# Neuroproteome Changes after Ischemia/Reperfusion Injury and Tissue Plasminogen Activator Administration in Rats: A Quantitative iTRAQ Proteomics Study

**DOI:** 10.1371/journal.pone.0098706

**Published:** 2014-05-30

**Authors:** Zamir Merali, Meah MingYang Gao, Tim Bowes, Jian Chen, Kenneth Evans, Andrea Kassner

**Affiliations:** 1 Department of Medical Imaging, University Of Toronto, Toronto, Ontario, Canada; 2 Physiology and Experimental Medicine, The Hospital For Sick Children, Toronto, Ontario, Canada; 3 Ontario Cancer Biomarker Network, Toronto, Ontario Canada; Massachusetts General Hospital, United States of America

## Abstract

The thrombolytic, recombinant tissue plasminogen activator (rt-PA) is the only approved therapy for acute ischemic stroke (AIS). When administered after AIS, rt-PA has many adverse pleiotropic actions, which are currently poorly understood. The identification of proteins showing differential expression after rt-PA administration may provide insight into these pleiotropic actions. In this study we used a 2D-LC MS/MS iTRAQ proteomic analysis, western blotting, and pathway analysis to analyze changes in protein expression 24-hours after rt-PA administration in the cortical brain tissue of 36 rats that underwent a sham or transient middle cerebral artery occlusion surgery. After rt-PA administration we reported alterations in the expressions of 18 proteins, many of which were involved in excitatory neurotransmitter function or cytoskeletal structure. The expression changes of GAD2 and EAAT1 were validated with western blot. The interactions between the identified proteins were analyzed with the IPA pathway analysis tool and three proteins: DPYSL2, RTN4, and the NF-kB complex, were found to have characteristics of being key proteins in the network. The differential protein expressions we observed may reflect pleiotropic actions of rt-PA after experimental stroke, and shine light on the mechanisms of rt-PA's adverse effects. This may have important implications in clinical settings where thrombolytic therapy is used to treat AIS.

## Introduction

A thrombolytic agent, rt-PA (recombinant tissue plasminogen activator) is the only therapeutic used in the treatment of acute ischemic stroke (AIS) [1]. Numerous exclusion criteria, such as a narrow therapeutic window of 4.5 hrs, limit the number of patients receiving rt-PA. Even amongst eligible patients, rt-PA is substantially underused, with only 3% to 8.5% of potentially eligible patients receiving the drug [2]. The association of rt-PA with severe adverse effects has contributed to reluctance amongst emergency physicians to utilize the drug [3].

rt-PA is capable of both beneficial reperfusion due to its interaction with plasminogen, and neurotoxicity due to its pleiotropic effects on other brain tissue substrates [4]. While many of the pleiotropic effects are poorly characterized, the interaction between the N-methyl-D-aspartate (NMDA) receptor and rt-PA has received substantial experimental attention. In the presence of rt-PA, NMDA receptor signaling is potentiated, and the ensuing dysregulation of intracellular calcium levels contributes to neuron and glia death after stroke [5]. NMDA receptor antagonists were seen as promising neuroprotective agents for their ability to disrupt this process and perhaps mediate the harmful effects of rt-PA in an animal model [6]. The ultimate failure, however, of the NMDA receptor antagonists in clinical trials was demonstrative of the simplification of the NMDA receptor’s role in the complex pathophysiology of stroke [7]. More broadly, it demonstrated the tendency to overemphasize the role of a single pathway in mediating a complex disease state. To achieve an understanding of how rt-PA contributes to the pathophysiology of AIS, it may not be sufficient to utilize a traditional biochemical approach, in which pathways are investigated in isolation [8]–[10].

With recent advances in proteomics technology, the overall protein expression of various samples can be compared within a single experiment. Analysis of these results with bioinformatics tools can generate a more comprehensive understanding of the involved pathways [11]. Neuroproteomic studies of AIS have varied in their methods, with many published before 2010 making use of the 2D-gel-based approach (2D-GE MS/MS) [12]–[15]. A small number of more recent studies have made use of LC MS/MS-based multi dimensional protein identification [9], [10]. When combined with the multiplex isobaric tag for relative and absolute quantification (iTRAQ) method, the LC MS/MS approach can simultaneously quantify proteins in 4 or 8 samples [16]. A number of studies have successfully utilized neuroproteomic methods to analyze differentially expressed proteins after ischemic stroke, and these results have deepened our understanding of stroke pathophysiology. However, although rt-PA administration alters the pathophysiology of stroke in a clinically significant way, no study that we are aware of has attempted to measure the proteome changes associated with rt-PA administration after AIS.

Since many of rt-PA’s pleiotropic interactions are uncharacterized, the identification of proteins showing differential expression after rt-PA administration may shed light on the molecular mechanisms underlying rt-PA’s adverse effects. We sought to apply 2D-LC MS/MS and iTRAQ proteomics directly to cortical brain tissue to analyze how rt-PA administration altered the proteome in a transient rodent stroke model. A further aim of this study was to probe the source of this protein regulation with a pathway analysis tool.

## Materials and Methods

### Reagents

All agents were purchased from Sigma-Aldrich (Oakville, Ontario) unless otherwise indicated.

### Ethics Statement

The Hospital For Sick Children Animal Research Committee approved all procedures and protocols in accordance with the protocols of the Canadian Council of Animal Care. At the experimental endpoint all rats were humanely sacrificed with an intra-peritoneal euthanyl injection in accordance with the Canadian Council of Animal Care guidelines.

### Proteomics Experimental Design

**Figure 1:**
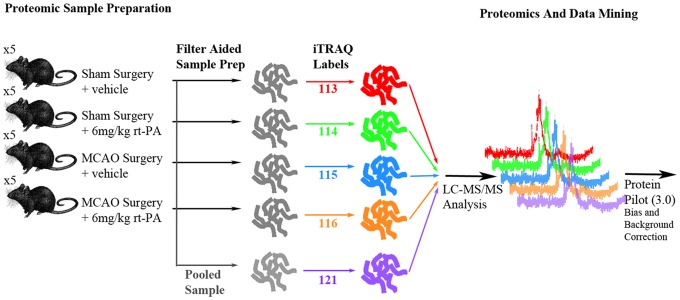
Schematic of 2D LC-MS/MS experimental design. Five iTRAQ labels were used: 113 for the Sham+distilled water injection. 114 for the Sham+rt-PA infusion. 115 for tMCAo+distilled water injection. 116 for tMCAo+rt-PA infusion. 121 for a pooled sample containing equal amounts from each group. Treated rats were given 6 mg/kg rt-PA 4 hrs after occlusion. All samples were pooled after iTRAQ labeling and subjected to mass spectrometric analysis.

Sprague dawley rats (300–350 g males, Charles-River Laboratories, Sherbrooke, Canada), in pathogen free housing, were made use of. The experimental design for the iTRAQ proteomics analysis is as shown in Figure 1. Rats were randomized into one of four groups with a computerized random number generator (five per group). These groups were: a Sham group - rats receiving a sham surgery followed by IV sterile water infusion 4 hours after occlusion; Sham+rt-PA Group - rats receiving a sham surgery followed by a 10 minute intravenous infusion of 6 mg/kg rt-PA 4 hours after occlusion; tMCAo Group - rats receiving a 1 hr tMCAo followed by an IV sterile water infusion 4 hours after occlusion; and tMCAo+rt-PA Group, rats receiving a 1 hr tMCAo followed by a 10 minute intravenous infusion of 6 mg/kg rt-PA 4 hours after occlusion. An endpoint of 24-hours was chosen because in the tMCAo rodent model the risks of hemorrhage and neuronal death reach a peak at this time-point. In addition, this choice of end-point allowed for maximum comparability with previous results, as previous neuroproteomic studies have predominantly chosen an end-point of 24-hours. All rats were euthanized with an IP euthanyl injection. For the 2D LC-MS/MS iTRAQ analysis 5 biologic replicates for each experimental group and 1 injection was performed.

### Transient Ischemia Model

A transient focal ischemia was induced in rats under isoflurane anesthesia with the transient middle cerebral artery occlusion (tMCAo) procedure as previously described [17]. A 0.39 mm diameter filament tip was used and the occlusion was maintained for 1 hr. For sham operations the carotid bifurcation was exposed, the external carotid was ligated, and the filament was inserted the correct distance before being immediately withdrawn. Core body temperature was monitored continuously and kept constant at 37°C. Rats were given 0.03 mg/kg buprenorphine for post-surgical pain relief. At 5-hours after occlusion rats were given 2 mL of subcutaneous fluids. During the surgery heart rate, and breathing rate were monitored periodically.

### Tissue Extraction

At 24-hours after euthanasia brains were quickly removed and sectioned into 2 mm slices over ice. The right hemisphere from a slice 0.3 mm anterior of bregma was carefully dissected under magnification over ice to isolate the cortical tissue. The cortical tissue was flash frozen in liquid nitrogen and stored at −80**°**C until proteomic analysis. The slices anterior and posterior to this slice were stained with Tetrazolium Chloride (TTC). Images of TTC stained sections were imported into the Image J software (NIH). The infarcted areas were manually traced, as were the total hemispheric areas. Rats with infarct sizes less than 40% cross-sectional area of the sampled right hemisphere sections were excluded from the proteomic analysis.

### Proteomics Sample Preparation

The protocol used was the Filter Aided Sample Preparation (FASP) method originally developed by Matthial Mann [18]. FASP is able to reduce SDS and contaminant levels, and increase the yield of membrane bound and nuclear proteins. Cortical tissue was weighed and dissolved into 800 uL of lysis buffer (3% SDS, 5 mM TCEP, 0.1 M Tris-HCl at pH 7.6). After sonication the samples were heated at 95 C for 5 min. The samples were then centrifuged at 14000 g in a microcentrifuge for 10 minutes. The supernatants were isolated and protein content was measured using the Bio-Rad DC assay, having diluted 10 µL of sample into 1 mL of assay reagent buffer to reduce TCEP concentration to below acceptable assay thresholds. Once the concentrations of each sample were determined, 60 µg from each animal in the same treatment were pooled to a total of 300 µg. Following this, Nanosep 10 kDa filters (Pall Life Sciences, Ann Arbor, MI, USA) were rinsed with 100 µL deionized, distilled water, and spun through the filter. Then, the filters were rinsed with 100 µL of fresh urea buffer (8 M urea in 0.1 M Tris-HCl, pH 8.5), which was also spun through and discarded. 200 µg of sample was loaded onto each filter and spun through for 15 min at 14000 g. Following this, 100 µL of 50 mM iodoacetamide in urea buffer was applied to the filters and mixed for 1 min at 600 rpm. The filters were incubated at room temperature for 20 min and the buffer was removed by centrifugation at 14000 g for 15 min. The filters were then rinsed with 100 µL of urea buffer and then rinsed with 100 µL of 0.5 M TEAB removing buffer by centrifugation after each rinse. 40 uL of 1 µg Trypsin in 0.5 M TEAB was added and the filters were incubated at 37 C for 18 h. The digested samples were isolated by centrifugation, spinning through another 40 µL of 0.5 M TEAB to improve tryptic peptide yield. Peptide concentration was determined using a Nanospec (Nanolytik, Germany).

### iTRAQ Labeling and 2D LC-MS/MS Analysis

The iTRAQ proteomics method provides extensive quantification coverage of around 88% with one technical replicate [19]. iTRAQ proteomics was performed using an 8-plex procedure (only 5 samples were analyzed) so that all experimental samples were processed simultaneously. The iTRAQ labels for each group were as follows: 113 - sham surgery and vehicle, 114 – sham surgery and rt-PA, 115 – tMCAo surgery and vehicle, 116 – tMCAo surgery and rt-PA. Thirty µg of protein was used for iTRAQ labeling following the manufacturer’s instructions with no modifications. Trypsin-digested and iTRAQ labeled samples were then pooled and subjected to strong cation exchange (SCX) fractionation on a Biobasic SCX column (4.6 mm I.D×15 cm L., Thermo, USA). Fifteen fractions were collected and injected into a nanoflow high-performance liquid chromatographer (HPLC) (Proxeon, Denmark), equipped with a 360 um filter and packed with Zorbax SB18-300 reversed phase (Agilent, USA). This HPLC set-up was coupled with a hybrid quadrupole/Time of flight MS (QStar Elite, AB Sciex, USA) and data was collected using Analyst QS 2.0 controlling software (AB Sciex, USA) through a nanospray ion source. ProteinPilot version 3.0 (AB Sciex, USA) was used for processing the raw acquired iTRAQ data files. The protein identification was performed by searching raw MS data against the Swissprot-Uniprot protein database (January, 2010), with species restriction to Rattus norvegicus. Default search parameters were predominantly used.

### PANTHER Gene Ontology

The PANTHER classification system v8.1 (California) was used according to a published protocol [20]. The Uniprot IDs of the 209 proteins identified by 2 or more peptides and 113 proteins identified by 1 peptide were uploaded separately to the PANTHER online tool. The Rattus norvegicus genome was chosen as the reference database. From the output report aggregate functional and sub-cellular location information was extracted. This was compared with the aggregate data from the entire Rattus norvegicus genome of 20966 proteins.

### Pathway and Centrality Analysis

The Uniprot ID of the 403 proteins identified by 2D LC-MS/MS iTRAQ with an FDR<0.01 were uploaded to the Ingenuity Pathway Analysis (IPA) version 7.5 (Redwood City, CA) software. IPA allows for the identification of protein-protein interactions and network interactions based on a database of manually curated published protein interactions. We utilized a p-value cut-off at 0.05 and no fold change cut-off. Each Uniprot ID meeting this cut-off was mapped to the corresponding gene object in the Ingenuity pathway knowledge base. These genes are referred to as focus genes, and were overlaid onto the curated global molecular networks contained within the Ingenuity knowledge base. Next, networks of the focus genes were generated based on protein-protein interactions and each network was scored based on a Fisher test. The network score represented the relevance of the network to the input Uniprot IDs. The highest scoring network was chosen and was represented as a graph that indicated the interactions (direct or indirect) between proteins. The relative expression data from the tMCAo+rt-PA comparison was overlaid onto this network. Each node indicated a single protein, and the green or red color indicated up- or down- regulation as seen in the 2D LC-MS/MS iTRAQ analysis.

Lastly, the degree centrality for each protein in the network was calculated as previously described using the IPA software [11]. Briefly, the degree centrality is the simplest measure of centrality and represents the number of connections (direct or indirect) made by each protein in the network regardless of the direction of the connection. To determine the proteins that had the highest levels of centrality we used the standard cut-off of 5% [21]. Thus, the proteins with degree centrality measures in the top 5% of the distribution were identified as having high degree centrality.

### Confirmation of Differentially Expressed Proteins by Western Blot

Separate animals were used for the western blot analysis (4 per group). At 24-hours after occlusion rats were euthanized with an IP euthanyl injection and the cortical tissue was extracted following the same procedure as for iTRAQ sample preparation. Whole-cell protein extracts were prepared from the cortical tissue. Primary antibodies used were: GAD2 (5834P, Cell Signalling), EAAT1 (5684P, Cell Signalling), and Vinculin (4650P, Cell Signalling). Infrared conjugated secondary antibodies (LI-COR) were used and luminescence was quantified using an Odyssey infrared imaging system.

### Statistical Analysis

For data processing the ProteinPilot software v.3.0 (AB SCIEX, Framingham, USA) was used. The false discovery rate (FDR) for each protein was estimated using a concatenated target-decoy database as described by Elias and Gygi. [22] This generated a probability measure for each protein. To determine iTRAQ protein quantification the Pro Group algorithm within ProteinPilot was used to calculate a p-value of each relative expression change. Quantitation data for each protein was based on the underlying peptides that were determined to be unique by Pro Group. Within Pro Group, the null-hypothesis was taken as a lognormal distribution of unique peptide quantities and the p-value of the expression change was calculated from the standard deviation in logspace.

We imposed a strict set of criteria to reduce false positive expression changes. For a protein to be accepted as having a differential expression between treatment groups we used the following criteria: The protein must have been identified in all 5 iTRAQ preparations. Only proteins that were identified with at least 2 peptides with greater than 95% confidence were considered. Proteins with 10% increase or decrease in expression (iTRAQ ratios 114∶113, 116∶115, or 113∶115 greater than 1.1 or less than −1.1) and a significant p-value (p<0.05), were considered to be differentially expressed.

The densitometry results of the western blot experiments were analyzed in the Excel program (Microsoft, 2013) with a two-tailed student’s T-test and p<0.05 was taken as being significant.

Degree centrality data from the IPA analysis was also analyzed with the Excel program. Degree centrality data was taken to have a normal distribution and proteins with high levels of degree centrality were identified by calculating the standard deviation within the distribution.

## Results

In total 44 rats underwent tMCAo or sham surgery and tissue extraction. Six rats died before the experimental end-point and were excluded from further analysis. Two rats had an insufficient stroke size (<40% hemispheric area) upon TTC staining and were also excluded from further analysis. This left 36 rats to undergo tissue analysis. Twenty of these rats underwent 2D LC-MS/MS iTRAQ analysis with 5 rats per experimental group. The remaining sixteen rats underwent western blotting experiments with 4 rats per experimental group.

**Figure 2:**
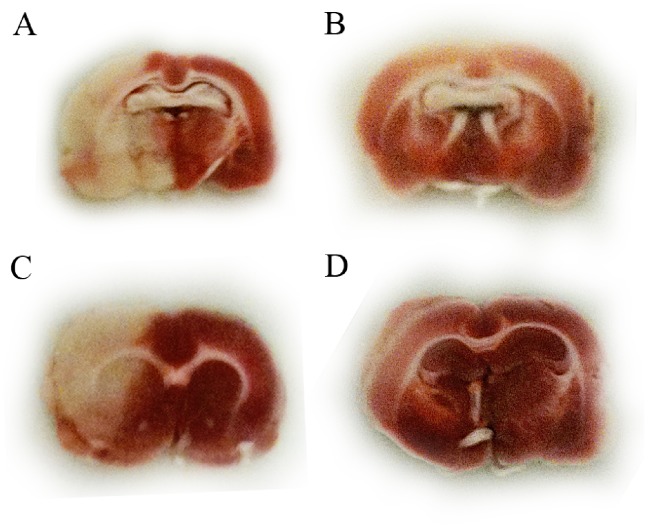
Representative tetrazolium chloride (TTC) stained sections of (A) transient middle cerebral artery occlusion (tMCAo)+rt-PA rats (B) sham+rt-PA rats (C) tMCAo+vehicle rats and (D) sham+vehicle rats. Dark areas reflect living viable tissue while white reflects infarcted tissue or areas of white matter. Infarcted areas occupy at least 50% of the right hemisphere in all tMCAo rats.

Physiologic parameters and core body temperature did not differ between groups (data not shown). All rats that underwent further analysis had large strokes through the basal ganglia and cortical regions (Figure 2) occupying at least 40% of the hemispheric cross-sectional area.

### Protein Identification and Relative Expression

**Figure 3:**
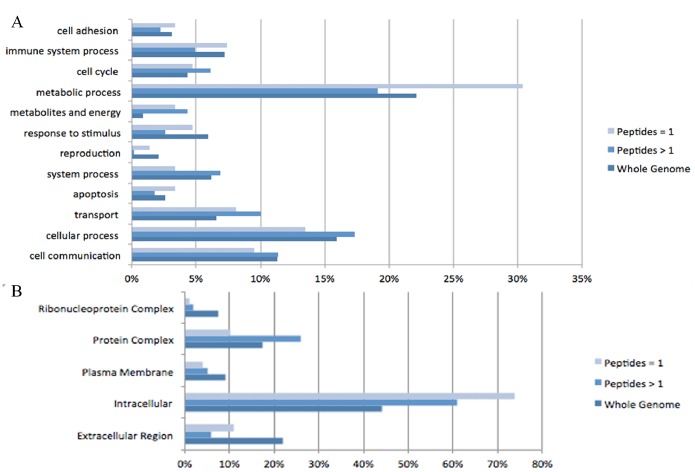
Functional distribution (A) and Sub-cellular distribution (B) of proteins identified with 1 peptide and 2 or more peptides compared to the entire Rattus norvegicus genome. Functional and spacial categories are those defined in the PANTHER database.

Overall our analysis identified 1143 distinct proteins, of which 403 had an estimated global False Discovery Rate (FDR)<0.01 (Table S1). As an assessment of quality we compared the distributions of biologic functions of these 403 proteins (209 identified with 2 or more peptides and 113 identified by 1 peptide) with the entire Rattus norvegicus genome of 20966 proteins (Figure 3A). 12 functional categories were defined utilizing the PANTHER gene ontology database. Proteins identified with 2 or more peptides are comparable in distribution to the overall genome. Proteins identified with 1 peptide are over-represented in the metabolic process category.

**Figure 4:**
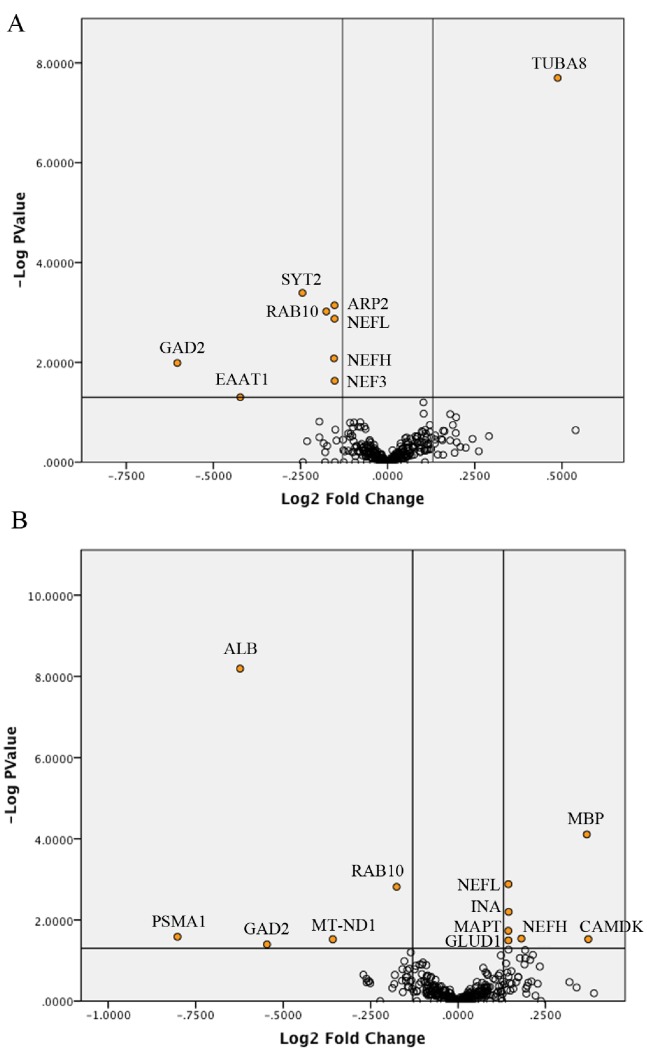
Volcano plots showing the distribution of significance and fold change of identified proteins in the (A) sham+rt-PA/sham comparison and (B) tMCAo+rt-PA/tMCAo comparison. All proteins with 2 or more peptides were plotted with fold change on the x-axis and p-value on the y-axis. Vertical lines mark a fold change of +/−10% and the horizontal line marks p  = 0.05. The 18 proteins marked in orange with text labels meet the criteria for having differential expression (>10% in either direction) and are significant (<p = 0.05).

We next compared the distribution based on sub-cellular location (Figure 3B). We have identified higher percentages of proteins from the cytoplasm, and lower percentages of proteins from the plasma membrane, extra cellular region, and nucleus. We chose to consider only proteins identified by 2 or more peptides in our comparative analysis, as there is higher reliability amongst these identifications ^20^. The 115 and 113 labels in our 2D LC-MS/MS iTRAQ analysis are similar to a previous neuroproteomic study by Datta et al., which compared protein expression in rat brain 24-hours after tMCAo surgery with a sham group. Comparison of the 115 and 113 labelled proteins from our analysis, with the Datta et al study, revealed a 74% overlap between the two lists of identified proteins [10].

(Table 1) summarizes the expressions changes (fold changes) of protein pairs identified in the experimental groups. (Figure 4) illustrates the distribution of fold changes amongst the identified proteins. The main expression changes were seen in the tMCAo+rt-PA comparison (Figure 4B), where 13 proteins were significantly up- or down-regulated. The same number of significant expression changes were seen in the tMCAo/sham comparison. Four proteins which were over-expressed after tMCAo were significantly down-regulated with tMCAo+rt-PA. These were glutamate decarboxylase 2 (GAD2), proteasome subunit alpha (PSMA2), Ras-related protein Rab-10 (RAB10), and Synapsin-2 (SYN2). Both the Myelin basic protein (MBP) and the Neurofilament heavy polypeptide (NEFH) were significantly down-regulated after tMCAo and significantly up-regulated after rt-PA administration following tMCAo.

**Table 1:** Between group comparisons of protein expression fold changes after tMCAo and rt-PA administration meeting the criteria outlined in ‘Statistical Analysis’.

Name	Uniprot ID	Peptides (95%)	control+rt-PA/control	tMCAO/control	tMCAO+rt-PA/tMCAO
			Fold Change	Fold Change	Fold Change
**Actin-related protein-2**	Q5M7U6	4	**−1.11**	−1.04	−1.09
**Alpha-internexin**	P23565	26	−1.03	1.09	**1.11**
**Calcium/calmodulin-dependent protein kinase type II subunit delta**	P15791	12	−1.09	**−1.48**	**1.29**
**Excitatory amino acid transporter 1**	P24942	4	**−1.34**	**−1.27**	1.07
**Glutamate decarboxylase 2**	Q05683	6	**−1.52**	**1.15**	**−1.46**
**Glutamate dehydrogenase 1, mitochondrial**	P10860	14	−1.03	−1.07	**1.10**
**Microtubule-associated protein tau**	P19332	5	−1.04	−1.08	**1.13**
**Myelin basic protein S**	P02688	20	1.03	**−1.22**	**1.29**
**NADH dehydrogenase [ubiquinone] flavoprotein 2, mitochondrial**	P19234	2	1.01	1.00	**−1.28**
**Neurofilament heavy polypeptide**	P16884	15	**−1.11**	**−1.20**	**1.19**
**Neurofilament light polypeptide**	P19527	32	**−1.11**	−1.09	**1.10**
**Neurofilament medium polypeptide**	P12839	27	**−1.11**	−1.07	−1.01
**Proteasome subunit alpha type-2**	P17220	2	−1.01	**1.16**	**−1.74**
**Ras-related protein Rab-10**	P35281	4	**−1.14**	**1.10**	**−1.16**
**Serum Albumin**	P02770	28	1.03	**1.89**	**−1.54**
**Synapsin-2**	Q63537	17	1.07	**1.14**	−1.09
**Synaptotagmin-2**	P29101	2	**−1.19**	**−1.25**	1.05
**Tubulin alpha-8 chain**	Q6AY56	55	**1.40**	1.00	−1.02

Fold changes in **bold** were significantly altered.

Six proteins that were unaffected after tMCAo alone had their expressions significantly altered when rt-PA was administered after the tMCAO. These included 3 cytoskeletal proteins: (INA, MAPT, NEFL), a nucleotide phosphodiesterase (CNP1), and two dehydrogenases (GLDH1, NDUFV2).

Surprisingly there were four proteins affected by rt-PA administration in the sham group, which were unaffected by tMCAo, and rt-PA administration after tMCAo. These were the cytoskeletal proteins (ARP2, NEF3, TUBA8) and the phosphatase subunit PPP1CB. Only 2 proteins (GAD2 and RAB10) had their expressions significantly affected in the same direction when rt-PA was administered to the sham group as when rt-PA was administered to the tMCAo group. There were two cytoskeletal proteins (NEFH and NEFL) that showed opposite expression changes when rt-PA was given to the sham group compared to when it was given to the tMCAo group.

### Pathway Analysis

**Figure 5:**
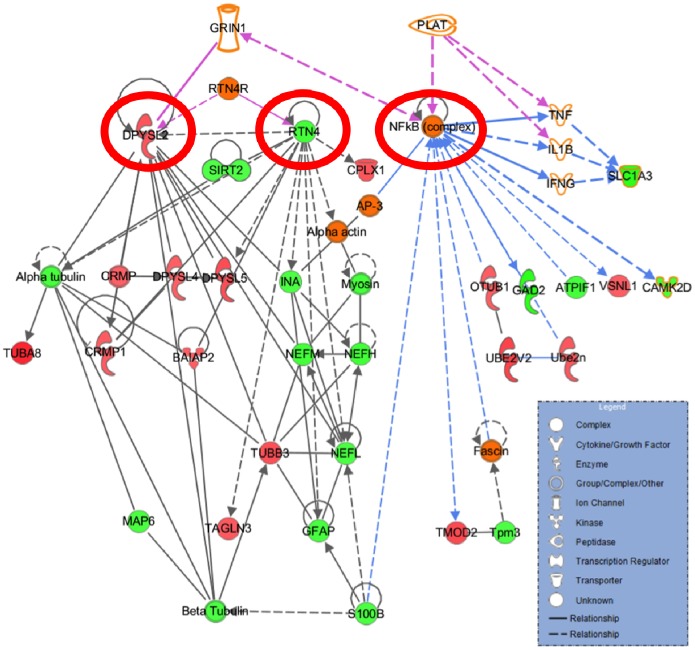
Results of an IPA pathway analysis of the tMCAo comparison. Encircled proteins are DPYSL2, RTN4, and NF-kB, which show a high centrality measure. Green nodes are up-regulated proteins, red nodes are down-regulated. A line between two nodes represents a direct or indirect interaction noted in the literature.

(Figure 5) represents the output of the IPA analysis with relative expression information overlaid. In this network Dihydropyrimidinase-related protein 2 (DPYSL2), reticulon 4 (RTN4), and the nuclear factor-kappa B (NF-kB) complex had degree centralities in the upper 5% of the distribution. Both DPYSL2 and RTN4 connected to 11 identified proteins, the majority of which were related to cytoskeleton structure/maintenance. The NF-kB interacted with 10 identified proteins, many of which were related to excitatory neurotransmitter function.

### Western Blot Validation

**Figure 6:**
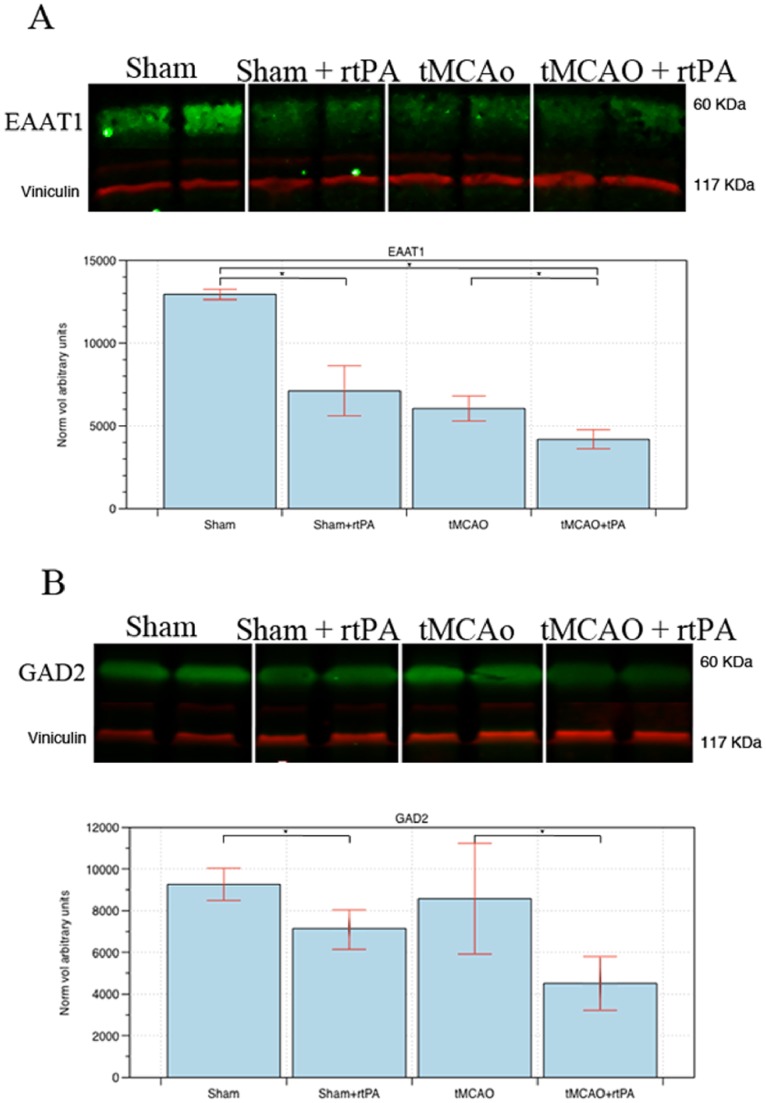
Quantification of protein expression by western blot. (A) Excitatory amino acid transporter 1 (EAAT1) and (B) Glutamate decarboxylase 2 (GAD2) expressions analyzed by western blot. Bands were quantified using scanned densitometry and normalized to viniculin expression. Vertical axis shows an arbitrary density unit. Asterisks mark significant changes (p<0.05, n = 4).

In order to confirm the results from the 2D LC-MS/MS experiment western blots were used to quantify the expression of GAD2 and EAAT1 (Figure 6). GAD2 and EAAT1 were chosen, as these are proteins that have previously been implicated in exacerbating cell death after AIS and may represent adverse pleiotropic actions of rt-PA. The western blot analysis of GAD2 and EAAT1 confirmed the iTRAQ data.

## Discussion

Recombinant tissue plasminogen activator is the only medical agent used in the acute treatment of AIS, but because of its pleiotropic actions it is associated with severe adverse effects [23], [24]. Since the extent of rt-PA’s pleiotropic interactions are unknown, the identification of proteins that show differential expression after rt-PA administration may shed light on the molecular mechanisms underlying rt-PA’s adverse effects. In this study we used a 2D LC-MS/MS iTRAQ proteomics approach to characterize the brain protein changes that result 24 hrs after rt-PA administration. We have identified 18 proteins showing differential expression after rt-PA administration to either sham rats or rats that underwent tMCAo. Many of these proteins were associated with either excitatory neurotransmitter function or cytoskeletal structure. By applying a pathway analysis tool to the results of the 2D LC-MS/MS iTRAQ analysis we identified 3 proteins: DPYSL2, RTN4, and the NF-kB complex as having characteristics of being key proteins in the interaction network. These results may provide insight into the potential mechanisms underlying the adverse effects of rt-PA use after AIS.

In this study, the rat tMCAo model and the iTRAQ MS analysis are potential sources of variation [15]. We utilized sample pooling, replicates, internal controls, and bioinformatic analyses to address these concerns, and assess their influence on the data, as these strategies have been validated [13], [25], [26]. The group of 209 proteins identified with 2 or more peptides showed similar functional and sub-cellular distributions to the entire Rattus norvegicus genome. The under-representation of nuclear and membrane bound proteins is an expected artifact, and our yield from these two compartments is comparable to another study utilizing the FASP protocol and exceeds that of studies which did not utilize FASP [27]. The comparable spacial and functional distributions yielded, as well as a high overlap between our results and the study by Datta et al. lend support to the reliability of our sample collection and analysis.

Excitatory neurotransmitter function is thought to become deregulated after stroke, leading to excitotoxic cell death through excessive activation of NMDA and AMPA type receptors [5], [28]. Previous work has shown that after stroke, rt-PA potentiates glutamate activity at NMDA receptors by proteolytically cleaving the NR-2 subunit [29]. In addition, excitatory amino acid transporters can reverse transport direction under ischemic conditions, leading to a further increase in synaptic glutamate levels. [30] Our results indicated that after sham and tMCAo surgery, rt-PA had caused down-regulation of the EAAT1 and GAD2 proteins, both of which are responsible for reducing the levels of synaptic glutamate and other excitatory neurotransmitters. GAD2 is an enzyme that converts synaptic glutamate into the inhibitory GABA [31]. In an animal model, GAD2 was neuroprotective against excitotoxic damage by reducing levels of available glutamate in pre-synaptic neurons [32]. EAAT1 is the predominant transporter responsible for removing glutamate and aspartate from the synapse [33]. Like GAD2, EAAT1 activity is neuroprotective by reducing synaptic glutamate levels [34]–[36]. In addition, our results showed alterations in the expressions of synaptic vesicle proteins RAB10, SYN2, and SYT2. RAB10 is a key regulator of intra-cellular membrane trafficking, and may be responsible for recycling AMPA receptors from intracellular compartments to the synapse [37]. SYN2 and SYT2 are both involved in facilitating neurotransmitter release into the synapse. Previous studies have indicated that dysfunctions in the expression levels of the SYN and SYT families can markedly affect the extent of ischemic brain injury [38], [39]. Our results also indicated that rt-PA had caused a change in the expression of the CaMK2δ, a protein responsible for sensing the rise in intracellular calcium levels due to excitatory neurotransmitter stimulation at the NMDA receptors [40]. Our results demonstrated that a number of proteins involved in excitatory neurotransmitter function were differentially expressed after rt-PA administration to sham and tMCAo rats. Many of these proteins have been previously implicated in neuronal damage after ischemic injury, and the observed differential expressions may reflect adverse pleiotropic effects of rt-PA administration.

Cytoskeletal structure is necessary for neurovascular integrity [41], [42]. Alterations of cytoskeletal function and composition occur after ischemic stroke [43]. Our results indicated differential expression of cytoskeletal and cytoskeleton regulatory proteins after rt-PA administration to both sham and tMCAo rats. The family of neurofilament proteins is a major element of the neuronal cytoskeleton, regulating axonal structure and diameter. The increase in neurofilament proteins we observed with rt-PA administration in tMCAo rats may indicate increased axonal growth [44]. In addition, our observations indicated that the expression of cytoskeletal proteins such as ARP2, INA, MAPT, and TUBA8 may be highly sensitive to rt-PA administration after tMCAo, leading to alterations in cytoskeletal structure and function.

Pathway analysis has become a standard method to gain insight into the underlying biology of differentially expressed proteins identified by proteomic analyses [45]. We generated an interaction network from the results of our 2D LC-MS/MS iTRAQ experiment and used the IPA software to perform a centrality analysis on this network. In a previous study, proteins with high centrality were 3 times more likely to be biologically essential [46]. It therefore appears that DPYSL2, RTN4, and NF-kB complex, which all showed high centrality in our protein-protein interaction network, may serve important functions in this network. DPYSL2 is involved in neuronal regeneration and multiple studies have identified changes in DPYSL2 expression and function shortly after ischemic brain injury [47]. RTN4 is also involved in neuronal regeneration, and has been proposed as a potential therapeutic target because of its role in exacerbating damage after experimental stroke [48]. The NF-kB complex regulates the expression of many proteins and its activation is known to exacerbate ischemic brain injury while inhibition of the NF-kB complex is neuroprotective [49]. The NF-kB complex can be activated by rt-PA through a pathway involving annexin A2/CD11b and integrin-linked kinase [50]. In our model the activated NF-kB may go on to cause a subset of the protein expression changes noticed in the 2D LC-MS/MS iTRAQ analysis. It is important to note that our pathway analysis is limited to simply suggesting that DPYSL2, RTN4, or the NF-kB complex may be relevant to rt-PA’s pleiotropic actions. Pathway analyses are limited in part by low-resolution knowledge databases, missing condition-specific interactions, and incomplete annotations. Despite this, however, these results may prompt further research into rt-PA’s pleiotropic effects on the cortex. For example, we are currently examining the effect of rt-PA on various proteins under the control of the NF-kB complex after experimental stroke.

In conclusion, a 2D LC-MS/MS iTRAQ analysis identified 18 proteins with differential expression 24-hours after rt-PA administration to rats that underwent sham or tMCAo surgery. Many of the identified proteins have been previously implicated in ischemic brain injury. The differential protein expressions we observed may reflect pleiotropic actions of rt-PA on the cortex of rats. Although further focused work is required to investigate these protein regulations in isolation, the general overview of protein regulation presented in our study provides insight into the potential mechanisms of rt-PA’s adverse pleiotropic actions. This work may guide future work and may have implications in clinical settings where rt-PA is used to treat AIS.

## Supporting Information

Table S1 **Output of false discovery rate (FDR) characteristics for identified peptides and proteins.**
(XLS)

Table S2 **Output of relative protein expression changes between iTRAQ labels 113/114 and 115/116.**
(PDF)
