# Supplementary material for: Neuroproteome Changes after Ischemia/Reperfusion Injury and Tissue Plasminogen Activator Administration in Rats: A Quantitative iTRAQ Proteomics Study
Source: PLoS One. 2014 May 30;9(5):e98706. doi: 10.1371/journal.pone.0098706 (PMC4039533; doi:10.1371/journal.pone.0098706)
Supplement: Table S2 — Output of relative protein expression changes between iTRAQ labels 113/114 and 115/116. (PDF) [file pone.0098706.s002.pdf]

N	Unused	Total	%Cov	%Cov(50)	%Cov(95)	Name	Species	Peptides(95%)	114:113	PVal 114:113	115:113	PVal 115:113
818	0.09	0.12	22.21000046	0	0	G-protein coupled receptor-associated sorting protein 1 OS=Rattus norvegicus GN=Gprasp1 PE=1 SV=1	RAT	0	1.564582944		1.453423977	
346	1.92	1.92	55.69999814	7.594999671	7.594999671	Low molecular weight phosphotyrosine protein phosphatase OS=Rattus norvegicus GN=Acp1 PE=1 SV=3	RAT	1	1.546200991		0.852979422	
592	0.22	0.25	22.81000018	0.279899989	0	Cadherin EGF LAG seven-pass G-type receptor 2 (Fragment) OS=Rattus norvegicus GN=Celsr2 PE=2 SV=1	RAT	0	1.511289001		0.893113315	
403	1	1	33.55000019	11.60999984	11.60999984	V-type proton ATPase 16 kDa proteolipid subunit OS=Rattus norvegicus GN=Atp6v0c PE=2 SV=1	RAT	1	1.502673984		1.106804013	
1006	0.06	0.07	34.09999907	0	0	REVERSED Cadherin EGF LAG seven-pass G-type receptor 2 (Fragment) OS=Rattus norvegicus GN=Celsr2 PE=2 SV=1	RAT	0	1.495154977		1.001330972	
1067	0.05	0.08	28.16999853	0	0	REVERSED Down syndrome cell adhesion molecule homolog OS=Rattus norvegicus GN=Dscam PE=1 SV=1	RAT	0	1.480672002	0.168339103	1.093742013	0.553084314
217	4.09	4.11	67.93000102	9.309999645	9.309999645	Enoyl-CoA hydratase, mitochondrial OS=Rattus norvegicus GN=Echs1 PE=1 SV=1	RAT	2	1.453307033	0.228512302	1.189098001	0.341521204
344	2	2	40.20999968	15.45999944	15.45999944	Mitochondrial import inner membrane translocase subunit Tim8 A OS=Rattus norvegicus GN=Timm8a PE=1 SV=1	RAT	1	1.451686025		1.608926058	
682	0.15	0.16	30.39999902	1.494999975	0	REVERSED Protein FAM194A OS=Rattus norvegicus GN=Fam194a PE=2 SV=1	RAT	0	1.435729027		1.384009004	
422	0.76	0.76	49.57000017	10.42999998	10.42999998	60S acidic ribosomal protein P2 OS=Rattus norvegicus GN=Rplp2 PE=1 SV=2	RAT	1	1.427633047		1.461395979	
718	0.13	0.15	36.41000092	4.084999859	0	Methylcrotonoyl-CoA carboxylase beta chain, mitochondrial OS=Rattus norvegicus GN=Mccc2 PE=2 SV=1	RAT	0	1.422505021		1.14338398	
798	0.1	0.1	18.00999939	1.401000004	0	Tyrosine-protein phosphatase non-receptor type 23 (Fragment) OS=Rattus norvegicus GN=Ptpn23 PE=1 SV=2	RAT	0	1.418805957		1.130720973	
1011	0.06	0.07	37.00000048	2.250000089	0	REVERSED Sphingosine 1-phosphate receptor 5 OS=Rattus norvegicus GN=S1pr5 PE=2 SV=1	RAT	0	1.415207982		1.220872998	
290	2.09	31.8	93.76000166	64.59000111	51.67000294	Tubulin alpha-8 chain OS=Rattus norvegicus GN=Tuba8 PE=2 SV=1	RAT	55	1.402178049		0.996187925	
932	0.07	0.09	27.12000012	0	0	Glycine N-acyltransferase-like protein Keg1 OS=Rattus norvegicus GN=Keg1 PE=1 SV=2	RAT	0	1.389631987		1.246685982	
363	1.7	1.72	76.35999918	5.454999954	5.454999954	40S ribosomal protein S10 OS=Rattus norvegicus GN=Rps10 PE=2 SV=1	RAT	1	1.386952996		1.375398993	
541	0.31	0.38	34.65999961	0.5076	0	REVERSED SH3 and multiple ankyrin repeat domains protein 1 OS=Rattus norvegicus GN=Shank1 PE=1 SV=1	RAT	0	1.353410959	0.2163876	0.982492387	0.802937925
657	0.17	0.17	26.03999972	1.49299996	0	REVERSED Polypeptide N-acetylgalactosaminyltransferase 10 OS=Rattus norvegicus GN=Galnt10 PE=2 SV=1	RAT	0	1.342342973		1.282811046	
840	0.09	0.09	30.12999892	4.675000161	0	REVERSED Coiled-coil domain-containing protein 130 OS=Rattus norvegicus GN=Ccdc130 PE=2 SV=1	RAT	0	1.335814953		0.936548412	
836	0.09	0.09	31.07999861	0.944100041	0	REVERSED Neogenin (Fragment) OS=Rattus norvegicus GN=Neo1 PE=2 SV=1	RAT	0	1.321954966		1.165173054	
819	0.09	0.12	23.75999987	0.601499993	0	REVERSED Ubinuclein-2 OS=Rattus norvegicus GN=Ubn2 PE=3 SV=1	RAT	0	1.318204999		1.307458997	
863	0.08	0.11	26.5899986	2.624999918	0	REVERSED Sodium/potassium/calcium exchanger 1 OS=Rattus norvegicus GN=Slc24a1 PE=2 SV=1	RAT	0	1.299716949		0.969774783	
446	0.63	0.66	42.48000085	3.398000076	3.398000076	REVERSED [Pyruvate dehydrogenase [lipoamide]] kinase isozyme 4, mitochondrial OS=Rattus norvegicus GN=Pdk4 PE=2 SV=1	RAT	1	1.296197057		0.873827219	
631	0.19	0.37	24.68000054	1.028000005	0	REVERSED Valyl-tRNA synthetase OS=Rattus norvegicus GN=Vars PE=2 SV=2	RAT	0	1.276432037	0.248952299	1.164769053	0.369920105
327	2	2.02	69.96999979	6.600999832	6.600999832	Monoglyceride lipase OS=Rattus norvegicus GN=Mgll PE=1 SV=1	RAT	1	1.268967032		1.270441055	
883	0.08	0.08	19.26999986	1.389000006	0	MAGUK p55 subfamily member 7 OS=Rattus norvegicus GN=Mpp7 PE=2 SV=2	RAT	0	1.265902042		1.221271992	
935	0.07	0.08	30.52999973	0.525100017	0	REVERSED Sister chromatid cohesion protein PDS5 homolog A OS=Rattus norvegicus GN=Pds5a PE=2 SV=1	RAT	0	1.260565042	0.540526211	1.378888011	0.391215712
1070	0.05	0.08	31.33000135	0	0	REVERSED ATP-binding cassette sub-family C member 9 OS=Rattus norvegicus GN=Abcc9 PE=2 SV=1	RAT	0	1.251474977	0.331819206	1.117879987	0.315204799
1100	0.05	0.06	34.11000073	1.589000039	0	Protein unc-45 homolog A OS=Rattus norvegicus GN=Unc45a PE=2 SV=1	RAT	0	1.249722958		1.261047006	
885	0.08	0.08	20.52000016	1.434999984	0	Long-chain-fatty-acid--CoA ligase 6 OS=Rattus norvegicus GN=Acsl6 PE=1 SV=1	RAT	0	1.247189045	0.530786693	1.028676987	0.958708227
219	4.07	4.07	54.43000197	23.24000001	23.24000001	Serine/threonine-protein phosphatase PP1-beta catalytic subunit OS=Rattus norvegicus GN=Ppp1cb PE=1 SV=3	RAT	4	1.246673942		1.135810018	
846	0.09	0.09	20.29999942	2.00500004	0	LanC-like protein 1 OS=Rattus norvegicus GN=Lancl1 PE=2 SV=2	RAT	0	1.242442012		1.088173985	
498	0.4	0.42	73.24000001	16.89999998	0	ATP synthase subunit e, mitochondrial OS=Rattus norvegicus GN=Atp5i PE=1 SV=3	RAT	0	1.241559982	0.39770329	1.034930944	0.819205403
692	0.14	0.22	37.99000084	0.337500009	0	REVERSED Striated muscle-specific serine/threonine-protein kinase OS=Rattus norvegicus GN=Speg PE=2 SV=2	RAT	0	1.232385993	0.368340999	1.007694006	0.934981525
502	0.39	0.4	45.12999952	3.895999864	0	REVERSED 3'(2'),5'-bisphosphate nucleotidase 1 OS=Rattus norvegicus GN=Bpnt1 PE=1 SV=1	RAT	0	1.231655002		1.144840956	
326	2	2.02	73.79999757	2.771000005	2.771000005	Acetyl-CoA acetyltransferase, cytosolic OS=Rattus norvegicus GN=Acat2 PE=1 SV=1	RAT	1	1.224786997		1.320888996	
349	1.88	1.88	58.75999928	18.0399999	18.0399999	Adenylate kinase isoenzyme 1 OS=Rattus norvegicus GN=Ak1 PE=1 SV=3	RAT	2	1.22311902	0.302144587	1.06160295	0.666210413
842	0.09	0.09	18.81999969	0	0	Methyltransferase-like protein 6 OS=Rattus norvegicus GN=Mettl6 PE=2 SV=1	RAT	0	1.220664978	0.564692914	1.055487037	0.77056253
773	0.11	0.11	30.88999987	2.703000046	0	Tonin OS=Rattus norvegicus GN=Klk2 PE=1 SV=1	RAT	0	1.218103051		1.069390059	
307	2.03	2.03	67.68000126	13.23000044	8.141999692	Isocitrate dehydrogenase [NAD] subunit gamma 1, mitochondrial OS=Rattus norvegicus GN=Idh3g PE=2 SV=2	RAT	1	1.217499971		1.053606987	
533	0.33	0.33	10.27999967	1.834999956	0	REVERSED Sterol O-acyltransferase 1 OS=Rattus norvegicus GN=Soat1 PE=2 SV=1	RAT	0	1.212617993		1.162250042	
558	0.27	0.28	47.74000049	2.160999924	0	Heat shock 70 kDa protein 14 OS=Rattus norvegicus GN=Hspa14 PE=2 SV=1	RAT	0	1.212617993		1.162250042	
598	0.22	0.22	36.48999929	9.730000049	0	Galanin receptor type 3 OS=Rattus norvegicus GN=Galr3 PE=2 SV=1	RAT	0	1.210584998	0.461366385	0.927197576	0.450583011
726	0.12	1.65	33.50999951	13.56000006	9.573999792	Alpha-centractin OS=Rattus norvegicus GN=Actr1a PE=1 SV=1	RAT	2	1.209287047		0.730521679	
741	0.12	0.13	24.60000068	3.162999824	0	REVERSED TBCC domain-containing protein 1 OS=Rattus norvegicus GN=Tbccd1 PE=2 SV=1	RAT	0	1.205682039		0.98789531	
1126	0.05	0.05	32.67999887	0	0	Echinoderm microtubule-associated protein-like 1 OS=Rattus norvegicus GN=Eml1 PE=2 SV=2	RAT	0	1.205639005	0.313560188	1.030696988	0.840607524
648	0.17	0.27	19.60999966	0.493199984	0	REVERSED DNA (cytosine-5)-methyltransferase 1 OS=Rattus norvegicus GN=Dnmt1 PE=1 SV=2	RAT	0	1.204753041		1.260100007	
340	2	2	60.14999747	10.53000018	10.53000018	Fatty acid-binding protein, heart OS=Rattus norvegicus GN=Fabp3 PE=1 SV=2	RAT	1	1.203099012		0.95738709	
781	0.1	0.16	30.64999878	0.404699985	0	Echinoderm microtubule-associated protein-like 5 OS=Rattus norvegicus GN=Eml5 PE=1 SV=2	RAT	0	1.201578975		0.970697105	
903	0.07	0.16	23.92999977	3.31799984	0	REVERSED DNA (cytosine-5)-methyltransferase 3-like OS=Rattus norvegicus GN=Dnmt3l PE=2 SV=1	RAT	0	1.200871944		0.996373117	
214	4.1	4.1	62.18000054	10.58000028	10.58000028	Pyridoxal kinase OS=Rattus norvegicus GN=Pdxk PE=1 SV=1	RAT	2	1.198642015	0.605821073	1.09977603	0.574756026
394	1.12	1.12	44.13999915	6.897000223	6.897000223	Ubiquitin-conjugating enzyme E2 variant 2 OS=Rattus norvegicus GN=Ube2v2 PE=1 SV=3	RAT	1	1.19857204		1.134032965	
298	2.06	2.06	98.6199975	6.207000092	6.207000092	40S ribosomal protein S19 OS=Rattus norvegicus GN=Rps19 PE=2 SV=3	RAT	1	1.197317004	0.343813986	1.025218964	0.862752616
325	2	2.03	96.10999823	8.948999643	8.948999643	6-phosphogluconolactonase OS=Rattus norvegicus GN=Pgls PE=1 SV=1	RAT	1	1.194538951		0.807516217	
511	0.37	0.39	33.57999921	1.700000092	0	REVERSED Coiled-coil and C2 domain-containing protein 1A OS=Rattus norvegicus GN=Cc2d1a PE=2 SV=2	RAT	0	1.19421196		1.056897998	
381	1.39	1.4	30.03999889	2.789999917	2.789999917	GRINL1A complex locus protein 1 OS=Rattus norvegicus GN=Gcom1 PE=1 SV=1	RAT	1	1.193932056		1.489002943	
767	0.11	0.12	29.0899992	4.431999847	0	REVERSED P2Y purinoceptor 4 OS=Rattus norvegicus GN=P2ry4 PE=2 SV=1	RAT	0	1.187909961	0.336813986	1.109385967	0.492427289
713	0.13	0.17	15.09000063	0.750700012	0	Neurogenic locus notch homolog protein 1 OS=Rattus norvegicus GN=Notch1 PE=2 SV=2	RAT	0	1.18392396		0.9799968	
680	0.15	0.17	39.1200006	1.645000093	0	REVERSED Partitioning defective 3 homolog OS=Rattus norvegicus GN=Pard3 PE=1 SV=1	RAT	0	1.183727026	0.339125603	0.98663038	0.914791286
249	3.06	3.06	38.46000135	16.92000031	16.92000031	Astrocytic phosphoprotein PEA-15 OS=Rattus norvegicus GN=Pea15 PE=1 SV=1	RAT	3	1.183663011	0.3417826	1.059959054	0.663943887
506	0.38	0.38	23.00000042	0.97190002	0	REVERSED Rap guanine nucleotide exchange factor 3 OS=Rattus norvegicus GN=Rapgef3 PE=2 SV=2	RAT	0	1.183447003		1.062999964	
873	0.08	0.09	32.69000053	0	0	Dual serine/threonine and tyrosine protein kinase OS=Rattus norvegicus GN=Dstyk PE=2 SV=1	RAT	0	1.182582974	0.193059802	1.131688952	0.639355481
367	1.63	1.63	31.36999905	10.77999994	10.77999994	Parathymosin OS=Rattus norvegicus GN=Ptms PE=1 SV=2	RAT	1	1.181632996		1.13954103	
1121	0.05	0.05	52.42000222	0	0	REVERSED Trifunctional enzyme subunit alpha, mitochondrial OS=Rattus norvegicus GN=Hadha PE=1 SV=2	RAT	0	1.181620002		1.039445996	
457	0.57	0.59	27.3999989	1.840000041	0	Mitochondrial-processing peptidase subunit beta OS=Rattus norvegicus GN=Pmpcb PE=1 SV=3	RAT	0	1.181318998		1.014330983	
720	0.13	0.14	30.55999875	6.509999931	0	Copine-9 OS=Rattus norvegicus GN=Cpne9 PE=2 SV=1	RAT	0	1.17919004	0.350625694	1.051246047	0.707714975
516	0.36	0.36	21.01999968	3.232999891	0	26S protease regulatory subunit 7 OS=Rattus norvegicus GN=Psmc2 PE=1 SV=3	RAT	0	1.176108956		0.843989372	
987	0.06	0.09	30.50000072	0	0	REVERSED RNA polymerase II-associated protein 1 OS=Rattus norvegicus GN=Rpap1 PE=2 SV=1	RAT	0	1.171450019	0.207788303	1.041321993	0.684738576
231	3.64	3.64	82.71999955	43.2099998	19.75000054	NEDD8 OS=Rattus norvegicus GN=Nedd8 PE=1 SV=1	RAT	3	1.168073058	0.514259219	1.052533031	0.613465011
1056	0.05	0.11	18.52999926	0.866699964	0	REVERSED Myosin-Vb OS=Rattus norvegicus GN=Myo5b PE=1 SV=1	RAT	0	1.166798949	0.465349704	1.049008012	0.786777377
509	0.37	0.42	31.63000047	2.716000006	0	Janus kinase and microtubule-interacting protein 1 OS=Rattus norvegicus GN=Jakmip1 PE=1 SV=1	RAT	0	1.166561961		1.065034986	
684	0.15	0.15	32.76999891	0.504700001	0	Teneurin-2 OS=Rattus norvegicus GN=Odz2 PE=2 SV=2	RAT	0	1.166542053	0.25167641	1.11432004	0.335729688
357	1.79	1.79	40.29999971	4.563000053	4.563000053	40S ribosomal protein S4, X isoform OS=Rattus norvegicus GN=Rps4x PE=2 SV=2	RAT	1	1.164105058	0.403306991	1.296386957	0.259378195

543	0.3	0.46	38.71999979	1.279000007	0	Neurabin-1 OS=Rattus norvegicus GN=Ppp1r9a PE=1 SV=1	RAT	0	1.156123996	0.381159514	1.044425011	0.779855311
75	14.68	14.68	92.94000268	69.70999837	57.64999986	Guanine nucleotide-binding protein G(I)/G(S)/G(T) subunit beta-1 OS=Rattus norvegicus GN=Gnb1 PE=1 SV=4	RAT	12	1.15545404	0.495663702	1.085162044	0.562011898
979	0.06	0.11	31.36999905	0	0	REVERSED Rho guanine nucleotide exchange factor 2 OS=Rattus norvegicus GN=Arhgef2 PE=2 SV=1	RAT	0	1.152398944	0.51129961	1.000671029	0.993391275
861	0.08	0.12	34.18000042	0	0	SNF-related serine/threonine-protein kinase OS=Rattus norvegicus GN=Snrk PE=1 SV=1	RAT	0	1.152346969	0.489030212	0.901485026	0.34609431
793	0.1	0.12	32.85000026	1.917999983	0	Sterile alpha motif domain-containing protein 14 OS=Rattus norvegicus GN=Samd14 PE=2 SV=1	RAT	0	1.152107954		1.221884966	
1038	0.06	0.06	20.0000003	0	0	REVERSED Deoxycytidine kinase OS=Rattus norvegicus GN=Dck PE=2 SV=1	RAT	0	1.150063038	0.393256903	0.900701582	0.483739108
544	0.3	0.31	28.52999866	4.20400016	0	REVERSED Homeobox protein Hox-A1 OS=Rattus norvegicus GN=Hoxa1 PE=2 SV=1	RAT	0	1.149701953		1.151975989	
256	2.89	2.89	83.13999772	7.987999916	7.987999916	Limbic system-associated membrane protein OS=Rattus norvegicus GN=Lsamp PE=1 SV=1	RAT	2	1.147251964	0.4001939	1.115144014	0.47288391
296	2.06	2.09	64.67000246	3.988999873	3.988999873	Tropomodulin-2 OS=Rattus norvegicus GN=Tmod2 PE=1 SV=1	RAT	1	1.146975994	0.410356492	0.989637911	0.94204253
56	18.24	18.24	92.51999855	64.56999779	53.53999734	Phosphoglycerate mutase 1 OS=Rattus norvegicus GN=Pgam1 PE=1 SV=4	RAT	23	1.14578402	0.126261696	1.03066802	0.568480074
490	0.42	0.83	28.83999944	0.743100001	0	REVERSED Cytoplasmic dynein 2 heavy chain 1 OS=Rattus norvegicus GN=Dync2h1 PE=1 SV=1	RAT	0	1.145645976		1.041640997	
191	5.15	5.19	58.96000266	9.453000128	9.453000128	Dynactin subunit 2 OS=Rattus norvegicus GN=Dctn2 PE=1 SV=1	RAT	3	1.145107985	0.260854304	0.994222283	0.953221083
784	0.1	0.14	37.09999919	1.854999922	0	Fragile X mental retardation protein 1 homolog OS=Rattus norvegicus GN=Fmr1 PE=2 SV=2	RAT	0	1.144101977		1.127974987	
317	2.01	2.01	54.90000248	8.06099996	4.357000068	NADH-ubiquinone oxidoreductase chain 4 OS=Rattus norvegicus GN=Mtnd4 PE=2 SV=2	RAT	1	1.143524051		1.517652988	
233	3.58	3.58	50.85999966	5.160000175	5.160000175	Cathepsin D OS=Rattus norvegicus GN=Ctsd PE=1 SV=1	RAT	2	1.142554998	0.64929539	0.965846419	0.786023319
733	0.12	0.14	33.4100008	1.116000023	0	Myb-binding protein 1A OS=Rattus norvegicus GN=Mybbp1a PE=2 SV=2	RAT	0	1.141111016		0.997455597	
754	0.11	0.4	21.86000049	0.088569999	0	REVERSED Dynein heavy chain 1, axonemal OS=Rattus norvegicus GN=Dnah1 PE=2 SV=2	RAT	0	1.139482975	0.168074593	1.098701954	0.284858495
595	0.22	0.22	32.48000145	0.457500014	0	REVERSED Neurexin-1-alpha OS=Rattus norvegicus GN=Nrxn1 PE=1 SV=3	RAT	0	1.139088035		0.99828881	
6	50.1	50.1	86.51999831	83.14999938	80.44999838	Tubulin beta-2A chain OS=Rattus norvegicus GN=Tubb2a PE=1 SV=1	RAT	88	1.138473988	0.180664107	1.066694021	0.371612489
1063	0.05	0.1	21.0800007	0	0	Heparanase OS=Rattus norvegicus GN=Hpse PE=2 SV=1	RAT	0	1.138025045	0.417184502	1.028265953	0.826058984
384	1.32	1.33	42.9399997	3.047000058	3.047000058	Septin-2 OS=Rattus norvegicus GN=Sept2 PE=1 SV=1	RAT	1	1.134847045		1.133312941	
356	1.8	1.8	48.64000082	12.06	12.06	Thioredoxin-dependent peroxide reductase, mitochondrial OS=Rattus norvegicus GN=Prdx3 PE=1 SV=2	RAT	1	1.133527994		1.03186202	
615	0.2	0.38	26.01999938	0.271699997	0	REVERSED Cadherin EGF LAG seven-pass G-type receptor 3 OS=Rattus norvegicus GN=Celsr3 PE=2 SV=1	RAT	0	1.13351202	0.45183751	1.071344018	0.490060002
596	0.22	0.22	34.31000113	0.912399963	0	REVERSED Inactive phospholipase C-like protein 1 OS=Rattus norvegicus GN=Plcl1 PE=1 SV=1	RAT	0	1.132524967		1.118538976	
60	17.4	17.4	95.24000287	95.24000287	86.39000058	Hemoglobin subunit beta-1 OS=Rattus norvegicus GN=Hbb PE=1 SV=3	RAT	14	1.132446051	0.109191202	1.100749016	0.107085302
208	4.21	4.21	55.9499979	9.911999851	9.911999851	Guanine deaminase OS=Rattus norvegicus GN=Gda PE=1 SV=1	RAT	3	1.132210016	0.36760959	1.070490956	0.608226776
626	0.2	0.2	15.81999958	7.909999788	0	Protein CutA OS=Rattus norvegicus GN=Cuta PE=1 SV=2	RAT	0	1.132099032		1.092414021	
624	0.2	0.2	31.81999922	2.405999973	0	REVERSED Ras-related GTP-binding protein B OS=Rattus norvegicus GN=RragB PE=1 SV=1	RAT	0	1.131677032		1.015285969	
832	0.09	0.1	42.64999926	0	0	60S ribosomal protein L15 OS=Rattus norvegicus GN=Rpl15 PE=1 SV=2	RAT	0	1.131543994	0.657038391	1.11016798	0.50186038
775	0.1	0.34	29.67000008	1.779999956	0	REVERSED Pannexin-2 OS=Rattus norvegicus GN=Panx2 PE=1 SV=2	RAT	0	1.131343007	0.4336254	1.000323057	0.997937977
800	0.1	0.1	31.86999857	1.820999943	0	REVERSED ATP-binding cassette sub-family D member 3 OS=Rattus norvegicus GN=Abcd3 PE=1 SV=3	RAT	0	1.130530953	0.584665	1.010743976	0.931820571
314	2.02	2.02	55.62999845	10.59999987	10.59999987	Myosin light polypeptide 6 OS=Rattus norvegicus GN=Myl6 PE=1 SV=3	RAT	1	1.130025983	0.433951706	1.013717055	0.913123608
916	0.07	0.1	35.04999876	1.027000044	0	REVERSED Radial spoke head 10 homolog B OS=Rattus norvegicus GN=Rsph10b PE=2 SV=1	RAT	0	1.129062057		1.480777979	
895	0.07	0.29	25.83000064	4.504999891	0	L-asparaginase OS=Rattus norvegicus GN=Asrgl1 PE=1 SV=1	RAT	0	1.128581047		0.936777294	
1101	0.05	0.06	34.1899991	0	0	REVERSED Nephrin OS=Rattus norvegicus GN=Nphs1 PE=1 SV=2	RAT	0	1.128204942	0.437123299	1.165884018	0.364260614
1014	0.06	0.07	36.77000105	5.161000043	0	REVERSED 28S ribosomal protein S10, mitochondrial OS=Rattus norvegicus GN=Mrps10 PE=2 SV=1	RAT	0	1.126929998		0.984076381	
462	0.55	0.64	33.97000134	3.325000033	2.37499997	UPF0415 protein C7orf25 homolog OS=Rattus norvegicus PE=2 SV=1	RAT	1	1.126306057		1.011036992	
929	0.07	0.09	17.5999999	0	0	Mesothelin OS=Rattus norvegicus GN=Msln PE=2 SV=2	RAT	0	1.125298023		1.039021969	
960	0.06	0.32	19.30000037	0.358700007	0	Kinesin-like protein KIF27 OS=Rattus norvegicus GN=Kif27 PE=2 SV=1	RAT	0	1.125270963		0.993181586	
1096	0.05	0.07	23.11999947	3.064000048	0	Tropomodulin-1 OS=Rattus norvegicus GN=Tmod1 PE=2 SV=1	RAT	0	1.125255942		1.072435021	
392	1.16	1.18	33.82000029	20.58999985	10.28999984	Guanine nucleotide-binding protein G(I)/G(S)/G(O) subunit gamma-7 OS=Rattus norvegicus GN=Gng7 PE=2 SV=2	RAT	1	1.125126004	0.685609818	1.207800031	0.563909888
655	0.17	0.17	76.96999907	7.272999734	0	Alanine and arginine-rich domain-containing protein OS=Rattus norvegicus GN=Aard PE=2 SV=1	RAT	0	1.123396993		1.037322044	
780	0.1	0.18	26.96999907	0	0	REVERSED Telomerase protein component 1 OS=Rattus norvegicus GN=Tep1 PE=1 SV=1	RAT	0	1.123255968	0.4475905	1.014309049	0.953065991
904	0.07	0.15	25.31000078	0.691600004	0	Voltage-dependent L-type calcium channel subunit alpha-1C OS=Rattus norvegicus GN=Cacna1c PE=1 SV=1	RAT	0	1.123128057		0.969796598	
364	1.69	1.78	63.41000199	1.788000017	1.23800002	Glycerol-3-phosphate dehydrogenase, mitochondrial OS=Rattus norvegicus GN=Gpd2 PE=1 SV=1	RAT	1	1.122094035	0.472341001	1.200386047	0.485462606
611	0.21	0.21	16.71999991	6.485000253	0	REVERSED Uncharacterized protein C12orf41 homolog OS=Rattus norvegicus PE=2 SV=1	RAT	0	1.121255994		1.033503056	
225	3.98	3.98	75.18000007	21.80999964	19.67999935	Dihydropyrimidinase-related protein 5 OS=Rattus norvegicus GN=Dpysl5 PE=1 SV=1	RAT	5	1.119686007	0.239670098	1.119794011	0.237280399
668	0.16	0.17	50	1.913999952	0	REVERSED Testis-specific H1 histone OS=Rattus norvegicus GN=H1fnt PE=2 SV=1	RAT	0	1.118996024	0.313765407	1.268038988	0.131734401
521	0.35	0.35	30.95999956	0.690399995	0	Rho GTPase-activating protein 27 OS=Rattus norvegicus GN=Arhgap27 PE=1 SV=1	RAT	0	1.117918015	0.245272994	0.943504214	0.506162584
650	0.17	0.22	27.86999941	2.246999927	0	REVERSED C-terminal-binding protein 2 OS=Rattus norvegicus GN=Ctbp2 PE=1 SV=2	RAT	0	1.117285967	0.469872713	1.126713991	0.448105305
847	0.09	0.09	16.30000025	3.762000054	0	Dehydrogenase/reductase SDR family member 9 OS=Rattus norvegicus GN=Dhrs9 PE=2 SV=1	RAT	0	1.117154002	0.47251451	0.94903928	0.696174026
206	4.28	4.28	76.09000206	18.4799999	14.12999928	Lactoylglutathione lyase OS=Rattus norvegicus GN=Glo1 PE=1 SV=3	RAT	2	1.116716027	0.235266507	0.944250703	0.499147207
156	6.88	19.36	75.85999966	18.82999986	16.32999927	Plasma membrane calcium-transporting ATPase 2 OS=Rattus norvegicus GN=Atp2b2 PE=2 SV=2	RAT	13	1.116567969	0.714793682	0.992555499	0.960498929
820	0.09	0.12	19.30000037	1.228000037	0	Interleukin-1 receptor accessory protein OS=Rattus norvegicus GN=Il1rap PE=2 SV=1	RAT	0	1.116345048		0.985104322	
433	0.68	0.68	12.03000024	3.773999959	3.773999959	REVERSED ATP-sensitive inward rectifier potassium channel 8 OS=Rattus norvegicus GN=Kcnj8 PE=2 SV=1	RAT	1	1.115749002	0.311208606	1.028630018	0.762393415
244	3.22	3.24	51.77000165	10.62000021	9.291999787	Isocitrate dehydrogenase [NADP], mitochondrial OS=Rattus norvegicus GN=Idh2 PE=1 SV=2	RAT	3	1.114771008	0.362544596	0.994195819	0.950453877
919	0.07	0.1	31.34999871	0	0	Kinesin-like protein KIF22 OS=Rattus norvegicus GN=Kif22 PE=2 SV=1	RAT	0	1.114369988	0.416914999	1.061687946	0.440403402
997	0.06	0.08	29.67999876	0	0	REVERSED Tuberin OS=Rattus norvegicus GN=Tsc2 PE=1 SV=1	RAT	0	1.113584042	0.321348906	1.082651019	0.436677903
530	0.33	0.44	28.7499994	1.094000041	0	Dynactin subunit 1 OS=Rattus norvegicus GN=Dctn1 PE=2 SV=2	RAT	0	1.112987041	0.597985327	1.06901598	0.623862982
536	0.32	0.38	54.03000116	2.685000002	0	REVERSED ADP/ATP translocase 2 OS=Rattus norvegicus GN=Slc25a5 PE=1 SV=3	RAT	0	1.112969041		1.056274056	
586	0.23	0.23	36.23000085	1.479000039	0	T-complex protein 1 subunit epsilon OS=Rattus norvegicus GN=Cct5 PE=1 SV=1	RAT	0	1.112884045	0.525404274	1.067543983	0.712467372
833	0.09	0.1	20.0000003	3.158000112	0	Flap endonuclease 1 OS=Rattus norvegicus GN=Fen1 PE=2 SV=1	RAT	0	1.112763047		1.107591987	
161	6.7	6.71	100	42.3999995	34.40000117	Histone H2B type 1 OS=Rattus norvegicus PE=1 SV=2	RAT	4	1.112501979	0.344904214	1.011469007	0.907196701
331	2	2	57.48000145	5.139999837	5.139999837	60S ribosomal protein L10 OS=Rattus norvegicus GN=Rpl10 PE=1 SV=3	RAT	1	1.111083031		1.166182995	
562	0.26	0.26	29.5599997	0.780500006	0	Dihydropyrimidine dehydrogenase [NADP+] OS=Rattus norvegicus GN=Dpyd PE=2 SV=1	RAT	0	1.110955954	0.667987525	1.081472993	0.574907005
613	0.2	3.81	77.56000161	22.43999988	10.72999984	Ras-related protein Rab-1A OS=Rattus norvegicus GN=Rab1A PE=1 SV=3	RAT	2	1.110885024	0.337100387	1.096791029	0.38630259
884	0.08	0.08	41.62999988	0	0	REVERSED Serine/arginine-rich splicing factor 2 OS=Rattus norvegicus GN=Srsf2 PE=1 SV=3	RAT	0	1.109930992	0.482981086	1.161649942	0.371196091
951	0.07	0.07	26.06000006	0	0	Long-chain-fatty-acid--CoA ligase 5 OS=Rattus norvegicus GN=Acsl5 PE=2 SV=1	RAT	0	1.107555032	0.241470203	0.950959682	0.626716495
612	0.21	0.21	2.848000079	2.848000079	0	POU domain, class 2, transcription factor 1 (Fragment) OS=Rattus norvegicus GN=Pou2f1 PE=2 SV=2	RAT	0	1.106927991		0.90307337	
777	0.1	0.27	26.94000006	0.172099995	0	Myomegalin OS=Rattus norvegicus GN=Pde4dip PE=1 SV=1	RAT	0	1.106235027	0.494735986	1.018478036	0.884039223
989	0.06	0.09	21.33000046	1.243999973	0	REVERSED Telomerase reverse transcriptase OS=Rattus norvegicus GN=Tert PE=2 SV=1	RAT	0	1.105391979		0.9867751	
252	2.93	3.04	54.60000038	9.194000065	5.232999846	Drebrin OS=Rattus norvegicus GN=Dbn1 PE=2 SV=3	RAT	2	1.104765058	0.707124472	0.976033509	0.848306417
1055	0.05	0.12	34.61999893	0	0	Protogenin OS=Rattus norvegicus GN=Prtg PE=2 SV=1	RAT	0	1.102373004	0.504920185	1.062533021	0.649877429
1120	0.05	0.05	71.53000236	0	0	REVERSED Creatine kinase U-type, mitochondrial OS=Rattus norvegicus GN=Ckmt1 PE=1 SV=1	RAT	0	1.101819038	0.367310613	0.878479421	0.253426611

1076	0.05	0.08	10.14999971	1.212000009	0	REVERSED Arginyl-tRNA synthetase, cytoplasmic OS=Rattus norvegicus GN=Rars PE=1 SV=2	RAT	0	1.100991964	0.545768678	0.956979811	0.831073225
758	0.11	0.14	26.28000081	0.320499996	0	Ral GTPase-activating protein subunit alpha-2 OS=Rattus norvegicus GN=Ralgapa2 PE=1 SV=1	RAT	0	1.100209951		0.99858582	
429	0.7	0.7	39.41999972	5.076000094	5.076000094	Calnexin OS=Rattus norvegicus GN=Canx PE=1 SV=1	RAT	2	1.100154996	0.514210284	0.886678576	0.441286594
628	0.19	12.83	99.32000041	85.0300014	70.0699985	Hemoglobin subunit beta-2 OS=Rattus norvegicus PE=1 SV=2	RAT	9	1.100093007	0.374814093	0.988237917	0.900166214
128	8.62	12.39	82.06999898	36.71999872	36.71999872	Elongation factor 1-alpha 2 OS=Rattus norvegicus GN=Eef1a2 PE=2 SV=1	RAT	20	1.099259973	0.291807085	1.043869019	0.846475601
54	19.07	38.35	86.66999936	64.88999724	58.89000297	Tubulin beta-3 chain OS=Rattus norvegicus GN=Tubb3 PE=1 SV=1	RAT	87	1.098664045	0.351831496	1.066616058	0.681060612
804	0.1	0.1	36.10000014	5.393999815	0	Chloride intracellular channel protein 1 OS=Rattus norvegicus GN=Clic1 PE=1 SV=1	RAT	0	1.098644018		1.182549953	
1060	0.05	0.1	33.01999867	0	0	Double-stranded RNA-specific adenosine deaminase OS=Rattus norvegicus GN=Adar PE=2 SV=1	RAT	0	1.098512053		1.038400054	
138	8.09	8.12	53.90999913	9.97299999	7.547000051	Synaptic vesicle glycoprotein 2A OS=Rattus norvegicus GN=Sv2a PE=1 SV=2	RAT	4	1.098384976	0.422472596	1.11233604	0.650308371
678	0.15	0.23	30.23000062	0.716000004	0	REVERSED Phosphatidylinositol-3,4,5-trisphosphate 5-phosphatase 2 OS=Rattus norvegicus GN=Inppl1 PE=1 SV=1	RAT	0	1.097548008	0.370613486	1.068161964	0.502160907
430	0.7	0.7	16.67000055	3.736000136	3.736000136	Neuronal growth regulator 1 OS=Rattus norvegicus GN=Negr1 PE=1 SV=1	RAT	1	1.095777988		1.271141052	
556	0.27	0.3	29.84000146	0.578799983	0.578799983	Regulating synaptic membrane exocytosis protein 2 OS=Rattus norvegicus GN=Rims2 PE=1 SV=1	RAT	1	1.095479965	0.380187899	1.052302957	0.596172214
870	0.08	0.1	34.2200011	0	0	Cytochrome P450 2F2 OS=Rattus norvegicus GN=Cyp2f2 PE=2 SV=1	RAT	0	1.095414042	0.525772512	1.15377295	0.384900987
389	1.2	1.2	24.33000058	4.752999917	2.66200006	Amine oxidase [flavin-containing] A OS=Rattus norvegicus GN=Maoa PE=1 SV=1	RAT	1	1.095396996	0.52934128	0.979257584	0.868534625
914	0.07	0.1	32.13	0.733300019	0	REVERSED Carbamoyl-phosphate synthase [ammonia], mitochondrial OS=Rattus norvegicus GN=Cps1 PE=1 SV=1	RAT	0	1.095067024		0.978665471	
345	2	2	33.86000097	12.6000002	12.6000002	Nuclear transport factor 2 OS=Rattus norvegicus GN=Nutf2 PE=1 SV=1	RAT	1	1.094769001		0.977440476	
479	0.47	0.55	29.89000082	4.214999825	0	REVERSED Paraspeckle component 1 OS=Rattus norvegicus GN=Pspc1 PE=2 SV=1	RAT	0	1.094030976	0.532622218	0.943812728	0.729192972
999	0.06	0.08	33.16000104	0	0	Receptor-type tyrosine-protein phosphatase-like N OS=Rattus norvegicus GN=Ptprn PE=2 SV=2	RAT	0	1.093108058	0.534728587	1.059970021	0.702706277
972	0.06	0.16	26.48000121	1.673999988	0	REVERSED Histidine ammonia-lyase OS=Rattus norvegicus GN=Hal PE=1 SV=1	RAT	0	1.092774987	0.555579007	1.284952998	0.474188209
1041	0.06	0.06	8.771999925	5.847999826	0	Neudesin OS=Rattus norvegicus GN=Nenf PE=2 SV=1	RAT	0	1.092254043		1.128872037	
210	4.14	8.66	77.20000148	9.976000339	7.362999767	Glycogen phosphorylase, muscle form OS=Rattus norvegicus GN=Pygm PE=2 SV=5	RAT	8	1.090963006	0.319119304	1.046875954	0.576855123
839	0.09	0.09	29.8299998	1.255999971	0	REVERSED ATP-dependent DNA helicase PIF1 OS=Rattus norvegicus GN=Pif1 PE=2 SV=1	RAT	0	1.090173006	0.545104027	0.990789771	0.941050828
286	2.14	2.15	89.63999748	41.8900013	41.8900013	Superoxide dismutase [Mn], mitochondrial OS=Rattus norvegicus GN=Sod2 PE=1 SV=2	RAT	4	1.090121984	0.543505788	1.147629976	0.396575689
300	2.05	2.06	67.91999936	5.895999819	5.895999819	Acetyl-CoA acetyltransferase, mitochondrial OS=Rattus norvegicus GN=Acat1 PE=1 SV=1	RAT	2	1.089501977	0.546116889	0.996498227	0.977460325
477	0.49	0.49	12.16999963	7.825999707	0	Macrophage migration inhibitory factor OS=Rattus norvegicus GN=Mif PE=1 SV=4	RAT	0	1.089488029		0.983302116	
1134	0.05	0.05	24.31000024	0	0	REVERSED Cannabinoid receptor 1 OS=Rattus norvegicus GN=Cnr1 PE=1 SV=1	RAT	0	1.089387059		1.033540964	
435	0.67	0.68	21.38999999	1.25099998	1.25099998	Metabotropic glutamate receptor 3 OS=Rattus norvegicus GN=Grm3 PE=1 SV=1	RAT	1	1.089380026	0.546311915	1.043450952	0.742743313
865	0.08	0.11	21.78000063	5.940999836	0	Endothelin-1 OS=Rattus norvegicus GN=Edn1 PE=1 SV=2	RAT	0	1.088896036		0.908577323	
910	0.07	0.12	65.71000218	2.449000068	0	REVERSED 14-3-3 protein theta OS=Rattus norvegicus GN=Ywhaq PE=1 SV=1	RAT	0	1.088896036		0.908577323	
949	0.07	0.07	38.35999966	3.438999876	0	1-phosphatidylinositol-4,5-bisphosphate phosphodiesterase delta-1 OS=Rattus norvegicus GN=Plcd1 PE=1 SV=1	RAT	0	1.088896036		0.908577323	
877	0.08	0.09	24.25000072	2.65800003	0	REVERSED Ribonuclease H2 subunit A OS=Rattus norvegicus GN=Rnaseh2a PE=2 SV=1	RAT	0	1.088799	0.553527474	0.988441586	0.926691711
908	0.07	0.13	27.75000036	0	0	Protein midA homolog, mitochondrial OS=Rattus norvegicus PE=2 SV=1	RAT	0	1.088683009	0.550115883	0.997289121	0.982597172
990	0.06	0.09	28.85999978	1.023999974	0	REVERSED Disks large-associated protein 3 OS=Rattus norvegicus GN=Dlgap3 PE=1 SV=2	RAT	0	1.087890029		1.126132965	
139	8.07	10.55	80.52999973	13.38	9.272000194	Dynamin-1-like protein OS=Rattus norvegicus GN=Dnm1l PE=1 SV=1	RAT	6	1.087826014	0.43948549	1.052291036	0.6841712
78	14.02	14.05	98.58999848	83.79999995	71.13000154	Hemoglobin subunit alpha-1/2 OS=Rattus norvegicus GN=Hba1 PE=1 SV=3	RAT	23	1.087563992	0.179292798	1.020215034	0.729536474
116	9.89	9.9	80.58999777	72.35000134	59.40999985	Calcineurin subunit B type 1 OS=Rattus norvegicus GN=Ppp3r1 PE=1 SV=2	RAT	5	1.087481022	0.249259293	1.073174	0.484264612
1017	0.06	0.07	5.234000087	1.652999967	0	Glycoprotein-N-acetylgalactosamine 3-beta-galactosyltransferase 1 OS=Rattus norvegicus GN=C1galt1 PE=1 SV=1	RAT	0	1.086227059		1.020290971	
874	0.08	0.09	30.05000055	0.930900034	0	REVERSED ATP-binding cassette sub-family B member 7, mitochondrial OS=Rattus norvegicus GN=Abcb7 PE=2 SV=1	RAT	0	1.086065054		1.059916019	
763	0.11	0.13	30.48000038	2.380999923	0	Pre-mRNA-splicing factor RBM22 OS=Rattus norvegicus GN=Rbm22 PE=2 SV=1	RAT	0	1.085860968		1.053940058	
689	0.15	0.15	30.16000092	11.11000031	0	Histidine triad nucleotide-binding protein 1 OS=Rattus norvegicus GN=Hint1 PE=1 SV=5	RAT	0	1.08543396		1.14360702	
226	3.92	3.92	67.58999825	25	25	Peptidyl-prolyl cis-trans isomerase FKBP1A OS=Rattus norvegicus GN=Fkbp1a PE=2 SV=3	RAT	2	1.085222006	0.560112298	1.036383033	0.779271722
549	0.28	0.32	20.09000033	0.172499998	0	Dynein heavy chain 7, axonemal OS=Rattus norvegicus GN=Dnah7 PE=2 SV=2	RAT	0	1.084280968	0.346819013	1.022681952	0.777828574
669	0.16	0.17	25.99999905	5.736000091	0	Succinate-semialdehyde dehydrogenase, mitochondrial OS=Rattus norvegicus GN=Aldh5a1 PE=1 SV=2	RAT	0	1.084184051	0.817793012	1.046488047	0.841280222
1000	0.06	0.08	45.82999945	0	0	Apolipoprotein E OS=Rattus norvegicus GN=Apoe PE=1 SV=2	RAT	0	1.084025025	0.630513608	1.025941014	0.841612279
513	0.36	2.96	72.81000018	19.47000027	9.122999758	Dihydropyrimidinase-related protein 3 OS=Rattus norvegicus GN=Dpysl3 PE=1 SV=2	RAT	4	1.083891988	0.439742595	0.971981525	0.827130079
588	0.23	0.23	14.94999975	4.298999906	0	EH domain-containing protein 3 OS=Rattus norvegicus GN=Ehd3 PE=1 SV=2	RAT	0	1.083889961		1.003666997	
1016	0.06	0.07	9.649000317	2.129999921	0	REVERSED PR domain zinc finger protein 4 OS=Rattus norvegicus GN=Prdm4 PE=2 SV=1	RAT	0	1.08357501	0.582030773	1.11628902	0.48149699
212	4.12	4.12	62.99999952	11.9099997	8.323000371	Catenin beta-1 OS=Rattus norvegicus GN=Ctnnb1 PE=1 SV=1	RAT	3	1.083495021	0.44307521	1.059622049	0.563257277
224	4	4.02	72.93000221	9.235999733	9.235999733	Mitochondrial 2-oxoglutarate/malate carrier protein OS=Rattus norvegicus GN=Slc25a11 PE=2 SV=3	RAT	2	1.081771016	0.572461903	1.088212013	0.54961139
802	0.1	0.1	32.91999996	1.875000075	0	Potassium voltage-gated channel subfamily G member 2 OS=Rattus norvegicus GN=Kcng2 PE=2 SV=1	RAT	0	1.081333995	0.461883903	1.036190987	0.801397979
130	8.44	8.44	74.00000095	20.08000016	13.19999993	NADH-ubiquinone oxidoreductase 75 kDa subunit, mitochondrial OS=Rattus norvegicus GN=Ndufs1 PE=1 SV=1	RAT	5	1.080867052	0.31967321	1.071470976	0.623002887
707	0.13	0.38	41.94000065	0.767999981	0	REVERSED Coiled-coil domain-containing protein 81 OS=Rattus norvegicus GN=Ccdc81 PE=2 SV=1	RAT	0	1.080809951	0.437659115	1.045243979	0.639399171
756	0.11	0.17	34.24000144	0.953000039	0	REVERSED Synaptojanin-1 OS=Rattus norvegicus GN=Synj1 PE=1 SV=3	RAT	0	1.080654025	0.576561391	0.90430671	0.49461621
173	6.07	6.18	55.41999936	5.033000186	4.157999903	Myosin-Va OS=Rattus norvegicus GN=Myo5a PE=2 SV=1	RAT	4	1.080530047	0.44815129	0.910609484	0.373324394
1135	0.05	0.05	30.32000065	2.445000038	0	REVERSED Z-DNA-binding protein 1 OS=Rattus norvegicus GN=Zbp1 PE=2 SV=1	RAT	0	1.079224944		1.19739902	
377	1.41	1.41	43.65999997	18.31	18.31	Glia maturation factor beta OS=Rattus norvegicus GN=Gmfb PE=1 SV=2	RAT	1	1.07880497	0.451594114	1.157825947	0.215897396
385	1.29	1.29	24.26999956	1.248000003	1.248000003	Far upstream element-binding protein 2 OS=Rattus norvegicus GN=Khsrp PE=1 SV=1	RAT	1	1.078750968	0.583455682	0.943512022	0.661452115
662	0.16	0.2	37.97000051	0	0	REVERSED Adenomatous polyposis coli protein OS=Rattus norvegicus GN=Apc PE=1 SV=1	RAT	0	1.078570962	0.297537804	1.020967007	0.758349001
459	0.56	0.61	23.78000021	3.909000009	0	REVERSED Hepatitis A virus cellular receptor 1 homolog OS=Rattus norvegicus GN=Havcr1 PE=2 SV=1	RAT	0	1.078379035	0.588358223	1.072024941	0.75344038
192	5.13	5.13	77.13999748	56.19000196	49.52000082	Cytochrome c, somatic OS=Rattus norvegicus GN=Cycs PE=1 SV=2	RAT	6	1.078354001	0.26699689	0.997928977	0.973960876
664	0.16	0.19	41.06999934	1.785999909	0	Formin-binding protein 1 OS=Rattus norvegicus GN=Fnbp1 PE=1 SV=2	RAT	0	1.078300953		0.980414927	
876	0.08	0.09	56.98999763	6.735999882	0	Protein-tyrosine phosphatase mitochondrial 1 OS=Rattus norvegicus GN=Ptpmt1 PE=2 SV=1	RAT	0	1.078148007		1.066406965	
271	2.5	2.5	51.56000257	5.778000131	5.778000131	Protein tweety homolog 1 OS=Rattus norvegicus GN=Ttyh1 PE=2 SV=1	RAT	2	1.078099012	0.58660382	0.994079709	0.976334214
120	9.78	9.78	100	52.42999792	31.06999993	Histone H4 OS=Rattus norvegicus GN=Hist1h4b PE=1 SV=2	RAT	8	1.077383995	0.226158202	1.001103044	0.984715223
283	2.19	2.24	70.28999925	7.427000254	3.448000178	NADH dehydrogenase [ubiquinone] 1 alpha subcomplex subunit 9, mitochondrial OS=Rattus norvegicus GN=Ndufa9 PE=1 SV=2	RAT	1	1.077214956	0.641860604	1.107519984	0.852450013
115	10.01	10.04	66.92000031	7.336000353	7.336000353	Reticulon-1 OS=Rattus norvegicus GN=Rtn1 PE=2 SV=1	RAT	5	1.077155948	0.364358604	1.110818982	0.277404398
127	8.73	47.98	86.51999831	82.46999979	80.44999838	Tubulin beta-2C chain OS=Rattus norvegicus GN=Tubb2c PE=1 SV=1	RAT	91	1.076949954		1.055860996	
1109	0.05	0.06	40.29999971	0	0	REVERSED Induced myeloid leukemia cell differentiation protein Mcl-1 homolog OS=Rattus norvegicus GN=Mcl1 PE=1 SV=2	RAT	0	1.076900959	0.379139811	1.060207963	0.474748611
303	2.04	2.07	74.47999716	1.04700001	1.04700001	Membrane-associated phosphatidylinositol transfer protein 1 OS=Rattus norvegicus GN=Pitpnm1 PE=2 SV=1	RAT	1	1.076693058	0.456845194	0.997959614	0.982110977
144	7.62	7.62	80.00000119	19.17999983	19.17999983	Myelin-oligodendrocyte glycoprotein OS=Rattus norvegicus GN=Mog PE=1 SV=1	RAT	4	1.076478004	0.398983687	1.039955974	0.594742417
86	12.71	12.78	75.63999891	38.20999861	32.82000124	Pyruvate dehydrogenase E1 component subunit alpha, somatic form, mitochondrial OS=Rattus norvegicus GN=Pdha1 PE=1 SV=2	RAT	7	1.076192975	0.590268016	1.084210992	0.223163903
848	0.09	0.09	12.25999966	4.515999928	0	28S ribosomal protein S10, mitochondrial OS=Rattus norvegicus GN=Mrps10 PE=2 SV=1	RAT	0	1.075721025		1.015849948	
265	2.68	2.79	32.8700006	16.32000059	10.33999994	Cell adhesion molecule 2 OS=Rattus norvegicus GN=Cadm2 PE=1 SV=2	RAT	3	1.075585008	0.559620202	1.115337014	0.311252594
772	0.11	0.11	23.63000065	2.305000089	0	REVERSED Microfibril-associated glycoprotein 3 OS=Rattus norvegicus GN=Mfap3 PE=2 SV=1	RAT	0	1.074898005		1.07198596	

725	0.12	6.45	67.04000235	22.82000035	14.65000063	Guanine nucleotide-binding protein G(i) subunit alpha-2 OS=Rattus norvegicus GN=Gnai2 PE=1 SV=3	RAT	4	1.074671984		1.100074053	
431	0.68	0.7	12.09999993	0.466800015	0	REVERSED SCO-spondin OS=Rattus norvegicus GN=Sspo PE=2 SV=1	RAT	0	1.074344993	0.601065814	1.033503056	0.795157015
73	14.99	15.3	70.09000182	16.59000069	9.588000178	Sarcoplasmic/endoplasmic reticulum calcium ATPase 2 OS=Rattus norvegicus GN=Atp2a2 PE=1 SV=1	RAT	7	1.074197054	0.106979601	1.090111971	0.092381597
16	41.34	41.43	99.23999906	68.23999882	63.8899982	ATP synthase subunit beta, mitochondrial OS=Rattus norvegicus GN=Atp5b PE=1 SV=2	RAT	51	1.073637009	0.063633919	1.041553974	0.271015286
427	0.7	0.75	43.20000112	1.941999979	1.941999979	DnaJ homolog subfamily A member 2 OS=Rattus norvegicus GN=Dnaja2 PE=2 SV=1	RAT	1	1.072836995		1.099629045	
122	9.56	9.56	86.10000014	81.81999922	63.09999824	Phosphatidylethanolamine-binding protein 1 OS=Rattus norvegicus GN=Pebp1 PE=1 SV=3	RAT	14	1.072649002	0.247013897	1.031713009	0.588456094
1008	0.06	0.07	33.98000002	0	0	Centrosomal protein of 95 kDa OS=Rattus norvegicus GN=Cep95 PE=2 SV=1	RAT	0	1.072533965		0.98290962	
337	2	2	51.05999708	10.63999981	10.63999981	PRA1 family protein 3 OS=Rattus norvegicus GN=Arl6ip5 PE=1 SV=1	RAT	1	1.072150946		1.124089956	
766	0.11	0.12	39.80000019	3.234000131	0	REVERSED Uncharacterized protein C1orf177 homolog OS=Rattus norvegicus PE=2 SV=1	RAT	0	1.072036982		0.881194413	
857	0.08	0.15	31.38000071	0	0	REVERSED WD repeat-containing protein 6 OS=Rattus norvegicus GN=Wdr6 PE=1 SV=2	RAT	0	1.07149601	0.482844412	0.909569025	0.36211279
1058	0.05	0.1	25.69999993	0	0	Serine/threonine-protein kinase mTOR OS=Rattus norvegicus GN=Mtor PE=1 SV=1	RAT	0	1.071369052	0.63650912	0.990283072	0.919565022
936	0.07	0.08	33.6800009	0	0	REVERSED Potassium voltage-gated channel subfamily H member 1 OS=Rattus norvegicus GN=Kcnh1 PE=1 SV=1	RAT	0	1.071241975	0.493210286	1.01659596	0.87563318
172	6.1	6.14	67.80999899	13.97999972	10.02999991	Heterogeneous nuclear ribonucleoprotein A3 OS=Rattus norvegicus GN=Hnrnpa3 PE=1 SV=1	RAT	3	1.070325017	0.4063088	1.000020027	1
1105	0.05	0.06	23.3799994	1.264000032	0	REVERSED ATP-dependent Clp protease ATP-binding subunit clpX-like, mitochondrial OS=Rattus norvegicus GN=Clpx PE=2 SV=1	RAT	0	1.070247054		1.12004602	
671	0.16	0.16	39.10999894	1.593999937	0	Coiled-coil and C2 domain-containing protein 1A OS=Rattus norvegicus GN=Cc2d1a PE=2 SV=2	RAT	0	1.069229007	0.626492202	0.998077691	0.989138603
794	0.1	0.11	19.17999983	1.099000033	0	REVERSED Phosphatidylinositol 4,5-bisphosphate 5-phosphatase A OS=Rattus norvegicus GN=Inpp5j PE=1 SV=1	RAT	0	1.068881035	0.624103725	1.036885023	0.778315127
276	2.34	2.37	57.35999942	11.28999963	2.377000079	Annexin A6 OS=Rattus norvegicus GN=Anxa6 PE=1 SV=2	RAT	1	1.06854105	0.369719803	1.021744967	0.758397818
1089	0.05	0.07	23.29999954	0.459800009	0	REVERSED Contactin-associated protein like 5-1 OS=Rattus norvegicus GN=Cntnap5a PE=2 SV=1	RAT	0	1.067855		0.930919886	
881	0.08	0.08	20.35000026	0.413599983	0	Calcium-activated potassium channel subunit alpha-1 OS=Rattus norvegicus GN=Kcnma1 PE=1 SV=3	RAT	0	1.067837		0.985624373	
243	3.23	3.23	73.90999794	21.73999995	13.59000057	Ras-related protein Rap-1A OS=Rattus norvegicus GN=Rap1a PE=1 SV=1	RAT	2	1.067626953	0.6118595	1.103315949	0.369675785
739	0.12	0.13	39.01000023	3.5459999	0	Uncharacterized protein C1orf131 homolog OS=Rattus norvegicus PE=2 SV=1	RAT	0	1.067625999		0.972894371	
358	1.77	1.77	16.89999998	6.103000045	6.103000045	REVERSED CDP-diacylglycerol--inositol 3-phosphatidyltransferase OS=Rattus norvegicus GN=Cdipt PE=1 SV=1	RAT	1	1.066998959		1.002941012	
970	0.06	0.17	18.67000014	0	0	REVERSED Nuclear receptor coactivator 3 (Fragment) OS=Rattus norvegicus GN=Ncoa3 PE=2 SV=1	RAT	0	1.066527009	0.770167828	1.067774057	0.517568111
184	5.63	5.63	77.60999799	41.04000032	41.04000032	Complexin-1 OS=Rattus norvegicus GN=Cplx1 PE=1 SV=1	RAT	5	1.066503048	0.632415712	0.947683513	0.682965517
202	4.4	4.4	51.71999931	17.15999991	14.86999989	Elongation factor 1-gamma OS=Rattus norvegicus GN=Eef1g PE=1 SV=3	RAT	3	1.066388965	0.386946589	1.045513034	0.53763622
1082	0.05	0.07	33.61000121	1.659999974	0	REVERSED Protein timeless homolog OS=Rattus norvegicus GN=Timeless PE=1 SV=1	RAT	0	1.065870047		1.133939981	
91	12.44	12.44	82.26000071	23.95000011	18.97999942	Transitional endoplasmic reticulum ATPase OS=Rattus norvegicus GN=Vcp PE=1 SV=3	RAT	7	1.064700007	0.513347924	1.070523977	0.208319306
460	0.56	0.58	19.15999949	2.199999988	0	Voltage-dependent calcium channel subunit alpha-2/delta-1 OS=Rattus norvegicus GN=Cacna2d1 PE=1 SV=1	RAT	0	1.063838959		1.08633399	
318	2.01	2.01	72.47999907	5.045999959	5.045999959	Hypoxanthine-guanine phosphoribosyltransferase OS=Rattus norvegicus GN=Hprt1 PE=1 SV=1	RAT	1	1.063138008		0.997452676	
918	0.07	0.1	34.09000039	1.331999991	0	Elongation factor G, mitochondrial OS=Rattus norvegicus GN=Gfm1 PE=1 SV=2	RAT	0	1.062677979	0.651699126	1.06811595	0.62882477
524	0.35	0.35	26.01000071	5.093999952	0	Probable G-protein coupled receptor 173 OS=Rattus norvegicus GN=Gpr173 PE=2 SV=1	RAT	0	1.061766982		1.116263986	
1001	0.06	0.08	18.42000037	0	0	Interphotoreceptor matrix proteoglycan 1 OS=Rattus norvegicus GN=Impg1 PE=2 SV=1	RAT	0	1.061635971	0.654506505	1.105607986	0.631469607
28	30.93	31.56	96.49999738	69.41000223	57.8700006	Dihydropyrimidinase-related protein 2 OS=Rattus norvegicus GN=Dpysl2 PE=1 SV=1	RAT	36	1.061480045	0.3051292	1.011307001	0.798336089
1047	0.05	0.19	33.25000107	0.39400002	0	REVERSED Protein strawberry notch homolog 1 OS=Rattus norvegicus GN=Sbno1 PE=2 SV=2	RAT	0	1.061359048	0.537917078	1.044566989	0.660707712
101	11.24	11.24	100	45.21999955	45.21999955	Prohibitin OS=Rattus norvegicus GN=Phb PE=1 SV=1	RAT	8	1.061009049	0.418458015	0.983702481	0.8161695
520	0.35	0.41	22.99000025	4.214999825	0	Calbindin OS=Rattus norvegicus GN=Calb1 PE=1 SV=2	RAT	0	1.060894966		0.977442384	
719	0.13	0.14	31.90999925	0.839599967	0	Structural maintenance of chromosomes protein 3 OS=Rattus norvegicus GN=Smc3 PE=1 SV=1	RAT	0	1.060603023	0.593375087	1.183748007	0.174940795
888	0.08	0.08	24.34999943	4.662999883	0	UPF0671 protein C14orf153 homolog OS=Rattus norvegicus PE=2 SV=1	RAT	0	1.060577989		0.931135893	
227	3.79	3.82	59.50999856	21.24000043	17.03999937	Elongation factor Tu, mitochondrial OS=Rattus norvegicus GN=Tufm PE=1 SV=1	RAT	6	1.060461044	0.415753186	1.050724983	0.434477299
582	0.23	0.37	44.72000003	9.888000041	0	Rab GDP dissociation inhibitor beta OS=Rattus norvegicus GN=Gdi2 PE=1 SV=2	RAT	0	1.060451031	0.662828922	1.134233952	0.428583413
100	11.3	11.3	88.59000206	43.18000078	25.49999952	Rab GDP dissociation inhibitor alpha OS=Rattus norvegicus GN=Gdi1 PE=1 SV=1	RAT	13	1.060274005	0.306425303	1.024608016	0.66176182
66	16.3	16.31	76.84000134	54.79999781	46.32999897	Guanine nucleotide-binding protein G(o) subunit alpha OS=Rattus norvegicus GN=Gnao1 PE=1 SV=2	RAT	15	1.06022501	0.315961599	1.041368961	0.477082908
1093	0.05	0.07	44.69000101	0	0	H/ACA ribonucleoprotein complex subunit 1 OS=Rattus norvegicus GN=Gar1 PE=2 SV=1	RAT	0	1.059793949	0.669941485	1.121798038	0.489242494
762	0.11	0.13	33.05999935	1.365999971	0	Acylamino-acid-releasing enzyme OS=Rattus norvegicus GN=Apeh PE=1 SV=1	RAT	0	1.059335947	0.572703421	0.955341816	0.648128271
370	1.6	1.6	40.34000039	12.61000037	12.61000037	40S ribosomal protein S20 OS=Rattus norvegicus GN=Rps20 PE=3 SV=1	RAT	1	1.059106946		1.128818035	
438	0.66	0.67	48.89999926	2.707000077	0	REVERSED ATPase family AAA domain-containing protein 3 OS=Rattus norvegicus GN=Atad3 PE=2 SV=1	RAT	0	1.058887959	0.555836797	0.899228275	0.322339892
899	0.07	0.17	33.59000087	0	0	REVERSED Myosin-IXa OS=Rattus norvegicus GN=Myo9a PE=1 SV=1	RAT	0	1.058837056	0.478262395	1.077383041	0.375175208
1114	0.05	0.06	18.42000037	3.158000112	0	REVERSED 39S ribosomal protein L38, mitochondrial OS=Rattus norvegicus GN=Mrpl38 PE=2 SV=2	RAT	0	1.05863595		0.827631414	
475	0.49	0.49	30.50999939	0.906299986	0	Hypoxia-inducible factor 3-alpha OS=Rattus norvegicus GN=Hif3a PE=1 SV=1	RAT	0	1.058513999	0.676491022	0.983616173	0.914368272
57	18.22	18.22	71.56999707	22.21000046	20.55999935	6-phosphofructokinase type C OS=Rattus norvegicus GN=Pfkp PE=1 SV=2	RAT	13	1.058166981	0.346315593	0.994092882	0.894311428
445	0.64	0.64	25.06999969	0.539099984	0	Cyclin-dependent kinase 12 OS=Rattus norvegicus GN=Cdk12 PE=1 SV=1	RAT	0	1.058027983	0.435072094	1.028728008	0.67678839
644	0.18	0.19	31.56000078	0.852899998	0	REVERSED Glutamate [NMDA] receptor subunit zeta-1 OS=Rattus norvegicus GN=Grin1 PE=1 SV=1	RAT	0	1.057386041		1.007889986	
436	0.67	0.68	18.95000041	2.823000029	2.823000029	Clathrin light chain A OS=Rattus norvegicus GN=Clta PE=1 SV=1	RAT	1	1.057023048	0.687726617	0.936451912	0.640655577
716	0.13	0.16	19.63	2.094000019	0	V-type proton ATPase subunit C 1 OS=Rattus norvegicus GN=Atp6v1c1 PE=2 SV=1	RAT	0	1.05675602	0.675832391	1.023920059	0.850600183
730	0.12	0.16	21.78999931	0.606699986	0	Sodium channel protein type 8 subunit alpha OS=Rattus norvegicus GN=Scn8a PE=2 SV=1	RAT	0	1.056596994	0.68362987	0.965713978	0.621133924
423	0.75	0.77	47.24999964	2.982000075	0	Ras GTPase-activating protein SynGAP OS=Rattus norvegicus GN=Syngap1 PE=1 SV=2	RAT	0	1.056421041	0.679989874	1.057589054	0.673576415
83	13.41	13.41	78.64000201	28.0099988	24.05000031	Dihydrolipoyllysine-residue acetyltransferase component of pyruvate dehydrogenase complex, mitochondrial OS=Rattus norvegicus GN=Dlat PE=1 SV=3	RAT	8	1.056409001	0.3040573	1.001001954	0.982937098
29	30.74	30.94	96.31000161	62.74999976	54.36000228	ADP/ATP translocase 1 OS=Rattus norvegicus GN=Slc25a4 PE=1 SV=3	RAT	21	1.056401968	0.467668414	0.9865731	0.854996383
962	0.06	0.24	37.47000098	1.532999985	0	Carbamoyl-phosphate synthase [ammonia], mitochondrial OS=Rattus norvegicus GN=Cps1 PE=1 SV=1	RAT	0	1.05635798		1.201452017	
878	0.08	0.09	17.64999926	17.64999926	0	REVERSED Ubiquitin-fold modifier 1 OS=Rattus norvegicus GN=Ufm1 PE=2 SV=1	RAT	0	1.055948019		0.90647769	
1057	0.05	0.11	21.26999944	0	0	REVERSED Sodium channel protein type 10 subunit alpha OS=Rattus norvegicus GN=Scn10a PE=1 SV=1	RAT	0	1.055852056	0.571914077	1.029091001	0.758023381
605	0.21	0.23	14.23999965	3.779000044	0	REVERSED Trace amine-associated receptor 8b OS=Rattus norvegicus GN=Taar8b PE=3 SV=1	RAT	0	1.055353999	0.715105176	1.051800013	0.781606317
791	0.1	0.12	36.41000092	0	0	Rho guanine nucleotide exchange factor 11 OS=Rattus norvegicus GN=Arhgef11 PE=1 SV=1	RAT	0	1.055241942	0.506359994	0.991415024	0.911193192
831	0.09	0.1	31.52999878	1.083000004	0	Vascular cell adhesion protein 1 OS=Rattus norvegicus GN=Vcam1 PE=2 SV=1	RAT	0	1.05506897		1.022979975	
398	1.06	1.09	24.67000037	1.396000013	1.396000013	Calcium-dependent secretion activator 1 OS=Rattus norvegicus GN=Cadps PE=1 SV=1	RAT	1	1.054963946	0.503290713	0.906960189	0.260276705
1023	0.06	0.06	52.24000216	6.089999899	0	Basal cell adhesion molecule OS=Rattus norvegicus GN=Bcam PE=2 SV=1	RAT	0	1.054183006		1.106541991	
299	2.05	2.08	72.00999856	2.865000069	2.865000069	Gephyrin OS=Rattus norvegicus GN=Gphn PE=1 SV=3	RAT	1	1.053979039	0.600077212	1.024441957	0.797364593
515	0.36	0.37	14.13999945	2.163000032	0	Serine/threonine-protein phosphatase 2A 65 kDa regulatory subunit A beta isoform OS=Rattus norvegicus GN=Ppp2r1b PE=2 SV=1	RAT	0	1.053962946	0.694674671	0.971686304	0.823279679
770	0.11	0.11	31.54999912	5.882000178	0	4-hydroxybenzoate polyprenyltransferase, mitochondrial OS=Rattus norvegicus GN=Coq2 PE=2 SV=1	RAT	0	1.053913951		1.142886043	
255	2.89	2.89	73.65000248	9.934999794	9.934999794	Heterogeneous nuclear ribonucleoprotein K OS=Rattus norvegicus GN=Hnrnpk PE=1 SV=1	RAT	3	1.053820014	0.71482712	0.934728503	0.898615479
672	0.16	0.16	86.90000176	14.48000073	0	REVERSED 40S ribosomal protein S19 OS=Rattus norvegicus GN=Rps19 PE=2 SV=3	RAT	0	1.053668022	0.699256897	1.04184103	0.75786382
996	0.06	0.08	34.4900012	0	0	REVERSED SH3 and multiple ankyrin repeat domains protein 3 OS=Rattus norvegicus GN=Shank3 PE=1 SV=2	RAT	0	1.053634048	0.714730799	0.962668717	0.766104102
99	11.34	15.61	75.19999743	26.01999938	26.01999938	14-3-3 protein eta OS=Rattus norvegicus GN=Ywhah PE=1 SV=2	RAT	9	1.053226948	0.518424213	1.073613048	0.563997507
1062	0.05	0.1	17.03000069	0	0	Neurogenic locus notch homolog protein 3 OS=Rattus norvegicus GN=Notch3 PE=2 SV=2	RAT	0	1.052718997	0.695619285	1.047992945	0.779176116

241	3.35	3.48	89.21999931	37.25000024	25.49000084	10 kDa heat shock protein, mitochondrial OS=Rattus norvegicus GN=Hspe1 PE=1 SV=3	RAT	2	1.052714944	0.480574101	1.046720982	0.528013825
428	0.7	0.72	23.08000028	2.91799996	2.91799996	Guanine nucleotide exchange factor for Rab-3A OS=Rattus norvegicus GN=Rab3il1 PE=1 SV=1	RAT	1	1.052656054	0.695569813	1.12202096	0.452792287
526	0.34	0.38	20.09000033	3.929999843	0	Clathrin light chain B OS=Rattus norvegicus GN=Cltb PE=1 SV=1	RAT	0	1.052484989	0.696791291	1.083724976	0.566056073
390	1.17	1.17	30.23000062	5.587000027	0	Serotransferrin OS=Rattus norvegicus GN=Tf PE=1 SV=3	RAT	0	1.052440047	0.459839404	1.226114035	0.064492837
260	2.78	2.78	76.88000202	29.15000021	9.044999629	Peroxiredoxin-1 OS=Rattus norvegicus GN=Prdx1 PE=1 SV=1	RAT	2	1.052178025	0.480494797	1.016644955	0.845312774
1122	0.05	0.05	32.60999918	0	0	Probable ATP-dependent RNA helicase DHX40 OS=Rattus norvegicus GN=Dhx40 PE=2 SV=1	RAT	0	1.052055955		0.875754476	
950	0.07	0.07	53.15999985	0	0	REVERSED Dihydroorotate dehydrogenase (quinone), mitochondrial OS=Rattus norvegicus GN=Dhodh PE=1 SV=1	RAT	0	1.051187992	0.704748094	0.969233274	0.824793994
179	5.96	5.96	82.76000023	35.3399992	35.3399992	Vesicle-associated membrane protein 2 OS=Rattus norvegicus GN=Vamp2 PE=1 SV=2	RAT	4	1.05104804	0.501801014	1.01187396	0.930304587
366	1.64	1.66	45.51999867	8.275999874	8.275999874	Synaptojanin-2-binding protein OS=Rattus norvegicus GN=Synj2bp PE=1 SV=2	RAT	1	1.050421		0.944197297	
419	0.79	0.8	20.16000003	0.445799995	0.445799995	Sodium channel protein type 5 subunit alpha OS=Rattus norvegicus GN=Scn5a PE=2 SV=1	RAT	1	1.05035603		0.885632575	
170	6.2	6.22	80.76999784	30.12999892	24.3599996	Ubiquitin-40S ribosomal protein S27a OS=Rattus norvegicus GN=Rps27a PE=1 SV=2	RAT	3	1.050333977	0.486882687	1.054911971	0.432489514
397	1.07	1.07	42.10999906	19.74000037	6.578999758	Ubiquitin-conjugating enzyme E2 N OS=Rattus norvegicus GN=Ube2n PE=1 SV=1	RAT	1	1.05004704	0.716477573	0.984094501	0.898958027
646	0.18	0.18	34.29999948	2.900999971	0	REVERSED Dual specificity tyrosine-phosphorylation-regulated kinase 3 OS=Rattus norvegicus GN=Dyrk3 PE=2 SV=1	RAT	0	1.049350977		0.993151784	
395	1.1	1.1	29.46999967	3.139999881	3.139999881	Secernin-1 OS=Rattus norvegicus GN=Scrn1 PE=1 SV=1	RAT	1	1.048529983	0.622845829	1.075147986	0.471959412
164	6.6	6.6	95.50999999	58.42999816	58.42999816	Cytochrome b-c1 complex subunit 6, mitochondrial OS=Rattus norvegicus GN=Uqcrh PE=2 SV=1	RAT	4	1.04845798	0.730912805	1.012704015	0.920726478
472	0.5	0.55	23.3799994	1.425999962	0	Adenylate cyclase type 5 OS=Rattus norvegicus GN=Adcy5 PE=2 SV=2	RAT	0	1.048051	0.718210101	1.010416985	0.933584392
776	0.1	0.28	29.1200012	0	0	REVERSED Apolipoprotein B-100 OS=Rattus norvegicus GN=Apob PE=1 SV=1	RAT	0	1.047495961	0.506737173	1.065780044	0.373630404
328	2	2	71.39000297	7.219000161	7.219000161	Alcohol dehydrogenase class-3 OS=Rattus norvegicus GN=Adh5 PE=1 SV=2	RAT	1	1.04716301		1.143373966	
30	29.37	29.46	96.28000259	75.34999847	67.9099977	Aspartate aminotransferase, mitochondrial OS=Rattus norvegicus GN=Got2 PE=1 SV=2	RAT	22	1.046151996	0.338498414	1.042898059	0.362682492
197	4.86	4.86	59.61999893	23.08000028	19.23000067	Carbonic anhydrase 2 OS=Rattus norvegicus GN=Ca2 PE=1 SV=2	RAT	3	1.045511961	0.678270996	0.896063328	0.358927488
911	0.07	0.11	26.28000081	0	0	Ubiquitin-like modifier-activating enzyme 1 OS=Rattus norvegicus GN=Uba1 PE=1 SV=1	RAT	0	1.045434952	0.580202818	1.046290994	0.622895598
471	0.51	0.51	28.67999971	1.150000002	0	Protein FAM65A OS=Rattus norvegicus GN=Fam65a PE=2 SV=2	RAT	0	1.045411944	0.73199749	1.053861976	0.725564718
376	1.41	1.43	17.84999967	3.150000051	3.150000051	cAMP-dependent protein kinase type I-beta regulatory subunit OS=Rattus norvegicus GN=Prkar1b PE=2 SV=2	RAT	1	1.04537499	0.743112624	0.993787408	0.962124527
159	6.76	6.76	70.70999742	19.19	19.19	Beta-soluble NSF attachment protein OS=Rattus norvegicus GN=Napb PE=1 SV=1	RAT	4	1.045367956	0.645761609	1.024474978	0.798094511
809	0.09	0.51	26.48000121	0.110700005	0	Dynein heavy chain 1, axonemal OS=Rattus norvegicus GN=Dnah1 PE=2 SV=2	RAT	0	1.045199037	0.600560725	0.98790431	0.926179528
454	0.58	0.58	32.85000026	1.197000034	0	Neuroendocrine convertase 1 OS=Rattus norvegicus GN=Pcsk1 PE=2 SV=1	RAT	0	1.044819951	0.740106523	1.090108991	0.551024199
1029	0.06	0.06	47.47999907	0	0	REVERSED Na(+)/H(+) exchange regulatory cofactor NHE-RF2 OS=Rattus norvegicus GN=Slc9a3r2 PE=1 SV=1	RAT	0	1.044366002	0.738925874	1.135550022	0.42462039
926	0.07	0.09	27.48000026	0	0	REVERSED Phosphatidylinositol-4,5-bisphosphate 3-kinase catalytic subunit beta isoform OS=Rattus norvegicus GN=Pik3cb PE=2 SV=1	RAT	0	1.043916941	0.739592075	1.00720799	0.954169989
1033	0.06	0.06	32.13	0	0	REVERSED CMP-N-acetylneuraminate-beta-galactosamide-alpha-2,3-sialyltransferase 4 OS=Rattus norvegicus GN=St3gal4 PE=2 SV=1	RAT	0	1.043558002	0.815769017	1.000288963	0.997117281
177	6.01	6.01	69.34999824	29.15000021	19.59999949	Transgelin-3 OS=Rattus norvegicus GN=Tagln3 PE=1 SV=2	RAT	3	1.043303967	0.598514974	1.124719977	0.142705306
69	15.94	50.59	93.56999993	86.47000194	84.70000029	Tubulin alpha-1A chain OS=Rattus norvegicus GN=Tuba1a PE=1 SV=1	RAT	152	1.043092012	0.602226496	1.079439998	0.370553613
287	2.14	2.14	54.83999848	11.4699997	7.88500011	Complement component 1 Q subcomponent-binding protein, mitochondrial OS=Rattus norvegicus GN=C1qbp PE=1 SV=2	RAT	2	1.042930961	0.747855484	1.144570947	0.407049596
871	0.08	0.1	22.34999985	2.535000071	0	ATP-sensitive inward rectifier potassium channel 14 OS=Rattus norvegicus GN=Kcnj14 PE=1 SV=1	RAT	0	1.042850018		1.093601942	
907	0.07	0.13	30.75000048	0	0	Zinc finger RNA-binding protein OS=Rattus norvegicus GN=Zfr PE=1 SV=2	RAT	0	1.041872025	0.749538422	1.406914949	0.438860714
656	0.17	0.17	26.05000138	0.803900044	0	Mitochondrial Rho GTPase 2 OS=Rattus norvegicus GN=Rhot2 PE=2 SV=1	RAT	0	1.041862011		1.09157896	
142	7.78	7.79	64.09999728	14.00000006	12.16999963	Fascin OS=Rattus norvegicus GN=Fscn1 PE=1 SV=2	RAT	5	1.041615009	0.497601986	1.120198011	0.085551582
43	22.41	22.41	86.94000244	53.43999863	42.98999906	Synaptotagmin-1 OS=Rattus norvegicus GN=Syt1 PE=1 SV=3	RAT	17	1.04114902	0.393736511	1.086063027	0.099969998
649	0.17	0.23	20.63000053	5.820000172	0	Receptor expression-enhancing protein 5 OS=Rattus norvegicus GN=Reep5 PE=1 SV=1	RAT	0	1.041120052		0.96337682	
32	28.31	28.31	97.03999758	74.26000237	66.86000228	Malate dehydrogenase, mitochondrial OS=Rattus norvegicus GN=Mdh2 PE=1 SV=2	RAT	21	1.040683985	0.446672499	1.036414027	0.429045588
608	0.21	0.21	26.32000148	0.736800022	0	Mitogen-activated protein kinase kinase kinase 1 OS=Rattus norvegicus GN=Map3k1 PE=1 SV=1	RAT	0	1.040406942	0.757521987	1.027462959	0.829727173
697	0.14	0.17	26.33000016	0.719399983	0	Zona pellucida sperm-binding protein 2 OS=Rattus norvegicus GN=Zp2 PE=1 SV=1	RAT	0	1.04024303		0.834861219	
309	2.02	2.35	67.69000292	3.333000094	2.178999968	6-phosphofructokinase, liver type OS=Rattus norvegicus GN=Pfkl PE=2 SV=3	RAT	1	1.040040016	0.849458873	1.03341496	0.804997921
88	12.66	12.83	69.599998	39.55999911	37.36000061	ATP synthase subunit gamma, mitochondrial OS=Rattus norvegicus GN=Atp5c1 PE=1 SV=2	RAT	7	1.040016055	0.558739424	0.979913116	0.791668415
418	0.82	0.82	56.99999928	19.32000071	6.763000041	Ras-related protein Rab-7a OS=Rattus norvegicus GN=Rab7a PE=1 SV=2	RAT	1	1.039273024	0.778868675	1.067968011	0.646499217
24	34.8	34.8	94.70000267	72.11999893	70.05000114	Gamma-enolase OS=Rattus norvegicus GN=Eno2 PE=1 SV=2	RAT	42	1.038964033	0.503153622	0.999302208	0.985479772
171	6.1	6.18	53.17000151	2.675000019	2.394999936	Fatty acid synthase OS=Rattus norvegicus GN=Fasn PE=1 SV=3	RAT	4	1.038759947	0.700488627	1.072060943	0.615302384
476	0.49	0.49	18.75	2.71700006	0	Hsc70-interacting protein OS=Rattus norvegicus GN=St13 PE=1 SV=1	RAT	0	1.038745999		0.958248317	
687	0.15	0.15	25.99999905	2.127999999	0	Mannose-6-phosphate isomerase OS=Rattus norvegicus GN=Mpi PE=2 SV=1	RAT	0	1.038411021		1.279595017	
391	1.16	1.18	50.51000118	5.888999999	4.983000085	Brevican core protein OS=Rattus norvegicus GN=Bcan PE=1 SV=2	RAT	2	1.038318038	0.757667184	0.967142582	0.682766676
20	36.96	37.04	84.04999971	59.38000083	50.81999898	Serum albumin OS=Rattus norvegicus GN=Alb PE=1 SV=2	RAT	28	1.038288951	0.319545388	1.871464014	1.56E-08
189	5.37	14.79	87.40000129	44.31000054	36.59000099	14-3-3 protein beta/alpha OS=Rattus norvegicus GN=Ywhab PE=1 SV=3	RAT	11	1.03786695	0.792149723	1.01799798	0.897972286
969	0.06	0.17	72.32000232	1.47599997	0	REVERSED Arginine and glutamate-rich protein 1 OS=Rattus norvegicus GN=Arglu1 PE=2 SV=1	RAT	0	1.037804008	0.691735387	0.907349229	0.353290588
315	2.02	2.02	68.90000105	14.0200004	14.0200004	CB1 cannabinoid receptor-interacting protein 1 OS=Rattus norvegicus GN=Cnrip1 PE=1 SV=1	RAT	1	1.037328959	0.779218972	1.07348001	0.611744702
47	21.04	21.11	82.96999931	32.80000091	32.80000091	Glucose-6-phosphate isomerase OS=Rattus norvegicus GN=Gpi PE=1 SV=1	RAT	15	1.037273049	0.392740488	1.005216002	0.888338685
213	4.11	4.17	91.29999876	12.37000003	12.37000003	Prohibitin-2 OS=Rattus norvegicus GN=Phb2 PE=1 SV=1	RAT	2	1.037258983	0.775146782	0.999391377	0.996092677
667	0.16	0.18	22.81000018	1.754000038	0	Probable G-protein coupled receptor 176 OS=Rattus norvegicus GN=Gpr176 PE=2 SV=2	RAT	0	1.037253022		1.11640203	
565	0.25	0.25	33.64000022	1.807999983	0	REVERSED Neurexin-2-alpha OS=Rattus norvegicus GN=Nrxn2 PE=1 SV=3	RAT	0	1.036836982	0.699658513	1.013702989	0.882614315
519	0.35	0.41	24.86999929	0.697400002	0	Chromodomain-helicase-DNA-binding protein 8 OS=Rattus norvegicus GN=Chd8 PE=1 SV=2	RAT	0	1.036753058	0.559858978	1.020378947	0.814699829
136	8.15	8.15	59.46999788	44.04999912	44.04999912	Protein-L-isoaspartate(D-aspartate) O-methyltransferase OS=Rattus norvegicus GN=Pcmt1 PE=1 SV=2	RAT	7	1.036581993	0.678345382	1.025102019	0.870634079
1054	0.05	0.12	39.39999938	0	0	REVERSED Vinculin OS=Rattus norvegicus GN=Vcl PE=1 SV=1	RAT	0	1.036489964	0.778578877	1.157068014	0.3796601
1046	0.05	0.2	24.76000041	0.430600019	0	REVERSED Diacylglycerol kinase zeta OS=Rattus norvegicus GN=Dgkz PE=2 SV=1	RAT	0	1.036352992		1.099107981	
1039	0.06	0.06	32.65999854	3.517999873	0	Uncharacterized protein C1orf105 homolog OS=Rattus norvegicus PE=2 SV=1	RAT	0	1.035702944		1.085111976	
470	0.51	0.56	33.77999961	1.617999934	0	Leucine zipper protein 1 OS=Rattus norvegicus GN=Luzp1 PE=2 SV=1	RAT	0	1.03569603	0.783043206	1.173416972	0.707964778
1013	0.06	0.07	20.67999989	0	0	Ectonucleotide pyrophosphatase/phosphodiesterase family member 6 OS=Rattus norvegicus GN=Enpp6 PE=2 SV=1	RAT	0	1.035637021	0.717215598	0.982198894	0.850435913
242	3.35	3.38	30.45000136	12.84999996	8.380000293	Mitogen-activated protein kinase 1 OS=Rattus norvegicus GN=Mapk1 PE=1 SV=3	RAT	2	1.035359979	0.714025915	1.005087018	0.962049723
412	3.35	3.38	30.45000136	12.84999996	8.380000293	Mitogen-activated protein kinase 1 OS=Rattus norvegicus GN=Mapk1 PE=1 SV=3	RAT	2	1.035359979	0.714025915	1.005087018	0.962049723
18	39.64	39.64	82.17999935	49.73999858	46.7900008	Aconitate hydratase, mitochondrial OS=Rattus norvegicus GN=Aco2 PE=1 SV=2	RAT	24	1.035240054	0.332621396	1.044327974	0.230763093
1138	0.05	0.05	15.52000046	1.410999987	0	REVERSED Angiopoietin-2 OS=Rattus norvegicus GN=Angpt2 PE=2 SV=2	RAT	0	1.034782052	0.788175821	1.198165059	0.318515211
467	0.53	0.58	30.45000136	3.21800001	0	Endophilin-B2 OS=Rattus norvegicus GN=Sh3glb2 PE=2 SV=2	RAT	0	1.03449595	0.79575181	1.109143972	0.495682508
1113	0.05	0.06	31.00000024	5.240000039	0	UPF0765 protein C10orf58 homolog OS=Rattus norvegicus PE=1 SV=1	RAT	0	1.034451962		1.030441999	
925	0.07	0.09	21.49000019	0	0	Protein timeless homolog OS=Rattus norvegicus GN=Timeless PE=1 SV=1	RAT	0	1.034000039		1.234197021	
204	4.33	4.34	44.94999945	12.12000027	9.342999756	Obg-like ATPase 1 OS=Rattus norvegicus GN=Ola1 PE=2 SV=1	RAT	2	1.033473015	0.827258408	0.932577789	0.609357715
320	2	4	55.61000109	15.81999958	15.81999958	Rho-related GTP-binding protein RhoB OS=Rattus norvegicus GN=Rhob PE=1 SV=1	RAT	2	1.033460021		0.88644731	
1094	0.05	0.07	32.39000142	0	0	Serum paraoxonase/arylesterase 1 OS=Rattus norvegicus GN=Pon1 PE=1 SV=3	RAT	0	1.033105016		0.931020319	

448	0.63	0.63	24.30000007	2.195999958	0	Amphiphysin OS=Rattus norvegicus GN=Amph PE=1 SV=1	RAT	0	1.032850981	0.802732587	0.933064401	0.615235388
135	8.18	13.77	83.78999829	17.1299994	14.21999931	78 kDa glucose-regulated protein OS=Rattus norvegicus GN=Hspa5 PE=1 SV=1	RAT	9	1.031975985	0.607560575	1.056933045	0.381140709
178	5.97	6.26	44.72000003	8.750999719	7.257000357	AP-2 complex subunit beta OS=Rattus norvegicus GN=Ap2b1 PE=1 SV=1	RAT	4	1.031913042	0.610353827	1.068202019	0.363141
26	32.16	32.16	85.55999994	53.35000157	45.82000077	Calcium/calmodulin-dependent protein kinase type II subunit alpha OS=Rattus norvegicus GN=Camk2a PE=1 SV=1	RAT	49	1.031481981	0.518685818	0.973216295	0.569774628
900	0.07	0.16	57.77000189	0.351199997	0	REVERSED Solute carrier family 12 member 5 OS=Rattus norvegicus GN=Slc12a5 PE=1 SV=2	RAT	0	1.03095305	0.742667019	1.053053021	0.589041471
901	0.07	0.16	38.9200002	0	0	E3 ubiquitin-protein ligase BRE1B OS=Rattus norvegicus GN=Rnf40 PE=1 SV=1	RAT	0	1.030833006	0.749563396	1.186630011	0.356532007
94	11.95	11.95	75.44999719	28.51999998	22.74000049	Myelin proteolipid protein OS=Rattus norvegicus GN=Plp1 PE=1 SV=2	RAT	9	1.030540943	0.658896923	1.003376961	0.952228785
539	0.32	0.32	31.31000102	4.797999933	0	Cell adhesion molecule 3 OS=Rattus norvegicus GN=Cadm3 PE=2 SV=1	RAT	0	1.029943943		1.091182947	
49	20.67	20.67	86.66999936	55.37999868	46.66999876	Myelin basic protein S OS=Rattus norvegicus GN=Mbp PE=1 SV=3	RAT	20	1.029878974	0.537047923	0.81826359	0.00153293
845	0.09	0.09	48.48000109	6.818000227	0	40S ribosomal protein S12 OS=Rattus norvegicus GN=Rps12 PE=1 SV=2	RAT	0	1.029353976		1.02975595	
632	0.19	0.25	14.30000067	0.457199989	0	Slit homolog 1 protein OS=Rattus norvegicus GN=Slit1 PE=1 SV=1	RAT	0	1.029312968		1.518504977	
310	2.02	2.08	56.33999705	8.450999856	8.450999856	AP-2 complex subunit sigma OS=Rattus norvegicus GN=Ap2s1 PE=2 SV=1	RAT	2	1.029266	0.837882578	0.947136104	0.692237318
797	0.1	0.1	52.42999792	0	0	REVERSED Protein-glutamine gamma-glutamyltransferase K OS=Rattus norvegicus GN=Tgm1 PE=2 SV=1	RAT	0	1.029032946	0.794976294	1.034891963	0.717416525
105	10.95	13.96	91.86999798	45.21999955	32.0600003	Creatine kinase U-type, mitochondrial OS=Rattus norvegicus GN=Ckmt1 PE=1 SV=1	RAT	8	1.028542995	0.554236829	1.012181044	0.797483981
864	0.08	0.11	43.04000139	1.133000012	0	REVERSED Nuclear RNA export factor 1 OS=Rattus norvegicus GN=Nxf1 PE=2 SV=1	RAT	0	1.028494954	0.824740171	1.056360006	0.679810405
316	2.01	2.89	99.2200017	17.97000021	17.97000021	Histone H2A.Z OS=Rattus norvegicus GN=H2afz PE=1 SV=2	RAT	2	1.028313994	0.837835789	0.915319681	0.560124516
952	0.07	0.07	26.6200006	2.976999991	0	REVERSED Double-stranded RNA-binding protein Staufen homolog 2 OS=Rattus norvegicus GN=Stau2 PE=1 SV=1	RAT	0	1.02828002	0.825529099	1.025544047	0.841150224
27	31.02	31.11	81.04000092	40.38000107	37.23999858	Heat shock protein HSP 90-alpha OS=Rattus norvegicus GN=Hsp90aa1 PE=1 SV=3	RAT	22	1.028123021	0.633111477	1.002624035	0.956272483
640	0.18	0.21	31.13000095	0.551000005	0	SH3 and multiple ankyrin repeat domains protein 3 OS=Rattus norvegicus GN=Shank3 PE=1 SV=2	RAT	0	1.027668953	0.771851301	1.039242983	0.684343219
812	0.09	0.14	35.21000147	0.6329	0	Valyl-tRNA synthetase OS=Rattus norvegicus GN=Vars PE=2 SV=2	RAT	0	1.027459979	0.857050121	0.952361524	0.624423325
270	2.51	2.53	30.66000044	10.90999991	4.173000157	Transketolase OS=Rattus norvegicus GN=Tkt PE=1 SV=1	RAT	1	1.027374029	0.734682083	0.965237081	0.658924103
151	7.11	7.11	66.36000276	33.18000138	23.18000048	Ras-related protein Rab-3A OS=Rattus norvegicus GN=Rab3a PE=1 SV=1	RAT	5	1.026888013	0.71484679	0.991641223	0.90727061
886	0.08	0.08	19.78999972	1.590000093	0	Macrophage colony-stimulating factor 1 OS=Rattus norvegicus GN=Csf1 PE=2 SV=1	RAT	0	1.026677012		1.093801975	
3	73.73	73.8	86.87000275	54.29000258	47.78000116	Sodium/potassium-transporting ATPase subunit alpha-3 OS=Rattus norvegicus GN=Atp1a3 PE=1 SV=2	RAT	55	1.026025057	0.422454089	1.012843966	0.689435303
931	0.07	0.09	24.60999936	5.468999967	0	REVERSED Lysine-rich coiled-coil protein 1 OS=Rattus norvegicus GN=Krcc1 PE=2 SV=1	RAT	0	1.025858045		1.008751035	
36	27.18	27.26	79.90000248	19.0200001	11.98000014	Microtubule-associated protein 2 OS=Rattus norvegicus GN=Map2 PE=1 SV=3	RAT	14	1.025565028	0.483665407	0.976254225	0.462175488
269	2.57	2.57	35.85000038	9.747999907	9.747999907	Ribose-phosphate pyrophosphokinase 1 OS=Rattus norvegicus GN=Prps1 PE=1 SV=2	RAT	2	1.025488973	0.842644811	1.033764005	0.795609772
902	0.07	0.16	42.41000116	0.627399981	0	REVERSED MAP/microtubule affinity-regulating kinase 3 OS=Rattus norvegicus GN=Mark3 PE=2 SV=1	RAT	0	1.025200009	0.880740285	1.126278996	0.318123311
686	0.15	0.15	45.82000077	1.772000082	0	ADP-ribosylation factor-like protein 13A OS=Rattus norvegicus GN=Arl13a PE=2 SV=1	RAT	0	1.024759054		0.979069412	
623	0.2	0.22	32.3300004	3.016999923	0	Protein lin-7 homolog A OS=Rattus norvegicus GN=Lin7a PE=1 SV=2	RAT	0	1.024739981	0.847397685	0.957014322	0.736231685
1112	0.05	0.06	17.63000041	0	0	39S ribosomal protein L38, mitochondrial OS=Rattus norvegicus GN=Mrpl38 PE=2 SV=2	RAT	0	1.024490952	0.793860376	0.94322139	0.546166122
432	0.68	0.68	26.75000131	1.396999974	1.396999974	REVERSED Solute carrier family 12 member 3 OS=Rattus norvegicus GN=Slc12a3 PE=2 SV=2	RAT	1	1.024307013		0.9737975	
168	6.39	6.41	71.06000185	18.90999973	16.32999927	Glycerol-3-phosphate dehydrogenase [NAD+], cytoplasmic OS=Rattus norvegicus GN=Gpd1 PE=1 SV=4	RAT	3	1.024266005	0.777821422	1.016103983	0.925097585
1083	0.05	0.07	29.15999889	0	0	REVERSED Glutamate [NMDA] receptor subunit 3A OS=Rattus norvegicus GN=Grin3a PE=1 SV=1	RAT	0	1.024260044	0.799211919	1.021195054	0.823410571
567	0.25	0.25	64.09999728	10.13000011	10.13000011	Dihydrolipoyllysine-residue succinyltransferase component of 2-oxoglutarate dehydrogenase complex, mitochondrial OS=Rattus norvegicus GN=Dlst PE=1 SV=2	RAT	3	1.024037004		0.819672585	
930	0.07	0.09	21.05000019	2.411999926	0	REVERSED Keratin, type I cytoskeletal 12 OS=Rattus norvegicus GN=Krt12 PE=2 SV=1	RAT	0	1.02401495	0.850766182	0.973040521	0.879558027
879	0.08	0.08	20.99999934	0	0	REVERSED Reelin OS=Rattus norvegicus GN=Reln PE=2 SV=1	RAT	0	1.023954988	0.725060821	0.992610216	0.911638618
312	2.02	2.02	39.48000073	5.178000033	5.178000033	Hydroxyacylglutathione hydrolase, mitochondrial OS=Rattus norvegicus GN=Hagh PE=1 SV=2	RAT	1	1.023769021		1.206223965	
351	1.85	1.87	59.32999849	4.306000099	4.306000099	26S protease regulatory subunit 6B OS=Rattus norvegicus GN=Psmc4 PE=1 SV=1	RAT	1	1.023494005	0.939386308	0.994386375	0.986340106
96	11.59	11.59	88.94000053	27.63999999	27.63999999	cAMP-dependent protein kinase type II-beta regulatory subunit OS=Rattus norvegicus GN=Prkar2b PE=1 SV=3	RAT	7	1.02344799	0.694531679	1.103167057	0.34451431
59	17.84	17.84	82.96999931	41.29000008	33.46000016	V-type proton ATPase subunit B, brain isoform OS=Rattus norvegicus GN=Atp6v1b2 PE=1 SV=1	RAT	12	1.023275971	0.653122187	0.961701512	0.40106371
461	0.56	0.58	8.292999864	1.138000004	0	REVERSED Nuclear receptor subfamily 1 group D member 1 OS=Rattus norvegicus GN=Nr1d1 PE=2 SV=2	RAT	0	1.022603035		1.157616019	
528	0.34	0.35	44.76000071	1.905000024	0	Transcriptional activator protein Pur-beta OS=Rattus norvegicus GN=Purb PE=1 SV=3	RAT	0	1.02235496	0.811793387	1.008118987	0.930275679
810	0.09	0.2	38.5800004	0	0	Tuberin OS=Rattus norvegicus GN=Tsc2 PE=1 SV=1	RAT	0	1.022346973	0.914492726	1.111366987	0.552723587
160	6.71	11.55	72.14000225	61.43000126	53.57000232	Alpha-synuclein OS=Rattus norvegicus GN=Snca PE=1 SV=1	RAT	6	1.022155046	0.753350317	0.945923388	0.442444593
378	1.4	1.4	12.6000002	8.660999686	8.660999686	Brain protein 44 OS=Rattus norvegicus GN=Brp44 PE=2 SV=1	RAT	1	1.021872997		0.949725628	
53	19.48	19.6	80.04000187	41.83999896	36.84999943	Serine/threonine-protein phosphatase 2B catalytic subunit alpha isoform OS=Rattus norvegicus GN=Ppp3ca PE=1 SV=1	RAT	14	1.021424055	0.676051617	1.068786979	0.446445614
855	0.08	0.16	29.31999862	0	0	REVERSED Nitric oxide synthase, brain OS=Rattus norvegicus GN=Nos1 PE=1 SV=1	RAT	0	1.021152973	0.820417702	1.006530046	0.943439424
80	13.54	13.54	98.58999848	32.85999894	32.85999894	Peroxiredoxin-5, mitochondrial OS=Rattus norvegicus GN=Prdx5 PE=1 SV=1	RAT	7	1.020900965	0.76906091	0.978536904	0.759304881
976	0.06	0.13	22.75999933	1.38600003	0	Immunoglobulin superfamily member 10 OS=Rattus norvegicus GN=Igsf10 PE=2 SV=1	RAT	0	1.020053983		1.037606001	
415	0.84	0.87	48.82999957	3.125	3.125	ATP synthase subunit b, mitochondrial OS=Rattus norvegicus GN=Atp5f1 PE=1 SV=1	RAT	1	1.019747019	0.803803325	0.968033195	0.711093605
939	0.07	0.08	22.61999995	0	0	Corepressor interacting with RBPJ 1 OS=Rattus norvegicus GN=Cir1 PE=2 SV=1	RAT	0	1.01967001	0.902791202	1.189805031	0.332637012
1080	0.05	0.07	37.99000084	0	0	REVERSED SH3 and multiple ankyrin repeat domains protein 2 OS=Rattus norvegicus GN=Shank2 PE=1 SV=2	RAT	0	1.019559979	0.838733673	0.927444518	0.4644036
15	41.52	41.52	99.21000004	84.50999856	77.17000246	Creatine kinase B-type OS=Rattus norvegicus GN=Ckb PE=1 SV=2	RAT	51	1.01949501	0.637719989	1.054420948	0.195060402
235	3.54	3.66	64.17999864	7.456000149	6.870999932	Rabphilin-3A OS=Rattus norvegicus GN=Rph3a PE=1 SV=1	RAT	3	1.019291043	0.888936281	0.95477277	0.742315114
882	0.08	0.08	38.58999908	5.454999954	0	REVERSED Potassium voltage-gated channel subfamily A member 1 OS=Rattus norvegicus GN=Kcna1 PE=1 SV=1	RAT	0	1.01926899	0.878795385	1.025856972	0.839212775
207	4.28	4.28	46.66999876	32.37999976	20.94999999	Thioredoxin OS=Rattus norvegicus GN=Txn PE=1 SV=2	RAT	2	1.018936038	0.791686118	0.993932128	0.931308687
353	1.81	1.81	50.45999885	12.83999979	5.505000055	Glutathione S-transferase Mu 1 OS=Rattus norvegicus GN=Gstm1 PE=1 SV=2	RAT	1	1.018717051	0.912700593	0.963807881	0.778483272
966	0.06	0.19	34.0200007	0.341899996	0	REVERSED Short transient receptor potential channel 2 OS=Rattus norvegicus GN=Trpc2 PE=2 SV=2	RAT	0	1.018429041	0.848471582	1.046058059	0.644957006
121	9.7	16.54	79.54999804	39.34000134	32.51999915	Dihydropyrimidinase-related protein 1 OS=Rattus norvegicus GN=Crmp1 PE=1 SV=1	RAT	11	1.018368006	0.769763827	0.976960301	0.68636322
67	16.16	16.41	73.03000093	20.53000033	17.41999984	Glycogen phosphorylase, brain form (Fragment) OS=Rattus norvegicus GN=Pygb PE=1 SV=3	RAT	13	1.017714024	0.74176091	1.054661989	0.453390509
109	10.7	10.79	55.14000058	5.652999878	4.758000001	Microtubule-associated protein 1B OS=Rattus norvegicus GN=Map1b PE=1 SV=2	RAT	7	1.017536998	0.866220176	1.054504037	0.523096502
913	0.07	0.1	20.73999941	0.617300021	0	Sushi, von Willebrand factor type A, EGF and pentraxin domain-containing protein 1 OS=Rattus norvegicus GN=Svep1 PE=1 SV=1	RAT	0	1.016875982	0.93522191	0.919619977	0.587826729
180	5.93	5.96	80.80000281	33.59999955	33.59999955	4-aminobutyrate aminotransferase, mitochondrial OS=Rattus norvegicus GN=Abat PE=1 SV=3	RAT	11	1.016252995	0.811033428	1.06703198	0.5163728
790	0.1	0.13	22.69999981	2.453999966	0	Protein phosphatase Slingshot homolog 3 OS=Rattus norvegicus GN=Ssh3 PE=2 SV=1	RAT	0	1.016222954		1.026342034	
485	0.45	0.45	80.18000126	4.504999891	0	REVERSED Glyceraldehyde-3-phosphate dehydrogenase OS=Rattus norvegicus GN=Gapdh PE=1 SV=3	RAT	0	1.015930057		0.887778819	
7	47.95	47.95	97.6000011	80.80000281	75.73000193	Actin, cytoplasmic 2 OS=Rattus norvegicus GN=Actg1 PE=1 SV=1	RAT	73	1.015833974	0.674590528	1.014125943	0.733573377
627	0.19	13.6	84.78999734	33.4100008	31.34000003	Beta-enolase OS=Rattus norvegicus GN=Eno3 PE=1 SV=3	RAT	19	1.015643001		1.046730042	
538	0.32	0.32	29.21999991	1.817999966	0	Low-density lipoprotein receptor-related protein 3 OS=Rattus norvegicus GN=Lrp3 PE=1 SV=1	RAT	0	1.015516043	0.905122697	0.93089211	0.61130631
728	0.12	0.18	29.19999957	0.620200019	0	REVERSED Myosin-7 OS=Rattus norvegicus GN=Myh7 PE=2 SV=2	RAT	0	1.015413046		1.00010705	
76	14.45	14.51	48.08999896	13.24999928	13.24999928	V-type proton ATPase 116 kDa subunit a isoform 1 OS=Rattus norvegicus GN=Atp6v0a1 PE=2 SV=1	RAT	10	1.015274048	0.741467714	1.041064024	0.343855411
786	0.1	0.13	26.94000006	0.601600017	0	Synaptojanin-2 OS=Rattus norvegicus GN=Synj2 PE=1 SV=2	RAT	0	1.014840007		0.888621628	
129	8.49	8.54	97.75999784	50.22000074	46.63999975	Ubiquitin carboxyl-terminal hydrolase isozyme L1 OS=Rattus norvegicus GN=Uchl1 PE=1 SV=2	RAT	14	1.01463902	0.839364886	0.997289419	0.974236012
150	7.18	7.18	71.82999849	17.46000051	13.51999938	NADH dehydrogenase [ubiquinone] 1 alpha subcomplex subunit 10, mitochondrial OS=Rattus norvegicus GN=Ndufa10 PE=1 SV=1	RAT	7	1.014448047	0.842012227	0.946775317	0.610131323

107	10.85	10.9	60.98999977	15.57999998	11.87999994	Alpha-actinin-1 OS=Rattus norvegicus GN=Actn1 PE=1 SV=1	RAT	8	1.013918996	0.893805385	1.152333021	0.571257889
153	7.09	7.09	83.24999809	47.63999879	47.63999879	Visinin-like protein 1 OS=Rattus norvegicus GN=Vsnl1 PE=1 SV=2	RAT	7	1.013877034	0.886778474	0.966884196	0.732757509
111	10.37	10.58	59.83999968	9.892000258	8.815000206	Contactin-1 OS=Rattus norvegicus GN=Cntn1 PE=1 SV=2	RAT	8	1.013499975	0.881185293	0.937732577	0.425496101
439	0.66	0.66	18.68000031	5.494999886	5.494999886	Vacuolar protein sorting-associated protein 29 OS=Rattus norvegicus GN=Vps29 PE=1 SV=2	RAT	1	1.01326704		1.051720977	
17	40.36	40.44	100	89.78999853	84.97999907	Glyceraldehyde-3-phosphate dehydrogenase OS=Rattus norvegicus GN=Gapdh PE=1 SV=3	RAT	79	1.013201952	0.735047817	1.080454946	0.05776662
375	1.44	5.42	53.68999839	10.36000028	4.642999917	Heat shock 70 kDa protein 4 OS=Rattus norvegicus GN=Hspa4 PE=1 SV=1	RAT	3	1.013106942	0.891503811	1.099043965	0.450123101
468	0.53	0.53	18.63999963	5.248999968	0	Cytosolic acyl coenzyme A thioester hydrolase OS=Rattus norvegicus GN=Acot7 PE=1 SV=4	RAT	0	1.013007998	0.920192778	1.028596044	0.828735113
710	0.13	0.21	55.44000268	1.468999963	0	REVERSED Contactin-1 OS=Rattus norvegicus GN=Cntn1 PE=1 SV=2	RAT	0	1.012959957	0.903112829	1.048285007	0.623451173
872	0.08	0.09	30.30000031	1.059999969	0	REVERSED Tyrosine-protein kinase JAK2 OS=Rattus norvegicus GN=Jak2 PE=2 SV=1	RAT	0	1.012776971		0.924372315	
1115	0.05	0.06	25.69000125	4.51399982	0	REVERSED Corticosteroid 11-beta-dehydrogenase isozyme 1 OS=Rattus norvegicus GN=Hsd11b1 PE=1 SV=2	RAT	0	1.012776971		0.924372315	
25	32.89	32.98	73.93000126	26.62999928	25.20000041	Plasma membrane calcium-transporting ATPase 1 OS=Rattus norvegicus GN=Atp2b1 PE=2 SV=2	RAT	19	1.012711048	0.831583679	1.007568002	0.898804128
137	8.09	8.18	65.67999721	16.24000072	16.24000072	Ubiquitin thioesterase OTUB1 OS=Rattus norvegicus GN=Otub1 PE=1 SV=1	RAT	5	1.012609005	0.879604518	1.078287959	0.430806786
288	2.12	2.12	71.03000283	4.673000053	4.673000053	Sideroflexin-3 OS=Rattus norvegicus GN=Sfxn3 PE=2 SV=1	RAT	1	1.012539029	0.924436629	1.054123044	0.598651528
835	0.09	0.1	24.60999936	1.733000018	0	Zinc finger with UFM1-specific peptidase domain protein OS=Rattus norvegicus GN=Zufsp PE=2 SV=1	RAT	0	1.01238203		1.125314951	
22	36.05	36.05	86.15999818	50.5400002	38.98000121	Vesicle-fusing ATPase OS=Rattus norvegicus GN=Nsf PE=1 SV=1	RAT	19	1.012086034	0.810733914	1.048501968	0.38785401
426	0.71	0.97	67.04000235	1.173000038	0	REVERSED Spectrin beta chain, brain 2 OS=Rattus norvegicus GN=Sptbn2 PE=1 SV=2	RAT	0	1.011814952	0.92514497	1.056097984	0.68184942
262	2.73	2.75	65.31999707	6.650999933	6.650999933	60S ribosomal protein L4 OS=Rattus norvegicus GN=Rpl4 PE=1 SV=3	RAT	2	1.011540055	0.908720374	1.058428049	0.566486001
140	8.05	8.08	76.10999942	24.77999926	24.77999926	V-type proton ATPase subunit E 1 OS=Rattus norvegicus GN=Atp6v1e1 PE=1 SV=1	RAT	7	1.011432052	0.903079808	1.007895947	0.950407982
58	18.09	18.09	82.03999996	56.80000186	54.36999798	Synaptosomal-associated protein 25 OS=Rattus norvegicus GN=Snap25 PE=1 SV=1	RAT	12	1.011401057	0.81419462	1.060840011	0.239706397
732	0.12	0.15	10.06999984	3.356999904	0	REVERSED Multidrug and toxin extrusion protein 1 OS=Rattus norvegicus GN=Slc47a1 PE=1 SV=1	RAT	0	1.011306047		0.912220001	
382	1.33	1.39	38.74000013	3.156000003	3.156000003	Protein kinase C gamma type OS=Rattus norvegicus GN=Prkcg PE=1 SV=1	RAT	1	1.011219025	0.930795193	1.111001015	0.491415888
416	0.84	0.87	34.09000039	4.830000177	4.830000177	Alpha-2-HS-glycoprotein OS=Rattus norvegicus GN=Ahsg PE=1 SV=2	RAT	1	1.010632992		1.724748015	
291	2.09	2.09	64.85999823	6.142999977	3.686000034	Eukaryotic initiation factor 4A-II OS=Rattus norvegicus GN=Eif4a2 PE=1 SV=1	RAT	1	1.010501981	0.937242627	0.957680285	0.803672671
940	0.07	0.08	28.74000072	0	0	REVERSED Flotillin-2 OS=Rattus norvegicus GN=Flot2 PE=1 SV=1	RAT	0	1.010342002	0.950744689	0.884146571	0.275906891
827	0.09	0.11	28.18000019	1.083999965	0	REVERSED Dipeptidyl peptidase 3 OS=Rattus norvegicus GN=Dpp3 PE=1 SV=2	RAT	0	1.010272026	0.945754588	0.941922188	0.666842103
503	0.38	0.58	34.58999991	0.558699993	0	REVERSED Protein bassoon OS=Rattus norvegicus GN=Bsn PE=1 SV=3	RAT	0	1.010035038	0.888298571	0.97017169	0.726503193
48	20.95	20.95	94.24999952	73.44999909	57.08000064	Neuromodulin OS=Rattus norvegicus GN=Gap43 PE=1 SV=1	RAT	13	1.009861946	0.853800118	1.075327992	0.249041602
1	122.02	122.02	90.53000212	49.27000105	37.09999919	Spectrin alpha chain, brain OS=Rattus norvegicus GN=Sptan1 PE=1 SV=2	RAT	83	1.009763002	0.576383412	0.995934784	0.814881682
253	2.93	2.93	45.84999979	18.05000007	18.05000007	Protein phosphatase 1 regulatory subunit 1B OS=Rattus norvegicus GN=Ppp1r1b PE=2 SV=1	RAT	2	1.009552956	0.939927399	1.078101993	0.590217888
938	0.07	0.08	27.5000006	0	0	Peroxisomal targeting signal 1 receptor OS=Rattus norvegicus GN=Pex5 PE=1 SV=2	RAT	0	1.009528041	0.940365076	0.988745689	0.928586185
597	0.22	0.22	26.64000094	1.824999973	0	von Willebrand factor A domain-containing protein 5A OS=Rattus norvegicus GN=Vwa5a PE=2 SV=1	RAT	0	1.00908196		0.940837383	
342	2	2	18.70000064	16.25999957	8.129999787	Anionic trypsin-1 OS=Rattus norvegicus GN=Prss1 PE=1 SV=1	RAT	5	1.008967996		0.997649908	
359	1.75	1.78	71.18999958	32.19999969	22.87999988	D-dopachrome decarboxylase OS=Rattus norvegicus GN=Ddt PE=1 SV=3	RAT	2	1.00888896	0.945485473	0.882833898	0.563257992
1074	0.05	0.08	24.4599998	0	0	NLR family member X1 OS=Rattus norvegicus GN=Nlrx1 PE=2 SV=1	RAT	0	1.008687973		0.972200871	
163	6.64	6.72	55.46000004	30.32999933	15.29999971	Isocitrate dehydrogenase [NAD] subunit alpha, mitochondrial OS=Rattus norvegicus GN=Idh3a PE=1 SV=1	RAT	4	1.008672953	0.855704427	1.012931943	0.787203729
203	4.38	4.38	34.5299989	9.446000308	9.446000308	Synaptophysin OS=Rattus norvegicus GN=Syp PE=1 SV=1	RAT	3	1.008535028	0.929574788	1.016283989	0.866575181
12	44.29	44.29	91.67000055	67.86000133	58.09999704	2',3'-cyclic-nucleotide 3'-phosphodiesterase OS=Rattus norvegicus GN=Cnp PE=1 SV=2	RAT	51	1.007606983	0.808463216	0.92242229	0.007179616
527	0.34	0.35	30.23999929	1.133000012	0	Glutamate receptor 2 OS=Rattus norvegicus GN=Gria2 PE=1 SV=2	RAT	0	1.007501006		0.97093147	
61	17.3	17.3	96.46999836	71.38000131	68.55000257	Voltage-dependent anion-selective channel protein 1 OS=Rattus norvegicus GN=Vdac1 PE=1 SV=4	RAT	17	1.006826997	0.882282078	1.044078946	0.355716586
1091	0.05	0.07	26.91000104	0	0	REVERSED SH3 domain-binding protein 5 OS=Rattus norvegicus GN=Sh3bp5 PE=1 SV=2	RAT	0	1.006778002	0.942230403	0.992272913	0.952165186
1007	0.06	0.07	26.3500005	0	0	REVERSED Canalicular multispecific organic anion transporter 1 OS=Rattus norvegicus GN=Abcc2 PE=2 SV=1	RAT	0	1.006669998	0.942348719	0.988905489	0.94615829
124	9.16	9.18	77.74999738	22.24999964	16.76000059	Succinyl-CoA ligase [GDP-forming] subunit alpha, mitochondrial OS=Rattus norvegicus GN=Suclg1 PE=2 SV=2	RAT	5	1.006171942	0.918363214	0.992973387	0.888331711
185	5.57	5.57	56.33999705	5.457000062	3.170999885	Tenascin-R OS=Rattus norvegicus GN=Tnr PE=1 SV=1	RAT	4	1.005697012	0.949725628	1.003765941	0.962368309
774	0.11	0.11	47.79999852	4.402999952	0	Succinate dehydrogenase [ubiquinone] cytochrome b small subunit, mitochondrial OS=Rattus norvegicus GN=Sdhd PE=2 SV=1	RAT	0	1.005692959	0.971167326	0.990401208	0.938303709
579	0.24	0.24	34.70999897	5.784999952	0	Microtubule-associated proteins 1A/1B light chain 3A OS=Rattus norvegicus GN=Map1lc3a PE=1 SV=1	RAT	0	1.005674005		0.909312308	
505	0.38	0.39	53.46000195	6.452000141	0	60S ribosomal protein L10a OS=Rattus norvegicus GN=Rpl10a PE=1 SV=2	RAT	0	1.005560994	0.965667903	1.002398968	0.985269427
125	8.98	9.04	81.27999902	12.15000004	12.15000004	Mitochondrial inner membrane protein (Fragment) OS=Rattus norvegicus GN=Immt PE=1 SV=1	RAT	6	1.005103946	0.94805789	0.994859219	0.946921885
40	24.75	24.93	73.44999909	10.33999994	8.291000128	Spectrin beta chain, brain 2 OS=Rattus norvegicus GN=Sptbn2 PE=1 SV=2	RAT	14	1.005095005	0.898974776	0.964095473	0.504204929
1129	0.05	0.05	15.02999961	0	0	REVERSED Calsyntenin-2 OS=Rattus norvegicus GN=Clstn2 PE=2 SV=1	RAT	0	1.004871011		1.017557025	
923	0.07	0.09	31.47000074	2.800999954	0	REVERSED Lysine-specific demethylase 3A OS=Rattus norvegicus GN=Kdm3a PE=2 SV=1	RAT	0	1.004171014		0.977261305	
748	0.12	0.12	29.92999852	2.386000007	0	Coronin-1A OS=Rattus norvegicus GN=Coro1a PE=1 SV=3	RAT	0	1.00385499	0.977423787	0.936602771	0.632494092
292	2.08	2.14	28.49000096	8.139999956	8.139999956	Translationally-controlled tumor protein OS=Rattus norvegicus GN=Tpt1 PE=1 SV=1	RAT	1	1.00376296	0.976271987	0.956442475	0.776270986
551	0.28	0.3	33.62999856	1.011999976	0	REVERSED Potassium voltage-gated channel subfamily C member 3 OS=Rattus norvegicus GN=Kcnc3 PE=2 SV=1	RAT	0	1.003738046		1.127218962	
158	6.79	6.79	82.31999874	45.44999897	45.44999897	Peroxiredoxin-2 OS=Rattus norvegicus GN=Prdx2 PE=1 SV=3	RAT	6	1.003443003	0.967509687	1.020256042	0.813406408
383	1.32	1.33	36.44999862	5.046999827	5.046999827	Brain-specific angiogenesis inhibitor 1-associated protein 2 OS=Rattus norvegicus GN=Baiap2 PE=1 SV=1	RAT	1	1.003414035	0.983633518	1.097259998	0.401548207
1004	0.06	0.08	33.61999989	2.029000036	0	Coiled-coil domain-containing protein KIAA1826 homolog OS=Rattus norvegicus PE=2 SV=1	RAT	0	1.003337979	0.978547275	1.05122304	0.702081919
683	0.15	0.16	27.63000131	11.84	0	Cytochrome c oxidase subunit 6C-2 OS=Rattus norvegicus GN=Cox6c2 PE=1 SV=3	RAT	0	1.003216982	0.979456782	0.906278312	0.50293529
497	0.4	0.42	27.32000053	3.195000067	3.195000067	Myelin-associated glycoprotein OS=Rattus norvegicus GN=Mag PE=1 SV=1	RAT	1	1.003216028	0.981874704	1.014785051	0.906243086
200	4.47	4.47	44.65999901	5.124000087	3.807000071	Synaptic vesicle glycoprotein 2B OS=Rattus norvegicus GN=Sv2b PE=1 SV=1	RAT	3	1.003015041	0.970054626	1.094606042	0.349060386
523	0.35	0.35	20.11000067	3.351999819	0	Neuronal-specific septin-3 OS=Rattus norvegicus GN=Sept3 PE=1 SV=2	RAT	0	1.002868056	0.981634974	1.010053039	0.936204374
71	15.17	15.17	98.18999767	62.65000105	56.62999749	Cofilin-1 OS=Rattus norvegicus GN=Cfl1 PE=1 SV=3	RAT	14	1.002814054	0.954160094	0.975235581	0.578847706
410	0.87	0.89	34.15000141	11.99999973	0	Alcohol dehydrogenase [NADP+] OS=Rattus norvegicus GN=Akr1a1 PE=1 SV=2	RAT	0	1.002683997	0.977537811	0.978903711	0.823333919
1136	0.05	0.05	31.25	10.10000035	0	REVERSED Uncharacterized protein C4orf51 homolog OS=Rattus norvegicus PE=2 SV=1	RAT	0	1.002562046	0.988012373	0.984379113	0.899592876
2	74.38	74.38	77.67000198	50.20999908	38.98999989	Clathrin heavy chain 1 OS=Rattus norvegicus GN=Cltc PE=1 SV=3	RAT	58	1.002298951	0.941793621	1.035858035	0.203705296
194	5.05	5.11	27.4300009	5.683000013	4.58999984	Clathrin coat assembly protein AP180 OS=Rattus norvegicus GN=Snap91 PE=1 SV=1	RAT	3	1.002292037	0.97719878	0.966140389	0.745055377
600	0.22	0.22	51.84999704	12.03999966	0	CDGSH iron-sulfur domain-containing protein 1 OS=Rattus norvegicus GN=Cisd1 PE=3 SV=1	RAT	0	1.002086997		0.950467229	
258	2.85	2.85	84.95000005	13.1099999	13.1099999	Ras-related protein Ral-A OS=Rattus norvegicus GN=Rala PE=1 SV=1	RAT	2	1.00199604	0.987276077	1.005558014	0.964737892
108	10.72	10.72	71.32999897	21.44999951	19.9300006	Neural cell adhesion molecule 1 OS=Rattus norvegicus GN=Ncam1 PE=1 SV=1	RAT	10	1.001777053	0.968000114	1.124135971	0.1177838
64	16.87	16.87	66.10000134	21.63999975	13.1099999	AP-2 complex subunit alpha-2 OS=Rattus norvegicus GN=Ap2a2 PE=1 SV=3	RAT	9	1.001611948	0.971333325	1.059512019	0.25311169
374	1.46	1.46	44.85000074	8.357000351	8.357000351	Guanine nucleotide-binding protein G(q) subunit alpha OS=Rattus norvegicus GN=Gnaq PE=1 SV=2	RAT	2	1.001391053	0.993394792	1.001531005	0.990148783
39	25.05	25.05	83.67000222	56.73000216	56.73000216	14-3-3 protein zeta/delta OS=Rattus norvegicus GN=Ywhaz PE=1 SV=1	RAT	21	1.001356959	0.975747406	1.068410993	0.155558795
237	3.46	3.48	67.72999763	19.32999939	9.042999893	Dihydropyrimidinase-related protein 4 (Fragment) OS=Rattus norvegicus GN=Dpysl4 PE=1 SV=1	RAT	3	1.001011968	0.991814911	0.990315676	0.922284722
982	0.06	0.1	29.03999984	0	0	Huntingtin OS=Rattus norvegicus GN=Htt PE=1 SV=1	RAT	0	1.000916004	0.990383804	0.921391189	0.326586396
474	0.49	0.51	32.92999864	1.706999913	0	Centromere protein U OS=Rattus norvegicus GN=Mlf1ip PE=2 SV=1	RAT	0	1.000488043		1.140854955	

236	3.5	3.5	80.11999726	22.98000008	13.0400002	Thy-1 membrane glycoprotein OS=Rattus norvegicus GN=Thy1 PE=1 SV=1	RAT	2	1		0.998354971	
166	6.58	6.62	84.21999812	19.52999979	18.15000027	Fumarate hydratase, mitochondrial OS=Rattus norvegicus GN=Fh PE=1 SV=1	RAT	9	0.999927819	0.999413788	0.982004881	0.82043308
868	0.08	0.1	28.79999876	1.178000029	0	REVERSED Transient receptor potential cation channel subfamily M member 8 OS=Rattus norvegicus GN=Trpm8 PE=2 SV=4	RAT	0	0.999854207	0.998779297	0.914521396	0.3957389
114	10.11	10.19	65.14000297	23.99999946	23.99999946	NAD-dependent deacetylase sirtuin-2 OS=Rattus norvegicus GN=Sirt2 PE=1 SV=1	RAT	9	0.999501586	0.99595499	0.966763616	0.759435296
245	3.18	3.26	61.3499999	4.687999934	2.604000084	Dynamin-like 120 kDa protein, mitochondrial OS=Rattus norvegicus GN=Opa1 PE=1 SV=1	RAT	3	0.998629987	0.984000087	1.012444973	0.856336176
196	4.91	4.91	68.90000105	14.68999982	13.60999942	NADH dehydrogenase [ubiquinone] iron-sulfur protein 2, mitochondrial OS=Rattus norvegicus GN=Ndufs2 PE=1 SV=1	RAT	4	0.998294413	0.986215174	0.994520187	0.955541611
5	51.27	51.49	95.28999925	71.93999887	66.2899971	Pyruvate kinase isozymes M1/M2 OS=Rattus norvegicus GN=Pkm2 PE=1 SV=3	RAT	37	0.998279989	0.952265918	1.020856977	0.474528998
285	2.14	9.19	91.01999998	37.54999936	29.80000079	14-3-3 protein theta OS=Rattus norvegicus GN=Ywhaq PE=1 SV=1	RAT	7	0.997533619	0.985007584	1.03382504	0.803965986
998	0.06	0.08	26.8900007	0	0	REVERSED Anion exchange protein 3 OS=Rattus norvegicus GN=Slc4a3 PE=2 SV=1	RAT	0	0.997412324		0.878107309	
463	0.55	0.57	30.25999963	1.164000016	0	Sodium-driven chloride bicarbonate exchanger OS=Rattus norvegicus GN=Slc4a10 PE=2 SV=1	RAT	0	0.997251928		0.80985707	
147	7.38	7.41	66.67000055	21.95000052	21.95000052	Septin-5 OS=Rattus norvegicus GN=Sept5 PE=1 SV=2	RAT	7	0.997213125	0.971167982	0.976953387	0.788756907
361	1.74	1.74	34.31999981	0.927499961	0.927499961	Arf-GAP with GTPase, ANK repeat and PH domain-containing protein 2 OS=Rattus norvegicus GN=Agap2 PE=1 SV=1	RAT	1	0.996850312		1.071015954	
955	0.07	0.07	26.94999874	0	0	REVERSED Serpin B10 OS=Rattus norvegicus GN=Serpinb10 PE=2 SV=1	RAT	0	0.996847928	0.977111876	0.973965526	0.778444707
634	0.19	0.23	30.77000082	1.737000048	0	REVERSED Sex hormone-binding globulin OS=Rattus norvegicus GN=Shbg PE=1 SV=1	RAT	0	0.996779621	0.979232073	0.927577317	0.586154521
154	7.08	7.08	68.83000135	24.16000068	24.16000068	Isocitrate dehydrogenase [NAD] subunit beta, mitochondrial OS=Rattus norvegicus GN=Idh3B PE=1 SV=1	RAT	6	0.996611774	0.978500724	0.918777287	0.431110889
42	22.58	22.99	68.23999882	10.74000001	10.53000018	Microtubule-associated protein 1A OS=Rattus norvegicus GN=Map1a PE=1 SV=1	RAT	17	0.996110022	0.927619576	0.959403872	0.341302514
574	0.24	0.25	38.60999942	4.633000121	0	REVERSED Estradiol 17-beta-dehydrogenase 8 OS=Rattus norvegicus GN=Hsd17b8 PE=2 SV=1	RAT	0	0.996048212	0.974541008	1.036082029	0.780911028
157	6.79	6.79	63.87000084	29.5599997	26.28000081	Cytochrome b-c1 complex subunit Rieske, mitochondrial OS=Rattus norvegicus GN=Uqcrfs1 PE=1 SV=2	RAT	4	0.996042013	0.957283974	0.977535486	0.761010706
182	5.71	5.71	64.63000178	16.32999927	14.74000067	Protein kinase C and casein kinase substrate in neurons protein 1 OS=Rattus norvegicus GN=Pacsin1 PE=1 SV=1	RAT	5	0.995894074	0.94001317	1.021332979	0.700341523
112	10.29	10.34	69.41000223	35.69000065	31.43999875	Heterogeneous nuclear ribonucleoproteins A2/B1 OS=Rattus norvegicus GN=Hnrnpa2b1 PE=1 SV=1	RAT	8	0.99562639	0.943551183	1.034204006	0.590405226
195	5.02	5.02	60.04999876	17.04999954	5.344000086	Dual specificity mitogen-activated protein kinase kinase 1 OS=Rattus norvegicus GN=Map2k1 PE=1 SV=2	RAT	2	0.995493472	0.938468575	1.034150004	0.57138598
736	0.12	0.14	35.87000072	0	0	REVERSED Sulfhydryl oxidase 1 OS=Rattus norvegicus GN=Qsox1 PE=1 SV=1	RAT	0	0.995243609	0.951392889	0.94514209	0.493279696
306	2.03	4.06	46.43999934	13.3100003	13.3100003	Serine/threonine-protein phosphatase PP1-gamma catalytic subunit OS=Rattus norvegicus GN=Ppp1cc PE=1 SV=1	RAT	3	0.995197773	0.980352998	1.028516054	0.831161618
77	14.25	51.31	83.92000198	42.84000099	38.42999935	Sodium/potassium-transporting ATPase subunit alpha-2 OS=Rattus norvegicus GN=Atp1a2 PE=1 SV=1	RAT	37	0.995087087	0.919163883	0.892310679	0.037139811
8	46.56	46.66	90.78000188	63.47000003	57.31999874	ATP synthase subunit alpha, mitochondrial OS=Rattus norvegicus GN=Atp5a1 PE=1 SV=2	RAT	35	0.995061219	0.89782548	1.025866985	0.39722079
941	0.07	0.08	34.70999897	0	0	Zinc transporter 1 OS=Rattus norvegicus GN=Slc30a1 PE=2 SV=1	RAT	0	0.995048404	0.968115091	1.067996979	0.626863003
661	0.16	0.24	27.700001	2.195999958	0	Protein kinase C zeta type OS=Rattus norvegicus GN=Prkcz PE=1 SV=2	RAT	0	0.994620085	0.991590977	0.997584999	0.989159822
33	28.2	28.64	63.20000291	6.782999635	4.349999875	Cytoplasmic dynein 1 heavy chain 1 OS=Rattus norvegicus GN=Dync1h1 PE=1 SV=1	RAT	15	0.99449873	0.888477802	0.997701228	0.945263922
123	9.43	9.49	79.97000217	9.572999924	9.572999924	Stress-70 protein, mitochondrial OS=Rattus norvegicus GN=Hspa9 PE=1 SV=3	RAT	5	0.994247615	0.91098237	0.949112475	0.2396411
824	0.09	0.11	32.39000142	0	0	REVERSED Thyroid hormone receptor-associated protein 3 OS=Rattus norvegicus GN=Thrap3 PE=1 SV=1	RAT	0	0.994071722	0.944156587	0.915145516	0.300020307
102	11.15	11.15	86.3499999	29.24999893	29.24999893	Pyruvate dehydrogenase E1 component subunit beta, mitochondrial OS=Rattus norvegicus GN=Pdhb PE=1 SV=2	RAT	9	0.993634224	0.920999825	0.969798684	0.629157186
550	0.28	0.31	24.42000061	0.313799991	0	REVERSED Myosin-XVI OS=Rattus norvegicus GN=Myo16 PE=1 SV=1	RAT	0	0.99357301	0.943871617	0.856983483	0.197759807
193	5.09	17.21	96.31000161	44.63	39.9300009	ADP/ATP translocase 2 OS=Rattus norvegicus GN=Slc25a5 PE=1 SV=3	RAT	13	0.993274212	0.930114925	0.997117221	0.96996361
176	6.02	6.07	87.66999841	17.81000048	17.35000014	Histone H1.2 OS=Rattus norvegicus GN=Hist1h1c PE=1 SV=3	RAT	4	0.993227422	0.92140162	1.022686005	0.746170282
759	0.11	0.13	23.8499999	1.173999999	0	REVERSED Myosin-Ie OS=Rattus norvegicus GN=Myo1e PE=2 SV=1	RAT	0	0.993188918	0.940951824	0.999882817	0.999054372
782	0.1	0.15	35.85999906	0.509600015	0	REVERSED Microtubule-associated serine/threonine-protein kinase 1 OS=Rattus norvegicus GN=Mast1 PE=2 SV=1	RAT	0	0.993156075	0.95589292	1.178056002	0.44290629
587	0.23	0.23	20.2000007	2.356999926	0	REVERSED Zinc finger protein 467 OS=Rattus norvegicus GN=Znf467 PE=2 SV=1	RAT	0	0.99282378		1.01383698	
362	1.72	1.74	27.39000022	6.066000089	3.125	Serine/threonine-protein kinase PAK 1 OS=Rattus norvegicus GN=Pak1 PE=1 SV=3	RAT	1	0.992505372	0.949083626	0.949452221	0.561508298
216	4.09	4.12	57.66999722	5.96299991	2.638999932	2-oxoglutarate dehydrogenase, mitochondrial OS=Rattus norvegicus GN=Ogdh PE=1 SV=1	RAT	2	0.992422819	0.901443005	1.121929049	0.104978897
466	0.53	0.97	24.97999966	0.985999964	0	REVERSED Protein FAM65A OS=Rattus norvegicus GN=Fam65a PE=2 SV=2	RAT	0	0.992006779	0.948427081	1.11134398	0.479005694
1075	0.05	0.08	35.96999943	0	0	REVERSED Sterile alpha motif domain-containing protein 14 OS=Rattus norvegicus GN=Samd14 PE=2 SV=1	RAT	0	0.99199909	0.949243426	1.038051009	0.772363305
354	1.81	1.81	26.89999938	2.923999913	2.923999913	Sideroflexin-5 OS=Rattus norvegicus GN=Sfxn5 PE=2 SV=1	RAT	1	0.991988182		0.947470427	
570	0.24	0.53	36.35999858	0.191999995	0	REVERSED Plectin OS=Rattus norvegicus GN=Plec PE=1 SV=2	RAT	0	0.991747022	0.910027385	0.991934717	0.893553317
9	46.03	46.03	93.94000173	63.63999844	57.58000016	Syntaxin-binding protein 1 OS=Rattus norvegicus GN=Stxbp1 PE=1 SV=1	RAT	34	0.991323888	0.803119421	1.03164196	0.37852639
301	2.05	2.05	73.89000058	11.06000021	11.06000021	CD9 antigen OS=Rattus norvegicus GN=Cd9 PE=1 SV=2	RAT	1	0.991018713	0.976843894	1.005542994	0.968801677
721	0.13	0.13	35.17000079	0	0	Nucleolar RNA helicase 2 OS=Rattus norvegicus GN=Ddx21 PE=2 SV=1	RAT	0	0.990432322	0.93837899	1.004853964	0.968939304
93	12.34	16.65	80.00000119	45.1000005	36.86000109	14-3-3 protein epsilon OS=Rattus norvegicus GN=Ywhae PE=1 SV=1	RAT	13	0.990001678	0.846583605	0.989293814	0.820624471
280	2.24	7.49	81.27999902	15.60000032	10.30000001	Heat shock 70 kDa protein 1A/1B OS=Rattus norvegicus GN=Hspa1a PE=2 SV=2	RAT	5	0.98936069	0.96995163	1.165305018	0.659893811
967	0.06	0.18	30.36000133	0	0	REVERSED Receptor-type tyrosine-protein phosphatase S OS=Rattus norvegicus GN=Ptprs PE=1 SV=2	RAT	0	0.989074171	0.953522801	1.051843047	0.597717583
717	0.13	0.15	26.6900003	0	0	Myosin-binding protein C, cardiac-type OS=Rattus norvegicus GN=Mybpc3 PE=2 SV=2	RAT	0	0.988306522	0.914882302	1.040740013	0.618577778
11	45.12	45.12	97.68000245	54.3299973	45.35999894	Heat shock cognate 71 kDa protein OS=Rattus norvegicus GN=Hspa8 PE=1 SV=1	RAT	32	0.988248706	0.714067578	0.984521925	0.628891587
1048	0.05	0.16	29.53000069	0	0	Complement C4 OS=Rattus norvegicus GN=C4 PE=1 SV=3	RAT	0	0.988222122		1.136890054	
1025	0.06	0.06	34.97000039	0	0	REVERSED Cytosolic phospholipase A2 OS=Rattus norvegicus GN=Pla2g4a PE=2 SV=1	RAT	0	0.988141179	0.923969328	0.93751812	0.633484483
13	42.96	42.96	95.3700006	48.84000123	35.76000035	Dynamin-1 OS=Rattus norvegicus GN=Dnm1 PE=1 SV=2	RAT	28	0.987603307	0.710852802	1.024438024	0.475595802
117	9.86	9.87	57.81000257	10.61000004	9.441000223	Heat shock protein 105 kDa OS=Rattus norvegicus GN=Hsph1 PE=1 SV=1	RAT	6	0.987544775	0.875752628	1.016644955	0.836381495
264	2.71	2.71	25.27999878	4.473999888	4.473999888	Serine/threonine-protein phosphatase 2A 55 kDa regulatory subunit B alpha isoform OS=Rattus norvegicus GN=Ppp2r2a PE=2 SV=1	RAT	2	0.987512171	0.873254716	1.002004981	0.979669273
34	28.11	28.11	72.32999802	30.50000072	27.55999863	Hexokinase-1 OS=Rattus norvegicus GN=Hk1 PE=1 SV=4	RAT	22	0.987335026	0.682258189	1.009363055	0.764480114
284	2.16	2.22	74.91999865	1.006999984	1.006999984	Liprin-alpha-3 OS=Rattus norvegicus GN=Ppfia3 PE=1 SV=2	RAT	1	0.987135589	0.860304117	1.046879053	0.590899587
308	2.03	2.03	89.34999704	4.563000053	4.563000053	Proteasome subunit beta type-5 OS=Rattus norvegicus GN=Psmb5 PE=1 SV=3	RAT	1	0.986968875	0.940314174	1.041221023	0.763885498
209	4.17	4.17	59.53999758	13.98999989	8.141999692	Neuroplastin OS=Rattus norvegicus GN=Nptn PE=1 SV=2	RAT	4	0.986853302	0.876347899	1.029284954	0.793339789
493	0.42	0.42	46.65000141	3.795000166	0	UPF0667 protein C1orf55 homolog OS=Rattus norvegicus PE=2 SV=2	RAT	0	0.986802876	0.914987326	0.944421589	0.717778206
546	0.3	0.3	36.35999858	4.544999823	0	60S ribosomal protein L18a OS=Rattus norvegicus GN=Rpl18a PE=2 SV=1	RAT	0	0.986703694		0.931434274	
275	2.38	2.38	54.6299994	16.30000025	4.405000061	Cytochrome c oxidase subunit 2 OS=Rattus norvegicus GN=Mtco2 PE=2 SV=2	RAT	1	0.986537397	0.889335573	1.001574039	0.995856822
23	35.51	35.59	95.99000216	51.66000128	50.78999996	60 kDa heat shock protein, mitochondrial OS=Rattus norvegicus GN=Hspd1 PE=1 SV=1	RAT	23	0.98623991	0.838886082	0.974530995	0.483400792
705	0.14	0.14	17.09000021	1.453000028	0	Ral guanine nucleotide dissociation stimulator OS=Rattus norvegicus GN=Ralgds PE=1 SV=1	RAT	0	0.986204684		0.865632892	
145	7.48	7.48	82.88000226	47.94999957	42.46999919	Cytochrome c oxidase subunit 5A, mitochondrial OS=Rattus norvegicus GN=Cox5a PE=1 SV=1	RAT	6	0.986078084	0.922420919	0.957780123	0.640558124
63	16.96	16.96	84.99000072	36.32000089	25.90999901	Aspartate aminotransferase, cytoplasmic OS=Rattus norvegicus GN=Got1 PE=1 SV=3	RAT	9	0.985431671	0.723308384	1.029863954	0.484152287
98	11.37	11.56	62.51000166	10.27000025	8.516000211	Solute carrier family 12 member 5 OS=Rattus norvegicus GN=Slc12a5 PE=1 SV=2	RAT	7	0.985414326	0.825491905	1.001798034	0.978565395
757	0.11	0.15	35.46999991	0.57529998	0	Glutamate receptor-interacting protein 2 OS=Rattus norvegicus GN=Grip2 PE=1 SV=1	RAT	0	0.9853158	0.905449271	0.963023722	0.768421888
518	0.35	0.42	28.72999907	0.914600026	0	DNA repair protein RAD50 OS=Rattus norvegicus GN=Rad50 PE=1 SV=1	RAT	0	0.984441578	0.87671262	1.084445953	0.426361293
456	0.57	0.61	56.61000013	6.102000177	0	Voltage-dependent anion-selective channel protein 2 OS=Rattus norvegicus GN=Vdac2 PE=1 SV=2	RAT	0	0.984350622	0.899398327	1.004521012	0.97097671
1053	0.05	0.12	64.67999816	0	0	REVERSED Liprin-alpha-3 OS=Rattus norvegicus GN=Ppfia3 PE=1 SV=2	RAT	0	0.984278381	0.836647391	1.078891993	0.530363798
616	0.2	0.36	34.36000049	0.634799991	0	Protein bassoon OS=Rattus norvegicus GN=Bsn PE=1 SV=3	RAT	0	0.984057307	0.811165929	0.952144504	0.480929792
849	0.08	2.3	57.92999864	0.713900011	0.713900011	Myosin-9 OS=Rattus norvegicus GN=Myh9 PE=1 SV=3	RAT	2	0.983977675	0.880172312	0.940906823	0.454240888

593	0.22	0.25	34.34000015	0.552800018	0	Transient receptor potential cation channel subfamily M member 1 OS=Rattus norvegicus GN=Trpm1 PE=2 SV=1	RAT	0	0.983976722		0.910471618	
267	2.63	2.68	43.99999976	1.190999988	0.714900019	Neural cell adhesion molecule L1 OS=Rattus norvegicus GN=L1cam PE=2 SV=3	RAT	1	0.983450115	0.830182612	0.961935222	0.663745224
620	0.2	0.23	25.27999878	0.966900028	0	Phosphatidylinositol 3-kinase regulatory subunit alpha OS=Rattus norvegicus GN=Pik3r1 PE=1 SV=1	RAT	0	0.982904375		0.938428819	
247	3.1	3.12	91.54000282	23.8499999	21.53999954	Histone H2A type 1 OS=Rattus norvegicus PE=1 SV=2	RAT	2	0.982669711	0.921072185	0.98918277	0.895957112
68	16	23.85	83.74999762	60.61000228	46.00999951	Fructose-bisphosphate aldolase C OS=Rattus norvegicus GN=Aldoc PE=1 SV=3	RAT	24	0.982363224	0.775461078	0.983645797	0.765115321
110	10.51	10.52	79.49000001	11.15000024	10.00000015	6-phosphofructokinase, muscle type OS=Rattus norvegicus GN=Pfkm PE=2 SV=3	RAT	6	0.98227489	0.847940326	0.986756027	0.829040587
257	2.87	2.98	48.67999852	7.301999629	6.491000205	Solute carrier family 2, facilitated glucose transporter member 3 OS=Rattus norvegicus GN=Slc2a3 PE=1 SV=1	RAT	2	0.982264519	0.886515319	1.000277996	0.998227894
234	3.56	3.56	59.54999924	46.07000053	32.58000016	Dynein light chain 1, cytoplasmic OS=Rattus norvegicus GN=Dynll1 PE=1 SV=1	RAT	2	0.982134819	0.850742996	0.928683579	0.473764002
266	2.67	2.67	61.37999892	2.89699994	1.516999956	Beta-adducin OS=Rattus norvegicus GN=Add2 PE=2 SV=4	RAT	1	0.981517076	0.876599014	1.039162993	0.671899199
297	2.06	2.06	79.11000252	3.39399986	3.39399986	Paralemmin-1 OS=Rattus norvegicus GN=Palm PE=1 SV=1	RAT	1	0.981405079	0.897975802	1.042613029	0.773621619
148	7.36	7.4	59.50000286	10.98000035	10.98000035	Glutaminase kidney isoform, mitochondrial OS=Rattus norvegicus GN=Gls PE=1 SV=2	RAT	4	0.981338382	0.854092419	0.980807483	0.790387273
440	0.65	0.65	22.46000022	0.33160001	0	REVERSED Cadherin-23 OS=Rattus norvegicus GN=Cdh23 PE=2 SV=1	RAT	0	0.98117429	0.917794228	1.286676049	0.3272928
659	0.17	0.17	4.681000113	4.681000113	0	Secretory carrier-associated membrane protein 5 OS=Rattus norvegicus GN=Scamp5 PE=2 SV=1	RAT	0	0.980655789		1.045559049	
169	6.38	6.39	84.82999802	20.50999999	13.4800002	Phosphate carrier protein, mitochondrial OS=Rattus norvegicus GN=Slc25a3 PE=1 SV=1	RAT	3	0.980629325	0.736158192	1.036985993	0.534676194
1015	0.06	0.07	39.01999891	0	0	REVERSED Bcl2 antagonist of cell death OS=Rattus norvegicus GN=Bad PE=1 SV=2	RAT	0	0.980217099		1.165603042	
65	16.73	16.73	87.52999902	46.03999853	35.96999943	Phosphoglycerate kinase 1 OS=Rattus norvegicus GN=Pgk1 PE=1 SV=2	RAT	9	0.980033398	0.615731478	0.977118373	0.56560123
1051	0.05	0.13	20.90999931	0	0	Latrophilin-2 OS=Rattus norvegicus GN=Lphn2 PE=1 SV=2	RAT	0	0.979925215	0.871245384	0.877884328	0.413372695
131	8.43	8.5	90.31999707	27.82000005	24.1899997	Tropomyosin alpha-3 chain OS=Rattus norvegicus GN=Tpm3 PE=1 SV=2	RAT	5	0.9798733	0.739705682	0.94496119	0.372145504
386	1.28	1.52	13.86000067	6.931000203	4.949999973	Leukocyte surface antigen CD47 OS=Rattus norvegicus GN=Cd47 PE=1 SV=1	RAT	1	0.979749084	0.877988219	1.071928978	0.630401373
119	9.83	21.18	83.75999928	40.77000022	34.86999869	Calcium/calmodulin-dependent protein kinase type II subunit beta OS=Rattus norvegicus GN=Camk2b PE=1 SV=1	RAT	37	0.979102075	0.697569013	1.005290031	0.922339082
609	0.21	0.21	15.18999934	3.164999932	0	C1GALT1-specific chaperone 1 OS=Rattus norvegicus GN=C1galt1c1 PE=1 SV=1	RAT	0	0.978785813		1.142254949	
84	12.92	12.96	70.34999728	27.64999866	23.22999984	Cytochrome b-c1 complex subunit 2, mitochondrial OS=Rattus norvegicus GN=Uqcrc2 PE=1 SV=2	RAT	14	0.978699327	0.767671227	1.044862986	0.505959392
591	0.22	0.3	26.26999915	0.599400001	0	REVERSED Serine/threonine-protein kinase TAO1 OS=Rattus norvegicus GN=Taok1 PE=1 SV=1	RAT	0	0.978559494	0.866641402	0.946485698	0.686116397
673	0.16	0.16	51.17999911	2.693999931	0	Calponin-1 OS=Rattus norvegicus GN=Cnn1 PE=2 SV=1	RAT	0	0.978547513		0.90425092	
594	0.22	0.23	24.86000061	4.926000163	0	REVERSED Programmed cell death 6-interacting protein OS=Rattus norvegicus GN=Pdcd6ip PE=1 SV=2	RAT	0	0.977701187	0.743263006	0.894599974	0.202945501
1052	0.05	0.13	27.73000002	3.150999919	0	REVERSED Non-POU domain-containing octamer-binding protein OS=Rattus norvegicus GN=Nono PE=2 SV=3	RAT	0	0.977551877		1.010131001	
201	4.43	4.43	72.5399971	46.63000107	16.06000066	Transforming protein RhoA OS=Rattus norvegicus GN=Rhoa PE=1 SV=1	RAT	2	0.977309883	0.787982285	0.940066218	0.487648606
393	1.15	1.28	32.4000001	5.361000076	0	Elongation factor 2 OS=Rattus norvegicus GN=Eef2 PE=1 SV=4	RAT	0	0.977287829	0.799238026	1.009209037	0.910879791
971	0.06	0.16	30.41999936	0.316899992	0	REVERSED Neurexin-3-alpha OS=Rattus norvegicus GN=Nrxn3 PE=1 SV=1	RAT	0	0.976634085	0.850765526	0.901922524	0.486226797
1037	0.06	0.06	20.58999985	7.353000343	0	60S ribosomal protein L27 OS=Rattus norvegicus GN=Rpl27 PE=2 SV=2	RAT	0	0.976477385		0.999210715	
559	0.27	0.28	29.6600014	3.145999834	0	Oxysterols receptor LXR-alpha OS=Rattus norvegicus GN=Nr1h3 PE=2 SV=1	RAT	0	0.975858629		0.940033019	
825	0.09	0.11	46.81000113	0	0	Coiled-coil domain-containing protein 8 OS=Rattus norvegicus GN=Ccdc8 PE=2 SV=1	RAT	0	0.975849926	0.75162369	0.937445283	0.42588231
106	10.9	10.95	83.1099987	12.63000071	6.649000198	Microtubule-associated protein tau OS=Rattus norvegicus GN=Mapt PE=1 SV=3	RAT	5	0.975802124	0.580430984	0.926401794	0.100604199
246	3.17	3.17	89.99999762	18.56999993	18.56999993	Profilin-1 OS=Rattus norvegicus GN=Pfn1 PE=1 SV=2	RAT	3	0.975750029	0.884545803	0.91848582	0.406249195
333	2	2	42.08000004	6.334999949	6.334999949	Fumarylacetoacetate hydrolase domain-containing protein 1 OS=Rattus norvegicus GN=Fahd1 PE=2 SV=1	RAT	1	0.975670576		0.935802221	
133	8.24	8.27	60.08999944	23.38999975	14.67999965	Septin-7 OS=Rattus norvegicus GN=Sept7 PE=1 SV=1	RAT	5	0.975378513	0.812268913	0.999422729	0.994992018
564	0.25	0.28	31.67999983	3.649999946	1.606000029	Stromal interaction molecule 1 OS=Rattus norvegicus GN=Stim1 PE=1 SV=1	RAT	1	0.975335121		1.118684053	
441	0.65	0.65	31.18999898	1.948999986	1.948999986	REVERSED WD40 repeat-containing protein SMU1 OS=Rattus norvegicus GN=Smu1 PE=2 SV=1	RAT	1	0.975040674	0.786430418	1.10265696	0.354905605
45	22.16	29.06	97.69999981	57.59999752	54.61000204	Alpha-enolase OS=Rattus norvegicus GN=Eno1 PE=1 SV=4	RAT	34	0.975009799	0.633777022	1.007230997	0.861540794
70	15.46	15.46	90.96000195	43.61000061	43.61000061	Dihydrolipoyl dehydrogenase, mitochondrial OS=Rattus norvegicus GN=Dld PE=1 SV=1	RAT	15	0.974986315	0.670411408	0.996661007	0.954879522
964	0.06	0.21	26.84000134	0	0	REVERSED Dynein heavy chain 12, axonemal OS=Rattus norvegicus GN=Dnah12 PE=2 SV=2	RAT	0	0.974918604		1.14329505	
698	0.14	0.17	27.79000103	1.420000009	0	REVERSED Cysteine sulfinic acid decarboxylase OS=Rattus norvegicus GN=Csad PE=1 SV=1	RAT	0	0.974850893	0.741534412	1.048959017	0.602779329
44	22.38	22.41	95.57999969	67.47000217	64.6600008	Triosephosphate isomerase OS=Rattus norvegicus GN=Tpi1 PE=1 SV=2	RAT	20	0.974788308	0.532869875	0.936096489	0.119934604
198	4.72	4.72	75	27.9399991	27.9399991	Rho GDP-dissociation inhibitor 1 OS=Rattus norvegicus GN=Arhgdia PE=1 SV=1	RAT	3	0.974239171	0.803889513	1.012403965	0.906301498
584	0.23	0.25	44.06000078	1.013999991	0	Heterogeneous nuclear ribonucleoprotein M OS=Rattus norvegicus GN=Hnrnpm PE=1 SV=4	RAT	0	0.974008083	0.741448283	0.993994296	0.939101517
132	8.29	8.31	85.42000055	42.71000028	37.85000145	Syntaxin-1A OS=Rattus norvegicus GN=Stx1a PE=1 SV=1	RAT	7	0.97350502	0.605242908	1.011477947	0.825885773
554	0.28	0.28	21.92000002	5.384999886	0	REVERSED Keratin, type II cytoskeletal 72 OS=Rattus norvegicus GN=Krt72 PE=2 SV=2	RAT	0	0.97346127	0.933052421	1.028010011	0.910446584
1042	0.05	7.8	72.72999883	11.96999997	7.594999671	Dynamin-3 OS=Rattus norvegicus GN=Dnm3 PE=1 SV=2	RAT	6	0.973460078	0.774670422	1.009376049	0.923401713
37	26.65	57.08	78.49000096	43.39999855	41.3500011	Sodium/potassium-transporting ATPase subunit alpha-1 OS=Rattus norvegicus GN=Atp1a1 PE=1 SV=1	RAT	44	0.973163724	0.447679788	0.973587871	0.454730809
1022	0.06	0.06	46.12999856	0	0	REVERSED Mitochondria-eating protein OS=Rattus norvegicus GN=Spata18 PE=2 SV=1	RAT	0	0.973064184	0.830216825	1.026116014	0.839752793
645	0.18	0.18	35.13000011	1.229999959	0	Collagen alpha-1(III) chain OS=Rattus norvegicus GN=Col3a1 PE=2 SV=3	RAT	0	0.973039687	0.725503922	1.076429963	0.464850307
55	18.76	19.18	93.66999865	53.3100009	45.48000097	L-lactate dehydrogenase A chain OS=Rattus norvegicus GN=Ldha PE=1 SV=1	RAT	15	0.972809792	0.63335079	1.171931982	0.110588998
534	0.32	3.63	75.7799983	5.401000008	5.401000008	Pyruvate kinase isozymes R/L OS=Rattus norvegicus GN=Pklr PE=2 SV=2	RAT	3	0.971983492	0.840860605	0.962988913	0.905939698
933	0.07	0.08	76.27000213	3.489999846	0	REVERSED 60 kDa heat shock protein, mitochondrial OS=Rattus norvegicus GN=Hspd1 PE=1 SV=1	RAT	0	0.971583486	0.825700581	1.050173998	0.719717085
82	13.5	13.5	71.27000093	46.41000032	45.30000091	ADP-ribosylation factor 3 OS=Rattus norvegicus GN=Arf3 PE=1 SV=2	RAT	10	0.97154671	0.576904476	1.046587944	0.419223011
948	0.07	0.07	21.95000052	0	0	REVERSED Kinesin-like protein KIF27 OS=Rattus norvegicus GN=Kif27 PE=2 SV=1	RAT	0	0.970865726	0.75556308	0.902993321	0.344179392
815	0.09	0.13	29.39999998	0	0	REVERSED Coiled-coil domain-containing protein 105 OS=Rattus norvegicus GN=Ccdc105 PE=2 SV=1	RAT	0	0.970733285	0.889638126	0.947937429	0.823098302
995	0.06	0.08	36.66000068	0	0	Adenomatous polyposis coli protein OS=Rattus norvegicus GN=Apc PE=1 SV=1	RAT	0	0.970674574	0.805154026	0.976522088	0.84483099
708	0.13	0.24	59.03999805	0	0	REVERSED Cytoplasmic dynein 1 heavy chain 1 OS=Rattus norvegicus GN=Dync1h1 PE=1 SV=1	RAT	0	0.970571518	0.831183016	0.995889723	0.968135476
239	3.38	3.5	34.41999853	7.599999756	7.599999756	Sodium/calcium exchanger 2 OS=Rattus norvegicus GN=Slc8a2 PE=2 SV=1	RAT	4	0.970506012	0.704011083	0.971323609	0.711732626
329	2	2	37.67000139	2.195999958	2.195999958	Bifunctional purine biosynthesis protein PURH OS=Rattus norvegicus GN=Atic PE=1 SV=2	RAT	1	0.970382512		0.864629328	
324	2	2.04	46.05999887	7.570999861	7.570999861	Protein IMPACT OS=Rattus norvegicus GN=Impact PE=2 SV=1	RAT	1	0.970130026		1.006880045	
603	0.21	0.37	28.16999853	1.131999958	0	REVERSED GRIP and coiled-coil domain-containing protein 2 OS=Rattus norvegicus GN=Gcc2 PE=1 SV=1	RAT	0	0.9701249	0.662336111	1.054774046	0.457289785
695	0.14	0.17	69.48000193	0	0	REVERSED Microtubule-associated protein 2 OS=Rattus norvegicus GN=Map2 PE=1 SV=3	RAT	0	0.969930172	0.751895189	0.958696187	0.498968691
958	0.07	0.07	23.96000028	6.25	0	Interferon alpha-1 OS=Rattus norvegicus GN=Ifna1 PE=3 SV=1	RAT	0	0.969797611		0.989167511	
334	2	2	41.96000099	5.490000173	5.490000173	Proteasome subunit alpha type-3 OS=Rattus norvegicus GN=Psma3 PE=1 SV=3	RAT	1	0.969793677		1.065698981	
607	0.21	0.21	30.89999855	0.433199992	0	Kinesin-like protein KIF15 OS=Rattus norvegicus GN=Kif15 PE=2 SV=1	RAT	0	0.969576597	0.807465971	0.984045386	0.911687315
752	0.12	0.12	31.60000145	5.18900007	0	Peroxisomal membrane protein 4 OS=Rattus norvegicus GN=Pxmp4 PE=1 SV=2	RAT	0	0.969477713	0.73791182	1.005612969	0.964272022
232	3.61	3.61	81.37000203	57.13999867	37.88999915	ATP synthase subunit d, mitochondrial OS=Rattus norvegicus GN=Atp5h PE=1 SV=3	RAT	3	0.969290078	0.85177052	0.996293485	0.987601578
700	0.14	0.14	36.10999882	1.697999984	0	Rab11 family-interacting protein 1 OS=Rattus norvegicus GN=Rab11fip1 PE=2 SV=1	RAT	0	0.968951821	0.803489089	1.042094946	0.779737115
373	1.48	1.48	31.60000145	11.32000014	11.32000014	Ras-related protein Rab-2A OS=Rattus norvegicus GN=Rab2a PE=1 SV=1	RAT	2	0.968812823	0.805870295	1.08416605	0.568556726
451	0.6	0.68	30.25000095	1.341000013	0	Protein kinase C beta type OS=Rattus norvegicus GN=Prkcb PE=1 SV=3	RAT	0	0.968629718	0.80997771	1.015406966	0.843720913
371	1.58	1.62	30.23000062	2.305999957	1.317999978	Neuronal cell adhesion molecule OS=Rattus norvegicus GN=Nrcam PE=1 SV=2	RAT	1	0.968109429	0.799712896	0.946091771	0.678913116
1072	0.05	0.08	29.15000021	0	0	Myosin phosphatase Rho-interacting protein OS=Rattus norvegicus GN=Mprip PE=1 SV=1	RAT	0	0.968071878	0.798870206	1	1

851	0.08	0.28	35.85000038	0.711200014	0	ProSAP-interacting protein 1 OS=Rattus norvegicus GN=Prosapip1 PE=1 SV=2	RAT	0	0.968009591	0.805065811	0.987800598	0.923601627
175	6.04	6.04	89.85000253	20.29999942	20.29999942	ES1 protein homolog, mitochondrial OS=Rattus norvegicus PE=1 SV=2	RAT	3	0.967970312	0.786729872	0.930733621	0.474440008
302	2.04	46.85	86.71000004	75.90000033	71.61999941	Tubulin beta-5 chain OS=Rattus norvegicus GN=Tubb5 PE=1 SV=1	RAT	94	0.967906713	0.79741329	0.922650516	0.565843701
387	1.24	1.24	58.60000253	5.09600006	5.09600006	60S ribosomal protein L24 OS=Rattus norvegicus GN=Rpl24 PE=1 SV=1	RAT	1	0.967903018		1.000349045	
188	5.48	5.5	70.38999796	41.44999981	28.94999981	Nucleoside diphosphate kinase B OS=Rattus norvegicus GN=Nme2 PE=1 SV=1	RAT	3	0.967778921	0.65557611	1.053693056	0.483937204
542	0.31	0.33	30.91000021	0.525100017	0	Aldehyde oxidase OS=Rattus norvegicus GN=Aox1 PE=2 SV=1	RAT	0	0.967671871	0.713339984	1.016567945	0.891376913
1104	0.05	0.06	38.90999854	0.997800007	0	REVERSED Aldehyde dehydrogenase family 1 member L1 OS=Rattus norvegicus GN=Aldh1l1 PE=1 SV=2	RAT	0	0.967649281		0.962325394	
103	11.02	11.05	85.32999754	38.9200002	31.43999875	Malate dehydrogenase, cytoplasmic OS=Rattus norvegicus GN=Mdh1 PE=1 SV=3	RAT	6	0.967433572	0.515432715	0.988850296	0.843676507
922	0.07	0.09	39.77999985	0	0	Collagen alpha-1(I) chain OS=Rattus norvegicus GN=Col1a1 PE=1 SV=5	RAT	0	0.967031777	0.719694614	1.081823945	0.436230391
795	0.1	0.11	22.63000011	0.729899993	0	REVERSED Basic fibroblast growth factor receptor 1 OS=Rattus norvegicus GN=Fgfr1 PE=1 SV=1	RAT	0	0.966505289	0.875490487	0.929310203	0.593926787
581	0.23	0.37	24.05000031	0.307999994	0	E3 ubiquitin-protein ligase UBR4 OS=Rattus norvegicus GN=Ubr4 PE=2 SV=2	RAT	0	0.966417193	0.832500517	0.911134005	0.172050402
273	2.45	2.45	58.57999921	23.08000028	10.64999998	Cytochrome c oxidase subunit 4 isoform 1, mitochondrial OS=Rattus norvegicus GN=Cox4i1 PE=1 SV=1	RAT	2	0.966286302	0.661366701	0.934207201	0.408114403
199	4.49	5.38	82.84000158	55.22000194	55.22000194	Complexin-2 OS=Rattus norvegicus GN=Cplx2 PE=1 SV=1	RAT	5	0.965791225	0.792116225	1.018512964	0.888541877
980	0.06	0.11	42.96999872	0	0	REVERSED Coiled-coil domain-containing protein 61 OS=Rattus norvegicus GN=Ccdc61 PE=2 SV=1	RAT	0	0.96559298	0.784027219	0.985188782	0.905048311
946	0.07	0.07	84.93999839	0	0	REVERSED Synapsin-1 OS=Rattus norvegicus GN=Syn1 PE=1 SV=3	RAT	0	0.965540111	0.804887712	0.900367618	0.405667514
981	0.06	0.11	41.13000035	0	0	Egl nine homolog 3, mitochondrial OS=Rattus norvegicus GN=Egln3 PE=1 SV=1	RAT	0	0.965367496	0.759817004	1.010475993	0.947017014
915	0.07	0.1	71.49999738	0	0	REVERSED Dihydropyrimidinase-related protein 1 OS=Rattus norvegicus GN=Crmp1 PE=1 SV=1	RAT	0	0.9650262	0.823039412	1.066689014	0.776412129
496	0.4	0.42	30.03999889	0.925900042	0	REVERSED Microtubule-associated protein 1S OS=Rattus norvegicus GN=Map1s PE=1 SV=1	RAT	0	0.964924872	0.779703021	0.964039505	0.774629891
126	8.9	8.96	60.86999774	51.09000206	51.09000206	Protein S100-B OS=Rattus norvegicus GN=S100b PE=1 SV=2	RAT	7	0.964744389	0.611605883	0.964996874	0.677206695
272	2.5	2.5	62.18000054	8.807999641	5.181000009	Neuron-specific calcium-binding protein hippocalcin OS=Rattus norvegicus GN=Hpca PE=1 SV=2	RAT	1	0.964421511	0.722011387	1.077079058	0.489458591
21	36.18	36.18	98.18000197	89.09000158	89.09000158	Brain acid soluble protein 1 OS=Rattus norvegicus GN=Basp1 PE=1 SV=2	RAT	26	0.964012504	0.387872189	1.094099045	0.024524409
1078	0.05	0.07	29.03999984	0	0	Cadherin EGF LAG seven-pass G-type receptor 3 OS=Rattus norvegicus GN=Celsr3 PE=2 SV=1	RAT	0	0.963543773	0.833001673	0.974510789	0.837654889
566	0.25	0.25	33.28000009	2.996000089	0	REVERSED Insulin receptor substrate 1 OS=Rattus norvegicus GN=Irs1 PE=1 SV=1	RAT	0	0.963348508		0.94748491	
92	12.42	12.44	78.14000249	37.45000064	27.05999911	Elongation factor 1-alpha 1 OS=Rattus norvegicus GN=Eef1a1 PE=1 SV=1	RAT	11	0.963263988	0.638782322	1.02206099	0.78060317
62	17.18	17.18	95.12000288	70.73000073	70.73000073	Peptidyl-prolyl cis-trans isomerase A OS=Rattus norvegicus GN=Ppia PE=1 SV=2	RAT	20	0.963214815	0.45234251	0.97356391	0.702111304
87	12.71	12.71	82.01000094	42.86000133	42.86000133	Protein DJ-1 OS=Rattus norvegicus GN=Park7 PE=1 SV=1	RAT	7	0.962849915	0.647551119	0.934530377	0.40489769
1003	0.06	0.08	14.18000013	0	0	REVERSED Chaperone activity of bc1 complex-like, mitochondrial OS=Rattus norvegicus GN=Adck3 PE=2 SV=1	RAT	0	0.962432325	0.682323873	1.037832975	0.731847227
651	0.17	0.21	30.18999994	1.391999982	0	Inositol-trisphosphate 3-kinase B OS=Rattus norvegicus GN=Itpkb PE=1 SV=3	RAT	0	0.962379873	0.632945716	1.088958025	0.322339296
829	0.09	0.11	32.39000142	1.408000011	0	REVERSED Angiopoietin-1 OS=Rattus norvegicus GN=Angpt1 PE=2 SV=2	RAT	0	0.962101102		0.968330681	
813	0.09	0.14	23.01999927	0.47129998	0	REVERSED Receptor-type tyrosine-protein phosphatase C OS=Rattus norvegicus GN=Ptprc PE=2 SV=2	RAT	0	0.962076724		1.142475963	
277	2.33	2.33	34.74000096	4.008999839	2.226999961	Heterogeneous nuclear ribonucleoprotein H2 OS=Rattus norvegicus GN=Hnrnph2 PE=1 SV=1	RAT	1	0.961784422	0.811255693	1.010239959	0.941784978
228	3.72	3.72	45.3700006	37.04000115	28.2400012	GTP-binding nuclear protein Ran OS=Rattus norvegicus GN=Ran PE=1 SV=3	RAT	2	0.961725116	0.74447763	0.948133707	0.65028882
89	12.62	12.69	87.38999963	24.68000054	21.63999975	Microtubule-associated protein 6 OS=Rattus norvegicus GN=Map6 PE=1 SV=1	RAT	7	0.960578084	0.522461295	1.040583014	0.727204025
575	0.24	0.25	22.99000025	2.135000005	0	Tyrosyl-DNA phosphodiesterase 1 OS=Rattus norvegicus GN=Tdp1 PE=2 SV=1	RAT	0	0.960488021	0.80458349	0.924522579	0.682302773
1066	0.05	0.09	17.3299998	0	0	REVERSED Aldehyde dehydrogenase family 16 member A1 OS=Rattus norvegicus GN=Aldh16a1 PE=2 SV=1	RAT	0	0.960456073	0.612435579	1.002526045	0.97421062
783	0.1	0.14	38.49999905	0	0	Roundabout homolog 4 OS=Rattus norvegicus GN=Robo4 PE=2 SV=1	RAT	0	0.960282922	0.760138929	0.954583287	0.721868277
411	0.87	0.87	52.38000154	37.5	8.332999796	ATP synthase subunit delta, mitochondrial OS=Rattus norvegicus GN=Atp5d PE=1 SV=2	RAT	1	0.959799528		1.10857904	
181	5.79	5.96	64.01000023	20.83999962	12.98999935	LETM1 and EF-hand domain-containing protein 1, mitochondrial OS=Rattus norvegicus GN=Letm1 PE=1 SV=1	RAT	4	0.959640026	0.655704319	0.956743479	0.526490271
746	0.12	0.12	22.12000042	0.611899979	0	Adenylate cyclase type 3 OS=Rattus norvegicus GN=Adcy3 PE=2 SV=1	RAT	0	0.959529102	0.830349386	1.097960949	0.517926216
761	0.11	0.13	42.68999994	3.880999982	0	REVERSED DNA polymerase beta OS=Rattus norvegicus GN=Polb PE=1 SV=4	RAT	0	0.959510088	0.747839987	0.955089211	0.722957671
1102	0.05	0.06	23.63999933	0	0	REVERSED Multidrug resistance-associated protein 6 OS=Rattus norvegicus GN=Abcc6 PE=2 SV=1	RAT	0	0.959296584	0.753838778	1.022053003	0.866408825
729	0.12	0.18	46.43000066	1.817999966	0	REVERSED Caskin-1 OS=Rattus norvegicus GN=Caskin1 PE=1 SV=1	RAT	0	0.959087312	0.676990271	0.980904222	0.845613301
190	5.18	5.18	50.67999959	9.353999794	5.612000078	Myc box-dependent-interacting protein 1 OS=Rattus norvegicus GN=Bin1 PE=1 SV=1	RAT	3	0.958946884	0.618790686	1.000247002	0.997592211
714	0.13	0.17	42.86000133	1.706000045	0	REVERSED SHC-transforming protein 1 OS=Rattus norvegicus GN=Shc1 PE=1 SV=1	RAT	0	0.958623528	0.742921412	1.085510015	0.559294105
289	2.1	2.1	92.47000217	15.07000029	6.848999858	40S ribosomal protein S16 OS=Rattus norvegicus GN=Rps16 PE=1 SV=2	RAT	1	0.958469927	0.831090212	0.817988217	0.351680398
167	6.56	6.56	46.30999863	30.86999953	30.86999953	Cell cycle exit and neuronal differentiation protein 1 OS=Rattus norvegicus GN=Cend1 PE=1 SV=1	RAT	3	0.958463788	0.679507315	0.997569978	0.980430722
95	11.67	11.67	82.99000263	41.67000055	38.53999972	Syntaxin-1B OS=Rattus norvegicus GN=Stx1b PE=1 SV=1	RAT	9	0.9583956	0.462372094	0.984187186	0.779815018
81	13.51	13.51	76.45999789	29.17000055	27.5000006	Cytochrome b-c1 complex subunit 1, mitochondrial OS=Rattus norvegicus GN=Uqcrc1 PE=1 SV=1	RAT	10	0.958176315	0.782633305	0.947777271	0.531863928
1107	0.05	0.06	46.2500006	0	0	REVERSED Lysophosphatidylcholine acyltransferase 1 OS=Rattus norvegicus GN=Lpcat1 PE=2 SV=2	RAT	0	0.958077729		1.16980505	
792	0.1	0.12	29.91000116	2.20199991	0	T-complex protein 1 subunit gamma OS=Rattus norvegicus GN=Cct3 PE=1 SV=1	RAT	0	0.957371175	0.768284976	1.006368995	0.979498625
174	6.05	6.05	44.78000104	7.710999995	7.710999995	Endoplasmin OS=Rattus norvegicus GN=Hsp90b1 PE=1 SV=2	RAT	3	0.957020283	0.744358003	0.8678689	0.399053305
10	45.54	45.54	93.95999908	82.41999745	74.44999814	Fructose-bisphosphate aldolase A OS=Rattus norvegicus GN=Aldoa PE=1 SV=2	RAT	38	0.956901371	0.218024105	0.973772407	0.455123186
928	0.07	0.09	45.26999891	0	0	REVERSED Glycerol-3-phosphate dehydrogenase [NAD+], cytoplasmic OS=Rattus norvegicus GN=Gpd1 PE=1 SV=4	RAT	0	0.956725478	0.82787329	0.978942215	0.917147279
571	0.24	0.3	17.97000021	0.435299985	0	Adenylate cyclase type 10 OS=Rattus norvegicus GN=Adcy10 PE=1 SV=1	RAT	0	0.956610918	0.732608199	1.080131054	0.579168499
1045	0.05	0.24	24.86000061	0	0	REVERSED E3 ubiquitin-protein ligase UBR4 OS=Rattus norvegicus GN=Ubr4 PE=2 SV=2	RAT	0	0.95607692	0.64870882	0.996695578	0.968023598
604	0.21	0.25	30.23999929	0.337300007	0	Fibronectin type III domain-containing protein 1 OS=Rattus norvegicus GN=Fndc1 PE=2 SV=2	RAT	0	0.955832779	0.727062881	0.936571121	0.627390921
437	0.67	0.67	28.67999971	0.744900014	0.744900014	Phospholipase D1 OS=Rattus norvegicus GN=Pld1 PE=1 SV=3	RAT	1	0.955758214	0.863692224	1.144569039	0.507833183
238	3.43	3.48	48.96000028	21.87999934	9.89599973	Ras-related C3 botulinum toxin substrate 1 OS=Rattus norvegicus GN=Rac1 PE=1 SV=1	RAT	2	0.955659091	0.573747873	0.97975558	0.794767618
240	3.38	3.49	42.28999913	2.411000058	1.279999968	Plectin OS=Rattus norvegicus GN=Plec PE=1 SV=2	RAT	3	0.95505178	0.1948331	0.958591282	0.233139098
909	0.07	0.12	30.07999957	0.723099988	0	REVERSED Insulin receptor OS=Rattus norvegicus GN=Insr PE=1 SV=1	RAT	0	0.954245389	0.790885389	1.08584094	0.568855226
282	2.2	2.23	86.04999781	22.48000056	10.08000001	Cytochrome c oxidase subunit 5B, mitochondrial OS=Rattus norvegicus GN=Cox5b PE=1 SV=2	RAT	1	0.954159081	0.551425278	0.940423071	0.447279394
259	2.8	2.86	68.48000288	29.69999909	18.17999929	Destrin OS=Rattus norvegicus GN=Dstn PE=1 SV=3	RAT	2	0.953928828	0.606075108	1.029356956	0.719701827
937	0.07	0.08	36.55999899	1.343999989	0	Tripartite motif-containing protein 3 OS=Rattus norvegicus GN=Trim3 PE=1 SV=1	RAT	0	0.953653693		0.962058902	
134	8.22	8.22	71.49000168	16.77999943	14.93999958	AP-2 complex subunit mu OS=Rattus norvegicus GN=Ap2m1 PE=1 SV=1	RAT	7	0.953638196	0.495815009	0.941062689	0.393182695
968	0.06	0.18	38.02999854	0.345700001	0	Voltage-dependent calcium channel subunit alpha-2/delta-2 OS=Rattus norvegicus GN=Cacna2d2 PE=2 SV=1	RAT	0	0.952366829	0.6102705	0.978263915	0.812463284
643	0.18	0.2	18.67000014	0.372399995	0	REVERSED General transcription factor 3C polypeptide 1 OS=Rattus norvegicus GN=Gtf3c1 PE=1 SV=1	RAT	0	0.952306211	0.607145488	1.085523963	0.417300493
74	14.74	14.74	75.8400023	69.80000138	69.80000138	Calmodulin OS=Rattus norvegicus GN=Calm1 PE=1 SV=2	RAT	17	0.951984882	0.577468514	1.008337975	0.896755397
149	7.21	7.21	79.42000031	16.86999947	7.682000101	Neurochondrin OS=Rattus norvegicus GN=Ncdn PE=1 SV=2	RAT	4	0.951798916	0.51763308	0.972633004	0.687969089
853	0.08	0.26	25.42999983	0.344599993	0	Fanconi anemia group D2 protein homolog OS=Rattus norvegicus GN=Fancd2 PE=2 SV=2	RAT	0	0.951578021	0.537038386	0.986062407	0.856291115
837	0.09	0.09	27.9399991	1.209999993	0	Peripheral plasma membrane protein CASK OS=Rattus norvegicus GN=Cask PE=1 SV=1	RAT	0	0.951470017	0.913052797	0.951993823	0.746820629
263	2.71	2.74	60.07000208	3.957000002	3.957000002	T-complex protein 1 subunit alpha OS=Rattus norvegicus GN=Tcp1 PE=1 SV=1	RAT	2	0.950993478	0.736795306	1.000283003	0.998180807
905	0.07	0.14	25.33999979	0	0	Serine/threonine-protein kinase TAO2 OS=Rattus norvegicus GN=Taok2 PE=1 SV=1	RAT	0	0.950586677	0.752659976	0.856544793	0.36522159
1108	0.05	0.06	24.95999932	0	0	Exocyst complex component 7 OS=Rattus norvegicus GN=Exoc7 PE=2 SV=1	RAT	0	0.949859679		1.056349993	
801	0.1	0.1	41.0100013	0	0	Perilipin-1 OS=Rattus norvegicus GN=Plin1 PE=1 SV=1	RAT	0	0.949791074	0.517440379	0.92581898	0.352838904

218	4.08	4.17	46.63000107	2.549000084	2.384999953	1-phosphatidylinositol-4,5-bisphosphate phosphodiesterase beta-1 OS=Rattus norvegicus GN=Plcb1 PE=1 SV=1	RAT	2	0.949788213	0.523105919	0.996364772	0.966886878
152	7.1	7.1	89.20000196	22.06999958	22.06999958	ATP synthase subunit O, mitochondrial OS=Rattus norvegicus GN=Atp5o PE=1 SV=1	RAT	6	0.949522376	0.432243198	0.961010218	0.509157896
417	0.83	0.83	35.06000042	5.029999837	1.219999976	Succinate dehydrogenase [ubiquinone] flavoprotein subunit, mitochondrial OS=Rattus norvegicus GN=Sdha PE=1 SV=1	RAT	1	0.948813677	0.517954886	1.027269959	0.732863188
1030	0.06	0.06	32.85999894	1.059999969	0	Beta,beta-carotene 15,15'-monooxygenase OS=Rattus norvegicus GN=Bcmo1 PE=2 SV=1	RAT	0	0.948722124	0.816053212	1.025766015	0.854941785
1140	0.05	0.05	38.01000118	4.977000132	0	REVERSED Ras-related protein Rab-28 OS=Rattus norvegicus GN=Rab28 PE=2 SV=1	RAT	0	0.94860822		1.042848945	
974	0.06	0.14	27.27000117	0.712999981	0	Meckel syndrome type 1 protein homolog OS=Rattus norvegicus GN=Mks1 PE=2 SV=2	RAT	0	0.948536515	0.511112273	1.026440978	0.739080012
789	0.1	0.13	23.92999977	2.778000012	0	Zinc finger protein 672 OS=Rattus norvegicus GN=Znf672 PE=2 SV=1	RAT	0	0.948457599		0.877836883	
1002	0.06	0.08	23.42000008	1.688000001	0	Abhydrolase domain-containing protein 16B OS=Rattus norvegicus GN=Abhd16b PE=2 SV=1	RAT	0	0.948354006		1.004603982	
52	19.6	19.6	99.26999807	92.69999862	92.69999862	Beta-synuclein OS=Rattus norvegicus GN=Sncb PE=1 SV=1	RAT	16	0.947762012	0.467054814	0.910568595	0.228691801
1127	0.05	0.05	39.14999962	0	0	REVERSED Prolyl 4-hydroxylase subunit alpha-3 OS=Rattus norvegicus GN=P4ha3 PE=2 SV=1	RAT	0	0.947714925	0.683155119	1.002074003	0.986659586
35	28.06	28.14	90.71999788	69.16000247	56.88999891	L-lactate dehydrogenase B chain OS=Rattus norvegicus GN=Ldhb PE=1 SV=2	RAT	19	0.947662175	0.197484404	1.024973989	0.545050323
1090	0.05	0.07	18.59000027	0	0	REVERSED Tyrosine-protein kinase Fer OS=Rattus norvegicus GN=Fer PE=2 SV=2	RAT	0	0.946998477	0.680160999	0.969151616	0.82751143
14	42.04	42.04	91.33999944	63.48999739	57.66999722	Synapsin-1 OS=Rattus norvegicus GN=Syn1 PE=1 SV=3	RAT	30	0.946915388	0.158659399	0.983485222	0.657849312
141	8.05	8.05	74.29000139	42.14000106	42.14000106	Profilin-2 OS=Rattus norvegicus GN=Pfn2 PE=1 SV=3	RAT	5	0.946804285	0.627936184	0.999399185	0.994919777
453	0.58	0.65	33.57999921	0.646900013	0	Collagen alpha-1(XXVII) chain OS=Rattus norvegicus GN=Col27a1 PE=2 SV=1	RAT	0	0.946766078	0.689099789	1.035447001	0.716142714
557	0.27	0.28	30.70000112	2.129999921	0	REVERSED Bromodomain-containing protein 2 OS=Rattus norvegicus GN=Brd2 PE=2 SV=1	RAT	0	0.945896387	0.683972418	0.935229719	0.631561399
1010	0.06	0.07	41.40999913	0	0	Kynurenine/alpha-aminoadipate aminotransferase, mitochondrial OS=Rattus norvegicus GN=Aadat PE=1 SV=1	RAT	0	0.945428371	0.563837886	0.93253988	0.483496606
507	0.38	0.38	35.83999872	2.376000024	0	Protein disulfide-isomerase A3 OS=Rattus norvegicus GN=Pdia3 PE=1 SV=2	RAT	0	0.945400298	0.672282875	0.943605125	0.663386703
1012	0.06	0.07	28.97999883	1.769999973	0	Caspase-2 OS=Rattus norvegicus GN=Casp2 PE=2 SV=3	RAT	0	0.945326388		0.957963526	
118	9.85	9.85	66.75999761	43.47000122	36.0799998	Endophilin-A1 OS=Rattus norvegicus GN=Sh3gl2 PE=1 SV=2	RAT	8	0.945278287	0.623819172	0.958105624	0.539041877
529	0.34	0.34	61.36000156	43.18000078	0	Thymosin beta-4 OS=Rattus norvegicus GN=Tmsb4x PE=1 SV=2	RAT	0	0.94524771	0.677254319	0.94324398	0.775201023
738	0.12	0.13	29.57000136	0.982800033	0	Deubiquitinating protein VCIP135 OS=Rattus norvegicus GN=Vcpip1 PE=1 SV=2	RAT	0	0.945100605	0.558364272	0.931390703	0.473162591
796	0.1	0.11	7.156000286	1.506999973	0	REVERSED Muscarinic acetylcholine receptor M5 OS=Rattus norvegicus GN=Chrm5 PE=1 SV=1	RAT	0	0.945060492	0.675521195	0.892388821	0.46157369
347	1.91	1.91	47.51999974	3.66399996	1.508999988	cGMP-dependent 3',5'-cyclic phosphodiesterase OS=Rattus norvegicus GN=Pde2a PE=1 SV=2	RAT	1	0.943089128	0.822464108	0.977466524	0.865219474
72	15.15	16.84	91.62999988	35.12000144	29.53000069	Glial fibrillary acidic protein OS=Rattus norvegicus GN=Gfap PE=1 SV=2	RAT	9	0.942793489	0.194988593	0.946052313	0.4471232
254	2.89	2.95	89.70999718	35.28999984	28.67999971	Histone H3.3 OS=Rattus norvegicus GN=H3f3b PE=1 SV=2	RAT	4	0.942260981	0.612522483	1.122946978	0.224481195
693	0.14	0.19	42.89000034	1.98800005	0	REVERSED Ras GTPase-activating protein SynGAP OS=Rattus norvegicus GN=Syngap1 PE=1 SV=2	RAT	0	0.942027628	0.653963625	1.052613974	0.695446372
927	0.07	0.09	28.92999947	0	0	REVERSED Leucine-rich repeat and fibronectin type-III domain-containing protein 2 OS=Rattus norvegicus GN=Lrfn2 PE=1 SV=1	RAT	0	0.941821277	0.65503782	1.150351048	0.57595098
1103	0.05	0.06	27.20000148	0	0	Ankycorbin OS=Rattus norvegicus GN=Rai14 PE=2 SV=2	RAT	0	0.941555798		1.04103601	
892	0.08	0.08	22.73000032	12.12000027	0	Stress-associated endoplasmic reticulum protein 1 OS=Rattus norvegicus GN=Serp1 PE=1 SV=2	RAT	0	0.940940976		0.980552077	
681	0.15	0.17	25.29000044	1.900999993	0	REVERSED Keratin, type I cytoskeletal 10 OS=Rattus norvegicus GN=Krt10 PE=2 SV=1	RAT	0	0.940727413		0.81401819	
639	0.18	0.46	32.10000098	0.165700004	0	REVERSED Microtubule-actin cross-linking factor 1 OS=Rattus norvegicus GN=Macf1 PE=1 SV=1	RAT	0	0.940662682	0.682108879	0.9755283	0.720327199
817	0.09	0.12	55.1699996	0.399200013	0	REVERSED Fatty acid synthase OS=Rattus norvegicus GN=Fasn PE=1 SV=3	RAT	0	0.94039458	0.646064878	0.934304476	0.745655775
229	3.71	7.72	62.15000153	31.63999915	10.72999984	Guanine nucleotide-binding protein G(i) subunit alpha-1 OS=Rattus norvegicus GN=Gnai1 PE=1 SV=3	RAT	3	0.940356672	0.474674106	1.022696018	0.784016371
4	52.47	52.47	94.87000108	91.74000025	88.38999867	Tubulin alpha-4A chain OS=Rattus norvegicus GN=Tuba4a PE=2 SV=1	RAT	128	0.93930459	0.473055691	0.971071482	0.725173414
293	2.08	2.1	70.4400003	3.201999888	3.201999888	26S protease regulatory subunit 8 OS=Rattus norvegicus GN=Psmc5 PE=1 SV=1	RAT	1	0.939100981	0.527219176	1.004421949	0.962451518
372	1.58	1.6	32.22000003	2.380999923	0.793700013	Ankyrin repeat and sterile alpha motif domain-containing protein 1B OS=Rattus norvegicus GN=Anks1b PE=1 SV=1	RAT	1	0.939061999	0.542362988	0.894128978	0.325631499
350	1.86	1.86	28.88000011	13.16000074	1.768000051	Tyrosine-protein phosphatase non-receptor type substrate 1 OS=Rattus norvegicus GN=Sirpa PE=1 SV=1	RAT	1	0.938889325	0.546762586	0.992375314	0.938460588
963	0.06	0.23	29.69999909	1.83600001	0	Rap guanine nucleotide exchange factor 3 OS=Rattus norvegicus GN=Rapgef3 PE=2 SV=2	RAT	0	0.93862468	0.641460717	0.978558779	0.869476378
866	0.08	0.1	25.76000094	0	0	REVERSED Rab3 GTPase-activating protein non-catalytic subunit OS=Rattus norvegicus GN=Rab3gap2 PE=1 SV=2	RAT	0	0.93826592	0.645834029	0.950250804	0.705062926
223	4	5.32	79.61000204	16.94999933	11.59000024	Vimentin OS=Rattus norvegicus GN=Vim PE=1 SV=2	RAT	5	0.937780976	0.761950791	1.023640037	0.880110204
1005	0.06	0.07	67.77999997	0	0	REVERSED Myosin-Va OS=Rattus norvegicus GN=Myo5a PE=2 SV=1	RAT	0	0.937597215	0.634246588	0.887666881	0.442590714
694	0.14	0.18	34.92000103	0.712599978	0	Myosin-Ia (Fragment) OS=Rattus norvegicus GN=Myo1a PE=2 SV=1	RAT	0	0.937578022	0.71693331	1.163007975	0.376264989
341	2	2	27.27000117	7.791999727	7.791999727	Eukaryotic translation initiation factor 5A-1 OS=Rattus norvegicus GN=Eif5a PE=1 SV=3	RAT	2	0.937212229		0.882151186	
143	7.76	13.82	94.33000088	55.87000251	51.41999722	14-3-3 protein gamma OS=Rattus norvegicus GN=Ywhag PE=1 SV=2	RAT	14	0.937021673	0.514451802	1.016381025	0.862838984
893	0.07	0.35	35.29999852	0.641799998	0	REVERSED Ribosome-releasing factor 2, mitochondrial OS=Rattus norvegicus GN=Gfm2 PE=2 SV=2	RAT	0	0.936664879	0.710635006	0.969670713	0.752969921
744	0.12	0.12	29.31999862	1.704999991	0	REVERSED NACHT, LRR and PYD domains-containing protein 6 OS=Rattus norvegicus GN=Nlrp6 PE=1 SV=3	RAT	0	0.936565995	0.638888896	1.020037055	0.875448227
715	0.13	0.16	23.80000055	2.266000025	0	Heterogeneous nuclear ribonucleoprotein D0 OS=Rattus norvegicus GN=Hnrnpd PE=1 SV=1	RAT	0	0.935999572	0.625639379	0.863342583	0.435049504
85	12.83	12.83	69.41000223	41.78000093	38.82000148	Sodium/potassium-transporting ATPase subunit beta-1 OS=Rattus norvegicus GN=Atp1b1 PE=1 SV=1	RAT	12	0.935162485	0.160960495	0.911783218	0.06255056
50	20.17	20.17	73.29999804	18.67000014	16.93000048	Excitatory amino acid transporter 2 OS=Rattus norvegicus GN=Slc1a2 PE=1 SV=2	RAT	11	0.934546411	0.382842988	0.935503423	0.162884995
368	1.62	1.68	65.17000198	37.07999885	24.71999973	Dynein light chain 2, cytoplasmic OS=Rattus norvegicus GN=Dynll2 PE=1 SV=1	RAT	1	0.934359372	0.500611484	0.98383069	0.882851422
647	0.17	0.31	32.73999989	0.153400004	0	Striated muscle-specific serine/threonine-protein kinase OS=Rattus norvegicus GN=Speg PE=2 SV=2	RAT	0	0.933992207	0.610654712	0.978808701	0.853340983
458	0.57	0.57	14.4600004	11.24000028	0	Vesicle-associated membrane protein-associated protein A OS=Rattus norvegicus GN=Vapa PE=1 SV=3	RAT	0	0.933765829	0.614360273	0.894024611	0.460453093
294	2.08	2.08	46.84000015	2.105000056	2.105000056	DNA damage-binding protein 1 OS=Rattus norvegicus GN=Ddb1 PE=2 SV=1	RAT	1	0.933737278		1.011211991	
220	4.04	19.42	71.46000266	13.19999993	12.16000021	Plasma membrane calcium-transporting ATPase 3 OS=Rattus norvegicus GN=Atp2b3 PE=2 SV=2	RAT	10	0.933600903	0.624735177	1.097972989	0.713230371
405	0.96	0.96	33.50999951	5.155000091	5.155000091	40S ribosomal protein S9 OS=Rattus norvegicus GN=Rps9 PE=1 SV=4	RAT	1	0.933599472	0.493599296	1.063537002	0.558386981
104	11	11	76.17999911	30.03999889	20.81999928	Citrate synthase, mitochondrial OS=Rattus norvegicus GN=Cs PE=1 SV=1	RAT	14	0.933247924	0.201655701	1.019917011	0.701265812
665	0.16	0.19	33.01999867	8.019000292	0	Fibroblast growth factor 20 OS=Rattus norvegicus GN=Fgf20 PE=2 SV=1	RAT	0	0.932425022		1.01888597	
186	5.53	8.34	47.20000029	15.03999978	10.86999997	Alpha-actinin-4 OS=Rattus norvegicus GN=Actn4 PE=1 SV=2	RAT	6	0.932355523	0.635265529	1.01207304	0.923166573
1059	0.05	0.1	30.34999967	0	0	Cystic fibrosis transmembrane conductance regulator OS=Rattus norvegicus GN=Cftr PE=1 SV=3	RAT	0	0.932198703	0.479243398	0.977662683	0.823749781
146	7.42	7.42	97.39999771	74.02999997	61.69000268	Superoxide dismutase [Cu-Zn] OS=Rattus norvegicus GN=Sod1 PE=1 SV=2	RAT	5	0.932143688	0.299705595	0.914272785	0.199639902
522	0.35	0.35	32.76999891	2.941000089	0	Histone deacetylase 7 (Fragment) OS=Rattus norvegicus GN=Hdac7 PE=2 SV=1	RAT	0	0.931939483		0.90499562	
880	0.08	0.08	39.86999989	0	0	REVERSED Melanoma-associated antigen D1 OS=Rattus norvegicus GN=Maged1 PE=1 SV=3	RAT	0	0.931784689	0.68645978	0.999049187	0.995406508
343	2	2	56.13999963	14.04000074	14.04000074	60S acidic ribosomal protein P1 OS=Rattus norvegicus GN=Rplp1 PE=3 SV=1	RAT	1	0.931723714		1.087968946	
379	1.39	1.41	52.48000026	4.255000129	4.255000129	Succinate dehydrogenase [ubiquinone] iron-sulfur subunit, mitochondrial OS=Rattus norvegicus GN=Sdhb PE=2 SV=2	RAT	1	0.931415975	0.63133949	0.953775287	0.5875386
572	0.24	0.28	35.96000075	0	0	REVERSED Myosin-IXb OS=Rattus norvegicus GN=Myo9b PE=1 SV=1	RAT	0	0.930744886	0.169526607	0.966014683	0.488648802
834	0.09	0.1	67.69999862	10.11999995	0	REVERSED Ras-related protein Rab-26 OS=Rattus norvegicus GN=Rab26 PE=2 SV=2	RAT	0	0.930680811		0.93397671	
753	0.12	0.12	47.72000015	2.806999907	0	REVERSED N(G),N(G)-dimethylarginine dimethylaminohydrolase 2 OS=Rattus norvegicus GN=Ddah2 PE=1 SV=1	RAT	0	0.930625916	0.656688571	1.118021011	0.461962193
712	0.13	0.17	32.31999874	2.208999917	0	Serine/threonine-protein kinase WNK4 OS=Rattus norvegicus GN=Wnk4 PE=2 SV=3	RAT	0	0.930526674	0.466555297	0.993087113	0.939352274
483	0.46	0.46	17.64999926	3.113999963	0	Growth hormone-regulated TBC protein 1 OS=Rattus norvegicus GN=Grtp1 PE=2 SV=1	RAT	0	0.929892778		0.96360302	
469	0.53	0.53	26.23000145	4.26199995	0	REVERSED Serine/threonine-protein phosphatase 6 catalytic subunit OS=Rattus norvegicus GN=Ppp6c PE=2 SV=2	RAT	0	0.929700673		0.821951985	
1111	0.05	0.06	15.96000046	0	0	Proprotein convertase subtilisin/kexin type 7 OS=Rattus norvegicus GN=Pcsk7 PE=1 SV=1	RAT	0	0.929229319	0.597801208	0.928976178	0.597568989
162	6.64	6.88	60.17000079	4.909000173	2.78299991	Myosin-10 OS=Rattus norvegicus GN=Myh10 PE=1 SV=1	RAT	5	0.928815603	0.271009713	0.982658029	0.780537188
560	0.26	0.38	28.20000052	0	0	Microtubule-actin cross-linking factor 1 OS=Rattus norvegicus GN=Macf1 PE=1 SV=1	RAT	0	0.928791583	0.168626294	1.114454031	0.290907413

230	3.67	3.7	41.11000001	2.096999995	1.398000028	Synaptojanin-1 OS=Rattus norvegicus GN=Synj1 PE=1 SV=3	RAT	2	0.927971005	0.311742187	0.956887007	0.534278274
79	13.75	13.75	71.05000019	37.52999902	35.38999856	Glutamine synthetase OS=Rattus norvegicus GN=Glul PE=1 SV=3	RAT	13	0.927904785	0.162367806	0.981549203	0.735053122
360	1.74	1.79	46.47000134	1.654000022	1.654000022	Prelamin-A/C OS=Rattus norvegicus GN=Lmna PE=1 SV=1	RAT	1	0.927588105	0.464314014	0.970516384	0.755163908
1079	0.05	0.07	30.82000017	0	0	Serine/threonine-protein kinase MRCK beta OS=Rattus norvegicus GN=Cdc42bpb PE=1 SV=1	RAT	0	0.927247584	0.804582298	1.046751022	0.728215814
396	1.07	1.16	33.32000077	0.723499991	0	Myosin-IXa OS=Rattus norvegicus GN=Myo9a PE=1 SV=1	RAT	0	0.927217782	0.452461809	1.015707016	0.919530928
388	1.24	1.24	43.14999878	5.393999815	5.393999815	Dihydropteridine reductase OS=Rattus norvegicus GN=Qdpr PE=1 SV=1	RAT	1	0.92705971		0.922356427	
278	2.31	2.31	71.03000283	14.94999975	7.477000356	ATPase inhibitor, mitochondrial OS=Rattus norvegicus GN=Atpif1 PE=2 SV=2	RAT	1	0.926818013	0.295214415	0.885496616	0.186258405
898	0.07	0.18	11.16999984	6.145000085	0	REVERSED Ubiquinone biosynthesis protein COQ7 homolog (Fragment) OS=Rattus norvegicus GN=Coq7 PE=2 SV=3	RAT	0	0.926655591		0.967031479	
319	2.01	2.01	37.81999946	8.807999641	8.807999641	Cysteine and glycine-rich protein 1 OS=Rattus norvegicus GN=Csrp1 PE=2 SV=2	RAT	1	0.926095188		0.89126271	
743	0.12	0.12	73.29999804	3.567000106	0	REVERSED Dynamin-3 OS=Rattus norvegicus GN=Dnm3 PE=1 SV=2	RAT	0	0.925622284	0.448844492	0.996383727	0.97067672
1084	0.05	0.07	25.44000149	0	0	REVERSED Phosphorylase b kinase regulatory subunit alpha, skeletal muscle isoform OS=Rattus norvegicus GN=Phka1 PE=2 SV=2	RAT	0	0.924749196	0.573948801	0.952674925	0.709669411
90	12.59	12.59	83.03999901	54.46000099	49.54999983	Peroxiredoxin-6 OS=Rattus norvegicus GN=Prdx6 PE=1 SV=3	RAT	7	0.924568474	0.266233593	0.936815619	0.346458107
618	0.2	0.24	28.99999917	5.333000049	0	Transcription factor E2F5 (Fragment) OS=Rattus norvegicus GN=E2f5 PE=2 SV=1	RAT	0	0.924542189		0.849002421	
215	4.1	4.1	43.70999932	19.9300006	19.9300006	F-actin-capping protein subunit alpha-2 OS=Rattus norvegicus GN=Capza2 PE=1 SV=1	RAT	3	0.924511611	0.591694415	0.941036701	0.666104198
822	0.09	0.12	34.58999991	6.917999685	0	Solute carrier family 25 member 42 OS=Rattus norvegicus GN=Slc25a42 PE=2 SV=1	RAT	0	0.923813283		1.143586993	
676	0.15	25.77	90.71999788	61.26999855	56.23000264	Actin, alpha cardiac muscle 1 OS=Rattus norvegicus GN=Actc1 PE=1 SV=1	RAT	32	0.923791528	0.578601182	0.986752272	0.923192918
978	0.06	0.12	24.78999943	0	0	Calcineurin-binding protein cabin-1 OS=Rattus norvegicus GN=Cabin1 PE=1 SV=1	RAT	0	0.923686326	0.417990297	0.994342387	0.943822384
606	0.21	0.22	35.01999974	1.240999997	0	REVERSED Nck-associated protein 1 OS=Rattus norvegicus GN=Nckap1 PE=2 SV=1	RAT	0	0.9230358		1.054173946	
654	0.17	0.17	20.03999949	0.715699978	0	Macrophage colony-stimulating factor 1 receptor OS=Rattus norvegicus GN=Csf1r PE=2 SV=1	RAT	0	0.922593892	0.427746713	0.916168511	0.3957026
619	0.2	0.23	51.55000091	0.918999966	0	SRC kinase signaling inhibitor 1 OS=Rattus norvegicus GN=Srcin1 PE=1 SV=1	RAT	0	0.922376275	0.584292173	0.89231509	0.69396472
658	0.17	0.17	27.59999931	1.982999966	0	REVERSED Dolichyl-diphosphooligosaccharide--protein glycosyltransferase subunit 1 OS=Rattus norvegicus GN=Rpn1 PE=2 SV=1	RAT	0	0.921400189	0.761493027	0.901309371	0.499425411
808	0.09	1.34	28.99999917	4.408000037	2.088000067	Septin-11 OS=Rattus norvegicus GN=Sept11 PE=1 SV=1	RAT	1	0.919836521	0.579322517	0.955450475	0.744000077
482	0.47	0.47	24.1899997	1.882000081	0	REVERSED Actin-related protein 2/3 complex subunit 1B OS=Rattus norvegicus GN=Arpc1b PE=2 SV=3	RAT	0	0.919642389		0.912011683	
638	0.19	0.19	44.78999972	6.25	0	REVERSED Serine/threonine-protein phosphatase PGAM5, mitochondrial OS=Rattus norvegicus GN=Pgam5 PE=2 SV=1	RAT	0	0.919360101	0.551809371	0.909946978	0.515822828
709	0.13	0.24	29.96999919	0.333900005	0	REVERSED Potassium/sodium hyperpolarization-activated cyclic nucleotide-gated channel 4 OS=Rattus norvegicus GN=Hcn4 PE=2 SV=1	RAT	0	0.919215202	0.32586199	1.050632954	0.542857409
1095	0.05	0.07	19.72000003	1.689999923	0	REVERSED Solute carrier organic anion transporter family member 3A1 OS=Rattus norvegicus GN=Slco3a1 PE=2 SV=1	RAT	0	0.918944776	0.593295097	0.989856124	0.964848876
823	0.09	0.11	19.74000037	0	0	Low-density lipoprotein receptor-related protein 2 OS=Rattus norvegicus GN=Lrp2 PE=1 SV=1	RAT	0	0.918181717	0.529231071	0.943383574	0.477223098
261	2.77	2.88	38.82000148	3.985999897	3.293000162	Moesin OS=Rattus norvegicus GN=Msn PE=1 SV=3	RAT	2	0.917958617	0.610932529	0.896294415	0.492086589
1125	0.05	0.05	54.83999848	0	0	Friend of PRMT1 protein OS=Rattus norvegicus GN=Fop PE=2 SV=1	RAT	0	0.916719615	0.54076612	0.935094416	0.620266616
338	2	2	65.52000046	17.23999977	17.23999977	NADH dehydrogenase [ubiquinone] 1 alpha subcomplex subunit 5 OS=Rattus norvegicus GN=Ndufa5 PE=1 SV=3	RAT	1	0.915944695		0.955346823	
838	0.09	0.09	29.98999953	0.590300001	0	A-kinase anchor protein 4 OS=Rattus norvegicus GN=Akap4 PE=1 SV=1	RAT	0	0.915849984	0.537223816	0.948629677	0.688144386
274	2.42	3.47	60.72999835	36.12999916	30.37	Cell division control protein 42 homolog OS=Rattus norvegicus GN=Cdc42 PE=1 SV=2	RAT	3	0.915801108	0.576552093	0.857865512	0.403284401
242	0.86	1.13	27.36999989	7.105000317	2.895000018	Mitogen-activated protein kinase 3 OS=Rattus norvegicus GN=Mapk3 PE=1 SV=5	RAT	1	0.91571188	0.547819316	0.801861286	0.276649594
412	0.86	1.13	27.36999989	7.105000317	2.895000018	Mitogen-activated protein kinase 3 OS=Rattus norvegicus GN=Mapk3 PE=1 SV=5	RAT	1	0.91571188	0.547819316	0.801861286	0.276649594
421	0.76	0.76	20.9800005	5.959000066	5.959000066	5-hydroxytryptamine receptor 1B OS=Rattus norvegicus GN=Htr1b PE=2 SV=1	RAT	1	0.915415525		0.889685571	
183	5.64	5.68	73.46000075	14.22999948	12.30999976	Succinyl-CoA:3-ketoacid-coenzyme A transferase 1, mitochondrial OS=Rattus norvegicus GN=Oxct1 PE=1 SV=1	RAT	3	0.915195584	0.355623007	0.980429828	0.824304521
722	0.13	0.13	35.83000004	1.737999916	0	Meprin A subunit alpha OS=Rattus norvegicus GN=Mep1a PE=1 SV=2	RAT	0	0.915148914		0.925607502	
555	0.28	0.28	14.24999982	3.166000172	0	Sialidase-2 OS=Rattus norvegicus GN=Neu2 PE=2 SV=1	RAT	0	0.913423419		1.106587052	
573	0.24	0.27	26.3500005	0.748999992	0	Zinc finger and BTB domain-containing protein 38 OS=Rattus norvegicus GN=Zbtb38 PE=2 SV=1	RAT	0	0.913264394	0.382069588	0.930585682	0.470394611
630	0.19	0.6	38.24999928	0.686899992	0	REVERSED PDZ domain-containing protein 2 OS=Rattus norvegicus GN=Pdzd2 PE=1 SV=1	RAT	0	0.912529886	0.223798394	1.00030601	0.996393323
961	0.06	0.3	24.2899999	0.547000021	0	Thyroid peroxidase OS=Rattus norvegicus GN=Tpo PE=2 SV=1	RAT	0	0.912343323	0.52617228	0.975928903	0.847985506
816	0.09	0.13	24.49000031	3.265000135	0	REVERSED Collagen triple helix repeat-containing protein 1 OS=Rattus norvegicus GN=Cthrc1 PE=1 SV=1	RAT	0	0.911711693	0.526108623	0.96503371	0.78418088
365	1.69	1.73	30.00999987	1.204000041	1.204000041	Reticulon-4 OS=Rattus norvegicus GN=Rtn4 PE=1 SV=1	RAT	1	0.911164224	0.583404422	0.895916522	0.3261365
652	0.17	0.17	64.3599987	10.05999967	10.05999967	Adenylyl cyclase-associated protein 2 OS=Rattus norvegicus GN=Cap2 PE=2 SV=1	RAT	2	0.911072016		1.004312038	
583	0.23	0.28	28.60000134	0.717899995	0.448699994	REVERSED Breast cancer type 2 susceptibility protein homolog OS=Rattus norvegicus GN=Brca2 PE=1 SV=1	RAT	1	0.910606027		0.989513576	
889	0.08	0.08	10.46999991	1.394999959	0	N-acylglucosamine 2-epimerase OS=Rattus norvegicus GN=Renbp PE=2 SV=2	RAT	0	0.91042459		0.895005882	
983	0.06	0.1	34.20000076	0	0	Kinesin-like protein KIF1B OS=Rattus norvegicus GN=Kif1b PE=1 SV=2	RAT	0	0.910329819	0.372391105	0.954130173	0.625258386
635	0.19	0.22	28.7499994	0.162600004	0	Cytoplasmic dynein 2 heavy chain 1 OS=Rattus norvegicus GN=Dync2h1 PE=1 SV=1	RAT	0	0.909841716	0.519785583	0.916674078	0.544112921
675	0.16	0.16	36.66999936	8.666999638	0	REVERSED Ribonuclease pancreatic delta-type OS=Rattus norvegicus GN=Rnase1d PE=3 SV=1	RAT	0	0.909574628		0.945015371	
622	0.2	0.22	31.47000074	0.7675	0	Metabotropic glutamate receptor 4 OS=Rattus norvegicus GN=Grm4 PE=1 SV=1	RAT	0	0.909332991	0.514082372	1.069715023	0.621647894
311	2.02	2.06	47.90000021	1.532000024	1.532000024	Neurofascin OS=Rattus norvegicus GN=Nfasc PE=1 SV=2	RAT	1	0.908887923	0.512542725	1.005586028	0.979068577
489	0.43	0.43	22.89000005	1.999999955	0	REVERSED Runt-related transcription factor 1 OS=Rattus norvegicus GN=Runx1 PE=2 SV=1	RAT	0	0.908689201	0.510448575	0.92378068	0.570260108
984	0.06	0.1	35.83999872	0	0	REVERSED Disabled homolog 2-interacting protein OS=Rattus norvegicus GN=Dab2ip PE=1 SV=1	RAT	0	0.90830791	0.517540276	0.876173019	0.41650781
585	0.23	0.25	19.07999963	1.155999955	0	DNA-binding protein RFX2 OS=Rattus norvegicus GN=Rfx2 PE=2 SV=1	RAT	0	0.907350481		0.898201883	
414	0.84	0.87	32.82999992	1.51800001	1.51800001	REVERSED Cyclin-L1 OS=Rattus norvegicus GN=Ccnl1 PE=1 SV=1	RAT	1	0.907140911	0.508893728	0.928964674	0.59642309
1088	0.05	0.07	42.68000126	0	0	REVERSED NGFI-A-binding protein 1 OS=Rattus norvegicus GN=Nab1 PE=2 SV=2	RAT	0	0.90695858	0.510458708	0.96099472	0.804403722
737	0.12	0.14	38.94000053	4.867000133	0	Neuron-specific vesicular protein calcyon OS=Rattus norvegicus GN=Caly PE=1 SV=1	RAT	0	0.906075716		0.935668826	
785	0.1	0.13	27.73000002	0.477200001	0	Nuclear pore membrane glycoprotein 210 OS=Rattus norvegicus GN=Pom210 PE=1 SV=1	RAT	0	0.905291021	0.49941051	0.957091689	0.735415399
641	0.18	0.21	38.35000098	0.958800036	0	REVERSED Glutamate receptor-interacting protein 2 OS=Rattus norvegicus GN=Grip2 PE=1 SV=1	RAT	0	0.904849708		0.994793713	
826	0.09	0.11	38.19999993	1.283999998	0	Leucine-rich repeat, immunoglobulin-like domain and transmembrane domain-containing protein 1 OS=Rattus norvegicus GN=Lrit1 PE=1 SV=2	RAT	0	0.903897583		0.915482521	
934	0.07	0.08	26.53000057	0	0	REVERSED Breast cancer type 1 susceptibility protein homolog OS=Rattus norvegicus GN=Brca1 PE=1 SV=1	RAT	0	0.90374732	0.352374405	1.011155009	0.90586549
165	6.59	6.71	50.33000112	7.479999959	6.667000055	Cullin-associated NEDD8-dissociated protein 1 OS=Rattus norvegicus GN=Cand1 PE=1 SV=1	RAT	5	0.903135598	0.36869669	1.033565044	0.67440629
642	0.18	0.21	19.65000033	1.889000088	0	Striatin-3 OS=Rattus norvegicus GN=Strn3 PE=2 SV=2	RAT	0	0.901988089	0.490673214	0.966386914	0.790705919
1065	0.05	0.09	24.77999926	0	0	Crooked neck-like protein 1 OS=Rattus norvegicus GN=Crnkl1 PE=2 SV=1	RAT	0	0.901583374	0.335681111	1.059393048	0.555731773
633	0.19	0.23	44.38000023	2.465000004	0	Leucine-rich repeat transmembrane protein FLRT3 OS=Rattus norvegicus GN=Flrt3 PE=2 SV=1	RAT	0	0.901211679	0.483752489	0.977207601	0.854112923
97	11.49	11.49	61.87999845	16.15999937	14.36000019	Disks large homolog 4 OS=Rattus norvegicus GN=Dlg4 PE=1 SV=1	RAT	6	0.901115716	0.223221496	0.939777315	0.306375295
755	0.11	0.27	39.0899986	0.7216	0	REVERSED Complement C3 OS=Rattus norvegicus GN=C3 PE=1 SV=3	RAT	0	0.901090384	0.489345789	0.906215489	0.507144213
322	2	2.1	37.54999936	3.376000002	2.531999908	Adenylyl cyclase-associated protein 1 OS=Rattus norvegicus GN=Cap1 PE=1 SV=3	RAT	1	0.900786817		0.972665012	
517	0.36	0.36	19.67999935	5.397000164	0	Heme oxygenase 2 OS=Rattus norvegicus GN=Hmox2 PE=1 SV=1	RAT	0	0.90076828		1.044955015	
750	0.12	0.12	10.66000015	0.861100014	0	REVERSED Urea transporter 2 OS=Rattus norvegicus GN=Slc14a2 PE=1 SV=2	RAT	0	0.899749994	0.669304311	0.955963373	0.738053679
867	0.08	0.1	28.36999893	0.446800003	0	REVERSED Vascular endothelial growth factor receptor 2 OS=Rattus norvegicus GN=Kdr PE=1 SV=1	RAT	0	0.898397624		1.039847016	
1027	0.06	0.06	30.68000078	0	0	Probable G-protein coupled receptor 37 OS=Rattus norvegicus GN=Gpr37 PE=2 SV=1	RAT	0	0.898396611	0.479914814	0.863593102	0.407716393
973	0.06	0.14	67.69000292	7.69200027	0	REVERSED ProSAAS OS=Rattus norvegicus GN=Pcsk1n PE=1 SV=1	RAT	0	0.898301482		0.908034384	
828	0.09	0.11	8.615999669	0.753299985	0	REVERSED Aggrecan core protein OS=Rattus norvegicus GN=Acan PE=1 SV=2	RAT	0	0.897975326	0.474312693	0.851167321	0.67918849

413	0.85	0.85	47.31999934	3.155000135	3.155000135	60S acidic ribosomal protein P0 OS=Rattus norvegicus GN=Rplp0 PE=1 SV=2	RAT	1	0.897789419		0.988365889	
399	1.04	1.06	36.26999855	2.590999939	2.590999939	Centrosomal protein of 44 kDa OS=Rattus norvegicus GN=Cep44 PE=2 SV=1	RAT	1	0.897020102		0.994035602	
690	0.14	2.22	72.7699995	12.21000031	5.164000019	Ras-related protein Rab-4B OS=Rattus norvegicus GN=Rab4b PE=2 SV=1	RAT	1	0.896878481		1.02472496	
660	0.16	0.35	33.03999901	0.078659999	0	REVERSED Protein piccolo OS=Rattus norvegicus GN=Pclo PE=1 SV=1	RAT	0	0.896481514	0.291155189	0.938928425	0.437066704
854	0.08	0.21	54.44999933	0.202400004	0	REVERSED Myosin-10 OS=Rattus norvegicus GN=Myh10 PE=1 SV=1	RAT	0	0.895570219	0.307142705	0.977721274	0.806969702
1132	0.05	0.05	27.61000097	1.971999928	0	Uncharacterized protein C9orf128 homolog OS=Rattus norvegicus PE=2 SV=1	RAT	0	0.895434499		0.874714911	
1028	0.06	0.06	22.72000015	1.69699993	0	Protein FAM5C OS=Rattus norvegicus GN=Fam5c PE=2 SV=1	RAT	0	0.895027876		0.954062819	
844	0.09	0.09	38.06000054	4.515999928	0	NAD-dependent deacetylase sirtuin-5 OS=Rattus norvegicus GN=Sirt5 PE=2 SV=1	RAT	0	0.893790424		1.121780038	
965	0.06	0.21	18.53999943	0.248000002	0	Transcription activator BRG1 OS=Rattus norvegicus GN=Smarca4 PE=1 SV=1	RAT	0	0.893335521	0.777427197	0.96941781	0.825354218
481	0.47	0.47	37.79000044	5.232999846	0	Neurotrimin OS=Rattus norvegicus GN=Ntm PE=1 SV=1	RAT	0	0.892511904	0.77265358	0.961660087	0.90681839
856	0.08	0.15	20.8100006	0	0	Zinc finger FYVE domain-containing protein 26 OS=Rattus norvegicus GN=Zfyve26 PE=3 SV=1	RAT	0	0.891604781	0.234339997	0.999979973	1
548	0.28	0.33	29.10000086	1.379000023	0	REVERSED Antigen peptide transporter 1 OS=Rattus norvegicus GN=Tap1 PE=1 SV=2	RAT	0	0.890711904	0.292333513	0.867730677	0.223078594
704	0.14	0.14	40.45999944	4.275999963	0	REVERSED DCN1-like protein 3 OS=Rattus norvegicus GN=Dcun1d3 PE=2 SV=1	RAT	0	0.889634728		0.973667026	
408	0.95	0.97	24.51000065	1.941999979	1.941999979	REVERSED Neuromedin-U receptor 1 OS=Rattus norvegicus GN=Nmur1 PE=1 SV=1	RAT	1	0.889537573	0.447807997	0.899224699	0.61216712
975	0.06	0.14	33.84000063	3.282999992	0	Macrophage erythroblast attacher OS=Rattus norvegicus GN=Maea PE=2 SV=2	RAT	0	0.889478028		0.818778813	
1137	0.05	0.05	14.01000023	2.137999982	0	Medium-chain specific acyl-CoA dehydrogenase, mitochondrial OS=Rattus norvegicus GN=Acadm PE=1 SV=1	RAT	0	0.889478028		0.818778813	
484	0.45	0.5	42.17000008	15.65999985	15.65999985	Cytochrome c oxidase subunit 7A2, mitochondrial OS=Rattus norvegicus GN=Cox7a2 PE=1 SV=1	RAT	1	0.88920927		0.963547289	
336	2	2	42.17999876	3.835000098	3.835000098	Cathepsin B OS=Rattus norvegicus GN=Ctsb PE=1 SV=2	RAT	1	0.888774872		0.789767921	
621	0.2	0.22	43.09999943	1.279999968	0	REVERSED Metastasis-associated protein MTA1 OS=Rattus norvegicus GN=Mta1 PE=1 SV=1	RAT	0	0.888768017		1.273406982	
537	0.32	0.34	26.06000006	1.057999954	0	REVERSED SH2B adapter protein 1 OS=Rattus norvegicus GN=Sh2b1 PE=1 SV=1	RAT	0	0.887476027		0.995983124	
702	0.14	0.14	37.13000119	1.374999993	0	Protein disulfide-isomerase OS=Rattus norvegicus GN=P4hb PE=1 SV=2	RAT	0	0.887444615	0.440165401	1.011219025	0.928459823
494	0.42	0.42	18.79999936	1.709000021	0	REVERSED Uncharacterized protein KIAA0895-like OS=Rattus norvegicus GN=Kiaa0895l PE=2 SV=1	RAT	0	0.887035728		1.092599034	
352	1.83	6.38	65.32999873	15.43000042	10.48000008	Serine/threonine-protein phosphatase 2B catalytic subunit beta isoform OS=Rattus norvegicus GN=Ppp3cb PE=2 SV=1	RAT	4	0.886578023	0.472408414	1.048145056	0.631139815
1131	0.05	0.05	43.81000102	0	0	REVERSED Cyclin-dependent kinase 11 OS=Rattus norvegicus GN=Cdk11 PE=2 SV=1	RAT	0	0.886142373	0.440065205	0.818205774	0.294924796
580	0.23	0.48	32.89000094	4.278000072	0	Transcription termination factor, mitochondrial OS=Rattus norvegicus GN=Mterf PE=1 SV=1	RAT	0	0.884889424		0.927380204	
1069	0.05	0.08	31.16999865	2.66200006	0	REVERSED Multidrug resistance protein 1 OS=Rattus norvegicus GN=Abcb1 PE=2 SV=1	RAT	0	0.883303225		0.919675112	
447	0.63	0.63	37.25000024	1.444000006	0	REVERSED Pleckstrin homology domain-containing family G member 5 OS=Rattus norvegicus GN=Plekhg5 PE=1 SV=1	RAT	0	0.883170784		0.954249918	
250	2.96	2.96	39.82999921	5.085000023	5.085000023	Hyaluronan and proteoglycan link protein 1 OS=Rattus norvegicus GN=Hapln1 PE=1 SV=2	RAT	2	0.883158505	0.630740285	0.943800628	0.670034587
248	3.06	5.18	46.00000083	17.00000018	17.00000018	Ras-related protein Rab-10 OS=Rattus norvegicus GN=Rab10 PE=2 SV=1	RAT	4	0.883041978		1.100311041	
917	0.07	0.1	32.13	0	0	REVERSED Integrin alpha-D OS=Rattus norvegicus GN=Itgad PE=2 SV=1	RAT	0	0.881926179	0.285620689	0.939745724	0.54919821
321	2	2.14	54.07999754	7.06499964	3.804000095	Endophilin-A2 OS=Rattus norvegicus GN=Sh3gl1 PE=1 SV=1	RAT	1	0.881727517		0.924104095	
268	2.59	2.61	70.23000121	8.37199986	8.37199986	C-terminal-binding protein 1 OS=Rattus norvegicus GN=Ctbp1 PE=1 SV=3	RAT	3	0.880424976	0.42160809	0.863655686	0.380573004
1021	0.06	0.06	27.41000056	0	0	REVERSED Xylosyltransferase 1 (Fragment) OS=Rattus norvegicus GN=Xylt1 PE=2 SV=1	RAT	0	0.87961942	0.596826077	0.932561219	0.754579425
843	0.09	0.09	21.78999931	0	0	REVERSED MAP kinase-interacting serine/threonine-protein kinase 1 OS=Rattus norvegicus GN=Mknk1 PE=2 SV=1	RAT	0	0.879576683	0.255713314	0.973835588	0.776309729
924	0.07	0.09	33.73000026	0	0	Ankyrin repeat and LEM domain-containing protein 2 OS=Rattus norvegicus GN=Ankle2 PE=2 SV=1	RAT	0	0.878757596		0.994241714	
1081	0.05	0.07	26.60000026	0	0	Ubiquitin carboxyl-terminal hydrolase 19 OS=Rattus norvegicus GN=Usp19 PE=1 SV=1	RAT	0	0.878743589	0.419686586	0.916797817	0.544361115
670	0.16	0.17	16.27999991	1.252999995	0	REVERSED Fibrinogen beta chain OS=Rattus norvegicus GN=Fgb PE=1 SV=4	RAT	0	0.875757396		0.89083612	
335	2	2	18.75	6.25	6.25	Calreticulin OS=Rattus norvegicus GN=Calr PE=1 SV=1	RAT	1	0.875515223		1.069322944	
279	2.25	2.25	67.96000004	8.414000273	6.148999929	Myristoylated alanine-rich C-kinase substrate OS=Rattus norvegicus GN=Marcks PE=1 SV=2	RAT	1	0.875291228	0.407908887	1.032727957	0.800847411
486	0.44	0.45	41.67000055	5.277999863	0	Protein phosphatase 1 regulatory subunit 7 OS=Rattus norvegicus GN=Ppp1r7 PE=1 SV=1	RAT	0	0.874726176	0.517506897	0.860213995	0.681079209
891	0.08	0.08	18.40000004	4.112999886	0	REVERSED Tissue alpha-L-fucosidase OS=Rattus norvegicus GN=Fuca1 PE=1 SV=1	RAT	0	0.874494672		0.896629274	
977	0.06	0.13	26.57999992	0	0	A-kinase anchor protein 6 OS=Rattus norvegicus GN=Akap6 PE=2 SV=1	RAT	0	0.874131918	0.649899006	0.991314113	0.960507214
402	1.01	1.03	31.06000125	4.924000055	4.924000055	40S ribosomal protein S3a OS=Rattus norvegicus GN=Rps3a PE=1 SV=2	RAT	1	0.8740049		0.973575771	
113	10.28	21.21	75.83000064	28.3100009	23.62000048	Heat shock protein HSP 90-beta OS=Rattus norvegicus GN=Hsp90ab1 PE=1 SV=4	RAT	13	0.873041689	0.318412006	0.973629773	0.680117726
251	2.95	3.22	84.5600009	46.97999954	18.11999977	Stathmin OS=Rattus norvegicus GN=Stmn1 PE=1 SV=2	RAT	2	0.873013616	0.154439002	0.938571215	0.44310689
1032	0.06	0.06	31.67999983	0	0	REVERSED Heterogeneous nuclear ribonucleoprotein D-like OS=Rattus norvegicus GN=Hnrpdl PE=2 SV=1	RAT	0	0.872656524	0.314247996	0.9434461	0.552967072
636	0.19	0.21	40.81999958	4.081999883	0	60S ribosomal protein L19 OS=Rattus norvegicus GN=Rpl19 PE=1 SV=1	RAT	0	0.871114194		0.946932614	
1064	0.05	0.09	31.15000129	0.648899982	0	Regulator of G-protein signaling 12 OS=Rattus norvegicus GN=Rgs12 PE=1 SV=1	RAT	0	0.870928884		0.760307074	
1044	0.05	0.25	35.71000099	0.121100002	0	Xin actin-binding repeat-containing protein 2 OS=Rattus norvegicus GN=Xirp2 PE=2 SV=1	RAT	0	0.869851828	0.1876508	0.865889072	0.210928798
858	0.08	0.13	32.73999989	1.889000088	0	Coatomer subunit beta OS=Rattus norvegicus GN=Copb1 PE=1 SV=1	RAT	0	0.868734896		0.870832503	
407	0.95	0.99	30.01999855	2.008000016	0	REVERSED Serine/threonine-protein kinase N1 OS=Rattus norvegicus GN=Pkn1 PE=1 SV=2	RAT	0	0.867298126	0.643454075	1.102502942	0.86992538
473	0.49	0.55	30.50999939	0.295199989	0	Apolipoprotein B-100 OS=Rattus norvegicus GN=Apob PE=1 SV=1	RAT	0	0.867218673	0.261909693	0.974502504	0.739161015
803	0.1	0.1	39.14000094	2.631999925	0	UPF0498 protein KIAA1191 homolog OS=Rattus norvegicus GN=Aa2-141 PE=2 SV=1	RAT	0	0.867001891		1.034258008	
313	2.02	2.02	87.30999827	3.846000135	3.846000135	ProSAAS OS=Rattus norvegicus GN=Pcsk1n PE=1 SV=1	RAT	1	0.866671324	0.400891513	0.951289713	0.713159025
578	0.24	0.24	31.20000064	7.19999969	0	REVERSED Pleckstrin homology-like domain family A member 3 OS=Rattus norvegicus GN=Phlda3 PE=2 SV=1	RAT	0	0.866601288		1.127923012	
1043	0.05	0.3	35.85000038	0.297800009	0	GRIP and coiled-coil domain-containing protein 2 OS=Rattus norvegicus GN=Gcc2 PE=1 SV=1	RAT	0	0.86517632	0.543442011	1.073225975	0.428114504
666	0.16	0.18	24.1899997	1.410000026	0	REVERSED Neuropilin-1 OS=Rattus norvegicus GN=Nrp1 PE=1 SV=1	RAT	0	0.862676919		0.928881586	
1034	0.06	0.06	22.80000001	4.560000077	0	REVERSED Thymidylate synthase OS=Rattus norvegicus GN=Tyms PE=1 SV=1	RAT	0	0.862011611		0.943984807	
590	0.22	0.31	23.39999974	1.032000035	0	REVERSED Dual oxidase 1 OS=Rattus norvegicus GN=Duox1 PE=1 SV=1	RAT	0	0.861848891	0.373483598	0.904445708	0.494877696
532	0.33	0.33	29.62000072	2.830000035	0	REVERSED Protein FAM48A OS=Rattus norvegicus GN=Fam48a PE=2 SV=1	RAT	0	0.857670188	0.662896872	0.973651826	0.842938781
1077	0.05	0.07	26.78000033	0	0	REVERSED Protocadherin Fat 3 OS=Rattus norvegicus GN=Fat3 PE=1 SV=1	RAT	0	0.856284201	0.119399697	0.967820883	0.678792715
685	0.15	0.15	36.01999879	1.200999971	0	Torsin-1A-interacting protein 1 OS=Rattus norvegicus GN=Tor1aip1 PE=1 SV=1	RAT	0	0.855987489	0.360781789	1.047773957	0.719583571
745	0.12	0.12	24.05000031	1.631999947	0	REVERSED Sarcosine dehydrogenase, mitochondrial OS=Rattus norvegicus GN=Sardh PE=1 SV=2	RAT	0	0.855933011	0.361534894	0.977768123	0.858687818
444	0.64	0.65	41.33999944	0.996300019	0	WD repeat-containing protein 78 OS=Rattus norvegicus GN=Wdr78 PE=2 SV=1	RAT	0	0.855475724		1.018407941	
841	0.09	0.09	29.2899996	2.31199991	0	Carcinoembryonic antigen-related cell adhesion molecule 1 OS=Rattus norvegicus GN=Ceacam1 PE=1 SV=3	RAT	0	0.852604389		1.166149974	
155	6.96	13.69	89.41000104	48.53000045	38.53000104	Guanine nucleotide-binding protein G(I)/G(S)/G(T) subunit beta-2 OS=Rattus norvegicus GN=Gnb2 PE=1 SV=4	RAT	10	0.851893425	0.381587297	1.058279037	0.695101321
992	0.06	0.09	25.94000101	2.786999941	0	REVERSED Phospholipase D2 OS=Rattus norvegicus GN=Pld2 PE=1 SV=2	RAT	0	0.851849914		0.840834975	
1099	0.05	0.06	30.27000129	0	0	ELKS/Rab6-interacting/CAST family member 1 OS=Rattus norvegicus GN=Erc1 PE=1 SV=1	RAT	0	0.851220071		0.706219614	
956	0.07	0.07	15.57999998	0	0	REVERSED Uncharacterized protein C2orf81 homolog OS=Rattus norvegicus PE=2 SV=1	RAT	0	0.850495219	0.450096786	1.031790018	0.806761384
1009	0.06	0.07	31.86999857	0	0	REVERSED Ribosome biogenesis protein BOP1 OS=Rattus norvegicus GN=Bop1 PE=2 SV=1	RAT	0	0.84893769	0.353371888	0.913177371	0.534992278
696	0.14	0.17	29.49999869	2.476999909	0	REVERSED Spermatogenesis-associated protein 1 OS=Rattus norvegicus GN=Spata1 PE=2 SV=1	RAT	0	0.847450316		0.849367976	
332	2	2	26.53999925	3.505999967	3.505999967	Sodium- and chloride-dependent GABA transporter 1 OS=Rattus norvegicus GN=Slc6a1 PE=1 SV=1	RAT	1	0.846504688		0.851337671	
576	0.24	0.24	21.97999954	0.729200011	0	Semaphorin-6C OS=Rattus norvegicus GN=Sema6c PE=2 SV=1	RAT	0	0.845144928	0.640251577	0.999079585	0.994383097
406	0.95	1.07	29.10999954	0.568399997	0.328999991	Breast cancer type 2 susceptibility protein homolog OS=Rattus norvegicus GN=Brca2 PE=1 SV=1	RAT	1	0.845063627		0.874297798	

348	1.89	2.74	68.48000288	7.819999754	7.819999754	Synaptotagmin-2 OS=Rattus norvegicus GN=Syt2 PE=1 SV=1	RAT	2	0.844850421		0.796865821	
805	0.1	0.1	25.49000084	4.90199998	0	REVERSED 60S ribosomal protein L15 OS=Rattus norvegicus GN=Rpl15 PE=1 SV=2	RAT	0	0.844133914	0.506416082	0.917531013	0.54439199
920	0.07	0.1	18.26000065	0.837500021	0	Kelch-like protein 21 OS=Rattus norvegicus GN=Klhl21 PE=3 SV=1	RAT	0	0.844129682		0.975335121	
637	0.19	0.2	18.70000064	2.033000067	0	Transmembrane protein 39B OS=Rattus norvegicus GN=Tmem39b PE=2 SV=1	RAT	0	0.84386301	0.338462114	1.004385948	0.972179174
994	0.06	0.09	28.81000042	4.70900014	0	Porphobilinogen deaminase OS=Rattus norvegicus GN=Hmbs PE=1 SV=2	RAT	0	0.84377718		0.935900688	
988	0.06	0.09	29.69999909	0	0	REVERSED Kinesin-1 heavy chain OS=Rattus norvegicus GN=Kif5b PE=2 SV=1	RAT	0	0.84368521	0.335916609	0.959470689	0.748135388
1133	0.05	0.05	25.40999949	1.319999993	0	WD repeat-containing protein 1 OS=Rattus norvegicus GN=Wdr1 PE=1 SV=3	RAT	0	0.843571723		0.927797914	
653	0.17	0.17	63.01000118	4.913000017	0	REVERSED Succinyl-CoA ligase [GDP-forming] subunit alpha, mitochondrial OS=Rattus norvegicus GN=Suclg1 PE=2 SV=2	RAT	0	0.842348874		0.903348684	
1141	0.05	0.05	21.86000049	3.720999882	0	Casein kinase II subunit beta OS=Rattus norvegicus GN=Csnk2b PE=1 SV=1	RAT	0	0.840076029		0.810816884	
561	0.26	0.28	15.82999974	2.422999963	0	Secretogranin-2 OS=Rattus norvegicus GN=Scg2 PE=2 SV=1	RAT	0	0.839918196		0.985144496	
852	0.08	0.26	26.33999884	0.20620001	0	Myosin-3 OS=Rattus norvegicus GN=Myh3 PE=2 SV=1	RAT	0	0.839210987		1.213274002	
305	2.04	2.04	58.09999704	7.619000226	7.619000226	Glutathione S-transferase P OS=Rattus norvegicus GN=Gstp1 PE=1 SV=2	RAT	1	0.838451505	0.338807493	0.943348587	0.666990817
986	0.06	0.1	20.74999958	1.494999975	0	Growth factor receptor-bound protein 7 OS=Rattus norvegicus GN=Grb7 PE=1 SV=1	RAT	0	0.837010384		0.961442888	
1085	0.05	0.07	33.12000036	0	0	REVERSED Zinc finger protein DZIP1L OS=Rattus norvegicus GN=Dzip1l PE=2 SV=1	RAT	0	0.833716094		1.119379044	
921	0.07	0.09	29.78999913	0	0	PHD and RING finger domain-containing protein 1 OS=Rattus norvegicus GN=Phrf1 PE=1 SV=2	RAT	0	0.833344519		0.952495873	
629	0.19	2.27	70.19000053	11.06000021	5.288000032	Ras-related protein Rab-6A OS=Rattus norvegicus GN=Rab6a PE=2 SV=2	RAT	1	0.828022003		1.010208964	
499	0.4	0.4	54.22000289	3.89200002	2.501999959	Electrogenic sodium bicarbonate cotransporter 1 OS=Rattus norvegicus GN=Slc4a4 PE=1 SV=1	RAT	1	0.824467182	0.301253289	1.143694997	0.4917714
339	2	2	88.45999837	19.23000067	19.23000067	Neurogranin OS=Rattus norvegicus GN=Nrgn PE=1 SV=1	RAT	1	0.823016405		0.697235227	
1061	0.05	0.1	50.40000081	0	0	REVERSED Keratin, type II cytoskeletal 1 OS=Rattus norvegicus GN=Krt1 PE=2 SV=1	RAT	0	0.817976594	0.299515396	1.037122965	0.778627813
380	1.39	1.41	16.60999954	3.80599983	3.80599983	Purine nucleoside phosphorylase OS=Rattus norvegicus GN=Pnp PE=1 SV=1	RAT	1	0.816842318	0.316565514	0.919458091	0.551975071
711	0.13	0.2	23.97000045	1.217	0	IQ and AAA domain-containing protein 1-like OS=Rattus norvegicus GN=Iqca1p1 PE=2 SV=2	RAT	0	0.815876126		0.9185552	
691	0.14	0.4	28.09999883	1.879999973	0	REVERSED Adenylate cyclase type 4 OS=Rattus norvegicus GN=Adcy4 PE=2 SV=1	RAT	0	0.813292921		0.937521517	
1035	0.06	0.06	30.48999906	2.845999971	0	REVERSED Proteasome subunit alpha type-6 OS=Rattus norvegicus GN=Psma6 PE=1 SV=1	RAT	0	0.812808514		0.914138913	
875	0.08	0.09	20.53000033	0	0	Epithelial splicing regulatory protein 2 OS=Rattus norvegicus GN=Esrp2 PE=2 SV=1	RAT	0	0.812549472	0.399789214	1.044648051	0.735093474
369	1.61	1.63	34.92000103	1.217999961	1.217999961	Rho guanine nucleotide exchange factor 2 OS=Rattus norvegicus GN=Arhgef2 PE=2 SV=1	RAT	1	0.809066117	0.090309419	0.90046382	0.173633203
747	0.12	0.12	51.33000016	4.255000129	0	Arginine/serine-rich coiled-coil protein 2 OS=Rattus norvegicus GN=Rsrc2 PE=2 SV=1	RAT	0	0.8081249	0.121770598	0.912899911	0.520260215
1139	0.05	0.05	60.55999994	4.930000007	0	ATP synthase lipid-binding protein, mitochondrial OS=Rattus norvegicus GN=Atp5g3 PE=2 SV=1	RAT	0	0.807396829		0.922084928	
957	0.07	0.07	47.67000079	5.426000059	0	REVERSED Synapse differentiation-inducing gene protein 1 OS=Rattus norvegicus GN=Syndig1 PE=2 SV=1	RAT	0	0.805480719		1.02077198	
404	0.96	0.96	52.07999945	5.832999945	5.832999945	Proteasome subunit beta type-1 OS=Rattus norvegicus GN=Psmb1 PE=1 SV=3	RAT	1	0.805127501		0.914520681	
779	0.1	0.21	28.58999968	2.563999966	0	Potassium/sodium hyperpolarization-activated cyclic nucleotide-gated channel 3 OS=Rattus norvegicus GN=Hcn3 PE=2 SV=1	RAT	0	0.803876281		0.880803406	
724	0.13	0.13	14.98000026	0.74260002	0	REVERSED Leucine-rich repeat-containing protein 41 OS=Rattus norvegicus GN=Lrrc41 PE=2 SV=1	RAT	0	0.803789914		0.968184471	
1020	0.06	0.06	35.13000011	1.107000001	0	TRMT1-like protein OS=Rattus norvegicus GN=Trmt1l PE=2 SV=1	RAT	0	0.803775787		0.771679819	
1097	0.05	0.07	11.64999977	2.981000021	0	REVERSED Tyrosine-protein phosphatase non-receptor type 5 OS=Rattus norvegicus GN=Ptpn5 PE=2 SV=2	RAT	0	0.803355217		1.065325022	
944	0.07	0.08	18.00000072	0.833300035	0	DNA polymerase alpha subunit B OS=Rattus norvegicus GN=Pola2 PE=2 SV=2	RAT	0	0.802886128		0.87403971	
1031	0.06	0.06	17.79000014	1.252999995	0	Cold shock domain-containing protein E1 OS=Rattus norvegicus GN=Csde1 PE=2 SV=1	RAT	0	0.802886128		0.87403971	
860	0.08	0.12	60.89000106	1.544999983	0	REVERSED Cullin-associated NEDD8-dissociated protein 1 OS=Rattus norvegicus GN=Cand1 PE=1 SV=1	RAT	0	0.802204788	0.508196712	1.000058055	0.999619305
688	0.15	0.15	11.99999973	1.71399992	0	Protein FAM118B OS=Rattus norvegicus GN=Fam118b PE=2 SV=1	RAT	0	0.801885724		1.071212053	
577	0.24	0.24	34.77999866	3.187999874	0	Ribosome production factor 1 OS=Rattus norvegicus GN=Rpf1 PE=2 SV=2	RAT	0	0.801627874	0.250476003	0.85596031	0.1235618
788	0.1	0.13	24.07999933	1.840000041	0	Aspartyl-tRNA synthetase, mitochondrial OS=Rattus norvegicus GN=Dars2 PE=2 SV=1	RAT	0	0.797746718		0.902975678	
614	0.2	1.41	45.62999904	5.330000073	1.919000037	Desmin OS=Rattus norvegicus GN=Des PE=2 SV=2	RAT	1	0.797311425	0.460240185	0.828581095	0.515876174
510	0.37	0.39	40.65999985	1.367000025	1.367000025	REVERSED Kinesin-like protein KIF1C OS=Rattus norvegicus GN=Kif1c PE=1 SV=1	RAT	1	0.786701083		0.97848171	
1128	0.05	0.05	15.92999995	0	0	REVERSED Semaphorin-3A OS=Rattus norvegicus GN=Sema3a PE=2 SV=1	RAT	0	0.786379278		0.793766022	
706	0.13	4.17	90.56000113	28.88999879	17.77999997	ADP-ribosylation factor 5 OS=Rattus norvegicus GN=Arf5 PE=1 SV=2	RAT	2	0.783687711		0.896233797	
896	0.07	0.21	28.2799989	0.202000001	0	Myosin-IXb OS=Rattus norvegicus GN=Myo9b PE=1 SV=1	RAT	0	0.779908419	0.420686811	0.852867305	0.530708313
1049	0.05	0.15	26.53000057	1.28899999	0	Scaffold attachment factor B1 OS=Rattus norvegicus GN=Safb PE=1 SV=2	RAT	0	0.776207685	0.331224412	0.901237428	0.484089911
424	0.72	0.72	43.97999942	1.91200003	1.91200003	REST corepressor 2 OS=Rattus norvegicus GN=Rcor2 PE=2 SV=1	RAT	1	0.772347987		0.947809875	
501	0.4	0.4	11.32000014	1.617000066	0	Protein NDRG2 OS=Rattus norvegicus GN=Ndrg2 PE=1 SV=1	RAT	0	0.769137323	0.229567498	0.880182922	0.41980651
787	0.1	0.13	48.08000028	2.559999935	0	REVERSED ProSAP-interacting protein 1 OS=Rattus norvegicus GN=Prosapip1 PE=1 SV=2	RAT	0	0.767265081	0.267996907	1.115494967	0.488029987
355	1.81	1.81	35.65999866	2.150999941	2.150999941	Abhydrolase domain-containing protein 16A OS=Rattus norvegicus GN=Abhd16a PE=2 SV=1	RAT	1	0.766885817	0.233975098	0.936567128	0.637171924
400	1.03	1.03	41.29999876	4.857999831	4.857999831	Acidic leucine-rich nuclear phosphoprotein 32 family member A OS=Rattus norvegicus GN=Anp32a PE=2 SV=1	RAT	1	0.76390028		0.953097284	
323	2	2.07	70.77000141	1.965999976	1.965999976	Trifunctional enzyme subunit alpha, mitochondrial OS=Rattus norvegicus GN=Hadha PE=1 SV=2	RAT	1	0.757308781		0.961523771	
1119	0.05	0.06	28.36000025	8.955000341	0	ATP synthase protein 8 OS=Rattus norvegicus GN=Mt-atp8 PE=1 SV=1	RAT	0	0.735721886		0.864794314	
1087	0.05	0.07	18.72999966	1.727000065	0	Guanylate cyclase 2G OS=Rattus norvegicus GN=Gucy2g PE=2 SV=2	RAT	0	0.730520189		0.782266319	
1019	0.06	0.06	32.49999881	0	0	Multidrug resistance protein 1 OS=Rattus norvegicus GN=Abcb1 PE=2 SV=1	RAT	0	0.730149806		0.909934878	
443	0.64	0.66	75.41000247	2.127999999	0	REVERSED Neurofilament medium polypeptide OS=Rattus norvegicus GN=Nefm PE=1 SV=4	RAT	0	0.729873776		0.926533282	
993	0.06	0.09	63.73999715	4.944999889	0	Uncharacterized protein C8orf42 homolog OS=Rattus norvegicus PE=2 SV=2	RAT	0	0.713079572		0.823220789	
1018	0.06	0.06	41.83999896	0.933100004	0	REVERSED CLIP-associating protein 2 OS=Rattus norvegicus GN=Clasp2 PE=2 SV=1	RAT	0	0.712625921		1.052765965	
1050	0.05	0.13	22.05000073	0	0	PH domain leucine-rich repeat protein phosphatase 1 OS=Rattus norvegicus GN=Phlpp1 PE=1 SV=1	RAT	0	0.687790811		0.899613917	
602	0.21	0.97	32.35000074	4.778999835	1.837999932	F-actin-capping protein subunit beta OS=Rattus norvegicus GN=Capzb PE=1 SV=1	RAT	1	0.679885626		1.083997011	
830	0.09	0.1	25.99999905	0.705899997	0	REVERSED Coiled-coil and C2 domain-containing protein 1B OS=Rattus norvegicus GN=Cc2d1b PE=2 SV=2	RAT	0	0.66885078		0.892305791	
1040	0.06	0.06	30.25999963	9.211000055	0	REVERSED Small VCP/p97-interacting protein OS=Rattus norvegicus GN=Svip PE=1 SV=1	RAT	0	0.66885078		0.892305791	
663	0.16	0.2	34.2200011	1.823000051	0	Nucleolin OS=Rattus norvegicus GN=Ncl PE=1 SV=3	RAT	0	0.642157316		0.700537622	
760	0.11	0.13	31.43000007	2.040999942	0	REVERSED Dual specificity protein kinase CLK3 OS=Rattus norvegicus GN=Clk3 PE=2 SV=2	RAT	0	0.628039181		0.745841622	
912	0.07	0.11	18.86000037	0.948399957	0	MAM domain-containing glycosylphosphatidylinositol anchor protein 2 OS=Rattus norvegicus GN=Mdga2 PE=2 SV=1	RAT	0	0.566261113		0.738467813	
7	0	47.95	93.33000183	80.80000281	75.73000193	Actin, cytoplasmic 1 OS=Rattus norvegicus GN=Actb PE=1 SV=1	RAT	73				
69	0	45.22	91.78000093	77.10999846	73.78000021	Tubulin alpha-3 chain OS=Rattus norvegicus GN=Tuba3a PE=2 SV=1	RAT	120				
82	0	13.45	81.22000098	46.41000032	45.30000091	ADP-ribosylation factor 1 OS=Rattus norvegicus GN=Arf1 PE=1 SV=2	RAT	10				
161	0.01	5.95	87.40000129	22.05000073	22.05000073	Histone H2B type 1-A OS=Rattus norvegicus GN=Hist1h2ba PE=1 SV=2	RAT	3				
170	0	6.2	80.4700017	36.71999872	29.69000041	Ubiquitin-60S ribosomal protein L40 OS=Rattus norvegicus GN=Uba52 PE=1 SV=2	RAT	3				
170	0	6.2	90.99000096	58.02000165	46.90999985	Polyubiquitin-C OS=Rattus norvegicus GN=Ubc PE=1 SV=1	RAT	3				
170	0	6.2	91.14999771	61.64000034	49.84000027	Polyubiquitin-B OS=Rattus norvegicus GN=Ubb PE=1 SV=1	RAT	3				
229	0	6.21	74.29000139	18.93000007	10.72999984	Guanine nucleotide-binding protein G(k) subunit alpha OS=Rattus norvegicus GN=Gnai3 PE=1 SV=3	RAT	3				
243	0	2.91	53.79999876	13.59000057	13.59000057	Ras-related protein Rap-1b OS=Rattus norvegicus GN=Rap1b PE=2 SV=2	RAT	2				
247	0.01	3.06	93.01999807	24.02999997	21.70999944	Histone H2A.J OS=Rattus norvegicus GN=H2afj PE=2 SV=1	RAT	2				

247	0.01	3.02	98.46000075	34.61999893	21.53999954	Histone H2A type 1-F OS=Rattus norvegicus PE=3 SV=3	RAT	2
247	0	3.12	91.54000282	23.8499999	21.53999954	Histone H2A type 2-A OS=Rattus norvegicus GN=Hist2h2aa3 PE=1 SV=1	RAT	2
247	0	3.04	91.54000282	23.8499999	21.53999954	Histone H2A type 4 OS=Rattus norvegicus PE=2 SV=2	RAT	2
247	0	3.03	91.54000282	23.8499999	21.53999954	Histone H2A type 1-E OS=Rattus norvegicus PE=1 SV=2	RAT	2
247	0	3.03	91.54000282	23.8499999	21.53999954	Histone H2A type 1-C OS=Rattus norvegicus PE=1 SV=2	RAT	2
247	0	3	91.54000282	23.8499999	21.53999954	Histone H2A type 3 OS=Rattus norvegicus PE=2 SV=3	RAT	2
248	0.01	3.65	86.47000194	14.01000023	10.62999964	Ras-related protein Rab-8A OS=Rattus norvegicus GN=Rab8a PE=2 SV=2	RAT	2
248	0.01	3.64	53.61999869	14.01000023	10.62999964	Ras-related protein Rab-8B OS=Rattus norvegicus GN=Rab8b PE=1 SV=1	RAT	2
248	0.01	1.42	71.42999768	5.418999866	5.418999866	Ras-related protein Rab-13 OS=Rattus norvegicus GN=Rab13 PE=2 SV=2	RAT	1
248	0	3.66	54.72999811	14.42999989	10.94999984	Ras-related protein Rab-1B OS=Rattus norvegicus GN=Rab1b PE=1 SV=1	RAT	2
248	0	3.53	61.69000268	10.94999984	10.94999984	Ras-related protein Rab-35 OS=Rattus norvegicus GN=Rab35 PE=2 SV=1	RAT	2
248	0	3.47	44.33999956	10.37999988	10.37999988	Ras-related protein Rab-15 OS=Rattus norvegicus GN=Rab15 PE=2 SV=1	RAT	2
261	0	2.78	20.47999948	3.24199982	3.24199982	Ezrin OS=Rattus norvegicus GN=Ezr PE=1 SV=3	RAT	2
264	0	1.72	30.23999929	2.428000048	2.428000048	Serine/threonine-protein phosphatase 2A 55 kDa regulatory subunit B delta isoform OS=Rattus norvegicus GN=Ppp2r2d PE=2 SV=1	RAT	1
277	0	2	26.98999941	2.410000004	2.410000004	Heterogeneous nuclear ribonucleoprotein F OS=Rattus norvegicus GN=Hnrnpf PE=1 SV=3	RAT	1
280	0	9.27	60.53000093	13.42000067	9.047999978	Heat shock 70 kDa protein 1-like OS=Rattus norvegicus GN=Hspa1l PE=2 SV=2	RAT	6
290	0	45.22	91.78000093	77.10999846	73.78000021	Tubulin alpha-3 chain OS=Rattus norvegicus GN=Tuba3a PE=2 SV=1	RAT	120
295	2.06	3	93.4400022	20.30999959	15.62999934	Heterogeneous nuclear ribonucleoprotein A1 OS=Rattus norvegicus GN=Hnrnpa1 PE=1 SV=3	RAT	3
304	2.04	2.06	59.4900012	1.982999966	1.982999966	Heat shock protein 75 kDa, mitochondrial OS=Rattus norvegicus GN=Trap1 PE=1 SV=1	RAT	1
306	0	4.02	44.24000084	13.03000003	13.03000003	Serine/threonine-protein phosphatase PP1-alpha catalytic subunit OS=Rattus norvegicus GN=Ppp1ca PE=1 SV=1	RAT	3
330	2	2	54.8600018	3.24300006	3.24300006	Peptidyl-prolyl cis-trans isomerase D OS=Rattus norvegicus GN=Ppid PE=1 SV=3	RAT	1
374	0	1.35	54.8699975	3.064000048	3.064000048	Guanine nucleotide-binding protein subunit alpha-11 OS=Rattus norvegicus GN=Gna11 PE=2 SV=1	RAT	1
376	0	1.39	24.41000044	3.150000051	3.150000051	cAMP-dependent protein kinase type I-alpha regulatory subunit OS=Rattus norvegicus GN=Prkar1a PE=2 SV=2	RAT	1
401	1.02	1.02	26.49999857	0.466000009	0.466000009	Multidrug resistance-associated protein 6 OS=Rattus norvegicus GN=Abcc6 PE=2 SV=1	RAT	1
401	0	1.02	37.38999963	2.077000029	2.077000029	Transaldolase OS=Rattus norvegicus GN=Taldo1 PE=1 SV=2	RAT	1
409	0.93	0.94	27.36000121	5.660000071	5.660000071	REVERSED WAP four-disulfide core domain protein 1 OS=Rattus norvegicus GN=Wfdc1 PE=1 SV=1	RAT	1
420	0.79	0.79	37.61000037	2.531999908	2.531999908	REVERSED Copine-9 OS=Rattus norvegicus GN=Cpne9 PE=2 SV=1	RAT	1
425	0.72	0.72	14.98000026	1.009999961	1.009999961	REVERSED Cryptochrome-2 OS=Rattus norvegicus GN=Cry2 PE=2 SV=1	RAT	1
425	0.02	0.06	41.99000001	1.656999998	0	REVERSED Sterol-4-alpha-carboxylate 3-dehydrogenase, decarboxylating OS=Rattus norvegicus GN=Nsdhl PE=2 SV=1	RAT	0
425	0	0.75	74.26999807	1.592000015	1.592000015	REVERSED NADH dehydrogenase [ubiquinone] 1 alpha subcomplex subunit 9, mitochondrial OS=Rattus norvegicus GN=Ndufa9 PE=1 SV=2	RAT	1
434	0.68	0.68	13.78999949	2.122000046	2.122000046	Histone deacetylase 8 OS=Rattus norvegicus GN=Hdac8 PE=2 SV=1	RAT	1
442	0.65	0.65	37.92999983	3.448000178	3.448000178	REVERSED Secreted phosphoprotein 24 OS=Rattus norvegicus GN=Spp2 PE=2 SV=2	RAT	1
449	0.62	0.62	45.3399986	11.80000007	0	Probable tRNA threonylcarbamoyladenosine biosynthesis protein Osgep (Fragment) OS=Rattus norvegicus GN=Osgep PE=2 SV=1	RAT	0
450	0.61	0.61	17.79000014	2.453999966	0	REVERSED Serine/threonine-protein kinase pim-3 OS=Rattus norvegicus GN=Pim3 PE=1 SV=3	RAT	0
452	0.6	0.6	43.32999885	1.499000005	1.499000005	Midline-1 OS=Rattus norvegicus GN=Mid1 PE=2 SV=1	RAT	1
455	0.58	0.58	43.95999908	2.349000052	0	REVERSED Mitochondrial 2-oxodicarboxylate carrier OS=Rattus norvegicus GN=Slc25a21 PE=2 SV=1	RAT	0
464	0.55	0.55	17.04999954	2.841000073	0	Protein NDRG4 OS=Rattus norvegicus GN=Ndrg4 PE=2 SV=1	RAT	0
465	0.54	0.58	39.68999982	0.699299993	0	Arf-GAP with SH3 domain, ANK repeat and PH domain-containing protein 1 OS=Rattus norvegicus GN=Asap1 PE=2 SV=2	RAT	0
475	0	0.47	25.20999908	0.72730002	0	Hypoxia-inducible factor 1-alpha OS=Rattus norvegicus GN=Hif1a PE=2 SV=1	RAT	0
478	0.48	0.5	15.79000056	7.237000018	0	Mitochondrial fission 1 protein OS=Rattus norvegicus GN=Fis1 PE=1 SV=1	RAT	0
480	0.47	0.47	28.63999903	0.734400004	0	Protein kintoun OS=Rattus norvegicus GN=Dnaaf2 PE=2 SV=2	RAT	0
480	0	0.03	34.77999866	3.260999918	0	Gastrokine-2 OS=Rattus norvegicus GN=Gkn2 PE=2 SV=1	RAT	0
487	0.44	0.44	42.1299994	7.343000174	7.343000174	NADP-dependent malic enzyme OS=Rattus norvegicus GN=Me1 PE=1 SV=2	RAT	1
488	0.43	0.46	27.57000029	1.992999949	0	PEX5-related protein OS=Rattus norvegicus GN=Pex5l PE=1 SV=1	RAT	0
491	0.42	0.45	30.7099998	4.148999974	0	Glutathione S-transferase omega-1 OS=Rattus norvegicus GN=Gsto1 PE=1 SV=2	RAT	0
492	0.42	0.44	36.14000082	1.944999956	0	REVERSED Filensin (Fragment) OS=Rattus norvegicus GN=Bfsp1 PE=2 SV=2	RAT	0
495	0.42	0.42	27.77999938	2.431000024	0	Phenazine biosynthesis-like domain-containing protein OS=Rattus norvegicus GN=Pbld PE=2 SV=1	RAT	0
500	0.4	0.4	33.39000046	1.42099997	0	REVERSED Beta-catenin-like protein 1 OS=Rattus norvegicus GN=Ctnnbl1 PE=2 SV=1	RAT	0
501	0	0.38	35.3700012	2.040999942	0	Cyclin-G1 OS=Rattus norvegicus GN=Ccng1 PE=2 SV=2	RAT	0
504	0.38	0.43	15.0000006	1.061000023	0	Protein O-linked-mannose beta-1,2-N-acetylglucosaminyltransferase 1 OS=Rattus norvegicus GN=Pomgnt1 PE=2 SV=1	RAT	0
508	0.38	0.38	22.36000001	4.348000139	0	REVERSED ATP synthase subunit d, mitochondrial OS=Rattus norvegicus GN=Atp5h PE=1 SV=3	RAT	0
512	0.37	0.37	34.36000049	1.322000008	0	REVERSED Uncharacterized protein C8orf45 homolog OS=Rattus norvegicus PE=2 SV=1	RAT	0
514	0.36	0.38	26.37000084	1.185000036	0	Intraflagellar transport protein 81 homolog OS=Rattus norvegicus GN=Ift81 PE=2 SV=1	RAT	0
520	0	0.35	23.25000018	4.058999941	0	Calretinin OS=Rattus norvegicus GN=Calb2 PE=1 SV=1	RAT	0
525	0.34	0.4	26.14000142	1.38299996	1.38299996	REVERSED Threonyl-tRNA synthetase, mitochondrial OS=Rattus norvegicus GN=Tars2 PE=2 SV=1	RAT	1
531	0.33	0.35	31.00000024	3.500000015	0	Cysteine dioxygenase type 1 OS=Rattus norvegicus GN=Cdo1 PE=1 SV=1	RAT	0
535	0.32	1.24	25.6099999	1.190999988	0.270699989	REVERSED Peripheral-type benzodiazepine receptor-associated protein 1 OS=Rattus norvegicus GN=Bzrap1 PE=1 SV=2	RAT	1
540	0.31	0.46	24.94000047	1.892000064	0	REVERSED Hamartin OS=Rattus norvegicus GN=Tsc1 PE=1 SV=1	RAT	0
545	0.3	0.3	19.14000064	1.154999994	0	Kelch repeat and BTB domain-containing protein 10 OS=Rattus norvegicus GN=Kbtbd10 PE=1 SV=1	RAT	0
547	0.29	0.31	23.17000031	2.015000023	0	DnaJ homolog subfamily A member 1 OS=Rattus norvegicus GN=Dnaja1 PE=2 SV=1	RAT	0
552	0.28	0.29	25.24999976	1.654000022	0	Glutamate receptor 1 OS=Rattus norvegicus GN=Gria1 PE=1 SV=2	RAT	0
553	0.28	0.29	25.88	4.152999818	0	Mu-crystallin homolog OS=Rattus norvegicus GN=Crym PE=1 SV=1	RAT	0
563	0.25	0.29	28.76000106	2.35399995	0	REVERSED Short transient receptor potential channel 4 OS=Rattus norvegicus GN=Trpc4 PE=1 SV=2	RAT	0
568	0.25	0.25	40.27999938	1.389000006	0	REVERSED Pre-mRNA-processing factor 19 OS=Rattus norvegicus GN=Prpf19 PE=2 SV=2	RAT	0
569	0.25	0.25	34.11999941	3.937000036	0	REVERSED NF-kappa-B inhibitor-like protein 1 OS=Rattus norvegicus GN=Nfkbil1 PE=2 SV=2	RAT	0
579	0	0.24	26.06000006	4.930000007	0	Microtubule-associated proteins 1A/1B light chain 3B OS=Rattus norvegicus GN=Map1lc3b PE=1 SV=3	RAT	0
589	0.23	0.23	12.89000064	5.031000078	5.031000078	Solute carrier family 25 member 34 OS=Rattus norvegicus GN=Slc25a34 PE=2 SV=2	RAT	1
595	0	0.2	28.42000127	1.496000029	0	REVERSED Neurexin-1-beta OS=Rattus norvegicus GN=Nrxn1 PE=1 SV=1	RAT	0
599	0.22	0.22	13.40000033	4.574999958	0	Macrophage asialoglycoprotein-binding protein 1 OS=Rattus norvegicus GN=Mgl1 PE=1 SV=1	RAT	0
601	0.21	1.3	68.62999797	7.379999757	2.95199994	Arginine and glutamate-rich protein 1 OS=Rattus norvegicus GN=Arglu1 PE=2 SV=1	RAT	1
610	0.21	0.21	10.44000015	3.846000135	0	Ferritin heavy chain OS=Rattus norvegicus GN=Fth1 PE=1 SV=3	RAT	0
617	0.2	0.25	8.122999966	4.202000052	0	UDP-GlcNAc:betaGal beta-1,3-N-acetylglucosaminyltransferase-like protein 1 OS=Rattus norvegicus GN=B3gntl1 PE=2 SV=1	RAT	0
625	0.2	0.2	38.83000016	3.190999851	0	REVERSED 60S ribosomal protein L18 OS=Rattus norvegicus GN=Rpl18 PE=2 SV=2	RAT	0
674	0.16	0.16	30.45000136	4.478000104	0	POU domain, class 5, transcription factor 2 OS=Rattus norvegicus GN=Pou5f2 PE=2 SV=2	RAT	0

676	0	23.68	95.23000121	58.35999846	53.32000256	Actin, aortic smooth muscle OS=Rattus norvegicus GN=Acta2 PE=1 SV=1	RAT	31		
676	0	21.48	89.6600008	61.79999709	56.49999976	Actin, alpha skeletal muscle OS=Rattus norvegicus GN=Acta1 PE=2 SV=1	RAT	27		
676	0	19.28	95.74000239	51.59999728	46.54000103	Actin, gamma-enteric smooth muscle OS=Rattus norvegicus GN=Actg2 PE=2 SV=1	RAT	25		
677	0.15	0.25	28.34999859	0.581899984	0	Metabotropic glutamate receptor 5 OS=Rattus norvegicus GN=Grm5 PE=1 SV=2	RAT	0		
679	0.15	0.19	34.94000137	1.727999933	0	REVERSED Leucine-rich repeat-containing protein 8A OS=Rattus norvegicus GN=Lrrc8a PE=2 SV=1	RAT	0		
686	0	0.15	18.71999949	2.978999913	0	REVERSED RAD9, RAD1, HUS1-interacting nuclear orphan protein OS=Rattus norvegicus GN=Rhino PE=2 SV=1	RAT	0		
699	0.14	0.15	8.931999654	1.164999977	0	REVERSED Fatty acyl-CoA reductase 1 OS=Rattus norvegicus GN=Far1 PE=2 SV=1	RAT	0		
701	0.14	0.14	35.58000028	2.404000051	0	Kelch-like ECH-associated protein 1 OS=Rattus norvegicus GN=Keap1 PE=2 SV=1	RAT	0		
703	0.14	0.14	54.28000093	2.974000014	2.974000014	Signal recognition particle receptor subunit beta OS=Rattus norvegicus GN=Srprb PE=2 SV=1	RAT	1		
723	0.13	0.13	21.35999948	0.784699991	0	Nitric oxide synthase, inducible OS=Rattus norvegicus GN=Nos2 PE=1 SV=2	RAT	0		
727	0.12	0.2	26.46999955	8.332999796	0	40S ribosomal protein S5 OS=Rattus norvegicus GN=Rps5 PE=2 SV=3	RAT	0		
731	0.12	0.16	19.33999956	0.875900034	0	Insulin-like growth factor 1 receptor OS=Rattus norvegicus GN=Igf1r PE=2 SV=2	RAT	0		
734	0.12	0.14	75.73999763	8.679000288	4.733999819	REVERSED Fumarate hydratase, mitochondrial OS=Rattus norvegicus GN=Fh PE=1 SV=1	RAT	1		
735	0.12	0.14	23.69000018	2.379000001	0	REVERSED Protein-tyrosine kinase 2-beta OS=Rattus norvegicus GN=Ptk2b PE=1 SV=1	RAT	0		
740	0.12	0.13	38.17000091	3.279000148	0	Protein FAM46B OS=Rattus norvegicus GN=Fam46b PE=2 SV=1	RAT	0		
742	0.12	0.13	11.94000021	2.070000023	0	REVERSED Nuclear receptor subfamily 4 group A member 3 OS=Rattus norvegicus GN=Nr4a3 PE=2 SV=1	RAT	0		
749	0.12	0.12	36.50000095	5.353000015	0	Class E basic helix-loop-helix protein 40 OS=Rattus norvegicus GN=Bhlhe40 PE=2 SV=1	RAT	0		
751	0.12	0.12	34.799999	11.8900001	0	REVERSED Cytochrome c oxidase subunit 2 OS=Rattus norvegicus GN=Mtco2 PE=2 SV=2	RAT	0		
764	0.11	0.12	30.55000007	0.643099984	0	REVERSED DNA mismatch repair protein Msh2 OS=Rattus norvegicus GN=Msh2 PE=2 SV=1	RAT	0		
765	0.11	0.12	33.88000131	0.952399988	0	REVERSED Serine/threonine-protein kinase BRSK2 OS=Rattus norvegicus GN=Brsk2 PE=2 SV=1	RAT	0		
765	0	0.11	44.72999871	0.899699982	0	REVERSED Serine/threonine-protein kinase BRSK1 OS=Rattus norvegicus GN=Brsk1 PE=1 SV=1	RAT	0		
768	0.11	0.11	31.88999891	1.367999986	0	Lon protease homolog, mitochondrial OS=Rattus norvegicus GN=Lonp1 PE=2 SV=1	RAT	0		
769	0.11	0.11	32.24000037	3.64000015	0	Insulin-like growth factor 2 mRNA-binding protein 1 OS=Rattus norvegicus GN=Igf2bp1 PE=1 SV=1	RAT	0		
771	0.11	0.11	25.78999996	2.42999997	0	Atrial natriuretic peptide receptor 3 OS=Rattus norvegicus GN=Npr3 PE=2 SV=1	RAT	0		
773	0	0.11	18.08999926	3.72299999	0	Glandular kallikrein-3, submandibular (Fragment) OS=Rattus norvegicus GN=Klk3 PE=2 SV=2	RAT	0		
778	0.1	0.27	28.49000096	1.303999964	0	REVERSED Synaptonemal complex protein 1 OS=Rattus norvegicus GN=Sycp1 PE=2 SV=2	RAT	0		
799	0.1	0.1	35.67999899	2.057999931	0	REVERSED Epsin-2 OS=Rattus norvegicus GN=Epn2 PE=1 SV=1	RAT	0		
806	0.1	0.1	23.86000007	3.268000111	0	REVERSED Macrophage asialoglycoprotein-binding protein 1 OS=Rattus norvegicus GN=Mgl1 PE=1 SV=1	RAT	0		
807	0.1	0.1	5.49999997	3.500000015	0	REVERSED Cysteine dioxygenase type 1 OS=Rattus norvegicus GN=Cdo1 PE=1 SV=1	RAT	0		
811	0.09	0.16	31.83000088	0.502499985	0	REVERSED Putative ATP-dependent RNA helicase DHX30 OS=Rattus norvegicus GN=Dhx30 PE=2 SV=1	RAT	0		
811	0	0.13	27.18999982	0.795799959	0	Coiled-coil domain-containing protein 164 OS=Rattus norvegicus GN=Ccdc164 PE=2 SV=1	RAT	0		
814	0.09	0.14	13.1400004	1.703000069	0	REVERSED Potassium channel subfamily K member 3 OS=Rattus norvegicus GN=Kcnk3 PE=2 SV=1	RAT	0		
821	0.09	0.12	37.95000017	9.069000185	0	REVERSED Carboxypeptidase A1 OS=Rattus norvegicus GN=Cpa1 PE=2 SV=2	RAT	0		
850	0.08	0.91	15.32000005	3.153000027	1.350999996	Tryptophan 5-hydroxylase 1 OS=Rattus norvegicus GN=Tph1 PE=2 SV=2	RAT	1		
859	0.08	0.13	34.43999887	3.641999885	0	Deoxyhypusine hydroxylase OS=Rattus norvegicus GN=Dohh PE=2 SV=1	RAT	0		
862	0.08	0.11	24.61999953	0.71109999	0	Telomerase reverse transcriptase OS=Rattus norvegicus GN=Tert PE=2 SV=1	RAT	0		
869	0.08	0.1	26.76999867	1.114999969	0	N-terminal kinase-like protein OS=Rattus norvegicus GN=Scyl1 PE=2 SV=1	RAT	0		
887	0.08	0.08	19.26999986	1.116999984	0	REVERSED Solute carrier organic anion transporter family member 1C1 OS=Rattus norvegicus GN=Slco1c1 PE=1 SV=1	RAT	0		
890	0.08	0.08	13.4800002	2.696999907	0	REVERSED Protein cereblon OS=Rattus norvegicus GN=Crbn PE=1 SV=1	RAT	0		
894	0.07	0.33	36.05999947	3.189999983	0	Centlein (Fragment) OS=Rattus norvegicus GN=Cntln PE=2 SV=1	RAT	0		
897	0.07	0.18	18.29999983	1.559999958	0	Dynein intermediate chain 1, axonemal OS=Rattus norvegicus GN=Dnai1 PE=2 SV=1	RAT	0		
906	0.07	0.14	28.0099988	1.851999946	0	REVERSED Adenosylhomocysteinase OS=Rattus norvegicus GN=Ahcy PE=1 SV=3	RAT	0		
942	0.07	0.08	26.28999949	1.29300002	0	Glycine receptor subunit alpha-3 OS=Rattus norvegicus GN=Glra3 PE=2 SV=1	RAT	0		
943	0.07	0.08	21.64999992	2.118000016	0	Intermediate conductance calcium-activated potassium channel protein 4 OS=Rattus norvegicus GN=Kcnn4 PE=2 SV=2	RAT	0		
945	0.07	0.08	25.08000135	2.006999962	0	REVERSED Troponin T, cardiac muscle OS=Rattus norvegicus GN=Tnnt2 PE=1 SV=2	RAT	0		
947	0.07	0.07	68.48999858	6.239999831	0	REVERSED Heat shock 70 kDa protein 1A/1B OS=Rattus norvegicus GN=Hspa1a PE=2 SV=2	RAT	0		
953	0.07	0.07	57.51000047	5.750999972	0	Fumarylacetoacetate hydrolase domain-containing protein 2 OS=Rattus norvegicus GN=Fahd2 PE=1 SV=1	RAT	0		
954	0.07	0.07	19.64000016	1.785999909	0	REVERSED Death-associated protein kinase 3 OS=Rattus norvegicus GN=Dapk3 PE=1 SV=1	RAT	0		
959	0.06	2.53	49.66000021	4.482999817	2.068999968	Dynamin-2 OS=Rattus norvegicus GN=Dnm2 PE=1 SV=1	RAT	2		
985	0.06	0.1	28.76000106	1.042999979	0	Spermatid perinuclear RNA-binding protein OS=Rattus norvegicus GN=Strbp PE=1 SV=1	RAT	0		
991	0.06	0.09	27.9399991	2.205999941	0	Usher syndrome type-1C protein-binding protein 1 OS=Rattus norvegicus GN=Ushbp1 PE=2 SV=1	RAT	0		
1024	0.06	0.06	27.52000093	1.455000043	0	5E5 antigen OS=Rattus norvegicus PE=2 SV=1	RAT	0		
1026	0.06	0.06	22.48000056	0	0	E3 ubiquitin-protein ligase UHRF1 OS=Rattus norvegicus GN=Uhrf1 PE=2 SV=2	RAT	0		
1036	0.06	0.06	21.10999972	3.055999987	0	REVERSED Inward rectifier potassium channel 13 OS=Rattus norvegicus GN=Kcnj13 PE=2 SV=1	RAT	0		
1068	0.05	0.08	22.41999954	1.265999954	0	Voltage-dependent P/Q-type calcium channel subunit alpha-1A OS=Rattus norvegicus GN=Cacna1a PE=1 SV=1	RAT	0		
1071	0.05	0.08	34.5299989	0.631600013	0	REVERSED Lon protease homolog, mitochondrial OS=Rattus norvegicus GN=Lonp1 PE=2 SV=1	RAT	0		
1071	0	0.06	8.828999847	1.018999983	0	Muscarinic acetylcholine receptor M3 OS=Rattus norvegicus GN=Chrm3 PE=1 SV=1	RAT	0		
1073	0.05	0.08	27.5000006	1.183999982	0	Rho GTPase-activating protein 20 OS=Rattus norvegicus GN=Arhgap20 PE=2 SV=2	RAT	0		
1086	0.05	0.07	59.89000201	3.621000051	0	REVERSED Pyruvate dehydrogenase E1 component subunit beta, mitochondrial OS=Rattus norvegicus GN=Pdhb PE=1 SV=2	RAT	0		
1092	0.05	0.07	44.35000122	2.301000059	0	REVERSED Patatin-like phospholipase domain-containing protein 2 OS=Rattus norvegicus GN=Pnpla2 PE=2 SV=1	RAT	0		
1098	0.05	0.06	40.75999856	0.580799999	0	REVERSED Potassium-transporting ATPase alpha chain 1 OS=Rattus norvegicus GN=Atp4a PE=2 SV=3	RAT	0		
1106	0.05	0.06	49.88999963	1.290000044	0	E3 ubiquitin-protein ligase parkin OS=Rattus norvegicus GN=Park2 PE=1 SV=1	RAT	0		
1110	0.05	0.06	24.07999933	0	0	Rap guanine nucleotide exchange factor 4 (Fragment) OS=Rattus norvegicus GN=Rapgef4 PE=2 SV=1	RAT	0		
1116	0.05	0.06	17.28000045	1.553000044	0	REVERSED UPF0464 protein C15orf44 homolog OS=Rattus norvegicus PE=2 SV=1	RAT	0		
1117	0.05	0.06	37.95000017	4.910999909	0	Claudin-19 OS=Rattus norvegicus GN=Cldn19 PE=2 SV=1	RAT	0		
1118	0.05	0.06	21.67000026	4.444000125	0	REVERSED Insulin-like growth factor II OS=Rattus norvegicus GN=Igf2 PE=1 SV=1	RAT	0		
1123	0.05	0.05	24.25000072	2.425000072	0	Glutamate [NMDA] receptor subunit epsilon-3 OS=Rattus norvegicus GN=Grin2c PE=1 SV=1	RAT	0		
1124	0.05	0.05	34.4599992	0	0	REVERSED Mitogen-activated protein kinase kinase kinase 12 OS=Rattus norvegicus GN=Map3k12 PE=1 SV=1	RAT	0		
1130	0.05	0.05	36.62999868	1.484999992	0	REVERSED 5-azacytidine-induced protein 2 OS=Rattus norvegicus GN=Azi2 PE=2 SV=1	RAT	0		
1139	0	0.05	37.59000003	4.964999855	0	ATP synthase lipid-binding protein, mitochondrial OS=Rattus norvegicus GN=Atp5g2 PE=2 SV=1	RAT	0		
1139	0	0.05	55.87999821	5.14700003	0	ATP synthase lipid-binding protein, mitochondrial OS=Rattus norvegicus GN=Atp5g1 PE=1 SV=1	RAT	0		
1142	0.05	0.05	5.235999823	3.141000122	0	Calcium-activated potassium channel subunit beta-1 OS=Rattus norvegicus GN=Kcnmb1 PE=2 SV=4	RAT	0		
N	Unused	Total	%Cov	%Cov(50)	%Cov(95)	Name	Species	Peptides(95%)	116:115	PVal 116:115

1	122.02	122.02	90.53000212	49.27000105	37.09999919	Spectrin alpha chain, brain OS=Rattus norvegicus GN=Sptan1 PE=1 SV=2	RAT	83	1.00425303	0.806862473
2	74.38	74.38	77.67000198	50.20999908	38.98999989	Clathrin heavy chain 1 OS=Rattus norvegicus GN=Cltc PE=1 SV=3	RAT	58	0.983734071	0.522550523
3	73.73	73.8	86.87000275	54.29000258	47.78000116	Sodium/potassium-transporting ATPase subunit alpha-3 OS=Rattus norvegicus GN=Atp1a3 PE=1 SV=2	RAT	55	1.004536033	0.887523711
4	52.47	52.47	94.87000108	91.74000025	88.38999867	Tubulin alpha-4A chain OS=Rattus norvegicus GN=Tuba4a PE=2 SV=1	RAT	128	0.981916487	0.83401382
5	51.27	51.49	95.28999925	71.93999887	66.2899971	Pyruvate kinase isozymes M1/M2 OS=Rattus norvegicus GN=Pkm2 PE=1 SV=3	RAT	37	0.983139515	0.620271504
6	50.1	50.1	86.51999831	83.14999938	80.44999838	Tubulin beta-2A chain OS=Rattus norvegicus GN=Tubb2a PE=1 SV=1	RAT	88	1.009174943	0.893610001
7	47.95	47.95	97.6000011	80.80000281	75.73000193	Actin, cytoplasmic 2 OS=Rattus norvegicus GN=Actg1 PE=1 SV=1	RAT	73	1.053627014	0.466044307
7	0	47.95	93.33000183	80.80000281	75.73000193	Actin, cytoplasmic 1 OS=Rattus norvegicus GN=Actb PE=1 SV=1	RAT	73		
8	46.56	46.66	90.78000188	63.47000003	57.31999874	ATP synthase subunit alpha, mitochondrial OS=Rattus norvegicus GN=Atp5a1 PE=1 SV=2	RAT	35	1.001080036	0.977985322
9	46.03	46.03	93.94000173	63.63999844	57.58000016	Syntaxin-binding protein 1 OS=Rattus norvegicus GN=Stxbp1 PE=1 SV=1	RAT	34	0.982756078	0.620549679
10	45.54	45.54	93.95999908	82.41999745	74.44999814	Fructose-bisphosphate aldolase A OS=Rattus norvegicus GN=Aldoa PE=1 SV=2	RAT	38	1.018641949	0.716568708
11	45.12	45.12	97.68000245	54.3299973	45.35999894	Heat shock cognate 71 kDa protein OS=Rattus norvegicus GN=Hspa8 PE=1 SV=1	RAT	32	1.051087022	0.151258498
12	44.29	44.29	91.67000055	67.86000133	58.09999704	2',3'-cyclic-nucleotide 3'-phosphodiesterase OS=Rattus norvegicus GN=Cnp PE=1 SV=2	RAT	51	1.104735017	0.001313483
13	42.96	42.96	95.3700006	48.84000123	35.76000035	Dynamin-1 OS=Rattus norvegicus GN=Dnm1 PE=1 SV=2	RAT	28	0.975227714	0.555558503
14	42.04	42.04	91.33999944	63.48999739	57.66999722	Synapsin-1 OS=Rattus norvegicus GN=Syn1 PE=1 SV=3	RAT	30	0.974568427	0.523089111
15	41.52	41.52	99.21000004	84.50999856	77.17000246	Creatine kinase B-type OS=Rattus norvegicus GN=Ckb PE=1 SV=2	RAT	51	0.939033329	0.129936904
16	41.34	41.43	99.23999906	68.23999882	63.8899982	ATP synthase subunit beta, mitochondrial OS=Rattus norvegicus GN=Atp5b PE=1 SV=2	RAT	51	1.02029705	0.58274442
17	40.36	40.44	100	89.78999853	84.97999907	Glyceraldehyde-3-phosphate dehydrogenase OS=Rattus norvegicus GN=Gapdh PE=1 SV=3	RAT	79	0.926583171	0.125733703
18	39.64	39.64	82.17999935	49.73999858	46.7900008	Aconitate hydratase, mitochondrial OS=Rattus norvegicus GN=Aco2 PE=1 SV=2	RAT	24	1.007171988	0.8555035
20	36.96	37.04	84.04999971	59.38000083	50.81999898	Serum albumin OS=Rattus norvegicus GN=Alb PE=1 SV=2	RAT	28	0.649255097	6.45E-09
21	36.18	36.18	98.18000197	89.09000158	89.09000158	Brain acid soluble protein 1 OS=Rattus norvegicus GN=Basp1 PE=1 SV=2	RAT	26	0.949621499	0.420698613
22	36.05	36.05	86.15999818	50.5400002	38.98000121	Vesicle-fusing ATPase OS=Rattus norvegicus GN=Nsf PE=1 SV=1	RAT	19	0.971226513	0.537291884
23	35.51	35.59	95.99000216	51.66000128	50.78999996	60 kDa heat shock protein, mitochondrial OS=Rattus norvegicus GN=Hspd1 PE=1 SV=1	RAT	23	1.038926959	0.425683886
24	34.8	34.8	94.70000267	72.11999893	70.05000114	Gamma-enolase OS=Rattus norvegicus GN=Eno2 PE=1 SV=2	RAT	42	1.038133979	0.336619914
25	32.89	32.98	73.93000126	26.62999928	25.20000041	Plasma membrane calcium-transporting ATPase 1 OS=Rattus norvegicus GN=Atp2b1 PE=2 SV=2	RAT	19	1.042935014	0.581642687
26	32.16	32.16	85.55999994	53.35000157	45.82000077	Calcium/calmodulin-dependent protein kinase type II subunit alpha OS=Rattus norvegicus GN=Camk2a PE=1 SV=1	RAT	49	1.015777946	0.74104172
27	31.02	31.11	81.04000092	40.38000107	37.23999858	Heat shock protein HSP 90-alpha OS=Rattus norvegicus GN=Hsp90aa1 PE=1 SV=3	RAT	22	1.048823953	0.352472097
28	30.93	31.56	96.49999738	69.41000223	57.8700006	Dihydropyrimidinase-related protein 2 OS=Rattus norvegicus GN=Dpysl2 PE=1 SV=1	RAT	36	1.02156496	0.630835474
29	30.74	30.94	96.31000161	62.74999976	54.36000228	ADP/ATP translocase 1 OS=Rattus norvegicus GN=Slc25a4 PE=1 SV=3	RAT	21	1.045654058	0.680754721
30	29.37	29.46	96.28000259	75.34999847	67.9099977	Aspartate aminotransferase, mitochondrial OS=Rattus norvegicus GN=Got2 PE=1 SV=2	RAT	22	0.999419212	0.994246185
32	28.31	28.31	97.03999758	74.26000237	66.86000228	Malate dehydrogenase, mitochondrial OS=Rattus norvegicus GN=Mdh2 PE=1 SV=2	RAT	21	0.988264382	0.755465984
33	28.2	28.64	63.20000291	6.782999635	4.349999875	Cytoplasmic dynein 1 heavy chain 1 OS=Rattus norvegicus GN=Dync1h1 PE=1 SV=1	RAT	15	0.995398879	0.909320295
34	28.11	28.11	72.32999802	30.50000072	27.55999863	Hexokinase-1 OS=Rattus norvegicus GN=Hk1 PE=1 SV=4	RAT	22	0.985318303	0.639094889
35	28.06	28.14	90.71999788	69.16000247	56.88999891	L-lactate dehydrogenase B chain OS=Rattus norvegicus GN=Ldhb PE=1 SV=2	RAT	19	0.966861784	0.425517708
36	27.18	27.26	79.90000248	19.0200001	11.98000014	Microtubule-associated protein 2 OS=Rattus norvegicus GN=Map2 PE=1 SV=3	RAT	14	1.008957982	0.783902586
37	26.65	57.08	78.49000096	43.39999855	41.3500011	Sodium/potassium-transporting ATPase subunit alpha-1 OS=Rattus norvegicus GN=Atp1a1 PE=1 SV=1	RAT	44	1.056367993	0.195483506
39	25.05	25.05	83.67000222	56.73000216	56.73000216	14-3-3 protein zeta/delta OS=Rattus norvegicus GN=Ywhaz PE=1 SV=1	RAT	21	0.930878103	0.126759201
40	24.75	24.93	73.44999909	10.33999994	8.291000128	Spectrin beta chain, brain 2 OS=Rattus norvegicus GN=Sptbn2 PE=1 SV=2	RAT	14	1.072749019	0.276142508
42	22.58	22.99	68.23999882	10.74000001	10.53000018	Microtubule-associated protein 1A OS=Rattus norvegicus GN=Map1a PE=1 SV=1	RAT	17	1.015377045	0.721542716
43	22.41	22.41	86.94000244	53.43999863	42.98999906	Synaptotagmin-1 OS=Rattus norvegicus GN=Syt1 PE=1 SV=3	RAT	17	0.963698328	0.437824309
44	22.38	22.41	95.57999969	67.47000217	64.6600008	Triosephosphate isomerase OS=Rattus norvegicus GN=Tpi1 PE=1 SV=2	RAT	20	1.088533998	0.074374221
45	22.16	29.06	97.69999981	57.59999752	54.61000204	Alpha-enolase OS=Rattus norvegicus GN=Eno1 PE=1 SV=4	RAT	34	1.008141041	0.844803572
47	21.04	21.11	82.96999931	32.80000091	32.80000091	Glucose-6-phosphate isomerase OS=Rattus norvegicus GN=Gpi PE=1 SV=1	RAT	15	0.994268	0.876901686
48	20.95	20.95	94.24999952	73.44999909	57.08000064	Neuromodulin OS=Rattus norvegicus GN=Gap43 PE=1 SV=1	RAT	13	1.053843021	0.669250607
49	20.67	20.67	86.66999936	55.37999868	46.66999876	Myelin basic protein S OS=Rattus norvegicus GN=Mbp PE=1 SV=3	RAT	20	1.290603042	7.82E-05
50	20.17	20.17	73.29999804	18.67000014	16.93000048	Excitatory amino acid transporter 2 OS=Rattus norvegicus GN=Slc1a2 PE=1 SV=2	RAT	11	1.099717021	0.117511302
52	19.6	19.6	99.26999807	92.69999862	92.69999862	Beta-synuclein OS=Rattus norvegicus GN=Sncb PE=1 SV=1	RAT	16	1.111302018	0.183157101
53	19.48	19.6	80.04000187	41.83999896	36.84999943	Serine/threonine-protein phosphatase 2B catalytic subunit alpha isoform OS=Rattus norvegicus GN=Ppp3ca PE=1 SV=1	RAT	14	0.939212024	0.479328305
54	19.07	38.35	86.66999936	64.88999724	58.89000297	Tubulin beta-3 chain OS=Rattus norvegicus GN=Tubb3 PE=1 SV=1	RAT	87	1.006610036	0.938007176
55	18.76	19.18	93.66999865	53.3100009	45.48000097	L-lactate dehydrogenase A chain OS=Rattus norvegicus GN=Ldha PE=1 SV=1	RAT	15	0.895494103	0.163020298
56	18.24	18.24	92.51999855	64.56999779	53.53999734	Phosphoglycerate mutase 1 OS=Rattus norvegicus GN=Pgam1 PE=1 SV=4	RAT	23	1.112149	0.375672013
57	18.22	18.22	71.56999707	22.21000046	20.55999935	6-phosphofructokinase type C OS=Rattus norvegicus GN=Pfkp PE=1 SV=2	RAT	13	0.939812779	0.255329788
58	18.09	18.09	82.03999996	56.80000186	54.36999798	Synaptosomal-associated protein 25 OS=Rattus norvegicus GN=Snap25 PE=1 SV=1	RAT	12	0.983065784	0.809708774
59	17.84	17.84	82.96999931	41.29000008	33.46000016	V-type proton ATPase subunit B, brain isoform OS=Rattus norvegicus GN=Atp6v1b2 PE=1 SV=1	RAT	12	1.035380006	0.454381198
60	17.4	17.4	95.24000287	95.24000287	86.39000058	Hemoglobin subunit beta-1 OS=Rattus norvegicus GN=Hbb PE=1 SV=3	RAT	14	0.917338312	0.163855895
61	17.3	17.3	96.46999836	71.38000131	68.55000257	Voltage-dependent anion-selective channel protein 1 OS=Rattus norvegicus GN=Vdac1 PE=1 SV=4	RAT	17	0.910621583	0.062905587
62	17.18	17.18	95.12000084	70.73000073	70.73000073	Peptidyl-prolyl cis-trans isomerase A OS=Rattus norvegicus GN=Ppia PE=1 SV=2	RAT	20	1.026793003	0.635373175
63	16.96	16.96	84.99000072	36.32000089	25.90999901	Aspartate aminotransferase, cytoplasmic OS=Rattus norvegicus GN=Got1 PE=1 SV=3	RAT	9	0.932758212	0.112077899
64	16.87	16.87	66.10000134	21.63999975	13.1099999	AP-2 complex subunit alpha-2 OS=Rattus norvegicus GN=Ap2a2 PE=1 SV=3	RAT	9	0.948184788	0.252499908
65	16.73	16.73	87.52999902	46.03999853	35.96999943	Phosphoglycerate kinase 1 OS=Rattus norvegicus GN=Pgk1 PE=1 SV=2	RAT	9	1.06043005	0.228786901
66	16.3	16.31	76.84000134	54.79999781	46.32999897	Guanine nucleotide-binding protein G(o) subunit alpha OS=Rattus norvegicus GN=Gnao1 PE=1 SV=2	RAT	15	0.978684187	0.767862916
67	16.16	16.41	73.03000093	20.53000033	17.41999984	Glycogen phosphorylase, brain form (Fragment) OS=Rattus norvegicus GN=Pygb PE=1 SV=3	RAT	13	0.953203976	0.398703992
68	16	23.85	83.74999762	60.61000228	46.00999951	Fructose-bisphosphate aldolase C OS=Rattus norvegicus GN=Aldoc PE=1 SV=3	RAT	24	1.015288949	0.784194171
69	15.94	50.59	93.56999993	86.47000194	84.70000029	Tubulin alpha-1A chain OS=Rattus norvegicus GN=Tuba1a PE=1 SV=1	RAT	152	0.971328914	0.716304719
69	0	45.22	91.78000093	77.10999846	73.78000021	Tubulin alpha-3 chain OS=Rattus norvegicus GN=Tuba3a PE=2 SV=1	RAT	120		
70	15.46	15.46	90.96000195	43.61000061	43.61000061	Dihydrolipoyl dehydrogenase, mitochondrial OS=Rattus norvegicus GN=Dld PE=1 SV=1	RAT	15	0.95621109	0.458855212
71	15.17	15.17	98.18999767	62.65000105	56.62999749	Cofilin-1 OS=Rattus norvegicus GN=Cfl1 PE=1 SV=3	RAT	14	1.059687972	0.212993398
72	15.15	16.84	91.62999988	35.12000144	29.53000069	Glial fibrillary acidic protein OS=Rattus norvegicus GN=Gfap PE=1 SV=2	RAT	9	1.13045001	0.087032892
73	14.99	15.3	70.09000182	16.59000069	9.588000178	Sarcoplasmic/endoplasmic reticulum calcium ATPase 2 OS=Rattus norvegicus GN=Atp2a2 PE=1 SV=1	RAT	7	0.956442177	0.298133612
74	14.74	14.74	75.8400023	69.80000138	69.80000138	Calmodulin OS=Rattus norvegicus GN=Calm1 PE=1 SV=2	RAT	17	0.88223052	0.226329297
75	14.68	14.68	92.94000268	69.70999837	57.64999986	Guanine nucleotide-binding protein G(I)/G(S)/G(T) subunit beta-1 OS=Rattus norvegicus GN=Gnb1 PE=1 SV=4	RAT	12	0.978810787	0.864772499
76	14.45	14.51	48.08999896	13.24999928	13.24999928	V-type proton ATPase 116 kDa subunit a isoform 1 OS=Rattus norvegicus GN=Atp6v0a1 PE=2 SV=1	RAT	10	0.960623026	0.469917208
77	14.25	51.31	83.92000198	42.84000099	38.42999935	Sodium/potassium-transporting ATPase subunit alpha-2 OS=Rattus norvegicus GN=Atp1a2 PE=1 SV=1	RAT	37	1.14279902	0.08757133
78	14.02	14.05	98.58999848	83.79999995	71.13000154	Hemoglobin subunit alpha-1/2 OS=Rattus norvegicus GN=Hba1 PE=1 SV=3	RAT	23	0.992566228	0.897084892

79	13.75	13.75	71.05000019	37.52999902	35.38999856	Glutamine synthetase OS=Rattus norvegicus GN=Glul PE=1 SV=3	RAT	13	0.972257316	0.579918325
80	13.54	13.54	98.58999848	32.85999894	32.85999894	Peroxiredoxin-5, mitochondrial OS=Rattus norvegicus GN=Prdx5 PE=1 SV=1	RAT	7	0.986400783	0.846178889
81	13.51	13.51	76.45999789	29.17000055	27.5000006	Cytochrome b-c1 complex subunit 1, mitochondrial OS=Rattus norvegicus GN=Uqcrc1 PE=1 SV=1	RAT	10	1.002432942	0.968867719
82	13.5	13.5	71.27000093	46.41000032	45.30000091	ADP-ribosylation factor 3 OS=Rattus norvegicus GN=Arf3 PE=1 SV=2	RAT	10	0.972434521	0.732462525
82	0	13.45	81.22000098	46.41000032	45.30000091	ADP-ribosylation factor 1 OS=Rattus norvegicus GN=Arf1 PE=1 SV=2	RAT	10		
83	13.41	13.41	78.64000201	28.0099988	24.05000031	Dihydrolipoyllysine-residue acetyltransferase component of pyruvate dehydrogenase complex, mitochondrial OS=Rattus norvegicus GN=Dlat PE=1 SV=3	RAT	8	1.083274007	0.233695403
84	12.92	12.96	70.34999728	27.64999866	23.22999984	Cytochrome b-c1 complex subunit 2, mitochondrial OS=Rattus norvegicus GN=Uqcrc2 PE=1 SV=2	RAT	14	0.934857726	0.321403503
85	12.83	12.83	69.41000223	41.78000093	38.82000148	Sodium/potassium-transporting ATPase subunit beta-1 OS=Rattus norvegicus GN=Atp1b1 PE=1 SV=1	RAT	12	1	1
86	12.71	12.78	75.63999891	38.20999861	32.82000124	Pyruvate dehydrogenase E1 component subunit alpha, somatic form, mitochondrial OS=Rattus norvegicus GN=Pdha1 PE=1 SV=2	RAT	7	0.950530291	0.435996085
87	12.71	12.71	82.01000094	42.86000133	42.86000133	Protein DJ-1 OS=Rattus norvegicus GN=Park7 PE=1 SV=1	RAT	7	1.102287054	0.197188199
88	12.66	12.83	69.599998	39.55999911	37.36000061	ATP synthase subunit gamma, mitochondrial OS=Rattus norvegicus GN=Atp5c1 PE=1 SV=2	RAT	7	1.076267958	0.294720292
89	12.62	12.69	87.38999963	24.68000054	21.63999975	Microtubule-associated protein 6 OS=Rattus norvegicus GN=Map6 PE=1 SV=1	RAT	7	1.008677006	0.938365996
90	12.59	12.59	83.03999901	54.46000099	49.54999983	Peroxiredoxin-6 OS=Rattus norvegicus GN=Prdx6 PE=1 SV=3	RAT	7	1.043980002	0.523793995
91	12.44	12.44	82.26000071	23.95000011	18.97999942	Transitional endoplasmic reticulum ATPase OS=Rattus norvegicus GN=Vcp PE=1 SV=3	RAT	7	1.004623055	0.931759417
92	12.42	12.44	78.14000249	37.45000064	27.05999911	Elongation factor 1-alpha 1 OS=Rattus norvegicus GN=Eef1a1 PE=1 SV=1	RAT	11	1.065369964	0.444575489
93	12.34	16.65	80.00000119	45.1000005	36.86000109	14-3-3 protein epsilon OS=Rattus norvegicus GN=Ywhae PE=1 SV=1	RAT	13	0.941461504	0.231813297
94	11.95	11.95	75.44999719	28.51999998	22.74000049	Myelin proteolipid protein OS=Rattus norvegicus GN=Plp1 PE=1 SV=2	RAT	9	0.986772716	0.813086092
95	11.67	11.67	82.99000263	41.67000055	38.53999972	Syntaxin-1B OS=Rattus norvegicus GN=Stx1b PE=1 SV=1	RAT	9	0.955801487	0.439103514
96	11.59	11.59	88.94000053	27.63999999	27.63999999	cAMP-dependent protein kinase type II-beta regulatory subunit OS=Rattus norvegicus GN=Prkar2b PE=1 SV=3	RAT	7	0.998375177	0.987314522
97	11.49	11.49	61.87999845	16.15999937	14.36000019	Disks large homolog 4 OS=Rattus norvegicus GN=Dlg4 PE=1 SV=1	RAT	6	1.003239036	0.955254376
98	11.37	11.56	62.51000166	10.27000025	8.516000211	Solute carrier family 12 member 5 OS=Rattus norvegicus GN=Slc12a5 PE=1 SV=2	RAT	7	1.020324945	0.763517022
99	11.34	15.61	75.19999743	26.01999938	26.01999938	14-3-3 protein eta OS=Rattus norvegicus GN=Ywhah PE=1 SV=2	RAT	9	0.828960299	0.223741293
100	11.3	11.3	88.59000206	43.18000078	25.49999952	Rab GDP dissociation inhibitor alpha OS=Rattus norvegicus GN=Gdi1 PE=1 SV=1	RAT	13	0.98033762	0.721784115
101	11.24	11.24	100	45.21999955	45.21999955	Prohibitin OS=Rattus norvegicus GN=Phb PE=1 SV=1	RAT	8	1.02462399	0.732546389
102	11.15	11.15	86.3499999	29.24999893	29.24999893	Pyruvate dehydrogenase E1 component subunit beta, mitochondrial OS=Rattus norvegicus GN=Pdhb PE=1 SV=2	RAT	9	0.959604919	0.520418406
103	11.02	11.05	85.32999754	38.9200002	31.43999875	Malate dehydrogenase, cytoplasmic OS=Rattus norvegicus GN=Mdh1 PE=1 SV=3	RAT	6	1.029165983	0.674049377
104	11	11	76.17999911	30.03999889	20.81999928	Citrate synthase, mitochondrial OS=Rattus norvegicus GN=Cs PE=1 SV=1	RAT	14	0.939497828	0.244374007
105	10.95	13.96	91.86999798	45.21999955	32.0600003	Creatine kinase U-type, mitochondrial OS=Rattus norvegicus GN=Ckmt1 PE=1 SV=1	RAT	8	1.026587963	0.582327485
106	10.9	10.95	83.1099987	12.63000071	6.649000198	Microtubule-associated protein tau OS=Rattus norvegicus GN=Mapt PE=1 SV=3	RAT	5	1.133633971	0.02886316
107	10.85	10.9	60.98999977	15.57999998	11.87999994	Alpha-actinin-1 OS=Rattus norvegicus GN=Actn1 PE=1 SV=1	RAT	8	0.881242096	0.379426807
108	10.72	10.72	71.32999897	21.44999951	19.9300006	Neural cell adhesion molecule 1 OS=Rattus norvegicus GN=Ncam1 PE=1 SV=1	RAT	10	0.941088676	0.395706505
109	10.7	10.79	55.14000058	5.652999878	4.758000001	Microtubule-associated protein 1B OS=Rattus norvegicus GN=Map1b PE=1 SV=2	RAT	7	0.945223093	0.553043187
110	10.51	10.52	79.49000001	11.15000024	10.00000015	6-phosphofructokinase, muscle type OS=Rattus norvegicus GN=Pfkm PE=2 SV=3	RAT	6	0.963422716	0.552214384
111	10.37	10.58	59.83999968	9.892000258	8.815000206	Contactin-1 OS=Rattus norvegicus GN=Cntn1 PE=1 SV=2	RAT	8	1.105519056	0.247245297
112	10.29	10.34	69.41000223	35.69000065	31.43999875	Heterogeneous nuclear ribonucleoproteins A2/B1 OS=Rattus norvegicus GN=Hnrnpa2b1 PE=1 SV=1	RAT	8	0.972171009	0.650127888
113	10.28	21.21	75.83000064	28.3100009	23.62000048	Heat shock protein HSP 90-beta OS=Rattus norvegicus GN=Hsp90ab1 PE=1 SV=4	RAT	13	0.916011512	0.594471872
114	10.11	10.19	65.14000297	23.99999946	23.99999946	NAD-dependent deacetylase sirtuin-2 OS=Rattus norvegicus GN=Sirt2 PE=1 SV=1	RAT	9	1.078889012	0.642801106
115	10.01	10.04	66.92000031	7.336000353	7.336000353	Reticulon-1 OS=Rattus norvegicus GN=Rtn1 PE=2 SV=1	RAT	5	0.905191481	0.434997708
116	9.89	9.9	80.58999777	72.35000134	59.40999985	Calcineurin subunit B type 1 OS=Rattus norvegicus GN=Ppp3r1 PE=1 SV=2	RAT	5	0.925565481	0.387600005
117	9.86	9.87	57.81000257	10.61000004	9.441000223	Heat shock protein 105 kDa OS=Rattus norvegicus GN=Hsph1 PE=1 SV=1	RAT	6	1.009935021	0.900785029
118	9.85	9.85	66.75999761	43.47000122	36.0799998	Endophilin-A1 OS=Rattus norvegicus GN=Sh3gl2 PE=1 SV=2	RAT	8	0.956355572	0.521812916
119	9.83	21.18	83.75999928	40.77000022	34.86999869	Calcium/calmodulin-dependent protein kinase type II subunit beta OS=Rattus norvegicus GN=Camk2b PE=1 SV=1	RAT	37	0.937276423	0.347697794
120	9.78	9.78	100	52.42999792	31.06999993	Histone H4 OS=Rattus norvegicus GN=Hist1h4b PE=1 SV=2	RAT	8	1.142148972	0.055291969
121	9.7	16.54	79.54999804	39.34000134	32.51999915	Dihydropyrimidinase-related protein 1 OS=Rattus norvegicus GN=Crmp1 PE=1 SV=1	RAT	11	1.056316972	0.435695499
122	9.56	9.56	86.10000014	81.81999922	63.09999824	Phosphatidylethanolamine-binding protein 1 OS=Rattus norvegicus GN=Pebp1 PE=1 SV=3	RAT	14	1.159705043	0.072603352
123	9.43	9.49	79.97000217	9.572999924	9.572999924	Stress-70 protein, mitochondrial OS=Rattus norvegicus GN=Hspa9 PE=1 SV=3	RAT	5	1.105010986	0.053850532
124	9.16	9.18	77.74999738	22.24999964	16.76000059	Succinyl-CoA ligase [GDP-forming] subunit alpha, mitochondrial OS=Rattus norvegicus GN=Suclg1 PE=2 SV=2	RAT	5	1.038432002	0.650031626
125	8.98	9.04	81.27999902	12.15000004	12.15000004	Mitochondrial inner membrane protein (Fragment) OS=Rattus norvegicus GN=Immt PE=1 SV=1	RAT	6	0.972337484	0.720447183
126	8.9	8.96	60.86999774	51.09000206	51.09000206	Protein S100-B OS=Rattus norvegicus GN=S100b PE=1 SV=2	RAT	7	1.090742946	0.410652608
127	8.73	47.98	86.51999831	82.46999979	80.44999838	Tubulin beta-2C chain OS=Rattus norvegicus GN=Tubb2c PE=1 SV=1	RAT	91	0.972759128	
128	8.62	12.39	82.06999898	36.71999872	36.71999872	Elongation factor 1-alpha 2 OS=Rattus norvegicus GN=Eef1a2 PE=2 SV=1	RAT	20	1.089782953	0.587937593
129	8.49	8.54	97.75999784	50.22000074	46.63999975	Ubiquitin carboxyl-terminal hydrolase isozyme L1 OS=Rattus norvegicus GN=Uchl1 PE=1 SV=2	RAT	14	0.993457019	0.948835373
130	8.44	8.44	74.00000095	20.08000016	13.19999993	NADH-ubiquinone oxidoreductase 75 kDa subunit, mitochondrial OS=Rattus norvegicus GN=Ndufs1 PE=1 SV=1	RAT	5	0.946473897	0.465784401
131	8.43	8.5	90.31999707	27.82000005	24.1899997	Tropomyosin alpha-3 chain OS=Rattus norvegicus GN=Tpm3 PE=1 SV=2	RAT	5	1.055755973	0.392582804
132	8.29	8.31	85.42000055	42.71000028	37.85000145	Syntaxin-1A OS=Rattus norvegicus GN=Stx1a PE=1 SV=1	RAT	7	1.053326964	0.460045904
133	8.24	8.27	60.08999944	23.38999957	14.67999965	Septin-7 OS=Rattus norvegicus GN=Sept7 PE=1 SV=1	RAT	5	1.053660035	0.580035388
134	8.22	8.22	71.49000168	16.77999943	14.93999958	AP-2 complex subunit mu OS=Rattus norvegicus GN=Ap2m1 PE=1 SV=1	RAT	7	1.027269006	0.694219828
135	8.18	13.77	83.78999829	17.1299994	14.21999931	78 kDa glucose-regulated protein OS=Rattus norvegicus GN=Hspa5 PE=1 SV=1	RAT	9	1.086287022	0.206525698
136	8.15	8.15	59.46999788	44.04999912	44.04999912	Protein-L-isoaspartate(D-aspartate) O-methyltransferase OS=Rattus norvegicus GN=Pcmt1 PE=1 SV=2	RAT	7	0.955994487	0.737380385
137	8.09	8.18	65.67999721	16.24000072	16.24000072	Ubiquitin thioesterase OTUB1 OS=Rattus norvegicus GN=Otub1 PE=1 SV=1	RAT	5	0.946335912	0.708997071
138	8.09	8.12	53.90999913	9.97299999	7.547000051	Synaptic vesicle glycoprotein 2A OS=Rattus norvegicus GN=Sv2a PE=1 SV=2	RAT	4	0.953429222	0.6273911
139	8.07	10.55	80.52999973	13.38	9.272000194	Dynamin-1-like protein OS=Rattus norvegicus GN=Dnm1l PE=1 SV=1	RAT	6	0.943544686	0.693580687
140	8.05	8.08	76.10999942	24.77999926	24.77999926	V-type proton ATPase subunit E 1 OS=Rattus norvegicus GN=Atp6v1e1 PE=1 SV=1	RAT	7	1.057409048	0.748781681
141	8.05	8.05	74.29000139	42.14000106	42.14000106	Profilin-2 OS=Rattus norvegicus GN=Pfn2 PE=1 SV=3	RAT	5	1.049659014	0.621077478
142	7.78	7.79	64.09999728	14.00000006	12.16999963	Fascin OS=Rattus norvegicus GN=Fscn1 PE=1 SV=2	RAT	5	0.898971677	0.103478998
143	7.76	13.82	94.33000088	55.87000251	51.41999722	14-3-3 protein gamma OS=Rattus norvegicus GN=Ywhag PE=1 SV=2	RAT	14	0.894899786	0.31280601
144	7.62	7.62	80.00000119	19.17999983	19.17999983	Myelin-oligodendrocyte glycoprotein OS=Rattus norvegicus GN=Mog PE=1 SV=1	RAT	4	0.90418911	0.250709593
145	7.48	7.48	82.88000226	47.94999957	42.46999919	Cytochrome c oxidase subunit 5A, mitochondrial OS=Rattus norvegicus GN=Cox5a PE=1 SV=1	RAT	6	1.017235041	0.892625213
146	7.42	7.42	97.39999771	74.02999997	61.69000268	Superoxide dismutase [Cu-Zn] OS=Rattus norvegicus GN=Sod1 PE=1 SV=2	RAT	5	1.153632998	0.096246406
147	7.38	7.41	66.67000055	21.95000052	21.95000052	Septin-5 OS=Rattus norvegicus GN=Sept5 PE=1 SV=2	RAT	7	0.940122187	0.597087085
148	7.36	7.4	59.50000286	10.98000035	10.98000035	Glutaminase kidney isoform, mitochondrial OS=Rattus norvegicus GN=Gls PE=1 SV=2	RAT	4	0.963620424	0.61430037
149	7.21	7.21	79.42000031	16.86999947	7.682000101	Neurochondrin OS=Rattus norvegicus GN=Ncdn PE=1 SV=2	RAT	4	1.019991994	0.780230403
150	7.18	7.18	71.82999849	17.46000051	13.51999938	NADH dehydrogenase [ubiquinone] 1 alpha subcomplex subunit 10, mitochondrial OS=Rattus norvegicus GN=Ndufa10 PE=1 SV=1	RAT	7	1.156383991	0.327101201
151	7.11	7.11	66.36000276	33.18000138	23.18000048	Ras-related protein Rab-3A OS=Rattus norvegicus GN=Rab3a PE=1 SV=1	RAT	5	1.036453009	0.695489407

152	7.1	7.1	89.20000196	22.06999958	22.06999958	ATP synthase subunit O, mitochondrial OS=Rattus norvegicus GN=Atp5o PE=1 SV=1	RAT	6	1.126708984	0.25777179
153	7.09	7.09	83.24999809	47.63999879	47.63999879	Visinin-like protein 1 OS=Rattus norvegicus GN=Vsnl1 PE=1 SV=2	RAT	7	1.055253029	0.596961677
154	7.08	7.08	68.83000135	24.16000068	24.16000068	Isocitrate dehydrogenase [NAD] subunit beta, mitochondrial OS=Rattus norvegicus GN=Idh3B PE=1 SV=1	RAT	6	1.126441956	0.253595501
155	6.96	13.69	89.41000104	48.53000045	38.53000104	Guanine nucleotide-binding protein G(I)/G(S)/G(T) subunit beta-2 OS=Rattus norvegicus GN=Gnb2 PE=1 SV=4	RAT	10	0.834154904	0.345095694
156	6.88	19.36	75.85999966	18.82999986	16.32999927	Plasma membrane calcium-transporting ATPase 2 OS=Rattus norvegicus GN=Atp2b2 PE=2 SV=2	RAT	13	1.144873977	0.654525816
157	6.79	6.79	63.87000084	29.5599997	26.28000081	Cytochrome b-c1 complex subunit Rieske, mitochondrial OS=Rattus norvegicus GN=Uqcrfs1 PE=1 SV=2	RAT	4	1.008012056	0.928483725
158	6.79	6.79	82.31999874	45.44999897	45.44999897	Peroxiredoxin-2 OS=Rattus norvegicus GN=Prdx2 PE=1 SV=3	RAT	6	1.01602602	0.861922324
159	6.76	6.76	70.70999742	19.19	19.19	Beta-soluble NSF attachment protein OS=Rattus norvegicus GN=Napb PE=1 SV=1	RAT	4	0.992136121	0.956676185
160	6.71	11.55	72.14000225	61.43000126	53.57000232	Alpha-synuclein OS=Rattus norvegicus GN=Snca PE=1 SV=1	RAT	6	1.061882019	0.451496214
161	6.7	6.71	100	42.3999995	34.40000117	Histone H2B type 1 OS=Rattus norvegicus PE=1 SV=2	RAT	4	1.108399034	0.358619094
161	0.01	5.95	87.40000129	22.05000073	22.05000073	Histone H2B type 1-A OS=Rattus norvegicus GN=Hist1h2ba PE=1 SV=2	RAT	3		
162	6.64	6.88	60.17000079	4.909000173	2.78299991	Myosin-10 OS=Rattus norvegicus GN=Myh10 PE=1 SV=1	RAT	5	1.006307006	0.919487119
163	6.64	6.72	55.46000004	30.32999933	15.29999971	Isocitrate dehydrogenase [NAD] subunit alpha, mitochondrial OS=Rattus norvegicus GN=Idh3a PE=1 SV=1	RAT	4	0.946754515	0.26552999
164	6.6	6.6	95.50999999	58.42999816	58.42999816	Cytochrome b-c1 complex subunit 6, mitochondrial OS=Rattus norvegicus GN=Uqcrh PE=2 SV=1	RAT	4	1.021680951	0.866239488
165	6.59	6.71	50.33000112	7.479999959	6.667000055	Cullin-associated NEDD8-dissociated protein 1 OS=Rattus norvegicus GN=Cand1 PE=1 SV=1	RAT	5	0.913038611	0.497532308
166	6.58	6.62	84.21999812	19.52999979	18.15000027	Fumarate hydratase, mitochondrial OS=Rattus norvegicus GN=Fh PE=1 SV=1	RAT	9	1.036775947	0.725514174
167	6.56	6.56	46.30999863	30.86999953	30.86999953	Cell cycle exit and neuronal differentiation protein 1 OS=Rattus norvegicus GN=Cend1 PE=1 SV=1	RAT	3	1.048835039	0.656253219
168	6.39	6.41	71.06000185	18.90999973	16.32999927	Glycerol-3-phosphate dehydrogenase [NAD+], cytoplasmic OS=Rattus norvegicus GN=Gpd1 PE=1 SV=4	RAT	3	1.092311025	0.66452682
169	6.38	6.39	84.82999802	20.50999999	13.4800002	Phosphate carrier protein, mitochondrial OS=Rattus norvegicus GN=Slc25a3 PE=1 SV=1	RAT	3	0.908245087	0.158646494
170	6.2	6.22	80.76999784	30.12999892	24.3599996	Ubiquitin-40S ribosomal protein S27a OS=Rattus norvegicus GN=Rps27a PE=1 SV=2	RAT	3	0.904257774	0.170371801
170	0	6.2	80.4700017	36.71999872	29.69000041	Ubiquitin-60S ribosomal protein L40 OS=Rattus norvegicus GN=Uba52 PE=1 SV=2	RAT	3		
170	0	6.2	90.99000096	58.02000165	46.90999985	Polyubiquitin-C OS=Rattus norvegicus GN=Ubc PE=1 SV=1	RAT	3		
170	0	6.2	91.14999771	61.64000034	49.84000027	Polyubiquitin-B OS=Rattus norvegicus GN=Ubb PE=1 SV=1	RAT	3		
171	6.1	6.18	53.17000151	2.675000019	2.394999936	Fatty acid synthase OS=Rattus norvegicus GN=Fasn PE=1 SV=3	RAT	4	0.833933115	0.280829191
172	6.1	6.14	67.80999899	13.97999972	10.02999991	Heterogeneous nuclear ribonucleoprotein A3 OS=Rattus norvegicus GN=Hnrnpa3 PE=1 SV=1	RAT	3	0.981502414	0.807603896
173	6.07	6.18	55.41999936	5.033000186	4.157999903	Myosin-Va OS=Rattus norvegicus GN=Myo5a PE=2 SV=1	RAT	4	1.060596943	0.550863802
174	6.05	6.05	44.78000104	7.710999995	7.710999995	Endoplasmin OS=Rattus norvegicus GN=Hsp90b1 PE=1 SV=2	RAT	3	1.264870048	0.460123897
175	6.04	6.04	89.85000253	20.29999942	20.29999942	ES1 protein homolog, mitochondrial OS=Rattus norvegicus PE=1 SV=2	RAT	3	1.046843052	0.669381022
176	6.02	6.07	87.66999841	17.81000048	17.35000014	Histone H1.2 OS=Rattus norvegicus GN=Hist1h1c PE=1 SV=3	RAT	4	1.041007996	0.612178624
177	6.01	6.01	69.34999824	29.15000021	19.59999949	Transgelin-3 OS=Rattus norvegicus GN=Tagln3 PE=1 SV=2	RAT	3	0.901843727	0.255604506
178	5.97	6.26	44.72000003	8.750999719	7.257000357	AP-2 complex subunit beta OS=Rattus norvegicus GN=Ap2b1 PE=1 SV=1	RAT	4	0.977003872	0.700535476
179	5.96	5.96	82.76000023	35.3399992	35.3399992	Vesicle-associated membrane protein 2 OS=Rattus norvegicus GN=Vamp2 PE=1 SV=2	RAT	4	1.072641969	0.717414498
180	5.93	5.96	80.80000281	33.59999955	33.59999955	4-aminobutyrate aminotransferase, mitochondrial OS=Rattus norvegicus GN=Abat PE=1 SV=3	RAT	11	0.941295803	0.626797378
181	5.79	5.96	64.01000023	20.83999962	12.98999935	LETM1 and EF-hand domain-containing protein 1, mitochondrial OS=Rattus norvegicus GN=Letm1 PE=1 SV=1	RAT	4	1.14072597	0.124683402
182	5.71	5.71	64.63000178	16.32999927	14.74000067	Protein kinase C and casein kinase substrate in neurons protein 1 OS=Rattus norvegicus GN=Pacsin1 PE=1 SV=1	RAT	5	0.930725813	0.215852097
183	5.64	5.68	73.46000075	14.22999948	12.30999976	Succinyl-CoA:3-ketoacid-coenzyme A transferase 1, mitochondrial OS=Rattus norvegicus GN=Oxct1 PE=1 SV=1	RAT	3	1.058370948	0.574860871
184	5.63	5.63	77.60999799	41.04000032	41.04000032	Complexin-1 OS=Rattus norvegicus GN=Cplx1 PE=1 SV=1	RAT	5	0.957011878	0.733750284
185	5.57	5.57	56.33999705	5.457000062	3.170999885	Tenascin-R OS=Rattus norvegicus GN=Tnr PE=1 SV=1	RAT	4	0.969411612	0.756978571
186	5.53	8.34	47.20000029	15.03999978	10.86999997	Alpha-actinin-4 OS=Rattus norvegicus GN=Actn4 PE=1 SV=2	RAT	6	0.947315991	0.681340516
188	5.48	5.5	70.38999796	41.44999981	28.94999981	Nucleoside diphosphate kinase B OS=Rattus norvegicus GN=Nme2 PE=1 SV=1	RAT	3	0.928894818	0.33739239
189	5.37	14.79	87.40000129	44.31000054	36.59000099	14-3-3 protein beta/alpha OS=Rattus norvegicus GN=Ywhab PE=1 SV=3	RAT	11	0.840298593	0.36220029
190	5.18	5.18	50.67999959	9.353999794	5.612000078	Myc box-dependent-interacting protein 1 OS=Rattus norvegicus GN=Bin1 PE=1 SV=1	RAT	3	1.019240022	0.817111015
191	5.15	5.19	58.96000266	9.453000128	9.453000128	Dynactin subunit 2 OS=Rattus norvegicus GN=Dctn2 PE=1 SV=1	RAT	3	0.988573015	0.954268098
192	5.13	5.13	77.13999748	56.19000196	49.52000082	Cytochrome c, somatic OS=Rattus norvegicus GN=Cycs PE=1 SV=2	RAT	6	1.092813015	0.329079688
193	5.09	17.21	96.31000161	44.63	39.9300009	ADP/ATP translocase 2 OS=Rattus norvegicus GN=Slc25a5 PE=1 SV=3	RAT	13	0.996994674	0.973412812
194	5.05	5.11	27.4300009	5.683000013	4.58999984	Clathrin coat assembly protein AP180 OS=Rattus norvegicus GN=Snap91 PE=1 SV=1	RAT	3	0.959420204	0.701181471
195	5.02	5.02	60.04999876	17.04999954	5.344000086	Dual specificity mitogen-activated protein kinase kinase 1 OS=Rattus norvegicus GN=Map2k1 PE=1 SV=2	RAT	2	0.955864787	0.450337887
196	4.91	4.91	68.90000105	14.68999982	13.60999942	NADH dehydrogenase [ubiquinone] iron-sulfur protein 2, mitochondrial OS=Rattus norvegicus GN=Ndufs2 PE=1 SV=1	RAT	4	1.048310995	0.659412086
197	4.86	4.86	59.61999893	23.08000028	19.23000067	Carbonic anhydrase 2 OS=Rattus norvegicus GN=Ca2 PE=1 SV=2	RAT	3	1.121749997	0.341807187
198	4.72	4.72	75	27.9399991	27.9399991	Rho GDP-dissociation inhibitor 1 OS=Rattus norvegicus GN=Arhgdia PE=1 SV=1	RAT	3	0.93847239	0.560101271
199	4.49	5.38	82.84000158	55.22000194	55.22000194	Complexin-2 OS=Rattus norvegicus GN=Cplx2 PE=1 SV=1	RAT	5	1.024479985	0.893017471
200	4.47	4.47	44.65999901	5.124000087	3.807000071	Synaptic vesicle glycoprotein 2B OS=Rattus norvegicus GN=Sv2b PE=1 SV=1	RAT	3	0.909750521	0.334642798
201	4.43	4.43	72.5399971	46.63000107	16.06000066	Transforming protein RhoA OS=Rattus norvegicus GN=Rhoa PE=1 SV=1	RAT	2	1.022359014	0.797146678
202	4.4	4.4	51.71999931	17.15999991	14.86999989	Elongation factor 1-gamma OS=Rattus norvegicus GN=Eef1g PE=1 SV=3	RAT	3	1.024867058	0.751235127
203	4.38	4.38	34.5299989	9.446000308	9.446000308	Synaptophysin OS=Rattus norvegicus GN=Syp PE=1 SV=1	RAT	3	0.962367415	0.69528681
204	4.33	4.34	44.94999945	12.12000027	9.342999756	Obg-like ATPase 1 OS=Rattus norvegicus GN=Ola1 PE=2 SV=1	RAT	2	0.998966813	0.993365586
206	4.28	4.28	76.09000206	18.4799999	14.12999928	Lactoylglutathione lyase OS=Rattus norvegicus GN=Glo1 PE=1 SV=3	RAT	2	1.175825	0.140605599
207	4.28	4.28	46.66999876	32.37999976	20.94999999	Thioredoxin OS=Rattus norvegicus GN=Txn PE=1 SV=2	RAT	2	1.036138058	0.694893181
208	4.21	4.21	55.9499979	9.911999851	9.911999851	Guanine deaminase OS=Rattus norvegicus GN=Gda PE=1 SV=1	RAT	3	1.084445953	0.43496111
209	4.17	4.17	59.53999758	13.98999989	8.141999692	Neuroplastin OS=Rattus norvegicus GN=Nptn PE=1 SV=2	RAT	4	0.991833985	0.921441913
210	4.14	8.66	77.20000148	9.976000339	7.362999767	Glycogen phosphorylase, muscle form OS=Rattus norvegicus GN=Pygm PE=2 SV=5	RAT	8	1.026734948	0.74193579
212	4.12	4.12	62.99999952	11.9099997	8.323000371	Catenin beta-1 OS=Rattus norvegicus GN=Ctnnb1 PE=1 SV=1	RAT	3	1.004580975	0.961713493
213	4.11	4.17	91.29999876	12.37000003	12.37000003	Prohibitin-2 OS=Rattus norvegicus GN=Phb2 PE=1 SV=1	RAT	2	0.921200693	0.560472488
214	4.1	4.1	62.18000054	10.58000028	10.58000028	Pyridoxal kinase OS=Rattus norvegicus GN=Pdxk PE=1 SV=1	RAT	2	1.247002006	0.339444786
215	4.1	4.1	43.70999932	19.9300006	19.9300006	F-actin-capping protein subunit alpha-2 OS=Rattus norvegicus GN=Capza2 PE=1 SV=1	RAT	3	1.171007037	0.373241007
216	4.09	4.12	57.66999722	5.96299991	2.638999932	2-oxoglutarate dehydrogenase, mitochondrial OS=Rattus norvegicus GN=Ogdh PE=1 SV=1	RAT	2	0.942153573	0.421735287
217	4.09	4.11	67.93000102	9.309999645	9.309999645	Enoyl-CoA hydratase, mitochondrial OS=Rattus norvegicus GN=Echs1 PE=1 SV=1	RAT	2	1.087967992	0.561508119
218	4.08	4.17	46.63000107	2.549000084	2.384999953	1-phosphatidylinositol-4,5-bisphosphate phosphodiesterase beta-1 OS=Rattus norvegicus GN=Plcb1 PE=1 SV=1	RAT	2	0.95885998	0.625778615
219	4.07	4.07	54.43000197	23.24000001	23.24000001	Serine/threonine-protein phosphatase PP1-beta catalytic subunit OS=Rattus norvegicus GN=Ppp1cb PE=1 SV=3	RAT	4	1.035122037	
220	4.04	19.42	71.46000266	13.19999993	12.16000021	Plasma membrane calcium-transporting ATPase 3 OS=Rattus norvegicus GN=Atp2b3 PE=2 SV=2	RAT	10	0.896374822	0.600936115
221	4.01	15.89	72.61000276	35.2699995	31.13999963	Calcium/calmodulin-dependent protein kinase type II subunit delta OS=Rattus norvegicus GN=Camk2d PE=1 SV=1	RAT	12	1.294273019	
223	4	5.32	79.61000204	16.94999933	11.59000024	Vimentin OS=Rattus norvegicus GN=Vim PE=1 SV=2	RAT	5	1.109164953	0.553018093
224	4	4.02	72.93000221	9.235999733	9.235999733	Mitochondrial 2-oxoglutarate/malate carrier protein OS=Rattus norvegicus GN=Slc25a11 PE=2 SV=3	RAT	2	0.984135628	0.897974372
225	3.98	3.98	75.18000007	21.80999964	19.67999935	Dihydropyrimidinase-related protein 5 OS=Rattus norvegicus GN=Dpysl5 PE=1 SV=1	RAT	5	0.960982084	0.639836371

226	3.92	3.92	67.58999825	25	25	Peptidyl-prolyl cis-trans isomerase FKBP1A OS=Rattus norvegicus GN=Fkbp1a PE=2 SV=3	RAT	2	1.021070957	0.867746413
227	3.79	3.82	59.50999856	21.24000043	17.03999937	Elongation factor Tu, mitochondrial OS=Rattus norvegicus GN=Tufm PE=1 SV=1	RAT	6	0.952970386	0.446734786
228	3.72	3.72	45.3700006	37.04000115	28.2400012	GTP-binding nuclear protein Ran OS=Rattus norvegicus GN=Ran PE=1 SV=3	RAT	2	0.95953387	0.662155271
229	3.71	7.72	62.15000153	31.63999915	10.72999984	Guanine nucleotide-binding protein G(i) subunit alpha-1 OS=Rattus norvegicus GN=Gnai1 PE=1 SV=3	RAT	3	0.911483526	0.306370497
229	0	6.21	74.29000139	18.93000007	10.72999984	Guanine nucleotide-binding protein G(k) subunit alpha OS=Rattus norvegicus GN=Gnai3 PE=1 SV=3	RAT	3		
230	3.67	3.7	41.11000001	2.096999995	1.398000028	Synaptojanin-1 OS=Rattus norvegicus GN=Synj1 PE=1 SV=3	RAT	2	1.040388942	0.573639691
231	3.64	3.64	82.71999955	43.2099998	19.75000054	NEDD8 OS=Rattus norvegicus GN=Nedd8 PE=1 SV=1	RAT	3	1.149191022	0.348421007
232	3.61	3.61	81.37000203	57.13999867	37.88999915	ATP synthase subunit d, mitochondrial OS=Rattus norvegicus GN=Atp5h PE=1 SV=3	RAT	3	1.165706992	0.734207988
233	3.58	3.58	50.85999966	5.160000175	5.160000175	Cathepsin D OS=Rattus norvegicus GN=Ctsd PE=1 SV=1	RAT	2	1.049746037	0.798707783
234	3.56	3.56	59.54999924	46.07000053	32.58000016	Dynein light chain 1, cytoplasmic OS=Rattus norvegicus GN=Dynll1 PE=1 SV=1	RAT	2	1.028480053	0.904224575
235	3.54	3.66	64.17999864	7.456000149	6.870999932	Rabphilin-3A OS=Rattus norvegicus GN=Rph3a PE=1 SV=1	RAT	3	1.101508021	0.537144482
236	3.5	3.5	80.11999726	22.98000008	13.0400002	Thy-1 membrane glycoprotein OS=Rattus norvegicus GN=Thy1 PE=1 SV=1	RAT	2	1.028115034	
237	3.46	3.48	67.72999763	19.32999939	9.042999893	Dihydropyrimidinase-related protein 4 (Fragment) OS=Rattus norvegicus GN=Dpysl4 PE=1 SV=1	RAT	3	1.049823046	0.652976513
238	3.43	3.48	48.96000028	21.87999934	9.89599973	Ras-related C3 botulinum toxin substrate 1 OS=Rattus norvegicus GN=Rac1 PE=1 SV=1	RAT	2	1.013859987	0.860190272
239	3.38	3.5	34.41999853	7.599999756	7.599999756	Sodium/calcium exchanger 2 OS=Rattus norvegicus GN=Slc8a2 PE=2 SV=1	RAT	4	1.003944993	0.959549785
240	3.38	3.49	42.28999913	2.411000058	1.279999968	Plectin OS=Rattus norvegicus GN=Plec PE=1 SV=2	RAT	3	1.023712993	0.502962112
241	3.35	3.48	89.21999931	37.25000024	25.49000084	10 kDa heat shock protein, mitochondrial OS=Rattus norvegicus GN=Hspe1 PE=1 SV=3	RAT	2	1.058614969	0.439112812
242	0.86	1.13	27.36999989	7.105000317	2.895000018	Mitogen-activated protein kinase 3 OS=Rattus norvegicus GN=Mapk3 PE=1 SV=5	RAT	1	1.173674941	0.363102108
242	3.35	3.38	30.45000136	12.84999996	8.380000293	Mitogen-activated protein kinase 1 OS=Rattus norvegicus GN=Mapk1 PE=1 SV=3	RAT	2	0.97287941	0.830055773
243	3.23	3.23	73.90999794	21.73999995	13.59000057	Ras-related protein Rap-1A OS=Rattus norvegicus GN=Rap1a PE=1 SV=1	RAT	2	0.980221093	0.836919725
243	0	2.91	53.79999876	13.59000057	13.59000057	Ras-related protein Rap-1b OS=Rattus norvegicus GN=Rap1b PE=2 SV=2	RAT	2		
244	3.22	3.24	51.77000165	10.62000021	9.291999787	Isocitrate dehydrogenase [NADP], mitochondrial OS=Rattus norvegicus GN=Idh2 PE=1 SV=2	RAT	3	1.09531796	0.386983395
245	3.18	3.26	61.3499999	4.687999934	2.604000084	Dynamin-like 120 kDa protein, mitochondrial OS=Rattus norvegicus GN=Opa1 PE=1 SV=1	RAT	3	0.980255485	0.770216405
246	3.17	3.17	89.99999762	18.56999993	18.56999993	Profilin-1 OS=Rattus norvegicus GN=Pfn1 PE=1 SV=2	RAT	3	1.066666007	0.713714182
247	3.1	3.12	91.54000282	23.8499999	21.53999954	Histone H2A type 1 OS=Rattus norvegicus PE=1 SV=2	RAT	2	1.034787059	0.685199678
247	0.01	3.06	93.01999807	24.02999997	21.70999944	Histone H2A.J OS=Rattus norvegicus GN=H2afj PE=2 SV=1	RAT	2		
247	0.01	3.02	98.46000075	34.61999893	21.53999954	Histone H2A type 1-F OS=Rattus norvegicus PE=3 SV=3	RAT	2		
247	0	3.12	91.54000282	23.8499999	21.53999954	Histone H2A type 2-A OS=Rattus norvegicus GN=Hist2h2aa3 PE=1 SV=1	RAT	2		
247	0	3.04	91.54000282	23.8499999	21.53999954	Histone H2A type 4 OS=Rattus norvegicus PE=2 SV=2	RAT	2		
247	0	3.03	91.54000282	23.8499999	21.53999954	Histone H2A type 1-E OS=Rattus norvegicus PE=1 SV=2	RAT	2		
247	0	3.03	91.54000282	23.8499999	21.53999954	Histone H2A type 1-C OS=Rattus norvegicus PE=1 SV=2	RAT	2		
247	0	3	91.54000282	23.8499999	21.53999954	Histone H2A type 3 OS=Rattus norvegicus PE=2 SV=3	RAT	2		
248	3.06	5.18	46.00000083	17.00000018	17.00000018	Ras-related protein Rab-10 OS=Rattus norvegicus GN=Rab10 PE=2 SV=1	RAT	4	0.857053578	
248	0.01	3.65	86.47000194	14.01000023	10.62999964	Ras-related protein Rab-8A OS=Rattus norvegicus GN=Rab8a PE=2 SV=2	RAT	2		
248	0.01	3.64	53.61999869	14.01000023	10.62999964	Ras-related protein Rab-8B OS=Rattus norvegicus GN=Rab8b PE=1 SV=1	RAT	2		
248	0.01	1.42	71.42999768	5.418999866	5.418999866	Ras-related protein Rab-13 OS=Rattus norvegicus GN=Rab13 PE=2 SV=2	RAT	1		
248	0	3.66	54.72999811	14.42999989	10.94999984	Ras-related protein Rab-1B OS=Rattus norvegicus GN=Rab1b PE=1 SV=1	RAT	2		
248	0	3.53	61.69000268	10.94999984	10.94999984	Ras-related protein Rab-35 OS=Rattus norvegicus GN=Rab35 PE=2 SV=1	RAT	2		
248	0	3.47	44.33999956	10.37999988	10.37999988	Ras-related protein Rab-15 OS=Rattus norvegicus GN=Rab15 PE=2 SV=1	RAT	2		
249	3.06	3.06	38.46000135	16.92000031	16.92000031	Astrocytic phosphoprotein PEA-15 OS=Rattus norvegicus GN=Pea15 PE=1 SV=1	RAT	3	0.99555558	0.971652985
250	2.96	2.96	39.82999921	5.085000023	5.085000023	Hyaluronan and proteoglycan link protein 1 OS=Rattus norvegicus GN=Hapln1 PE=1 SV=2	RAT	2	1.082373023	0.57747829
251	2.95	3.22	84.5600009	46.97999954	18.11999977	Stathmin OS=Rattus norvegicus GN=Stmn1 PE=1 SV=2	RAT	2	1.047209978	0.566674471
252	2.93	3.04	54.60000038	9.194000065	5.232999846	Drebrin OS=Rattus norvegicus GN=Dbn1 PE=2 SV=3	RAT	2	1.014016032	0.912038624
253	2.93	2.93	45.84999979	18.05000007	18.05000007	Protein phosphatase 1 regulatory subunit 1B OS=Rattus norvegicus GN=Ppp1r1b PE=2 SV=1	RAT	2	0.941882312	0.720453322
254	2.89	2.95	89.70999718	35.28999984	28.67999971	Histone H3.3 OS=Rattus norvegicus GN=H3f3b PE=1 SV=2	RAT	4	0.901871681	0.316878587
255	2.89	2.89	73.65000248	9.934999794	9.934999794	Heterogeneous nuclear ribonucleoprotein K OS=Rattus norvegicus GN=Hnrnpk PE=1 SV=1	RAT	3	1.089439034	0.85674578
256	2.89	2.89	83.13999772	7.987999916	7.987999916	Limbic system-associated membrane protein OS=Rattus norvegicus GN=Lsamp PE=1 SV=1	RAT	2	1.015063047	0.905664623
257	2.87	2.98	48.67999852	7.301999629	6.491000205	Solute carrier family 2, facilitated glucose transporter member 3 OS=Rattus norvegicus GN=Slc2a3 PE=1 SV=1	RAT	2	1.143856049	0.490378499
258	2.85	2.85	84.95000005	13.1099999	13.1099999	Ras-related protein Ral-A OS=Rattus norvegicus GN=Rala PE=1 SV=1	RAT	2	1.052567005	0.698156178
259	2.8	2.86	68.48000288	29.69999909	18.17999929	Destrin OS=Rattus norvegicus GN=Dstn PE=1 SV=3	RAT	2	1.033406973	0.749839723
260	2.78	2.78	76.88000202	29.15000021	9.044999629	Peroxiredoxin-1 OS=Rattus norvegicus GN=Prdx1 PE=1 SV=1	RAT	2	1.028676033	0.837528884
261	2.77	2.88	38.82000148	3.985999897	3.293000162	Moesin OS=Rattus norvegicus GN=Msn PE=1 SV=3	RAT	2	1.044986963	0.75101459
261	0	2.78	20.47999948	3.24199982	3.24199982	Ezrin OS=Rattus norvegicus GN=Ezr PE=1 SV=3	RAT	2		
262	2.73	2.75	65.31999707	6.650999933	6.650999933	60S ribosomal protein L4 OS=Rattus norvegicus GN=Rpl4 PE=1 SV=3	RAT	2	1.025149941	0.793262899
263	2.71	2.74	60.07000208	3.957000002	3.957000002	T-complex protein 1 subunit alpha OS=Rattus norvegicus GN=Tcp1 PE=1 SV=1	RAT	2	1.058634043	0.667459488
264	2.71	2.71	25.27999878	4.473999888	4.473999888	Serine/threonine-protein phosphatase 2A 55 kDa regulatory subunit B alpha isoform OS=Rattus norvegicus GN=Ppp2r2a PE=2 SV=1	RAT	2	0.980889976	0.807023227
264	0	1.72	30.23999929	2.428000048	2.428000048	Serine/threonine-protein phosphatase 2A 55 kDa regulatory subunit B delta isoform OS=Rattus norvegicus GN=Ppp2r2d PE=2 SV=1	RAT	1		
265	2.68	2.79	32.8700006	16.32000059	10.33999994	Cell adhesion molecule 2 OS=Rattus norvegicus GN=Cadm2 PE=1 SV=2	RAT	3	0.961893201	0.698879719
266	2.67	2.67	61.37999892	2.89699994	1.516999956	Beta-adducin OS=Rattus norvegicus GN=Add2 PE=2 SV=4	RAT	1	0.933998525	0.638910592
267	2.63	2.68	43.99999976	1.190999988	0.714900019	Neural cell adhesion molecule L1 OS=Rattus norvegicus GN=L1cam PE=2 SV=3	RAT	1	1.038581014	0.722756684
268	2.59	2.61	70.23000121	8.37199986	8.37199986	C-terminal-binding protein 1 OS=Rattus norvegicus GN=Ctbp1 PE=1 SV=3	RAT	3	0.997116387	0.981596172
269	2.57	2.57	35.85000038	9.747999907	9.747999907	Ribose-phosphate pyrophosphokinase 1 OS=Rattus norvegicus GN=Prps1 PE=1 SV=2	RAT	2	0.938400686	0.638300002
270	2.51	2.53	30.66000044	10.90999991	4.173000157	Transketolase OS=Rattus norvegicus GN=Tkt PE=1 SV=1	RAT	1	0.971158087	0.713059604
271	2.5	2.5	51.56000257	5.778000131	5.778000131	Protein tweety homolog 1 OS=Rattus norvegicus GN=Ttyh1 PE=2 SV=1	RAT	2	1.028059006	0.901369989
272	2.5	2.5	62.18000054	8.807999641	5.181000009	Neuron-specific calcium-binding protein hippocalcin OS=Rattus norvegicus GN=Hpca PE=1 SV=2	RAT	1	0.819296479	0.153558597
273	2.45	2.45	58.57999921	23.08000028	10.64999998	Cytochrome c oxidase subunit 4 isoform 1, mitochondrial OS=Rattus norvegicus GN=Cox4i1 PE=1 SV=1	RAT	2	1.059237957	0.476375401
274	2.42	3.47	60.72999835	36.12999916	30.37	Cell division control protein 42 homolog OS=Rattus norvegicus GN=Cdc42 PE=1 SV=2	RAT	3	0.990097702	0.943889499
275	2.38	2.38	54.6299994	16.30000025	4.405000061	Cytochrome c oxidase subunit 2 OS=Rattus norvegicus GN=Mtco2 PE=2 SV=2	RAT	1	0.903734982	0.594401121
276	2.34	2.37	57.35999942	11.28999963	2.377000079	Annexin A6 OS=Rattus norvegicus GN=Anxa6 PE=1 SV=2	RAT	1	0.974326611	0.709865212
277	2.33	2.33	34.74000096	4.008999839	2.226999961	Heterogeneous nuclear ribonucleoprotein H2 OS=Rattus norvegicus GN=Hnrnph2 PE=1 SV=1	RAT	1	0.964648783	0.861110508
277	0	2	26.98999941	2.410000004	2.410000004	Heterogeneous nuclear ribonucleoprotein F OS=Rattus norvegicus GN=Hnrnpf PE=1 SV=3	RAT	1		
278	2.31	2.31	71.03000283	14.94999975	7.477000356	ATPase inhibitor, mitochondrial OS=Rattus norvegicus GN=Atpif1 PE=2 SV=2	RAT	1	1.115733027	0.365127295
279	2.25	2.25	67.96000004	8.414000273	6.148999929	Myristoylated alanine-rich C-kinase substrate OS=Rattus norvegicus GN=Marcks PE=1 SV=2	RAT	1	0.90410012	0.495862991
280	2.24	7.49	81.27999902	15.60000032	10.30000001	Heat shock 70 kDa protein 1A/1B OS=Rattus norvegicus GN=Hspa1a PE=2 SV=2	RAT	5	1.309183955	0.639838874

280	0	9.27	60.53000093	13.42000067	9.047999978	Heat shock 70 kDa protein 1-like OS=Rattus norvegicus GN=Hspa1l PE=2 SV=2	RAT	6		
282	2.2	2.23	86.04999781	22.48000056	10.08000001	Cytochrome c oxidase subunit 5B, mitochondrial OS=Rattus norvegicus GN=Cox5b PE=1 SV=2	RAT	1	1.00257194	0.973194897
283	2.19	2.24	70.28999925	7.427000254	3.448000178	NADH dehydrogenase [ubiquinone] 1 alpha subcomplex subunit 9, mitochondrial OS=Rattus norvegicus GN=Ndufa9 PE=1 SV=2	RAT	1	0.86718148	0.422454804
284	2.16	2.22	74.91999865	1.006999984	1.006999984	Liprin-alpha-3 OS=Rattus norvegicus GN=Ppfia3 PE=1 SV=2	RAT	1	0.991488218	0.904520392
285	2.14	9.19	91.01999998	37.54999936	29.80000079	14-3-3 protein theta OS=Rattus norvegicus GN=Ywhaq PE=1 SV=1	RAT	7	0.922110498	0.578083873
286	2.14	2.15	89.63999748	41.8900013	41.8900013	Superoxide dismutase [Mn], mitochondrial OS=Rattus norvegicus GN=Sod2 PE=1 SV=2	RAT	4	0.838653326	0.325825393
287	2.14	2.14	54.83999848	11.4699997	7.88500011	Complement component 1 Q subcomponent-binding protein, mitochondrial OS=Rattus norvegicus GN=C1qbp PE=1 SV=2	RAT	2	0.83732599	0.327536106
288	2.12	2.12	71.03000283	4.673000053	4.673000053	Sideroflexin-3 OS=Rattus norvegicus GN=Sfxn3 PE=2 SV=1	RAT	1	1.063361049	0.693144619
289	2.1	2.1	92.47000217	15.07000029	6.848999858	40S ribosomal protein S16 OS=Rattus norvegicus GN=Rps16 PE=1 SV=2	RAT	1	1.063992023	0.646135688
290	2.09	31.8	93.76000166	64.59000111	51.67000294	Tubulin alpha-8 chain OS=Rattus norvegicus GN=Tuba8 PE=2 SV=1	RAT	55	0.982314825	
290	0	45.22	91.78000093	77.10999846	73.78000021	Tubulin alpha-3 chain OS=Rattus norvegicus GN=Tuba3a PE=2 SV=1	RAT	120		
291	2.09	2.09	64.85999823	6.142999977	3.686000034	Eukaryotic initiation factor 4A-II OS=Rattus norvegicus GN=Eif4a2 PE=1 SV=1	RAT	1	0.960995972	0.854707301
292	2.08	2.14	28.49000096	8.139999956	8.139999956	Translationally-controlled tumor protein OS=Rattus norvegicus GN=Tpt1 PE=1 SV=1	RAT	1	1.036946058	0.780570686
293	2.08	2.1	70.4400003	3.201999888	3.201999888	26S protease regulatory subunit 8 OS=Rattus norvegicus GN=Psmc5 PE=1 SV=1	RAT	1	0.927096486	0.456633896
294	2.08	2.08	46.84000015	2.105000056	2.105000056	DNA damage-binding protein 1 OS=Rattus norvegicus GN=Ddb1 PE=2 SV=1	RAT	1	0.955619812	
295	2.06	3	93.4400022	20.30999959	15.62999934	Heterogeneous nuclear ribonucleoprotein A1 OS=Rattus norvegicus GN=Hnrnpa1 PE=1 SV=3	RAT	3		
296	2.06	2.09	64.67000246	3.988999873	3.988999873	Tropomodulin-2 OS=Rattus norvegicus GN=Tmod2 PE=1 SV=1	RAT	1	1.012037992	0.925675094
297	2.06	2.06	79.11000252	3.39399986	3.39399986	Paralemmin-1 OS=Rattus norvegicus GN=Palm PE=1 SV=1	RAT	1	1.078544021	0.596702874
298	2.06	2.06	98.6199975	6.207000092	6.207000092	40S ribosomal protein S19 OS=Rattus norvegicus GN=Rps19 PE=2 SV=3	RAT	1	1.004731059	0.992006421
299	2.05	2.08	72.00999856	2.865000069	2.865000069	Gephyrin OS=Rattus norvegicus GN=Gphn PE=1 SV=3	RAT	1	0.978641093	0.847767115
300	2.05	2.06	67.91999936	5.895999819	5.895999819	Acetyl-CoA acetyltransferase, mitochondrial OS=Rattus norvegicus GN=Acat1 PE=1 SV=1	RAT	2	0.948978186	0.689857781
301	2.05	2.05	73.89000058	11.06000021	11.06000021	CD9 antigen OS=Rattus norvegicus GN=Cd9 PE=1 SV=2	RAT	1	0.968194127	0.801858187
302	2.04	46.85	86.71000004	75.90000033	71.61999941	Tubulin beta-5 chain OS=Rattus norvegicus GN=Tubb5 PE=1 SV=1	RAT	94	1.115640998	0.496414006
303	2.04	2.07	74.47999716	1.04700001	1.04700001	Membrane-associated phosphatidylinositol transfer protein 1 OS=Rattus norvegicus GN=Pitpnm1 PE=2 SV=1	RAT	1	0.965748787	0.726619422
304	2.04	2.06	59.4900012	1.982999966	1.982999966	Heat shock protein 75 kDa, mitochondrial OS=Rattus norvegicus GN=Trap1 PE=1 SV=1	RAT	1		
305	2.04	2.04	58.09999704	7.619000226	7.619000226	Glutathione S-transferase P OS=Rattus norvegicus GN=Gstp1 PE=1 SV=2	RAT	1	0.951722085	0.710650802
306	2.03	4.06	46.43999934	13.3100003	13.3100003	Serine/threonine-protein phosphatase PP1-gamma catalytic subunit OS=Rattus norvegicus GN=Ppp1cc PE=1 SV=1	RAT	3	1.076763034	0.772408724
306	0	4.02	44.24000084	13.03000003	13.03000003	Serine/threonine-protein phosphatase PP1-alpha catalytic subunit OS=Rattus norvegicus GN=Ppp1ca PE=1 SV=1	RAT	3		
307	2.03	2.03	67.68000126	13.23000044	8.141999692	Isocitrate dehydrogenase [NAD] subunit gamma 1, mitochondrial OS=Rattus norvegicus GN=Idh3g PE=2 SV=2	RAT	1	1.031479955	
308	2.03	2.03	89.34999704	4.563000053	4.563000053	Proteasome subunit beta type-5 OS=Rattus norvegicus GN=Psmb5 PE=1 SV=3	RAT	1	0.961628795	0.769962072
309	2.02	2.35	67.69000292	3.333000094	2.178999968	6-phosphofructokinase, liver type OS=Rattus norvegicus GN=Pfkl PE=2 SV=3	RAT	1	0.982731283	0.893775821
310	2.02	2.08	56.33999705	8.450999856	8.450999856	AP-2 complex subunit sigma OS=Rattus norvegicus GN=Ap2s1 PE=2 SV=1	RAT	2	1.059203029	0.677260816
311	2.02	2.06	47.90000021	1.532000024	1.532000024	Neurofascin OS=Rattus norvegicus GN=Nfasc PE=1 SV=2	RAT	1	0.886892021	0.528710783
312	2.02	2.02	39.48000073	5.178000033	5.178000033	Hydroxyacylglutathione hydrolase, mitochondrial OS=Rattus norvegicus GN=Hagh PE=1 SV=2	RAT	1	1.04914701	
313	2.02	2.02	87.30999827	3.846000135	3.846000135	ProSAAS OS=Rattus norvegicus GN=Pcsk1n PE=1 SV=1	RAT	1	1.00542295	0.966723919
314	2.02	2.02	55.62999845	10.59999987	10.59999987	Myosin light polypeptide 6 OS=Rattus norvegicus GN=Myl6 PE=1 SV=3	RAT	1	1.081231952	0.575244427
315	2.02	2.02	68.90000105	14.0200004	14.0200004	CB1 cannabinoid receptor-interacting protein 1 OS=Rattus norvegicus GN=Cnrip1 PE=1 SV=1	RAT	1	0.920094371	0.564416111
316	2.01	2.89	99.2200017	17.97000021	17.97000021	Histone H2A.Z OS=Rattus norvegicus GN=H2afz PE=1 SV=2	RAT	2	1.146868944	0.420876592
317	2.01	2.01	54.90000248	8.06099996	4.357000068	NADH-ubiquinone oxidoreductase chain 4 OS=Rattus norvegicus GN=Mtnd4 PE=2 SV=2	RAT	1	0.59106952	
318	2.01	2.01	72.47999907	5.045999959	5.045999959	Hypoxanthine-guanine phosphoribosyltransferase OS=Rattus norvegicus GN=Hprt1 PE=1 SV=1	RAT	1	0.933160603	
319	2.01	2.01	37.81999946	8.807999641	8.807999641	Cysteine and glycine-rich protein 1 OS=Rattus norvegicus GN=Csrp1 PE=2 SV=2	RAT	1	1.058894038	
320	2	4	55.61000109	15.81999958	15.81999958	Rho-related GTP-binding protein RhoB OS=Rattus norvegicus GN=Rhob PE=1 SV=1	RAT	2	1.091357946	
321	2	2.14	54.07999754	7.06499964	3.804000095	Endophilin-A2 OS=Rattus norvegicus GN=Sh3gl1 PE=1 SV=1	RAT	1	1.196429968	
322	2	2.1	37.54999936	3.376000002	2.531999908	Adenylyl cyclase-associated protein 1 OS=Rattus norvegicus GN=Cap1 PE=1 SV=3	RAT	1	0.919032216	
323	2	2.07	70.77000141	1.965999976	1.965999976	Trifunctional enzyme subunit alpha, mitochondrial OS=Rattus norvegicus GN=Hadha PE=1 SV=2	RAT	1	1.192371964	
324	2	2.04	46.05999887	7.570999861	7.570999861	Protein IMPACT OS=Rattus norvegicus GN=Impact PE=2 SV=1	RAT	1	1.008188963	
325	2	2.03	96.10999823	8.948999643	8.948999643	6-phosphogluconolactonase OS=Rattus norvegicus GN=Pgls PE=1 SV=1	RAT	1	1.359398961	
326	2	2.02	73.79999757	2.771000005	2.771000005	Acetyl-CoA acetyltransferase, cytosolic OS=Rattus norvegicus GN=Acat2 PE=1 SV=1	RAT	1	0.78174752	
327	2	2.02	69.96999979	6.600999832	6.600999832	Monoglyceride lipase OS=Rattus norvegicus GN=Mgll PE=1 SV=1	RAT	1	0.758483171	
328	2	2	71.39000297	7.219000161	7.219000161	Alcohol dehydrogenase class-3 OS=Rattus norvegicus GN=Adh5 PE=1 SV=2	RAT	1	0.864289105	
329	2	2	37.67000139	2.195999958	2.195999958	Bifunctional purine biosynthesis protein PURH OS=Rattus norvegicus GN=Atic PE=1 SV=2	RAT	1	1.119292974	
330	2	2	54.8600018	3.24300006	3.24300006	Peptidyl-prolyl cis-trans isomerase D OS=Rattus norvegicus GN=Ppid PE=1 SV=3	RAT	1		
331	2	2	57.48000145	5.139999837	5.139999837	60S ribosomal protein L10 OS=Rattus norvegicus GN=Rpl10 PE=1 SV=3	RAT	1	0.908524215	
332	2	2	26.53999925	3.505999967	3.505999967	Sodium- and chloride-dependent GABA transporter 1 OS=Rattus norvegicus GN=Slc6a1 PE=1 SV=1	RAT	1	1.211755991	
333	2	2	42.08000004	6.334999949	6.334999949	Fumarylacetoacetate hydrolase domain-containing protein 1 OS=Rattus norvegicus GN=Fahd1 PE=2 SV=1	RAT	1	1.045030951	
334	2	2	41.96000099	5.490000173	5.490000173	Proteasome subunit alpha type-3 OS=Rattus norvegicus GN=Psma3 PE=1 SV=3	RAT	1	0.982057571	
335	2	2	18.75	6.25	6.25	Calreticulin OS=Rattus norvegicus GN=Calr PE=1 SV=1	RAT	1	0.961023688	
336	2	2	42.17999876	3.835000098	3.835000098	Cathepsin B OS=Rattus norvegicus GN=Ctsb PE=1 SV=2	RAT	1	1.109349966	
337	2	2	51.05999708	10.63999981	10.63999981	PRA1 family protein 3 OS=Rattus norvegicus GN=Arl6ip5 PE=1 SV=1	RAT	1	0.918147624	
338	2	2	65.52000046	17.23999977	17.23999977	NADH dehydrogenase [ubiquinone] 1 alpha subcomplex subunit 5 OS=Rattus norvegicus GN=Ndufa5 PE=1 SV=3	RAT	1	0.876940608	
339	2	2	88.45999837	19.23000067	19.23000067	Neurogranin OS=Rattus norvegicus GN=Nrgn PE=1 SV=1	RAT	1	1.230332017	
340	2	2	60.14999747	10.53000018	10.53000018	Fatty acid-binding protein, heart OS=Rattus norvegicus GN=Fabp3 PE=1 SV=2	RAT	1	1.015890956	
341	2	2	27.27000117	7.791999727	7.791999727	Eukaryotic translation initiation factor 5A-1 OS=Rattus norvegicus GN=Eif5a PE=1 SV=3	RAT	2	1.064898014	
342	2	2	18.70000064	16.25999957	8.129999787	Anionic trypsin-1 OS=Rattus norvegicus GN=Prss1 PE=1 SV=1	RAT	5	0.953276873	
343	2	2	56.13999963	14.04000074	14.04000074	60S acidic ribosomal protein P1 OS=Rattus norvegicus GN=Rplp1 PE=3 SV=1	RAT	1	0.846308589	
344	2	2	40.20999968	15.45999944	15.45999944	Mitochondrial import inner membrane translocase subunit Tim8 A OS=Rattus norvegicus GN=Timm8a PE=1 SV=1	RAT	1	0.880110919	
345	2	2	33.86000097	12.6000002	12.6000002	Nuclear transport factor 2 OS=Rattus norvegicus GN=Nutf2 PE=1 SV=1	RAT	1	0.808645725	
346	1.92	1.92	55.69999814	7.594999671	7.594999671	Low molecular weight phosphotyrosine protein phosphatase OS=Rattus norvegicus GN=Acp1 PE=1 SV=3	RAT	1	1.298213005	
347	1.91	1.91	47.51999974	3.66399996	1.508999988	cGMP-dependent 3',5'-cyclic phosphodiesterase OS=Rattus norvegicus GN=Pde2a PE=1 SV=2	RAT	1	1.066117048	0.656512022
348	1.89	2.74	68.48000288	7.819999754	7.819999754	Synaptotagmin-2 OS=Rattus norvegicus GN=Syt2 PE=1 SV=1	RAT	2	1.045575023	
349	1.88	1.88	58.75999928	18.0399999	18.0399999	Adenylate kinase isoenzyme 1 OS=Rattus norvegicus GN=Ak1 PE=1 SV=3	RAT	2	1.040850043	0.762803614
350	1.86	1.86	28.88000011	13.16000074	1.768000051	Tyrosine-protein phosphatase non-receptor type substrate 1 OS=Rattus norvegicus GN=Sirpa PE=1 SV=1	RAT	1	0.931828916	0.512992918
351	1.85	1.87	59.32999849	4.306000099	4.306000099	26S protease regulatory subunit 6B OS=Rattus norvegicus GN=Psmc4 PE=1 SV=1	RAT	1	0.90816468	0.648672402
352	1.83	6.38	65.32999873	15.43000042	10.48000008	Serine/threonine-protein phosphatase 2B catalytic subunit beta isoform OS=Rattus norvegicus GN=Ppp3cb PE=2 SV=1	RAT	4	0.877852023	0.476312101

353	1.81	1.81	50.45999885	12.83999979	5.505000055	Glutathione S-transferase Mu 1 OS=Rattus norvegicus GN=Gstm1 PE=1 SV=2	RAT	1	1.151654959	0.397502691
354	1.81	1.81	26.89999938	2.923999913	2.923999913	Sideroflexin-5 OS=Rattus norvegicus GN=Sfxn5 PE=2 SV=1	RAT	1	1.137467027	
355	1.81	1.81	35.65999866	2.150999941	2.150999941	Abhydrolase domain-containing protein 16A OS=Rattus norvegicus GN=Abhd16a PE=2 SV=1	RAT	1	0.89357239	0.471651405
356	1.8	1.8	48.64000082	12.06	12.06	Thioredoxin-dependent peroxide reductase, mitochondrial OS=Rattus norvegicus GN=Prdx3 PE=1 SV=2	RAT	1	0.987304628	
357	1.79	1.79	40.29999971	4.563000053	4.563000053	40S ribosomal protein S4, X isoform OS=Rattus norvegicus GN=Rps4x PE=2 SV=2	RAT	1	0.889264286	0.48630631
358	1.77	1.77	16.89999998	6.103000045	6.103000045	REVERSED CDP-diacylglycerol--inositol 3-phosphatidyltransferase OS=Rattus norvegicus GN=Cdipt PE=1 SV=1	RAT	1	0.870278001	
359	1.75	1.78	71.18999958	32.19999969	22.87999988	D-dopachrome decarboxylase OS=Rattus norvegicus GN=Ddt PE=1 SV=3	RAT	2	1.029232979	0.88445729
360	1.74	1.79	46.47000134	1.654000022	1.654000022	Prelamin-A/C OS=Rattus norvegicus GN=Lmna PE=1 SV=1	RAT	1	1.013473034	0.909177125
361	1.74	1.74	34.31999981	0.927499961	0.927499961	Arf-GAP with GTPase, ANK repeat and PH domain-containing protein 2 OS=Rattus norvegicus GN=Agap2 PE=1 SV=1	RAT	1	0.940844476	
362	1.72	1.74	27.39000022	6.066000089	3.125	Serine/threonine-protein kinase PAK 1 OS=Rattus norvegicus GN=Pak1 PE=1 SV=3	RAT	1	1.075149059	0.431686193
363	1.7	1.72	76.35999918	5.454999954	5.454999954	40S ribosomal protein S10 OS=Rattus norvegicus GN=Rps10 PE=2 SV=1	RAT	1	1.285544038	
364	1.69	1.78	63.41000199	1.788000017	1.23800002	Glycerol-3-phosphate dehydrogenase, mitochondrial OS=Rattus norvegicus GN=Gpd2 PE=1 SV=1	RAT	1	0.903324604	0.590119779
365	1.69	1.73	30.00999987	1.204000041	1.204000041	Reticulon-4 OS=Rattus norvegicus GN=Rtn4 PE=1 SV=1	RAT	1	1.041859984	0.678289711
366	1.64	1.66	45.51999867	8.275999874	8.275999874	Synaptojanin-2-binding protein OS=Rattus norvegicus GN=Synj2bp PE=1 SV=2	RAT	1	1.040922999	
367	1.63	1.63	31.36999905	10.77999994	10.77999994	Parathymosin OS=Rattus norvegicus GN=Ptms PE=1 SV=2	RAT	1	0.958800614	
368	1.62	1.68	65.17000198	37.07999885	24.71999973	Dynein light chain 2, cytoplasmic OS=Rattus norvegicus GN=Dynll2 PE=1 SV=1	RAT	1	1.064726949	0.710020781
369	1.61	1.63	34.92000103	1.217999961	1.217999961	Rho guanine nucleotide exchange factor 2 OS=Rattus norvegicus GN=Arhgef2 PE=2 SV=1	RAT	1	0.93485719	0.347740203
370	1.6	1.6	40.34000039	12.61000037	12.61000037	40S ribosomal protein S20 OS=Rattus norvegicus GN=Rps20 PE=3 SV=1	RAT	1	0.901078999	
371	1.58	1.62	30.23000062	2.305999957	1.317999978	Neuronal cell adhesion molecule OS=Rattus norvegicus GN=Nrcam PE=1 SV=2	RAT	1	0.970654428	0.856735528
372	1.58	1.6	32.22000003	2.380999923	0.793700013	Ankyrin repeat and sterile alpha motif domain-containing protein 1B OS=Rattus norvegicus GN=Anks1b PE=1 SV=1	RAT	1	0.912233829	0.625681281
373	1.48	1.48	31.60000145	11.32000014	11.32000014	Ras-related protein Rab-2A OS=Rattus norvegicus GN=Rab2a PE=1 SV=1	RAT	2	0.963695586	0.774793088
374	1.46	1.46	44.85000074	8.357000351	8.357000351	Guanine nucleotide-binding protein G(q) subunit alpha OS=Rattus norvegicus GN=Gnaq PE=1 SV=2	RAT	2	0.959208429	0.746149421
374	0	1.35	54.8699975	3.064000048	3.064000048	Guanine nucleotide-binding protein subunit alpha-11 OS=Rattus norvegicus GN=Gna11 PE=2 SV=1	RAT	1		
375	1.44	5.42	53.68999839	10.36000028	4.642999917	Heat shock 70 kDa protein 4 OS=Rattus norvegicus GN=Hspa4 PE=1 SV=1	RAT	3	0.906899571	0.450732797
376	1.41	1.43	17.84999967	3.150000051	3.150000051	cAMP-dependent protein kinase type I-beta regulatory subunit OS=Rattus norvegicus GN=Prkar1b PE=2 SV=2	RAT	1	0.94169867	0.668160915
376	0	1.39	24.41000044	3.150000051	3.150000051	cAMP-dependent protein kinase type I-alpha regulatory subunit OS=Rattus norvegicus GN=Prkar1a PE=2 SV=2	RAT	1		
377	1.41	1.41	43.65999997	18.31	18.31	Glia maturation factor beta OS=Rattus norvegicus GN=Gmfb PE=1 SV=2	RAT	1	1.003504992	0.969895422
378	1.4	1.4	12.6000002	8.660999686	8.660999686	Brain protein 44 OS=Rattus norvegicus GN=Brp44 PE=2 SV=1	RAT	1	1.099015951	
379	1.39	1.41	52.48000026	4.255000129	4.255000129	Succinate dehydrogenase [ubiquinone] iron-sulfur subunit, mitochondrial OS=Rattus norvegicus GN=Sdhb PE=2 SV=2	RAT	1	1.040467024	0.648096502
380	1.39	1.41	16.60999954	3.80599983	3.80599983	Purine nucleoside phosphorylase OS=Rattus norvegicus GN=Pnp PE=1 SV=1	RAT	1	1.052152991	0.697689593
381	1.39	1.4	30.03999889	2.789999917	2.789999917	GRINL1A complex locus protein 1 OS=Rattus norvegicus GN=Gcom1 PE=1 SV=1	RAT	1	1.055353999	
382	1.33	1.39	38.74000013	3.156000003	3.156000003	Protein kinase C gamma type OS=Rattus norvegicus GN=Prkcg PE=1 SV=1	RAT	1	0.915307581	0.544701517
383	1.32	1.33	36.44999862	5.046999827	5.046999827	Brain-specific angiogenesis inhibitor 1-associated protein 2 OS=Rattus norvegicus GN=Baiap2 PE=1 SV=1	RAT	1	0.971682727	0.7564978
384	1.32	1.33	42.9399997	3.047000058	3.047000058	Septin-2 OS=Rattus norvegicus GN=Sept2 PE=1 SV=1	RAT	1	0.874568522	
385	1.29	1.29	24.26999956	1.248000003	1.248000003	Far upstream element-binding protein 2 OS=Rattus norvegicus GN=Khsrp PE=1 SV=1	RAT	1	1.089892983	0.543824792
386	1.28	1.52	13.86000067	6.931000203	4.949999973	Leukocyte surface antigen CD47 OS=Rattus norvegicus GN=Cd47 PE=1 SV=1	RAT	1	1.030882001	0.822162092
387	1.24	1.24	58.60000253	5.09600006	5.09600006	60S ribosomal protein L24 OS=Rattus norvegicus GN=Rpl24 PE=1 SV=1	RAT	1	1.011214018	
388	1.24	1.24	43.14999878	5.393999815	5.393999815	Dihydropteridine reductase OS=Rattus norvegicus GN=Qdpr PE=1 SV=1	RAT	1	1.016594052	
389	1.2	1.2	24.33000058	4.752999917	2.66200006	Amine oxidase [flavin-containing] A OS=Rattus norvegicus GN=Maoa PE=1 SV=1	RAT	1	1.068794012	0.733657718
390	1.17	1.17	30.23000062	5.587000027	0	Serotransferrin OS=Rattus norvegicus GN=Tf PE=1 SV=3	RAT	0	0.855274677	0.067080528
391	1.16	1.18	50.51000118	5.888999999	4.983000085	Brevican core protein OS=Rattus norvegicus GN=Bcan PE=1 SV=2	RAT	2	1.016232967	0.868008614
392	1.16	1.18	33.82000029	20.58999985	10.28999984	Guanine nucleotide-binding protein G(I)/G(S)/G(O) subunit gamma-7 OS=Rattus norvegicus GN=Gng7 PE=2 SV=2	RAT	1	0.901733816	0.488105208
393	1.15	1.28	32.4000001	5.361000076	0	Elongation factor 2 OS=Rattus norvegicus GN=Eef2 PE=1 SV=4	RAT	0	1.059679985	0.471866995
394	1.12	1.12	44.13999915	6.897000223	6.897000223	Ubiquitin-conjugating enzyme E2 variant 2 OS=Rattus norvegicus GN=Ube2v2 PE=1 SV=3	RAT	1	0.949803114	
395	1.1	1.1	29.46999967	3.139999881	3.139999881	Secernin-1 OS=Rattus norvegicus GN=Scrn1 PE=1 SV=1	RAT	1	1.033915997	0.725633025
396	1.07	1.16	33.32000077	0.723499991	0	Myosin-IXa OS=Rattus norvegicus GN=Myo9a PE=1 SV=1	RAT	0	0.918689013	0.407792985
397	1.07	1.07	42.10999906	19.74000037	6.578999758	Ubiquitin-conjugating enzyme E2 N OS=Rattus norvegicus GN=Ube2n PE=1 SV=1	RAT	1	1.032724023	0.802424729
398	1.06	1.09	24.67000037	1.396000013	1.396000013	Calcium-dependent secretion activator 1 OS=Rattus norvegicus GN=Cadps PE=1 SV=1	RAT	1	1.104102015	0.255320489
399	1.04	1.06	36.26999855	2.590999939	2.590999939	Centrosomal protein of 44 kDa OS=Rattus norvegicus GN=Cep44 PE=2 SV=1	RAT	1	0.955126524	
400	1.03	1.03	41.29999876	4.857999831	4.857999831	Acidic leucine-rich nuclear phosphoprotein 32 family member A OS=Rattus norvegicus GN=Anp32a PE=2 SV=1	RAT	1	0.990840018	
401	1.02	1.02	26.49999857	0.466000009	0.466000009	Multidrug resistance-associated protein 6 OS=Rattus norvegicus GN=Abcc6 PE=2 SV=1	RAT	1		
401	0	1.02	37.38999963	2.077000029	2.077000029	Transaldolase OS=Rattus norvegicus GN=Taldo1 PE=1 SV=2	RAT	1		
402	1.01	1.03	31.06000125	4.924000055	4.924000055	40S ribosomal protein S3a OS=Rattus norvegicus GN=Rps3a PE=1 SV=2	RAT	1	0.859001517	
403	1	1	33.55000019	11.60999984	11.60999984	V-type proton ATPase 16 kDa proteolipid subunit OS=Rattus norvegicus GN=Atp6v0c PE=2 SV=1	RAT	1	1.157888055	
404	0.96	0.96	52.07999945	5.832999945	5.832999945	Proteasome subunit beta type-1 OS=Rattus norvegicus GN=Psmb1 PE=1 SV=3	RAT	1	1.087007046	
405	0.96	0.96	33.50999951	5.155000091	5.155000091	40S ribosomal protein S9 OS=Rattus norvegicus GN=Rps9 PE=1 SV=4	RAT	1	1.065827012	0.570123672
406	0.95	1.07	29.10999954	0.568399997	0.328999991	Breast cancer type 2 susceptibility protein homolog OS=Rattus norvegicus GN=Brca2 PE=1 SV=1	RAT	1	1.087543964	
407	0.95	0.99	30.01999855	2.008000016	0	REVERSED Serine/threonine-protein kinase N1 OS=Rattus norvegicus GN=Pkn1 PE=1 SV=2	RAT	0	0.809099615	0.53217268
408	0.95	0.97	24.51000065	1.941999979	1.941999979	REVERSED Neuromedin-U receptor 1 OS=Rattus norvegicus GN=Nmur1 PE=1 SV=1	RAT	1	1.210401058	0.305290699
409	0.93	0.94	27.36000121	5.660000071	5.660000071	REVERSED WAP four-disulfide core domain protein 1 OS=Rattus norvegicus GN=Wfdc1 PE=1 SV=1	RAT	1		
410	0.87	0.89	34.15000141	11.99999973	0	Alcohol dehydrogenase [NADP+] OS=Rattus norvegicus GN=Akr1a1 PE=1 SV=2	RAT	0	1.013075948	0.900121689
411	0.87	0.87	52.38000154	37.5	8.332999796	ATP synthase subunit delta, mitochondrial OS=Rattus norvegicus GN=Atp5d PE=1 SV=2	RAT	1	0.852037787	
412	0.86	1.13	27.36999989	7.105000317	2.895000018	Mitogen-activated protein kinase 3 OS=Rattus norvegicus GN=Mapk3 PE=1 SV=5	RAT	1	1.173674941	0.363102108
412	3.35	3.38	30.45000136	12.84999996	8.380000293	Mitogen-activated protein kinase 1 OS=Rattus norvegicus GN=Mapk1 PE=1 SV=3	RAT	2	0.97287941	0.830055773
413	0.85	0.85	47.31999934	3.155000135	3.155000135	60S acidic ribosomal protein P0 OS=Rattus norvegicus GN=Rplp0 PE=1 SV=2	RAT	1	1.089761972	
414	0.84	0.87	32.82999992	1.51800001	1.51800001	REVERSED Cyclin-L1 OS=Rattus norvegicus GN=Ccnl1 PE=1 SV=1	RAT	1	1.103903055	0.505030274
415	0.84	0.87	48.82999957	3.125	3.125	ATP synthase subunit b, mitochondrial OS=Rattus norvegicus GN=Atp5f1 PE=1 SV=1	RAT	1	1.026195049	0.750145018
416	0.84	0.87	34.09000039	4.830000177	4.830000177	Alpha-2-HS-glycoprotein OS=Rattus norvegicus GN=Ahsg PE=1 SV=2	RAT	1	0.702407777	
417	0.83	0.83	35.06000042	5.029999837	1.219999976	Succinate dehydrogenase [ubiquinone] flavoprotein subunit, mitochondrial OS=Rattus norvegicus GN=Sdha PE=1 SV=1	RAT	1	0.960516214	0.613745213
418	0.82	0.82	56.99999928	19.32000071	6.763000041	Ras-related protein Rab-7a OS=Rattus norvegicus GN=Rab7a PE=1 SV=2	RAT	1	0.997018576	0.982073188
419	0.79	0.8	20.16000003	0.445799995	0.445799995	Sodium channel protein type 5 subunit alpha OS=Rattus norvegicus GN=Scn5a PE=2 SV=1	RAT	1	1.054057002	
420	0.79	0.79	37.61000037	2.531999908	2.531999908	REVERSED Copine-9 OS=Rattus norvegicus GN=Cpne9 PE=2 SV=1	RAT	1		
421	0.76	0.76	20.9800005	5.959000066	5.959000066	5-hydroxytryptamine receptor 1B OS=Rattus norvegicus GN=Htr1b PE=2 SV=1	RAT	1	1.129009962	
422	0.76	0.76	49.57000017	10.42999998	10.42999998	60S acidic ribosomal protein P2 OS=Rattus norvegicus GN=Rplp2 PE=1 SV=2	RAT	1	0.754190087	

423	0.75	0.77	47.24999964	2.982000075	0	Ras GTPase-activating protein SynGAP OS=Rattus norvegicus GN=Syngap1 PE=1 SV=2	RAT	0	1.05013895	0.709013581
424	0.72	0.72	43.97999942	1.91200003	1.91200003	REST corepressor 2 OS=Rattus norvegicus GN=Rcor2 PE=2 SV=1	RAT	1	0.820307374	
425	0.72	0.72	14.98000026	1.009999961	1.009999961	REVERSED Cryptochrome-2 OS=Rattus norvegicus GN=Cry2 PE=2 SV=1	RAT	1		
425	0.02	0.06	41.99000001	1.656999998	0	REVERSED Sterol-4-alpha-carboxylate 3-dehydrogenase, decarboxylating OS=Rattus norvegicus GN=Nsdhl PE=2 SV=1	RAT	0		
425	0	0.75	74.26999807	1.592000015	1.592000015	REVERSED NADH dehydrogenase [ubiquinone] 1 alpha subcomplex subunit 9, mitochondrial OS=Rattus norvegicus GN=Ndufa9 PE=1 SV=2	RAT	1		
426	0.71	0.97	67.04000235	1.173000038	0	REVERSED Spectrin beta chain, brain 2 OS=Rattus norvegicus GN=Sptbn2 PE=1 SV=2	RAT	0	0.914956272	0.537959099
427	0.7	0.75	43.20000112	1.941999979	1.941999979	DnaJ homolog subfamily A member 2 OS=Rattus norvegicus GN=Dnaja2 PE=2 SV=1	RAT	1	1.008090019	
428	0.7	0.72	23.08000028	2.91799996	2.91799996	Guanine nucleotide exchange factor for Rab-3A OS=Rattus norvegicus GN=Rab3il1 PE=1 SV=1	RAT	1	0.943993807	0.665189028
429	0.7	0.7	39.41999972	5.076000094	5.076000094	Calnexin OS=Rattus norvegicus GN=Canx PE=1 SV=1	RAT	2	1.11233604	0.479170591
430	0.7	0.7	16.67000055	3.736000136	3.736000136	Neuronal growth regulator 1 OS=Rattus norvegicus GN=Negr1 PE=1 SV=1	RAT	1	0.867324412	
431	0.68	0.7	12.09999993	0.466800015	0	REVERSED SCO-spondin OS=Rattus norvegicus GN=Sspo PE=2 SV=1	RAT	0	0.848902225	0.345722288
432	0.68	0.68	26.75000131	1.396999974	1.396999974	REVERSED Solute carrier family 12 member 3 OS=Rattus norvegicus GN=Slc12a3 PE=2 SV=2	RAT	1	0.902620971	
433	0.68	0.68	12.03000024	3.773999959	3.773999959	REVERSED ATP-sensitive inward rectifier potassium channel 8 OS=Rattus norvegicus GN=Kcnj8 PE=2 SV=1	RAT	1	1.061053991	0.543302178
434	0.68	0.68	13.78999949	2.122000046	2.122000046	Histone deacetylase 8 OS=Rattus norvegicus GN=Hdac8 PE=2 SV=1	RAT	1		
435	0.67	0.68	21.38999999	1.25099998	1.25099998	Metabotropic glutamate receptor 3 OS=Rattus norvegicus GN=Grm3 PE=1 SV=1	RAT	1	0.947075725	0.681522071
436	0.67	0.68	18.95000041	2.823000029	2.823000029	Clathrin light chain A OS=Rattus norvegicus GN=Clta PE=1 SV=1	RAT	1	1.201176047	0.330360115
437	0.67	0.67	28.67999971	0.744900014	0.744900014	Phospholipase D1 OS=Rattus norvegicus GN=Pld1 PE=1 SV=3	RAT	1	0.970851481	0.815110803
438	0.66	0.67	48.89999926	2.707000077	0	REVERSED ATPase family AAA domain-containing protein 3 OS=Rattus norvegicus GN=Atad3 PE=2 SV=1	RAT	0	1.09604001	0.377479702
439	0.66	0.66	18.68000031	5.494999886	5.494999886	Vacuolar protein sorting-associated protein 29 OS=Rattus norvegicus GN=Vps29 PE=1 SV=2	RAT	1	1.047142982	
440	0.65	0.65	22.46000022	0.33160001	0	REVERSED Cadherin-23 OS=Rattus norvegicus GN=Cdh23 PE=2 SV=1	RAT	0	0.821195185	0.371261686
441	0.65	0.65	31.18999898	1.948999986	1.948999986	REVERSED WD40 repeat-containing protein SMU1 OS=Rattus norvegicus GN=Smu1 PE=2 SV=1	RAT	1	0.907629073	0.357557714
442	0.65	0.65	37.92999983	3.448000178	3.448000178	REVERSED Secreted phosphoprotein 24 OS=Rattus norvegicus GN=Spp2 PE=2 SV=2	RAT	1		
443	0.64	0.66	75.41000247	2.127999999	0	REVERSED Neurofilament medium polypeptide OS=Rattus norvegicus GN=Nefm PE=1 SV=4	RAT	0	0.882085383	
444	0.64	0.65	41.33999944	0.996300019	0	WD repeat-containing protein 78 OS=Rattus norvegicus GN=Wdr78 PE=2 SV=1	RAT	0	1.119423985	
445	0.64	0.64	25.06999969	0.539099984	0	Cyclin-dependent kinase 12 OS=Rattus norvegicus GN=Cdk12 PE=1 SV=1	RAT	0	1.003350019	0.960227013
446	0.63	0.66	42.48000085	3.398000076	3.398000076	REVERSED [Pyruvate dehydrogenase [lipoamide]] kinase isozyme 4, mitochondrial OS=Rattus norvegicus GN=Pdk4 PE=2 SV=1	RAT	1	1.129904032	
447	0.63	0.63	37.25000024	1.444000006	0	REVERSED Pleckstrin homology domain-containing family G member 5 OS=Rattus norvegicus GN=Plekhg5 PE=1 SV=1	RAT	0	1.098242998	
448	0.63	0.63	24.30000007	2.195999958	0	Amphiphysin OS=Rattus norvegicus GN=Amph PE=1 SV=1	RAT	0	1.043720007	0.743370414
449	0.62	0.62	45.3399986	11.80000007	0	Probable tRNA threonylcarbamoyladenosine biosynthesis protein Osgep (Fragment) OS=Rattus norvegicus GN=Osgep PE=2 SV=1	RAT	0		
450	0.61	0.61	17.79000014	2.453999966	0	REVERSED Serine/threonine-protein kinase pim-3 OS=Rattus norvegicus GN=Pim3 PE=1 SV=3	RAT	0		
451	0.6	0.68	30.25000095	1.341000013	0	Protein kinase C beta type OS=Rattus norvegicus GN=Prkcb PE=1 SV=3	RAT	0	1.008095026	0.923739195
452	0.6	0.6	43.32999885	1.499000005	1.499000005	Midline-1 OS=Rattus norvegicus GN=Mid1 PE=2 SV=1	RAT	1		
453	0.58	0.65	33.57999921	0.646900013	0	Collagen alpha-1(XXVII) chain OS=Rattus norvegicus GN=Col27a1 PE=2 SV=1	RAT	0	1.011844993	0.90033561
454	0.58	0.58	32.85000026	1.197000034	0	Neuroendocrine convertase 1 OS=Rattus norvegicus GN=Pcsk1 PE=2 SV=1	RAT	0	0.928277016	0.59671241
455	0.58	0.58	43.95999908	2.349000052	0	REVERSED Mitochondrial 2-oxodicarboxylate carrier OS=Rattus norvegicus GN=Slc25a21 PE=2 SV=1	RAT	0		
456	0.57	0.61	56.61000013	6.102000177	0	Voltage-dependent anion-selective channel protein 2 OS=Rattus norvegicus GN=Vdac2 PE=1 SV=2	RAT	0	0.939434588	0.761745095
457	0.57	0.59	27.3999989	1.840000041	0	Mitochondrial-processing peptidase subunit beta OS=Rattus norvegicus GN=Pmpcb PE=1 SV=3	RAT	0	1.05697	
458	0.57	0.57	14.4600004	11.24000028	0	Vesicle-associated membrane protein-associated protein A OS=Rattus norvegicus GN=Vapa PE=1 SV=3	RAT	0	1.173493981	0.352517486
459	0.56	0.61	23.78000021	3.909000009	0	REVERSED Hepatitis A virus cellular receptor 1 homolog OS=Rattus norvegicus GN=Havcr1 PE=2 SV=1	RAT	0	0.987327516	0.91872561
460	0.56	0.58	19.15999949	2.199999988	0	Voltage-dependent calcium channel subunit alpha-2/delta-1 OS=Rattus norvegicus GN=Cacna2d1 PE=1 SV=1	RAT	0	1.017114997	
461	0.56	0.58	8.292999864	1.138000004	0	REVERSED Nuclear receptor subfamily 1 group D member 1 OS=Rattus norvegicus GN=Nr1d1 PE=2 SV=2	RAT	0	1.005010962	
462	0.55	0.64	33.97000134	3.325000033	2.37499997	UPF0415 protein C7orf25 homolog OS=Rattus norvegicus PE=2 SV=1	RAT	1	0.874788821	
463	0.55	0.57	30.25999963	1.164000016	0	Sodium-driven chloride bicarbonate exchanger OS=Rattus norvegicus GN=Slc4a10 PE=2 SV=1	RAT	0	1.114418983	
464	0.55	0.55	17.04999954	2.841000073	0	Protein NDRG4 OS=Rattus norvegicus GN=Ndrg4 PE=2 SV=1	RAT	0		
465	0.54	0.58	39.68999982	0.699299993	0	Arf-GAP with SH3 domain, ANK repeat and PH domain-containing protein 1 OS=Rattus norvegicus GN=Asap1 PE=2 SV=2	RAT	0		
466	0.53	0.97	24.97999966	0.985999964	0	REVERSED Protein FAM65A OS=Rattus norvegicus GN=Fam65a PE=2 SV=2	RAT	0	0.80422157	0.271147102
467	0.53	0.58	30.45000136	3.21800001	0	Endophilin-B2 OS=Rattus norvegicus GN=Sh3glb2 PE=2 SV=2	RAT	0	0.915564299	0.547486186
468	0.53	0.53	18.63999963	5.248999968	0	Cytosolic acyl coenzyme A thioester hydrolase OS=Rattus norvegicus GN=Acot7 PE=1 SV=4	RAT	0	1.006801963	0.957873821
469	0.53	0.53	26.23000145	4.26199995	0	REVERSED Serine/threonine-protein phosphatase 6 catalytic subunit OS=Rattus norvegicus GN=Ppp6c PE=2 SV=2	RAT	0	1.117459059	
470	0.51	0.56	33.77999961	1.617999934	0	Leucine zipper protein 1 OS=Rattus norvegicus GN=Luzp1 PE=2 SV=1	RAT	0	0.857667983	0.529022813
471	0.51	0.51	28.67999971	1.150000002	0	Protein FAM65A OS=Rattus norvegicus GN=Fam65a PE=2 SV=2	RAT	0	0.973467827	0.83170712
472	0.5	0.55	23.3799994	1.425999962	0	Adenylate cyclase type 5 OS=Rattus norvegicus GN=Adcy5 PE=2 SV=2	RAT	0	0.968630314	0.818259895
473	0.49	0.55	30.50999939	0.295199989	0	Apolipoprotein B-100 OS=Rattus norvegicus GN=Apob PE=1 SV=1	RAT	0	0.916976511	0.307723492
474	0.49	0.51	32.92999864	1.706999913	0	Centromere protein U OS=Rattus norvegicus GN=Mlf1ip PE=2 SV=1	RAT	0	0.738651276	
475	0.49	0.49	30.50999939	0.906299986	0	Hypoxia-inducible factor 3-alpha OS=Rattus norvegicus GN=Hif3a PE=1 SV=1	RAT	0	0.99176538	0.957261682
475	0	0.47	25.20999908	0.72730002	0	Hypoxia-inducible factor 1-alpha OS=Rattus norvegicus GN=Hif1a PE=2 SV=1	RAT	0		
476	0.49	0.49	18.75	2.71700006	0	Hsc70-interacting protein OS=Rattus norvegicus GN=St13 PE=1 SV=1	RAT	0	1.136262059	
477	0.49	0.49	12.16999963	7.825999707	0	Macrophage migration inhibitory factor OS=Rattus norvegicus GN=Mif PE=1 SV=4	RAT	0	1.164525986	
478	0.48	0.5	15.79000056	7.237000018	0	Mitochondrial fission 1 protein OS=Rattus norvegicus GN=Fis1 PE=1 SV=1	RAT	0		
479	0.47	0.55	29.89000082	4.214999825	0	REVERSED Paraspeckle component 1 OS=Rattus norvegicus GN=Pspc1 PE=2 SV=1	RAT	0	1.157768011	0.382043391
480	0.47	0.47	28.63999903	0.734400004	0	Protein kintoun OS=Rattus norvegicus GN=Dnaaf2 PE=2 SV=2	RAT	0		
480	0	0.03	34.77999866	3.260999918	0	Gastrokine-2 OS=Rattus norvegicus GN=Gkn2 PE=2 SV=1	RAT	0		
481	0.47	0.47	37.79000044	5.232999846	0	Neurotrimin OS=Rattus norvegicus GN=Ntm PE=1 SV=1	RAT	0	0.967113018	0.818103075
482	0.47	0.47	24.1899997	1.882000081	0	REVERSED Actin-related protein 2/3 complex subunit 1B OS=Rattus norvegicus GN=Arpc1b PE=2 SV=3	RAT	0	1.000098944	
483	0.46	0.46	17.64999926	3.113999963	0	Growth hormone-regulated TBC protein 1 OS=Rattus norvegicus GN=Grtp1 PE=2 SV=1	RAT	0	1.030521035	
484	0.45	0.5	42.17000008	15.65999985	15.65999985	Cytochrome c oxidase subunit 7A2, mitochondrial OS=Rattus norvegicus GN=Cox7a2 PE=1 SV=1	RAT	1	0.855812073	
485	0.45	0.45	80.18000126	4.504999891	0	REVERSED Glyceraldehyde-3-phosphate dehydrogenase OS=Rattus norvegicus GN=Gapdh PE=1 SV=3	RAT	0	1.029644012	
486	0.44	0.45	41.67000055	5.277999863	0	Protein phosphatase 1 regulatory subunit 7 OS=Rattus norvegicus GN=Ppp1r7 PE=1 SV=1	RAT	0	1.042703032	0.893937528
487	0.44	0.44	42.1299994	7.343000174	7.343000174	NADP-dependent malic enzyme OS=Rattus norvegicus GN=Me1 PE=1 SV=2	RAT	1		
488	0.43	0.46	27.57000029	1.992999949	0	PEX5-related protein OS=Rattus norvegicus GN=Pex5l PE=1 SV=1	RAT	0		
489	0.43	0.43	22.89000005	1.999999955	0	REVERSED Runt-related transcription factor 1 OS=Rattus norvegicus GN=Runx1 PE=2 SV=1	RAT	0	1.028910041	0.821641386
490	0.42	0.83	28.83999944	0.743100001	0	REVERSED Cytoplasmic dynein 2 heavy chain 1 OS=Rattus norvegicus GN=Dync2h1 PE=1 SV=1	RAT	0	1.211102962	
491	0.42	0.45	30.7099998	4.148999974	0	Glutathione S-transferase omega-1 OS=Rattus norvegicus GN=Gsto1 PE=1 SV=2	RAT	0		
492	0.42	0.44	36.14000082	1.944999956	0	REVERSED Filensin (Fragment) OS=Rattus norvegicus GN=Bfsp1 PE=2 SV=2	RAT	0		

493	0.42	0.42	46.65000141	3.795000166	0	UPF0667 protein C1orf55 homolog OS=Rattus norvegicus PE=2 SV=2	RAT	0	1.173231959	0.35318619
494	0.42	0.42	18.79999936	1.709000021	0	REVERSED Uncharacterized protein KIAA0895-like OS=Rattus norvegicus GN=Kiaa0895l PE=2 SV=1	RAT	0	0.786236823	
495	0.42	0.42	27.77999938	2.431000024	0	Phenazine biosynthesis-like domain-containing protein OS=Rattus norvegicus GN=Pbld PE=2 SV=1	RAT	0		
496	0.4	0.42	30.03999889	0.925900042	0	REVERSED Microtubule-associated protein 1S OS=Rattus norvegicus GN=Map1s PE=1 SV=1	RAT	0	1.18038404	0.342879593
497	0.4	0.42	27.32000053	3.195000067	3.195000067	Myelin-associated glycoprotein OS=Rattus norvegicus GN=Mag PE=1 SV=1	RAT	1	1.017760038	0.887856185
498	0.4	0.42	73.24000001	16.89999998	0	ATP synthase subunit e, mitochondrial OS=Rattus norvegicus GN=Atp5i PE=1 SV=3	RAT	0	1.034770012	0.903511882
499	0.4	0.4	54.22000289	3.89200002	2.501999959	Electrogenic sodium bicarbonate cotransporter 1 OS=Rattus norvegicus GN=Slc4a4 PE=1 SV=1	RAT	1	0.7213341	0.269489497
500	0.4	0.4	33.39000046	1.42099997	0	REVERSED Beta-catenin-like protein 1 OS=Rattus norvegicus GN=Ctnnbl1 PE=2 SV=1	RAT	0		
501	0.4	0.4	11.32000014	1.617000066	0	Protein NDRG2 OS=Rattus norvegicus GN=Ndrg2 PE=1 SV=1	RAT	0	1.002352953	0.992588222
501	0	0.38	35.3700012	2.040999942	0	Cyclin-G1 OS=Rattus norvegicus GN=Ccng1 PE=2 SV=2	RAT	0		
502	0.39	0.4	45.12999952	3.895999864	0	REVERSED 3'(2'),5'-bisphosphate nucleotidase 1 OS=Rattus norvegicus GN=Bpnt1 PE=1 SV=1	RAT	0	0.800802529	
503	0.38	0.58	34.58999991	0.558699993	0	REVERSED Protein bassoon OS=Rattus norvegicus GN=Bsn PE=1 SV=3	RAT	0	0.996453702	0.971726716
504	0.38	0.43	15.0000006	1.061000023	0	Protein O-linked-mannose beta-1,2-N-acetylglucosaminyltransferase 1 OS=Rattus norvegicus GN=Pomgnt1 PE=2 SV=1	RAT	0		
505	0.38	0.39	53.46000195	6.452000141	0	60S ribosomal protein L10a OS=Rattus norvegicus GN=Rpl10a PE=1 SV=2	RAT	0	1.148700953	0.592272699
506	0.38	0.38	23.00000042	0.97190002	0	REVERSED Rap guanine nucleotide exchange factor 3 OS=Rattus norvegicus GN=Rapgef3 PE=2 SV=2	RAT	0	0.892666817	
507	0.38	0.38	35.83999872	2.376000024	0	Protein disulfide-isomerase A3 OS=Rattus norvegicus GN=Pdia3 PE=1 SV=2	RAT	0	1.034605026	0.790665925
508	0.38	0.38	22.36000001	4.348000139	0	REVERSED ATP synthase subunit d, mitochondrial OS=Rattus norvegicus GN=Atp5h PE=1 SV=3	RAT	0		
509	0.37	0.42	31.63000047	2.716000006	0	Janus kinase and microtubule-interacting protein 1 OS=Rattus norvegicus GN=Jakmip1 PE=1 SV=1	RAT	0	1.105965018	
510	0.37	0.39	40.65999985	1.367000025	1.367000025	REVERSED Kinesin-like protein KIF1C OS=Rattus norvegicus GN=Kif1c PE=1 SV=1	RAT	1	0.993269026	
511	0.37	0.39	33.57999921	1.700000092	0	REVERSED Coiled-coil and C2 domain-containing protein 1A OS=Rattus norvegicus GN=Cc2d1a PE=2 SV=2	RAT	0	1.057858944	
512	0.37	0.37	34.36000049	1.322000008	0	REVERSED Uncharacterized protein C8orf45 homolog OS=Rattus norvegicus PE=2 SV=1	RAT	0		
513	0.36	2.96	72.81000018	19.47000027	9.122999758	Dihydropyrimidinase-related protein 3 OS=Rattus norvegicus GN=Dpysl3 PE=1 SV=2	RAT	4	1.007382989	0.9493922
514	0.36	0.38	26.37000084	1.185000036	0	Intraflagellar transport protein 81 homolog OS=Rattus norvegicus GN=Ift81 PE=2 SV=1	RAT	0		
515	0.36	0.37	14.13999945	2.163000032	0	Serine/threonine-protein phosphatase 2A 65 kDa regulatory subunit A beta isoform OS=Rattus norvegicus GN=Ppp2r1b PE=2 SV=1	RAT	0	1.136487961	0.424608409
516	0.36	0.36	21.01999968	3.232999891	0	26S protease regulatory subunit 7 OS=Rattus norvegicus GN=Psmc2 PE=1 SV=3	RAT	0	1.097167969	
517	0.36	0.36	19.67999935	5.397000164	0	Heme oxygenase 2 OS=Rattus norvegicus GN=Hmox2 PE=1 SV=1	RAT	0	0.905168116	
518	0.35	0.42	28.72999907	0.914600026	0	DNA repair protein RAD50 OS=Rattus norvegicus GN=Rad50 PE=1 SV=1	RAT	0	0.994325221	0.950955093
519	0.35	0.41	24.86999929	0.697400002	0	Chromodomain-helicase-DNA-binding protein 8 OS=Rattus norvegicus GN=Chd8 PE=1 SV=2	RAT	0	0.96165669	0.635459781
520	0.35	0.41	22.99000025	4.214999825	0	Calbindin OS=Rattus norvegicus GN=Calb1 PE=1 SV=2	RAT	0	1.217167974	
520	0	0.35	23.25000018	4.058999941	0	Calretinin OS=Rattus norvegicus GN=Calb2 PE=1 SV=1	RAT	0		
521	0.35	0.35	30.95999956	0.690399995	0	Rho GTPase-activating protein 27 OS=Rattus norvegicus GN=Arhgap27 PE=1 SV=1	RAT	0	1.04776001	0.644164026
522	0.35	0.35	32.76999891	2.941000089	0	Histone deacetylase 7 (Fragment) OS=Rattus norvegicus GN=Hdac7 PE=2 SV=1	RAT	0	1.029194951	
523	0.35	0.35	20.11000067	3.351999819	0	Neuronal-specific septin-3 OS=Rattus norvegicus GN=Sept3 PE=1 SV=2	RAT	0	0.921194613	0.561683476
524	0.35	0.35	26.01000071	5.093999952	0	Probable G-protein coupled receptor 173 OS=Rattus norvegicus GN=Gpr173 PE=2 SV=1	RAT	0	0.97818768	
525	0.34	0.4	26.14000142	1.38299996	1.38299996	REVERSED Threonyl-tRNA synthetase, mitochondrial OS=Rattus norvegicus GN=Tars2 PE=2 SV=1	RAT	1		
526	0.34	0.38	20.09000033	3.929999843	0	Clathrin light chain B OS=Rattus norvegicus GN=Cltb PE=1 SV=1	RAT	0	0.854000092	0.357419908
527	0.34	0.35	30.23999929	1.133000012	0	Glutamate receptor 2 OS=Rattus norvegicus GN=Gria2 PE=1 SV=2	RAT	0	1.036836028	
528	0.34	0.35	44.76000071	1.905000024	0	Transcriptional activator protein Pur-beta OS=Rattus norvegicus GN=Purb PE=1 SV=3	RAT	0	0.92665112	0.449748993
529	0.34	0.34	61.36000156	43.18000078	0	Thymosin beta-4 OS=Rattus norvegicus GN=Tmsb4x PE=1 SV=2	RAT	0	1.064839005	0.792363524
530	0.33	0.44	28.7499994	1.094000041	0	Dynactin subunit 1 OS=Rattus norvegicus GN=Dctn1 PE=2 SV=2	RAT	0	0.988981009	0.929370582
531	0.33	0.35	31.00000024	3.500000015	0	Cysteine dioxygenase type 1 OS=Rattus norvegicus GN=Cdo1 PE=1 SV=1	RAT	0		
532	0.33	0.33	29.62000072	2.830000035	0	REVERSED Protein FAM48A OS=Rattus norvegicus GN=Fam48a PE=2 SV=1	RAT	0	0.849751174	0.368155897
533	0.33	0.33	10.27999967	1.834999956	0	REVERSED Sterol O-acyltransferase 1 OS=Rattus norvegicus GN=Soat1 PE=2 SV=1	RAT	0	1.121855974	
534	0.32	3.63	75.7799983	5.401000008	5.401000008	Pyruvate kinase isozymes R/L OS=Rattus norvegicus GN=Pklr PE=2 SV=2	RAT	3	1.000386	0.998709023
535	0.32	1.24	25.6099999	1.190999988	0.270699989	REVERSED Peripheral-type benzodiazepine receptor-associated protein 1 OS=Rattus norvegicus GN=Bzrap1 PE=1 SV=2	RAT	1		
536	0.32	0.38	54.03000116	2.685000002	0	REVERSED ADP/ATP translocase 2 OS=Rattus norvegicus GN=Slc25a5 PE=1 SV=3	RAT	0	0.922605693	
537	0.32	0.34	26.06000006	1.057999954	0	REVERSED SH2B adapter protein 1 OS=Rattus norvegicus GN=Sh2b1 PE=1 SV=1	RAT	0	0.989504516	
538	0.32	0.32	29.21999991	1.817999966	0	Low-density lipoprotein receptor-related protein 3 OS=Rattus norvegicus GN=Lrp3 PE=1 SV=1	RAT	0	1.094969034	0.539325893
539	0.32	0.32	31.31000102	4.797999933	0	Cell adhesion molecule 3 OS=Rattus norvegicus GN=Cadm3 PE=2 SV=1	RAT	0	0.897759199	
540	0.31	0.46	24.94000047	1.892000064	0	REVERSED Hamartin OS=Rattus norvegicus GN=Tsc1 PE=1 SV=1	RAT	0		
541	0.31	0.38	34.65999961	0.5076	0	REVERSED SH3 and multiple ankyrin repeat domains protein 1 OS=Rattus norvegicus GN=Shank1 PE=1 SV=1	RAT	0	0.993843615	0.946489513
542	0.31	0.33	30.91000021	0.525100017	0	Aldehyde oxidase OS=Rattus norvegicus GN=Aox1 PE=2 SV=1	RAT	0	1.031522036	0.663031816
543	0.3	0.46	38.71999979	1.279000007	0	Neurabin-1 OS=Rattus norvegicus GN=Ppp1r9a PE=1 SV=1	RAT	0	1.032112956	0.803899884
544	0.3	0.31	28.52999866	4.20400016	0	REVERSED Homeobox protein Hox-A1 OS=Rattus norvegicus GN=Hoxa1 PE=2 SV=1	RAT	0	0.952976108	
545	0.3	0.3	19.14000064	1.154999994	0	Kelch repeat and BTB domain-containing protein 10 OS=Rattus norvegicus GN=Kbtbd10 PE=1 SV=1	RAT	0		
546	0.3	0.3	36.35999858	4.544999823	0	60S ribosomal protein L18a OS=Rattus norvegicus GN=Rpl18a PE=2 SV=1	RAT	0	1.127789021	
547	0.29	0.31	23.17000031	2.015000023	0	DnaJ homolog subfamily A member 1 OS=Rattus norvegicus GN=Dnaja1 PE=2 SV=1	RAT	0		
548	0.28	0.33	29.10000086	1.379000023	0	REVERSED Antigen peptide transporter 1 OS=Rattus norvegicus GN=Tap1 PE=1 SV=2	RAT	0	0.974465311	0.785093188
549	0.28	0.32	20.09000033	0.172499998	0	Dynein heavy chain 7, axonemal OS=Rattus norvegicus GN=Dnah7 PE=2 SV=2	RAT	0	0.979863226	0.798017323
550	0.28	0.31	24.42000061	0.313799991	0	REVERSED Myosin-XVI OS=Rattus norvegicus GN=Myo16 PE=1 SV=1	RAT	0	1.139780998	0.248690099
551	0.28	0.3	33.62999856	1.011999976	0	REVERSED Potassium voltage-gated channel subfamily C member 3 OS=Rattus norvegicus GN=Kcnc3 PE=2 SV=1	RAT	0	1.012066007	
552	0.28	0.29	25.24999976	1.654000022	0	Glutamate receptor 1 OS=Rattus norvegicus GN=Gria1 PE=1 SV=2	RAT	0		
553	0.28	0.29	25.88	4.152999818	0	Mu-crystallin homolog OS=Rattus norvegicus GN=Crym PE=1 SV=1	RAT	0		
554	0.28	0.28	21.92000002	5.384999886	0	REVERSED Keratin, type II cytoskeletal 72 OS=Rattus norvegicus GN=Krt72 PE=2 SV=2	RAT	0	1.011448979	0.927220523
555	0.28	0.28	14.24999982	3.166000172	0	Sialidase-2 OS=Rattus norvegicus GN=Neu2 PE=2 SV=1	RAT	0	0.819362402	
556	0.27	0.3	29.84000146	0.578799983	0.578799983	Regulating synaptic membrane exocytosis protein 2 OS=Rattus norvegicus GN=Rims2 PE=1 SV=1	RAT	1	1.033040047	0.760091603
557	0.27	0.28	30.70000112	2.129999921	0	REVERSED Bromodomain-containing protein 2 OS=Rattus norvegicus GN=Brd2 PE=2 SV=1	RAT	0	1.109735966	0.494575113
558	0.27	0.28	47.74000049	2.160999924	0	Heat shock 70 kDa protein 14 OS=Rattus norvegicus GN=Hspa14 PE=2 SV=1	RAT	0	1.121855974	
559	0.27	0.28	29.6600014	3.145999834	0	Oxysterols receptor LXR-alpha OS=Rattus norvegicus GN=Nr1h3 PE=2 SV=1	RAT	0	1.007490993	
560	0.26	0.38	28.20000052	0	0	Microtubule-actin cross-linking factor 1 OS=Rattus norvegicus GN=Macf1 PE=1 SV=1	RAT	0	0.869941592	0.101972602
561	0.26	0.28	15.82999974	2.422999963	0	Secretogranin-2 OS=Rattus norvegicus GN=Scg2 PE=2 SV=1	RAT	0	0.847759783	
562	0.26	0.26	29.5599997	0.780500006	0	Dihydropyrimidine dehydrogenase [NADP+] OS=Rattus norvegicus GN=Dpyd PE=2 SV=1	RAT	0	1.035153031	0.786377728
563	0.25	0.29	28.76000106	2.35399995	0	REVERSED Short transient receptor potential channel 4 OS=Rattus norvegicus GN=Trpc4 PE=1 SV=2	RAT	0		
564	0.25	0.28	31.67999983	3.649999946	1.606000029	Stromal interaction molecule 1 OS=Rattus norvegicus GN=Stim1 PE=1 SV=1	RAT	1	0.986146212	

565	0.25	0.25	33.64000022	1.807999983	0	REVERSED Neurexin-2-alpha OS=Rattus norvegicus GN=Nrxn2 PE=1 SV=3	RAT	0	1.006126046	0.953186691
566	0.25	0.25	33.28000009	2.996000089	0	REVERSED Insulin receptor substrate 1 OS=Rattus norvegicus GN=Irs1 PE=1 SV=1	RAT	0	1.096928	
567	0.25	0.25	64.09999728	10.13000011	10.13000011	Dihydrolipoyllysine-residue succinyltransferase component of 2-oxoglutarate dehydrogenase complex, mitochondrial OS=Rattus norvegicus GN=Dlst PE=1 SV=2	RAT	3	1.288012981	
568	0.25	0.25	40.27999938	1.389000006	0	REVERSED Pre-mRNA-processing factor 19 OS=Rattus norvegicus GN=Prpf19 PE=2 SV=2	RAT	0		
569	0.25	0.25	34.11999941	3.937000036	0	REVERSED NF-kappa-B inhibitor-like protein 1 OS=Rattus norvegicus GN=Nfkbil1 PE=2 SV=2	RAT	0		
570	0.24	0.53	36.35999858	0.191999995	0	REVERSED Plectin OS=Rattus norvegicus GN=Plec PE=1 SV=2	RAT	0	0.975352526	0.682823122
571	0.24	0.3	17.97000021	0.435299985	0	Adenylate cyclase type 10 OS=Rattus norvegicus GN=Adcy10 PE=1 SV=1	RAT	0	0.988157272	0.923830211
572	0.24	0.28	35.96000075	0	0	REVERSED Myosin-IXb OS=Rattus norvegicus GN=Myo9b PE=1 SV=1	RAT	0	1.010084987	0.886107385
573	0.24	0.27	26.3500005	0.748099992	0	Zinc finger and BTB domain-containing protein 38 OS=Rattus norvegicus GN=Zbtb38 PE=2 SV=1	RAT	0	1.048858047	0.617502093
574	0.24	0.25	38.60999942	4.633000121	0	REVERSED Estradiol 17-beta-dehydrogenase 8 OS=Rattus norvegicus GN=Hsd17b8 PE=2 SV=1	RAT	0	0.922293782	0.563676119
575	0.24	0.25	22.99000025	2.135000005	0	Tyrosyl-DNA phosphodiesterase 1 OS=Rattus norvegicus GN=Tdp1 PE=2 SV=1	RAT	0	1.013368964	0.933364391
576	0.24	0.24	21.97999954	0.729200011	0	Semaphorin-6C OS=Rattus norvegicus GN=Sema6c PE=2 SV=1	RAT	0	0.971531689	0.827745497
577	0.24	0.24	34.77999866	3.187999874	0	Ribosome production factor 1 OS=Rattus norvegicus GN=Rpf1 PE=2 SV=2	RAT	0	0.953685224	0.643023789
578	0.24	0.24	31.20000064	7.19999969	0	REVERSED Pleckstrin homology-like domain family A member 3 OS=Rattus norvegicus GN=Phlda3 PE=2 SV=1	RAT	0	0.837429583	
579	0.24	0.24	34.70999897	5.784999952	0	Microtubule-associated proteins 1A/1B light chain 3A OS=Rattus norvegicus GN=Map1lc3a PE=1 SV=1	RAT	0	1.011430025	
579	0	0.24	26.06000006	4.930000007	0	Microtubule-associated proteins 1A/1B light chain 3B OS=Rattus norvegicus GN=Map1lc3b PE=1 SV=3	RAT	0		
580	0.23	0.48	32.89000094	4.278000072	0	Transcription termination factor, mitochondrial OS=Rattus norvegicus GN=Mterf PE=1 SV=1	RAT	0	0.854160428	
581	0.23	0.37	24.05000031	0.307999994	0	E3 ubiquitin-protein ligase UBR4 OS=Rattus norvegicus GN=Ubr4 PE=2 SV=2	RAT	0	1.078613997	0.334848613
582	0.23	0.37	44.72000003	9.888000041	0	Rab GDP dissociation inhibitor beta OS=Rattus norvegicus GN=Gdi2 PE=1 SV=2	RAT	0	0.985264599	0.906897604
583	0.23	0.28	28.60000134	0.717899995	0.448699994	REVERSED Breast cancer type 2 susceptibility protein homolog OS=Rattus norvegicus GN=Brca2 PE=1 SV=1	RAT	1	0.848103225	
584	0.23	0.25	44.06000078	1.013999991	0	Heterogeneous nuclear ribonucleoprotein M OS=Rattus norvegicus GN=Hnrnpm PE=1 SV=4	RAT	0	0.9889552	0.888083696
585	0.23	0.25	19.07999963	1.155999955	0	DNA-binding protein RFX2 OS=Rattus norvegicus GN=Rfx2 PE=2 SV=1	RAT	0	0.905487776	
586	0.23	0.23	36.23000085	1.479000039	0	T-complex protein 1 subunit epsilon OS=Rattus norvegicus GN=Cct5 PE=1 SV=1	RAT	0	0.992348075	0.950904071
587	0.23	0.23	20.2000007	2.356999926	0	REVERSED Zinc finger protein 467 OS=Rattus norvegicus GN=Znf467 PE=2 SV=1	RAT	0	1.043182015	
588	0.23	0.23	14.94999975	4.298999906	0	EH domain-containing protein 3 OS=Rattus norvegicus GN=Ehd3 PE=1 SV=2	RAT	0	1.330237985	
589	0.23	0.23	12.89000064	5.031000078	5.031000078	Solute carrier family 25 member 34 OS=Rattus norvegicus GN=Slc25a34 PE=2 SV=2	RAT	1		
590	0.22	0.31	23.39999974	1.032000035	0	REVERSED Dual oxidase 1 OS=Rattus norvegicus GN=Duox1 PE=1 SV=1	RAT	0	0.823765695	0.300120294
591	0.22	0.3	26.26999915	0.599400001	0	REVERSED Serine/threonine-protein kinase TAO1 OS=Rattus norvegicus GN=Taok1 PE=1 SV=1	RAT	0	1.104536057	0.50947839
592	0.22	0.25	22.81000018	0.279899989	0	Cadherin EGF LAG seven-pass G-type receptor 2 (Fragment) OS=Rattus norvegicus GN=Celsr2 PE=2 SV=1	RAT	0	1.502560019	
593	0.22	0.25	34.34000015	0.552800018	0	Transient receptor potential cation channel subfamily M member 1 OS=Rattus norvegicus GN=Trpm1 PE=2 SV=1	RAT	0	1.003898025	
594	0.22	0.23	24.86000061	4.926000163	0	REVERSED Programmed cell death 6-interacting protein OS=Rattus norvegicus GN=Pdcd6ip PE=1 SV=2	RAT	0	1.046231985	0.521004975
595	0.22	0.22	32.48000145	0.457500014	0	REVERSED Neurexin-1-alpha OS=Rattus norvegicus GN=Nrxn1 PE=1 SV=3	RAT	0	1.072257996	
595	0	0.2	28.42000127	1.496000029	0	REVERSED Neurexin-1-beta OS=Rattus norvegicus GN=Nrxn1 PE=1 SV=1	RAT	0		
596	0.22	0.22	34.31000113	0.912399963	0	REVERSED Inactive phospholipase C-like protein 1 OS=Rattus norvegicus GN=Plcl1 PE=1 SV=1	RAT	0	0.939804912	
597	0.22	0.22	26.64000094	1.824999973	0	von Willebrand factor A domain-containing protein 5A OS=Rattus norvegicus GN=Vwa5a PE=2 SV=1	RAT	0	1.063199997	
598	0.22	0.22	36.48999929	9.730000049	0	Galanin receptor type 3 OS=Rattus norvegicus GN=Galr3 PE=2 SV=1	RAT	0	1.057685018	0.746798575
599	0.22	0.22	13.40000033	4.574999958	0	Macrophage asialoglycoprotein-binding protein 1 OS=Rattus norvegicus GN=Mgl1 PE=1 SV=1	RAT	0		
600	0.22	0.22	51.84999704	12.03999966	0	CDGSH iron-sulfur domain-containing protein 1 OS=Rattus norvegicus GN=Cisd1 PE=3 SV=1	RAT	0	1.036219001	
601	0.21	1.3	68.62999797	7.379999757	2.95199994	Arginine and glutamate-rich protein 1 OS=Rattus norvegicus GN=Arglu1 PE=2 SV=1	RAT	1		
602	0.21	0.97	32.35000074	4.778999835	1.837999932	F-actin-capping protein subunit beta OS=Rattus norvegicus GN=Capzb PE=1 SV=1	RAT	1	0.766353905	
603	0.21	0.37	28.16999853	1.131999958	0	REVERSED GRIP and coiled-coil domain-containing protein 2 OS=Rattus norvegicus GN=Gcc2 PE=1 SV=1	RAT	0	0.954603076	0.628728211
604	0.21	0.25	30.23999929	0.337300007	0	Fibronectin type III domain-containing protein 1 OS=Rattus norvegicus GN=Fndc1 PE=2 SV=2	RAT	0	0.978470802	0.862026811
605	0.21	0.23	14.23999965	3.779000044	0	REVERSED Trace amine-associated receptor 8b OS=Rattus norvegicus GN=Taar8b PE=3 SV=1	RAT	0	0.879446507	0.449844599
606	0.21	0.22	35.01999974	1.240999997	0	REVERSED Nck-associated protein 1 OS=Rattus norvegicus GN=Nckap1 PE=2 SV=1	RAT	0	0.907117128	
607	0.21	0.21	30.89999855	0.433199992	0	Kinesin-like protein KIF15 OS=Rattus norvegicus GN=Kif15 PE=2 SV=1	RAT	0	0.927209973	0.584579289
608	0.21	0.21	26.32000148	0.736800022	0	Mitogen-activated protein kinase kinase kinase 1 OS=Rattus norvegicus GN=Map3k1 PE=1 SV=1	RAT	0	0.948462427	0.687128782
609	0.21	0.21	15.18999934	3.164999932	0	C1GALT1-specific chaperone 1 OS=Rattus norvegicus GN=C1galt1c1 PE=1 SV=1	RAT	0	0.786800086	
610	0.21	0.21	10.44000015	3.846000135	0	Ferritin heavy chain OS=Rattus norvegicus GN=Fth1 PE=1 SV=3	RAT	0		
611	0.21	0.21	16.71999991	6.485000253	0	REVERSED Uncharacterized protein C12orf41 homolog OS=Rattus norvegicus PE=2 SV=1	RAT	0	0.82386142	
612	0.21	0.21	2.848000079	2.848000079	0	POU domain, class 2, transcription factor 1 (Fragment) OS=Rattus norvegicus GN=Pou2f1 PE=2 SV=2	RAT	0	1.295647979	
613	0.2	3.81	77.56000161	22.43999988	10.72999984	Ras-related protein Rab-1A OS=Rattus norvegicus GN=Rab1A PE=1 SV=3	RAT	2	1.004709005	0.960526884
614	0.2	1.41	45.62999904	5.330000073	1.919000037	Desmin OS=Rattus norvegicus GN=Des PE=2 SV=2	RAT	1	1.08265698	0.787352204
615	0.2	0.38	26.01999938	0.271699997	0	REVERSED Cadherin EGF LAG seven-pass G-type receptor 3 OS=Rattus norvegicus GN=Celsr3 PE=2 SV=1	RAT	0	0.951004386	0.735002697
616	0.2	0.36	34.36000049	0.634799991	0	Protein bassoon OS=Rattus norvegicus GN=Bsn PE=1 SV=3	RAT	0	1.059193015	0.414183587
617	0.2	0.25	8.122999966	4.202000052	0	UDP-GlcNAc:betaGal beta-1,3-N-acetylglucosaminyltransferase-like protein 1 OS=Rattus norvegicus GN=B3gntl1 PE=2 SV=1	RAT	0		
618	0.2	0.24	28.99999917	5.333000049	0	Transcription factor E2F5 (Fragment) OS=Rattus norvegicus GN=E2f5 PE=2 SV=1	RAT	0	1.017542005	
619	0.2	0.23	51.55000091	0.918999966	0	SRC kinase signaling inhibitor 1 OS=Rattus norvegicus GN=Srcin1 PE=1 SV=1	RAT	0	0.992663503	0.984912813
620	0.2	0.23	25.27999878	0.966900028	0	Phosphatidylinositol 3-kinase regulatory subunit alpha OS=Rattus norvegicus GN=Pik3r1 PE=1 SV=1	RAT	0	1.271801949	
621	0.2	0.22	43.09999943	1.279999968	0	REVERSED Metastasis-associated protein MTA1 OS=Rattus norvegicus GN=Mta1 PE=1 SV=1	RAT	0	0.747656524	
622	0.2	0.22	31.47000074	0.7675	0	Metabotropic glutamate receptor 4 OS=Rattus norvegicus GN=Grm4 PE=1 SV=1	RAT	0	0.913780928	0.531866372
623	0.2	0.22	32.3300004	3.016999923	0	Protein lin-7 homolog A OS=Rattus norvegicus GN=Lin7a PE=1 SV=2	RAT	0	1.12846601	0.461041689
624	0.2	0.2	31.81999922	2.405999973	0	REVERSED Ras-related GTP-binding protein B OS=Rattus norvegicus GN=RragB PE=1 SV=1	RAT	0	0.96949178	
625	0.2	0.2	38.83000016	3.190999851	0	REVERSED 60S ribosomal protein L18 OS=Rattus norvegicus GN=Rpl18 PE=2 SV=2	RAT	0		
626	0.2	0.2	15.81999958	7.909999788	0	Protein CutA OS=Rattus norvegicus GN=Cuta PE=1 SV=2	RAT	0	0.889075994	
627	0.19	13.6	84.78999734	33.4100008	31.34000003	Beta-enolase OS=Rattus norvegicus GN=Eno3 PE=1 SV=3	RAT	19	0.988058388	
628	0.19	12.83	99.32000041	85.0300014	70.0699985	Hemoglobin subunit beta-2 OS=Rattus norvegicus PE=1 SV=2	RAT	9	1.212404013	0.325935513
629	0.19	2.27	70.19000053	11.06000021	5.288000032	Ras-related protein Rab-6A OS=Rattus norvegicus GN=Rab6a PE=2 SV=2	RAT	1	1.073837042	
630	0.19	0.6	38.24999928	0.686899992	0	REVERSED PDZ domain-containing protein 2 OS=Rattus norvegicus GN=Pdzd2 PE=1 SV=1	RAT	0	0.911183596	0.218512297
631	0.19	0.37	24.68000054	1.028000005	0	REVERSED Valyl-tRNA synthetase OS=Rattus norvegicus GN=Vars PE=2 SV=2	RAT	0	0.86766839	0.391258806
632	0.19	0.25	14.30000067	0.457199989	0	Slit homolog 1 protein OS=Rattus norvegicus GN=Slit1 PE=1 SV=1	RAT	0	0.80084908	
633	0.19	0.23	44.38000023	2.465000004	0	Leucine-rich repeat transmembrane protein FLRT3 OS=Rattus norvegicus GN=Flrt3 PE=2 SV=1	RAT	0	0.905068398	0.497297406
634	0.19	0.23	30.77000082	1.737000048	0	REVERSED Sex hormone-binding globulin OS=Rattus norvegicus GN=Shbg PE=1 SV=1	RAT	0	1.112751007	0.475413501
635	0.19	0.22	28.7499994	0.162600004	0	Cytoplasmic dynein 2 heavy chain 1 OS=Rattus norvegicus GN=Dync2h1 PE=1 SV=1	RAT	0	1.011759043	0.944816887
636	0.19	0.21	40.81999958	4.081999883	0	60S ribosomal protein L19 OS=Rattus norvegicus GN=Rpl19 PE=1 SV=1	RAT	0	1.270419002	

637	0.19	0.2	18.70000064	2.033000067	0	Transmembrane protein 39B OS=Rattus norvegicus GN=Tmem39b PE=2 SV=1	RAT	0	0.850450277	0.352120787
638	0.19	0.19	44.78999972	6.25	0	REVERSED Serine/threonine-protein phosphatase PGAM5, mitochondrial OS=Rattus norvegicus GN=Pgam5 PE=2 SV=1	RAT	0	1.061280966	0.656267583
639	0.18	0.46	32.10000098	0.165700004	0	REVERSED Microtubule-actin cross-linking factor 1 OS=Rattus norvegicus GN=Macf1 PE=1 SV=1	RAT	0	1.007861972	0.909618616
640	0.18	0.21	31.13000095	0.551000005	0	SH3 and multiple ankyrin repeat domains protein 3 OS=Rattus norvegicus GN=Shank3 PE=1 SV=2	RAT	0	0.90214771	0.335540086
641	0.18	0.21	38.35000098	0.958800036	0	REVERSED Glutamate receptor-interacting protein 2 OS=Rattus norvegicus GN=Grip2 PE=1 SV=1	RAT	0	0.899584711	
642	0.18	0.21	19.65000033	1.889000088	0	Striatin-3 OS=Rattus norvegicus GN=Strn3 PE=2 SV=2	RAT	0	1.01593101	0.900696218
643	0.18	0.2	18.67000014	0.372399995	0	REVERSED General transcription factor 3C polypeptide 1 OS=Rattus norvegicus GN=Gtf3c1 PE=1 SV=1	RAT	0	0.866687477	0.219163299
644	0.18	0.19	31.56000078	0.852899998	0	REVERSED Glutamate [NMDA] receptor subunit zeta-1 OS=Rattus norvegicus GN=Grin1 PE=1 SV=1	RAT	0	0.959866703	
645	0.18	0.18	35.13000011	1.229999959	0	Collagen alpha-1(III) chain OS=Rattus norvegicus GN=Col3a1 PE=2 SV=3	RAT	0	0.96040231	0.802985013
646	0.18	0.18	34.29999948	2.900999971	0	REVERSED Dual specificity tyrosine-phosphorylation-regulated kinase 3 OS=Rattus norvegicus GN=Dyrk3 PE=2 SV=1	RAT	0	1.122174025	
647	0.17	0.31	32.73999989	0.153400004	0	Striated muscle-specific serine/threonine-protein kinase OS=Rattus norvegicus GN=Speg PE=2 SV=2	RAT	0	0.982969582	0.832930028
648	0.17	0.27	19.60999966	0.493199984	0	REVERSED DNA (cytosine-5)-methyltransferase 1 OS=Rattus norvegicus GN=Dnmt1 PE=1 SV=2	RAT	0	0.829192579	
649	0.17	0.23	20.63000053	5.820000172	0	Receptor expression-enhancing protein 5 OS=Rattus norvegicus GN=Reep5 PE=1 SV=1	RAT	0	0.882941008	
650	0.17	0.22	27.86999941	2.246999927	0	REVERSED C-terminal-binding protein 2 OS=Rattus norvegicus GN=Ctbp2 PE=1 SV=2	RAT	0	0.85387218	0.360852003
651	0.17	0.21	30.18999994	1.391999982	0	Inositol-trisphosphate 3-kinase B OS=Rattus norvegicus GN=Itpkb PE=1 SV=3	RAT	0	0.74597162	0.02659245
652	0.17	0.17	64.3599987	10.05999967	10.05999967	Adenylyl cyclase-associated protein 2 OS=Rattus norvegicus GN=Cap2 PE=2 SV=1	RAT	2	1.059945941	
653	0.17	0.17	63.01000118	4.913000017	0	REVERSED Succinyl-CoA ligase [GDP-forming] subunit alpha, mitochondrial OS=Rattus norvegicus GN=Suclg1 PE=2 SV=2	RAT	0	1.229567051	
654	0.17	0.17	20.03999949	0.715699978	0	Macrophage colony-stimulating factor 1 receptor OS=Rattus norvegicus GN=Csf1r PE=2 SV=1	RAT	0	1.182816982	0.175854906
655	0.17	0.17	76.96999907	7.272999734	0	Alanine and arginine-rich domain-containing protein OS=Rattus norvegicus GN=Aard PE=2 SV=1	RAT	0	1.038285971	
656	0.17	0.17	26.05000138	0.803900044	0	Mitochondrial Rho GTPase 2 OS=Rattus norvegicus GN=Rhot2 PE=2 SV=1	RAT	0	0.91488862	
657	0.17	0.17	26.03999972	1.49299996	0	REVERSED Polypeptide N-acetylgalactosaminyltransferase 10 OS=Rattus norvegicus GN=Galnt10 PE=2 SV=1	RAT	0	0.860205114	
658	0.17	0.17	27.59999931	1.982999966	0	REVERSED Dolichyl-diphosphooligosaccharide--protein glycosyltransferase subunit 1 OS=Rattus norvegicus GN=Rpn1 PE=2 SV=1	RAT	0	1.052237988	0.708103001
659	0.17	0.17	4.681000113	4.681000113	0	Secretory carrier-associated membrane protein 5 OS=Rattus norvegicus GN=Scamp5 PE=2 SV=1	RAT	0	0.902948976	
660	0.16	0.35	33.03999901	0.078659999	0	REVERSED Protein piccolo OS=Rattus norvegicus GN=Pclo PE=1 SV=1	RAT	0	1.026293039	0.833123088
661	0.16	0.24	27.700001	2.195999958	0	Protein kinase C zeta type OS=Rattus norvegicus GN=Prkcz PE=1 SV=2	RAT	0	0.878944993	0.429344714
662	0.16	0.2	37.97000051	0	0	REVERSED Adenomatous polyposis coli protein OS=Rattus norvegicus GN=Apc PE=1 SV=1	RAT	0	0.963817775	0.589711487
663	0.16	0.2	34.2200011	1.823000051	0	Nucleolin OS=Rattus norvegicus GN=Ncl PE=1 SV=3	RAT	0	1.148301005	
664	0.16	0.19	41.06999934	1.785999909	0	Formin-binding protein 1 OS=Rattus norvegicus GN=Fnbp1 PE=1 SV=2	RAT	0	1.467087984	
665	0.16	0.19	33.01999867	8.019000292	0	Fibroblast growth factor 20 OS=Rattus norvegicus GN=Fgf20 PE=2 SV=1	RAT	0	0.889677703	
666	0.16	0.18	24.1899997	1.410000026	0	REVERSED Neuropilin-1 OS=Rattus norvegicus GN=Nrp1 PE=1 SV=1	RAT	0	0.973678589	
667	0.16	0.18	22.81000018	1.754000038	0	Probable G-protein coupled receptor 176 OS=Rattus norvegicus GN=Gpr176 PE=2 SV=2	RAT	0	0.90293318	
668	0.16	0.17	50	1.913999952	0	REVERSED Testis-specific H1 histone OS=Rattus norvegicus GN=H1fnt PE=2 SV=1	RAT	0	0.905372322	0.252275795
669	0.16	0.17	25.99999905	5.736000091	0	Succinate-semialdehyde dehydrogenase, mitochondrial OS=Rattus norvegicus GN=Aldh5a1 PE=1 SV=2	RAT	0	0.884141088	0.458645403
670	0.16	0.17	16.27999991	1.252999995	0	REVERSED Fibrinogen beta chain OS=Rattus norvegicus GN=Fgb PE=1 SV=4	RAT	0	1.046265006	
671	0.16	0.16	39.10999894	1.593999937	0	Coiled-coil and C2 domain-containing protein 1A OS=Rattus norvegicus GN=Cc2d1a PE=2 SV=2	RAT	0	0.927971721	0.750895917
672	0.16	0.16	86.90000176	14.48000073	0	REVERSED 40S ribosomal protein S19 OS=Rattus norvegicus GN=Rps19 PE=2 SV=3	RAT	0	0.988373876	0.960820913
673	0.16	0.16	51.17999911	2.693999931	0	Calponin-1 OS=Rattus norvegicus GN=Cnn1 PE=2 SV=1	RAT	0	1.210420966	
674	0.16	0.16	30.45000136	4.478000104	0	POU domain, class 5, transcription factor 2 OS=Rattus norvegicus GN=Pou5f2 PE=2 SV=2	RAT	0		
675	0.16	0.16	36.66999936	8.666999638	0	REVERSED Ribonuclease pancreatic delta-type OS=Rattus norvegicus GN=Rnase1d PE=3 SV=1	RAT	0	1.113572955	
676	0.15	25.77	90.71999788	61.26999855	56.23000264	Actin, alpha cardiac muscle 1 OS=Rattus norvegicus GN=Actc1 PE=1 SV=1	RAT	32	1.194573998	0.561941803
676	0	23.68	95.23000121	58.35999846	53.32000256	Actin, aortic smooth muscle OS=Rattus norvegicus GN=Acta2 PE=1 SV=1	RAT	31		
676	0	21.48	89.6600008	61.79999709	56.49999976	Actin, alpha skeletal muscle OS=Rattus norvegicus GN=Acta1 PE=2 SV=1	RAT	27		
676	0	19.28	95.74000239	51.59999728	46.54000103	Actin, gamma-enteric smooth muscle OS=Rattus norvegicus GN=Actg2 PE=2 SV=1	RAT	25		
677	0.15	0.25	28.34999859	0.581899984	0	Metabotropic glutamate receptor 5 OS=Rattus norvegicus GN=Grm5 PE=1 SV=2	RAT	0		
678	0.15	0.23	30.23000062	0.716000004	0	REVERSED Phosphatidylinositol-3,4,5-trisphosphate 5-phosphatase 2 OS=Rattus norvegicus GN=Inppl1 PE=1 SV=1	RAT	0	1.008980989	0.922394395
679	0.15	0.19	34.94000137	1.727999933	0	REVERSED Leucine-rich repeat-containing protein 8A OS=Rattus norvegicus GN=Lrrc8a PE=2 SV=1	RAT	0		
680	0.15	0.17	39.1200006	1.645000093	0	REVERSED Partitioning defective 3 homolog OS=Rattus norvegicus GN=Pard3 PE=1 SV=1	RAT	0	1.151435971	0.392574787
681	0.15	0.17	25.29000044	1.900999993	0	REVERSED Keratin, type I cytoskeletal 10 OS=Rattus norvegicus GN=Krt10 PE=2 SV=1	RAT	0	1.184363008	
682	0.15	0.16	30.39999902	1.494999975	0	REVERSED Protein FAM194A OS=Rattus norvegicus GN=Fam194a PE=2 SV=1	RAT	0	0.936340213	
683	0.15	0.16	27.63000131	11.84	0	Cytochrome c oxidase subunit 6C-2 OS=Rattus norvegicus GN=Cox6c2 PE=1 SV=3	RAT	0	1.033663034	0.795193672
684	0.15	0.15	32.76999891	0.504700001	0	Teneurin-2 OS=Rattus norvegicus GN=Odz2 PE=2 SV=2	RAT	0	0.99177289	0.947434902
685	0.15	0.15	36.01999879	1.200999971	0	Torsin-1A-interacting protein 1 OS=Rattus norvegicus GN=Tor1aip1 PE=1 SV=1	RAT	0	0.928725302	0.592167377
686	0.15	0.15	45.82000077	1.772000082	0	ADP-ribosylation factor-like protein 13A OS=Rattus norvegicus GN=Arl13a PE=2 SV=1	RAT	0	0.910989225	
686	0	0.15	18.71999949	2.978999913	0	REVERSED RAD9, RAD1, HUS1-interacting nuclear orphan protein OS=Rattus norvegicus GN=Rhino PE=2 SV=1	RAT	0		
687	0.15	0.15	25.99999905	2.127999999	0	Mannose-6-phosphate isomerase OS=Rattus norvegicus GN=Mpi PE=2 SV=1	RAT	0	0.708447218	
688	0.15	0.15	11.99999973	1.71399992	0	Protein FAM118B OS=Rattus norvegicus GN=Fam118b PE=2 SV=1	RAT	0	0.880357981	
689	0.15	0.15	30.16000092	11.11000031	0	Histidine triad nucleotide-binding protein 1 OS=Rattus norvegicus GN=Hint1 PE=1 SV=5	RAT	0	0.985191822	
690	0.14	2.22	72.7699995	12.21000031	5.164000019	Ras-related protein Rab-4B OS=Rattus norvegicus GN=Rab4b PE=2 SV=1	RAT	1	0.949697971	
691	0.14	0.4	28.09999883	1.879999973	0	REVERSED Adenylate cyclase type 4 OS=Rattus norvegicus GN=Adcy4 PE=2 SV=1	RAT	0	1.109190941	
692	0.14	0.22	37.99000084	0.337500009	0	REVERSED Striated muscle-specific serine/threonine-protein kinase OS=Rattus norvegicus GN=Speg PE=2 SV=2	RAT	0	1.058812976	0.557240784
693	0.14	0.19	42.89000034	1.98800005	0	REVERSED Ras GTPase-activating protein SynGAP OS=Rattus norvegicus GN=Syngap1 PE=1 SV=2	RAT	0	0.937816799	0.633383811
694	0.14	0.18	34.92000103	0.712599978	0	Myosin-Ia (Fragment) OS=Rattus norvegicus GN=Myo1a PE=2 SV=1	RAT	0	0.866162717	0.390320808
695	0.14	0.17	69.48000193	0	0	REVERSED Microtubule-associated protein 2 OS=Rattus norvegicus GN=Map2 PE=1 SV=3	RAT	0	1.040030003	0.528856277
696	0.14	0.17	29.49999869	2.476999909	0	REVERSED Spermatogenesis-associated protein 1 OS=Rattus norvegicus GN=Spata1 PE=2 SV=1	RAT	0	1.156013012	
697	0.14	0.17	26.33000016	0.719399983	0	Zona pellucida sperm-binding protein 2 OS=Rattus norvegicus GN=Zp2 PE=1 SV=1	RAT	0	1.098721027	
698	0.14	0.17	27.79000103	1.420000009	0	REVERSED Cysteine sulfinic acid decarboxylase OS=Rattus norvegicus GN=Csad PE=1 SV=1	RAT	0	0.915711105	0.433804691
699	0.14	0.15	8.931999654	1.164999977	0	REVERSED Fatty acyl-CoA reductase 1 OS=Rattus norvegicus GN=Far1 PE=2 SV=1	RAT	0		
700	0.14	0.14	36.10999882	1.697999984	0	Rab11 family-interacting protein 1 OS=Rattus norvegicus GN=Rab11fip1 PE=2 SV=1	RAT	0	0.902334273	0.489563495
701	0.14	0.14	35.58000028	2.404000051	0	Kelch-like ECH-associated protein 1 OS=Rattus norvegicus GN=Keap1 PE=2 SV=1	RAT	0		
702	0.14	0.14	37.13000119	1.374999993	0	Protein disulfide-isomerase OS=Rattus norvegicus GN=P4hb PE=1 SV=2	RAT	0	0.953110993	0.712060988
703	0.14	0.14	54.28000093	2.974000014	2.974000014	Signal recognition particle receptor subunit beta OS=Rattus norvegicus GN=Srprb PE=2 SV=1	RAT	1		
704	0.14	0.14	40.45999944	4.275999963	0	REVERSED DCN1-like protein 3 OS=Rattus norvegicus GN=Dcun1d3 PE=2 SV=1	RAT	0	1.083042026	
705	0.14	0.14	17.09000021	1.453000028	0	Ral guanine nucleotide dissociation stimulator OS=Rattus norvegicus GN=Ralgds PE=1 SV=1	RAT	0	1.169329047	
706	0.13	4.17	90.56000113	28.88999879	17.77999997	ADP-ribosylation factor 5 OS=Rattus norvegicus GN=Arf5 PE=1 SV=2	RAT	2	1.115383029	

707	0.13	0.38	41.94000065	0.767999981	0	REVERSED Coiled-coil domain-containing protein 81 OS=Rattus norvegicus GN=Ccdc81 PE=2 SV=1	RAT	0	1.009246945	0.936488211
708	0.13	0.24	59.03999805	0	0	REVERSED Cytoplasmic dynein 1 heavy chain 1 OS=Rattus norvegicus GN=Dync1h1 PE=1 SV=1	RAT	0	0.941365719	0.597374976
709	0.13	0.24	29.96999919	0.333900005	0	REVERSED Potassium/sodium hyperpolarization-activated cyclic nucleotide-gated channel 4 OS=Rattus norvegicus GN=Hcn4 PE=2 SV=1	RAT	0	0.937785983	0.437854707
710	0.13	0.21	55.44000268	1.468999963	0	REVERSED Contactin-1 OS=Rattus norvegicus GN=Cntn1 PE=1 SV=2	RAT	0	0.949576378	0.592711627
711	0.13	0.2	23.97000045	1.217	0	IQ and AAA domain-containing protein 1-like OS=Rattus norvegicus GN=Iqca1p1 PE=2 SV=2	RAT	0	0.871480227	
712	0.13	0.17	32.31999874	2.208999917	0	Serine/threonine-protein kinase WNK4 OS=Rattus norvegicus GN=Wnk4 PE=2 SV=3	RAT	0	1.073099017	0.474485904
713	0.13	0.17	15.09000063	0.750700012	0	Neurogenic locus notch homolog protein 1 OS=Rattus norvegicus GN=Notch1 PE=2 SV=2	RAT	0	0.975340903	
714	0.13	0.17	42.86000133	1.706000045	0	REVERSED SHC-transforming protein 1 OS=Rattus norvegicus GN=Shc1 PE=1 SV=1	RAT	0	0.94092977	0.648904324
715	0.13	0.16	23.80000055	2.266000025	0	Heterogeneous nuclear ribonucleoprotein D0 OS=Rattus norvegicus GN=Hnrnpd PE=1 SV=1	RAT	0	1.160096049	0.478951097
716	0.13	0.16	19.63	2.094000019	0	V-type proton ATPase subunit C 1 OS=Rattus norvegicus GN=Atp6v1c1 PE=2 SV=1	RAT	0	1.022233963	0.873298407
717	0.13	0.15	26.6900003	0	0	Myosin-binding protein C, cardiac-type OS=Rattus norvegicus GN=Mybpc3 PE=2 SV=2	RAT	0	0.905247986	0.263810188
718	0.13	0.15	36.41000092	4.084999859	0	Methylcrotonoyl-CoA carboxylase beta chain, mitochondrial OS=Rattus norvegicus GN=Mccc2 PE=2 SV=1	RAT	0	1.020215988	
719	0.13	0.14	31.90999925	0.839599967	0	Structural maintenance of chromosomes protein 3 OS=Rattus norvegicus GN=Smc3 PE=1 SV=1	RAT	0	0.870744884	0.233059496
720	0.13	0.14	30.55999875	6.509999931	0	Copine-9 OS=Rattus norvegicus GN=Cpne9 PE=2 SV=1	RAT	0	1.010972977	0.962470293
721	0.13	0.13	35.17000079	0	0	Nucleolar RNA helicase 2 OS=Rattus norvegicus GN=Ddx21 PE=2 SV=1	RAT	0	0.938943684	0.640802979
722	0.13	0.13	35.83000004	1.737999916	0	Meprin A subunit alpha OS=Rattus norvegicus GN=Mep1a PE=1 SV=2	RAT	0	0.932596624	
723	0.13	0.13	21.35999948	0.784699991	0	Nitric oxide synthase, inducible OS=Rattus norvegicus GN=Nos2 PE=1 SV=2	RAT	0		
724	0.13	0.13	14.98000026	0.74260002	0	REVERSED Leucine-rich repeat-containing protein 41 OS=Rattus norvegicus GN=Lrrc41 PE=2 SV=1	RAT	0	0.955002904	
725	0.12	6.45	67.04000235	22.82000035	14.65000063	Guanine nucleotide-binding protein G(i) subunit alpha-2 OS=Rattus norvegicus GN=Gnai2 PE=1 SV=3	RAT	4	0.883865476	
726	0.12	1.65	33.50999951	13.56000006	9.573999792	Alpha-centractin OS=Rattus norvegicus GN=Actr1a PE=1 SV=1	RAT	2	1.345931053	
727	0.12	0.2	26.46999955	8.332999796	0	40S ribosomal protein S5 OS=Rattus norvegicus GN=Rps5 PE=2 SV=3	RAT	0		
728	0.12	0.18	29.19999957	0.620200019	0	REVERSED Myosin-7 OS=Rattus norvegicus GN=Myh7 PE=2 SV=2	RAT	0	1.050616026	
729	0.12	0.18	46.43000066	1.817999966	0	REVERSED Caskin-1 OS=Rattus norvegicus GN=Caskin1 PE=1 SV=1	RAT	0	1.02480495	0.79106909
730	0.12	0.16	21.78999931	0.606699986	0	Sodium channel protein type 8 subunit alpha OS=Rattus norvegicus GN=Scn8a PE=2 SV=1	RAT	0	1.065919995	0.383522213
731	0.12	0.16	19.33999956	0.875900034	0	Insulin-like growth factor 1 receptor OS=Rattus norvegicus GN=Igf1r PE=2 SV=2	RAT	0		
732	0.12	0.15	10.06999984	3.356999904	0	REVERSED Multidrug and toxin extrusion protein 1 OS=Rattus norvegicus GN=Slc47a1 PE=1 SV=1	RAT	0	1.259883046	
733	0.12	0.14	33.4100008	1.116000023	0	Myb-binding protein 1A OS=Rattus norvegicus GN=Mybbp1a PE=2 SV=2	RAT	0	1.211629987	
734	0.12	0.14	75.73999763	8.679000288	4.733999819	REVERSED Fumarate hydratase, mitochondrial OS=Rattus norvegicus GN=Fh PE=1 SV=1	RAT	1		
735	0.12	0.14	23.69000018	2.379000001	0	REVERSED Protein-tyrosine kinase 2-beta OS=Rattus norvegicus GN=Ptk2b PE=1 SV=1	RAT	0		
736	0.12	0.14	35.87000072	0	0	REVERSED Sulfhydryl oxidase 1 OS=Rattus norvegicus GN=Qsox1 PE=1 SV=1	RAT	0	0.942233682	0.546626508
737	0.12	0.14	38.94000053	4.867000133	0	Neuron-specific vesicular protein calcyon OS=Rattus norvegicus GN=Caly PE=1 SV=1	RAT	0	1.115653038	
738	0.12	0.13	29.57000136	0.982800033	0	Deubiquitinating protein VCIP135 OS=Rattus norvegicus GN=Vcpip1 PE=1 SV=2	RAT	0	0.900258422	0.324998587
739	0.12	0.13	39.01000023	3.5459999	0	Uncharacterized protein C1orf131 homolog OS=Rattus norvegicus PE=2 SV=1	RAT	0	1.069161057	
740	0.12	0.13	38.17000091	3.279000148	0	Protein FAM46B OS=Rattus norvegicus GN=Fam46b PE=2 SV=1	RAT	0		
741	0.12	0.13	24.60000068	3.162999824	0	REVERSED TBCC domain-containing protein 1 OS=Rattus norvegicus GN=Tbccd1 PE=2 SV=1	RAT	0	1.133895993	
742	0.12	0.13	11.94000021	2.070000023	0	REVERSED Nuclear receptor subfamily 4 group A member 3 OS=Rattus norvegicus GN=Nr4a3 PE=2 SV=1	RAT	0		
743	0.12	0.12	73.29999804	3.567000106	0	REVERSED Dynamin-3 OS=Rattus norvegicus GN=Dnm3 PE=1 SV=2	RAT	0	0.904226422	0.346500695
744	0.12	0.12	29.31999862	1.704999991	0	REVERSED NACHT, LRR and PYD domains-containing protein 6 OS=Rattus norvegicus GN=Nlrp6 PE=1 SV=3	RAT	0	0.914299488	0.535796285
745	0.12	0.12	24.05000031	1.631999947	0	REVERSED Sarcosine dehydrogenase, mitochondrial OS=Rattus norvegicus GN=Sardh PE=1 SV=2	RAT	0	1.006101012	0.96117419
746	0.12	0.12	22.12000042	0.611899979	0	Adenylate cyclase type 3 OS=Rattus norvegicus GN=Adcy3 PE=2 SV=1	RAT	0	0.838816285	0.326099485
747	0.12	0.12	51.33000016	4.255000129	0	Arginine/serine-rich coiled-coil protein 2 OS=Rattus norvegicus GN=Rsrc2 PE=2 SV=1	RAT	0	1.063871026	0.711457491
748	0.12	0.12	29.92999852	2.386000007	0	Coronin-1A OS=Rattus norvegicus GN=Coro1a PE=1 SV=3	RAT	0	1.140898943	0.431703091
749	0.12	0.12	36.50000095	5.353000015	0	Class E basic helix-loop-helix protein 40 OS=Rattus norvegicus GN=Bhlhe40 PE=2 SV=1	RAT	0		
750	0.12	0.12	10.66000015	0.861100014	0	REVERSED Urea transporter 2 OS=Rattus norvegicus GN=Slc14a2 PE=1 SV=2	RAT	0	1.04844296	0.724783778
751	0.12	0.12	34.799999	11.8900001	0	REVERSED Cytochrome c oxidase subunit 2 OS=Rattus norvegicus GN=Mtco2 PE=2 SV=2	RAT	0		
752	0.12	0.12	31.60000145	5.18900007	0	Peroxisomal membrane protein 4 OS=Rattus norvegicus GN=Pxmp4 PE=1 SV=2	RAT	0	0.935296178	0.567003608
753	0.12	0.12	47.72000015	2.806999907	0	REVERSED N(G),N(G)-dimethylarginine dimethylaminohydrolase 2 OS=Rattus norvegicus GN=Ddah2 PE=1 SV=1	RAT	0	0.972838223	0.827430308
754	0.11	0.4	21.86000049	0.088569999	0	REVERSED Dynein heavy chain 1, axonemal OS=Rattus norvegicus GN=Dnah1 PE=2 SV=2	RAT	0	1.034780025	0.78404671
755	0.11	0.27	39.0899986	0.7216	0	REVERSED Complement C3 OS=Rattus norvegicus GN=C3 PE=1 SV=3	RAT	0	1.068277001	0.63143158
756	0.11	0.17	34.24000144	0.953000039	0	REVERSED Synaptojanin-1 OS=Rattus norvegicus GN=Synj1 PE=1 SV=3	RAT	0	1.024324059	0.848239779
757	0.11	0.15	35.46999991	0.57529998	0	Glutamate receptor-interacting protein 2 OS=Rattus norvegicus GN=Grip2 PE=1 SV=1	RAT	0	1.074975967	0.598556876
758	0.11	0.14	26.28000081	0.320499996	0	Ral GTPase-activating protein subunit alpha-2 OS=Rattus norvegicus GN=Ralgapa2 PE=1 SV=1	RAT	0	1.007117987	
759	0.11	0.13	23.8499999	1.173999999	0	REVERSED Myosin-Ie OS=Rattus norvegicus GN=Myo1e PE=2 SV=1	RAT	0	0.943694413	0.553631723
760	0.11	0.13	31.43000007	2.040999942	0	REVERSED Dual specificity protein kinase CLK3 OS=Rattus norvegicus GN=Clk3 PE=2 SV=2	RAT	0	1.132392049	
761	0.11	0.13	42.68999994	3.880999982	0	REVERSED DNA polymerase beta OS=Rattus norvegicus GN=Polb PE=1 SV=4	RAT	0	1.146507025	0.398546398
762	0.11	0.13	33.05999935	1.365999971	0	Acylamino-acid-releasing enzyme OS=Rattus norvegicus GN=Apeh PE=1 SV=1	RAT	0	0.978416681	0.823685229
763	0.11	0.13	30.48000038	2.380999923	0	Pre-mRNA-splicing factor RBM22 OS=Rattus norvegicus GN=Rbm22 PE=2 SV=1	RAT	0	0.980933785	
764	0.11	0.12	30.55000007	0.643099984	0	REVERSED DNA mismatch repair protein Msh2 OS=Rattus norvegicus GN=Msh2 PE=2 SV=1	RAT	0		
765	0.11	0.12	33.88000131	0.952399988	0	REVERSED Serine/threonine-protein kinase BRSK2 OS=Rattus norvegicus GN=Brsk2 PE=2 SV=1	RAT	0		
765	0	0.11	44.72999871	0.899699982	0	REVERSED Serine/threonine-protein kinase BRSK1 OS=Rattus norvegicus GN=Brsk1 PE=1 SV=1	RAT	0		
766	0.11	0.12	39.80000019	3.234000131	0	REVERSED Uncharacterized protein C1orf177 homolog OS=Rattus norvegicus PE=2 SV=1	RAT	0	0.951318502	
767	0.11	0.12	29.0899992	4.431999847	0	REVERSED P2Y purinoceptor 4 OS=Rattus norvegicus GN=P2ry4 PE=2 SV=1	RAT	0	0.882854998	0.434844196
768	0.11	0.11	31.88999891	1.367999986	0	Lon protease homolog, mitochondrial OS=Rattus norvegicus GN=Lonp1 PE=2 SV=1	RAT	0		
769	0.11	0.11	32.24000037	3.64000015	0	Insulin-like growth factor 2 mRNA-binding protein 1 OS=Rattus norvegicus GN=Igf2bp1 PE=1 SV=1	RAT	0		
770	0.11	0.11	31.54999912	5.882000178	0	4-hydroxybenzoate polyprenyltransferase, mitochondrial OS=Rattus norvegicus GN=Coq2 PE=2 SV=1	RAT	0	0.897316813	
771	0.11	0.11	25.78999996	2.42999997	0	Atrial natriuretic peptide receptor 3 OS=Rattus norvegicus GN=Npr3 PE=2 SV=1	RAT	0		
772	0.11	0.11	23.63000065	2.305000089	0	REVERSED Microfibril-associated glycoprotein 3 OS=Rattus norvegicus GN=Mfap3 PE=2 SV=1	RAT	0	0.939859927	
773	0.11	0.11	30.88999987	2.703000046	0	Tonin OS=Rattus norvegicus GN=Klk2 PE=1 SV=1	RAT	0	1.175963998	
773	0	0.11	18.08999926	3.72299999	0	Glandular kallikrein-3, submandibular (Fragment) OS=Rattus norvegicus GN=Klk3 PE=2 SV=2	RAT	0		
774	0.11	0.11	47.79999852	4.402999952	0	Succinate dehydrogenase [ubiquinone] cytochrome b small subunit, mitochondrial OS=Rattus norvegicus GN=Sdhd PE=2 SV=1	RAT	0	0.935517192	0.62285161
775	0.1	0.34	29.67000008	1.779999956	0	REVERSED Pannexin-2 OS=Rattus norvegicus GN=Panx2 PE=1 SV=2	RAT	0	1.103602052	0.709703922
776	0.1	0.28	29.1200012	0	0	REVERSED Apolipoprotein B-100 OS=Rattus norvegicus GN=Apob PE=1 SV=1	RAT	0	1.011420965	0.866616189
777	0.1	0.27	26.94000006	0.172099995	0	Myomegalin OS=Rattus norvegicus GN=Pde4dip PE=1 SV=1	RAT	0	0.909329116	0.513719976
778	0.1	0.27	28.49000096	1.303999964	0	REVERSED Synaptonemal complex protein 1 OS=Rattus norvegicus GN=Sycp1 PE=2 SV=2	RAT	0		

779	0.1	0.21	28.58999968	2.563999966	0	Potassium/sodium hyperpolarization-activated cyclic nucleotide-gated channel 3 OS=Rattus norvegicus GN=Hcn3 PE=2 SV=1	RAT	0	0.964085221	
780	0.1	0.18	26.96999907	0	0	REVERSED Telomerase protein component 1 OS=Rattus norvegicus GN=Tep1 PE=1 SV=1	RAT	0	0.908815026	0.431726187
781	0.1	0.16	30.64999878	0.404699985	0	Echinoderm microtubule-associated protein-like 5 OS=Rattus norvegicus GN=Eml5 PE=1 SV=2	RAT	0	1.226462007	
782	0.1	0.15	35.85999906	0.509600015	0	REVERSED Microtubule-associated serine/threonine-protein kinase 1 OS=Rattus norvegicus GN=Mast1 PE=2 SV=1	RAT	0	0.894364715	0.526637077
783	0.1	0.14	38.49999905	0	0	Roundabout homolog 4 OS=Rattus norvegicus GN=Robo4 PE=2 SV=1	RAT	0	1.101897955	0.636708498
784	0.1	0.14	37.09999919	1.854999922	0	Fragile X mental retardation protein 1 homolog OS=Rattus norvegicus GN=Fmr1 PE=2 SV=2	RAT	0	0.854474723	
785	0.1	0.13	27.73000002	0.477200001	0	Nuclear pore membrane glycoprotein 210 OS=Rattus norvegicus GN=Pom210 PE=1 SV=1	RAT	0	0.935756981	0.625258327
786	0.1	0.13	26.94000006	0.601600017	0	Synaptojanin-2 OS=Rattus norvegicus GN=Synj2 PE=1 SV=2	RAT	0	1.126721025	
787	0.1	0.13	48.08000028	2.559999935	0	REVERSED ProSAP-interacting protein 1 OS=Rattus norvegicus GN=Prosapip1 PE=1 SV=2	RAT	0	0.797074974	0.28276071
788	0.1	0.13	24.07999933	1.840000041	0	Aspartyl-tRNA synthetase, mitochondrial OS=Rattus norvegicus GN=Dars2 PE=2 SV=1	RAT	0	1.099575043	
789	0.1	0.13	23.92999977	2.778000012	0	Zinc finger protein 672 OS=Rattus norvegicus GN=Znf672 PE=2 SV=1	RAT	0	1.11679399	
790	0.1	0.13	22.69999981	2.453999966	0	Protein phosphatase Slingshot homolog 3 OS=Rattus norvegicus GN=Ssh3 PE=2 SV=1	RAT	0	0.937383175	
791	0.1	0.12	36.41000092	0	0	Rho guanine nucleotide exchange factor 11 OS=Rattus norvegicus GN=Arhgef11 PE=1 SV=1	RAT	0	0.966867089	0.77870822
792	0.1	0.12	29.91000116	2.20199991	0	T-complex protein 1 subunit gamma OS=Rattus norvegicus GN=Cct3 PE=1 SV=1	RAT	0	0.973597825	0.854717612
793	0.1	0.12	32.85000026	1.917999983	0	Sterile alpha motif domain-containing protein 14 OS=Rattus norvegicus GN=Samd14 PE=2 SV=1	RAT	0	0.904348612	
794	0.1	0.11	19.17999983	1.099000033	0	REVERSED Phosphatidylinositol 4,5-bisphosphate 5-phosphatase A OS=Rattus norvegicus GN=Inpp5j PE=1 SV=1	RAT	0	0.949406981	0.695451319
795	0.1	0.11	22.63000011	0.729899993	0	REVERSED Basic fibroblast growth factor receptor 1 OS=Rattus norvegicus GN=Fgfr1 PE=1 SV=1	RAT	0	1.002975941	0.980896294
796	0.1	0.11	7.156000286	1.506999973	0	REVERSED Muscarinic acetylcholine receptor M5 OS=Rattus norvegicus GN=Chrm5 PE=1 SV=1	RAT	0	1.13784802	0.423552006
797	0.1	0.1	52.42999792	0	0	REVERSED Protein-glutamine gamma-glutamyltransferase K OS=Rattus norvegicus GN=Tgm1 PE=2 SV=1	RAT	0	0.883266687	0.269114405
798	0.1	0.1	18.00999939	1.401000004	0	Tyrosine-protein phosphatase non-receptor type 23 (Fragment) OS=Rattus norvegicus GN=Ptpn23 PE=1 SV=2	RAT	0	1.088392973	
799	0.1	0.1	35.67999899	2.057999931	0	REVERSED Epsin-2 OS=Rattus norvegicus GN=Epn2 PE=1 SV=1	RAT	0		
800	0.1	0.1	31.86999857	1.820999943	0	REVERSED ATP-binding cassette sub-family D member 3 OS=Rattus norvegicus GN=Abcd3 PE=1 SV=3	RAT	0	1.072540998	0.610067725
801	0.1	0.1	41.0100013	0	0	Perilipin-1 OS=Rattus norvegicus GN=Plin1 PE=1 SV=1	RAT	0	1.056522012	0.491156399
802	0.1	0.1	32.91999996	1.875000075	0	Potassium voltage-gated channel subfamily G member 2 OS=Rattus norvegicus GN=Kcng2 PE=2 SV=1	RAT	0	0.948952675	0.83158052
803	0.1	0.1	39.14000094	2.631999925	0	UPF0498 protein KIAA1191 homolog OS=Rattus norvegicus GN=Aa2-141 PE=2 SV=1	RAT	0	1.063627005	
804	0.1	0.1	36.10000014	5.393999815	0	Chloride intracellular channel protein 1 OS=Rattus norvegicus GN=Clic1 PE=1 SV=1	RAT	0	0.85410732	
805	0.1	0.1	25.49000084	4.90199998	0	REVERSED 60S ribosomal protein L15 OS=Rattus norvegicus GN=Rpl15 PE=1 SV=2	RAT	0	1.061609983	0.728277326
806	0.1	0.1	23.86000007	3.268000111	0	REVERSED Macrophage asialoglycoprotein-binding protein 1 OS=Rattus norvegicus GN=Mgl1 PE=1 SV=1	RAT	0		
807	0.1	0.1	5.49999997	3.500000015	0	REVERSED Cysteine dioxygenase type 1 OS=Rattus norvegicus GN=Cdo1 PE=1 SV=1	RAT	0		
808	0.09	1.34	28.99999917	4.408000037	2.088000067	Septin-11 OS=Rattus norvegicus GN=Sept11 PE=1 SV=1	RAT	1	1.101973057	0.53011328
809	0.09	0.51	26.48000121	0.110700005	0	Dynein heavy chain 1, axonemal OS=Rattus norvegicus GN=Dnah1 PE=2 SV=2	RAT	0	1.100250006	0.570494115
810	0.09	0.2	38.5800004	0	0	Tuberin OS=Rattus norvegicus GN=Tsc2 PE=1 SV=1	RAT	0	0.845956922	0.340128392
811	0.09	0.16	31.83000088	0.502499985	0	REVERSED Putative ATP-dependent RNA helicase DHX30 OS=Rattus norvegicus GN=Dhx30 PE=2 SV=1	RAT	0		
811	0	0.13	27.18999982	0.795799959	0	Coiled-coil domain-containing protein 164 OS=Rattus norvegicus GN=Ccdc164 PE=2 SV=1	RAT	0		
812	0.09	0.14	35.21000147	0.6329	0	Valyl-tRNA synthetase OS=Rattus norvegicus GN=Vars PE=2 SV=2	RAT	0	1.182513952	0.277787089
813	0.09	0.14	23.01999927	0.47129998	0	REVERSED Receptor-type tyrosine-protein phosphatase C OS=Rattus norvegicus GN=Ptprc PE=2 SV=2	RAT	0	0.920615911	
814	0.09	0.14	13.1400004	1.703000069	0	REVERSED Potassium channel subfamily K member 3 OS=Rattus norvegicus GN=Kcnk3 PE=2 SV=1	RAT	0		
815	0.09	0.13	29.39999998	0	0	REVERSED Coiled-coil domain-containing protein 105 OS=Rattus norvegicus GN=Ccdc105 PE=2 SV=1	RAT	0	0.906265914	0.771542311
816	0.09	0.13	24.49000031	3.265000135	0	REVERSED Collagen triple helix repeat-containing protein 1 OS=Rattus norvegicus GN=Cthrc1 PE=1 SV=1	RAT	0	0.981955171	0.886334002
817	0.09	0.12	55.1699996	0.399200013	0	REVERSED Fatty acid synthase OS=Rattus norvegicus GN=Fasn PE=1 SV=3	RAT	0	0.989496827	0.932240486
818	0.09	0.12	22.21000046	0	0	G-protein coupled receptor-associated sorting protein 1 OS=Rattus norvegicus GN=Gprasp1 PE=1 SV=1	RAT	0	1.127300024	
819	0.09	0.12	23.75999987	0.601499993	0	REVERSED Ubinuclein-2 OS=Rattus norvegicus GN=Ubn2 PE=3 SV=1	RAT	0	0.843946576	
820	0.09	0.12	19.30000037	1.228000037	0	Interleukin-1 receptor accessory protein OS=Rattus norvegicus GN=Il1rap PE=2 SV=1	RAT	0	1.266649008	
821	0.09	0.12	37.95000017	9.069000185	0	REVERSED Carboxypeptidase A1 OS=Rattus norvegicus GN=Cpa1 PE=2 SV=2	RAT	0		
822	0.09	0.12	34.58999991	6.917999685	0	Solute carrier family 25 member 42 OS=Rattus norvegicus GN=Slc25a42 PE=2 SV=1	RAT	0	0.943865478	
823	0.09	0.11	19.74000037	0	0	Low-density lipoprotein receptor-related protein 2 OS=Rattus norvegicus GN=Lrp2 PE=1 SV=1	RAT	0	1.042883992	0.600101829
824	0.09	0.11	32.39000142	0	0	REVERSED Thyroid hormone receptor-associated protein 3 OS=Rattus norvegicus GN=Thrap3 PE=1 SV=1	RAT	0	1.014871955	0.867271185
825	0.09	0.11	46.81000113	0	0	Coiled-coil domain-containing protein 8 OS=Rattus norvegicus GN=Ccdc8 PE=2 SV=1	RAT	0	1.128422022	0.278257102
826	0.09	0.11	38.19999993	1.283999998	0	Leucine-rich repeat, immunoglobulin-like domain and transmembrane domain-containing protein 1 OS=Rattus norvegicus GN=Lrit1 PE=1 SV=2	RAT	0	0.878375471	
827	0.09	0.11	28.18000019	1.083999965	0	REVERSED Dipeptidyl peptidase 3 OS=Rattus norvegicus GN=Dpp3 PE=1 SV=2	RAT	0	0.976960182	0.875848711
828	0.09	0.11	8.615999669	0.753299985	0	REVERSED Aggrecan core protein OS=Rattus norvegicus GN=Acan PE=1 SV=2	RAT	0	1.064687014	0.640857279
829	0.09	0.11	32.39000142	1.408000011	0	REVERSED Angiopoietin-1 OS=Rattus norvegicus GN=Angpt1 PE=2 SV=2	RAT	0	0.958992779	
830	0.09	0.1	25.99999905	0.705899997	0	REVERSED Coiled-coil and C2 domain-containing protein 1B OS=Rattus norvegicus GN=Cc2d1b PE=2 SV=2	RAT	0	0.960086286	
831	0.09	0.1	31.52999878	1.083000004	0	Vascular cell adhesion protein 1 OS=Rattus norvegicus GN=Vcam1 PE=2 SV=1	RAT	0	0.912550271	
832	0.09	0.1	42.64999926	0	0	60S ribosomal protein L15 OS=Rattus norvegicus GN=Rpl15 PE=1 SV=2	RAT	0	1.10216701	0.521663785
833	0.09	0.1	20.0000003	3.158000112	0	Flap endonuclease 1 OS=Rattus norvegicus GN=Fen1 PE=2 SV=1	RAT	0	1.001888037	
834	0.09	0.1	67.69999862	10.11999995	0	REVERSED Ras-related protein Rab-26 OS=Rattus norvegicus GN=Rab26 PE=2 SV=2	RAT	0	0.938253224	
835	0.09	0.1	24.60999936	1.733000018	0	Zinc finger with UFM1-specific peptidase domain protein OS=Rattus norvegicus GN=Zufsp PE=2 SV=1	RAT	0	0.937377274	
836	0.09	0.09	31.07999861	0.944100041	0	REVERSED Neogenin (Fragment) OS=Rattus norvegicus GN=Neo1 PE=2 SV=1	RAT	0	1.093505025	
837	0.09	0.09	27.9399991	1.209999993	0	Peripheral plasma membrane protein CASK OS=Rattus norvegicus GN=Cask PE=1 SV=1	RAT	0	0.988344371	0.930015385
838	0.09	0.09	29.98999953	0.590300001	0	A-kinase anchor protein 4 OS=Rattus norvegicus GN=Akap4 PE=1 SV=1	RAT	0	1.012874007	0.918198407
839	0.09	0.09	29.8299998	1.255999971	0	REVERSED ATP-dependent DNA helicase PIF1 OS=Rattus norvegicus GN=Pif1 PE=2 SV=1	RAT	0	1.031046987	0.810341597
840	0.09	0.09	30.12999892	4.675000161	0	REVERSED Coiled-coil domain-containing protein 130 OS=Rattus norvegicus GN=Ccdc130 PE=2 SV=1	RAT	0	1.412430048	
841	0.09	0.09	29.2899996	2.31199991	0	Carcinoembryonic antigen-related cell adhesion molecule 1 OS=Rattus norvegicus GN=Ceacam1 PE=1 SV=3	RAT	0	0.843399286	
842	0.09	0.09	18.81999969	0	0	Methyltransferase-like protein 6 OS=Rattus norvegicus GN=Mettl6 PE=2 SV=1	RAT	0	0.97746098	0.868067026
843	0.09	0.09	21.78999931	0	0	REVERSED MAP kinase-interacting serine/threonine-protein kinase 1 OS=Rattus norvegicus GN=Mknk1 PE=2 SV=1	RAT	0	0.96424669	0.809206307
844	0.09	0.09	38.06000054	4.515999928	0	NAD-dependent deacetylase sirtuin-5 OS=Rattus norvegicus GN=Sirt5 PE=2 SV=1	RAT	0	0.760806322	
845	0.09	0.09	48.48000109	6.818000227	0	40S ribosomal protein S12 OS=Rattus norvegicus GN=Rps12 PE=1 SV=2	RAT	0	1.034016013	
846	0.09	0.09	20.29999942	2.00500004	0	LanC-like protein 1 OS=Rattus norvegicus GN=Lancl1 PE=2 SV=2	RAT	0	1.077345967	
847	0.09	0.09	16.30000025	3.762000054	0	Dehydrogenase/reductase SDR family member 9 OS=Rattus norvegicus GN=Dhrs9 PE=2 SV=1	RAT	0	0.985899687	0.92581588
848	0.09	0.09	12.25999966	4.515999928	0	28S ribosomal protein S10, mitochondrial OS=Rattus norvegicus GN=Mrps10 PE=2 SV=1	RAT	0	1.199990988	
849	0.08	2.3	57.92999864	0.713900011	0.713900011	Myosin-9 OS=Rattus norvegicus GN=Myh9 PE=1 SV=3	RAT	2	1.042691946	0.607923329
850	0.08	0.91	15.32000005	3.153000027	1.350999996	Tryptophan 5-hydroxylase 1 OS=Rattus norvegicus GN=Tph1 PE=2 SV=2	RAT	1		
851	0.08	0.28	35.85000038	0.711200014	0	ProSAP-interacting protein 1 OS=Rattus norvegicus GN=Prosapip1 PE=1 SV=2	RAT	0	1.13449502	0.666056395

852	0.08	0.26	26.33999884	0.20620001	0	Myosin-3 OS=Rattus norvegicus GN=Myh3 PE=2 SV=1	RAT	0	0.804342806	
853	0.08	0.26	25.42999983	0.344599993	0	Fanconi anemia group D2 protein homolog OS=Rattus norvegicus GN=Fancd2 PE=2 SV=2	RAT	0	1.008486986	0.912818074
854	0.08	0.21	54.44999933	0.202400004	0	REVERSED Myosin-10 OS=Rattus norvegicus GN=Myh10 PE=1 SV=1	RAT	0	0.98394382	0.860022902
855	0.08	0.16	29.31999862	0	0	REVERSED Nitric oxide synthase, brain OS=Rattus norvegicus GN=Nos1 PE=1 SV=1	RAT	0	1.156177998	0.452754498
856	0.08	0.15	20.8100006	0	0	Zinc finger FYVE domain-containing protein 26 OS=Rattus norvegicus GN=Zfyve26 PE=3 SV=1	RAT	0	0.949503124	0.554883778
857	0.08	0.15	31.38000071	0	0	REVERSED WD repeat-containing protein 6 OS=Rattus norvegicus GN=Wdr6 PE=1 SV=2	RAT	0	1.012004972	0.949957073
858	0.08	0.13	32.73999989	1.889000088	0	Coatomer subunit beta OS=Rattus norvegicus GN=Copb1 PE=1 SV=1	RAT	0	1.03045404	
859	0.08	0.13	34.43999887	3.641999885	0	Deoxyhypusine hydroxylase OS=Rattus norvegicus GN=Dohh PE=2 SV=1	RAT	0		
860	0.08	0.12	60.89000106	1.544999983	0	REVERSED Cullin-associated NEDD8-dissociated protein 1 OS=Rattus norvegicus GN=Cand1 PE=1 SV=1	RAT	0	0.932328522	0.764355421
861	0.08	0.12	34.18000042	0	0	SNF-related serine/threonine-protein kinase OS=Rattus norvegicus GN=Snrk PE=1 SV=1	RAT	0	1.02895999	0.767653823
862	0.08	0.11	24.61999953	0.71109999	0	Telomerase reverse transcriptase OS=Rattus norvegicus GN=Tert PE=2 SV=1	RAT	0		
863	0.08	0.11	26.5899986	2.624999918	0	REVERSED Sodium/potassium/calcium exchanger 1 OS=Rattus norvegicus GN=Slc24a1 PE=2 SV=1	RAT	0	1.012585998	
864	0.08	0.11	43.04000139	1.133000012	0	REVERSED Nuclear RNA export factor 1 OS=Rattus norvegicus GN=Nxf1 PE=2 SV=1	RAT	0	0.882005095	0.445828497
865	0.08	0.11	21.78000063	5.940999836	0	Endothelin-1 OS=Rattus norvegicus GN=Edn1 PE=1 SV=2	RAT	0	1.074633956	
866	0.08	0.1	25.76000094	0	0	REVERSED Rab3 GTPase-activating protein non-catalytic subunit OS=Rattus norvegicus GN=Rab3gap2 PE=1 SV=2	RAT	0	0.936623514	0.636633694
867	0.08	0.1	28.36999893	0.446800003	0	REVERSED Vascular endothelial growth factor receptor 2 OS=Rattus norvegicus GN=Kdr PE=1 SV=1	RAT	0	0.937941074	
868	0.08	0.1	28.79999876	1.178000029	0	REVERSED Transient receptor potential cation channel subfamily M member 8 OS=Rattus norvegicus GN=Trpm8 PE=2 SV=4	RAT	0	0.996038675	0.966410518
869	0.08	0.1	26.76999867	1.114999969	0	N-terminal kinase-like protein OS=Rattus norvegicus GN=Scyl1 PE=2 SV=1	RAT	0		
870	0.08	0.1	34.2200011	0	0	Cytochrome P450 2F2 OS=Rattus norvegicus GN=Cyp2f2 PE=2 SV=1	RAT	0	0.911875188	0.521895707
871	0.08	0.1	22.34999985	2.535000071	0	ATP-sensitive inward rectifier potassium channel 14 OS=Rattus norvegicus GN=Kcnj14 PE=1 SV=1	RAT	0	1.030747056	
872	0.08	0.09	30.30000031	1.059999969	0	REVERSED Tyrosine-protein kinase JAK2 OS=Rattus norvegicus GN=Jak2 PE=2 SV=1	RAT	0	1.258103967	
873	0.08	0.09	32.69000053	0	0	Dual serine/threonine and tyrosine protein kinase OS=Rattus norvegicus GN=Dstyk PE=2 SV=1	RAT	0	0.957795382	0.689588487
874	0.08	0.09	30.05000055	0.930900034	0	REVERSED ATP-binding cassette sub-family B member 7, mitochondrial OS=Rattus norvegicus GN=Abcb7 PE=2 SV=1	RAT	0	1.031569004	
875	0.08	0.09	20.53000033	0	0	Epithelial splicing regulatory protein 2 OS=Rattus norvegicus GN=Esrp2 PE=2 SV=1	RAT	0	0.987643182	0.920341492
876	0.08	0.09	56.98999763	6.735999882	0	Protein-tyrosine phosphatase mitochondrial 1 OS=Rattus norvegicus GN=Ptpmt1 PE=2 SV=1	RAT	0	1.030925035	
877	0.08	0.09	24.25000072	2.65800003	0	REVERSED Ribonuclease H2 subunit A OS=Rattus norvegicus GN=Rnaseh2a PE=2 SV=1	RAT	0	1.067968011	0.631038189
878	0.08	0.09	17.64999926	17.64999926	0	REVERSED Ubiquitin-fold modifier 1 OS=Rattus norvegicus GN=Ufm1 PE=2 SV=1	RAT	0	1.057863951	
879	0.08	0.08	20.99999934	0	0	REVERSED Reelin OS=Rattus norvegicus GN=Reln PE=2 SV=1	RAT	0	1.015215993	0.823320925
880	0.08	0.08	39.86999989	0	0	REVERSED Melanoma-associated antigen D1 OS=Rattus norvegicus GN=Maged1 PE=1 SV=3	RAT	0	1.024317026	0.832526326
881	0.08	0.08	20.35000026	0.413599983	0	Calcium-activated potassium channel subunit alpha-1 OS=Rattus norvegicus GN=Kcnma1 PE=1 SV=3	RAT	0	0.924718022	
882	0.08	0.08	38.58999908	5.454999954	0	REVERSED Potassium voltage-gated channel subfamily A member 1 OS=Rattus norvegicus GN=Kcna1 PE=1 SV=1	RAT	0	1.002213955	0.985776782
883	0.08	0.08	19.26999986	1.389000006	0	MAGUK p55 subfamily member 7 OS=Rattus norvegicus GN=Mpp7 PE=2 SV=2	RAT	0	0.891111016	
884	0.08	0.08	41.62999988	0	0	REVERSED Serine/arginine-rich splicing factor 2 OS=Rattus norvegicus GN=Srsf2 PE=1 SV=3	RAT	0	0.84974283	0.364911407
885	0.08	0.08	20.52000016	1.434999984	0	Long-chain-fatty-acid--CoA ligase 6 OS=Rattus norvegicus GN=Acsl6 PE=1 SV=1	RAT	0	1.106973052	0.791894495
886	0.08	0.08	19.78999972	1.590000093	0	Macrophage colony-stimulating factor 1 OS=Rattus norvegicus GN=Csf1 PE=2 SV=1	RAT	0	0.924383283	
887	0.08	0.08	19.26999986	1.116999984	0	REVERSED Solute carrier organic anion transporter family member 1C1 OS=Rattus norvegicus GN=Slco1c1 PE=1 SV=1	RAT	0		
888	0.08	0.08	24.34999943	4.662999883	0	UPF0671 protein C14orf153 homolog OS=Rattus norvegicus PE=2 SV=1	RAT	0	1.020138979	
889	0.08	0.08	10.46999991	1.394999959	0	N-acylglucosamine 2-epimerase OS=Rattus norvegicus GN=Renbp PE=2 SV=2	RAT	0	1.021386981	
890	0.08	0.08	13.4800002	2.696999907	0	REVERSED Protein cereblon OS=Rattus norvegicus GN=Crbn PE=1 SV=1	RAT	0		
891	0.08	0.08	18.40000004	4.112999886	0	REVERSED Tissue alpha-L-fucosidase OS=Rattus norvegicus GN=Fuca1 PE=1 SV=1	RAT	0	1.041308999	
892	0.08	0.08	22.73000032	12.12000027	0	Stress-associated endoplasmic reticulum protein 1 OS=Rattus norvegicus GN=Serp1 PE=1 SV=2	RAT	0	0.876230299	
893	0.07	0.35	35.29999852	0.641799998	0	REVERSED Ribosome-releasing factor 2, mitochondrial OS=Rattus norvegicus GN=Gfm2 PE=2 SV=2	RAT	0	0.919622481	0.431159496
894	0.07	0.33	36.05999947	3.189999983	0	Centlein (Fragment) OS=Rattus norvegicus GN=Cntln PE=2 SV=1	RAT	0		
895	0.07	0.29	25.83000064	4.504999891	0	L-asparaginase OS=Rattus norvegicus GN=Asrgl1 PE=1 SV=1	RAT	0	0.990896881	
896	0.07	0.21	28.2799989	0.202000001	0	Myosin-IXb OS=Rattus norvegicus GN=Myo9b PE=1 SV=1	RAT	0	1.15004003	0.420395106
897	0.07	0.18	18.29999983	1.559999958	0	Dynein intermediate chain 1, axonemal OS=Rattus norvegicus GN=Dnai1 PE=2 SV=1	RAT	0		
898	0.07	0.18	11.16999984	6.145000085	0	REVERSED Ubiquinone biosynthesis protein COQ7 homolog (Fragment) OS=Rattus norvegicus GN=Coq7 PE=2 SV=3	RAT	0	0.975473523	
899	0.07	0.17	33.59000087	0	0	REVERSED Myosin-IXa OS=Rattus norvegicus GN=Myo9a PE=1 SV=1	RAT	0	1.003873944	0.967166483
900	0.07	0.16	57.77000189	0.351199997	0	REVERSED Solute carrier family 12 member 5 OS=Rattus norvegicus GN=Slc12a5 PE=1 SV=2	RAT	0	0.952989697	0.677662313
901	0.07	0.16	38.9200002	0	0	E3 ubiquitin-protein ligase BRE1B OS=Rattus norvegicus GN=Rnf40 PE=1 SV=1	RAT	0	0.855245829	0.193949595
902	0.07	0.16	42.41000116	0.627399981	0	REVERSED MAP/microtubule affinity-regulating kinase 3 OS=Rattus norvegicus GN=Mark3 PE=2 SV=1	RAT	0	0.911232173	0.483372509
903	0.07	0.16	23.92999977	3.31799984	0	REVERSED DNA (cytosine-5)-methyltransferase 3-like OS=Rattus norvegicus GN=Dnmt3l PE=2 SV=1	RAT	0	1.034139037	
904	0.07	0.15	25.31000078	0.691600004	0	Voltage-dependent L-type calcium channel subunit alpha-1C OS=Rattus norvegicus GN=Cacna1c PE=1 SV=1	RAT	0	0.942739189	
905	0.07	0.14	25.33999979	0	0	Serine/threonine-protein kinase TAO2 OS=Rattus norvegicus GN=Taok2 PE=1 SV=1	RAT	0	1.136072993	0.423780203
906	0.07	0.14	28.0099988	1.851999946	0	REVERSED Adenosylhomocysteinase OS=Rattus norvegicus GN=Ahcy PE=1 SV=3	RAT	0		
907	0.07	0.13	30.75000048	0	0	Zinc finger RNA-binding protein OS=Rattus norvegicus GN=Zfr PE=1 SV=2	RAT	0	0.807824075	0.556643307
908	0.07	0.13	27.75000036	0	0	Protein midA homolog, mitochondrial OS=Rattus norvegicus PE=2 SV=1	RAT	0	0.952883482	0.733290315
909	0.07	0.12	30.07999957	0.723099988	0	REVERSED Insulin receptor OS=Rattus norvegicus GN=Insr PE=1 SV=1	RAT	0	1.013355017	0.918734908
910	0.07	0.12	65.71000218	2.449000068	0	REVERSED 14-3-3 protein theta OS=Rattus norvegicus GN=Ywhaq PE=1 SV=1	RAT	0	1.074633956	
911	0.07	0.11	26.28000081	0	0	Ubiquitin-like modifier-activating enzyme 1 OS=Rattus norvegicus GN=Uba1 PE=1 SV=1	RAT	0	0.946748912	0.673630595
912	0.07	0.11	18.86000037	0.948399957	0	MAM domain-containing glycosylphosphatidylinositol anchor protein 2 OS=Rattus norvegicus GN=Mdga2 PE=2 SV=1	RAT	0	1.236675978	
913	0.07	0.1	20.73999941	0.617300021	0	Sushi, von Willebrand factor type A, EGF and pentraxin domain-containing protein 1 OS=Rattus norvegicus GN=Svep1 PE=1 SV=1	RAT	0	1.126409054	0.476871699
914	0.07	0.1	32.13	0.733300019	0	REVERSED Carbamoyl-phosphate synthase [ammonia], mitochondrial OS=Rattus norvegicus GN=Cps1 PE=1 SV=1	RAT	0	1.165843964	
915	0.07	0.1	71.49999738	0	0	REVERSED Dihydropyrimidinase-related protein 1 OS=Rattus norvegicus GN=Crmp1 PE=1 SV=1	RAT	0	1.087249994	0.560014307
916	0.07	0.1	35.04999876	1.027000044	0	REVERSED Radial spoke head 10 homolog B OS=Rattus norvegicus GN=Rsph10b PE=2 SV=1	RAT	0	0.661975205	
917	0.07	0.1	32.13	0	0	REVERSED Integrin alpha-D OS=Rattus norvegicus GN=Itgad PE=2 SV=1	RAT	0	0.972160995	0.778234303
918	0.07	0.1	34.09000039	1.331999991	0	Elongation factor G, mitochondrial OS=Rattus norvegicus GN=Gfm1 PE=1 SV=2	RAT	0	1.028826952	0.823513389
919	0.07	0.1	31.34999871	0	0	Kinesin-like protein KIF22 OS=Rattus norvegicus GN=Kif22 PE=2 SV=1	RAT	0	0.997869372	0.982403576
920	0.07	0.1	18.26000065	0.837500021	0	Kelch-like protein 21 OS=Rattus norvegicus GN=Klhl21 PE=3 SV=1	RAT	0	1.029870987	
921	0.07	0.09	29.78999913	0	0	PHD and RING finger domain-containing protein 1 OS=Rattus norvegicus GN=Phrf1 PE=1 SV=2	RAT	0	0.929063499	
922	0.07	0.09	39.77999985	0	0	Collagen alpha-1(I) chain OS=Rattus norvegicus GN=Col1a1 PE=1 SV=5	RAT	0	0.905160725	0.345889598
923	0.07	0.09	31.47000074	2.800999954	0	REVERSED Lysine-specific demethylase 3A OS=Rattus norvegicus GN=Kdm3a PE=2 SV=1	RAT	0	1.260579944	
924	0.07	0.09	33.73000026	0	0	Ankyrin repeat and LEM domain-containing protein 2 OS=Rattus norvegicus GN=Ankle2 PE=2 SV=1	RAT	0	1.043517947	
925	0.07	0.09	21.49000019	0	0	Protein timeless homolog OS=Rattus norvegicus GN=Timeless PE=1 SV=1	RAT	0	1.000509024	

926	0.07	0.09	27.48000026	0	0	REVERSED Phosphatidylinositol-4,5-bisphosphate 3-kinase catalytic subunit beta isoform OS=Rattus norvegicus GN=Pik3cb PE=2 SV=1	RAT	0	0.952575386	0.711727917
927	0.07	0.09	28.92999947	0	0	REVERSED Leucine-rich repeat and fibronectin type-III domain-containing protein 2 OS=Rattus norvegicus GN=Lrfn2 PE=1 SV=1	RAT	0	1.017771006	0.91003418
928	0.07	0.09	45.26999891	0	0	REVERSED Glycerol-3-phosphate dehydrogenase [NAD+], cytoplasmic OS=Rattus norvegicus GN=Gpd1 PE=1 SV=4	RAT	0	1.061243057	0.744253874
929	0.07	0.09	17.5999999	0	0	Mesothelin OS=Rattus norvegicus GN=Msln PE=2 SV=2	RAT	0	0.961652517	
930	0.07	0.09	21.05000019	2.411999926	0	REVERSED Keratin, type I cytoskeletal 12 OS=Rattus norvegicus GN=Krt12 PE=2 SV=1	RAT	0	0.945995688	0.677488506
931	0.07	0.09	24.60999936	5.468999967	0	REVERSED Lysine-rich coiled-coil protein 1 OS=Rattus norvegicus GN=Krcc1 PE=2 SV=1	RAT	0	1.011711001	
932	0.07	0.09	27.12000012	0	0	Glycine N-acyltransferase-like protein Keg1 OS=Rattus norvegicus GN=Keg1 PE=1 SV=2	RAT	0	1.115803003	
933	0.07	0.08	76.27000213	3.489999846	0	REVERSED 60 kDa heat shock protein, mitochondrial OS=Rattus norvegicus GN=Hspd1 PE=1 SV=1	RAT	0	1.03926301	0.763845026
934	0.07	0.08	26.53000057	0	0	REVERSED Breast cancer type 1 susceptibility protein homolog OS=Rattus norvegicus GN=Brca1 PE=1 SV=1	RAT	0	0.977317512	0.807542086
935	0.07	0.08	30.52999973	0.525100017	0	REVERSED Sister chromatid cohesion protein PDS5 homolog A OS=Rattus norvegicus GN=Pds5a PE=2 SV=1	RAT	0	0.895040691	0.584677398
936	0.07	0.08	33.6800009	0	0	REVERSED Potassium voltage-gated channel subfamily H member 1 OS=Rattus norvegicus GN=Kcnh1 PE=1 SV=1	RAT	0	0.860664427	0.36965251
937	0.07	0.08	36.55999899	1.343999989	0	Tripartite motif-containing protein 3 OS=Rattus norvegicus GN=Trim3 PE=1 SV=1	RAT	0	0.940406322	
938	0.07	0.08	27.5000006	0	0	Peroxisomal targeting signal 1 receptor OS=Rattus norvegicus GN=Pex5 PE=1 SV=2	RAT	0	0.946994483	0.683286428
939	0.07	0.08	22.61999995	0	0	Corepressor interacting with RBPJ 1 OS=Rattus norvegicus GN=Cir1 PE=2 SV=1	RAT	0	0.835133314	0.322498709
940	0.07	0.08	28.74000072	0	0	REVERSED Flotillin-2 OS=Rattus norvegicus GN=Flot2 PE=1 SV=1	RAT	0	1.189972043	0.171215504
941	0.07	0.08	34.70999897	0	0	Zinc transporter 1 OS=Rattus norvegicus GN=Slc30a1 PE=2 SV=1	RAT	0	0.936928093	0.629442811
942	0.07	0.08	26.28999949	1.29300002	0	Glycine receptor subunit alpha-3 OS=Rattus norvegicus GN=Glra3 PE=2 SV=1	RAT	0		
943	0.07	0.08	21.64999992	2.118000016	0	Intermediate conductance calcium-activated potassium channel protein 4 OS=Rattus norvegicus GN=Kcnn4 PE=2 SV=2	RAT	0		
944	0.07	0.08	18.00000072	0.833300035	0	DNA polymerase alpha subunit B OS=Rattus norvegicus GN=Pola2 PE=2 SV=2	RAT	0	0.981677294	
945	0.07	0.08	25.08000135	2.006999962	0	REVERSED Troponin T, cardiac muscle OS=Rattus norvegicus GN=Tnnt2 PE=1 SV=2	RAT	0		
946	0.07	0.07	84.93999839	0	0	REVERSED Synapsin-1 OS=Rattus norvegicus GN=Syn1 PE=1 SV=3	RAT	0	1.212674975	0.477102697
947	0.07	0.07	68.48999858	6.239999831	0	REVERSED Heat shock 70 kDa protein 1A/1B OS=Rattus norvegicus GN=Hspa1a PE=2 SV=2	RAT	0		
948	0.07	0.07	21.95000052	0	0	REVERSED Kinesin-like protein KIF27 OS=Rattus norvegicus GN=Kif27 PE=2 SV=1	RAT	0	1.101727009	0.363138407
949	0.07	0.07	38.35999966	3.438999876	0	1-phosphatidylinositol-4,5-bisphosphate phosphodiesterase delta-1 OS=Rattus norvegicus GN=Plcd1 PE=1 SV=1	RAT	0	1.074633956	
950	0.07	0.07	53.15999985	0	0	REVERSED Dihydroorotate dehydrogenase (quinone), mitochondrial OS=Rattus norvegicus GN=Dhodh PE=1 SV=1	RAT	0	1.029314041	0.8202914
951	0.07	0.07	26.06000006	0	0	Long-chain-fatty-acid--CoA ligase 5 OS=Rattus norvegicus GN=Acsl5 PE=2 SV=1	RAT	0	1.079437971	0.489125192
952	0.07	0.07	26.6200006	2.976999991	0	REVERSED Double-stranded RNA-binding protein Staufen homolog 2 OS=Rattus norvegicus GN=Stau2 PE=1 SV=1	RAT	0	1.022879004	0.8568964
953	0.07	0.07	57.51000047	5.750999972	0	Fumarylacetoacetate hydrolase domain-containing protein 2 OS=Rattus norvegicus GN=Fahd2 PE=1 SV=1	RAT	0		
954	0.07	0.07	19.64000016	1.785999909	0	REVERSED Death-associated protein kinase 3 OS=Rattus norvegicus GN=Dapk3 PE=1 SV=1	RAT	0		
955	0.07	0.07	26.94999874	0	0	REVERSED Serpin B10 OS=Rattus norvegicus GN=Serpinb10 PE=2 SV=1	RAT	0	1.074812055	0.472162008
956	0.07	0.07	15.57999998	0	0	REVERSED Uncharacterized protein C2orf81 homolog OS=Rattus norvegicus PE=2 SV=1	RAT	0	0.870993674	0.667004526
957	0.07	0.07	47.67000079	5.426000059	0	REVERSED Synapse differentiation-inducing gene protein 1 OS=Rattus norvegicus GN=Syndig1 PE=2 SV=1	RAT	0	0.913465023	
958	0.07	0.07	23.96000028	6.25	0	Interferon alpha-1 OS=Rattus norvegicus GN=Ifna1 PE=3 SV=1	RAT	0	0.984088302	
959	0.06	2.53	49.66000021	4.482999817	2.068999968	Dynamin-2 OS=Rattus norvegicus GN=Dnm2 PE=1 SV=1	RAT	2		
960	0.06	0.32	19.30000037	0.358700007	0	Kinesin-like protein KIF27 OS=Rattus norvegicus GN=Kif27 PE=2 SV=1	RAT	0	0.886862874	
961	0.06	0.3	24.2899999	0.547000021	0	Thyroid peroxidase OS=Rattus norvegicus GN=Tpo PE=2 SV=1	RAT	0	1.017061949	0.904926479
962	0.06	0.24	37.47000098	1.532999985	0	Carbamoyl-phosphate synthase [ammonia], mitochondrial OS=Rattus norvegicus GN=Cps1 PE=1 SV=1	RAT	0	0.927382171	
963	0.06	0.23	29.69999909	1.83600001	0	Rap guanine nucleotide exchange factor 3 OS=Rattus norvegicus GN=Rapgef3 PE=2 SV=2	RAT	0	0.92373389	0.688676119
964	0.06	0.21	26.84000134	0	0	REVERSED Dynein heavy chain 12, axonemal OS=Rattus norvegicus GN=Dnah12 PE=2 SV=2	RAT	0	0.986299217	
965	0.06	0.21	18.53999943	0.248000002	0	Transcription activator BRG1 OS=Rattus norvegicus GN=Smarca4 PE=1 SV=1	RAT	0	0.966691673	0.808457315
966	0.06	0.19	34.0200007	0.341899996	0	REVERSED Short transient receptor potential channel 2 OS=Rattus norvegicus GN=Trpc2 PE=2 SV=2	RAT	0	0.9460935	0.578216314
967	0.06	0.18	30.36000133	0	0	REVERSED Receptor-type tyrosine-protein phosphatase S OS=Rattus norvegicus GN=Ptprs PE=1 SV=2	RAT	0	1.082754016	0.432143688
968	0.06	0.18	38.02999854	0.345700001	0	Voltage-dependent calcium channel subunit alpha-2/delta-2 OS=Rattus norvegicus GN=Cacna2d2 PE=2 SV=1	RAT	0	0.949872613	0.655168712
969	0.06	0.17	72.32000232	1.47599997	0	REVERSED Arginine and glutamate-rich protein 1 OS=Rattus norvegicus GN=Arglu1 PE=2 SV=1	RAT	0	1.091871977	0.391797215
970	0.06	0.17	18.67000014	0	0	REVERSED Nuclear receptor coactivator 3 (Fragment) OS=Rattus norvegicus GN=Ncoa3 PE=2 SV=1	RAT	0	0.944574177	0.593254626
971	0.06	0.16	30.41999936	0.316899992	0	REVERSED Neurexin-3-alpha OS=Rattus norvegicus GN=Nrxn3 PE=1 SV=1	RAT	0	1.059893966	0.661417484
972	0.06	0.16	26.48000121	1.673999988	0	REVERSED Histidine ammonia-lyase OS=Rattus norvegicus GN=Hal PE=1 SV=1	RAT	0	0.958381772	0.741724491
973	0.06	0.14	67.69000292	7.69200027	0	REVERSED ProSAAS OS=Rattus norvegicus GN=Pcsk1n PE=1 SV=1	RAT	0	1.059422016	
974	0.06	0.14	27.27000117	0.712999981	0	Meckel syndrome type 1 protein homolog OS=Rattus norvegicus GN=Mks1 PE=2 SV=2	RAT	0	0.951582611	0.654952288
975	0.06	0.14	33.84000063	3.282999992	0	Macrophage erythroblast attacher OS=Rattus norvegicus GN=Maea PE=2 SV=2	RAT	0	0.978549182	
976	0.06	0.13	22.75999933	1.38600003	0	Immunoglobulin superfamily member 10 OS=Rattus norvegicus GN=Igsf10 PE=2 SV=1	RAT	0	0.942041576	
977	0.06	0.13	26.57999992	0	0	A-kinase anchor protein 6 OS=Rattus norvegicus GN=Akap6 PE=2 SV=1	RAT	0	0.87530762	0.424144804
978	0.06	0.12	24.78999943	0	0	Calcineurin-binding protein cabin-1 OS=Rattus norvegicus GN=Cabin1 PE=1 SV=1	RAT	0	1.006597042	0.935014606
979	0.06	0.11	31.36999905	0	0	REVERSED Rho guanine nucleotide exchange factor 2 OS=Rattus norvegicus GN=Arhgef2 PE=2 SV=1	RAT	0	1.097643018	0.530187011
980	0.06	0.11	42.96999872	0	0	REVERSED Coiled-coil domain-containing protein 61 OS=Rattus norvegicus GN=Ccdc61 PE=2 SV=1	RAT	0	1.103886008	0.50159049
981	0.06	0.11	41.13000035	0	0	Egl nine homolog 3, mitochondrial OS=Rattus norvegicus GN=Egln3 PE=1 SV=1	RAT	0	0.983099401	0.854447424
982	0.06	0.1	29.03999984	0	0	Huntingtin OS=Rattus norvegicus GN=Htt PE=1 SV=1	RAT	0	1.198724031	0.08124309
983	0.06	0.1	34.20000076	0	0	Kinesin-like protein KIF1B OS=Rattus norvegicus GN=Kif1b PE=1 SV=2	RAT	0	1.052850008	0.594896078
984	0.06	0.1	35.83999872	0	0	REVERSED Disabled homolog 2-interacting protein OS=Rattus norvegicus GN=Dab2ip PE=1 SV=1	RAT	0	1.163359046	0.375344306
985	0.06	0.1	28.76000106	1.042999979	0	Spermatid perinuclear RNA-binding protein OS=Rattus norvegicus GN=Strbp PE=1 SV=1	RAT	0		
986	0.06	0.1	20.74999958	1.494999975	0	Growth factor receptor-bound protein 7 OS=Rattus norvegicus GN=Grb7 PE=1 SV=1	RAT	0	0.903288901	
987	0.06	0.09	30.50000072	0	0	REVERSED RNA polymerase II-associated protein 1 OS=Rattus norvegicus GN=Rpap1 PE=2 SV=1	RAT	0	0.976603329	0.809685171
988	0.06	0.09	29.69999909	0	0	REVERSED Kinesin-1 heavy chain OS=Rattus norvegicus GN=Kif5b PE=2 SV=1	RAT	0	1.027143002	0.831984699
989	0.06	0.09	21.33000046	1.243999973	0	REVERSED Telomerase reverse transcriptase OS=Rattus norvegicus GN=Tert PE=2 SV=1	RAT	0	0.904485106	
990	0.06	0.09	28.85999978	1.023999974	0	REVERSED Disks large-associated protein 3 OS=Rattus norvegicus GN=Dlgap3 PE=1 SV=2	RAT	0	0.968433619	
991	0.06	0.09	27.9399991	2.205999941	0	Usher syndrome type-1C protein-binding protein 1 OS=Rattus norvegicus GN=Ushbp1 PE=2 SV=1	RAT	0		
992	0.06	0.09	25.94000101	2.786999941	0	REVERSED Phospholipase D2 OS=Rattus norvegicus GN=Pld2 PE=1 SV=2	RAT	0	1.133334041	
993	0.06	0.09	63.73999715	4.944999889	0	Uncharacterized protein C8orf42 homolog OS=Rattus norvegicus PE=2 SV=2	RAT	0	1.61886096	
994	0.06	0.09	28.81000042	4.70900014	0	Porphobilinogen deaminase OS=Rattus norvegicus GN=Hmbs PE=1 SV=2	RAT	0	0.850988686	
995	0.06	0.08	36.66000068	0	0	Adenomatous polyposis coli protein OS=Rattus norvegicus GN=Apc PE=1 SV=1	RAT	0	1.093783975	0.4683218
996	0.06	0.08	34.4900012	0	0	REVERSED SH3 and multiple ankyrin repeat domains protein 3 OS=Rattus norvegicus GN=Shank3 PE=1 SV=2	RAT	0	1.008283973	0.946999073
997	0.06	0.08	29.67999876	0	0	REVERSED Tuberin OS=Rattus norvegicus GN=Tsc2 PE=1 SV=1	RAT	0	1.014695048	0.876083076
998	0.06	0.08	26.8900007	0	0	REVERSED Anion exchange protein 3 OS=Rattus norvegicus GN=Slc4a3 PE=2 SV=1	RAT	0	1.07268095	
999	0.06	0.08	33.16000104	0	0	Receptor-type tyrosine-protein phosphatase-like N OS=Rattus norvegicus GN=Ptprn PE=2 SV=2	RAT	0	0.892987013	0.570584178

1000	0.06	0.08	45.82999945	0	0	Apolipoprotein E OS=Rattus norvegicus GN=Apoe PE=1 SV=2	RAT	0	1.128072023	0.444891989
1001	0.06	0.08	18.42000037	0	0	Interphotoreceptor matrix proteoglycan 1 OS=Rattus norvegicus GN=Impg1 PE=2 SV=1	RAT	0	0.910070002	0.785086513
1002	0.06	0.08	23.42000008	1.688000001	0	Abhydrolase domain-containing protein 16B OS=Rattus norvegicus GN=Abhd16b PE=2 SV=1	RAT	0	0.943732619	
1003	0.06	0.08	14.18000013	0	0	REVERSED Chaperone activity of bc1 complex-like, mitochondrial OS=Rattus norvegicus GN=Adck3 PE=2 SV=1	RAT	0	0.956458688	0.682883322
1004	0.06	0.08	33.61999989	2.029000036	0	Coiled-coil domain-containing protein KIAA1826 homolog OS=Rattus norvegicus PE=2 SV=1	RAT	0	0.973076224	0.828513682
1005	0.06	0.07	67.77999997	0	0	REVERSED Myosin-Va OS=Rattus norvegicus GN=Myo5a PE=2 SV=1	RAT	0	1.074398994	0.601795197
1006	0.06	0.07	34.09999907	0	0	REVERSED Cadherin EGF LAG seven-pass G-type receptor 2 (Fragment) OS=Rattus norvegicus GN=Celsr2 PE=2 SV=1	RAT	0	1.180924058	
1007	0.06	0.07	26.3500005	0	0	REVERSED Canalicular multispecific organic anion transporter 1 OS=Rattus norvegicus GN=Abcc2 PE=2 SV=1	RAT	0	0.901370287	0.56735009
1008	0.06	0.07	33.98000002	0	0	Centrosomal protein of 95 kDa OS=Rattus norvegicus GN=Cep95 PE=2 SV=1	RAT	0	1.108829021	
1009	0.06	0.07	31.86999857	0	0	REVERSED Ribosome biogenesis protein BOP1 OS=Rattus norvegicus GN=Bop1 PE=2 SV=1	RAT	0	1.150979042	0.397821993
1010	0.06	0.07	41.40999913	0	0	Kynurenine/alpha-aminoadipate aminotransferase, mitochondrial OS=Rattus norvegicus GN=Aadat PE=1 SV=1	RAT	0	1.065809965	0.518042624
1011	0.06	0.07	37.00000048	2.250000089	0	REVERSED Sphingosine 1-phosphate receptor 5 OS=Rattus norvegicus GN=S1pr5 PE=2 SV=1	RAT	0	0.785014629	
1012	0.06	0.07	28.97999883	1.769999973	0	Caspase-2 OS=Rattus norvegicus GN=Casp2 PE=2 SV=3	RAT	0	0.963306725	
1013	0.06	0.07	20.67999989	0	0	Ectonucleotide pyrophosphatase/phosphodiesterase family member 6 OS=Rattus norvegicus GN=Enpp6 PE=2 SV=1	RAT	0	1.055928946	0.648006022
1014	0.06	0.07	36.77000105	5.161000043	0	REVERSED 28S ribosomal protein S10, mitochondrial OS=Rattus norvegicus GN=Mrps10 PE=2 SV=1	RAT	0	1.150341988	
1015	0.06	0.07	39.01999891	0	0	REVERSED Bcl2 antagonist of cell death OS=Rattus norvegicus GN=Bad PE=1 SV=2	RAT	0	0.993915498	
1016	0.06	0.07	9.649000317	2.129999921	0	REVERSED PR domain zinc finger protein 4 OS=Rattus norvegicus GN=Prdm4 PE=2 SV=1	RAT	0	0.806769729	0.364742786
1017	0.06	0.07	5.234000087	1.652999967	0	Glycoprotein-N-acetylgalactosamine 3-beta-galactosyltransferase 1 OS=Rattus norvegicus GN=C1galt1 PE=1 SV=1	RAT	0	1.111202955	
1018	0.06	0.06	41.83999896	0.933100004	0	REVERSED CLIP-associating protein 2 OS=Rattus norvegicus GN=Clasp2 PE=2 SV=1	RAT	0	0.90899092	
1019	0.06	0.06	32.49999881	0	0	Multidrug resistance protein 1 OS=Rattus norvegicus GN=Abcb1 PE=2 SV=1	RAT	0	0.914269328	
1020	0.06	0.06	35.13000011	1.107000001	0	TRMT1-like protein OS=Rattus norvegicus GN=Trmt1l PE=2 SV=1	RAT	0	0.947221994	
1021	0.06	0.06	27.41000056	0	0	REVERSED Xylosyltransferase 1 (Fragment) OS=Rattus norvegicus GN=Xylt1 PE=2 SV=1	RAT	0	0.976697505	0.864278018
1022	0.06	0.06	46.12999856	0	0	REVERSED Mitochondria-eating protein OS=Rattus norvegicus GN=Spata18 PE=2 SV=1	RAT	0	0.853914797	0.360822707
1023	0.06	0.06	52.24000216	6.089999899	0	Basal cell adhesion molecule OS=Rattus norvegicus GN=Bcam PE=2 SV=1	RAT	0	1.035372019	
1024	0.06	0.06	27.52000093	1.455000043	0	5E5 antigen OS=Rattus norvegicus PE=2 SV=1	RAT	0		
1025	0.06	0.06	34.97000039	0	0	REVERSED Cytosolic phospholipase A2 OS=Rattus norvegicus GN=Pla2g4a PE=2 SV=1	RAT	0	1.172513962	0.354939312
1026	0.06	0.06	22.48000056	0	0	E3 ubiquitin-protein ligase UHRF1 OS=Rattus norvegicus GN=Uhrf1 PE=2 SV=2	RAT	0		
1027	0.06	0.06	30.68000078	0	0	Probable G-protein coupled receptor 37 OS=Rattus norvegicus GN=Gpr37 PE=2 SV=1	RAT	0	1.123551011	0.45500499
1028	0.06	0.06	22.72000015	1.69699993	0	Protein FAM5C OS=Rattus norvegicus GN=Fam5c PE=2 SV=1	RAT	0	1.040824056	
1029	0.06	0.06	47.47999907	0	0	REVERSED Na(+)/H(+) exchange regulatory cofactor NHE-RF2 OS=Rattus norvegicus GN=Slc9a3r2 PE=1 SV=1	RAT	0	0.862437189	0.379925013
1030	0.06	0.06	32.85999894	1.059999969	0	Beta,beta-carotene 15,15'-monooxygenase OS=Rattus norvegicus GN=Bcmo1 PE=2 SV=1	RAT	0	0.897601128	0.489659905
1031	0.06	0.06	17.79000014	1.252999995	0	Cold shock domain-containing protein E1 OS=Rattus norvegicus GN=Csde1 PE=2 SV=1	RAT	0	0.981677294	
1032	0.06	0.06	31.67999983	0	0	REVERSED Heterogeneous nuclear ribonucleoprotein D-like OS=Rattus norvegicus GN=Hnrpdl PE=2 SV=1	RAT	0	0.992099822	0.948658526
1033	0.06	0.06	32.13	0	0	REVERSED CMP-N-acetylneuraminate-beta-galactosamide-alpha-2,3-sialyltransferase 4 OS=Rattus norvegicus GN=St3gal4 PE=2 SV=1	RAT	0	0.966092229	0.672733188
1034	0.06	0.06	22.80000001	4.560000077	0	REVERSED Thymidylate synthase OS=Rattus norvegicus GN=Tyms PE=1 SV=1	RAT	0	0.861804187	
1035	0.06	0.06	30.48999906	2.845999971	0	REVERSED Proteasome subunit alpha type-6 OS=Rattus norvegicus GN=Psma6 PE=1 SV=1	RAT	0	0.888889372	
1036	0.06	0.06	21.10999972	3.055999987	0	REVERSED Inward rectifier potassium channel 13 OS=Rattus norvegicus GN=Kcnj13 PE=2 SV=1	RAT	0		
1037	0.06	0.06	20.58999985	7.353000343	0	60S ribosomal protein L27 OS=Rattus norvegicus GN=Rpl27 PE=2 SV=2	RAT	0	0.991199791	
1038	0.06	0.06	20.0000003	0	0	REVERSED Deoxycytidine kinase OS=Rattus norvegicus GN=Dck PE=2 SV=1	RAT	0	1.188436985	0.332186401
1039	0.06	0.06	32.65999854	3.517999873	0	Uncharacterized protein C1orf105 homolog OS=Rattus norvegicus PE=2 SV=1	RAT	0	0.887878776	
1040	0.06	0.06	30.25999963	9.211000055	0	REVERSED Small VCP/p97-interacting protein OS=Rattus norvegicus GN=Svip PE=1 SV=1	RAT	0	0.960086286	
1041	0.06	0.06	8.771999925	5.847999826	0	Neudesin OS=Rattus norvegicus GN=Nenf PE=2 SV=1	RAT	0	0.890047073	
1042	0.05	7.8	72.72999883	11.96999997	7.594999671	Dynamin-3 OS=Rattus norvegicus GN=Dnm3 PE=1 SV=2	RAT	6	0.957279623	0.750396788
1043	0.05	0.3	35.85000038	0.297800009	0	GRIP and coiled-coil domain-containing protein 2 OS=Rattus norvegicus GN=Gcc2 PE=1 SV=1	RAT	0	0.863301992	0.158732593
1044	0.05	0.25	35.71000099	0.121100002	0	Xin actin-binding repeat-containing protein 2 OS=Rattus norvegicus GN=Xirp2 PE=2 SV=1	RAT	0	1.041304946	0.739043474
1045	0.05	0.24	24.86000061	0	0	REVERSED E3 ubiquitin-protein ligase UBR4 OS=Rattus norvegicus GN=Ubr4 PE=2 SV=2	RAT	0	1.030586004	0.821106076
1046	0.05	0.2	24.76000041	0.430600019	0	REVERSED Diacylglycerol kinase zeta OS=Rattus norvegicus GN=Dgkz PE=2 SV=1	RAT	0	1.059589028	
1047	0.05	0.19	33.25000107	0.39400002	0	REVERSED Protein strawberry notch homolog 1 OS=Rattus norvegicus GN=Sbno1 PE=2 SV=2	RAT	0	1.116003036	0.308183491
1048	0.05	0.16	29.53000069	0	0	Complement C4 OS=Rattus norvegicus GN=C4 PE=1 SV=3	RAT	0	0.731824815	
1049	0.05	0.15	26.53000057	1.28899999	0	Scaffold attachment factor B1 OS=Rattus norvegicus GN=Safb PE=1 SV=2	RAT	0	0.911934376	0.579383373
1050	0.05	0.13	22.05000073	0	0	PH domain leucine-rich repeat protein phosphatase 1 OS=Rattus norvegicus GN=Phlpp1 PE=1 SV=1	RAT	0	0.92564559	
1051	0.05	0.13	20.90999931	0	0	Latrophilin-2 OS=Rattus norvegicus GN=Lphn2 PE=1 SV=2	RAT	0	1.140843034	0.409961402
1052	0.05	0.13	27.73000002	3.150999919	0	REVERSED Non-POU domain-containing octamer-binding protein OS=Rattus norvegicus GN=Nono PE=2 SV=3	RAT	0	0.911723375	
1053	0.05	0.12	64.67999816	0	0	REVERSED Liprin-alpha-3 OS=Rattus norvegicus GN=Ppfia3 PE=1 SV=2	RAT	0	0.912774682	0.39059329
1054	0.05	0.12	39.39999938	0	0	REVERSED Vinculin OS=Rattus norvegicus GN=Vcl PE=1 SV=1	RAT	0	1.06873703	0.759241521
1055	0.05	0.12	34.61999893	0	0	Protogenin OS=Rattus norvegicus GN=Prtg PE=2 SV=1	RAT	0	1.038544059	0.767648816
1056	0.05	0.11	18.52999926	0.866699964	0	REVERSED Myosin-Vb OS=Rattus norvegicus GN=Myo5b PE=1 SV=1	RAT	0	1.026049018	0.838158727
1057	0.05	0.11	21.26999944	0	0	REVERSED Sodium channel protein type 10 subunit alpha OS=Rattus norvegicus GN=Scn10a PE=1 SV=1	RAT	0	0.944317222	0.553937376
1058	0.05	0.1	25.69999993	0	0	Serine/threonine-protein kinase mTOR OS=Rattus norvegicus GN=Mtor PE=1 SV=1	RAT	0	0.993554115	0.946468115
1059	0.05	0.1	30.34999967	0	0	Cystic fibrosis transmembrane conductance regulator OS=Rattus norvegicus GN=Cftr PE=1 SV=3	RAT	0	1.050176978	0.607066095
1060	0.05	0.1	33.01999867	0	0	Double-stranded RNA-specific adenosine deaminase OS=Rattus norvegicus GN=Adar PE=2 SV=1	RAT	0	0.957899928	
1061	0.05	0.1	50.40000081	0	0	REVERSED Keratin, type II cytoskeletal 1 OS=Rattus norvegicus GN=Krt1 PE=2 SV=1	RAT	0	1.006006956	0.962199688
1062	0.05	0.1	17.03000069	0	0	Neurogenic locus notch homolog protein 3 OS=Rattus norvegicus GN=Notch3 PE=2 SV=2	RAT	0	1.071578026	0.613606215
1063	0.05	0.1	21.0800007	0	0	Heparanase OS=Rattus norvegicus GN=Hpse PE=2 SV=1	RAT	0	0.964771807	0.840804815
1064	0.05	0.09	31.15000129	0.648899982	0	Regulator of G-protein signaling 12 OS=Rattus norvegicus GN=Rgs12 PE=1 SV=1	RAT	0	1.024690032	
1065	0.05	0.09	24.77999926	0	0	Crooked neck-like protein 1 OS=Rattus norvegicus GN=Crnkl1 PE=2 SV=1	RAT	0	0.944989622	0.56262958
1066	0.05	0.09	17.3299998	0	0	REVERSED Aldehyde dehydrogenase family 16 member A1 OS=Rattus norvegicus GN=Aldh16a1 PE=2 SV=1	RAT	0	1.01256001	0.872790515
1067	0.05	0.08	28.16999853	0	0	REVERSED Down syndrome cell adhesion molecule homolog OS=Rattus norvegicus GN=Dscam PE=1 SV=1	RAT	0	1.057773948	0.691904783
1068	0.05	0.08	22.41999954	1.265999954	0	Voltage-dependent P/Q-type calcium channel subunit alpha-1A OS=Rattus norvegicus GN=Cacna1a PE=1 SV=1	RAT	0		
1069	0.05	0.08	31.16999865	2.66200006	0	REVERSED Multidrug resistance protein 1 OS=Rattus norvegicus GN=Abcb1 PE=2 SV=1	RAT	0	1.199329972	
1070	0.05	0.08	31.33000135	0	0	REVERSED ATP-binding cassette sub-family C member 9 OS=Rattus norvegicus GN=Abcc9 PE=2 SV=1	RAT	0	1.020812035	0.827795506
1071	0.05	0.08	34.5299989	0.631600013	0	REVERSED Lon protease homolog, mitochondrial OS=Rattus norvegicus GN=Lonp1 PE=2 SV=1	RAT	0		
1071	0	0.06	8.828999847	1.018999983	0	Muscarinic acetylcholine receptor M3 OS=Rattus norvegicus GN=Chrm3 PE=1 SV=1	RAT	0		
1072	0.05	0.08	29.15000021	0	0	Myosin phosphatase Rho-interacting protein OS=Rattus norvegicus GN=Mprip PE=1 SV=1	RAT	0	1.05528295	0.682964623

1073	0.05	0.08	27.5000006	1.183999982	0	Rho GTPase-activating protein 20 OS=Rattus norvegicus GN=Arhgap20 PE=2 SV=2	RAT	0		
1074	0.05	0.08	24.4599998	0	0	NLR family member X1 OS=Rattus norvegicus GN=Nlrx1 PE=2 SV=1	RAT	0	0.896664083	
1075	0.05	0.08	35.96999943	0	0	REVERSED Sterile alpha motif domain-containing protein 14 OS=Rattus norvegicus GN=Samd14 PE=2 SV=1	RAT	0	0.822708428	0.301668614
1076	0.05	0.08	10.14999971	1.212000009	0	REVERSED Arginyl-tRNA synthetase, cytoplasmic OS=Rattus norvegicus GN=Rars PE=1 SV=2	RAT	0	1.010928035	0.937102675
1077	0.05	0.07	26.78000033	0	0	REVERSED Protocadherin Fat 3 OS=Rattus norvegicus GN=Fat3 PE=1 SV=1	RAT	0	0.918620884	0.356927007
1078	0.05	0.07	29.03999984	0	0	Cadherin EGF LAG seven-pass G-type receptor 3 OS=Rattus norvegicus GN=Celsr3 PE=2 SV=1	RAT	0	0.941351414	0.65107739
1079	0.05	0.07	30.82000017	0	0	Serine/threonine-protein kinase MRCK beta OS=Rattus norvegicus GN=Cdc42bpb PE=1 SV=1	RAT	0	0.874985218	0.529793382
1080	0.05	0.07	37.99000084	0	0	REVERSED SH3 and multiple ankyrin repeat domains protein 2 OS=Rattus norvegicus GN=Shank2 PE=1 SV=2	RAT	0	1.103368998	0.364073604
1081	0.05	0.07	26.60000026	0	0	Ubiquitin carboxyl-terminal hydrolase 19 OS=Rattus norvegicus GN=Usp19 PE=1 SV=1	RAT	0	1.272333026	0.251008898
1082	0.05	0.07	33.61000121	1.659999974	0	REVERSED Protein timeless homolog OS=Rattus norvegicus GN=Timeless PE=1 SV=1	RAT	0	0.962234378	
1083	0.05	0.07	29.15999889	0	0	REVERSED Glutamate [NMDA] receptor subunit 3A OS=Rattus norvegicus GN=Grin3a PE=1 SV=1	RAT	0	0.987483323	0.8925246
1084	0.05	0.07	25.44000149	0	0	REVERSED Phosphorylase b kinase regulatory subunit alpha, skeletal muscle isoform OS=Rattus norvegicus GN=Phka1 PE=2 SV=2	RAT	0	0.925043702	0.57499361
1085	0.05	0.07	33.12000036	0	0	REVERSED Zinc finger protein DZIP1L OS=Rattus norvegicus GN=Dzip1l PE=2 SV=1	RAT	0	0.859223723	
1086	0.05	0.07	59.89000201	3.621000051	0	REVERSED Pyruvate dehydrogenase E1 component subunit beta, mitochondrial OS=Rattus norvegicus GN=Pdhb PE=1 SV=2	RAT	0		
1087	0.05	0.07	18.72999966	1.727000065	0	Guanylate cyclase 2G OS=Rattus norvegicus GN=Gucy2g PE=2 SV=2	RAT	0	1.192283988	
1088	0.05	0.07	42.68000126	0	0	REVERSED NGFI-A-binding protein 1 OS=Rattus norvegicus GN=Nab1 PE=2 SV=2	RAT	0	0.942474723	0.661143482
1089	0.05	0.07	23.29999954	0.459800009	0	REVERSED Contactin-associated protein like 5-1 OS=Rattus norvegicus GN=Cntnap5a PE=2 SV=1	RAT	0	1.01177597	
1090	0.05	0.07	18.59000027	0	0	REVERSED Tyrosine-protein kinase Fer OS=Rattus norvegicus GN=Fer PE=2 SV=2	RAT	0	1.073253989	0.671077907
1091	0.05	0.07	26.91000104	0	0	REVERSED SH3 domain-binding protein 5 OS=Rattus norvegicus GN=Sh3bp5 PE=1 SV=2	RAT	0	0.994921684	0.961770415
1092	0.05	0.07	44.35000122	2.301000059	0	REVERSED Patatin-like phospholipase domain-containing protein 2 OS=Rattus norvegicus GN=Pnpla2 PE=2 SV=1	RAT	0		
1093	0.05	0.07	44.69000101	0	0	H/ACA ribonucleoprotein complex subunit 1 OS=Rattus norvegicus GN=Gar1 PE=2 SV=1	RAT	0	0.833078682	0.324280888
1094	0.05	0.07	32.39000142	0	0	Serum paraoxonase/arylesterase 1 OS=Rattus norvegicus GN=Pon1 PE=1 SV=3	RAT	0	1.081537008	
1095	0.05	0.07	19.72000003	1.689999923	0	REVERSED Solute carrier organic anion transporter family member 3A1 OS=Rattus norvegicus GN=Slco3a1 PE=2 SV=1	RAT	0	1.001361012	0.994511425
1096	0.05	0.07	23.11999947	3.064000048	0	Tropomodulin-1 OS=Rattus norvegicus GN=Tmod1 PE=2 SV=1	RAT	0	1.100944996	
1097	0.05	0.07	11.64999977	2.981000021	0	REVERSED Tyrosine-protein phosphatase non-receptor type 5 OS=Rattus norvegicus GN=Ptpn5 PE=2 SV=2	RAT	0	0.767886102	
1098	0.05	0.06	40.75999856	0.580799999	0	REVERSED Potassium-transporting ATPase alpha chain 1 OS=Rattus norvegicus GN=Atp4a PE=2 SV=3	RAT	0		
1099	0.05	0.06	30.27000129	0	0	ELKS/Rab6-interacting/CAST family member 1 OS=Rattus norvegicus GN=Erc1 PE=1 SV=1	RAT	0	1.137336016	
1100	0.05	0.06	34.11000073	1.589000039	0	Protein unc-45 homolog A OS=Rattus norvegicus GN=Unc45a PE=2 SV=1	RAT	0	0.90258348	
1101	0.05	0.06	34.1899991	0	0	REVERSED Nephrin OS=Rattus norvegicus GN=Nphs1 PE=1 SV=2	RAT	0	0.916048408	0.555169225
1102	0.05	0.06	23.63999933	0	0	REVERSED Multidrug resistance-associated protein 6 OS=Rattus norvegicus GN=Abcc6 PE=2 SV=1	RAT	0	0.867742777	0.397914201
1103	0.05	0.06	27.20000148	0	0	Ankycorbin OS=Rattus norvegicus GN=Rai14 PE=2 SV=2	RAT	0	0.817273617	
1104	0.05	0.06	38.90999854	0.997800007	0	REVERSED Aldehyde dehydrogenase family 1 member L1 OS=Rattus norvegicus GN=Aldh1l1 PE=1 SV=2	RAT	0	0.981759012	
1105	0.05	0.06	23.3799994	1.264000032	0	REVERSED ATP-dependent Clp protease ATP-binding subunit clpX-like, mitochondrial OS=Rattus norvegicus GN=Clpx PE=2 SV=1	RAT	0	0.846912622	
1106	0.05	0.06	49.88999963	1.290000044	0	E3 ubiquitin-protein ligase parkin OS=Rattus norvegicus GN=Park2 PE=1 SV=1	RAT	0		
1107	0.05	0.06	46.2500006	0	0	REVERSED Lysophosphatidylcholine acyltransferase 1 OS=Rattus norvegicus GN=Lpcat1 PE=2 SV=2	RAT	0	0.894353271	
1108	0.05	0.06	24.95999932	0	0	Exocyst complex component 7 OS=Rattus norvegicus GN=Exoc7 PE=2 SV=1	RAT	0	0.687576175	
1109	0.05	0.06	40.29999971	0	0	REVERSED Induced myeloid leukemia cell differentiation protein Mcl-1 homolog OS=Rattus norvegicus GN=Mcl1 PE=1 SV=2	RAT	0	0.934430301	0.415139705
1110	0.05	0.06	24.07999933	0	0	Rap guanine nucleotide exchange factor 4 (Fragment) OS=Rattus norvegicus GN=Rapgef4 PE=2 SV=1	RAT	0		
1111	0.05	0.06	15.96000046	0	0	Proprotein convertase subtilisin/kexin type 7 OS=Rattus norvegicus GN=Pcsk7 PE=1 SV=1	RAT	0	1.072234035	0.614597619
1112	0.05	0.06	17.63000041	0	0	39S ribosomal protein L38, mitochondrial OS=Rattus norvegicus GN=Mrpl38 PE=2 SV=2	RAT	0	1.065814018	0.514831185
1113	0.05	0.06	31.00000024	5.240000039	0	UPF0765 protein C10orf58 homolog OS=Rattus norvegicus PE=1 SV=1	RAT	0	0.906886578	
1114	0.05	0.06	18.42000037	3.158000112	0	REVERSED 39S ribosomal protein L38, mitochondrial OS=Rattus norvegicus GN=Mrpl38 PE=2 SV=2	RAT	0	1.217334032	
1115	0.05	0.06	25.69000125	4.51399982	0	REVERSED Corticosteroid 11-beta-dehydrogenase isozyme 1 OS=Rattus norvegicus GN=Hsd11b1 PE=1 SV=2	RAT	0	1.258103967	
1116	0.05	0.06	17.28000045	1.553000044	0	REVERSED UPF0464 protein C15orf44 homolog OS=Rattus norvegicus PE=2 SV=1	RAT	0		
1117	0.05	0.06	37.95000017	4.910999909	0	Claudin-19 OS=Rattus norvegicus GN=Cldn19 PE=2 SV=1	RAT	0		
1118	0.05	0.06	21.67000026	4.444000125	0	REVERSED Insulin-like growth factor II OS=Rattus norvegicus GN=Igf2 PE=1 SV=1	RAT	0		
1119	0.05	0.06	28.36000025	8.955000341	0	ATP synthase protein 8 OS=Rattus norvegicus GN=Mt-atp8 PE=1 SV=1	RAT	0	1.015629053	
1120	0.05	0.05	71.53000236	0	0	REVERSED Creatine kinase U-type, mitochondrial OS=Rattus norvegicus GN=Ckmt1 PE=1 SV=1	RAT	0	1.155729055	0.217710406
1121	0.05	0.05	52.42000222	0	0	REVERSED Trifunctional enzyme subunit alpha, mitochondrial OS=Rattus norvegicus GN=Hadha PE=1 SV=2	RAT	0	1.079455972	
1122	0.05	0.05	32.60999918	0	0	Probable ATP-dependent RNA helicase DHX40 OS=Rattus norvegicus GN=Dhx40 PE=2 SV=1	RAT	0	1.139835	
1123	0.05	0.05	24.25000072	2.425000072	0	Glutamate [NMDA] receptor subunit epsilon-3 OS=Rattus norvegicus GN=Grin2c PE=1 SV=1	RAT	0		
1124	0.05	0.05	34.4599992	0	0	REVERSED Mitogen-activated protein kinase kinase kinase 12 OS=Rattus norvegicus GN=Map3k12 PE=1 SV=1	RAT	0		
1125	0.05	0.05	54.83999848	0	0	Friend of PRMT1 protein OS=Rattus norvegicus GN=Fop PE=2 SV=1	RAT	0	1.066357017	0.648474872
1126	0.05	0.05	32.67999887	0	0	Echinoderm microtubule-associated protein-like 1 OS=Rattus norvegicus GN=Eml1 PE=2 SV=2	RAT	0	0.981691182	0.904366612
1127	0.05	0.05	39.14999962	0	0	REVERSED Prolyl 4-hydroxylase subunit alpha-3 OS=Rattus norvegicus GN=P4ha3 PE=2 SV=1	RAT	0	0.95285058	0.710625589
1128	0.05	0.05	15.92999995	0	0	REVERSED Semaphorin-3A OS=Rattus norvegicus GN=Sema3a PE=2 SV=1	RAT	0	1.046229005	
1129	0.05	0.05	15.02999961	0	0	REVERSED Calsyntenin-2 OS=Rattus norvegicus GN=Clstn2 PE=2 SV=1	RAT	0	1.02955997	
1130	0.05	0.05	36.62999868	1.484999992	0	REVERSED 5-azacytidine-induced protein 2 OS=Rattus norvegicus GN=Azi2 PE=2 SV=1	RAT	0		
1131	0.05	0.05	43.81000102	0	0	REVERSED Cyclin-dependent kinase 11 OS=Rattus norvegicus GN=Cdk11 PE=2 SV=1	RAT	0	1.109109998	0.489100486
1132	0.05	0.05	27.61000097	1.971999928	0	Uncharacterized protein C9orf128 homolog OS=Rattus norvegicus PE=2 SV=1	RAT	0	1.031594038	
1133	0.05	0.05	25.40999949	1.319999993	0	WD repeat-containing protein 1 OS=Rattus norvegicus GN=Wdr1 PE=1 SV=3	RAT	0	0.983479083	
1134	0.05	0.05	24.31000024	0	0	REVERSED Cannabinoid receptor 1 OS=Rattus norvegicus GN=Cnr1 PE=1 SV=1	RAT	0	1.163089037	
1135	0.05	0.05	30.32000065	2.445000038	0	REVERSED Z-DNA-binding protein 1 OS=Rattus norvegicus GN=Zbp1 PE=2 SV=1	RAT	0	0.937187195	
1136	0.05	0.05	31.25	10.10000035	0	REVERSED Uncharacterized protein C4orf51 homolog OS=Rattus norvegicus PE=2 SV=1	RAT	0	0.912447989	0.525371015
1137	0.05	0.05	14.01000023	2.137999982	0	Medium-chain specific acyl-CoA dehydrogenase, mitochondrial OS=Rattus norvegicus GN=Acadm PE=1 SV=1	RAT	0	0.978549182	
1138	0.05	0.05	15.52000046	1.410999987	0	REVERSED Angiopoietin-2 OS=Rattus norvegicus GN=Angpt2 PE=2 SV=2	RAT	0	0.795760512	0.259955704
1139	0.05	0.05	60.55999994	4.930000007	0	ATP synthase lipid-binding protein, mitochondrial OS=Rattus norvegicus GN=Atp5g3 PE=2 SV=1	RAT	0	0.860621512	
1139	0	0.05	37.59000003	4.964999855	0	ATP synthase lipid-binding protein, mitochondrial OS=Rattus norvegicus GN=Atp5g2 PE=2 SV=1	RAT	0		
1139	0	0.05	55.87999821	5.14700003	0	ATP synthase lipid-binding protein, mitochondrial OS=Rattus norvegicus GN=Atp5g1 PE=1 SV=1	RAT	0		
1140	0.05	0.05	38.01000118	4.977000132	0	REVERSED Ras-related protein Rab-28 OS=Rattus norvegicus GN=Rab28 PE=2 SV=1	RAT	0	0.901447415	
1141	0.05	0.05	21.86000049	3.720999882	0	Casein kinase II subunit beta OS=Rattus norvegicus GN=Csnk2b PE=1 SV=1	RAT	0	0.91908741	
1142	0.05	0.05	5.235999823	3.141000122	0	Calcium-activated potassium channel subunit beta-1 OS=Rattus norvegicus GN=Kcnmb1 PE=2 SV=4	RAT	0		

Peptides

Title	Accessions	Names	Sequence
>	sp|P16086|SPT	Spectrin alpha chain, brain OS=Rattus norvegicus GN=Sptan1 PE=1 SV=2	AALLELWELR
>	sp|P16086|SPT	Spectrin alpha chain, brain OS=Rattus norvegicus GN=Sptan1 PE=1 SV=2	AALLELWELRR
>	sp|P16086|SPT	Spectrin alpha chain, brain OS=Rattus norvegicus GN=Sptan1 PE=1 SV=2	ADVVESWIGEKENSLK
>	sp|P16086|SPT	Spectrin alpha chain, brain OS=Rattus norvegicus GN=Sptan1 PE=1 SV=2	AGTFQAFEQFGQQLLAHGHYASPEIK
>	sp|P16086|SPT	Spectrin alpha chain, brain OS=Rattus norvegicus GN=Sptan1 PE=1 SV=2	AQLADSFHLQQFFR
>	sp|P16086|SPT	Spectrin alpha chain, brain OS=Rattus norvegicus GN=Sptan1 PE=1 SV=2	DLAALEDKVK
>	sp|P16086|SPT	Spectrin alpha chain, brain OS=Rattus norvegicus GN=Sptan1 PE=1 SV=2	DLAALGDKVNSLGETAQR
>	sp|P16086|SPT	Spectrin alpha chain, brain OS=Rattus norvegicus GN=Sptan1 PE=1 SV=2	DLASVQALLR
>	sp|P16086|SPT	Spectrin alpha chain, brain OS=Rattus norvegicus GN=Sptan1 PE=1 SV=2	DLSSVQTLLTK
>	sp|P16086|SPT	Spectrin alpha chain, brain OS=Rattus norvegicus GN=Sptan1 PE=1 SV=2	DVEDEETWIR
>	sp|P16086|SPT	Spectrin alpha chain, brain OS=Rattus norvegicus GN=Sptan1 PE=1 SV=2	EAALTSEEVGADLEQVEVLQK
>	sp|P16086|SPT	Spectrin alpha chain, brain OS=Rattus norvegicus GN=Sptan1 PE=1 SV=2	EKEPIAASTNR
>	sp|P16086|SPT	Spectrin alpha chain, brain OS=Rattus norvegicus GN=Sptan1 PE=1 SV=2	FKELSTLR
>	sp|P16086|SPT	Spectrin alpha chain, brain OS=Rattus norvegicus GN=Sptan1 PE=1 SV=2	GKDLIGVQNLLK
>	sp|P16086|SPT	Spectrin alpha chain, brain OS=Rattus norvegicus GN=Sptan1 PE=1 SV=2	GLVSSDELAKDVTGAEALLER
>	sp|P16086|SPT	Spectrin alpha chain, brain OS=Rattus norvegicus GN=Sptan1 PE=1 SV=2	GRELPTAFDYVEFTR
>	sp|P16086|SPT	Spectrin alpha chain, brain OS=Rattus norvegicus GN=Sptan1 PE=1 SV=2	HALLEADVAAHQDR
>	sp|P16086|SPT	Spectrin alpha chain, brain OS=Rattus norvegicus GN=Sptan1 PE=1 SV=2	HQAFEAELHANADR
>	sp|P16086|SPT	Spectrin alpha chain, brain OS=Rattus norvegicus GN=Sptan1 PE=1 SV=2	HQAFEAELSANQSR
>	sp|P16086|SPT	Spectrin alpha chain, brain OS=Rattus norvegicus GN=Sptan1 PE=1 SV=2	HQALQAEIAGHEPR
>	sp|P16086|SPT	Spectrin alpha chain, brain OS=Rattus norvegicus GN=Sptan1 PE=1 SV=2	IAALQAFADQLIAVDHYAK
>	sp|P16086|SPT	Spectrin alpha chain, brain OS=Rattus norvegicus GN=Sptan1 PE=1 SV=2	KGDILTLLNSTNKDWWK
>	sp|P16086|SPT	Spectrin alpha chain, brain OS=Rattus norvegicus GN=Sptan1 PE=1 SV=2	KHALLEADVAAHQDRIDGITIQAR
>	sp|P16086|SPT	Spectrin alpha chain, brain OS=Rattus norvegicus GN=Sptan1 PE=1 SV=2	KHEAFETDFTVHKDR
>	sp|P16086|SPT	Spectrin alpha chain, brain OS=Rattus norvegicus GN=Sptan1 PE=1 SV=2	KHEALMSDLSAYGSSIQALR
>	sp|P16086|SPT	Spectrin alpha chain, brain OS=Rattus norvegicus GN=Sptan1 PE=1 SV=2	KIEDLGAAMEEALILDNK
>	sp|P16086|SPT	Spectrin alpha chain, brain OS=Rattus norvegicus GN=Sptan1 PE=1 SV=2	KLLVSSEDYGR
>	sp|P16086|SPT	Spectrin alpha chain, brain OS=Rattus norvegicus GN=Sptan1 PE=1 SV=2	LAALADQWQFLVQK
>	sp|P16086|SPT	Spectrin alpha chain, brain OS=Rattus norvegicus GN=Sptan1 PE=1 SV=2	LAQFVEHWK
>	sp|P16086|SPT	Spectrin alpha chain, brain OS=Rattus norvegicus GN=Sptan1 PE=1 SV=2	LFGAAEVQR
>	sp|P16086|SPT	Spectrin alpha chain, brain OS=Rattus norvegicus GN=Sptan1 PE=1 SV=2	LGDSHDLQR
>	sp|P16086|SPT	Spectrin alpha chain, brain OS=Rattus norvegicus GN=Sptan1 PE=1 SV=2	LLEAQSHFR
>	sp|P16086|SPT	Spectrin alpha chain, brain OS=Rattus norvegicus GN=Sptan1 PE=1 SV=2	LSDDNTIGQEEIQQR
>	sp|P16086|SPT	Spectrin alpha chain, brain OS=Rattus norvegicus GN=Sptan1 PE=1 SV=2	MMLDHCLELQLFHR
>	sp|P16086|SPT	Spectrin alpha chain, brain OS=Rattus norvegicus GN=Sptan1 PE=1 SV=2	MNEVISLWK
>	sp|P16086|SPT	Spectrin alpha chain, brain OS=Rattus norvegicus GN=Sptan1 PE=1 SV=2	MQHNLEQQIQAR
>	sp|P16086|SPT	Spectrin alpha chain, brain OS=Rattus norvegicus GN=Sptan1 PE=1 SV=2	MTLVASEDYGDTLAAIQGLLK
>	sp|P16086|SPT	Spectrin alpha chain, brain OS=Rattus norvegicus GN=Sptan1 PE=1 SV=2	NVEDIELWLYEVEGHLASDDYGKDLTNVQNLQK
>	sp|P16086|SPT	Spectrin alpha chain, brain OS=Rattus norvegicus GN=Sptan1 PE=1 SV=2	QEFAQHANAFHQWIQETR
>	sp|P16086|SPT	Spectrin alpha chain, brain OS=Rattus norvegicus GN=Sptan1 PE=1 SV=2	QETFDAGLQAFQQEGIANITALKDQLLAAK
>	sp|P16086|SPT	Spectrin alpha chain, brain OS=Rattus norvegicus GN=Sptan1 PE=1 SV=2	REELITNWEQIR
>	sp|P16086|SPT	Spectrin alpha chain, brain OS=Rattus norvegicus GN=Sptan1 PE=1 SV=2	RLEAELAAHEPAIQGVLDTGKK
>	sp|P16086|SPT	Spectrin alpha chain, brain OS=Rattus norvegicus GN=Sptan1 PE=1 SV=2	RQDLEDSLQAQQYFADANEAESWMR
>	sp|P16086|SPT	Spectrin alpha chain, brain OS=Rattus norvegicus GN=Sptan1 PE=1 SV=2	SADESGQALLAAGHYASDEVR
>	sp|P16086|SPT	Spectrin alpha chain, brain OS=Rattus norvegicus GN=Sptan1 PE=1 SV=2	SSLSSAQADFNQLAELDR
>	sp|P16086|SPT	Spectrin alpha chain, brain OS=Rattus norvegicus GN=Sptan1 PE=1 SV=2	TKQEEVNAAWQR
>	sp|P16086|SPT	Spectrin alpha chain, brain OS=Rattus norvegicus GN=Sptan1 PE=1 SV=2	LIQSHPESAEDLKEK
>	sp|P16086|SPT	Spectrin alpha chain, brain OS=Rattus norvegicus GN=Sptan1 PE=1 SV=2	LEAELAAHEPAIQGVLDTGK
>	sp|P16086|SPT	Spectrin alpha chain, brain OS=Rattus norvegicus GN=Sptan1 PE=1 SV=2	ENLLEEQGSIALR
>	sp|P16086|SPT	Spectrin alpha chain, brain OS=Rattus norvegicus GN=Sptan1 PE=1 SV=2	RLEAELAAHEPAIQGVLDTGK
>	sp|P16086|SPT	Spectrin alpha chain, brain OS=Rattus norvegicus GN=Sptan1 PE=1 SV=2	KASAFNSWFENAEEDLTDPVR
>	sp|P16086|SPT	Spectrin alpha chain, brain OS=Rattus norvegicus GN=Sptan1 PE=1 SV=2	LIQNNHYAMEDVATR
>	sp|P16086|SPT	Spectrin alpha chain, brain OS=Rattus norvegicus GN=Sptan1 PE=1 SV=2	KVEDLFLTFAK
>	sp|P16086|SPT	Spectrin alpha chain, brain OS=Rattus norvegicus GN=Sptan1 PE=1 SV=2	HQAFEAEVQANSGAIVK
>	sp|P16086|SPT	Spectrin alpha chain, brain OS=Rattus norvegicus GN=Sptan1 PE=1 SV=2	KLSDDNTIGQEEIQQR
>	sp|P16086|SPT	Spectrin alpha chain, brain OS=Rattus norvegicus GN=Sptan1 PE=1 SV=2	KFEEFQTDLAAHEER
>	sp|P16086|SPT	Spectrin alpha chain, brain OS=Rattus norvegicus GN=Sptan1 PE=1 SV=2	QEAFLLNEDLGDSLDSVEALLK
>	sp|P16086|SPT	Spectrin alpha chain, brain OS=Rattus norvegicus GN=Sptan1 PE=1 SV=2	LQQSHPLSANQIQVK
>	sp|P16086|SPT	Spectrin alpha chain, brain OS=Rattus norvegicus GN=Sptan1 PE=1 SV=2	ADVVESWIGEKENSLK
>	sp|P16086|SPT	Spectrin alpha chain, brain OS=Rattus norvegicus GN=Sptan1 PE=1 SV=2	AGTFQAFEQFGQQLLAHGHYASPEIK
>	sp|P16086|SPT	Spectrin alpha chain, brain OS=Rattus norvegicus GN=Sptan1 PE=1 SV=2	AGTFQAFEQFGQQLLAHGHYASPEIK
>	sp|P16086|SPT	Spectrin alpha chain, brain OS=Rattus norvegicus GN=Sptan1 PE=1 SV=2	AGTFQAFEQFGQQLLAHGHYASPEIK
>	sp|P16086|SPT	Spectrin alpha chain, brain OS=Rattus norvegicus GN=Sptan1 PE=1 SV=2	AGTFQAFEQFGQQLLAHGHYASPEIK
>	sp|P16086|SPT	Spectrin alpha chain, brain OS=Rattus norvegicus GN=Sptan1 PE=1 SV=2	AGTFQAFEQFGQQLLAHGHYASPEIK
>	sp|P16086|SPT	Spectrin alpha chain, brain OS=Rattus norvegicus GN=Sptan1 PE=1 SV=2	AGTFQAFEQFGQQLLAHGHYASPEIK
>	sp|P16086|SPT	Spectrin alpha chain, brain OS=Rattus norvegicus GN=Sptan1 PE=1 SV=2	EAALTSEEVGADLEQVEVLQK
>	sp|P16086|SPT	Spectrin alpha chain, brain OS=Rattus norvegicus GN=Sptan1 PE=1 SV=2	EAALTSEEVGADLEQVEVLQK
>	sp|P16086|SPT	Spectrin alpha chain, brain OS=Rattus norvegicus GN=Sptan1 PE=1 SV=2	EKEPIAASTNR
>	sp|P16086|SPT	Spectrin alpha chain, brain OS=Rattus norvegicus GN=Sptan1 PE=1 SV=2	HALLEADVAAHQDR
>	sp|P16086|SPT	Spectrin alpha chain, brain OS=Rattus norvegicus GN=Sptan1 PE=1 SV=2	HALLEADVAAHQDR
>	sp|P16086|SPT	Spectrin alpha chain, brain OS=Rattus norvegicus GN=Sptan1 PE=1 SV=2	HALLEADVAAHQDR
>	sp|P16086|SPT	Spectrin alpha chain, brain OS=Rattus norvegicus GN=Sptan1 PE=1 SV=2	HQAFEAELHANADR
>	sp|P16086|SPT	Spectrin alpha chain, brain OS=Rattus norvegicus GN=Sptan1 PE=1 SV=2	HQAFEAELSANQSR

>	sp|P16086|SPT Spectrin alpha chain, brain OS=Rattus norvegicus GN=Sptan1 PE=1 SV=2	HQAFEAELSANQSR
>	sp|P16086|SPT Spectrin alpha chain, brain OS=Rattus norvegicus GN=Sptan1 PE=1 SV=2	HQAFEAELSANQSR
>	sp|P16086|SPT Spectrin alpha chain, brain OS=Rattus norvegicus GN=Sptan1 PE=1 SV=2	HQAFEAELSANQSR
>	sp|P16086|SPT Spectrin alpha chain, brain OS=Rattus norvegicus GN=Sptan1 PE=1 SV=2	HQAFEAEVQANSGAIVK
>	sp|P16086|SPT Spectrin alpha chain, brain OS=Rattus norvegicus GN=Sptan1 PE=1 SV=2	HQALQAEIAGHEPR
>	sp|P16086|SPT Spectrin alpha chain, brain OS=Rattus norvegicus GN=Sptan1 PE=1 SV=2	HQALQAEIAGHEPR
>	sp|P16086|SPT Spectrin alpha chain, brain OS=Rattus norvegicus GN=Sptan1 PE=1 SV=2	IAALQAFADQLIAVDHYAK
>	sp|P16086|SPT Spectrin alpha chain, brain OS=Rattus norvegicus GN=Sptan1 PE=1 SV=2	IAALQAFADQLIAVDHYAK
>	sp|P16086|SPT Spectrin alpha chain, brain OS=Rattus norvegicus GN=Sptan1 PE=1 SV=2	IAALQAFADQLIAVDHYAK
>	sp|P16086|SPT Spectrin alpha chain, brain OS=Rattus norvegicus GN=Sptan1 PE=1 SV=2	IAALQAFADQLIAVDHYAK
>	sp|P16086|SPT Spectrin alpha chain, brain OS=Rattus norvegicus GN=Sptan1 PE=1 SV=2	IAALQAFADQLIAVDHYAK
>	sp|P16086|SPT Spectrin alpha chain, brain OS=Rattus norvegicus GN=Sptan1 PE=1 SV=2	IAALQAFADQLIAVDHYAK
>	sp|P16086|SPT Spectrin alpha chain, brain OS=Rattus norvegicus GN=Sptan1 PE=1 SV=2	KASAFNSWFENAEEDLTDPVR
>	sp|P16086|SPT Spectrin alpha chain, brain OS=Rattus norvegicus GN=Sptan1 PE=1 SV=2	KASAFNSWFENAEEDLTDPVR
>	sp|P16086|SPT Spectrin alpha chain, brain OS=Rattus norvegicus GN=Sptan1 PE=1 SV=2	KFEEFQTDLAAHEER
>	sp|P16086|SPT Spectrin alpha chain, brain OS=Rattus norvegicus GN=Sptan1 PE=1 SV=2	KGDILTLLNSTNKDWWK
>	sp|P16086|SPT Spectrin alpha chain, brain OS=Rattus norvegicus GN=Sptan1 PE=1 SV=2	KHALLEADVAAHQDRIDGITIQAR
>	sp|P16086|SPT Spectrin alpha chain, brain OS=Rattus norvegicus GN=Sptan1 PE=1 SV=2	KHALLEADVAAHQDRIDGITIQAR
>	sp|P16086|SPT Spectrin alpha chain, brain OS=Rattus norvegicus GN=Sptan1 PE=1 SV=2	KHEAFETDFTVHKDR
>	sp|P16086|SPT Spectrin alpha chain, brain OS=Rattus norvegicus GN=Sptan1 PE=1 SV=2	KHEAFETDFTVHKDR
>	sp|P16086|SPT Spectrin alpha chain, brain OS=Rattus norvegicus GN=Sptan1 PE=1 SV=2	KHEALMSDLSAYGSSIQALR
>	sp|P16086|SPT Spectrin alpha chain, brain OS=Rattus norvegicus GN=Sptan1 PE=1 SV=2	KHEALMSDLSAYGSSIQALR
>	sp|P16086|SPT Spectrin alpha chain, brain OS=Rattus norvegicus GN=Sptan1 PE=1 SV=2	KIEDLGAAMEEALILDNK
>	sp|P16086|SPT Spectrin alpha chain, brain OS=Rattus norvegicus GN=Sptan1 PE=1 SV=2	KIEDLGAAMEEALILDNK
>	sp|P16086|SPT Spectrin alpha chain, brain OS=Rattus norvegicus GN=Sptan1 PE=1 SV=2	KIEDLGAAMEEALILDNK
>	sp|P16086|SPT Spectrin alpha chain, brain OS=Rattus norvegicus GN=Sptan1 PE=1 SV=2	KIEDLGAAMEEALILDNK
>	sp|P16086|SPT Spectrin alpha chain, brain OS=Rattus norvegicus GN=Sptan1 PE=1 SV=2	KIEDLGAAMEEALILDNK
>	sp|P16086|SPT Spectrin alpha chain, brain OS=Rattus norvegicus GN=Sptan1 PE=1 SV=2	KIEDLGAAMEEALILDNK
>	sp|P16086|SPT Spectrin alpha chain, brain OS=Rattus norvegicus GN=Sptan1 PE=1 SV=2	KIEDLGAAMEEALILDNK
>	sp|P16086|SPT Spectrin alpha chain, brain OS=Rattus norvegicus GN=Sptan1 PE=1 SV=2	KIEDLGAAMEEALILDNK
>	sp|P16086|SPT Spectrin alpha chain, brain OS=Rattus norvegicus GN=Sptan1 PE=1 SV=2	KIEDLGAAMEEALILDNK
>	sp|P16086|SPT Spectrin alpha chain, brain OS=Rattus norvegicus GN=Sptan1 PE=1 SV=2	KIEDLGAAMEEALILDNK
>	sp|P16086|SPT Spectrin alpha chain, brain OS=Rattus norvegicus GN=Sptan1 PE=1 SV=2	KIEDLGAAMEEALILDNK
>	sp|P16086|SPT Spectrin alpha chain, brain OS=Rattus norvegicus GN=Sptan1 PE=1 SV=2	KIEDLGAAMEEALILDNK
>	sp|P16086|SPT Spectrin alpha chain, brain OS=Rattus norvegicus GN=Sptan1 PE=1 SV=2	KIEDLGAAMEEALILDNK
>	sp|P16086|SPT Spectrin alpha chain, brain OS=Rattus norvegicus GN=Sptan1 PE=1 SV=2	KIEDLGAAMEEALILDNK
>	sp|P16086|SPT Spectrin alpha chain, brain OS=Rattus norvegicus GN=Sptan1 PE=1 SV=2	KIEDLGAAMEEALILDNK
>	sp|P16086|SPT Spectrin alpha chain, brain OS=Rattus norvegicus GN=Sptan1 PE=1 SV=2	KIEDLGAAMEEALILDNK
>	sp|P16086|SPT Spectrin alpha chain, brain OS=Rattus norvegicus GN=Sptan1 PE=1 SV=2	KIEDLGAAMEEALILDNK
>	sp|P16086|SPT Spectrin alpha chain, brain OS=Rattus norvegicus GN=Sptan1 PE=1 SV=2	KVEDLFLTFAK
>	sp|P16086|SPT Spectrin alpha chain, brain OS=Rattus norvegicus GN=Sptan1 PE=1 SV=2	LAALADQWQFLVQK
>	sp|P16086|SPT Spectrin alpha chain, brain OS=Rattus norvegicus GN=Sptan1 PE=1 SV=2	LAALADQWQFLVQK
>	sp|P16086|SPT Spectrin alpha chain, brain OS=Rattus norvegicus GN=Sptan1 PE=1 SV=2	LAQFVEHWK
>	sp|P16086|SPT Spectrin alpha chain, brain OS=Rattus norvegicus GN=Sptan1 PE=1 SV=2	LIQSHPESAEDLKEK
>	sp|P16086|SPT Spectrin alpha chain, brain OS=Rattus norvegicus GN=Sptan1 PE=1 SV=2	LIQSHPESAEDLKEK
>	sp|P16086|SPT Spectrin alpha chain, brain OS=Rattus norvegicus GN=Sptan1 PE=1 SV=2	MMLDHCLELQLFHR
>	sp|P16086|SPT Spectrin alpha chain, brain OS=Rattus norvegicus GN=Sptan1 PE=1 SV=2	MMLDHCLELQLFHR
>	sp|P16086|SPT Spectrin alpha chain, brain OS=Rattus norvegicus GN=Sptan1 PE=1 SV=2	MNEVISLWK
>	sp|P16086|SPT Spectrin alpha chain, brain OS=Rattus norvegicus GN=Sptan1 PE=1 SV=2	MQHNLEQQIQAR
>	sp|P16086|SPT Spectrin alpha chain, brain OS=Rattus norvegicus GN=Sptan1 PE=1 SV=2	MQHNLEQQIQAR
>	sp|P16086|SPT Spectrin alpha chain, brain OS=Rattus norvegicus GN=Sptan1 PE=1 SV=2	MQHNLEQQIQAR
>	sp|P16086|SPT Spectrin alpha chain, brain OS=Rattus norvegicus GN=Sptan1 PE=1 SV=2	MTLVASEDYGDTLAAIQGLLK
>	sp|P16086|SPT Spectrin alpha chain, brain OS=Rattus norvegicus GN=Sptan1 PE=1 SV=2	NVEDIELWLYEVEGHLASDDYGKDLTNVQNLQK
>	sp|P16086|SPT Spectrin alpha chain, brain OS=Rattus norvegicus GN=Sptan1 PE=1 SV=2	QEFAQHANAFHQWIQETR
>	sp|P16086|SPT Spectrin alpha chain, brain OS=Rattus norvegicus GN=Sptan1 PE=1 SV=2	QETFDAGLQAFQQEGIANITALKDQLLAAK
>	sp|P16086|SPT Spectrin alpha chain, brain OS=Rattus norvegicus GN=Sptan1 PE=1 SV=2	REELITNWEQIR
>	sp|P16086|SPT Spectrin alpha chain, brain OS=Rattus norvegicus GN=Sptan1 PE=1 SV=2	REELITNWEQIR
>	sp|P16086|SPT Spectrin alpha chain, brain OS=Rattus norvegicus GN=Sptan1 PE=1 SV=2	RLEAELAAHEPAIQGVLDTGKK
>	sp|P16086|SPT Spectrin alpha chain, brain OS=Rattus norvegicus GN=Sptan1 PE=1 SV=2	RLEAELAAHEPAIQGVLDTGKK
>	sp|P16086|SPT Spectrin alpha chain, brain OS=Rattus norvegicus GN=Sptan1 PE=1 SV=2	RLEAELAAHEPAIQGVLDTGKK
>	sp|P16086|SPT Spectrin alpha chain, brain OS=Rattus norvegicus GN=Sptan1 PE=1 SV=2	RLEAELAAHEPAIQGVLDTGKK
>	sp|P16086|SPT Spectrin alpha chain, brain OS=Rattus norvegicus GN=Sptan1 PE=1 SV=2	RLEAELAAHEPAIQGVLDTGKK
>	sp|P16086|SPT Spectrin alpha chain, brain OS=Rattus norvegicus GN=Sptan1 PE=1 SV=2	RLEAELAAHEPAIQGVLDTGKK
>	sp|P16086|SPT Spectrin alpha chain, brain OS=Rattus norvegicus GN=Sptan1 PE=1 SV=2	RQDLEDSLQAQQYFADANEAESWMR
>	sp|P16086|SPT Spectrin alpha chain, brain OS=Rattus norvegicus GN=Sptan1 PE=1 SV=2	SADESGQALLAAGHYASDEVR
>	sp|P16086|SPT Spectrin alpha chain, brain OS=Rattus norvegicus GN=Sptan1 PE=1 SV=2	SADESGQALLAAGHYASDEVR
>	sp|P16086|SPT Spectrin alpha chain, brain OS=Rattus norvegicus GN=Sptan1 PE=1 SV=2	TKQEEVNAAWQR
>	sp|P16086|SPT Spectrin alpha chain, brain OS=Rattus norvegicus GN=Sptan1 PE=1 SV=2	AQLADSFHLQQFFR
>	sp|P16086|SPT Spectrin alpha chain, brain OS=Rattus norvegicus GN=Sptan1 PE=1 SV=2	MMLDHCLELQLFHR
>	sp|P16086|SPT Spectrin alpha chain, brain OS=Rattus norvegicus GN=Sptan1 PE=1 SV=2	GVIDMGNSLIER
>	sp|P16086|SPT Spectrin alpha chain, brain OS=Rattus norvegicus GN=Sptan1 PE=1 SV=2	ITALDEFATK
>	sp|P16086|SPT Spectrin alpha chain, brain OS=Rattus norvegicus GN=Sptan1 PE=1 SV=2	RQDLEDSLQAQQYFADANEAESWMR
>	sp|P16086|SPT Spectrin alpha chain, brain OS=Rattus norvegicus GN=Sptan1 PE=1 SV=2	HQALQAEIAGHEPR
>	sp|P16086|SPT Spectrin alpha chain, brain OS=Rattus norvegicus GN=Sptan1 PE=1 SV=2	LGESQTLQQFSR

>	sp|P16086|SPT	Spectrin alpha chain, brain OS=Rattus norvegicus GN=Sptan1 PE=1 SV=2	LDETGNLMISEGHFASETIR
>	sp|P16086|SPT	Spectrin alpha chain, brain OS=Rattus norvegicus GN=Sptan1 PE=1 SV=2	LLEATELK
>	sp|P16086|SPT	Spectrin alpha chain, brain OS=Rattus norvegicus GN=Sptan1 PE=1 SV=2	LGESQTLQQFSR
>	sp|P16086|SPT	Spectrin alpha chain, brain OS=Rattus norvegicus GN=Sptan1 PE=1 SV=2	MMLDHCLELQLFHR
>	sp|P16086|SPT	Spectrin alpha chain, brain OS=Rattus norvegicus GN=Sptan1 PE=1 SV=2	MMLDHCLELQLFHR
>	sp|P16086|SPT	Spectrin alpha chain, brain OS=Rattus norvegicus GN=Sptan1 PE=1 SV=2	GVIDMGNSLIER
>	sp|P16086|SPT	Spectrin alpha chain, brain OS=Rattus norvegicus GN=Sptan1 PE=1 SV=2	RQDLEDSLQAQQYFADANEAESWMR
>	sp|P16086|SPT	Spectrin alpha chain, brain OS=Rattus norvegicus GN=Sptan1 PE=1 SV=2	TKQEEVNAAWQR
>	sp|P16086|SPT	Spectrin alpha chain, brain OS=Rattus norvegicus GN=Sptan1 PE=1 SV=2	MMLDHCLELQLFHR
>	sp|P16086|SPT	Spectrin alpha chain, brain OS=Rattus norvegicus GN=Sptan1 PE=1 SV=2	AGTFQAFEQFGQQLLAHGHYASPEIK
>	sp|P16086|SPT	Spectrin alpha chain, brain OS=Rattus norvegicus GN=Sptan1 PE=1 SV=2	LAQFVEHWK
>	sp|P16086|SPT	Spectrin alpha chain, brain OS=Rattus norvegicus GN=Sptan1 PE=1 SV=2	DLSSVQTLLTK
>	sp|P16086|SPT	Spectrin alpha chain, brain OS=Rattus norvegicus GN=Sptan1 PE=1 SV=2	VNEVNQFAAK
>	sp|P16086|SPT	Spectrin alpha chain, brain OS=Rattus norvegicus GN=Sptan1 PE=1 SV=2	ADVVESWIGEK
>	sp|P16086|SPT	Spectrin alpha chain, brain OS=Rattus norvegicus GN=Sptan1 PE=1 SV=2	NVEDIELWLYEVEGHLASDDYGK
>	sp|P16086|SPT	Spectrin alpha chain, brain OS=Rattus norvegicus GN=Sptan1 PE=1 SV=2	GKDLIGVQNLLK
>	sp|P16086|SPT	Spectrin alpha chain, brain OS=Rattus norvegicus GN=Sptan1 PE=1 SV=2	SADESGQALLAAGHYASDEVR
>	sp|P16086|SPT	Spectrin alpha chain, brain OS=Rattus norvegicus GN=Sptan1 PE=1 SV=2	SADESGQALLAAGHYASDEVR
>	sp|P16086|SPT	Spectrin alpha chain, brain OS=Rattus norvegicus GN=Sptan1 PE=1 SV=2	FKELSTLR
>	sp|P16086|SPT	Spectrin alpha chain, brain OS=Rattus norvegicus GN=Sptan1 PE=1 SV=2	MMLDHCLELQLFHR
>	sp|P16086|SPT	Spectrin alpha chain, brain OS=Rattus norvegicus GN=Sptan1 PE=1 SV=2	DLTGVQNLR
>	sp|P16086|SPT	Spectrin alpha chain, brain OS=Rattus norvegicus GN=Sptan1 PE=1 SV=2	AQLADSFHLQQFFR
>	sp|P16086|SPT	Spectrin alpha chain, brain OS=Rattus norvegicus GN=Sptan1 PE=1 SV=2	AGTFQAFEQFGQQLLAHGHYASPEIK
>	sp|P16086|SPT	Spectrin alpha chain, brain OS=Rattus norvegicus GN=Sptan1 PE=1 SV=2	KFEEFQTDLAAHEER
>	sp|P16086|SPT	Spectrin alpha chain, brain OS=Rattus norvegicus GN=Sptan1 PE=1 SV=2	SLQQLAEER
>	sp|P16086|SPT	Spectrin alpha chain, brain OS=Rattus norvegicus GN=Sptan1 PE=1 SV=2	ITALDEFATK
>	sp|P16086|SPT	Spectrin alpha chain, brain OS=Rattus norvegicus GN=Sptan1 PE=1 SV=2	AGTFQAFEQFGQQLLAHGHYASPEIK
>	sp|P16086|SPT	Spectrin alpha chain, brain OS=Rattus norvegicus GN=Sptan1 PE=1 SV=2	KFEEFQTDLAAHEER
>	sp|P16086|SPT	Spectrin alpha chain, brain OS=Rattus norvegicus GN=Sptan1 PE=1 SV=2	DLAALGDK
>	sp|P16086|SPT	Spectrin alpha chain, brain OS=Rattus norvegicus GN=Sptan1 PE=1 SV=2	SSEEIESAFR
>	sp|P16086|SPT	Spectrin alpha chain, brain OS=Rattus norvegicus GN=Sptan1 PE=1 SV=2	DVDEIEAWISEK
>	sp|P16086|SPT	Spectrin alpha chain, brain OS=Rattus norvegicus GN=Sptan1 PE=1 SV=2	AQLADSFHLQQFFR
>	sp|P16086|SPT	Spectrin alpha chain, brain OS=Rattus norvegicus GN=Sptan1 PE=1 SV=2	NQALNTDNYGHDLASVQALQR
>	sp|P16086|SPT	Spectrin alpha chain, brain OS=Rattus norvegicus GN=Sptan1 PE=1 SV=2	AQLADSFHLQQFFR
>	sp|P16086|SPT	Spectrin alpha chain, brain OS=Rattus norvegicus GN=Sptan1 PE=1 SV=2	DLAALEDKVK
>	sp|P16086|SPT	Spectrin alpha chain, brain OS=Rattus norvegicus GN=Sptan1 PE=1 SV=2	DVDETIGWIK
>	sp|P16086|SPT	Spectrin alpha chain, brain OS=Rattus norvegicus GN=Sptan1 PE=1 SV=2	EKEQLMASDDFGR
>	sp|P16086|SPT	Spectrin alpha chain, brain OS=Rattus norvegicus GN=Sptan1 PE=1 SV=2	ELPTAFDYVEFTR
>	sp|P16086|SPT	Spectrin alpha chain, brain OS=Rattus norvegicus GN=Sptan1 PE=1 SV=2	KFEEFQTDLAAHEER
>	sp|P16086|SPT	Spectrin alpha chain, brain OS=Rattus norvegicus GN=Sptan1 PE=1 SV=2	QEFAQHANAFHQWIQETR
>	sp|P16086|SPT	Spectrin alpha chain, brain OS=Rattus norvegicus GN=Sptan1 PE=1 SV=2	ELPTAFDYVEFTR
>	sp|P16086|SPT	Spectrin alpha chain, brain OS=Rattus norvegicus GN=Sptan1 PE=1 SV=2	KIEDLGAAMEEALILDNKYTEHSTVGLAQQWDQLDQLGMR
>	sp|P16086|SPT	Spectrin alpha chain, brain OS=Rattus norvegicus GN=Sptan1 PE=1 SV=2	DADETKEWIEEK
>	sp|P16086|SPT	Spectrin alpha chain, brain OS=Rattus norvegicus GN=Sptan1 PE=1 SV=2	DADETKEWIEEK
>	sp|P16086|SPT	Spectrin alpha chain, brain OS=Rattus norvegicus GN=Sptan1 PE=1 SV=2	KGDILTLLNSTNKDWWK
>	sp|P16086|SPT	Spectrin alpha chain, brain OS=Rattus norvegicus GN=Sptan1 PE=1 SV=2	ELPTAFDYVEFTR
>	sp|P16086|SPT	Spectrin alpha chain, brain OS=Rattus norvegicus GN=Sptan1 PE=1 SV=2	EKEPIVGSTDYGKDEDSAEALLK
>	sp|P16086|SPT	Spectrin alpha chain, brain OS=Rattus norvegicus GN=Sptan1 PE=1 SV=2	FNRDVDETIGWIK
>	sp|P16086|SPT	Spectrin alpha chain, brain OS=Rattus norvegicus GN=Sptan1 PE=1 SV=2	AQLADSFHLQQFFR
>	sp|P16086|SPT	Spectrin alpha chain, brain OS=Rattus norvegicus GN=Sptan1 PE=1 SV=2	ALINADELANDVAGAEALLDRHQEHKGEIDAHEDSFK
>	sp|P16086|SPT	Spectrin alpha chain, brain OS=Rattus norvegicus GN=Sptan1 PE=1 SV=2	QQVAPMDDETGKELVLALYDYQEK
>	sp|P16086|SPT	Spectrin alpha chain, brain OS=Rattus norvegicus GN=Sptan1 PE=1 SV=2	QEFAQHANAFHQWIQETR
>	sp|P16086|SPT	Spectrin alpha chain, brain OS=Rattus norvegicus GN=Sptan1 PE=1 SV=2	GVIDMGNSLIER
>	sp|P16086|SPT	Spectrin alpha chain, brain OS=Rattus norvegicus GN=Sptan1 PE=1 SV=2	LFGAAEVQR
>	sp|P16086|SPT	Spectrin alpha chain, brain OS=Rattus norvegicus GN=Sptan1 PE=1 SV=2	QQVAPMDDETGKELVLALYDYQEK
>	sp|P16086|SPT	Spectrin alpha chain, brain OS=Rattus norvegicus GN=Sptan1 PE=1 SV=2	HALLEADVAAHQDR
>	sp|P16086|SPT	Spectrin alpha chain, brain OS=Rattus norvegicus GN=Sptan1 PE=1 SV=2	KIEDLGAAMEEALILDNKYTEHSTVGLAQQWDQLDQLGMR
>	sp|P16086|SPT	Spectrin alpha chain, brain OS=Rattus norvegicus GN=Sptan1 PE=1 SV=2	HQAFEAELHANADR
>	sp|P16086|SPT	Spectrin alpha chain, brain OS=Rattus norvegicus GN=Sptan1 PE=1 SV=2	NVEDIELWLYEVEGHLASDDYGKDLTNVQNLQK
>	sp|P16086|SPT	Spectrin alpha chain, brain OS=Rattus norvegicus GN=Sptan1 PE=1 SV=2	KASAFNSWFENAEEDLTDPVR
>	sp|P16086|SPT	Spectrin alpha chain, brain OS=Rattus norvegicus GN=Sptan1 PE=1 SV=2	LIQEQHPEEELIK
>	sp|P16086|SPT	Spectrin alpha chain, brain OS=Rattus norvegicus GN=Sptan1 PE=1 SV=2	KGDILTLLNSTNKDWWK
>	sp|P16086|SPT	Spectrin alpha chain, brain OS=Rattus norvegicus GN=Sptan1 PE=1 SV=2	ECEDVMDWINDKEAIVTSEELGQDLEHVEVLQK
>	sp|P11442|CLH	Clathrin heavy chain 1 OS=Rattus norvegicus GN=Cltc PE=1 SV=3	ADDPSSYMEVVQAANTSGNWEELVK
>	sp|P11442|CLH	Clathrin heavy chain 1 OS=Rattus norvegicus GN=Cltc PE=1 SV=3	AFMTADLPNELIELLEK
>	sp|P11442|CLH	Clathrin heavy chain 1 OS=Rattus norvegicus GN=Cltc PE=1 SV=3	AHMGMFTELAILYSK
>	sp|P11442|CLH	Clathrin heavy chain 1 OS=Rattus norvegicus GN=Cltc PE=1 SV=3	DAMQYASESKDTELAEELLQWFLQEEKR
>	sp|P11442|CLH	Clathrin heavy chain 1 OS=Rattus norvegicus GN=Cltc PE=1 SV=3	EHLELFWSR
>	sp|P11442|CLH	Clathrin heavy chain 1 OS=Rattus norvegicus GN=Cltc PE=1 SV=3	IHEGCEEPATHNALAK
>	sp|P11442|CLH	Clathrin heavy chain 1 OS=Rattus norvegicus GN=Cltc PE=1 SV=3	ISGETIFVTAPHEATAGIIGVNR
>	sp|P11442|CLH	Clathrin heavy chain 1 OS=Rattus norvegicus GN=Cltc PE=1 SV=3	KFDVNTSAVQVLIEHIGNLDR
>	sp|P11442|CLH	Clathrin heavy chain 1 OS=Rattus norvegicus GN=Cltc PE=1 SV=3	KGQVLSVCVEEENIIPYITNVLQNPDLALR
>	sp|P11442|CLH	Clathrin heavy chain 1 OS=Rattus norvegicus GN=Cltc PE=1 SV=3	KVGYTPDWIFLLR
>	sp|P11442|CLH	Clathrin heavy chain 1 OS=Rattus norvegicus GN=Cltc PE=1 SV=3	LASTLVHLGEYQAAVDGAR

>	sp|P11442|CLH Clathrin heavy chain 1 OS=Rattus norvegicus GN=Cltc PE=1 SV=3	LEKHELIEFR
>	sp|P11442|CLH Clathrin heavy chain 1 OS=Rattus norvegicus GN=Cltc PE=1 SV=3	LLEMNLMHAPQVADAILGNQMFTHYDR
>	sp|P11442|CLH Clathrin heavy chain 1 OS=Rattus norvegicus GN=Cltc PE=1 SV=3	LLLPWLEAR
>	sp|P11442|CLH Clathrin heavy chain 1 OS=Rattus norvegicus GN=Cltc PE=1 SV=3	MREHLELFWSR
>	sp|P11442|CLH Clathrin heavy chain 1 OS=Rattus norvegicus GN=Cltc PE=1 SV=3	NLQNLLILTAIK
>	sp|P11442|CLH Clathrin heavy chain 1 OS=Rattus norvegicus GN=Cltc PE=1 SV=3	NNLAGAEELFAR
>	sp|P11442|CLH Clathrin heavy chain 1 OS=Rattus norvegicus GN=Cltc PE=1 SV=3	RDPHLACVAYER
>	sp|P11442|CLH Clathrin heavy chain 1 OS=Rattus norvegicus GN=Cltc PE=1 SV=3	RKDPELWGSVLLESNPYR
>	sp|P11442|CLH Clathrin heavy chain 1 OS=Rattus norvegicus GN=Cltc PE=1 SV=3	RPISADSAIMNPASK
>	sp|P11442|CLH Clathrin heavy chain 1 OS=Rattus norvegicus GN=Cltc PE=1 SV=3	RPLIDQVVQTALSETQDPEEVSVTVK
>	sp|P11442|CLH Clathrin heavy chain 1 OS=Rattus norvegicus GN=Cltc PE=1 SV=3	SVDPTLALSVYLR
>	sp|P11442|CLH Clathrin heavy chain 1 OS=Rattus norvegicus GN=Cltc PE=1 SV=3	TSIDAYDNFDNISLAQR
>	sp|P11442|CLH Clathrin heavy chain 1 OS=Rattus norvegicus GN=Cltc PE=1 SV=3	VGEQAQVVIIDMNDPSNPIR
>	sp|P11442|CLH Clathrin heavy chain 1 OS=Rattus norvegicus GN=Cltc PE=1 SV=3	WLLLTGISAQQNR
>	sp|P11442|CLH Clathrin heavy chain 1 OS=Rattus norvegicus GN=Cltc PE=1 SV=3	KDPELWGSVLLESNPYR
>	sp|P11442|CLH Clathrin heavy chain 1 OS=Rattus norvegicus GN=Cltc PE=1 SV=3	ESYVETELIFALAK
>	sp|P11442|CLH Clathrin heavy chain 1 OS=Rattus norvegicus GN=Cltc PE=1 SV=3	GQFSTDELVAEVEKR
>	sp|P11442|CLH Clathrin heavy chain 1 OS=Rattus norvegicus GN=Cltc PE=1 SV=3	WLKEDKLECSEELGDLVK
>	sp|P11442|CLH Clathrin heavy chain 1 OS=Rattus norvegicus GN=Cltc PE=1 SV=3	GQCDLELINVCNENSLFK
>	sp|P11442|CLH Clathrin heavy chain 1 OS=Rattus norvegicus GN=Cltc PE=1 SV=3	HSSLAGCQIINYR
>	sp|P11442|CLH Clathrin heavy chain 1 OS=Rattus norvegicus GN=Cltc PE=1 SV=3	KFNALFAQGNYSEAAK
>	sp|P11442|CLH Clathrin heavy chain 1 OS=Rattus norvegicus GN=Cltc PE=1 SV=3	TLQIFNIEMK
>	sp|P11442|CLH Clathrin heavy chain 1 OS=Rattus norvegicus GN=Cltc PE=1 SV=3	CNEPAVWSQLAK
>	sp|P11442|CLH Clathrin heavy chain 1 OS=Rattus norvegicus GN=Cltc PE=1 SV=3	SVNESLNNLFITEEDYQALRTSI
>	sp|P11442|CLH Clathrin heavy chain 1 OS=Rattus norvegicus GN=Cltc PE=1 SV=3	ADDPSSYMEVVQAANTSGNWEELVK
>	sp|P11442|CLH Clathrin heavy chain 1 OS=Rattus norvegicus GN=Cltc PE=1 SV=3	AFMTADLPNELIELLEK
>	sp|P11442|CLH Clathrin heavy chain 1 OS=Rattus norvegicus GN=Cltc PE=1 SV=3	AHMGMFTELAILYSK
>	sp|P11442|CLH Clathrin heavy chain 1 OS=Rattus norvegicus GN=Cltc PE=1 SV=3	AHMGMFTELAILYSK
>	sp|P11442|CLH Clathrin heavy chain 1 OS=Rattus norvegicus GN=Cltc PE=1 SV=3	EHLELFWSR
>	sp|P11442|CLH Clathrin heavy chain 1 OS=Rattus norvegicus GN=Cltc PE=1 SV=3	EHLELFWSR
>	sp|P11442|CLH Clathrin heavy chain 1 OS=Rattus norvegicus GN=Cltc PE=1 SV=3	ESYVETELIFALAK
>	sp|P11442|CLH Clathrin heavy chain 1 OS=Rattus norvegicus GN=Cltc PE=1 SV=3	ESYVETELIFALAK
>	sp|P11442|CLH Clathrin heavy chain 1 OS=Rattus norvegicus GN=Cltc PE=1 SV=3	ESYVETELIFALAK
>	sp|P11442|CLH Clathrin heavy chain 1 OS=Rattus norvegicus GN=Cltc PE=1 SV=3	ESYVETELIFALAK
>	sp|P11442|CLH Clathrin heavy chain 1 OS=Rattus norvegicus GN=Cltc PE=1 SV=3	GQCDLELINVCNENSLFK
>	sp|P11442|CLH Clathrin heavy chain 1 OS=Rattus norvegicus GN=Cltc PE=1 SV=3	IHEGCEEPATHNALAK
>	sp|P11442|CLH Clathrin heavy chain 1 OS=Rattus norvegicus GN=Cltc PE=1 SV=3	IHEGCEEPATHNALAK
>	sp|P11442|CLH Clathrin heavy chain 1 OS=Rattus norvegicus GN=Cltc PE=1 SV=3	IHEGCEEPATHNALAK
>	sp|P11442|CLH Clathrin heavy chain 1 OS=Rattus norvegicus GN=Cltc PE=1 SV=3	IHEGCEEPATHNALAK
>	sp|P11442|CLH Clathrin heavy chain 1 OS=Rattus norvegicus GN=Cltc PE=1 SV=3	IHEGCEEPATHNALAK
>	sp|P11442|CLH Clathrin heavy chain 1 OS=Rattus norvegicus GN=Cltc PE=1 SV=3	IHEGCEEPATHNALAK
>	sp|P11442|CLH Clathrin heavy chain 1 OS=Rattus norvegicus GN=Cltc PE=1 SV=3	ISGETIFVTAPHEATAGIIGVNR
>	sp|P11442|CLH Clathrin heavy chain 1 OS=Rattus norvegicus GN=Cltc PE=1 SV=3	ISGETIFVTAPHEATAGIIGVNR
>	sp|P11442|CLH Clathrin heavy chain 1 OS=Rattus norvegicus GN=Cltc PE=1 SV=3	ISGETIFVTAPHEATAGIIGVNR
>	sp|P11442|CLH Clathrin heavy chain 1 OS=Rattus norvegicus GN=Cltc PE=1 SV=3	KDPELWGSVLLESNPYR
>	sp|P11442|CLH Clathrin heavy chain 1 OS=Rattus norvegicus GN=Cltc PE=1 SV=3	KDPELWGSVLLESNPYR
>	sp|P11442|CLH Clathrin heavy chain 1 OS=Rattus norvegicus GN=Cltc PE=1 SV=3	KFDVNTSAVQVLIEHIGNLDR
>	sp|P11442|CLH Clathrin heavy chain 1 OS=Rattus norvegicus GN=Cltc PE=1 SV=3	KFDVNTSAVQVLIEHIGNLDR
>	sp|P11442|CLH Clathrin heavy chain 1 OS=Rattus norvegicus GN=Cltc PE=1 SV=3	KFDVNTSAVQVLIEHIGNLDR
>	sp|P11442|CLH Clathrin heavy chain 1 OS=Rattus norvegicus GN=Cltc PE=1 SV=3	KFDVNTSAVQVLIEHIGNLDR
>	sp|P11442|CLH Clathrin heavy chain 1 OS=Rattus norvegicus GN=Cltc PE=1 SV=3	KFDVNTSAVQVLIEHIGNLDR
>	sp|P11442|CLH Clathrin heavy chain 1 OS=Rattus norvegicus GN=Cltc PE=1 SV=3	KGQVLSVCVEEENIIPYITNVLQNPDLALR
>	sp|P11442|CLH Clathrin heavy chain 1 OS=Rattus norvegicus GN=Cltc PE=1 SV=3	KGQVLSVCVEEENIIPYITNVLQNPDLALR
>	sp|P11442|CLH Clathrin heavy chain 1 OS=Rattus norvegicus GN=Cltc PE=1 SV=3	KVGYTPDWIFLLR
>	sp|P11442|CLH Clathrin heavy chain 1 OS=Rattus norvegicus GN=Cltc PE=1 SV=3	KVGYTPDWIFLLR
>	sp|P11442|CLH Clathrin heavy chain 1 OS=Rattus norvegicus GN=Cltc PE=1 SV=3	KVGYTPDWIFLLR
>	sp|P11442|CLH Clathrin heavy chain 1 OS=Rattus norvegicus GN=Cltc PE=1 SV=3	KVGYTPDWIFLLR
>	sp|P11442|CLH Clathrin heavy chain 1 OS=Rattus norvegicus GN=Cltc PE=1 SV=3	KVGYTPDWIFLLR
>	sp|P11442|CLH Clathrin heavy chain 1 OS=Rattus norvegicus GN=Cltc PE=1 SV=3	LASTLVHLGEYQAAVDGAR
>	sp|P11442|CLH Clathrin heavy chain 1 OS=Rattus norvegicus GN=Cltc PE=1 SV=3	LASTLVHLGEYQAAVDGAR
>	sp|P11442|CLH Clathrin heavy chain 1 OS=Rattus norvegicus GN=Cltc PE=1 SV=3	LASTLVHLGEYQAAVDGAR
>	sp|P11442|CLH Clathrin heavy chain 1 OS=Rattus norvegicus GN=Cltc PE=1 SV=3	LEKHELIEFR
>	sp|P11442|CLH Clathrin heavy chain 1 OS=Rattus norvegicus GN=Cltc PE=1 SV=3	LEKHELIEFR
>	sp|P11442|CLH Clathrin heavy chain 1 OS=Rattus norvegicus GN=Cltc PE=1 SV=3	LLEMNLMHAPQVADAILGNQMFTHYDR
>	sp|P11442|CLH Clathrin heavy chain 1 OS=Rattus norvegicus GN=Cltc PE=1 SV=3	LLEMNLMHAPQVADAILGNQMFTHYDR
>	sp|P11442|CLH Clathrin heavy chain 1 OS=Rattus norvegicus GN=Cltc PE=1 SV=3	LLEMNLMHAPQVADAILGNQMFTHYDR
>	sp|P11442|CLH Clathrin heavy chain 1 OS=Rattus norvegicus GN=Cltc PE=1 SV=3	LLEMNLMHAPQVADAILGNQMFTHYDR
>	sp|P11442|CLH Clathrin heavy chain 1 OS=Rattus norvegicus GN=Cltc PE=1 SV=3	LLEMNLMHAPQVADAILGNQMFTHYDR
>	sp|P11442|CLH Clathrin heavy chain 1 OS=Rattus norvegicus GN=Cltc PE=1 SV=3	LLEMNLMHAPQVADAILGNQMFTHYDR
>	sp|P11442|CLH Clathrin heavy chain 1 OS=Rattus norvegicus GN=Cltc PE=1 SV=3	LLEMNLMHAPQVADAILGNQMFTHYDR
>	sp|P11442|CLH Clathrin heavy chain 1 OS=Rattus norvegicus GN=Cltc PE=1 SV=3	LLEMNLMHAPQVADAILGNQMFTHYDR
>	sp|P11442|CLH Clathrin heavy chain 1 OS=Rattus norvegicus GN=Cltc PE=1 SV=3	LLEMNLMHAPQVADAILGNQMFTHYDR
>	sp|P11442|CLH Clathrin heavy chain 1 OS=Rattus norvegicus GN=Cltc PE=1 SV=3	LLEMNLMHAPQVADAILGNQMFTHYDR
>	sp|P11442|CLH Clathrin heavy chain 1 OS=Rattus norvegicus GN=Cltc PE=1 SV=3	LLEMNLMHAPQVADAILGNQMFTHYDR

>	sp|P11442|CLH	Clathrin heavy chain 1 OS=Rattus norvegicus GN=Cltc PE=1 SV=3	LLEMNLMHAPQVADAILGNQMFTHYDR
>	sp|P11442|CLH	Clathrin heavy chain 1 OS=Rattus norvegicus GN=Cltc PE=1 SV=3	LLEMNLMHAPQVADAILGNQMFTHYDR
>	sp|P11442|CLH	Clathrin heavy chain 1 OS=Rattus norvegicus GN=Cltc PE=1 SV=3	LLEMNLMHAPQVADAILGNQMFTHYDR
>	sp|P11442|CLH	Clathrin heavy chain 1 OS=Rattus norvegicus GN=Cltc PE=1 SV=3	LLEMNLMHAPQVADAILGNQMFTHYDR
>	sp|P11442|CLH	Clathrin heavy chain 1 OS=Rattus norvegicus GN=Cltc PE=1 SV=3	LLEMNLMHAPQVADAILGNQMFTHYDR
>	sp|P11442|CLH	Clathrin heavy chain 1 OS=Rattus norvegicus GN=Cltc PE=1 SV=3	MREHLELFWSR
>	sp|P11442|CLH	Clathrin heavy chain 1 OS=Rattus norvegicus GN=Cltc PE=1 SV=3	RKDPELWGSVLLESNPYR
>	sp|P11442|CLH	Clathrin heavy chain 1 OS=Rattus norvegicus GN=Cltc PE=1 SV=3	RKDPELWGSVLLESNPYR
>	sp|P11442|CLH	Clathrin heavy chain 1 OS=Rattus norvegicus GN=Cltc PE=1 SV=3	RPLIDQVVQTALSETQDPEEVSVTVK
>	sp|P11442|CLH	Clathrin heavy chain 1 OS=Rattus norvegicus GN=Cltc PE=1 SV=3	RPLIDQVVQTALSETQDPEEVSVTVK
>	sp|P11442|CLH	Clathrin heavy chain 1 OS=Rattus norvegicus GN=Cltc PE=1 SV=3	RPLIDQVVQTALSETQDPEEVSVTVK
>	sp|P11442|CLH	Clathrin heavy chain 1 OS=Rattus norvegicus GN=Cltc PE=1 SV=3	RPLIDQVVQTALSETQDPEEVSVTVK
>	sp|P11442|CLH	Clathrin heavy chain 1 OS=Rattus norvegicus GN=Cltc PE=1 SV=3	RPLIDQVVQTALSETQDPEEVSVTVK
>	sp|P11442|CLH	Clathrin heavy chain 1 OS=Rattus norvegicus GN=Cltc PE=1 SV=3	RPLIDQVVQTALSETQDPEEVSVTVK
>	sp|P11442|CLH	Clathrin heavy chain 1 OS=Rattus norvegicus GN=Cltc PE=1 SV=3	RPLIDQVVQTALSETQDPEEVSVTVK
>	sp|P11442|CLH	Clathrin heavy chain 1 OS=Rattus norvegicus GN=Cltc PE=1 SV=3	RPLIDQVVQTALSETQDPEEVSVTVK
>	sp|P11442|CLH	Clathrin heavy chain 1 OS=Rattus norvegicus GN=Cltc PE=1 SV=3	SVDPTLALSVYLR
>	sp|P11442|CLH	Clathrin heavy chain 1 OS=Rattus norvegicus GN=Cltc PE=1 SV=3	TSIDAYDNFDNISLAQR
>	sp|P11442|CLH	Clathrin heavy chain 1 OS=Rattus norvegicus GN=Cltc PE=1 SV=3	TSIDAYDNFDNISLAQR
>	sp|P11442|CLH	Clathrin heavy chain 1 OS=Rattus norvegicus GN=Cltc PE=1 SV=3	WLLLTGISAQQNR
>	sp|P11442|CLH	Clathrin heavy chain 1 OS=Rattus norvegicus GN=Cltc PE=1 SV=3	LHIIEVGTPPTGNQPFPK
>	sp|P11442|CLH	Clathrin heavy chain 1 OS=Rattus norvegicus GN=Cltc PE=1 SV=3	IHEGCEEPATHNALAK
>	sp|P11442|CLH	Clathrin heavy chain 1 OS=Rattus norvegicus GN=Cltc PE=1 SV=3	RPISADSAIMNPASK
>	sp|P11442|CLH	Clathrin heavy chain 1 OS=Rattus norvegicus GN=Cltc PE=1 SV=3	HELIEFR
>	sp|P11442|CLH	Clathrin heavy chain 1 OS=Rattus norvegicus GN=Cltc PE=1 SV=3	IHEGCEEPATHNALAK
>	sp|P11442|CLH	Clathrin heavy chain 1 OS=Rattus norvegicus GN=Cltc PE=1 SV=3	VIQCFAETGQVQK
>	sp|P11442|CLH	Clathrin heavy chain 1 OS=Rattus norvegicus GN=Cltc PE=1 SV=3	GQFSTDELVAEVEKR
>	sp|P11442|CLH	Clathrin heavy chain 1 OS=Rattus norvegicus GN=Cltc PE=1 SV=3	SVNESLNNLFITEEDYQALR
>	sp|P11442|CLH	Clathrin heavy chain 1 OS=Rattus norvegicus GN=Cltc PE=1 SV=3	KGQVLSVCVEEENIIPYITNVLQNPDLALR
>	sp|P11442|CLH	Clathrin heavy chain 1 OS=Rattus norvegicus GN=Cltc PE=1 SV=3	LHIIEVGTPPTGNQPFPK
>	sp|P11442|CLH	Clathrin heavy chain 1 OS=Rattus norvegicus GN=Cltc PE=1 SV=3	ALEHFTDLYDIK
>	sp|P11442|CLH	Clathrin heavy chain 1 OS=Rattus norvegicus GN=Cltc PE=1 SV=3	NLQNLLILTAIK
>	sp|P11442|CLH	Clathrin heavy chain 1 OS=Rattus norvegicus GN=Cltc PE=1 SV=3	SVNESLNNLFITEEDYQALR
>	sp|P11442|CLH	Clathrin heavy chain 1 OS=Rattus norvegicus GN=Cltc PE=1 SV=3	KGQVLSVCVEEENIIPYITNVLQNPDLALR
>	sp|P11442|CLH	Clathrin heavy chain 1 OS=Rattus norvegicus GN=Cltc PE=1 SV=3	GQFSTDELVAEVEKR
>	sp|P11442|CLH	Clathrin heavy chain 1 OS=Rattus norvegicus GN=Cltc PE=1 SV=3	KFNALFAQGNYSEAAK
>	sp|P11442|CLH	Clathrin heavy chain 1 OS=Rattus norvegicus GN=Cltc PE=1 SV=3	TLQIFNIEMK
>	sp|P11442|CLH	Clathrin heavy chain 1 OS=Rattus norvegicus GN=Cltc PE=1 SV=3	LHIIEVGTPPTGNQPFPK
>	sp|P11442|CLH	Clathrin heavy chain 1 OS=Rattus norvegicus GN=Cltc PE=1 SV=3	WISLNTVALVTDNAVYHWSMEGESQPVK
>	sp|P11442|CLH	Clathrin heavy chain 1 OS=Rattus norvegicus GN=Cltc PE=1 SV=3	LHIIEVGTPPTGNQPFPK
>	sp|P11442|CLH	Clathrin heavy chain 1 OS=Rattus norvegicus GN=Cltc PE=1 SV=3	RPLIDQVVQTALSETQDPEEVSVTVK
>	sp|P11442|CLH	Clathrin heavy chain 1 OS=Rattus norvegicus GN=Cltc PE=1 SV=3	EHLELFWSR
>	sp|P11442|CLH	Clathrin heavy chain 1 OS=Rattus norvegicus GN=Cltc PE=1 SV=3	VNPSRLPVVIGGLLDVDCSEDVIK
>	sp|P11442|CLH	Clathrin heavy chain 1 OS=Rattus norvegicus GN=Cltc PE=1 SV=3	KFNALFAQGNYSEAAK
>	sp|P11442|CLH	Clathrin heavy chain 1 OS=Rattus norvegicus GN=Cltc PE=1 SV=3	QLPLVKPYLR
>	sp|P11442|CLH	Clathrin heavy chain 1 OS=Rattus norvegicus GN=Cltc PE=1 SV=3	IYIDSNNNPER
>	sp|P11442|CLH	Clathrin heavy chain 1 OS=Rattus norvegicus GN=Cltc PE=1 SV=3	NLQNLLILTAIK
>	sp|P11442|CLH	Clathrin heavy chain 1 OS=Rattus norvegicus GN=Cltc PE=1 SV=3	LAELEEFINGPNNAHIQQVGDR
>	sp|P11442|CLH	Clathrin heavy chain 1 OS=Rattus norvegicus GN=Cltc PE=1 SV=3	ISGETIFVTAPHEATAGIIGVNR
>	sp|P11442|CLH	Clathrin heavy chain 1 OS=Rattus norvegicus GN=Cltc PE=1 SV=3	IAAYLFK
>	sp|P11442|CLH	Clathrin heavy chain 1 OS=Rattus norvegicus GN=Cltc PE=1 SV=3	ALEHFTDLYDIK
>	sp|P11442|CLH	Clathrin heavy chain 1 OS=Rattus norvegicus GN=Cltc PE=1 SV=3	VMEYINR
>	sp|P11442|CLH	Clathrin heavy chain 1 OS=Rattus norvegicus GN=Cltc PE=1 SV=3	KGQVLSVCVEEENIIPYITNVLQNPDLALR
>	sp|P11442|CLH	Clathrin heavy chain 1 OS=Rattus norvegicus GN=Cltc PE=1 SV=3	IVLYAK
>	sp|P11442|CLH	Clathrin heavy chain 1 OS=Rattus norvegicus GN=Cltc PE=1 SV=3	VVGAMQLYSVDR
>	sp|P11442|CLH	Clathrin heavy chain 1 OS=Rattus norvegicus GN=Cltc PE=1 SV=3	KVGYTPDWIFLLR
>	sp|P11442|CLH	Clathrin heavy chain 1 OS=Rattus norvegicus GN=Cltc PE=1 SV=3	TSIDAYDNFDNISLAQR
>	sp|P11442|CLH	Clathrin heavy chain 1 OS=Rattus norvegicus GN=Cltc PE=1 SV=3	IAAYLFK
>	sp|P11442|CLH	Clathrin heavy chain 1 OS=Rattus norvegicus GN=Cltc PE=1 SV=3	NLQNLLILTAIK
>	sp|P11442|CLH	Clathrin heavy chain 1 OS=Rattus norvegicus GN=Cltc PE=1 SV=3	GQFSTDELVAEVEKR
>	sp|P11442|CLH	Clathrin heavy chain 1 OS=Rattus norvegicus GN=Cltc PE=1 SV=3	EAIDSYIK
>	sp|P11442|CLH	Clathrin heavy chain 1 OS=Rattus norvegicus GN=Cltc PE=1 SV=3	FQSVPAQPGQTSPLLQYFGILLDQGQLNKYESLELCRPVLQQGR
>	sp|P11442|CLH	Clathrin heavy chain 1 OS=Rattus norvegicus GN=Cltc PE=1 SV=3	HELIEFR
>	sp|P11442|CLH	Clathrin heavy chain 1 OS=Rattus norvegicus GN=Cltc PE=1 SV=3	SVDPTLALSVYLR
>	sp|P11442|CLH	Clathrin heavy chain 1 OS=Rattus norvegicus GN=Cltc PE=1 SV=3	HDVVFLITK
>	sp|P11442|CLH	Clathrin heavy chain 1 OS=Rattus norvegicus GN=Cltc PE=1 SV=3	RFQSVPAQPGQTSPLLQYFGILLDQGQLNKYESLELCRPVLQQGR
>	sp|P11442|CLH	Clathrin heavy chain 1 OS=Rattus norvegicus GN=Cltc PE=1 SV=3	VNPSRLPVVIGGLLDVDCSEDVIK
>	sp|P11442|CLH	Clathrin heavy chain 1 OS=Rattus norvegicus GN=Cltc PE=1 SV=3	KFNALFAQGNYSEAAK
>	sp|P11442|CLH	Clathrin heavy chain 1 OS=Rattus norvegicus GN=Cltc PE=1 SV=3	ISGETIFVTAPHEATAGIIGVNR
>	sp|P11442|CLH	Clathrin heavy chain 1 OS=Rattus norvegicus GN=Cltc PE=1 SV=3	IAAYLFK
>	sp|P06687|AT1	Sodium/potassium-transporting ATPase subunit alpha-3 OS=Rattus norvegicus GN=Atp1a3 PE=1 SV=2	AAVPDAVGK
>	sp|P06687|AT1	Sodium/potassium-transporting ATPase subunit alpha-3 OS=Rattus norvegicus GN=Atp1a3 PE=1 SV=2	ACVIHGTDLKDFTSEQIDEILQNHTEIVFAR
>	sp|P06687|AT1	Sodium/potassium-transporting ATPase subunit alpha-3 OS=Rattus norvegicus GN=Atp1a3 PE=1 SV=2	ADIGVAMGIAGSDVSK
>	sp|P06687|AT1	Sodium/potassium-transporting ATPase subunit alpha-3 OS=Rattus norvegicus GN=Atp1a3 PE=1 SV=2	AVFKGGQDNIPVLK

>	sp|P06687|AT1 Sodium/potassium-transporting ATPase subunit alpha-3 OS=Rattus norvegicus GN=Atp1a3 PE=1 SV=2	CATILLQGK
>	sp|P06687|AT1 Sodium/potassium-transporting ATPase subunit alpha-3 OS=Rattus norvegicus GN=Atp1a3 PE=1 SV=2	DFTSEQIDEILQNHTEIVFAR
>	sp|P06687|AT1 Sodium/potassium-transporting ATPase subunit alpha-3 OS=Rattus norvegicus GN=Atp1a3 PE=1 SV=2	DVAGDASESALLK
>	sp|P06687|AT1 Sodium/potassium-transporting ATPase subunit alpha-3 OS=Rattus norvegicus GN=Atp1a3 PE=1 SV=2	EQPLDEEMKEAFQNAYLELGGLGER
>	sp|P06687|AT1 Sodium/potassium-transporting ATPase subunit alpha-3 OS=Rattus norvegicus GN=Atp1a3 PE=1 SV=2	GGQDNIPVLKR
>	sp|P06687|AT1 Sodium/potassium-transporting ATPase subunit alpha-3 OS=Rattus norvegicus GN=Atp1a3 PE=1 SV=2	GVGIISEGNETVEDIAAR
>	sp|P06687|AT1 Sodium/potassium-transporting ATPase subunit alpha-3 OS=Rattus norvegicus GN=Atp1a3 PE=1 SV=2	KADIGVAMGIAGSDVSK
>	sp|P06687|AT1 Sodium/potassium-transporting ATPase subunit alpha-3 OS=Rattus norvegicus GN=Atp1a3 PE=1 SV=2	KYNTDCVQGLTHSK
>	sp|P06687|AT1 Sodium/potassium-transporting ATPase subunit alpha-3 OS=Rattus norvegicus GN=Atp1a3 PE=1 SV=2	LCFVGLMSMIDPPR
>	sp|P06687|AT1 Sodium/potassium-transporting ATPase subunit alpha-3 OS=Rattus norvegicus GN=Atp1a3 PE=1 SV=2	LIIVEGCQR
>	sp|P06687|AT1 Sodium/potassium-transporting ATPase subunit alpha-3 OS=Rattus norvegicus GN=Atp1a3 PE=1 SV=2	MQVNAEEVVVGDLVEIK
>	sp|P06687|AT1 Sodium/potassium-transporting ATPase subunit alpha-3 OS=Rattus norvegicus GN=Atp1a3 PE=1 SV=2	MTVAHMWFDNQIHEADTTEDQSGTSFDK
>	sp|P06687|AT1 Sodium/potassium-transporting ATPase subunit alpha-3 OS=Rattus norvegicus GN=Atp1a3 PE=1 SV=2	NITFFSTNCVEGTAR
>	sp|P06687|AT1 Sodium/potassium-transporting ATPase subunit alpha-3 OS=Rattus norvegicus GN=Atp1a3 PE=1 SV=2	NLEAVETLGSTSTICSDK
>	sp|P06687|AT1 Sodium/potassium-transporting ATPase subunit alpha-3 OS=Rattus norvegicus GN=Atp1a3 PE=1 SV=2	NLEAVETLGSTSTICSDKTGTLTQNR
>	sp|P06687|AT1 Sodium/potassium-transporting ATPase subunit alpha-3 OS=Rattus norvegicus GN=Atp1a3 PE=1 SV=2	NMVPQQALVIR
>	sp|P06687|AT1 Sodium/potassium-transporting ATPase subunit alpha-3 OS=Rattus norvegicus GN=Atp1a3 PE=1 SV=2	QAADMILLDDNFASIVTGVEEGR
>	sp|P06687|AT1 Sodium/potassium-transporting ATPase subunit alpha-3 OS=Rattus norvegicus GN=Atp1a3 PE=1 SV=2	QGAIVAVTGDGVNDSPALK
>	sp|P06687|AT1 Sodium/potassium-transporting ATPase subunit alpha-3 OS=Rattus norvegicus GN=Atp1a3 PE=1 SV=2	RNPGGWVEKETYY
>	sp|P06687|AT1 Sodium/potassium-transporting ATPase subunit alpha-3 OS=Rattus norvegicus GN=Atp1a3 PE=1 SV=2	RNSVFQQGMK
>	sp|P06687|AT1 Sodium/potassium-transporting ATPase subunit alpha-3 OS=Rattus norvegicus GN=Atp1a3 PE=1 SV=2	SPDCTHDNPLETR
>	sp|P06687|AT1 Sodium/potassium-transporting ATPase subunit alpha-3 OS=Rattus norvegicus GN=Atp1a3 PE=1 SV=2	SSHTWVALSHIAGLCNR
>	sp|P06687|AT1 Sodium/potassium-transporting ATPase subunit alpha-3 OS=Rattus norvegicus GN=Atp1a3 PE=1 SV=2	TDKLVNER
>	sp|P06687|AT1 Sodium/potassium-transporting ATPase subunit alpha-3 OS=Rattus norvegicus GN=Atp1a3 PE=1 SV=2	VAEIPFNSTNK
>	sp|P06687|AT1 Sodium/potassium-transporting ATPase subunit alpha-3 OS=Rattus norvegicus GN=Atp1a3 PE=1 SV=2	VDNSSLTGESEPQTR
>	sp|P06687|AT1 Sodium/potassium-transporting ATPase subunit alpha-3 OS=Rattus norvegicus GN=Atp1a3 PE=1 SV=2	VLGFCHYYLPEEQFPK
>	sp|P06687|AT1 Sodium/potassium-transporting ATPase subunit alpha-3 OS=Rattus norvegicus GN=Atp1a3 PE=1 SV=2	VIMVTGDHPITAK
>	sp|P06687|AT1 Sodium/potassium-transporting ATPase subunit alpha-3 OS=Rattus norvegicus GN=Atp1a3 PE=1 SV=2	LNIPVSQVNPR
>	sp|P06687|AT1 Sodium/potassium-transporting ATPase subunit alpha-3 OS=Rattus norvegicus GN=Atp1a3 PE=1 SV=2	IATLASGLEVGK
>	sp|P06687|AT1 Sodium/potassium-transporting ATPase subunit alpha-3 OS=Rattus norvegicus GN=Atp1a3 PE=1 SV=2	LIFDNLKK
>	sp|P06687|AT1 Sodium/potassium-transporting ATPase subunit alpha-3 OS=Rattus norvegicus GN=Atp1a3 PE=1 SV=2	AAVPDAVGK
>	sp|P06687|AT1 Sodium/potassium-transporting ATPase subunit alpha-3 OS=Rattus norvegicus GN=Atp1a3 PE=1 SV=2	AAVPDAVGK
>	sp|P06687|AT1 Sodium/potassium-transporting ATPase subunit alpha-3 OS=Rattus norvegicus GN=Atp1a3 PE=1 SV=2	ACVIHGTDLKDFTSEQIDEILQNHTEIVFAR
>	sp|P06687|AT1 Sodium/potassium-transporting ATPase subunit alpha-3 OS=Rattus norvegicus GN=Atp1a3 PE=1 SV=2	ACVIHGTDLKDFTSEQIDEILQNHTEIVFAR
>	sp|P06687|AT1 Sodium/potassium-transporting ATPase subunit alpha-3 OS=Rattus norvegicus GN=Atp1a3 PE=1 SV=2	ACVIHGTDLKDFTSEQIDEILQNHTEIVFAR
>	sp|P06687|AT1 Sodium/potassium-transporting ATPase subunit alpha-3 OS=Rattus norvegicus GN=Atp1a3 PE=1 SV=2	ACVIHGTDLKDFTSEQIDEILQNHTEIVFAR
>	sp|P06687|AT1 Sodium/potassium-transporting ATPase subunit alpha-3 OS=Rattus norvegicus GN=Atp1a3 PE=1 SV=2	ACVIHGTDLKDFTSEQIDEILQNHTEIVFAR
>	sp|P06687|AT1 Sodium/potassium-transporting ATPase subunit alpha-3 OS=Rattus norvegicus GN=Atp1a3 PE=1 SV=2	ACVIHGTDLKDFTSEQIDEILQNHTEIVFAR
>	sp|P06687|AT1 Sodium/potassium-transporting ATPase subunit alpha-3 OS=Rattus norvegicus GN=Atp1a3 PE=1 SV=2	ACVIHGTDLKDFTSEQIDEILQNHTEIVFAR
>	sp|P06687|AT1 Sodium/potassium-transporting ATPase subunit alpha-3 OS=Rattus norvegicus GN=Atp1a3 PE=1 SV=2	ACVIHGTDLKDFTSEQIDEILQNHTEIVFAR
>	sp|P06687|AT1 Sodium/potassium-transporting ATPase subunit alpha-3 OS=Rattus norvegicus GN=Atp1a3 PE=1 SV=2	ACVIHGTDLKDFTSEQIDEILQNHTEIVFAR
>	sp|P06687|AT1 Sodium/potassium-transporting ATPase subunit alpha-3 OS=Rattus norvegicus GN=Atp1a3 PE=1 SV=2	ACVIHGTDLKDFTSEQIDEILQNHTEIVFAR
>	sp|P06687|AT1 Sodium/potassium-transporting ATPase subunit alpha-3 OS=Rattus norvegicus GN=Atp1a3 PE=1 SV=2	ACVIHGTDLKDFTSEQIDEILQNHTEIVFAR
>	sp|P06687|AT1 Sodium/potassium-transporting ATPase subunit alpha-3 OS=Rattus norvegicus GN=Atp1a3 PE=1 SV=2	ACVIHGTDLKDFTSEQIDEILQNHTEIVFAR
>	sp|P06687|AT1 Sodium/potassium-transporting ATPase subunit alpha-3 OS=Rattus norvegicus GN=Atp1a3 PE=1 SV=2	ACVIHGTDLKDFTSEQIDEILQNHTEIVFAR
>	sp|P06687|AT1 Sodium/potassium-transporting ATPase subunit alpha-3 OS=Rattus norvegicus GN=Atp1a3 PE=1 SV=2	ADIGVAMGIAGSDVSK
>	sp|P06687|AT1 Sodium/potassium-transporting ATPase subunit alpha-3 OS=Rattus norvegicus GN=Atp1a3 PE=1 SV=2	DVAGDASESALLK
>	sp|P06687|AT1 Sodium/potassium-transporting ATPase subunit alpha-3 OS=Rattus norvegicus GN=Atp1a3 PE=1 SV=2	EQPLDEEMKEAFQNAYLELGGLGER
>	sp|P06687|AT1 Sodium/potassium-transporting ATPase subunit alpha-3 OS=Rattus norvegicus GN=Atp1a3 PE=1 SV=2	EQPLDEEMKEAFQNAYLELGGLGER
>	sp|P06687|AT1 Sodium/potassium-transporting ATPase subunit alpha-3 OS=Rattus norvegicus GN=Atp1a3 PE=1 SV=2	EQPLDEEMKEAFQNAYLELGGLGER
>	sp|P06687|AT1 Sodium/potassium-transporting ATPase subunit alpha-3 OS=Rattus norvegicus GN=Atp1a3 PE=1 SV=2	EQPLDEEMKEAFQNAYLELGGLGER
>	sp|P06687|AT1 Sodium/potassium-transporting ATPase subunit alpha-3 OS=Rattus norvegicus GN=Atp1a3 PE=1 SV=2	EQPLDEEMKEAFQNAYLELGGLGER
>	sp|P06687|AT1 Sodium/potassium-transporting ATPase subunit alpha-3 OS=Rattus norvegicus GN=Atp1a3 PE=1 SV=2	EQPLDEEMKEAFQNAYLELGGLGER
>	sp|P06687|AT1 Sodium/potassium-transporting ATPase subunit alpha-3 OS=Rattus norvegicus GN=Atp1a3 PE=1 SV=2	EQPLDEEMKEAFQNAYLELGGLGER
>	sp|P06687|AT1 Sodium/potassium-transporting ATPase subunit alpha-3 OS=Rattus norvegicus GN=Atp1a3 PE=1 SV=2	EQPLDEEMKEAFQNAYLELGGLGER
>	sp|P06687|AT1 Sodium/potassium-transporting ATPase subunit alpha-3 OS=Rattus norvegicus GN=Atp1a3 PE=1 SV=2	GVGIISEGNETVEDIAAR
>	sp|P06687|AT1 Sodium/potassium-transporting ATPase subunit alpha-3 OS=Rattus norvegicus GN=Atp1a3 PE=1 SV=2	GVGIISEGNETVEDIAAR
>	sp|P06687|AT1 Sodium/potassium-transporting ATPase subunit alpha-3 OS=Rattus norvegicus GN=Atp1a3 PE=1 SV=2	GVGIISEGNETVEDIAAR
>	sp|P06687|AT1 Sodium/potassium-transporting ATPase subunit alpha-3 OS=Rattus norvegicus GN=Atp1a3 PE=1 SV=2	GVGIISEGNETVEDIAAR
>	sp|P06687|AT1 Sodium/potassium-transporting ATPase subunit alpha-3 OS=Rattus norvegicus GN=Atp1a3 PE=1 SV=2	GVGIISEGNETVEDIAAR
>	sp|P06687|AT1 Sodium/potassium-transporting ATPase subunit alpha-3 OS=Rattus norvegicus GN=Atp1a3 PE=1 SV=2	KADIGVAMGIAGSDVSK
>	sp|P06687|AT1 Sodium/potassium-transporting ATPase subunit alpha-3 OS=Rattus norvegicus GN=Atp1a3 PE=1 SV=2	KADIGVAMGIAGSDVSK
>	sp|P06687|AT1 Sodium/potassium-transporting ATPase subunit alpha-3 OS=Rattus norvegicus GN=Atp1a3 PE=1 SV=2	KADIGVAMGIAGSDVSK
>	sp|P06687|AT1 Sodium/potassium-transporting ATPase subunit alpha-3 OS=Rattus norvegicus GN=Atp1a3 PE=1 SV=2	KADIGVAMGIAGSDVSK
>	sp|P06687|AT1 Sodium/potassium-transporting ATPase subunit alpha-3 OS=Rattus norvegicus GN=Atp1a3 PE=1 SV=2	KADIGVAMGIAGSDVSK
>	sp|P06687|AT1 Sodium/potassium-transporting ATPase subunit alpha-3 OS=Rattus norvegicus GN=Atp1a3 PE=1 SV=2	KYNTDCVQGLTHSK
>	sp|P06687|AT1 Sodium/potassium-transporting ATPase subunit alpha-3 OS=Rattus norvegicus GN=Atp1a3 PE=1 SV=2	LCFVGLMSMIDPPR
>	sp|P06687|AT1 Sodium/potassium-transporting ATPase subunit alpha-3 OS=Rattus norvegicus GN=Atp1a3 PE=1 SV=2	MQVNAEEVVVGDLVEIK
>	sp|P06687|AT1 Sodium/potassium-transporting ATPase subunit alpha-3 OS=Rattus norvegicus GN=Atp1a3 PE=1 SV=2	MQVNAEEVVVGDLVEIK
>	sp|P06687|AT1 Sodium/potassium-transporting ATPase subunit alpha-3 OS=Rattus norvegicus GN=Atp1a3 PE=1 SV=2	MQVNAEEVVVGDLVEIK
>	sp|P06687|AT1 Sodium/potassium-transporting ATPase subunit alpha-3 OS=Rattus norvegicus GN=Atp1a3 PE=1 SV=2	MTVAHMWFDNQIHEADTTEDQSGTSFDK
>	sp|P06687|AT1 Sodium/potassium-transporting ATPase subunit alpha-3 OS=Rattus norvegicus GN=Atp1a3 PE=1 SV=2	MTVAHMWFDNQIHEADTTEDQSGTSFDK
>	sp|P06687|AT1 Sodium/potassium-transporting ATPase subunit alpha-3 OS=Rattus norvegicus GN=Atp1a3 PE=1 SV=2	MTVAHMWFDNQIHEADTTEDQSGTSFDK
>	sp|P06687|AT1 Sodium/potassium-transporting ATPase subunit alpha-3 OS=Rattus norvegicus GN=Atp1a3 PE=1 SV=2	MTVAHMWFDNQIHEADTTEDQSGTSFDK

<table>
<tr><td>></td><td>sp|P06687|AT1 Sodium/potassium-transporting ATPase subunit alpha-3 OS=Rattus norvegicus GN=Atp1a3 PE=1 SV=2</td><td>MTVAHMWFDNQIHEADTTEDQSGTSFDK</td></tr>
<tr><td>></td><td>sp|P06687|AT1 Sodium/potassium-transporting ATPase subunit alpha-3 OS=Rattus norvegicus GN=Atp1a3 PE=1 SV=2</td><td>MTVAHMWFDNQIHEADTTEDQSGTSFDK</td></tr>
<tr><td>></td><td>sp|P06687|AT1 Sodium/potassium-transporting ATPase subunit alpha-3 OS=Rattus norvegicus GN=Atp1a3 PE=1 SV=2</td><td>MTVAHMWFDNQIHEADTTEDQSGTSFDK</td></tr>
<tr><td>></td><td>sp|P06687|AT1 Sodium/potassium-transporting ATPase subunit alpha-3 OS=Rattus norvegicus GN=Atp1a3 PE=1 SV=2</td><td>NITFFSTNCVEGTAR</td></tr>
<tr><td>></td><td>sp|P06687|AT1 Sodium/potassium-transporting ATPase subunit alpha-3 OS=Rattus norvegicus GN=Atp1a3 PE=1 SV=2</td><td>NLEAVETLGSTSTICSDK</td></tr>
<tr><td>></td><td>sp|P06687|AT1 Sodium/potassium-transporting ATPase subunit alpha-3 OS=Rattus norvegicus GN=Atp1a3 PE=1 SV=2</td><td>NLEAVETLGSTSTICSDK</td></tr>
<tr><td>></td><td>sp|P06687|AT1 Sodium/potassium-transporting ATPase subunit alpha-3 OS=Rattus norvegicus GN=Atp1a3 PE=1 SV=2</td><td>NLEAVETLGSTSTICSDK</td></tr>
<tr><td>></td><td>sp|P06687|AT1 Sodium/potassium-transporting ATPase subunit alpha-3 OS=Rattus norvegicus GN=Atp1a3 PE=1 SV=2</td><td>NLEAVETLGSTSTICSDK</td></tr>
<tr><td>></td><td>sp|P06687|AT1 Sodium/potassium-transporting ATPase subunit alpha-3 OS=Rattus norvegicus GN=Atp1a3 PE=1 SV=2</td><td>NLEAVETLGSTSTICSDKTGTLTQNR</td></tr>
<tr><td>></td><td>sp|P06687|AT1 Sodium/potassium-transporting ATPase subunit alpha-3 OS=Rattus norvegicus GN=Atp1a3 PE=1 SV=2</td><td>NLEAVETLGSTSTICSDKTGTLTQNR</td></tr>
<tr><td>></td><td>sp|P06687|AT1 Sodium/potassium-transporting ATPase subunit alpha-3 OS=Rattus norvegicus GN=Atp1a3 PE=1 SV=2</td><td>NLEAVETLGSTSTICSDKTGTLTQNR</td></tr>
<tr><td>></td><td>sp|P06687|AT1 Sodium/potassium-transporting ATPase subunit alpha-3 OS=Rattus norvegicus GN=Atp1a3 PE=1 SV=2</td><td>NMVPQQALVIR</td></tr>
<tr><td>></td><td>sp|P06687|AT1 Sodium/potassium-transporting ATPase subunit alpha-3 OS=Rattus norvegicus GN=Atp1a3 PE=1 SV=2</td><td>NMVPQQALVIR</td></tr>
<tr><td>></td><td>sp|P06687|AT1 Sodium/potassium-transporting ATPase subunit alpha-3 OS=Rattus norvegicus GN=Atp1a3 PE=1 SV=2</td><td>QAADMILLDDNFASIVTGVEEGR</td></tr>
<tr><td>></td><td>sp|P06687|AT1 Sodium/potassium-transporting ATPase subunit alpha-3 OS=Rattus norvegicus GN=Atp1a3 PE=1 SV=2</td><td>QAADMILLDDNFASIVTGVEEGR</td></tr>
<tr><td>></td><td>sp|P06687|AT1 Sodium/potassium-transporting ATPase subunit alpha-3 OS=Rattus norvegicus GN=Atp1a3 PE=1 SV=2</td><td>QGAIVAVTGDGVNDSPALK</td></tr>
<tr><td>></td><td>sp|P06687|AT1 Sodium/potassium-transporting ATPase subunit alpha-3 OS=Rattus norvegicus GN=Atp1a3 PE=1 SV=2</td><td>QGAIVAVTGDGVNDSPALK</td></tr>
<tr><td>></td><td>sp|P06687|AT1 Sodium/potassium-transporting ATPase subunit alpha-3 OS=Rattus norvegicus GN=Atp1a3 PE=1 SV=2</td><td>QGAIVAVTGDGVNDSPALK</td></tr>
<tr><td>></td><td>sp|P06687|AT1 Sodium/potassium-transporting ATPase subunit alpha-3 OS=Rattus norvegicus GN=Atp1a3 PE=1 SV=2</td><td>QGAIVAVTGDGVNDSPALK</td></tr>
<tr><td>></td><td>sp|P06687|AT1 Sodium/potassium-transporting ATPase subunit alpha-3 OS=Rattus norvegicus GN=Atp1a3 PE=1 SV=2</td><td>SPDCTHDNPLETR</td></tr>
<tr><td>></td><td>sp|P06687|AT1 Sodium/potassium-transporting ATPase subunit alpha-3 OS=Rattus norvegicus GN=Atp1a3 PE=1 SV=2</td><td>SPDCTHDNPLETR</td></tr>
<tr><td>></td><td>sp|P06687|AT1 Sodium/potassium-transporting ATPase subunit alpha-3 OS=Rattus norvegicus GN=Atp1a3 PE=1 SV=2</td><td>SPDCTHDNPLETR</td></tr>
<tr><td>></td><td>sp|P06687|AT1 Sodium/potassium-transporting ATPase subunit alpha-3 OS=Rattus norvegicus GN=Atp1a3 PE=1 SV=2</td><td>SPDCTHDNPLETR</td></tr>
<tr><td>></td><td>sp|P06687|AT1 Sodium/potassium-transporting ATPase subunit alpha-3 OS=Rattus norvegicus GN=Atp1a3 PE=1 SV=2</td><td>SPDCTHDNPLETR</td></tr>
<tr><td>></td><td>sp|P06687|AT1 Sodium/potassium-transporting ATPase subunit alpha-3 OS=Rattus norvegicus GN=Atp1a3 PE=1 SV=2</td><td>SSHTWVALSHIAGLCNR</td></tr>
<tr><td>></td><td>sp|P06687|AT1 Sodium/potassium-transporting ATPase subunit alpha-3 OS=Rattus norvegicus GN=Atp1a3 PE=1 SV=2</td><td>SSHTWVALSHIAGLCNR</td></tr>
<tr><td>></td><td>sp|P06687|AT1 Sodium/potassium-transporting ATPase subunit alpha-3 OS=Rattus norvegicus GN=Atp1a3 PE=1 SV=2</td><td>VAEIPFNSTNK</td></tr>
<tr><td>></td><td>sp|P06687|AT1 Sodium/potassium-transporting ATPase subunit alpha-3 OS=Rattus norvegicus GN=Atp1a3 PE=1 SV=2</td><td>VAEIPFNSTNK</td></tr>
<tr><td>></td><td>sp|P06687|AT1 Sodium/potassium-transporting ATPase subunit alpha-3 OS=Rattus norvegicus GN=Atp1a3 PE=1 SV=2</td><td>VDNSSLTGESEPQTR</td></tr>
<tr><td>></td><td>sp|P06687|AT1 Sodium/potassium-transporting ATPase subunit alpha-3 OS=Rattus norvegicus GN=Atp1a3 PE=1 SV=2</td><td>VDNSSLTGESEPQTR</td></tr>
<tr><td>></td><td>sp|P06687|AT1 Sodium/potassium-transporting ATPase subunit alpha-3 OS=Rattus norvegicus GN=Atp1a3 PE=1 SV=2</td><td>VDNSSLTGESEPQTR</td></tr>
<tr><td>></td><td>sp|P06687|AT1 Sodium/potassium-transporting ATPase subunit alpha-3 OS=Rattus norvegicus GN=Atp1a3 PE=1 SV=2</td><td>VIMVTGDHPITAK</td></tr>
<tr><td>></td><td>sp|P06687|AT1 Sodium/potassium-transporting ATPase subunit alpha-3 OS=Rattus norvegicus GN=Atp1a3 PE=1 SV=2</td><td>VIMVTGDHPITAK</td></tr>
<tr><td>></td><td>sp|P06687|AT1 Sodium/potassium-transporting ATPase subunit alpha-3 OS=Rattus norvegicus GN=Atp1a3 PE=1 SV=2</td><td>VIMVTGDHPITAK</td></tr>
<tr><td>></td><td>sp|P06687|AT1 Sodium/potassium-transporting ATPase subunit alpha-3 OS=Rattus norvegicus GN=Atp1a3 PE=1 SV=2</td><td>VLGFCHYYLPEEQFPK</td></tr>
<tr><td>></td><td>sp|P06687|AT1 Sodium/potassium-transporting ATPase subunit alpha-3 OS=Rattus norvegicus GN=Atp1a3 PE=1 SV=2</td><td>DFTSEQIDEILQNHTEIVFAR</td></tr>
<tr><td>></td><td>sp|P06687|AT1 Sodium/potassium-transporting ATPase subunit alpha-3 OS=Rattus norvegicus GN=Atp1a3 PE=1 SV=2</td><td>NITFFSTNCVEGTAR</td></tr>
<tr><td>></td><td>sp|P06687|AT1 Sodium/potassium-transporting ATPase subunit alpha-3 OS=Rattus norvegicus GN=Atp1a3 PE=1 SV=2</td><td>EQPLDEEMKEAFQNAYLELGGLGER</td></tr>
<tr><td>></td><td>sp|P06687|AT1 Sodium/potassium-transporting ATPase subunit alpha-3 OS=Rattus norvegicus GN=Atp1a3 PE=1 SV=2</td><td>MQVNAEEVVVGDLVEIK</td></tr>
<tr><td>></td><td>sp|P06687|AT1 Sodium/potassium-transporting ATPase subunit alpha-3 OS=Rattus norvegicus GN=Atp1a3 PE=1 SV=2</td><td>DFTSEQIDEILQNHTEIVFAR</td></tr>
<tr><td>></td><td>sp|P06687|AT1 Sodium/potassium-transporting ATPase subunit alpha-3 OS=Rattus norvegicus GN=Atp1a3 PE=1 SV=2</td><td>NLEAVETLGSTSTICSDK</td></tr>
<tr><td>></td><td>sp|P06687|AT1 Sodium/potassium-transporting ATPase subunit alpha-3 OS=Rattus norvegicus GN=Atp1a3 PE=1 SV=2</td><td>IATLASGLEVGK</td></tr>
<tr><td>></td><td>sp|P06687|AT1 Sodium/potassium-transporting ATPase subunit alpha-3 OS=Rattus norvegicus GN=Atp1a3 PE=1 SV=2</td><td>NLEAVETLGSTSTICSDK</td></tr>
<tr><td>></td><td>sp|P06687|AT1 Sodium/potassium-transporting ATPase subunit alpha-3 OS=Rattus norvegicus GN=Atp1a3 PE=1 SV=2</td><td>DVAGDASESALLK</td></tr>
<tr><td>></td><td>sp|P06687|AT1 Sodium/potassium-transporting ATPase subunit alpha-3 OS=Rattus norvegicus GN=Atp1a3 PE=1 SV=2</td><td>SPDCTHDNPLETR</td></tr>
<tr><td>></td><td>sp|P06687|AT1 Sodium/potassium-transporting ATPase subunit alpha-3 OS=Rattus norvegicus GN=Atp1a3 PE=1 SV=2</td><td>AQEILAR</td></tr>
<tr><td>></td><td>sp|P06687|AT1 Sodium/potassium-transporting ATPase subunit alpha-3 OS=Rattus norvegicus GN=Atp1a3 PE=1 SV=2</td><td>KADIGVAMGIAGSDVSK</td></tr>
<tr><td>></td><td>sp|P06687|AT1 Sodium/potassium-transporting ATPase subunit alpha-3 OS=Rattus norvegicus GN=Atp1a3 PE=1 SV=2</td><td>LNIPVSQVNPR</td></tr>
<tr><td>></td><td>sp|P06687|AT1 Sodium/potassium-transporting ATPase subunit alpha-3 OS=Rattus norvegicus GN=Atp1a3 PE=1 SV=2</td><td>NMVPQQALVIR</td></tr>
<tr><td>></td><td>sp|P06687|AT1 Sodium/potassium-transporting ATPase subunit alpha-3 OS=Rattus norvegicus GN=Atp1a3 PE=1 SV=2</td><td>DGPNALTPPPTTPEWVK</td></tr>
<tr><td>></td><td>sp|P06687|AT1 Sodium/potassium-transporting ATPase subunit alpha-3 OS=Rattus norvegicus GN=Atp1a3 PE=1 SV=2</td><td>IATLASGLEVGK</td></tr>
<tr><td>></td><td>sp|P06687|AT1 Sodium/potassium-transporting ATPase subunit alpha-3 OS=Rattus norvegicus GN=Atp1a3 PE=1 SV=2</td><td>ACVIHGTDLKDFTSEQIDEILQNHTEIVFAR</td></tr>
<tr><td>></td><td>sp|P06687|AT1 Sodium/potassium-transporting ATPase subunit alpha-3 OS=Rattus norvegicus GN=Atp1a3 PE=1 SV=2</td><td>NMVPQQALVIR</td></tr>
<tr><td>></td><td>sp|P06687|AT1 Sodium/potassium-transporting ATPase subunit alpha-3 OS=Rattus norvegicus GN=Atp1a3 PE=1 SV=2</td><td>LNIPVSQVNPR</td></tr>
<tr><td>></td><td>sp|P06687|AT1 Sodium/potassium-transporting ATPase subunit alpha-3 OS=Rattus norvegicus GN=Atp1a3 PE=1 SV=2</td><td>KADIGVAMGIAGSDVSK</td></tr>
<tr><td>></td><td>sp|P06687|AT1 Sodium/potassium-transporting ATPase subunit alpha-3 OS=Rattus norvegicus GN=Atp1a3 PE=1 SV=2</td><td>MQVNAEEVVVGDLVEIK</td></tr>
<tr><td>></td><td>sp|P06687|AT1 Sodium/potassium-transporting ATPase subunit alpha-3 OS=Rattus norvegicus GN=Atp1a3 PE=1 SV=2</td><td>NMVPQQALVIR</td></tr>
<tr><td>></td><td>sp|P06687|AT1 Sodium/potassium-transporting ATPase subunit alpha-3 OS=Rattus norvegicus GN=Atp1a3 PE=1 SV=2</td><td>RDVAGDASESALLK</td></tr>
<tr><td>></td><td>sp|P06687|AT1 Sodium/potassium-transporting ATPase subunit alpha-3 OS=Rattus norvegicus GN=Atp1a3 PE=1 SV=2</td><td>NSVFQQGMK</td></tr>
<tr><td>></td><td>sp|P06687|AT1 Sodium/potassium-transporting ATPase subunit alpha-3 OS=Rattus norvegicus GN=Atp1a3 PE=1 SV=2</td><td>LNIPVSQVNPR</td></tr>
<tr><td>></td><td>sp|P06687|AT1 Sodium/potassium-transporting ATPase subunit alpha-3 OS=Rattus norvegicus GN=Atp1a3 PE=1 SV=2</td><td>LIFDNLK</td></tr>
<tr><td>></td><td>sp|P06687|AT1 Sodium/potassium-transporting ATPase subunit alpha-3 OS=Rattus norvegicus GN=Atp1a3 PE=1 SV=2</td><td>VIMVTGDHPITAK</td></tr>
<tr><td>></td><td>sp|P06687|AT1 Sodium/potassium-transporting ATPase subunit alpha-3 OS=Rattus norvegicus GN=Atp1a3 PE=1 SV=2</td><td>NLEAVETLGSTSTICSDK</td></tr>
<tr><td>></td><td>sp|P06687|AT1 Sodium/potassium-transporting ATPase subunit alpha-3 OS=Rattus norvegicus GN=Atp1a3 PE=1 SV=2</td><td>GGQDNIPVLK</td></tr>
<tr><td>></td><td>sp|P06687|AT1 Sodium/potassium-transporting ATPase subunit alpha-3 OS=Rattus norvegicus GN=Atp1a3 PE=1 SV=2</td><td>YQLSIHETEDPNDNR</td></tr>
<tr><td>></td><td>sp|P06687|AT1 Sodium/potassium-transporting ATPase subunit alpha-3 OS=Rattus norvegicus GN=Atp1a3 PE=1 SV=2</td><td>MQVNAEEVVVGDLVEIK</td></tr>
<tr><td>></td><td>sp|P06687|AT1 Sodium/potassium-transporting ATPase subunit alpha-3 OS=Rattus norvegicus GN=Atp1a3 PE=1 SV=2</td><td>MVTGDHPITAK</td></tr>
<tr><td>></td><td>sp|P06687|AT1 Sodium/potassium-transporting ATPase subunit alpha-3 OS=Rattus norvegicus GN=Atp1a3 PE=1 SV=2</td><td>VIMVTGDHPITAK</td></tr>
<tr><td>></td><td>sp|P06687|AT1 Sodium/potassium-transporting ATPase subunit alpha-3 OS=Rattus norvegicus GN=Atp1a3 PE=1 SV=2</td><td>LIIVEGCQR</td></tr>
<tr><td>></td><td>sp|P06687|AT1 Sodium/potassium-transporting ATPase subunit alpha-3 OS=Rattus norvegicus GN=Atp1a3 PE=1 SV=2</td><td>KNCLVK</td></tr>
<tr><td>></td><td>sp|P06687|AT1 Sodium/potassium-transporting ATPase subunit alpha-3 OS=Rattus norvegicus GN=Atp1a3 PE=1 SV=2</td><td>MTVAHMWFDNQIHEADTTEDQSGTSFDK</td></tr>
<tr><td>></td><td>sp|P06687|AT1 Sodium/potassium-transporting ATPase subunit alpha-3 OS=Rattus norvegicus GN=Atp1a3 PE=1 SV=2</td><td>ACVIHGTDLKDFTSEQIDEILQNHTEIVFAR</td></tr>
<tr><td>></td><td>sp|P06687|AT1 Sodium/potassium-transporting ATPase subunit alpha-3 OS=Rattus norvegicus GN=Atp1a3 PE=1 SV=2</td><td>DVAGDASESALLK</td></tr>
<tr><td>></td><td>sp|P06687|AT1 Sodium/potassium-transporting ATPase subunit alpha-3 OS=Rattus norvegicus GN=Atp1a3 PE=1 SV=2</td><td>LNIPVSQVNPR</td></tr>
</table>

>	sp|P06687|AT1 Sodium/potassium-transporting ATPase subunit alpha-3 OS=Rattus norvegicus GN=Atp1a3 PE=1 SV=2	DVAGDASESALLK
>	sp|P06687|AT1 Sodium/potassium-transporting ATPase subunit alpha-3 OS=Rattus norvegicus GN=Atp1a3 PE=1 SV=2	EQPLDEEMKEAFQNAYLELGGLGER
>	sp|P06687|AT1 Sodium/potassium-transporting ATPase subunit alpha-3 OS=Rattus norvegicus GN=Atp1a3 PE=1 SV=2	NLEAVETLGSTSTICSDK
>	sp|P06687|AT1 Sodium/potassium-transporting ATPase subunit alpha-3 OS=Rattus norvegicus GN=Atp1a3 PE=1 SV=2	KADIGVAMGIAGSDVSK
>	sp|P06687|AT1 Sodium/potassium-transporting ATPase subunit alpha-3 OS=Rattus norvegicus GN=Atp1a3 PE=1 SV=2	TVNDLEDSYGQQWTYEQR
>	sp|P06687|AT1 Sodium/potassium-transporting ATPase subunit alpha-3 OS=Rattus norvegicus GN=Atp1a3 PE=1 SV=2	TVNDLEDSYGQQWTYEQR
>	sp|P06687|AT1 Sodium/potassium-transporting ATPase subunit alpha-3 OS=Rattus norvegicus GN=Atp1a3 PE=1 SV=2	AQEILAR
>	sp|P06687|AT1 Sodium/potassium-transporting ATPase subunit alpha-3 OS=Rattus norvegicus GN=Atp1a3 PE=1 SV=2	GVVVATGDR
>	sp|P06687|AT1 Sodium/potassium-transporting ATPase subunit alpha-3 OS=Rattus norvegicus GN=Atp1a3 PE=1 SV=2	KNCLVK
>	sp|P06687|AT1 Sodium/potassium-transporting ATPase subunit alpha-3 OS=Rattus norvegicus GN=Atp1a3 PE=1 SV=2	KADIGVAMGIAGSDVSK
>	sp|P06687|AT1 Sodium/potassium-transporting ATPase subunit alpha-3 OS=Rattus norvegicus GN=Atp1a3 PE=1 SV=2	IATLASGLEVGK
>	sp|P06687|AT1 Sodium/potassium-transporting ATPase subunit alpha-3 OS=Rattus norvegicus GN=Atp1a3 PE=1 SV=2	DVAGDASESALLK
>	sp|Q5XIF6|TBA Tubulin alpha-4A chain OS=Rattus norvegicus GN=Tuba4a PE=2 SV=1	AFVHWYVGEGMEEGEFSEAR
>	sp|Q5XIF6|TBA Tubulin alpha-4A chain OS=Rattus norvegicus GN=Tuba4a PE=2 SV=1	AFVHWYVGEGMEEGEFSEAREDMAALEK
>	sp|Q5XIF6|TBA Tubulin alpha-4A chain OS=Rattus norvegicus GN=Tuba4a PE=2 SV=1	AVCMLSNTTAIAEAWAR
>	sp|Q5XIF6|TBA Tubulin alpha-4A chain OS=Rattus norvegicus GN=Tuba4a PE=2 SV=1	AVFVDLEPTVIDEIR
>	sp|Q5XIF6|TBA Tubulin alpha-4A chain OS=Rattus norvegicus GN=Tuba4a PE=2 SV=1	AYHEQLSVAEITNACFEPANQMVK
>	sp|Q5XIF6|TBA Tubulin alpha-4A chain OS=Rattus norvegicus GN=Tuba4a PE=2 SV=1	DVNAAIAAIK
>	sp|Q5XIF6|TBA Tubulin alpha-4A chain OS=Rattus norvegicus GN=Tuba4a PE=2 SV=1	ECISVHVGQAGVQMGNACWELYCLEHGIQPDGQMPSDK
>	sp|Q5XIF6|TBA Tubulin alpha-4A chain OS=Rattus norvegicus GN=Tuba4a PE=2 SV=1	ECISVHVGQAGVQMGNACWELYCLEHGIQPDGQMPSDKTIGGGDDSFTTFFCETGAGK
>	sp|Q5XIF6|TBA Tubulin alpha-4A chain OS=Rattus norvegicus GN=Tuba4a PE=2 SV=1	EDAANNYAR
>	sp|Q5XIF6|TBA Tubulin alpha-4A chain OS=Rattus norvegicus GN=Tuba4a PE=2 SV=1	EIIDPVLDR
>	sp|Q5XIF6|TBA Tubulin alpha-4A chain OS=Rattus norvegicus GN=Tuba4a PE=2 SV=1	FDGALNVDLTEFQTNLVPYPR
>	sp|Q5XIF6|TBA Tubulin alpha-4A chain OS=Rattus norvegicus GN=Tuba4a PE=2 SV=1	IHFPLATYAPVISAEK
>	sp|Q5XIF6|TBA Tubulin alpha-4A chain OS=Rattus norvegicus GN=Tuba4a PE=2 SV=1	KLSDQCTGLQGFLVFHSFGGGTGSGFTSLLMER
>	sp|Q5XIF6|TBA Tubulin alpha-4A chain OS=Rattus norvegicus GN=Tuba4a PE=2 SV=1	LDHKFDLMYAK
>	sp|Q5XIF6|TBA Tubulin alpha-4A chain OS=Rattus norvegicus GN=Tuba4a PE=2 SV=1	LEFSIYPAPQVSTAVVEPYNSILTTHTTLEHSDCAFMVDNEAIYDICR
>	sp|Q5XIF6|TBA Tubulin alpha-4A chain OS=Rattus norvegicus GN=Tuba4a PE=2 SV=1	NLDIERPTYTNLNR
>	sp|Q5XIF6|TBA Tubulin alpha-4A chain OS=Rattus norvegicus GN=Tuba4a PE=2 SV=1	NLDIERPTYTNLNRLISQIVSSITASLR
>	sp|Q5XIF6|TBA Tubulin alpha-4A chain OS=Rattus norvegicus GN=Tuba4a PE=2 SV=1	NLDIERPTYTNLNRLISQIVSSITASLRFDGALNVDLTEFQTNLVPYPR
>	sp|Q5XIF6|TBA Tubulin alpha-4A chain OS=Rattus norvegicus GN=Tuba4a PE=2 SV=1	QLFHPEQLITGK
>	sp|Q5XIF6|TBA Tubulin alpha-4A chain OS=Rattus norvegicus GN=Tuba4a PE=2 SV=1	QLFHPEQLITGKEDAANNYAR
>	sp|Q5XIF6|TBA Tubulin alpha-4A chain OS=Rattus norvegicus GN=Tuba4a PE=2 SV=1	RAFVHWYVGEGMEEGEFSEAR
>	sp|Q5XIF6|TBA Tubulin alpha-4A chain OS=Rattus norvegicus GN=Tuba4a PE=2 SV=1	SKLEFSIYPAPQVSTAVVEPYNSILTTHTTLEHSDCAFMVDNEAIYDICR
>	sp|Q5XIF6|TBA Tubulin alpha-4A chain OS=Rattus norvegicus GN=Tuba4a PE=2 SV=1	VGINYQPPTVVPGGDLAK
>	sp|Q5XIF6|TBA Tubulin alpha-4A chain OS=Rattus norvegicus GN=Tuba4a PE=2 SV=1	YMACCLLYR
>	sp|Q5XIF6|TBA Tubulin alpha-4A chain OS=Rattus norvegicus GN=Tuba4a PE=2 SV=1	SIQFVDWCPTGFK
>	sp|Q5XIF6|TBA Tubulin alpha-4A chain OS=Rattus norvegicus GN=Tuba4a PE=2 SV=1	AYHEQLSVAEITN
>	sp|Q5XIF6|TBA Tubulin alpha-4A chain OS=Rattus norvegicus GN=Tuba4a PE=2 SV=1	PGGDLAK
>	sp|Q5XIF6|TBA Tubulin alpha-4A chain OS=Rattus norvegicus GN=Tuba4a PE=2 SV=1	PTVIDEIR
>	sp|Q5XIF6|TBA Tubulin alpha-4A chain OS=Rattus norvegicus GN=Tuba4a PE=2 SV=1	IHFPLATYAPVIS
>	sp|Q5XIF6|TBA Tubulin alpha-4A chain OS=Rattus norvegicus GN=Tuba4a PE=2 SV=1	PPTVVPGGDLAK
>	sp|Q5XIF6|TBA Tubulin alpha-4A chain OS=Rattus norvegicus GN=Tuba4a PE=2 SV=1	MRECISVHVGQAGVQMGNACWELYCLEHGIQPDGQMPSDKTIGGGDDSFTTFFCETGAGK
>	sp|Q5XIF6|TBA Tubulin alpha-4A chain OS=Rattus norvegicus GN=Tuba4a PE=2 SV=1	NGPYRQLFHPEQLITGKEDAANNYAR
>	sp|Q5XIF6|TBA Tubulin alpha-4A chain OS=Rattus norvegicus GN=Tuba4a PE=2 SV=1	AFVHWYVGEGMEEGEFSEAR
>	sp|Q5XIF6|TBA Tubulin alpha-4A chain OS=Rattus norvegicus GN=Tuba4a PE=2 SV=1	AFVHWYVGEGMEEGEFSEAR
>	sp|Q5XIF6|TBA Tubulin alpha-4A chain OS=Rattus norvegicus GN=Tuba4a PE=2 SV=1	AFVHWYVGEGMEEGEFSEAR
>	sp|Q5XIF6|TBA Tubulin alpha-4A chain OS=Rattus norvegicus GN=Tuba4a PE=2 SV=1	AFVHWYVGEGMEEGEFSEAR
>	sp|Q5XIF6|TBA Tubulin alpha-4A chain OS=Rattus norvegicus GN=Tuba4a PE=2 SV=1	AFVHWYVGEGMEEGEFSEAR
>	sp|Q5XIF6|TBA Tubulin alpha-4A chain OS=Rattus norvegicus GN=Tuba4a PE=2 SV=1	AFVHWYVGEGMEEGEFSEAR
>	sp|Q5XIF6|TBA Tubulin alpha-4A chain OS=Rattus norvegicus GN=Tuba4a PE=2 SV=1	AFVHWYVGEGMEEGEFSEAR
>	sp|Q5XIF6|TBA Tubulin alpha-4A chain OS=Rattus norvegicus GN=Tuba4a PE=2 SV=1	AFVHWYVGEGMEEGEFSEAR
>	sp|Q5XIF6|TBA Tubulin alpha-4A chain OS=Rattus norvegicus GN=Tuba4a PE=2 SV=1	AFVHWYVGEGMEEGEFSEAR
>	sp|Q5XIF6|TBA Tubulin alpha-4A chain OS=Rattus norvegicus GN=Tuba4a PE=2 SV=1	AFVHWYVGEGMEEGEFSEAR
>	sp|Q5XIF6|TBA Tubulin alpha-4A chain OS=Rattus norvegicus GN=Tuba4a PE=2 SV=1	AFVHWYVGEGMEEGEFSEAR
>	sp|Q5XIF6|TBA Tubulin alpha-4A chain OS=Rattus norvegicus GN=Tuba4a PE=2 SV=1	AFVHWYVGEGMEEGEFSEAR
>	sp|Q5XIF6|TBA Tubulin alpha-4A chain OS=Rattus norvegicus GN=Tuba4a PE=2 SV=1	AFVHWYVGEGMEEGEFSEAR
>	sp|Q5XIF6|TBA Tubulin alpha-4A chain OS=Rattus norvegicus GN=Tuba4a PE=2 SV=1	AFVHWYVGEGMEEGEFSEAREDMAALEK
>	sp|Q5XIF6|TBA Tubulin alpha-4A chain OS=Rattus norvegicus GN=Tuba4a PE=2 SV=1	AFVHWYVGEGMEEGEFSEAREDMAALEK
>	sp|Q5XIF6|TBA Tubulin alpha-4A chain OS=Rattus norvegicus GN=Tuba4a PE=2 SV=1	AFVHWYVGEGMEEGEFSEAREDMAALEK
>	sp|Q5XIF6|TBA Tubulin alpha-4A chain OS=Rattus norvegicus GN=Tuba4a PE=2 SV=1	AVCMLSNTTAIAEAWAR
>	sp|Q5XIF6|TBA Tubulin alpha-4A chain OS=Rattus norvegicus GN=Tuba4a PE=2 SV=1	AVCMLSNTTAIAEAWAR
>	sp|Q5XIF6|TBA Tubulin alpha-4A chain OS=Rattus norvegicus GN=Tuba4a PE=2 SV=1	AVCMLSNTTAIAEAWAR
>	sp|Q5XIF6|TBA Tubulin alpha-4A chain OS=Rattus norvegicus GN=Tuba4a PE=2 SV=1	AVCMLSNTTAIAEAWAR
>	sp|Q5XIF6|TBA Tubulin alpha-4A chain OS=Rattus norvegicus GN=Tuba4a PE=2 SV=1	AVCMLSNTTAIAEAWAR
>	sp|Q5XIF6|TBA Tubulin alpha-4A chain OS=Rattus norvegicus GN=Tuba4a PE=2 SV=1	AVCMLSNTTAIAEAWAR
>	sp|Q5XIF6|TBA Tubulin alpha-4A chain OS=Rattus norvegicus GN=Tuba4a PE=2 SV=1	AVCMLSNTTAIAEAWAR
>	sp|Q5XIF6|TBA Tubulin alpha-4A chain OS=Rattus norvegicus GN=Tuba4a PE=2 SV=1	AVCMLSNTTAIAEAWAR
>	sp|Q5XIF6|TBA Tubulin alpha-4A chain OS=Rattus norvegicus GN=Tuba4a PE=2 SV=1	AVCMLSNTTAIAEAWAR
>	sp|Q5XIF6|TBA Tubulin alpha-4A chain OS=Rattus norvegicus GN=Tuba4a PE=2 SV=1	AVCMLSNTTAIAEAWAR
>	sp|Q5XIF6|TBA Tubulin alpha-4A chain OS=Rattus norvegicus GN=Tuba4a PE=2 SV=1	AVCMLSNTTAIAEAWAR
>	sp|Q5XIF6|TBA Tubulin alpha-4A chain OS=Rattus norvegicus GN=Tuba4a PE=2 SV=1	AVCMLSNTTAIAEAWAR
>	sp|Q5XIF6|TBA Tubulin alpha-4A chain OS=Rattus norvegicus GN=Tuba4a PE=2 SV=1	AVCMLSNTTAIAEAWAR
>	sp|Q5XIF6|TBA Tubulin alpha-4A chain OS=Rattus norvegicus GN=Tuba4a PE=2 SV=1	AVCMLSNTTAIAEAWAR

>	sp|Q5XIF6|TBA Tubulin alpha-4A chain OS=Rattus norvegicus GN=Tuba4a PE=2 SV=1	AVCMLSNTTAIAEAWAR
>	sp|Q5XIF6|TBA Tubulin alpha-4A chain OS=Rattus norvegicus GN=Tuba4a PE=2 SV=1	AVFVDLEPTVIDEIR
>	sp|Q5XIF6|TBA Tubulin alpha-4A chain OS=Rattus norvegicus GN=Tuba4a PE=2 SV=1	AVFVDLEPTVIDEIR
>	sp|Q5XIF6|TBA Tubulin alpha-4A chain OS=Rattus norvegicus GN=Tuba4a PE=2 SV=1	AYHEQLSVAEITNACFEPANQMVK
>	sp|Q5XIF6|TBA Tubulin alpha-4A chain OS=Rattus norvegicus GN=Tuba4a PE=2 SV=1	AYHEQLSVAEITNACFEPANQMVK
>	sp|Q5XIF6|TBA Tubulin alpha-4A chain OS=Rattus norvegicus GN=Tuba4a PE=2 SV=1	AYHEQLSVAEITNACFEPANQMVK
>	sp|Q5XIF6|TBA Tubulin alpha-4A chain OS=Rattus norvegicus GN=Tuba4a PE=2 SV=1	AYHEQLSVAEITNACFEPANQMVK
>	sp|Q5XIF6|TBA Tubulin alpha-4A chain OS=Rattus norvegicus GN=Tuba4a PE=2 SV=1	AYHEQLSVAEITNACFEPANQMVK
>	sp|Q5XIF6|TBA Tubulin alpha-4A chain OS=Rattus norvegicus GN=Tuba4a PE=2 SV=1	AYHEQLSVAEITNACFEPANQMVK
>	sp|Q5XIF6|TBA Tubulin alpha-4A chain OS=Rattus norvegicus GN=Tuba4a PE=2 SV=1	AYHEQLSVAEITNACFEPANQMVK
>	sp|Q5XIF6|TBA Tubulin alpha-4A chain OS=Rattus norvegicus GN=Tuba4a PE=2 SV=1	AYHEQLSVAEITNACFEPANQMVK
>	sp|Q5XIF6|TBA Tubulin alpha-4A chain OS=Rattus norvegicus GN=Tuba4a PE=2 SV=1	AYHEQLSVAEITNACFEPANQMVK
>	sp|Q5XIF6|TBA Tubulin alpha-4A chain OS=Rattus norvegicus GN=Tuba4a PE=2 SV=1	AYHEQLSVAEITNACFEPANQMVK
>	sp|Q5XIF6|TBA Tubulin alpha-4A chain OS=Rattus norvegicus GN=Tuba4a PE=2 SV=1	AYHEQLSVAEITNACFEPANQMVK
>	sp|Q5XIF6|TBA Tubulin alpha-4A chain OS=Rattus norvegicus GN=Tuba4a PE=2 SV=1	AYHEQLSVAEITNACFEPANQMVK
>	sp|Q5XIF6|TBA Tubulin alpha-4A chain OS=Rattus norvegicus GN=Tuba4a PE=2 SV=1	AYHEQLSVAEITNACFEPANQMVK
>	sp|Q5XIF6|TBA Tubulin alpha-4A chain OS=Rattus norvegicus GN=Tuba4a PE=2 SV=1	AYHEQLSVAEITNACFEPANQMVK
>	sp|Q5XIF6|TBA Tubulin alpha-4A chain OS=Rattus norvegicus GN=Tuba4a PE=2 SV=1	AYHEQLSVAEITNACFEPANQMVK
>	sp|Q5XIF6|TBA Tubulin alpha-4A chain OS=Rattus norvegicus GN=Tuba4a PE=2 SV=1	AYHEQLSVAEITNACFEPANQMVK
>	sp|Q5XIF6|TBA Tubulin alpha-4A chain OS=Rattus norvegicus GN=Tuba4a PE=2 SV=1	AYHEQLSVAEITNACFEPANQMVK
>	sp|Q5XIF6|TBA Tubulin alpha-4A chain OS=Rattus norvegicus GN=Tuba4a PE=2 SV=1	AYHEQLSVAEITNACFEPANQMVK
>	sp|Q5XIF6|TBA Tubulin alpha-4A chain OS=Rattus norvegicus GN=Tuba4a PE=2 SV=1	AYHEQLSVAEITNACFEPANQMVK
>	sp|Q5XIF6|TBA Tubulin alpha-4A chain OS=Rattus norvegicus GN=Tuba4a PE=2 SV=1	AYHEQLSVAEITNACFEPANQMVK
>	sp|Q5XIF6|TBA Tubulin alpha-4A chain OS=Rattus norvegicus GN=Tuba4a PE=2 SV=1	AYHEQLSVAEITNACFEPANQMVK
>	sp|Q5XIF6|TBA Tubulin alpha-4A chain OS=Rattus norvegicus GN=Tuba4a PE=2 SV=1	AYHEQLSVAEITNACFEPANQMVK
>	sp|Q5XIF6|TBA Tubulin alpha-4A chain OS=Rattus norvegicus GN=Tuba4a PE=2 SV=1	AYHEQLSVAEITNACFEPANQMVK
>	sp|Q5XIF6|TBA Tubulin alpha-4A chain OS=Rattus norvegicus GN=Tuba4a PE=2 SV=1	AYHEQLSVAEITNACFEPANQMVK
>	sp|Q5XIF6|TBA Tubulin alpha-4A chain OS=Rattus norvegicus GN=Tuba4a PE=2 SV=1	AYHEQLSVAEITNACFEPANQMVK
>	sp|Q5XIF6|TBA Tubulin alpha-4A chain OS=Rattus norvegicus GN=Tuba4a PE=2 SV=1	AYHEQLSVAEITNACFEPANQMVK
>	sp|Q5XIF6|TBA Tubulin alpha-4A chain OS=Rattus norvegicus GN=Tuba4a PE=2 SV=1	AYHEQLSVAEITNACFEPANQMVK
>	sp|Q5XIF6|TBA Tubulin alpha-4A chain OS=Rattus norvegicus GN=Tuba4a PE=2 SV=1	AYHEQLSVAEITNACFEPANQMVK
>	sp|Q5XIF6|TBA Tubulin alpha-4A chain OS=Rattus norvegicus GN=Tuba4a PE=2 SV=1	AYHEQLSVAEITNACFEPANQMVK
>	sp|Q5XIF6|TBA Tubulin alpha-4A chain OS=Rattus norvegicus GN=Tuba4a PE=2 SV=1	AYHEQLSVAEITNACFEPANQMVK
>	sp|Q5XIF6|TBA Tubulin alpha-4A chain OS=Rattus norvegicus GN=Tuba4a PE=2 SV=1	AYHEQLSVAEITNACFEPANQMVK
>	sp|Q5XIF6|TBA Tubulin alpha-4A chain OS=Rattus norvegicus GN=Tuba4a PE=2 SV=1	AYHEQLSVAEITNACFEPANQMVK
>	sp|Q5XIF6|TBA Tubulin alpha-4A chain OS=Rattus norvegicus GN=Tuba4a PE=2 SV=1	AYHEQLSVAEITNACFEPANQMVK
>	sp|Q5XIF6|TBA Tubulin alpha-4A chain OS=Rattus norvegicus GN=Tuba4a PE=2 SV=1	AYHEQLSVAEITNACFEPANQMVK
>	sp|Q5XIF6|TBA Tubulin alpha-4A chain OS=Rattus norvegicus GN=Tuba4a PE=2 SV=1	AYHEQLSVAEITNACFEPANQMVK
>	sp|Q5XIF6|TBA Tubulin alpha-4A chain OS=Rattus norvegicus GN=Tuba4a PE=2 SV=1	AYHEQLSVAEITNACFEPANQMVK
>	sp|Q5XIF6|TBA Tubulin alpha-4A chain OS=Rattus norvegicus GN=Tuba4a PE=2 SV=1	AYHEQLSVAEITNACFEPANQMVK
>	sp|Q5XIF6|TBA Tubulin alpha-4A chain OS=Rattus norvegicus GN=Tuba4a PE=2 SV=1	AYHEQLSVAEITNACFEPANQMVK
>	sp|Q5XIF6|TBA Tubulin alpha-4A chain OS=Rattus norvegicus GN=Tuba4a PE=2 SV=1	AYHEQLSVAEITNACFEPANQMVK
>	sp|Q5XIF6|TBA Tubulin alpha-4A chain OS=Rattus norvegicus GN=Tuba4a PE=2 SV=1	AYHEQLSVAEITNACFEPANQMVK
>	sp|Q5XIF6|TBA Tubulin alpha-4A chain OS=Rattus norvegicus GN=Tuba4a PE=2 SV=1	AYHEQLSVAEITNACFEPANQMVK
>	sp|Q5XIF6|TBA Tubulin alpha-4A chain OS=Rattus norvegicus GN=Tuba4a PE=2 SV=1	AYHEQLSVAEITNACFEPANQMVK
>	sp|Q5XIF6|TBA Tubulin alpha-4A chain OS=Rattus norvegicus GN=Tuba4a PE=2 SV=1	DVNAAIAAIK
>	sp|Q5XIF6|TBA Tubulin alpha-4A chain OS=Rattus norvegicus GN=Tuba4a PE=2 SV=1	DVNAAIAAIK
>	sp|Q5XIF6|TBA Tubulin alpha-4A chain OS=Rattus norvegicus GN=Tuba4a PE=2 SV=1	DVNAAIAAIK
>	sp|Q5XIF6|TBA Tubulin alpha-4A chain OS=Rattus norvegicus GN=Tuba4a PE=2 SV=1	DVNAAIAAIK
>	sp|Q5XIF6|TBA Tubulin alpha-4A chain OS=Rattus norvegicus GN=Tuba4a PE=2 SV=1	ECISVHVGQAGVQMGNACWELYCLEHGIQPDGQMPSDK
>	sp|Q5XIF6|TBA Tubulin alpha-4A chain OS=Rattus norvegicus GN=Tuba4a PE=2 SV=1	ECISVHVGQAGVQMGNACWELYCLEHGIQPDGQMPSDK
>	sp|Q5XIF6|TBA Tubulin alpha-4A chain OS=Rattus norvegicus GN=Tuba4a PE=2 SV=1	ECISVHVGQAGVQMGNACWELYCLEHGIQPDGQMPSDK
>	sp|Q5XIF6|TBA Tubulin alpha-4A chain OS=Rattus norvegicus GN=Tuba4a PE=2 SV=1	ECISVHVGQAGVQMGNACWELYCLEHGIQPDGQMPSDK
>	sp|Q5XIF6|TBA Tubulin alpha-4A chain OS=Rattus norvegicus GN=Tuba4a PE=2 SV=1	ECISVHVGQAGVQMGNACWELYCLEHGIQPDGQMPSDK
>	sp|Q5XIF6|TBA Tubulin alpha-4A chain OS=Rattus norvegicus GN=Tuba4a PE=2 SV=1	ECISVHVGQAGVQMGNACWELYCLEHGIQPDGQMPSDK
>	sp|Q5XIF6|TBA Tubulin alpha-4A chain OS=Rattus norvegicus GN=Tuba4a PE=2 SV=1	ECISVHVGQAGVQMGNACWELYCLEHGIQPDGQMPSDK
>	sp|Q5XIF6|TBA Tubulin alpha-4A chain OS=Rattus norvegicus GN=Tuba4a PE=2 SV=1	ECISVHVGQAGVQMGNACWELYCLEHGIQPDGQMPSDK
>	sp|Q5XIF6|TBA Tubulin alpha-4A chain OS=Rattus norvegicus GN=Tuba4a PE=2 SV=1	ECISVHVGQAGVQMGNACWELYCLEHGIQPDGQMPSDK
>	sp|Q5XIF6|TBA Tubulin alpha-4A chain OS=Rattus norvegicus GN=Tuba4a PE=2 SV=1	ECISVHVGQAGVQMGNACWELYCLEHGIQPDGQMPSDK
>	sp|Q5XIF6|TBA Tubulin alpha-4A chain OS=Rattus norvegicus GN=Tuba4a PE=2 SV=1	FDGALNVDLTEFQTNLVPYPR
>	sp|Q5XIF6|TBA Tubulin alpha-4A chain OS=Rattus norvegicus GN=Tuba4a PE=2 SV=1	FDGALNVDLTEFQTNLVPYPR
>	sp|Q5XIF6|TBA Tubulin alpha-4A chain OS=Rattus norvegicus GN=Tuba4a PE=2 SV=1	FDGALNVDLTEFQTNLVPYPR
>	sp|Q5XIF6|TBA Tubulin alpha-4A chain OS=Rattus norvegicus GN=Tuba4a PE=2 SV=1	FDGALNVDLTEFQTNLVPYPR
>	sp|Q5XIF6|TBA Tubulin alpha-4A chain OS=Rattus norvegicus GN=Tuba4a PE=2 SV=1	FDGALNVDLTEFQTNLVPYPR
>	sp|Q5XIF6|TBA Tubulin alpha-4A chain OS=Rattus norvegicus GN=Tuba4a PE=2 SV=1	IHFPLATYAPVISAEK
>	sp|Q5XIF6|TBA Tubulin alpha-4A chain OS=Rattus norvegicus GN=Tuba4a PE=2 SV=1	IHFPLATYAPVISAEK
>	sp|Q5XIF6|TBA Tubulin alpha-4A chain OS=Rattus norvegicus GN=Tuba4a PE=2 SV=1	IHFPLATYAPVISAEK
>	sp|Q5XIF6|TBA Tubulin alpha-4A chain OS=Rattus norvegicus GN=Tuba4a PE=2 SV=1	IHFPLATYAPVISAEK
>	sp|Q5XIF6|TBA Tubulin alpha-4A chain OS=Rattus norvegicus GN=Tuba4a PE=2 SV=1	IHFPLATYAPVISAEK
>	sp|Q5XIF6|TBA Tubulin alpha-4A chain OS=Rattus norvegicus GN=Tuba4a PE=2 SV=1	IHFPLATYAPVISAEK
>	sp|Q5XIF6|TBA Tubulin alpha-4A chain OS=Rattus norvegicus GN=Tuba4a PE=2 SV=1	IHFPLATYAPVISAEK
>	sp|Q5XIF6|TBA Tubulin alpha-4A chain OS=Rattus norvegicus GN=Tuba4a PE=2 SV=1	IHFPLATYAPVISAEK
>	sp|Q5XIF6|TBA Tubulin alpha-4A chain OS=Rattus norvegicus GN=Tuba4a PE=2 SV=1	IHFPLATYAPVISAEK
>	sp|Q5XIF6|TBA Tubulin alpha-4A chain OS=Rattus norvegicus GN=Tuba4a PE=2 SV=1	IHFPLATYAPVISAEK

>	sp|Q5XIF6|TBA Tubulin alpha-4A chain OS=Rattus norvegicus GN=Tuba4a PE=2 SV=1	IHFPLATYAPVISAEK
>	sp|Q5XIF6|TBA Tubulin alpha-4A chain OS=Rattus norvegicus GN=Tuba4a PE=2 SV=1	IHFPLATYAPVISAEK
>	sp|Q5XIF6|TBA Tubulin alpha-4A chain OS=Rattus norvegicus GN=Tuba4a PE=2 SV=1	IHFPLATYAPVISAEK
>	sp|Q5XIF6|TBA Tubulin alpha-4A chain OS=Rattus norvegicus GN=Tuba4a PE=2 SV=1	IHFPLATYAPVISAEK
>	sp|Q5XIF6|TBA Tubulin alpha-4A chain OS=Rattus norvegicus GN=Tuba4a PE=2 SV=1	IHFPLATYAPVISAEK
>	sp|Q5XIF6|TBA Tubulin alpha-4A chain OS=Rattus norvegicus GN=Tuba4a PE=2 SV=1	IHFPLATYAPVISAEK
>	sp|Q5XIF6|TBA Tubulin alpha-4A chain OS=Rattus norvegicus GN=Tuba4a PE=2 SV=1	IHFPLATYAPVISAEK
>	sp|Q5XIF6|TBA Tubulin alpha-4A chain OS=Rattus norvegicus GN=Tuba4a PE=2 SV=1	IHFPLATYAPVISAEK
>	sp|Q5XIF6|TBA Tubulin alpha-4A chain OS=Rattus norvegicus GN=Tuba4a PE=2 SV=1	IHFPLATYAPVISAEK
>	sp|Q5XIF6|TBA Tubulin alpha-4A chain OS=Rattus norvegicus GN=Tuba4a PE=2 SV=1	IHFPLATYAPVISAEK
>	sp|Q5XIF6|TBA Tubulin alpha-4A chain OS=Rattus norvegicus GN=Tuba4a PE=2 SV=1	IHFPLATYAPVISAEK
>	sp|Q5XIF6|TBA Tubulin alpha-4A chain OS=Rattus norvegicus GN=Tuba4a PE=2 SV=1	IHFPLATYAPVISAEK
>	sp|Q5XIF6|TBA Tubulin alpha-4A chain OS=Rattus norvegicus GN=Tuba4a PE=2 SV=1	IHFPLATYAPVISAEK
>	sp|Q5XIF6|TBA Tubulin alpha-4A chain OS=Rattus norvegicus GN=Tuba4a PE=2 SV=1	IHFPLATYAPVISAEK
>	sp|Q5XIF6|TBA Tubulin alpha-4A chain OS=Rattus norvegicus GN=Tuba4a PE=2 SV=1	IHFPLATYAPVISAEK
>	sp|Q5XIF6|TBA Tubulin alpha-4A chain OS=Rattus norvegicus GN=Tuba4a PE=2 SV=1	IHFPLATYAPVISAEK
>	sp|Q5XIF6|TBA Tubulin alpha-4A chain OS=Rattus norvegicus GN=Tuba4a PE=2 SV=1	IHFPLATYAPVISAEK
>	sp|Q5XIF6|TBA Tubulin alpha-4A chain OS=Rattus norvegicus GN=Tuba4a PE=2 SV=1	IHFPLATYAPVISAEK
>	sp|Q5XIF6|TBA Tubulin alpha-4A chain OS=Rattus norvegicus GN=Tuba4a PE=2 SV=1	IHFPLATYAPVISAEK
>	sp|Q5XIF6|TBA Tubulin alpha-4A chain OS=Rattus norvegicus GN=Tuba4a PE=2 SV=1	IHFPLATYAPVISAEK
>	sp|Q5XIF6|TBA Tubulin alpha-4A chain OS=Rattus norvegicus GN=Tuba4a PE=2 SV=1	IHFPLATYAPVISAEK
>	sp|Q5XIF6|TBA Tubulin alpha-4A chain OS=Rattus norvegicus GN=Tuba4a PE=2 SV=1	IHFPLATYAPVISAEK
>	sp|Q5XIF6|TBA Tubulin alpha-4A chain OS=Rattus norvegicus GN=Tuba4a PE=2 SV=1	IHFPLATYAPVISAEK
>	sp|Q5XIF6|TBA Tubulin alpha-4A chain OS=Rattus norvegicus GN=Tuba4a PE=2 SV=1	IHFPLATYAPVISAEK
>	sp|Q5XIF6|TBA Tubulin alpha-4A chain OS=Rattus norvegicus GN=Tuba4a PE=2 SV=1	IHFPLATYAPVISAEK
>	sp|Q5XIF6|TBA Tubulin alpha-4A chain OS=Rattus norvegicus GN=Tuba4a PE=2 SV=1	IHFPLATYAPVISAEK
>	sp|Q5XIF6|TBA Tubulin alpha-4A chain OS=Rattus norvegicus GN=Tuba4a PE=2 SV=1	IHFPLATYAPVISAEK
>	sp|Q5XIF6|TBA Tubulin alpha-4A chain OS=Rattus norvegicus GN=Tuba4a PE=2 SV=1	IHFPLATYAPVISAEK
>	sp|Q5XIF6|TBA Tubulin alpha-4A chain OS=Rattus norvegicus GN=Tuba4a PE=2 SV=1	IHFPLATYAPVISAEK
>	sp|Q5XIF6|TBA Tubulin alpha-4A chain OS=Rattus norvegicus GN=Tuba4a PE=2 SV=1	IHFPLATYAPVISAEK
>	sp|Q5XIF6|TBA Tubulin alpha-4A chain OS=Rattus norvegicus GN=Tuba4a PE=2 SV=1	IHFPLATYAPVISAEK
>	sp|Q5XIF6|TBA Tubulin alpha-4A chain OS=Rattus norvegicus GN=Tuba4a PE=2 SV=1	IHFPLATYAPVISAEK
>	sp|Q5XIF6|TBA Tubulin alpha-4A chain OS=Rattus norvegicus GN=Tuba4a PE=2 SV=1	IHFPLATYAPVISAEK
>	sp|Q5XIF6|TBA Tubulin alpha-4A chain OS=Rattus norvegicus GN=Tuba4a PE=2 SV=1	IHFPLATYAPVISAEK
>	sp|Q5XIF6|TBA Tubulin alpha-4A chain OS=Rattus norvegicus GN=Tuba4a PE=2 SV=1	IHFPLATYAPVISAEK
>	sp|Q5XIF6|TBA Tubulin alpha-4A chain OS=Rattus norvegicus GN=Tuba4a PE=2 SV=1	IHFPLATYAPVISAEK
>	sp|Q5XIF6|TBA Tubulin alpha-4A chain OS=Rattus norvegicus GN=Tuba4a PE=2 SV=1	IHFPLATYAPVISAEK
>	sp|Q5XIF6|TBA Tubulin alpha-4A chain OS=Rattus norvegicus GN=Tuba4a PE=2 SV=1	IHFPLATYAPVISAEK
>	sp|Q5XIF6|TBA Tubulin alpha-4A chain OS=Rattus norvegicus GN=Tuba4a PE=2 SV=1	IHFPLATYAPVISAEK
>	sp|Q5XIF6|TBA Tubulin alpha-4A chain OS=Rattus norvegicus GN=Tuba4a PE=2 SV=1	IHFPLATYAPVISAEK
>	sp|Q5XIF6|TBA Tubulin alpha-4A chain OS=Rattus norvegicus GN=Tuba4a PE=2 SV=1	IHFPLATYAPVISAEK
>	sp|Q5XIF6|TBA Tubulin alpha-4A chain OS=Rattus norvegicus GN=Tuba4a PE=2 SV=1	IHFPLATYAPVISAEK
>	sp|Q5XIF6|TBA Tubulin alpha-4A chain OS=Rattus norvegicus GN=Tuba4a PE=2 SV=1	IHFPLATYAPVISAEK
>	sp|Q5XIF6|TBA Tubulin alpha-4A chain OS=Rattus norvegicus GN=Tuba4a PE=2 SV=1	IHFPLATYAPVISAEK
>	sp|Q5XIF6|TBA Tubulin alpha-4A chain OS=Rattus norvegicus GN=Tuba4a PE=2 SV=1	IHFPLATYAPVISAEK
>	sp|Q5XIF6|TBA Tubulin alpha-4A chain OS=Rattus norvegicus GN=Tuba4a PE=2 SV=1	IHFPLATYAPVISAEK
>	sp|Q5XIF6|TBA Tubulin alpha-4A chain OS=Rattus norvegicus GN=Tuba4a PE=2 SV=1	IHFPLATYAPVISAEK
>	sp|Q5XIF6|TBA Tubulin alpha-4A chain OS=Rattus norvegicus GN=Tuba4a PE=2 SV=1	IHFPLATYAPVISAEK
>	sp|Q5XIF6|TBA Tubulin alpha-4A chain OS=Rattus norvegicus GN=Tuba4a PE=2 SV=1	IHFPLATYAPVISAEK
>	sp|Q5XIF6|TBA Tubulin alpha-4A chain OS=Rattus norvegicus GN=Tuba4a PE=2 SV=1	IHFPLATYAPVISAEK
>	sp|Q5XIF6|TBA Tubulin alpha-4A chain OS=Rattus norvegicus GN=Tuba4a PE=2 SV=1	KLSDQCTGLQGFLVFHSFGGGTGSGFTSLLMER
>	sp|Q5XIF6|TBA Tubulin alpha-4A chain OS=Rattus norvegicus GN=Tuba4a PE=2 SV=1	KLSDQCTGLQGFLVFHSFGGGTGSGFTSLLMER
>	sp|Q5XIF6|TBA Tubulin alpha-4A chain OS=Rattus norvegicus GN=Tuba4a PE=2 SV=1	KLSDQCTGLQGFLVFHSFGGGTGSGFTSLLMER
>	sp|Q5XIF6|TBA Tubulin alpha-4A chain OS=Rattus norvegicus GN=Tuba4a PE=2 SV=1	KLSDQCTGLQGFLVFHSFGGGTGSGFTSLLMER
>	sp|Q5XIF6|TBA Tubulin alpha-4A chain OS=Rattus norvegicus GN=Tuba4a PE=2 SV=1	LDHKFDLMYAK
>	sp|Q5XIF6|TBA Tubulin alpha-4A chain OS=Rattus norvegicus GN=Tuba4a PE=2 SV=1	LDHKFDLMYAK
>	sp|Q5XIF6|TBA Tubulin alpha-4A chain OS=Rattus norvegicus GN=Tuba4a PE=2 SV=1	LEFSIYPAPQVSTAVVEPYNSILTTHTTLEHSDCAFMVDNEAIYDICR
>	sp|Q5XIF6|TBA Tubulin alpha-4A chain OS=Rattus norvegicus GN=Tuba4a PE=2 SV=1	LEFSIYPAPQVSTAVVEPYNSILTTHTTLEHSDCAFMVDNEAIYDICR
>	sp|Q5XIF6|TBA Tubulin alpha-4A chain OS=Rattus norvegicus GN=Tuba4a PE=2 SV=1	LEFSIYPAPQVSTAVVEPYNSILTTHTTLEHSDCAFMVDNEAIYDICR
>	sp|Q5XIF6|TBA Tubulin alpha-4A chain OS=Rattus norvegicus GN=Tuba4a PE=2 SV=1	LEFSIYPAPQVSTAVVEPYNSILTTHTTLEHSDCAFMVDNEAIYDICR
>	sp|Q5XIF6|TBA Tubulin alpha-4A chain OS=Rattus norvegicus GN=Tuba4a PE=2 SV=1	LEFSIYPAPQVSTAVVEPYNSILTTHTTLEHSDCAFMVDNEAIYDICR
>	sp|Q5XIF6|TBA Tubulin alpha-4A chain OS=Rattus norvegicus GN=Tuba4a PE=2 SV=1	LEFSIYPAPQVSTAVVEPYNSILTTHTTLEHSDCAFMVDNEAIYDICR
>	sp|Q5XIF6|TBA Tubulin alpha-4A chain OS=Rattus norvegicus GN=Tuba4a PE=2 SV=1	LEFSIYPAPQVSTAVVEPYNSILTTHTTLEHSDCAFMVDNEAIYDICR
>	sp|Q5XIF6|TBA Tubulin alpha-4A chain OS=Rattus norvegicus GN=Tuba4a PE=2 SV=1	LEFSIYPAPQVSTAVVEPYNSILTTHTTLEHSDCAFMVDNEAIYDICR
>	sp|Q5XIF6|TBA Tubulin alpha-4A chain OS=Rattus norvegicus GN=Tuba4a PE=2 SV=1	LEFSIYPAPQVSTAVVEPYNSILTTHTTLEHSDCAFMVDNEAIYDICR
>	sp|Q5XIF6|TBA Tubulin alpha-4A chain OS=Rattus norvegicus GN=Tuba4a PE=2 SV=1	LEFSIYPAPQVSTAVVEPYNSILTTHTTLEHSDCAFMVDNEAIYDICR
>	sp|Q5XIF6|TBA Tubulin alpha-4A chain OS=Rattus norvegicus GN=Tuba4a PE=2 SV=1	LEFSIYPAPQVSTAVVEPYNSILTTHTTLEHSDCAFMVDNEAIYDICR
>	sp|Q5XIF6|TBA Tubulin alpha-4A chain OS=Rattus norvegicus GN=Tuba4a PE=2 SV=1	LEFSIYPAPQVSTAVVEPYNSILTTHTTLEHSDCAFMVDNEAIYDICR
>	sp|Q5XIF6|TBA Tubulin alpha-4A chain OS=Rattus norvegicus GN=Tuba4a PE=2 SV=1	LEFSIYPAPQVSTAVVEPYNSILTTHTTLEHSDCAFMVDNEAIYDICR
>	sp|Q5XIF6|TBA Tubulin alpha-4A chain OS=Rattus norvegicus GN=Tuba4a PE=2 SV=1	LEFSIYPAPQVSTAVVEPYNSILTTHTTLEHSDCAFMVDNEAIYDICR
>	sp|Q5XIF6|TBA Tubulin alpha-4A chain OS=Rattus norvegicus GN=Tuba4a PE=2 SV=1	LEFSIYPAPQVSTAVVEPYNSILTTHTTLEHSDCAFMVDNEAIYDICR
>	sp|Q5XIF6|TBA Tubulin alpha-4A chain OS=Rattus norvegicus GN=Tuba4a PE=2 SV=1	LEFSIYPAPQVSTAVVEPYNSILTTHTTLEHSDCAFMVDNEAIYDICR
>	sp|Q5XIF6|TBA Tubulin alpha-4A chain OS=Rattus norvegicus GN=Tuba4a PE=2 SV=1	LEFSIYPAPQVSTAVVEPYNSILTTHTTLEHSDCAFMVDNEAIYDICR
>	sp|Q5XIF6|TBA Tubulin alpha-4A chain OS=Rattus norvegicus GN=Tuba4a PE=2 SV=1	LEFSIYPAPQVSTAVVEPYNSILTTHTTLEHSDCAFMVDNEAIYDICR

>	sp|Q5XIF6|TBA Tubulin alpha-4A chain OS=Rattus norvegicus GN=Tuba4a PE=2 SV=1	LEFSIYPAPQVSTAVVEPYNSILTTHTTLEHSDCAFMVDNEAIYDICR
>	sp|Q5XIF6|TBA Tubulin alpha-4A chain OS=Rattus norvegicus GN=Tuba4a PE=2 SV=1	LEFSIYPAPQVSTAVVEPYNSILTTHTTLEHSDCAFMVDNEAIYDICR
>	sp|Q5XIF6|TBA Tubulin alpha-4A chain OS=Rattus norvegicus GN=Tuba4a PE=2 SV=1	LEFSIYPAPQVSTAVVEPYNSILTTHTTLEHSDCAFMVDNEAIYDICR
>	sp|Q5XIF6|TBA Tubulin alpha-4A chain OS=Rattus norvegicus GN=Tuba4a PE=2 SV=1	NLDIERPTYTNLNRLISQIVSSITASLRFDGALNVDLTEFQTNLVPYPR
>	sp|Q5XIF6|TBA Tubulin alpha-4A chain OS=Rattus norvegicus GN=Tuba4a PE=2 SV=1	NLDIERPTYTNLNRLISQIVSSITASLRFDGALNVDLTEFQTNLVPYPR
>	sp|Q5XIF6|TBA Tubulin alpha-4A chain OS=Rattus norvegicus GN=Tuba4a PE=2 SV=1	NLDIERPTYTNLNRLISQIVSSITASLRFDGALNVDLTEFQTNLVPYPR
>	sp|Q5XIF6|TBA Tubulin alpha-4A chain OS=Rattus norvegicus GN=Tuba4a PE=2 SV=1	QLFHPEQLITGK
>	sp|Q5XIF6|TBA Tubulin alpha-4A chain OS=Rattus norvegicus GN=Tuba4a PE=2 SV=1	QLFHPEQLITGK
>	sp|Q5XIF6|TBA Tubulin alpha-4A chain OS=Rattus norvegicus GN=Tuba4a PE=2 SV=1	QLFHPEQLITGK
>	sp|Q5XIF6|TBA Tubulin alpha-4A chain OS=Rattus norvegicus GN=Tuba4a PE=2 SV=1	QLFHPEQLITGK
>	sp|Q5XIF6|TBA Tubulin alpha-4A chain OS=Rattus norvegicus GN=Tuba4a PE=2 SV=1	QLFHPEQLITGKEDAANNYAR
>	sp|Q5XIF6|TBA Tubulin alpha-4A chain OS=Rattus norvegicus GN=Tuba4a PE=2 SV=1	QLFHPEQLITGKEDAANNYAR
>	sp|Q5XIF6|TBA Tubulin alpha-4A chain OS=Rattus norvegicus GN=Tuba4a PE=2 SV=1	QLFHPEQLITGKEDAANNYAR
>	sp|Q5XIF6|TBA Tubulin alpha-4A chain OS=Rattus norvegicus GN=Tuba4a PE=2 SV=1	QLFHPEQLITGKEDAANNYAR
>	sp|Q5XIF6|TBA Tubulin alpha-4A chain OS=Rattus norvegicus GN=Tuba4a PE=2 SV=1	QLFHPEQLITGKEDAANNYAR
>	sp|Q5XIF6|TBA Tubulin alpha-4A chain OS=Rattus norvegicus GN=Tuba4a PE=2 SV=1	QLFHPEQLITGKEDAANNYAR
>	sp|Q5XIF6|TBA Tubulin alpha-4A chain OS=Rattus norvegicus GN=Tuba4a PE=2 SV=1	QLFHPEQLITGKEDAANNYAR
>	sp|Q5XIF6|TBA Tubulin alpha-4A chain OS=Rattus norvegicus GN=Tuba4a PE=2 SV=1	QLFHPEQLITGKEDAANNYAR
>	sp|Q5XIF6|TBA Tubulin alpha-4A chain OS=Rattus norvegicus GN=Tuba4a PE=2 SV=1	QLFHPEQLITGKEDAANNYAR
>	sp|Q5XIF6|TBA Tubulin alpha-4A chain OS=Rattus norvegicus GN=Tuba4a PE=2 SV=1	QLFHPEQLITGKEDAANNYAR
>	sp|Q5XIF6|TBA Tubulin alpha-4A chain OS=Rattus norvegicus GN=Tuba4a PE=2 SV=1	QLFHPEQLITGKEDAANNYAR
>	sp|Q5XIF6|TBA Tubulin alpha-4A chain OS=Rattus norvegicus GN=Tuba4a PE=2 SV=1	QLFHPEQLITGKEDAANNYAR
>	sp|Q5XIF6|TBA Tubulin alpha-4A chain OS=Rattus norvegicus GN=Tuba4a PE=2 SV=1	QLFHPEQLITGKEDAANNYAR
>	sp|Q5XIF6|TBA Tubulin alpha-4A chain OS=Rattus norvegicus GN=Tuba4a PE=2 SV=1	QLFHPEQLITGKEDAANNYAR
>	sp|Q5XIF6|TBA Tubulin alpha-4A chain OS=Rattus norvegicus GN=Tuba4a PE=2 SV=1	QLFHPEQLITGKEDAANNYAR
>	sp|Q5XIF6|TBA Tubulin alpha-4A chain OS=Rattus norvegicus GN=Tuba4a PE=2 SV=1	QLFHPEQLITGKEDAANNYAR
>	sp|Q5XIF6|TBA Tubulin alpha-4A chain OS=Rattus norvegicus GN=Tuba4a PE=2 SV=1	QLFHPEQLITGKEDAANNYAR
>	sp|Q5XIF6|TBA Tubulin alpha-4A chain OS=Rattus norvegicus GN=Tuba4a PE=2 SV=1	QLFHPEQLITGKEDAANNYAR
>	sp|Q5XIF6|TBA Tubulin alpha-4A chain OS=Rattus norvegicus GN=Tuba4a PE=2 SV=1	QLFHPEQLITGKEDAANNYAR
>	sp|Q5XIF6|TBA Tubulin alpha-4A chain OS=Rattus norvegicus GN=Tuba4a PE=2 SV=1	QLFHPEQLITGKEDAANNYAR
>	sp|Q5XIF6|TBA Tubulin alpha-4A chain OS=Rattus norvegicus GN=Tuba4a PE=2 SV=1	QLFHPEQLITGKEDAANNYAR
>	sp|Q5XIF6|TBA Tubulin alpha-4A chain OS=Rattus norvegicus GN=Tuba4a PE=2 SV=1	QLFHPEQLITGKEDAANNYAR
>	sp|Q5XIF6|TBA Tubulin alpha-4A chain OS=Rattus norvegicus GN=Tuba4a PE=2 SV=1	QLFHPEQLITGKEDAANNYAR
>	sp|Q5XIF6|TBA Tubulin alpha-4A chain OS=Rattus norvegicus GN=Tuba4a PE=2 SV=1	QLFHPEQLITGKEDAANNYAR
>	sp|Q5XIF6|TBA Tubulin alpha-4A chain OS=Rattus norvegicus GN=Tuba4a PE=2 SV=1	QLFHPEQLITGKEDAANNYAR
>	sp|Q5XIF6|TBA Tubulin alpha-4A chain OS=Rattus norvegicus GN=Tuba4a PE=2 SV=1	QLFHPEQLITGKEDAANNYAR
>	sp|Q5XIF6|TBA Tubulin alpha-4A chain OS=Rattus norvegicus GN=Tuba4a PE=2 SV=1	QLFHPEQLITGKEDAANNYAR
>	sp|Q5XIF6|TBA Tubulin alpha-4A chain OS=Rattus norvegicus GN=Tuba4a PE=2 SV=1	QLFHPEQLITGKEDAANNYAR
>	sp|Q5XIF6|TBA Tubulin alpha-4A chain OS=Rattus norvegicus GN=Tuba4a PE=2 SV=1	QLFHPEQLITGKEDAANNYAR
>	sp|Q5XIF6|TBA Tubulin alpha-4A chain OS=Rattus norvegicus GN=Tuba4a PE=2 SV=1	RAFVHWYVGEGMEEGEFSEAR
>	sp|Q5XIF6|TBA Tubulin alpha-4A chain OS=Rattus norvegicus GN=Tuba4a PE=2 SV=1	RAFVHWYVGEGMEEGEFSEAR
>	sp|Q5XIF6|TBA Tubulin alpha-4A chain OS=Rattus norvegicus GN=Tuba4a PE=2 SV=1	RAFVHWYVGEGMEEGEFSEAR
>	sp|Q5XIF6|TBA Tubulin alpha-4A chain OS=Rattus norvegicus GN=Tuba4a PE=2 SV=1	SIQFVDWCPTGFK
>	sp|Q5XIF6|TBA Tubulin alpha-4A chain OS=Rattus norvegicus GN=Tuba4a PE=2 SV=1	SIQFVDWCPTGFK
>	sp|Q5XIF6|TBA Tubulin alpha-4A chain OS=Rattus norvegicus GN=Tuba4a PE=2 SV=1	SIQFVDWCPTGFK
>	sp|Q5XIF6|TBA Tubulin alpha-4A chain OS=Rattus norvegicus GN=Tuba4a PE=2 SV=1	SIQFVDWCPTGFK
>	sp|Q5XIF6|TBA Tubulin alpha-4A chain OS=Rattus norvegicus GN=Tuba4a PE=2 SV=1	SIQFVDWCPTGFK
>	sp|Q5XIF6|TBA Tubulin alpha-4A chain OS=Rattus norvegicus GN=Tuba4a PE=2 SV=1	SIQFVDWCPTGFK
>	sp|Q5XIF6|TBA Tubulin alpha-4A chain OS=Rattus norvegicus GN=Tuba4a PE=2 SV=1	SIQFVDWCPTGFK
>	sp|Q5XIF6|TBA Tubulin alpha-4A chain OS=Rattus norvegicus GN=Tuba4a PE=2 SV=1	SIQFVDWCPTGFK
>	sp|Q5XIF6|TBA Tubulin alpha-4A chain OS=Rattus norvegicus GN=Tuba4a PE=2 SV=1	SKLEFSIYPAPQVSTAVVEPYNSILTTHTTLEHSDCAFMVDNEAIYDICR
>	sp|Q5XIF6|TBA Tubulin alpha-4A chain OS=Rattus norvegicus GN=Tuba4a PE=2 SV=1	SKLEFSIYPAPQVSTAVVEPYNSILTTHTTLEHSDCAFMVDNEAIYDICR
>	sp|Q5XIF6|TBA Tubulin alpha-4A chain OS=Rattus norvegicus GN=Tuba4a PE=2 SV=1	SKLEFSIYPAPQVSTAVVEPYNSILTTHTTLEHSDCAFMVDNEAIYDICR
>	sp|Q5XIF6|TBA Tubulin alpha-4A chain OS=Rattus norvegicus GN=Tuba4a PE=2 SV=1	SKLEFSIYPAPQVSTAVVEPYNSILTTHTTLEHSDCAFMVDNEAIYDICR
>	sp|Q5XIF6|TBA Tubulin alpha-4A chain OS=Rattus norvegicus GN=Tuba4a PE=2 SV=1	SKLEFSIYPAPQVSTAVVEPYNSILTTHTTLEHSDCAFMVDNEAIYDICR
>	sp|Q5XIF6|TBA Tubulin alpha-4A chain OS=Rattus norvegicus GN=Tuba4a PE=2 SV=1	SKLEFSIYPAPQVSTAVVEPYNSILTTHTTLEHSDCAFMVDNEAIYDICR
>	sp|Q5XIF6|TBA Tubulin alpha-4A chain OS=Rattus norvegicus GN=Tuba4a PE=2 SV=1	SKLEFSIYPAPQVSTAVVEPYNSILTTHTTLEHSDCAFMVDNEAIYDICR
>	sp|Q5XIF6|TBA Tubulin alpha-4A chain OS=Rattus norvegicus GN=Tuba4a PE=2 SV=1	SKLEFSIYPAPQVSTAVVEPYNSILTTHTTLEHSDCAFMVDNEAIYDICR
>	sp|Q5XIF6|TBA Tubulin alpha-4A chain OS=Rattus norvegicus GN=Tuba4a PE=2 SV=1	SKLEFSIYPAPQVSTAVVEPYNSILTTHTTLEHSDCAFMVDNEAIYDICR
>	sp|Q5XIF6|TBA Tubulin alpha-4A chain OS=Rattus norvegicus GN=Tuba4a PE=2 SV=1	SKLEFSIYPAPQVSTAVVEPYNSILTTHTTLEHSDCAFMVDNEAIYDICR
>	sp|Q5XIF6|TBA Tubulin alpha-4A chain OS=Rattus norvegicus GN=Tuba4a PE=2 SV=1	SKLEFSIYPAPQVSTAVVEPYNSILTTHTTLEHSDCAFMVDNEAIYDICR
>	sp|Q5XIF6|TBA Tubulin alpha-4A chain OS=Rattus norvegicus GN=Tuba4a PE=2 SV=1	SKLEFSIYPAPQVSTAVVEPYNSILTTHTTLEHSDCAFMVDNEAIYDICR
>	sp|Q5XIF6|TBA Tubulin alpha-4A chain OS=Rattus norvegicus GN=Tuba4a PE=2 SV=1	SKLEFSIYPAPQVSTAVVEPYNSILTTHTTLEHSDCAFMVDNEAIYDICR
>	sp|Q5XIF6|TBA Tubulin alpha-4A chain OS=Rattus norvegicus GN=Tuba4a PE=2 SV=1	SKLEFSIYPAPQVSTAVVEPYNSILTTHTTLEHSDCAFMVDNEAIYDICR
>	sp|Q5XIF6|TBA Tubulin alpha-4A chain OS=Rattus norvegicus GN=Tuba4a PE=2 SV=1	SKLEFSIYPAPQVSTAVVEPYNSILTTHTTLEHSDCAFMVDNEAIYDICR
>	sp|Q5XIF6|TBA Tubulin alpha-4A chain OS=Rattus norvegicus GN=Tuba4a PE=2 SV=1	SKLEFSIYPAPQVSTAVVEPYNSILTTHTTLEHSDCAFMVDNEAIYDICR
>	sp|Q5XIF6|TBA Tubulin alpha-4A chain OS=Rattus norvegicus GN=Tuba4a PE=2 SV=1	SKLEFSIYPAPQVSTAVVEPYNSILTTHTTLEHSDCAFMVDNEAIYDICR
>	sp|Q5XIF6|TBA Tubulin alpha-4A chain OS=Rattus norvegicus GN=Tuba4a PE=2 SV=1	SKLEFSIYPAPQVSTAVVEPYNSILTTHTTLEHSDCAFMVDNEAIYDICR
>	sp|Q5XIF6|TBA Tubulin alpha-4A chain OS=Rattus norvegicus GN=Tuba4a PE=2 SV=1	SKLEFSIYPAPQVSTAVVEPYNSILTTHTTLEHSDCAFMVDNEAIYDICR
>	sp|Q5XIF6|TBA Tubulin alpha-4A chain OS=Rattus norvegicus GN=Tuba4a PE=2 SV=1	SKLEFSIYPAPQVSTAVVEPYNSILTTHTTLEHSDCAFMVDNEAIYDICR
>	sp|Q5XIF6|TBA Tubulin alpha-4A chain OS=Rattus norvegicus GN=Tuba4a PE=2 SV=1	SKLEFSIYPAPQVSTAVVEPYNSILTTHTTLEHSDCAFMVDNEAIYDICR
>	sp|Q5XIF6|TBA Tubulin alpha-4A chain OS=Rattus norvegicus GN=Tuba4a PE=2 SV=1	SKLEFSIYPAPQVSTAVVEPYNSILTTHTTLEHSDCAFMVDNEAIYDICR
>	sp|Q5XIF6|TBA Tubulin alpha-4A chain OS=Rattus norvegicus GN=Tuba4a PE=2 SV=1	SKLEFSIYPAPQVSTAVVEPYNSILTTHTTLEHSDCAFMVDNEAIYDICR
>	sp|Q5XIF6|TBA Tubulin alpha-4A chain OS=Rattus norvegicus GN=Tuba4a PE=2 SV=1	SKLEFSIYPAPQVSTAVVEPYNSILTTHTTLEHSDCAFMVDNEAIYDICR

>	sp|Q5XIF6|TBA Tubulin alpha-4A chain OS=Rattus norvegicus GN=Tuba4a PE=2 SV=1	SKLEFSIYPAPQVSTAVVEPYNSILTTHTTLEHSDCAFMVDNEAIYDICR
>	sp|Q5XIF6|TBA Tubulin alpha-4A chain OS=Rattus norvegicus GN=Tuba4a PE=2 SV=1	SKLEFSIYPAPQVSTAVVEPYNSILTTHTTLEHSDCAFMVDNEAIYDICR
>	sp|Q5XIF6|TBA Tubulin alpha-4A chain OS=Rattus norvegicus GN=Tuba4a PE=2 SV=1	SKLEFSIYPAPQVSTAVVEPYNSILTTHTTLEHSDCAFMVDNEAIYDICR
>	sp|Q5XIF6|TBA Tubulin alpha-4A chain OS=Rattus norvegicus GN=Tuba4a PE=2 SV=1	SKLEFSIYPAPQVSTAVVEPYNSILTTHTTLEHSDCAFMVDNEAIYDICR
>	sp|Q5XIF6|TBA Tubulin alpha-4A chain OS=Rattus norvegicus GN=Tuba4a PE=2 SV=1	SKLEFSIYPAPQVSTAVVEPYNSILTTHTTLEHSDCAFMVDNEAIYDICR
>	sp|Q5XIF6|TBA Tubulin alpha-4A chain OS=Rattus norvegicus GN=Tuba4a PE=2 SV=1	SKLEFSIYPAPQVSTAVVEPYNSILTTHTTLEHSDCAFMVDNEAIYDICR
>	sp|Q5XIF6|TBA Tubulin alpha-4A chain OS=Rattus norvegicus GN=Tuba4a PE=2 SV=1	SKLEFSIYPAPQVSTAVVEPYNSILTTHTTLEHSDCAFMVDNEAIYDICR
>	sp|Q5XIF6|TBA Tubulin alpha-4A chain OS=Rattus norvegicus GN=Tuba4a PE=2 SV=1	SKLEFSIYPAPQVSTAVVEPYNSILTTHTTLEHSDCAFMVDNEAIYDICR
>	sp|Q5XIF6|TBA Tubulin alpha-4A chain OS=Rattus norvegicus GN=Tuba4a PE=2 SV=1	SKLEFSIYPAPQVSTAVVEPYNSILTTHTTLEHSDCAFMVDNEAIYDICR
>	sp|Q5XIF6|TBA Tubulin alpha-4A chain OS=Rattus norvegicus GN=Tuba4a PE=2 SV=1	SKLEFSIYPAPQVSTAVVEPYNSILTTHTTLEHSDCAFMVDNEAIYDICR
>	sp|Q5XIF6|TBA Tubulin alpha-4A chain OS=Rattus norvegicus GN=Tuba4a PE=2 SV=1	SKLEFSIYPAPQVSTAVVEPYNSILTTHTTLEHSDCAFMVDNEAIYDICR
>	sp|Q5XIF6|TBA Tubulin alpha-4A chain OS=Rattus norvegicus GN=Tuba4a PE=2 SV=1	SKLEFSIYPAPQVSTAVVEPYNSILTTHTTLEHSDCAFMVDNEAIYDICR
>	sp|Q5XIF6|TBA Tubulin alpha-4A chain OS=Rattus norvegicus GN=Tuba4a PE=2 SV=1	SKLEFSIYPAPQVSTAVVEPYNSILTTHTTLEHSDCAFMVDNEAIYDICR
>	sp|Q5XIF6|TBA Tubulin alpha-4A chain OS=Rattus norvegicus GN=Tuba4a PE=2 SV=1	SKLEFSIYPAPQVSTAVVEPYNSILTTHTTLEHSDCAFMVDNEAIYDICR
>	sp|Q5XIF6|TBA Tubulin alpha-4A chain OS=Rattus norvegicus GN=Tuba4a PE=2 SV=1	SKLEFSIYPAPQVSTAVVEPYNSILTTHTTLEHSDCAFMVDNEAIYDICR
>	sp|Q5XIF6|TBA Tubulin alpha-4A chain OS=Rattus norvegicus GN=Tuba4a PE=2 SV=1	VGINYQPPTVVPGGDLAK
>	sp|Q5XIF6|TBA Tubulin alpha-4A chain OS=Rattus norvegicus GN=Tuba4a PE=2 SV=1	VGINYQPPTVVPGGDLAK
>	sp|Q5XIF6|TBA Tubulin alpha-4A chain OS=Rattus norvegicus GN=Tuba4a PE=2 SV=1	VGINYQPPTVVPGGDLAK
>	sp|Q5XIF6|TBA Tubulin alpha-4A chain OS=Rattus norvegicus GN=Tuba4a PE=2 SV=1	VGINYQPPTVVPGGDLAK
>	sp|Q5XIF6|TBA Tubulin alpha-4A chain OS=Rattus norvegicus GN=Tuba4a PE=2 SV=1	VGINYQPPTVVPGGDLAK
>	sp|Q5XIF6|TBA Tubulin alpha-4A chain OS=Rattus norvegicus GN=Tuba4a PE=2 SV=1	VGINYQPPTVVPGGDLAK
>	sp|Q5XIF6|TBA Tubulin alpha-4A chain OS=Rattus norvegicus GN=Tuba4a PE=2 SV=1	VGINYQPPTVVPGGDLAK
>	sp|Q5XIF6|TBA Tubulin alpha-4A chain OS=Rattus norvegicus GN=Tuba4a PE=2 SV=1	VGINYQPPTVVPGGDLAK
>	sp|Q5XIF6|TBA Tubulin alpha-4A chain OS=Rattus norvegicus GN=Tuba4a PE=2 SV=1	VGINYQPPTVVPGGDLAK
>	sp|Q5XIF6|TBA Tubulin alpha-4A chain OS=Rattus norvegicus GN=Tuba4a PE=2 SV=1	VGINYQPPTVVPGGDLAK
>	sp|Q5XIF6|TBA Tubulin alpha-4A chain OS=Rattus norvegicus GN=Tuba4a PE=2 SV=1	VGINYQPPTVVPGGDLAK
>	sp|Q5XIF6|TBA Tubulin alpha-4A chain OS=Rattus norvegicus GN=Tuba4a PE=2 SV=1	VGINYQPPTVVPGGDLAK
>	sp|Q5XIF6|TBA Tubulin alpha-4A chain OS=Rattus norvegicus GN=Tuba4a PE=2 SV=1	YMACCLLYR
>	sp|Q5XIF6|TBA Tubulin alpha-4A chain OS=Rattus norvegicus GN=Tuba4a PE=2 SV=1	IHFPLATYAPVISAEK
>	sp|Q5XIF6|TBA Tubulin alpha-4A chain OS=Rattus norvegicus GN=Tuba4a PE=2 SV=1	AYHEQLSVAEITNACFEPANQMVK
>	sp|Q5XIF6|TBA Tubulin alpha-4A chain OS=Rattus norvegicus GN=Tuba4a PE=2 SV=1	IHFPLATYAPVISAEK
>	sp|Q5XIF6|TBA Tubulin alpha-4A chain OS=Rattus norvegicus GN=Tuba4a PE=2 SV=1	QLFHPEQLITGKEDAANNYAR
>	sp|Q5XIF6|TBA Tubulin alpha-4A chain OS=Rattus norvegicus GN=Tuba4a PE=2 SV=1	LEFSIYPAPQVSTAVVEPYNSILTTHTTLEHSDCAFMVDNEAIYDICR
>	sp|Q5XIF6|TBA Tubulin alpha-4A chain OS=Rattus norvegicus GN=Tuba4a PE=2 SV=1	SKLEFSIYPAPQVSTAVVEPYNSILTTHTTLEHSDCAFMVDNEAIYDICR
>	sp|Q5XIF6|TBA Tubulin alpha-4A chain OS=Rattus norvegicus GN=Tuba4a PE=2 SV=1	QLFHPEQLITGKEDAANNYAR
>	sp|Q5XIF6|TBA Tubulin alpha-4A chain OS=Rattus norvegicus GN=Tuba4a PE=2 SV=1	LSVDYGK
>	sp|Q5XIF6|TBA Tubulin alpha-4A chain OS=Rattus norvegicus GN=Tuba4a PE=2 SV=1	ECISVHVGQAGVQMGNACWELYCLEHGIQPDGQMPSDK
>	sp|Q5XIF6|TBA Tubulin alpha-4A chain OS=Rattus norvegicus GN=Tuba4a PE=2 SV=1	SIQFVDWCPTGFK
>	sp|Q5XIF6|TBA Tubulin alpha-4A chain OS=Rattus norvegicus GN=Tuba4a PE=2 SV=1	IHFPLATYAPVISAEK
>	sp|Q5XIF6|TBA Tubulin alpha-4A chain OS=Rattus norvegicus GN=Tuba4a PE=2 SV=1	QLFHPEQLITGKEDAANNYAR
>	sp|Q5XIF6|TBA Tubulin alpha-4A chain OS=Rattus norvegicus GN=Tuba4a PE=2 SV=1	VGINYQPPTVVPGGDLAK
>	sp|Q5XIF6|TBA Tubulin alpha-4A chain OS=Rattus norvegicus GN=Tuba4a PE=2 SV=1	SIQFVDWCPTGFK
>	sp|Q5XIF6|TBA Tubulin alpha-4A chain OS=Rattus norvegicus GN=Tuba4a PE=2 SV=1	SIQFVDWCPTGFK
>	sp|Q5XIF6|TBA Tubulin alpha-4A chain OS=Rattus norvegicus GN=Tuba4a PE=2 SV=1	IHFPLATYAPVISAE
>	sp|Q5XIF6|TBA Tubulin alpha-4A chain OS=Rattus norvegicus GN=Tuba4a PE=2 SV=1	IHFPLATYAPVISAEK
>	sp|Q5XIF6|TBA Tubulin alpha-4A chain OS=Rattus norvegicus GN=Tuba4a PE=2 SV=1	QLFHPEQLITGKEDAANNYAR
>	sp|Q5XIF6|TBA Tubulin alpha-4A chain OS=Rattus norvegicus GN=Tuba4a PE=2 SV=1	SKLEFSIYPAPQVSTAVVEPYNSILTTHTTLEHSDCAFMVDNEAIYDICR
>	sp|Q5XIF6|TBA Tubulin alpha-4A chain OS=Rattus norvegicus GN=Tuba4a PE=2 SV=1	KFDLMYAKRAFVHWYVGEGMEEGEFSEAR
>	sp|Q5XIF6|TBA Tubulin alpha-4A chain OS=Rattus norvegicus GN=Tuba4a PE=2 SV=1	KFDLMYAKRAFVHWYVGEGMEEGEFSEAR
>	sp|Q5XIF6|TBA Tubulin alpha-4A chain OS=Rattus norvegicus GN=Tuba4a PE=2 SV=1	IHFPLATYAPVISAEK
>	sp|Q5XIF6|TBA Tubulin alpha-4A chain OS=Rattus norvegicus GN=Tuba4a PE=2 SV=1	AVCMLSNTTAIAEAWAR
>	sp|Q5XIF6|TBA Tubulin alpha-4A chain OS=Rattus norvegicus GN=Tuba4a PE=2 SV=1	AYHEQLSVAEITNACFEPANQMVK
>	sp|Q5XIF6|TBA Tubulin alpha-4A chain OS=Rattus norvegicus GN=Tuba4a PE=2 SV=1	VGINYQPPTVVPGGDLAK
>	sp|Q5XIF6|TBA Tubulin alpha-4A chain OS=Rattus norvegicus GN=Tuba4a PE=2 SV=1	SKLEFSIYPAPQVSTAVVEPYNSILTTHTTLEHSDCAFMVDNEAIYDICR
>	sp|Q5XIF6|TBA Tubulin alpha-4A chain OS=Rattus norvegicus GN=Tuba4a PE=2 SV=1	SKLEFSIYPAPQVSTAVVEPYNSILTTHTTLEHSDCAFMVDNEAIYDICR
>	sp|Q5XIF6|TBA Tubulin alpha-4A chain OS=Rattus norvegicus GN=Tuba4a PE=2 SV=1	VGINYQPPTVVPGGDLAK
>	sp|Q5XIF6|TBA Tubulin alpha-4A chain OS=Rattus norvegicus GN=Tuba4a PE=2 SV=1	AYHEQLSVAEITNACFEPANQMVK
>	sp|Q5XIF6|TBA Tubulin alpha-4A chain OS=Rattus norvegicus GN=Tuba4a PE=2 SV=1	VGINYQPPTVVPGGDLAK
>	sp|Q5XIF6|TBA Tubulin alpha-4A chain OS=Rattus norvegicus GN=Tuba4a PE=2 SV=1	QLFHPEQLITGK
>	sp|Q5XIF6|TBA Tubulin alpha-4A chain OS=Rattus norvegicus GN=Tuba4a PE=2 SV=1	YMACCLLYR
>	sp|Q5XIF6|TBA Tubulin alpha-4A chain OS=Rattus norvegicus GN=Tuba4a PE=2 SV=1	AYHEQLSVAEITNACFEPANQMVK
>	sp|Q5XIF6|TBA Tubulin alpha-4A chain OS=Rattus norvegicus GN=Tuba4a PE=2 SV=1	QLFHPEQLITGKEDAANNYAR
>	sp|Q5XIF6|TBA Tubulin alpha-4A chain OS=Rattus norvegicus GN=Tuba4a PE=2 SV=1	QLFHPEQLITGKEDAANNYAR
>	sp|Q5XIF6|TBA Tubulin alpha-4A chain OS=Rattus norvegicus GN=Tuba4a PE=2 SV=1	QLFHPEQLITGKEDAANNYAR
>	sp|Q5XIF6|TBA Tubulin alpha-4A chain OS=Rattus norvegicus GN=Tuba4a PE=2 SV=1	AYHEQLSVAEITNACFEPANQMVK
>	sp|Q5XIF6|TBA Tubulin alpha-4A chain OS=Rattus norvegicus GN=Tuba4a PE=2 SV=1	LEFSIYPAPQVSTAVVEPYNSILTTHTTLEHSDCAFMVDNEAIYDICR
>	sp|Q5XIF6|TBA Tubulin alpha-4A chain OS=Rattus norvegicus GN=Tuba4a PE=2 SV=1	FDGALNVDLTEFQTNLVPYPR
>	sp|Q5XIF6|TBA Tubulin alpha-4A chain OS=Rattus norvegicus GN=Tuba4a PE=2 SV=1	SIQFVDWCPTGFK
>	sp|Q5XIF6|TBA Tubulin alpha-4A chain OS=Rattus norvegicus GN=Tuba4a PE=2 SV=1	QLFHPEQLITGKEDAANNYAR
>	sp|Q5XIF6|TBA Tubulin alpha-4A chain OS=Rattus norvegicus GN=Tuba4a PE=2 SV=1	QLFHPEQLITGKEDAANNYAR
>	sp|Q5XIF6|TBA Tubulin alpha-4A chain OS=Rattus norvegicus GN=Tuba4a PE=2 SV=1	YMACCLLYR
>	sp|Q5XIF6|TBA Tubulin alpha-4A chain OS=Rattus norvegicus GN=Tuba4a PE=2 SV=1	PPTVVPGGDLAK
>	sp|Q5XIF6|TBA Tubulin alpha-4A chain OS=Rattus norvegicus GN=Tuba4a PE=2 SV=1	SIQFVDWCPTGFK
>	sp|Q5XIF6|TBA Tubulin alpha-4A chain OS=Rattus norvegicus GN=Tuba4a PE=2 SV=1	VGINYQPPTVVPGGDLAK

> sp|Q5XIF6|TBA Tubulin alpha-4A chain OS=Rattus norvegicus GN=Tuba4a PE=2 SV=1 AVFVDLE
> sp|Q5XIF6|TBA Tubulin alpha-4A chain OS=Rattus norvegicus GN=Tuba4a PE=2 SV=1 SIQFVDWCPTGFK
> sp|Q5XIF6|TBA Tubulin alpha-4A chain OS=Rattus norvegicus GN=Tuba4a PE=2 SV=1 SIQFVDWCPTGFK
> sp|Q5XIF6|TBA Tubulin alpha-4A chain OS=Rattus norvegicus GN=Tuba4a PE=2 SV=1 AVCMLSNTTAIAEAWAR
> sp|Q5XIF6|TBA Tubulin alpha-4A chain OS=Rattus norvegicus GN=Tuba4a PE=2 SV=1 YMACCLLYR
> sp|Q5XIF6|TBA Tubulin alpha-4A chain OS=Rattus norvegicus GN=Tuba4a PE=2 SV=1 ECISVHVGQAGVQMGNACWELYCLEHGIQPDGQMPSDK
> sp|Q5XIF6|TBA Tubulin alpha-4A chain OS=Rattus norvegicus GN=Tuba4a PE=2 SV=1 AYHEQLSVAEITNACFEPANQMVK
> sp|Q5XIF6|TBA Tubulin alpha-4A chain OS=Rattus norvegicus GN=Tuba4a PE=2 SV=1 AFVHWYVGEGMEEGEFSEAREDMAALEK
> sp|Q5XIF6|TBA Tubulin alpha-4A chain OS=Rattus norvegicus GN=Tuba4a PE=2 SV=1 YMACCLLYR
> sp|Q5XIF6|TBA Tubulin alpha-4A chain OS=Rattus norvegicus GN=Tuba4a PE=2 SV=1 FDGALNVDLTEFQTNLVPYPR
> sp|Q5XIF6|TBA Tubulin alpha-4A chain OS=Rattus norvegicus GN=Tuba4a PE=2 SV=1 AFVHWYVGEGMEEGEFSEAR
> sp|Q5XIF6|TBA Tubulin alpha-4A chain OS=Rattus norvegicus GN=Tuba4a PE=2 SV=1 KLSDQCTGLQGFLVFHSFGGGTGSGFTSLLMER
> sp|Q5XIF6|TBA Tubulin alpha-4A chain OS=Rattus norvegicus GN=Tuba4a PE=2 SV=1 AYHEQLSVAEITNACFEPANQMVK
> sp|Q5XIF6|TBA Tubulin alpha-4A chain OS=Rattus norvegicus GN=Tuba4a PE=2 SV=1 KLSDQCTGLQGFLVFHSFGGGTGSGFTSLLMER
> sp|Q5XIF6|TBA Tubulin alpha-4A chain OS=Rattus norvegicus GN=Tuba4a PE=2 SV=1 AYHEQLSVAEITNACFEPANQMVK
> sp|Q5XIF6|TBA Tubulin alpha-4A chain OS=Rattus norvegicus GN=Tuba4a PE=2 SV=1 IHFPLATYAPVISAEK
> sp|Q5XIF6|TBA Tubulin alpha-4A chain OS=Rattus norvegicus GN=Tuba4a PE=2 SV=1 GHYTIGKEIIDPVLDR
> sp|Q5XIF6|TBA Tubulin alpha-4A chain OS=Rattus norvegicus GN=Tuba4a PE=2 SV=1 GDVVPK
> sp|Q5XIF6|TBA Tubulin alpha-4A chain OS=Rattus norvegicus GN=Tuba4a PE=2 SV=1 AYHEQLSVAEITNACFEPANQMVK
> sp|Q5XIF6|TBA Tubulin alpha-4A chain OS=Rattus norvegicus GN=Tuba4a PE=2 SV=1 DVNAAIAAIK
> sp|Q5XIF6|TBA Tubulin alpha-4A chain OS=Rattus norvegicus GN=Tuba4a PE=2 SV=1 YMACCLLYR
> sp|Q5XIF6|TBA Tubulin alpha-4A chain OS=Rattus norvegicus GN=Tuba4a PE=2 SV=1 LEFSIYPAPQVSTAVVEPYNSILTTHTTLEHSDCAFMVDNEAIYDICR
> sp|Q5XIF6|TBA Tubulin alpha-4A chain OS=Rattus norvegicus GN=Tuba4a PE=2 SV=1 LEFSIYPAPQVSTAVVEPYNSILTTHTTLEHSDCAFMVDNEAIYDICR
> sp|Q5XIF6|TBA Tubulin alpha-4A chain OS=Rattus norvegicus GN=Tuba4a PE=2 SV=1 FDGALNVDLTEFQTNLVPYPR
> sp|Q5XIF6|TBA Tubulin alpha-4A chain OS=Rattus norvegicus GN=Tuba4a PE=2 SV=1 ECISVHVGQAGVQMGNACWELYCLEHGIQPDGQMPSDKTIGGGDDSFTTFFCETGAGK
> sp|Q5XIF6|TBA Tubulin alpha-4A chain OS=Rattus norvegicus GN=Tuba4a PE=2 SV=1 LDHKFDLMYAK
> sp|Q5XIF6|TBA Tubulin alpha-4A chain OS=Rattus norvegicus GN=Tuba4a PE=2 SV=1 KLSDQCTGLQGFLVFHSFGGGTGSGFTSLLMER
> sp|Q5XIF6|TBA Tubulin alpha-4A chain OS=Rattus norvegicus GN=Tuba4a PE=2 SV=1 QLFHPEQLITGKEDAANNYAR
> sp|Q5XIF6|TBA Tubulin alpha-4A chain OS=Rattus norvegicus GN=Tuba4a PE=2 SV=1 FDGALNVDLTEFQTNLVPYPR
> sp|Q5XIF6|TBA Tubulin alpha-4A chain OS=Rattus norvegicus GN=Tuba4a PE=2 SV=1 SKLEFSIYPAPQVSTAVVEPYNSILTTHTTLEHSDCAFMVDNEAIYDICR
> sp|Q5XIF6|TBA Tubulin alpha-4A chain OS=Rattus norvegicus GN=Tuba4a PE=2 SV=1 TIGGGDDSFTTFFCETGAGK
> sp|Q5XIF6|TBA Tubulin alpha-4A chain OS=Rattus norvegicus GN=Tuba4a PE=2 SV=1 PGGDLAK
> sp|Q5XIF6|TBA Tubulin alpha-4A chain OS=Rattus norvegicus GN=Tuba4a PE=2 SV=1 SKLEFSIYPAPQVSTAVVEPYNSILTTHTTLEHSDCAFMVDNEAIYDICR
> sp|Q5XIF6|TBA Tubulin alpha-4A chain OS=Rattus norvegicus GN=Tuba4a PE=2 SV=1 TVIDEIRNGPYRQLFHPEQLITGKEDAANNYAR
> sp|Q5XIF6|TBA Tubulin alpha-4A chain OS=Rattus norvegicus GN=Tuba4a PE=2 SV=1 AYHEQLSVAEITNACFEPANQMVK
> sp|Q5XIF6|TBA Tubulin alpha-4A chain OS=Rattus norvegicus GN=Tuba4a PE=2 SV=1 QLFHPEQLITGKEDAANNYAR
> sp|Q5XIF6|TBA Tubulin alpha-4A chain OS=Rattus norvegicus GN=Tuba4a PE=2 SV=1 QLFHPEQLITGK
> sp|Q5XIF6|TBA Tubulin alpha-4A chain OS=Rattus norvegicus GN=Tuba4a PE=2 SV=1 SIQFVDWCPTGFK
> sp|Q5XIF6|TBA Tubulin alpha-4A chain OS=Rattus norvegicus GN=Tuba4a PE=2 SV=1 AYHEQLSVAEITN
> sp|Q5XIF6|TBA Tubulin alpha-4A chain OS=Rattus norvegicus GN=Tuba4a PE=2 SV=1 TVIDEIRNGPYRQLFHPEQLITGKEDAANNYAR
> sp|Q5XIF6|TBA Tubulin alpha-4A chain OS=Rattus norvegicus GN=Tuba4a PE=2 SV=1 AYHEQLSVAEITNACFEPANQMVK
> sp|Q5XIF6|TBA Tubulin alpha-4A chain OS=Rattus norvegicus GN=Tuba4a PE=2 SV=1 SIQFVDWCPTGFK
> sp|Q5XIF6|TBA Tubulin alpha-4A chain OS=Rattus norvegicus GN=Tuba4a PE=2 SV=1 IHFPLATYAPVIS
> sp|Q5XIF6|TBA Tubulin alpha-4A chain OS=Rattus norvegicus GN=Tuba4a PE=2 SV=1 AYHEQLSVAEITNACFEPANQMVK
> sp|Q5XIF6|TBA Tubulin alpha-4A chain OS=Rattus norvegicus GN=Tuba4a PE=2 SV=1 VGINYQPPTVVPGGDLAK
> sp|Q5XIF6|TBA Tubulin alpha-4A chain OS=Rattus norvegicus GN=Tuba4a PE=2 SV=1 AFVHWYVGEGMEEGEFSEAR
> sp|Q5XIF6|TBA Tubulin alpha-4A chain OS=Rattus norvegicus GN=Tuba4a PE=2 SV=1 VGINYQPPTVVPGGDLAK
> sp|Q5XIF6|TBA Tubulin alpha-4A chain OS=Rattus norvegicus GN=Tuba4a PE=2 SV=1 SKLEFSIYPAPQVSTAVVEPYNSILTTHTTLEHSDCAFMVDNEAIYDICR
> sp|Q5XIF6|TBA Tubulin alpha-4A chain OS=Rattus norvegicus GN=Tuba4a PE=2 SV=1 MRECISVHVGQAGVQMGNACWELYCLEHGIQPDGQMPSDKTIGGGDDSFTTFFCETGAGK
> sp|Q5XIF6|TBA Tubulin alpha-4A chain OS=Rattus norvegicus GN=Tuba4a PE=2 SV=1 SIQFVDWCPTGFK
> sp|Q5XIF6|TBA Tubulin alpha-4A chain OS=Rattus norvegicus GN=Tuba4a PE=2 SV=1 YMACCLLYR
> sp|Q5XIF6|TBA Tubulin alpha-4A chain OS=Rattus norvegicus GN=Tuba4a PE=2 SV=1 DVNAAIAAIK
> sp|Q5XIF6|TBA Tubulin alpha-4A chain OS=Rattus norvegicus GN=Tuba4a PE=2 SV=1 AYHEQLSVAEITNACFEPANQMVK
> sp|Q5XIF6|TBA Tubulin alpha-4A chain OS=Rattus norvegicus GN=Tuba4a PE=2 SV=1 LSVDYGK
> sp|Q5XIF6|TBA Tubulin alpha-4A chain OS=Rattus norvegicus GN=Tuba4a PE=2 SV=1 SKLEFSIYPAPQVSTAVVEPYNSILTTHTTLEHSDCAFMVDNEAIYDICR
> sp|Q5XIF6|TBA Tubulin alpha-4A chain OS=Rattus norvegicus GN=Tuba4a PE=2 SV=1 AYHEQLSVAEITNACFEPANQMVK
> sp|Q5XIF6|TBA Tubulin alpha-4A chain OS=Rattus norvegicus GN=Tuba4a PE=2 SV=1 QLFHPEQLITGKEDAANNYAR
> sp|Q5XIF6|TBA Tubulin alpha-4A chain OS=Rattus norvegicus GN=Tuba4a PE=2 SV=1 SKLEFSIYPAPQVSTAVVEPYNSILTTHTTLEHSDCAFMVDNEAIYDICR
> sp|Q5XIF6|TBA Tubulin alpha-4A chain OS=Rattus norvegicus GN=Tuba4a PE=2 SV=1 SKLEFSIYPAPQVSTAVVEPYNSILTTHTTLEHSDCAFMVDNEAIYDICR
> sp|Q5XIF6|TBA Tubulin alpha-4A chain OS=Rattus norvegicus GN=Tuba4a PE=2 SV=1 SIQFVDWCPTGFK
> sp|Q5XIF6|TBA Tubulin alpha-4A chain OS=Rattus norvegicus GN=Tuba4a PE=2 SV=1 SIQFVDWCPTGFK
> sp|Q5XIF6|TBA Tubulin alpha-4A chain OS=Rattus norvegicus GN=Tuba4a PE=2 SV=1 AYHEQLSVAEITNACFEPANQMVK
> sp|Q5XIF6|TBA Tubulin alpha-4A chain OS=Rattus norvegicus GN=Tuba4a PE=2 SV=1 SKLEFSIYPAPQVSTAVVEPYNSILTTHTTLEHSDCAFMVDNEAIYDICR
> sp|Q5XIF6|TBA Tubulin alpha-4A chain OS=Rattus norvegicus GN=Tuba4a PE=2 SV=1 IHFPLATYAPVISA
> sp|Q5XIF6|TBA Tubulin alpha-4A chain OS=Rattus norvegicus GN=Tuba4a PE=2 SV=1 QLFHPEQLITGKEDAANNYAR
> sp|Q5XIF6|TBA Tubulin alpha-4A chain OS=Rattus norvegicus GN=Tuba4a PE=2 SV=1 AFVHWYVGEGMEEGEFSEAR
> sp|Q5XIF6|TBA Tubulin alpha-4A chain OS=Rattus norvegicus GN=Tuba4a PE=2 SV=1 LSVDYGKK
> sp|Q5XIF6|TBA Tubulin alpha-4A chain OS=Rattus norvegicus GN=Tuba4a PE=2 SV=1 VGINYQPPTVVPGGDLAK
> sp|Q5XIF6|TBA Tubulin alpha-4A chain OS=Rattus norvegicus GN=Tuba4a PE=2 SV=1 LDHKFDLMYAK
> sp|Q5XIF6|TBA Tubulin alpha-4A chain OS=Rattus norvegicus GN=Tuba4a PE=2 SV=1 IHFPLATYAPVISA
> sp|Q5XIF6|TBA Tubulin alpha-4A chain OS=Rattus norvegicus GN=Tuba4a PE=2 SV=1 ECISVHVGQAGVQMGNACWELYCLEHGIQPDGQMPSDK
> sp|Q5XIF6|TBA Tubulin alpha-4A chain OS=Rattus norvegicus GN=Tuba4a PE=2 SV=1 QLFHPEQLITGKEDAANNYAR
> sp|Q5XIF6|TBA Tubulin alpha-4A chain OS=Rattus norvegicus GN=Tuba4a PE=2 SV=1 AYHEQLSVAEITNACFEPANQMVK
> sp|Q5XIF6|TBA Tubulin alpha-4A chain OS=Rattus norvegicus GN=Tuba4a PE=2 SV=1 IHFPLATYAP

>	sp|Q5XIF6|TBA Tubulin alpha-4A chain OS=Rattus norvegicus GN=Tuba4a PE=2 SV=1	VGINYQPPTVVPGGDLAK
>	sp|Q5XIF6|TBA Tubulin alpha-4A chain OS=Rattus norvegicus GN=Tuba4a PE=2 SV=1	IHFPLATYAPVISAEK
>	sp|Q5XIF6|TBA Tubulin alpha-4A chain OS=Rattus norvegicus GN=Tuba4a PE=2 SV=1	AFVHWYVGEGMEEGEFSEAREDMAALEK
>	sp|Q5XIF6|TBA Tubulin alpha-4A chain OS=Rattus norvegicus GN=Tuba4a PE=2 SV=1	QLFHPEQLITGKEDAANNYAR
>	sp|Q5XIF6|TBA Tubulin alpha-4A chain OS=Rattus norvegicus GN=Tuba4a PE=2 SV=1	QLFHPEQLITGKEDAANNYAR
>	sp|Q5XIF6|TBA Tubulin alpha-4A chain OS=Rattus norvegicus GN=Tuba4a PE=2 SV=1	AYHEQLSVAEITNACFEPANQMVK
>	sp|Q5XIF6|TBA Tubulin alpha-4A chain OS=Rattus norvegicus GN=Tuba4a PE=2 SV=1	LEFSIYPAPQVSTAVVEPYNSILTTHTTLEHSDCAFMVDNEAIYDICR
>	sp|Q5XIF6|TBA Tubulin alpha-4A chain OS=Rattus norvegicus GN=Tuba4a PE=2 SV=1	SKLEFSIYPAPQVSTAVVEPYNSILTTHTTLEHSDCAFMVDNEAIYDICR
>	sp|Q5XIF6|TBA Tubulin alpha-4A chain OS=Rattus norvegicus GN=Tuba4a PE=2 SV=1	IHFPLATYAP
>	sp|Q5XIF6|TBA Tubulin alpha-4A chain OS=Rattus norvegicus GN=Tuba4a PE=2 SV=1	ECISVHVGQAGVQMGNACWELYCLEHGIQPDGQMPSDK
>	sp|Q5XIF6|TBA Tubulin alpha-4A chain OS=Rattus norvegicus GN=Tuba4a PE=2 SV=1	AVFVDLEPTVIDEIR
>	sp|Q5XIF6|TBA Tubulin alpha-4A chain OS=Rattus norvegicus GN=Tuba4a PE=2 SV=1	AVCMLSNTTAIAEAWAR
>	sp|Q5XIF6|TBA Tubulin alpha-4A chain OS=Rattus norvegicus GN=Tuba4a PE=2 SV=1	SKLEFSIYPAPQVSTAVVEPYNSILTTHTTLEHSDCAFMVDNEAIYDICR
>	sp|Q5XIF6|TBA Tubulin alpha-4A chain OS=Rattus norvegicus GN=Tuba4a PE=2 SV=1	QLFHPEQLITGKEDAANNYAR
>	sp|Q5XIF6|TBA Tubulin alpha-4A chain OS=Rattus norvegicus GN=Tuba4a PE=2 SV=1	LEFSIYPAPQVSTAVVEPYNSILTTHTTLEHSDCAFMVDNEAIYDICR
>	sp|Q5XIF6|TBA Tubulin alpha-4A chain OS=Rattus norvegicus GN=Tuba4a PE=2 SV=1	NLDIERPTYTNLNRLISQIVSSITASLR
>	sp|Q5XIF6|TBA Tubulin alpha-4A chain OS=Rattus norvegicus GN=Tuba4a PE=2 SV=1	EIIDPVLDR
>	sp|Q5XIF6|TBA Tubulin alpha-4A chain OS=Rattus norvegicus GN=Tuba4a PE=2 SV=1	LDHKFDLMYAK
>	sp|Q5XIF6|TBA Tubulin alpha-4A chain OS=Rattus norvegicus GN=Tuba4a PE=2 SV=1	AVCMLSNTTAIAEAWAR
>	sp|Q5XIF6|TBA Tubulin alpha-4A chain OS=Rattus norvegicus GN=Tuba4a PE=2 SV=1	LEFSIYPAPQVSTAVVEPYNSILTTHTTLEHSDCAFMVDNEAIYDICR
>	sp|P11980|KPY Pyruvate kinase isozymes M1/M2 OS=Rattus norvegicus GN=Pkm2 PE=1 SV=3	AADVHEVRK
>	sp|P11980|KPY Pyruvate kinase isozymes M1/M2 OS=Rattus norvegicus GN=Pkm2 PE=1 SV=3	AEGSDVANAVLDGADCIMLSGETAK
>	sp|P11980|KPY Pyruvate kinase isozymes M1/M2 OS=Rattus norvegicus GN=Pkm2 PE=1 SV=3	AEGSDVANAVLDGADCIMLSGETAKGDYPLEAVR
>	sp|P11980|KPY Pyruvate kinase isozymes M1/M2 OS=Rattus norvegicus GN=Pkm2 PE=1 SV=3	AGKPVICATQMLESMIK
>	sp|P11980|KPY Pyruvate kinase isozymes M1/M2 OS=Rattus norvegicus GN=Pkm2 PE=1 SV=3	CDENILWLDYK
>	sp|P11980|KPY Pyruvate kinase isozymes M1/M2 OS=Rattus norvegicus GN=Pkm2 PE=1 SV=3	CLAAALIVLTESGR
>	sp|P11980|KPY Pyruvate kinase isozymes M1/M2 OS=Rattus norvegicus GN=Pkm2 PE=1 SV=3	DAVLDAWAEDVDLR
>	sp|P11980|KPY Pyruvate kinase isozymes M1/M2 OS=Rattus norvegicus GN=Pkm2 PE=1 SV=3	GADYLVTEVENGGSLGSK
>	sp|P11980|KPY Pyruvate kinase isozymes M1/M2 OS=Rattus norvegicus GN=Pkm2 PE=1 SV=3	GDLGIEIPAEK
>	sp|P11980|KPY Pyruvate kinase isozymes M1/M2 OS=Rattus norvegicus GN=Pkm2 PE=1 SV=3	GFFKKGDVVIVLTGWRPGSGFTNTMR
>	sp|P11980|KPY Pyruvate kinase isozymes M1/M2 OS=Rattus norvegicus GN=Pkm2 PE=1 SV=3	ITLDNAYMEK
>	sp|P11980|KPY Pyruvate kinase isozymes M1/M2 OS=Rattus norvegicus GN=Pkm2 PE=1 SV=3	KAADVHEVR
>	sp|P11980|KPY Pyruvate kinase isozymes M1/M2 OS=Rattus norvegicus GN=Pkm2 PE=1 SV=3	KGDVVIVLTGWRPGSGFTNTMR
>	sp|P11980|KPY Pyruvate kinase isozymes M1/M2 OS=Rattus norvegicus GN=Pkm2 PE=1 SV=3	KGVNLPGAAVDLPAVSEK
>	sp|P11980|KPY Pyruvate kinase isozymes M1/M2 OS=Rattus norvegicus GN=Pkm2 PE=1 SV=3	NTGIICTIGPASR
>	sp|P11980|KPY Pyruvate kinase isozymes M1/M2 OS=Rattus norvegicus GN=Pkm2 PE=1 SV=3	PKPDSEAGTAFIQTQQLHAAMADTFLEHMCR
>	sp|P11980|KPY Pyruvate kinase isozymes M1/M2 OS=Rattus norvegicus GN=Pkm2 PE=1 SV=3	RFDEILEASDGIMVAR
>	sp|P11980|KPY Pyruvate kinase isozymes M1/M2 OS=Rattus norvegicus GN=Pkm2 PE=1 SV=3	VNLAMNVGK
>	sp|P11980|KPY Pyruvate kinase isozymes M1/M2 OS=Rattus norvegicus GN=Pkm2 PE=1 SV=3	GVNLPGAAVDLPAVSEKDIQDLK
>	sp|P11980|KPY Pyruvate kinase isozymes M1/M2 OS=Rattus norvegicus GN=Pkm2 PE=1 SV=3	AATESFASDPILYRPVAVALDTK
>	sp|P11980|KPY Pyruvate kinase isozymes M1/M2 OS=Rattus norvegicus GN=Pkm2 PE=1 SV=3	EAEAAVFHR
>	sp|P11980|KPY Pyruvate kinase isozymes M1/M2 OS=Rattus norvegicus GN=Pkm2 PE=1 SV=3	KGVNLPGAAVDLPAVSEKDIQDLK
>	sp|P11980|KPY Pyruvate kinase isozymes M1/M2 OS=Rattus norvegicus GN=Pkm2 PE=1 SV=3	LDIDSAPITAR
>	sp|P11980|KPY Pyruvate kinase isozymes M1/M2 OS=Rattus norvegicus GN=Pkm2 PE=1 SV=3	IYVDDGLISLQVK
>	sp|P11980|KPY Pyruvate kinase isozymes M1/M2 OS=Rattus norvegicus GN=Pkm2 PE=1 SV=3	FGVEQDVDMVFASFIR
>	sp|P11980|KPY Pyruvate kinase isozymes M1/M2 OS=Rattus norvegicus GN=Pkm2 PE=1 SV=3	AEGSDVANAVLDGADCIMLSGETAK
>	sp|P11980|KPY Pyruvate kinase isozymes M1/M2 OS=Rattus norvegicus GN=Pkm2 PE=1 SV=3	AEGSDVANAVLDGADCIMLSGETAK
>	sp|P11980|KPY Pyruvate kinase isozymes M1/M2 OS=Rattus norvegicus GN=Pkm2 PE=1 SV=3	AEGSDVANAVLDGADCIMLSGETAK
>	sp|P11980|KPY Pyruvate kinase isozymes M1/M2 OS=Rattus norvegicus GN=Pkm2 PE=1 SV=3	AEGSDVANAVLDGADCIMLSGETAK
>	sp|P11980|KPY Pyruvate kinase isozymes M1/M2 OS=Rattus norvegicus GN=Pkm2 PE=1 SV=3	AEGSDVANAVLDGADCIMLSGETAK
>	sp|P11980|KPY Pyruvate kinase isozymes M1/M2 OS=Rattus norvegicus GN=Pkm2 PE=1 SV=3	AEGSDVANAVLDGADCIMLSGETAKGDYPLEAVR
>	sp|P11980|KPY Pyruvate kinase isozymes M1/M2 OS=Rattus norvegicus GN=Pkm2 PE=1 SV=3	AEGSDVANAVLDGADCIMLSGETAKGDYPLEAVR
>	sp|P11980|KPY Pyruvate kinase isozymes M1/M2 OS=Rattus norvegicus GN=Pkm2 PE=1 SV=3	AEGSDVANAVLDGADCIMLSGETAKGDYPLEAVR
>	sp|P11980|KPY Pyruvate kinase isozymes M1/M2 OS=Rattus norvegicus GN=Pkm2 PE=1 SV=3	AGKPVICATQMLESMIK
>	sp|P11980|KPY Pyruvate kinase isozymes M1/M2 OS=Rattus norvegicus GN=Pkm2 PE=1 SV=3	AGKPVICATQMLESMIK
>	sp|P11980|KPY Pyruvate kinase isozymes M1/M2 OS=Rattus norvegicus GN=Pkm2 PE=1 SV=3	AGKPVICATQMLESMIK
>	sp|P11980|KPY Pyruvate kinase isozymes M1/M2 OS=Rattus norvegicus GN=Pkm2 PE=1 SV=3	AGKPVICATQMLESMIK
>	sp|P11980|KPY Pyruvate kinase isozymes M1/M2 OS=Rattus norvegicus GN=Pkm2 PE=1 SV=3	AGKPVICATQMLESMIK
>	sp|P11980|KPY Pyruvate kinase isozymes M1/M2 OS=Rattus norvegicus GN=Pkm2 PE=1 SV=3	AGKPVICATQMLESMIK
>	sp|P11980|KPY Pyruvate kinase isozymes M1/M2 OS=Rattus norvegicus GN=Pkm2 PE=1 SV=3	AGKPVICATQMLESMIK
>	sp|P11980|KPY Pyruvate kinase isozymes M1/M2 OS=Rattus norvegicus GN=Pkm2 PE=1 SV=3	AGKPVICATQMLESMIK
>	sp|P11980|KPY Pyruvate kinase isozymes M1/M2 OS=Rattus norvegicus GN=Pkm2 PE=1 SV=3	AGKPVICATQMLESMIK
>	sp|P11980|KPY Pyruvate kinase isozymes M1/M2 OS=Rattus norvegicus GN=Pkm2 PE=1 SV=3	AGKPVICATQMLESMIK
>	sp|P11980|KPY Pyruvate kinase isozymes M1/M2 OS=Rattus norvegicus GN=Pkm2 PE=1 SV=3	AGKPVICATQMLESMIK
>	sp|P11980|KPY Pyruvate kinase isozymes M1/M2 OS=Rattus norvegicus GN=Pkm2 PE=1 SV=3	AGKPVICATQMLESMIK
>	sp|P11980|KPY Pyruvate kinase isozymes M1/M2 OS=Rattus norvegicus GN=Pkm2 PE=1 SV=3	CLAAALIVLTESGR
>	sp|P11980|KPY Pyruvate kinase isozymes M1/M2 OS=Rattus norvegicus GN=Pkm2 PE=1 SV=3	DAVLDAWAEDVDLR
>	sp|P11980|KPY Pyruvate kinase isozymes M1/M2 OS=Rattus norvegicus GN=Pkm2 PE=1 SV=3	DAVLDAWAEDVDLR
>	sp|P11980|KPY Pyruvate kinase isozymes M1/M2 OS=Rattus norvegicus GN=Pkm2 PE=1 SV=3	EAEAAVFHR
>	sp|P11980|KPY Pyruvate kinase isozymes M1/M2 OS=Rattus norvegicus GN=Pkm2 PE=1 SV=3	EAEAAVFHR
>	sp|P11980|KPY Pyruvate kinase isozymes M1/M2 OS=Rattus norvegicus GN=Pkm2 PE=1 SV=3	EAEAAVFHR
>	sp|P11980|KPY Pyruvate kinase isozymes M1/M2 OS=Rattus norvegicus GN=Pkm2 PE=1 SV=3	EAEAAVFHR
>	sp|P11980|KPY Pyruvate kinase isozymes M1/M2 OS=Rattus norvegicus GN=Pkm2 PE=1 SV=3	EAEAAVFHR
>	sp|P11980|KPY Pyruvate kinase isozymes M1/M2 OS=Rattus norvegicus GN=Pkm2 PE=1 SV=3	FGVEQDVDMVFASFIR

>	sp|P11980|KPY Pyruvate kinase isozymes M1/M2 OS=Rattus norvegicus GN=Pkm2 PE=1 SV=3	GADYLVTEVENGGSLGSK
>	sp|P11980|KPY Pyruvate kinase isozymes M1/M2 OS=Rattus norvegicus GN=Pkm2 PE=1 SV=3	GDLGIEIPAEK
>	sp|P11980|KPY Pyruvate kinase isozymes M1/M2 OS=Rattus norvegicus GN=Pkm2 PE=1 SV=3	GDLGIEIPAEK
>	sp|P11980|KPY Pyruvate kinase isozymes M1/M2 OS=Rattus norvegicus GN=Pkm2 PE=1 SV=3	GFFKKGDVVIVLTGWRPGSGFTNTMR
>	sp|P11980|KPY Pyruvate kinase isozymes M1/M2 OS=Rattus norvegicus GN=Pkm2 PE=1 SV=3	GFFKKGDVVIVLTGWRPGSGFTNTMR
>	sp|P11980|KPY Pyruvate kinase isozymes M1/M2 OS=Rattus norvegicus GN=Pkm2 PE=1 SV=3	GVNLPGAAVDLPAVSEKDIQDLK
>	sp|P11980|KPY Pyruvate kinase isozymes M1/M2 OS=Rattus norvegicus GN=Pkm2 PE=1 SV=3	KGDVVIVLTGWRPGSGFTNTMR
>	sp|P11980|KPY Pyruvate kinase isozymes M1/M2 OS=Rattus norvegicus GN=Pkm2 PE=1 SV=3	KGDVVIVLTGWRPGSGFTNTMR
>	sp|P11980|KPY Pyruvate kinase isozymes M1/M2 OS=Rattus norvegicus GN=Pkm2 PE=1 SV=3	KGDVVIVLTGWRPGSGFTNTMR
>	sp|P11980|KPY Pyruvate kinase isozymes M1/M2 OS=Rattus norvegicus GN=Pkm2 PE=1 SV=3	KGDVVIVLTGWRPGSGFTNTMR
>	sp|P11980|KPY Pyruvate kinase isozymes M1/M2 OS=Rattus norvegicus GN=Pkm2 PE=1 SV=3	KGVNLPGAAVDLPAVSEK
>	sp|P11980|KPY Pyruvate kinase isozymes M1/M2 OS=Rattus norvegicus GN=Pkm2 PE=1 SV=3	KGVNLPGAAVDLPAVSEK
>	sp|P11980|KPY Pyruvate kinase isozymes M1/M2 OS=Rattus norvegicus GN=Pkm2 PE=1 SV=3	KGVNLPGAAVDLPAVSEK
>	sp|P11980|KPY Pyruvate kinase isozymes M1/M2 OS=Rattus norvegicus GN=Pkm2 PE=1 SV=3	KGVNLPGAAVDLPAVSEKDIQDLK
>	sp|P11980|KPY Pyruvate kinase isozymes M1/M2 OS=Rattus norvegicus GN=Pkm2 PE=1 SV=3	KGVNLPGAAVDLPAVSEKDIQDLK
>	sp|P11980|KPY Pyruvate kinase isozymes M1/M2 OS=Rattus norvegicus GN=Pkm2 PE=1 SV=3	NTGIICTIGPASR
>	sp|P11980|KPY Pyruvate kinase isozymes M1/M2 OS=Rattus norvegicus GN=Pkm2 PE=1 SV=3	NTGIICTIGPASR
>	sp|P11980|KPY Pyruvate kinase isozymes M1/M2 OS=Rattus norvegicus GN=Pkm2 PE=1 SV=3	NTGIICTIGPASR
>	sp|P11980|KPY Pyruvate kinase isozymes M1/M2 OS=Rattus norvegicus GN=Pkm2 PE=1 SV=3	PKPDSEAGTAFIQTQQLHAAMADTFLEHMCR
>	sp|P11980|KPY Pyruvate kinase isozymes M1/M2 OS=Rattus norvegicus GN=Pkm2 PE=1 SV=3	PKPDSEAGTAFIQTQQLHAAMADTFLEHMCR
>	sp|P11980|KPY Pyruvate kinase isozymes M1/M2 OS=Rattus norvegicus GN=Pkm2 PE=1 SV=3	PKPDSEAGTAFIQTQQLHAAMADTFLEHMCR
>	sp|P11980|KPY Pyruvate kinase isozymes M1/M2 OS=Rattus norvegicus GN=Pkm2 PE=1 SV=3	PKPDSEAGTAFIQTQQLHAAMADTFLEHMCR
>	sp|P11980|KPY Pyruvate kinase isozymes M1/M2 OS=Rattus norvegicus GN=Pkm2 PE=1 SV=3	PKPDSEAGTAFIQTQQLHAAMADTFLEHMCR
>	sp|P11980|KPY Pyruvate kinase isozymes M1/M2 OS=Rattus norvegicus GN=Pkm2 PE=1 SV=3	RFDEILEASDGIMVAR
>	sp|P11980|KPY Pyruvate kinase isozymes M1/M2 OS=Rattus norvegicus GN=Pkm2 PE=1 SV=3	RFDEILEASDGIMVAR
>	sp|P11980|KPY Pyruvate kinase isozymes M1/M2 OS=Rattus norvegicus GN=Pkm2 PE=1 SV=3	RFDEILEASDGIMVAR
>	sp|P11980|KPY Pyruvate kinase isozymes M1/M2 OS=Rattus norvegicus GN=Pkm2 PE=1 SV=3	RFDEILEASDGIMVAR
>	sp|P11980|KPY Pyruvate kinase isozymes M1/M2 OS=Rattus norvegicus GN=Pkm2 PE=1 SV=3	RFDEILEASDGIMVAR
>	sp|P11980|KPY Pyruvate kinase isozymes M1/M2 OS=Rattus norvegicus GN=Pkm2 PE=1 SV=3	RFDEILEASDGIMVAR
>	sp|P11980|KPY Pyruvate kinase isozymes M1/M2 OS=Rattus norvegicus GN=Pkm2 PE=1 SV=3	RFDEILEASDGIMVAR
>	sp|P11980|KPY Pyruvate kinase isozymes M1/M2 OS=Rattus norvegicus GN=Pkm2 PE=1 SV=3	RFDEILEASDGIMVAR
>	sp|P11980|KPY Pyruvate kinase isozymes M1/M2 OS=Rattus norvegicus GN=Pkm2 PE=1 SV=3	RFDEILEASDGIMVAR
>	sp|P11980|KPY Pyruvate kinase isozymes M1/M2 OS=Rattus norvegicus GN=Pkm2 PE=1 SV=3	RFDEILEASDGIMVAR
>	sp|P11980|KPY Pyruvate kinase isozymes M1/M2 OS=Rattus norvegicus GN=Pkm2 PE=1 SV=3	RFDEILEASDGIMVAR
>	sp|P11980|KPY Pyruvate kinase isozymes M1/M2 OS=Rattus norvegicus GN=Pkm2 PE=1 SV=3	RFDEILEASDGIMVAR
>	sp|P11980|KPY Pyruvate kinase isozymes M1/M2 OS=Rattus norvegicus GN=Pkm2 PE=1 SV=3	RFDEILEASDGIMVAR
>	sp|P11980|KPY Pyruvate kinase isozymes M1/M2 OS=Rattus norvegicus GN=Pkm2 PE=1 SV=3	RFDEILEASDGIMVAR
>	sp|P11980|KPY Pyruvate kinase isozymes M1/M2 OS=Rattus norvegicus GN=Pkm2 PE=1 SV=3	RFDEILEASDGIMVAR
>	sp|P11980|KPY Pyruvate kinase isozymes M1/M2 OS=Rattus norvegicus GN=Pkm2 PE=1 SV=3	VNLAMNVGK
>	sp|P11980|KPY Pyruvate kinase isozymes M1/M2 OS=Rattus norvegicus GN=Pkm2 PE=1 SV=3	EAEAAVFHR
>	sp|P11980|KPY Pyruvate kinase isozymes M1/M2 OS=Rattus norvegicus GN=Pkm2 PE=1 SV=3	GIFPVLCK
>	sp|P11980|KPY Pyruvate kinase isozymes M1/M2 OS=Rattus norvegicus GN=Pkm2 PE=1 SV=3	VFLAQK
>	sp|P11980|KPY Pyruvate kinase isozymes M1/M2 OS=Rattus norvegicus GN=Pkm2 PE=1 SV=3	GIFPVLCK
>	sp|P11980|KPY Pyruvate kinase isozymes M1/M2 OS=Rattus norvegicus GN=Pkm2 PE=1 SV=3	RFDEILEASDGIMVAR
>	sp|P11980|KPY Pyruvate kinase isozymes M1/M2 OS=Rattus norvegicus GN=Pkm2 PE=1 SV=3	GVNLPGAAVDLPAVSEKDIQDLK
>	sp|P11980|KPY Pyruvate kinase isozymes M1/M2 OS=Rattus norvegicus GN=Pkm2 PE=1 SV=3	GDYPLEAVR
>	sp|P11980|KPY Pyruvate kinase isozymes M1/M2 OS=Rattus norvegicus GN=Pkm2 PE=1 SV=3	GADYLVTEVENGGSLGSK
>	sp|P11980|KPY Pyruvate kinase isozymes M1/M2 OS=Rattus norvegicus GN=Pkm2 PE=1 SV=3	NTGIICTIGPASR
>	sp|P11980|KPY Pyruvate kinase isozymes M1/M2 OS=Rattus norvegicus GN=Pkm2 PE=1 SV=3	GADYLVTEVENGGSLGSK
>	sp|P11980|KPY Pyruvate kinase isozymes M1/M2 OS=Rattus norvegicus GN=Pkm2 PE=1 SV=3	AEGSDVANAVLDGADCIMLSGETAKGDYPLEAVR
>	sp|P11980|KPY Pyruvate kinase isozymes M1/M2 OS=Rattus norvegicus GN=Pkm2 PE=1 SV=3	EAEAAVFHR
>	sp|P11980|KPY Pyruvate kinase isozymes M1/M2 OS=Rattus norvegicus GN=Pkm2 PE=1 SV=3	RFDEILEASDGIMVAR
>	sp|P11980|KPY Pyruvate kinase isozymes M1/M2 OS=Rattus norvegicus GN=Pkm2 PE=1 SV=3	LDIDSAPITAR
>	sp|P11980|KPY Pyruvate kinase isozymes M1/M2 OS=Rattus norvegicus GN=Pkm2 PE=1 SV=3	AATESFASDPILYRPVAVALDTK
>	sp|P11980|KPY Pyruvate kinase isozymes M1/M2 OS=Rattus norvegicus GN=Pkm2 PE=1 SV=3	LDIDSAPITAR
>	sp|P11980|KPY Pyruvate kinase isozymes M1/M2 OS=Rattus norvegicus GN=Pkm2 PE=1 SV=3	GIFPVLCK
>	sp|P11980|KPY Pyruvate kinase isozymes M1/M2 OS=Rattus norvegicus GN=Pkm2 PE=1 SV=3	GIFPVLCK
>	sp|P11980|KPY Pyruvate kinase isozymes M1/M2 OS=Rattus norvegicus GN=Pkm2 PE=1 SV=3	CDENILWLDYK
>	sp|P11980|KPY Pyruvate kinase isozymes M1/M2 OS=Rattus norvegicus GN=Pkm2 PE=1 SV=3	EAEAAVFHR
>	sp|P11980|KPY Pyruvate kinase isozymes M1/M2 OS=Rattus norvegicus GN=Pkm2 PE=1 SV=3	VVIVLTGWRPGSGFTNTMR
>	sp|P11980|KPY Pyruvate kinase isozymes M1/M2 OS=Rattus norvegicus GN=Pkm2 PE=1 SV=3	LLFEELAR
>	sp|P11980|KPY Pyruvate kinase isozymes M1/M2 OS=Rattus norvegicus GN=Pkm2 PE=1 SV=3	AEGSDVANAVLDGADCIMLSGETAK
>	sp|P11980|KPY Pyruvate kinase isozymes M1/M2 OS=Rattus norvegicus GN=Pkm2 PE=1 SV=3	GDLGIEIPAEK
>	sp|P11980|KPY Pyruvate kinase isozymes M1/M2 OS=Rattus norvegicus GN=Pkm2 PE=1 SV=3	RFDEILEASDGIMVAR
>	sp|P11980|KPY Pyruvate kinase isozymes M1/M2 OS=Rattus norvegicus GN=Pkm2 PE=1 SV=3	CDENILWLDYK
>	sp|P11980|KPY Pyruvate kinase isozymes M1/M2 OS=Rattus norvegicus GN=Pkm2 PE=1 SV=3	VNLAMNVGK
>	sp|P11980|KPY Pyruvate kinase isozymes M1/M2 OS=Rattus norvegicus GN=Pkm2 PE=1 SV=3	VFLAQK
>	sp|P11980|KPY Pyruvate kinase isozymes M1/M2 OS=Rattus norvegicus GN=Pkm2 PE=1 SV=3	KGVNLPGAAVDLPAVSEKDIQDLK
>	sp|P11980|KPY Pyruvate kinase isozymes M1/M2 OS=Rattus norvegicus GN=Pkm2 PE=1 SV=3	RFDEILEASDGIMVAR
>	sp|P11980|KPY Pyruvate kinase isozymes M1/M2 OS=Rattus norvegicus GN=Pkm2 PE=1 SV=3	KGVNLPGAAVDLPAVSEKDIQDLK
>	sp|P11980|KPY Pyruvate kinase isozymes M1/M2 OS=Rattus norvegicus GN=Pkm2 PE=1 SV=3	LLFEELAR
>	sp|P11980|KPY Pyruvate kinase isozymes M1/M2 OS=Rattus norvegicus GN=Pkm2 PE=1 SV=3	RFDEILEASDGIMVAR
>	sp|P11980|KPY Pyruvate kinase isozymes M1/M2 OS=Rattus norvegicus GN=Pkm2 PE=1 SV=3	IENHEGVR
>	sp|P11980|KPY Pyruvate kinase isozymes M1/M2 OS=Rattus norvegicus GN=Pkm2 PE=1 SV=3	LLFEELAR

>	sp|P11980|KPY Pyruvate kinase isozymes M1/M2 OS=Rattus norvegicus GN=Pkm2 PE=1 SV=3	GIFPVLCK
>	sp|P11980|KPY Pyruvate kinase isozymes M1/M2 OS=Rattus norvegicus GN=Pkm2 PE=1 SV=3	LDIDSAPITAR
>	sp|P11980|KPY Pyruvate kinase isozymes M1/M2 OS=Rattus norvegicus GN=Pkm2 PE=1 SV=3	AADVHEVR
>	sp|P11980|KPY Pyruvate kinase isozymes M1/M2 OS=Rattus norvegicus GN=Pkm2 PE=1 SV=3	ASSQSTDPLEAMAMGSVEASYK
>	sp|P11980|KPY Pyruvate kinase isozymes M1/M2 OS=Rattus norvegicus GN=Pkm2 PE=1 SV=3	PKPDSEAGTAFIQTQQLHAAMADTFLEHMCR
>	sp|P11980|KPY Pyruvate kinase isozymes M1/M2 OS=Rattus norvegicus GN=Pkm2 PE=1 SV=3	KGVNLPGAAVDLPAVSEKDIQDLK
>	sp|P11980|KPY Pyruvate kinase isozymes M1/M2 OS=Rattus norvegicus GN=Pkm2 PE=1 SV=3	RFDEILEASDGIMVAR
>	sp|P11980|KPY Pyruvate kinase isozymes M1/M2 OS=Rattus norvegicus GN=Pkm2 PE=1 SV=3	KGVNLPGAAVDLPAVSEKDIQDLK
>	sp|P11980|KPY Pyruvate kinase isozymes M1/M2 OS=Rattus norvegicus GN=Pkm2 PE=1 SV=3	KGVNLPGAAVDLPAVSEKDIQDLK
>	sp|P11980|KPY Pyruvate kinase isozymes M1/M2 OS=Rattus norvegicus GN=Pkm2 PE=1 SV=3	KGVNLPGAAVDLPAVSEK
>	sp|P11980|KPY Pyruvate kinase isozymes M1/M2 OS=Rattus norvegicus GN=Pkm2 PE=1 SV=3	KGVNLPGAAVDLPAVSEKDIQDLK
>	sp|P11980|KPY Pyruvate kinase isozymes M1/M2 OS=Rattus norvegicus GN=Pkm2 PE=1 SV=3	RFDEILEASDGIMVAR
>	sp|P11980|KPY Pyruvate kinase isozymes M1/M2 OS=Rattus norvegicus GN=Pkm2 PE=1 SV=3	IENHEGVR
>	sp|P11980|KPY Pyruvate kinase isozymes M1/M2 OS=Rattus norvegicus GN=Pkm2 PE=1 SV=3	RFDEILEASDGIMVAR
>	sp|P11980|KPY Pyruvate kinase isozymes M1/M2 OS=Rattus norvegicus GN=Pkm2 PE=1 SV=3	KGVNLPGAAVDLPAVSEKDIQDLK
>	sp|P11980|KPY Pyruvate kinase isozymes M1/M2 OS=Rattus norvegicus GN=Pkm2 PE=1 SV=3	GVNLPGAAVDLPAVSEKDIQDLK
>	sp|P11980|KPY Pyruvate kinase isozymes M1/M2 OS=Rattus norvegicus GN=Pkm2 PE=1 SV=3	LLFEELAR
>	sp|P11980|KPY Pyruvate kinase isozymes M1/M2 OS=Rattus norvegicus GN=Pkm2 PE=1 SV=3	RFDEILEASDGIMVAR
>	sp|P85108|TBE Tubulin beta-2A chain OS=Rattus norvegicus GN=Tubb2a PE=1 SV=1	AILVDLEPGTMDSVR
>	sp|P85108|TBE Tubulin beta-2A chain OS=Rattus norvegicus GN=Tubb2a PE=1 SV=1	ALTVPELTQQMFDSK
>	sp|P85108|TBE Tubulin beta-2A chain OS=Rattus norvegicus GN=Tubb2a PE=1 SV=1	EIVHIQAGQCGNQIGAK
>	sp|P85108|TBE Tubulin beta-2A chain OS=Rattus norvegicus GN=Tubb2a PE=1 SV=1	EVDEQMLNVQNK
>	sp|P85108|TBE Tubulin beta-2A chain OS=Rattus norvegicus GN=Tubb2a PE=1 SV=1	FPGQLNADLR
>	sp|P85108|TBE Tubulin beta-2A chain OS=Rattus norvegicus GN=Tubb2a PE=1 SV=1	FWEVISDEHGIDPTGSYHGDSDLQLER
>	sp|P85108|TBE Tubulin beta-2A chain OS=Rattus norvegicus GN=Tubb2a PE=1 SV=1	GHYTEGAELVDSVLDVVR
>	sp|P85108|TBE Tubulin beta-2A chain OS=Rattus norvegicus GN=Tubb2a PE=1 SV=1	GHYTEGAELVDSVLDVVRK
>	sp|P85108|TBE Tubulin beta-2A chain OS=Rattus norvegicus GN=Tubb2a PE=1 SV=1	IMNTFSVMPSPK
>	sp|P85108|TBE Tubulin beta-2A chain OS=Rattus norvegicus GN=Tubb2a PE=1 SV=1	INVYYNEAAGNK
>	sp|P85108|TBE Tubulin beta-2A chain OS=Rattus norvegicus GN=Tubb2a PE=1 SV=1	IREEYPDR
>	sp|P85108|TBE Tubulin beta-2A chain OS=Rattus norvegicus GN=Tubb2a PE=1 SV=1	KLAVNMVPFPR
>	sp|P85108|TBE Tubulin beta-2A chain OS=Rattus norvegicus GN=Tubb2a PE=1 SV=1	LAVNMVPFPR
>	sp|P85108|TBE Tubulin beta-2A chain OS=Rattus norvegicus GN=Tubb2a PE=1 SV=1	LHFFMPGFAPLTSR
>	sp|P85108|TBE Tubulin beta-2A chain OS=Rattus norvegicus GN=Tubb2a PE=1 SV=1	LTTPTYGDLNHLVSATMSGVTTCLR
>	sp|P85108|TBE Tubulin beta-2A chain OS=Rattus norvegicus GN=Tubb2a PE=1 SV=1	MSATFIGNSTAIQELFKRISEQFTAMFR
>	sp|P85108|TBE Tubulin beta-2A chain OS=Rattus norvegicus GN=Tubb2a PE=1 SV=1	MSMKEVDEQMLNVQNK
>	sp|P85108|TBE Tubulin beta-2A chain OS=Rattus norvegicus GN=Tubb2a PE=1 SV=1	SGPFGQIFRPDNFVFGQSGAGNNWAK
>	sp|P85108|TBE Tubulin beta-2A chain OS=Rattus norvegicus GN=Tubb2a PE=1 SV=1	TAVCDIPPR
>	sp|P85108|TBE Tubulin beta-2A chain OS=Rattus norvegicus GN=Tubb2a PE=1 SV=1	VSDTVVEPYNATLSVHQLVENTDETYSIDNEALYDICFR
>	sp|P85108|TBE Tubulin beta-2A chain OS=Rattus norvegicus GN=Tubb2a PE=1 SV=1	PGFAPLTSR
>	sp|P85108|TBE Tubulin beta-2A chain OS=Rattus norvegicus GN=Tubb2a PE=1 SV=1	KLAVNMVPFPRLHFFMPGFAPLTSR
>	sp|P85108|TBE Tubulin beta-2A chain OS=Rattus norvegicus GN=Tubb2a PE=1 SV=1	FPGQLNADLRK
>	sp|P85108|TBE Tubulin beta-2A chain OS=Rattus norvegicus GN=Tubb2a PE=1 SV=1	KESESCDCLQGFQLTHSLGGGTGSGMGTLLISK
>	sp|P85108|TBE Tubulin beta-2A chain OS=Rattus norvegicus GN=Tubb2a PE=1 SV=1	NSSYFVEWIPNNVK
>	sp|P85108|TBE Tubulin beta-2A chain OS=Rattus norvegicus GN=Tubb2a PE=1 SV=1	PGQLNADLR
>	sp|P85108|TBE Tubulin beta-2A chain OS=Rattus norvegicus GN=Tubb2a PE=1 SV=1	PGTMDSVR
>	sp|P85108|TBE Tubulin beta-2A chain OS=Rattus norvegicus GN=Tubb2a PE=1 SV=1	PGQLNADLRK
>	sp|P85108|TBE Tubulin beta-2A chain OS=Rattus norvegicus GN=Tubb2a PE=1 SV=1	AILVDLEPGTMDSVR
>	sp|P85108|TBE Tubulin beta-2A chain OS=Rattus norvegicus GN=Tubb2a PE=1 SV=1	AILVDLEPGTMDSVR
>	sp|P85108|TBE Tubulin beta-2A chain OS=Rattus norvegicus GN=Tubb2a PE=1 SV=1	AILVDLEPGTMDSVR
>	sp|P85108|TBE Tubulin beta-2A chain OS=Rattus norvegicus GN=Tubb2a PE=1 SV=1	AILVDLEPGTMDSVR
>	sp|P85108|TBE Tubulin beta-2A chain OS=Rattus norvegicus GN=Tubb2a PE=1 SV=1	AILVDLEPGTMDSVR
>	sp|P85108|TBE Tubulin beta-2A chain OS=Rattus norvegicus GN=Tubb2a PE=1 SV=1	AILVDLEPGTMDSVR
>	sp|P85108|TBE Tubulin beta-2A chain OS=Rattus norvegicus GN=Tubb2a PE=1 SV=1	AILVDLEPGTMDSVR
>	sp|P85108|TBE Tubulin beta-2A chain OS=Rattus norvegicus GN=Tubb2a PE=1 SV=1	AILVDLEPGTMDSVR
>	sp|P85108|TBE Tubulin beta-2A chain OS=Rattus norvegicus GN=Tubb2a PE=1 SV=1	AILVDLEPGTMDSVR
>	sp|P85108|TBE Tubulin beta-2A chain OS=Rattus norvegicus GN=Tubb2a PE=1 SV=1	AILVDLEPGTMDSVR
>	sp|P85108|TBE Tubulin beta-2A chain OS=Rattus norvegicus GN=Tubb2a PE=1 SV=1	ALTVPELTQQMFDSK
>	sp|P85108|TBE Tubulin beta-2A chain OS=Rattus norvegicus GN=Tubb2a PE=1 SV=1	EIVHIQAGQCGNQIGAK
>	sp|P85108|TBE Tubulin beta-2A chain OS=Rattus norvegicus GN=Tubb2a PE=1 SV=1	EIVHIQAGQCGNQIGAK
>	sp|P85108|TBE Tubulin beta-2A chain OS=Rattus norvegicus GN=Tubb2a PE=1 SV=1	EIVHIQAGQCGNQIGAK
>	sp|P85108|TBE Tubulin beta-2A chain OS=Rattus norvegicus GN=Tubb2a PE=1 SV=1	EIVHIQAGQCGNQIGAK
>	sp|P85108|TBE Tubulin beta-2A chain OS=Rattus norvegicus GN=Tubb2a PE=1 SV=1	EIVHIQAGQCGNQIGAK
>	sp|P85108|TBE Tubulin beta-2A chain OS=Rattus norvegicus GN=Tubb2a PE=1 SV=1	EIVHIQAGQCGNQIGAK
>	sp|P85108|TBE Tubulin beta-2A chain OS=Rattus norvegicus GN=Tubb2a PE=1 SV=1	EIVHIQAGQCGNQIGAK
>	sp|P85108|TBE Tubulin beta-2A chain OS=Rattus norvegicus GN=Tubb2a PE=1 SV=1	EIVHIQAGQCGNQIGAK
>	sp|P85108|TBE Tubulin beta-2A chain OS=Rattus norvegicus GN=Tubb2a PE=1 SV=1	EIVHIQAGQCGNQIGAK
>	sp|P85108|TBE Tubulin beta-2A chain OS=Rattus norvegicus GN=Tubb2a PE=1 SV=1	EIVHIQAGQCGNQIGAK
>	sp|P85108|TBE Tubulin beta-2A chain OS=Rattus norvegicus GN=Tubb2a PE=1 SV=1	EIVHIQAGQCGNQIGAK
>	sp|P85108|TBE Tubulin beta-2A chain OS=Rattus norvegicus GN=Tubb2a PE=1 SV=1	EIVHIQAGQCGNQIGAK
>	sp|P85108|TBE Tubulin beta-2A chain OS=Rattus norvegicus GN=Tubb2a PE=1 SV=1	EIVHIQAGQCGNQIGAK
>	sp|P85108|TBE Tubulin beta-2A chain OS=Rattus norvegicus GN=Tubb2a PE=1 SV=1	EIVHIQAGQCGNQIGAK
>	sp|P85108|TBE Tubulin beta-2A chain OS=Rattus norvegicus GN=Tubb2a PE=1 SV=1	EIVHIQAGQCGNQIGAK
>	sp|P85108|TBE Tubulin beta-2A chain OS=Rattus norvegicus GN=Tubb2a PE=1 SV=1	EIVHIQAGQCGNQIGAK
>	sp|P85108|TBE Tubulin beta-2A chain OS=Rattus norvegicus GN=Tubb2a PE=1 SV=1	EIVHIQAGQCGNQIGAK

<table>
<tr><td>></td><td>sp|P85108|TBE Tubulin beta-2A chain OS=Rattus norvegicus GN=Tubb2a PE=1 SV=1</td><td>EIVHIQAGQCGNQIGAK</td></tr>
<tr><td>></td><td>sp|P85108|TBE Tubulin beta-2A chain OS=Rattus norvegicus GN=Tubb2a PE=1 SV=1</td><td>EIVHIQAGQCGNQIGAK</td></tr>
<tr><td>></td><td>sp|P85108|TBE Tubulin beta-2A chain OS=Rattus norvegicus GN=Tubb2a PE=1 SV=1</td><td>EIVHIQAGQCGNQIGAK</td></tr>
<tr><td>></td><td>sp|P85108|TBE Tubulin beta-2A chain OS=Rattus norvegicus GN=Tubb2a PE=1 SV=1</td><td>EIVHIQAGQCGNQIGAK</td></tr>
<tr><td>></td><td>sp|P85108|TBE Tubulin beta-2A chain OS=Rattus norvegicus GN=Tubb2a PE=1 SV=1</td><td>EIVHIQAGQCGNQIGAK</td></tr>
<tr><td>></td><td>sp|P85108|TBE Tubulin beta-2A chain OS=Rattus norvegicus GN=Tubb2a PE=1 SV=1</td><td>EIVHIQAGQCGNQIGAK</td></tr>
<tr><td>></td><td>sp|P85108|TBE Tubulin beta-2A chain OS=Rattus norvegicus GN=Tubb2a PE=1 SV=1</td><td>EIVHIQAGQCGNQIGAK</td></tr>
<tr><td>></td><td>sp|P85108|TBE Tubulin beta-2A chain OS=Rattus norvegicus GN=Tubb2a PE=1 SV=1</td><td>EIVHIQAGQCGNQIGAK</td></tr>
<tr><td>></td><td>sp|P85108|TBE Tubulin beta-2A chain OS=Rattus norvegicus GN=Tubb2a PE=1 SV=1</td><td>EIVHIQAGQCGNQIGAK</td></tr>
<tr><td>></td><td>sp|P85108|TBE Tubulin beta-2A chain OS=Rattus norvegicus GN=Tubb2a PE=1 SV=1</td><td>EVDEQMLNVQNK</td></tr>
<tr><td>></td><td>sp|P85108|TBE Tubulin beta-2A chain OS=Rattus norvegicus GN=Tubb2a PE=1 SV=1</td><td>EVDEQMLNVQNK</td></tr>
<tr><td>></td><td>sp|P85108|TBE Tubulin beta-2A chain OS=Rattus norvegicus GN=Tubb2a PE=1 SV=1</td><td>EVDEQMLNVQNK</td></tr>
<tr><td>></td><td>sp|P85108|TBE Tubulin beta-2A chain OS=Rattus norvegicus GN=Tubb2a PE=1 SV=1</td><td>FPGQLNADLR</td></tr>
<tr><td>></td><td>sp|P85108|TBE Tubulin beta-2A chain OS=Rattus norvegicus GN=Tubb2a PE=1 SV=1</td><td>FPGQLNADLR</td></tr>
<tr><td>></td><td>sp|P85108|TBE Tubulin beta-2A chain OS=Rattus norvegicus GN=Tubb2a PE=1 SV=1</td><td>FPGQLNADLR</td></tr>
<tr><td>></td><td>sp|P85108|TBE Tubulin beta-2A chain OS=Rattus norvegicus GN=Tubb2a PE=1 SV=1</td><td>FPGQLNADLR</td></tr>
<tr><td>></td><td>sp|P85108|TBE Tubulin beta-2A chain OS=Rattus norvegicus GN=Tubb2a PE=1 SV=1</td><td>FPGQLNADLR</td></tr>
<tr><td>></td><td>sp|P85108|TBE Tubulin beta-2A chain OS=Rattus norvegicus GN=Tubb2a PE=1 SV=1</td><td>FWEVISDEHGIDPTGSYHGDSDLQLER</td></tr>
<tr><td>></td><td>sp|P85108|TBE Tubulin beta-2A chain OS=Rattus norvegicus GN=Tubb2a PE=1 SV=1</td><td>FWEVISDEHGIDPTGSYHGDSDLQLER</td></tr>
<tr><td>></td><td>sp|P85108|TBE Tubulin beta-2A chain OS=Rattus norvegicus GN=Tubb2a PE=1 SV=1</td><td>FWEVISDEHGIDPTGSYHGDSDLQLER</td></tr>
<tr><td>></td><td>sp|P85108|TBE Tubulin beta-2A chain OS=Rattus norvegicus GN=Tubb2a PE=1 SV=1</td><td>FWEVISDEHGIDPTGSYHGDSDLQLER</td></tr>
<tr><td>></td><td>sp|P85108|TBE Tubulin beta-2A chain OS=Rattus norvegicus GN=Tubb2a PE=1 SV=1</td><td>GHYTEGAELVDSVLDVVR</td></tr>
<tr><td>></td><td>sp|P85108|TBE Tubulin beta-2A chain OS=Rattus norvegicus GN=Tubb2a PE=1 SV=1</td><td>GHYTEGAELVDSVLDVVR</td></tr>
<tr><td>></td><td>sp|P85108|TBE Tubulin beta-2A chain OS=Rattus norvegicus GN=Tubb2a PE=1 SV=1</td><td>GHYTEGAELVDSVLDVVR</td></tr>
<tr><td>></td><td>sp|P85108|TBE Tubulin beta-2A chain OS=Rattus norvegicus GN=Tubb2a PE=1 SV=1</td><td>GHYTEGAELVDSVLDVVR</td></tr>
<tr><td>></td><td>sp|P85108|TBE Tubulin beta-2A chain OS=Rattus norvegicus GN=Tubb2a PE=1 SV=1</td><td>GHYTEGAELVDSVLDVVR</td></tr>
<tr><td>></td><td>sp|P85108|TBE Tubulin beta-2A chain OS=Rattus norvegicus GN=Tubb2a PE=1 SV=1</td><td>GHYTEGAELVDSVLDVVR</td></tr>
<tr><td>></td><td>sp|P85108|TBE Tubulin beta-2A chain OS=Rattus norvegicus GN=Tubb2a PE=1 SV=1</td><td>GHYTEGAELVDSVLDVVR</td></tr>
<tr><td>></td><td>sp|P85108|TBE Tubulin beta-2A chain OS=Rattus norvegicus GN=Tubb2a PE=1 SV=1</td><td>GHYTEGAELVDSVLDVVR</td></tr>
<tr><td>></td><td>sp|P85108|TBE Tubulin beta-2A chain OS=Rattus norvegicus GN=Tubb2a PE=1 SV=1</td><td>GHYTEGAELVDSVLDVVR</td></tr>
<tr><td>></td><td>sp|P85108|TBE Tubulin beta-2A chain OS=Rattus norvegicus GN=Tubb2a PE=1 SV=1</td><td>GHYTEGAELVDSVLDVVR</td></tr>
<tr><td>></td><td>sp|P85108|TBE Tubulin beta-2A chain OS=Rattus norvegicus GN=Tubb2a PE=1 SV=1</td><td>GHYTEGAELVDSVLDVVR</td></tr>
<tr><td>></td><td>sp|P85108|TBE Tubulin beta-2A chain OS=Rattus norvegicus GN=Tubb2a PE=1 SV=1</td><td>GHYTEGAELVDSVLDVVR</td></tr>
<tr><td>></td><td>sp|P85108|TBE Tubulin beta-2A chain OS=Rattus norvegicus GN=Tubb2a PE=1 SV=1</td><td>GHYTEGAELVDSVLDVVR</td></tr>
<tr><td>></td><td>sp|P85108|TBE Tubulin beta-2A chain OS=Rattus norvegicus GN=Tubb2a PE=1 SV=1</td><td>GHYTEGAELVDSVLDVVR</td></tr>
<tr><td>></td><td>sp|P85108|TBE Tubulin beta-2A chain OS=Rattus norvegicus GN=Tubb2a PE=1 SV=1</td><td>GHYTEGAELVDSVLDVVR</td></tr>
<tr><td>></td><td>sp|P85108|TBE Tubulin beta-2A chain OS=Rattus norvegicus GN=Tubb2a PE=1 SV=1</td><td>GHYTEGAELVDSVLDVVR</td></tr>
<tr><td>></td><td>sp|P85108|TBE Tubulin beta-2A chain OS=Rattus norvegicus GN=Tubb2a PE=1 SV=1</td><td>GHYTEGAELVDSVLDVVR</td></tr>
<tr><td>></td><td>sp|P85108|TBE Tubulin beta-2A chain OS=Rattus norvegicus GN=Tubb2a PE=1 SV=1</td><td>GHYTEGAELVDSVLDVVR</td></tr>
<tr><td>></td><td>sp|P85108|TBE Tubulin beta-2A chain OS=Rattus norvegicus GN=Tubb2a PE=1 SV=1</td><td>GHYTEGAELVDSVLDVVR</td></tr>
<tr><td>></td><td>sp|P85108|TBE Tubulin beta-2A chain OS=Rattus norvegicus GN=Tubb2a PE=1 SV=1</td><td>GHYTEGAELVDSVLDVVR</td></tr>
<tr><td>></td><td>sp|P85108|TBE Tubulin beta-2A chain OS=Rattus norvegicus GN=Tubb2a PE=1 SV=1</td><td>GHYTEGAELVDSVLDVVR</td></tr>
<tr><td>></td><td>sp|P85108|TBE Tubulin beta-2A chain OS=Rattus norvegicus GN=Tubb2a PE=1 SV=1</td><td>GHYTEGAELVDSVLDVVR</td></tr>
<tr><td>></td><td>sp|P85108|TBE Tubulin beta-2A chain OS=Rattus norvegicus GN=Tubb2a PE=1 SV=1</td><td>GHYTEGAELVDSVLDVVR</td></tr>
<tr><td>></td><td>sp|P85108|TBE Tubulin beta-2A chain OS=Rattus norvegicus GN=Tubb2a PE=1 SV=1</td><td>GHYTEGAELVDSVLDVVR</td></tr>
<tr><td>></td><td>sp|P85108|TBE Tubulin beta-2A chain OS=Rattus norvegicus GN=Tubb2a PE=1 SV=1</td><td>GHYTEGAELVDSVLDVVR</td></tr>
<tr><td>></td><td>sp|P85108|TBE Tubulin beta-2A chain OS=Rattus norvegicus GN=Tubb2a PE=1 SV=1</td><td>GHYTEGAELVDSVLDVVR</td></tr>
<tr><td>></td><td>sp|P85108|TBE Tubulin beta-2A chain OS=Rattus norvegicus GN=Tubb2a PE=1 SV=1</td><td>GHYTEGAELVDSVLDVVR</td></tr>
<tr><td>></td><td>sp|P85108|TBE Tubulin beta-2A chain OS=Rattus norvegicus GN=Tubb2a PE=1 SV=1</td><td>GHYTEGAELVDSVLDVVR</td></tr>
<tr><td>></td><td>sp|P85108|TBE Tubulin beta-2A chain OS=Rattus norvegicus GN=Tubb2a PE=1 SV=1</td><td>GHYTEGAELVDSVLDVVR</td></tr>
<tr><td>></td><td>sp|P85108|TBE Tubulin beta-2A chain OS=Rattus norvegicus GN=Tubb2a PE=1 SV=1</td><td>GHYTEGAELVDSVLDVVR</td></tr>
<tr><td>></td><td>sp|P85108|TBE Tubulin beta-2A chain OS=Rattus norvegicus GN=Tubb2a PE=1 SV=1</td><td>GHYTEGAELVDSVLDVVR</td></tr>
<tr><td>></td><td>sp|P85108|TBE Tubulin beta-2A chain OS=Rattus norvegicus GN=Tubb2a PE=1 SV=1</td><td>IMNTFSVMPSPK</td></tr>
<tr><td>></td><td>sp|P85108|TBE Tubulin beta-2A chain OS=Rattus norvegicus GN=Tubb2a PE=1 SV=1</td><td>INVYYNEAAGNK</td></tr>
<tr><td>></td><td>sp|P85108|TBE Tubulin beta-2A chain OS=Rattus norvegicus GN=Tubb2a PE=1 SV=1</td><td>INVYYNEAAGNK</td></tr>
<tr><td>></td><td>sp|P85108|TBE Tubulin beta-2A chain OS=Rattus norvegicus GN=Tubb2a PE=1 SV=1</td><td>KESESCDCLQGFQLTHSLGGGTGSGMGTLLISK</td></tr>
<tr><td>></td><td>sp|P85108|TBE Tubulin beta-2A chain OS=Rattus norvegicus GN=Tubb2a PE=1 SV=1</td><td>KESESCDCLQGFQLTHSLGGGTGSGMGTLLISK</td></tr>
<tr><td>></td><td>sp|P85108|TBE Tubulin beta-2A chain OS=Rattus norvegicus GN=Tubb2a PE=1 SV=1</td><td>KLAVNMVPFPR</td></tr>
<tr><td>></td><td>sp|P85108|TBE Tubulin beta-2A chain OS=Rattus norvegicus GN=Tubb2a PE=1 SV=1</td><td>KLAVNMVPFPR</td></tr>
<tr><td>></td><td>sp|P85108|TBE Tubulin beta-2A chain OS=Rattus norvegicus GN=Tubb2a PE=1 SV=1</td><td>KLAVNMVPFPR</td></tr>
<tr><td>></td><td>sp|P85108|TBE Tubulin beta-2A chain OS=Rattus norvegicus GN=Tubb2a PE=1 SV=1</td><td>KLAVNMVPFPR</td></tr>
<tr><td>></td><td>sp|P85108|TBE Tubulin beta-2A chain OS=Rattus norvegicus GN=Tubb2a PE=1 SV=1</td><td>KLAVNMVPFPR</td></tr>
<tr><td>></td><td>sp|P85108|TBE Tubulin beta-2A chain OS=Rattus norvegicus GN=Tubb2a PE=1 SV=1</td><td>KLAVNMVPFPR</td></tr>
<tr><td>></td><td>sp|P85108|TBE Tubulin beta-2A chain OS=Rattus norvegicus GN=Tubb2a PE=1 SV=1</td><td>KLAVNMVPFPR</td></tr>
<tr><td>></td><td>sp|P85108|TBE Tubulin beta-2A chain OS=Rattus norvegicus GN=Tubb2a PE=1 SV=1</td><td>KLAVNMVPFPR</td></tr>
<tr><td>></td><td>sp|P85108|TBE Tubulin beta-2A chain OS=Rattus norvegicus GN=Tubb2a PE=1 SV=1</td><td>KLAVNMVPFPR</td></tr>
<tr><td>></td><td>sp|P85108|TBE Tubulin beta-2A chain OS=Rattus norvegicus GN=Tubb2a PE=1 SV=1</td><td>KLAVNMVPFPR</td></tr>
<tr><td>></td><td>sp|P85108|TBE Tubulin beta-2A chain OS=Rattus norvegicus GN=Tubb2a PE=1 SV=1</td><td>KLAVNMVPFPR</td></tr>
<tr><td>></td><td>sp|P85108|TBE Tubulin beta-2A chain OS=Rattus norvegicus GN=Tubb2a PE=1 SV=1</td><td>KLAVNMVPFPR</td></tr>
<tr><td>></td><td>sp|P85108|TBE Tubulin beta-2A chain OS=Rattus norvegicus GN=Tubb2a PE=1 SV=1</td><td>KLAVNMVPFPR</td></tr>
<tr><td>></td><td>sp|P85108|TBE Tubulin beta-2A chain OS=Rattus norvegicus GN=Tubb2a PE=1 SV=1</td><td>KLAVNMVPFPRLHFFMPGFAPLTSR</td></tr>
<tr><td>></td><td>sp|P85108|TBE Tubulin beta-2A chain OS=Rattus norvegicus GN=Tubb2a PE=1 SV=1</td><td>KLAVNMVPFPRLHFFMPGFAPLTSR</td></tr>
<tr><td>></td><td>sp|P85108|TBE Tubulin beta-2A chain OS=Rattus norvegicus GN=Tubb2a PE=1 SV=1</td><td>LAVNMVPFPR</td></tr>
<tr><td>></td><td>sp|P85108|TBE Tubulin beta-2A chain OS=Rattus norvegicus GN=Tubb2a PE=1 SV=1</td><td>LAVNMVPFPR</td></tr>
</table>

<table>
<tr><td>></td><td>sp|P85108|TBE Tubulin beta-2A chain OS=Rattus norvegicus GN=Tubb2a PE=1 SV=1</td><td>LAVNMVPFPR</td></tr>
<tr><td>></td><td>sp|P85108|TBE Tubulin beta-2A chain OS=Rattus norvegicus GN=Tubb2a PE=1 SV=1</td><td>LAVNMVPFPR</td></tr>
<tr><td>></td><td>sp|P85108|TBE Tubulin beta-2A chain OS=Rattus norvegicus GN=Tubb2a PE=1 SV=1</td><td>LAVNMVPFPR</td></tr>
<tr><td>></td><td>sp|P85108|TBE Tubulin beta-2A chain OS=Rattus norvegicus GN=Tubb2a PE=1 SV=1</td><td>LAVNMVPFPR</td></tr>
<tr><td>></td><td>sp|P85108|TBE Tubulin beta-2A chain OS=Rattus norvegicus GN=Tubb2a PE=1 SV=1</td><td>LAVNMVPFPR</td></tr>
<tr><td>></td><td>sp|P85108|TBE Tubulin beta-2A chain OS=Rattus norvegicus GN=Tubb2a PE=1 SV=1</td><td>LHFFMPGFAPLTSR</td></tr>
<tr><td>></td><td>sp|P85108|TBE Tubulin beta-2A chain OS=Rattus norvegicus GN=Tubb2a PE=1 SV=1</td><td>LHFFMPGFAPLTSR</td></tr>
<tr><td>></td><td>sp|P85108|TBE Tubulin beta-2A chain OS=Rattus norvegicus GN=Tubb2a PE=1 SV=1</td><td>LHFFMPGFAPLTSR</td></tr>
<tr><td>></td><td>sp|P85108|TBE Tubulin beta-2A chain OS=Rattus norvegicus GN=Tubb2a PE=1 SV=1</td><td>LHFFMPGFAPLTSR</td></tr>
<tr><td>></td><td>sp|P85108|TBE Tubulin beta-2A chain OS=Rattus norvegicus GN=Tubb2a PE=1 SV=1</td><td>LHFFMPGFAPLTSR</td></tr>
<tr><td>></td><td>sp|P85108|TBE Tubulin beta-2A chain OS=Rattus norvegicus GN=Tubb2a PE=1 SV=1</td><td>LHFFMPGFAPLTSR</td></tr>
<tr><td>></td><td>sp|P85108|TBE Tubulin beta-2A chain OS=Rattus norvegicus GN=Tubb2a PE=1 SV=1</td><td>LHFFMPGFAPLTSR</td></tr>
<tr><td>></td><td>sp|P85108|TBE Tubulin beta-2A chain OS=Rattus norvegicus GN=Tubb2a PE=1 SV=1</td><td>LHFFMPGFAPLTSR</td></tr>
<tr><td>></td><td>sp|P85108|TBE Tubulin beta-2A chain OS=Rattus norvegicus GN=Tubb2a PE=1 SV=1</td><td>LHFFMPGFAPLTSR</td></tr>
<tr><td>></td><td>sp|P85108|TBE Tubulin beta-2A chain OS=Rattus norvegicus GN=Tubb2a PE=1 SV=1</td><td>LHFFMPGFAPLTSR</td></tr>
<tr><td>></td><td>sp|P85108|TBE Tubulin beta-2A chain OS=Rattus norvegicus GN=Tubb2a PE=1 SV=1</td><td>LHFFMPGFAPLTSR</td></tr>
<tr><td>></td><td>sp|P85108|TBE Tubulin beta-2A chain OS=Rattus norvegicus GN=Tubb2a PE=1 SV=1</td><td>LHFFMPGFAPLTSR</td></tr>
<tr><td>></td><td>sp|P85108|TBE Tubulin beta-2A chain OS=Rattus norvegicus GN=Tubb2a PE=1 SV=1</td><td>LHFFMPGFAPLTSR</td></tr>
<tr><td>></td><td>sp|P85108|TBE Tubulin beta-2A chain OS=Rattus norvegicus GN=Tubb2a PE=1 SV=1</td><td>LHFFMPGFAPLTSR</td></tr>
<tr><td>></td><td>sp|P85108|TBE Tubulin beta-2A chain OS=Rattus norvegicus GN=Tubb2a PE=1 SV=1</td><td>LHFFMPGFAPLTSR</td></tr>
<tr><td>></td><td>sp|P85108|TBE Tubulin beta-2A chain OS=Rattus norvegicus GN=Tubb2a PE=1 SV=1</td><td>LHFFMPGFAPLTSR</td></tr>
<tr><td>></td><td>sp|P85108|TBE Tubulin beta-2A chain OS=Rattus norvegicus GN=Tubb2a PE=1 SV=1</td><td>LHFFMPGFAPLTSR</td></tr>
<tr><td>></td><td>sp|P85108|TBE Tubulin beta-2A chain OS=Rattus norvegicus GN=Tubb2a PE=1 SV=1</td><td>LHFFMPGFAPLTSR</td></tr>
<tr><td>></td><td>sp|P85108|TBE Tubulin beta-2A chain OS=Rattus norvegicus GN=Tubb2a PE=1 SV=1</td><td>LHFFMPGFAPLTSR</td></tr>
<tr><td>></td><td>sp|P85108|TBE Tubulin beta-2A chain OS=Rattus norvegicus GN=Tubb2a PE=1 SV=1</td><td>LHFFMPGFAPLTSR</td></tr>
<tr><td>></td><td>sp|P85108|TBE Tubulin beta-2A chain OS=Rattus norvegicus GN=Tubb2a PE=1 SV=1</td><td>LHFFMPGFAPLTSR</td></tr>
<tr><td>></td><td>sp|P85108|TBE Tubulin beta-2A chain OS=Rattus norvegicus GN=Tubb2a PE=1 SV=1</td><td>LHFFMPGFAPLTSR</td></tr>
<tr><td>></td><td>sp|P85108|TBE Tubulin beta-2A chain OS=Rattus norvegicus GN=Tubb2a PE=1 SV=1</td><td>LHFFMPGFAPLTSR</td></tr>
<tr><td>></td><td>sp|P85108|TBE Tubulin beta-2A chain OS=Rattus norvegicus GN=Tubb2a PE=1 SV=1</td><td>LHFFMPGFAPLTSR</td></tr>
<tr><td>></td><td>sp|P85108|TBE Tubulin beta-2A chain OS=Rattus norvegicus GN=Tubb2a PE=1 SV=1</td><td>LHFFMPGFAPLTSR</td></tr>
<tr><td>></td><td>sp|P85108|TBE Tubulin beta-2A chain OS=Rattus norvegicus GN=Tubb2a PE=1 SV=1</td><td>LHFFMPGFAPLTSR</td></tr>
<tr><td>></td><td>sp|P85108|TBE Tubulin beta-2A chain OS=Rattus norvegicus GN=Tubb2a PE=1 SV=1</td><td>LHFFMPGFAPLTSR</td></tr>
<tr><td>></td><td>sp|P85108|TBE Tubulin beta-2A chain OS=Rattus norvegicus GN=Tubb2a PE=1 SV=1</td><td>LHFFMPGFAPLTSR</td></tr>
<tr><td>></td><td>sp|P85108|TBE Tubulin beta-2A chain OS=Rattus norvegicus GN=Tubb2a PE=1 SV=1</td><td>LHFFMPGFAPLTSR</td></tr>
<tr><td>></td><td>sp|P85108|TBE Tubulin beta-2A chain OS=Rattus norvegicus GN=Tubb2a PE=1 SV=1</td><td>LHFFMPGFAPLTSR</td></tr>
<tr><td>></td><td>sp|P85108|TBE Tubulin beta-2A chain OS=Rattus norvegicus GN=Tubb2a PE=1 SV=1</td><td>LHFFMPGFAPLTSR</td></tr>
<tr><td>></td><td>sp|P85108|TBE Tubulin beta-2A chain OS=Rattus norvegicus GN=Tubb2a PE=1 SV=1</td><td>LHFFMPGFAPLTSR</td></tr>
<tr><td>></td><td>sp|P85108|TBE Tubulin beta-2A chain OS=Rattus norvegicus GN=Tubb2a PE=1 SV=1</td><td>LHFFMPGFAPLTSR</td></tr>
<tr><td>></td><td>sp|P85108|TBE Tubulin beta-2A chain OS=Rattus norvegicus GN=Tubb2a PE=1 SV=1</td><td>LHFFMPGFAPLTSR</td></tr>
<tr><td>></td><td>sp|P85108|TBE Tubulin beta-2A chain OS=Rattus norvegicus GN=Tubb2a PE=1 SV=1</td><td>LHFFMPGFAPLTSR</td></tr>
<tr><td>></td><td>sp|P85108|TBE Tubulin beta-2A chain OS=Rattus norvegicus GN=Tubb2a PE=1 SV=1</td><td>LHFFMPGFAPLTSR</td></tr>
<tr><td>></td><td>sp|P85108|TBE Tubulin beta-2A chain OS=Rattus norvegicus GN=Tubb2a PE=1 SV=1</td><td>LHFFMPGFAPLTSR</td></tr>
<tr><td>></td><td>sp|P85108|TBE Tubulin beta-2A chain OS=Rattus norvegicus GN=Tubb2a PE=1 SV=1</td><td>LHFFMPGFAPLTSR</td></tr>
<tr><td>></td><td>sp|P85108|TBE Tubulin beta-2A chain OS=Rattus norvegicus GN=Tubb2a PE=1 SV=1</td><td>LHFFMPGFAPLTSR</td></tr>
<tr><td>></td><td>sp|P85108|TBE Tubulin beta-2A chain OS=Rattus norvegicus GN=Tubb2a PE=1 SV=1</td><td>LHFFMPGFAPLTSR</td></tr>
<tr><td>></td><td>sp|P85108|TBE Tubulin beta-2A chain OS=Rattus norvegicus GN=Tubb2a PE=1 SV=1</td><td>LHFFMPGFAPLTSR</td></tr>
<tr><td>></td><td>sp|P85108|TBE Tubulin beta-2A chain OS=Rattus norvegicus GN=Tubb2a PE=1 SV=1</td><td>LHFFMPGFAPLTSR</td></tr>
<tr><td>></td><td>sp|P85108|TBE Tubulin beta-2A chain OS=Rattus norvegicus GN=Tubb2a PE=1 SV=1</td><td>LHFFMPGFAPLTSR</td></tr>
<tr><td>></td><td>sp|P85108|TBE Tubulin beta-2A chain OS=Rattus norvegicus GN=Tubb2a PE=1 SV=1</td><td>LHFFMPGFAPLTSR</td></tr>
<tr><td>></td><td>sp|P85108|TBE Tubulin beta-2A chain OS=Rattus norvegicus GN=Tubb2a PE=1 SV=1</td><td>LTTPTYGDLNHLVSATMSGVTTCLR</td></tr>
<tr><td>></td><td>sp|P85108|TBE Tubulin beta-2A chain OS=Rattus norvegicus GN=Tubb2a PE=1 SV=1</td><td>LTTPTYGDLNHLVSATMSGVTTCLR</td></tr>
<tr><td>></td><td>sp|P85108|TBE Tubulin beta-2A chain OS=Rattus norvegicus GN=Tubb2a PE=1 SV=1</td><td>LTTPTYGDLNHLVSATMSGVTTCLR</td></tr>
<tr><td>></td><td>sp|P85108|TBE Tubulin beta-2A chain OS=Rattus norvegicus GN=Tubb2a PE=1 SV=1</td><td>LTTPTYGDLNHLVSATMSGVTTCLR</td></tr>
<tr><td>></td><td>sp|P85108|TBE Tubulin beta-2A chain OS=Rattus norvegicus GN=Tubb2a PE=1 SV=1</td><td>LTTPTYGDLNHLVSATMSGVTTCLR</td></tr>
<tr><td>></td><td>sp|P85108|TBE Tubulin beta-2A chain OS=Rattus norvegicus GN=Tubb2a PE=1 SV=1</td><td>LTTPTYGDLNHLVSATMSGVTTCLR</td></tr>
<tr><td>></td><td>sp|P85108|TBE Tubulin beta-2A chain OS=Rattus norvegicus GN=Tubb2a PE=1 SV=1</td><td>LTTPTYGDLNHLVSATMSGVTTCLR</td></tr>
<tr><td>></td><td>sp|P85108|TBE Tubulin beta-2A chain OS=Rattus norvegicus GN=Tubb2a PE=1 SV=1</td><td>LTTPTYGDLNHLVSATMSGVTTCLR</td></tr>
<tr><td>></td><td>sp|P85108|TBE Tubulin beta-2A chain OS=Rattus norvegicus GN=Tubb2a PE=1 SV=1</td><td>LTTPTYGDLNHLVSATMSGVTTCLR</td></tr>
<tr><td>></td><td>sp|P85108|TBE Tubulin beta-2A chain OS=Rattus norvegicus GN=Tubb2a PE=1 SV=1</td><td>LTTPTYGDLNHLVSATMSGVTTCLR</td></tr>
<tr><td>></td><td>sp|P85108|TBE Tubulin beta-2A chain OS=Rattus norvegicus GN=Tubb2a PE=1 SV=1</td><td>LTTPTYGDLNHLVSATMSGVTTCLR</td></tr>
<tr><td>></td><td>sp|P85108|TBE Tubulin beta-2A chain OS=Rattus norvegicus GN=Tubb2a PE=1 SV=1</td><td>LTTPTYGDLNHLVSATMSGVTTCLR</td></tr>
<tr><td>></td><td>sp|P85108|TBE Tubulin beta-2A chain OS=Rattus norvegicus GN=Tubb2a PE=1 SV=1</td><td>LTTPTYGDLNHLVSATMSGVTTCLR</td></tr>
<tr><td>></td><td>sp|P85108|TBE Tubulin beta-2A chain OS=Rattus norvegicus GN=Tubb2a PE=1 SV=1</td><td>LTTPTYGDLNHLVSATMSGVTTCLR</td></tr>
<tr><td>></td><td>sp|P85108|TBE Tubulin beta-2A chain OS=Rattus norvegicus GN=Tubb2a PE=1 SV=1</td><td>LTTPTYGDLNHLVSATMSGVTTCLR</td></tr>
<tr><td>></td><td>sp|P85108|TBE Tubulin beta-2A chain OS=Rattus norvegicus GN=Tubb2a PE=1 SV=1</td><td>LTTPTYGDLNHLVSATMSGVTTCLR</td></tr>
<tr><td>></td><td>sp|P85108|TBE Tubulin beta-2A chain OS=Rattus norvegicus GN=Tubb2a PE=1 SV=1</td><td>LTTPTYGDLNHLVSATMSGVTTCLR</td></tr>
<tr><td>></td><td>sp|P85108|TBE Tubulin beta-2A chain OS=Rattus norvegicus GN=Tubb2a PE=1 SV=1</td><td>LTTPTYGDLNHLVSATMSGVTTCLR</td></tr>
<tr><td>></td><td>sp|P85108|TBE Tubulin beta-2A chain OS=Rattus norvegicus GN=Tubb2a PE=1 SV=1</td><td>LTTPTYGDLNHLVSATMSGVTTCLR</td></tr>
<tr><td>></td><td>sp|P85108|TBE Tubulin beta-2A chain OS=Rattus norvegicus GN=Tubb2a PE=1 SV=1</td><td>LTTPTYGDLNHLVSATMSGVTTCLR</td></tr>
<tr><td>></td><td>sp|P85108|TBE Tubulin beta-2A chain OS=Rattus norvegicus GN=Tubb2a PE=1 SV=1</td><td>LTTPTYGDLNHLVSATMSGVTTCLR</td></tr>
<tr><td>></td><td>sp|P85108|TBE Tubulin beta-2A chain OS=Rattus norvegicus GN=Tubb2a PE=1 SV=1</td><td>LTTPTYGDLNHLVSATMSGVTTCLR</td></tr>
<tr><td>></td><td>sp|P85108|TBE Tubulin beta-2A chain OS=Rattus norvegicus GN=Tubb2a PE=1 SV=1</td><td>MSMKEVDEQMLNVQNK</td></tr>
<tr><td>></td><td>sp|P85108|TBE Tubulin beta-2A chain OS=Rattus norvegicus GN=Tubb2a PE=1 SV=1</td><td>NSSYFVEWIPNNVK</td></tr>
<tr><td>></td><td>sp|P85108|TBE Tubulin beta-2A chain OS=Rattus norvegicus GN=Tubb2a PE=1 SV=1</td><td>NSSYFVEWIPNNVK</td></tr>
</table>

<table>
<tr><td>></td><td>sp|P85108|TBE Tubulin beta-2A chain OS=Rattus norvegicus GN=Tubb2a PE=1 SV=1</td><td>NSSYFVEWIPNNVK</td></tr>
<tr><td>></td><td>sp|P85108|TBE Tubulin beta-2A chain OS=Rattus norvegicus GN=Tubb2a PE=1 SV=1</td><td>PGTMDSVR</td></tr>
<tr><td>></td><td>sp|P85108|TBE Tubulin beta-2A chain OS=Rattus norvegicus GN=Tubb2a PE=1 SV=1</td><td>SGPFGQIFRPDNFVFGQSGAGNNWAK</td></tr>
<tr><td>></td><td>sp|P85108|TBE Tubulin beta-2A chain OS=Rattus norvegicus GN=Tubb2a PE=1 SV=1</td><td>SGPFGQIFRPDNFVFGQSGAGNNWAK</td></tr>
<tr><td>></td><td>sp|P85108|TBE Tubulin beta-2A chain OS=Rattus norvegicus GN=Tubb2a PE=1 SV=1</td><td>SGPFGQIFRPDNFVFGQSGAGNNWAK</td></tr>
<tr><td>></td><td>sp|P85108|TBE Tubulin beta-2A chain OS=Rattus norvegicus GN=Tubb2a PE=1 SV=1</td><td>SGPFGQIFRPDNFVFGQSGAGNNWAK</td></tr>
<tr><td>></td><td>sp|P85108|TBE Tubulin beta-2A chain OS=Rattus norvegicus GN=Tubb2a PE=1 SV=1</td><td>SGPFGQIFRPDNFVFGQSGAGNNWAK</td></tr>
<tr><td>></td><td>sp|P85108|TBE Tubulin beta-2A chain OS=Rattus norvegicus GN=Tubb2a PE=1 SV=1</td><td>SGPFGQIFRPDNFVFGQSGAGNNWAK</td></tr>
<tr><td>></td><td>sp|P85108|TBE Tubulin beta-2A chain OS=Rattus norvegicus GN=Tubb2a PE=1 SV=1</td><td>SGPFGQIFRPDNFVFGQSGAGNNWAK</td></tr>
<tr><td>></td><td>sp|P85108|TBE Tubulin beta-2A chain OS=Rattus norvegicus GN=Tubb2a PE=1 SV=1</td><td>SGPFGQIFRPDNFVFGQSGAGNNWAK</td></tr>
<tr><td>></td><td>sp|P85108|TBE Tubulin beta-2A chain OS=Rattus norvegicus GN=Tubb2a PE=1 SV=1</td><td>SGPFGQIFRPDNFVFGQSGAGNNWAK</td></tr>
<tr><td>></td><td>sp|P85108|TBE Tubulin beta-2A chain OS=Rattus norvegicus GN=Tubb2a PE=1 SV=1</td><td>SGPFGQIFRPDNFVFGQSGAGNNWAK</td></tr>
<tr><td>></td><td>sp|P85108|TBE Tubulin beta-2A chain OS=Rattus norvegicus GN=Tubb2a PE=1 SV=1</td><td>SGPFGQIFRPDNFVFGQSGAGNNWAK</td></tr>
<tr><td>></td><td>sp|P85108|TBE Tubulin beta-2A chain OS=Rattus norvegicus GN=Tubb2a PE=1 SV=1</td><td>SGPFGQIFRPDNFVFGQSGAGNNWAK</td></tr>
<tr><td>></td><td>sp|P85108|TBE Tubulin beta-2A chain OS=Rattus norvegicus GN=Tubb2a PE=1 SV=1</td><td>SGPFGQIFRPDNFVFGQSGAGNNWAK</td></tr>
<tr><td>></td><td>sp|P85108|TBE Tubulin beta-2A chain OS=Rattus norvegicus GN=Tubb2a PE=1 SV=1</td><td>SGPFGQIFRPDNFVFGQSGAGNNWAK</td></tr>
<tr><td>></td><td>sp|P85108|TBE Tubulin beta-2A chain OS=Rattus norvegicus GN=Tubb2a PE=1 SV=1</td><td>SGPFGQIFRPDNFVFGQSGAGNNWAK</td></tr>
<tr><td>></td><td>sp|P85108|TBE Tubulin beta-2A chain OS=Rattus norvegicus GN=Tubb2a PE=1 SV=1</td><td>SGPFGQIFRPDNFVFGQSGAGNNWAK</td></tr>
<tr><td>></td><td>sp|P85108|TBE Tubulin beta-2A chain OS=Rattus norvegicus GN=Tubb2a PE=1 SV=1</td><td>TAVCDIPPR</td></tr>
<tr><td>></td><td>sp|P85108|TBE Tubulin beta-2A chain OS=Rattus norvegicus GN=Tubb2a PE=1 SV=1</td><td>TAVCDIPPR</td></tr>
<tr><td>></td><td>sp|P85108|TBE Tubulin beta-2A chain OS=Rattus norvegicus GN=Tubb2a PE=1 SV=1</td><td>TAVCDIPPR</td></tr>
<tr><td>></td><td>sp|P85108|TBE Tubulin beta-2A chain OS=Rattus norvegicus GN=Tubb2a PE=1 SV=1</td><td>TAVCDIPPR</td></tr>
<tr><td>></td><td>sp|P85108|TBE Tubulin beta-2A chain OS=Rattus norvegicus GN=Tubb2a PE=1 SV=1</td><td>TAVCDIPPR</td></tr>
<tr><td>></td><td>sp|P85108|TBE Tubulin beta-2A chain OS=Rattus norvegicus GN=Tubb2a PE=1 SV=1</td><td>TAVCDIPPR</td></tr>
<tr><td>></td><td>sp|P85108|TBE Tubulin beta-2A chain OS=Rattus norvegicus GN=Tubb2a PE=1 SV=1</td><td>TAVCDIPPR</td></tr>
<tr><td>></td><td>sp|P85108|TBE Tubulin beta-2A chain OS=Rattus norvegicus GN=Tubb2a PE=1 SV=1</td><td>TAVCDIPPR</td></tr>
<tr><td>></td><td>sp|P85108|TBE Tubulin beta-2A chain OS=Rattus norvegicus GN=Tubb2a PE=1 SV=1</td><td>VSDTVVEPYNATLSVHQLVENTDETYSIDNEALYDICFR</td></tr>
<tr><td>></td><td>sp|P85108|TBE Tubulin beta-2A chain OS=Rattus norvegicus GN=Tubb2a PE=1 SV=1</td><td>VSDTVVEPYNATLSVHQLVENTDETYSIDNEALYDICFR</td></tr>
<tr><td>></td><td>sp|P85108|TBE Tubulin beta-2A chain OS=Rattus norvegicus GN=Tubb2a PE=1 SV=1</td><td>VSDTVVEPYNATLSVHQLVENTDETYSIDNEALYDICFR</td></tr>
<tr><td>></td><td>sp|P85108|TBE Tubulin beta-2A chain OS=Rattus norvegicus GN=Tubb2a PE=1 SV=1</td><td>VSDTVVEPYNATLSVHQLVENTDETYSIDNEALYDICFR</td></tr>
<tr><td>></td><td>sp|P85108|TBE Tubulin beta-2A chain OS=Rattus norvegicus GN=Tubb2a PE=1 SV=1</td><td>VSDTVVEPYNATLSVHQLVENTDETYSIDNEALYDICFR</td></tr>
<tr><td>></td><td>sp|P85108|TBE Tubulin beta-2A chain OS=Rattus norvegicus GN=Tubb2a PE=1 SV=1</td><td>VSDTVVEPYNATLSVHQLVENTDETYSIDNEALYDICFR</td></tr>
<tr><td>></td><td>sp|P85108|TBE Tubulin beta-2A chain OS=Rattus norvegicus GN=Tubb2a PE=1 SV=1</td><td>VSDTVVEPYNATLSVHQLVENTDETYSIDNEALYDICFR</td></tr>
<tr><td>></td><td>sp|P85108|TBE Tubulin beta-2A chain OS=Rattus norvegicus GN=Tubb2a PE=1 SV=1</td><td>VSDTVVEPYNATLSVHQLVENTDETYSIDNEALYDICFR</td></tr>
<tr><td>></td><td>sp|P85108|TBE Tubulin beta-2A chain OS=Rattus norvegicus GN=Tubb2a PE=1 SV=1</td><td>VSDTVVEPYNATLSVHQLVENTDETYSIDNEALYDICFR</td></tr>
<tr><td>></td><td>sp|P85108|TBE Tubulin beta-2A chain OS=Rattus norvegicus GN=Tubb2a PE=1 SV=1</td><td>VSDTVVEPYNATLSVHQLVENTDETYSIDNEALYDICFR</td></tr>
<tr><td>></td><td>sp|P85108|TBE Tubulin beta-2A chain OS=Rattus norvegicus GN=Tubb2a PE=1 SV=1</td><td>VSDTVVEPYNATLSVHQLVENTDETYSIDNEALYDICFR</td></tr>
<tr><td>></td><td>sp|P85108|TBE Tubulin beta-2A chain OS=Rattus norvegicus GN=Tubb2a PE=1 SV=1</td><td>VSDTVVEPYNATLSVHQLVENTDETYSIDNEALYDICFR</td></tr>
<tr><td>></td><td>sp|P85108|TBE Tubulin beta-2A chain OS=Rattus norvegicus GN=Tubb2a PE=1 SV=1</td><td>VSDTVVEPYNATLSVHQLVENTDETYSIDNEALYDICFR</td></tr>
<tr><td>></td><td>sp|P85108|TBE Tubulin beta-2A chain OS=Rattus norvegicus GN=Tubb2a PE=1 SV=1</td><td>VSDTVVEPYNATLSVHQLVENTDETYSIDNEALYDICFR</td></tr>
<tr><td>></td><td>sp|P85108|TBE Tubulin beta-2A chain OS=Rattus norvegicus GN=Tubb2a PE=1 SV=1</td><td>VSDTVVEPYNATLSVHQLVENTDETYSIDNEALYDICFR</td></tr>
<tr><td>></td><td>sp|P85108|TBE Tubulin beta-2A chain OS=Rattus norvegicus GN=Tubb2a PE=1 SV=1</td><td>VSDTVVEPYNATLSVHQLVENTDETYSIDNEALYDICFR</td></tr>
<tr><td>></td><td>sp|P85108|TBE Tubulin beta-2A chain OS=Rattus norvegicus GN=Tubb2a PE=1 SV=1</td><td>VSDTVVEPYNATLSVHQLVENTDETYSIDNEALYDICFR</td></tr>
<tr><td>></td><td>sp|P85108|TBE Tubulin beta-2A chain OS=Rattus norvegicus GN=Tubb2a PE=1 SV=1</td><td>VSDTVVEPYNATLSVHQLVENTDETYSIDNEALYDICFR</td></tr>
<tr><td>></td><td>sp|P85108|TBE Tubulin beta-2A chain OS=Rattus norvegicus GN=Tubb2a PE=1 SV=1</td><td>VSDTVVEPYNATLSVHQLVENTDETYSIDNEALYDICFR</td></tr>
<tr><td>></td><td>sp|P85108|TBE Tubulin beta-2A chain OS=Rattus norvegicus GN=Tubb2a PE=1 SV=1</td><td>VSDTVVEPYNATLSVHQLVENTDETYSIDNEALYDICFR</td></tr>
<tr><td>></td><td>sp|P85108|TBE Tubulin beta-2A chain OS=Rattus norvegicus GN=Tubb2a PE=1 SV=1</td><td>VSDTVVEPYNATLSVHQLVENTDETYSIDNEALYDICFR</td></tr>
<tr><td>></td><td>sp|P85108|TBE Tubulin beta-2A chain OS=Rattus norvegicus GN=Tubb2a PE=1 SV=1</td><td>VSDTVVEPYNATLSVHQLVENTDETYSIDNEALYDICFR</td></tr>
<tr><td>></td><td>sp|P85108|TBE Tubulin beta-2A chain OS=Rattus norvegicus GN=Tubb2a PE=1 SV=1</td><td>VSDTVVEPYNATLSVHQLVENTDETYSIDNEALYDICFR</td></tr>
<tr><td>></td><td>sp|P85108|TBE Tubulin beta-2A chain OS=Rattus norvegicus GN=Tubb2a PE=1 SV=1</td><td>VSDTVVEPYNATLSVHQLVENTDETYSIDNEALYDICFR</td></tr>
<tr><td>></td><td>sp|P85108|TBE Tubulin beta-2A chain OS=Rattus norvegicus GN=Tubb2a PE=1 SV=1</td><td>VSDTVVEPYNATLSVHQLVENTDETYSIDNEALYDICFR</td></tr>
<tr><td>></td><td>sp|P85108|TBE Tubulin beta-2A chain OS=Rattus norvegicus GN=Tubb2a PE=1 SV=1</td><td>VSDTVVEPYNATLSVHQLVENTDETYSIDNEALYDICFR</td></tr>
<tr><td>></td><td>sp|P85108|TBE Tubulin beta-2A chain OS=Rattus norvegicus GN=Tubb2a PE=1 SV=1</td><td>VSDTVVEPYNATLSVHQLVENTDETYSIDNEALYDICFR</td></tr>
<tr><td>></td><td>sp|P85108|TBE Tubulin beta-2A chain OS=Rattus norvegicus GN=Tubb2a PE=1 SV=1</td><td>VSDTVVEPYNATLSVHQLVENTDETYSIDNEALYDICFR</td></tr>
<tr><td>></td><td>sp|P85108|TBE Tubulin beta-2A chain OS=Rattus norvegicus GN=Tubb2a PE=1 SV=1</td><td>IMNTFSVMPSPK</td></tr>
<tr><td>></td><td>sp|P85108|TBE Tubulin beta-2A chain OS=Rattus norvegicus GN=Tubb2a PE=1 SV=1</td><td>TAVCDIPPR</td></tr>
<tr><td>></td><td>sp|P85108|TBE Tubulin beta-2A chain OS=Rattus norvegicus GN=Tubb2a PE=1 SV=1</td><td>NSSYFVEWIPNNVK</td></tr>
<tr><td>></td><td>sp|P85108|TBE Tubulin beta-2A chain OS=Rattus norvegicus GN=Tubb2a PE=1 SV=1</td><td>YLTVAAIFR</td></tr>
<tr><td>></td><td>sp|P85108|TBE Tubulin beta-2A chain OS=Rattus norvegicus GN=Tubb2a PE=1 SV=1</td><td>EIVHIQAGQCGNQIGAK</td></tr>
<tr><td>></td><td>sp|P85108|TBE Tubulin beta-2A chain OS=Rattus norvegicus GN=Tubb2a PE=1 SV=1</td><td>TAVCDIPPR</td></tr>
<tr><td>></td><td>sp|P85108|TBE Tubulin beta-2A chain OS=Rattus norvegicus GN=Tubb2a PE=1 SV=1</td><td>EIVHIQAGQCGNQIGAK</td></tr>
<tr><td>></td><td>sp|P85108|TBE Tubulin beta-2A chain OS=Rattus norvegicus GN=Tubb2a PE=1 SV=1</td><td>LHFFMPGFAPLTSR</td></tr>
<tr><td>></td><td>sp|P85108|TBE Tubulin beta-2A chain OS=Rattus norvegicus GN=Tubb2a PE=1 SV=1</td><td>VSDTVVEPYNATLSVHQLVENTDETYSIDNEALYDICFR</td></tr>
<tr><td>></td><td>sp|P85108|TBE Tubulin beta-2A chain OS=Rattus norvegicus GN=Tubb2a PE=1 SV=1</td><td>TAVCDIPPR</td></tr>
<tr><td>></td><td>sp|P85108|TBE Tubulin beta-2A chain OS=Rattus norvegicus GN=Tubb2a PE=1 SV=1</td><td>VSDTVVEPYNATLSVHQLVENTDETYSIDNEALYDICFR</td></tr>
<tr><td>></td><td>sp|P85108|TBE Tubulin beta-2A chain OS=Rattus norvegicus GN=Tubb2a PE=1 SV=1</td><td>FPGQLNADLR</td></tr>
<tr><td>></td><td>sp|P85108|TBE Tubulin beta-2A chain OS=Rattus norvegicus GN=Tubb2a PE=1 SV=1</td><td>KLAVNMVPFPR</td></tr>
<tr><td>></td><td>sp|P85108|TBE Tubulin beta-2A chain OS=Rattus norvegicus GN=Tubb2a PE=1 SV=1</td><td>NSSYFVEWIPNNVK</td></tr>
<tr><td>></td><td>sp|P85108|TBE Tubulin beta-2A chain OS=Rattus norvegicus GN=Tubb2a PE=1 SV=1</td><td>NSSYFVEWIPNNVK</td></tr>
<tr><td>></td><td>sp|P85108|TBE Tubulin beta-2A chain OS=Rattus norvegicus GN=Tubb2a PE=1 SV=1</td><td>VSDTVVEPYNATLSVHQLVENTDETYSIDNEALYDICFR</td></tr>
<tr><td>></td><td>sp|P85108|TBE Tubulin beta-2A chain OS=Rattus norvegicus GN=Tubb2a PE=1 SV=1</td><td>LHFFMPGFAPLTSR</td></tr>
<tr><td>></td><td>sp|P85108|TBE Tubulin beta-2A chain OS=Rattus norvegicus GN=Tubb2a PE=1 SV=1</td><td>NSSYFVEWIPNNVK</td></tr>
<tr><td>></td><td>sp|P85108|TBE Tubulin beta-2A chain OS=Rattus norvegicus GN=Tubb2a PE=1 SV=1</td><td>YLTVAAIFR</td></tr>
</table>

<table>
<tr><td>></td><td>sp|P85108|TBE Tubulin beta-2A chain OS=Rattus norvegicus GN=Tubb2a PE=1 SV=1</td><td>LHFFMPGFAPLTSR</td></tr>
<tr><td>></td><td>sp|P85108|TBE Tubulin beta-2A chain OS=Rattus norvegicus GN=Tubb2a PE=1 SV=1</td><td>GHYTEGAELVDSVLDVVR</td></tr>
<tr><td>></td><td>sp|P85108|TBE Tubulin beta-2A chain OS=Rattus norvegicus GN=Tubb2a PE=1 SV=1</td><td>LHFFMPGFAPLTSR</td></tr>
<tr><td>></td><td>sp|P85108|TBE Tubulin beta-2A chain OS=Rattus norvegicus GN=Tubb2a PE=1 SV=1</td><td>ALTVPELTQQMFDSK</td></tr>
<tr><td>></td><td>sp|P85108|TBE Tubulin beta-2A chain OS=Rattus norvegicus GN=Tubb2a PE=1 SV=1</td><td>EIVHIQAGQCGNQIGAK</td></tr>
<tr><td>></td><td>sp|P85108|TBE Tubulin beta-2A chain OS=Rattus norvegicus GN=Tubb2a PE=1 SV=1</td><td>GHYTEGAELVDSVLDVVR</td></tr>
<tr><td>></td><td>sp|P85108|TBE Tubulin beta-2A chain OS=Rattus norvegicus GN=Tubb2a PE=1 SV=1</td><td>GHYTEGAELVDSVLDVVR</td></tr>
<tr><td>></td><td>sp|P85108|TBE Tubulin beta-2A chain OS=Rattus norvegicus GN=Tubb2a PE=1 SV=1</td><td>GHYTEGAELVDSVLDVVR</td></tr>
<tr><td>></td><td>sp|P85108|TBE Tubulin beta-2A chain OS=Rattus norvegicus GN=Tubb2a PE=1 SV=1</td><td>AILVDLEPGTMDSVR</td></tr>
<tr><td>></td><td>sp|P85108|TBE Tubulin beta-2A chain OS=Rattus norvegicus GN=Tubb2a PE=1 SV=1</td><td>VSDTVVEPYNATLSVHQLVENTDETYSIDNEALYDICFR</td></tr>
<tr><td>></td><td>sp|P85108|TBE Tubulin beta-2A chain OS=Rattus norvegicus GN=Tubb2a PE=1 SV=1</td><td>VSDTVVEPYNATLSVHQLVENTDETYSIDNEALYDICFR</td></tr>
<tr><td>></td><td>sp|P85108|TBE Tubulin beta-2A chain OS=Rattus norvegicus GN=Tubb2a PE=1 SV=1</td><td>EIVHIQAGQCGNQIGAK</td></tr>
<tr><td>></td><td>sp|P85108|TBE Tubulin beta-2A chain OS=Rattus norvegicus GN=Tubb2a PE=1 SV=1</td><td>EVDEQMLNVQNK</td></tr>
<tr><td>></td><td>sp|P85108|TBE Tubulin beta-2A chain OS=Rattus norvegicus GN=Tubb2a PE=1 SV=1</td><td>KLAVNMVPFPR</td></tr>
<tr><td>></td><td>sp|P85108|TBE Tubulin beta-2A chain OS=Rattus norvegicus GN=Tubb2a PE=1 SV=1</td><td>AILVDLEPGTMDSVR</td></tr>
<tr><td>></td><td>sp|P85108|TBE Tubulin beta-2A chain OS=Rattus norvegicus GN=Tubb2a PE=1 SV=1</td><td>EIVHIQAGQCGNQIGAK</td></tr>
<tr><td>></td><td>sp|P85108|TBE Tubulin beta-2A chain OS=Rattus norvegicus GN=Tubb2a PE=1 SV=1</td><td>FWEVISDEHGIDPTGSYHGDSDLQLER</td></tr>
<tr><td>></td><td>sp|P85108|TBE Tubulin beta-2A chain OS=Rattus norvegicus GN=Tubb2a PE=1 SV=1</td><td>EIVHIQAGQCGNQIGAK</td></tr>
<tr><td>></td><td>sp|P85108|TBE Tubulin beta-2A chain OS=Rattus norvegicus GN=Tubb2a PE=1 SV=1</td><td>LHFFMPGFAPLTSR</td></tr>
<tr><td>></td><td>sp|P85108|TBE Tubulin beta-2A chain OS=Rattus norvegicus GN=Tubb2a PE=1 SV=1</td><td>LAVNMVPFPR</td></tr>
<tr><td>></td><td>sp|P85108|TBE Tubulin beta-2A chain OS=Rattus norvegicus GN=Tubb2a PE=1 SV=1</td><td>KLAVNMVPFPR</td></tr>
<tr><td>></td><td>sp|P85108|TBE Tubulin beta-2A chain OS=Rattus norvegicus GN=Tubb2a PE=1 SV=1</td><td>PGTMDSVR</td></tr>
<tr><td>></td><td>sp|P85108|TBE Tubulin beta-2A chain OS=Rattus norvegicus GN=Tubb2a PE=1 SV=1</td><td>GHYTEGAELVDSVLDVVR</td></tr>
<tr><td>></td><td>sp|P85108|TBE Tubulin beta-2A chain OS=Rattus norvegicus GN=Tubb2a PE=1 SV=1</td><td>LTTPTYGDLNHLVSATMSGVTTCLR</td></tr>
<tr><td>></td><td>sp|P85108|TBE Tubulin beta-2A chain OS=Rattus norvegicus GN=Tubb2a PE=1 SV=1</td><td>AILVDLEPGTMDSVR</td></tr>
<tr><td>></td><td>sp|P85108|TBE Tubulin beta-2A chain OS=Rattus norvegicus GN=Tubb2a PE=1 SV=1</td><td>AILVDLEPGTMDSVR</td></tr>
<tr><td>></td><td>sp|P85108|TBE Tubulin beta-2A chain OS=Rattus norvegicus GN=Tubb2a PE=1 SV=1</td><td>SGPFGQIFRPDNFVFGQSGAGNNWAK</td></tr>
<tr><td>></td><td>sp|P85108|TBE Tubulin beta-2A chain OS=Rattus norvegicus GN=Tubb2a PE=1 SV=1</td><td>SGPFGQIFRPDNFVFGQSGAGNNWAK</td></tr>
<tr><td>></td><td>sp|P85108|TBE Tubulin beta-2A chain OS=Rattus norvegicus GN=Tubb2a PE=1 SV=1</td><td>EIVHIQAGQCGNQIGAK</td></tr>
<tr><td>></td><td>sp|P85108|TBE Tubulin beta-2A chain OS=Rattus norvegicus GN=Tubb2a PE=1 SV=1</td><td>KLAVNMVPFPR</td></tr>
<tr><td>></td><td>sp|P85108|TBE Tubulin beta-2A chain OS=Rattus norvegicus GN=Tubb2a PE=1 SV=1</td><td>LHFFMPGFAPLTSR</td></tr>
<tr><td>></td><td>sp|P85108|TBE Tubulin beta-2A chain OS=Rattus norvegicus GN=Tubb2a PE=1 SV=1</td><td>KLAVNMVPFPRLHFFMPGFAPLTSR</td></tr>
<tr><td>></td><td>sp|P85108|TBE Tubulin beta-2A chain OS=Rattus norvegicus GN=Tubb2a PE=1 SV=1</td><td>FPGQLNADLRK</td></tr>
<tr><td>></td><td>sp|P85108|TBE Tubulin beta-2A chain OS=Rattus norvegicus GN=Tubb2a PE=1 SV=1</td><td>VSDTVVEPYNATLSVHQLVENTDETYSIDNEALYDICFR</td></tr>
<tr><td>></td><td>sp|P85108|TBE Tubulin beta-2A chain OS=Rattus norvegicus GN=Tubb2a PE=1 SV=1</td><td>KLAVNMVPFPR</td></tr>
<tr><td>></td><td>sp|P85108|TBE Tubulin beta-2A chain OS=Rattus norvegicus GN=Tubb2a PE=1 SV=1</td><td>KLAVNMVPFPR</td></tr>
<tr><td>></td><td>sp|P85108|TBE Tubulin beta-2A chain OS=Rattus norvegicus GN=Tubb2a PE=1 SV=1</td><td>LAVNMVPFPR</td></tr>
<tr><td>></td><td>sp|P85108|TBE Tubulin beta-2A chain OS=Rattus norvegicus GN=Tubb2a PE=1 SV=1</td><td>KLAVNMVPFPR</td></tr>
<tr><td>></td><td>sp|P85108|TBE Tubulin beta-2A chain OS=Rattus norvegicus GN=Tubb2a PE=1 SV=1</td><td>KLAVNMVPFPR</td></tr>
<tr><td>></td><td>sp|P85108|TBE Tubulin beta-2A chain OS=Rattus norvegicus GN=Tubb2a PE=1 SV=1</td><td>GHYTEGAELVDSVLDVVR</td></tr>
<tr><td>></td><td>sp|P85108|TBE Tubulin beta-2A chain OS=Rattus norvegicus GN=Tubb2a PE=1 SV=1</td><td>KESESCDCLQGFQLTHSLGGGTGSGMGTLLISK</td></tr>
<tr><td>></td><td>sp|P85108|TBE Tubulin beta-2A chain OS=Rattus norvegicus GN=Tubb2a PE=1 SV=1</td><td>YLTVAAIFR</td></tr>
<tr><td>></td><td>sp|P85108|TBE Tubulin beta-2A chain OS=Rattus norvegicus GN=Tubb2a PE=1 SV=1</td><td>LTTPTYGDLNHLVSATMSGVTTCLR</td></tr>
<tr><td>></td><td>sp|P85108|TBE Tubulin beta-2A chain OS=Rattus norvegicus GN=Tubb2a PE=1 SV=1</td><td>KLAVNMVPFPR</td></tr>
<tr><td>></td><td>sp|P85108|TBE Tubulin beta-2A chain OS=Rattus norvegicus GN=Tubb2a PE=1 SV=1</td><td>LAVNMVPFPR</td></tr>
<tr><td>></td><td>sp|P85108|TBE Tubulin beta-2A chain OS=Rattus norvegicus GN=Tubb2a PE=1 SV=1</td><td>SGPFGQIFRPDNFVFGQSGAGNNWAK</td></tr>
<tr><td>></td><td>sp|P85108|TBE Tubulin beta-2A chain OS=Rattus norvegicus GN=Tubb2a PE=1 SV=1</td><td>LHFFMPGFAPLTSR</td></tr>
<tr><td>></td><td>sp|P85108|TBE Tubulin beta-2A chain OS=Rattus norvegicus GN=Tubb2a PE=1 SV=1</td><td>GHYTEGAELVDSVLDVVR</td></tr>
<tr><td>></td><td>sp|P85108|TBE Tubulin beta-2A chain OS=Rattus norvegicus GN=Tubb2a PE=1 SV=1</td><td>VSDTVVEPYNATLSVHQLVENTDETYSIDNEALYDICFR</td></tr>
<tr><td>></td><td>sp|P85108|TBE Tubulin beta-2A chain OS=Rattus norvegicus GN=Tubb2a PE=1 SV=1</td><td>EIVHIQAGQCGNQIGAK</td></tr>
<tr><td>></td><td>sp|P85108|TBE Tubulin beta-2A chain OS=Rattus norvegicus GN=Tubb2a PE=1 SV=1</td><td>KLAVNMVPFPR</td></tr>
<tr><td>></td><td>sp|P85108|TBE Tubulin beta-2A chain OS=Rattus norvegicus GN=Tubb2a PE=1 SV=1</td><td>KLAVNMVPFPRLHFFMPGFAPLTSR</td></tr>
<tr><td>></td><td>sp|P85108|TBE Tubulin beta-2A chain OS=Rattus norvegicus GN=Tubb2a PE=1 SV=1</td><td>KESESCDCLQGFQLTHSLGGGTGSGMGTLLISK</td></tr>
<tr><td>></td><td>sp|P85108|TBE Tubulin beta-2A chain OS=Rattus norvegicus GN=Tubb2a PE=1 SV=1</td><td>ALTVPELTQQMFDSK</td></tr>
<tr><td>></td><td>sp|P85108|TBE Tubulin beta-2A chain OS=Rattus norvegicus GN=Tubb2a PE=1 SV=1</td><td>TAVCDIPPR</td></tr>
<tr><td>></td><td>sp|P85108|TBE Tubulin beta-2A chain OS=Rattus norvegicus GN=Tubb2a PE=1 SV=1</td><td>EIVHIQAGQCGNQIGAK</td></tr>
<tr><td>></td><td>sp|P85108|TBE Tubulin beta-2A chain OS=Rattus norvegicus GN=Tubb2a PE=1 SV=1</td><td>LHFFMPGFAPLTSR</td></tr>
<tr><td>></td><td>sp|P85108|TBE Tubulin beta-2A chain OS=Rattus norvegicus GN=Tubb2a PE=1 SV=1</td><td>FPGQLNADLR</td></tr>
<tr><td>></td><td>sp|P85108|TBE Tubulin beta-2A chain OS=Rattus norvegicus GN=Tubb2a PE=1 SV=1</td><td>AILVDLE</td></tr>
<tr><td>></td><td>sp|P85108|TBE Tubulin beta-2A chain OS=Rattus norvegicus GN=Tubb2a PE=1 SV=1</td><td>YLTVAAIFR</td></tr>
<tr><td>></td><td>sp|P85108|TBE Tubulin beta-2A chain OS=Rattus norvegicus GN=Tubb2a PE=1 SV=1</td><td>LTTPTYGDLNHLVSATMSGVTTCLR</td></tr>
<tr><td>></td><td>sp|P85108|TBE Tubulin beta-2A chain OS=Rattus norvegicus GN=Tubb2a PE=1 SV=1</td><td>KLAVNMVPFPR</td></tr>
<tr><td>></td><td>sp|P85108|TBE Tubulin beta-2A chain OS=Rattus norvegicus GN=Tubb2a PE=1 SV=1</td><td>EIVHIQAGQCGNQIGAK</td></tr>
<tr><td>></td><td>sp|P85108|TBE Tubulin beta-2A chain OS=Rattus norvegicus GN=Tubb2a PE=1 SV=1</td><td>NSSYFVEWIPNNVK</td></tr>
<tr><td>></td><td>sp|P85108|TBE Tubulin beta-2A chain OS=Rattus norvegicus GN=Tubb2a PE=1 SV=1</td><td>VSDTVVEPYNATLSVHQLVENTDETYSIDNEALYDICFR</td></tr>
<tr><td>></td><td>sp|P85108|TBE Tubulin beta-2A chain OS=Rattus norvegicus GN=Tubb2a PE=1 SV=1</td><td>NSSYFVEWIPNNVK</td></tr>
<tr><td>></td><td>sp|P85108|TBE Tubulin beta-2A chain OS=Rattus norvegicus GN=Tubb2a PE=1 SV=1</td><td>NMMAACDPR</td></tr>
<tr><td>></td><td>sp|P85108|TBE Tubulin beta-2A chain OS=Rattus norvegicus GN=Tubb2a PE=1 SV=1</td><td>VSDTVVEPYNATLSVHQLVENTDETYSIDNEALYDICFR</td></tr>
<tr><td>></td><td>sp|P85108|TBE Tubulin beta-2A chain OS=Rattus norvegicus GN=Tubb2a PE=1 SV=1</td><td>PELTQQMFDSK</td></tr>
<tr><td>></td><td>sp|P85108|TBE Tubulin beta-2A chain OS=Rattus norvegicus GN=Tubb2a PE=1 SV=1</td><td>VSDTVVEPYNATLSVHQLVENTDETYSIDNEALYDICFR</td></tr>
<tr><td>></td><td>sp|P85108|TBE Tubulin beta-2A chain OS=Rattus norvegicus GN=Tubb2a PE=1 SV=1</td><td>NSSYFVEWIPNNVK</td></tr>
<tr><td>></td><td>sp|P85108|TBE Tubulin beta-2A chain OS=Rattus norvegicus GN=Tubb2a PE=1 SV=1</td><td>KESESCDCLQGFQLTHSLGGGTGSGMGTLLISK</td></tr>
<tr><td>></td><td>sp|P85108|TBE Tubulin beta-2A chain OS=Rattus norvegicus GN=Tubb2a PE=1 SV=1</td><td>KLAVNMVPFPR</td></tr>
<tr><td>></td><td>sp|P85108|TBE Tubulin beta-2A chain OS=Rattus norvegicus GN=Tubb2a PE=1 SV=1</td><td>ISEQFTAMFR</td></tr>
</table>

<table>
<tr><td>></td><td>sp|P85108|TBE Tubulin beta-2A chain OS=Rattus norvegicus GN=Tubb2a PE=1 SV=1</td><td>LTTPTYGDLNHLVSATMSGVTTCLR</td></tr>
<tr><td>></td><td>sp|P85108|TBE Tubulin beta-2A chain OS=Rattus norvegicus GN=Tubb2a PE=1 SV=1</td><td>SVMPSPKVSDTVVEPYNATLSVHQLVENTDETYSIDNEALYDICFR</td></tr>
<tr><td>></td><td>sp|P85108|TBE Tubulin beta-2A chain OS=Rattus norvegicus GN=Tubb2a PE=1 SV=1</td><td>VSDTVVEPYNATLSVHQLVENTDETYSIDNEALYDICFR</td></tr>
<tr><td>></td><td>sp|P85108|TBE Tubulin beta-2A chain OS=Rattus norvegicus GN=Tubb2a PE=1 SV=1</td><td>TAVCDIPPR</td></tr>
<tr><td>></td><td>sp|P85108|TBE Tubulin beta-2A chain OS=Rattus norvegicus GN=Tubb2a PE=1 SV=1</td><td>SGPFGQIFRPDNFVFGQSGAGNNWAK</td></tr>
<tr><td>></td><td>sp|P85108|TBE Tubulin beta-2A chain OS=Rattus norvegicus GN=Tubb2a PE=1 SV=1</td><td>EIVHIQAGQCGNQIGAK</td></tr>
<tr><td>></td><td>sp|P85108|TBE Tubulin beta-2A chain OS=Rattus norvegicus GN=Tubb2a PE=1 SV=1</td><td>FPGQLNADLR</td></tr>
<tr><td>></td><td>sp|P85108|TBE Tubulin beta-2A chain OS=Rattus norvegicus GN=Tubb2a PE=1 SV=1</td><td>KLAVNMVPFPR</td></tr>
<tr><td>></td><td>sp|P85108|TBE Tubulin beta-2A chain OS=Rattus norvegicus GN=Tubb2a PE=1 SV=1</td><td>VSDTVVEPYNATLSVHQLVENTDETYSIDNEALYDICFR</td></tr>
<tr><td>></td><td>sp|P85108|TBE Tubulin beta-2A chain OS=Rattus norvegicus GN=Tubb2a PE=1 SV=1</td><td>FWEVISDEHGIDPTGSYHGDSDLQLER</td></tr>
<tr><td>></td><td>sp|P85108|TBE Tubulin beta-2A chain OS=Rattus norvegicus GN=Tubb2a PE=1 SV=1</td><td>LTTPTYGDLNHLVSATMSGVTTCLR</td></tr>
<tr><td>></td><td>sp|P85108|TBE Tubulin beta-2A chain OS=Rattus norvegicus GN=Tubb2a PE=1 SV=1</td><td>TAVCDIPPR</td></tr>
<tr><td>></td><td>sp|P85108|TBE Tubulin beta-2A chain OS=Rattus norvegicus GN=Tubb2a PE=1 SV=1</td><td>EIVHIQAGQCGNQIGAK</td></tr>
<tr><td>></td><td>sp|P85108|TBE Tubulin beta-2A chain OS=Rattus norvegicus GN=Tubb2a PE=1 SV=1</td><td>VSDTVVEPYNATLSVHQLVENTDETYSIDNEALYDICFR</td></tr>
<tr><td>></td><td>sp|P85108|TBE Tubulin beta-2A chain OS=Rattus norvegicus GN=Tubb2a PE=1 SV=1</td><td>LTTPTYGDLNHLVSATMSGVTTCLR</td></tr>
<tr><td>></td><td>sp|P85108|TBE Tubulin beta-2A chain OS=Rattus norvegicus GN=Tubb2a PE=1 SV=1</td><td>FWEVISDEHGIDPTGSYHGDSDLQLER</td></tr>
<tr><td>></td><td>sp|P85108|TBE Tubulin beta-2A chain OS=Rattus norvegicus GN=Tubb2a PE=1 SV=1</td><td>LVENTDETYSIDNEALYDICFRTLKLTTPTYGDLNHLVSATMSGVTTCLR</td></tr>
<tr><td>></td><td>sp|P85108|TBE Tubulin beta-2A chain OS=Rattus norvegicus GN=Tubb2a PE=1 SV=1</td><td>EIVHIQAGQCGNQIGAK</td></tr>
<tr><td>></td><td>sp|P85108|TBE Tubulin beta-2A chain OS=Rattus norvegicus GN=Tubb2a PE=1 SV=1</td><td>KLAVNMVPFPR</td></tr>
<tr><td>></td><td>sp|P85108|TBE Tubulin beta-2A chain OS=Rattus norvegicus GN=Tubb2a PE=1 SV=1</td><td>EIVHIQAGQCGNQIGAK</td></tr>
<tr><td>></td><td>sp|P85108|TBE Tubulin beta-2A chain OS=Rattus norvegicus GN=Tubb2a PE=1 SV=1</td><td>EVDEQMLNVQNK</td></tr>
<tr><td>></td><td>sp|P85108|TBE Tubulin beta-2A chain OS=Rattus norvegicus GN=Tubb2a PE=1 SV=1</td><td>TAVCDIPPR</td></tr>
<tr><td>></td><td>sp|P85108|TBE Tubulin beta-2A chain OS=Rattus norvegicus GN=Tubb2a PE=1 SV=1</td><td>KESESCDCLQGFQLTHSLGGGTGSGMGTLLISKI</td></tr>
<tr><td>></td><td>sp|P85108|TBE Tubulin beta-2A chain OS=Rattus norvegicus GN=Tubb2a PE=1 SV=1</td><td>FWEVISDEHGIDPTGSYHGDSDLQLER</td></tr>
<tr><td>></td><td>sp|P85108|TBE Tubulin beta-2A chain OS=Rattus norvegicus GN=Tubb2a PE=1 SV=1</td><td>LTTPTYGDLNHLVSATMSGVTTCLR</td></tr>
<tr><td>></td><td>sp|P85108|TBE Tubulin beta-2A chain OS=Rattus norvegicus GN=Tubb2a PE=1 SV=1</td><td>VSDTVVEPYNATLSVHQLVENTDETYSIDNEALYDICFR</td></tr>
<tr><td>></td><td>sp|P85108|TBE Tubulin beta-2A chain OS=Rattus norvegicus GN=Tubb2a PE=1 SV=1</td><td>EIVHIQAGQCGNQIGAK</td></tr>
<tr><td>></td><td>sp|P85108|TBE Tubulin beta-2A chain OS=Rattus norvegicus GN=Tubb2a PE=1 SV=1</td><td>VSDTVVEPYNATLSVHQLVENTDETYSIDNEALYDICFR</td></tr>
<tr><td>></td><td>sp|P85108|TBE Tubulin beta-2A chain OS=Rattus norvegicus GN=Tubb2a PE=1 SV=1</td><td>VSDTVVEPYNATLSVHQLVENTDETYSIDNEALYDICFR</td></tr>
<tr><td>></td><td>sp|P85108|TBE Tubulin beta-2A chain OS=Rattus norvegicus GN=Tubb2a PE=1 SV=1</td><td>FPGQLNADLR</td></tr>
<tr><td>></td><td>sp|P85108|TBE Tubulin beta-2A chain OS=Rattus norvegicus GN=Tubb2a PE=1 SV=1</td><td>KLAVNMVPFPR</td></tr>
<tr><td>></td><td>sp|P85108|TBE Tubulin beta-2A chain OS=Rattus norvegicus GN=Tubb2a PE=1 SV=1</td><td>ALTVPELTQQMFDSK</td></tr>
<tr><td>></td><td>sp|P85108|TBE Tubulin beta-2A chain OS=Rattus norvegicus GN=Tubb2a PE=1 SV=1</td><td>MSMKEVDEQMLNVQNK</td></tr>
<tr><td>></td><td>sp|P85108|TBE Tubulin beta-2A chain OS=Rattus norvegicus GN=Tubb2a PE=1 SV=1</td><td>NSSYFVEWIPNNVK</td></tr>
<tr><td>></td><td>sp|P85108|TBE Tubulin beta-2A chain OS=Rattus norvegicus GN=Tubb2a PE=1 SV=1</td><td>KLAVNMVPFPR</td></tr>
<tr><td>></td><td>sp|P85108|TBE Tubulin beta-2A chain OS=Rattus norvegicus GN=Tubb2a PE=1 SV=1</td><td>KLAVNMVPFPR</td></tr>
<tr><td>></td><td>sp|P85108|TBE Tubulin beta-2A chain OS=Rattus norvegicus GN=Tubb2a PE=1 SV=1</td><td>KLAVNMVPFPR</td></tr>
<tr><td>></td><td>sp|P85108|TBE Tubulin beta-2A chain OS=Rattus norvegicus GN=Tubb2a PE=1 SV=1</td><td>EVDEQMLNVQNK</td></tr>
<tr><td>></td><td>sp|P85108|TBE Tubulin beta-2A chain OS=Rattus norvegicus GN=Tubb2a PE=1 SV=1</td><td>VSDTVVEPYNATLSVHQLVENTDETYSIDNEALYDICFR</td></tr>
<tr><td>></td><td>sp|P85108|TBE Tubulin beta-2A chain OS=Rattus norvegicus GN=Tubb2a PE=1 SV=1</td><td>GHYTEGAELVDSVLDVVR</td></tr>
<tr><td>></td><td>sp|P85108|TBE Tubulin beta-2A chain OS=Rattus norvegicus GN=Tubb2a PE=1 SV=1</td><td>LTTPTYGDLNHLVSATMSGVTTCLR</td></tr>
<tr><td>></td><td>sp|P85108|TBE Tubulin beta-2A chain OS=Rattus norvegicus GN=Tubb2a PE=1 SV=1</td><td>LTTPTYGDLNHLVSATMSGVTTCLR</td></tr>
<tr><td>></td><td>sp|P63259|ACT Actin, cytoplasmic 2 OS=Rattus norvegicus GN=Actg1 PE=1 SV=1</td><td>AVFPSIVGRPR</td></tr>
<tr><td>></td><td>sp|P63259|ACT Actin, cytoplasmic 2 OS=Rattus norvegicus GN=Actg1 PE=1 SV=1</td><td>DLYANTVLSGGTTMYPGIADR</td></tr>
<tr><td>></td><td>sp|P63259|ACT Actin, cytoplasmic 2 OS=Rattus norvegicus GN=Actg1 PE=1 SV=1</td><td>DSYVGDEAQSK</td></tr>
<tr><td>></td><td>sp|P63259|ACT Actin, cytoplasmic 2 OS=Rattus norvegicus GN=Actg1 PE=1 SV=1</td><td>DSYVGDEAQSKR</td></tr>
<tr><td>></td><td>sp|P63259|ACT Actin, cytoplasmic 2 OS=Rattus norvegicus GN=Actg1 PE=1 SV=1</td><td>EKLCYVALDFEQEMATAASSSSLEK</td></tr>
<tr><td>></td><td>sp|P63259|ACT Actin, cytoplasmic 2 OS=Rattus norvegicus GN=Actg1 PE=1 SV=1</td><td>EKMTQIMFETFNTPAMYVAIQAVLSLYASGR</td></tr>
<tr><td>></td><td>sp|P63259|ACT Actin, cytoplasmic 2 OS=Rattus norvegicus GN=Actg1 PE=1 SV=1</td><td>GYSFTTTAER</td></tr>
<tr><td>></td><td>sp|P63259|ACT Actin, cytoplasmic 2 OS=Rattus norvegicus GN=Actg1 PE=1 SV=1</td><td>HQGVMVGMGQK</td></tr>
<tr><td>></td><td>sp|P63259|ACT Actin, cytoplasmic 2 OS=Rattus norvegicus GN=Actg1 PE=1 SV=1</td><td>IIAPPER</td></tr>
<tr><td>></td><td>sp|P63259|ACT Actin, cytoplasmic 2 OS=Rattus norvegicus GN=Actg1 PE=1 SV=1</td><td>IWHHTFYNELR</td></tr>
<tr><td>></td><td>sp|P63259|ACT Actin, cytoplasmic 2 OS=Rattus norvegicus GN=Actg1 PE=1 SV=1</td><td>KDLYANTVLSGGTTMYPGIADR</td></tr>
<tr><td>></td><td>sp|P63259|ACT Actin, cytoplasmic 2 OS=Rattus norvegicus GN=Actg1 PE=1 SV=1</td><td>KYSVWIGGSILASLSTFQQMWISKQEYDESGPSIVHR</td></tr>
<tr><td>></td><td>sp|P63259|ACT Actin, cytoplasmic 2 OS=Rattus norvegicus GN=Actg1 PE=1 SV=1</td><td>LCYVALDFEQEMATAASSSSLEK</td></tr>
<tr><td>></td><td>sp|P63259|ACT Actin, cytoplasmic 2 OS=Rattus norvegicus GN=Actg1 PE=1 SV=1</td><td>LDLAGR</td></tr>
<tr><td>></td><td>sp|P63259|ACT Actin, cytoplasmic 2 OS=Rattus norvegicus GN=Actg1 PE=1 SV=1</td><td>SYELPDGQVITIGNER</td></tr>
<tr><td>></td><td>sp|P63259|ACT Actin, cytoplasmic 2 OS=Rattus norvegicus GN=Actg1 PE=1 SV=1</td><td>TTGIVMDSGDGVTHTVPIYEGYALPHAILR</td></tr>
<tr><td>></td><td>sp|P63259|ACT Actin, cytoplasmic 2 OS=Rattus norvegicus GN=Actg1 PE=1 SV=1</td><td>VAPEEHPVLLTEA</td></tr>
<tr><td>></td><td>sp|P63259|ACT Actin, cytoplasmic 2 OS=Rattus norvegicus GN=Actg1 PE=1 SV=1</td><td>VAPEEHPVLLTEAPLN</td></tr>
<tr><td>></td><td>sp|P63259|ACT Actin, cytoplasmic 2 OS=Rattus norvegicus GN=Actg1 PE=1 SV=1</td><td>VAPEEHPVLLTEAPLNPK</td></tr>
<tr><td>></td><td>sp|P63259|ACT Actin, cytoplasmic 2 OS=Rattus norvegicus GN=Actg1 PE=1 SV=1</td><td>YPIEHGIVTNWDDMEK</td></tr>
<tr><td>></td><td>sp|P63259|ACT Actin, cytoplasmic 2 OS=Rattus norvegicus GN=Actg1 PE=1 SV=1</td><td>YSVWIGGSILASLSTFQQMWISKQEYDESGPSIVHR</td></tr>
<tr><td>></td><td>sp|P63259|ACT Actin, cytoplasmic 2 OS=Rattus norvegicus GN=Actg1 PE=1 SV=1</td><td>KYSVWIGGSIL</td></tr>
<tr><td>></td><td>sp|P63259|ACT Actin, cytoplasmic 2 OS=Rattus norvegicus GN=Actg1 PE=1 SV=1</td><td>AGFAGDDAPR</td></tr>
<tr><td>></td><td>sp|P63259|ACT Actin, cytoplasmic 2 OS=Rattus norvegicus GN=Actg1 PE=1 SV=1</td><td>EITALAPSTMK</td></tr>
<tr><td>></td><td>sp|P63259|ACT Actin, cytoplasmic 2 OS=Rattus norvegicus GN=Actg1 PE=1 SV=1</td><td>MTQIMFETFNTPAMYVAIQAVLSLYASGR</td></tr>
<tr><td>></td><td>sp|P63259|ACT Actin, cytoplasmic 2 OS=Rattus norvegicus GN=Actg1 PE=1 SV=1</td><td>AGFAGDDAPR</td></tr>
<tr><td>></td><td>sp|P63259|ACT Actin, cytoplasmic 2 OS=Rattus norvegicus GN=Actg1 PE=1 SV=1</td><td>AGFAGDDAPR</td></tr>
<tr><td>></td><td>sp|P63259|ACT Actin, cytoplasmic 2 OS=Rattus norvegicus GN=Actg1 PE=1 SV=1</td><td>AGFAGDDAPR</td></tr>
<tr><td>></td><td>sp|P63259|ACT Actin, cytoplasmic 2 OS=Rattus norvegicus GN=Actg1 PE=1 SV=1</td><td>AVFPSIVGRPR</td></tr>
<tr><td>></td><td>sp|P63259|ACT Actin, cytoplasmic 2 OS=Rattus norvegicus GN=Actg1 PE=1 SV=1</td><td>AVFPSIVGRPR</td></tr>
<tr><td>></td><td>sp|P63259|ACT Actin, cytoplasmic 2 OS=Rattus norvegicus GN=Actg1 PE=1 SV=1</td><td>AVFPSIVGRPR</td></tr>
<tr><td>></td><td>sp|P63259|ACT Actin, cytoplasmic 2 OS=Rattus norvegicus GN=Actg1 PE=1 SV=1</td><td>AVFPSIVGRPR</td></tr>
</table>

>	sp|P63259|ACT Actin, cytoplasmic 2 OS=Rattus norvegicus GN=Actg1 PE=1 SV=1	AVFPSIVGRPR
>	sp|P63259|ACT Actin, cytoplasmic 2 OS=Rattus norvegicus GN=Actg1 PE=1 SV=1	AVFPSIVGRPR
>	sp|P63259|ACT Actin, cytoplasmic 2 OS=Rattus norvegicus GN=Actg1 PE=1 SV=1	AVFPSIVGRPR
>	sp|P63259|ACT Actin, cytoplasmic 2 OS=Rattus norvegicus GN=Actg1 PE=1 SV=1	AVFPSIVGRPR
>	sp|P63259|ACT Actin, cytoplasmic 2 OS=Rattus norvegicus GN=Actg1 PE=1 SV=1	AVFPSIVGRPR
>	sp|P63259|ACT Actin, cytoplasmic 2 OS=Rattus norvegicus GN=Actg1 PE=1 SV=1	AVFPSIVGRPR
>	sp|P63259|ACT Actin, cytoplasmic 2 OS=Rattus norvegicus GN=Actg1 PE=1 SV=1	AVFPSIVGRPR
>	sp|P63259|ACT Actin, cytoplasmic 2 OS=Rattus norvegicus GN=Actg1 PE=1 SV=1	AVFPSIVGRPR
>	sp|P63259|ACT Actin, cytoplasmic 2 OS=Rattus norvegicus GN=Actg1 PE=1 SV=1	DLYANTVLSGGTTMYPGIADR
>	sp|P63259|ACT Actin, cytoplasmic 2 OS=Rattus norvegicus GN=Actg1 PE=1 SV=1	DSYVGDEAQSK
>	sp|P63259|ACT Actin, cytoplasmic 2 OS=Rattus norvegicus GN=Actg1 PE=1 SV=1	DSYVGDEAQSK
>	sp|P63259|ACT Actin, cytoplasmic 2 OS=Rattus norvegicus GN=Actg1 PE=1 SV=1	DSYVGDEAQSK
>	sp|P63259|ACT Actin, cytoplasmic 2 OS=Rattus norvegicus GN=Actg1 PE=1 SV=1	DSYVGDEAQSK
>	sp|P63259|ACT Actin, cytoplasmic 2 OS=Rattus norvegicus GN=Actg1 PE=1 SV=1	DSYVGDEAQSK
>	sp|P63259|ACT Actin, cytoplasmic 2 OS=Rattus norvegicus GN=Actg1 PE=1 SV=1	DSYVGDEAQSKR
>	sp|P63259|ACT Actin, cytoplasmic 2 OS=Rattus norvegicus GN=Actg1 PE=1 SV=1	EITALAPSTMK
>	sp|P63259|ACT Actin, cytoplasmic 2 OS=Rattus norvegicus GN=Actg1 PE=1 SV=1	EITALAPSTMK
>	sp|P63259|ACT Actin, cytoplasmic 2 OS=Rattus norvegicus GN=Actg1 PE=1 SV=1	EITALAPSTMK
>	sp|P63259|ACT Actin, cytoplasmic 2 OS=Rattus norvegicus GN=Actg1 PE=1 SV=1	EITALAPSTMK
>	sp|P63259|ACT Actin, cytoplasmic 2 OS=Rattus norvegicus GN=Actg1 PE=1 SV=1	EKLCYVALDFEQEMATAASSSSLEK
>	sp|P63259|ACT Actin, cytoplasmic 2 OS=Rattus norvegicus GN=Actg1 PE=1 SV=1	EKLCYVALDFEQEMATAASSSSLEK
>	sp|P63259|ACT Actin, cytoplasmic 2 OS=Rattus norvegicus GN=Actg1 PE=1 SV=1	EKLCYVALDFEQEMATAASSSSLEK
>	sp|P63259|ACT Actin, cytoplasmic 2 OS=Rattus norvegicus GN=Actg1 PE=1 SV=1	EKLCYVALDFEQEMATAASSSSLEK
>	sp|P63259|ACT Actin, cytoplasmic 2 OS=Rattus norvegicus GN=Actg1 PE=1 SV=1	EKLCYVALDFEQEMATAASSSSLEK
>	sp|P63259|ACT Actin, cytoplasmic 2 OS=Rattus norvegicus GN=Actg1 PE=1 SV=1	EKLCYVALDFEQEMATAASSSSLEK
>	sp|P63259|ACT Actin, cytoplasmic 2 OS=Rattus norvegicus GN=Actg1 PE=1 SV=1	EKLCYVALDFEQEMATAASSSSLEK
>	sp|P63259|ACT Actin, cytoplasmic 2 OS=Rattus norvegicus GN=Actg1 PE=1 SV=1	HQGVMVGMGQK
>	sp|P63259|ACT Actin, cytoplasmic 2 OS=Rattus norvegicus GN=Actg1 PE=1 SV=1	HQGVMVGMGQK
>	sp|P63259|ACT Actin, cytoplasmic 2 OS=Rattus norvegicus GN=Actg1 PE=1 SV=1	KDLYANTVLSGGTTMYPGIADR
>	sp|P63259|ACT Actin, cytoplasmic 2 OS=Rattus norvegicus GN=Actg1 PE=1 SV=1	KDLYANTVLSGGTTMYPGIADR
>	sp|P63259|ACT Actin, cytoplasmic 2 OS=Rattus norvegicus GN=Actg1 PE=1 SV=1	KDLYANTVLSGGTTMYPGIADR
>	sp|P63259|ACT Actin, cytoplasmic 2 OS=Rattus norvegicus GN=Actg1 PE=1 SV=1	KDLYANTVLSGGTTMYPGIADR
>	sp|P63259|ACT Actin, cytoplasmic 2 OS=Rattus norvegicus GN=Actg1 PE=1 SV=1	KDLYANTVLSGGTTMYPGIADR
>	sp|P63259|ACT Actin, cytoplasmic 2 OS=Rattus norvegicus GN=Actg1 PE=1 SV=1	KDLYANTVLSGGTTMYPGIADR
>	sp|P63259|ACT Actin, cytoplasmic 2 OS=Rattus norvegicus GN=Actg1 PE=1 SV=1	KDLYANTVLSGGTTMYPGIADR
>	sp|P63259|ACT Actin, cytoplasmic 2 OS=Rattus norvegicus GN=Actg1 PE=1 SV=1	KDLYANTVLSGGTTMYPGIADR
>	sp|P63259|ACT Actin, cytoplasmic 2 OS=Rattus norvegicus GN=Actg1 PE=1 SV=1	KDLYANTVLSGGTTMYPGIADR
>	sp|P63259|ACT Actin, cytoplasmic 2 OS=Rattus norvegicus GN=Actg1 PE=1 SV=1	KDLYANTVLSGGTTMYPGIADR
>	sp|P63259|ACT Actin, cytoplasmic 2 OS=Rattus norvegicus GN=Actg1 PE=1 SV=1	KDLYANTVLSGGTTMYPGIADR
>	sp|P63259|ACT Actin, cytoplasmic 2 OS=Rattus norvegicus GN=Actg1 PE=1 SV=1	KDLYANTVLSGGTTMYPGIADR
>	sp|P63259|ACT Actin, cytoplasmic 2 OS=Rattus norvegicus GN=Actg1 PE=1 SV=1	KDLYANTVLSGGTTMYPGIADR
>	sp|P63259|ACT Actin, cytoplasmic 2 OS=Rattus norvegicus GN=Actg1 PE=1 SV=1	KDLYANTVLSGGTTMYPGIADR
>	sp|P63259|ACT Actin, cytoplasmic 2 OS=Rattus norvegicus GN=Actg1 PE=1 SV=1	KDLYANTVLSGGTTMYPGIADR
>	sp|P63259|ACT Actin, cytoplasmic 2 OS=Rattus norvegicus GN=Actg1 PE=1 SV=1	KDLYANTVLSGGTTMYPGIADR
>	sp|P63259|ACT Actin, cytoplasmic 2 OS=Rattus norvegicus GN=Actg1 PE=1 SV=1	KDLYANTVLSGGTTMYPGIADR
>	sp|P63259|ACT Actin, cytoplasmic 2 OS=Rattus norvegicus GN=Actg1 PE=1 SV=1	KYSVWIGGSILASLSTFQQMWISKQEYDESGPSIVHR
>	sp|P63259|ACT Actin, cytoplasmic 2 OS=Rattus norvegicus GN=Actg1 PE=1 SV=1	LCYVALDFEQEMATAASSSSLEK
>	sp|P63259|ACT Actin, cytoplasmic 2 OS=Rattus norvegicus GN=Actg1 PE=1 SV=1	LCYVALDFEQEMATAASSSSLEK
>	sp|P63259|ACT Actin, cytoplasmic 2 OS=Rattus norvegicus GN=Actg1 PE=1 SV=1	LCYVALDFEQEMATAASSSSLEK
>	sp|P63259|ACT Actin, cytoplasmic 2 OS=Rattus norvegicus GN=Actg1 PE=1 SV=1	LCYVALDFEQEMATAASSSSLEK
>	sp|P63259|ACT Actin, cytoplasmic 2 OS=Rattus norvegicus GN=Actg1 PE=1 SV=1	LCYVALDFEQEMATAASSSSLEK
>	sp|P63259|ACT Actin, cytoplasmic 2 OS=Rattus norvegicus GN=Actg1 PE=1 SV=1	LCYVALDFEQEMATAASSSSLEK
>	sp|P63259|ACT Actin, cytoplasmic 2 OS=Rattus norvegicus GN=Actg1 PE=1 SV=1	LCYVALDFEQEMATAASSSSLEK
>	sp|P63259|ACT Actin, cytoplasmic 2 OS=Rattus norvegicus GN=Actg1 PE=1 SV=1	LCYVALDFEQEMATAASSSSLEK
>	sp|P63259|ACT Actin, cytoplasmic 2 OS=Rattus norvegicus GN=Actg1 PE=1 SV=1	LCYVALDFEQEMATAASSSSLEK
>	sp|P63259|ACT Actin, cytoplasmic 2 OS=Rattus norvegicus GN=Actg1 PE=1 SV=1	LCYVALDFEQEMATAASSSSLEK
>	sp|P63259|ACT Actin, cytoplasmic 2 OS=Rattus norvegicus GN=Actg1 PE=1 SV=1	LCYVALDFEQEMATAASSSSLEK
>	sp|P63259|ACT Actin, cytoplasmic 2 OS=Rattus norvegicus GN=Actg1 PE=1 SV=1	LCYVALDFEQEMATAASSSSLEK
>	sp|P63259|ACT Actin, cytoplasmic 2 OS=Rattus norvegicus GN=Actg1 PE=1 SV=1	LCYVALDFEQEMATAASSSSLEK
>	sp|P63259|ACT Actin, cytoplasmic 2 OS=Rattus norvegicus GN=Actg1 PE=1 SV=1	LCYVALDFEQEMATAASSSSLEK
>	sp|P63259|ACT Actin, cytoplasmic 2 OS=Rattus norvegicus GN=Actg1 PE=1 SV=1	MTQIMFETFNTPAMYVAIQAVLSLYASGR
>	sp|P63259|ACT Actin, cytoplasmic 2 OS=Rattus norvegicus GN=Actg1 PE=1 SV=1	SYELPDGQVITIGNER
>	sp|P63259|ACT Actin, cytoplasmic 2 OS=Rattus norvegicus GN=Actg1 PE=1 SV=1	SYELPDGQVITIGNER
>	sp|P63259|ACT Actin, cytoplasmic 2 OS=Rattus norvegicus GN=Actg1 PE=1 SV=1	SYELPDGQVITIGNER
>	sp|P63259|ACT Actin, cytoplasmic 2 OS=Rattus norvegicus GN=Actg1 PE=1 SV=1	SYELPDGQVITIGNER
>	sp|P63259|ACT Actin, cytoplasmic 2 OS=Rattus norvegicus GN=Actg1 PE=1 SV=1	SYELPDGQVITIGNER
>	sp|P63259|ACT Actin, cytoplasmic 2 OS=Rattus norvegicus GN=Actg1 PE=1 SV=1	SYELPDGQVITIGNER
>	sp|P63259|ACT Actin, cytoplasmic 2 OS=Rattus norvegicus GN=Actg1 PE=1 SV=1	SYELPDGQVITIGNER
>	sp|P63259|ACT Actin, cytoplasmic 2 OS=Rattus norvegicus GN=Actg1 PE=1 SV=1	SYELPDGQVITIGNER
>	sp|P63259|ACT Actin, cytoplasmic 2 OS=Rattus norvegicus GN=Actg1 PE=1 SV=1	SYELPDGQVITIGNER
>	sp|P63259|ACT Actin, cytoplasmic 2 OS=Rattus norvegicus GN=Actg1 PE=1 SV=1	SYELPDGQVITIGNER
>	sp|P63259|ACT Actin, cytoplasmic 2 OS=Rattus norvegicus GN=Actg1 PE=1 SV=1	SYELPDGQVITIGNER
>	sp|P63259|ACT Actin, cytoplasmic 2 OS=Rattus norvegicus GN=Actg1 PE=1 SV=1	SYELPDGQVITIGNER
>	sp|P63259|ACT Actin, cytoplasmic 2 OS=Rattus norvegicus GN=Actg1 PE=1 SV=1	SYELPDGQVITIGNER

>	sp|P63259|ACT Actin, cytoplasmic 2 OS=Rattus norvegicus GN=Actg1 PE=1 SV=1	TTGIVMDSGDGVTHTVPIYEGYALPHAILR
>	sp|P63259|ACT Actin, cytoplasmic 2 OS=Rattus norvegicus GN=Actg1 PE=1 SV=1	TTGIVMDSGDGVTHTVPIYEGYALPHAILR
>	sp|P63259|ACT Actin, cytoplasmic 2 OS=Rattus norvegicus GN=Actg1 PE=1 SV=1	TTGIVMDSGDGVTHTVPIYEGYALPHAILR
>	sp|P63259|ACT Actin, cytoplasmic 2 OS=Rattus norvegicus GN=Actg1 PE=1 SV=1	TTGIVMDSGDGVTHTVPIYEGYALPHAILR
>	sp|P63259|ACT Actin, cytoplasmic 2 OS=Rattus norvegicus GN=Actg1 PE=1 SV=1	TTGIVMDSGDGVTHTVPIYEGYALPHAILR
>	sp|P63259|ACT Actin, cytoplasmic 2 OS=Rattus norvegicus GN=Actg1 PE=1 SV=1	TTGIVMDSGDGVTHTVPIYEGYALPHAILR
>	sp|P63259|ACT Actin, cytoplasmic 2 OS=Rattus norvegicus GN=Actg1 PE=1 SV=1	TTGIVMDSGDGVTHTVPIYEGYALPHAILR
>	sp|P63259|ACT Actin, cytoplasmic 2 OS=Rattus norvegicus GN=Actg1 PE=1 SV=1	TTGIVMDSGDGVTHTVPIYEGYALPHAILR
>	sp|P63259|ACT Actin, cytoplasmic 2 OS=Rattus norvegicus GN=Actg1 PE=1 SV=1	TTGIVMDSGDGVTHTVPIYEGYALPHAILR
>	sp|P63259|ACT Actin, cytoplasmic 2 OS=Rattus norvegicus GN=Actg1 PE=1 SV=1	TTGIVMDSGDGVTHTVPIYEGYALPHAILR
>	sp|P63259|ACT Actin, cytoplasmic 2 OS=Rattus norvegicus GN=Actg1 PE=1 SV=1	TTGIVMDSGDGVTHTVPIYEGYALPHAILR
>	sp|P63259|ACT Actin, cytoplasmic 2 OS=Rattus norvegicus GN=Actg1 PE=1 SV=1	TTGIVMDSGDGVTHTVPIYEGYALPHAILR
>	sp|P63259|ACT Actin, cytoplasmic 2 OS=Rattus norvegicus GN=Actg1 PE=1 SV=1	TTGIVMDSGDGVTHTVPIYEGYALPHAILR
>	sp|P63259|ACT Actin, cytoplasmic 2 OS=Rattus norvegicus GN=Actg1 PE=1 SV=1	TTGIVMDSGDGVTHTVPIYEGYALPHAILR
>	sp|P63259|ACT Actin, cytoplasmic 2 OS=Rattus norvegicus GN=Actg1 PE=1 SV=1	TTGIVMDSGDGVTHTVPIYEGYALPHAILR
>	sp|P63259|ACT Actin, cytoplasmic 2 OS=Rattus norvegicus GN=Actg1 PE=1 SV=1	TTGIVMDSGDGVTHTVPIYEGYALPHAILR
>	sp|P63259|ACT Actin, cytoplasmic 2 OS=Rattus norvegicus GN=Actg1 PE=1 SV=1	TTGIVMDSGDGVTHTVPIYEGYALPHAILR
>	sp|P63259|ACT Actin, cytoplasmic 2 OS=Rattus norvegicus GN=Actg1 PE=1 SV=1	TTGIVMDSGDGVTHTVPIYEGYALPHAILR
>	sp|P63259|ACT Actin, cytoplasmic 2 OS=Rattus norvegicus GN=Actg1 PE=1 SV=1	TTGIVMDSGDGVTHTVPIYEGYALPHAILR
>	sp|P63259|ACT Actin, cytoplasmic 2 OS=Rattus norvegicus GN=Actg1 PE=1 SV=1	TTGIVMDSGDGVTHTVPIYEGYALPHAILR
>	sp|P63259|ACT Actin, cytoplasmic 2 OS=Rattus norvegicus GN=Actg1 PE=1 SV=1	TTGIVMDSGDGVTHTVPIYEGYALPHAILR
>	sp|P63259|ACT Actin, cytoplasmic 2 OS=Rattus norvegicus GN=Actg1 PE=1 SV=1	TTGIVMDSGDGVTHTVPIYEGYALPHAILR
>	sp|P63259|ACT Actin, cytoplasmic 2 OS=Rattus norvegicus GN=Actg1 PE=1 SV=1	TTGIVMDSGDGVTHTVPIYEGYALPHAILR
>	sp|P63259|ACT Actin, cytoplasmic 2 OS=Rattus norvegicus GN=Actg1 PE=1 SV=1	TTGIVMDSGDGVTHTVPIYEGYALPHAILR
>	sp|P63259|ACT Actin, cytoplasmic 2 OS=Rattus norvegicus GN=Actg1 PE=1 SV=1	VAPEEHPVLLTEA
>	sp|P63259|ACT Actin, cytoplasmic 2 OS=Rattus norvegicus GN=Actg1 PE=1 SV=1	VAPEEHPVLLTEA
>	sp|P63259|ACT Actin, cytoplasmic 2 OS=Rattus norvegicus GN=Actg1 PE=1 SV=1	VAPEEHPVLLTEAPLN
>	sp|P63259|ACT Actin, cytoplasmic 2 OS=Rattus norvegicus GN=Actg1 PE=1 SV=1	VAPEEHPVLLTEAPLNPK
>	sp|P63259|ACT Actin, cytoplasmic 2 OS=Rattus norvegicus GN=Actg1 PE=1 SV=1	VAPEEHPVLLTEAPLNPK
>	sp|P63259|ACT Actin, cytoplasmic 2 OS=Rattus norvegicus GN=Actg1 PE=1 SV=1	VAPEEHPVLLTEAPLNPK
>	sp|P63259|ACT Actin, cytoplasmic 2 OS=Rattus norvegicus GN=Actg1 PE=1 SV=1	VAPEEHPVLLTEAPLNPK
>	sp|P63259|ACT Actin, cytoplasmic 2 OS=Rattus norvegicus GN=Actg1 PE=1 SV=1	VAPEEHPVLLTEAPLNPK
>	sp|P63259|ACT Actin, cytoplasmic 2 OS=Rattus norvegicus GN=Actg1 PE=1 SV=1	VAPEEHPVLLTEAPLNPK
>	sp|P63259|ACT Actin, cytoplasmic 2 OS=Rattus norvegicus GN=Actg1 PE=1 SV=1	VAPEEHPVLLTEAPLNPK
>	sp|P63259|ACT Actin, cytoplasmic 2 OS=Rattus norvegicus GN=Actg1 PE=1 SV=1	VAPEEHPVLLTEAPLNPK
>	sp|P63259|ACT Actin, cytoplasmic 2 OS=Rattus norvegicus GN=Actg1 PE=1 SV=1	VAPEEHPVLLTEAPLNPK
>	sp|P63259|ACT Actin, cytoplasmic 2 OS=Rattus norvegicus GN=Actg1 PE=1 SV=1	VAPEEHPVLLTEAPLNPK
>	sp|P63259|ACT Actin, cytoplasmic 2 OS=Rattus norvegicus GN=Actg1 PE=1 SV=1	VAPEEHPVLLTEAPLNPK
>	sp|P63259|ACT Actin, cytoplasmic 2 OS=Rattus norvegicus GN=Actg1 PE=1 SV=1	VAPEEHPVLLTEAPLNPK
>	sp|P63259|ACT Actin, cytoplasmic 2 OS=Rattus norvegicus GN=Actg1 PE=1 SV=1	VAPEEHPVLLTEAPLNPK
>	sp|P63259|ACT Actin, cytoplasmic 2 OS=Rattus norvegicus GN=Actg1 PE=1 SV=1	VAPEEHPVLLTEAPLNPK
>	sp|P63259|ACT Actin, cytoplasmic 2 OS=Rattus norvegicus GN=Actg1 PE=1 SV=1	VAPEEHPVLLTEAPLNPK
>	sp|P63259|ACT Actin, cytoplasmic 2 OS=Rattus norvegicus GN=Actg1 PE=1 SV=1	VAPEEHPVLLTEAPLNPK
>	sp|P63259|ACT Actin, cytoplasmic 2 OS=Rattus norvegicus GN=Actg1 PE=1 SV=1	VAPEEHPVLLTEAPLNPK
>	sp|P63259|ACT Actin, cytoplasmic 2 OS=Rattus norvegicus GN=Actg1 PE=1 SV=1	VAPEEHPVLLTEAPLNPK
>	sp|P63259|ACT Actin, cytoplasmic 2 OS=Rattus norvegicus GN=Actg1 PE=1 SV=1	VAPEEHPVLLTEAPLNPK
>	sp|P63259|ACT Actin, cytoplasmic 2 OS=Rattus norvegicus GN=Actg1 PE=1 SV=1	VAPEEHPVLLTEAPLNPK
>	sp|P63259|ACT Actin, cytoplasmic 2 OS=Rattus norvegicus GN=Actg1 PE=1 SV=1	VAPEEHPVLLTEAPLNPK
>	sp|P63259|ACT Actin, cytoplasmic 2 OS=Rattus norvegicus GN=Actg1 PE=1 SV=1	VAPEEHPVLLTEAPLNPK
>	sp|P63259|ACT Actin, cytoplasmic 2 OS=Rattus norvegicus GN=Actg1 PE=1 SV=1	VAPEEHPVLLTEAPLNPK
>	sp|P63259|ACT Actin, cytoplasmic 2 OS=Rattus norvegicus GN=Actg1 PE=1 SV=1	VAPEEHPVLLTEAPLNPK
>	sp|P63259|ACT Actin, cytoplasmic 2 OS=Rattus norvegicus GN=Actg1 PE=1 SV=1	VAPEEHPVLLTEAPLNPK
>	sp|P63259|ACT Actin, cytoplasmic 2 OS=Rattus norvegicus GN=Actg1 PE=1 SV=1	VAPEEHPVLLTEAPLNPK
>	sp|P63259|ACT Actin, cytoplasmic 2 OS=Rattus norvegicus GN=Actg1 PE=1 SV=1	VAPEEHPVLLTEAPLNPK
>	sp|P63259|ACT Actin, cytoplasmic 2 OS=Rattus norvegicus GN=Actg1 PE=1 SV=1	VAPEEHPVLLTEAPLNPK
>	sp|P63259|ACT Actin, cytoplasmic 2 OS=Rattus norvegicus GN=Actg1 PE=1 SV=1	VAPEEHPVLLTEAPLNPK
>	sp|P63259|ACT Actin, cytoplasmic 2 OS=Rattus norvegicus GN=Actg1 PE=1 SV=1	VAPEEHPVLLTEAPLNPK
>	sp|P63259|ACT Actin, cytoplasmic 2 OS=Rattus norvegicus GN=Actg1 PE=1 SV=1	YSVWIGGSILASLSTFQQMWISKQEYDESGPSIVHR
>	sp|P63259|ACT Actin, cytoplasmic 2 OS=Rattus norvegicus GN=Actg1 PE=1 SV=1	YSVWIGGSILASLSTFQQMWISKQEYDESGPSIVHR
>	sp|P63259|ACT Actin, cytoplasmic 2 OS=Rattus norvegicus GN=Actg1 PE=1 SV=1	YSVWIGGSILASLSTFQQMWISKQEYDESGPSIVHR
>	sp|P63259|ACT Actin, cytoplasmic 2 OS=Rattus norvegicus GN=Actg1 PE=1 SV=1	YSVWIGGSILASLSTFQQMWISKQEYDESGPSIVHR
>	sp|P63259|ACT Actin, cytoplasmic 2 OS=Rattus norvegicus GN=Actg1 PE=1 SV=1	YSVWIGGSILASLSTFQQMWISKQEYDESGPSIVHR
>	sp|P63259|ACT Actin, cytoplasmic 2 OS=Rattus norvegicus GN=Actg1 PE=1 SV=1	AVFPSIVGRPR
>	sp|P63259|ACT Actin, cytoplasmic 2 OS=Rattus norvegicus GN=Actg1 PE=1 SV=1	EKLCYVALDFEQEMATAASSSSLEK
>	sp|P63259|ACT Actin, cytoplasmic 2 OS=Rattus norvegicus GN=Actg1 PE=1 SV=1	KDLYANTVLSGGTTMYPGIADR
>	sp|P63259|ACT Actin, cytoplasmic 2 OS=Rattus norvegicus GN=Actg1 PE=1 SV=1	VAPEEHPVLLTEAPLNPK
>	sp|P63259|ACT Actin, cytoplasmic 2 OS=Rattus norvegicus GN=Actg1 PE=1 SV=1	SYELPDGQVITIGNER
>	sp|P63259|ACT Actin, cytoplasmic 2 OS=Rattus norvegicus GN=Actg1 PE=1 SV=1	VAPEEHPVLLTEAPLNPK
>	sp|P63259|ACT Actin, cytoplasmic 2 OS=Rattus norvegicus GN=Actg1 PE=1 SV=1	LCYVALDFEQEMATAASSSSLEK
>	sp|P63259|ACT Actin, cytoplasmic 2 OS=Rattus norvegicus GN=Actg1 PE=1 SV=1	AVFPSIVGRPR
>	sp|P63259|ACT Actin, cytoplasmic 2 OS=Rattus norvegicus GN=Actg1 PE=1 SV=1	VAPEEHPVLLTEAPLNPK
>	sp|P63259|ACT Actin, cytoplasmic 2 OS=Rattus norvegicus GN=Actg1 PE=1 SV=1	VAPEEHPVLLTEAPLNPK
>	sp|P63259|ACT Actin, cytoplasmic 2 OS=Rattus norvegicus GN=Actg1 PE=1 SV=1	VAPEEHPVLLTEAPLNPK
>	sp|P63259|ACT Actin, cytoplasmic 2 OS=Rattus norvegicus GN=Actg1 PE=1 SV=1	KYSVWIGGSILASLSTFQQMWISKQEYDESGPSIVHR

>	sp|P63259|ACT Actin, cytoplasmic 2 OS=Rattus norvegicus GN=Actg1 PE=1 SV=1	SYELPDGQVITIGNER
>	sp|P63259|ACT Actin, cytoplasmic 2 OS=Rattus norvegicus GN=Actg1 PE=1 SV=1	AGFAGDDAPR
>	sp|P63259|ACT Actin, cytoplasmic 2 OS=Rattus norvegicus GN=Actg1 PE=1 SV=1	IIAPPER
>	sp|P63259|ACT Actin, cytoplasmic 2 OS=Rattus norvegicus GN=Actg1 PE=1 SV=1	DLYANTVLSGGTTMYPGIADR
>	sp|P63259|ACT Actin, cytoplasmic 2 OS=Rattus norvegicus GN=Actg1 PE=1 SV=1	AGFAGDDAPR
>	sp|P63259|ACT Actin, cytoplasmic 2 OS=Rattus norvegicus GN=Actg1 PE=1 SV=1	TTGIVMDSGDGVTHTVPIYEGYALPHAILR
>	sp|P63259|ACT Actin, cytoplasmic 2 OS=Rattus norvegicus GN=Actg1 PE=1 SV=1	AGFAGDDAPR
>	sp|P63259|ACT Actin, cytoplasmic 2 OS=Rattus norvegicus GN=Actg1 PE=1 SV=1	KDLYANTVLSGGTTMYPGIADR
>	sp|P63259|ACT Actin, cytoplasmic 2 OS=Rattus norvegicus GN=Actg1 PE=1 SV=1	VAPEEHPVLLTEAPL
>	sp|P63259|ACT Actin, cytoplasmic 2 OS=Rattus norvegicus GN=Actg1 PE=1 SV=1	SYELPDGQVITIGNER
>	sp|P63259|ACT Actin, cytoplasmic 2 OS=Rattus norvegicus GN=Actg1 PE=1 SV=1	YPIEHGIVTNWDDMEK
>	sp|P63259|ACT Actin, cytoplasmic 2 OS=Rattus norvegicus GN=Actg1 PE=1 SV=1	SSSSLEKSYELPDGQVITIGNER
>	sp|P63259|ACT Actin, cytoplasmic 2 OS=Rattus norvegicus GN=Actg1 PE=1 SV=1	AGFAGDDAPR
>	sp|P63259|ACT Actin, cytoplasmic 2 OS=Rattus norvegicus GN=Actg1 PE=1 SV=1	KDLYANTVLSGGTTMYPGIADR
>	sp|P63259|ACT Actin, cytoplasmic 2 OS=Rattus norvegicus GN=Actg1 PE=1 SV=1	KDLYANTVLSGGTTMYPGIADR
>	sp|P63259|ACT Actin, cytoplasmic 2 OS=Rattus norvegicus GN=Actg1 PE=1 SV=1	KDLYANTVLSGGTTMYPGIADR
>	sp|P63259|ACT Actin, cytoplasmic 2 OS=Rattus norvegicus GN=Actg1 PE=1 SV=1	KDLYANTVLSGGTTMYPGIADR
>	sp|P63259|ACT Actin, cytoplasmic 2 OS=Rattus norvegicus GN=Actg1 PE=1 SV=1	KDLYANTVLSGGTTMYPGIADR
>	sp|P63259|ACT Actin, cytoplasmic 2 OS=Rattus norvegicus GN=Actg1 PE=1 SV=1	KDLYANTVLSGGTTMYPGIADR
>	sp|P63259|ACT Actin, cytoplasmic 2 OS=Rattus norvegicus GN=Actg1 PE=1 SV=1	TTGIVMDSGDGVTHTVPIYEGYALPHAILR
>	sp|P63259|ACT Actin, cytoplasmic 2 OS=Rattus norvegicus GN=Actg1 PE=1 SV=1	TTGIVMDSGDGVTHTVPIYEGYALPHAILR
>	sp|P63259|ACT Actin, cytoplasmic 2 OS=Rattus norvegicus GN=Actg1 PE=1 SV=1	IIAPPER
>	sp|P63259|ACT Actin, cytoplasmic 2 OS=Rattus norvegicus GN=Actg1 PE=1 SV=1	KDLYANTVLSGGTTMYPGIADR
>	sp|P63259|ACT Actin, cytoplasmic 2 OS=Rattus norvegicus GN=Actg1 PE=1 SV=1	DLYANTVLSGGTTMYPGIADR
>	sp|P63259|ACT Actin, cytoplasmic 2 OS=Rattus norvegicus GN=Actg1 PE=1 SV=1	IIAPPER
>	sp|P63259|ACT Actin, cytoplasmic 2 OS=Rattus norvegicus GN=Actg1 PE=1 SV=1	IIAPPER
>	sp|P63259|ACT Actin, cytoplasmic 2 OS=Rattus norvegicus GN=Actg1 PE=1 SV=1	TTGIVMDSGDGVTHTVPIYEGYALPHAILR
>	sp|P63259|ACT Actin, cytoplasmic 2 OS=Rattus norvegicus GN=Actg1 PE=1 SV=1	LCYVALDFEQEMATAASSSSLEK
>	sp|P63259|ACT Actin, cytoplasmic 2 OS=Rattus norvegicus GN=Actg1 PE=1 SV=1	YPIEHGIVTNWDDMEK
>	sp|P63259|ACT Actin, cytoplasmic 2 OS=Rattus norvegicus GN=Actg1 PE=1 SV=1	IIAPPER
>	sp|P63259|ACT Actin, cytoplasmic 2 OS=Rattus norvegicus GN=Actg1 PE=1 SV=1	DSYVGDEAQSKR
>	sp|P63259|ACT Actin, cytoplasmic 2 OS=Rattus norvegicus GN=Actg1 PE=1 SV=1	IIAPPER
>	sp|P63259|ACT Actin, cytoplasmic 2 OS=Rattus norvegicus GN=Actg1 PE=1 SV=1	EKLCYVALDFEQEMATAASSSSLEK
>	sp|P63259|ACT Actin, cytoplasmic 2 OS=Rattus norvegicus GN=Actg1 PE=1 SV=1	AVFPSIVGRPR
>	sp|P63259|ACT Actin, cytoplasmic 2 OS=Rattus norvegicus GN=Actg1 PE=1 SV=1	VAPEEHPVLLTEAPLNPK
>	sp|P63259|ACT Actin, cytoplasmic 2 OS=Rattus norvegicus GN=Actg1 PE=1 SV=1	YPIEHGIVTNWDDMEK
>	sp|P63259|ACT Actin, cytoplasmic 2 OS=Rattus norvegicus GN=Actg1 PE=1 SV=1	VAPEEHPVLLTEAPLNPK
>	sp|P63259|ACT Actin, cytoplasmic 2 OS=Rattus norvegicus GN=Actg1 PE=1 SV=1	KDLYANTVLSGGTTMYPGIADR
>	sp|P63259|ACT Actin, cytoplasmic 2 OS=Rattus norvegicus GN=Actg1 PE=1 SV=1	LCYVALDFEQEMATAASSSSLEK
>	sp|P63259|ACT Actin, cytoplasmic 2 OS=Rattus norvegicus GN=Actg1 PE=1 SV=1	VAPEEHPVLLTEAPLNPK
>	sp|P63259|ACT Actin, cytoplasmic 2 OS=Rattus norvegicus GN=Actg1 PE=1 SV=1	VAPEEHPVLLTEAPLNPK
>	sp|P63259|ACT Actin, cytoplasmic 2 OS=Rattus norvegicus GN=Actg1 PE=1 SV=1	TTGIVMDSGDGVTHTVPIYEGYALPHAILR
>	sp|P63259|ACT Actin, cytoplasmic 2 OS=Rattus norvegicus GN=Actg1 PE=1 SV=1	VAPEEHPVLLTEAPLNPK
>	sp|P63259|ACT Actin, cytoplasmic 2 OS=Rattus norvegicus GN=Actg1 PE=1 SV=1	LDLAGR
>	sp|P63259|ACT Actin, cytoplasmic 2 OS=Rattus norvegicus GN=Actg1 PE=1 SV=1	DLYANTVLSGGTTMYPGIADR
>	sp|P63259|ACT Actin, cytoplasmic 2 OS=Rattus norvegicus GN=Actg1 PE=1 SV=1	LCYVALDFEQEMATAASSSSLEK
>	sp|P63259|ACT Actin, cytoplasmic 2 OS=Rattus norvegicus GN=Actg1 PE=1 SV=1	GYSFTTTAER
>	sp|P63259|ACT Actin, cytoplasmic 2 OS=Rattus norvegicus GN=Actg1 PE=1 SV=1	KDLYANTVLSGGTTMYPGIADR
>	sp|P63259|ACT Actin, cytoplasmic 2 OS=Rattus norvegicus GN=Actg1 PE=1 SV=1	KDLYANTVLSGGTTMYPGIADR
>	sp|P63259|ACT Actin, cytoplasmic 2 OS=Rattus norvegicus GN=Actg1 PE=1 SV=1	KYSVWIGGSILASLSTFQQMWISKQEYDESGPSIVHR
>	sp|P63259|ACT Actin, cytoplasmic 2 OS=Rattus norvegicus GN=Actg1 PE=1 SV=1	LCYVALDFEQEMATAASSSSLEK
>	sp|P63259|ACT Actin, cytoplasmic 2 OS=Rattus norvegicus GN=Actg1 PE=1 SV=1	KDLYANTVLSGGTTMYPGIADR
>	sp|P63259|ACT Actin, cytoplasmic 2 OS=Rattus norvegicus GN=Actg1 PE=1 SV=1	EKMTQIMFETFNTPAMYVAIQAVLSLYASGR
>	sp|P63259|ACT Actin, cytoplasmic 2 OS=Rattus norvegicus GN=Actg1 PE=1 SV=1	SYELPDGQVITIGNER
>	sp|P63259|ACT Actin, cytoplasmic 2 OS=Rattus norvegicus GN=Actg1 PE=1 SV=1	YPIEHGIVTNWDDMEK
>	sp|P63259|ACT Actin, cytoplasmic 2 OS=Rattus norvegicus GN=Actg1 PE=1 SV=1	VAPEEHPVLLTEAPLN
>	sp|P63259|ACT Actin, cytoplasmic 2 OS=Rattus norvegicus GN=Actg1 PE=1 SV=1	LDLAGRDLTDYLMK
>	sp|P63259|ACT Actin, cytoplasmic 2 OS=Rattus norvegicus GN=Actg1 PE=1 SV=1	VAPEEHPVLLTEAPLNPK
>	sp|P63259|ACT Actin, cytoplasmic 2 OS=Rattus norvegicus GN=Actg1 PE=1 SV=1	DSYVGDEAQSK
>	sp|P63259|ACT Actin, cytoplasmic 2 OS=Rattus norvegicus GN=Actg1 PE=1 SV=1	TTGIVMDSGDGVTHTVPIYEGYALPHAILR
>	sp|P63259|ACT Actin, cytoplasmic 2 OS=Rattus norvegicus GN=Actg1 PE=1 SV=1	YPIEHGIVTNWDDMEK
>	sp|P63259|ACT Actin, cytoplasmic 2 OS=Rattus norvegicus GN=Actg1 PE=1 SV=1	VAPEEHPVLLTEAPLNPK
>	sp|P63259|ACT Actin, cytoplasmic 2 OS=Rattus norvegicus GN=Actg1 PE=1 SV=1	VAPEEHPVLLTEAPLNPK
>	sp|P63259|ACT Actin, cytoplasmic 2 OS=Rattus norvegicus GN=Actg1 PE=1 SV=1	YPIEHGIVTNWDDMEK
>	sp|P63259|ACT Actin, cytoplasmic 2 OS=Rattus norvegicus GN=Actg1 PE=1 SV=1	LCYVALDFEQEMATAASSSSLEK
>	sp|P63259|ACT Actin, cytoplasmic 2 OS=Rattus norvegicus GN=Actg1 PE=1 SV=1	EITALAPSTMK
>	sp|P63259|ACT Actin, cytoplasmic 2 OS=Rattus norvegicus GN=Actg1 PE=1 SV=1	VAPEEHPVLLTEAPLNPK
>	sp|P63259|ACT Actin, cytoplasmic 2 OS=Rattus norvegicus GN=Actg1 PE=1 SV=1	VAPEEHPVLLTEAPLNPK
>	sp|P63259|ACT Actin, cytoplasmic 2 OS=Rattus norvegicus GN=Actg1 PE=1 SV=1	AGFAGDDAPR
>	sp|P63259|ACT Actin, cytoplasmic 2 OS=Rattus norvegicus GN=Actg1 PE=1 SV=1	GILTLK
>	sp|P63259|ACT Actin, cytoplasmic 2 OS=Rattus norvegicus GN=Actg1 PE=1 SV=1	QEYDESGPSIVHR
>	sp|P63259|ACT Actin, cytoplasmic 2 OS=Rattus norvegicus GN=Actg1 PE=1 SV=1	ILTER
>	sp|P63259|ACT Actin, cytoplasmic 2 OS=Rattus norvegicus GN=Actg1 PE=1 SV=1	VAPEEHPVLLTEAPLNPK
>	sp|P63259|ACT Actin, cytoplasmic 2 OS=Rattus norvegicus GN=Actg1 PE=1 SV=1	LCYVALDFEQEMATAASSSSLEK

>	sp|P63259|ACT Actin, cytoplasmic 2 OS=Rattus norvegicus GN=Actg1 PE=1 SV=1	SYELPDGQVITIGNER
>	sp|P63259|ACT Actin, cytoplasmic 2 OS=Rattus norvegicus GN=Actg1 PE=1 SV=1	GILTLK
>	sp|P63259|ACT Actin, cytoplasmic 2 OS=Rattus norvegicus GN=Actg1 PE=1 SV=1	EKLCYVALDFEQEMATAASSSSLEK
>	sp|P63259|ACT Actin, cytoplasmic 2 OS=Rattus norvegicus GN=Actg1 PE=1 SV=1	VAPEEHPVLLTEAPLNPK
>	sp|P63259|ACT Actin, cytoplasmic 2 OS=Rattus norvegicus GN=Actg1 PE=1 SV=1	HQGVMVGMGQK
>	sp|P63259|ACT Actin, cytoplasmic 2 OS=Rattus norvegicus GN=Actg1 PE=1 SV=1	AVFPSIVGRPR
>	sp|P63259|ACT Actin, cytoplasmic 2 OS=Rattus norvegicus GN=Actg1 PE=1 SV=1	YPIEHGIVTNWDDMEK
>	sp|P63259|ACT Actin, cytoplasmic 2 OS=Rattus norvegicus GN=Actg1 PE=1 SV=1	TTGIVMDSGDGVTHTVPIYEGYALPHAILR
>	sp|P63259|ACT Actin, cytoplasmic 2 OS=Rattus norvegicus GN=Actg1 PE=1 SV=1	LCYVALDFEQEMATAASSSSLEK
>	sp|P63259|ACT Actin, cytoplasmic 2 OS=Rattus norvegicus GN=Actg1 PE=1 SV=1	GILTLK
>	sp|P63259|ACT Actin, cytoplasmic 2 OS=Rattus norvegicus GN=Actg1 PE=1 SV=1	DLYANTVLSGGTTMYPGIADR
>	sp|P63259|ACT Actin, cytoplasmic 2 OS=Rattus norvegicus GN=Actg1 PE=1 SV=1	HQGVMVGMGQK
>	sp|P63259|ACT Actin, cytoplasmic 2 OS=Rattus norvegicus GN=Actg1 PE=1 SV=1	KDLYANTVLSGGTTMYPGIADR
>	sp|P63259|ACT Actin, cytoplasmic 2 OS=Rattus norvegicus GN=Actg1 PE=1 SV=1	LDLAGR
>	sp|P63259|ACT Actin, cytoplasmic 2 OS=Rattus norvegicus GN=Actg1 PE=1 SV=1	EITALAPSTMK
>	sp|P63259|ACT Actin, cytoplasmic 2 OS=Rattus norvegicus GN=Actg1 PE=1 SV=1	IWHHTFYNELR
>	sp|P15999|ATF ATP synthase subunit alpha, mitochondrial OS=Rattus norvegicus GN=Atp5a1 PE=1 SV=2	AVDSLVPIGR
>	sp|P15999|ATF ATP synthase subunit alpha, mitochondrial OS=Rattus norvegicus GN=Atp5a1 PE=1 SV=2	EAYPGDVFYLHSR
>	sp|P15999|ATF ATP synthase subunit alpha, mitochondrial OS=Rattus norvegicus GN=Atp5a1 PE=1 SV=2	EVAAFAQFGSDLDAATQQLLSR
>	sp|P15999|ATF ATP synthase subunit alpha, mitochondrial OS=Rattus norvegicus GN=Atp5a1 PE=1 SV=2	GIRPAINVGLSVSR
>	sp|P15999|ATF ATP synthase subunit alpha, mitochondrial OS=Rattus norvegicus GN=Atp5a1 PE=1 SV=2	ILGADTSVDLEETGR
>	sp|P15999|ATF ATP synthase subunit alpha, mitochondrial OS=Rattus norvegicus GN=Atp5a1 PE=1 SV=2	ISEQSDAK
>	sp|P15999|ATF ATP synthase subunit alpha, mitochondrial OS=Rattus norvegicus GN=Atp5a1 PE=1 SV=2	ITKFESAFLSHVVSQHQSLLGNIR
>	sp|P15999|ATF ATP synthase subunit alpha, mitochondrial OS=Rattus norvegicus GN=Atp5a1 PE=1 SV=2	KLYCIYVAIGQK
>	sp|P15999|ATF ATP synthase subunit alpha, mitochondrial OS=Rattus norvegicus GN=Atp5a1 PE=1 SV=2	LKEIVTNFLAGFEP
>	sp|P15999|ATF ATP synthase subunit alpha, mitochondrial OS=Rattus norvegicus GN=Atp5a1 PE=1 SV=2	LYCIYVAIGQK
>	sp|P15999|ATF ATP synthase subunit alpha, mitochondrial OS=Rattus norvegicus GN=Atp5a1 PE=1 SV=2	NVQAEEMVEFSSGLK
>	sp|P15999|ATF ATP synthase subunit alpha, mitochondrial OS=Rattus norvegicus GN=Atp5a1 PE=1 SV=2	QGQYSPMAIEEQVAVIYAGVR
>	sp|P15999|ATF ATP synthase subunit alpha, mitochondrial OS=Rattus norvegicus GN=Atp5a1 PE=1 SV=2	SDGKISEQSDAK
>	sp|P15999|ATF ATP synthase subunit alpha, mitochondrial OS=Rattus norvegicus GN=Atp5a1 PE=1 SV=2	TGAIVDVPVGDELLGR
>	sp|P15999|ATF ATP synthase subunit alpha, mitochondrial OS=Rattus norvegicus GN=Atp5a1 PE=1 SV=2	TGTAEMSSILEER
>	sp|P15999|ATF ATP synthase subunit alpha, mitochondrial OS=Rattus norvegicus GN=Atp5a1 PE=1 SV=2	TSIAIDTIINQK
>	sp|P15999|ATF ATP synthase subunit alpha, mitochondrial OS=Rattus norvegicus GN=Atp5a1 PE=1 SV=2	VLSIGDGIAR
>	sp|P15999|ATF ATP synthase subunit alpha, mitochondrial OS=Rattus norvegicus GN=Atp5a1 PE=1 SV=2	VVDALGNAIDGK
>	sp|P15999|ATF ATP synthase subunit alpha, mitochondrial OS=Rattus norvegicus GN=Atp5a1 PE=1 SV=2	DNGKHALIIYDDLSK
>	sp|P15999|ATF ATP synthase subunit alpha, mitochondrial OS=Rattus norvegicus GN=Atp5a1 PE=1 SV=2	YTIVVSATASDAAPLQYLAPYSGCSMGEYFR
>	sp|P15999|ATF ATP synthase subunit alpha, mitochondrial OS=Rattus norvegicus GN=Atp5a1 PE=1 SV=2	GMSLNLEPDNVGVVVFGNDK
>	sp|P15999|ATF ATP synthase subunit alpha, mitochondrial OS=Rattus norvegicus GN=Atp5a1 PE=1 SV=2	AVDSLVPIGR
>	sp|P15999|ATF ATP synthase subunit alpha, mitochondrial OS=Rattus norvegicus GN=Atp5a1 PE=1 SV=2	AVDSLVPIGR
>	sp|P15999|ATF ATP synthase subunit alpha, mitochondrial OS=Rattus norvegicus GN=Atp5a1 PE=1 SV=2	DNGKHALIIYDDLSK
>	sp|P15999|ATF ATP synthase subunit alpha, mitochondrial OS=Rattus norvegicus GN=Atp5a1 PE=1 SV=2	DNGKHALIIYDDLSK
>	sp|P15999|ATF ATP synthase subunit alpha, mitochondrial OS=Rattus norvegicus GN=Atp5a1 PE=1 SV=2	EAYPGDVFYLHSR
>	sp|P15999|ATF ATP synthase subunit alpha, mitochondrial OS=Rattus norvegicus GN=Atp5a1 PE=1 SV=2	EAYPGDVFYLHSR
>	sp|P15999|ATF ATP synthase subunit alpha, mitochondrial OS=Rattus norvegicus GN=Atp5a1 PE=1 SV=2	EVAAFAQFGSDLDAATQQLLSR
>	sp|P15999|ATF ATP synthase subunit alpha, mitochondrial OS=Rattus norvegicus GN=Atp5a1 PE=1 SV=2	EVAAFAQFGSDLDAATQQLLSR
>	sp|P15999|ATF ATP synthase subunit alpha, mitochondrial OS=Rattus norvegicus GN=Atp5a1 PE=1 SV=2	EVAAFAQFGSDLDAATQQLLSR
>	sp|P15999|ATF ATP synthase subunit alpha, mitochondrial OS=Rattus norvegicus GN=Atp5a1 PE=1 SV=2	EVAAFAQFGSDLDAATQQLLSR
>	sp|P15999|ATF ATP synthase subunit alpha, mitochondrial OS=Rattus norvegicus GN=Atp5a1 PE=1 SV=2	GIRPAINVGLSVSR
>	sp|P15999|ATF ATP synthase subunit alpha, mitochondrial OS=Rattus norvegicus GN=Atp5a1 PE=1 SV=2	GIRPAINVGLSVSR
>	sp|P15999|ATF ATP synthase subunit alpha, mitochondrial OS=Rattus norvegicus GN=Atp5a1 PE=1 SV=2	GIRPAINVGLSVSR
>	sp|P15999|ATF ATP synthase subunit alpha, mitochondrial OS=Rattus norvegicus GN=Atp5a1 PE=1 SV=2	GIRPAINVGLSVSR
>	sp|P15999|ATF ATP synthase subunit alpha, mitochondrial OS=Rattus norvegicus GN=Atp5a1 PE=1 SV=2	GIRPAINVGLSVSR
>	sp|P15999|ATF ATP synthase subunit alpha, mitochondrial OS=Rattus norvegicus GN=Atp5a1 PE=1 SV=2	GIRPAINVGLSVSR
>	sp|P15999|ATF ATP synthase subunit alpha, mitochondrial OS=Rattus norvegicus GN=Atp5a1 PE=1 SV=2	GIRPAINVGLSVSR
>	sp|P15999|ATF ATP synthase subunit alpha, mitochondrial OS=Rattus norvegicus GN=Atp5a1 PE=1 SV=2	GIRPAINVGLSVSR
>	sp|P15999|ATF ATP synthase subunit alpha, mitochondrial OS=Rattus norvegicus GN=Atp5a1 PE=1 SV=2	GIRPAINVGLSVSR
>	sp|P15999|ATF ATP synthase subunit alpha, mitochondrial OS=Rattus norvegicus GN=Atp5a1 PE=1 SV=2	GIRPAINVGLSVSR
>	sp|P15999|ATF ATP synthase subunit alpha, mitochondrial OS=Rattus norvegicus GN=Atp5a1 PE=1 SV=2	GIRPAINVGLSVSR
>	sp|P15999|ATF ATP synthase subunit alpha, mitochondrial OS=Rattus norvegicus GN=Atp5a1 PE=1 SV=2	GIRPAINVGLSVSR
>	sp|P15999|ATF ATP synthase subunit alpha, mitochondrial OS=Rattus norvegicus GN=Atp5a1 PE=1 SV=2	GIRPAINVGLSVSR
>	sp|P15999|ATF ATP synthase subunit alpha, mitochondrial OS=Rattus norvegicus GN=Atp5a1 PE=1 SV=2	ILGADTSVDLEETGR
>	sp|P15999|ATF ATP synthase subunit alpha, mitochondrial OS=Rattus norvegicus GN=Atp5a1 PE=1 SV=2	ILGADTSVDLEETGR
>	sp|P15999|ATF ATP synthase subunit alpha, mitochondrial OS=Rattus norvegicus GN=Atp5a1 PE=1 SV=2	ITKFESAFLSHVVSQHQSLLGNIR
>	sp|P15999|ATF ATP synthase subunit alpha, mitochondrial OS=Rattus norvegicus GN=Atp5a1 PE=1 SV=2	KLYCIYVAIGQK
>	sp|P15999|ATF ATP synthase subunit alpha, mitochondrial OS=Rattus norvegicus GN=Atp5a1 PE=1 SV=2	KLYCIYVAIGQK
>	sp|P15999|ATF ATP synthase subunit alpha, mitochondrial OS=Rattus norvegicus GN=Atp5a1 PE=1 SV=2	KLYCIYVAIGQK
>	sp|P15999|ATF ATP synthase subunit alpha, mitochondrial OS=Rattus norvegicus GN=Atp5a1 PE=1 SV=2	LKEIVTNFLAGFEP
>	sp|P15999|ATF ATP synthase subunit alpha, mitochondrial OS=Rattus norvegicus GN=Atp5a1 PE=1 SV=2	LYCIYVAIGQK
>	sp|P15999|ATF ATP synthase subunit alpha, mitochondrial OS=Rattus norvegicus GN=Atp5a1 PE=1 SV=2	LYCIYVAIGQK
>	sp|P15999|ATF ATP synthase subunit alpha, mitochondrial OS=Rattus norvegicus GN=Atp5a1 PE=1 SV=2	NVQAEEMVEFSSGLK
>	sp|P15999|ATF ATP synthase subunit alpha, mitochondrial OS=Rattus norvegicus GN=Atp5a1 PE=1 SV=2	NVQAEEMVEFSSGLK
>	sp|P15999|ATF ATP synthase subunit alpha, mitochondrial OS=Rattus norvegicus GN=Atp5a1 PE=1 SV=2	QGQYSPMAIEEQVAVIYAGVR
>	sp|P15999|ATF ATP synthase subunit alpha, mitochondrial OS=Rattus norvegicus GN=Atp5a1 PE=1 SV=2	QGQYSPMAIEEQVAVIYAGVR
>	sp|P15999|ATF ATP synthase subunit alpha, mitochondrial OS=Rattus norvegicus GN=Atp5a1 PE=1 SV=2	TGAIVDVPVGDELLGR

<table>
<tr><td>></td><td>sp|P15999|ATF ATP synthase subunit alpha, mitochondrial OS=Rattus norvegicus GN=Atp5a1 PE=1 SV=2</td><td>TGAIVDVPVGDELLGR</td></tr>
<tr><td>></td><td>sp|P15999|ATF ATP synthase subunit alpha, mitochondrial OS=Rattus norvegicus GN=Atp5a1 PE=1 SV=2</td><td>TGAIVDVPVGDELLGR</td></tr>
<tr><td>></td><td>sp|P15999|ATF ATP synthase subunit alpha, mitochondrial OS=Rattus norvegicus GN=Atp5a1 PE=1 SV=2</td><td>TGTAEMSSILEER</td></tr>
<tr><td>></td><td>sp|P15999|ATF ATP synthase subunit alpha, mitochondrial OS=Rattus norvegicus GN=Atp5a1 PE=1 SV=2</td><td>TGTAEMSSILEER</td></tr>
<tr><td>></td><td>sp|P15999|ATF ATP synthase subunit alpha, mitochondrial OS=Rattus norvegicus GN=Atp5a1 PE=1 SV=2</td><td>TGTAEMSSILEER</td></tr>
<tr><td>></td><td>sp|P15999|ATF ATP synthase subunit alpha, mitochondrial OS=Rattus norvegicus GN=Atp5a1 PE=1 SV=2</td><td>TGTAEMSSILEER</td></tr>
<tr><td>></td><td>sp|P15999|ATF ATP synthase subunit alpha, mitochondrial OS=Rattus norvegicus GN=Atp5a1 PE=1 SV=2</td><td>TGTAEMSSILEER</td></tr>
<tr><td>></td><td>sp|P15999|ATF ATP synthase subunit alpha, mitochondrial OS=Rattus norvegicus GN=Atp5a1 PE=1 SV=2</td><td>TGTAEMSSILEER</td></tr>
<tr><td>></td><td>sp|P15999|ATF ATP synthase subunit alpha, mitochondrial OS=Rattus norvegicus GN=Atp5a1 PE=1 SV=2</td><td>TGTAEMSSILEER</td></tr>
<tr><td>></td><td>sp|P15999|ATF ATP synthase subunit alpha, mitochondrial OS=Rattus norvegicus GN=Atp5a1 PE=1 SV=2</td><td>VVDALGNAIDGK</td></tr>
<tr><td>></td><td>sp|P15999|ATF ATP synthase subunit alpha, mitochondrial OS=Rattus norvegicus GN=Atp5a1 PE=1 SV=2</td><td>VVDALGNAIDGK</td></tr>
<tr><td>></td><td>sp|P15999|ATF ATP synthase subunit alpha, mitochondrial OS=Rattus norvegicus GN=Atp5a1 PE=1 SV=2</td><td>VVDALGNAIDGK</td></tr>
<tr><td>></td><td>sp|P15999|ATF ATP synthase subunit alpha, mitochondrial OS=Rattus norvegicus GN=Atp5a1 PE=1 SV=2</td><td>VVDALGNAIDGK</td></tr>
<tr><td>></td><td>sp|P15999|ATF ATP synthase subunit alpha, mitochondrial OS=Rattus norvegicus GN=Atp5a1 PE=1 SV=2</td><td>VVDALGNAIDGK</td></tr>
<tr><td>></td><td>sp|P15999|ATF ATP synthase subunit alpha, mitochondrial OS=Rattus norvegicus GN=Atp5a1 PE=1 SV=2</td><td>ITKFESAFLSHVVSQHQSLLGNIR</td></tr>
<tr><td>></td><td>sp|P15999|ATF ATP synthase subunit alpha, mitochondrial OS=Rattus norvegicus GN=Atp5a1 PE=1 SV=2</td><td>ITKFESAFLSHVVSQHQSLLGNIR</td></tr>
<tr><td>></td><td>sp|P15999|ATF ATP synthase subunit alpha, mitochondrial OS=Rattus norvegicus GN=Atp5a1 PE=1 SV=2</td><td>VVDALGNAIDGK</td></tr>
<tr><td>></td><td>sp|P15999|ATF ATP synthase subunit alpha, mitochondrial OS=Rattus norvegicus GN=Atp5a1 PE=1 SV=2</td><td>EGDIVKR</td></tr>
<tr><td>></td><td>sp|P15999|ATF ATP synthase subunit alpha, mitochondrial OS=Rattus norvegicus GN=Atp5a1 PE=1 SV=2</td><td>TGTAEMSSILEER</td></tr>
<tr><td>></td><td>sp|P15999|ATF ATP synthase subunit alpha, mitochondrial OS=Rattus norvegicus GN=Atp5a1 PE=1 SV=2</td><td>LYCIYVAIGQK</td></tr>
<tr><td>></td><td>sp|P15999|ATF ATP synthase subunit alpha, mitochondrial OS=Rattus norvegicus GN=Atp5a1 PE=1 SV=2</td><td>TGTAEMSSILEER</td></tr>
<tr><td>></td><td>sp|P15999|ATF ATP synthase subunit alpha, mitochondrial OS=Rattus norvegicus GN=Atp5a1 PE=1 SV=2</td><td>GMSLNLEPDNVGVVVFGNDKLIK</td></tr>
<tr><td>></td><td>sp|P15999|ATF ATP synthase subunit alpha, mitochondrial OS=Rattus norvegicus GN=Atp5a1 PE=1 SV=2</td><td>VVDALGNAIDGKGPVGSK</td></tr>
<tr><td>></td><td>sp|P15999|ATF ATP synthase subunit alpha, mitochondrial OS=Rattus norvegicus GN=Atp5a1 PE=1 SV=2</td><td>AVDSLVPIGR</td></tr>
<tr><td>></td><td>sp|P15999|ATF ATP synthase subunit alpha, mitochondrial OS=Rattus norvegicus GN=Atp5a1 PE=1 SV=2</td><td>AVDSLVPIGR</td></tr>
<tr><td>></td><td>sp|P15999|ATF ATP synthase subunit alpha, mitochondrial OS=Rattus norvegicus GN=Atp5a1 PE=1 SV=2</td><td>YTIVVSATASDAAPLQYLAPYSGCSMGEYFR</td></tr>
<tr><td>></td><td>sp|P15999|ATF ATP synthase subunit alpha, mitochondrial OS=Rattus norvegicus GN=Atp5a1 PE=1 SV=2</td><td>LYCIYVAIGQK</td></tr>
<tr><td>></td><td>sp|P15999|ATF ATP synthase subunit alpha, mitochondrial OS=Rattus norvegicus GN=Atp5a1 PE=1 SV=2</td><td>NVQAEEMVEFSSGLK</td></tr>
<tr><td>></td><td>sp|P15999|ATF ATP synthase subunit alpha, mitochondrial OS=Rattus norvegicus GN=Atp5a1 PE=1 SV=2</td><td>VLSIGDGIAR</td></tr>
<tr><td>></td><td>sp|P15999|ATF ATP synthase subunit alpha, mitochondrial OS=Rattus norvegicus GN=Atp5a1 PE=1 SV=2</td><td>ISEQSDAK</td></tr>
<tr><td>></td><td>sp|P15999|ATF ATP synthase subunit alpha, mitochondrial OS=Rattus norvegicus GN=Atp5a1 PE=1 SV=2</td><td>LTDADAMK</td></tr>
<tr><td>></td><td>sp|P15999|ATF ATP synthase subunit alpha, mitochondrial OS=Rattus norvegicus GN=Atp5a1 PE=1 SV=2</td><td>LELAQYR</td></tr>
<tr><td>></td><td>sp|P15999|ATF ATP synthase subunit alpha, mitochondrial OS=Rattus norvegicus GN=Atp5a1 PE=1 SV=2</td><td>VGLKAPGIIPR</td></tr>
<tr><td>></td><td>sp|P15999|ATF ATP synthase subunit alpha, mitochondrial OS=Rattus norvegicus GN=Atp5a1 PE=1 SV=2</td><td>GIRPAINVGLSVSR</td></tr>
<tr><td>></td><td>sp|P15999|ATF ATP synthase subunit alpha, mitochondrial OS=Rattus norvegicus GN=Atp5a1 PE=1 SV=2</td><td>VVDALGNAIDGK</td></tr>
<tr><td>></td><td>sp|P15999|ATF ATP synthase subunit alpha, mitochondrial OS=Rattus norvegicus GN=Atp5a1 PE=1 SV=2</td><td>VGLKAPGIIPR</td></tr>
<tr><td>></td><td>sp|P15999|ATF ATP synthase subunit alpha, mitochondrial OS=Rattus norvegicus GN=Atp5a1 PE=1 SV=2</td><td>AVDSLVPIGR</td></tr>
<tr><td>></td><td>sp|P15999|ATF ATP synthase subunit alpha, mitochondrial OS=Rattus norvegicus GN=Atp5a1 PE=1 SV=2</td><td>VVDALGNAIDGK</td></tr>
<tr><td>></td><td>sp|P15999|ATF ATP synthase subunit alpha, mitochondrial OS=Rattus norvegicus GN=Atp5a1 PE=1 SV=2</td><td>VLSIGDGIAR</td></tr>
<tr><td>></td><td>sp|P15999|ATF ATP synthase subunit alpha, mitochondrial OS=Rattus norvegicus GN=Atp5a1 PE=1 SV=2</td><td>VVDALGNAIDGK</td></tr>
<tr><td>></td><td>sp|P15999|ATF ATP synthase subunit alpha, mitochondrial OS=Rattus norvegicus GN=Atp5a1 PE=1 SV=2</td><td>VVDALGNAIDGK</td></tr>
<tr><td>></td><td>sp|P15999|ATF ATP synthase subunit alpha, mitochondrial OS=Rattus norvegicus GN=Atp5a1 PE=1 SV=2</td><td>AVDSLVPIGR</td></tr>
<tr><td>></td><td>sp|P15999|ATF ATP synthase subunit alpha, mitochondrial OS=Rattus norvegicus GN=Atp5a1 PE=1 SV=2</td><td>VGLKAPGIIPR</td></tr>
<tr><td>></td><td>sp|P15999|ATF ATP synthase subunit alpha, mitochondrial OS=Rattus norvegicus GN=Atp5a1 PE=1 SV=2</td><td>GMSLNLEPDNVGVVVFGNDK</td></tr>
<tr><td>></td><td>sp|P15999|ATF ATP synthase subunit alpha, mitochondrial OS=Rattus norvegicus GN=Atp5a1 PE=1 SV=2</td><td>LYCIYVAIGQK</td></tr>
<tr><td>></td><td>sp|P15999|ATF ATP synthase subunit alpha, mitochondrial OS=Rattus norvegicus GN=Atp5a1 PE=1 SV=2</td><td>YTIVVSATASDAAPLQYLAPYSGCSMGEYFR</td></tr>
<tr><td>></td><td>sp|P15999|ATF ATP synthase subunit alpha, mitochondrial OS=Rattus norvegicus GN=Atp5a1 PE=1 SV=2</td><td>YTIVVSATASDAAPLQYLAPYSGCSMGEYFR</td></tr>
<tr><td>></td><td>sp|P15999|ATF ATP synthase subunit alpha, mitochondrial OS=Rattus norvegicus GN=Atp5a1 PE=1 SV=2</td><td>NVQAEEMVEFSSGLK</td></tr>
<tr><td>></td><td>sp|P15999|ATF ATP synthase subunit alpha, mitochondrial OS=Rattus norvegicus GN=Atp5a1 PE=1 SV=2</td><td>YTIVVSATASDAAPLQYLAPYSGCSMGEYFR</td></tr>
<tr><td>></td><td>sp|P15999|ATF ATP synthase subunit alpha, mitochondrial OS=Rattus norvegicus GN=Atp5a1 PE=1 SV=2</td><td>HALIIYDDLSK</td></tr>
<tr><td>></td><td>sp|P61765|STX Syntaxin-binding protein 1 OS=Rattus norvegicus GN=Stxbp1 PE=1 SV=1</td><td>AAHVFFTDSCPDALFNELVK</td></tr>
<tr><td>></td><td>sp|P61765|STX Syntaxin-binding protein 1 OS=Rattus norvegicus GN=Stxbp1 PE=1 SV=1</td><td>ADDPTMGEGPDKAR</td></tr>
<tr><td>></td><td>sp|P61765|STX Syntaxin-binding protein 1 OS=Rattus norvegicus GN=Stxbp1 PE=1 SV=1</td><td>AQMKNPILERLAEQIATLCATLKEYPAVR</td></tr>
<tr><td>></td><td>sp|P61765|STX Syntaxin-binding protein 1 OS=Rattus norvegicus GN=Stxbp1 PE=1 SV=1</td><td>AVVGEK</td></tr>
<tr><td>></td><td>sp|P61765|STX Syntaxin-binding protein 1 OS=Rattus norvegicus GN=Stxbp1 PE=1 SV=1</td><td>DIMEDTIEDKLDTK</td></tr>
<tr><td>></td><td>sp|P61765|STX Syntaxin-binding protein 1 OS=Rattus norvegicus GN=Stxbp1 PE=1 SV=1</td><td>GFDPSSPVLHELTFQAMSYDLLPIENDVYKYETSGIGEAR</td></tr>
<tr><td>></td><td>sp|P61765|STX Syntaxin-binding protein 1 OS=Rattus norvegicus GN=Stxbp1 PE=1 SV=1</td><td>HIAEVSQEVTR</td></tr>
<tr><td>></td><td>sp|P61765|STX Syntaxin-binding protein 1 OS=Rattus norvegicus GN=Stxbp1 PE=1 SV=1</td><td>IMHDVIK</td></tr>
<tr><td>></td><td>sp|P61765|STX Syntaxin-binding protein 1 OS=Rattus norvegicus GN=Stxbp1 PE=1 SV=1</td><td>LAEQIATLCATLK</td></tr>
<tr><td>></td><td>sp|P61765|STX Syntaxin-binding protein 1 OS=Rattus norvegicus GN=Stxbp1 PE=1 SV=1</td><td>LAEQIATLCATLKEYPAVR</td></tr>
<tr><td>></td><td>sp|P61765|STX Syntaxin-binding protein 1 OS=Rattus norvegicus GN=Stxbp1 PE=1 SV=1</td><td>LIQHAQIPPEDSEIITNMAHLGVPIVTDSTLR</td></tr>
<tr><td>></td><td>sp|P61765|STX Syntaxin-binding protein 1 OS=Rattus norvegicus GN=Stxbp1 PE=1 SV=1</td><td>MTDIMTEGITIVEDINKR</td></tr>
<tr><td>></td><td>sp|P61765|STX Syntaxin-binding protein 1 OS=Rattus norvegicus GN=Stxbp1 PE=1 SV=1</td><td>REPLPSLEAVYLITPSEK</td></tr>
<tr><td>></td><td>sp|P61765|STX Syntaxin-binding protein 1 OS=Rattus norvegicus GN=Stxbp1 PE=1 SV=1</td><td>SQLLILDR</td></tr>
<tr><td>></td><td>sp|P61765|STX Syntaxin-binding protein 1 OS=Rattus norvegicus GN=Stxbp1 PE=1 SV=1</td><td>SVHSLISDFKDPPTAK</td></tr>
<tr><td>></td><td>sp|P61765|STX Syntaxin-binding protein 1 OS=Rattus norvegicus GN=Stxbp1 PE=1 SV=1</td><td>TLTEINIAFLPYESQVYSLDSADSFQSFYSPHK</td></tr>
<tr><td>></td><td>sp|P61765|STX Syntaxin-binding protein 1 OS=Rattus norvegicus GN=Stxbp1 PE=1 SV=1</td><td>VEQDLAMGTDAEGEK</td></tr>
<tr><td>></td><td>sp|P61765|STX Syntaxin-binding protein 1 OS=Rattus norvegicus GN=Stxbp1 PE=1 SV=1</td><td>VKEVLLDEDDDLWIALR</td></tr>
<tr><td>></td><td>sp|P61765|STX Syntaxin-binding protein 1 OS=Rattus norvegicus GN=Stxbp1 PE=1 SV=1</td><td>WEVLIGSTHILTPQK</td></tr>
<tr><td>></td><td>sp|P61765|STX Syntaxin-binding protein 1 OS=Rattus norvegicus GN=Stxbp1 PE=1 SV=1</td><td>WTPIIKDIMEDTIEDKLDTK</td></tr>
<tr><td>></td><td>sp|P61765|STX Syntaxin-binding protein 1 OS=Rattus norvegicus GN=Stxbp1 PE=1 SV=1</td><td>YRGEYKDNALLAQLIQDKLDAYK</td></tr>
<tr><td>></td><td>sp|P61765|STX Syntaxin-binding protein 1 OS=Rattus norvegicus GN=Stxbp1 PE=1 SV=1</td><td>DNALLAQLIQDKLDAYK</td></tr>
<tr><td>></td><td>sp|P61765|STX Syntaxin-binding protein 1 OS=Rattus norvegicus GN=Stxbp1 PE=1 SV=1</td><td>AAHVFFTDSCPDALFNELVK</td></tr>
<tr><td>></td><td>sp|P61765|STX Syntaxin-binding protein 1 OS=Rattus norvegicus GN=Stxbp1 PE=1 SV=1</td><td>AAHVFFTDSCPDALFNELVK</td></tr>
</table>

>	sp|P61765|STX Syntaxin-binding protein 1 OS=Rattus norvegicus GN=Stxbp1 PE=1 SV=1	AAHVFFTDSCPDALFNELVK
>	sp|P61765|STX Syntaxin-binding protein 1 OS=Rattus norvegicus GN=Stxbp1 PE=1 SV=1	AAHVFFTDSCPDALFNELVK
>	sp|P61765|STX Syntaxin-binding protein 1 OS=Rattus norvegicus GN=Stxbp1 PE=1 SV=1	AAHVFFTDSCPDALFNELVK
>	sp|P61765|STX Syntaxin-binding protein 1 OS=Rattus norvegicus GN=Stxbp1 PE=1 SV=1	AAHVFFTDSCPDALFNELVK
>	sp|P61765|STX Syntaxin-binding protein 1 OS=Rattus norvegicus GN=Stxbp1 PE=1 SV=1	AAHVFFTDSCPDALFNELVK
>	sp|P61765|STX Syntaxin-binding protein 1 OS=Rattus norvegicus GN=Stxbp1 PE=1 SV=1	AAHVFFTDSCPDALFNELVK
>	sp|P61765|STX Syntaxin-binding protein 1 OS=Rattus norvegicus GN=Stxbp1 PE=1 SV=1	AAHVFFTDSCPDALFNELVK
>	sp|P61765|STX Syntaxin-binding protein 1 OS=Rattus norvegicus GN=Stxbp1 PE=1 SV=1	AAHVFFTDSCPDALFNELVK
>	sp|P61765|STX Syntaxin-binding protein 1 OS=Rattus norvegicus GN=Stxbp1 PE=1 SV=1	AAHVFFTDSCPDALFNELVK
>	sp|P61765|STX Syntaxin-binding protein 1 OS=Rattus norvegicus GN=Stxbp1 PE=1 SV=1	AAHVFFTDSCPDALFNELVK
>	sp|P61765|STX Syntaxin-binding protein 1 OS=Rattus norvegicus GN=Stxbp1 PE=1 SV=1	AAHVFFTDSCPDALFNELVK
>	sp|P61765|STX Syntaxin-binding protein 1 OS=Rattus norvegicus GN=Stxbp1 PE=1 SV=1	AAHVFFTDSCPDALFNELVK
>	sp|P61765|STX Syntaxin-binding protein 1 OS=Rattus norvegicus GN=Stxbp1 PE=1 SV=1	AAHVFFTDSCPDALFNELVK
>	sp|P61765|STX Syntaxin-binding protein 1 OS=Rattus norvegicus GN=Stxbp1 PE=1 SV=1	AAHVFFTDSCPDALFNELVK
>	sp|P61765|STX Syntaxin-binding protein 1 OS=Rattus norvegicus GN=Stxbp1 PE=1 SV=1	AAHVFFTDSCPDALFNELVK
>	sp|P61765|STX Syntaxin-binding protein 1 OS=Rattus norvegicus GN=Stxbp1 PE=1 SV=1	AAHVFFTDSCPDALFNELVK
>	sp|P61765|STX Syntaxin-binding protein 1 OS=Rattus norvegicus GN=Stxbp1 PE=1 SV=1	AAHVFFTDSCPDALFNELVK
>	sp|P61765|STX Syntaxin-binding protein 1 OS=Rattus norvegicus GN=Stxbp1 PE=1 SV=1	AAHVFFTDSCPDALFNELVK
>	sp|P61765|STX Syntaxin-binding protein 1 OS=Rattus norvegicus GN=Stxbp1 PE=1 SV=1	AAHVFFTDSCPDALFNELVK
>	sp|P61765|STX Syntaxin-binding protein 1 OS=Rattus norvegicus GN=Stxbp1 PE=1 SV=1	AAHVFFTDSCPDALFNELVK
>	sp|P61765|STX Syntaxin-binding protein 1 OS=Rattus norvegicus GN=Stxbp1 PE=1 SV=1	AAHVFFTDSCPDALFNELVK
>	sp|P61765|STX Syntaxin-binding protein 1 OS=Rattus norvegicus GN=Stxbp1 PE=1 SV=1	AAHVFFTDSCPDALFNELVK
>	sp|P61765|STX Syntaxin-binding protein 1 OS=Rattus norvegicus GN=Stxbp1 PE=1 SV=1	AQMKNPILERLAEQIATLCATLKEYPAVR
>	sp|P61765|STX Syntaxin-binding protein 1 OS=Rattus norvegicus GN=Stxbp1 PE=1 SV=1	AQMKNPILERLAEQIATLCATLKEYPAVR
>	sp|P61765|STX Syntaxin-binding protein 1 OS=Rattus norvegicus GN=Stxbp1 PE=1 SV=1	AQMKNPILERLAEQIATLCATLKEYPAVR
>	sp|P61765|STX Syntaxin-binding protein 1 OS=Rattus norvegicus GN=Stxbp1 PE=1 SV=1	AQMKNPILERLAEQIATLCATLKEYPAVR
>	sp|P61765|STX Syntaxin-binding protein 1 OS=Rattus norvegicus GN=Stxbp1 PE=1 SV=1	AQMKNPILERLAEQIATLCATLKEYPAVR
>	sp|P61765|STX Syntaxin-binding protein 1 OS=Rattus norvegicus GN=Stxbp1 PE=1 SV=1	AQMKNPILERLAEQIATLCATLKEYPAVR
>	sp|P61765|STX Syntaxin-binding protein 1 OS=Rattus norvegicus GN=Stxbp1 PE=1 SV=1	DNALLAQLIQDKLDAYK
>	sp|P61765|STX Syntaxin-binding protein 1 OS=Rattus norvegicus GN=Stxbp1 PE=1 SV=1	GFDPSSPVLHELTFQAMSYDLLPIENDVYKYETSGIGEAR
>	sp|P61765|STX Syntaxin-binding protein 1 OS=Rattus norvegicus GN=Stxbp1 PE=1 SV=1	GFDPSSPVLHELTFQAMSYDLLPIENDVYKYETSGIGEAR
>	sp|P61765|STX Syntaxin-binding protein 1 OS=Rattus norvegicus GN=Stxbp1 PE=1 SV=1	GFDPSSPVLHELTFQAMSYDLLPIENDVYKYETSGIGEAR
>	sp|P61765|STX Syntaxin-binding protein 1 OS=Rattus norvegicus GN=Stxbp1 PE=1 SV=1	GFDPSSPVLHELTFQAMSYDLLPIENDVYKYETSGIGEAR
>	sp|P61765|STX Syntaxin-binding protein 1 OS=Rattus norvegicus GN=Stxbp1 PE=1 SV=1	GFDPSSPVLHELTFQAMSYDLLPIENDVYKYETSGIGEAR
>	sp|P61765|STX Syntaxin-binding protein 1 OS=Rattus norvegicus GN=Stxbp1 PE=1 SV=1	HIAEVSQEVTR
>	sp|P61765|STX Syntaxin-binding protein 1 OS=Rattus norvegicus GN=Stxbp1 PE=1 SV=1	HIAEVSQEVTR
>	sp|P61765|STX Syntaxin-binding protein 1 OS=Rattus norvegicus GN=Stxbp1 PE=1 SV=1	HIAEVSQEVTR
>	sp|P61765|STX Syntaxin-binding protein 1 OS=Rattus norvegicus GN=Stxbp1 PE=1 SV=1	HIAEVSQEVTR
>	sp|P61765|STX Syntaxin-binding protein 1 OS=Rattus norvegicus GN=Stxbp1 PE=1 SV=1	HIAEVSQEVTR
>	sp|P61765|STX Syntaxin-binding protein 1 OS=Rattus norvegicus GN=Stxbp1 PE=1 SV=1	IMHDVIK
>	sp|P61765|STX Syntaxin-binding protein 1 OS=Rattus norvegicus GN=Stxbp1 PE=1 SV=1	LAEQIATLCATLK
>	sp|P61765|STX Syntaxin-binding protein 1 OS=Rattus norvegicus GN=Stxbp1 PE=1 SV=1	LAEQIATLCATLK
>	sp|P61765|STX Syntaxin-binding protein 1 OS=Rattus norvegicus GN=Stxbp1 PE=1 SV=1	LAEQIATLCATLK
>	sp|P61765|STX Syntaxin-binding protein 1 OS=Rattus norvegicus GN=Stxbp1 PE=1 SV=1	LAEQIATLCATLK
>	sp|P61765|STX Syntaxin-binding protein 1 OS=Rattus norvegicus GN=Stxbp1 PE=1 SV=1	LAEQIATLCATLK
>	sp|P61765|STX Syntaxin-binding protein 1 OS=Rattus norvegicus GN=Stxbp1 PE=1 SV=1	LAEQIATLCATLKEYPAVR
>	sp|P61765|STX Syntaxin-binding protein 1 OS=Rattus norvegicus GN=Stxbp1 PE=1 SV=1	LAEQIATLCATLKEYPAVR
>	sp|P61765|STX Syntaxin-binding protein 1 OS=Rattus norvegicus GN=Stxbp1 PE=1 SV=1	LIQHAQIPPEDSEIITNMAHLGVPIVTDSTLR
>	sp|P61765|STX Syntaxin-binding protein 1 OS=Rattus norvegicus GN=Stxbp1 PE=1 SV=1	LIQHAQIPPEDSEIITNMAHLGVPIVTDSTLR
>	sp|P61765|STX Syntaxin-binding protein 1 OS=Rattus norvegicus GN=Stxbp1 PE=1 SV=1	LIQHAQIPPEDSEIITNMAHLGVPIVTDSTLR
>	sp|P61765|STX Syntaxin-binding protein 1 OS=Rattus norvegicus GN=Stxbp1 PE=1 SV=1	LIQHAQIPPEDSEIITNMAHLGVPIVTDSTLR
>	sp|P61765|STX Syntaxin-binding protein 1 OS=Rattus norvegicus GN=Stxbp1 PE=1 SV=1	MTDIMTEGITIVEDINKR
>	sp|P61765|STX Syntaxin-binding protein 1 OS=Rattus norvegicus GN=Stxbp1 PE=1 SV=1	MTDIMTEGITIVEDINKR
>	sp|P61765|STX Syntaxin-binding protein 1 OS=Rattus norvegicus GN=Stxbp1 PE=1 SV=1	MTDIMTEGITIVEDINKR
>	sp|P61765|STX Syntaxin-binding protein 1 OS=Rattus norvegicus GN=Stxbp1 PE=1 SV=1	REPLPSLEAVYLITPSEK
>	sp|P61765|STX Syntaxin-binding protein 1 OS=Rattus norvegicus GN=Stxbp1 PE=1 SV=1	REPLPSLEAVYLITPSEK
>	sp|P61765|STX Syntaxin-binding protein 1 OS=Rattus norvegicus GN=Stxbp1 PE=1 SV=1	REPLPSLEAVYLITPSEK
>	sp|P61765|STX Syntaxin-binding protein 1 OS=Rattus norvegicus GN=Stxbp1 PE=1 SV=1	REPLPSLEAVYLITPSEK
>	sp|P61765|STX Syntaxin-binding protein 1 OS=Rattus norvegicus GN=Stxbp1 PE=1 SV=1	REPLPSLEAVYLITPSEK
>	sp|P61765|STX Syntaxin-binding protein 1 OS=Rattus norvegicus GN=Stxbp1 PE=1 SV=1	SQLLILDR
>	sp|P61765|STX Syntaxin-binding protein 1 OS=Rattus norvegicus GN=Stxbp1 PE=1 SV=1	SVHSLISDFKDPPTAK
>	sp|P61765|STX Syntaxin-binding protein 1 OS=Rattus norvegicus GN=Stxbp1 PE=1 SV=1	VEQDLAMGTDAEGEK
>	sp|P61765|STX Syntaxin-binding protein 1 OS=Rattus norvegicus GN=Stxbp1 PE=1 SV=1	VKEVLLDEDDDLWIALR
>	sp|P61765|STX Syntaxin-binding protein 1 OS=Rattus norvegicus GN=Stxbp1 PE=1 SV=1	VKEVLLDEDDDLWIALR
>	sp|P61765|STX Syntaxin-binding protein 1 OS=Rattus norvegicus GN=Stxbp1 PE=1 SV=1	VKEVLLDEDDDLWIALR
>	sp|P61765|STX Syntaxin-binding protein 1 OS=Rattus norvegicus GN=Stxbp1 PE=1 SV=1	VKEVLLDEDDDLWIALR
>	sp|P61765|STX Syntaxin-binding protein 1 OS=Rattus norvegicus GN=Stxbp1 PE=1 SV=1	VKEVLLDEDDDLWIALR
>	sp|P61765|STX Syntaxin-binding protein 1 OS=Rattus norvegicus GN=Stxbp1 PE=1 SV=1	WEVLIGSTHILTPQK
>	sp|P61765|STX Syntaxin-binding protein 1 OS=Rattus norvegicus GN=Stxbp1 PE=1 SV=1	WTPIIKDIMEDTIEDKLDTK
>	sp|P61765|STX Syntaxin-binding protein 1 OS=Rattus norvegicus GN=Stxbp1 PE=1 SV=1	WTPIIKDIMEDTIEDKLDTK
>	sp|P61765|STX Syntaxin-binding protein 1 OS=Rattus norvegicus GN=Stxbp1 PE=1 SV=1	WTPIIKDIMEDTIEDKLDTK
>	sp|P61765|STX Syntaxin-binding protein 1 OS=Rattus norvegicus GN=Stxbp1 PE=1 SV=1	WTPIIKDIMEDTIEDKLDTK
>	sp|P61765|STX Syntaxin-binding protein 1 OS=Rattus norvegicus GN=Stxbp1 PE=1 SV=1	WTPIIKDIMEDTIEDKLDTK
>	sp|P61765|STX Syntaxin-binding protein 1 OS=Rattus norvegicus GN=Stxbp1 PE=1 SV=1	WTPIIKDIMEDTIEDKLDTK

>	sp|P61765|STX Syntaxin-binding protein 1 OS=Rattus norvegicus GN=Stxbp1 PE=1 SV=1	WTPIIKDIMEDTIEDKLDTK
>	sp|P61765|STX Syntaxin-binding protein 1 OS=Rattus norvegicus GN=Stxbp1 PE=1 SV=1	WTPIIKDIMEDTIEDKLDTK
>	sp|P61765|STX Syntaxin-binding protein 1 OS=Rattus norvegicus GN=Stxbp1 PE=1 SV=1	WTPIIKDIMEDTIEDKLDTK
>	sp|P61765|STX Syntaxin-binding protein 1 OS=Rattus norvegicus GN=Stxbp1 PE=1 SV=1	WTPIIKDIMEDTIEDKLDTK
>	sp|P61765|STX Syntaxin-binding protein 1 OS=Rattus norvegicus GN=Stxbp1 PE=1 SV=1	WTPIIKDIMEDTIEDKLDTK
>	sp|P61765|STX Syntaxin-binding protein 1 OS=Rattus norvegicus GN=Stxbp1 PE=1 SV=1	YRGEYKDNALLAQLIQDKLDAYK
>	sp|P61765|STX Syntaxin-binding protein 1 OS=Rattus norvegicus GN=Stxbp1 PE=1 SV=1	YRGEYKDNALLAQLIQDKLDAYK
>	sp|P61765|STX Syntaxin-binding protein 1 OS=Rattus norvegicus GN=Stxbp1 PE=1 SV=1	YRGEYKDNALLAQLIQDKLDAYK
>	sp|P61765|STX Syntaxin-binding protein 1 OS=Rattus norvegicus GN=Stxbp1 PE=1 SV=1	WTPIIKDIMEDTIEDKLDTK
>	sp|P61765|STX Syntaxin-binding protein 1 OS=Rattus norvegicus GN=Stxbp1 PE=1 SV=1	REPLPSLEAVYLITPSEK
>	sp|P61765|STX Syntaxin-binding protein 1 OS=Rattus norvegicus GN=Stxbp1 PE=1 SV=1	VEQDLAMGTDAEGEK
>	sp|P61765|STX Syntaxin-binding protein 1 OS=Rattus norvegicus GN=Stxbp1 PE=1 SV=1	MTDIMTEGITIVEDINK
>	sp|P61765|STX Syntaxin-binding protein 1 OS=Rattus norvegicus GN=Stxbp1 PE=1 SV=1	LIQHAQIPPEDSEIITNMAHLGVPIVTDSTLR
>	sp|P61765|STX Syntaxin-binding protein 1 OS=Rattus norvegicus GN=Stxbp1 PE=1 SV=1	REPLPSLEAVYLITPSEK
>	sp|P61765|STX Syntaxin-binding protein 1 OS=Rattus norvegicus GN=Stxbp1 PE=1 SV=1	REPLPSLEAVYLITPSEK
>	sp|P61765|STX Syntaxin-binding protein 1 OS=Rattus norvegicus GN=Stxbp1 PE=1 SV=1	VKEVLLDEDDDLWIALR
>	sp|P61765|STX Syntaxin-binding protein 1 OS=Rattus norvegicus GN=Stxbp1 PE=1 SV=1	VKEVLLDEDDDLWIALR
>	sp|P61765|STX Syntaxin-binding protein 1 OS=Rattus norvegicus GN=Stxbp1 PE=1 SV=1	MTDIMTEGITIVEDINK
>	sp|P61765|STX Syntaxin-binding protein 1 OS=Rattus norvegicus GN=Stxbp1 PE=1 SV=1	YRGEYKDNALLAQLIQDKLDAYK
>	sp|P61765|STX Syntaxin-binding protein 1 OS=Rattus norvegicus GN=Stxbp1 PE=1 SV=1	HIAEVSQEVTR
>	sp|P61765|STX Syntaxin-binding protein 1 OS=Rattus norvegicus GN=Stxbp1 PE=1 SV=1	IMHDVIK
>	sp|P61765|STX Syntaxin-binding protein 1 OS=Rattus norvegicus GN=Stxbp1 PE=1 SV=1	REPLPSLEAVYLITPSEK
>	sp|P61765|STX Syntaxin-binding protein 1 OS=Rattus norvegicus GN=Stxbp1 PE=1 SV=1	WTPIIKDIMEDTIEDKLDTK
>	sp|P61765|STX Syntaxin-binding protein 1 OS=Rattus norvegicus GN=Stxbp1 PE=1 SV=1	VKEVLLDEDDDLWIALR
>	sp|P61765|STX Syntaxin-binding protein 1 OS=Rattus norvegicus GN=Stxbp1 PE=1 SV=1	WTPIIKDIMEDTIEDKLDTK
>	sp|P61765|STX Syntaxin-binding protein 1 OS=Rattus norvegicus GN=Stxbp1 PE=1 SV=1	AAHVFFTDSCPDALFNELVK
>	sp|P61765|STX Syntaxin-binding protein 1 OS=Rattus norvegicus GN=Stxbp1 PE=1 SV=1	TLTEINIAFLPYESQVYSLDSADSFQSFYSPHK
>	sp|P61765|STX Syntaxin-binding protein 1 OS=Rattus norvegicus GN=Stxbp1 PE=1 SV=1	AAHVFFTDSCPDALFNELVK
>	sp|P61765|STX Syntaxin-binding protein 1 OS=Rattus norvegicus GN=Stxbp1 PE=1 SV=1	REPLPSLEAVYLITPSEK
>	sp|P61765|STX Syntaxin-binding protein 1 OS=Rattus norvegicus GN=Stxbp1 PE=1 SV=1	YRGEYKDNALLAQLIQDKLDAYK
>	sp|P61765|STX Syntaxin-binding protein 1 OS=Rattus norvegicus GN=Stxbp1 PE=1 SV=1	TLTEINIAFLPYESQVYSLDSADSFQSFYSPHK
>	sp|P61765|STX Syntaxin-binding protein 1 OS=Rattus norvegicus GN=Stxbp1 PE=1 SV=1	TLTEINIAFLPYESQVYSLDSADSFQSFYSPHK
>	sp|P61765|STX Syntaxin-binding protein 1 OS=Rattus norvegicus GN=Stxbp1 PE=1 SV=1	TLTEINIAFLPYESQVYSLDSADSFQSFYSPHK
>	sp|P61765|STX Syntaxin-binding protein 1 OS=Rattus norvegicus GN=Stxbp1 PE=1 SV=1	YRGEYKDNALLAQLIQDKLDAYK
>	sp|P61765|STX Syntaxin-binding protein 1 OS=Rattus norvegicus GN=Stxbp1 PE=1 SV=1	WTPIIKDIMEDTIEDKLDTK
>	sp|P61765|STX Syntaxin-binding protein 1 OS=Rattus norvegicus GN=Stxbp1 PE=1 SV=1	LIQHAQIPPEDSEIITNMAHLGVPIVTDSTLR
>	sp|P61765|STX Syntaxin-binding protein 1 OS=Rattus norvegicus GN=Stxbp1 PE=1 SV=1	DLSQMLK
>	sp|P61765|STX Syntaxin-binding protein 1 OS=Rattus norvegicus GN=Stxbp1 PE=1 SV=1	YRGEYKDNALLAQLIQDKLDAYK
>	sp|P61765|STX Syntaxin-binding protein 1 OS=Rattus norvegicus GN=Stxbp1 PE=1 SV=1	YSTHLHLAEDCMK
>	sp|P61765|STX Syntaxin-binding protein 1 OS=Rattus norvegicus GN=Stxbp1 PE=1 SV=1	LAEQIATLCATLK
>	sp|P61765|STX Syntaxin-binding protein 1 OS=Rattus norvegicus GN=Stxbp1 PE=1 SV=1	RREPLPSLEAVYLITPSEK
>	sp|P61765|STX Syntaxin-binding protein 1 OS=Rattus norvegicus GN=Stxbp1 PE=1 SV=1	LIQHAQIPPEDSEIITNMAHLGVPIVTDSTLR
>	sp|P61765|STX Syntaxin-binding protein 1 OS=Rattus norvegicus GN=Stxbp1 PE=1 SV=1	NKAPGEYR
>	sp|P61765|STX Syntaxin-binding protein 1 OS=Rattus norvegicus GN=Stxbp1 PE=1 SV=1	SQLLILDR
>	sp|P61765|STX Syntaxin-binding protein 1 OS=Rattus norvegicus GN=Stxbp1 PE=1 SV=1	WTPIIKDIMEDTIEDKLDTKHYPYISTR
>	sp|P61765|STX Syntaxin-binding protein 1 OS=Rattus norvegicus GN=Stxbp1 PE=1 SV=1	VKEVLLDEDDDLWIALR
>	sp|P61765|STX Syntaxin-binding protein 1 OS=Rattus norvegicus GN=Stxbp1 PE=1 SV=1	AAHVFFTDSCPDALFNELVK
>	sp|P61765|STX Syntaxin-binding protein 1 OS=Rattus norvegicus GN=Stxbp1 PE=1 SV=1	HIAEVSQEVTR
>	sp|P61765|STX Syntaxin-binding protein 1 OS=Rattus norvegicus GN=Stxbp1 PE=1 SV=1	GFDPSSPVLHELTFQAMSYDLLPIENDVYKYETSGIGEAR
>	sp|P61765|STX Syntaxin-binding protein 1 OS=Rattus norvegicus GN=Stxbp1 PE=1 SV=1	DLSQMLK
>	sp|P05065|ALD Fructose-bisphosphate aldolase A OS=Rattus norvegicus GN=Aldoa PE=1 SV=2	AAQEEYIKR
>	sp|P05065|ALD Fructose-bisphosphate aldolase A OS=Rattus norvegicus GN=Aldoa PE=1 SV=2	ADDGRPFPQVIK
>	sp|P05065|ALD Fructose-bisphosphate aldolase A OS=Rattus norvegicus GN=Aldoa PE=1 SV=2	ALANSLACQGK
>	sp|P05065|ALD Fructose-bisphosphate aldolase A OS=Rattus norvegicus GN=Aldoa PE=1 SV=2	ALQASALK
>	sp|P05065|ALD Fructose-bisphosphate aldolase A OS=Rattus norvegicus GN=Aldoa PE=1 SV=2	ELADIAHR
>	sp|P05065|ALD Fructose-bisphosphate aldolase A OS=Rattus norvegicus GN=Aldoa PE=1 SV=2	FSNEEIAMATVTALR
>	sp|P05065|ALD Fructose-bisphosphate aldolase A OS=Rattus norvegicus GN=Aldoa PE=1 SV=2	FSNEEIAMATVTALRR
>	sp|P05065|ALD Fructose-bisphosphate aldolase A OS=Rattus norvegicus GN=Aldoa PE=1 SV=2	GILAADESTGSIAK
>	sp|P05065|ALD Fructose-bisphosphate aldolase A OS=Rattus norvegicus GN=Aldoa PE=1 SV=2	GVVPLAGTNGETTTQGLDGLSER
>	sp|P05065|ALD Fructose-bisphosphate aldolase A OS=Rattus norvegicus GN=Aldoa PE=1 SV=2	IGEHTPSSLAIMENANVLAR
>	sp|P05065|ALD Fructose-bisphosphate aldolase A OS=Rattus norvegicus GN=Aldoa PE=1 SV=2	LQSIGTENTEENRR
>	sp|P05065|ALD Fructose-bisphosphate aldolase A OS=Rattus norvegicus GN=Aldoa PE=1 SV=2	QLLLTADDR
>	sp|P05065|ALD Fructose-bisphosphate aldolase A OS=Rattus norvegicus GN=Aldoa PE=1 SV=2	RLQSIGTENTEENR
>	sp|P05065|ALD Fructose-bisphosphate aldolase A OS=Rattus norvegicus GN=Aldoa PE=1 SV=2	SKGGVVGIK
>	sp|P05065|ALD Fructose-bisphosphate aldolase A OS=Rattus norvegicus GN=Aldoa PE=1 SV=2	TVPPAVPGVTFLSGGQSEEEASINLNAINKCPLLKPWALTFSYGR
>	sp|P05065|ALD Fructose-bisphosphate aldolase A OS=Rattus norvegicus GN=Aldoa PE=1 SV=2	VDKGVVPLAGTNGETTTQGLDGLSER
>	sp|P05065|ALD Fructose-bisphosphate aldolase A OS=Rattus norvegicus GN=Aldoa PE=1 SV=2	YASICQQNGIVPIVEPEILPDGDHDLK
>	sp|P05065|ALD Fructose-bisphosphate aldolase A OS=Rattus norvegicus GN=Aldoa PE=1 SV=2	YASICQQNGIVPIVEPEILPDGDHDLKR
>	sp|P05065|ALD Fructose-bisphosphate aldolase A OS=Rattus norvegicus GN=Aldoa PE=1 SV=2	ALSDHHVYLEGTLLKPNMVTPGHACTQK
>	sp|P05065|ALD Fructose-bisphosphate aldolase A OS=Rattus norvegicus GN=Aldoa PE=1 SV=2	AAQEEYIKR
>	sp|P05065|ALD Fructose-bisphosphate aldolase A OS=Rattus norvegicus GN=Aldoa PE=1 SV=2	ADDGRPFPQVIK
>	sp|P05065|ALD Fructose-bisphosphate aldolase A OS=Rattus norvegicus GN=Aldoa PE=1 SV=2	ADDGRPFPQVIK
>	sp|P05065|ALD Fructose-bisphosphate aldolase A OS=Rattus norvegicus GN=Aldoa PE=1 SV=2	ADDGRPFPQVIK
>	sp|P05065|ALD Fructose-bisphosphate aldolase A OS=Rattus norvegicus GN=Aldoa PE=1 SV=2	ALQASALK

>	sp|P05065|ALD	Fructose-bisphosphate aldolase A OS=Rattus norvegicus GN=Aldoa PE=1 SV=2	ALSDHHVYLEGTLLKPNMVTPGHACTQK
>	sp|P05065|ALD	Fructose-bisphosphate aldolase A OS=Rattus norvegicus GN=Aldoa PE=1 SV=2	ELADIAHR
>	sp|P05065|ALD	Fructose-bisphosphate aldolase A OS=Rattus norvegicus GN=Aldoa PE=1 SV=2	ELADIAHR
>	sp|P05065|ALD	Fructose-bisphosphate aldolase A OS=Rattus norvegicus GN=Aldoa PE=1 SV=2	ELADIAHR
>	sp|P05065|ALD	Fructose-bisphosphate aldolase A OS=Rattus norvegicus GN=Aldoa PE=1 SV=2	FSNEEIAMATVTALR
>	sp|P05065|ALD	Fructose-bisphosphate aldolase A OS=Rattus norvegicus GN=Aldoa PE=1 SV=2	GILAADESTGSIAK
>	sp|P05065|ALD	Fructose-bisphosphate aldolase A OS=Rattus norvegicus GN=Aldoa PE=1 SV=2	GILAADESTGSIAK
>	sp|P05065|ALD	Fructose-bisphosphate aldolase A OS=Rattus norvegicus GN=Aldoa PE=1 SV=2	GILAADESTGSIAK
>	sp|P05065|ALD	Fructose-bisphosphate aldolase A OS=Rattus norvegicus GN=Aldoa PE=1 SV=2	IGEHTPSSLAIMENANVLAR
>	sp|P05065|ALD	Fructose-bisphosphate aldolase A OS=Rattus norvegicus GN=Aldoa PE=1 SV=2	IGEHTPSSLAIMENANVLAR
>	sp|P05065|ALD	Fructose-bisphosphate aldolase A OS=Rattus norvegicus GN=Aldoa PE=1 SV=2	IGEHTPSSLAIMENANVLAR
>	sp|P05065|ALD	Fructose-bisphosphate aldolase A OS=Rattus norvegicus GN=Aldoa PE=1 SV=2	IGEHTPSSLAIMENANVLAR
>	sp|P05065|ALD	Fructose-bisphosphate aldolase A OS=Rattus norvegicus GN=Aldoa PE=1 SV=2	TVPPAVPGVTFLSGGQSEEEASINLNAINKCPLLKPWALTFSYGR
>	sp|P05065|ALD	Fructose-bisphosphate aldolase A OS=Rattus norvegicus GN=Aldoa PE=1 SV=2	TVPPAVPGVTFLSGGQSEEEASINLNAINKCPLLKPWALTFSYGR
>	sp|P05065|ALD	Fructose-bisphosphate aldolase A OS=Rattus norvegicus GN=Aldoa PE=1 SV=2	TVPPAVPGVTFLSGGQSEEEASINLNAINKCPLLKPWALTFSYGR
>	sp|P05065|ALD	Fructose-bisphosphate aldolase A OS=Rattus norvegicus GN=Aldoa PE=1 SV=2	TVPPAVPGVTFLSGGQSEEEASINLNAINKCPLLKPWALTFSYGR
>	sp|P05065|ALD	Fructose-bisphosphate aldolase A OS=Rattus norvegicus GN=Aldoa PE=1 SV=2	TVPPAVPGVTFLSGGQSEEEASINLNAINKCPLLKPWALTFSYGR
>	sp|P05065|ALD	Fructose-bisphosphate aldolase A OS=Rattus norvegicus GN=Aldoa PE=1 SV=2	TVPPAVPGVTFLSGGQSEEEASINLNAINKCPLLKPWALTFSYGR
>	sp|P05065|ALD	Fructose-bisphosphate aldolase A OS=Rattus norvegicus GN=Aldoa PE=1 SV=2	VDKGVVPLAGTNGETTTQGLDGLSER
>	sp|P05065|ALD	Fructose-bisphosphate aldolase A OS=Rattus norvegicus GN=Aldoa PE=1 SV=2	VDKGVVPLAGTNGETTTQGLDGLSER
>	sp|P05065|ALD	Fructose-bisphosphate aldolase A OS=Rattus norvegicus GN=Aldoa PE=1 SV=2	VDKGVVPLAGTNGETTTQGLDGLSER
>	sp|P05065|ALD	Fructose-bisphosphate aldolase A OS=Rattus norvegicus GN=Aldoa PE=1 SV=2	VDKGVVPLAGTNGETTTQGLDGLSER
>	sp|P05065|ALD	Fructose-bisphosphate aldolase A OS=Rattus norvegicus GN=Aldoa PE=1 SV=2	YASICQQNGIVPIVEPEILPDGDHDLK
>	sp|P05065|ALD	Fructose-bisphosphate aldolase A OS=Rattus norvegicus GN=Aldoa PE=1 SV=2	YASICQQNGIVPIVEPEILPDGDHDLK
>	sp|P05065|ALD	Fructose-bisphosphate aldolase A OS=Rattus norvegicus GN=Aldoa PE=1 SV=2	YASICQQNGIVPIVEPEILPDGDHDLK
>	sp|P05065|ALD	Fructose-bisphosphate aldolase A OS=Rattus norvegicus GN=Aldoa PE=1 SV=2	YASICQQNGIVPIVEPEILPDGDHDLK
>	sp|P05065|ALD	Fructose-bisphosphate aldolase A OS=Rattus norvegicus GN=Aldoa PE=1 SV=2	YASICQQNGIVPIVEPEILPDGDHDLKR
>	sp|P05065|ALD	Fructose-bisphosphate aldolase A OS=Rattus norvegicus GN=Aldoa PE=1 SV=2	YASICQQNGIVPIVEPEILPDGDHDLKR
>	sp|P05065|ALD	Fructose-bisphosphate aldolase A OS=Rattus norvegicus GN=Aldoa PE=1 SV=2	YASICQQNGIVPIVEPEILPDGDHDLKR
>	sp|P05065|ALD	Fructose-bisphosphate aldolase A OS=Rattus norvegicus GN=Aldoa PE=1 SV=2	YASICQQNGIVPIVEPEILPDGDHDLKR
>	sp|P05065|ALD	Fructose-bisphosphate aldolase A OS=Rattus norvegicus GN=Aldoa PE=1 SV=2	YASICQQNGIVPIVEPEILPDGDHDLKR
>	sp|P05065|ALD	Fructose-bisphosphate aldolase A OS=Rattus norvegicus GN=Aldoa PE=1 SV=2	YASICQQNGIVPIVEPEILPDGDHDLKR
>	sp|P05065|ALD	Fructose-bisphosphate aldolase A OS=Rattus norvegicus GN=Aldoa PE=1 SV=2	YASICQQNGIVPIVEPEILPDGDHDLKR
>	sp|P05065|ALD	Fructose-bisphosphate aldolase A OS=Rattus norvegicus GN=Aldoa PE=1 SV=2	YASICQQNGIVPIVEPEILPDGDHDLKR
>	sp|P05065|ALD	Fructose-bisphosphate aldolase A OS=Rattus norvegicus GN=Aldoa PE=1 SV=2	YASICQQNGIVPIVEPEILPDGDHDLKR
>	sp|P05065|ALD	Fructose-bisphosphate aldolase A OS=Rattus norvegicus GN=Aldoa PE=1 SV=2	YASICQQNGIVPIVEPEILPDGDHDLKR
>	sp|P05065|ALD	Fructose-bisphosphate aldolase A OS=Rattus norvegicus GN=Aldoa PE=1 SV=2	YASICQQNGIVPIVEPEILPDGDHDLKR
>	sp|P05065|ALD	Fructose-bisphosphate aldolase A OS=Rattus norvegicus GN=Aldoa PE=1 SV=2	YASICQQNGIVPIVEPEILPDGDHDLKR
>	sp|P05065|ALD	Fructose-bisphosphate aldolase A OS=Rattus norvegicus GN=Aldoa PE=1 SV=2	YASICQQNGIVPIVEPEILPDGDHDLKR
>	sp|P05065|ALD	Fructose-bisphosphate aldolase A OS=Rattus norvegicus GN=Aldoa PE=1 SV=2	YASICQQNGIVPIVEPEILPDGDHDLKR
>	sp|P05065|ALD	Fructose-bisphosphate aldolase A OS=Rattus norvegicus GN=Aldoa PE=1 SV=2	YASICQQNGIVPIVEPEILPDGDHDLKR
>	sp|P05065|ALD	Fructose-bisphosphate aldolase A OS=Rattus norvegicus GN=Aldoa PE=1 SV=2	YASICQQNGIVPIVEPEILPDGDHDLKR
>	sp|P05065|ALD	Fructose-bisphosphate aldolase A OS=Rattus norvegicus GN=Aldoa PE=1 SV=2	YASICQQNGIVPIVEPEILPDGDHDLKR
>	sp|P05065|ALD	Fructose-bisphosphate aldolase A OS=Rattus norvegicus GN=Aldoa PE=1 SV=2	YASICQQNGIVPIVEPEILPDGDHDLKR
>	sp|P05065|ALD	Fructose-bisphosphate aldolase A OS=Rattus norvegicus GN=Aldoa PE=1 SV=2	YASICQQNGIVPIVEPEILPDGDHDLKR
>	sp|P05065|ALD	Fructose-bisphosphate aldolase A OS=Rattus norvegicus GN=Aldoa PE=1 SV=2	YASICQQNGIVPIVEPEILPDGDHDLKR
>	sp|P05065|ALD	Fructose-bisphosphate aldolase A OS=Rattus norvegicus GN=Aldoa PE=1 SV=2	YASICQQNGIVPIVEPEILPDGDHDLKR
>	sp|P05065|ALD	Fructose-bisphosphate aldolase A OS=Rattus norvegicus GN=Aldoa PE=1 SV=2	YASICQQNGIVPIVEPEILPDGDHDLKR
>	sp|P05065|ALD	Fructose-bisphosphate aldolase A OS=Rattus norvegicus GN=Aldoa PE=1 SV=2	YASICQQNGIVPIVEPEILPDGDHDLKR
>	sp|P05065|ALD	Fructose-bisphosphate aldolase A OS=Rattus norvegicus GN=Aldoa PE=1 SV=2	YASICQQNGIVPIVEPEILPDGDHDLKR
>	sp|P05065|ALD	Fructose-bisphosphate aldolase A OS=Rattus norvegicus GN=Aldoa PE=1 SV=2	YASICQQNGIVPIVEPEILPDGDHDLKR
>	sp|P05065|ALD	Fructose-bisphosphate aldolase A OS=Rattus norvegicus GN=Aldoa PE=1 SV=2	YASICQQNGIVPIVEPEILPDGDHDLKR
>	sp|P05065|ALD	Fructose-bisphosphate aldolase A OS=Rattus norvegicus GN=Aldoa PE=1 SV=2	YASICQQNGIVPIVEPEILPDGDHDLKR
>	sp|P05065|ALD	Fructose-bisphosphate aldolase A OS=Rattus norvegicus GN=Aldoa PE=1 SV=2	YASICQQNGIVPIVEPEILPDGDHDLKR
>	sp|P05065|ALD	Fructose-bisphosphate aldolase A OS=Rattus norvegicus GN=Aldoa PE=1 SV=2	YASICQQNGIVPIVEPEILPDGDHDLKR
>	sp|P05065|ALD	Fructose-bisphosphate aldolase A OS=Rattus norvegicus GN=Aldoa PE=1 SV=2	FSNEEIAMATVTALR
>	sp|P05065|ALD	Fructose-bisphosphate aldolase A OS=Rattus norvegicus GN=Aldoa PE=1 SV=2	FSNEEIAMATVTALR
>	sp|P05065|ALD	Fructose-bisphosphate aldolase A OS=Rattus norvegicus GN=Aldoa PE=1 SV=2	TVPPAVPGVTFLSGGQSEEEASINLNAINKCPLLKPWALTFSYGR
>	sp|P05065|ALD	Fructose-bisphosphate aldolase A OS=Rattus norvegicus GN=Aldoa PE=1 SV=2	ALQASALK
>	sp|P05065|ALD	Fructose-bisphosphate aldolase A OS=Rattus norvegicus GN=Aldoa PE=1 SV=2	IVAPGK
>	sp|P05065|ALD	Fructose-bisphosphate aldolase A OS=Rattus norvegicus GN=Aldoa PE=1 SV=2	GVVPLAGTNGETTTQGLDGLSER
>	sp|P05065|ALD	Fructose-bisphosphate aldolase A OS=Rattus norvegicus GN=Aldoa PE=1 SV=2	IGEHTPSSLAIMENANVLAR
>	sp|P05065|ALD	Fructose-bisphosphate aldolase A OS=Rattus norvegicus GN=Aldoa PE=1 SV=2	ADDGRPFPQVIK
>	sp|P05065|ALD	Fructose-bisphosphate aldolase A OS=Rattus norvegicus GN=Aldoa PE=1 SV=2	ALQASALK
>	sp|P05065|ALD	Fructose-bisphosphate aldolase A OS=Rattus norvegicus GN=Aldoa PE=1 SV=2	YASICQQNGIVPIVEPEILPDGDHDLKR
>	sp|P05065|ALD	Fructose-bisphosphate aldolase A OS=Rattus norvegicus GN=Aldoa PE=1 SV=2	IGEHTPSSLAIMENANVLAR
>	sp|P05065|ALD	Fructose-bisphosphate aldolase A OS=Rattus norvegicus GN=Aldoa PE=1 SV=2	ALQASALK
>	sp|P05065|ALD	Fructose-bisphosphate aldolase A OS=Rattus norvegicus GN=Aldoa PE=1 SV=2	YASICQQNGIVPIVEPEILPDGDHDLKR
>	sp|P05065|ALD	Fructose-bisphosphate aldolase A OS=Rattus norvegicus GN=Aldoa PE=1 SV=2	DGADFAK
>	sp|P05065|ALD	Fructose-bisphosphate aldolase A OS=Rattus norvegicus GN=Aldoa PE=1 SV=2	ALSDHHVYLEGTLLKPNMVTPGHACTQK
>	sp|P05065|ALD	Fructose-bisphosphate aldolase A OS=Rattus norvegicus GN=Aldoa PE=1 SV=2	VDKGVVPLAGTNGETTTQGLDGLSER
>	sp|P05065|ALD	Fructose-bisphosphate aldolase A OS=Rattus norvegicus GN=Aldoa PE=1 SV=2	ADDGRPFPQVIK
>	sp|P05065|ALD	Fructose-bisphosphate aldolase A OS=Rattus norvegicus GN=Aldoa PE=1 SV=2	ADDGRPFPQVIK
>	sp|P05065|ALD	Fructose-bisphosphate aldolase A OS=Rattus norvegicus GN=Aldoa PE=1 SV=2	IVAPGK

>	sp|P05065|ALD Fructose-bisphosphate aldolase A OS=Rattus norvegicus GN=Aldoa PE=1 SV=2	TVPPAVPGVTFLSGGQSEEEASINLNAINKCPLLKPWALTFSYGR
>	sp|P05065|ALD Fructose-bisphosphate aldolase A OS=Rattus norvegicus GN=Aldoa PE=1 SV=2	GGVVGIK
>	sp|P05065|ALD Fructose-bisphosphate aldolase A OS=Rattus norvegicus GN=Aldoa PE=1 SV=2	ALSDHHVYLEGTLLKPNMVTPGHACTQK
>	sp|P05065|ALD Fructose-bisphosphate aldolase A OS=Rattus norvegicus GN=Aldoa PE=1 SV=2	ALQASALK
>	sp|P05065|ALD Fructose-bisphosphate aldolase A OS=Rattus norvegicus GN=Aldoa PE=1 SV=2	LQSIGTENTEENRR
>	sp|P05065|ALD Fructose-bisphosphate aldolase A OS=Rattus norvegicus GN=Aldoa PE=1 SV=2	IVAPGK
>	sp|P05065|ALD Fructose-bisphosphate aldolase A OS=Rattus norvegicus GN=Aldoa PE=1 SV=2	AAQEEYIK
>	sp|P05065|ALD Fructose-bisphosphate aldolase A OS=Rattus norvegicus GN=Aldoa PE=1 SV=2	YASICQQNGIVPIVEPEILPDGDHDLKR
>	sp|P05065|ALD Fructose-bisphosphate aldolase A OS=Rattus norvegicus GN=Aldoa PE=1 SV=2	GILAADESTGSIAK
>	sp|P05065|ALD Fructose-bisphosphate aldolase A OS=Rattus norvegicus GN=Aldoa PE=1 SV=2	IVAPGK
>	sp|P05065|ALD Fructose-bisphosphate aldolase A OS=Rattus norvegicus GN=Aldoa PE=1 SV=2	TVPPAVPGVTFLSGGQSEEEASINLNAINKCPLLKPWALTFSYGR
>	sp|P05065|ALD Fructose-bisphosphate aldolase A OS=Rattus norvegicus GN=Aldoa PE=1 SV=2	IGEHTPSSLAIMENANVLAR
>	sp|P05065|ALD Fructose-bisphosphate aldolase A OS=Rattus norvegicus GN=Aldoa PE=1 SV=2	ELADIAHR
>	sp|P05065|ALD Fructose-bisphosphate aldolase A OS=Rattus norvegicus GN=Aldoa PE=1 SV=2	RLQSIGTENTEENR
>	sp|P05065|ALD Fructose-bisphosphate aldolase A OS=Rattus norvegicus GN=Aldoa PE=1 SV=2	LQSIGTENTEENR
>	sp|P05065|ALD Fructose-bisphosphate aldolase A OS=Rattus norvegicus GN=Aldoa PE=1 SV=2	RLQSIGTENTEENR
>	sp|P05065|ALD Fructose-bisphosphate aldolase A OS=Rattus norvegicus GN=Aldoa PE=1 SV=2	VLAAVYK
>	sp|P05065|ALD Fructose-bisphosphate aldolase A OS=Rattus norvegicus GN=Aldoa PE=1 SV=2	IVAPGK
>	sp|P05065|ALD Fructose-bisphosphate aldolase A OS=Rattus norvegicus GN=Aldoa PE=1 SV=2	YASICQQNGIVPIVEPEILPDGDHDLK
>	sp|P05065|ALD Fructose-bisphosphate aldolase A OS=Rattus norvegicus GN=Aldoa PE=1 SV=2	LQSIGTENTEENRR
>	sp|P05065|ALD Fructose-bisphosphate aldolase A OS=Rattus norvegicus GN=Aldoa PE=1 SV=2	AAQEEYIK
>	sp|P05065|ALD Fructose-bisphosphate aldolase A OS=Rattus norvegicus GN=Aldoa PE=1 SV=2	TVPPAVPGVTFLSGGQSEEEASINLNAINKCPLLKPWALTFSYGR
>	sp|P05065|ALD Fructose-bisphosphate aldolase A OS=Rattus norvegicus GN=Aldoa PE=1 SV=2	ADDGRPFPQVIK
>	sp|P05065|ALD Fructose-bisphosphate aldolase A OS=Rattus norvegicus GN=Aldoa PE=1 SV=2	CPLLKPWALTFSYGR
>	sp|P05065|ALD Fructose-bisphosphate aldolase A OS=Rattus norvegicus GN=Aldoa PE=1 SV=2	YASICQQNGIVPIVEPEILPDGDHDLKR
>	sp|P05065|ALD Fructose-bisphosphate aldolase A OS=Rattus norvegicus GN=Aldoa PE=1 SV=2	YASICQQNGIVPIVEPEILPDGDHDLKR
>	sp|P05065|ALD Fructose-bisphosphate aldolase A OS=Rattus norvegicus GN=Aldoa PE=1 SV=2	IVAPGK
>	sp|P05065|ALD Fructose-bisphosphate aldolase A OS=Rattus norvegicus GN=Aldoa PE=1 SV=2	FSNEEIAMATVTALR
>	sp|P05065|ALD Fructose-bisphosphate aldolase A OS=Rattus norvegicus GN=Aldoa PE=1 SV=2	YASICQQNGIVPIVEPEILPDGDHDLKR
>	sp|P05065|ALD Fructose-bisphosphate aldolase A OS=Rattus norvegicus GN=Aldoa PE=1 SV=2	VLAAVYK
>	sp|P05065|ALD Fructose-bisphosphate aldolase A OS=Rattus norvegicus GN=Aldoa PE=1 SV=2	YASICQQNGIVPIVEPEILPDGDHDLKR
>	sp|P05065|ALD Fructose-bisphosphate aldolase A OS=Rattus norvegicus GN=Aldoa PE=1 SV=2	YTPSGQSGAAASESLFISNHAY
>	sp|P05065|ALD Fructose-bisphosphate aldolase A OS=Rattus norvegicus GN=Aldoa PE=1 SV=2	TVPPAVPGVTFLSGGQSEEEASINLNAINKCPLLKPWALTFSYGR
>	sp|P63018|HSF Heat shock cognate 71 kDa protein OS=Rattus norvegicus GN=Hspa8 PE=1 SV=1	ARFEELNADLFR
>	sp|P63018|HSF Heat shock cognate 71 kDa protein OS=Rattus norvegicus GN=Hspa8 PE=1 SV=1	DAGTIAGLNVLR
>	sp|P63018|HSF Heat shock cognate 71 kDa protein OS=Rattus norvegicus GN=Hspa8 PE=1 SV=1	FEELNADLFR
>	sp|P63018|HSF Heat shock cognate 71 kDa protein OS=Rattus norvegicus GN=Hspa8 PE=1 SV=1	HWPFMVVNDAGRPK
>	sp|P63018|HSF Heat shock cognate 71 kDa protein OS=Rattus norvegicus GN=Hspa8 PE=1 SV=1	IINEPTAAAIAYGLDK
>	sp|P63018|HSF Heat shock cognate 71 kDa protein OS=Rattus norvegicus GN=Hspa8 PE=1 SV=1	IINEPTAAAIAYGLDKK
>	sp|P63018|HSF Heat shock cognate 71 kDa protein OS=Rattus norvegicus GN=Hspa8 PE=1 SV=1	IPKIQKLLQDFFNGKELNK
>	sp|P63018|HSF Heat shock cognate 71 kDa protein OS=Rattus norvegicus GN=Hspa8 PE=1 SV=1	LDKSQIHDIVLVGGSTR
>	sp|P63018|HSF Heat shock cognate 71 kDa protein OS=Rattus norvegicus GN=Hspa8 PE=1 SV=1	LLQDFFNGKELNK
>	sp|P63018|HSF Heat shock cognate 71 kDa protein OS=Rattus norvegicus GN=Hspa8 PE=1 SV=1	MKEIAEAYLGK
>	sp|P63018|HSF Heat shock cognate 71 kDa protein OS=Rattus norvegicus GN=Hspa8 PE=1 SV=1	MVNHFIAEFK
>	sp|P63018|HSF Heat shock cognate 71 kDa protein OS=Rattus norvegicus GN=Hspa8 PE=1 SV=1	NQVAMNPTNTVFDAK
>	sp|P63018|HSF Heat shock cognate 71 kDa protein OS=Rattus norvegicus GN=Hspa8 PE=1 SV=1	STAGDTHLGGEDFDNR
>	sp|P63018|HSF Heat shock cognate 71 kDa protein OS=Rattus norvegicus GN=Hspa8 PE=1 SV=1	TTPSYVAFTDTER
>	sp|P63018|HSF Heat shock cognate 71 kDa protein OS=Rattus norvegicus GN=Hspa8 PE=1 SV=1	VEIIANDQGNR
>	sp|P63018|HSF Heat shock cognate 71 kDa protein OS=Rattus norvegicus GN=Hspa8 PE=1 SV=1	VQVEYKGETK
>	sp|P63018|HSF Heat shock cognate 71 kDa protein OS=Rattus norvegicus GN=Hspa8 PE=1 SV=1	TVTNAVVTVPAYFNDSQR
>	sp|P63018|HSF Heat shock cognate 71 kDa protein OS=Rattus norvegicus GN=Hspa8 PE=1 SV=1	RFDDAVVQSDMK
>	sp|P63018|HSF Heat shock cognate 71 kDa protein OS=Rattus norvegicus GN=Hspa8 PE=1 SV=1	GPAVGIDLGTTYSCVGVFQHGK
>	sp|P63018|HSF Heat shock cognate 71 kDa protein OS=Rattus norvegicus GN=Hspa8 PE=1 SV=1	NQTAEKEEFEHQQK
>	sp|P63018|HSF Heat shock cognate 71 kDa protein OS=Rattus norvegicus GN=Hspa8 PE=1 SV=1	ARFEELNADLFR
>	sp|P63018|HSF Heat shock cognate 71 kDa protein OS=Rattus norvegicus GN=Hspa8 PE=1 SV=1	DAGTIAGLNVLR
>	sp|P63018|HSF Heat shock cognate 71 kDa protein OS=Rattus norvegicus GN=Hspa8 PE=1 SV=1	DAGTIAGLNVLR
>	sp|P63018|HSF Heat shock cognate 71 kDa protein OS=Rattus norvegicus GN=Hspa8 PE=1 SV=1	FEELNADLFR
>	sp|P63018|HSF Heat shock cognate 71 kDa protein OS=Rattus norvegicus GN=Hspa8 PE=1 SV=1	GPAVGIDLGTTYSCVGVFQHGK
>	sp|P63018|HSF Heat shock cognate 71 kDa protein OS=Rattus norvegicus GN=Hspa8 PE=1 SV=1	GPAVGIDLGTTYSCVGVFQHGK
>	sp|P63018|HSF Heat shock cognate 71 kDa protein OS=Rattus norvegicus GN=Hspa8 PE=1 SV=1	GPAVGIDLGTTYSCVGVFQHGK
>	sp|P63018|HSF Heat shock cognate 71 kDa protein OS=Rattus norvegicus GN=Hspa8 PE=1 SV=1	HWPFMVVNDAGRPK
>	sp|P63018|HSF Heat shock cognate 71 kDa protein OS=Rattus norvegicus GN=Hspa8 PE=1 SV=1	HWPFMVVNDAGRPK
>	sp|P63018|HSF Heat shock cognate 71 kDa protein OS=Rattus norvegicus GN=Hspa8 PE=1 SV=1	HWPFMVVNDAGRPK
>	sp|P63018|HSF Heat shock cognate 71 kDa protein OS=Rattus norvegicus GN=Hspa8 PE=1 SV=1	HWPFMVVNDAGRPK
>	sp|P63018|HSF Heat shock cognate 71 kDa protein OS=Rattus norvegicus GN=Hspa8 PE=1 SV=1	IINEPTAAAIAYGLDKK
>	sp|P63018|HSF Heat shock cognate 71 kDa protein OS=Rattus norvegicus GN=Hspa8 PE=1 SV=1	IINEPTAAAIAYGLDKK
>	sp|P63018|HSF Heat shock cognate 71 kDa protein OS=Rattus norvegicus GN=Hspa8 PE=1 SV=1	IINEPTAAAIAYGLDKK
>	sp|P63018|HSF Heat shock cognate 71 kDa protein OS=Rattus norvegicus GN=Hspa8 PE=1 SV=1	IINEPTAAAIAYGLDKK
>	sp|P63018|HSF Heat shock cognate 71 kDa protein OS=Rattus norvegicus GN=Hspa8 PE=1 SV=1	IINEPTAAAIAYGLDKK
>	sp|P63018|HSF Heat shock cognate 71 kDa protein OS=Rattus norvegicus GN=Hspa8 PE=1 SV=1	IINEPTAAAIAYGLDKK
>	sp|P63018|HSF Heat shock cognate 71 kDa protein OS=Rattus norvegicus GN=Hspa8 PE=1 SV=1	IINEPTAAAIAYGLDKK
>	sp|P63018|HSF Heat shock cognate 71 kDa protein OS=Rattus norvegicus GN=Hspa8 PE=1 SV=1	IINEPTAAAIAYGLDKK
>	sp|P63018|HSF Heat shock cognate 71 kDa protein OS=Rattus norvegicus GN=Hspa8 PE=1 SV=1	IINEPTAAAIAYGLDKK
>	sp|P63018|HSF Heat shock cognate 71 kDa protein OS=Rattus norvegicus GN=Hspa8 PE=1 SV=1	IINEPTAAAIAYGLDKK

>	sp|P63018|HSF Heat shock cognate 71 kDa protein OS=Rattus norvegicus GN=Hspa8 PE=1 SV=1	IINEPTAAAIAYGLDKK
>	sp|P63018|HSF Heat shock cognate 71 kDa protein OS=Rattus norvegicus GN=Hspa8 PE=1 SV=1	LDKSQIHDIVLVGGSTR
>	sp|P63018|HSF Heat shock cognate 71 kDa protein OS=Rattus norvegicus GN=Hspa8 PE=1 SV=1	LDKSQIHDIVLVGGSTR
>	sp|P63018|HSF Heat shock cognate 71 kDa protein OS=Rattus norvegicus GN=Hspa8 PE=1 SV=1	LDKSQIHDIVLVGGSTR
>	sp|P63018|HSF Heat shock cognate 71 kDa protein OS=Rattus norvegicus GN=Hspa8 PE=1 SV=1	LDKSQIHDIVLVGGSTR
>	sp|P63018|HSF Heat shock cognate 71 kDa protein OS=Rattus norvegicus GN=Hspa8 PE=1 SV=1	LDKSQIHDIVLVGGSTR
>	sp|P63018|HSF Heat shock cognate 71 kDa protein OS=Rattus norvegicus GN=Hspa8 PE=1 SV=1	LLQDFFNGKELNK
>	sp|P63018|HSF Heat shock cognate 71 kDa protein OS=Rattus norvegicus GN=Hspa8 PE=1 SV=1	LLQDFFNGKELNK
>	sp|P63018|HSF Heat shock cognate 71 kDa protein OS=Rattus norvegicus GN=Hspa8 PE=1 SV=1	MKEIAEAYLGK
>	sp|P63018|HSF Heat shock cognate 71 kDa protein OS=Rattus norvegicus GN=Hspa8 PE=1 SV=1	MKEIAEAYLGK
>	sp|P63018|HSF Heat shock cognate 71 kDa protein OS=Rattus norvegicus GN=Hspa8 PE=1 SV=1	MVNHFIAEFK
>	sp|P63018|HSF Heat shock cognate 71 kDa protein OS=Rattus norvegicus GN=Hspa8 PE=1 SV=1	MVNHFIAEFK
>	sp|P63018|HSF Heat shock cognate 71 kDa protein OS=Rattus norvegicus GN=Hspa8 PE=1 SV=1	MVNHFIAEFK
>	sp|P63018|HSF Heat shock cognate 71 kDa protein OS=Rattus norvegicus GN=Hspa8 PE=1 SV=1	MVNHFIAEFK
>	sp|P63018|HSF Heat shock cognate 71 kDa protein OS=Rattus norvegicus GN=Hspa8 PE=1 SV=1	MVNHFIAEFK
>	sp|P63018|HSF Heat shock cognate 71 kDa protein OS=Rattus norvegicus GN=Hspa8 PE=1 SV=1	MVNHFIAEFK
>	sp|P63018|HSF Heat shock cognate 71 kDa protein OS=Rattus norvegicus GN=Hspa8 PE=1 SV=1	MVNHFIAEFK
>	sp|P63018|HSF Heat shock cognate 71 kDa protein OS=Rattus norvegicus GN=Hspa8 PE=1 SV=1	NQVAMNPTNTVFDAK
>	sp|P63018|HSF Heat shock cognate 71 kDa protein OS=Rattus norvegicus GN=Hspa8 PE=1 SV=1	NQVAMNPTNTVFDAK
>	sp|P63018|HSF Heat shock cognate 71 kDa protein OS=Rattus norvegicus GN=Hspa8 PE=1 SV=1	NQVAMNPTNTVFDAK
>	sp|P63018|HSF Heat shock cognate 71 kDa protein OS=Rattus norvegicus GN=Hspa8 PE=1 SV=1	RFDDAVVQSDMK
>	sp|P63018|HSF Heat shock cognate 71 kDa protein OS=Rattus norvegicus GN=Hspa8 PE=1 SV=1	STAGDTHLGGEDFDNR
>	sp|P63018|HSF Heat shock cognate 71 kDa protein OS=Rattus norvegicus GN=Hspa8 PE=1 SV=1	STAGDTHLGGEDFDNR
>	sp|P63018|HSF Heat shock cognate 71 kDa protein OS=Rattus norvegicus GN=Hspa8 PE=1 SV=1	STAGDTHLGGEDFDNR
>	sp|P63018|HSF Heat shock cognate 71 kDa protein OS=Rattus norvegicus GN=Hspa8 PE=1 SV=1	STAGDTHLGGEDFDNR
>	sp|P63018|HSF Heat shock cognate 71 kDa protein OS=Rattus norvegicus GN=Hspa8 PE=1 SV=1	VEIIANDQGNR
>	sp|P63018|HSF Heat shock cognate 71 kDa protein OS=Rattus norvegicus GN=Hspa8 PE=1 SV=1	VEIIANDQGNR
>	sp|P63018|HSF Heat shock cognate 71 kDa protein OS=Rattus norvegicus GN=Hspa8 PE=1 SV=1	VQVEYKGETK
>	sp|P63018|HSF Heat shock cognate 71 kDa protein OS=Rattus norvegicus GN=Hspa8 PE=1 SV=1	VQVEYKGETK
>	sp|P63018|HSF Heat shock cognate 71 kDa protein OS=Rattus norvegicus GN=Hspa8 PE=1 SV=1	VQVEYKGETK
>	sp|P63018|HSF Heat shock cognate 71 kDa protein OS=Rattus norvegicus GN=Hspa8 PE=1 SV=1	LLQDFFNGKELNK
>	sp|P63018|HSF Heat shock cognate 71 kDa protein OS=Rattus norvegicus GN=Hspa8 PE=1 SV=1	GPAVGIDLGTTYSCVGVFQHGK
>	sp|P63018|HSF Heat shock cognate 71 kDa protein OS=Rattus norvegicus GN=Hspa8 PE=1 SV=1	LDKSQIHDIVLVGGSTR
>	sp|P63018|HSF Heat shock cognate 71 kDa protein OS=Rattus norvegicus GN=Hspa8 PE=1 SV=1	FEELNADLFR
>	sp|P63018|HSF Heat shock cognate 71 kDa protein OS=Rattus norvegicus GN=Hspa8 PE=1 SV=1	IINEPTAAAIAYGLDK
>	sp|P63018|HSF Heat shock cognate 71 kDa protein OS=Rattus norvegicus GN=Hspa8 PE=1 SV=1	LLQDFFNGKELNK
>	sp|P63018|HSF Heat shock cognate 71 kDa protein OS=Rattus norvegicus GN=Hspa8 PE=1 SV=1	IINEPTAAAIAYGLDKK
>	sp|P63018|HSF Heat shock cognate 71 kDa protein OS=Rattus norvegicus GN=Hspa8 PE=1 SV=1	HWPFMVVNDAGRPK
>	sp|P63018|HSF Heat shock cognate 71 kDa protein OS=Rattus norvegicus GN=Hspa8 PE=1 SV=1	GTLDPVEK
>	sp|P63018|HSF Heat shock cognate 71 kDa protein OS=Rattus norvegicus GN=Hspa8 PE=1 SV=1	TTPSYVAFTDTER
>	sp|P63018|HSF Heat shock cognate 71 kDa protein OS=Rattus norvegicus GN=Hspa8 PE=1 SV=1	LLQDFFNGKELNK
>	sp|P63018|HSF Heat shock cognate 71 kDa protein OS=Rattus norvegicus GN=Hspa8 PE=1 SV=1	GTLDPVEK
>	sp|P63018|HSF Heat shock cognate 71 kDa protein OS=Rattus norvegicus GN=Hspa8 PE=1 SV=1	IINEPTAAAIAYGLDK
>	sp|P63018|HSF Heat shock cognate 71 kDa protein OS=Rattus norvegicus GN=Hspa8 PE=1 SV=1	GPAVGIDLGTTYSCVGVFQHGK
>	sp|P63018|HSF Heat shock cognate 71 kDa protein OS=Rattus norvegicus GN=Hspa8 PE=1 SV=1	LDKSQIHDIVLVGGSTR
>	sp|P63018|HSF Heat shock cognate 71 kDa protein OS=Rattus norvegicus GN=Hspa8 PE=1 SV=1	QTQTFTTYSDNQPGVLIQVYEGER
>	sp|P63018|HSF Heat shock cognate 71 kDa protein OS=Rattus norvegicus GN=Hspa8 PE=1 SV=1	VEIIANDQGNR
>	sp|P63018|HSF Heat shock cognate 71 kDa protein OS=Rattus norvegicus GN=Hspa8 PE=1 SV=1	TTPSYVAFTDTER
>	sp|P63018|HSF Heat shock cognate 71 kDa protein OS=Rattus norvegicus GN=Hspa8 PE=1 SV=1	LLQDFFNGKELNK
>	sp|P63018|HSF Heat shock cognate 71 kDa protein OS=Rattus norvegicus GN=Hspa8 PE=1 SV=1	NSLESYAFNMK
>	sp|P63018|HSF Heat shock cognate 71 kDa protein OS=Rattus norvegicus GN=Hspa8 PE=1 SV=1	LIGDAAK
>	sp|P63018|HSF Heat shock cognate 71 kDa protein OS=Rattus norvegicus GN=Hspa8 PE=1 SV=1	LLQDFFNGKELNK
>	sp|P63018|HSF Heat shock cognate 71 kDa protein OS=Rattus norvegicus GN=Hspa8 PE=1 SV=1	IINEPTAAAIAYGLDKK
>	sp|P63018|HSF Heat shock cognate 71 kDa protein OS=Rattus norvegicus GN=Hspa8 PE=1 SV=1	IINEPTAAAIAYGLDKK
>	sp|P63018|HSF Heat shock cognate 71 kDa protein OS=Rattus norvegicus GN=Hspa8 PE=1 SV=1	LLQDFFNGKELNK
>	sp|P63018|HSF Heat shock cognate 71 kDa protein OS=Rattus norvegicus GN=Hspa8 PE=1 SV=1	HWPFMVVNDAGRPK
>	sp|P63018|HSF Heat shock cognate 71 kDa protein OS=Rattus norvegicus GN=Hspa8 PE=1 SV=1	HWPFMVVNDAGRPK
>	sp|P63018|HSF Heat shock cognate 71 kDa protein OS=Rattus norvegicus GN=Hspa8 PE=1 SV=1	LLQDFFNGKELNK
>	sp|P63018|HSF Heat shock cognate 71 kDa protein OS=Rattus norvegicus GN=Hspa8 PE=1 SV=1	GPAVGIDLGTTYSCVGVFQHGK
>	sp|P63018|HSF Heat shock cognate 71 kDa protein OS=Rattus norvegicus GN=Hspa8 PE=1 SV=1	MKEIAEAYLGK
>	sp|P63018|HSF Heat shock cognate 71 kDa protein OS=Rattus norvegicus GN=Hspa8 PE=1 SV=1	TTPSYVAFTDTER
>	sp|P63018|HSF Heat shock cognate 71 kDa protein OS=Rattus norvegicus GN=Hspa8 PE=1 SV=1	MKEIAEAYLGK
>	sp|P63018|HSF Heat shock cognate 71 kDa protein OS=Rattus norvegicus GN=Hspa8 PE=1 SV=1	ILDKCNEIISWLDKNQTAEKEEFEHQQK
>	sp|P63018|HSF Heat shock cognate 71 kDa protein OS=Rattus norvegicus GN=Hspa8 PE=1 SV=1	ARFEELNADLFR
>	sp|P63018|HSF Heat shock cognate 71 kDa protein OS=Rattus norvegicus GN=Hspa8 PE=1 SV=1	FEELNADLFR
>	sp|P63018|HSF Heat shock cognate 71 kDa protein OS=Rattus norvegicus GN=Hspa8 PE=1 SV=1	MKEIAEAYLGK
>	sp|P63018|HSF Heat shock cognate 71 kDa protein OS=Rattus norvegicus GN=Hspa8 PE=1 SV=1	LIGDAAK
>	sp|P63018|HSF Heat shock cognate 71 kDa protein OS=Rattus norvegicus GN=Hspa8 PE=1 SV=1	NSLESYAFNMK
>	sp|P63018|HSF Heat shock cognate 71 kDa protein OS=Rattus norvegicus GN=Hspa8 PE=1 SV=1	GTLDPVEK
>	sp|P63018|HSF Heat shock cognate 71 kDa protein OS=Rattus norvegicus GN=Hspa8 PE=1 SV=1	HWPFMVVNDAGRPK
>	sp|P63018|HSF Heat shock cognate 71 kDa protein OS=Rattus norvegicus GN=Hspa8 PE=1 SV=1	MVNHFIAEFKR
>	sp|P63018|HSF Heat shock cognate 71 kDa protein OS=Rattus norvegicus GN=Hspa8 PE=1 SV=1	HWPFMVVNDAGRPK
>	sp|P63018|HSF Heat shock cognate 71 kDa protein OS=Rattus norvegicus GN=Hspa8 PE=1 SV=1	FDDAVVQSDMK
>	sp|P63018|HSF Heat shock cognate 71 kDa protein OS=Rattus norvegicus GN=Hspa8 PE=1 SV=1	HWPFMVVNDAGRPK

<table>
<tr><td>></td><td>sp|P63018|HSF Heat shock cognate 71 kDa protein OS=Rattus norvegicus GN=Hspa8 PE=1 SV=1</td><td>VSAVDK</td></tr>
<tr><td>></td><td>sp|P63018|HSF Heat shock cognate 71 kDa protein OS=Rattus norvegicus GN=Hspa8 PE=1 SV=1</td><td>VCNPIITK</td></tr>
<tr><td>></td><td>sp|P63018|HSF Heat shock cognate 71 kDa protein OS=Rattus norvegicus GN=Hspa8 PE=1 SV=1</td><td>TVTNAVVTVPAYFNDSQR</td></tr>
<tr><td>></td><td>sp|P63018|HSF Heat shock cognate 71 kDa protein OS=Rattus norvegicus GN=Hspa8 PE=1 SV=1</td><td>SFYPEEVSSMVLTK</td></tr>
<tr><td>></td><td>sp|P13233|CN 2',3'-cyclic-nucleotide 3'-phosphodiesterase OS=Rattus norvegicus GN=Cnp PE=1 SV=2</td><td>AGQVFLEELGNHK</td></tr>
<tr><td>></td><td>sp|P13233|CN 2',3'-cyclic-nucleotide 3'-phosphodiesterase OS=Rattus norvegicus GN=Cnp PE=1 SV=2</td><td>AHVTLGCAADVQPVQTGLDLLEILQQVK</td></tr>
<tr><td>></td><td>sp|P13233|CN 2',3'-cyclic-nucleotide 3'-phosphodiesterase OS=Rattus norvegicus GN=Cnp PE=1 SV=2</td><td>ATGAEEYAQQDVVR</td></tr>
<tr><td>></td><td>sp|P13233|CN 2',3'-cyclic-nucleotide 3'-phosphodiesterase OS=Rattus norvegicus GN=Cnp PE=1 SV=2</td><td>DKPELQFPFLQDEDTVATLHECK</td></tr>
<tr><td>></td><td>sp|P13233|CN 2',3'-cyclic-nucleotide 3'-phosphodiesterase OS=Rattus norvegicus GN=Cnp PE=1 SV=2</td><td>ERLDQLFEMADQYQYQVVLVEPK</td></tr>
<tr><td>></td><td>sp|P13233|CN 2',3'-cyclic-nucleotide 3'-phosphodiesterase OS=Rattus norvegicus GN=Cnp PE=1 SV=2</td><td>GGSQGEEVGELPR</td></tr>
<tr><td>></td><td>sp|P13233|CN 2',3'-cyclic-nucleotide 3'-phosphodiesterase OS=Rattus norvegicus GN=Cnp PE=1 SV=2</td><td>KAGQVFLEELGNHK</td></tr>
<tr><td>></td><td>sp|P13233|CN 2',3'-cyclic-nucleotide 3'-phosphodiesterase OS=Rattus norvegicus GN=Cnp PE=1 SV=2</td><td>KLKPGLEKDFLPLYFGWFLTK</td></tr>
<tr><td>></td><td>sp|P13233|CN 2',3'-cyclic-nucleotide 3'-phosphodiesterase OS=Rattus norvegicus GN=Cnp PE=1 SV=2</td><td>KLKPGLEKDFLPLYFGWFLTKK</td></tr>
<tr><td>></td><td>sp|P13233|CN 2',3'-cyclic-nucleotide 3'-phosphodiesterase OS=Rattus norvegicus GN=Cnp PE=1 SV=2</td><td>LDLVSYFGK</td></tr>
<tr><td>></td><td>sp|P13233|CN 2',3'-cyclic-nucleotide 3'-phosphodiesterase OS=Rattus norvegicus GN=Cnp PE=1 SV=2</td><td>LDQLFEMADQYQYQVVLVEPK</td></tr>
<tr><td>></td><td>sp|P13233|CN 2',3'-cyclic-nucleotide 3'-phosphodiesterase OS=Rattus norvegicus GN=Cnp PE=1 SV=2</td><td>LKPGLEKDFLPLYFGWFLTK</td></tr>
<tr><td>></td><td>sp|P13233|CN 2',3'-cyclic-nucleotide 3'-phosphodiesterase OS=Rattus norvegicus GN=Cnp PE=1 SV=2</td><td>LKPGLEKDFLPLYFGWFLTKK</td></tr>
<tr><td>></td><td>sp|P13233|CN 2',3'-cyclic-nucleotide 3'-phosphodiesterase OS=Rattus norvegicus GN=Cnp PE=1 SV=2</td><td>NQWQLSLDDLK</td></tr>
<tr><td>></td><td>sp|P13233|CN 2',3'-cyclic-nucleotide 3'-phosphodiesterase OS=Rattus norvegicus GN=Cnp PE=1 SV=2</td><td>RLDEDLAGYCR</td></tr>
<tr><td>></td><td>sp|P13233|CN 2',3'-cyclic-nucleotide 3'-phosphodiesterase OS=Rattus norvegicus GN=Cnp PE=1 SV=2</td><td>TAGAQVVLNEQELQLWPSDLDKPSSSESLPPGSR</td></tr>
<tr><td>></td><td>sp|P13233|CN 2',3'-cyclic-nucleotide 3'-phosphodiesterase OS=Rattus norvegicus GN=Cnp PE=1 SV=2</td><td>EKLDLVSYFGK</td></tr>
<tr><td>></td><td>sp|P13233|CN 2',3'-cyclic-nucleotide 3'-phosphodiesterase OS=Rattus norvegicus GN=Cnp PE=1 SV=2</td><td>LSISALFVTPK</td></tr>
<tr><td>></td><td>sp|P13233|CN 2',3'-cyclic-nucleotide 3'-phosphodiesterase OS=Rattus norvegicus GN=Cnp PE=1 SV=2</td><td>NQWQLSLDDLKK</td></tr>
<tr><td>></td><td>sp|P13233|CN 2',3'-cyclic-nucleotide 3'-phosphodiesterase OS=Rattus norvegicus GN=Cnp PE=1 SV=2</td><td>EKNQWQLSLDDLKK</td></tr>
<tr><td>></td><td>sp|P13233|CN 2',3'-cyclic-nucleotide 3'-phosphodiesterase OS=Rattus norvegicus GN=Cnp PE=1 SV=2</td><td>AHVTLGCAADVQPVQTGLDLLEILQQVK</td></tr>
<tr><td>></td><td>sp|P13233|CN 2',3'-cyclic-nucleotide 3'-phosphodiesterase OS=Rattus norvegicus GN=Cnp PE=1 SV=2</td><td>AHVTLGCAADVQPVQTGLDLLEILQQVK</td></tr>
<tr><td>></td><td>sp|P13233|CN 2',3'-cyclic-nucleotide 3'-phosphodiesterase OS=Rattus norvegicus GN=Cnp PE=1 SV=2</td><td>AHVTLGCAADVQPVQTGLDLLEILQQVK</td></tr>
<tr><td>></td><td>sp|P13233|CN 2',3'-cyclic-nucleotide 3'-phosphodiesterase OS=Rattus norvegicus GN=Cnp PE=1 SV=2</td><td>AHVTLGCAADVQPVQTGLDLLEILQQVK</td></tr>
<tr><td>></td><td>sp|P13233|CN 2',3'-cyclic-nucleotide 3'-phosphodiesterase OS=Rattus norvegicus GN=Cnp PE=1 SV=2</td><td>AHVTLGCAADVQPVQTGLDLLEILQQVK</td></tr>
<tr><td>></td><td>sp|P13233|CN 2',3'-cyclic-nucleotide 3'-phosphodiesterase OS=Rattus norvegicus GN=Cnp PE=1 SV=2</td><td>AHVTLGCAADVQPVQTGLDLLEILQQVK</td></tr>
<tr><td>></td><td>sp|P13233|CN 2',3'-cyclic-nucleotide 3'-phosphodiesterase OS=Rattus norvegicus GN=Cnp PE=1 SV=2</td><td>AHVTLGCAADVQPVQTGLDLLEILQQVK</td></tr>
<tr><td>></td><td>sp|P13233|CN 2',3'-cyclic-nucleotide 3'-phosphodiesterase OS=Rattus norvegicus GN=Cnp PE=1 SV=2</td><td>AHVTLGCAADVQPVQTGLDLLEILQQVK</td></tr>
<tr><td>></td><td>sp|P13233|CN 2',3'-cyclic-nucleotide 3'-phosphodiesterase OS=Rattus norvegicus GN=Cnp PE=1 SV=2</td><td>AHVTLGCAADVQPVQTGLDLLEILQQVK</td></tr>
<tr><td>></td><td>sp|P13233|CN 2',3'-cyclic-nucleotide 3'-phosphodiesterase OS=Rattus norvegicus GN=Cnp PE=1 SV=2</td><td>AHVTLGCAADVQPVQTGLDLLEILQQVK</td></tr>
<tr><td>></td><td>sp|P13233|CN 2',3'-cyclic-nucleotide 3'-phosphodiesterase OS=Rattus norvegicus GN=Cnp PE=1 SV=2</td><td>AHVTLGCAADVQPVQTGLDLLEILQQVK</td></tr>
<tr><td>></td><td>sp|P13233|CN 2',3'-cyclic-nucleotide 3'-phosphodiesterase OS=Rattus norvegicus GN=Cnp PE=1 SV=2</td><td>AHVTLGCAADVQPVQTGLDLLEILQQVK</td></tr>
<tr><td>></td><td>sp|P13233|CN 2',3'-cyclic-nucleotide 3'-phosphodiesterase OS=Rattus norvegicus GN=Cnp PE=1 SV=2</td><td>AHVTLGCAADVQPVQTGLDLLEILQQVK</td></tr>
<tr><td>></td><td>sp|P13233|CN 2',3'-cyclic-nucleotide 3'-phosphodiesterase OS=Rattus norvegicus GN=Cnp PE=1 SV=2</td><td>AHVTLGCAADVQPVQTGLDLLEILQQVK</td></tr>
<tr><td>></td><td>sp|P13233|CN 2',3'-cyclic-nucleotide 3'-phosphodiesterase OS=Rattus norvegicus GN=Cnp PE=1 SV=2</td><td>AHVTLGCAADVQPVQTGLDLLEILQQVK</td></tr>
<tr><td>></td><td>sp|P13233|CN 2',3'-cyclic-nucleotide 3'-phosphodiesterase OS=Rattus norvegicus GN=Cnp PE=1 SV=2</td><td>AHVTLGCAADVQPVQTGLDLLEILQQVK</td></tr>
<tr><td>></td><td>sp|P13233|CN 2',3'-cyclic-nucleotide 3'-phosphodiesterase OS=Rattus norvegicus GN=Cnp PE=1 SV=2</td><td>AHVTLGCAADVQPVQTGLDLLEILQQVK</td></tr>
<tr><td>></td><td>sp|P13233|CN 2',3'-cyclic-nucleotide 3'-phosphodiesterase OS=Rattus norvegicus GN=Cnp PE=1 SV=2</td><td>AHVTLGCAADVQPVQTGLDLLEILQQVK</td></tr>
<tr><td>></td><td>sp|P13233|CN 2',3'-cyclic-nucleotide 3'-phosphodiesterase OS=Rattus norvegicus GN=Cnp PE=1 SV=2</td><td>AHVTLGCAADVQPVQTGLDLLEILQQVK</td></tr>
<tr><td>></td><td>sp|P13233|CN 2',3'-cyclic-nucleotide 3'-phosphodiesterase OS=Rattus norvegicus GN=Cnp PE=1 SV=2</td><td>AHVTLGCAADVQPVQTGLDLLEILQQVK</td></tr>
<tr><td>></td><td>sp|P13233|CN 2',3'-cyclic-nucleotide 3'-phosphodiesterase OS=Rattus norvegicus GN=Cnp PE=1 SV=2</td><td>AHVTLGCAADVQPVQTGLDLLEILQQVK</td></tr>
<tr><td>></td><td>sp|P13233|CN 2',3'-cyclic-nucleotide 3'-phosphodiesterase OS=Rattus norvegicus GN=Cnp PE=1 SV=2</td><td>AHVTLGCAADVQPVQTGLDLLEILQQVK</td></tr>
<tr><td>></td><td>sp|P13233|CN 2',3'-cyclic-nucleotide 3'-phosphodiesterase OS=Rattus norvegicus GN=Cnp PE=1 SV=2</td><td>AHVTLGCAADVQPVQTGLDLLEILQQVK</td></tr>
<tr><td>></td><td>sp|P13233|CN 2',3'-cyclic-nucleotide 3'-phosphodiesterase OS=Rattus norvegicus GN=Cnp PE=1 SV=2</td><td>AHVTLGCAADVQPVQTGLDLLEILQQVK</td></tr>
<tr><td>></td><td>sp|P13233|CN 2',3'-cyclic-nucleotide 3'-phosphodiesterase OS=Rattus norvegicus GN=Cnp PE=1 SV=2</td><td>AHVTLGCAADVQPVQTGLDLLEILQQVK</td></tr>
<tr><td>></td><td>sp|P13233|CN 2',3'-cyclic-nucleotide 3'-phosphodiesterase OS=Rattus norvegicus GN=Cnp PE=1 SV=2</td><td>AHVTLGCAADVQPVQTGLDLLEILQQVK</td></tr>
<tr><td>></td><td>sp|P13233|CN 2',3'-cyclic-nucleotide 3'-phosphodiesterase OS=Rattus norvegicus GN=Cnp PE=1 SV=2</td><td>ATGAEEYAQQDVVR</td></tr>
<tr><td>></td><td>sp|P13233|CN 2',3'-cyclic-nucleotide 3'-phosphodiesterase OS=Rattus norvegicus GN=Cnp PE=1 SV=2</td><td>ATGAEEYAQQDVVR</td></tr>
<tr><td>></td><td>sp|P13233|CN 2',3'-cyclic-nucleotide 3'-phosphodiesterase OS=Rattus norvegicus GN=Cnp PE=1 SV=2</td><td>ATGAEEYAQQDVVR</td></tr>
<tr><td>></td><td>sp|P13233|CN 2',3'-cyclic-nucleotide 3'-phosphodiesterase OS=Rattus norvegicus GN=Cnp PE=1 SV=2</td><td>DKPELQFPFLQDEDTVATLHECK</td></tr>
<tr><td>></td><td>sp|P13233|CN 2',3'-cyclic-nucleotide 3'-phosphodiesterase OS=Rattus norvegicus GN=Cnp PE=1 SV=2</td><td>DKPELQFPFLQDEDTVATLHECK</td></tr>
<tr><td>></td><td>sp|P13233|CN 2',3'-cyclic-nucleotide 3'-phosphodiesterase OS=Rattus norvegicus GN=Cnp PE=1 SV=2</td><td>DKPELQFPFLQDEDTVATLHECK</td></tr>
<tr><td>></td><td>sp|P13233|CN 2',3'-cyclic-nucleotide 3'-phosphodiesterase OS=Rattus norvegicus GN=Cnp PE=1 SV=2</td><td>DKPELQFPFLQDEDTVATLHECK</td></tr>
<tr><td>></td><td>sp|P13233|CN 2',3'-cyclic-nucleotide 3'-phosphodiesterase OS=Rattus norvegicus GN=Cnp PE=1 SV=2</td><td>DKPELQFPFLQDEDTVATLHECK</td></tr>
<tr><td>></td><td>sp|P13233|CN 2',3'-cyclic-nucleotide 3'-phosphodiesterase OS=Rattus norvegicus GN=Cnp PE=1 SV=2</td><td>DKPELQFPFLQDEDTVATLHECK</td></tr>
<tr><td>></td><td>sp|P13233|CN 2',3'-cyclic-nucleotide 3'-phosphodiesterase OS=Rattus norvegicus GN=Cnp PE=1 SV=2</td><td>DKPELQFPFLQDEDTVATLHECK</td></tr>
<tr><td>></td><td>sp|P13233|CN 2',3'-cyclic-nucleotide 3'-phosphodiesterase OS=Rattus norvegicus GN=Cnp PE=1 SV=2</td><td>DKPELQFPFLQDEDTVATLHECK</td></tr>
<tr><td>></td><td>sp|P13233|CN 2',3'-cyclic-nucleotide 3'-phosphodiesterase OS=Rattus norvegicus GN=Cnp PE=1 SV=2</td><td>DKPELQFPFLQDEDTVATLHECK</td></tr>
<tr><td>></td><td>sp|P13233|CN 2',3'-cyclic-nucleotide 3'-phosphodiesterase OS=Rattus norvegicus GN=Cnp PE=1 SV=2</td><td>DKPELQFPFLQDEDTVATLHECK</td></tr>
<tr><td>></td><td>sp|P13233|CN 2',3'-cyclic-nucleotide 3'-phosphodiesterase OS=Rattus norvegicus GN=Cnp PE=1 SV=2</td><td>DKPELQFPFLQDEDTVATLHECK</td></tr>
<tr><td>></td><td>sp|P13233|CN 2',3'-cyclic-nucleotide 3'-phosphodiesterase OS=Rattus norvegicus GN=Cnp PE=1 SV=2</td><td>DKPELQFPFLQDEDTVATLHECK</td></tr>
<tr><td>></td><td>sp|P13233|CN 2',3'-cyclic-nucleotide 3'-phosphodiesterase OS=Rattus norvegicus GN=Cnp PE=1 SV=2</td><td>DKPELQFPFLQDEDTVATLHECK</td></tr>
<tr><td>></td><td>sp|P13233|CN 2',3'-cyclic-nucleotide 3'-phosphodiesterase OS=Rattus norvegicus GN=Cnp PE=1 SV=2</td><td>DKPELQFPFLQDEDTVATLHECK</td></tr>
<tr><td>></td><td>sp|P13233|CN 2',3'-cyclic-nucleotide 3'-phosphodiesterase OS=Rattus norvegicus GN=Cnp PE=1 SV=2</td><td>DKPELQFPFLQDEDTVATLHECK</td></tr>
<tr><td>></td><td>sp|P13233|CN 2',3'-cyclic-nucleotide 3'-phosphodiesterase OS=Rattus norvegicus GN=Cnp PE=1 SV=2</td><td>DKPELQFPFLQDEDTVATLHECK</td></tr>
<tr><td>></td><td>sp|P13233|CN 2',3'-cyclic-nucleotide 3'-phosphodiesterase OS=Rattus norvegicus GN=Cnp PE=1 SV=2</td><td>DKPELQFPFLQDEDTVATLHECK</td></tr>
<tr><td>></td><td>sp|P13233|CN 2',3'-cyclic-nucleotide 3'-phosphodiesterase OS=Rattus norvegicus GN=Cnp PE=1 SV=2</td><td>DKPELQFPFLQDEDTVATLHECK</td></tr>
<tr><td>></td><td>sp|P13233|CN 2',3'-cyclic-nucleotide 3'-phosphodiesterase OS=Rattus norvegicus GN=Cnp PE=1 SV=2</td><td>EKLDLVSYFGK</td></tr>
<tr><td>></td><td>sp|P13233|CN 2',3'-cyclic-nucleotide 3'-phosphodiesterase OS=Rattus norvegicus GN=Cnp PE=1 SV=2</td><td>EKLDLVSYFGK</td></tr>
<tr><td>></td><td>sp|P13233|CN 2',3'-cyclic-nucleotide 3'-phosphodiesterase OS=Rattus norvegicus GN=Cnp PE=1 SV=2</td><td>ERLDQLFEMADQYQYQVVLVEPK</td></tr>
<tr><td>></td><td>sp|P13233|CN 2',3'-cyclic-nucleotide 3'-phosphodiesterase OS=Rattus norvegicus GN=Cnp PE=1 SV=2</td><td>ERLDQLFEMADQYQYQVVLVEPK</td></tr>
</table>

>	sp|P13233|CN 2',3'-cyclic-nucleotide 3'-phosphodiesterase OS=Rattus norvegicus GN=Cnp PE=1 SV=2	ERLDQLFEMADQYQYQVVLVEPK
>	sp|P13233|CN 2',3'-cyclic-nucleotide 3'-phosphodiesterase OS=Rattus norvegicus GN=Cnp PE=1 SV=2	ERLDQLFEMADQYQYQVVLVEPK
>	sp|P13233|CN 2',3'-cyclic-nucleotide 3'-phosphodiesterase OS=Rattus norvegicus GN=Cnp PE=1 SV=2	ERLDQLFEMADQYQYQVVLVEPK
>	sp|P13233|CN 2',3'-cyclic-nucleotide 3'-phosphodiesterase OS=Rattus norvegicus GN=Cnp PE=1 SV=2	ERLDQLFEMADQYQYQVVLVEPK
>	sp|P13233|CN 2',3'-cyclic-nucleotide 3'-phosphodiesterase OS=Rattus norvegicus GN=Cnp PE=1 SV=2	ERLDQLFEMADQYQYQVVLVEPK
>	sp|P13233|CN 2',3'-cyclic-nucleotide 3'-phosphodiesterase OS=Rattus norvegicus GN=Cnp PE=1 SV=2	ERLDQLFEMADQYQYQVVLVEPK
>	sp|P13233|CN 2',3'-cyclic-nucleotide 3'-phosphodiesterase OS=Rattus norvegicus GN=Cnp PE=1 SV=2	ERLDQLFEMADQYQYQVVLVEPK
>	sp|P13233|CN 2',3'-cyclic-nucleotide 3'-phosphodiesterase OS=Rattus norvegicus GN=Cnp PE=1 SV=2	ERLDQLFEMADQYQYQVVLVEPK
>	sp|P13233|CN 2',3'-cyclic-nucleotide 3'-phosphodiesterase OS=Rattus norvegicus GN=Cnp PE=1 SV=2	GGSQGEEVGELPR
>	sp|P13233|CN 2',3'-cyclic-nucleotide 3'-phosphodiesterase OS=Rattus norvegicus GN=Cnp PE=1 SV=2	GGSQGEEVGELPR
>	sp|P13233|CN 2',3'-cyclic-nucleotide 3'-phosphodiesterase OS=Rattus norvegicus GN=Cnp PE=1 SV=2	GGSQGEEVGELPR
>	sp|P13233|CN 2',3'-cyclic-nucleotide 3'-phosphodiesterase OS=Rattus norvegicus GN=Cnp PE=1 SV=2	GGSQGEEVGELPR
>	sp|P13233|CN 2',3'-cyclic-nucleotide 3'-phosphodiesterase OS=Rattus norvegicus GN=Cnp PE=1 SV=2	GGSQGEEVGELPR
>	sp|P13233|CN 2',3'-cyclic-nucleotide 3'-phosphodiesterase OS=Rattus norvegicus GN=Cnp PE=1 SV=2	GGSQGEEVGELPR
>	sp|P13233|CN 2',3'-cyclic-nucleotide 3'-phosphodiesterase OS=Rattus norvegicus GN=Cnp PE=1 SV=2	GGSQGEEVGELPR
>	sp|P13233|CN 2',3'-cyclic-nucleotide 3'-phosphodiesterase OS=Rattus norvegicus GN=Cnp PE=1 SV=2	KAGQVFLEELGNHK
>	sp|P13233|CN 2',3'-cyclic-nucleotide 3'-phosphodiesterase OS=Rattus norvegicus GN=Cnp PE=1 SV=2	KLKPGLEKDFLPLYFGWFLTKK
>	sp|P13233|CN 2',3'-cyclic-nucleotide 3'-phosphodiesterase OS=Rattus norvegicus GN=Cnp PE=1 SV=2	LDLVSYFGK
>	sp|P13233|CN 2',3'-cyclic-nucleotide 3'-phosphodiesterase OS=Rattus norvegicus GN=Cnp PE=1 SV=2	LDLVSYFGK
>	sp|P13233|CN 2',3'-cyclic-nucleotide 3'-phosphodiesterase OS=Rattus norvegicus GN=Cnp PE=1 SV=2	LDLVSYFGK
>	sp|P13233|CN 2',3'-cyclic-nucleotide 3'-phosphodiesterase OS=Rattus norvegicus GN=Cnp PE=1 SV=2	LDQLFEMADQYQYQVVLVEPK
>	sp|P13233|CN 2',3'-cyclic-nucleotide 3'-phosphodiesterase OS=Rattus norvegicus GN=Cnp PE=1 SV=2	LDQLFEMADQYQYQVVLVEPK
>	sp|P13233|CN 2',3'-cyclic-nucleotide 3'-phosphodiesterase OS=Rattus norvegicus GN=Cnp PE=1 SV=2	LDQLFEMADQYQYQVVLVEPK
>	sp|P13233|CN 2',3'-cyclic-nucleotide 3'-phosphodiesterase OS=Rattus norvegicus GN=Cnp PE=1 SV=2	LKPGLEKDFLPLYFGWFLTK
>	sp|P13233|CN 2',3'-cyclic-nucleotide 3'-phosphodiesterase OS=Rattus norvegicus GN=Cnp PE=1 SV=2	LKPGLEKDFLPLYFGWFLTK
>	sp|P13233|CN 2',3'-cyclic-nucleotide 3'-phosphodiesterase OS=Rattus norvegicus GN=Cnp PE=1 SV=2	LKPGLEKDFLPLYFGWFLTK
>	sp|P13233|CN 2',3'-cyclic-nucleotide 3'-phosphodiesterase OS=Rattus norvegicus GN=Cnp PE=1 SV=2	LKPGLEKDFLPLYFGWFLTK
>	sp|P13233|CN 2',3'-cyclic-nucleotide 3'-phosphodiesterase OS=Rattus norvegicus GN=Cnp PE=1 SV=2	LKPGLEKDFLPLYFGWFLTK
>	sp|P13233|CN 2',3'-cyclic-nucleotide 3'-phosphodiesterase OS=Rattus norvegicus GN=Cnp PE=1 SV=2	LKPGLEKDFLPLYFGWFLTK
>	sp|P13233|CN 2',3'-cyclic-nucleotide 3'-phosphodiesterase OS=Rattus norvegicus GN=Cnp PE=1 SV=2	LKPGLEKDFLPLYFGWFLTK
>	sp|P13233|CN 2',3'-cyclic-nucleotide 3'-phosphodiesterase OS=Rattus norvegicus GN=Cnp PE=1 SV=2	LKPGLEKDFLPLYFGWFLTK
>	sp|P13233|CN 2',3'-cyclic-nucleotide 3'-phosphodiesterase OS=Rattus norvegicus GN=Cnp PE=1 SV=2	LKPGLEKDFLPLYFGWFLTK
>	sp|P13233|CN 2',3'-cyclic-nucleotide 3'-phosphodiesterase OS=Rattus norvegicus GN=Cnp PE=1 SV=2	LKPGLEKDFLPLYFGWFLTK
>	sp|P13233|CN 2',3'-cyclic-nucleotide 3'-phosphodiesterase OS=Rattus norvegicus GN=Cnp PE=1 SV=2	LKPGLEKDFLPLYFGWFLTK
>	sp|P13233|CN 2',3'-cyclic-nucleotide 3'-phosphodiesterase OS=Rattus norvegicus GN=Cnp PE=1 SV=2	LKPGLEKDFLPLYFGWFLTK
>	sp|P13233|CN 2',3'-cyclic-nucleotide 3'-phosphodiesterase OS=Rattus norvegicus GN=Cnp PE=1 SV=2	LKPGLEKDFLPLYFGWFLTK
>	sp|P13233|CN 2',3'-cyclic-nucleotide 3'-phosphodiesterase OS=Rattus norvegicus GN=Cnp PE=1 SV=2	LKPGLEKDFLPLYFGWFLTK
>	sp|P13233|CN 2',3'-cyclic-nucleotide 3'-phosphodiesterase OS=Rattus norvegicus GN=Cnp PE=1 SV=2	LKPGLEKDFLPLYFGWFLTKK
>	sp|P13233|CN 2',3'-cyclic-nucleotide 3'-phosphodiesterase OS=Rattus norvegicus GN=Cnp PE=1 SV=2	LKPGLEKDFLPLYFGWFLTKK
>	sp|P13233|CN 2',3'-cyclic-nucleotide 3'-phosphodiesterase OS=Rattus norvegicus GN=Cnp PE=1 SV=2	LKPGLEKDFLPLYFGWFLTKK
>	sp|P13233|CN 2',3'-cyclic-nucleotide 3'-phosphodiesterase OS=Rattus norvegicus GN=Cnp PE=1 SV=2	LKPGLEKDFLPLYFGWFLTKK
>	sp|P13233|CN 2',3'-cyclic-nucleotide 3'-phosphodiesterase OS=Rattus norvegicus GN=Cnp PE=1 SV=2	LKPGLEKDFLPLYFGWFLTKK
>	sp|P13233|CN 2',3'-cyclic-nucleotide 3'-phosphodiesterase OS=Rattus norvegicus GN=Cnp PE=1 SV=2	LKPGLEKDFLPLYFGWFLTKK
>	sp|P13233|CN 2',3'-cyclic-nucleotide 3'-phosphodiesterase OS=Rattus norvegicus GN=Cnp PE=1 SV=2	LKPGLEKDFLPLYFGWFLTKK
>	sp|P13233|CN 2',3'-cyclic-nucleotide 3'-phosphodiesterase OS=Rattus norvegicus GN=Cnp PE=1 SV=2	LKPGLEKDFLPLYFGWFLTKK
>	sp|P13233|CN 2',3'-cyclic-nucleotide 3'-phosphodiesterase OS=Rattus norvegicus GN=Cnp PE=1 SV=2	LKPGLEKDFLPLYFGWFLTKK
>	sp|P13233|CN 2',3'-cyclic-nucleotide 3'-phosphodiesterase OS=Rattus norvegicus GN=Cnp PE=1 SV=2	LKPGLEKDFLPLYFGWFLTKK
>	sp|P13233|CN 2',3'-cyclic-nucleotide 3'-phosphodiesterase OS=Rattus norvegicus GN=Cnp PE=1 SV=2	LKPGLEKDFLPLYFGWFLTKK
>	sp|P13233|CN 2',3'-cyclic-nucleotide 3'-phosphodiesterase OS=Rattus norvegicus GN=Cnp PE=1 SV=2	LKPGLEKDFLPLYFGWFLTKK
>	sp|P13233|CN 2',3'-cyclic-nucleotide 3'-phosphodiesterase OS=Rattus norvegicus GN=Cnp PE=1 SV=2	LKPGLEKDFLPLYFGWFLTKK
>	sp|P13233|CN 2',3'-cyclic-nucleotide 3'-phosphodiesterase OS=Rattus norvegicus GN=Cnp PE=1 SV=2	LKPGLEKDFLPLYFGWFLTKK
>	sp|P13233|CN 2',3'-cyclic-nucleotide 3'-phosphodiesterase OS=Rattus norvegicus GN=Cnp PE=1 SV=2	LKPGLEKDFLPLYFGWFLTKK
>	sp|P13233|CN 2',3'-cyclic-nucleotide 3'-phosphodiesterase OS=Rattus norvegicus GN=Cnp PE=1 SV=2	LKPGLEKDFLPLYFGWFLTKK
>	sp|P13233|CN 2',3'-cyclic-nucleotide 3'-phosphodiesterase OS=Rattus norvegicus GN=Cnp PE=1 SV=2	LKPGLEKDFLPLYFGWFLTKK
>	sp|P13233|CN 2',3'-cyclic-nucleotide 3'-phosphodiesterase OS=Rattus norvegicus GN=Cnp PE=1 SV=2	LSISALFVTPK
>	sp|P13233|CN 2',3'-cyclic-nucleotide 3'-phosphodiesterase OS=Rattus norvegicus GN=Cnp PE=1 SV=2	LSISALFVTPK
>	sp|P13233|CN 2',3'-cyclic-nucleotide 3'-phosphodiesterase OS=Rattus norvegicus GN=Cnp PE=1 SV=2	LSISALFVTPK
>	sp|P13233|CN 2',3'-cyclic-nucleotide 3'-phosphodiesterase OS=Rattus norvegicus GN=Cnp PE=1 SV=2	NQWQLSLDDLK
>	sp|P13233|CN 2',3'-cyclic-nucleotide 3'-phosphodiesterase OS=Rattus norvegicus GN=Cnp PE=1 SV=2	RLDEDLAGYCR
>	sp|P13233|CN 2',3'-cyclic-nucleotide 3'-phosphodiesterase OS=Rattus norvegicus GN=Cnp PE=1 SV=2	RLDEDLAGYCR
>	sp|P13233|CN 2',3'-cyclic-nucleotide 3'-phosphodiesterase OS=Rattus norvegicus GN=Cnp PE=1 SV=2	RLDEDLAGYCR
>	sp|P13233|CN 2',3'-cyclic-nucleotide 3'-phosphodiesterase OS=Rattus norvegicus GN=Cnp PE=1 SV=2	RLDEDLAGYCR
>	sp|P13233|CN 2',3'-cyclic-nucleotide 3'-phosphodiesterase OS=Rattus norvegicus GN=Cnp PE=1 SV=2	RLDEDLAGYCR
>	sp|P13233|CN 2',3'-cyclic-nucleotide 3'-phosphodiesterase OS=Rattus norvegicus GN=Cnp PE=1 SV=2	RLDEDLAGYCR
>	sp|P13233|CN 2',3'-cyclic-nucleotide 3'-phosphodiesterase OS=Rattus norvegicus GN=Cnp PE=1 SV=2	RLDEDLAGYCR
>	sp|P13233|CN 2',3'-cyclic-nucleotide 3'-phosphodiesterase OS=Rattus norvegicus GN=Cnp PE=1 SV=2	RLDEDLAGYCR
>	sp|P13233|CN 2',3'-cyclic-nucleotide 3'-phosphodiesterase OS=Rattus norvegicus GN=Cnp PE=1 SV=2	TAGAQVVLNEQELQLWPSDLDKPSSSESLPPGSR
>	sp|P13233|CN 2',3'-cyclic-nucleotide 3'-phosphodiesterase OS=Rattus norvegicus GN=Cnp PE=1 SV=2	AGQVFLEELGNHK
>	sp|P13233|CN 2',3'-cyclic-nucleotide 3'-phosphodiesterase OS=Rattus norvegicus GN=Cnp PE=1 SV=2	DKPELQFPFLQDEDTVATLHECK
>	sp|P13233|CN 2',3'-cyclic-nucleotide 3'-phosphodiesterase OS=Rattus norvegicus GN=Cnp PE=1 SV=2	ATGAEEYAQQDVVR
>	sp|P13233|CN 2',3'-cyclic-nucleotide 3'-phosphodiesterase OS=Rattus norvegicus GN=Cnp PE=1 SV=2	DKPELQFPFLQDEDTVATLHECK
>	sp|P13233|CN 2',3'-cyclic-nucleotide 3'-phosphodiesterase OS=Rattus norvegicus GN=Cnp PE=1 SV=2	GGSQGEEVGELPR
>	sp|P13233|CN 2',3'-cyclic-nucleotide 3'-phosphodiesterase OS=Rattus norvegicus GN=Cnp PE=1 SV=2	VLVLDDTNHERER
>	sp|P13233|CN 2',3'-cyclic-nucleotide 3'-phosphodiesterase OS=Rattus norvegicus GN=Cnp PE=1 SV=2	LDLVSYFGK

>	sp|P13233|CN	2',3'-cyclic-nucleotide 3'-phosphodiesterase OS=Rattus norvegicus GN=Cnp PE=1 SV=2	AHVTLGCAADVQPVQTGLDLLEILQQVK
>	sp|P13233|CN	2',3'-cyclic-nucleotide 3'-phosphodiesterase OS=Rattus norvegicus GN=Cnp PE=1 SV=2	AHVTLGCAADVQPVQTGLDLLEILQQVK
>	sp|P13233|CN	2',3'-cyclic-nucleotide 3'-phosphodiesterase OS=Rattus norvegicus GN=Cnp PE=1 SV=2	DKPELQFPFLQDEDTVATLHECK
>	sp|P13233|CN	2',3'-cyclic-nucleotide 3'-phosphodiesterase OS=Rattus norvegicus GN=Cnp PE=1 SV=2	DKPELQFPFLQDEDTVATLHECK
>	sp|P13233|CN	2',3'-cyclic-nucleotide 3'-phosphodiesterase OS=Rattus norvegicus GN=Cnp PE=1 SV=2	LDEDLAGYCR
>	sp|P13233|CN	2',3'-cyclic-nucleotide 3'-phosphodiesterase OS=Rattus norvegicus GN=Cnp PE=1 SV=2	WMLSLAK
>	sp|P13233|CN	2',3'-cyclic-nucleotide 3'-phosphodiesterase OS=Rattus norvegicus GN=Cnp PE=1 SV=2	LDQLFEMADQYQYQVVLVEPK
>	sp|P13233|CN	2',3'-cyclic-nucleotide 3'-phosphodiesterase OS=Rattus norvegicus GN=Cnp PE=1 SV=2	LDLVSYFGK
>	sp|P13233|CN	2',3'-cyclic-nucleotide 3'-phosphodiesterase OS=Rattus norvegicus GN=Cnp PE=1 SV=2	AHVTLGCAADVQPVQTGLDLLEILQQVK
>	sp|P13233|CN	2',3'-cyclic-nucleotide 3'-phosphodiesterase OS=Rattus norvegicus GN=Cnp PE=1 SV=2	AHVTLGCAADVQPVQTGLDLLEILQQVK
>	sp|P13233|CN	2',3'-cyclic-nucleotide 3'-phosphodiesterase OS=Rattus norvegicus GN=Cnp PE=1 SV=2	AHVTLGCAADVQPVQTGLDLLEILQQVK
>	sp|P13233|CN	2',3'-cyclic-nucleotide 3'-phosphodiesterase OS=Rattus norvegicus GN=Cnp PE=1 SV=2	GGSQGEEVGELPR
>	sp|P13233|CN	2',3'-cyclic-nucleotide 3'-phosphodiesterase OS=Rattus norvegicus GN=Cnp PE=1 SV=2	KAGQVFLEELGNHK
>	sp|P13233|CN	2',3'-cyclic-nucleotide 3'-phosphodiesterase OS=Rattus norvegicus GN=Cnp PE=1 SV=2	LDLVSYFGK
>	sp|P13233|CN	2',3'-cyclic-nucleotide 3'-phosphodiesterase OS=Rattus norvegicus GN=Cnp PE=1 SV=2	LDLVSYFGK
>	sp|P13233|CN	2',3'-cyclic-nucleotide 3'-phosphodiesterase OS=Rattus norvegicus GN=Cnp PE=1 SV=2	NQWQLSLDDLKK
>	sp|P13233|CN	2',3'-cyclic-nucleotide 3'-phosphodiesterase OS=Rattus norvegicus GN=Cnp PE=1 SV=2	VLVLDDTNHER
>	sp|P13233|CN	2',3'-cyclic-nucleotide 3'-phosphodiesterase OS=Rattus norvegicus GN=Cnp PE=1 SV=2	DKPELQFPFLQDEDTVATLHECK
>	sp|P13233|CN	2',3'-cyclic-nucleotide 3'-phosphodiesterase OS=Rattus norvegicus GN=Cnp PE=1 SV=2	NQWQLSLDDLK
>	sp|P13233|CN	2',3'-cyclic-nucleotide 3'-phosphodiesterase OS=Rattus norvegicus GN=Cnp PE=1 SV=2	NQWQLSLDDLKK
>	sp|P13233|CN	2',3'-cyclic-nucleotide 3'-phosphodiesterase OS=Rattus norvegicus GN=Cnp PE=1 SV=2	LKPGLEKDFLPLYFGWFLTK
>	sp|P13233|CN	2',3'-cyclic-nucleotide 3'-phosphodiesterase OS=Rattus norvegicus GN=Cnp PE=1 SV=2	NQWQLSLDDLK
>	sp|P13233|CN	2',3'-cyclic-nucleotide 3'-phosphodiesterase OS=Rattus norvegicus GN=Cnp PE=1 SV=2	HFISGDEPKEK
>	sp|P13233|CN	2',3'-cyclic-nucleotide 3'-phosphodiesterase OS=Rattus norvegicus GN=Cnp PE=1 SV=2	ATGAEEYAQQDVVR
>	sp|P13233|CN	2',3'-cyclic-nucleotide 3'-phosphodiesterase OS=Rattus norvegicus GN=Cnp PE=1 SV=2	KLKPGLEKDFLPLYFGWFLTKK
>	sp|P13233|CN	2',3'-cyclic-nucleotide 3'-phosphodiesterase OS=Rattus norvegicus GN=Cnp PE=1 SV=2	AHVTLGCAADVQPVQTGLDLLEILQQVK
>	sp|P13233|CN	2',3'-cyclic-nucleotide 3'-phosphodiesterase OS=Rattus norvegicus GN=Cnp PE=1 SV=2	WMLSLAK
>	sp|P13233|CN	2',3'-cyclic-nucleotide 3'-phosphodiesterase OS=Rattus norvegicus GN=Cnp PE=1 SV=2	NQWQLSLDDLKK
>	sp|P13233|CN	2',3'-cyclic-nucleotide 3'-phosphodiesterase OS=Rattus norvegicus GN=Cnp PE=1 SV=2	ADFSEEYKR
>	sp|P13233|CN	2',3'-cyclic-nucleotide 3'-phosphodiesterase OS=Rattus norvegicus GN=Cnp PE=1 SV=2	LDEDLAGYCR
>	sp|P13233|CN	2',3'-cyclic-nucleotide 3'-phosphodiesterase OS=Rattus norvegicus GN=Cnp PE=1 SV=2	RLDEDLAGYCR
>	sp|P13233|CN	2',3'-cyclic-nucleotide 3'-phosphodiesterase OS=Rattus norvegicus GN=Cnp PE=1 SV=2	DKPELQFPFLQDEDTVATLHECK
>	sp|P13233|CN	2',3'-cyclic-nucleotide 3'-phosphodiesterase OS=Rattus norvegicus GN=Cnp PE=1 SV=2	ATGAEEYAQQDVVRR
>	sp|P13233|CN	2',3'-cyclic-nucleotide 3'-phosphodiesterase OS=Rattus norvegicus GN=Cnp PE=1 SV=2	LDLVSYFGK
>	sp|P13233|CN	2',3'-cyclic-nucleotide 3'-phosphodiesterase OS=Rattus norvegicus GN=Cnp PE=1 SV=2	LEELGNHK
>	sp|P13233|CN	2',3'-cyclic-nucleotide 3'-phosphodiesterase OS=Rattus norvegicus GN=Cnp PE=1 SV=2	VLVLDDTNHER
>	sp|P13233|CN	2',3'-cyclic-nucleotide 3'-phosphodiesterase OS=Rattus norvegicus GN=Cnp PE=1 SV=2	LKPGLEKDFLPLYFGWFLTK
>	sp|P13233|CN	2',3'-cyclic-nucleotide 3'-phosphodiesterase OS=Rattus norvegicus GN=Cnp PE=1 SV=2	VLVLDDTNHER
>	sp|P13233|CN	2',3'-cyclic-nucleotide 3'-phosphodiesterase OS=Rattus norvegicus GN=Cnp PE=1 SV=2	KAGQVFLEELGNHK
>	sp|P13233|CN	2',3'-cyclic-nucleotide 3'-phosphodiesterase OS=Rattus norvegicus GN=Cnp PE=1 SV=2	DKPELQFPFLQDEDTVATLHECK
>	sp|P13233|CN	2',3'-cyclic-nucleotide 3'-phosphodiesterase OS=Rattus norvegicus GN=Cnp PE=1 SV=2	DKPELQFPFLQDEDTVATLHECK
>	sp|P13233|CN	2',3'-cyclic-nucleotide 3'-phosphodiesterase OS=Rattus norvegicus GN=Cnp PE=1 SV=2	KAGQVFLEELGNHK
>	sp|P13233|CN	2',3'-cyclic-nucleotide 3'-phosphodiesterase OS=Rattus norvegicus GN=Cnp PE=1 SV=2	KAGQVFLEELGNHK
>	sp|P13233|CN	2',3'-cyclic-nucleotide 3'-phosphodiesterase OS=Rattus norvegicus GN=Cnp PE=1 SV=2	AHVTLGCAADVQPVQTGLDLLEILQQVK
>	sp|P13233|CN	2',3'-cyclic-nucleotide 3'-phosphodiesterase OS=Rattus norvegicus GN=Cnp PE=1 SV=2	HFISGDEPK
>	sp|P13233|CN	2',3'-cyclic-nucleotide 3'-phosphodiesterase OS=Rattus norvegicus GN=Cnp PE=1 SV=2	TAGAQVVLNEQELQLWPSDLDKPSSSESLPPGSR
>	sp|P13233|CN	2',3'-cyclic-nucleotide 3'-phosphodiesterase OS=Rattus norvegicus GN=Cnp PE=1 SV=2	GGSQGEEVGELPR
>	sp|P13233|CN	2',3'-cyclic-nucleotide 3'-phosphodiesterase OS=Rattus norvegicus GN=Cnp PE=1 SV=2	LKPGLEKDFLPLYFGWFLTK
>	sp|P13233|CN	2',3'-cyclic-nucleotide 3'-phosphodiesterase OS=Rattus norvegicus GN=Cnp PE=1 SV=2	LEELGNHK
>	sp|P13233|CN	2',3'-cyclic-nucleotide 3'-phosphodiesterase OS=Rattus norvegicus GN=Cnp PE=1 SV=2	KAGQVFLEELGNHK
>	sp|P13233|CN	2',3'-cyclic-nucleotide 3'-phosphodiesterase OS=Rattus norvegicus GN=Cnp PE=1 SV=2	WMLSLAK
>	sp|P13233|CN	2',3'-cyclic-nucleotide 3'-phosphodiesterase OS=Rattus norvegicus GN=Cnp PE=1 SV=2	AGQVFLEELGNHK
>	sp|P13233|CN	2',3'-cyclic-nucleotide 3'-phosphodiesterase OS=Rattus norvegicus GN=Cnp PE=1 SV=2	GGSQGEEVGELPR
>	sp|P13233|CN	2',3'-cyclic-nucleotide 3'-phosphodiesterase OS=Rattus norvegicus GN=Cnp PE=1 SV=2	AHVTLGCAADVQPVQTGLDLLEILQQVK
>	sp|P13233|CN	2',3'-cyclic-nucleotide 3'-phosphodiesterase OS=Rattus norvegicus GN=Cnp PE=1 SV=2	MVSADAYK
>	sp|P13233|CN	2',3'-cyclic-nucleotide 3'-phosphodiesterase OS=Rattus norvegicus GN=Cnp PE=1 SV=2	LSISALFVTPK
>	sp|P13233|CN	2',3'-cyclic-nucleotide 3'-phosphodiesterase OS=Rattus norvegicus GN=Cnp PE=1 SV=2	RPPGVLHCTTK
>	sp|P13233|CN	2',3'-cyclic-nucleotide 3'-phosphodiesterase OS=Rattus norvegicus GN=Cnp PE=1 SV=2	DKPELQFPFLQDEDTVATLHECK
>	sp|P13233|CN	2',3'-cyclic-nucleotide 3'-phosphodiesterase OS=Rattus norvegicus GN=Cnp PE=1 SV=2	HFISGDEPKEK
>	sp|P13233|CN	2',3'-cyclic-nucleotide 3'-phosphodiesterase OS=Rattus norvegicus GN=Cnp PE=1 SV=2	KAGQVFLEELGNHK
>	sp|P21575|DY	Dynamin-1 OS=Rattus norvegicus GN=Dnm1 PE=1 SV=2	ALLQMVQQFAVDFEK
>	sp|P21575|DY	Dynamin-1 OS=Rattus norvegicus GN=Dnm1 PE=1 SV=2	ALLQMVQQFAVDFEKR
>	sp|P21575|DY	Dynamin-1 OS=Rattus norvegicus GN=Dnm1 PE=1 SV=2	HIFALFNTEQR
>	sp|P21575|DY	Dynamin-1 OS=Rattus norvegicus GN=Dnm1 PE=1 SV=2	KDITAALAAER
>	sp|P21575|DY	Dynamin-1 OS=Rattus norvegicus GN=Dnm1 PE=1 SV=2	KGWLTINNIGIMK
>	sp|P21575|DY	Dynamin-1 OS=Rattus norvegicus GN=Dnm1 PE=1 SV=2	LQDAFSAIGQNADLDLPQIAVVGGQSAGK
>	sp|P21575|DY	Dynamin-1 OS=Rattus norvegicus GN=Dnm1 PE=1 SV=2	NKLQSQLLSIEKEVDEYK
>	sp|P21575|DY	Dynamin-1 OS=Rattus norvegicus GN=Dnm1 PE=1 SV=2	NLVDSYMAIVNK
>	sp|P21575|DY	Dynamin-1 OS=Rattus norvegicus GN=Dnm1 PE=1 SV=2	PGMTK
>	sp|P21575|DY	Dynamin-1 OS=Rattus norvegicus GN=Dnm1 PE=1 SV=2	QVETIRNLVDSYMAIVNKTVRDLMPK
>	sp|P21575|DY	Dynamin-1 OS=Rattus norvegicus GN=Dnm1 PE=1 SV=2	RIEGSGDQIDTYELSGGAR
>	sp|P21575|DY	Dynamin-1 OS=Rattus norvegicus GN=Dnm1 PE=1 SV=2	RPLVLQLVNSTTEYAEFLHCK
>	sp|P21575|DY	Dynamin-1 OS=Rattus norvegicus GN=Dnm1 PE=1 SV=2	TGLFTPDLAFEATVK
>	sp|P21575|DY	Dynamin-1 OS=Rattus norvegicus GN=Dnm1 PE=1 SV=2	VPVGDQPPDIEFQIR

>	sp|P21575|DYN	Dynamin-1 OS=Rattus norvegicus GN=Dnm1 PE=1 SV=2	LQSQLLSIEKEVDEYKNFRPDDPAR
>	sp|P21575|DYN	Dynamin-1 OS=Rattus norvegicus GN=Dnm1 PE=1 SV=2	TKEQVMLLIDIELAYMNTNHEDFIGFANAQQR
>	sp|P21575|DYN	Dynamin-1 OS=Rattus norvegicus GN=Dnm1 PE=1 SV=2	ALLQMVQQFAVDFEK
>	sp|P21575|DYN	Dynamin-1 OS=Rattus norvegicus GN=Dnm1 PE=1 SV=2	ALLQMVQQFAVDFEK
>	sp|P21575|DYN	Dynamin-1 OS=Rattus norvegicus GN=Dnm1 PE=1 SV=2	HIFALFNTEQR
>	sp|P21575|DYN	Dynamin-1 OS=Rattus norvegicus GN=Dnm1 PE=1 SV=2	HIFALFNTEQR
>	sp|P21575|DYN	Dynamin-1 OS=Rattus norvegicus GN=Dnm1 PE=1 SV=2	HIFALFNTEQR
>	sp|P21575|DYN	Dynamin-1 OS=Rattus norvegicus GN=Dnm1 PE=1 SV=2	HIFALFNTEQR
>	sp|P21575|DYN	Dynamin-1 OS=Rattus norvegicus GN=Dnm1 PE=1 SV=2	HIFALFNTEQR
>	sp|P21575|DYN	Dynamin-1 OS=Rattus norvegicus GN=Dnm1 PE=1 SV=2	HIFALFNTEQR
>	sp|P21575|DYN	Dynamin-1 OS=Rattus norvegicus GN=Dnm1 PE=1 SV=2	HIFALFNTEQR
>	sp|P21575|DYN	Dynamin-1 OS=Rattus norvegicus GN=Dnm1 PE=1 SV=2	HIFALFNTEQR
>	sp|P21575|DYN	Dynamin-1 OS=Rattus norvegicus GN=Dnm1 PE=1 SV=2	KDITAALAAER
>	sp|P21575|DYN	Dynamin-1 OS=Rattus norvegicus GN=Dnm1 PE=1 SV=2	KDITAALAAER
>	sp|P21575|DYN	Dynamin-1 OS=Rattus norvegicus GN=Dnm1 PE=1 SV=2	KGWLTINNIGIMK
>	sp|P21575|DYN	Dynamin-1 OS=Rattus norvegicus GN=Dnm1 PE=1 SV=2	KGWLTINNIGIMK
>	sp|P21575|DYN	Dynamin-1 OS=Rattus norvegicus GN=Dnm1 PE=1 SV=2	KGWLTINNIGIMK
>	sp|P21575|DYN	Dynamin-1 OS=Rattus norvegicus GN=Dnm1 PE=1 SV=2	KGWLTINNIGIMK
>	sp|P21575|DYN	Dynamin-1 OS=Rattus norvegicus GN=Dnm1 PE=1 SV=2	LQSQLLSIEKEVDEYKNFRPDDPAR
>	sp|P21575|DYN	Dynamin-1 OS=Rattus norvegicus GN=Dnm1 PE=1 SV=2	NKLQSQLLSIEKEVDEYK
>	sp|P21575|DYN	Dynamin-1 OS=Rattus norvegicus GN=Dnm1 PE=1 SV=2	NKLQSQLLSIEKEVDEYK
>	sp|P21575|DYN	Dynamin-1 OS=Rattus norvegicus GN=Dnm1 PE=1 SV=2	NKLQSQLLSIEKEVDEYK
>	sp|P21575|DYN	Dynamin-1 OS=Rattus norvegicus GN=Dnm1 PE=1 SV=2	RIEGSGDQIDTYELSGGAR
>	sp|P21575|DYN	Dynamin-1 OS=Rattus norvegicus GN=Dnm1 PE=1 SV=2	RIEGSGDQIDTYELSGGAR
>	sp|P21575|DYN	Dynamin-1 OS=Rattus norvegicus GN=Dnm1 PE=1 SV=2	RPLVLQLVNSTTEYAEFLHCK
>	sp|P21575|DYN	Dynamin-1 OS=Rattus norvegicus GN=Dnm1 PE=1 SV=2	RPLVLQLVNSTTEYAEFLHCK
>	sp|P21575|DYN	Dynamin-1 OS=Rattus norvegicus GN=Dnm1 PE=1 SV=2	RPLVLQLVNSTTEYAEFLHCK
>	sp|P21575|DYN	Dynamin-1 OS=Rattus norvegicus GN=Dnm1 PE=1 SV=2	RPLVLQLVNSTTEYAEFLHCK
>	sp|P21575|DYN	Dynamin-1 OS=Rattus norvegicus GN=Dnm1 PE=1 SV=2	RPLVLQLVNSTTEYAEFLHCK
>	sp|P21575|DYN	Dynamin-1 OS=Rattus norvegicus GN=Dnm1 PE=1 SV=2	RPLVLQLVNSTTEYAEFLHCK
>	sp|P21575|DYN	Dynamin-1 OS=Rattus norvegicus GN=Dnm1 PE=1 SV=2	RPLVLQLVNSTTEYAEFLHCK
>	sp|P21575|DYN	Dynamin-1 OS=Rattus norvegicus GN=Dnm1 PE=1 SV=2	RPLVLQLVNSTTEYAEFLHCK
>	sp|P21575|DYN	Dynamin-1 OS=Rattus norvegicus GN=Dnm1 PE=1 SV=2	TKEQVMLLIDIELAYMNTNHEDFIGFANAQQR
>	sp|P21575|DYN	Dynamin-1 OS=Rattus norvegicus GN=Dnm1 PE=1 SV=2	RPLVLQLVNSTTEYAEFLHCK
>	sp|P21575|DYN	Dynamin-1 OS=Rattus norvegicus GN=Dnm1 PE=1 SV=2	RPLVLQLVNSTTEYAEFLHCK
>	sp|P21575|DYN	Dynamin-1 OS=Rattus norvegicus GN=Dnm1 PE=1 SV=2	LDLMDEGTDAR
>	sp|P21575|DYN	Dynamin-1 OS=Rattus norvegicus GN=Dnm1 PE=1 SV=2	QLELACETQEEVDSWK
>	sp|P21575|DYN	Dynamin-1 OS=Rattus norvegicus GN=Dnm1 PE=1 SV=2	QLELACETQEEVDSWK
>	sp|P21575|DYN	Dynamin-1 OS=Rattus norvegicus GN=Dnm1 PE=1 SV=2	VLNQQLTNHIR
>	sp|P21575|DYN	Dynamin-1 OS=Rattus norvegicus GN=Dnm1 PE=1 SV=2	GMEDLIPLVNR
>	sp|P21575|DYN	Dynamin-1 OS=Rattus norvegicus GN=Dnm1 PE=1 SV=2	FPFELVK
>	sp|P21575|DYN	Dynamin-1 OS=Rattus norvegicus GN=Dnm1 PE=1 SV=2	NLVDSYMAIVNK
>	sp|P21575|DYN	Dynamin-1 OS=Rattus norvegicus GN=Dnm1 PE=1 SV=2	NLVDSYMAIVNK
>	sp|P21575|DYN	Dynamin-1 OS=Rattus norvegicus GN=Dnm1 PE=1 SV=2	KGWLTINNIGIMK
>	sp|P21575|DYN	Dynamin-1 OS=Rattus norvegicus GN=Dnm1 PE=1 SV=2	CVDMVVSELTSTIR
>	sp|P21575|DYN	Dynamin-1 OS=Rattus norvegicus GN=Dnm1 PE=1 SV=2	FTDFEEVRLEIEAETDRVTGTNK
>	sp|P21575|DYN	Dynamin-1 OS=Rattus norvegicus GN=Dnm1 PE=1 SV=2	GPAPGPPPAGSALGGAPPVPSRPGASPDPFGPPPQVPSRPNR
>	sp|P21575|DYN	Dynamin-1 OS=Rattus norvegicus GN=Dnm1 PE=1 SV=2	KGWLTINNIGIMK
>	sp|P21575|DYN	Dynamin-1 OS=Rattus norvegicus GN=Dnm1 PE=1 SV=2	TKEQVMLLIDIELAYMNTNHEDFIGFANAQQR
>	sp|P21575|DYN	Dynamin-1 OS=Rattus norvegicus GN=Dnm1 PE=1 SV=2	NLVDSYMAIVNK
>	sp|P21575|DYN	Dynamin-1 OS=Rattus norvegicus GN=Dnm1 PE=1 SV=2	VLNQQLTNHIRDTLPGLR
>	sp|P21575|DYN	Dynamin-1 OS=Rattus norvegicus GN=Dnm1 PE=1 SV=2	GISPVPINLR
>	sp|P21575|DYN	Dynamin-1 OS=Rattus norvegicus GN=Dnm1 PE=1 SV=2	KGWLTINNIGIMK
>	sp|P21575|DYN	Dynamin-1 OS=Rattus norvegicus GN=Dnm1 PE=1 SV=2	LQSQLLSIEKEVDEYKNFRPDDPAR
>	sp|P21575|DYN	Dynamin-1 OS=Rattus norvegicus GN=Dnm1 PE=1 SV=2	NFRPDDPAR
>	sp|P21575|DYN	Dynamin-1 OS=Rattus norvegicus GN=Dnm1 PE=1 SV=2	TKEQVMLLIDIELAYMNTNHEDFIGFANAQQR
>	sp|P21575|DYN	Dynamin-1 OS=Rattus norvegicus GN=Dnm1 PE=1 SV=2	QLELACETQEEVDSWK
>	sp|P21575|DYN	Dynamin-1 OS=Rattus norvegicus GN=Dnm1 PE=1 SV=2	RPLVLQLVNSTTEYAEFLHCK
>	sp|P21575|DYN	Dynamin-1 OS=Rattus norvegicus GN=Dnm1 PE=1 SV=2	ENCLILAVSPANSDLANSDALK
>	sp|P09951|SYN	Synapsin-1 OS=Rattus norvegicus GN=Syn1 PE=1 SV=3	DGRDHIIEVVGSSMPLIGDHQDEDK
>	sp|P09951|SYN	Synapsin-1 OS=Rattus norvegicus GN=Syn1 PE=1 SV=3	DGRDHIIEVVGSSMPLIGDHQDEDKQLIVELVVNK
>	sp|P09951|SYN	Synapsin-1 OS=Rattus norvegicus GN=Syn1 PE=1 SV=3	DHIIEVVGSSMPLIGDHQDEDKQLIVELVVNK
>	sp|P09951|SYN	Synapsin-1 OS=Rattus norvegicus GN=Syn1 PE=1 SV=3	GSHSQTPSPGALPLGR
>	sp|P09951|SYN	Synapsin-1 OS=Rattus norvegicus GN=Syn1 PE=1 SV=3	IHGEIDIK
>	sp|P09951|SYN	Synapsin-1 OS=Rattus norvegicus GN=Syn1 PE=1 SV=3	KLGTEEFPLIDQTFYPNHK
>	sp|P09951|SYN	Synapsin-1 OS=Rattus norvegicus GN=Syn1 PE=1 SV=3	LWVDTCSEIFGGLDICAVEALHGK
>	sp|P09951|SYN	Synapsin-1 OS=Rattus norvegicus GN=Syn1 PE=1 SV=3	MTQALPR
>	sp|P09951|SYN	Synapsin-1 OS=Rattus norvegicus GN=Syn1 PE=1 SV=3	PSGPGPAGRPTKPQLAQKPSQDVPPPIIAAAGGPPHPQLNK
>	sp|P09951|SYN	Synapsin-1 OS=Rattus norvegicus GN=Syn1 PE=1 SV=3	QGPPQKPPGPAGPIR
>	sp|P09951|SYN	Synapsin-1 OS=Rattus norvegicus GN=Syn1 PE=1 SV=3	QLIVELVVNK
>	sp|P09951|SYN	Synapsin-1 OS=Rattus norvegicus GN=Syn1 PE=1 SV=3	QSRPVAGGPGAPPAARPPASPSPQR
>	sp|P09951|SYN	Synapsin-1 OS=Rattus norvegicus GN=Syn1 PE=1 SV=3	QTSQQPAGPPAQQRPPPQGGPPQPGPGPQR
>	sp|P09951|SYN	Synapsin-1 OS=Rattus norvegicus GN=Syn1 PE=1 SV=3	QTTAAAAATFSEQVGGGSGGAGR
>	sp|P09951|SYN	Synapsin-1 OS=Rattus norvegicus GN=Syn1 PE=1 SV=3	SLVIGLQYAGIPSVNSLHSVYNFCDKPWVFAQMVR

>	sp|P09951|SYN Synapsin-1 OS=Rattus norvegicus GN=Syn1 PE=1 SV=3	TGPPTTQQPRPSGPGPAGRPTKPQLAQKPSQDVPPPIIAAAGGPPHPQLNK
>	sp|P09951|SYN Synapsin-1 OS=Rattus norvegicus GN=Syn1 PE=1 SV=3	TNTGSAMLEQIAMSDR
>	sp|P09951|SYN Synapsin-1 OS=Rattus norvegicus GN=Syn1 PE=1 SV=3	VDNQHDFQDIASVVALTK
>	sp|P09951|SYN Synapsin-1 OS=Rattus norvegicus GN=Syn1 PE=1 SV=3	VKVDNQHDFQDIASVVALTK
>	sp|P09951|SYN Synapsin-1 OS=Rattus norvegicus GN=Syn1 PE=1 SV=3	VLLVIDEPHTDWAK
>	sp|P09951|SYN Synapsin-1 OS=Rattus norvegicus GN=Syn1 PE=1 SV=3	QGPPLQQRPPPQGQQHLSGLGPPAGSPLPQRLPSPTAAPQQSASQATPMTQGQGR
>	sp|P09951|SYN Synapsin-1 OS=Rattus norvegicus GN=Syn1 PE=1 SV=3	DGRDHIIEVVGSSMPLIGDHQDEDK
>	sp|P09951|SYN Synapsin-1 OS=Rattus norvegicus GN=Syn1 PE=1 SV=3	DGRDHIIEVVGSSMPLIGDHQDEDKQLIVELVVNK
>	sp|P09951|SYN Synapsin-1 OS=Rattus norvegicus GN=Syn1 PE=1 SV=3	DGRDHIIEVVGSSMPLIGDHQDEDKQLIVELVVNK
>	sp|P09951|SYN Synapsin-1 OS=Rattus norvegicus GN=Syn1 PE=1 SV=3	DGRDHIIEVVGSSMPLIGDHQDEDKQLIVELVVNK
>	sp|P09951|SYN Synapsin-1 OS=Rattus norvegicus GN=Syn1 PE=1 SV=3	DGRDHIIEVVGSSMPLIGDHQDEDKQLIVELVVNK
>	sp|P09951|SYN Synapsin-1 OS=Rattus norvegicus GN=Syn1 PE=1 SV=3	DGRDHIIEVVGSSMPLIGDHQDEDKQLIVELVVNK
>	sp|P09951|SYN Synapsin-1 OS=Rattus norvegicus GN=Syn1 PE=1 SV=3	DGRDHIIEVVGSSMPLIGDHQDEDKQLIVELVVNK
>	sp|P09951|SYN Synapsin-1 OS=Rattus norvegicus GN=Syn1 PE=1 SV=3	DGRDHIIEVVGSSMPLIGDHQDEDKQLIVELVVNK
>	sp|P09951|SYN Synapsin-1 OS=Rattus norvegicus GN=Syn1 PE=1 SV=3	DGRDHIIEVVGSSMPLIGDHQDEDKQLIVELVVNK
>	sp|P09951|SYN Synapsin-1 OS=Rattus norvegicus GN=Syn1 PE=1 SV=3	DHIIEVVGSSMPLIGDHQDEDKQLIVELVVNK
>	sp|P09951|SYN Synapsin-1 OS=Rattus norvegicus GN=Syn1 PE=1 SV=3	DHIIEVVGSSMPLIGDHQDEDKQLIVELVVNK
>	sp|P09951|SYN Synapsin-1 OS=Rattus norvegicus GN=Syn1 PE=1 SV=3	DHIIEVVGSSMPLIGDHQDEDKQLIVELVVNK
>	sp|P09951|SYN Synapsin-1 OS=Rattus norvegicus GN=Syn1 PE=1 SV=3	IHGEIDIK
>	sp|P09951|SYN Synapsin-1 OS=Rattus norvegicus GN=Syn1 PE=1 SV=3	KLGTEEFPLIDQTFYPNHK
>	sp|P09951|SYN Synapsin-1 OS=Rattus norvegicus GN=Syn1 PE=1 SV=3	KLGTEEFPLIDQTFYPNHK
>	sp|P09951|SYN Synapsin-1 OS=Rattus norvegicus GN=Syn1 PE=1 SV=3	PSGPGPAGRPTKPQLAQKPSQDVPPPIIAAAGGPPHPQLNK
>	sp|P09951|SYN Synapsin-1 OS=Rattus norvegicus GN=Syn1 PE=1 SV=3	PSGPGPAGRPTKPQLAQKPSQDVPPPIIAAAGGPPHPQLNK
>	sp|P09951|SYN Synapsin-1 OS=Rattus norvegicus GN=Syn1 PE=1 SV=3	QGPPQKPPGPAGPIR
>	sp|P09951|SYN Synapsin-1 OS=Rattus norvegicus GN=Syn1 PE=1 SV=3	QGPPQKPPGPAGPIR
>	sp|P09951|SYN Synapsin-1 OS=Rattus norvegicus GN=Syn1 PE=1 SV=3	QLIVELVVNK
>	sp|P09951|SYN Synapsin-1 OS=Rattus norvegicus GN=Syn1 PE=1 SV=3	QLIVELVVNK
>	sp|P09951|SYN Synapsin-1 OS=Rattus norvegicus GN=Syn1 PE=1 SV=3	QLIVELVVNK
>	sp|P09951|SYN Synapsin-1 OS=Rattus norvegicus GN=Syn1 PE=1 SV=3	QSRPVAGGPGAPPAARPPASPSPQR
>	sp|P09951|SYN Synapsin-1 OS=Rattus norvegicus GN=Syn1 PE=1 SV=3	QSRPVAGGPGAPPAARPPASPSPQR
>	sp|P09951|SYN Synapsin-1 OS=Rattus norvegicus GN=Syn1 PE=1 SV=3	QSRPVAGGPGAPPAARPPASPSPQR
>	sp|P09951|SYN Synapsin-1 OS=Rattus norvegicus GN=Syn1 PE=1 SV=3	QSRPVAGGPGAPPAARPPASPSPQR
>	sp|P09951|SYN Synapsin-1 OS=Rattus norvegicus GN=Syn1 PE=1 SV=3	QTSQQPAGPPAQQRPPPQGGPPQPGPGPQR
>	sp|P09951|SYN Synapsin-1 OS=Rattus norvegicus GN=Syn1 PE=1 SV=3	QTTAAAAATFSEQVGGGSGGAGR
>	sp|P09951|SYN Synapsin-1 OS=Rattus norvegicus GN=Syn1 PE=1 SV=3	QTTAAAAATFSEQVGGGSGGAGR
>	sp|P09951|SYN Synapsin-1 OS=Rattus norvegicus GN=Syn1 PE=1 SV=3	SLVIGLQYAGIPSVNSLHSVYNFCDKPWVFAQMVR
>	sp|P09951|SYN Synapsin-1 OS=Rattus norvegicus GN=Syn1 PE=1 SV=3	TNTGSAMLEQIAMSDR
>	sp|P09951|SYN Synapsin-1 OS=Rattus norvegicus GN=Syn1 PE=1 SV=3	TNTGSAMLEQIAMSDR
>	sp|P09951|SYN Synapsin-1 OS=Rattus norvegicus GN=Syn1 PE=1 SV=3	TNTGSAMLEQIAMSDR
>	sp|P09951|SYN Synapsin-1 OS=Rattus norvegicus GN=Syn1 PE=1 SV=3	VDNQHDFQDIASVVALTK
>	sp|P09951|SYN Synapsin-1 OS=Rattus norvegicus GN=Syn1 PE=1 SV=3	VKVDNQHDFQDIASVVALTK
>	sp|P09951|SYN Synapsin-1 OS=Rattus norvegicus GN=Syn1 PE=1 SV=3	VKVDNQHDFQDIASVVALTK
>	sp|P09951|SYN Synapsin-1 OS=Rattus norvegicus GN=Syn1 PE=1 SV=3	VKVDNQHDFQDIASVVALTK
>	sp|P09951|SYN Synapsin-1 OS=Rattus norvegicus GN=Syn1 PE=1 SV=3	VKVDNQHDFQDIASVVALTK
>	sp|P09951|SYN Synapsin-1 OS=Rattus norvegicus GN=Syn1 PE=1 SV=3	VKVDNQHDFQDIASVVALTK
>	sp|P09951|SYN Synapsin-1 OS=Rattus norvegicus GN=Syn1 PE=1 SV=3	VLLVIDEPHTDWAK
>	sp|P09951|SYN Synapsin-1 OS=Rattus norvegicus GN=Syn1 PE=1 SV=3	VLLVIDEPHTDWAK
>	sp|P09951|SYN Synapsin-1 OS=Rattus norvegicus GN=Syn1 PE=1 SV=3	VLLVIDEPHTDWAK
>	sp|P09951|SYN Synapsin-1 OS=Rattus norvegicus GN=Syn1 PE=1 SV=3	QGPPQKPPGPAGPIR
>	sp|P09951|SYN Synapsin-1 OS=Rattus norvegicus GN=Syn1 PE=1 SV=3	VDNQHDFQDIASVVALTK
>	sp|P09951|SYN Synapsin-1 OS=Rattus norvegicus GN=Syn1 PE=1 SV=3	VKVDNQHDFQDIASVVALTK
>	sp|P09951|SYN Synapsin-1 OS=Rattus norvegicus GN=Syn1 PE=1 SV=3	VDNQHDFQDIASVVALTK
>	sp|P09951|SYN Synapsin-1 OS=Rattus norvegicus GN=Syn1 PE=1 SV=3	DGRDHIIEVVGSSMPLIGDHQDEDK
>	sp|P09951|SYN Synapsin-1 OS=Rattus norvegicus GN=Syn1 PE=1 SV=3	DGRDHIIEVVGSSMPLIGDHQDEDK
>	sp|P09951|SYN Synapsin-1 OS=Rattus norvegicus GN=Syn1 PE=1 SV=3	QGPPQKPPGPAGPIR
>	sp|P09951|SYN Synapsin-1 OS=Rattus norvegicus GN=Syn1 PE=1 SV=3	PSGPGPAGRPTKPQLAQKPSQDVPPPIIAAAGGPPHPQLNK
>	sp|P09951|SYN Synapsin-1 OS=Rattus norvegicus GN=Syn1 PE=1 SV=3	DGRDHIIEVVGSSMPLIGDHQDEDK
>	sp|P09951|SYN Synapsin-1 OS=Rattus norvegicus GN=Syn1 PE=1 SV=3	DGRDHIIEVVGSSMPLIGDHQDEDK
>	sp|P09951|SYN Synapsin-1 OS=Rattus norvegicus GN=Syn1 PE=1 SV=3	TNTGSAMLEQIAMSDR
>	sp|P09951|SYN Synapsin-1 OS=Rattus norvegicus GN=Syn1 PE=1 SV=3	QGPPLQQRPPPQGQQHLSGLGPPAGSPLPQRLPSPTAAPQQSASQATPMTQGQGR
>	sp|P09951|SYN Synapsin-1 OS=Rattus norvegicus GN=Syn1 PE=1 SV=3	QLIVELVVNK
>	sp|P09951|SYN Synapsin-1 OS=Rattus norvegicus GN=Syn1 PE=1 SV=3	KLGTEEFPLIDQTFYPNHK
>	sp|P09951|SYN Synapsin-1 OS=Rattus norvegicus GN=Syn1 PE=1 SV=3	VDNQHDFQDIASVVALTK
>	sp|P09951|SYN Synapsin-1 OS=Rattus norvegicus GN=Syn1 PE=1 SV=3	QSRPVAGGPGAPPAARPPASPSPQR
>	sp|P09951|SYN Synapsin-1 OS=Rattus norvegicus GN=Syn1 PE=1 SV=3	EMLSSTTYPVVVK
>	sp|P09951|SYN Synapsin-1 OS=Rattus norvegicus GN=Syn1 PE=1 SV=3	QLIVELVVNK
>	sp|P09951|SYN Synapsin-1 OS=Rattus norvegicus GN=Syn1 PE=1 SV=3	QSRPVAGGPGAPPAARPPASPSPQR
>	sp|P09951|SYN Synapsin-1 OS=Rattus norvegicus GN=Syn1 PE=1 SV=3	VLLVIDEPHTDWAK
>	sp|P09951|SYN Synapsin-1 OS=Rattus norvegicus GN=Syn1 PE=1 SV=3	VKVDNQHDFQDIASVVALTK
>	sp|P09951|SYN Synapsin-1 OS=Rattus norvegicus GN=Syn1 PE=1 SV=3	VDNQHDFQDIASVVALTK
>	sp|P09951|SYN Synapsin-1 OS=Rattus norvegicus GN=Syn1 PE=1 SV=3	LGTEEFPLIDQTFYPNHK
>	sp|P09951|SYN Synapsin-1 OS=Rattus norvegicus GN=Syn1 PE=1 SV=3	LGTEEFPLIDQTFYPNHK
>	sp|P09951|SYN Synapsin-1 OS=Rattus norvegicus GN=Syn1 PE=1 SV=3	VDNQHDFQDIASVVALTK
>	sp|P09951|SYN Synapsin-1 OS=Rattus norvegicus GN=Syn1 PE=1 SV=3	QGPPQKPPGPAGPIR

>	sp|P09951|SYN	Synapsin-1 OS=Rattus norvegicus GN=Syn1 PE=1 SV=3	DHIIEVVGSSMPLIGDHQDEDKQLIVELVVNK
>	sp|P09951|SYN	Synapsin-1 OS=Rattus norvegicus GN=Syn1 PE=1 SV=3	SLVIGLQYAGIPSVNSLHSVYNFCDKPWVFAQMVR
>	sp|P09951|SYN	Synapsin-1 OS=Rattus norvegicus GN=Syn1 PE=1 SV=3	SLVIGLQYAGIPSVNSLHSVYNFCDKPWVFAQMVR
>	sp|P09951|SYN	Synapsin-1 OS=Rattus norvegicus GN=Syn1 PE=1 SV=3	VKVDNQHDFQDIASVVALTK
>	sp|P09951|SYN	Synapsin-1 OS=Rattus norvegicus GN=Syn1 PE=1 SV=3	DGRDHIIEVVGSSMPLIGDHQDEDK
>	sp|P07335|KCF	Creatine kinase B-type OS=Rattus norvegicus GN=Ckb PE=1 SV=2	CTPSGFTLDDAIQTGVDNPGHPYIMTVGAVAGDEESYDVFKDLFDPIIEDR
>	sp|P07335|KCF	Creatine kinase B-type OS=Rattus norvegicus GN=Ckb PE=1 SV=2	DLFDPIIEDR
>	sp|P07335|KCF	Creatine kinase B-type OS=Rattus norvegicus GN=Ckb PE=1 SV=2	FCTGLTQIETLFK
>	sp|P07335|KCF	Creatine kinase B-type OS=Rattus norvegicus GN=Ckb PE=1 SV=2	GIWHNDNK
>	sp|P07335|KCF	Creatine kinase B-type OS=Rattus norvegicus GN=Ckb PE=1 SV=2	GTGGVDTAAVGGVFDVSNADR
>	sp|P07335|KCF	Creatine kinase B-type OS=Rattus norvegicus GN=Ckb PE=1 SV=2	HGGYQPSDEHKTDLNPDNLQGGDDLDPNYVLSSR
>	sp|P07335|KCF	Creatine kinase B-type OS=Rattus norvegicus GN=Ckb PE=1 SV=2	LAVEALSSLDGDLSGR
>	sp|P07335|KCF	Creatine kinase B-type OS=Rattus norvegicus GN=Ckb PE=1 SV=2	LEQGQPIDDLMPAQK
>	sp|P07335|KCF	Creatine kinase B-type OS=Rattus norvegicus GN=Ckb PE=1 SV=2	LGFSEVELVQMVVDGVK
>	sp|P07335|KCF	Creatine kinase B-type OS=Rattus norvegicus GN=Ckb PE=1 SV=2	LGYILTCPSNLGTGLR
>	sp|P07335|KCF	Creatine kinase B-type OS=Rattus norvegicus GN=Ckb PE=1 SV=2	LLIEMEQR
>	sp|P07335|KCF	Creatine kinase B-type OS=Rattus norvegicus GN=Ckb PE=1 SV=2	LRFPAEDEFPDLSSHNNHMAK
>	sp|P07335|KCF	Creatine kinase B-type OS=Rattus norvegicus GN=Ckb PE=1 SV=2	PGHPYIMTVGAVAGDEESYDVFKDLFDPIIEDR
>	sp|P07335|KCF	Creatine kinase B-type OS=Rattus norvegicus GN=Ckb PE=1 SV=2	RGTGGVDTAAVGGVFDVSNADR
>	sp|P07335|KCF	Creatine kinase B-type OS=Rattus norvegicus GN=Ckb PE=1 SV=2	SKNYEFMWNPHLGYILTCPSNLGTGLR
>	sp|P07335|KCF	Creatine kinase B-type OS=Rattus norvegicus GN=Ckb PE=1 SV=2	SMTEAEQQQLIDDHFLFDKPVSPLLLASGMAR
>	sp|P07335|KCF	Creatine kinase B-type OS=Rattus norvegicus GN=Ckb PE=1 SV=2	TFLVWINEEDHLR
>	sp|P07335|KCF	Creatine kinase B-type OS=Rattus norvegicus GN=Ckb PE=1 SV=2	VLTPELYAELR
>	sp|P07335|KCF	Creatine kinase B-type OS=Rattus norvegicus GN=Ckb PE=1 SV=2	AGVHIK
>	sp|P07335|KCF	Creatine kinase B-type OS=Rattus norvegicus GN=Ckb PE=1 SV=2	TDLNPDNLQGGDDLDPNYVLSSR
>	sp|P07335|KCF	Creatine kinase B-type OS=Rattus norvegicus GN=Ckb PE=1 SV=2	NYEFMWNPHLGYILTCPSNLGTGLR
>	sp|P07335|KCF	Creatine kinase B-type OS=Rattus norvegicus GN=Ckb PE=1 SV=2	CTPSGFTLDDAIQTGVDNPGHPYIMTVGAVAGDEESYDVFKDLFDPIIEDR
>	sp|P07335|KCF	Creatine kinase B-type OS=Rattus norvegicus GN=Ckb PE=1 SV=2	CTPSGFTLDDAIQTGVDNPGHPYIMTVGAVAGDEESYDVFKDLFDPIIEDR
>	sp|P07335|KCF	Creatine kinase B-type OS=Rattus norvegicus GN=Ckb PE=1 SV=2	CTPSGFTLDDAIQTGVDNPGHPYIMTVGAVAGDEESYDVFKDLFDPIIEDR
>	sp|P07335|KCF	Creatine kinase B-type OS=Rattus norvegicus GN=Ckb PE=1 SV=2	CTPSGFTLDDAIQTGVDNPGHPYIMTVGAVAGDEESYDVFKDLFDPIIEDR
>	sp|P07335|KCF	Creatine kinase B-type OS=Rattus norvegicus GN=Ckb PE=1 SV=2	FCTGLTQIETLFK
>	sp|P07335|KCF	Creatine kinase B-type OS=Rattus norvegicus GN=Ckb PE=1 SV=2	FCTGLTQIETLFK
>	sp|P07335|KCF	Creatine kinase B-type OS=Rattus norvegicus GN=Ckb PE=1 SV=2	FCTGLTQIETLFK
>	sp|P07335|KCF	Creatine kinase B-type OS=Rattus norvegicus GN=Ckb PE=1 SV=2	FCTGLTQIETLFK
>	sp|P07335|KCF	Creatine kinase B-type OS=Rattus norvegicus GN=Ckb PE=1 SV=2	FCTGLTQIETLFK
>	sp|P07335|KCF	Creatine kinase B-type OS=Rattus norvegicus GN=Ckb PE=1 SV=2	FCTGLTQIETLFK
>	sp|P07335|KCF	Creatine kinase B-type OS=Rattus norvegicus GN=Ckb PE=1 SV=2	GIWHNDNK
>	sp|P07335|KCF	Creatine kinase B-type OS=Rattus norvegicus GN=Ckb PE=1 SV=2	GIWHNDNK
>	sp|P07335|KCF	Creatine kinase B-type OS=Rattus norvegicus GN=Ckb PE=1 SV=2	GIWHNDNK
>	sp|P07335|KCF	Creatine kinase B-type OS=Rattus norvegicus GN=Ckb PE=1 SV=2	GTGGVDTAAVGGVFDVSNADR
>	sp|P07335|KCF	Creatine kinase B-type OS=Rattus norvegicus GN=Ckb PE=1 SV=2	GTGGVDTAAVGGVFDVSNADR
>	sp|P07335|KCF	Creatine kinase B-type OS=Rattus norvegicus GN=Ckb PE=1 SV=2	GTGGVDTAAVGGVFDVSNADR
>	sp|P07335|KCF	Creatine kinase B-type OS=Rattus norvegicus GN=Ckb PE=1 SV=2	HGGYQPSDEHKTDLNPDNLQGGDDLDPNYVLSSR
>	sp|P07335|KCF	Creatine kinase B-type OS=Rattus norvegicus GN=Ckb PE=1 SV=2	LAVEALSSLDGDLSGR
>	sp|P07335|KCF	Creatine kinase B-type OS=Rattus norvegicus GN=Ckb PE=1 SV=2	LAVEALSSLDGDLSGR
>	sp|P07335|KCF	Creatine kinase B-type OS=Rattus norvegicus GN=Ckb PE=1 SV=2	LAVEALSSLDGDLSGR
>	sp|P07335|KCF	Creatine kinase B-type OS=Rattus norvegicus GN=Ckb PE=1 SV=2	LAVEALSSLDGDLSGR
>	sp|P07335|KCF	Creatine kinase B-type OS=Rattus norvegicus GN=Ckb PE=1 SV=2	LAVEALSSLDGDLSGR
>	sp|P07335|KCF	Creatine kinase B-type OS=Rattus norvegicus GN=Ckb PE=1 SV=2	LAVEALSSLDGDLSGR
>	sp|P07335|KCF	Creatine kinase B-type OS=Rattus norvegicus GN=Ckb PE=1 SV=2	LAVEALSSLDGDLSGR
>	sp|P07335|KCF	Creatine kinase B-type OS=Rattus norvegicus GN=Ckb PE=1 SV=2	LAVEALSSLDGDLSGR
>	sp|P07335|KCF	Creatine kinase B-type OS=Rattus norvegicus GN=Ckb PE=1 SV=2	LAVEALSSLDGDLSGR
>	sp|P07335|KCF	Creatine kinase B-type OS=Rattus norvegicus GN=Ckb PE=1 SV=2	LAVEALSSLDGDLSGR
>	sp|P07335|KCF	Creatine kinase B-type OS=Rattus norvegicus GN=Ckb PE=1 SV=2	LAVEALSSLDGDLSGR
>	sp|P07335|KCF	Creatine kinase B-type OS=Rattus norvegicus GN=Ckb PE=1 SV=2	LAVEALSSLDGDLSGR
>	sp|P07335|KCF	Creatine kinase B-type OS=Rattus norvegicus GN=Ckb PE=1 SV=2	LAVEALSSLDGDLSGR
>	sp|P07335|KCF	Creatine kinase B-type OS=Rattus norvegicus GN=Ckb PE=1 SV=2	LAVEALSSLDGDLSGR
>	sp|P07335|KCF	Creatine kinase B-type OS=Rattus norvegicus GN=Ckb PE=1 SV=2	LAVEALSSLDGDLSGR
>	sp|P07335|KCF	Creatine kinase B-type OS=Rattus norvegicus GN=Ckb PE=1 SV=2	LAVEALSSLDGDLSGR
>	sp|P07335|KCF	Creatine kinase B-type OS=Rattus norvegicus GN=Ckb PE=1 SV=2	LAVEALSSLDGDLSGR
>	sp|P07335|KCF	Creatine kinase B-type OS=Rattus norvegicus GN=Ckb PE=1 SV=2	LAVEALSSLDGDLSGR
>	sp|P07335|KCF	Creatine kinase B-type OS=Rattus norvegicus GN=Ckb PE=1 SV=2	LAVEALSSLDGDLSGR
>	sp|P07335|KCF	Creatine kinase B-type OS=Rattus norvegicus GN=Ckb PE=1 SV=2	LEQGQPIDDLMPAQK
>	sp|P07335|KCF	Creatine kinase B-type OS=Rattus norvegicus GN=Ckb PE=1 SV=2	LEQGQPIDDLMPAQK
>	sp|P07335|KCF	Creatine kinase B-type OS=Rattus norvegicus GN=Ckb PE=1 SV=2	LGFSEVELVQMVVDGVK
>	sp|P07335|KCF	Creatine kinase B-type OS=Rattus norvegicus GN=Ckb PE=1 SV=2	LGFSEVELVQMVVDGVK
>	sp|P07335|KCF	Creatine kinase B-type OS=Rattus norvegicus GN=Ckb PE=1 SV=2	LGFSEVELVQMVVDGVK
>	sp|P07335|KCF	Creatine kinase B-type OS=Rattus norvegicus GN=Ckb PE=1 SV=2	LGFSEVELVQMVVDGVK
>	sp|P07335|KCF	Creatine kinase B-type OS=Rattus norvegicus GN=Ckb PE=1 SV=2	LGFSEVELVQMVVDGVK
>	sp|P07335|KCF	Creatine kinase B-type OS=Rattus norvegicus GN=Ckb PE=1 SV=2	LGFSEVELVQMVVDGVK
>	sp|P07335|KCF	Creatine kinase B-type OS=Rattus norvegicus GN=Ckb PE=1 SV=2	LGYILTCPSNLGTGLR
>	sp|P07335|KCF	Creatine kinase B-type OS=Rattus norvegicus GN=Ckb PE=1 SV=2	LGYILTCPSNLGTGLR
>	sp|P07335|KCF	Creatine kinase B-type OS=Rattus norvegicus GN=Ckb PE=1 SV=2	NYEFMWNPHLGYILTCPSNLGTGLR
>	sp|P07335|KCF	Creatine kinase B-type OS=Rattus norvegicus GN=Ckb PE=1 SV=2	NYEFMWNPHLGYILTCPSNLGTGLR

>	sp|P07335|KCF	Creatine kinase B-type OS=Rattus norvegicus GN=Ckb PE=1 SV=2	SKNYEFMWNPHLGYILTCPSNLGTGLR
>	sp|P07335|KCF	Creatine kinase B-type OS=Rattus norvegicus GN=Ckb PE=1 SV=2	SKNYEFMWNPHLGYILTCPSNLGTGLR
>	sp|P07335|KCF	Creatine kinase B-type OS=Rattus norvegicus GN=Ckb PE=1 SV=2	SKNYEFMWNPHLGYILTCPSNLGTGLR
>	sp|P07335|KCF	Creatine kinase B-type OS=Rattus norvegicus GN=Ckb PE=1 SV=2	SMTEAEQQQLIDDHFLFDKPVSPLLLASGMAR
>	sp|P07335|KCF	Creatine kinase B-type OS=Rattus norvegicus GN=Ckb PE=1 SV=2	SMTEAEQQQLIDDHFLFDKPVSPLLLASGMAR
>	sp|P07335|KCF	Creatine kinase B-type OS=Rattus norvegicus GN=Ckb PE=1 SV=2	SMTEAEQQQLIDDHFLFDKPVSPLLLASGMAR
>	sp|P07335|KCF	Creatine kinase B-type OS=Rattus norvegicus GN=Ckb PE=1 SV=2	SMTEAEQQQLIDDHFLFDKPVSPLLLASGMAR
>	sp|P07335|KCF	Creatine kinase B-type OS=Rattus norvegicus GN=Ckb PE=1 SV=2	SMTEAEQQQLIDDHFLFDKPVSPLLLASGMAR
>	sp|P07335|KCF	Creatine kinase B-type OS=Rattus norvegicus GN=Ckb PE=1 SV=2	SMTEAEQQQLIDDHFLFDKPVSPLLLASGMAR
>	sp|P07335|KCF	Creatine kinase B-type OS=Rattus norvegicus GN=Ckb PE=1 SV=2	SMTEAEQQQLIDDHFLFDKPVSPLLLASGMAR
>	sp|P07335|KCF	Creatine kinase B-type OS=Rattus norvegicus GN=Ckb PE=1 SV=2	SMTEAEQQQLIDDHFLFDKPVSPLLLASGMAR
>	sp|P07335|KCF	Creatine kinase B-type OS=Rattus norvegicus GN=Ckb PE=1 SV=2	SMTEAEQQQLIDDHFLFDKPVSPLLLASGMAR
>	sp|P07335|KCF	Creatine kinase B-type OS=Rattus norvegicus GN=Ckb PE=1 SV=2	SMTEAEQQQLIDDHFLFDKPVSPLLLASGMAR
>	sp|P07335|KCF	Creatine kinase B-type OS=Rattus norvegicus GN=Ckb PE=1 SV=2	SMTEAEQQQLIDDHFLFDKPVSPLLLASGMAR
>	sp|P07335|KCF	Creatine kinase B-type OS=Rattus norvegicus GN=Ckb PE=1 SV=2	SMTEAEQQQLIDDHFLFDKPVSPLLLASGMAR
>	sp|P07335|KCF	Creatine kinase B-type OS=Rattus norvegicus GN=Ckb PE=1 SV=2	SMTEAEQQQLIDDHFLFDKPVSPLLLASGMAR
>	sp|P07335|KCF	Creatine kinase B-type OS=Rattus norvegicus GN=Ckb PE=1 SV=2	SMTEAEQQQLIDDHFLFDKPVSPLLLASGMAR
>	sp|P07335|KCF	Creatine kinase B-type OS=Rattus norvegicus GN=Ckb PE=1 SV=2	SMTEAEQQQLIDDHFLFDKPVSPLLLASGMAR
>	sp|P07335|KCF	Creatine kinase B-type OS=Rattus norvegicus GN=Ckb PE=1 SV=2	SMTEAEQQQLIDDHFLFDKPVSPLLLASGMAR
>	sp|P07335|KCF	Creatine kinase B-type OS=Rattus norvegicus GN=Ckb PE=1 SV=2	SMTEAEQQQLIDDHFLFDKPVSPLLLASGMAR
>	sp|P07335|KCF	Creatine kinase B-type OS=Rattus norvegicus GN=Ckb PE=1 SV=2	SMTEAEQQQLIDDHFLFDKPVSPLLLASGMAR
>	sp|P07335|KCF	Creatine kinase B-type OS=Rattus norvegicus GN=Ckb PE=1 SV=2	SMTEAEQQQLIDDHFLFDKPVSPLLLASGMAR
>	sp|P07335|KCF	Creatine kinase B-type OS=Rattus norvegicus GN=Ckb PE=1 SV=2	SMTEAEQQQLIDDHFLFDKPVSPLLLASGMAR
>	sp|P07335|KCF	Creatine kinase B-type OS=Rattus norvegicus GN=Ckb PE=1 SV=2	SMTEAEQQQLIDDHFLFDKPVSPLLLASGMAR
>	sp|P07335|KCF	Creatine kinase B-type OS=Rattus norvegicus GN=Ckb PE=1 SV=2	SMTEAEQQQLIDDHFLFDKPVSPLLLASGMAR
>	sp|P07335|KCF	Creatine kinase B-type OS=Rattus norvegicus GN=Ckb PE=1 SV=2	SMTEAEQQQLIDDHFLFDKPVSPLLLASGMAR
>	sp|P07335|KCF	Creatine kinase B-type OS=Rattus norvegicus GN=Ckb PE=1 SV=2	SMTEAEQQQLIDDHFLFDKPVSPLLLASGMAR
>	sp|P07335|KCF	Creatine kinase B-type OS=Rattus norvegicus GN=Ckb PE=1 SV=2	SMTEAEQQQLIDDHFLFDKPVSPLLLASGMAR
>	sp|P07335|KCF	Creatine kinase B-type OS=Rattus norvegicus GN=Ckb PE=1 SV=2	SMTEAEQQQLIDDHFLFDKPVSPLLLASGMAR
>	sp|P07335|KCF	Creatine kinase B-type OS=Rattus norvegicus GN=Ckb PE=1 SV=2	SMTEAEQQQLIDDHFLFDKPVSPLLLASGMAR
>	sp|P07335|KCF	Creatine kinase B-type OS=Rattus norvegicus GN=Ckb PE=1 SV=2	TFLVWINEEDHLR
>	sp|P07335|KCF	Creatine kinase B-type OS=Rattus norvegicus GN=Ckb PE=1 SV=2	TFLVWINEEDHLR
>	sp|P07335|KCF	Creatine kinase B-type OS=Rattus norvegicus GN=Ckb PE=1 SV=2	TFLVWINEEDHLR
>	sp|P07335|KCF	Creatine kinase B-type OS=Rattus norvegicus GN=Ckb PE=1 SV=2	TFLVWINEEDHLR
>	sp|P07335|KCF	Creatine kinase B-type OS=Rattus norvegicus GN=Ckb PE=1 SV=2	TFLVWINEEDHLR
>	sp|P07335|KCF	Creatine kinase B-type OS=Rattus norvegicus GN=Ckb PE=1 SV=2	TFLVWINEEDHLR
>	sp|P07335|KCF	Creatine kinase B-type OS=Rattus norvegicus GN=Ckb PE=1 SV=2	TFLVWINEEDHLR
>	sp|P07335|KCF	Creatine kinase B-type OS=Rattus norvegicus GN=Ckb PE=1 SV=2	TFLVWINEEDHLR
>	sp|P07335|KCF	Creatine kinase B-type OS=Rattus norvegicus GN=Ckb PE=1 SV=2	TFLVWINEEDHLR
>	sp|P07335|KCF	Creatine kinase B-type OS=Rattus norvegicus GN=Ckb PE=1 SV=2	TFLVWINEEDHLR
>	sp|P07335|KCF	Creatine kinase B-type OS=Rattus norvegicus GN=Ckb PE=1 SV=2	TFLVWINEEDHLR
>	sp|P07335|KCF	Creatine kinase B-type OS=Rattus norvegicus GN=Ckb PE=1 SV=2	TFLVWINEEDHLR
>	sp|P07335|KCF	Creatine kinase B-type OS=Rattus norvegicus GN=Ckb PE=1 SV=2	TFLVWINEEDHLR
>	sp|P07335|KCF	Creatine kinase B-type OS=Rattus norvegicus GN=Ckb PE=1 SV=2	TFLVWINEEDHLR
>	sp|P07335|KCF	Creatine kinase B-type OS=Rattus norvegicus GN=Ckb PE=1 SV=2	TFLVWINEEDHLR
>	sp|P07335|KCF	Creatine kinase B-type OS=Rattus norvegicus GN=Ckb PE=1 SV=2	TFLVWINEEDHLR
>	sp|P07335|KCF	Creatine kinase B-type OS=Rattus norvegicus GN=Ckb PE=1 SV=2	TFLVWINEEDHLR
>	sp|P07335|KCF	Creatine kinase B-type OS=Rattus norvegicus GN=Ckb PE=1 SV=2	TFLVWINEEDHLR
>	sp|P07335|KCF	Creatine kinase B-type OS=Rattus norvegicus GN=Ckb PE=1 SV=2	VLTPELYAELR
>	sp|P07335|KCF	Creatine kinase B-type OS=Rattus norvegicus GN=Ckb PE=1 SV=2	VLTPELYAELR
>	sp|P07335|KCF	Creatine kinase B-type OS=Rattus norvegicus GN=Ckb PE=1 SV=2	VLTPELYAELR
>	sp|P07335|KCF	Creatine kinase B-type OS=Rattus norvegicus GN=Ckb PE=1 SV=2	RGTGGVDTAAVGGVFDVSNADR
>	sp|P07335|KCF	Creatine kinase B-type OS=Rattus norvegicus GN=Ckb PE=1 SV=2	GIWHNDNKTFLVWINEEDHLR
>	sp|P07335|KCF	Creatine kinase B-type OS=Rattus norvegicus GN=Ckb PE=1 SV=2	CTPSGFTLDDAIQTGVDNPGHPYIMTVGAVAGDEESYDVFKDLFDPIIEDR
>	sp|P07335|KCF	Creatine kinase B-type OS=Rattus norvegicus GN=Ckb PE=1 SV=2	LGFSEVELVQMVVDGV
>	sp|P07335|KCF	Creatine kinase B-type OS=Rattus norvegicus GN=Ckb PE=1 SV=2	LGFSEVELVQMVVDGVK
>	sp|P07335|KCF	Creatine kinase B-type OS=Rattus norvegicus GN=Ckb PE=1 SV=2	SMTEAEQQQLIDDHFLFDKPVSPLLLASGMAR
>	sp|P07335|KCF	Creatine kinase B-type OS=Rattus norvegicus GN=Ckb PE=1 SV=2	SMTEAEQQQLIDDHFLFDKPVSPLLLASGMAR
>	sp|P07335|KCF	Creatine kinase B-type OS=Rattus norvegicus GN=Ckb PE=1 SV=2	SMTEAEQQQLIDDHFLFDKPVSPLLLASGMAR
>	sp|P07335|KCF	Creatine kinase B-type OS=Rattus norvegicus GN=Ckb PE=1 SV=2	TFLVWINEEDHLR
>	sp|P07335|KCF	Creatine kinase B-type OS=Rattus norvegicus GN=Ckb PE=1 SV=2	LLIEMEQR
>	sp|P07335|KCF	Creatine kinase B-type OS=Rattus norvegicus GN=Ckb PE=1 SV=2	LRFPAEDEFPDLSSHNNHMAK
>	sp|P07335|KCF	Creatine kinase B-type OS=Rattus norvegicus GN=Ckb PE=1 SV=2	CTPSGFTLDDAIQTGVDNPGHPYIMTVGAVAGDEESYDVFKDLFDPIIEDR
>	sp|P07335|KCF	Creatine kinase B-type OS=Rattus norvegicus GN=Ckb PE=1 SV=2	SMTEAEQQQLIDDHFLFDKPVSPLLLASGMAR
>	sp|P07335|KCF	Creatine kinase B-type OS=Rattus norvegicus GN=Ckb PE=1 SV=2	DLFDPIIEDR
>	sp|P07335|KCF	Creatine kinase B-type OS=Rattus norvegicus GN=Ckb PE=1 SV=2	LLIEMEQR
>	sp|P07335|KCF	Creatine kinase B-type OS=Rattus norvegicus GN=Ckb PE=1 SV=2	CTPSGFTLDDAIQTGVDNPGHPYIMTVGAVAGDEESYDVFK
>	sp|P07335|KCF	Creatine kinase B-type OS=Rattus norvegicus GN=Ckb PE=1 SV=2	SKNYEFMWNPHLGYILTCPSNLGTGLR
>	sp|P07335|KCF	Creatine kinase B-type OS=Rattus norvegicus GN=Ckb PE=1 SV=2	HGGYQPSDEHKTDLNPDNLQGGDDLDPNYVLSSR
>	sp|P07335|KCF	Creatine kinase B-type OS=Rattus norvegicus GN=Ckb PE=1 SV=2	TFLVWINEEDHLR
>	sp|P07335|KCF	Creatine kinase B-type OS=Rattus norvegicus GN=Ckb PE=1 SV=2	SKNYEFMWNPHLGYILTCPSNLGTGLR
>	sp|P07335|KCF	Creatine kinase B-type OS=Rattus norvegicus GN=Ckb PE=1 SV=2	LLIEMEQR
>	sp|P07335|KCF	Creatine kinase B-type OS=Rattus norvegicus GN=Ckb PE=1 SV=2	NYEFMWNPHLGYILTCPSNLGTGLR
>	sp|P07335|KCF	Creatine kinase B-type OS=Rattus norvegicus GN=Ckb PE=1 SV=2	VLTPELYAELR

> sp|P07335|KCF Creatine kinase B-type OS=Rattus norvegicus GN=Ckb PE=1 SV=2 GIWHNDNK
> sp|P07335|KCF Creatine kinase B-type OS=Rattus norvegicus GN=Ckb PE=1 SV=2 SMTEAEQQQLIDDHFLFDKPVSPLLLASGMAR
> sp|P07335|KCF Creatine kinase B-type OS=Rattus norvegicus GN=Ckb PE=1 SV=2 SMTEAEQQQLIDDHFLFDKPVSPLLLASGMAR
> sp|P07335|KCF Creatine kinase B-type OS=Rattus norvegicus GN=Ckb PE=1 SV=2 LRFPAEDEFPDLSSHNNHMAK
> sp|P07335|KCF Creatine kinase B-type OS=Rattus norvegicus GN=Ckb PE=1 SV=2 VLTPELYAELR
> sp|P07335|KCF Creatine kinase B-type OS=Rattus norvegicus GN=Ckb PE=1 SV=2 GIWHNDNK
> sp|P07335|KCF Creatine kinase B-type OS=Rattus norvegicus GN=Ckb PE=1 SV=2 RGTGGVDTAAVGGVFDVSNADR
> sp|P07335|KCF Creatine kinase B-type OS=Rattus norvegicus GN=Ckb PE=1 SV=2 SMTEAEQQQLIDDHFLFDKPVSPLLLASGMAR
> sp|P07335|KCF Creatine kinase B-type OS=Rattus norvegicus GN=Ckb PE=1 SV=2 SMTEAEQQQLIDDHFLFDKPVSPLLLASGMAR
> sp|P07335|KCF Creatine kinase B-type OS=Rattus norvegicus GN=Ckb PE=1 SV=2 SMTEAEQQQLIDDHFLFDKPVSPLLLASGMAR
> sp|P07335|KCF Creatine kinase B-type OS=Rattus norvegicus GN=Ckb PE=1 SV=2 TFLVWINEEDHLR
> sp|P07335|KCF Creatine kinase B-type OS=Rattus norvegicus GN=Ckb PE=1 SV=2 TFLVWINEEDHLR
> sp|P07335|KCF Creatine kinase B-type OS=Rattus norvegicus GN=Ckb PE=1 SV=2 SMTEAEQQQLIDDHFLFDKPVSPLLLASGMAR
> sp|P07335|KCF Creatine kinase B-type OS=Rattus norvegicus GN=Ckb PE=1 SV=2 DWPDAR
> sp|P07335|KCF Creatine kinase B-type OS=Rattus norvegicus GN=Ckb PE=1 SV=2 LPHLGK
> sp|P07335|KCF Creatine kinase B-type OS=Rattus norvegicus GN=Ckb PE=1 SV=2 TFLVWINEEDHLR
> sp|P07335|KCF Creatine kinase B-type OS=Rattus norvegicus GN=Ckb PE=1 SV=2 SMTEAEQQQLIDDHFLFDKPVSPLLLASGMAR
> sp|P07335|KCF Creatine kinase B-type OS=Rattus norvegicus GN=Ckb PE=1 SV=2 TFLVWINEEDHLR
> sp|P07335|KCF Creatine kinase B-type OS=Rattus norvegicus GN=Ckb PE=1 SV=2 RGTGGVDTAAVGGVFDVSNADR
> sp|P07335|KCF Creatine kinase B-type OS=Rattus norvegicus GN=Ckb PE=1 SV=2 SMTEAEQQQLIDDHFLFDKPVSPLLLASGMAR
> sp|P07335|KCF Creatine kinase B-type OS=Rattus norvegicus GN=Ckb PE=1 SV=2 LPHLGK
> sp|P07335|KCF Creatine kinase B-type OS=Rattus norvegicus GN=Ckb PE=1 SV=2 LLIEMEQR
> sp|P07335|KCF Creatine kinase B-type OS=Rattus norvegicus GN=Ckb PE=1 SV=2 VLTPELYAELR
> sp|P07335|KCF Creatine kinase B-type OS=Rattus norvegicus GN=Ckb PE=1 SV=2 LPHLGK
> sp|P07335|KCF Creatine kinase B-type OS=Rattus norvegicus GN=Ckb PE=1 SV=2 NYEFMWNPHLGYILTCPSNLGTGLR
> sp|P07335|KCF Creatine kinase B-type OS=Rattus norvegicus GN=Ckb PE=1 SV=2 LGFSEVELVQMVVDGVK
> sp|P07335|KCF Creatine kinase B-type OS=Rattus norvegicus GN=Ckb PE=1 SV=2 VQMVVDGVK
> sp|P07335|KCF Creatine kinase B-type OS=Rattus norvegicus GN=Ckb PE=1 SV=2 TFLVWINEEDHLR
> sp|P07335|KCF Creatine kinase B-type OS=Rattus norvegicus GN=Ckb PE=1 SV=2 SMTEAEQQQLIDDHFLFDKPVSPLLLA
> sp|P07335|KCF Creatine kinase B-type OS=Rattus norvegicus GN=Ckb PE=1 SV=2 GIWHNDNK
> sp|P07335|KCF Creatine kinase B-type OS=Rattus norvegicus GN=Ckb PE=1 SV=2 AGVHIK
> sp|P07335|KCF Creatine kinase B-type OS=Rattus norvegicus GN=Ckb PE=1 SV=2 SMTEAEQQQLIDDHFLFDKPVSPLLLASGMAR
> sp|P07335|KCF Creatine kinase B-type OS=Rattus norvegicus GN=Ckb PE=1 SV=2 CTPSGFTLDDAIQTGVDNPGHPYIMTVGAVAGDEESYDVFKDLFDPIIEDR
> sp|P07335|KCF Creatine kinase B-type OS=Rattus norvegicus GN=Ckb PE=1 SV=2 CTPSGFTLDDAIQTGVDNPGHPYIMTVGAVAGDEESYDVFKDLFDPIIEDR
> sp|P07335|KCF Creatine kinase B-type OS=Rattus norvegicus GN=Ckb PE=1 SV=2 SMTEAEQQQLIDDHFLFDKPVSPLLLASGMAR
> sp|P07335|KCF Creatine kinase B-type OS=Rattus norvegicus GN=Ckb PE=1 SV=2 SMTEAEQQQLIDDHFLFDKPVSPLLLASGMAR
> sp|P07335|KCF Creatine kinase B-type OS=Rattus norvegicus GN=Ckb PE=1 SV=2 VQMVVDGVK
> sp|P07335|KCF Creatine kinase B-type OS=Rattus norvegicus GN=Ckb PE=1 SV=2 CTPSGFTLDDAIQTGVDNPGHPYIMTVGAVAGDEESYDVFKDLFDPIIEDR
> sp|P07335|KCF Creatine kinase B-type OS=Rattus norvegicus GN=Ckb PE=1 SV=2 CTPSGFTLDDAIQTGVDNPGHPYIMTVGAVAGDEESYDVFKDLFDPIIEDR
> sp|P07335|KCF Creatine kinase B-type OS=Rattus norvegicus GN=Ckb PE=1 SV=2 LAVEALSSLDGDLSGR
> sp|P07335|KCF Creatine kinase B-type OS=Rattus norvegicus GN=Ckb PE=1 SV=2 SMTEAEQQQLIDDHFLFDKPVSPLLLASGMAR
> sp|P07335|KCF Creatine kinase B-type OS=Rattus norvegicus GN=Ckb PE=1 SV=2 SMTEAEQQQLIDDHFLFDKPVSPLLLASGMAR
> sp|P07335|KCF Creatine kinase B-type OS=Rattus norvegicus GN=Ckb PE=1 SV=2 SMTEAEQQQLIDDHFLFDKPVSPLLLASGMAR
> sp|P07335|KCF Creatine kinase B-type OS=Rattus norvegicus GN=Ckb PE=1 SV=2 SMTEAEQQQLIDDHFLFDKPVSPLLLASGMAR
> sp|P07335|KCF Creatine kinase B-type OS=Rattus norvegicus GN=Ckb PE=1 SV=2 TFLVWINEEDHLR
> sp|P07335|KCF Creatine kinase B-type OS=Rattus norvegicus GN=Ckb PE=1 SV=2 TFLVWINEEDHLR
> sp|P07335|KCF Creatine kinase B-type OS=Rattus norvegicus GN=Ckb PE=1 SV=2 VISMQK
> sp|P07335|KCF Creatine kinase B-type OS=Rattus norvegicus GN=Ckb PE=1 SV=2 LEQGQPIDDLMPAQK
> sp|P10719|ATF ATP synthase subunit beta, mitochondrial OS=Rattus norvegicus GN=Atp5b PE=1 SV=2 AHGGYSVFAGVGER
> sp|P10719|ATF ATP synthase subunit beta, mitochondrial OS=Rattus norvegicus GN=Atp5b PE=1 SV=2 AIAELGIYPAVDPLDSTSR
> sp|P10719|ATF ATP synthase subunit beta, mitochondrial OS=Rattus norvegicus GN=Atp5b PE=1 SV=2 FLSQPFQVAEVFTGHMGK
> sp|P10719|ATF ATP synthase subunit beta, mitochondrial OS=Rattus norvegicus GN=Atp5b PE=1 SV=2 FTQAGSEVSALLGR
> sp|P10719|ATF ATP synthase subunit beta, mitochondrial OS=Rattus norvegicus GN=Atp5b PE=1 SV=2 IGLFGGAGVGK
> sp|P10719|ATF ATP synthase subunit beta, mitochondrial OS=Rattus norvegicus GN=Atp5b PE=1 SV=2 IGLFGGAGVGKTVLIMELINNVAK
> sp|P10719|ATF ATP synthase subunit beta, mitochondrial OS=Rattus norvegicus GN=Atp5b PE=1 SV=2 IGLFGGAGVGKTVLIMELINNVAKAHGGYSVFAGVGER
> sp|P10719|ATF ATP synthase subunit beta, mitochondrial OS=Rattus norvegicus GN=Atp5b PE=1 SV=2 IMDPNIVGSEHYDVAR
> sp|P10719|ATF ATP synthase subunit beta, mitochondrial OS=Rattus norvegicus GN=Atp5b PE=1 SV=2 IMNVIGEPIDER
> sp|P10719|ATF ATP synthase subunit beta, mitochondrial OS=Rattus norvegicus GN=Atp5b PE=1 SV=2 KGSITSVQAIYVPADDLTDPAPATTFAHLDATTVLSR
> sp|P10719|ATF ATP synthase subunit beta, mitochondrial OS=Rattus norvegicus GN=Atp5b PE=1 SV=2 LVLEVAQHLGESTVR
> sp|P10719|ATF ATP synthase subunit beta, mitochondrial OS=Rattus norvegicus GN=Atp5b PE=1 SV=2 QFAPIHAEAPEFIEMSVEQEILVTGIK
> sp|P10719|ATF ATP synthase subunit beta, mitochondrial OS=Rattus norvegicus GN=Atp5b PE=1 SV=2 TIAMDGTEGLVR
> sp|P10719|ATF ATP synthase subunit beta, mitochondrial OS=Rattus norvegicus GN=Atp5b PE=1 SV=2 TREGNDLYHEMIESGVINLK
> sp|P10719|ATF ATP synthase subunit beta, mitochondrial OS=Rattus norvegicus GN=Atp5b PE=1 SV=2 TVLIMELINNVAK
> sp|P10719|ATF ATP synthase subunit beta, mitochondrial OS=Rattus norvegicus GN=Atp5b PE=1 SV=2 VALVYGQMNEPPGAR
> sp|P10719|ATF ATP synthase subunit beta, mitochondrial OS=Rattus norvegicus GN=Atp5b PE=1 SV=2 VLDSGAPIKIPVGPETLGR
> sp|P10719|ATF ATP synthase subunit beta, mitochondrial OS=Rattus norvegicus GN=Atp5b PE=1 SV=2 AAQSSAAPK
> sp|P10719|ATF ATP synthase subunit beta, mitochondrial OS=Rattus norvegicus GN=Atp5b PE=1 SV=2 VVDLLAPYAK
> sp|P10719|ATF ATP synthase subunit beta, mitochondrial OS=Rattus norvegicus GN=Atp5b PE=1 SV=2 GGKIGLFGGAGVGKTVLIMELINNVAKAHGGYSVFAGVGER
> sp|P10719|ATF ATP synthase subunit beta, mitochondrial OS=Rattus norvegicus GN=Atp5b PE=1 SV=2 PFQVAEVFTGHMGK
> sp|P10719|ATF ATP synthase subunit beta, mitochondrial OS=Rattus norvegicus GN=Atp5b PE=1 SV=2 SLQDIIAILGMDELSEEDKLTVSR
> sp|P10719|ATF ATP synthase subunit beta, mitochondrial OS=Rattus norvegicus GN=Atp5b PE=1 SV=2 GPIEEAVAK
> sp|P10719|ATF ATP synthase subunit beta, mitochondrial OS=Rattus norvegicus GN=Atp5b PE=1 SV=2 EGNDLYHEMIESGVINLK
> sp|P10719|ATF ATP synthase subunit beta, mitochondrial OS=Rattus norvegicus GN=Atp5b PE=1 SV=2 GFQQILAGDYDHLPEQAFYMVGPIEEAVAK
> sp|P10719|ATF ATP synthase subunit beta, mitochondrial OS=Rattus norvegicus GN=Atp5b PE=1 SV=2 AHGGYSVFAGVGER

>	sp|P10719|ATF ATP synthase subunit beta, mitochondrial OS=Rattus norvegicus GN=Atp5b PE=1 SV=2	AHGGYSVFAGVGER
>	sp|P10719|ATF ATP synthase subunit beta, mitochondrial OS=Rattus norvegicus GN=Atp5b PE=1 SV=2	AHGGYSVFAGVGER
>	sp|P10719|ATF ATP synthase subunit beta, mitochondrial OS=Rattus norvegicus GN=Atp5b PE=1 SV=2	AHGGYSVFAGVGER
>	sp|P10719|ATF ATP synthase subunit beta, mitochondrial OS=Rattus norvegicus GN=Atp5b PE=1 SV=2	AIAELGIYPAVDPLDSTSR
>	sp|P10719|ATF ATP synthase subunit beta, mitochondrial OS=Rattus norvegicus GN=Atp5b PE=1 SV=2	AIAELGIYPAVDPLDSTSR
>	sp|P10719|ATF ATP synthase subunit beta, mitochondrial OS=Rattus norvegicus GN=Atp5b PE=1 SV=2	AIAELGIYPAVDPLDSTSR
>	sp|P10719|ATF ATP synthase subunit beta, mitochondrial OS=Rattus norvegicus GN=Atp5b PE=1 SV=2	FLSQPFQVAEVFTGHMGK
>	sp|P10719|ATF ATP synthase subunit beta, mitochondrial OS=Rattus norvegicus GN=Atp5b PE=1 SV=2	FLSQPFQVAEVFTGHMGK
>	sp|P10719|ATF ATP synthase subunit beta, mitochondrial OS=Rattus norvegicus GN=Atp5b PE=1 SV=2	FLSQPFQVAEVFTGHMGK
>	sp|P10719|ATF ATP synthase subunit beta, mitochondrial OS=Rattus norvegicus GN=Atp5b PE=1 SV=2	FLSQPFQVAEVFTGHMGK
>	sp|P10719|ATF ATP synthase subunit beta, mitochondrial OS=Rattus norvegicus GN=Atp5b PE=1 SV=2	FLSQPFQVAEVFTGHMGK
>	sp|P10719|ATF ATP synthase subunit beta, mitochondrial OS=Rattus norvegicus GN=Atp5b PE=1 SV=2	FLSQPFQVAEVFTGHMGK
>	sp|P10719|ATF ATP synthase subunit beta, mitochondrial OS=Rattus norvegicus GN=Atp5b PE=1 SV=2	FLSQPFQVAEVFTGHMGK
>	sp|P10719|ATF ATP synthase subunit beta, mitochondrial OS=Rattus norvegicus GN=Atp5b PE=1 SV=2	FLSQPFQVAEVFTGHMGK
>	sp|P10719|ATF ATP synthase subunit beta, mitochondrial OS=Rattus norvegicus GN=Atp5b PE=1 SV=2	FLSQPFQVAEVFTGHMGK
>	sp|P10719|ATF ATP synthase subunit beta, mitochondrial OS=Rattus norvegicus GN=Atp5b PE=1 SV=2	FLSQPFQVAEVFTGHMGK
>	sp|P10719|ATF ATP synthase subunit beta, mitochondrial OS=Rattus norvegicus GN=Atp5b PE=1 SV=2	FLSQPFQVAEVFTGHMGK
>	sp|P10719|ATF ATP synthase subunit beta, mitochondrial OS=Rattus norvegicus GN=Atp5b PE=1 SV=2	FLSQPFQVAEVFTGHMGK
>	sp|P10719|ATF ATP synthase subunit beta, mitochondrial OS=Rattus norvegicus GN=Atp5b PE=1 SV=2	FLSQPFQVAEVFTGHMGK
>	sp|P10719|ATF ATP synthase subunit beta, mitochondrial OS=Rattus norvegicus GN=Atp5b PE=1 SV=2	FLSQPFQVAEVFTGHMGK
>	sp|P10719|ATF ATP synthase subunit beta, mitochondrial OS=Rattus norvegicus GN=Atp5b PE=1 SV=2	FLSQPFQVAEVFTGHMGK
>	sp|P10719|ATF ATP synthase subunit beta, mitochondrial OS=Rattus norvegicus GN=Atp5b PE=1 SV=2	FLSQPFQVAEVFTGHMGK
>	sp|P10719|ATF ATP synthase subunit beta, mitochondrial OS=Rattus norvegicus GN=Atp5b PE=1 SV=2	FLSQPFQVAEVFTGHMGK
>	sp|P10719|ATF ATP synthase subunit beta, mitochondrial OS=Rattus norvegicus GN=Atp5b PE=1 SV=2	FLSQPFQVAEVFTGHMGK
>	sp|P10719|ATF ATP synthase subunit beta, mitochondrial OS=Rattus norvegicus GN=Atp5b PE=1 SV=2	FLSQPFQVAEVFTGHMGK
>	sp|P10719|ATF ATP synthase subunit beta, mitochondrial OS=Rattus norvegicus GN=Atp5b PE=1 SV=2	FLSQPFQVAEVFTGHMGK
>	sp|P10719|ATF ATP synthase subunit beta, mitochondrial OS=Rattus norvegicus GN=Atp5b PE=1 SV=2	FLSQPFQVAEVFTGHMGK
>	sp|P10719|ATF ATP synthase subunit beta, mitochondrial OS=Rattus norvegicus GN=Atp5b PE=1 SV=2	FLSQPFQVAEVFTGHMGK
>	sp|P10719|ATF ATP synthase subunit beta, mitochondrial OS=Rattus norvegicus GN=Atp5b PE=1 SV=2	FLSQPFQVAEVFTGHMGK
>	sp|P10719|ATF ATP synthase subunit beta, mitochondrial OS=Rattus norvegicus GN=Atp5b PE=1 SV=2	FLSQPFQVAEVFTGHMGK
>	sp|P10719|ATF ATP synthase subunit beta, mitochondrial OS=Rattus norvegicus GN=Atp5b PE=1 SV=2	FLSQPFQVAEVFTGHMGK
>	sp|P10719|ATF ATP synthase subunit beta, mitochondrial OS=Rattus norvegicus GN=Atp5b PE=1 SV=2	FLSQPFQVAEVFTGHMGK
>	sp|P10719|ATF ATP synthase subunit beta, mitochondrial OS=Rattus norvegicus GN=Atp5b PE=1 SV=2	FLSQPFQVAEVFTGHMGK
>	sp|P10719|ATF ATP synthase subunit beta, mitochondrial OS=Rattus norvegicus GN=Atp5b PE=1 SV=2	FTQAGSEVSALLGR
>	sp|P10719|ATF ATP synthase subunit beta, mitochondrial OS=Rattus norvegicus GN=Atp5b PE=1 SV=2	GFQQILAGDYDHLPEQAFYMVGPIEEAVAK
>	sp|P10719|ATF ATP synthase subunit beta, mitochondrial OS=Rattus norvegicus GN=Atp5b PE=1 SV=2	GFQQILAGDYDHLPEQAFYMVGPIEEAVAK
>	sp|P10719|ATF ATP synthase subunit beta, mitochondrial OS=Rattus norvegicus GN=Atp5b PE=1 SV=2	IGLFGGAGVGK
>	sp|P10719|ATF ATP synthase subunit beta, mitochondrial OS=Rattus norvegicus GN=Atp5b PE=1 SV=2	IGLFGGAGVGK
>	sp|P10719|ATF ATP synthase subunit beta, mitochondrial OS=Rattus norvegicus GN=Atp5b PE=1 SV=2	IGLFGGAGVGK
>	sp|P10719|ATF ATP synthase subunit beta, mitochondrial OS=Rattus norvegicus GN=Atp5b PE=1 SV=2	IGLFGGAGVGK
>	sp|P10719|ATF ATP synthase subunit beta, mitochondrial OS=Rattus norvegicus GN=Atp5b PE=1 SV=2	IGLFGGAGVGK
>	sp|P10719|ATF ATP synthase subunit beta, mitochondrial OS=Rattus norvegicus GN=Atp5b PE=1 SV=2	IGLFGGAGVGK
>	sp|P10719|ATF ATP synthase subunit beta, mitochondrial OS=Rattus norvegicus GN=Atp5b PE=1 SV=2	IGLFGGAGVGK
>	sp|P10719|ATF ATP synthase subunit beta, mitochondrial OS=Rattus norvegicus GN=Atp5b PE=1 SV=2	IGLFGGAGVGKTVLIMELINNVAKAHGGYSVFAGVGER
>	sp|P10719|ATF ATP synthase subunit beta, mitochondrial OS=Rattus norvegicus GN=Atp5b PE=1 SV=2	IGLFGGAGVGKTVLIMELINNVAKAHGGYSVFAGVGER
>	sp|P10719|ATF ATP synthase subunit beta, mitochondrial OS=Rattus norvegicus GN=Atp5b PE=1 SV=2	IGLFGGAGVGKTVLIMELINNVAKAHGGYSVFAGVGER
>	sp|P10719|ATF ATP synthase subunit beta, mitochondrial OS=Rattus norvegicus GN=Atp5b PE=1 SV=2	IMDPNIVGSEHYDVAR
>	sp|P10719|ATF ATP synthase subunit beta, mitochondrial OS=Rattus norvegicus GN=Atp5b PE=1 SV=2	IMNVIGEPIDER
>	sp|P10719|ATF ATP synthase subunit beta, mitochondrial OS=Rattus norvegicus GN=Atp5b PE=1 SV=2	KGSITSVQAIYVPADDLTDPAPATTFAHLDATTVLSR
>	sp|P10719|ATF ATP synthase subunit beta, mitochondrial OS=Rattus norvegicus GN=Atp5b PE=1 SV=2	KGSITSVQAIYVPADDLTDPAPATTFAHLDATTVLSR
>	sp|P10719|ATF ATP synthase subunit beta, mitochondrial OS=Rattus norvegicus GN=Atp5b PE=1 SV=2	KGSITSVQAIYVPADDLTDPAPATTFAHLDATTVLSR
>	sp|P10719|ATF ATP synthase subunit beta, mitochondrial OS=Rattus norvegicus GN=Atp5b PE=1 SV=2	KGSITSVQAIYVPADDLTDPAPATTFAHLDATTVLSR
>	sp|P10719|ATF ATP synthase subunit beta, mitochondrial OS=Rattus norvegicus GN=Atp5b PE=1 SV=2	KGSITSVQAIYVPADDLTDPAPATTFAHLDATTVLSR
>	sp|P10719|ATF ATP synthase subunit beta, mitochondrial OS=Rattus norvegicus GN=Atp5b PE=1 SV=2	KGSITSVQAIYVPADDLTDPAPATTFAHLDATTVLSR
>	sp|P10719|ATF ATP synthase subunit beta, mitochondrial OS=Rattus norvegicus GN=Atp5b PE=1 SV=2	KGSITSVQAIYVPADDLTDPAPATTFAHLDATTVLSR
>	sp|P10719|ATF ATP synthase subunit beta, mitochondrial OS=Rattus norvegicus GN=Atp5b PE=1 SV=2	KGSITSVQAIYVPADDLTDPAPATTFAHLDATTVLSR
>	sp|P10719|ATF ATP synthase subunit beta, mitochondrial OS=Rattus norvegicus GN=Atp5b PE=1 SV=2	KGSITSVQAIYVPADDLTDPAPATTFAHLDATTVLSR
>	sp|P10719|ATF ATP synthase subunit beta, mitochondrial OS=Rattus norvegicus GN=Atp5b PE=1 SV=2	KGSITSVQAIYVPADDLTDPAPATTFAHLDATTVLSR
>	sp|P10719|ATF ATP synthase subunit beta, mitochondrial OS=Rattus norvegicus GN=Atp5b PE=1 SV=2	KGSITSVQAIYVPADDLTDPAPATTFAHLDATTVLSR
>	sp|P10719|ATF ATP synthase subunit beta, mitochondrial OS=Rattus norvegicus GN=Atp5b PE=1 SV=2	KGSITSVQAIYVPADDLTDPAPATTFAHLDATTVLSR
>	sp|P10719|ATF ATP synthase subunit beta, mitochondrial OS=Rattus norvegicus GN=Atp5b PE=1 SV=2	LVLEVAQHLGESTVR
>	sp|P10719|ATF ATP synthase subunit beta, mitochondrial OS=Rattus norvegicus GN=Atp5b PE=1 SV=2	LVLEVAQHLGESTVR
>	sp|P10719|ATF ATP synthase subunit beta, mitochondrial OS=Rattus norvegicus GN=Atp5b PE=1 SV=2	LVLEVAQHLGESTVR
>	sp|P10719|ATF ATP synthase subunit beta, mitochondrial OS=Rattus norvegicus GN=Atp5b PE=1 SV=2	LVLEVAQHLGESTVR
>	sp|P10719|ATF ATP synthase subunit beta, mitochondrial OS=Rattus norvegicus GN=Atp5b PE=1 SV=2	LVLEVAQHLGESTVR
>	sp|P10719|ATF ATP synthase subunit beta, mitochondrial OS=Rattus norvegicus GN=Atp5b PE=1 SV=2	LVLEVAQHLGESTVR
>	sp|P10719|ATF ATP synthase subunit beta, mitochondrial OS=Rattus norvegicus GN=Atp5b PE=1 SV=2	LVLEVAQHLGESTVR
>	sp|P10719|ATF ATP synthase subunit beta, mitochondrial OS=Rattus norvegicus GN=Atp5b PE=1 SV=2	LVLEVAQHLGESTVR
>	sp|P10719|ATF ATP synthase subunit beta, mitochondrial OS=Rattus norvegicus GN=Atp5b PE=1 SV=2	LVLEVAQHLGESTVR
>	sp|P10719|ATF ATP synthase subunit beta, mitochondrial OS=Rattus norvegicus GN=Atp5b PE=1 SV=2	LVLEVAQHLGESTVR
>	sp|P10719|ATF ATP synthase subunit beta, mitochondrial OS=Rattus norvegicus GN=Atp5b PE=1 SV=2	LVLEVAQHLGESTVR
>	sp|P10719|ATF ATP synthase subunit beta, mitochondrial OS=Rattus norvegicus GN=Atp5b PE=1 SV=2	LVLEVAQHLGESTVR
>	sp|P10719|ATF ATP synthase subunit beta, mitochondrial OS=Rattus norvegicus GN=Atp5b PE=1 SV=2	LVLEVAQHLGESTVR
>	sp|P10719|ATF ATP synthase subunit beta, mitochondrial OS=Rattus norvegicus GN=Atp5b PE=1 SV=2	LVLEVAQHLGESTVR

>	sp|P10719|ATF ATP synthase subunit beta, mitochondrial OS=Rattus norvegicus GN=Atp5b PE=1 SV=2	LVLEVAQHLGESTVR
>	sp|P10719|ATF ATP synthase subunit beta, mitochondrial OS=Rattus norvegicus GN=Atp5b PE=1 SV=2	LVLEVAQHLGESTVR
>	sp|P10719|ATF ATP synthase subunit beta, mitochondrial OS=Rattus norvegicus GN=Atp5b PE=1 SV=2	LVLEVAQHLGESTVR
>	sp|P10719|ATF ATP synthase subunit beta, mitochondrial OS=Rattus norvegicus GN=Atp5b PE=1 SV=2	LVLEVAQHLGESTVR
>	sp|P10719|ATF ATP synthase subunit beta, mitochondrial OS=Rattus norvegicus GN=Atp5b PE=1 SV=2	LVLEVAQHLGESTVR
>	sp|P10719|ATF ATP synthase subunit beta, mitochondrial OS=Rattus norvegicus GN=Atp5b PE=1 SV=2	LVLEVAQHLGESTVR
>	sp|P10719|ATF ATP synthase subunit beta, mitochondrial OS=Rattus norvegicus GN=Atp5b PE=1 SV=2	LVLEVAQHLGESTVR
>	sp|P10719|ATF ATP synthase subunit beta, mitochondrial OS=Rattus norvegicus GN=Atp5b PE=1 SV=2	LVLEVAQHLGESTVR
>	sp|P10719|ATF ATP synthase subunit beta, mitochondrial OS=Rattus norvegicus GN=Atp5b PE=1 SV=2	LVLEVAQHLGESTVR
>	sp|P10719|ATF ATP synthase subunit beta, mitochondrial OS=Rattus norvegicus GN=Atp5b PE=1 SV=2	LVLEVAQHLGESTVR
>	sp|P10719|ATF ATP synthase subunit beta, mitochondrial OS=Rattus norvegicus GN=Atp5b PE=1 SV=2	LVLEVAQHLGESTVR
>	sp|P10719|ATF ATP synthase subunit beta, mitochondrial OS=Rattus norvegicus GN=Atp5b PE=1 SV=2	LVLEVAQHLGESTVR
>	sp|P10719|ATF ATP synthase subunit beta, mitochondrial OS=Rattus norvegicus GN=Atp5b PE=1 SV=2	PFQVAEVFTGHMGK
>	sp|P10719|ATF ATP synthase subunit beta, mitochondrial OS=Rattus norvegicus GN=Atp5b PE=1 SV=2	PFQVAEVFTGHMGK
>	sp|P10719|ATF ATP synthase subunit beta, mitochondrial OS=Rattus norvegicus GN=Atp5b PE=1 SV=2	QFAPIHAEAPEFIEMSVEQEILVTGIK
>	sp|P10719|ATF ATP synthase subunit beta, mitochondrial OS=Rattus norvegicus GN=Atp5b PE=1 SV=2	QFAPIHAEAPEFIEMSVEQEILVTGIK
>	sp|P10719|ATF ATP synthase subunit beta, mitochondrial OS=Rattus norvegicus GN=Atp5b PE=1 SV=2	QFAPIHAEAPEFIEMSVEQEILVTGIK
>	sp|P10719|ATF ATP synthase subunit beta, mitochondrial OS=Rattus norvegicus GN=Atp5b PE=1 SV=2	QFAPIHAEAPEFIEMSVEQEILVTGIK
>	sp|P10719|ATF ATP synthase subunit beta, mitochondrial OS=Rattus norvegicus GN=Atp5b PE=1 SV=2	QFAPIHAEAPEFIEMSVEQEILVTGIK
>	sp|P10719|ATF ATP synthase subunit beta, mitochondrial OS=Rattus norvegicus GN=Atp5b PE=1 SV=2	QFAPIHAEAPEFIEMSVEQEILVTGIK
>	sp|P10719|ATF ATP synthase subunit beta, mitochondrial OS=Rattus norvegicus GN=Atp5b PE=1 SV=2	QFAPIHAEAPEFIEMSVEQEILVTGIK
>	sp|P10719|ATF ATP synthase subunit beta, mitochondrial OS=Rattus norvegicus GN=Atp5b PE=1 SV=2	QFAPIHAEAPEFIEMSVEQEILVTGIK
>	sp|P10719|ATF ATP synthase subunit beta, mitochondrial OS=Rattus norvegicus GN=Atp5b PE=1 SV=2	QFAPIHAEAPEFIEMSVEQEILVTGIK
>	sp|P10719|ATF ATP synthase subunit beta, mitochondrial OS=Rattus norvegicus GN=Atp5b PE=1 SV=2	QFAPIHAEAPEFIEMSVEQEILVTGIK
>	sp|P10719|ATF ATP synthase subunit beta, mitochondrial OS=Rattus norvegicus GN=Atp5b PE=1 SV=2	QFAPIHAEAPEFIEMSVEQEILVTGIK
>	sp|P10719|ATF ATP synthase subunit beta, mitochondrial OS=Rattus norvegicus GN=Atp5b PE=1 SV=2	QFAPIHAEAPEFIEMSVEQEILVTGIK
>	sp|P10719|ATF ATP synthase subunit beta, mitochondrial OS=Rattus norvegicus GN=Atp5b PE=1 SV=2	QFAPIHAEAPEFIEMSVEQEILVTGIK
>	sp|P10719|ATF ATP synthase subunit beta, mitochondrial OS=Rattus norvegicus GN=Atp5b PE=1 SV=2	QFAPIHAEAPEFIEMSVEQEILVTGIK
>	sp|P10719|ATF ATP synthase subunit beta, mitochondrial OS=Rattus norvegicus GN=Atp5b PE=1 SV=2	QFAPIHAEAPEFIEMSVEQEILVTGIK
>	sp|P10719|ATF ATP synthase subunit beta, mitochondrial OS=Rattus norvegicus GN=Atp5b PE=1 SV=2	SLQDIIAILGMDELSEEDKLTVSR
>	sp|P10719|ATF ATP synthase subunit beta, mitochondrial OS=Rattus norvegicus GN=Atp5b PE=1 SV=2	TIAMDGTEGLVR
>	sp|P10719|ATF ATP synthase subunit beta, mitochondrial OS=Rattus norvegicus GN=Atp5b PE=1 SV=2	TREGNDLYHEMIESGVINLK
>	sp|P10719|ATF ATP synthase subunit beta, mitochondrial OS=Rattus norvegicus GN=Atp5b PE=1 SV=2	TVLIMELINNVAK
>	sp|P10719|ATF ATP synthase subunit beta, mitochondrial OS=Rattus norvegicus GN=Atp5b PE=1 SV=2	TVLIMELINNVAK
>	sp|P10719|ATF ATP synthase subunit beta, mitochondrial OS=Rattus norvegicus GN=Atp5b PE=1 SV=2	TVLIMELINNVAK
>	sp|P10719|ATF ATP synthase subunit beta, mitochondrial OS=Rattus norvegicus GN=Atp5b PE=1 SV=2	TVLIMELINNVAK
>	sp|P10719|ATF ATP synthase subunit beta, mitochondrial OS=Rattus norvegicus GN=Atp5b PE=1 SV=2	TVLIMELINNVAK
>	sp|P10719|ATF ATP synthase subunit beta, mitochondrial OS=Rattus norvegicus GN=Atp5b PE=1 SV=2	TVLIMELINNVAK
>	sp|P10719|ATF ATP synthase subunit beta, mitochondrial OS=Rattus norvegicus GN=Atp5b PE=1 SV=2	VALVYGQMNEPPGAR
>	sp|P10719|ATF ATP synthase subunit beta, mitochondrial OS=Rattus norvegicus GN=Atp5b PE=1 SV=2	VALVYGQMNEPPGAR
>	sp|P10719|ATF ATP synthase subunit beta, mitochondrial OS=Rattus norvegicus GN=Atp5b PE=1 SV=2	VALVYGQMNEPPGAR
>	sp|P10719|ATF ATP synthase subunit beta, mitochondrial OS=Rattus norvegicus GN=Atp5b PE=1 SV=2	VLDSGAPIKIPVGPETLGR
>	sp|P10719|ATF ATP synthase subunit beta, mitochondrial OS=Rattus norvegicus GN=Atp5b PE=1 SV=2	VLDSGAPIKIPVGPETLGR
>	sp|P10719|ATF ATP synthase subunit beta, mitochondrial OS=Rattus norvegicus GN=Atp5b PE=1 SV=2	VLDSGAPIKIPVGPETLGR
>	sp|P10719|ATF ATP synthase subunit beta, mitochondrial OS=Rattus norvegicus GN=Atp5b PE=1 SV=2	VLDSGAPIKIPVGPETLGR
>	sp|P10719|ATF ATP synthase subunit beta, mitochondrial OS=Rattus norvegicus GN=Atp5b PE=1 SV=2	VLDSGAPIKIPVGPETLGR
>	sp|P10719|ATF ATP synthase subunit beta, mitochondrial OS=Rattus norvegicus GN=Atp5b PE=1 SV=2	VLDSGAPIKIPVGPETLGR
>	sp|P10719|ATF ATP synthase subunit beta, mitochondrial OS=Rattus norvegicus GN=Atp5b PE=1 SV=2	VLDSGAPIKIPVGPETLGR
>	sp|P10719|ATF ATP synthase subunit beta, mitochondrial OS=Rattus norvegicus GN=Atp5b PE=1 SV=2	VLDSGAPIKIPVGPETLGR
>	sp|P10719|ATF ATP synthase subunit beta, mitochondrial OS=Rattus norvegicus GN=Atp5b PE=1 SV=2	VLDSGAPIKIPVGPETLGR
>	sp|P10719|ATF ATP synthase subunit beta, mitochondrial OS=Rattus norvegicus GN=Atp5b PE=1 SV=2	VLDSGAPIKIPVGPETLGR
>	sp|P10719|ATF ATP synthase subunit beta, mitochondrial OS=Rattus norvegicus GN=Atp5b PE=1 SV=2	VLDSGAPIKIPVGPETLGR
>	sp|P10719|ATF ATP synthase subunit beta, mitochondrial OS=Rattus norvegicus GN=Atp5b PE=1 SV=2	VLDSGAPIKIPVGPETLGR
>	sp|P10719|ATF ATP synthase subunit beta, mitochondrial OS=Rattus norvegicus GN=Atp5b PE=1 SV=2	VLDSGAPIKIPVGPETLGR
>	sp|P10719|ATF ATP synthase subunit beta, mitochondrial OS=Rattus norvegicus GN=Atp5b PE=1 SV=2	VLDSGAPIKIPVGPETLGR
>	sp|P10719|ATF ATP synthase subunit beta, mitochondrial OS=Rattus norvegicus GN=Atp5b PE=1 SV=2	VVDLLAPYAK
>	sp|P10719|ATF ATP synthase subunit beta, mitochondrial OS=Rattus norvegicus GN=Atp5b PE=1 SV=2	VVDLLAPYAK
>	sp|P10719|ATF ATP synthase subunit beta, mitochondrial OS=Rattus norvegicus GN=Atp5b PE=1 SV=2	VVDLLAPYAK
>	sp|P10719|ATF ATP synthase subunit beta, mitochondrial OS=Rattus norvegicus GN=Atp5b PE=1 SV=2	VVDLLAPYAK
>	sp|P10719|ATF ATP synthase subunit beta, mitochondrial OS=Rattus norvegicus GN=Atp5b PE=1 SV=2	VVDLLAPYAK
>	sp|P10719|ATF ATP synthase subunit beta, mitochondrial OS=Rattus norvegicus GN=Atp5b PE=1 SV=2	VVDLLAPYAK
>	sp|P10719|ATF ATP synthase subunit beta, mitochondrial OS=Rattus norvegicus GN=Atp5b PE=1 SV=2	VVDLLAPYAK
>	sp|P10719|ATF ATP synthase subunit beta, mitochondrial OS=Rattus norvegicus GN=Atp5b PE=1 SV=2	VVDLLAPYAK
>	sp|P10719|ATF ATP synthase subunit beta, mitochondrial OS=Rattus norvegicus GN=Atp5b PE=1 SV=2	VLDSGAPIKIPVGPETLGR
>	sp|P10719|ATF ATP synthase subunit beta, mitochondrial OS=Rattus norvegicus GN=Atp5b PE=1 SV=2	TVLIMELINNVAK
>	sp|P10719|ATF ATP synthase subunit beta, mitochondrial OS=Rattus norvegicus GN=Atp5b PE=1 SV=2	GFQQILAGDYDHLPEQAFYMVGPIEEAVAK
>	sp|P10719|ATF ATP synthase subunit beta, mitochondrial OS=Rattus norvegicus GN=Atp5b PE=1 SV=2	IGLFGGAGVGK
>	sp|P10719|ATF ATP synthase subunit beta, mitochondrial OS=Rattus norvegicus GN=Atp5b PE=1 SV=2	KGSITSVQAIYVPADDLTDPAPATTFAHLDATTVLSR
>	sp|P10719|ATF ATP synthase subunit beta, mitochondrial OS=Rattus norvegicus GN=Atp5b PE=1 SV=2	SLQDIIAILGMDELSEEDKLTVSR
>	sp|P10719|ATF ATP synthase subunit beta, mitochondrial OS=Rattus norvegicus GN=Atp5b PE=1 SV=2	LVLEVAQHLGESTVR
>	sp|P10719|ATF ATP synthase subunit beta, mitochondrial OS=Rattus norvegicus GN=Atp5b PE=1 SV=2	VLDSGAPIKIPVGPETLGR
>	sp|P10719|ATF ATP synthase subunit beta, mitochondrial OS=Rattus norvegicus GN=Atp5b PE=1 SV=2	FTQAGSEVSALLGR
>	sp|P10719|ATF ATP synthase subunit beta, mitochondrial OS=Rattus norvegicus GN=Atp5b PE=1 SV=2	VVDLLAPYAK
>	sp|P10719|ATF ATP synthase subunit beta, mitochondrial OS=Rattus norvegicus GN=Atp5b PE=1 SV=2	KGSITSVQAIYVPADDLTDPAPATTFAHLDATTVLSR

>	sp|P10719|ATF ATP synthase subunit beta, mitochondrial OS=Rattus norvegicus GN=Atp5b PE=1 SV=2	EGNDLYHEMIESGVINLK
>	sp|P10719|ATF ATP synthase subunit beta, mitochondrial OS=Rattus norvegicus GN=Atp5b PE=1 SV=2	IMNVIGEPIDER
>	sp|P10719|ATF ATP synthase subunit beta, mitochondrial OS=Rattus norvegicus GN=Atp5b PE=1 SV=2	VVDLLAPYAK
>	sp|P10719|ATF ATP synthase subunit beta, mitochondrial OS=Rattus norvegicus GN=Atp5b PE=1 SV=2	VALVYGQMNEPPGAR
>	sp|P10719|ATF ATP synthase subunit beta, mitochondrial OS=Rattus norvegicus GN=Atp5b PE=1 SV=2	LVLEVAQHLGESTVR
>	sp|P10719|ATF ATP synthase subunit beta, mitochondrial OS=Rattus norvegicus GN=Atp5b PE=1 SV=2	QFAPIHAEAPEFIEMSVEQEILVTGIK
>	sp|P10719|ATF ATP synthase subunit beta, mitochondrial OS=Rattus norvegicus GN=Atp5b PE=1 SV=2	VALVYGQMNEPPGAR
>	sp|P10719|ATF ATP synthase subunit beta, mitochondrial OS=Rattus norvegicus GN=Atp5b PE=1 SV=2	VVDLLAPYAK
>	sp|P10719|ATF ATP synthase subunit beta, mitochondrial OS=Rattus norvegicus GN=Atp5b PE=1 SV=2	VLDSGAPIKIPVGPETLGR
>	sp|P10719|ATF ATP synthase subunit beta, mitochondrial OS=Rattus norvegicus GN=Atp5b PE=1 SV=2	KGSITSVQAIYVPADDLTDPAPATTFAHLDATTVLSR
>	sp|P10719|ATF ATP synthase subunit beta, mitochondrial OS=Rattus norvegicus GN=Atp5b PE=1 SV=2	TIAMDGTEGLVR
>	sp|P10719|ATF ATP synthase subunit beta, mitochondrial OS=Rattus norvegicus GN=Atp5b PE=1 SV=2	VVDLLAPYAK
>	sp|P10719|ATF ATP synthase subunit beta, mitochondrial OS=Rattus norvegicus GN=Atp5b PE=1 SV=2	QFAPIHAEAPEFIEMSVEQEILVTGIK
>	sp|P10719|ATF ATP synthase subunit beta, mitochondrial OS=Rattus norvegicus GN=Atp5b PE=1 SV=2	VVDLLAPYAK
>	sp|P10719|ATF ATP synthase subunit beta, mitochondrial OS=Rattus norvegicus GN=Atp5b PE=1 SV=2	IMNVIGEPIDER
>	sp|P10719|ATF ATP synthase subunit beta, mitochondrial OS=Rattus norvegicus GN=Atp5b PE=1 SV=2	VVDLLAPYAK
>	sp|P10719|ATF ATP synthase subunit beta, mitochondrial OS=Rattus norvegicus GN=Atp5b PE=1 SV=2	VLDSGAPIKIPVGPETLGR
>	sp|P10719|ATF ATP synthase subunit beta, mitochondrial OS=Rattus norvegicus GN=Atp5b PE=1 SV=2	LVLEVAQHLGESTVR
>	sp|P10719|ATF ATP synthase subunit beta, mitochondrial OS=Rattus norvegicus GN=Atp5b PE=1 SV=2	IMNVIGEPIDERGPIK
>	sp|P10719|ATF ATP synthase subunit beta, mitochondrial OS=Rattus norvegicus GN=Atp5b PE=1 SV=2	KGSITSVQAIYVPADDLTDPAPATTFAHLDATTVLSR
>	sp|P10719|ATF ATP synthase subunit beta, mitochondrial OS=Rattus norvegicus GN=Atp5b PE=1 SV=2	TVLIMELINNVAK
>	sp|P10719|ATF ATP synthase subunit beta, mitochondrial OS=Rattus norvegicus GN=Atp5b PE=1 SV=2	GFQQILAGDYDHLPEQAFYMVGPIEEAVAK
>	sp|P10719|ATF ATP synthase subunit beta, mitochondrial OS=Rattus norvegicus GN=Atp5b PE=1 SV=2	ADKLAEEHGS
>	sp|P10719|ATF ATP synthase subunit beta, mitochondrial OS=Rattus norvegicus GN=Atp5b PE=1 SV=2	FLSQPFQVAEVFTGHMGK
>	sp|P10719|ATF ATP synthase subunit beta, mitochondrial OS=Rattus norvegicus GN=Atp5b PE=1 SV=2	GGKIGLFGGAGVGKTVLIMELINNVAKAHGGYSVFAGVGER
>	sp|P10719|ATF ATP synthase subunit beta, mitochondrial OS=Rattus norvegicus GN=Atp5b PE=1 SV=2	GGKIGLFGGAGVGKTVLIMELINNVAKAHGGYSVFAGVGER
>	sp|P10719|ATF ATP synthase subunit beta, mitochondrial OS=Rattus norvegicus GN=Atp5b PE=1 SV=2	KGSITSVQAIYVPADDLTDPAPATTFAHLDATTVLSR
>	sp|P10719|ATF ATP synthase subunit beta, mitochondrial OS=Rattus norvegicus GN=Atp5b PE=1 SV=2	AAQSSAAPK
>	sp|P10719|ATF ATP synthase subunit beta, mitochondrial OS=Rattus norvegicus GN=Atp5b PE=1 SV=2	TVLIMELINNVAK
>	sp|P10719|ATF ATP synthase subunit beta, mitochondrial OS=Rattus norvegicus GN=Atp5b PE=1 SV=2	GFQQILAGDYDHLPEQAFYMVGPIEEAVAK
>	sp|P10719|ATF ATP synthase subunit beta, mitochondrial OS=Rattus norvegicus GN=Atp5b PE=1 SV=2	IMDPNIVGSEHYDVAR
>	sp|P10719|ATF ATP synthase subunit beta, mitochondrial OS=Rattus norvegicus GN=Atp5b PE=1 SV=2	KGSITSVQAIYVPADDLTDPAPATTFAHLDATTVLSR
>	sp|P10719|ATF ATP synthase subunit beta, mitochondrial OS=Rattus norvegicus GN=Atp5b PE=1 SV=2	VVDLLAPYAK
>	sp|P10719|ATF ATP synthase subunit beta, mitochondrial OS=Rattus norvegicus GN=Atp5b PE=1 SV=2	VVDLLAPYAK
>	sp|P10719|ATF ATP synthase subunit beta, mitochondrial OS=Rattus norvegicus GN=Atp5b PE=1 SV=2	FLSQPFQVAEVFTGHMGK
>	sp|P10719|ATF ATP synthase subunit beta, mitochondrial OS=Rattus norvegicus GN=Atp5b PE=1 SV=2	VLDSGAPIKIPVGPETLGR
>	sp|P10719|ATF ATP synthase subunit beta, mitochondrial OS=Rattus norvegicus GN=Atp5b PE=1 SV=2	VVDLLAPYAK
>	sp|P10719|ATF ATP synthase subunit beta, mitochondrial OS=Rattus norvegicus GN=Atp5b PE=1 SV=2	KGSITSVQAIYVPADDLTDPAPATTFAHLDATTVLSR
>	sp|P10719|ATF ATP synthase subunit beta, mitochondrial OS=Rattus norvegicus GN=Atp5b PE=1 SV=2	LVLEVAQHLGESTVRTIAMDGTEGLVR
>	sp|P10719|ATF ATP synthase subunit beta, mitochondrial OS=Rattus norvegicus GN=Atp5b PE=1 SV=2	VVDLLAPYAK
>	sp|P10719|ATF ATP synthase subunit beta, mitochondrial OS=Rattus norvegicus GN=Atp5b PE=1 SV=2	IMDPNIVGSEHYDVAR
>	sp|P10719|ATF ATP synthase subunit beta, mitochondrial OS=Rattus norvegicus GN=Atp5b PE=1 SV=2	IMDPNIVGSEHYDVAR
>	sp|P10719|ATF ATP synthase subunit beta, mitochondrial OS=Rattus norvegicus GN=Atp5b PE=1 SV=2	IMNVIGEPIDER
>	sp|P10719|ATF ATP synthase subunit beta, mitochondrial OS=Rattus norvegicus GN=Atp5b PE=1 SV=2	AIAELGIYPAVDPLDSTSR
>	sp|P10719|ATF ATP synthase subunit beta, mitochondrial OS=Rattus norvegicus GN=Atp5b PE=1 SV=2	AHGGYSVFAGVGER
>	sp|P10719|ATF ATP synthase subunit beta, mitochondrial OS=Rattus norvegicus GN=Atp5b PE=1 SV=2	VVDLLAPYAK
>	sp|P10719|ATF ATP synthase subunit beta, mitochondrial OS=Rattus norvegicus GN=Atp5b PE=1 SV=2	FTGHMGK
>	sp|P10719|ATF ATP synthase subunit beta, mitochondrial OS=Rattus norvegicus GN=Atp5b PE=1 SV=2	EGNDLYHEMIESGVINLK
>	sp|P10719|ATF ATP synthase subunit beta, mitochondrial OS=Rattus norvegicus GN=Atp5b PE=1 SV=2	KGSITSVQAIYVPADDLTDPAPATTFAHLDATTVLSR
>	sp|P10719|ATF ATP synthase subunit beta, mitochondrial OS=Rattus norvegicus GN=Atp5b PE=1 SV=2	TIAMDGTEGLVR
>	sp|P10719|ATF ATP synthase subunit beta, mitochondrial OS=Rattus norvegicus GN=Atp5b PE=1 SV=2	VVDLLAPYAK
>	sp|P10719|ATF ATP synthase subunit beta, mitochondrial OS=Rattus norvegicus GN=Atp5b PE=1 SV=2	KGSITSVQAIYVPADDLTDPAPATTFAHLDATTVLSR
>	sp|P10719|ATF ATP synthase subunit beta, mitochondrial OS=Rattus norvegicus GN=Atp5b PE=1 SV=2	LVLEVAQHLGESTVRTIAMDGTEGLVR
>	sp|P10719|ATF ATP synthase subunit beta, mitochondrial OS=Rattus norvegicus GN=Atp5b PE=1 SV=2	VVDLLAPYAK
>	sp|P10719|ATF ATP synthase subunit beta, mitochondrial OS=Rattus norvegicus GN=Atp5b PE=1 SV=2	KGSITSVQAIYVPADDLTDPAPATTFAHLDATTVLSR
>	sp|P10719|ATF ATP synthase subunit beta, mitochondrial OS=Rattus norvegicus GN=Atp5b PE=1 SV=2	KGSITSVQAIYVPADDLTDPAPATTFAHLDATTVLSR
>	sp|P04797|G3I Glyceraldehyde-3-phosphate dehydrogenase OS=Rattus norvegicus GN=Gapdh PE=1 SV=3	AAFSCDKVDIVAINDPFIDLNYMVYMFQYDSTHGK
>	sp|P04797|G3I Glyceraldehyde-3-phosphate dehydrogenase OS=Rattus norvegicus GN=Gapdh PE=1 SV=3	GAAQNIIPASTGAAK
>	sp|P04797|G3I Glyceraldehyde-3-phosphate dehydrogenase OS=Rattus norvegicus GN=Gapdh PE=1 SV=3	LEKPAKYDDIKK
>	sp|P04797|G3I Glyceraldehyde-3-phosphate dehydrogenase OS=Rattus norvegicus GN=Gapdh PE=1 SV=3	LTGMAFR
>	sp|P04797|G3I Glyceraldehyde-3-phosphate dehydrogenase OS=Rattus norvegicus GN=Gapdh PE=1 SV=3	LVINGKPITIFQERDPANIK
>	sp|P04797|G3I Glyceraldehyde-3-phosphate dehydrogenase OS=Rattus norvegicus GN=Gapdh PE=1 SV=3	RVIISAPSADAPMFVMGVNHEK
>	sp|P04797|G3I Glyceraldehyde-3-phosphate dehydrogenase OS=Rattus norvegicus GN=Gapdh PE=1 SV=3	VDIVAINDPFIDLNYMVYMFQYDSTHGK
>	sp|P04797|G3I Glyceraldehyde-3-phosphate dehydrogenase OS=Rattus norvegicus GN=Gapdh PE=1 SV=3	VIHDNFGIVEGLMTTVHAITATQK
>	sp|P04797|G3I Glyceraldehyde-3-phosphate dehydrogenase OS=Rattus norvegicus GN=Gapdh PE=1 SV=3	VIHDNFGIVEGLMTTVHAITATQKTVDGPS
>	sp|P04797|G3I Glyceraldehyde-3-phosphate dehydrogenase OS=Rattus norvegicus GN=Gapdh PE=1 SV=3	VIISAPSADAPMFVMGVNHEK
>	sp|P04797|G3I Glyceraldehyde-3-phosphate dehydrogenase OS=Rattus norvegicus GN=Gapdh PE=1 SV=3	VIPELNGK
>	sp|P04797|G3I Glyceraldehyde-3-phosphate dehydrogenase OS=Rattus norvegicus GN=Gapdh PE=1 SV=3	VPTPNVSVVDLTCR
>	sp|P04797|G3I Glyceraldehyde-3-phosphate dehydrogenase OS=Rattus norvegicus GN=Gapdh PE=1 SV=3	VVDLMAYMASK
>	sp|P04797|G3I Glyceraldehyde-3-phosphate dehydrogenase OS=Rattus norvegicus GN=Gapdh PE=1 SV=3	VVDLMAYMASKE
>	sp|P04797|G3I Glyceraldehyde-3-phosphate dehydrogenase OS=Rattus norvegicus GN=Gapdh PE=1 SV=3	WGDAGAEYVVESTGVFTTMEK
>	sp|P04797|G3I Glyceraldehyde-3-phosphate dehydrogenase OS=Rattus norvegicus GN=Gapdh PE=1 SV=3	VIPELNGKLTGMAFR
>	sp|P04797|G3I Glyceraldehyde-3-phosphate dehydrogenase OS=Rattus norvegicus GN=Gapdh PE=1 SV=3	AENGKLVINGKPITIFQER
>	sp|P04797|G3I Glyceraldehyde-3-phosphate dehydrogenase OS=Rattus norvegicus GN=Gapdh PE=1 SV=3	IVSNASCTTNCLAPLAK

<table>
<tr><td>></td><td>sp|P04797|G3F Glyceraldehyde-3-phosphate dehydrogenase OS=Rattus norvegicus GN=Gapdh PE=1 SV=3</td><td>AENGKLVINGKPITIFQERDPANIK</td></tr>
<tr><td>></td><td>sp|P04797|G3F Glyceraldehyde-3-phosphate dehydrogenase OS=Rattus norvegicus GN=Gapdh PE=1 SV=3</td><td>VIHDNFGIVEGLMTTVHAITATQ</td></tr>
<tr><td>></td><td>sp|P04797|G3F Glyceraldehyde-3-phosphate dehydrogenase OS=Rattus norvegicus GN=Gapdh PE=1 SV=3</td><td>VIHDNFGIVEGLMTTVHAI</td></tr>
<tr><td>></td><td>sp|P04797|G3F Glyceraldehyde-3-phosphate dehydrogenase OS=Rattus norvegicus GN=Gapdh PE=1 SV=3</td><td>PSADAPMFVMGVNHEK</td></tr>
<tr><td>></td><td>sp|P04797|G3F Glyceraldehyde-3-phosphate dehydrogenase OS=Rattus norvegicus GN=Gapdh PE=1 SV=3</td><td>QAAEGPLK</td></tr>
<tr><td>></td><td>sp|P04797|G3F Glyceraldehyde-3-phosphate dehydrogenase OS=Rattus norvegicus GN=Gapdh PE=1 SV=3</td><td>VIHDNFGIVEGLMTTVHAITAT</td></tr>
<tr><td>></td><td>sp|P04797|G3F Glyceraldehyde-3-phosphate dehydrogenase OS=Rattus norvegicus GN=Gapdh PE=1 SV=3</td><td>VIHDNFGIVEGLMTTVHAITA</td></tr>
<tr><td>></td><td>sp|P04797|G3F Glyceraldehyde-3-phosphate dehydrogenase OS=Rattus norvegicus GN=Gapdh PE=1 SV=3</td><td>VIHDNFGIVEGL</td></tr>
<tr><td>></td><td>sp|P04797|G3F Glyceraldehyde-3-phosphate dehydrogenase OS=Rattus norvegicus GN=Gapdh PE=1 SV=3</td><td>VIHDNFGIVEGLMTTVHAITATQKTVDGP</td></tr>
<tr><td>></td><td>sp|P04797|G3F Glyceraldehyde-3-phosphate dehydrogenase OS=Rattus norvegicus GN=Gapdh PE=1 SV=3</td><td>GILGYTEDQVVSCDFNSNSHSSTFDAGAGIALNDNFVK</td></tr>
<tr><td>></td><td>sp|P04797|G3F Glyceraldehyde-3-phosphate dehydrogenase OS=Rattus norvegicus GN=Gapdh PE=1 SV=3</td><td>LISWYDNEYGYSNR</td></tr>
<tr><td>></td><td>sp|P04797|G3F Glyceraldehyde-3-phosphate dehydrogenase OS=Rattus norvegicus GN=Gapdh PE=1 SV=3</td><td>VIHDNFGIVEG</td></tr>
<tr><td>></td><td>sp|P04797|G3F Glyceraldehyde-3-phosphate dehydrogenase OS=Rattus norvegicus GN=Gapdh PE=1 SV=3</td><td>VIHDNFGIVEGLMTTVHA</td></tr>
<tr><td>></td><td>sp|P04797|G3F Glyceraldehyde-3-phosphate dehydrogenase OS=Rattus norvegicus GN=Gapdh PE=1 SV=3</td><td>VIHDNFGIVEGLMTTVHAITATQKTVDGPSGKLWR</td></tr>
<tr><td>></td><td>sp|P04797|G3F Glyceraldehyde-3-phosphate dehydrogenase OS=Rattus norvegicus GN=Gapdh PE=1 SV=3</td><td>AAFSCDKVDIVAINDPFIDLNYMVYMFQYDSTHGK</td></tr>
<tr><td>></td><td>sp|P04797|G3F Glyceraldehyde-3-phosphate dehydrogenase OS=Rattus norvegicus GN=Gapdh PE=1 SV=3</td><td>AAFSCDKVDIVAINDPFIDLNYMVYMFQYDSTHGK</td></tr>
<tr><td>></td><td>sp|P04797|G3F Glyceraldehyde-3-phosphate dehydrogenase OS=Rattus norvegicus GN=Gapdh PE=1 SV=3</td><td>AAFSCDKVDIVAINDPFIDLNYMVYMFQYDSTHGK</td></tr>
<tr><td>></td><td>sp|P04797|G3F Glyceraldehyde-3-phosphate dehydrogenase OS=Rattus norvegicus GN=Gapdh PE=1 SV=3</td><td>AAFSCDKVDIVAINDPFIDLNYMVYMFQYDSTHGK</td></tr>
<tr><td>></td><td>sp|P04797|G3F Glyceraldehyde-3-phosphate dehydrogenase OS=Rattus norvegicus GN=Gapdh PE=1 SV=3</td><td>AAFSCDKVDIVAINDPFIDLNYMVYMFQYDSTHGK</td></tr>
<tr><td>></td><td>sp|P04797|G3F Glyceraldehyde-3-phosphate dehydrogenase OS=Rattus norvegicus GN=Gapdh PE=1 SV=3</td><td>AAFSCDKVDIVAINDPFIDLNYMVYMFQYDSTHGK</td></tr>
<tr><td>></td><td>sp|P04797|G3F Glyceraldehyde-3-phosphate dehydrogenase OS=Rattus norvegicus GN=Gapdh PE=1 SV=3</td><td>AAFSCDKVDIVAINDPFIDLNYMVYMFQYDSTHGK</td></tr>
<tr><td>></td><td>sp|P04797|G3F Glyceraldehyde-3-phosphate dehydrogenase OS=Rattus norvegicus GN=Gapdh PE=1 SV=3</td><td>AAFSCDKVDIVAINDPFIDLNYMVYMFQYDSTHGK</td></tr>
<tr><td>></td><td>sp|P04797|G3F Glyceraldehyde-3-phosphate dehydrogenase OS=Rattus norvegicus GN=Gapdh PE=1 SV=3</td><td>AENGKLVINGKPITIFQER</td></tr>
<tr><td>></td><td>sp|P04797|G3F Glyceraldehyde-3-phosphate dehydrogenase OS=Rattus norvegicus GN=Gapdh PE=1 SV=3</td><td>AENGKLVINGKPITIFQER</td></tr>
<tr><td>></td><td>sp|P04797|G3F Glyceraldehyde-3-phosphate dehydrogenase OS=Rattus norvegicus GN=Gapdh PE=1 SV=3</td><td>AENGKLVINGKPITIFQERDPANIK</td></tr>
<tr><td>></td><td>sp|P04797|G3F Glyceraldehyde-3-phosphate dehydrogenase OS=Rattus norvegicus GN=Gapdh PE=1 SV=3</td><td>AENGKLVINGKPITIFQERDPANIK</td></tr>
<tr><td>></td><td>sp|P04797|G3F Glyceraldehyde-3-phosphate dehydrogenase OS=Rattus norvegicus GN=Gapdh PE=1 SV=3</td><td>GAAQNIIPASTGAAK</td></tr>
<tr><td>></td><td>sp|P04797|G3F Glyceraldehyde-3-phosphate dehydrogenase OS=Rattus norvegicus GN=Gapdh PE=1 SV=3</td><td>GAAQNIIPASTGAAK</td></tr>
<tr><td>></td><td>sp|P04797|G3F Glyceraldehyde-3-phosphate dehydrogenase OS=Rattus norvegicus GN=Gapdh PE=1 SV=3</td><td>LISWYDNEYGYSNR</td></tr>
<tr><td>></td><td>sp|P04797|G3F Glyceraldehyde-3-phosphate dehydrogenase OS=Rattus norvegicus GN=Gapdh PE=1 SV=3</td><td>LTGMAFR</td></tr>
<tr><td>></td><td>sp|P04797|G3F Glyceraldehyde-3-phosphate dehydrogenase OS=Rattus norvegicus GN=Gapdh PE=1 SV=3</td><td>LTGMAFR</td></tr>
<tr><td>></td><td>sp|P04797|G3F Glyceraldehyde-3-phosphate dehydrogenase OS=Rattus norvegicus GN=Gapdh PE=1 SV=3</td><td>RVIISAPSADAPMFVMGVNHEK</td></tr>
<tr><td>></td><td>sp|P04797|G3F Glyceraldehyde-3-phosphate dehydrogenase OS=Rattus norvegicus GN=Gapdh PE=1 SV=3</td><td>RVIISAPSADAPMFVMGVNHEK</td></tr>
<tr><td>></td><td>sp|P04797|G3F Glyceraldehyde-3-phosphate dehydrogenase OS=Rattus norvegicus GN=Gapdh PE=1 SV=3</td><td>RVIISAPSADAPMFVMGVNHEK</td></tr>
<tr><td>></td><td>sp|P04797|G3F Glyceraldehyde-3-phosphate dehydrogenase OS=Rattus norvegicus GN=Gapdh PE=1 SV=3</td><td>RVIISAPSADAPMFVMGVNHEK</td></tr>
<tr><td>></td><td>sp|P04797|G3F Glyceraldehyde-3-phosphate dehydrogenase OS=Rattus norvegicus GN=Gapdh PE=1 SV=3</td><td>RVIISAPSADAPMFVMGVNHEK</td></tr>
<tr><td>></td><td>sp|P04797|G3F Glyceraldehyde-3-phosphate dehydrogenase OS=Rattus norvegicus GN=Gapdh PE=1 SV=3</td><td>RVIISAPSADAPMFVMGVNHEK</td></tr>
<tr><td>></td><td>sp|P04797|G3F Glyceraldehyde-3-phosphate dehydrogenase OS=Rattus norvegicus GN=Gapdh PE=1 SV=3</td><td>VDIVAINDPFIDLNYMVYMFQYDSTHGK</td></tr>
<tr><td>></td><td>sp|P04797|G3F Glyceraldehyde-3-phosphate dehydrogenase OS=Rattus norvegicus GN=Gapdh PE=1 SV=3</td><td>VDIVAINDPFIDLNYMVYMFQYDSTHGK</td></tr>
<tr><td>></td><td>sp|P04797|G3F Glyceraldehyde-3-phosphate dehydrogenase OS=Rattus norvegicus GN=Gapdh PE=1 SV=3</td><td>VDIVAINDPFIDLNYMVYMFQYDSTHGK</td></tr>
<tr><td>></td><td>sp|P04797|G3F Glyceraldehyde-3-phosphate dehydrogenase OS=Rattus norvegicus GN=Gapdh PE=1 SV=3</td><td>VDIVAINDPFIDLNYMVYMFQYDSTHGK</td></tr>
<tr><td>></td><td>sp|P04797|G3F Glyceraldehyde-3-phosphate dehydrogenase OS=Rattus norvegicus GN=Gapdh PE=1 SV=3</td><td>VIHDNFGIVEG</td></tr>
<tr><td>></td><td>sp|P04797|G3F Glyceraldehyde-3-phosphate dehydrogenase OS=Rattus norvegicus GN=Gapdh PE=1 SV=3</td><td>VIHDNFGIVEGLMTTVHAITAT</td></tr>
<tr><td>></td><td>sp|P04797|G3F Glyceraldehyde-3-phosphate dehydrogenase OS=Rattus norvegicus GN=Gapdh PE=1 SV=3</td><td>VIHDNFGIVEGLMTTVHAITATQ</td></tr>
<tr><td>></td><td>sp|P04797|G3F Glyceraldehyde-3-phosphate dehydrogenase OS=Rattus norvegicus GN=Gapdh PE=1 SV=3</td><td>VIHDNFGIVEGLMTTVHAITATQ</td></tr>
<tr><td>></td><td>sp|P04797|G3F Glyceraldehyde-3-phosphate dehydrogenase OS=Rattus norvegicus GN=Gapdh PE=1 SV=3</td><td>VIHDNFGIVEGLMTTVHAITATQ</td></tr>
<tr><td>></td><td>sp|P04797|G3F Glyceraldehyde-3-phosphate dehydrogenase OS=Rattus norvegicus GN=Gapdh PE=1 SV=3</td><td>VIHDNFGIVEGLMTTVHAITATQK</td></tr>
<tr><td>></td><td>sp|P04797|G3F Glyceraldehyde-3-phosphate dehydrogenase OS=Rattus norvegicus GN=Gapdh PE=1 SV=3</td><td>VIHDNFGIVEGLMTTVHAITATQK</td></tr>
<tr><td>></td><td>sp|P04797|G3F Glyceraldehyde-3-phosphate dehydrogenase OS=Rattus norvegicus GN=Gapdh PE=1 SV=3</td><td>VIHDNFGIVEGLMTTVHAITATQK</td></tr>
<tr><td>></td><td>sp|P04797|G3F Glyceraldehyde-3-phosphate dehydrogenase OS=Rattus norvegicus GN=Gapdh PE=1 SV=3</td><td>VIHDNFGIVEGLMTTVHAITATQK</td></tr>
<tr><td>></td><td>sp|P04797|G3F Glyceraldehyde-3-phosphate dehydrogenase OS=Rattus norvegicus GN=Gapdh PE=1 SV=3</td><td>VIHDNFGIVEGLMTTVHAITATQK</td></tr>
<tr><td>></td><td>sp|P04797|G3F Glyceraldehyde-3-phosphate dehydrogenase OS=Rattus norvegicus GN=Gapdh PE=1 SV=3</td><td>VIHDNFGIVEGLMTTVHAITATQK</td></tr>
<tr><td>></td><td>sp|P04797|G3F Glyceraldehyde-3-phosphate dehydrogenase OS=Rattus norvegicus GN=Gapdh PE=1 SV=3</td><td>VIHDNFGIVEGLMTTVHAITATQK</td></tr>
<tr><td>></td><td>sp|P04797|G3F Glyceraldehyde-3-phosphate dehydrogenase OS=Rattus norvegicus GN=Gapdh PE=1 SV=3</td><td>VIHDNFGIVEGLMTTVHAITATQK</td></tr>
<tr><td>></td><td>sp|P04797|G3F Glyceraldehyde-3-phosphate dehydrogenase OS=Rattus norvegicus GN=Gapdh PE=1 SV=3</td><td>VIHDNFGIVEGLMTTVHAITATQK</td></tr>
<tr><td>></td><td>sp|P04797|G3F Glyceraldehyde-3-phosphate dehydrogenase OS=Rattus norvegicus GN=Gapdh PE=1 SV=3</td><td>VIHDNFGIVEGLMTTVHAITATQK</td></tr>
<tr><td>></td><td>sp|P04797|G3F Glyceraldehyde-3-phosphate dehydrogenase OS=Rattus norvegicus GN=Gapdh PE=1 SV=3</td><td>VIHDNFGIVEGLMTTVHAITATQK</td></tr>
<tr><td>></td><td>sp|P04797|G3F Glyceraldehyde-3-phosphate dehydrogenase OS=Rattus norvegicus GN=Gapdh PE=1 SV=3</td><td>VIHDNFGIVEGLMTTVHAITATQK</td></tr>
<tr><td>></td><td>sp|P04797|G3F Glyceraldehyde-3-phosphate dehydrogenase OS=Rattus norvegicus GN=Gapdh PE=1 SV=3</td><td>VIHDNFGIVEGLMTTVHAITATQK</td></tr>
<tr><td>></td><td>sp|P04797|G3F Glyceraldehyde-3-phosphate dehydrogenase OS=Rattus norvegicus GN=Gapdh PE=1 SV=3</td><td>VIHDNFGIVEGLMTTVHAITATQK</td></tr>
<tr><td>></td><td>sp|P04797|G3F Glyceraldehyde-3-phosphate dehydrogenase OS=Rattus norvegicus GN=Gapdh PE=1 SV=3</td><td>VIHDNFGIVEGLMTTVHAITATQK</td></tr>
<tr><td>></td><td>sp|P04797|G3F Glyceraldehyde-3-phosphate dehydrogenase OS=Rattus norvegicus GN=Gapdh PE=1 SV=3</td><td>VIHDNFGIVEGLMTTVHAITATQK</td></tr>
<tr><td>></td><td>sp|P04797|G3F Glyceraldehyde-3-phosphate dehydrogenase OS=Rattus norvegicus GN=Gapdh PE=1 SV=3</td><td>VIHDNFGIVEGLMTTVHAITATQK</td></tr>
<tr><td>></td><td>sp|P04797|G3F Glyceraldehyde-3-phosphate dehydrogenase OS=Rattus norvegicus GN=Gapdh PE=1 SV=3</td><td>VIHDNFGIVEGLMTTVHAITATQK</td></tr>
<tr><td>></td><td>sp|P04797|G3F Glyceraldehyde-3-phosphate dehydrogenase OS=Rattus norvegicus GN=Gapdh PE=1 SV=3</td><td>VIHDNFGIVEGLMTTVHAITATQK</td></tr>
<tr><td>></td><td>sp|P04797|G3F Glyceraldehyde-3-phosphate dehydrogenase OS=Rattus norvegicus GN=Gapdh PE=1 SV=3</td><td>VIHDNFGIVEGLMTTVHAITATQK</td></tr>
<tr><td>></td><td>sp|P04797|G3F Glyceraldehyde-3-phosphate dehydrogenase OS=Rattus norvegicus GN=Gapdh PE=1 SV=3</td><td>VIHDNFGIVEGLMTTVHAITATQK</td></tr>
<tr><td>></td><td>sp|P04797|G3F Glyceraldehyde-3-phosphate dehydrogenase OS=Rattus norvegicus GN=Gapdh PE=1 SV=3</td><td>VIHDNFGIVEGLMTTVHAITATQK</td></tr>
<tr><td>></td><td>sp|P04797|G3F Glyceraldehyde-3-phosphate dehydrogenase OS=Rattus norvegicus GN=Gapdh PE=1 SV=3</td><td>VIHDNFGIVEGLMTTVHAITATQK</td></tr>
<tr><td>></td><td>sp|P04797|G3F Glyceraldehyde-3-phosphate dehydrogenase OS=Rattus norvegicus GN=Gapdh PE=1 SV=3</td><td>VIHDNFGIVEGLMTTVHAITATQK</td></tr>
<tr><td>></td><td>sp|P04797|G3F Glyceraldehyde-3-phosphate dehydrogenase OS=Rattus norvegicus GN=Gapdh PE=1 SV=3</td><td>VIHDNFGIVEGLMTTVHAITATQK</td></tr>
<tr><td>></td><td>sp|P04797|G3F Glyceraldehyde-3-phosphate dehydrogenase OS=Rattus norvegicus GN=Gapdh PE=1 SV=3</td><td>VIHDNFGIVEGLMTTVHAITATQK</td></tr>
<tr><td>></td><td>sp|P04797|G3F Glyceraldehyde-3-phosphate dehydrogenase OS=Rattus norvegicus GN=Gapdh PE=1 SV=3</td><td>VIHDNFGIVEGLMTTVHAITATQK</td></tr>
<tr><td>></td><td>sp|P04797|G3F Glyceraldehyde-3-phosphate dehydrogenase OS=Rattus norvegicus GN=Gapdh PE=1 SV=3</td><td>VIHDNFGIVEGLMTTVHAITATQK</td></tr>
</table>

>	sp|P04797|G3P Glyceraldehyde-3-phosphate dehydrogenase OS=Rattus norvegicus GN=Gapdh PE=1 SV=3	VIHDNFGIVEGLMTTVHAITATQK
>	sp|P04797|G3P Glyceraldehyde-3-phosphate dehydrogenase OS=Rattus norvegicus GN=Gapdh PE=1 SV=3	VIHDNFGIVEGLMTTVHAITATQK
>	sp|P04797|G3P Glyceraldehyde-3-phosphate dehydrogenase OS=Rattus norvegicus GN=Gapdh PE=1 SV=3	VIHDNFGIVEGLMTTVHAITATQK
>	sp|P04797|G3P Glyceraldehyde-3-phosphate dehydrogenase OS=Rattus norvegicus GN=Gapdh PE=1 SV=3	VIHDNFGIVEGLMTTVHAITATQK
>	sp|P04797|G3P Glyceraldehyde-3-phosphate dehydrogenase OS=Rattus norvegicus GN=Gapdh PE=1 SV=3	VIHDNFGIVEGLMTTVHAITATQK
>	sp|P04797|G3P Glyceraldehyde-3-phosphate dehydrogenase OS=Rattus norvegicus GN=Gapdh PE=1 SV=3	VIHDNFGIVEGLMTTVHAITATQK
>	sp|P04797|G3P Glyceraldehyde-3-phosphate dehydrogenase OS=Rattus norvegicus GN=Gapdh PE=1 SV=3	VIHDNFGIVEGLMTTVHAITATQK
>	sp|P04797|G3P Glyceraldehyde-3-phosphate dehydrogenase OS=Rattus norvegicus GN=Gapdh PE=1 SV=3	VIHDNFGIVEGLMTTVHAITATQK
>	sp|P04797|G3P Glyceraldehyde-3-phosphate dehydrogenase OS=Rattus norvegicus GN=Gapdh PE=1 SV=3	VIHDNFGIVEGLMTTVHAITATQK
>	sp|P04797|G3P Glyceraldehyde-3-phosphate dehydrogenase OS=Rattus norvegicus GN=Gapdh PE=1 SV=3	VIHDNFGIVEGLMTTVHAITATQK
>	sp|P04797|G3P Glyceraldehyde-3-phosphate dehydrogenase OS=Rattus norvegicus GN=Gapdh PE=1 SV=3	VIHDNFGIVEGLMTTVHAITATQK
>	sp|P04797|G3P Glyceraldehyde-3-phosphate dehydrogenase OS=Rattus norvegicus GN=Gapdh PE=1 SV=3	VIHDNFGIVEGLMTTVHAITATQK
>	sp|P04797|G3P Glyceraldehyde-3-phosphate dehydrogenase OS=Rattus norvegicus GN=Gapdh PE=1 SV=3	VIHDNFGIVEGLMTTVHAITATQK
>	sp|P04797|G3P Glyceraldehyde-3-phosphate dehydrogenase OS=Rattus norvegicus GN=Gapdh PE=1 SV=3	VIHDNFGIVEGLMTTVHAITATQKTVDGPS
>	sp|P04797|G3P Glyceraldehyde-3-phosphate dehydrogenase OS=Rattus norvegicus GN=Gapdh PE=1 SV=3	VIISAPSADAPMFVMGVNHEK
>	sp|P04797|G3P Glyceraldehyde-3-phosphate dehydrogenase OS=Rattus norvegicus GN=Gapdh PE=1 SV=3	VIISAPSADAPMFVMGVNHEK
>	sp|P04797|G3P Glyceraldehyde-3-phosphate dehydrogenase OS=Rattus norvegicus GN=Gapdh PE=1 SV=3	VIISAPSADAPMFVMGVNHEK
>	sp|P04797|G3P Glyceraldehyde-3-phosphate dehydrogenase OS=Rattus norvegicus GN=Gapdh PE=1 SV=3	VIISAPSADAPMFVMGVNHEK
>	sp|P04797|G3P Glyceraldehyde-3-phosphate dehydrogenase OS=Rattus norvegicus GN=Gapdh PE=1 SV=3	VIISAPSADAPMFVMGVNHEK
>	sp|P04797|G3P Glyceraldehyde-3-phosphate dehydrogenase OS=Rattus norvegicus GN=Gapdh PE=1 SV=3	VIISAPSADAPMFVMGVNHEK
>	sp|P04797|G3P Glyceraldehyde-3-phosphate dehydrogenase OS=Rattus norvegicus GN=Gapdh PE=1 SV=3	VIISAPSADAPMFVMGVNHEK
>	sp|P04797|G3P Glyceraldehyde-3-phosphate dehydrogenase OS=Rattus norvegicus GN=Gapdh PE=1 SV=3	VIISAPSADAPMFVMGVNHEK
>	sp|P04797|G3P Glyceraldehyde-3-phosphate dehydrogenase OS=Rattus norvegicus GN=Gapdh PE=1 SV=3	VIISAPSADAPMFVMGVNHEK
>	sp|P04797|G3P Glyceraldehyde-3-phosphate dehydrogenase OS=Rattus norvegicus GN=Gapdh PE=1 SV=3	VPTPNVSVVDLTCR
>	sp|P04797|G3P Glyceraldehyde-3-phosphate dehydrogenase OS=Rattus norvegicus GN=Gapdh PE=1 SV=3	VPTPNVSVVDLTCR
>	sp|P04797|G3P Glyceraldehyde-3-phosphate dehydrogenase OS=Rattus norvegicus GN=Gapdh PE=1 SV=3	VPTPNVSVVDLTCR
>	sp|P04797|G3P Glyceraldehyde-3-phosphate dehydrogenase OS=Rattus norvegicus GN=Gapdh PE=1 SV=3	VPTPNVSVVDLTCR
>	sp|P04797|G3P Glyceraldehyde-3-phosphate dehydrogenase OS=Rattus norvegicus GN=Gapdh PE=1 SV=3	VPTPNVSVVDLTCR
>	sp|P04797|G3P Glyceraldehyde-3-phosphate dehydrogenase OS=Rattus norvegicus GN=Gapdh PE=1 SV=3	VPTPNVSVVDLTCR
>	sp|P04797|G3P Glyceraldehyde-3-phosphate dehydrogenase OS=Rattus norvegicus GN=Gapdh PE=1 SV=3	VVDLMAYMASK
>	sp|P04797|G3P Glyceraldehyde-3-phosphate dehydrogenase OS=Rattus norvegicus GN=Gapdh PE=1 SV=3	VVDLMAYMASK
>	sp|P04797|G3P Glyceraldehyde-3-phosphate dehydrogenase OS=Rattus norvegicus GN=Gapdh PE=1 SV=3	VVDLMAYMASK
>	sp|P04797|G3P Glyceraldehyde-3-phosphate dehydrogenase OS=Rattus norvegicus GN=Gapdh PE=1 SV=3	VVDLMAYMASK
>	sp|P04797|G3P Glyceraldehyde-3-phosphate dehydrogenase OS=Rattus norvegicus GN=Gapdh PE=1 SV=3	VVDLMAYMASK
>	sp|P04797|G3P Glyceraldehyde-3-phosphate dehydrogenase OS=Rattus norvegicus GN=Gapdh PE=1 SV=3	VVDLMAYMASK
>	sp|P04797|G3P Glyceraldehyde-3-phosphate dehydrogenase OS=Rattus norvegicus GN=Gapdh PE=1 SV=3	VVDLMAYMASK
>	sp|P04797|G3P Glyceraldehyde-3-phosphate dehydrogenase OS=Rattus norvegicus GN=Gapdh PE=1 SV=3	VVDLMAYMASK
>	sp|P04797|G3P Glyceraldehyde-3-phosphate dehydrogenase OS=Rattus norvegicus GN=Gapdh PE=1 SV=3	VVDLMAYMASKE
>	sp|P04797|G3P Glyceraldehyde-3-phosphate dehydrogenase OS=Rattus norvegicus GN=Gapdh PE=1 SV=3	VVDLMAYMASKE
>	sp|P04797|G3P Glyceraldehyde-3-phosphate dehydrogenase OS=Rattus norvegicus GN=Gapdh PE=1 SV=3	VVDLMAYMASKE
>	sp|P04797|G3P Glyceraldehyde-3-phosphate dehydrogenase OS=Rattus norvegicus GN=Gapdh PE=1 SV=3	VVDLMAYMASKE
>	sp|P04797|G3P Glyceraldehyde-3-phosphate dehydrogenase OS=Rattus norvegicus GN=Gapdh PE=1 SV=3	VVDLMAYMASKE
>	sp|P04797|G3P Glyceraldehyde-3-phosphate dehydrogenase OS=Rattus norvegicus GN=Gapdh PE=1 SV=3	VVDLMAYMASKE
>	sp|P04797|G3P Glyceraldehyde-3-phosphate dehydrogenase OS=Rattus norvegicus GN=Gapdh PE=1 SV=3	VVDLMAYMASKE
>	sp|P04797|G3P Glyceraldehyde-3-phosphate dehydrogenase OS=Rattus norvegicus GN=Gapdh PE=1 SV=3	VVDLMAYMASKE
>	sp|P04797|G3P Glyceraldehyde-3-phosphate dehydrogenase OS=Rattus norvegicus GN=Gapdh PE=1 SV=3	VVDLMAYMASKE
>	sp|P04797|G3P Glyceraldehyde-3-phosphate dehydrogenase OS=Rattus norvegicus GN=Gapdh PE=1 SV=3	VVDLMAYMASKE
>	sp|P04797|G3P Glyceraldehyde-3-phosphate dehydrogenase OS=Rattus norvegicus GN=Gapdh PE=1 SV=3	VVDLMAYMASKE
>	sp|P04797|G3P Glyceraldehyde-3-phosphate dehydrogenase OS=Rattus norvegicus GN=Gapdh PE=1 SV=3	VVDLMAYMASKE
>	sp|P04797|G3P Glyceraldehyde-3-phosphate dehydrogenase OS=Rattus norvegicus GN=Gapdh PE=1 SV=3	VVDLMAYMASKE
>	sp|P04797|G3P Glyceraldehyde-3-phosphate dehydrogenase OS=Rattus norvegicus GN=Gapdh PE=1 SV=3	VVDLMAYMASKE
>	sp|P04797|G3P Glyceraldehyde-3-phosphate dehydrogenase OS=Rattus norvegicus GN=Gapdh PE=1 SV=3	VVDLMAYMASKE
>	sp|P04797|G3P Glyceraldehyde-3-phosphate dehydrogenase OS=Rattus norvegicus GN=Gapdh PE=1 SV=3	VVDLMAYMASKE
>	sp|P04797|G3P Glyceraldehyde-3-phosphate dehydrogenase OS=Rattus norvegicus GN=Gapdh PE=1 SV=3	VVDLMAYMASKE
>	sp|P04797|G3P Glyceraldehyde-3-phosphate dehydrogenase OS=Rattus norvegicus GN=Gapdh PE=1 SV=3	VVDLMAYMASKE
>	sp|P04797|G3P Glyceraldehyde-3-phosphate dehydrogenase OS=Rattus norvegicus GN=Gapdh PE=1 SV=3	VVDLMAYMASKE
>	sp|P04797|G3P Glyceraldehyde-3-phosphate dehydrogenase OS=Rattus norvegicus GN=Gapdh PE=1 SV=3	VVDLMAYMASKE
>	sp|P04797|G3P Glyceraldehyde-3-phosphate dehydrogenase OS=Rattus norvegicus GN=Gapdh PE=1 SV=3	VVDLMAYMASKE
>	sp|P04797|G3P Glyceraldehyde-3-phosphate dehydrogenase OS=Rattus norvegicus GN=Gapdh PE=1 SV=3	VVDLMAYMASKE
>	sp|P04797|G3P Glyceraldehyde-3-phosphate dehydrogenase OS=Rattus norvegicus GN=Gapdh PE=1 SV=3	WGDAGAEYVVESTGVFTTMEK
>	sp|P04797|G3P Glyceraldehyde-3-phosphate dehydrogenase OS=Rattus norvegicus GN=Gapdh PE=1 SV=3	WGDAGAEYVVESTGVFTTMEK
>	sp|P04797|G3P Glyceraldehyde-3-phosphate dehydrogenase OS=Rattus norvegicus GN=Gapdh PE=1 SV=3	WGDAGAEYVVESTGVFTTMEK
>	sp|P04797|G3P Glyceraldehyde-3-phosphate dehydrogenase OS=Rattus norvegicus GN=Gapdh PE=1 SV=3	WGDAGAEYVVESTGVFTTMEK
>	sp|P04797|G3P Glyceraldehyde-3-phosphate dehydrogenase OS=Rattus norvegicus GN=Gapdh PE=1 SV=3	WGDAGAEYVVESTGVFTTMEK
>	sp|P04797|G3P Glyceraldehyde-3-phosphate dehydrogenase OS=Rattus norvegicus GN=Gapdh PE=1 SV=3	WGDAGAEYVVESTGVFTTMEK
>	sp|P04797|G3P Glyceraldehyde-3-phosphate dehydrogenase OS=Rattus norvegicus GN=Gapdh PE=1 SV=3	WGDAGAEYVVESTGVFTTMEK
>	sp|P04797|G3P Glyceraldehyde-3-phosphate dehydrogenase OS=Rattus norvegicus GN=Gapdh PE=1 SV=3	VIHDNFGIVEGLMTTVHAITATQKT
>	sp|P04797|G3P Glyceraldehyde-3-phosphate dehydrogenase OS=Rattus norvegicus GN=Gapdh PE=1 SV=3	VPTPNVSVVDLTCR
>	sp|P04797|G3P Glyceraldehyde-3-phosphate dehydrogenase OS=Rattus norvegicus GN=Gapdh PE=1 SV=3	VIHDNFGIVEGLMTTVHAITATQKTVDGPSGK
>	sp|P04797|G3P Glyceraldehyde-3-phosphate dehydrogenase OS=Rattus norvegicus GN=Gapdh PE=1 SV=3	QAAEGPLK
>	sp|P04797|G3P Glyceraldehyde-3-phosphate dehydrogenase OS=Rattus norvegicus GN=Gapdh PE=1 SV=3	VIISAPSADAPMFVMGVNHEK
>	sp|P04797|G3P Glyceraldehyde-3-phosphate dehydrogenase OS=Rattus norvegicus GN=Gapdh PE=1 SV=3	VIPELNGKLTGMAFR
>	sp|P04797|G3P Glyceraldehyde-3-phosphate dehydrogenase OS=Rattus norvegicus GN=Gapdh PE=1 SV=3	GAAQNIIPASTGAAK

> sp|P04797|G3F Glyceraldehyde-3-phosphate dehydrogenase OS=Rattus norvegicus GN=Gapdh PE=1 SV=3 VIHDNFGIVEGLMTTVHAITATQK
> sp|P04797|G3F Glyceraldehyde-3-phosphate dehydrogenase OS=Rattus norvegicus GN=Gapdh PE=1 SV=3 VVDLMAYMASKE
> sp|P04797|G3F Glyceraldehyde-3-phosphate dehydrogenase OS=Rattus norvegicus GN=Gapdh PE=1 SV=3 GILGYTEDQVVSCDFNSNSHSSTFDAGAGIALNDNFVK
> sp|P04797|G3F Glyceraldehyde-3-phosphate dehydrogenase OS=Rattus norvegicus GN=Gapdh PE=1 SV=3 IVSNASCTTNCLAPLAK
> sp|P04797|G3F Glyceraldehyde-3-phosphate dehydrogenase OS=Rattus norvegicus GN=Gapdh PE=1 SV=3 VIISAPSADAPMFVMGVNHEK
> sp|P04797|G3F Glyceraldehyde-3-phosphate dehydrogenase OS=Rattus norvegicus GN=Gapdh PE=1 SV=3 WGDAGAEYVVESTGVFTTMEK
> sp|P04797|G3F Glyceraldehyde-3-phosphate dehydrogenase OS=Rattus norvegicus GN=Gapdh PE=1 SV=3 VIPELNGK
> sp|P04797|G3F Glyceraldehyde-3-phosphate dehydrogenase OS=Rattus norvegicus GN=Gapdh PE=1 SV=3 VIHDNFGIVEGLMTTVHAITATQK
> sp|P04797|G3F Glyceraldehyde-3-phosphate dehydrogenase OS=Rattus norvegicus GN=Gapdh PE=1 SV=3 LVINGKPITIFQERDPANIK
> sp|P04797|G3F Glyceraldehyde-3-phosphate dehydrogenase OS=Rattus norvegicus GN=Gapdh PE=1 SV=3 VVDLMAYMASKE
> sp|P04797|G3F Glyceraldehyde-3-phosphate dehydrogenase OS=Rattus norvegicus GN=Gapdh PE=1 SV=3 VPTPNVSVVDLTCR
> sp|P04797|G3F Glyceraldehyde-3-phosphate dehydrogenase OS=Rattus norvegicus GN=Gapdh PE=1 SV=3 WGDAGAEYVVESTGVFTTMEK
> sp|P04797|G3F Glyceraldehyde-3-phosphate dehydrogenase OS=Rattus norvegicus GN=Gapdh PE=1 SV=3 VIHDNFGIVEGLMTTVHAITATQKT
> sp|P04797|G3F Glyceraldehyde-3-phosphate dehydrogenase OS=Rattus norvegicus GN=Gapdh PE=1 SV=3 VIISAPSADAPMFVMGVNHEK
> sp|P04797|G3F Glyceraldehyde-3-phosphate dehydrogenase OS=Rattus norvegicus GN=Gapdh PE=1 SV=3 VVDLMAYMASKE
> sp|P04797|G3F Glyceraldehyde-3-phosphate dehydrogenase OS=Rattus norvegicus GN=Gapdh PE=1 SV=3 LVINGKPITIFQER
> sp|P04797|G3F Glyceraldehyde-3-phosphate dehydrogenase OS=Rattus norvegicus GN=Gapdh PE=1 SV=3 VVDLMAYMASKE
> sp|P04797|G3F Glyceraldehyde-3-phosphate dehydrogenase OS=Rattus norvegicus GN=Gapdh PE=1 SV=3 LVINGKPITIFQERDPANIK
> sp|P04797|G3F Glyceraldehyde-3-phosphate dehydrogenase OS=Rattus norvegicus GN=Gapdh PE=1 SV=3 RVIISAPSADAPMFVMGVNHEK
> sp|P04797|G3F Glyceraldehyde-3-phosphate dehydrogenase OS=Rattus norvegicus GN=Gapdh PE=1 SV=3 VIPELNGK
> sp|P04797|G3F Glyceraldehyde-3-phosphate dehydrogenase OS=Rattus norvegicus GN=Gapdh PE=1 SV=3 VVDLMAYMASKE
> sp|P04797|G3F Glyceraldehyde-3-phosphate dehydrogenase OS=Rattus norvegicus GN=Gapdh PE=1 SV=3 IVSNASCTTNCLAPLAK
> sp|P04797|G3F Glyceraldehyde-3-phosphate dehydrogenase OS=Rattus norvegicus GN=Gapdh PE=1 SV=3 PSADAPMFVMGVNHEK
> sp|P04797|G3F Glyceraldehyde-3-phosphate dehydrogenase OS=Rattus norvegicus GN=Gapdh PE=1 SV=3 VIPELNGK
> sp|P04797|G3F Glyceraldehyde-3-phosphate dehydrogenase OS=Rattus norvegicus GN=Gapdh PE=1 SV=3 LISWYDNEYGYSNR
> sp|P04797|G3F Glyceraldehyde-3-phosphate dehydrogenase OS=Rattus norvegicus GN=Gapdh PE=1 SV=3 VPTPNVSVVDLTCR
> sp|P04797|G3F Glyceraldehyde-3-phosphate dehydrogenase OS=Rattus norvegicus GN=Gapdh PE=1 SV=3 LVINGKPITIFQERDPANIK
> sp|P04797|G3F Glyceraldehyde-3-phosphate dehydrogenase OS=Rattus norvegicus GN=Gapdh PE=1 SV=3 IVSNASCTTNCLAPLAK
> sp|P04797|G3F Glyceraldehyde-3-phosphate dehydrogenase OS=Rattus norvegicus GN=Gapdh PE=1 SV=3 LISWYDNEYGYSNR
> sp|P04797|G3F Glyceraldehyde-3-phosphate dehydrogenase OS=Rattus norvegicus GN=Gapdh PE=1 SV=3 VGVNGFGR
> sp|P04797|G3F Glyceraldehyde-3-phosphate dehydrogenase OS=Rattus norvegicus GN=Gapdh PE=1 SV=3 PSADAPMFVMGVNHEK
> sp|P04797|G3F Glyceraldehyde-3-phosphate dehydrogenase OS=Rattus norvegicus GN=Gapdh PE=1 SV=3 VIHDNFGIVEGLMTT
> sp|P04797|G3F Glyceraldehyde-3-phosphate dehydrogenase OS=Rattus norvegicus GN=Gapdh PE=1 SV=3 AENGKLVINGKPITIFQER
> sp|P04797|G3F Glyceraldehyde-3-phosphate dehydrogenase OS=Rattus norvegicus GN=Gapdh PE=1 SV=3 VIHDNFGIVEGLMTTVHAITATQK
> sp|P04797|G3F Glyceraldehyde-3-phosphate dehydrogenase OS=Rattus norvegicus GN=Gapdh PE=1 SV=3 VIISAPSADAPMFVMGVNHEK
> sp|P04797|G3F Glyceraldehyde-3-phosphate dehydrogenase OS=Rattus norvegicus GN=Gapdh PE=1 SV=3 VIHDNFGIVEGLMTTVHAITATQK
> sp|P04797|G3F Glyceraldehyde-3-phosphate dehydrogenase OS=Rattus norvegicus GN=Gapdh PE=1 SV=3 VIISAPSADAPMFVMGVNHEK
> sp|P04797|G3F Glyceraldehyde-3-phosphate dehydrogenase OS=Rattus norvegicus GN=Gapdh PE=1 SV=3 GAAQNIIPASTGAAK
> sp|P04797|G3F Glyceraldehyde-3-phosphate dehydrogenase OS=Rattus norvegicus GN=Gapdh PE=1 SV=3 VIISAPSADAPMFVMGVNHEK
> sp|P04797|G3F Glyceraldehyde-3-phosphate dehydrogenase OS=Rattus norvegicus GN=Gapdh PE=1 SV=3 IVSNASCTTNCLAPLAK
> sp|P04797|G3F Glyceraldehyde-3-phosphate dehydrogenase OS=Rattus norvegicus GN=Gapdh PE=1 SV=3 VIHDNFGIVEGLMTTVHAITATQK
> sp|P04797|G3F Glyceraldehyde-3-phosphate dehydrogenase OS=Rattus norvegicus GN=Gapdh PE=1 SV=3 VIPELNGK
> sp|P04797|G3F Glyceraldehyde-3-phosphate dehydrogenase OS=Rattus norvegicus GN=Gapdh PE=1 SV=3 VVDLMAYMASKE
> sp|P04797|G3F Glyceraldehyde-3-phosphate dehydrogenase OS=Rattus norvegicus GN=Gapdh PE=1 SV=3 VVDLMAYMASKE
> sp|P04797|G3F Glyceraldehyde-3-phosphate dehydrogenase OS=Rattus norvegicus GN=Gapdh PE=1 SV=3 VIISAPSADAPMFVMGVNHEK
> sp|P04797|G3F Glyceraldehyde-3-phosphate dehydrogenase OS=Rattus norvegicus GN=Gapdh PE=1 SV=3 VIISAPSADAPMFVMGVNHEK
> sp|P04797|G3F Glyceraldehyde-3-phosphate dehydrogenase OS=Rattus norvegicus GN=Gapdh PE=1 SV=3 VIPELNGK
> sp|P04797|G3F Glyceraldehyde-3-phosphate dehydrogenase OS=Rattus norvegicus GN=Gapdh PE=1 SV=3 PMFVMGVNHEK
> sp|P04797|G3F Glyceraldehyde-3-phosphate dehydrogenase OS=Rattus norvegicus GN=Gapdh PE=1 SV=3 AAFSCDK
> sp|P04797|G3F Glyceraldehyde-3-phosphate dehydrogenase OS=Rattus norvegicus GN=Gapdh PE=1 SV=3 VIHDNFGIVEGLMTTVHAITATQK
> sp|P04797|G3F Glyceraldehyde-3-phosphate dehydrogenase OS=Rattus norvegicus GN=Gapdh PE=1 SV=3 VGVNGFGR
> sp|P04797|G3F Glyceraldehyde-3-phosphate dehydrogenase OS=Rattus norvegicus GN=Gapdh PE=1 SV=3 AENGKLVINGKPITIFQERDPANIK
> sp|P04797|G3F Glyceraldehyde-3-phosphate dehydrogenase OS=Rattus norvegicus GN=Gapdh PE=1 SV=3 VIHDNFGIVEGLMTTVHAITATQK
> sp|P04797|G3F Glyceraldehyde-3-phosphate dehydrogenase OS=Rattus norvegicus GN=Gapdh PE=1 SV=3 IVSNASCTTNCLAPLAK
> sp|P04797|G3F Glyceraldehyde-3-phosphate dehydrogenase OS=Rattus norvegicus GN=Gapdh PE=1 SV=3 IVSNASCTTNCLAPLAK
> sp|P04797|G3F Glyceraldehyde-3-phosphate dehydrogenase OS=Rattus norvegicus GN=Gapdh PE=1 SV=3 LISWYDNEYGYSNR
> sp|P04797|G3F Glyceraldehyde-3-phosphate dehydrogenase OS=Rattus norvegicus GN=Gapdh PE=1 SV=3 VDIVAINDPFIDLNYMVYMFQYDSTHGK
> sp|P04797|G3F Glyceraldehyde-3-phosphate dehydrogenase OS=Rattus norvegicus GN=Gapdh PE=1 SV=3 VGVNGFGR
> sp|P04797|G3F Glyceraldehyde-3-phosphate dehydrogenase OS=Rattus norvegicus GN=Gapdh PE=1 SV=3 GILGYTEDQVVSCDFNSNSHSSTFDAGAGIALNDNFVK
> sp|P04797|G3F Glyceraldehyde-3-phosphate dehydrogenase OS=Rattus norvegicus GN=Gapdh PE=1 SV=3 AAFSCDKVDIVAINDPFIDLNYMVYMFQYDSTHGK
> sp|P04797|G3F Glyceraldehyde-3-phosphate dehydrogenase OS=Rattus norvegicus GN=Gapdh PE=1 SV=3 GILGYTEDQVVSCDFNSNSHSSTFDAGAGIALNDNFVK
> sp|P04797|G3F Glyceraldehyde-3-phosphate dehydrogenase OS=Rattus norvegicus GN=Gapdh PE=1 SV=3 RVIISAPSADAPMFVMGVNHEKYDNSLK
> sp|P04797|G3F Glyceraldehyde-3-phosphate dehydrogenase OS=Rattus norvegicus GN=Gapdh PE=1 SV=3 IVSNASCTTNCLAPLAK
> sp|P04797|G3F Glyceraldehyde-3-phosphate dehydrogenase OS=Rattus norvegicus GN=Gapdh PE=1 SV=3 AENGKLVINGKPITIFQER
> sp|P04797|G3F Glyceraldehyde-3-phosphate dehydrogenase OS=Rattus norvegicus GN=Gapdh PE=1 SV=3 VIHDNFGIVEGLMTTVHAITATQK
> sp|P04797|G3F Glyceraldehyde-3-phosphate dehydrogenase OS=Rattus norvegicus GN=Gapdh PE=1 SV=3 VKVGVNGFGR
> sp|P04797|G3F Glyceraldehyde-3-phosphate dehydrogenase OS=Rattus norvegicus GN=Gapdh PE=1 SV=3 WGDAGAEYVVESTGVFTTMEK
> sp|P04797|G3F Glyceraldehyde-3-phosphate dehydrogenase OS=Rattus norvegicus GN=Gapdh PE=1 SV=3 LTGMAFRVPTPNVSVVDLTCR
> sp|Q9ER34|AC(Aconitate hydratase, mitochondrial OS=Rattus norvegicus GN=Aco2 PE=1 SV=2 ADIANLAEEFKDHLVPDPGCQYDQVIEINLNELKPHINGPFTPDLAHPVADVGTVAEK
> sp|Q9ER34|AC(Aconitate hydratase, mitochondrial OS=Rattus norvegicus GN=Aco2 PE=1 SV=2 AKDINQEVYNFLATAGAK
> sp|Q9ER34|AC(Aconitate hydratase, mitochondrial OS=Rattus norvegicus GN=Aco2 PE=1 SV=2 DVGGIVLANACGPCIGQWDR
> sp|Q9ER34|AC(Aconitate hydratase, mitochondrial OS=Rattus norvegicus GN=Aco2 PE=1 SV=2 FKLEAPDADELPR
> sp|Q9ER34|AC(Aconitate hydratase, mitochondrial OS=Rattus norvegicus GN=Aco2 PE=1 SV=2 GHLDNISNNLLIGAINIENGK
> sp|Q9ER34|AC(Aconitate hydratase, mitochondrial OS=Rattus norvegicus GN=Aco2 PE=1 SV=2 GHLDNISNNLLIGAINIENGKANSVR

>	sp|Q9ER34|AC	Aconitate hydratase, mitochondrial OS=Rattus norvegicus GN=Aco2 PE=1 SV=2	HPNGTQETILLNHTFNETQIEWFR
>	sp|Q9ER34|AC	Aconitate hydratase, mitochondrial OS=Rattus norvegicus GN=Aco2 PE=1 SV=2	IVYGHLDDPANQEIER
>	sp|Q9ER34|AC	Aconitate hydratase, mitochondrial OS=Rattus norvegicus GN=Aco2 PE=1 SV=2	LNRPLTLSEK
>	sp|Q9ER34|AC	Aconitate hydratase, mitochondrial OS=Rattus norvegicus GN=Aco2 PE=1 SV=2	NAVTQEFGPVPDTAR
>	sp|Q9ER34|AC	Aconitate hydratase, mitochondrial OS=Rattus norvegicus GN=Aco2 PE=1 SV=2	NDANPETHAFVTSPEIVTALAIAGTLK
>	sp|Q9ER34|AC	Aconitate hydratase, mitochondrial OS=Rattus norvegicus GN=Aco2 PE=1 SV=2	QALAHGLK
>	sp|Q9ER34|AC	Aconitate hydratase, mitochondrial OS=Rattus norvegicus GN=Aco2 PE=1 SV=2	VAMQDATAQMAMLQFISSGLPK
>	sp|Q9ER34|AC	Aconitate hydratase, mitochondrial OS=Rattus norvegicus GN=Aco2 PE=1 SV=2	VAMSHFEPSEYIR
>	sp|Q9ER34|AC	Aconitate hydratase, mitochondrial OS=Rattus norvegicus GN=Aco2 PE=1 SV=2	VAVPSTIHCDHLIEAQLGGEK
>	sp|Q9ER34|AC	Aconitate hydratase, mitochondrial OS=Rattus norvegicus GN=Aco2 PE=1 SV=2	VAVPSTIHCDHLIEAQLGGEKDLR
>	sp|Q9ER34|AC	Aconitate hydratase, mitochondrial OS=Rattus norvegicus GN=Aco2 PE=1 SV=2	WVVIGDENYGEGSSR
>	sp|Q9ER34|AC	Aconitate hydratase, mitochondrial OS=Rattus norvegicus GN=Aco2 PE=1 SV=2	LTGTLSGWTSPKDVILKVAGILTVK
>	sp|Q9ER34|AC	Aconitate hydratase, mitochondrial OS=Rattus norvegicus GN=Aco2 PE=1 SV=2	DINQEVYNFLATAGAK
>	sp|Q9ER34|AC	Aconitate hydratase, mitochondrial OS=Rattus norvegicus GN=Aco2 PE=1 SV=2	AKDINQEVYNFLATAGAK
>	sp|Q9ER34|AC	Aconitate hydratase, mitochondrial OS=Rattus norvegicus GN=Aco2 PE=1 SV=2	AKDINQEVYNFLATAGAK
>	sp|Q9ER34|AC	Aconitate hydratase, mitochondrial OS=Rattus norvegicus GN=Aco2 PE=1 SV=2	AKDINQEVYNFLATAGAK
>	sp|Q9ER34|AC	Aconitate hydratase, mitochondrial OS=Rattus norvegicus GN=Aco2 PE=1 SV=2	AKDINQEVYNFLATAGAK
>	sp|Q9ER34|AC	Aconitate hydratase, mitochondrial OS=Rattus norvegicus GN=Aco2 PE=1 SV=2	AKDINQEVYNFLATAGAK
>	sp|Q9ER34|AC	Aconitate hydratase, mitochondrial OS=Rattus norvegicus GN=Aco2 PE=1 SV=2	AKDINQEVYNFLATAGAK
>	sp|Q9ER34|AC	Aconitate hydratase, mitochondrial OS=Rattus norvegicus GN=Aco2 PE=1 SV=2	AKDINQEVYNFLATAGAK
>	sp|Q9ER34|AC	Aconitate hydratase, mitochondrial OS=Rattus norvegicus GN=Aco2 PE=1 SV=2	FKLEAPDADELPR
>	sp|Q9ER34|AC	Aconitate hydratase, mitochondrial OS=Rattus norvegicus GN=Aco2 PE=1 SV=2	GHLDNISNNLLIGAINIENGK
>	sp|Q9ER34|AC	Aconitate hydratase, mitochondrial OS=Rattus norvegicus GN=Aco2 PE=1 SV=2	GHLDNISNNLLIGAINIENGK
>	sp|Q9ER34|AC	Aconitate hydratase, mitochondrial OS=Rattus norvegicus GN=Aco2 PE=1 SV=2	GHLDNISNNLLIGAINIENGK
>	sp|Q9ER34|AC	Aconitate hydratase, mitochondrial OS=Rattus norvegicus GN=Aco2 PE=1 SV=2	GHLDNISNNLLIGAINIENGK
>	sp|Q9ER34|AC	Aconitate hydratase, mitochondrial OS=Rattus norvegicus GN=Aco2 PE=1 SV=2	IVYGHLDDPANQEIER
>	sp|Q9ER34|AC	Aconitate hydratase, mitochondrial OS=Rattus norvegicus GN=Aco2 PE=1 SV=2	IVYGHLDDPANQEIER
>	sp|Q9ER34|AC	Aconitate hydratase, mitochondrial OS=Rattus norvegicus GN=Aco2 PE=1 SV=2	IVYGHLDDPANQEIER
>	sp|Q9ER34|AC	Aconitate hydratase, mitochondrial OS=Rattus norvegicus GN=Aco2 PE=1 SV=2	IVYGHLDDPANQEIER
>	sp|Q9ER34|AC	Aconitate hydratase, mitochondrial OS=Rattus norvegicus GN=Aco2 PE=1 SV=2	IVYGHLDDPANQEIER
>	sp|Q9ER34|AC	Aconitate hydratase, mitochondrial OS=Rattus norvegicus GN=Aco2 PE=1 SV=2	IVYGHLDDPANQEIER
>	sp|Q9ER34|AC	Aconitate hydratase, mitochondrial OS=Rattus norvegicus GN=Aco2 PE=1 SV=2	IVYGHLDDPANQEIER
>	sp|Q9ER34|AC	Aconitate hydratase, mitochondrial OS=Rattus norvegicus GN=Aco2 PE=1 SV=2	LNRPLTLSEK
>	sp|Q9ER34|AC	Aconitate hydratase, mitochondrial OS=Rattus norvegicus GN=Aco2 PE=1 SV=2	NAVTQEFGPVPDTAR
>	sp|Q9ER34|AC	Aconitate hydratase, mitochondrial OS=Rattus norvegicus GN=Aco2 PE=1 SV=2	NAVTQEFGPVPDTAR
>	sp|Q9ER34|AC	Aconitate hydratase, mitochondrial OS=Rattus norvegicus GN=Aco2 PE=1 SV=2	NAVTQEFGPVPDTAR
>	sp|Q9ER34|AC	Aconitate hydratase, mitochondrial OS=Rattus norvegicus GN=Aco2 PE=1 SV=2	NDANPETHAFVTSPEIVTALAIAGTLK
>	sp|Q9ER34|AC	Aconitate hydratase, mitochondrial OS=Rattus norvegicus GN=Aco2 PE=1 SV=2	NDANPETHAFVTSPEIVTALAIAGTLK
>	sp|Q9ER34|AC	Aconitate hydratase, mitochondrial OS=Rattus norvegicus GN=Aco2 PE=1 SV=2	NDANPETHAFVTSPEIVTALAIAGTLK
>	sp|Q9ER34|AC	Aconitate hydratase, mitochondrial OS=Rattus norvegicus GN=Aco2 PE=1 SV=2	NDANPETHAFVTSPEIVTALAIAGTLK
>	sp|Q9ER34|AC	Aconitate hydratase, mitochondrial OS=Rattus norvegicus GN=Aco2 PE=1 SV=2	NDANPETHAFVTSPEIVTALAIAGTLK
>	sp|Q9ER34|AC	Aconitate hydratase, mitochondrial OS=Rattus norvegicus GN=Aco2 PE=1 SV=2	NDANPETHAFVTSPEIVTALAIAGTLK
>	sp|Q9ER34|AC	Aconitate hydratase, mitochondrial OS=Rattus norvegicus GN=Aco2 PE=1 SV=2	NDANPETHAFVTSPEIVTALAIAGTLK
>	sp|Q9ER34|AC	Aconitate hydratase, mitochondrial OS=Rattus norvegicus GN=Aco2 PE=1 SV=2	NDANPETHAFVTSPEIVTALAIAGTLK
>	sp|Q9ER34|AC	Aconitate hydratase, mitochondrial OS=Rattus norvegicus GN=Aco2 PE=1 SV=2	NDANPETHAFVTSPEIVTALAIAGTLK
>	sp|Q9ER34|AC	Aconitate hydratase, mitochondrial OS=Rattus norvegicus GN=Aco2 PE=1 SV=2	NDANPETHAFVTSPEIVTALAIAGTLK
>	sp|Q9ER34|AC	Aconitate hydratase, mitochondrial OS=Rattus norvegicus GN=Aco2 PE=1 SV=2	NDANPETHAFVTSPEIVTALAIAGTLK
>	sp|Q9ER34|AC	Aconitate hydratase, mitochondrial OS=Rattus norvegicus GN=Aco2 PE=1 SV=2	NDANPETHAFVTSPEIVTALAIAGTLK
>	sp|Q9ER34|AC	Aconitate hydratase, mitochondrial OS=Rattus norvegicus GN=Aco2 PE=1 SV=2	NDANPETHAFVTSPEIVTALAIAGTLK
>	sp|Q9ER34|AC	Aconitate hydratase, mitochondrial OS=Rattus norvegicus GN=Aco2 PE=1 SV=2	VAMQDATAQMAMLQFISSGLPK
>	sp|Q9ER34|AC	Aconitate hydratase, mitochondrial OS=Rattus norvegicus GN=Aco2 PE=1 SV=2	VAMQDATAQMAMLQFISSGLPK
>	sp|Q9ER34|AC	Aconitate hydratase, mitochondrial OS=Rattus norvegicus GN=Aco2 PE=1 SV=2	VAMQDATAQMAMLQFISSGLPK
>	sp|Q9ER34|AC	Aconitate hydratase, mitochondrial OS=Rattus norvegicus GN=Aco2 PE=1 SV=2	VAMQDATAQMAMLQFISSGLPK
>	sp|Q9ER34|AC	Aconitate hydratase, mitochondrial OS=Rattus norvegicus GN=Aco2 PE=1 SV=2	VAMSHFEPSEYIR
>	sp|Q9ER34|AC	Aconitate hydratase, mitochondrial OS=Rattus norvegicus GN=Aco2 PE=1 SV=2	VAMSHFEPSEYIR
>	sp|Q9ER34|AC	Aconitate hydratase, mitochondrial OS=Rattus norvegicus GN=Aco2 PE=1 SV=2	VAVPSTIHCDHLIEAQLGGEK
>	sp|Q9ER34|AC	Aconitate hydratase, mitochondrial OS=Rattus norvegicus GN=Aco2 PE=1 SV=2	VAVPSTIHCDHLIEAQLGGEKDLR
>	sp|Q9ER34|AC	Aconitate hydratase, mitochondrial OS=Rattus norvegicus GN=Aco2 PE=1 SV=2	WVVIGDENYGEGSSR
>	sp|Q9ER34|AC	Aconitate hydratase, mitochondrial OS=Rattus norvegicus GN=Aco2 PE=1 SV=2	FNPETDFLTGK
>	sp|Q9ER34|AC	Aconitate hydratase, mitochondrial OS=Rattus norvegicus GN=Aco2 PE=1 SV=2	VAMSHFEPSEYIR
>	sp|Q9ER34|AC	Aconitate hydratase, mitochondrial OS=Rattus norvegicus GN=Aco2 PE=1 SV=2	NAVTQEFGPVPDTAR
>	sp|Q9ER34|AC	Aconitate hydratase, mitochondrial OS=Rattus norvegicus GN=Aco2 PE=1 SV=2	IVYGHLDDPANQEIER
>	sp|Q9ER34|AC	Aconitate hydratase, mitochondrial OS=Rattus norvegicus GN=Aco2 PE=1 SV=2	FKLEAPDADELPR
>	sp|Q9ER34|AC	Aconitate hydratase, mitochondrial OS=Rattus norvegicus GN=Aco2 PE=1 SV=2	DINQEVYNFLATAGAK
>	sp|Q9ER34|AC	Aconitate hydratase, mitochondrial OS=Rattus norvegicus GN=Aco2 PE=1 SV=2	GHLDNISNNLLIGAINIENGK
>	sp|Q9ER34|AC	Aconitate hydratase, mitochondrial OS=Rattus norvegicus GN=Aco2 PE=1 SV=2	VAMQDATAQMAMLQFISSGLPK
>	sp|Q9ER34|AC	Aconitate hydratase, mitochondrial OS=Rattus norvegicus GN=Aco2 PE=1 SV=2	SQFTITPGSEQIR
>	sp|Q9ER34|AC	Aconitate hydratase, mitochondrial OS=Rattus norvegicus GN=Aco2 PE=1 SV=2	GHLDNISNNLLIGAINIENGK
>	sp|Q9ER34|AC	Aconitate hydratase, mitochondrial OS=Rattus norvegicus GN=Aco2 PE=1 SV=2	SQFTITPGSEQIR
>	sp|Q9ER34|AC	Aconitate hydratase, mitochondrial OS=Rattus norvegicus GN=Aco2 PE=1 SV=2	LTIQGLK
>	sp|Q9ER34|AC	Aconitate hydratase, mitochondrial OS=Rattus norvegicus GN=Aco2 PE=1 SV=2	FKLEAPDADELPR
>	sp|Q9ER34|AC	Aconitate hydratase, mitochondrial OS=Rattus norvegicus GN=Aco2 PE=1 SV=2	SQFTITPGSEQIR
>	sp|Q9ER34|AC	Aconitate hydratase, mitochondrial OS=Rattus norvegicus GN=Aco2 PE=1 SV=2	IVYGHLDDPANQEIER
>	sp|Q9ER34|AC	Aconitate hydratase, mitochondrial OS=Rattus norvegicus GN=Aco2 PE=1 SV=2	VAGILTVK

>	sp|Q9ER34|AC	Aconitate hydratase, mitochondrial OS=Rattus norvegicus GN=Aco2 PE=1 SV=2	GHLDNISNNLLIGAINIENGK
>	sp|Q9ER34|AC	Aconitate hydratase, mitochondrial OS=Rattus norvegicus GN=Aco2 PE=1 SV=2	LQLLEPFDKWDGKDLEDLQILIK
>	sp|Q9ER34|AC	Aconitate hydratase, mitochondrial OS=Rattus norvegicus GN=Aco2 PE=1 SV=2	IVYGHLDDPANQEIER
>	sp|Q9ER34|AC	Aconitate hydratase, mitochondrial OS=Rattus norvegicus GN=Aco2 PE=1 SV=2	FKLEAPDADELPR
>	sp|Q9ER34|AC	Aconitate hydratase, mitochondrial OS=Rattus norvegicus GN=Aco2 PE=1 SV=2	FKLEAPDADELPR
>	sp|Q9ER34|AC	Aconitate hydratase, mitochondrial OS=Rattus norvegicus GN=Aco2 PE=1 SV=2	SQFTITPGSEQIR
>	sp|Q9ER34|AC	Aconitate hydratase, mitochondrial OS=Rattus norvegicus GN=Aco2 PE=1 SV=2	HPNGTQETILLNHTFNETQIEWFR
>	sp|Q9ER34|AC	Aconitate hydratase, mitochondrial OS=Rattus norvegicus GN=Aco2 PE=1 SV=2	AKDINQEVYNFLATAGAK
>	sp|Q9ER34|AC	Aconitate hydratase, mitochondrial OS=Rattus norvegicus GN=Aco2 PE=1 SV=2	LTGTLSGWTSPKDVILKVAGILTVK
>	sp|Q9ER34|AC	Aconitate hydratase, mitochondrial OS=Rattus norvegicus GN=Aco2 PE=1 SV=2	AKDINQEVYNFLATAGAK
>	sp|Q9ER34|AC	Aconitate hydratase, mitochondrial OS=Rattus norvegicus GN=Aco2 PE=1 SV=2	FKLEAPDADELPR
>	sp|P19527|NFL	Neurofilament light polypeptide OS=Rattus norvegicus GN=Nefl PE=1 SV=3	ALYEQEIR
>	sp|P19527|NFL	Neurofilament light polypeptide OS=Rattus norvegicus GN=Nefl PE=1 SV=3	FASFIER
>	sp|P19527|NFL	Neurofilament light polypeptide OS=Rattus norvegicus GN=Nefl PE=1 SV=3	IDSLMDEIAFLK
>	sp|P19527|NFL	Neurofilament light polypeptide OS=Rattus norvegicus GN=Nefl PE=1 SV=3	KGADEAALAR
>	sp|P19527|NFL	Neurofilament light polypeptide OS=Rattus norvegicus GN=Nefl PE=1 SV=3	KLLEGEETR
>	sp|P19527|NFL	Neurofilament light polypeptide OS=Rattus norvegicus GN=Nefl PE=1 SV=3	KVHEEEIAELQAQIQ
>	sp|P19527|NFL	Neurofilament light polypeptide OS=Rattus norvegicus GN=Nefl PE=1 SV=3	KVHEEEIAELQAQIQYAQISVEMDVSSKPDLSAALK
>	sp|P19527|NFL	Neurofilament light polypeptide OS=Rattus norvegicus GN=Nefl PE=1 SV=3	KVHEEEIAELQAQIQYAQISVEMDVSSKPDLSAALKDIR
>	sp|P19527|NFL	Neurofilament light polypeptide OS=Rattus norvegicus GN=Nefl PE=1 SV=3	LLEGEETR
>	sp|P19527|NFL	Neurofilament light polypeptide OS=Rattus norvegicus GN=Nefl PE=1 SV=3	VHEEEIAELQAQIQYAQISVEMDVSSKPDLSAALK
>	sp|P19527|NFL	Neurofilament light polypeptide OS=Rattus norvegicus GN=Nefl PE=1 SV=3	VLEAELLVLR
>	sp|P19527|NFL	Neurofilament light polypeptide OS=Rattus norvegicus GN=Nefl PE=1 SV=3	MALDIEIAAYR
>	sp|P19527|NFL	Neurofilament light polypeptide OS=Rattus norvegicus GN=Nefl PE=1 SV=3	FTVLTESAAK
>	sp|P19527|NFL	Neurofilament light polypeptide OS=Rattus norvegicus GN=Nefl PE=1 SV=3	AAKDEVSESR
>	sp|P19527|NFL	Neurofilament light polypeptide OS=Rattus norvegicus GN=Nefl PE=1 SV=3	YEEEVLSREDAEGR
>	sp|P19527|NFL	Neurofilament light polypeptide OS=Rattus norvegicus GN=Nefl PE=1 SV=3	QLQELEDKQNADISAMQDTINKLENELR
>	sp|P19527|NFL	Neurofilament light polypeptide OS=Rattus norvegicus GN=Nefl PE=1 SV=3	RIDSLMDEIAFLK
>	sp|P19527|NFL	Neurofilament light polypeptide OS=Rattus norvegicus GN=Nefl PE=1 SV=3	AAKDEVSESR
>	sp|P19527|NFL	Neurofilament light polypeptide OS=Rattus norvegicus GN=Nefl PE=1 SV=3	IDSLMDEIAFLK
>	sp|P19527|NFL	Neurofilament light polypeptide OS=Rattus norvegicus GN=Nefl PE=1 SV=3	KLLEGEETR
>	sp|P19527|NFL	Neurofilament light polypeptide OS=Rattus norvegicus GN=Nefl PE=1 SV=3	KLLEGEETR
>	sp|P19527|NFL	Neurofilament light polypeptide OS=Rattus norvegicus GN=Nefl PE=1 SV=3	KLLEGEETR
>	sp|P19527|NFL	Neurofilament light polypeptide OS=Rattus norvegicus GN=Nefl PE=1 SV=3	KLLEGEETR
>	sp|P19527|NFL	Neurofilament light polypeptide OS=Rattus norvegicus GN=Nefl PE=1 SV=3	KVHEEEIAELQAQIQYAQISVEMDVSSKPDLSAALK
>	sp|P19527|NFL	Neurofilament light polypeptide OS=Rattus norvegicus GN=Nefl PE=1 SV=3	KVHEEEIAELQAQIQYAQISVEMDVSSKPDLSAALK
>	sp|P19527|NFL	Neurofilament light polypeptide OS=Rattus norvegicus GN=Nefl PE=1 SV=3	KVHEEEIAELQAQIQYAQISVEMDVSSKPDLSAALK
>	sp|P19527|NFL	Neurofilament light polypeptide OS=Rattus norvegicus GN=Nefl PE=1 SV=3	KVHEEEIAELQAQIQYAQISVEMDVSSKPDLSAALK
>	sp|P19527|NFL	Neurofilament light polypeptide OS=Rattus norvegicus GN=Nefl PE=1 SV=3	KVHEEEIAELQAQIQYAQISVEMDVSSKPDLSAALK
>	sp|P19527|NFL	Neurofilament light polypeptide OS=Rattus norvegicus GN=Nefl PE=1 SV=3	KVHEEEIAELQAQIQYAQISVEMDVSSKPDLSAALK
>	sp|P19527|NFL	Neurofilament light polypeptide OS=Rattus norvegicus GN=Nefl PE=1 SV=3	KVHEEEIAELQAQIQYAQISVEMDVSSKPDLSAALK
>	sp|P19527|NFL	Neurofilament light polypeptide OS=Rattus norvegicus GN=Nefl PE=1 SV=3	KVHEEEIAELQAQIQYAQISVEMDVSSKPDLSAALK
>	sp|P19527|NFL	Neurofilament light polypeptide OS=Rattus norvegicus GN=Nefl PE=1 SV=3	KVHEEEIAELQAQIQYAQISVEMDVSSKPDLSAALK
>	sp|P19527|NFL	Neurofilament light polypeptide OS=Rattus norvegicus GN=Nefl PE=1 SV=3	KVHEEEIAELQAQIQYAQISVEMDVSSKPDLSAALK
>	sp|P19527|NFL	Neurofilament light polypeptide OS=Rattus norvegicus GN=Nefl PE=1 SV=3	KVHEEEIAELQAQIQYAQISVEMDVSSKPDLSAALK
>	sp|P19527|NFL	Neurofilament light polypeptide OS=Rattus norvegicus GN=Nefl PE=1 SV=3	KVHEEEIAELQAQIQYAQISVEMDVSSKPDLSAALK
>	sp|P19527|NFL	Neurofilament light polypeptide OS=Rattus norvegicus GN=Nefl PE=1 SV=3	KVHEEEIAELQAQIQYAQISVEMDVSSKPDLSAALK
>	sp|P19527|NFL	Neurofilament light polypeptide OS=Rattus norvegicus GN=Nefl PE=1 SV=3	KVHEEEIAELQAQIQYAQISVEMDVSSKPDLSAALKDIR
>	sp|P19527|NFL	Neurofilament light polypeptide OS=Rattus norvegicus GN=Nefl PE=1 SV=3	KVHEEEIAELQAQIQYAQISVEMDVSSKPDLSAALKDIR
>	sp|P19527|NFL	Neurofilament light polypeptide OS=Rattus norvegicus GN=Nefl PE=1 SV=3	LLEGEETR
>	sp|P19527|NFL	Neurofilament light polypeptide OS=Rattus norvegicus GN=Nefl PE=1 SV=3	QLQELEDKQNADISAMQDTINKLENELR
>	sp|P19527|NFL	Neurofilament light polypeptide OS=Rattus norvegicus GN=Nefl PE=1 SV=3	RIDSLMDEIAFLK
>	sp|P19527|NFL	Neurofilament light polypeptide OS=Rattus norvegicus GN=Nefl PE=1 SV=3	RIDSLMDEIAFLK
>	sp|P19527|NFL	Neurofilament light polypeptide OS=Rattus norvegicus GN=Nefl PE=1 SV=3	RIDSLMDEIAFLK
>	sp|P19527|NFL	Neurofilament light polypeptide OS=Rattus norvegicus GN=Nefl PE=1 SV=3	RIDSLMDEIAFLK
>	sp|P19527|NFL	Neurofilament light polypeptide OS=Rattus norvegicus GN=Nefl PE=1 SV=3	VHEEEIAELQAQIQYAQISVEMDVSSKPDLSAALK
>	sp|P19527|NFL	Neurofilament light polypeptide OS=Rattus norvegicus GN=Nefl PE=1 SV=3	VHEEEIAELQAQIQYAQISVEMDVSSKPDLSAALK
>	sp|P19527|NFL	Neurofilament light polypeptide OS=Rattus norvegicus GN=Nefl PE=1 SV=3	VHEEEIAELQAQIQYAQISVEMDVSSKPDLSAALK
>	sp|P19527|NFL	Neurofilament light polypeptide OS=Rattus norvegicus GN=Nefl PE=1 SV=3	VHEEEIAELQAQIQYAQISVEMDVSSKPDLSAALK
>	sp|P19527|NFL	Neurofilament light polypeptide OS=Rattus norvegicus GN=Nefl PE=1 SV=3	VHEEEIAELQAQIQYAQISVEMDVSSKPDLSAALK
>	sp|P19527|NFL	Neurofilament light polypeptide OS=Rattus norvegicus GN=Nefl PE=1 SV=3	VHEEEIAELQAQIQYAQISVEMDVSSKPDLSAALK
>	sp|P19527|NFL	Neurofilament light polypeptide OS=Rattus norvegicus GN=Nefl PE=1 SV=3	VHEEEIAELQAQIQYAQISVEMDVSSKPDLSAALK
>	sp|P19527|NFL	Neurofilament light polypeptide OS=Rattus norvegicus GN=Nefl PE=1 SV=3	EGLEETLR
>	sp|P19527|NFL	Neurofilament light polypeptide OS=Rattus norvegicus GN=Nefl PE=1 SV=3	AAKDEVSESR
>	sp|P19527|NFL	Neurofilament light polypeptide OS=Rattus norvegicus GN=Nefl PE=1 SV=3	RIDSLMDEIAFLK
>	sp|P19527|NFL	Neurofilament light polypeptide OS=Rattus norvegicus GN=Nefl PE=1 SV=3	EYQDLLNVK
>	sp|P19527|NFL	Neurofilament light polypeptide OS=Rattus norvegicus GN=Nefl PE=1 SV=3	LAAEDATNEK
>	sp|P19527|NFL	Neurofilament light polypeptide OS=Rattus norvegicus GN=Nefl PE=1 SV=3	IDSLMDEIAFLK
>	sp|P19527|NFL	Neurofilament light polypeptide OS=Rattus norvegicus GN=Nefl PE=1 SV=3	LLEGEETR
>	sp|P19527|NFL	Neurofilament light polypeptide OS=Rattus norvegicus GN=Nefl PE=1 SV=3	AAKDEVSESR
>	sp|P19527|NFL	Neurofilament light polypeptide OS=Rattus norvegicus GN=Nefl PE=1 SV=3	AAKDEVSESR
>	sp|P19527|NFL	Neurofilament light polypeptide OS=Rattus norvegicus GN=Nefl PE=1 SV=3	LLEGEETR
>	sp|P19527|NFL	Neurofilament light polypeptide OS=Rattus norvegicus GN=Nefl PE=1 SV=3	LLEGEETR
>	sp|P19527|NFL	Neurofilament light polypeptide OS=Rattus norvegicus GN=Nefl PE=1 SV=3	RIDSLMDEIAFLK

>	sp|P19527|NFL Neurofilament light polypeptide OS=Rattus norvegicus GN=Nefl PE=1 SV=3	QLQELEDKQNADISAMQDTINKLENELR
>	sp|P19527|NFL Neurofilament light polypeptide OS=Rattus norvegicus GN=Nefl PE=1 SV=3	NMQNAEEWFK
>	sp|P19527|NFL Neurofilament light polypeptide OS=Rattus norvegicus GN=Nefl PE=1 SV=3	LLEGEETR
>	sp|P19527|NFL Neurofilament light polypeptide OS=Rattus norvegicus GN=Nefl PE=1 SV=3	FTVLTESAAK
>	sp|P19527|NFL Neurofilament light polypeptide OS=Rattus norvegicus GN=Nefl PE=1 SV=3	VHELEQQNK
>	sp|P19527|NFL Neurofilament light polypeptide OS=Rattus norvegicus GN=Nefl PE=1 SV=3	VHELEQQNK
>	sp|P19527|NFL Neurofilament light polypeptide OS=Rattus norvegicus GN=Nefl PE=1 SV=3	KVHEEEIAELQAQIQYAQISVEMDVSSKPDLSAALKDIR
>	sp|P19527|NFL Neurofilament light polypeptide OS=Rattus norvegicus GN=Nefl PE=1 SV=3	QLQELEDKQNADISAMQDTINKLENELR
>	sp|P19527|NFL Neurofilament light polypeptide OS=Rattus norvegicus GN=Nefl PE=1 SV=3	QALQGEREGLEETLR
>	sp|P19527|NFL Neurofilament light polypeptide OS=Rattus norvegicus GN=Nefl PE=1 SV=3	VHEEEIAELQAQIQYAQISVEMDVSSKPDLSAALK
>	sp|P19527|NFL Neurofilament light polypeptide OS=Rattus norvegicus GN=Nefl PE=1 SV=3	FTVLTESAAK
>	sp|P19527|NFL Neurofilament light polypeptide OS=Rattus norvegicus GN=Nefl PE=1 SV=3	KVHEEEIAELQAQIQYAQISVEMDVSSKPDLSAALK
>	sp|P19527|NFL Neurofilament light polypeptide OS=Rattus norvegicus GN=Nefl PE=1 SV=3	QLQELEDKQNADISAMQDTINKLENELR
>	sp|P19527|NFL Neurofilament light polypeptide OS=Rattus norvegicus GN=Nefl PE=1 SV=3	QLQELEDKQNADISAMQDTINKLENELR
>	sp|P19527|NFL Neurofilament light polypeptide OS=Rattus norvegicus GN=Nefl PE=1 SV=3	AAKDEVSESR
>	sp|P19527|NFL Neurofilament light polypeptide OS=Rattus norvegicus GN=Nefl PE=1 SV=3	AAKDEVSESR
>	sp|P19527|NFL Neurofilament light polypeptide OS=Rattus norvegicus GN=Nefl PE=1 SV=3	LLEGEETR
>	sp|P19527|NFL Neurofilament light polypeptide OS=Rattus norvegicus GN=Nefl PE=1 SV=3	MALDIEIAAYR
>	sp|P02770|ALB Serum albumin OS=Rattus norvegicus GN=Alb PE=1 SV=2	AADKDNCFATEGPNLVAR
>	sp|P02770|ALB Serum albumin OS=Rattus norvegicus GN=Alb PE=1 SV=2	APQVSTPTLVEAAR
>	sp|P02770|ALB Serum albumin OS=Rattus norvegicus GN=Alb PE=1 SV=2	DVFLGTFLYEYSR
>	sp|P02770|ALB Serum albumin OS=Rattus norvegicus GN=Alb PE=1 SV=2	ECCHGDLLECADDRAELAK
>	sp|P02770|ALB Serum albumin OS=Rattus norvegicus GN=Alb PE=1 SV=2	FKDLGEQHFK
>	sp|P02770|ALB Serum albumin OS=Rattus norvegicus GN=Alb PE=1 SV=2	GLVLIAFSQYLQK
>	sp|P02770|ALB Serum albumin OS=Rattus norvegicus GN=Alb PE=1 SV=2	INKECCHGDLLECADDRAELAK
>	sp|P02770|ALB Serum albumin OS=Rattus norvegicus GN=Alb PE=1 SV=2	LPCVEDYLSAILNR
>	sp|P02770|ALB Serum albumin OS=Rattus norvegicus GN=Alb PE=1 SV=2	LRDNYGELADCCAK
>	sp|P02770|ALB Serum albumin OS=Rattus norvegicus GN=Alb PE=1 SV=2	LVQEVTDFAK
>	sp|P02770|ALB Serum albumin OS=Rattus norvegicus GN=Alb PE=1 SV=2	QTALAELVK
>	sp|P02770|ALB Serum albumin OS=Rattus norvegicus GN=Alb PE=1 SV=2	RPCFSALTVDETYVPK
>	sp|P02770|ALB Serum albumin OS=Rattus norvegicus GN=Alb PE=1 SV=2	SQCLAEIEHDNIPADLPSIAADFVEDKEVCK
>	sp|P02770|ALB Serum albumin OS=Rattus norvegicus GN=Alb PE=1 SV=2	TCVADENAENCDK
>	sp|P02770|ALB Serum albumin OS=Rattus norvegicus GN=Alb PE=1 SV=2	TVMGDFAQFVDK
>	sp|P02770|ALB Serum albumin OS=Rattus norvegicus GN=Alb PE=1 SV=2	RHPDYSVSLLLR
>	sp|P02770|ALB Serum albumin OS=Rattus norvegicus GN=Alb PE=1 SV=2	YMCENQATISSK
>	sp|P02770|ALB Serum albumin OS=Rattus norvegicus GN=Alb PE=1 SV=2	FPNAEFAEITK
>	sp|P02770|ALB Serum albumin OS=Rattus norvegicus GN=Alb PE=1 SV=2	EFKAETFTFHSDICTLPDKEK
>	sp|P02770|ALB Serum albumin OS=Rattus norvegicus GN=Alb PE=1 SV=2	LGEYGFQNAVLVR
>	sp|P02770|ALB Serum albumin OS=Rattus norvegicus GN=Alb PE=1 SV=2	AADKDNCFATEGPNLVAR
>	sp|P02770|ALB Serum albumin OS=Rattus norvegicus GN=Alb PE=1 SV=2	AADKDNCFATEGPNLVAR
>	sp|P02770|ALB Serum albumin OS=Rattus norvegicus GN=Alb PE=1 SV=2	AADKDNCFATEGPNLVAR
>	sp|P02770|ALB Serum albumin OS=Rattus norvegicus GN=Alb PE=1 SV=2	AADKDNCFATEGPNLVAR
>	sp|P02770|ALB Serum albumin OS=Rattus norvegicus GN=Alb PE=1 SV=2	AADKDNCFATEGPNLVAR
>	sp|P02770|ALB Serum albumin OS=Rattus norvegicus GN=Alb PE=1 SV=2	AADKDNCFATEGPNLVAR
>	sp|P02770|ALB Serum albumin OS=Rattus norvegicus GN=Alb PE=1 SV=2	AADKDNCFATEGPNLVAR
>	sp|P02770|ALB Serum albumin OS=Rattus norvegicus GN=Alb PE=1 SV=2	APQVSTPTLVEAAR
>	sp|P02770|ALB Serum albumin OS=Rattus norvegicus GN=Alb PE=1 SV=2	DVFLGTFLYEYSR
>	sp|P02770|ALB Serum albumin OS=Rattus norvegicus GN=Alb PE=1 SV=2	DVFLGTFLYEYSR
>	sp|P02770|ALB Serum albumin OS=Rattus norvegicus GN=Alb PE=1 SV=2	DVFLGTFLYEYSR
>	sp|P02770|ALB Serum albumin OS=Rattus norvegicus GN=Alb PE=1 SV=2	DVFLGTFLYEYSR
>	sp|P02770|ALB Serum albumin OS=Rattus norvegicus GN=Alb PE=1 SV=2	DVFLGTFLYEYSR
>	sp|P02770|ALB Serum albumin OS=Rattus norvegicus GN=Alb PE=1 SV=2	ECCHGDLLECADDRAELAK
>	sp|P02770|ALB Serum albumin OS=Rattus norvegicus GN=Alb PE=1 SV=2	ECCHGDLLECADDRAELAK
>	sp|P02770|ALB Serum albumin OS=Rattus norvegicus GN=Alb PE=1 SV=2	GLVLIAFSQYLQK
>	sp|P02770|ALB Serum albumin OS=Rattus norvegicus GN=Alb PE=1 SV=2	GLVLIAFSQYLQK
>	sp|P02770|ALB Serum albumin OS=Rattus norvegicus GN=Alb PE=1 SV=2	GLVLIAFSQYLQK
>	sp|P02770|ALB Serum albumin OS=Rattus norvegicus GN=Alb PE=1 SV=2	GLVLIAFSQYLQK
>	sp|P02770|ALB Serum albumin OS=Rattus norvegicus GN=Alb PE=1 SV=2	GLVLIAFSQYLQK
>	sp|P02770|ALB Serum albumin OS=Rattus norvegicus GN=Alb PE=1 SV=2	GLVLIAFSQYLQK
>	sp|P02770|ALB Serum albumin OS=Rattus norvegicus GN=Alb PE=1 SV=2	GLVLIAFSQYLQK
>	sp|P02770|ALB Serum albumin OS=Rattus norvegicus GN=Alb PE=1 SV=2	GLVLIAFSQYLQK
>	sp|P02770|ALB Serum albumin OS=Rattus norvegicus GN=Alb PE=1 SV=2	GLVLIAFSQYLQK
>	sp|P02770|ALB Serum albumin OS=Rattus norvegicus GN=Alb PE=1 SV=2	GLVLIAFSQYLQK
>	sp|P02770|ALB Serum albumin OS=Rattus norvegicus GN=Alb PE=1 SV=2	GLVLIAFSQYLQKCPYEEHIK
>	sp|P02770|ALB Serum albumin OS=Rattus norvegicus GN=Alb PE=1 SV=2	INKECCHGDLLECADDRAELAK
>	sp|P02770|ALB Serum albumin OS=Rattus norvegicus GN=Alb PE=1 SV=2	LVQEVTDFAK
>	sp|P02770|ALB Serum albumin OS=Rattus norvegicus GN=Alb PE=1 SV=2	LVQEVTDFAK
>	sp|P02770|ALB Serum albumin OS=Rattus norvegicus GN=Alb PE=1 SV=2	RPCFSALTVDETYVPK
>	sp|P02770|ALB Serum albumin OS=Rattus norvegicus GN=Alb PE=1 SV=2	SQCLAEIEHDNIPADLPSIAADFVEDKEVCK
>	sp|P02770|ALB Serum albumin OS=Rattus norvegicus GN=Alb PE=1 SV=2	SQCLAEIEHDNIPADLPSIAADFVEDKEVCK
>	sp|P02770|ALB Serum albumin OS=Rattus norvegicus GN=Alb PE=1 SV=2	SQCLAEIEHDNIPADLPSIAADFVEDKEVCK
>	sp|P02770|ALB Serum albumin OS=Rattus norvegicus GN=Alb PE=1 SV=2	TCVADENAENCDK
>	sp|P02770|ALB Serum albumin OS=Rattus norvegicus GN=Alb PE=1 SV=2	TCVADENAENCDK
>	sp|P02770|ALB Serum albumin OS=Rattus norvegicus GN=Alb PE=1 SV=2	TCVADENAENCDK

>	sp|P02770|ALB	Serum albumin OS=Rattus norvegicus GN=Alb PE=1 SV=2	TVMGDFAQFVDK
>	sp|P02770|ALB	Serum albumin OS=Rattus norvegicus GN=Alb PE=1 SV=2	TVMGDFAQFVDK
>	sp|P02770|ALB	Serum albumin OS=Rattus norvegicus GN=Alb PE=1 SV=2	TCVADENAENCDK
>	sp|P02770|ALB	Serum albumin OS=Rattus norvegicus GN=Alb PE=1 SV=2	TVMGDFAQFVDK
>	sp|P02770|ALB	Serum albumin OS=Rattus norvegicus GN=Alb PE=1 SV=2	YMCENQATISSK
>	sp|P02770|ALB	Serum albumin OS=Rattus norvegicus GN=Alb PE=1 SV=2	QTALAELVK
>	sp|P02770|ALB	Serum albumin OS=Rattus norvegicus GN=Alb PE=1 SV=2	INKECCHGDLLECADDRAELAK
>	sp|P02770|ALB	Serum albumin OS=Rattus norvegicus GN=Alb PE=1 SV=2	LVQEVTDFAK
>	sp|P02770|ALB	Serum albumin OS=Rattus norvegicus GN=Alb PE=1 SV=2	DVFLGTFLYEYSR
>	sp|P02770|ALB	Serum albumin OS=Rattus norvegicus GN=Alb PE=1 SV=2	RHPYFYAPELLYYAEK
>	sp|P02770|ALB	Serum albumin OS=Rattus norvegicus GN=Alb PE=1 SV=2	LVQEVTDFAK
>	sp|P02770|ALB	Serum albumin OS=Rattus norvegicus GN=Alb PE=1 SV=2	ALVAAVR
>	sp|P02770|ALB	Serum albumin OS=Rattus norvegicus GN=Alb PE=1 SV=2	LVQEVTDFAK
>	sp|P02770|ALB	Serum albumin OS=Rattus norvegicus GN=Alb PE=1 SV=2	ALVAAVR
>	sp|P02770|ALB	Serum albumin OS=Rattus norvegicus GN=Alb PE=1 SV=2	FKDLGEQHFKGLVLIAFSQYLQK
>	sp|P02770|ALB	Serum albumin OS=Rattus norvegicus GN=Alb PE=1 SV=2	ECCHGDLLECADDRAELAK
>	sp|P02770|ALB	Serum albumin OS=Rattus norvegicus GN=Alb PE=1 SV=2	AADKDNCFATEGPNLVAR
>	sp|P02770|ALB	Serum albumin OS=Rattus norvegicus GN=Alb PE=1 SV=2	FPNAEFAEITK
>	sp|P02770|ALB	Serum albumin OS=Rattus norvegicus GN=Alb PE=1 SV=2	TVMGDFAQFVDK
>	sp|P02770|ALB	Serum albumin OS=Rattus norvegicus GN=Alb PE=1 SV=2	LRDNYGELADCCAK
>	sp|P02770|ALB	Serum albumin OS=Rattus norvegicus GN=Alb PE=1 SV=2	YMCENQATISSK
>	sp|P02770|ALB	Serum albumin OS=Rattus norvegicus GN=Alb PE=1 SV=2	LVQEVTDFAK
>	sp|P02770|ALB	Serum albumin OS=Rattus norvegicus GN=Alb PE=1 SV=2	RPCFSALTVDETYVPK
>	sp|P02770|ALB	Serum albumin OS=Rattus norvegicus GN=Alb PE=1 SV=2	ECCHGDLLECADDRAELAK
>	sp|P02770|ALB	Serum albumin OS=Rattus norvegicus GN=Alb PE=1 SV=2	LQACCDKPVLQK
>	sp|P02770|ALB	Serum albumin OS=Rattus norvegicus GN=Alb PE=1 SV=2	FKDLGEQHFK
>	sp|P02770|ALB	Serum albumin OS=Rattus norvegicus GN=Alb PE=1 SV=2	FPNAEFAEITK
>	sp|P02770|ALB	Serum albumin OS=Rattus norvegicus GN=Alb PE=1 SV=2	QEPERNECFLQHK
>	sp|P02770|ALB	Serum albumin OS=Rattus norvegicus GN=Alb PE=1 SV=2	RHPDYSVSLLLR
>	sp|P02770|ALB	Serum albumin OS=Rattus norvegicus GN=Alb PE=1 SV=2	LCVLHEK
>	sp|P02770|ALB	Serum albumin OS=Rattus norvegicus GN=Alb PE=1 SV=2	SQCLAEIEHDNIPADLPSIAADFVEDKEVCK
>	sp|P02770|ALB	Serum albumin OS=Rattus norvegicus GN=Alb PE=1 SV=2	SQCLAEIEHDNIPADLPSIAADFVEDKEVCK
>	sp|P02770|ALB	Serum albumin OS=Rattus norvegicus GN=Alb PE=1 SV=2	FPNAEFAEITK
>	sp|P02770|ALB	Serum albumin OS=Rattus norvegicus GN=Alb PE=1 SV=2	DVFLGTFLYEYSR
>	sp|P02770|ALB	Serum albumin OS=Rattus norvegicus GN=Alb PE=1 SV=2	ECCHGDLLECADDRAELAK
>	sp|P02770|ALB	Serum albumin OS=Rattus norvegicus GN=Alb PE=1 SV=2	RPCFSALTVDETYVPK
>	sp|P02770|ALB	Serum albumin OS=Rattus norvegicus GN=Alb PE=1 SV=2	QTALAELVK
>	sp|P02770|ALB	Serum albumin OS=Rattus norvegicus GN=Alb PE=1 SV=2	LQACCDKPVLQK
>	sp|P02770|ALB	Serum albumin OS=Rattus norvegicus GN=Alb PE=1 SV=2	DVFLGTFLYEYSR
>	sp|P02770|ALB	Serum albumin OS=Rattus norvegicus GN=Alb PE=1 SV=2	YMCENQATISSK
>	sp|P02770|ALB	Serum albumin OS=Rattus norvegicus GN=Alb PE=1 SV=2	SQCLAEIEHDNIPADLPSIAADFVEDKEVCK
>	sp|P02770|ALB	Serum albumin OS=Rattus norvegicus GN=Alb PE=1 SV=2	SQCLAEIEHDNIPADLPSIAADFVEDKEVCK
>	sp|P02770|ALB	Serum albumin OS=Rattus norvegicus GN=Alb PE=1 SV=2	QTALAELVK
>	sp|P02770|ALB	Serum albumin OS=Rattus norvegicus GN=Alb PE=1 SV=2	LQACCDKPVLQK
>	sp|P02770|ALB	Serum albumin OS=Rattus norvegicus GN=Alb PE=1 SV=2	SIHTLFGDK
>	sp|P02770|ALB	Serum albumin OS=Rattus norvegicus GN=Alb PE=1 SV=2	EFKAETFTFHSDICTLPDKEK
>	sp|P02770|ALB	Serum albumin OS=Rattus norvegicus GN=Alb PE=1 SV=2	APQVSTPTLVEAAR
>	sp|P02770|ALB	Serum albumin OS=Rattus norvegicus GN=Alb PE=1 SV=2	RPCFSALTVDETYVPK
>	sp|P02770|ALB	Serum albumin OS=Rattus norvegicus GN=Alb PE=1 SV=2	FKDLGEQHFKGLVLIAFSQYLQK
>	sp|Q05175|BAS	Brain acid soluble protein 1 OS=Rattus norvegicus GN=Basp1 PE=1 SV=2	AAAKEEAPKAEPEKSEGAAEEQPEPAPAPEQEAAAPGPAAGGEAPK
>	sp|Q05175|BAS	Brain acid soluble protein 1 OS=Rattus norvegicus GN=Basp1 PE=1 SV=2	AEPEKSEGAAEEQPEPAPAPEQEAAAPGPAAGGEAPK
>	sp|Q05175|BAS	Brain acid soluble protein 1 OS=Rattus norvegicus GN=Basp1 PE=1 SV=2	AGEASAESTGAADGAPQEEGEAK
>	sp|Q05175|BAS	Brain acid soluble protein 1 OS=Rattus norvegicus GN=Basp1 PE=1 SV=2	APAPAAPAAEPQAEAPVASSEQSVAVKE
>	sp|Q05175|BAS	Brain acid soluble protein 1 OS=Rattus norvegicus GN=Basp1 PE=1 SV=2	DAADGEAKAEEKEADKAAAK
>	sp|Q05175|BAS	Brain acid soluble protein 1 OS=Rattus norvegicus GN=Basp1 PE=1 SV=2	EADKAAAKEEAPKAEPEKSEGAAEEQPEPAPAPEQEAAAPGPAAGGEAPK
>	sp|Q05175|BAS	Brain acid soluble protein 1 OS=Rattus norvegicus GN=Basp1 PE=1 SV=2	EEAPKAEPEKSEGAAEEQPEPAPAPEQEAAAPGPAAGGEAPK
>	sp|Q05175|BAS	Brain acid soluble protein 1 OS=Rattus norvegicus GN=Basp1 PE=1 SV=2	ESEPQAAADATEVK
>	sp|Q05175|BAS	Brain acid soluble protein 1 OS=Rattus norvegicus GN=Basp1 PE=1 SV=2	ETPAASEAPSSAAK
>	sp|Q05175|BAS	Brain acid soluble protein 1 OS=Rattus norvegicus GN=Basp1 PE=1 SV=2	KAEGAGTEEEGTQK
>	sp|Q05175|BAS	Brain acid soluble protein 1 OS=Rattus norvegicus GN=Basp1 PE=1 SV=2	KAEGAGTEEEGTQKESEPQAAADATEVKESAEEKPK
>	sp|Q05175|BAS	Brain acid soluble protein 1 OS=Rattus norvegicus GN=Basp1 PE=1 SV=2	KTEAPAAGPEAK
>	sp|Q05175|BAS	Brain acid soluble protein 1 OS=Rattus norvegicus GN=Basp1 PE=1 SV=2	SDAAPAASDSKPSTEPAPSSK
>	sp|Q05175|BAS	Brain acid soluble protein 1 OS=Rattus norvegicus GN=Basp1 PE=1 SV=2	AEGAGTEEEGTQK
>	sp|Q05175|BAS	Brain acid soluble protein 1 OS=Rattus norvegicus GN=Basp1 PE=1 SV=2	ESEPQAAADATEVKESAEEKPK
>	sp|Q05175|BAS	Brain acid soluble protein 1 OS=Rattus norvegicus GN=Basp1 PE=1 SV=2	ESEPQAAADATEVKESAEEKPKDAADGEAK
>	sp|Q05175|BAS	Brain acid soluble protein 1 OS=Rattus norvegicus GN=Basp1 PE=1 SV=2	AEGAGTEEEGTQKESEPQAAADATEVKESAEEKPK
>	sp|Q05175|BAS	Brain acid soluble protein 1 OS=Rattus norvegicus GN=Basp1 PE=1 SV=2	KAEGAGTEEEGTQKESEPQAAADATEVK
>	sp|Q05175|BAS	Brain acid soluble protein 1 OS=Rattus norvegicus GN=Basp1 PE=1 SV=2	AAAKEEAPKAEPEKSEGAAEEQPEPAPAPEQEAAAPGPAAGGEAPK
>	sp|Q05175|BAS	Brain acid soluble protein 1 OS=Rattus norvegicus GN=Basp1 PE=1 SV=2	AAAKEEAPKAEPEKSEGAAEEQPEPAPAPEQEAAAPGPAAGGEAPK
>	sp|Q05175|BAS	Brain acid soluble protein 1 OS=Rattus norvegicus GN=Basp1 PE=1 SV=2	AAAKEEAPKAEPEKSEGAAEEQPEPAPAPEQEAAAPGPAAGGEAPK
>	sp|Q05175|BAS	Brain acid soluble protein 1 OS=Rattus norvegicus GN=Basp1 PE=1 SV=2	AAAKEEAPKAEPEKSEGAAEEQPEPAPAPEQEAAAPGPAAGGEAPK
>	sp|Q05175|BAS	Brain acid soluble protein 1 OS=Rattus norvegicus GN=Basp1 PE=1 SV=2	AAAKEEAPKAEPEKSEGAAEEQPEPAPAPEQEAAAPGPAAGGEAPK
>	sp|Q05175|BAS	Brain acid soluble protein 1 OS=Rattus norvegicus GN=Basp1 PE=1 SV=2	AAAKEEAPKAEPEKSEGAAEEQPEPAPAPEQEAAAPGPAAGGEAPK
>	sp|Q05175|BAS	Brain acid soluble protein 1 OS=Rattus norvegicus GN=Basp1 PE=1 SV=2	AAAKEEAPKAEPEKSEGAAEEQPEPAPAPEQEAAAPGPAAGGEAPK

>	sp|Q05175|BAS	Brain acid soluble protein 1 OS=Rattus norvegicus GN=Basp1 PE=1 SV=2	AAAKEEAPKAEPEKSEGAAEEQPEPAPAPEQEAAAPGPAAGGEAPK
>	sp|Q05175|BAS	Brain acid soluble protein 1 OS=Rattus norvegicus GN=Basp1 PE=1 SV=2	AAAKEEAPKAEPEKSEGAAEEQPEPAPAPEQEAAAPGPAAGGEAPK
>	sp|Q05175|BAS	Brain acid soluble protein 1 OS=Rattus norvegicus GN=Basp1 PE=1 SV=2	AAAKEEAPKAEPEKSEGAAEEQPEPAPAPEQEAAAPGPAAGGEAPK
>	sp|Q05175|BAS	Brain acid soluble protein 1 OS=Rattus norvegicus GN=Basp1 PE=1 SV=2	AAAKEEAPKAEPEKSEGAAEEQPEPAPAPEQEAAAPGPAAGGEAPK
>	sp|Q05175|BAS	Brain acid soluble protein 1 OS=Rattus norvegicus GN=Basp1 PE=1 SV=2	AEPEKSEGAAEEQPEPAPAPEQEAAAPGPAAGGEAPK
>	sp|Q05175|BAS	Brain acid soluble protein 1 OS=Rattus norvegicus GN=Basp1 PE=1 SV=2	AEPEKSEGAAEEQPEPAPAPEQEAAAPGPAAGGEAPK
>	sp|Q05175|BAS	Brain acid soluble protein 1 OS=Rattus norvegicus GN=Basp1 PE=1 SV=2	AEPEKSEGAAEEQPEPAPAPEQEAAAPGPAAGGEAPK
>	sp|Q05175|BAS	Brain acid soluble protein 1 OS=Rattus norvegicus GN=Basp1 PE=1 SV=2	AGEASAESTGAADGAPQEEGEAK
>	sp|Q05175|BAS	Brain acid soluble protein 1 OS=Rattus norvegicus GN=Basp1 PE=1 SV=2	AGEASAESTGAADGAPQEEGEAK
>	sp|Q05175|BAS	Brain acid soluble protein 1 OS=Rattus norvegicus GN=Basp1 PE=1 SV=2	AGEASAESTGAADGAPQEEGEAK
>	sp|Q05175|BAS	Brain acid soluble protein 1 OS=Rattus norvegicus GN=Basp1 PE=1 SV=2	APAPAAPAAEPQAEAPVASSEQSVAVKE
>	sp|Q05175|BAS	Brain acid soluble protein 1 OS=Rattus norvegicus GN=Basp1 PE=1 SV=2	APAPAAPAAEPQAEAPVASSEQSVAVKE
>	sp|Q05175|BAS	Brain acid soluble protein 1 OS=Rattus norvegicus GN=Basp1 PE=1 SV=2	EEAPKAEPEKSEGAAEEQPEPAPAPEQEAAAPGPAAGGEAPK
>	sp|Q05175|BAS	Brain acid soluble protein 1 OS=Rattus norvegicus GN=Basp1 PE=1 SV=2	ESEPQAAADATEVK
>	sp|Q05175|BAS	Brain acid soluble protein 1 OS=Rattus norvegicus GN=Basp1 PE=1 SV=2	ESEPQAAADATEVKESAEEKPK
>	sp|Q05175|BAS	Brain acid soluble protein 1 OS=Rattus norvegicus GN=Basp1 PE=1 SV=2	ESEPQAAADATEVKESAEEKPK
>	sp|Q05175|BAS	Brain acid soluble protein 1 OS=Rattus norvegicus GN=Basp1 PE=1 SV=2	ESEPQAAADATEVKESAEEKPK
>	sp|Q05175|BAS	Brain acid soluble protein 1 OS=Rattus norvegicus GN=Basp1 PE=1 SV=2	ETPAASEAPSSAAK
>	sp|Q05175|BAS	Brain acid soluble protein 1 OS=Rattus norvegicus GN=Basp1 PE=1 SV=2	ETPAASEAPSSAAK
>	sp|Q05175|BAS	Brain acid soluble protein 1 OS=Rattus norvegicus GN=Basp1 PE=1 SV=2	ETPAASEAPSSAAK
>	sp|Q05175|BAS	Brain acid soluble protein 1 OS=Rattus norvegicus GN=Basp1 PE=1 SV=2	KAEGAGTEEEGTQK
>	sp|Q05175|BAS	Brain acid soluble protein 1 OS=Rattus norvegicus GN=Basp1 PE=1 SV=2	KAEGAGTEEEGTQK
>	sp|Q05175|BAS	Brain acid soluble protein 1 OS=Rattus norvegicus GN=Basp1 PE=1 SV=2	KAEGAGTEEEGTQK
>	sp|Q05175|BAS	Brain acid soluble protein 1 OS=Rattus norvegicus GN=Basp1 PE=1 SV=2	KAEGAGTEEEGTQK
>	sp|Q05175|BAS	Brain acid soluble protein 1 OS=Rattus norvegicus GN=Basp1 PE=1 SV=2	KAEGAGTEEEGTQKESEPQAAADATEVKESAEEKPK
>	sp|Q05175|BAS	Brain acid soluble protein 1 OS=Rattus norvegicus GN=Basp1 PE=1 SV=2	KAEGAGTEEEGTQKESEPQAAADATEVKESAEEKPK
>	sp|Q05175|BAS	Brain acid soluble protein 1 OS=Rattus norvegicus GN=Basp1 PE=1 SV=2	KAEGAGTEEEGTQKESEPQAAADATEVKESAEEKPK
>	sp|Q05175|BAS	Brain acid soluble protein 1 OS=Rattus norvegicus GN=Basp1 PE=1 SV=2	KAEGAGTEEEGTQKESEPQAAADATEVKESAEEKPK
>	sp|Q05175|BAS	Brain acid soluble protein 1 OS=Rattus norvegicus GN=Basp1 PE=1 SV=2	SDAAPAASDSKPSTEPAPSSK
>	sp|Q05175|BAS	Brain acid soluble protein 1 OS=Rattus norvegicus GN=Basp1 PE=1 SV=2	SDAAPAASDSKPSTEPAPSSK
>	sp|Q05175|BAS	Brain acid soluble protein 1 OS=Rattus norvegicus GN=Basp1 PE=1 SV=2	KAEGAGTEEEGTQK
>	sp|Q05175|BAS	Brain acid soluble protein 1 OS=Rattus norvegicus GN=Basp1 PE=1 SV=2	ESAEEKPKDAADGEAK
>	sp|Q05175|BAS	Brain acid soluble protein 1 OS=Rattus norvegicus GN=Basp1 PE=1 SV=2	DAADGEAK
>	sp|Q05175|BAS	Brain acid soluble protein 1 OS=Rattus norvegicus GN=Basp1 PE=1 SV=2	ETPAASEAPSSAAK
>	sp|Q05175|BAS	Brain acid soluble protein 1 OS=Rattus norvegicus GN=Basp1 PE=1 SV=2	DAADGEAK
>	sp|Q05175|BAS	Brain acid soluble protein 1 OS=Rattus norvegicus GN=Basp1 PE=1 SV=2	AGEASAESTGAADGAPQEEGEAK
>	sp|Q05175|BAS	Brain acid soluble protein 1 OS=Rattus norvegicus GN=Basp1 PE=1 SV=2	AEPEKSEGAAEEQPEPAPAPEQEAAAPGPAAGGEAPK
>	sp|Q05175|BAS	Brain acid soluble protein 1 OS=Rattus norvegicus GN=Basp1 PE=1 SV=2	AEPEKSEGAAEEQPEPAPAPEQEAAAPGPAAGGEAPK
>	sp|Q05175|BAS	Brain acid soluble protein 1 OS=Rattus norvegicus GN=Basp1 PE=1 SV=2	ESAEEKPK
>	sp|Q05175|BAS	Brain acid soluble protein 1 OS=Rattus norvegicus GN=Basp1 PE=1 SV=2	ESEPQAAADATEVK
>	sp|Q05175|BAS	Brain acid soluble protein 1 OS=Rattus norvegicus GN=Basp1 PE=1 SV=2	KAEGAGTEEEGTQKESEPQAAADATEVK
>	sp|Q05175|BAS	Brain acid soluble protein 1 OS=Rattus norvegicus GN=Basp1 PE=1 SV=2	ESAEEKPK
>	sp|Q05175|BAS	Brain acid soluble protein 1 OS=Rattus norvegicus GN=Basp1 PE=1 SV=2	ESEPQAAADATEVKESAEEKPKDAADGEAK
>	sp|Q05175|BAS	Brain acid soluble protein 1 OS=Rattus norvegicus GN=Basp1 PE=1 SV=2	AAAKEEAPKAEPEKSEGAAEEQPEPAPAPEQEAAAPGPAAGGEAPK
>	sp|Q05175|BAS	Brain acid soluble protein 1 OS=Rattus norvegicus GN=Basp1 PE=1 SV=2	AEGAGTEEEGTQK
>	sp|Q05175|BAS	Brain acid soluble protein 1 OS=Rattus norvegicus GN=Basp1 PE=1 SV=2	AAAKEEAPKAEPEKSEGAAEEQPEPAPAPEQEAAAPGPAAGGEAPK
>	sp|Q05175|BAS	Brain acid soluble protein 1 OS=Rattus norvegicus GN=Basp1 PE=1 SV=2	KAEGAGTEEEGTQKESEPQAAADATEVK
>	sp|Q05175|BAS	Brain acid soluble protein 1 OS=Rattus norvegicus GN=Basp1 PE=1 SV=2	KTEAPAAGPEAK
>	sp|Q05175|BAS	Brain acid soluble protein 1 OS=Rattus norvegicus GN=Basp1 PE=1 SV=2	AEGAGTEEEGTQKESEPQAAADATEVKESAEEKPK
>	sp|Q05175|BAS	Brain acid soluble protein 1 OS=Rattus norvegicus GN=Basp1 PE=1 SV=2	SDAAPAASDSKPSTEPAPSSK
>	sp|Q05175|BAS	Brain acid soluble protein 1 OS=Rattus norvegicus GN=Basp1 PE=1 SV=2	KAEGAGTEEEGTQKESEPQAAADATEVKESAEEKPK
>	sp|Q05175|BAS	Brain acid soluble protein 1 OS=Rattus norvegicus GN=Basp1 PE=1 SV=2	AAAKEEAPKAEPEKSEGAAEEQPEPAPAPEQEAAAPGPAAGGEAPK
>	sp|Q05175|BAS	Brain acid soluble protein 1 OS=Rattus norvegicus GN=Basp1 PE=1 SV=2	ESEPQAAADATEVKESAEEKPK
>	sp|Q05175|BAS	Brain acid soluble protein 1 OS=Rattus norvegicus GN=Basp1 PE=1 SV=2	AEPEKSEGAAEEQPEPAPAPEQEAAAPGPAAGGEAPK
>	sp|Q05175|BAS	Brain acid soluble protein 1 OS=Rattus norvegicus GN=Basp1 PE=1 SV=2	ESAEEKPKDAADGEAKAEEK
>	sp|Q05175|BAS	Brain acid soluble protein 1 OS=Rattus norvegicus GN=Basp1 PE=1 SV=2	AEEKEADKAAAK
>	sp|Q05175|BAS	Brain acid soluble protein 1 OS=Rattus norvegicus GN=Basp1 PE=1 SV=2	KAEGAGTEEEGTQK
>	sp|Q9QUL6|NS	Vesicle-fusing ATPase OS=Rattus norvegicus GN=Nsf PE=1 SV=1	FSNLVLQALLVLLKKAPPQGR
>	sp|Q9QUL6|NS	Vesicle-fusing ATPase OS=Rattus norvegicus GN=Nsf PE=1 SV=1	GHQLLSADVDIKELAVETK
>	sp|Q9QUL6|NS	Vesicle-fusing ATPase OS=Rattus norvegicus GN=Nsf PE=1 SV=1	IDGVEQLNNILVIGMTNRPDLIDEALLRPGR
>	sp|Q9QUL6|NS	Vesicle-fusing ATPase OS=Rattus norvegicus GN=Nsf PE=1 SV=1	IDGVEQLNNILVIGMTNRPDLIDEALLRPGRLEVK
>	sp|Q9QUL6|NS	Vesicle-fusing ATPase OS=Rattus norvegicus GN=Nsf PE=1 SV=1	KDVLQEMEMLNAFSTTIHVPNIATGEQLLEALELLGNFKDKER
>	sp|Q9QUL6|NS	Vesicle-fusing ATPase OS=Rattus norvegicus GN=Nsf PE=1 SV=1	KLLIIGTTSR
>	sp|Q9QUL6|NS	Vesicle-fusing ATPase OS=Rattus norvegicus GN=Nsf PE=1 SV=1	LQILHIHTAR
>	sp|Q9QUL6|NS	Vesicle-fusing ATPase OS=Rattus norvegicus GN=Nsf PE=1 SV=1	MGIGGLDKEFSDIFR
>	sp|Q9QUL6|NS	Vesicle-fusing ATPase OS=Rattus norvegicus GN=Nsf PE=1 SV=1	NFSGAELEGLVR
>	sp|Q9QUL6|NS	Vesicle-fusing ATPase OS=Rattus norvegicus GN=Nsf PE=1 SV=1	NSDRTPLVSVLLEGPPHSGK
>	sp|Q9QUL6|NS	Vesicle-fusing ATPase OS=Rattus norvegicus GN=Nsf PE=1 SV=1	TPLVSVLLEGPPHSGK
>	sp|Q9QUL6|NS	Vesicle-fusing ATPase OS=Rattus norvegicus GN=Nsf PE=1 SV=1	AENSSLNLIGK
>	sp|Q9QUL6|NS	Vesicle-fusing ATPase OS=Rattus norvegicus GN=Nsf PE=1 SV=1	IAEESNFPFIK
>	sp|Q9QUL6|NS	Vesicle-fusing ATPase OS=Rattus norvegicus GN=Nsf PE=1 SV=1	QKIEVGLVVGNSQVAFEK
>	sp|Q9QUL6|NS	Vesicle-fusing ATPase OS=Rattus norvegicus GN=Nsf PE=1 SV=1	SQLSCVVVDDIER
>	sp|Q9QUL6|NS	Vesicle-fusing ATPase OS=Rattus norvegicus GN=Nsf PE=1 SV=1	KLFADAEEEQR
>	sp|Q9QUL6|NS	Vesicle-fusing ATPase OS=Rattus norvegicus GN=Nsf PE=1 SV=1	YVGESEANIR

>	sp|Q9QUL6|NS Vesicle-fusing ATPase OS=Rattus norvegicus GN=Nsf PE=1 SV=1	FSNLVLQALLVLLKKAPPQGR
>	sp|Q9QUL6|NS Vesicle-fusing ATPase OS=Rattus norvegicus GN=Nsf PE=1 SV=1	FSNLVLQALLVLLKKAPPQGR
>	sp|Q9QUL6|NS Vesicle-fusing ATPase OS=Rattus norvegicus GN=Nsf PE=1 SV=1	FSNLVLQALLVLLKKAPPQGR
>	sp|Q9QUL6|NS Vesicle-fusing ATPase OS=Rattus norvegicus GN=Nsf PE=1 SV=1	IDGVEQLNNILVIGMTNRPDLIDEALLRPGR
>	sp|Q9QUL6|NS Vesicle-fusing ATPase OS=Rattus norvegicus GN=Nsf PE=1 SV=1	IDGVEQLNNILVIGMTNRPDLIDEALLRPGRLEVK
>	sp|Q9QUL6|NS Vesicle-fusing ATPase OS=Rattus norvegicus GN=Nsf PE=1 SV=1	IDGVEQLNNILVIGMTNRPDLIDEALLRPGRLEVK
>	sp|Q9QUL6|NS Vesicle-fusing ATPase OS=Rattus norvegicus GN=Nsf PE=1 SV=1	IDGVEQLNNILVIGMTNRPDLIDEALLRPGRLEVK
>	sp|Q9QUL6|NS Vesicle-fusing ATPase OS=Rattus norvegicus GN=Nsf PE=1 SV=1	KDVLQEMEMLNAFSTTIHVPNIATGEQLLEALELLGNFKDKER
>	sp|Q9QUL6|NS Vesicle-fusing ATPase OS=Rattus norvegicus GN=Nsf PE=1 SV=1	KDVLQEMEMLNAFSTTIHVPNIATGEQLLEALELLGNFKDKER
>	sp|Q9QUL6|NS Vesicle-fusing ATPase OS=Rattus norvegicus GN=Nsf PE=1 SV=1	KDVLQEMEMLNAFSTTIHVPNIATGEQLLEALELLGNFKDKER
>	sp|Q9QUL6|NS Vesicle-fusing ATPase OS=Rattus norvegicus GN=Nsf PE=1 SV=1	KDVLQEMEMLNAFSTTIHVPNIATGEQLLEALELLGNFKDKER
>	sp|Q9QUL6|NS Vesicle-fusing ATPase OS=Rattus norvegicus GN=Nsf PE=1 SV=1	KLFADAEEEQR
>	sp|Q9QUL6|NS Vesicle-fusing ATPase OS=Rattus norvegicus GN=Nsf PE=1 SV=1	KLLIIGTTSR
>	sp|Q9QUL6|NS Vesicle-fusing ATPase OS=Rattus norvegicus GN=Nsf PE=1 SV=1	KLLIIGTTSR
>	sp|Q9QUL6|NS Vesicle-fusing ATPase OS=Rattus norvegicus GN=Nsf PE=1 SV=1	MGIGGLDKEFSDIFR
>	sp|Q9QUL6|NS Vesicle-fusing ATPase OS=Rattus norvegicus GN=Nsf PE=1 SV=1	MGIGGLDKEFSDIFR
>	sp|Q9QUL6|NS Vesicle-fusing ATPase OS=Rattus norvegicus GN=Nsf PE=1 SV=1	MGIGGLDKEFSDIFR
>	sp|Q9QUL6|NS Vesicle-fusing ATPase OS=Rattus norvegicus GN=Nsf PE=1 SV=1	MGIGGLDKEFSDIFR
>	sp|Q9QUL6|NS Vesicle-fusing ATPase OS=Rattus norvegicus GN=Nsf PE=1 SV=1	MGIGGLDKEFSDIFR
>	sp|Q9QUL6|NS Vesicle-fusing ATPase OS=Rattus norvegicus GN=Nsf PE=1 SV=1	MGIGGLDKEFSDIFR
>	sp|Q9QUL6|NS Vesicle-fusing ATPase OS=Rattus norvegicus GN=Nsf PE=1 SV=1	MGIGGLDKEFSDIFR
>	sp|Q9QUL6|NS Vesicle-fusing ATPase OS=Rattus norvegicus GN=Nsf PE=1 SV=1	MGIGGLDKEFSDIFR
>	sp|Q9QUL6|NS Vesicle-fusing ATPase OS=Rattus norvegicus GN=Nsf PE=1 SV=1	MGIGGLDKEFSDIFR
>	sp|Q9QUL6|NS Vesicle-fusing ATPase OS=Rattus norvegicus GN=Nsf PE=1 SV=1	SQLSCVVVDDIER
>	sp|Q9QUL6|NS Vesicle-fusing ATPase OS=Rattus norvegicus GN=Nsf PE=1 SV=1	TPLVSVLLEGPPHSGK
>	sp|Q9QUL6|NS Vesicle-fusing ATPase OS=Rattus norvegicus GN=Nsf PE=1 SV=1	TPLVSVLLEGPPHSGK
>	sp|Q9QUL6|NS Vesicle-fusing ATPase OS=Rattus norvegicus GN=Nsf PE=1 SV=1	TPLVSVLLEGPPHSGK
>	sp|Q9QUL6|NS Vesicle-fusing ATPase OS=Rattus norvegicus GN=Nsf PE=1 SV=1	TPLVSVLLEGPPHSGK
>	sp|Q9QUL6|NS Vesicle-fusing ATPase OS=Rattus norvegicus GN=Nsf PE=1 SV=1	TPLVSVLLEGPPHSGK
>	sp|Q9QUL6|NS Vesicle-fusing ATPase OS=Rattus norvegicus GN=Nsf PE=1 SV=1	TPLVSVLLEGPPHSGK
>	sp|Q9QUL6|NS Vesicle-fusing ATPase OS=Rattus norvegicus GN=Nsf PE=1 SV=1	WAGLSIGQDIEVALYSFDK
>	sp|Q9QUL6|NS Vesicle-fusing ATPase OS=Rattus norvegicus GN=Nsf PE=1 SV=1	GILLYGPPGCGK
>	sp|Q9QUL6|NS Vesicle-fusing ATPase OS=Rattus norvegicus GN=Nsf PE=1 SV=1	GHQLLSADVDIKELAVETK
>	sp|Q9QUL6|NS Vesicle-fusing ATPase OS=Rattus norvegicus GN=Nsf PE=1 SV=1	NFSGAELEGLVR
>	sp|Q9QUL6|NS Vesicle-fusing ATPase OS=Rattus norvegicus GN=Nsf PE=1 SV=1	MGIGGLDKEFSDIFR
>	sp|Q9QUL6|NS Vesicle-fusing ATPase OS=Rattus norvegicus GN=Nsf PE=1 SV=1	MEIGLPDEK
>	sp|Q9QUL6|NS Vesicle-fusing ATPase OS=Rattus norvegicus GN=Nsf PE=1 SV=1	RLGANSGLHIIIFDEIDAICK
>	sp|Q9QUL6|NS Vesicle-fusing ATPase OS=Rattus norvegicus GN=Nsf PE=1 SV=1	IAEESNFPFIK
>	sp|Q9QUL6|NS Vesicle-fusing ATPase OS=Rattus norvegicus GN=Nsf PE=1 SV=1	GHQLLSADVDIK
>	sp|Q9QUL6|NS Vesicle-fusing ATPase OS=Rattus norvegicus GN=Nsf PE=1 SV=1	TTIAQQVK
>	sp|Q9QUL6|NS Vesicle-fusing ATPase OS=Rattus norvegicus GN=Nsf PE=1 SV=1	KFLALMR
>	sp|Q9QUL6|NS Vesicle-fusing ATPase OS=Rattus norvegicus GN=Nsf PE=1 SV=1	GILLYGPPGCGK
>	sp|Q9QUL6|NS Vesicle-fusing ATPase OS=Rattus norvegicus GN=Nsf PE=1 SV=1	KFLALMR
>	sp|P63039|CH6 60 kDa heat shock protein, mitochondrial OS=Rattus norvegicus GN=Hspd1 PE=1 SV=1	AAVEEGIVLGGGCALLR
>	sp|P63039|CH6 60 kDa heat shock protein, mitochondrial OS=Rattus norvegicus GN=Hspd1 PE=1 SV=1	ALMLQGVDLLADAVAVTMGPK
>	sp|P63039|CH6 60 kDa heat shock protein, mitochondrial OS=Rattus norvegicus GN=Hspd1 PE=1 SV=1	DMAIATGGAVFGEEGLNLNLEDVQAHDLGK
>	sp|P63039|CH6 60 kDa heat shock protein, mitochondrial OS=Rattus norvegicus GN=Hspd1 PE=1 SV=1	GVMLAVDAVIAELK
>	sp|P63039|CH6 60 kDa heat shock protein, mitochondrial OS=Rattus norvegicus GN=Hspd1 PE=1 SV=1	GVMLAVDAVIAELKK
>	sp|P63039|CH6 60 kDa heat shock protein, mitochondrial OS=Rattus norvegicus GN=Hspd1 PE=1 SV=1	IGIEIIKR
>	sp|P63039|CH6 60 kDa heat shock protein, mitochondrial OS=Rattus norvegicus GN=Hspd1 PE=1 SV=1	ISSVQSIVPALEIANAHR
>	sp|P63039|CH6 60 kDa heat shock protein, mitochondrial OS=Rattus norvegicus GN=Hspd1 PE=1 SV=1	KPLVIIAEDVDGEALSTLVLNR
>	sp|P63039|CH6 60 kDa heat shock protein, mitochondrial OS=Rattus norvegicus GN=Hspd1 PE=1 SV=1	LSDGVAVLK
>	sp|P63039|CH6 60 kDa heat shock protein, mitochondrial OS=Rattus norvegicus GN=Hspd1 PE=1 SV=1	QSKPVTTPEEIAQVATISANGDKDIGNIISDAMK
>	sp|P63039|CH6 60 kDa heat shock protein, mitochondrial OS=Rattus norvegicus GN=Hspd1 PE=1 SV=1	RGVMLAVDAVIAELK
>	sp|P63039|CH6 60 kDa heat shock protein, mitochondrial OS=Rattus norvegicus GN=Hspd1 PE=1 SV=1	RGVMLAVDAVIAELKK
>	sp|P63039|CH6 60 kDa heat shock protein, mitochondrial OS=Rattus norvegicus GN=Hspd1 PE=1 SV=1	TALLDAAGVASLLTTAEAVVTEIPKEEKDPGMGAMGGMGGGMGGGMF
>	sp|P63039|CH6 60 kDa heat shock protein, mitochondrial OS=Rattus norvegicus GN=Hspd1 PE=1 SV=1	TLNDELEIIEGMK
>	sp|P63039|CH6 60 kDa heat shock protein, mitochondrial OS=Rattus norvegicus GN=Hspd1 PE=1 SV=1	VGEVIVTKDDAMLLK
>	sp|P63039|CH6 60 kDa heat shock protein, mitochondrial OS=Rattus norvegicus GN=Hspd1 PE=1 SV=1	VTDALNATR
>	sp|P63039|CH6 60 kDa heat shock protein, mitochondrial OS=Rattus norvegicus GN=Hspd1 PE=1 SV=1	IQEITEQLDITTSEYEKEK
>	sp|P63039|CH6 60 kDa heat shock protein, mitochondrial OS=Rattus norvegicus GN=Hspd1 PE=1 SV=1	KISSVQSIVPALEIANAHR
>	sp|P63039|CH6 60 kDa heat shock protein, mitochondrial OS=Rattus norvegicus GN=Hspd1 PE=1 SV=1	ALMLQGVDLLADAVAVTMGPK
>	sp|P63039|CH6 60 kDa heat shock protein, mitochondrial OS=Rattus norvegicus GN=Hspd1 PE=1 SV=1	ALMLQGVDLLADAVAVTMGPK
>	sp|P63039|CH6 60 kDa heat shock protein, mitochondrial OS=Rattus norvegicus GN=Hspd1 PE=1 SV=1	ALMLQGVDLLADAVAVTMGPK
>	sp|P63039|CH6 60 kDa heat shock protein, mitochondrial OS=Rattus norvegicus GN=Hspd1 PE=1 SV=1	ALMLQGVDLLADAVAVTMGPK
>	sp|P63039|CH6 60 kDa heat shock protein, mitochondrial OS=Rattus norvegicus GN=Hspd1 PE=1 SV=1	GVMLAVDAVIAELK
>	sp|P63039|CH6 60 kDa heat shock protein, mitochondrial OS=Rattus norvegicus GN=Hspd1 PE=1 SV=1	GVMLAVDAVIAELKK
>	sp|P63039|CH6 60 kDa heat shock protein, mitochondrial OS=Rattus norvegicus GN=Hspd1 PE=1 SV=1	GVMLAVDAVIAELKK
>	sp|P63039|CH6 60 kDa heat shock protein, mitochondrial OS=Rattus norvegicus GN=Hspd1 PE=1 SV=1	GVMLAVDAVIAELKK
>	sp|P63039|CH6 60 kDa heat shock protein, mitochondrial OS=Rattus norvegicus GN=Hspd1 PE=1 SV=1	GVMLAVDAVIAELKK
>	sp|P63039|CH6 60 kDa heat shock protein, mitochondrial OS=Rattus norvegicus GN=Hspd1 PE=1 SV=1	GVMLAVDAVIAELKK
>	sp|P63039|CH6 60 kDa heat shock protein, mitochondrial OS=Rattus norvegicus GN=Hspd1 PE=1 SV=1	IGIEIIKR
>	sp|P63039|CH6 60 kDa heat shock protein, mitochondrial OS=Rattus norvegicus GN=Hspd1 PE=1 SV=1	IQEITEQLDITTSEYEKEK
>	sp|P63039|CH6 60 kDa heat shock protein, mitochondrial OS=Rattus norvegicus GN=Hspd1 PE=1 SV=1	IQEITEQLDITTSEYEKEK

>	sp|P63039|CH(	60 kDa heat shock protein, mitochondrial OS=Rattus norvegicus GN=Hspd1 PE=1 SV=1	KISSVQSIVPALEIANAHR
>	sp|P63039|CH(	60 kDa heat shock protein, mitochondrial OS=Rattus norvegicus GN=Hspd1 PE=1 SV=1	KISSVQSIVPALEIANAHR
>	sp|P63039|CH(	60 kDa heat shock protein, mitochondrial OS=Rattus norvegicus GN=Hspd1 PE=1 SV=1	KISSVQSIVPALEIANAHR
>	sp|P63039|CH(	60 kDa heat shock protein, mitochondrial OS=Rattus norvegicus GN=Hspd1 PE=1 SV=1	KISSVQSIVPALEIANAHR
>	sp|P63039|CH(	60 kDa heat shock protein, mitochondrial OS=Rattus norvegicus GN=Hspd1 PE=1 SV=1	KISSVQSIVPALEIANAHR
>	sp|P63039|CH(	60 kDa heat shock protein, mitochondrial OS=Rattus norvegicus GN=Hspd1 PE=1 SV=1	KISSVQSIVPALEIANAHR
>	sp|P63039|CH(	60 kDa heat shock protein, mitochondrial OS=Rattus norvegicus GN=Hspd1 PE=1 SV=1	KPLVIIAEDVDGEALSTLVLNR
>	sp|P63039|CH(	60 kDa heat shock protein, mitochondrial OS=Rattus norvegicus GN=Hspd1 PE=1 SV=1	KPLVIIAEDVDGEALSTLVLNR
>	sp|P63039|CH(	60 kDa heat shock protein, mitochondrial OS=Rattus norvegicus GN=Hspd1 PE=1 SV=1	KPLVIIAEDVDGEALSTLVLNR
>	sp|P63039|CH(	60 kDa heat shock protein, mitochondrial OS=Rattus norvegicus GN=Hspd1 PE=1 SV=1	KPLVIIAEDVDGEALSTLVLNR
>	sp|P63039|CH(	60 kDa heat shock protein, mitochondrial OS=Rattus norvegicus GN=Hspd1 PE=1 SV=1	KPLVIIAEDVDGEALSTLVLNR
>	sp|P63039|CH(	60 kDa heat shock protein, mitochondrial OS=Rattus norvegicus GN=Hspd1 PE=1 SV=1	QSKPVTTPEEIAQVATISANGDKDIGNIISDAMK
>	sp|P63039|CH(	60 kDa heat shock protein, mitochondrial OS=Rattus norvegicus GN=Hspd1 PE=1 SV=1	RGVMLAVDAVIAELK
>	sp|P63039|CH(	60 kDa heat shock protein, mitochondrial OS=Rattus norvegicus GN=Hspd1 PE=1 SV=1	TLNDELEIIEGMK
>	sp|P63039|CH(	60 kDa heat shock protein, mitochondrial OS=Rattus norvegicus GN=Hspd1 PE=1 SV=1	AAVEEGIVLGGGCALLR
>	sp|P63039|CH(	60 kDa heat shock protein, mitochondrial OS=Rattus norvegicus GN=Hspd1 PE=1 SV=1	DMAIATGGAVFGEEGLNLNLEDVQAHDLGK
>	sp|P63039|CH(	60 kDa heat shock protein, mitochondrial OS=Rattus norvegicus GN=Hspd1 PE=1 SV=1	VGGTSDVEVNEK
>	sp|P63039|CH(	60 kDa heat shock protein, mitochondrial OS=Rattus norvegicus GN=Hspd1 PE=1 SV=1	KISSVQSIVPALEIANAHR
>	sp|P63039|CH(	60 kDa heat shock protein, mitochondrial OS=Rattus norvegicus GN=Hspd1 PE=1 SV=1	VTDALNATR
>	sp|P63039|CH(	60 kDa heat shock protein, mitochondrial OS=Rattus norvegicus GN=Hspd1 PE=1 SV=1	IQEITEQLDITTSEYEKEK
>	sp|P63039|CH(	60 kDa heat shock protein, mitochondrial OS=Rattus norvegicus GN=Hspd1 PE=1 SV=1	IGIEIIK
>	sp|P63039|CH(	60 kDa heat shock protein, mitochondrial OS=Rattus norvegicus GN=Hspd1 PE=1 SV=1	VTDALNATR
>	sp|P63039|CH(	60 kDa heat shock protein, mitochondrial OS=Rattus norvegicus GN=Hspd1 PE=1 SV=1	VTDALNATR
>	sp|P63039|CH(	60 kDa heat shock protein, mitochondrial OS=Rattus norvegicus GN=Hspd1 PE=1 SV=1	ISSVQSIVPALEIANAHR
>	sp|P63039|CH(	60 kDa heat shock protein, mitochondrial OS=Rattus norvegicus GN=Hspd1 PE=1 SV=1	IGIEIIK
>	sp|P63039|CH(	60 kDa heat shock protein, mitochondrial OS=Rattus norvegicus GN=Hspd1 PE=1 SV=1	IQEITEQLDITTSEYEKEK
>	sp|P63039|CH(	60 kDa heat shock protein, mitochondrial OS=Rattus norvegicus GN=Hspd1 PE=1 SV=1	AAVEEGIVLGGGCALLR
>	sp|P63039|CH(	60 kDa heat shock protein, mitochondrial OS=Rattus norvegicus GN=Hspd1 PE=1 SV=1	KNQLKDMAIATGGAVFGEEGLNLNLEDVQAHDLGK
>	sp|P63039|CH(	60 kDa heat shock protein, mitochondrial OS=Rattus norvegicus GN=Hspd1 PE=1 SV=1	ISSVQSIVPALEIANAHR
>	sp|P07323|EN(	Gamma-enolase OS=Rattus norvegicus GN=Eno2 PE=1 SV=2	AAVPSGASTGIYEALELR
>	sp|P07323|EN(	Gamma-enolase OS=Rattus norvegicus GN=Eno2 PE=1 SV=2	AGAAEKDLPLYR
>	sp|P07323|EN(	Gamma-enolase OS=Rattus norvegicus GN=Eno2 PE=1 SV=2	AVDHINSTIAPALISSGLSVVEQEK
>	sp|P07323|EN(	Gamma-enolase OS=Rattus norvegicus GN=Eno2 PE=1 SV=2	AVDHINSTIAPALISSGLSVVEQEKLDNLMLELDGTENK
>	sp|P07323|EN(	Gamma-enolase OS=Rattus norvegicus GN=Eno2 PE=1 SV=2	CITGDQLGALYQDFVR
>	sp|P07323|EN(	Gamma-enolase OS=Rattus norvegicus GN=Eno2 PE=1 SV=2	DATNVGDEGGFAPNILENSEALELVK
>	sp|P07323|EN(	Gamma-enolase OS=Rattus norvegicus GN=Eno2 PE=1 SV=2	FGANAILGVSLAVCK
>	sp|P07323|EN(	Gamma-enolase OS=Rattus norvegicus GN=Eno2 PE=1 SV=2	HIAQLAGNSDLILPVPAFNVINGGSHAGNK
>	sp|P07323|EN(	Gamma-enolase OS=Rattus norvegicus GN=Eno2 PE=1 SV=2	IEEELGEEAR
>	sp|P07323|EN(	Gamma-enolase OS=Rattus norvegicus GN=Eno2 PE=1 SV=2	LAMQEFMILPVGAESFR
>	sp|P07323|EN(	Gamma-enolase OS=Rattus norvegicus GN=Eno2 PE=1 SV=2	LAQENGWGVMVSHR
>	sp|P07323|EN(	Gamma-enolase OS=Rattus norvegicus GN=Eno2 PE=1 SV=2	MVIGMDVAASEFYR
>	sp|P07323|EN(	Gamma-enolase OS=Rattus norvegicus GN=Eno2 PE=1 SV=2	NYPVVSIEDPFDQDDWAAWSK
>	sp|P07323|EN(	Gamma-enolase OS=Rattus norvegicus GN=Eno2 PE=1 SV=2	SGETEDTFIADLVVGLCTGQIK
>	sp|P07323|EN(	Gamma-enolase OS=Rattus norvegicus GN=Eno2 PE=1 SV=2	VNQIGSVTEAIQACK
>	sp|P07323|EN(	Gamma-enolase OS=Rattus norvegicus GN=Eno2 PE=1 SV=2	GNPTVEVDLHTAK
>	sp|P07323|EN(	Gamma-enolase OS=Rattus norvegicus GN=Eno2 PE=1 SV=2	FTANVGIQIVGDDLTVTNPK
>	sp|P07323|EN(	Gamma-enolase OS=Rattus norvegicus GN=Eno2 PE=1 SV=2	PVPAFNVINGGSHAGNK
>	sp|P07323|EN(	Gamma-enolase OS=Rattus norvegicus GN=Eno2 PE=1 SV=2	AAVPSGASTGIYEALELR
>	sp|P07323|EN(	Gamma-enolase OS=Rattus norvegicus GN=Eno2 PE=1 SV=2	AAVPSGASTGIYEALELR
>	sp|P07323|EN(	Gamma-enolase OS=Rattus norvegicus GN=Eno2 PE=1 SV=2	AAVPSGASTGIYEALELR
>	sp|P07323|EN(	Gamma-enolase OS=Rattus norvegicus GN=Eno2 PE=1 SV=2	AAVPSGASTGIYEALELR
>	sp|P07323|EN(	Gamma-enolase OS=Rattus norvegicus GN=Eno2 PE=1 SV=2	AAVPSGASTGIYEALELR
>	sp|P07323|EN(	Gamma-enolase OS=Rattus norvegicus GN=Eno2 PE=1 SV=2	AAVPSGASTGIYEALELR
>	sp|P07323|EN(	Gamma-enolase OS=Rattus norvegicus GN=Eno2 PE=1 SV=2	AAVPSGASTGIYEALELR
>	sp|P07323|EN(	Gamma-enolase OS=Rattus norvegicus GN=Eno2 PE=1 SV=2	AVDHINSTIAPALISSGLSVVEQEK
>	sp|P07323|EN(	Gamma-enolase OS=Rattus norvegicus GN=Eno2 PE=1 SV=2	AVDHINSTIAPALISSGLSVVEQEK
>	sp|P07323|EN(	Gamma-enolase OS=Rattus norvegicus GN=Eno2 PE=1 SV=2	AVDHINSTIAPALISSGLSVVEQEK
>	sp|P07323|EN(	Gamma-enolase OS=Rattus norvegicus GN=Eno2 PE=1 SV=2	AVDHINSTIAPALISSGLSVVEQEK
>	sp|P07323|EN(	Gamma-enolase OS=Rattus norvegicus GN=Eno2 PE=1 SV=2	AVDHINSTIAPALISSGLSVVEQEK
>	sp|P07323|EN(	Gamma-enolase OS=Rattus norvegicus GN=Eno2 PE=1 SV=2	AVDHINSTIAPALISSGLSVVEQEKLDNLMLELDGTENK
>	sp|P07323|EN(	Gamma-enolase OS=Rattus norvegicus GN=Eno2 PE=1 SV=2	AVDHINSTIAPALISSGLSVVEQEKLDNLMLELDGTENK
>	sp|P07323|EN(	Gamma-enolase OS=Rattus norvegicus GN=Eno2 PE=1 SV=2	AVDHINSTIAPALISSGLSVVEQEKLDNLMLELDGTENK
>	sp|P07323|EN(	Gamma-enolase OS=Rattus norvegicus GN=Eno2 PE=1 SV=2	AVDHINSTIAPALISSGLSVVEQEKLDNLMLELDGTENK
>	sp|P07323|EN(	Gamma-enolase OS=Rattus norvegicus GN=Eno2 PE=1 SV=2	AVDHINSTIAPALISSGLSVVEQEKLDNLMLELDGTENK
>	sp|P07323|EN(	Gamma-enolase OS=Rattus norvegicus GN=Eno2 PE=1 SV=2	AVDHINSTIAPALISSGLSVVEQEKLDNLMLELDGTENK
>	sp|P07323|EN(	Gamma-enolase OS=Rattus norvegicus GN=Eno2 PE=1 SV=2	AVDHINSTIAPALISSGLSVVEQEKLDNLMLELDGTENK
>	sp|P07323|EN(	Gamma-enolase OS=Rattus norvegicus GN=Eno2 PE=1 SV=2	AVDHINSTIAPALISSGLSVVEQEKLDNLMLELDGTENK
>	sp|P07323|EN(	Gamma-enolase OS=Rattus norvegicus GN=Eno2 PE=1 SV=2	AVDHINSTIAPALISSGLSVVEQEKLDNLMLELDGTENK
>	sp|P07323|EN(	Gamma-enolase OS=Rattus norvegicus GN=Eno2 PE=1 SV=2	AVDHINSTIAPALISSGLSVVEQEKLDNLMLELDGTENK
>	sp|P07323|EN(	Gamma-enolase OS=Rattus norvegicus GN=Eno2 PE=1 SV=2	AVDHINSTIAPALISSGLSVVEQEKLDNLMLELDGTENK
>	sp|P07323|EN(	Gamma-enolase OS=Rattus norvegicus GN=Eno2 PE=1 SV=2	AVDHINSTIAPALISSGLSVVEQEKLDNLMLELDGTENK
>	sp|P07323|EN(	Gamma-enolase OS=Rattus norvegicus GN=Eno2 PE=1 SV=2	AVDHINSTIAPALISSGLSVVEQEKLDNLMLELDGTENK
>	sp|P07323|EN(	Gamma-enolase OS=Rattus norvegicus GN=Eno2 PE=1 SV=2	AVDHINSTIAPALISSGLSVVEQEKLDNLMLELDGTENK
>	sp|P07323|EN(	Gamma-enolase OS=Rattus norvegicus GN=Eno2 PE=1 SV=2	AVDHINSTIAPALISSGLSVVEQEKLDNLMLELDGTENK

>	sp|P07323|EN(	Gamma-enolase OS=Rattus norvegicus GN=Eno2 PE=1 SV=2	AVDHINSTIAPALISSGLSVVEQEKLDNLMLELDGTENK
>	sp|P07323|EN(	Gamma-enolase OS=Rattus norvegicus GN=Eno2 PE=1 SV=2	AVDHINSTIAPALISSGLSVVEQEKLDNLMLELDGTENK
>	sp|P07323|EN(	Gamma-enolase OS=Rattus norvegicus GN=Eno2 PE=1 SV=2	AVDHINSTIAPALISSGLSVVEQEKLDNLMLELDGTENK
>	sp|P07323|EN(	Gamma-enolase OS=Rattus norvegicus GN=Eno2 PE=1 SV=2	AVDHINSTIAPALISSGLSVVEQEKLDNLMLELDGTENK
>	sp|P07323|EN(	Gamma-enolase OS=Rattus norvegicus GN=Eno2 PE=1 SV=2	AVDHINSTIAPALISSGLSVVEQEKLDNLMLELDGTENK
>	sp|P07323|EN(	Gamma-enolase OS=Rattus norvegicus GN=Eno2 PE=1 SV=2	AVDHINSTIAPALISSGLSVVEQEKLDNLMLELDGTENK
>	sp|P07323|EN(	Gamma-enolase OS=Rattus norvegicus GN=Eno2 PE=1 SV=2	AVDHINSTIAPALISSGLSVVEQEKLDNLMLELDGTENK
>	sp|P07323|EN(	Gamma-enolase OS=Rattus norvegicus GN=Eno2 PE=1 SV=2	AVDHINSTIAPALISSGLSVVEQEKLDNLMLELDGTENK
>	sp|P07323|EN(	Gamma-enolase OS=Rattus norvegicus GN=Eno2 PE=1 SV=2	AVDHINSTIAPALISSGLSVVEQEKLDNLMLELDGTENK
>	sp|P07323|EN(	Gamma-enolase OS=Rattus norvegicus GN=Eno2 PE=1 SV=2	AVDHINSTIAPALISSGLSVVEQEKLDNLMLELDGTENK
>	sp|P07323|EN(	Gamma-enolase OS=Rattus norvegicus GN=Eno2 PE=1 SV=2	AVDHINSTIAPALISSGLSVVEQEKLDNLMLELDGTENK
>	sp|P07323|EN(	Gamma-enolase OS=Rattus norvegicus GN=Eno2 PE=1 SV=2	AVDHINSTIAPALISSGLSVVEQEKLDNLMLELDGTENK
>	sp|P07323|EN(	Gamma-enolase OS=Rattus norvegicus GN=Eno2 PE=1 SV=2	AVDHINSTIAPALISSGLSVVEQEKLDNLMLELDGTENK
>	sp|P07323|EN(	Gamma-enolase OS=Rattus norvegicus GN=Eno2 PE=1 SV=2	AVDHINSTIAPALISSGLSVVEQEKLDNLMLELDGTENK
>	sp|P07323|EN(	Gamma-enolase OS=Rattus norvegicus GN=Eno2 PE=1 SV=2	AVDHINSTIAPALISSGLSVVEQEKLDNLMLELDGTENK
>	sp|P07323|EN(	Gamma-enolase OS=Rattus norvegicus GN=Eno2 PE=1 SV=2	AVDHINSTIAPALISSGLSVVEQEKLDNLMLELDGTENK
>	sp|P07323|EN(	Gamma-enolase OS=Rattus norvegicus GN=Eno2 PE=1 SV=2	AVDHINSTIAPALISSGLSVVEQEKLDNLMLELDGTENK
>	sp|P07323|EN(	Gamma-enolase OS=Rattus norvegicus GN=Eno2 PE=1 SV=2	AVDHINSTIAPALISSGLSVVEQEKLDNLMLELDGTENK
>	sp|P07323|EN(	Gamma-enolase OS=Rattus norvegicus GN=Eno2 PE=1 SV=2	AVDHINSTIAPALISSGLSVVEQEKLDNLMLELDGTENK
>	sp|P07323|EN(	Gamma-enolase OS=Rattus norvegicus GN=Eno2 PE=1 SV=2	AVDHINSTIAPALISSGLSVVEQEKLDNLMLELDGTENK
>	sp|P07323|EN(	Gamma-enolase OS=Rattus norvegicus GN=Eno2 PE=1 SV=2	AVDHINSTIAPALISSGLSVVEQEKLDNLMLELDGTENK
>	sp|P07323|EN(	Gamma-enolase OS=Rattus norvegicus GN=Eno2 PE=1 SV=2	AVDHINSTIAPALISSGLSVVEQEKLDNLMLELDGTENK
>	sp|P07323|EN(	Gamma-enolase OS=Rattus norvegicus GN=Eno2 PE=1 SV=2	CITGDQLGALYQDFVR
>	sp|P07323|EN(	Gamma-enolase OS=Rattus norvegicus GN=Eno2 PE=1 SV=2	CITGDQLGALYQDFVR
>	sp|P07323|EN(	Gamma-enolase OS=Rattus norvegicus GN=Eno2 PE=1 SV=2	DATNVGDEGGFAPNILENSEALELVK
>	sp|P07323|EN(	Gamma-enolase OS=Rattus norvegicus GN=Eno2 PE=1 SV=2	DATNVGDEGGFAPNILENSEALELVK
>	sp|P07323|EN(	Gamma-enolase OS=Rattus norvegicus GN=Eno2 PE=1 SV=2	HIAQLAGNSDLILPVPAFNVINGGSHAGNK
>	sp|P07323|EN(	Gamma-enolase OS=Rattus norvegicus GN=Eno2 PE=1 SV=2	HIAQLAGNSDLILPVPAFNVINGGSHAGNK
>	sp|P07323|EN(	Gamma-enolase OS=Rattus norvegicus GN=Eno2 PE=1 SV=2	HIAQLAGNSDLILPVPAFNVINGGSHAGNK
>	sp|P07323|EN(	Gamma-enolase OS=Rattus norvegicus GN=Eno2 PE=1 SV=2	HIAQLAGNSDLILPVPAFNVINGGSHAGNK
>	sp|P07323|EN(	Gamma-enolase OS=Rattus norvegicus GN=Eno2 PE=1 SV=2	HIAQLAGNSDLILPVPAFNVINGGSHAGNK
>	sp|P07323|EN(	Gamma-enolase OS=Rattus norvegicus GN=Eno2 PE=1 SV=2	HIAQLAGNSDLILPVPAFNVINGGSHAGNK
>	sp|P07323|EN(	Gamma-enolase OS=Rattus norvegicus GN=Eno2 PE=1 SV=2	HIAQLAGNSDLILPVPAFNVINGGSHAGNK
>	sp|P07323|EN(	Gamma-enolase OS=Rattus norvegicus GN=Eno2 PE=1 SV=2	HIAQLAGNSDLILPVPAFNVINGGSHAGNK
>	sp|P07323|EN(	Gamma-enolase OS=Rattus norvegicus GN=Eno2 PE=1 SV=2	HIAQLAGNSDLILPVPAFNVINGGSHAGNK
>	sp|P07323|EN(	Gamma-enolase OS=Rattus norvegicus GN=Eno2 PE=1 SV=2	HIAQLAGNSDLILPVPAFNVINGGSHAGNK
>	sp|P07323|EN(	Gamma-enolase OS=Rattus norvegicus GN=Eno2 PE=1 SV=2	HIAQLAGNSDLILPVPAFNVINGGSHAGNK
>	sp|P07323|EN(	Gamma-enolase OS=Rattus norvegicus GN=Eno2 PE=1 SV=2	HIAQLAGNSDLILPVPAFNVINGGSHAGNK
>	sp|P07323|EN(	Gamma-enolase OS=Rattus norvegicus GN=Eno2 PE=1 SV=2	IEEELGEEAR
>	sp|P07323|EN(	Gamma-enolase OS=Rattus norvegicus GN=Eno2 PE=1 SV=2	LAMQEFMILPVGAESFR
>	sp|P07323|EN(	Gamma-enolase OS=Rattus norvegicus GN=Eno2 PE=1 SV=2	LAMQEFMILPVGAESFR
>	sp|P07323|EN(	Gamma-enolase OS=Rattus norvegicus GN=Eno2 PE=1 SV=2	LAQENGWGVMVSHR
>	sp|P07323|EN(	Gamma-enolase OS=Rattus norvegicus GN=Eno2 PE=1 SV=2	LAQENGWGVMVSHR
>	sp|P07323|EN(	Gamma-enolase OS=Rattus norvegicus GN=Eno2 PE=1 SV=2	LAQENGWGVMVSHR
>	sp|P07323|EN(	Gamma-enolase OS=Rattus norvegicus GN=Eno2 PE=1 SV=2	MVIGMDVAASEFYR
>	sp|P07323|EN(	Gamma-enolase OS=Rattus norvegicus GN=Eno2 PE=1 SV=2	MVIGMDVAASEFYR
>	sp|P07323|EN(	Gamma-enolase OS=Rattus norvegicus GN=Eno2 PE=1 SV=2	MVIGMDVAASEFYR
>	sp|P07323|EN(	Gamma-enolase OS=Rattus norvegicus GN=Eno2 PE=1 SV=2	SGETEDTFIADLVVGLCTGQIK
>	sp|P07323|EN(	Gamma-enolase OS=Rattus norvegicus GN=Eno2 PE=1 SV=2	SGETEDTFIADLVVGLCTGQIK
>	sp|P07323|EN(	Gamma-enolase OS=Rattus norvegicus GN=Eno2 PE=1 SV=2	SGETEDTFIADLVVGLCTGQIK
>	sp|P07323|EN(	Gamma-enolase OS=Rattus norvegicus GN=Eno2 PE=1 SV=2	SGETEDTFIADLVVGLCTGQIK
>	sp|P07323|EN(	Gamma-enolase OS=Rattus norvegicus GN=Eno2 PE=1 SV=2	SGETEDTFIADLVVGLCTGQIK
>	sp|P07323|EN(	Gamma-enolase OS=Rattus norvegicus GN=Eno2 PE=1 SV=2	VNQIGSVTEAIQACK
>	sp|P07323|EN(	Gamma-enolase OS=Rattus norvegicus GN=Eno2 PE=1 SV=2	VNQIGSVTEAIQACK
>	sp|P07323|EN(	Gamma-enolase OS=Rattus norvegicus GN=Eno2 PE=1 SV=2	VNQIGSVTEAIQACK
>	sp|P07323|EN(	Gamma-enolase OS=Rattus norvegicus GN=Eno2 PE=1 SV=2	HIAQLAGNSDLILPVPAFNVINGGSHAGNK
>	sp|P07323|EN(	Gamma-enolase OS=Rattus norvegicus GN=Eno2 PE=1 SV=2	FGANAILGVSLAVCK
>	sp|P07323|EN(	Gamma-enolase OS=Rattus norvegicus GN=Eno2 PE=1 SV=2	LAQENGWGVMVSHR
>	sp|P07323|EN(	Gamma-enolase OS=Rattus norvegicus GN=Eno2 PE=1 SV=2	LAMQEFMILPVGAESFR
>	sp|P07323|EN(	Gamma-enolase OS=Rattus norvegicus GN=Eno2 PE=1 SV=2	LAQENGWGVMVSHR
>	sp|P07323|EN(	Gamma-enolase OS=Rattus norvegicus GN=Eno2 PE=1 SV=2	FTANVGIQIVGDDLTVTNPK
>	sp|P07323|EN(	Gamma-enolase OS=Rattus norvegicus GN=Eno2 PE=1 SV=2	AVDHINSTIAPALISSGLSVVEQEK
>	sp|P07323|EN(	Gamma-enolase OS=Rattus norvegicus GN=Eno2 PE=1 SV=2	AVDHINSTIAPALISSGLSVVEQEK
>	sp|P07323|EN(	Gamma-enolase OS=Rattus norvegicus GN=Eno2 PE=1 SV=2	HIAQLAGNSDLILPVPAFNVINGGSHAGNK
>	sp|P07323|EN(	Gamma-enolase OS=Rattus norvegicus GN=Eno2 PE=1 SV=2	AVDHINSTIAPALISSGLSVVEQEKLDNLMLELDGTENK
>	sp|P07323|EN(	Gamma-enolase OS=Rattus norvegicus GN=Eno2 PE=1 SV=2	FGANAILGVSLAVCK
>	sp|P07323|EN(	Gamma-enolase OS=Rattus norvegicus GN=Eno2 PE=1 SV=2	GNPTVEVDLHTAK
>	sp|P07323|EN(	Gamma-enolase OS=Rattus norvegicus GN=Eno2 PE=1 SV=2	FGANAILGVSLAVCK
>	sp|P07323|EN(	Gamma-enolase OS=Rattus norvegicus GN=Eno2 PE=1 SV=2	FRAAVPSGASTGIYEALELR
>	sp|P07323|EN(	Gamma-enolase OS=Rattus norvegicus GN=Eno2 PE=1 SV=2	NYPVVSIEDPFDQDDWAAWSK
>	sp|P07323|EN(	Gamma-enolase OS=Rattus norvegicus GN=Eno2 PE=1 SV=2	HIAQLAGNSDLILPVPAFNVINGGSHAGNK
>	sp|P07323|EN(	Gamma-enolase OS=Rattus norvegicus GN=Eno2 PE=1 SV=2	AAVPSGASTGIYEALELR
>	sp|P07323|EN(	Gamma-enolase OS=Rattus norvegicus GN=Eno2 PE=1 SV=2	HIAQLAGNSDLILPVPAFNVINGGSHAGNK
>	sp|P07323|EN(	Gamma-enolase OS=Rattus norvegicus GN=Eno2 PE=1 SV=2	LAMQEFMILPVGAESFR

>	sp|P07323|ENO	Gamma-enolase OS=Rattus norvegicus GN=Eno2 PE=1 SV=2	HIAQLAGNSDLILPVPAFNVINGGSHAGNK
>	sp|P07323|ENO	Gamma-enolase OS=Rattus norvegicus GN=Eno2 PE=1 SV=2	NYPVVSIEDPFDQDDWAAWSK
>	sp|P07323|ENO	Gamma-enolase OS=Rattus norvegicus GN=Eno2 PE=1 SV=2	DGKYDLDFK
>	sp|P07323|ENO	Gamma-enolase OS=Rattus norvegicus GN=Eno2 PE=1 SV=2	HIAQLAGNSDLILPVPAFNVINGGSHAGNK
>	sp|P07323|ENO	Gamma-enolase OS=Rattus norvegicus GN=Eno2 PE=1 SV=2	GNPTVEVDLHTAK
>	sp|P07323|ENO	Gamma-enolase OS=Rattus norvegicus GN=Eno2 PE=1 SV=2	NYPVVSIEDPFDQDDWAAWSK
>	sp|P07323|ENO	Gamma-enolase OS=Rattus norvegicus GN=Eno2 PE=1 SV=2	DLPLYR
>	sp|P07323|ENO	Gamma-enolase OS=Rattus norvegicus GN=Eno2 PE=1 SV=2	LAQENGWGVMVSHR
>	sp|P07323|ENO	Gamma-enolase OS=Rattus norvegicus GN=Eno2 PE=1 SV=2	HIAQLAGNSDLILPVPAFNVINGGSHAGNK
>	sp|P07323|ENO	Gamma-enolase OS=Rattus norvegicus GN=Eno2 PE=1 SV=2	MVIGMDVAASEFYR
>	sp|P07323|ENO	Gamma-enolase OS=Rattus norvegicus GN=Eno2 PE=1 SV=2	HIAQLAGNSDLILPVPAFNVINGGSHAGNK
>	sp|P07323|ENO	Gamma-enolase OS=Rattus norvegicus GN=Eno2 PE=1 SV=2	AVDHINSTIAPALISSGLSVVEQEKLDNLMLELDGTENK
>	sp|P07323|ENO	Gamma-enolase OS=Rattus norvegicus GN=Eno2 PE=1 SV=2	AVDHINSTIAPALISSGLSVVEQEKLDNLMLELDGTENK
>	sp|P07323|ENO	Gamma-enolase OS=Rattus norvegicus GN=Eno2 PE=1 SV=2	AVDHINSTIAPALISSGLSVVEQEKLDNLMLELDGTENK
>	sp|P07323|ENO	Gamma-enolase OS=Rattus norvegicus GN=Eno2 PE=1 SV=2	LAQENGWGVMVSHR
>	sp|P07323|ENO	Gamma-enolase OS=Rattus norvegicus GN=Eno2 PE=1 SV=2	PSGASTGIYEALELR
>	sp|P07323|ENO	Gamma-enolase OS=Rattus norvegicus GN=Eno2 PE=1 SV=2	DLPLYR
>	sp|P07323|ENO	Gamma-enolase OS=Rattus norvegicus GN=Eno2 PE=1 SV=2	AVDHINSTIAPALISSGLSVVEQEKLDNLMLELDGTENK
>	sp|P07323|ENO	Gamma-enolase OS=Rattus norvegicus GN=Eno2 PE=1 SV=2	FTANVGIQIVGDDLTVTNPK
>	sp|P07323|ENO	Gamma-enolase OS=Rattus norvegicus GN=Eno2 PE=1 SV=2	LAQENGWGVMVSHR
>	sp|P07323|ENO	Gamma-enolase OS=Rattus norvegicus GN=Eno2 PE=1 SV=2	AVDHINSTIAPALISSGLSVVEQEKLDNLMLELDGTENK
>	sp|P07323|ENO	Gamma-enolase OS=Rattus norvegicus GN=Eno2 PE=1 SV=2	AVDHINSTIAPALISSGLSVVEQEKLDNLMLELDGTENK
>	sp|P11505|AT2	Plasma membrane calcium-transporting ATPase 1 OS=Rattus norvegicus GN=Atp2b1 PE=2 SV=2	AQDGAAMEMQPLKSEEGGDGDEKDKK
>	sp|P11505|AT2	Plasma membrane calcium-transporting ATPase 1 OS=Rattus norvegicus GN=Atp2b1 PE=2 SV=2	DFPAGEPEPEWDNENDVVTGLTCIAVVGIEDPVRPEVPEAIKK
>	sp|P11505|AT2	Plasma membrane calcium-transporting ATPase 1 OS=Rattus norvegicus GN=Atp2b1 PE=2 SV=2	EAGHGTQKEEIPEEELAEDVEEIDHAER
>	sp|P11505|AT2	Plasma membrane calcium-transporting ATPase 1 OS=Rattus norvegicus GN=Atp2b1 PE=2 SV=2	GGQVIQIPVADITVGDIAQVK
>	sp|P11505|AT2	Plasma membrane calcium-transporting ATPase 1 OS=Rattus norvegicus GN=Atp2b1 PE=2 SV=2	HLDACETMGNATAICSDK
>	sp|P11505|AT2	Plasma membrane calcium-transporting ATPase 1 OS=Rattus norvegicus GN=Atp2b1 PE=2 SV=2	HVGNKTECALLGFLLDLKR
>	sp|P11505|AT2	Plasma membrane calcium-transporting ATPase 1 OS=Rattus norvegicus GN=Atp2b1 PE=2 SV=2	KADVGFAMGIAGTDVAK
>	sp|P11505|AT2	Plasma membrane calcium-transporting ATPase 1 OS=Rattus norvegicus GN=Atp2b1 PE=2 SV=2	MVTGDNINTAR
>	sp|P11505|AT2	Plasma membrane calcium-transporting ATPase 1 OS=Rattus norvegicus GN=Atp2b1 PE=2 SV=2	NEKGEIEQER
>	sp|P11505|AT2	Plasma membrane calcium-transporting ATPase 1 OS=Rattus norvegicus GN=Atp2b1 PE=2 SV=2	TECALLGFLLDLKR
>	sp|P11505|AT2	Plasma membrane calcium-transporting ATPase 1 OS=Rattus norvegicus GN=Atp2b1 PE=2 SV=2	TSPNEGLSGNPADLER
>	sp|P11505|AT2	Plasma membrane calcium-transporting ATPase 1 OS=Rattus norvegicus GN=Atp2b1 PE=2 SV=2	TVIEPMASEGLR
>	sp|P11505|AT2	Plasma membrane calcium-transporting ATPase 1 OS=Rattus norvegicus GN=Atp2b1 PE=2 SV=2	MVVTAVGVNSQTGIIFTLLGAGGEEEEKKDEK
>	sp|P11505|AT2	Plasma membrane calcium-transporting ATPase 1 OS=Rattus norvegicus GN=Atp2b1 PE=2 SV=2	QVVAVTGDGTNDGPALK
>	sp|P11505|AT2	Plasma membrane calcium-transporting ATPase 1 OS=Rattus norvegicus GN=Atp2b1 PE=2 SV=2	CGILHPGEDFLCLEGK
>	sp|P11505|AT2	Plasma membrane calcium-transporting ATPase 1 OS=Rattus norvegicus GN=Atp2b1 PE=2 SV=2	AQDGAAMEMQPLKSEEGGDGDEKDKK
>	sp|P11505|AT2	Plasma membrane calcium-transporting ATPase 1 OS=Rattus norvegicus GN=Atp2b1 PE=2 SV=2	KADVGFAMGIAGTDVAK
>	sp|P11505|AT2	Plasma membrane calcium-transporting ATPase 1 OS=Rattus norvegicus GN=Atp2b1 PE=2 SV=2	KADVGFAMGIAGTDVAK
>	sp|P11505|AT2	Plasma membrane calcium-transporting ATPase 1 OS=Rattus norvegicus GN=Atp2b1 PE=2 SV=2	KADVGFAMGIAGTDVAK
>	sp|P11505|AT2	Plasma membrane calcium-transporting ATPase 1 OS=Rattus norvegicus GN=Atp2b1 PE=2 SV=2	KADVGFAMGIAGTDVAK
>	sp|P11505|AT2	Plasma membrane calcium-transporting ATPase 1 OS=Rattus norvegicus GN=Atp2b1 PE=2 SV=2	MVVTAVGVNSQTGIIFTLLGAGGEEEEKKDEK
>	sp|P11505|AT2	Plasma membrane calcium-transporting ATPase 1 OS=Rattus norvegicus GN=Atp2b1 PE=2 SV=2	MVVTAVGVNSQTGIIFTLLGAGGEEEEKKDEK
>	sp|P11505|AT2	Plasma membrane calcium-transporting ATPase 1 OS=Rattus norvegicus GN=Atp2b1 PE=2 SV=2	NEKGEIEQER
>	sp|P11505|AT2	Plasma membrane calcium-transporting ATPase 1 OS=Rattus norvegicus GN=Atp2b1 PE=2 SV=2	KVPEPEAIPPNILSYLVTGISVNCAYTSK
>	sp|P11505|AT2	Plasma membrane calcium-transporting ATPase 1 OS=Rattus norvegicus GN=Atp2b1 PE=2 SV=2	HLDACETMGNATAICSDK
>	sp|P11505|AT2	Plasma membrane calcium-transporting ATPase 1 OS=Rattus norvegicus GN=Atp2b1 PE=2 SV=2	LKTSPNEGLSGNPADLER
>	sp|P11505|AT2	Plasma membrane calcium-transporting ATPase 1 OS=Rattus norvegicus GN=Atp2b1 PE=2 SV=2	HLDACETMGNATAICSDK
>	sp|P11505|AT2	Plasma membrane calcium-transporting ATPase 1 OS=Rattus norvegicus GN=Atp2b1 PE=2 SV=2	GGQVIQIPVADITVGDIAQVK
>	sp|P11505|AT2	Plasma membrane calcium-transporting ATPase 1 OS=Rattus norvegicus GN=Atp2b1 PE=2 SV=2	LKTSPNEGLSGNPADLER
>	sp|P11505|AT2	Plasma membrane calcium-transporting ATPase 1 OS=Rattus norvegicus GN=Atp2b1 PE=2 SV=2	MVVTAVGVNSQTGIIFTLLGAGGEEEEKKDEK
>	sp|P11505|AT2	Plasma membrane calcium-transporting ATPase 1 OS=Rattus norvegicus GN=Atp2b1 PE=2 SV=2	MVVTAVGVNSQTGIIFTLLGAGGEEEEKKDEK
>	sp|P11505|AT2	Plasma membrane calcium-transporting ATPase 1 OS=Rattus norvegicus GN=Atp2b1 PE=2 SV=2	GGQVIQIPVADITVGDIAQVK
>	sp|P11505|AT2	Plasma membrane calcium-transporting ATPase 1 OS=Rattus norvegicus GN=Atp2b1 PE=2 SV=2	RGQILWFR
>	sp|P11505|AT2	Plasma membrane calcium-transporting ATPase 1 OS=Rattus norvegicus GN=Atp2b1 PE=2 SV=2	HTLVK
>	sp|P11505|AT2	Plasma membrane calcium-transporting ATPase 1 OS=Rattus norvegicus GN=Atp2b1 PE=2 SV=2	DFPAGEPEPEWDNENDVVTGLTCIAVVGIEDPVRPEVPEAIKK
>	sp|P11505|AT2	Plasma membrane calcium-transporting ATPase 1 OS=Rattus norvegicus GN=Atp2b1 PE=2 SV=2	AQDGAAMEMQPLKSEEGGDGDEKDKK
>	sp|P11505|AT2	Plasma membrane calcium-transporting ATPase 1 OS=Rattus norvegicus GN=Atp2b1 PE=2 SV=2	HLDACETMGNATAICSDK
>	sp|P11505|AT2	Plasma membrane calcium-transporting ATPase 1 OS=Rattus norvegicus GN=Atp2b1 PE=2 SV=2	TSPNEGLSGNPADLER
>	sp|P11505|AT2	Plasma membrane calcium-transporting ATPase 1 OS=Rattus norvegicus GN=Atp2b1 PE=2 SV=2	LAELR
>	sp|P11275|KCC	Calcium/calmodulin-dependent protein kinase type II subunit alpha OS=Rattus norvegicus GN=Camk2a PE=1 SV=1	DGKWQIVHFHR
>	sp|P11275|KCC	Calcium/calmodulin-dependent protein kinase type II subunit alpha OS=Rattus norvegicus GN=Camk2a PE=1 SV=1	DLKPENLLLASK
>	sp|P11275|KCC	Calcium/calmodulin-dependent protein kinase type II subunit alpha OS=Rattus norvegicus GN=Camk2a PE=1 SV=1	ESSESTNTTIEDEDTKVR
>	sp|P11275|KCC	Calcium/calmodulin-dependent protein kinase type II subunit alpha OS=Rattus norvegicus GN=Camk2a PE=1 SV=1	EYYSEADASHCIQQILEAVLHCHQMGVVHR
>	sp|P11275|KCC	Calcium/calmodulin-dependent protein kinase type II subunit alpha OS=Rattus norvegicus GN=Camk2a PE=1 SV=1	GAILTTMLATR
>	sp|P11275|KCC	Calcium/calmodulin-dependent protein kinase type II subunit alpha OS=Rattus norvegicus GN=Camk2a PE=1 SV=1	ITAAEALK
>	sp|P11275|KCC	Calcium/calmodulin-dependent protein kinase type II subunit alpha OS=Rattus norvegicus GN=Camk2a PE=1 SV=1	LKGAILTTMLATR
>	sp|P11275|KCC	Calcium/calmodulin-dependent protein kinase type II subunit alpha OS=Rattus norvegicus GN=Camk2a PE=1 SV=1	MCDPGMTAFEPEALGNLVEGLDFHR
>	sp|P11275|KCC	Calcium/calmodulin-dependent protein kinase type II subunit alpha OS=Rattus norvegicus GN=Camk2a PE=1 SV=1	VLAGQEYAAK
>	sp|P11275|KCC	Calcium/calmodulin-dependent protein kinase type II subunit alpha OS=Rattus norvegicus GN=Camk2a PE=1 SV=1	WQIVHFHR
>	sp|P11275|KCC	Calcium/calmodulin-dependent protein kinase type II subunit alpha OS=Rattus norvegicus GN=Camk2a PE=1 SV=1	VTEQLIEAISNGDFESYTK
>	sp|P11275|KCC	Calcium/calmodulin-dependent protein kinase type II subunit alpha OS=Rattus norvegicus GN=Camk2a PE=1 SV=1	AGAYDFPSPEWDTVTPEAK
>	sp|P11275|KCC	Calcium/calmodulin-dependent protein kinase type II subunit alpha OS=Rattus norvegicus GN=Camk2a PE=1 SV=1	ITQYLDAGGIPR

>	sp|P11275|KCC Calcium/calmodulin-dependent protein kinase type II subunit alpha OS=Rattus norvegicus GN=Camk2a PE=1 SV=1	KQEIIKVTEQLIEAISNGDFESYTK
>	sp|P11275|KCC Calcium/calmodulin-dependent protein kinase type II subunit alpha OS=Rattus norvegicus GN=Camk2a PE=1 SV=1	FTEEYQLFEELGK
>	sp|P11275|KCC Calcium/calmodulin-dependent protein kinase type II subunit alpha OS=Rattus norvegicus GN=Camk2a PE=1 SV=1	LEAVLHCHQMGVVHR
>	sp|P11275|KCC Calcium/calmodulin-dependent protein kinase type II subunit alpha OS=Rattus norvegicus GN=Camk2a PE=1 SV=1	AGAYDFPSPEWDTVTPEAK
>	sp|P11275|KCC Calcium/calmodulin-dependent protein kinase type II subunit alpha OS=Rattus norvegicus GN=Camk2a PE=1 SV=1	AGAYDFPSPEWDTVTPEAK
>	sp|P11275|KCC Calcium/calmodulin-dependent protein kinase type II subunit alpha OS=Rattus norvegicus GN=Camk2a PE=1 SV=1	AGAYDFPSPEWDTVTPEAK
>	sp|P11275|KCC Calcium/calmodulin-dependent protein kinase type II subunit alpha OS=Rattus norvegicus GN=Camk2a PE=1 SV=1	ESSESTNTTIEDEDTKVR
>	sp|P11275|KCC Calcium/calmodulin-dependent protein kinase type II subunit alpha OS=Rattus norvegicus GN=Camk2a PE=1 SV=1	EYYSEADASHCIQQILEAVLHCHQMGVVHR
>	sp|P11275|KCC Calcium/calmodulin-dependent protein kinase type II subunit alpha OS=Rattus norvegicus GN=Camk2a PE=1 SV=1	EYYSEADASHCIQQILEAVLHCHQMGVVHR
>	sp|P11275|KCC Calcium/calmodulin-dependent protein kinase type II subunit alpha OS=Rattus norvegicus GN=Camk2a PE=1 SV=1	EYYSEADASHCIQQILEAVLHCHQMGVVHR
>	sp|P11275|KCC Calcium/calmodulin-dependent protein kinase type II subunit alpha OS=Rattus norvegicus GN=Camk2a PE=1 SV=1	EYYSEADASHCIQQILEAVLHCHQMGVVHR
>	sp|P11275|KCC Calcium/calmodulin-dependent protein kinase type II subunit alpha OS=Rattus norvegicus GN=Camk2a PE=1 SV=1	EYYSEADASHCIQQILEAVLHCHQMGVVHR
>	sp|P11275|KCC Calcium/calmodulin-dependent protein kinase type II subunit alpha OS=Rattus norvegicus GN=Camk2a PE=1 SV=1	EYYSEADASHCIQQILEAVLHCHQMGVVHR
>	sp|P11275|KCC Calcium/calmodulin-dependent protein kinase type II subunit alpha OS=Rattus norvegicus GN=Camk2a PE=1 SV=1	EYYSEADASHCIQQILEAVLHCHQMGVVHR
>	sp|P11275|KCC Calcium/calmodulin-dependent protein kinase type II subunit alpha OS=Rattus norvegicus GN=Camk2a PE=1 SV=1	EYYSEADASHCIQQILEAVLHCHQMGVVHR
>	sp|P11275|KCC Calcium/calmodulin-dependent protein kinase type II subunit alpha OS=Rattus norvegicus GN=Camk2a PE=1 SV=1	EYYSEADASHCIQQILEAVLHCHQMGVVHR
>	sp|P11275|KCC Calcium/calmodulin-dependent protein kinase type II subunit alpha OS=Rattus norvegicus GN=Camk2a PE=1 SV=1	EYYSEADASHCIQQILEAVLHCHQMGVVHR
>	sp|P11275|KCC Calcium/calmodulin-dependent protein kinase type II subunit alpha OS=Rattus norvegicus GN=Camk2a PE=1 SV=1	EYYSEADASHCIQQILEAVLHCHQMGVVHR
>	sp|P11275|KCC Calcium/calmodulin-dependent protein kinase type II subunit alpha OS=Rattus norvegicus GN=Camk2a PE=1 SV=1	EYYSEADASHCIQQILEAVLHCHQMGVVHR
>	sp|P11275|KCC Calcium/calmodulin-dependent protein kinase type II subunit alpha OS=Rattus norvegicus GN=Camk2a PE=1 SV=1	EYYSEADASHCIQQILEAVLHCHQMGVVHR
>	sp|P11275|KCC Calcium/calmodulin-dependent protein kinase type II subunit alpha OS=Rattus norvegicus GN=Camk2a PE=1 SV=1	EYYSEADASHCIQQILEAVLHCHQMGVVHR
>	sp|P11275|KCC Calcium/calmodulin-dependent protein kinase type II subunit alpha OS=Rattus norvegicus GN=Camk2a PE=1 SV=1	EYYSEADASHCIQQILEAVLHCHQMGVVHR
>	sp|P11275|KCC Calcium/calmodulin-dependent protein kinase type II subunit alpha OS=Rattus norvegicus GN=Camk2a PE=1 SV=1	EYYSEADASHCIQQILEAVLHCHQMGVVHR
>	sp|P11275|KCC Calcium/calmodulin-dependent protein kinase type II subunit alpha OS=Rattus norvegicus GN=Camk2a PE=1 SV=1	EYYSEADASHCIQQILEAVLHCHQMGVVHR
>	sp|P11275|KCC Calcium/calmodulin-dependent protein kinase type II subunit alpha OS=Rattus norvegicus GN=Camk2a PE=1 SV=1	EYYSEADASHCIQQILEAVLHCHQMGVVHR
>	sp|P11275|KCC Calcium/calmodulin-dependent protein kinase type II subunit alpha OS=Rattus norvegicus GN=Camk2a PE=1 SV=1	EYYSEADASHCIQQILEAVLHCHQMGVVHR
>	sp|P11275|KCC Calcium/calmodulin-dependent protein kinase type II subunit alpha OS=Rattus norvegicus GN=Camk2a PE=1 SV=1	EYYSEADASHCIQQILEAVLHCHQMGVVHR
>	sp|P11275|KCC Calcium/calmodulin-dependent protein kinase type II subunit alpha OS=Rattus norvegicus GN=Camk2a PE=1 SV=1	EYYSEADASHCIQQILEAVLHCHQMGVVHR
>	sp|P11275|KCC Calcium/calmodulin-dependent protein kinase type II subunit alpha OS=Rattus norvegicus GN=Camk2a PE=1 SV=1	EYYSEADASHCIQQILEAVLHCHQMGVVHR
>	sp|P11275|KCC Calcium/calmodulin-dependent protein kinase type II subunit alpha OS=Rattus norvegicus GN=Camk2a PE=1 SV=1	EYYSEADASHCIQQILEAVLHCHQMGVVHR
>	sp|P11275|KCC Calcium/calmodulin-dependent protein kinase type II subunit alpha OS=Rattus norvegicus GN=Camk2a PE=1 SV=1	EYYSEADASHCIQQILEAVLHCHQMGVVHR
>	sp|P11275|KCC Calcium/calmodulin-dependent protein kinase type II subunit alpha OS=Rattus norvegicus GN=Camk2a PE=1 SV=1	EYYSEADASHCIQQILEAVLHCHQMGVVHR
>	sp|P11275|KCC Calcium/calmodulin-dependent protein kinase type II subunit alpha OS=Rattus norvegicus GN=Camk2a PE=1 SV=1	EYYSEADASHCIQQILEAVLHCHQMGVVHR
>	sp|P11275|KCC Calcium/calmodulin-dependent protein kinase type II subunit alpha OS=Rattus norvegicus GN=Camk2a PE=1 SV=1	EYYSEADASHCIQQILEAVLHCHQMGVVHR
>	sp|P11275|KCC Calcium/calmodulin-dependent protein kinase type II subunit alpha OS=Rattus norvegicus GN=Camk2a PE=1 SV=1	EYYSEADASHCIQQILEAVLHCHQMGVVHR
>	sp|P11275|KCC Calcium/calmodulin-dependent protein kinase type II subunit alpha OS=Rattus norvegicus GN=Camk2a PE=1 SV=1	EYYSEADASHCIQQILEAVLHCHQMGVVHR
>	sp|P11275|KCC Calcium/calmodulin-dependent protein kinase type II subunit alpha OS=Rattus norvegicus GN=Camk2a PE=1 SV=1	EYYSEADASHCIQQILEAVLHCHQMGVVHR
>	sp|P11275|KCC Calcium/calmodulin-dependent protein kinase type II subunit alpha OS=Rattus norvegicus GN=Camk2a PE=1 SV=1	EYYSEADASHCIQQILEAVLHCHQMGVVHR
>	sp|P11275|KCC Calcium/calmodulin-dependent protein kinase type II subunit alpha OS=Rattus norvegicus GN=Camk2a PE=1 SV=1	EYYSEADASHCIQQILEAVLHCHQMGVVHR
>	sp|P11275|KCC Calcium/calmodulin-dependent protein kinase type II subunit alpha OS=Rattus norvegicus GN=Camk2a PE=1 SV=1	EYYSEADASHCIQQILEAVLHCHQMGVVHR
>	sp|P11275|KCC Calcium/calmodulin-dependent protein kinase type II subunit alpha OS=Rattus norvegicus GN=Camk2a PE=1 SV=1	EYYSEADASHCIQQILEAVLHCHQMGVVHR
>	sp|P11275|KCC Calcium/calmodulin-dependent protein kinase type II subunit alpha OS=Rattus norvegicus GN=Camk2a PE=1 SV=1	EYYSEADASHCIQQILEAVLHCHQMGVVHR
>	sp|P11275|KCC Calcium/calmodulin-dependent protein kinase type II subunit alpha OS=Rattus norvegicus GN=Camk2a PE=1 SV=1	EYYSEADASHCIQQILEAVLHCHQMGVVHR
>	sp|P11275|KCC Calcium/calmodulin-dependent protein kinase type II subunit alpha OS=Rattus norvegicus GN=Camk2a PE=1 SV=1	EYYSEADASHCIQQILEAVLHCHQMGVVHR
>	sp|P11275|KCC Calcium/calmodulin-dependent protein kinase type II subunit alpha OS=Rattus norvegicus GN=Camk2a PE=1 SV=1	EYYSEADASHCIQQILEAVLHCHQMGVVHR
>	sp|P11275|KCC Calcium/calmodulin-dependent protein kinase type II subunit alpha OS=Rattus norvegicus GN=Camk2a PE=1 SV=1	EYYSEADASHCIQQILEAVLHCHQMGVVHR
>	sp|P11275|KCC Calcium/calmodulin-dependent protein kinase type II subunit alpha OS=Rattus norvegicus GN=Camk2a PE=1 SV=1	EYYSEADASHCIQQILEAVLHCHQMGVVHR
>	sp|P11275|KCC Calcium/calmodulin-dependent protein kinase type II subunit alpha OS=Rattus norvegicus GN=Camk2a PE=1 SV=1	EYYSEADASHCIQQILEAVLHCHQMGVVHR
>	sp|P11275|KCC Calcium/calmodulin-dependent protein kinase type II subunit alpha OS=Rattus norvegicus GN=Camk2a PE=1 SV=1	FTEEYQLFEELGK
>	sp|P11275|KCC Calcium/calmodulin-dependent protein kinase type II subunit alpha OS=Rattus norvegicus GN=Camk2a PE=1 SV=1	GAILTTMLATR
>	sp|P11275|KCC Calcium/calmodulin-dependent protein kinase type II subunit alpha OS=Rattus norvegicus GN=Camk2a PE=1 SV=1	GAILTTMLATR
>	sp|P11275|KCC Calcium/calmodulin-dependent protein kinase type II subunit alpha OS=Rattus norvegicus GN=Camk2a PE=1 SV=1	ITAAEALK
>	sp|P11275|KCC Calcium/calmodulin-dependent protein kinase type II subunit alpha OS=Rattus norvegicus GN=Camk2a PE=1 SV=1	ITAAEALK
>	sp|P11275|KCC Calcium/calmodulin-dependent protein kinase type II subunit alpha OS=Rattus norvegicus GN=Camk2a PE=1 SV=1	ITQYLDAGGIPR
>	sp|P11275|KCC Calcium/calmodulin-dependent protein kinase type II subunit alpha OS=Rattus norvegicus GN=Camk2a PE=1 SV=1	ITQYLDAGGIPR
>	sp|P11275|KCC Calcium/calmodulin-dependent protein kinase type II subunit alpha OS=Rattus norvegicus GN=Camk2a PE=1 SV=1	ITQYLDAGGIPR
>	sp|P11275|KCC Calcium/calmodulin-dependent protein kinase type II subunit alpha OS=Rattus norvegicus GN=Camk2a PE=1 SV=1	ITQYLDAGGIPR
>	sp|P11275|KCC Calcium/calmodulin-dependent protein kinase type II subunit alpha OS=Rattus norvegicus GN=Camk2a PE=1 SV=1	ITQYLDAGGIPR
>	sp|P11275|KCC Calcium/calmodulin-dependent protein kinase type II subunit alpha OS=Rattus norvegicus GN=Camk2a PE=1 SV=1	ITQYLDAGGIPR
>	sp|P11275|KCC Calcium/calmodulin-dependent protein kinase type II subunit alpha OS=Rattus norvegicus GN=Camk2a PE=1 SV=1	ITQYLDAGGIPR
>	sp|P11275|KCC Calcium/calmodulin-dependent protein kinase type II subunit alpha OS=Rattus norvegicus GN=Camk2a PE=1 SV=1	LEAVLHCHQMGVVHR
>	sp|P11275|KCC Calcium/calmodulin-dependent protein kinase type II subunit alpha OS=Rattus norvegicus GN=Camk2a PE=1 SV=1	LEAVLHCHQMGVVHR
>	sp|P11275|KCC Calcium/calmodulin-dependent protein kinase type II subunit alpha OS=Rattus norvegicus GN=Camk2a PE=1 SV=1	MCDPGMTAFEPEALGNLVEGLDFHR
>	sp|P11275|KCC Calcium/calmodulin-dependent protein kinase type II subunit alpha OS=Rattus norvegicus GN=Camk2a PE=1 SV=1	MCDPGMTAFEPEALGNLVEGLDFHR
>	sp|P11275|KCC Calcium/calmodulin-dependent protein kinase type II subunit alpha OS=Rattus norvegicus GN=Camk2a PE=1 SV=1	MCDPGMTAFEPEALGNLVEGLDFHR
>	sp|P11275|KCC Calcium/calmodulin-dependent protein kinase type II subunit alpha OS=Rattus norvegicus GN=Camk2a PE=1 SV=1	VTEQLIEAISNGDFESYTK
>	sp|P11275|KCC Calcium/calmodulin-dependent protein kinase type II subunit alpha OS=Rattus norvegicus GN=Camk2a PE=1 SV=1	VTEQLIEAISNGDFESYTK
>	sp|P11275|KCC Calcium/calmodulin-dependent protein kinase type II subunit alpha OS=Rattus norvegicus GN=Camk2a PE=1 SV=1	VTEQLIEAISNGDFESYTK
>	sp|P11275|KCC Calcium/calmodulin-dependent protein kinase type II subunit alpha OS=Rattus norvegicus GN=Camk2a PE=1 SV=1	VTEQLIEAISNGDFESYTK
>	sp|P11275|KCC Calcium/calmodulin-dependent protein kinase type II subunit alpha OS=Rattus norvegicus GN=Camk2a PE=1 SV=1	VTEQLIEAISNGDFESYTK
>	sp|P11275|KCC Calcium/calmodulin-dependent protein kinase type II subunit alpha OS=Rattus norvegicus GN=Camk2a PE=1 SV=1	VTEQLIEAISNGDFESYTK
>	sp|P11275|KCC Calcium/calmodulin-dependent protein kinase type II subunit alpha OS=Rattus norvegicus GN=Camk2a PE=1 SV=1	VTEQLIEAISNGDFESYTK
>	sp|P11275|KCC Calcium/calmodulin-dependent protein kinase type II subunit alpha OS=Rattus norvegicus GN=Camk2a PE=1 SV=1	ITQYLDAGGIPR
>	sp|P11275|KCC Calcium/calmodulin-dependent protein kinase type II subunit alpha OS=Rattus norvegicus GN=Camk2a PE=1 SV=1	FTEEYQLFEELGK

>	sp|P11275|KCC	Calcium/calmodulin-dependent protein kinase type II subunit alpha OS=Rattus norvegicus GN=Camk2a PE=1 SV=1	ITQYLDAGGIPR
>	sp|P11275|KCC	Calcium/calmodulin-dependent protein kinase type II subunit alpha OS=Rattus norvegicus GN=Camk2a PE=1 SV=1	HPWISHR
>	sp|P11275|KCC	Calcium/calmodulin-dependent protein kinase type II subunit alpha OS=Rattus norvegicus GN=Camk2a PE=1 SV=1	VTEQLIEAISNGDFESYTK
>	sp|P11275|KCC	Calcium/calmodulin-dependent protein kinase type II subunit alpha OS=Rattus norvegicus GN=Camk2a PE=1 SV=1	EYYSEADASHCIQQILEAVLHCHQMGVVHR
>	sp|P11275|KCC	Calcium/calmodulin-dependent protein kinase type II subunit alpha OS=Rattus norvegicus GN=Camk2a PE=1 SV=1	EYYSEADASHCIQQILEAVLHCHQMGVVHR
>	sp|P11275|KCC	Calcium/calmodulin-dependent protein kinase type II subunit alpha OS=Rattus norvegicus GN=Camk2a PE=1 SV=1	ITQYLDAGGIPR
>	sp|P11275|KCC	Calcium/calmodulin-dependent protein kinase type II subunit alpha OS=Rattus norvegicus GN=Camk2a PE=1 SV=1	FTEEYQLFEELGK
>	sp|P11275|KCC	Calcium/calmodulin-dependent protein kinase type II subunit alpha OS=Rattus norvegicus GN=Camk2a PE=1 SV=1	ESSESTNTTIEDEDTKVR
>	sp|P11275|KCC	Calcium/calmodulin-dependent protein kinase type II subunit alpha OS=Rattus norvegicus GN=Camk2a PE=1 SV=1	VLAGQEYAAK
>	sp|P11275|KCC	Calcium/calmodulin-dependent protein kinase type II subunit alpha OS=Rattus norvegicus GN=Camk2a PE=1 SV=1	FTEEYQLFEELGK
>	sp|P11275|KCC	Calcium/calmodulin-dependent protein kinase type II subunit alpha OS=Rattus norvegicus GN=Camk2a PE=1 SV=1	VLAGQEYAAK
>	sp|P11275|KCC	Calcium/calmodulin-dependent protein kinase type II subunit alpha OS=Rattus norvegicus GN=Camk2a PE=1 SV=1	QETVDCLKK
>	sp|P11275|KCC	Calcium/calmodulin-dependent protein kinase type II subunit alpha OS=Rattus norvegicus GN=Camk2a PE=1 SV=1	VTEQLIEAISNGDFESYTK
>	sp|P11275|KCC	Calcium/calmodulin-dependent protein kinase type II subunit alpha OS=Rattus norvegicus GN=Camk2a PE=1 SV=1	VTEQLIEAISNGDFESYTK
>	sp|P11275|KCC	Calcium/calmodulin-dependent protein kinase type II subunit alpha OS=Rattus norvegicus GN=Camk2a PE=1 SV=1	AGAYDFPSPEWDTVTPEAK
>	sp|P11275|KCC	Calcium/calmodulin-dependent protein kinase type II subunit alpha OS=Rattus norvegicus GN=Camk2a PE=1 SV=1	DLKPENLLLASK
>	sp|P11275|KCC	Calcium/calmodulin-dependent protein kinase type II subunit alpha OS=Rattus norvegicus GN=Camk2a PE=1 SV=1	VTEQLIEAISNGDFESYTK
>	sp|P11275|KCC	Calcium/calmodulin-dependent protein kinase type II subunit alpha OS=Rattus norvegicus GN=Camk2a PE=1 SV=1	EYYSEADASHCIQQILEAVLHCHQMGVVHR
>	sp|P11275|KCC	Calcium/calmodulin-dependent protein kinase type II subunit alpha OS=Rattus norvegicus GN=Camk2a PE=1 SV=1	EYYSEADASHCIQQILEAVLHCHQMGVVHR
>	sp|P11275|KCC	Calcium/calmodulin-dependent protein kinase type II subunit alpha OS=Rattus norvegicus GN=Camk2a PE=1 SV=1	MCDPGMTAFEPEALGNLVEGLDFHR
>	sp|P11275|KCC	Calcium/calmodulin-dependent protein kinase type II subunit alpha OS=Rattus norvegicus GN=Camk2a PE=1 SV=1	EYYSEADASHCIQQILEAVLHCHQMGVVHR
>	sp|P11275|KCC	Calcium/calmodulin-dependent protein kinase type II subunit alpha OS=Rattus norvegicus GN=Camk2a PE=1 SV=1	EYYSEADASHCIQQILEAVLHCHQMGVVHR
>	sp|P11275|KCC	Calcium/calmodulin-dependent protein kinase type II subunit alpha OS=Rattus norvegicus GN=Camk2a PE=1 SV=1	VLAGQEYAAK
>	sp|P11275|KCC	Calcium/calmodulin-dependent protein kinase type II subunit alpha OS=Rattus norvegicus GN=Camk2a PE=1 SV=1	LKGAAVK
>	sp|P11275|KCC	Calcium/calmodulin-dependent protein kinase type II subunit alpha OS=Rattus norvegicus GN=Camk2a PE=1 SV=1	ITAAEALK
>	sp|P11275|KCC	Calcium/calmodulin-dependent protein kinase type II subunit alpha OS=Rattus norvegicus GN=Camk2a PE=1 SV=1	EYYSEADASHCIQQILEAVLHCHQMGVVHR
>	sp|P11275|KCC	Calcium/calmodulin-dependent protein kinase type II subunit alpha OS=Rattus norvegicus GN=Camk2a PE=1 SV=1	VTEQLIEAISNGDFESYTK
>	sp|P11275|KCC	Calcium/calmodulin-dependent protein kinase type II subunit alpha OS=Rattus norvegicus GN=Camk2a PE=1 SV=1	GGELFEDIVAREYYSEADASHCIQQILEAVLHCHQMGVVHR
>	sp|P11275|KCC	Calcium/calmodulin-dependent protein kinase type II subunit alpha OS=Rattus norvegicus GN=Camk2a PE=1 SV=1	VTEQLIEAISNGDFESYTK
>	sp|P11275|KCC	Calcium/calmodulin-dependent protein kinase type II subunit alpha OS=Rattus norvegicus GN=Camk2a PE=1 SV=1	DLKPENLLLASK
>	sp|P11275|KCC	Calcium/calmodulin-dependent protein kinase type II subunit alpha OS=Rattus norvegicus GN=Camk2a PE=1 SV=1	EYYSEADASHCIQQILEAVLHCHQMGVVHR
>	sp|P11275|KCC	Calcium/calmodulin-dependent protein kinase type II subunit alpha OS=Rattus norvegicus GN=Camk2a PE=1 SV=1	GGELFEDIVAREYYSEADASHCIQQILEAVLHCHQMGVVHR
>	sp|P11275|KCC	Calcium/calmodulin-dependent protein kinase type II subunit alpha OS=Rattus norvegicus GN=Camk2a PE=1 SV=1	VLAGQEYAAK
>	sp|P11275|KCC	Calcium/calmodulin-dependent protein kinase type II subunit alpha OS=Rattus norvegicus GN=Camk2a PE=1 SV=1	EYYSEADASHCIQQILEAVLHCHQMGVVHR
>	sp|P11275|KCC	Calcium/calmodulin-dependent protein kinase type II subunit alpha OS=Rattus norvegicus GN=Camk2a PE=1 SV=1	EYYSEADASHCIQQILEAVLHCHQMGVVHR
>	sp|P11275|KCC	Calcium/calmodulin-dependent protein kinase type II subunit alpha OS=Rattus norvegicus GN=Camk2a PE=1 SV=1	LLKHPNIVR
>	sp|P11275|KCC	Calcium/calmodulin-dependent protein kinase type II subunit alpha OS=Rattus norvegicus GN=Camk2a PE=1 SV=1	MCDPGMTAFEPEALGNLVEGLDFHR
>	sp|P11275|KCC	Calcium/calmodulin-dependent protein kinase type II subunit alpha OS=Rattus norvegicus GN=Camk2a PE=1 SV=1	FYFENLWSR
>	sp|P11275|KCC	Calcium/calmodulin-dependent protein kinase type II subunit alpha OS=Rattus norvegicus GN=Camk2a PE=1 SV=1	EYYSEADASHCIQQILEAVLHCHQMGVVHR
>	sp|P11275|KCC	Calcium/calmodulin-dependent protein kinase type II subunit alpha OS=Rattus norvegicus GN=Camk2a PE=1 SV=1	VTEQLIEAISNGDFESYTK
>	sp|P11275|KCC	Calcium/calmodulin-dependent protein kinase type II subunit alpha OS=Rattus norvegicus GN=Camk2a PE=1 SV=1	VLAGQEYAAK
>	sp|P11275|KCC	Calcium/calmodulin-dependent protein kinase type II subunit alpha OS=Rattus norvegicus GN=Camk2a PE=1 SV=1	VLAGQEYAAK
>	sp|P11275|KCC	Calcium/calmodulin-dependent protein kinase type II subunit alpha OS=Rattus norvegicus GN=Camk2a PE=1 SV=1	FTEEYQLFEELGK
>	sp|P11275|KCC	Calcium/calmodulin-dependent protein kinase type II subunit alpha OS=Rattus norvegicus GN=Camk2a PE=1 SV=1	LIFDLVTGGELFEDIVAREYYSEADASHCIQQILEAVLHCHQMGVVHR
>	sp|P11275|KCC	Calcium/calmodulin-dependent protein kinase type II subunit alpha OS=Rattus norvegicus GN=Camk2a PE=1 SV=1	DLKPENLLLASK
>	sp|P11275|KCC	Calcium/calmodulin-dependent protein kinase type II subunit alpha OS=Rattus norvegicus GN=Camk2a PE=1 SV=1	DLKPENLLLASK
>	sp|P11275|KCC	Calcium/calmodulin-dependent protein kinase type II subunit alpha OS=Rattus norvegicus GN=Camk2a PE=1 SV=1	EYYSEADASHCIQQILEAVLHCHQMGVVHR
>	sp|P11275|KCC	Calcium/calmodulin-dependent protein kinase type II subunit alpha OS=Rattus norvegicus GN=Camk2a PE=1 SV=1	VLAGQEYAAK
>	sp|P11275|KCC	Calcium/calmodulin-dependent protein kinase type II subunit alpha OS=Rattus norvegicus GN=Camk2a PE=1 SV=1	LLKHPNIVR
>	sp|P11275|KCC	Calcium/calmodulin-dependent protein kinase type II subunit alpha OS=Rattus norvegicus GN=Camk2a PE=1 SV=1	DLKPENLLLASK
>	sp|P11275|KCC	Calcium/calmodulin-dependent protein kinase type II subunit alpha OS=Rattus norvegicus GN=Camk2a PE=1 SV=1	ITQYLDAGGIPR
>	sp|P82995|HS9	Heat shock protein HSP 90-alpha OS=Rattus norvegicus GN=Hsp90aa1 PE=1 SV=3	ADLINNLGTIAK
>	sp|P82995|HS9	Heat shock protein HSP 90-alpha OS=Rattus norvegicus GN=Hsp90aa1 PE=1 SV=3	GVVDSEDLPLNISR
>	sp|P82995|HS9	Heat shock protein HSP 90-alpha OS=Rattus norvegicus GN=Hsp90aa1 PE=1 SV=3	HLEINPDHSIIETLR
>	sp|P82995|HS9	Heat shock protein HSP 90-alpha OS=Rattus norvegicus GN=Hsp90aa1 PE=1 SV=3	HNDDEQYAWESSAGGSFTVR
>	sp|P82995|HS9	Heat shock protein HSP 90-alpha OS=Rattus norvegicus GN=Hsp90aa1 PE=1 SV=3	KCLELFTELAEDKENYKK
>	sp|P82995|HS9	Heat shock protein HSP 90-alpha OS=Rattus norvegicus GN=Hsp90aa1 PE=1 SV=3	KHLEINPDHSIIETLR
>	sp|P82995|HS9	Heat shock protein HSP 90-alpha OS=Rattus norvegicus GN=Hsp90aa1 PE=1 SV=3	KHSQFIGYPITLFVEK
>	sp|P82995|HS9	Heat shock protein HSP 90-alpha OS=Rattus norvegicus GN=Hsp90aa1 PE=1 SV=3	QKAEADKNDKSVKDLVILLYETALLSSGFSLEDPQTHANR
>	sp|P82995|HS9	Heat shock protein HSP 90-alpha OS=Rattus norvegicus GN=Hsp90aa1 PE=1 SV=3	SVKDLVILLYETALLSSGFSLEDPQTHANR
>	sp|P82995|HS9	Heat shock protein HSP 90-alpha OS=Rattus norvegicus GN=Hsp90aa1 PE=1 SV=3	TLTIVDTGIGMTK
>	sp|P82995|HS9	Heat shock protein HSP 90-alpha OS=Rattus norvegicus GN=Hsp90aa1 PE=1 SV=3	RAPFDLFENR
>	sp|P82995|HS9	Heat shock protein HSP 90-alpha OS=Rattus norvegicus GN=Hsp90aa1 PE=1 SV=3	ELHINLIPNKQDR
>	sp|P82995|HS9	Heat shock protein HSP 90-alpha OS=Rattus norvegicus GN=Hsp90aa1 PE=1 SV=3	LVTSPCCIVTSTYGWTANMER
>	sp|P82995|HS9	Heat shock protein HSP 90-alpha OS=Rattus norvegicus GN=Hsp90aa1 PE=1 SV=3	CLELFTELAEDKENYKK
>	sp|P82995|HS9	Heat shock protein HSP 90-alpha OS=Rattus norvegicus GN=Hsp90aa1 PE=1 SV=3	HSQFIGYPITLFVEK
>	sp|P82995|HS9	Heat shock protein HSP 90-alpha OS=Rattus norvegicus GN=Hsp90aa1 PE=1 SV=3	VILHLKEDQTEYLEER
>	sp|P82995|HS9	Heat shock protein HSP 90-alpha OS=Rattus norvegicus GN=Hsp90aa1 PE=1 SV=3	GVVDSEDLPLNISR
>	sp|P82995|HS9	Heat shock protein HSP 90-alpha OS=Rattus norvegicus GN=Hsp90aa1 PE=1 SV=3	GVVDSEDLPLNISR
>	sp|P82995|HS9	Heat shock protein HSP 90-alpha OS=Rattus norvegicus GN=Hsp90aa1 PE=1 SV=3	HLEINPDHSIIETLR
>	sp|P82995|HS9	Heat shock protein HSP 90-alpha OS=Rattus norvegicus GN=Hsp90aa1 PE=1 SV=3	HNDDEQYAWESSAGGSFTVR
>	sp|P82995|HS9	Heat shock protein HSP 90-alpha OS=Rattus norvegicus GN=Hsp90aa1 PE=1 SV=3	HNDDEQYAWESSAGGSFTVR
>	sp|P82995|HS9	Heat shock protein HSP 90-alpha OS=Rattus norvegicus GN=Hsp90aa1 PE=1 SV=3	HNDDEQYAWESSAGGSFTVR
>	sp|P82995|HS9	Heat shock protein HSP 90-alpha OS=Rattus norvegicus GN=Hsp90aa1 PE=1 SV=3	KHLEINPDHSIIETLR

>	sp|P82995|HS9	Heat shock protein HSP 90-alpha OS=Rattus norvegicus GN=Hsp90aa1 PE=1 SV=3	KHSQFIGYPITLFVEK
>	sp|P82995|HS9	Heat shock protein HSP 90-alpha OS=Rattus norvegicus GN=Hsp90aa1 PE=1 SV=3	RAPFDLFENR
>	sp|P82995|HS9	Heat shock protein HSP 90-alpha OS=Rattus norvegicus GN=Hsp90aa1 PE=1 SV=3	RAPFDLFENR
>	sp|P82995|HS9	Heat shock protein HSP 90-alpha OS=Rattus norvegicus GN=Hsp90aa1 PE=1 SV=3	VILHLKEDQTEYLEER
>	sp|P82995|HS9	Heat shock protein HSP 90-alpha OS=Rattus norvegicus GN=Hsp90aa1 PE=1 SV=3	ADLINNLGTIAK
>	sp|P82995|HS9	Heat shock protein HSP 90-alpha OS=Rattus norvegicus GN=Hsp90aa1 PE=1 SV=3	LGIHEDSQNR
>	sp|P82995|HS9	Heat shock protein HSP 90-alpha OS=Rattus norvegicus GN=Hsp90aa1 PE=1 SV=3	KHGLEVIYMIEPIDEYCVQQLK
>	sp|P82995|HS9	Heat shock protein HSP 90-alpha OS=Rattus norvegicus GN=Hsp90aa1 PE=1 SV=3	ADLINNLGTIAK
>	sp|P82995|HS9	Heat shock protein HSP 90-alpha OS=Rattus norvegicus GN=Hsp90aa1 PE=1 SV=3	VILHLKEDQTEYLEER
>	sp|P82995|HS9	Heat shock protein HSP 90-alpha OS=Rattus norvegicus GN=Hsp90aa1 PE=1 SV=3	GTKVILHLKEDQTEYLEER
>	sp|P82995|HS9	Heat shock protein HSP 90-alpha OS=Rattus norvegicus GN=Hsp90aa1 PE=1 SV=3	YIDQEELNK
>	sp|P82995|HS9	Heat shock protein HSP 90-alpha OS=Rattus norvegicus GN=Hsp90aa1 PE=1 SV=3	RVFIMDNCEELIPEYLNFIR
>	sp|P82995|HS9	Heat shock protein HSP 90-alpha OS=Rattus norvegicus GN=Hsp90aa1 PE=1 SV=3	RAPFDLFENR
>	sp|P82995|HS9	Heat shock protein HSP 90-alpha OS=Rattus norvegicus GN=Hsp90aa1 PE=1 SV=3	KHGLEVIYMIEPIDEYCVQQLK
>	sp|P82995|HS9	Heat shock protein HSP 90-alpha OS=Rattus norvegicus GN=Hsp90aa1 PE=1 SV=3	ELHINLIPNKQDR
>	sp|P82995|HS9	Heat shock protein HSP 90-alpha OS=Rattus norvegicus GN=Hsp90aa1 PE=1 SV=3	ADLINNLGTIAK
>	sp|P82995|HS9	Heat shock protein HSP 90-alpha OS=Rattus norvegicus GN=Hsp90aa1 PE=1 SV=3	YIDQEELNK
>	sp|P82995|HS9	Heat shock protein HSP 90-alpha OS=Rattus norvegicus GN=Hsp90aa1 PE=1 SV=3	RAPFDLFENR
>	sp|P82995|HS9	Heat shock protein HSP 90-alpha OS=Rattus norvegicus GN=Hsp90aa1 PE=1 SV=3	LSELLR
>	sp|P82995|HS9	Heat shock protein HSP 90-alpha OS=Rattus norvegicus GN=Hsp90aa1 PE=1 SV=3	ALLFVPR
>	sp|P82995|HS9	Heat shock protein HSP 90-alpha OS=Rattus norvegicus GN=Hsp90aa1 PE=1 SV=3	YIDQEELNK
>	sp|P82995|HS9	Heat shock protein HSP 90-alpha OS=Rattus norvegicus GN=Hsp90aa1 PE=1 SV=3	LVTSPCCIVTSTYGWTANMER
>	sp|P82995|HS9	Heat shock protein HSP 90-alpha OS=Rattus norvegicus GN=Hsp90aa1 PE=1 SV=3	HLEINPDHSIIETLR
>	sp|P82995|HS9	Heat shock protein HSP 90-alpha OS=Rattus norvegicus GN=Hsp90aa1 PE=1 SV=3	YIDQEELNK
>	sp|P82995|HS9	Heat shock protein HSP 90-alpha OS=Rattus norvegicus GN=Hsp90aa1 PE=1 SV=3	HGLEVIYMIEPIDEYCVQQLK
>	sp|P82995|HS9	Heat shock protein HSP 90-alpha OS=Rattus norvegicus GN=Hsp90aa1 PE=1 SV=3	KHGLEVIYMIEPIDEYCVQQLK
>	sp|P82995|HS9	Heat shock protein HSP 90-alpha OS=Rattus norvegicus GN=Hsp90aa1 PE=1 SV=3	YIDQEELNK
>	sp|P47942|DPY	Dihydropyrimidinase-related protein 2 OS=Rattus norvegicus GN=Dpysl2 PE=1 SV=1	AALAGGTTMIIDHVVPEPGTSLLAAFDQWR
>	sp|P47942|DPY	Dihydropyrimidinase-related protein 2 OS=Rattus norvegicus GN=Dpysl2 PE=1 SV=1	DNFTLIPEGTNGTEER
>	sp|P47942|DPY	Dihydropyrimidinase-related protein 2 OS=Rattus norvegicus GN=Dpysl2 PE=1 SV=1	GIQEEMEALVK
>	sp|P47942|DPY	Dihydropyrimidinase-related protein 2 OS=Rattus norvegicus GN=Dpysl2 PE=1 SV=1	GLYDGPVCEVSVTPK
>	sp|P47942|DPY	Dihydropyrimidinase-related protein 2 OS=Rattus norvegicus GN=Dpysl2 PE=1 SV=1	GSPLVVISQGK
>	sp|P47942|DPY	Dihydropyrimidinase-related protein 2 OS=Rattus norvegicus GN=Dpysl2 PE=1 SV=1	ILDLGITGPEGHVLSRPEEVEAEAVNR
>	sp|P47942|DPY	Dihydropyrimidinase-related protein 2 OS=Rattus norvegicus GN=Dpysl2 PE=1 SV=1	ISVGSDADLVIWDPDSVK
>	sp|P47942|DPY	Dihydropyrimidinase-related protein 2 OS=Rattus norvegicus GN=Dpysl2 PE=1 SV=1	IVLEDGTLHVTEGSGR
>	sp|P47942|DPY	Dihydropyrimidinase-related protein 2 OS=Rattus norvegicus GN=Dpysl2 PE=1 SV=1	MDENQFVAVTSTNAAK
>	sp|P47942|DPY	Dihydropyrimidinase-related protein 2 OS=Rattus norvegicus GN=Dpysl2 PE=1 SV=1	QIGENLIVPGGVK
>	sp|P47942|DPY	Dihydropyrimidinase-related protein 2 OS=Rattus norvegicus GN=Dpysl2 PE=1 SV=1	SITIANQTNCPLYVTK
>	sp|P47942|DPY	Dihydropyrimidinase-related protein 2 OS=Rattus norvegicus GN=Dpysl2 PE=1 SV=1	THNSALEYNIFEGMECR
>	sp|P47942|DPY	Dihydropyrimidinase-related protein 2 OS=Rattus norvegicus GN=Dpysl2 PE=1 SV=1	AVGKDNFTLIPEGTNGTEER
>	sp|P47942|DPY	Dihydropyrimidinase-related protein 2 OS=Rattus norvegicus GN=Dpysl2 PE=1 SV=1	VFNLYPR
>	sp|P47942|DPY	Dihydropyrimidinase-related protein 2 OS=Rattus norvegicus GN=Dpysl2 PE=1 SV=1	PGGIDVHTR
>	sp|P47942|DPY	Dihydropyrimidinase-related protein 2 OS=Rattus norvegicus GN=Dpysl2 PE=1 SV=1	SAAEVIAQAR
>	sp|P47942|DPY	Dihydropyrimidinase-related protein 2 OS=Rattus norvegicus GN=Dpysl2 PE=1 SV=1	MVIPGGIDVHTR
>	sp|P47942|DPY	Dihydropyrimidinase-related protein 2 OS=Rattus norvegicus GN=Dpysl2 PE=1 SV=1	AAAFVTSPPLSPDPTTPDFLNSLLSCGDLQVTGSAHCTFNTAQK
>	sp|P47942|DPY	Dihydropyrimidinase-related protein 2 OS=Rattus norvegicus GN=Dpysl2 PE=1 SV=1	AALAGGTTMIIDHVVPEPGTSLLAAFDQWREWADSK
>	sp|P47942|DPY	Dihydropyrimidinase-related protein 2 OS=Rattus norvegicus GN=Dpysl2 PE=1 SV=1	AAAFVTSPPLSPDPTTPDFLNSLLSCGDLQVTGSAHCTFNTAQK
>	sp|P47942|DPY	Dihydropyrimidinase-related protein 2 OS=Rattus norvegicus GN=Dpysl2 PE=1 SV=1	AAAFVTSPPLSPDPTTPDFLNSLLSCGDLQVTGSAHCTFNTAQK
>	sp|P47942|DPY	Dihydropyrimidinase-related protein 2 OS=Rattus norvegicus GN=Dpysl2 PE=1 SV=1	AAAFVTSPPLSPDPTTPDFLNSLLSCGDLQVTGSAHCTFNTAQK
>	sp|P47942|DPY	Dihydropyrimidinase-related protein 2 OS=Rattus norvegicus GN=Dpysl2 PE=1 SV=1	AAAFVTSPPLSPDPTTPDFLNSLLSCGDLQVTGSAHCTFNTAQK
>	sp|P47942|DPY	Dihydropyrimidinase-related protein 2 OS=Rattus norvegicus GN=Dpysl2 PE=1 SV=1	AALAGGTTMIIDHVVPEPGTSLLAAFDQWR
>	sp|P47942|DPY	Dihydropyrimidinase-related protein 2 OS=Rattus norvegicus GN=Dpysl2 PE=1 SV=1	AALAGGTTMIIDHVVPEPGTSLLAAFDQWR
>	sp|P47942|DPY	Dihydropyrimidinase-related protein 2 OS=Rattus norvegicus GN=Dpysl2 PE=1 SV=1	AVGKDNFTLIPEGTNGTEER
>	sp|P47942|DPY	Dihydropyrimidinase-related protein 2 OS=Rattus norvegicus GN=Dpysl2 PE=1 SV=1	DNFTLIPEGTNGTEER
>	sp|P47942|DPY	Dihydropyrimidinase-related protein 2 OS=Rattus norvegicus GN=Dpysl2 PE=1 SV=1	GIQEEMEALVK
>	sp|P47942|DPY	Dihydropyrimidinase-related protein 2 OS=Rattus norvegicus GN=Dpysl2 PE=1 SV=1	GLYDGPVCEVSVTPK
>	sp|P47942|DPY	Dihydropyrimidinase-related protein 2 OS=Rattus norvegicus GN=Dpysl2 PE=1 SV=1	GLYDGPVCEVSVTPK
>	sp|P47942|DPY	Dihydropyrimidinase-related protein 2 OS=Rattus norvegicus GN=Dpysl2 PE=1 SV=1	GSPLVVISQGK
>	sp|P47942|DPY	Dihydropyrimidinase-related protein 2 OS=Rattus norvegicus GN=Dpysl2 PE=1 SV=1	ILDLGITGPEGHVLSRPEEVEAEAVNR
>	sp|P47942|DPY	Dihydropyrimidinase-related protein 2 OS=Rattus norvegicus GN=Dpysl2 PE=1 SV=1	ILDLGITGPEGHVLSRPEEVEAEAVNR
>	sp|P47942|DPY	Dihydropyrimidinase-related protein 2 OS=Rattus norvegicus GN=Dpysl2 PE=1 SV=1	ILDLGITGPEGHVLSRPEEVEAEAVNR
>	sp|P47942|DPY	Dihydropyrimidinase-related protein 2 OS=Rattus norvegicus GN=Dpysl2 PE=1 SV=1	ILDLGITGPEGHVLSRPEEVEAEAVNR
>	sp|P47942|DPY	Dihydropyrimidinase-related protein 2 OS=Rattus norvegicus GN=Dpysl2 PE=1 SV=1	ILDLGITGPEGHVLSRPEEVEAEAVNR
>	sp|P47942|DPY	Dihydropyrimidinase-related protein 2 OS=Rattus norvegicus GN=Dpysl2 PE=1 SV=1	ILDLGITGPEGHVLSRPEEVEAEAVNR
>	sp|P47942|DPY	Dihydropyrimidinase-related protein 2 OS=Rattus norvegicus GN=Dpysl2 PE=1 SV=1	ILDLGITGPEGHVLSRPEEVEAEAVNR
>	sp|P47942|DPY	Dihydropyrimidinase-related protein 2 OS=Rattus norvegicus GN=Dpysl2 PE=1 SV=1	ILDLGITGPEGHVLSRPEEVEAEAVNR
>	sp|P47942|DPY	Dihydropyrimidinase-related protein 2 OS=Rattus norvegicus GN=Dpysl2 PE=1 SV=1	ILDLGITGPEGHVLSRPEEVEAEAVNR
>	sp|P47942|DPY	Dihydropyrimidinase-related protein 2 OS=Rattus norvegicus GN=Dpysl2 PE=1 SV=1	ILDLGITGPEGHVLSRPEEVEAEAVNR
>	sp|P47942|DPY	Dihydropyrimidinase-related protein 2 OS=Rattus norvegicus GN=Dpysl2 PE=1 SV=1	ILDLGITGPEGHVLSRPEEVEAEAVNR
>	sp|P47942|DPY	Dihydropyrimidinase-related protein 2 OS=Rattus norvegicus GN=Dpysl2 PE=1 SV=1	ILDLGITGPEGHVLSRPEEVEAEAVNR
>	sp|P47942|DPY	Dihydropyrimidinase-related protein 2 OS=Rattus norvegicus GN=Dpysl2 PE=1 SV=1	ILDLGITGPEGHVLSRPEEVEAEAVNR
>	sp|P47942|DPY	Dihydropyrimidinase-related protein 2 OS=Rattus norvegicus GN=Dpysl2 PE=1 SV=1	ILDLGITGPEGHVLSRPEEVEAEAVNR
>	sp|P47942|DPY	Dihydropyrimidinase-related protein 2 OS=Rattus norvegicus GN=Dpysl2 PE=1 SV=1	ILDLGITGPEGHVLSRPEEVEAEAVNR
>	sp|P47942|DPY	Dihydropyrimidinase-related protein 2 OS=Rattus norvegicus GN=Dpysl2 PE=1 SV=1	ILDLGITGPEGHVLSRPEEVEAEAVNR

>	sp|P47942|DPY	Dihydropyrimidinase-related protein 2 OS=Rattus norvegicus GN=Dpysl2 PE=1 SV=1	ISVGSDADLVIWDPDSVK
>	sp|P47942|DPY	Dihydropyrimidinase-related protein 2 OS=Rattus norvegicus GN=Dpysl2 PE=1 SV=1	ISVGSDADLVIWDPDSVK
>	sp|P47942|DPY	Dihydropyrimidinase-related protein 2 OS=Rattus norvegicus GN=Dpysl2 PE=1 SV=1	ISVGSDADLVIWDPDSVK
>	sp|P47942|DPY	Dihydropyrimidinase-related protein 2 OS=Rattus norvegicus GN=Dpysl2 PE=1 SV=1	ISVGSDADLVIWDPDSVK
>	sp|P47942|DPY	Dihydropyrimidinase-related protein 2 OS=Rattus norvegicus GN=Dpysl2 PE=1 SV=1	MDENQFVAVTSTNAAK
>	sp|P47942|DPY	Dihydropyrimidinase-related protein 2 OS=Rattus norvegicus GN=Dpysl2 PE=1 SV=1	MDENQFVAVTSTNAAK
>	sp|P47942|DPY	Dihydropyrimidinase-related protein 2 OS=Rattus norvegicus GN=Dpysl2 PE=1 SV=1	MDENQFVAVTSTNAAK
>	sp|P47942|DPY	Dihydropyrimidinase-related protein 2 OS=Rattus norvegicus GN=Dpysl2 PE=1 SV=1	MVIPGGIDVHTR
>	sp|P47942|DPY	Dihydropyrimidinase-related protein 2 OS=Rattus norvegicus GN=Dpysl2 PE=1 SV=1	MVIPGGIDVHTR
>	sp|P47942|DPY	Dihydropyrimidinase-related protein 2 OS=Rattus norvegicus GN=Dpysl2 PE=1 SV=1	MVIPGGIDVHTR
>	sp|P47942|DPY	Dihydropyrimidinase-related protein 2 OS=Rattus norvegicus GN=Dpysl2 PE=1 SV=1	MVIPGGIDVHTR
>	sp|P47942|DPY	Dihydropyrimidinase-related protein 2 OS=Rattus norvegicus GN=Dpysl2 PE=1 SV=1	MVIPGGIDVHTR
>	sp|P47942|DPY	Dihydropyrimidinase-related protein 2 OS=Rattus norvegicus GN=Dpysl2 PE=1 SV=1	QIGENLIVPGGVK
>	sp|P47942|DPY	Dihydropyrimidinase-related protein 2 OS=Rattus norvegicus GN=Dpysl2 PE=1 SV=1	QIGENLIVPGGVK
>	sp|P47942|DPY	Dihydropyrimidinase-related protein 2 OS=Rattus norvegicus GN=Dpysl2 PE=1 SV=1	SAAEVIAQAR
>	sp|P47942|DPY	Dihydropyrimidinase-related protein 2 OS=Rattus norvegicus GN=Dpysl2 PE=1 SV=1	SAAEVIAQAR
>	sp|P47942|DPY	Dihydropyrimidinase-related protein 2 OS=Rattus norvegicus GN=Dpysl2 PE=1 SV=1	SAAEVIAQAR
>	sp|P47942|DPY	Dihydropyrimidinase-related protein 2 OS=Rattus norvegicus GN=Dpysl2 PE=1 SV=1	SITIANQTNCPLYVTK
>	sp|P47942|DPY	Dihydropyrimidinase-related protein 2 OS=Rattus norvegicus GN=Dpysl2 PE=1 SV=1	THNSALEYNIFEGMECR
>	sp|P47942|DPY	Dihydropyrimidinase-related protein 2 OS=Rattus norvegicus GN=Dpysl2 PE=1 SV=1	THNSALEYNIFEGMECR
>	sp|P47942|DPY	Dihydropyrimidinase-related protein 2 OS=Rattus norvegicus GN=Dpysl2 PE=1 SV=1	VFNLYPR
>	sp|P47942|DPY	Dihydropyrimidinase-related protein 2 OS=Rattus norvegicus GN=Dpysl2 PE=1 SV=1	MVIPGGIDVHTR
>	sp|P47942|DPY	Dihydropyrimidinase-related protein 2 OS=Rattus norvegicus GN=Dpysl2 PE=1 SV=1	PGGIDVHTR
>	sp|P47942|DPY	Dihydropyrimidinase-related protein 2 OS=Rattus norvegicus GN=Dpysl2 PE=1 SV=1	AVGKDNFTLIPEGTNGTEER
>	sp|P47942|DPY	Dihydropyrimidinase-related protein 2 OS=Rattus norvegicus GN=Dpysl2 PE=1 SV=1	QIGENLIVPGGVK
>	sp|P47942|DPY	Dihydropyrimidinase-related protein 2 OS=Rattus norvegicus GN=Dpysl2 PE=1 SV=1	AVGKDNFTLIPEGTNGTEER
>	sp|P47942|DPY	Dihydropyrimidinase-related protein 2 OS=Rattus norvegicus GN=Dpysl2 PE=1 SV=1	QIGENLIVPGGVK
>	sp|P47942|DPY	Dihydropyrimidinase-related protein 2 OS=Rattus norvegicus GN=Dpysl2 PE=1 SV=1	AVGKDNFTLIPEGTNGTEER
>	sp|P47942|DPY	Dihydropyrimidinase-related protein 2 OS=Rattus norvegicus GN=Dpysl2 PE=1 SV=1	IVNDDQSFYADIYMEDGLIK
>	sp|P47942|DPY	Dihydropyrimidinase-related protein 2 OS=Rattus norvegicus GN=Dpysl2 PE=1 SV=1	GLYDGPVCEVSVTPK
>	sp|P47942|DPY	Dihydropyrimidinase-related protein 2 OS=Rattus norvegicus GN=Dpysl2 PE=1 SV=1	AAAFVTSPPLSPDPTTPDFLNSLLSCGDLQVTGSAHCTFNTAQK
>	sp|P47942|DPY	Dihydropyrimidinase-related protein 2 OS=Rattus norvegicus GN=Dpysl2 PE=1 SV=1	AAAFVTSPPLSPDPTTPDFLNSLLSCGDLQVTGSAHCTFNTAQK
>	sp|P47942|DPY	Dihydropyrimidinase-related protein 2 OS=Rattus norvegicus GN=Dpysl2 PE=1 SV=1	IVLEDGTLHVTEGSGR
>	sp|P47942|DPY	Dihydropyrimidinase-related protein 2 OS=Rattus norvegicus GN=Dpysl2 PE=1 SV=1	GSPLVVISQGK
>	sp|P47942|DPY	Dihydropyrimidinase-related protein 2 OS=Rattus norvegicus GN=Dpysl2 PE=1 SV=1	MDENQFVAVTSTNAAK
>	sp|P47942|DPY	Dihydropyrimidinase-related protein 2 OS=Rattus norvegicus GN=Dpysl2 PE=1 SV=1	AALAGGTTMIIDHVVPEPGTSLLAAFDQWR
>	sp|P47942|DPY	Dihydropyrimidinase-related protein 2 OS=Rattus norvegicus GN=Dpysl2 PE=1 SV=1	DIGAIAQVHAENGDIIAEEQQR
>	sp|P47942|DPY	Dihydropyrimidinase-related protein 2 OS=Rattus norvegicus GN=Dpysl2 PE=1 SV=1	ILDLGITGPEGHVLSRPEEVEAEAVNR
>	sp|P47942|DPY	Dihydropyrimidinase-related protein 2 OS=Rattus norvegicus GN=Dpysl2 PE=1 SV=1	PEGTNGTEER
>	sp|P47942|DPY	Dihydropyrimidinase-related protein 2 OS=Rattus norvegicus GN=Dpysl2 PE=1 SV=1	VFNLYPR
>	sp|P47942|DPY	Dihydropyrimidinase-related protein 2 OS=Rattus norvegicus GN=Dpysl2 PE=1 SV=1	ISVGSDADLVIWDPDSVK
>	sp|P47942|DPY	Dihydropyrimidinase-related protein 2 OS=Rattus norvegicus GN=Dpysl2 PE=1 SV=1	KKGTVVYGEPITASLGTDGSHYWSK
>	sp|P47942|DPY	Dihydropyrimidinase-related protein 2 OS=Rattus norvegicus GN=Dpysl2 PE=1 SV=1	VFNLYPR
>	sp|P47942|DPY	Dihydropyrimidinase-related protein 2 OS=Rattus norvegicus GN=Dpysl2 PE=1 SV=1	AVGKDNFTLIPEGTNGTEER
>	sp|P47942|DPY	Dihydropyrimidinase-related protein 2 OS=Rattus norvegicus GN=Dpysl2 PE=1 SV=1	IVLEDGTLHVTEGSGR
>	sp|P47942|DPY	Dihydropyrimidinase-related protein 2 OS=Rattus norvegicus GN=Dpysl2 PE=1 SV=1	NLHQSGFSLSGAQIDDNIPR
>	sp|P47942|DPY	Dihydropyrimidinase-related protein 2 OS=Rattus norvegicus GN=Dpysl2 PE=1 SV=1	AAAFVTSPPLSPDPTTPDFLNSLLSCGDLQVTGSAHCTFNTAQK
>	sp|P47942|DPY	Dihydropyrimidinase-related protein 2 OS=Rattus norvegicus GN=Dpysl2 PE=1 SV=1	IVLEDGTLHVTEGSGR
>	sp|P47942|DPY	Dihydropyrimidinase-related protein 2 OS=Rattus norvegicus GN=Dpysl2 PE=1 SV=1	AVGKDNFTLIPEGTNGTEER
>	sp|P47942|DPY	Dihydropyrimidinase-related protein 2 OS=Rattus norvegicus GN=Dpysl2 PE=1 SV=1	SITIANQTNCPLYVTK
>	sp|P47942|DPY	Dihydropyrimidinase-related protein 2 OS=Rattus norvegicus GN=Dpysl2 PE=1 SV=1	AAAFVTSPPLSPDPTTPDFLNSLLSCGDLQVTGSAHCTFNTAQK
>	sp|P47942|DPY	Dihydropyrimidinase-related protein 2 OS=Rattus norvegicus GN=Dpysl2 PE=1 SV=1	PGGIDVHTR
>	sp|P47942|DPY	Dihydropyrimidinase-related protein 2 OS=Rattus norvegicus GN=Dpysl2 PE=1 SV=1	MDENQFVAVTSTNAAK
>	sp|P47942|DPY	Dihydropyrimidinase-related protein 2 OS=Rattus norvegicus GN=Dpysl2 PE=1 SV=1	GSPLVVISQGK
>	sp|P47942|DPY	Dihydropyrimidinase-related protein 2 OS=Rattus norvegicus GN=Dpysl2 PE=1 SV=1	AAAFVTSPPLSPDPTTPDFLNSLLSCGDLQVTGSAHCTFNTAQK
>	sp|P47942|DPY	Dihydropyrimidinase-related protein 2 OS=Rattus norvegicus GN=Dpysl2 PE=1 SV=1	IVLEDGTLHVTEGSGR
>	sp|P47942|DPY	Dihydropyrimidinase-related protein 2 OS=Rattus norvegicus GN=Dpysl2 PE=1 SV=1	IVAPPGGR
>	sp|P47942|DPY	Dihydropyrimidinase-related protein 2 OS=Rattus norvegicus GN=Dpysl2 PE=1 SV=1	AVGKDNFTLIPEGTNGTEER
>	sp|P47942|DPY	Dihydropyrimidinase-related protein 2 OS=Rattus norvegicus GN=Dpysl2 PE=1 SV=1	AAAFVTSPPLSPDPTTPDFLNSLLSCGDLQVTGSAHCTFNTAQK
>	sp|P47942|DPY	Dihydropyrimidinase-related protein 2 OS=Rattus norvegicus GN=Dpysl2 PE=1 SV=1	IVAPPGGR
>	sp|P47942|DPY	Dihydropyrimidinase-related protein 2 OS=Rattus norvegicus GN=Dpysl2 PE=1 SV=1	LAELR
>	sp|P47942|DPY	Dihydropyrimidinase-related protein 2 OS=Rattus norvegicus GN=Dpysl2 PE=1 SV=1	GSPLVVISQGK
>	sp|P47942|DPY	Dihydropyrimidinase-related protein 2 OS=Rattus norvegicus GN=Dpysl2 PE=1 SV=1	SITIANQTNCPLYVTK
>	sp|Q05962|AD	ADP/ATP translocase 1 OS=Rattus norvegicus GN=Slc25a4 PE=1 SV=3	DFLAGGIAAAVSK
>	sp|Q05962|AD	ADP/ATP translocase 1 OS=Rattus norvegicus GN=Slc25a4 PE=1 SV=3	EFNGLGDCLTK
>	sp|Q05962|AD	ADP/ATP translocase 1 OS=Rattus norvegicus GN=Slc25a4 PE=1 SV=3	GADIMYTGTVDCWR
>	sp|Q05962|AD	ADP/ATP translocase 1 OS=Rattus norvegicus GN=Slc25a4 PE=1 SV=3	GDQALSFLKDFLAGGIAAAVSK
>	sp|Q05962|AD	ADP/ATP translocase 1 OS=Rattus norvegicus GN=Slc25a4 PE=1 SV=3	GMGGAFVLVLYDEIKK
>	sp|Q05962|AD	ADP/ATP translocase 1 OS=Rattus norvegicus GN=Slc25a4 PE=1 SV=3	GMGGAFVLVLYDEIKKYV
>	sp|Q05962|AD	ADP/ATP translocase 1 OS=Rattus norvegicus GN=Slc25a4 PE=1 SV=3	IPKEQGFLSFWR
>	sp|Q05962|AD	ADP/ATP translocase 1 OS=Rattus norvegicus GN=Slc25a4 PE=1 SV=3	KGADIMYTGTVDCWR
>	sp|Q05962|AD	ADP/ATP translocase 1 OS=Rattus norvegicus GN=Slc25a4 PE=1 SV=3	TAVAPIER
>	sp|Q05962|AD	ADP/ATP translocase 1 OS=Rattus norvegicus GN=Slc25a4 PE=1 SV=3	YFAGNLASGGAAGATSLCFVYPLDFAR
>	sp|Q05962|AD	ADP/ATP translocase 1 OS=Rattus norvegicus GN=Slc25a4 PE=1 SV=3	YFPTQALNFAFK

>	sp|Q05962|AD	ADP/ATP translocase 1 OS=Rattus norvegicus GN=Slc25a4 PE=1 SV=3	GAWSNVLR
>	sp|Q05962|AD	ADP/ATP translocase 1 OS=Rattus norvegicus GN=Slc25a4 PE=1 SV=3	LLLQVQHASK
>	sp|Q05962|AD	ADP/ATP translocase 1 OS=Rattus norvegicus GN=Slc25a4 PE=1 SV=3	GDQALSFLKDFLAGGIAAAVSK
>	sp|Q05962|AD	ADP/ATP translocase 1 OS=Rattus norvegicus GN=Slc25a4 PE=1 SV=3	GMGGAFVLVLYDEIKK
>	sp|Q05962|AD	ADP/ATP translocase 1 OS=Rattus norvegicus GN=Slc25a4 PE=1 SV=3	GMGGAFVLVLYDEIKK
>	sp|Q05962|AD	ADP/ATP translocase 1 OS=Rattus norvegicus GN=Slc25a4 PE=1 SV=3	GMGGAFVLVLYDEIKK
>	sp|Q05962|AD	ADP/ATP translocase 1 OS=Rattus norvegicus GN=Slc25a4 PE=1 SV=3	GMGGAFVLVLYDEIKK
>	sp|Q05962|AD	ADP/ATP translocase 1 OS=Rattus norvegicus GN=Slc25a4 PE=1 SV=3	GMGGAFVLVLYDEIKK
>	sp|Q05962|AD	ADP/ATP translocase 1 OS=Rattus norvegicus GN=Slc25a4 PE=1 SV=3	GMGGAFVLVLYDEIKK
>	sp|Q05962|AD	ADP/ATP translocase 1 OS=Rattus norvegicus GN=Slc25a4 PE=1 SV=3	IPKEQGFLSFWR
>	sp|Q05962|AD	ADP/ATP translocase 1 OS=Rattus norvegicus GN=Slc25a4 PE=1 SV=3	KGADIMYTGTVDCWR
>	sp|Q05962|AD	ADP/ATP translocase 1 OS=Rattus norvegicus GN=Slc25a4 PE=1 SV=3	KGADIMYTGTVDCWR
>	sp|Q05962|AD	ADP/ATP translocase 1 OS=Rattus norvegicus GN=Slc25a4 PE=1 SV=3	KGADIMYTGTVDCWR
>	sp|Q05962|AD	ADP/ATP translocase 1 OS=Rattus norvegicus GN=Slc25a4 PE=1 SV=3	KGADIMYTGTVDCWR
>	sp|Q05962|AD	ADP/ATP translocase 1 OS=Rattus norvegicus GN=Slc25a4 PE=1 SV=3	KGADIMYTGTVDCWR
>	sp|Q05962|AD	ADP/ATP translocase 1 OS=Rattus norvegicus GN=Slc25a4 PE=1 SV=3	KGADIMYTGTVDCWR
>	sp|Q05962|AD	ADP/ATP translocase 1 OS=Rattus norvegicus GN=Slc25a4 PE=1 SV=3	TAVAPIER
>	sp|Q05962|AD	ADP/ATP translocase 1 OS=Rattus norvegicus GN=Slc25a4 PE=1 SV=3	TAVAPIER
>	sp|Q05962|AD	ADP/ATP translocase 1 OS=Rattus norvegicus GN=Slc25a4 PE=1 SV=3	YFAGNLASGGAAGATSLCFVYPLDFAR
>	sp|Q05962|AD	ADP/ATP translocase 1 OS=Rattus norvegicus GN=Slc25a4 PE=1 SV=3	YFAGNLASGGAAGATSLCFVYPLDFAR
>	sp|Q05962|AD	ADP/ATP translocase 1 OS=Rattus norvegicus GN=Slc25a4 PE=1 SV=3	YFAGNLASGGAAGATSLCFVYPLDFAR
>	sp|Q05962|AD	ADP/ATP translocase 1 OS=Rattus norvegicus GN=Slc25a4 PE=1 SV=3	YFAGNLASGGAAGATSLCFVYPLDFAR
>	sp|Q05962|AD	ADP/ATP translocase 1 OS=Rattus norvegicus GN=Slc25a4 PE=1 SV=3	YFAGNLASGGAAGATSLCFVYPLDFAR
>	sp|Q05962|AD	ADP/ATP translocase 1 OS=Rattus norvegicus GN=Slc25a4 PE=1 SV=3	YFAGNLASGGAAGATSLCFVYPLDFAR
>	sp|Q05962|AD	ADP/ATP translocase 1 OS=Rattus norvegicus GN=Slc25a4 PE=1 SV=3	YFAGNLASGGAAGATSLCFVYPLDFAR
>	sp|Q05962|AD	ADP/ATP translocase 1 OS=Rattus norvegicus GN=Slc25a4 PE=1 SV=3	YFAGNLASGGAAGATSLCFVYPLDFAR
>	sp|Q05962|AD	ADP/ATP translocase 1 OS=Rattus norvegicus GN=Slc25a4 PE=1 SV=3	YFAGNLASGGAAGATSLCFVYPLDFAR
>	sp|Q05962|AD	ADP/ATP translocase 1 OS=Rattus norvegicus GN=Slc25a4 PE=1 SV=3	YFAGNLASGGAAGATSLCFVYPLDFAR
>	sp|Q05962|AD	ADP/ATP translocase 1 OS=Rattus norvegicus GN=Slc25a4 PE=1 SV=3	YFAGNLASGGAAGATSLCFVYPLDFAR
>	sp|Q05962|AD	ADP/ATP translocase 1 OS=Rattus norvegicus GN=Slc25a4 PE=1 SV=3	YFAGNLASGGAAGATSLCFVYPLDFAR
>	sp|Q05962|AD	ADP/ATP translocase 1 OS=Rattus norvegicus GN=Slc25a4 PE=1 SV=3	YFAGNLASGGAAGATSLCFVYPLDFAR
>	sp|Q05962|AD	ADP/ATP translocase 1 OS=Rattus norvegicus GN=Slc25a4 PE=1 SV=3	YFPTQALNFAFK
>	sp|Q05962|AD	ADP/ATP translocase 1 OS=Rattus norvegicus GN=Slc25a4 PE=1 SV=3	YFPTQALNFAFK
>	sp|Q05962|AD	ADP/ATP translocase 1 OS=Rattus norvegicus GN=Slc25a4 PE=1 SV=3	GMGGAFVLVLYDEIKK
>	sp|Q05962|AD	ADP/ATP translocase 1 OS=Rattus norvegicus GN=Slc25a4 PE=1 SV=3	TAVAPIER
>	sp|Q05962|AD	ADP/ATP translocase 1 OS=Rattus norvegicus GN=Slc25a4 PE=1 SV=3	GMGGAFVLVLYDEIKK
>	sp|Q05962|AD	ADP/ATP translocase 1 OS=Rattus norvegicus GN=Slc25a4 PE=1 SV=3	KGADIMYTGTVDCWR
>	sp|Q05962|AD	ADP/ATP translocase 1 OS=Rattus norvegicus GN=Slc25a4 PE=1 SV=3	QIFLGGVDR
>	sp|Q05962|AD	ADP/ATP translocase 1 OS=Rattus norvegicus GN=Slc25a4 PE=1 SV=3	AFFKGAWSNVLRGMGGAFVLVLYDEIKKYV
>	sp|Q05962|AD	ADP/ATP translocase 1 OS=Rattus norvegicus GN=Slc25a4 PE=1 SV=3	QISAEK
>	sp|Q05962|AD	ADP/ATP translocase 1 OS=Rattus norvegicus GN=Slc25a4 PE=1 SV=3	GAWSNVLR
>	sp|Q05962|AD	ADP/ATP translocase 1 OS=Rattus norvegicus GN=Slc25a4 PE=1 SV=3	QISAEK
>	sp|Q05962|AD	ADP/ATP translocase 1 OS=Rattus norvegicus GN=Slc25a4 PE=1 SV=3	IPKEQGFLSFWR
>	sp|Q05962|AD	ADP/ATP translocase 1 OS=Rattus norvegicus GN=Slc25a4 PE=1 SV=3	EFNGLGDCLTK
>	sp|Q05962|AD	ADP/ATP translocase 1 OS=Rattus norvegicus GN=Slc25a4 PE=1 SV=3	GMLPDPK
>	sp|Q05962|AD	ADP/ATP translocase 1 OS=Rattus norvegicus GN=Slc25a4 PE=1 SV=3	AAYFGVYDTAK
>	sp|Q05962|AD	ADP/ATP translocase 1 OS=Rattus norvegicus GN=Slc25a4 PE=1 SV=3	KGADIMYTGTVDCWR
>	sp|Q05962|AD	ADP/ATP translocase 1 OS=Rattus norvegicus GN=Slc25a4 PE=1 SV=3	KGADIMYTGTVDCWR
>	sp|Q05962|AD	ADP/ATP translocase 1 OS=Rattus norvegicus GN=Slc25a4 PE=1 SV=3	LAADVGK
>	sp|Q05962|AD	ADP/ATP translocase 1 OS=Rattus norvegicus GN=Slc25a4 PE=1 SV=3	LLLQVQHASK
>	sp|Q05962|AD	ADP/ATP translocase 1 OS=Rattus norvegicus GN=Slc25a4 PE=1 SV=3	QIFLGGVDR
>	sp|P00507|AA1	Aspartate aminotransferase, mitochondrial OS=Rattus norvegicus GN=Got2 PE=1 SV=2	ASAELALGENSEVLK
>	sp|P00507|AA1	Aspartate aminotransferase, mitochondrial OS=Rattus norvegicus GN=Got2 PE=1 SV=2	EGSSHNWQHITDQIGMFCFTGLKPEQVER
>	sp|P00507|AA1	Aspartate aminotransferase, mitochondrial OS=Rattus norvegicus GN=Got2 PE=1 SV=2	FVTVQTISGTGALR
>	sp|P00507|AA1	Aspartate aminotransferase, mitochondrial OS=Rattus norvegicus GN=Got2 PE=1 SV=2	IAATILTSPDLR
>	sp|P00507|AA1	Aspartate aminotransferase, mitochondrial OS=Rattus norvegicus GN=Got2 PE=1 SV=2	IAATILTSPDLRKQWLQEVKGMADRIISMR
>	sp|P00507|AA1	Aspartate aminotransferase, mitochondrial OS=Rattus norvegicus GN=Got2 PE=1 SV=2	ISVAGVTSGNVGYLAHAIHQVTK
>	sp|P00507|AA1	Aspartate aminotransferase, mitochondrial OS=Rattus norvegicus GN=Got2 PE=1 SV=2	KAEAQIAGK
>	sp|P00507|AA1	Aspartate aminotransferase, mitochondrial OS=Rattus norvegicus GN=Got2 PE=1 SV=2	KKNLFAFFDMAYQGFASGDGDKDAWAVR
>	sp|P00507|AA1	Aspartate aminotransferase, mitochondrial OS=Rattus norvegicus GN=Got2 PE=1 SV=2	KMNLGVGAYRDDNGKPYVLPSVR
>	sp|P00507|AA1	Aspartate aminotransferase, mitochondrial OS=Rattus norvegicus GN=Got2 PE=1 SV=2	NLDKEYLPIGGLADFCK
>	sp|P00507|AA1	Aspartate aminotransferase, mitochondrial OS=Rattus norvegicus GN=Got2 PE=1 SV=2	SSWWTHVEMGPPDPILGVTEAFKR
>	sp|P00507|AA1	Aspartate aminotransferase, mitochondrial OS=Rattus norvegicus GN=Got2 PE=1 SV=2	TCGFDFSGALEDISKIPEQSVLLLHACAHNPTGVDPRPEQWK
>	sp|P00507|AA1	Aspartate aminotransferase, mitochondrial OS=Rattus norvegicus GN=Got2 PE=1 SV=2	DAEEAK
>	sp|P00507|AA1	Aspartate aminotransferase, mitochondrial OS=Rattus norvegicus GN=Got2 PE=1 SV=2	VGAFTVVCK
>	sp|P00507|AA1	Aspartate aminotransferase, mitochondrial OS=Rattus norvegicus GN=Got2 PE=1 SV=2	HFIEQGINVCLCQSYAK
>	sp|P00507|AA1	Aspartate aminotransferase, mitochondrial OS=Rattus norvegicus GN=Got2 PE=1 SV=2	AASARASSWWTHVEMGPPDPILGVTEAFKR
>	sp|P00507|AA1	Aspartate aminotransferase, mitochondrial OS=Rattus norvegicus GN=Got2 PE=1 SV=2	SSWWTHVEMGPPDPILGVTEAFK
>	sp|P00507|AA1	Aspartate aminotransferase, mitochondrial OS=Rattus norvegicus GN=Got2 PE=1 SV=2	ASAELALGENSEVLK
>	sp|P00507|AA1	Aspartate aminotransferase, mitochondrial OS=Rattus norvegicus GN=Got2 PE=1 SV=2	ASAELALGENSEVLK
>	sp|P00507|AA1	Aspartate aminotransferase, mitochondrial OS=Rattus norvegicus GN=Got2 PE=1 SV=2	HFIEQGINVCLCQSYAK
>	sp|P00507|AA1	Aspartate aminotransferase, mitochondrial OS=Rattus norvegicus GN=Got2 PE=1 SV=2	IAATILTSPDLRKQWLQEVKGMADRIISMR
>	sp|P00507|AA1	Aspartate aminotransferase, mitochondrial OS=Rattus norvegicus GN=Got2 PE=1 SV=2	ISVAGVTSGNVGYLAHAIHQVTK
>	sp|P00507|AA1	Aspartate aminotransferase, mitochondrial OS=Rattus norvegicus GN=Got2 PE=1 SV=2	ISVAGVTSGNVGYLAHAIHQVTK

>	sp|P00507|AA1	Aspartate aminotransferase, mitochondrial OS=Rattus norvegicus GN=Got2 PE=1 SV=2	ISVAGVTSGNVGYLAHAIHQVTK
>	sp|P00507|AA1	Aspartate aminotransferase, mitochondrial OS=Rattus norvegicus GN=Got2 PE=1 SV=2	ISVAGVTSGNVGYLAHAIHQVTK
>	sp|P00507|AA1	Aspartate aminotransferase, mitochondrial OS=Rattus norvegicus GN=Got2 PE=1 SV=2	ISVAGVTSGNVGYLAHAIHQVTK
>	sp|P00507|AA1	Aspartate aminotransferase, mitochondrial OS=Rattus norvegicus GN=Got2 PE=1 SV=2	ISVAGVTSGNVGYLAHAIHQVTK
>	sp|P00507|AA1	Aspartate aminotransferase, mitochondrial OS=Rattus norvegicus GN=Got2 PE=1 SV=2	ISVAGVTSGNVGYLAHAIHQVTK
>	sp|P00507|AA1	Aspartate aminotransferase, mitochondrial OS=Rattus norvegicus GN=Got2 PE=1 SV=2	ISVAGVTSGNVGYLAHAIHQVTK
>	sp|P00507|AA1	Aspartate aminotransferase, mitochondrial OS=Rattus norvegicus GN=Got2 PE=1 SV=2	KKNLFAFFDMAYQGFASGDGDKDAWAVR
>	sp|P00507|AA1	Aspartate aminotransferase, mitochondrial OS=Rattus norvegicus GN=Got2 PE=1 SV=2	KKNLFAFFDMAYQGFASGDGDKDAWAVR
>	sp|P00507|AA1	Aspartate aminotransferase, mitochondrial OS=Rattus norvegicus GN=Got2 PE=1 SV=2	KKNLFAFFDMAYQGFASGDGDKDAWAVR
>	sp|P00507|AA1	Aspartate aminotransferase, mitochondrial OS=Rattus norvegicus GN=Got2 PE=1 SV=2	KKNLFAFFDMAYQGFASGDGDKDAWAVR
>	sp|P00507|AA1	Aspartate aminotransferase, mitochondrial OS=Rattus norvegicus GN=Got2 PE=1 SV=2	KKNLFAFFDMAYQGFASGDGDKDAWAVR
>	sp|P00507|AA1	Aspartate aminotransferase, mitochondrial OS=Rattus norvegicus GN=Got2 PE=1 SV=2	KKNLFAFFDMAYQGFASGDGDKDAWAVR
>	sp|P00507|AA1	Aspartate aminotransferase, mitochondrial OS=Rattus norvegicus GN=Got2 PE=1 SV=2	KKNLFAFFDMAYQGFASGDGDKDAWAVR
>	sp|P00507|AA1	Aspartate aminotransferase, mitochondrial OS=Rattus norvegicus GN=Got2 PE=1 SV=2	NLDKEYLPIGGLADFCK
>	sp|P00507|AA1	Aspartate aminotransferase, mitochondrial OS=Rattus norvegicus GN=Got2 PE=1 SV=2	NLDKEYLPIGGLADFCK
>	sp|P00507|AA1	Aspartate aminotransferase, mitochondrial OS=Rattus norvegicus GN=Got2 PE=1 SV=2	NLDKEYLPIGGLADFCK
>	sp|P00507|AA1	Aspartate aminotransferase, mitochondrial OS=Rattus norvegicus GN=Got2 PE=1 SV=2	SSWWTHVEMGPPDPILGVTEAFKR
>	sp|P00507|AA1	Aspartate aminotransferase, mitochondrial OS=Rattus norvegicus GN=Got2 PE=1 SV=2	TCGFDFSGALEDISKIPEQSVLLLHACAHNPTGVDPRPEQWK
>	sp|P00507|AA1	Aspartate aminotransferase, mitochondrial OS=Rattus norvegicus GN=Got2 PE=1 SV=2	TCGFDFSGALEDISK
>	sp|P00507|AA1	Aspartate aminotransferase, mitochondrial OS=Rattus norvegicus GN=Got2 PE=1 SV=2	VGAFTVVCK
>	sp|P00507|AA1	Aspartate aminotransferase, mitochondrial OS=Rattus norvegicus GN=Got2 PE=1 SV=2	NLDKEYLPIGGLADFCK
>	sp|P00507|AA1	Aspartate aminotransferase, mitochondrial OS=Rattus norvegicus GN=Got2 PE=1 SV=2	DDNGKPYVLPSVR
>	sp|P00507|AA1	Aspartate aminotransferase, mitochondrial OS=Rattus norvegicus GN=Got2 PE=1 SV=2	VGAFTVVCK
>	sp|P00507|AA1	Aspartate aminotransferase, mitochondrial OS=Rattus norvegicus GN=Got2 PE=1 SV=2	AASARASSWWTHVEMGPPDPILGVTEAFKR
>	sp|P00507|AA1	Aspartate aminotransferase, mitochondrial OS=Rattus norvegicus GN=Got2 PE=1 SV=2	KEGSSHNWQHITDQIGMFCFTGLKPEQVER
>	sp|P00507|AA1	Aspartate aminotransferase, mitochondrial OS=Rattus norvegicus GN=Got2 PE=1 SV=2	ASAELALGENSEVLK
>	sp|P00507|AA1	Aspartate aminotransferase, mitochondrial OS=Rattus norvegicus GN=Got2 PE=1 SV=2	ILIRPLYSNPPLNGAR
>	sp|P00507|AA1	Aspartate aminotransferase, mitochondrial OS=Rattus norvegicus GN=Got2 PE=1 SV=2	EGSSHNWQHITDQIGMFCFTGLKPEQVER
>	sp|P00507|AA1	Aspartate aminotransferase, mitochondrial OS=Rattus norvegicus GN=Got2 PE=1 SV=2	ISVAGVTSGNVGYLAHAIHQVTK
>	sp|P00507|AA1	Aspartate aminotransferase, mitochondrial OS=Rattus norvegicus GN=Got2 PE=1 SV=2	KAEAQIAGK
>	sp|P00507|AA1	Aspartate aminotransferase, mitochondrial OS=Rattus norvegicus GN=Got2 PE=1 SV=2	KMNLGVGAYRDDNGKPYVLPSVR
>	sp|P00507|AA1	Aspartate aminotransferase, mitochondrial OS=Rattus norvegicus GN=Got2 PE=1 SV=2	TCGFDFSGALEDISKIPEQSVLLLHACAHNPTGVDPRPEQWK
>	sp|P00507|AA1	Aspartate aminotransferase, mitochondrial OS=Rattus norvegicus GN=Got2 PE=1 SV=2	TCGFDFSGALEDISKIPEQSVLLLHACAHNPTGVDPRPEQWK
>	sp|P00507|AA1	Aspartate aminotransferase, mitochondrial OS=Rattus norvegicus GN=Got2 PE=1 SV=2	KEGSSHNWQHITDQIGMFCFTGLKPEQVER
>	sp|P12839|NFI	Neurofilament medium polypeptide OS=Rattus norvegicus GN=Nefm PE=1 SV=4	AGKDEQKEEEKVEEK
>	sp|P12839|NFI	Neurofilament medium polypeptide OS=Rattus norvegicus GN=Nefm PE=1 SV=4	AQVQLDSDHLEEDIHR
>	sp|P12839|NFI	Neurofilament medium polypeptide OS=Rattus norvegicus GN=Nefm PE=1 SV=4	ATLEMVNHEK
>	sp|P12839|NFI	Neurofilament medium polypeptide OS=Rattus norvegicus GN=Nefm PE=1 SV=4	AVEEMITITK
>	sp|P12839|NFI	Neurofilament medium polypeptide OS=Rattus norvegicus GN=Nefm PE=1 SV=4	EIEAEIHALR
>	sp|P12839|NFI	Neurofilament medium polypeptide OS=Rattus norvegicus GN=Nefm PE=1 SV=4	HNHDLSSYQDTIQQLENELR
>	sp|P12839|NFI	Neurofilament medium polypeptide OS=Rattus norvegicus GN=Nefm PE=1 SV=4	LRDDTEAAIR
>	sp|P12839|NFI	Neurofilament medium polypeptide OS=Rattus norvegicus GN=Nefm PE=1 SV=4	QASHAQLGDAYDQEIR
>	sp|P12839|NFI	Neurofilament medium polypeptide OS=Rattus norvegicus GN=Nefm PE=1 SV=4	SNEKEQLQGLNDR
>	sp|P12839|NFI	Neurofilament medium polypeptide OS=Rattus norvegicus GN=Nefm PE=1 SV=4	SNHEEEVADLLAQIQASHITVER
>	sp|P12839|NFI	Neurofilament medium polypeptide OS=Rattus norvegicus GN=Nefm PE=1 SV=4	VHYLEQQNKEIEAEIHALR
>	sp|P12839|NFI	Neurofilament medium polypeptide OS=Rattus norvegicus GN=Nefm PE=1 SV=4	VQSLQDEVAFLR
>	sp|P12839|NFI	Neurofilament medium polypeptide OS=Rattus norvegicus GN=Nefm PE=1 SV=4	DTKEEKPQQQEK
>	sp|P12839|NFI	Neurofilament medium polypeptide OS=Rattus norvegicus GN=Nefm PE=1 SV=4	SAKEEIAEYR
>	sp|P12839|NFI	Neurofilament medium polypeptide OS=Rattus norvegicus GN=Nefm PE=1 SV=4	AGKDEQKEEEKVEEK
>	sp|P12839|NFI	Neurofilament medium polypeptide OS=Rattus norvegicus GN=Nefm PE=1 SV=4	AGKDEQKEEEKVEEK
>	sp|P12839|NFI	Neurofilament medium polypeptide OS=Rattus norvegicus GN=Nefm PE=1 SV=4	AVEEMITITK
>	sp|P12839|NFI	Neurofilament medium polypeptide OS=Rattus norvegicus GN=Nefm PE=1 SV=4	AVEEMITITK
>	sp|P12839|NFI	Neurofilament medium polypeptide OS=Rattus norvegicus GN=Nefm PE=1 SV=4	DTKEEKPQQQEK
>	sp|P12839|NFI	Neurofilament medium polypeptide OS=Rattus norvegicus GN=Nefm PE=1 SV=4	EIEAEIHALR
>	sp|P12839|NFI	Neurofilament medium polypeptide OS=Rattus norvegicus GN=Nefm PE=1 SV=4	EIEAEIHALR
>	sp|P12839|NFI	Neurofilament medium polypeptide OS=Rattus norvegicus GN=Nefm PE=1 SV=4	EIEAEIHALR
>	sp|P12839|NFI	Neurofilament medium polypeptide OS=Rattus norvegicus GN=Nefm PE=1 SV=4	EIEAEIHALR
>	sp|P12839|NFI	Neurofilament medium polypeptide OS=Rattus norvegicus GN=Nefm PE=1 SV=4	EIEAEIHALR
>	sp|P12839|NFI	Neurofilament medium polypeptide OS=Rattus norvegicus GN=Nefm PE=1 SV=4	HNHDLSSYQDTIQQLENELR
>	sp|P12839|NFI	Neurofilament medium polypeptide OS=Rattus norvegicus GN=Nefm PE=1 SV=4	HNHDLSSYQDTIQQLENELR
>	sp|P12839|NFI	Neurofilament medium polypeptide OS=Rattus norvegicus GN=Nefm PE=1 SV=4	KLLEGEETR
>	sp|P12839|NFI	Neurofilament medium polypeptide OS=Rattus norvegicus GN=Nefm PE=1 SV=4	KLLEGEETR
>	sp|P12839|NFI	Neurofilament medium polypeptide OS=Rattus norvegicus GN=Nefm PE=1 SV=4	KLLEGEETR
>	sp|P12839|NFI	Neurofilament medium polypeptide OS=Rattus norvegicus GN=Nefm PE=1 SV=4	KLLEGEETR
>	sp|P12839|NFI	Neurofilament medium polypeptide OS=Rattus norvegicus GN=Nefm PE=1 SV=4	KLLEGEETR
>	sp|P12839|NFI	Neurofilament medium polypeptide OS=Rattus norvegicus GN=Nefm PE=1 SV=4	LLEGEETR
>	sp|P12839|NFI	Neurofilament medium polypeptide OS=Rattus norvegicus GN=Nefm PE=1 SV=4	LLEGEETR
>	sp|P12839|NFI	Neurofilament medium polypeptide OS=Rattus norvegicus GN=Nefm PE=1 SV=4	LRDDTEAAIR
>	sp|P12839|NFI	Neurofilament medium polypeptide OS=Rattus norvegicus GN=Nefm PE=1 SV=4	LRDDTEAAIR
>	sp|P12839|NFI	Neurofilament medium polypeptide OS=Rattus norvegicus GN=Nefm PE=1 SV=4	LRDDTEAAIR
>	sp|P12839|NFI	Neurofilament medium polypeptide OS=Rattus norvegicus GN=Nefm PE=1 SV=4	MALDIEIAAYR
>	sp|P12839|NFI	Neurofilament medium polypeptide OS=Rattus norvegicus GN=Nefm PE=1 SV=4	QASHAQLGDAYDQEIR
>	sp|P12839|NFI	Neurofilament medium polypeptide OS=Rattus norvegicus GN=Nefm PE=1 SV=4	QASHAQLGDAYDQEIR
>	sp|P12839|NFI	Neurofilament medium polypeptide OS=Rattus norvegicus GN=Nefm PE=1 SV=4	QASHAQLGDAYDQEIR

>	sp|P12839|NFM	Neurofilament medium polypeptide OS=Rattus norvegicus GN=Nefm PE=1 SV=4	QASHAQLGDAYDQEIR
>	sp|P12839|NFM	Neurofilament medium polypeptide OS=Rattus norvegicus GN=Nefm PE=1 SV=4	SNEKEQLQGLNDR
>	sp|P12839|NFM	Neurofilament medium polypeptide OS=Rattus norvegicus GN=Nefm PE=1 SV=4	SNEKEQLQGLNDR
>	sp|P12839|NFM	Neurofilament medium polypeptide OS=Rattus norvegicus GN=Nefm PE=1 SV=4	SNHEEEVADLLAQIQASHITVER
>	sp|P12839|NFM	Neurofilament medium polypeptide OS=Rattus norvegicus GN=Nefm PE=1 SV=4	SNHEEEVADLLAQIQASHITVER
>	sp|P12839|NFM	Neurofilament medium polypeptide OS=Rattus norvegicus GN=Nefm PE=1 SV=4	SNHEEEVADLLAQIQASHITVER
>	sp|P12839|NFM	Neurofilament medium polypeptide OS=Rattus norvegicus GN=Nefm PE=1 SV=4	SNHEEEVADLLAQIQASHITVER
>	sp|P12839|NFM	Neurofilament medium polypeptide OS=Rattus norvegicus GN=Nefm PE=1 SV=4	SNHEEEVADLLAQIQASHITVER
>	sp|P12839|NFM	Neurofilament medium polypeptide OS=Rattus norvegicus GN=Nefm PE=1 SV=4	SNHEEEVADLLAQIQASHITVER
>	sp|P12839|NFM	Neurofilament medium polypeptide OS=Rattus norvegicus GN=Nefm PE=1 SV=4	SNHEEEVADLLAQIQASHITVER
>	sp|P12839|NFM	Neurofilament medium polypeptide OS=Rattus norvegicus GN=Nefm PE=1 SV=4	VHYLEQQNKEIEAEIHALR
>	sp|P12839|NFM	Neurofilament medium polypeptide OS=Rattus norvegicus GN=Nefm PE=1 SV=4	EYQDLLNVK
>	sp|P12839|NFM	Neurofilament medium polypeptide OS=Rattus norvegicus GN=Nefm PE=1 SV=4	LLEGEETR
>	sp|P12839|NFM	Neurofilament medium polypeptide OS=Rattus norvegicus GN=Nefm PE=1 SV=4	LLEGEETR
>	sp|P12839|NFM	Neurofilament medium polypeptide OS=Rattus norvegicus GN=Nefm PE=1 SV=4	EIEAEIHALR
>	sp|P12839|NFM	Neurofilament medium polypeptide OS=Rattus norvegicus GN=Nefm PE=1 SV=4	LLEGEETR
>	sp|P12839|NFM	Neurofilament medium polypeptide OS=Rattus norvegicus GN=Nefm PE=1 SV=4	LRDDTEAAIR
>	sp|P12839|NFM	Neurofilament medium polypeptide OS=Rattus norvegicus GN=Nefm PE=1 SV=4	LRDDTEAAIR
>	sp|P12839|NFM	Neurofilament medium polypeptide OS=Rattus norvegicus GN=Nefm PE=1 SV=4	SNHEEEVADLLAQIQASHITVER
>	sp|P12839|NFM	Neurofilament medium polypeptide OS=Rattus norvegicus GN=Nefm PE=1 SV=4	LLEGEETR
>	sp|P12839|NFM	Neurofilament medium polypeptide OS=Rattus norvegicus GN=Nefm PE=1 SV=4	VTSHAIVK
>	sp|P12839|NFM	Neurofilament medium polypeptide OS=Rattus norvegicus GN=Nefm PE=1 SV=4	EIEAEIHALR
>	sp|P12839|NFM	Neurofilament medium polypeptide OS=Rattus norvegicus GN=Nefm PE=1 SV=4	ATLEMVNHEK
>	sp|P12839|NFM	Neurofilament medium polypeptide OS=Rattus norvegicus GN=Nefm PE=1 SV=4	LRDDTEAAIR
>	sp|P12839|NFM	Neurofilament medium polypeptide OS=Rattus norvegicus GN=Nefm PE=1 SV=4	FSTFSGSITGPLYTHR
>	sp|P12839|NFM	Neurofilament medium polypeptide OS=Rattus norvegicus GN=Nefm PE=1 SV=4	SNEKEQLQGLNDR
>	sp|P12839|NFM	Neurofilament medium polypeptide OS=Rattus norvegicus GN=Nefm PE=1 SV=4	SNEKEQLQGLNDR
>	sp|P12839|NFM	Neurofilament medium polypeptide OS=Rattus norvegicus GN=Nefm PE=1 SV=4	SIELESVR
>	sp|P12839|NFM	Neurofilament medium polypeptide OS=Rattus norvegicus GN=Nefm PE=1 SV=4	DTKEEKPQQQEK
>	sp|P12839|NFM	Neurofilament medium polypeptide OS=Rattus norvegicus GN=Nefm PE=1 SV=4	SNEKEQLQGLNDR
>	sp|P12839|NFM	Neurofilament medium polypeptide OS=Rattus norvegicus GN=Nefm PE=1 SV=4	TDISTALK
>	sp|P12839|NFM	Neurofilament medium polypeptide OS=Rattus norvegicus GN=Nefm PE=1 SV=4	LLEGEETR
>	sp|P12839|NFM	Neurofilament medium polypeptide OS=Rattus norvegicus GN=Nefm PE=1 SV=4	MALDIEIAAYR
>	sp|P04636|MD	Malate dehydrogenase, mitochondrial OS=Rattus norvegicus GN=Mdh2 PE=1 SV=2	AGAGSATLSMAYAGAR
>	sp|P04636|MD	Malate dehydrogenase, mitochondrial OS=Rattus norvegicus GN=Mdh2 PE=1 SV=2	DDLFNTNATIVATLTAACAQHCPEAMICIISNPVNSTIPITAEVFKK
>	sp|P04636|MD	Malate dehydrogenase, mitochondrial OS=Rattus norvegicus GN=Mdh2 PE=1 SV=2	ETECTYFSTPLLLGK
>	sp|P04636|MD	Malate dehydrogenase, mitochondrial OS=Rattus norvegicus GN=Mdh2 PE=1 SV=2	FVFSLVDAMNGK
>	sp|P04636|MD	Malate dehydrogenase, mitochondrial OS=Rattus norvegicus GN=Mdh2 PE=1 SV=2	GCDVVVIPAGVPR
>	sp|P04636|MD	Malate dehydrogenase, mitochondrial OS=Rattus norvegicus GN=Mdh2 PE=1 SV=2	IFGVTTLDIVR
>	sp|P04636|MD	Malate dehydrogenase, mitochondrial OS=Rattus norvegicus GN=Mdh2 PE=1 SV=2	IQEAGTEVVK
>	sp|P04636|MD	Malate dehydrogenase, mitochondrial OS=Rattus norvegicus GN=Mdh2 PE=1 SV=2	LTLYDIAHTPGVAADLSHIETR
>	sp|P04636|MD	Malate dehydrogenase, mitochondrial OS=Rattus norvegicus GN=Mdh2 PE=1 SV=2	TIIPLISQCTPK
>	sp|P04636|MD	Malate dehydrogenase, mitochondrial OS=Rattus norvegicus GN=Mdh2 PE=1 SV=2	VAVLGASGGIGQPLSLLLK
>	sp|P04636|MD	Malate dehydrogenase, mitochondrial OS=Rattus norvegicus GN=Mdh2 PE=1 SV=2	VDFPQDQLATLTGR
>	sp|P04636|MD	Malate dehydrogenase, mitochondrial OS=Rattus norvegicus GN=Mdh2 PE=1 SV=2	VNVPVIGGHAGK
>	sp|P04636|MD	Malate dehydrogenase, mitochondrial OS=Rattus norvegicus GN=Mdh2 PE=1 SV=2	MIAEAIPELK
>	sp|P04636|MD	Malate dehydrogenase, mitochondrial OS=Rattus norvegicus GN=Mdh2 PE=1 SV=2	GYLGPEQLPDCLK
>	sp|P04636|MD	Malate dehydrogenase, mitochondrial OS=Rattus norvegicus GN=Mdh2 PE=1 SV=2	AGAGSATLSMAYAGAR
>	sp|P04636|MD	Malate dehydrogenase, mitochondrial OS=Rattus norvegicus GN=Mdh2 PE=1 SV=2	AGAGSATLSMAYAGAR
>	sp|P04636|MD	Malate dehydrogenase, mitochondrial OS=Rattus norvegicus GN=Mdh2 PE=1 SV=2	FVFSLVDAMNGK
>	sp|P04636|MD	Malate dehydrogenase, mitochondrial OS=Rattus norvegicus GN=Mdh2 PE=1 SV=2	FVFSLVDAMNGK
>	sp|P04636|MD	Malate dehydrogenase, mitochondrial OS=Rattus norvegicus GN=Mdh2 PE=1 SV=2	FVFSLVDAMNGK
>	sp|P04636|MD	Malate dehydrogenase, mitochondrial OS=Rattus norvegicus GN=Mdh2 PE=1 SV=2	FVFSLVDAMNGK
>	sp|P04636|MD	Malate dehydrogenase, mitochondrial OS=Rattus norvegicus GN=Mdh2 PE=1 SV=2	GCDVVVIPAGVPR
>	sp|P04636|MD	Malate dehydrogenase, mitochondrial OS=Rattus norvegicus GN=Mdh2 PE=1 SV=2	GYLGPEQLPDCLK
>	sp|P04636|MD	Malate dehydrogenase, mitochondrial OS=Rattus norvegicus GN=Mdh2 PE=1 SV=2	IFGVTTLDIVR
>	sp|P04636|MD	Malate dehydrogenase, mitochondrial OS=Rattus norvegicus GN=Mdh2 PE=1 SV=2	IFGVTTLDIVR
>	sp|P04636|MD	Malate dehydrogenase, mitochondrial OS=Rattus norvegicus GN=Mdh2 PE=1 SV=2	LTLYDIAHTPGVAADLSHIETR
>	sp|P04636|MD	Malate dehydrogenase, mitochondrial OS=Rattus norvegicus GN=Mdh2 PE=1 SV=2	LTLYDIAHTPGVAADLSHIETR
>	sp|P04636|MD	Malate dehydrogenase, mitochondrial OS=Rattus norvegicus GN=Mdh2 PE=1 SV=2	LTLYDIAHTPGVAADLSHIETR
>	sp|P04636|MD	Malate dehydrogenase, mitochondrial OS=Rattus norvegicus GN=Mdh2 PE=1 SV=2	LTLYDIAHTPGVAADLSHIETR
>	sp|P04636|MD	Malate dehydrogenase, mitochondrial OS=Rattus norvegicus GN=Mdh2 PE=1 SV=2	LTLYDIAHTPGVAADLSHIETR
>	sp|P04636|MD	Malate dehydrogenase, mitochondrial OS=Rattus norvegicus GN=Mdh2 PE=1 SV=2	LTLYDIAHTPGVAADLSHIETR
>	sp|P04636|MD	Malate dehydrogenase, mitochondrial OS=Rattus norvegicus GN=Mdh2 PE=1 SV=2	LTLYDIAHTPGVAADLSHIETR
>	sp|P04636|MD	Malate dehydrogenase, mitochondrial OS=Rattus norvegicus GN=Mdh2 PE=1 SV=2	LTLYDIAHTPGVAADLSHIETR
>	sp|P04636|MD	Malate dehydrogenase, mitochondrial OS=Rattus norvegicus GN=Mdh2 PE=1 SV=2	LTLYDIAHTPGVAADLSHIETR
>	sp|P04636|MD	Malate dehydrogenase, mitochondrial OS=Rattus norvegicus GN=Mdh2 PE=1 SV=2	LTLYDIAHTPGVAADLSHIETR
>	sp|P04636|MD	Malate dehydrogenase, mitochondrial OS=Rattus norvegicus GN=Mdh2 PE=1 SV=2	LTLYDIAHTPGVAADLSHIETR
>	sp|P04636|MD	Malate dehydrogenase, mitochondrial OS=Rattus norvegicus GN=Mdh2 PE=1 SV=2	LTLYDIAHTPGVAADLSHIETR
>	sp|P04636|MD	Malate dehydrogenase, mitochondrial OS=Rattus norvegicus GN=Mdh2 PE=1 SV=2	LTLYDIAHTPGVAADLSHIETR
>	sp|P04636|MD	Malate dehydrogenase, mitochondrial OS=Rattus norvegicus GN=Mdh2 PE=1 SV=2	LTLYDIAHTPGVAADLSHIETR
>	sp|P04636|MD	Malate dehydrogenase, mitochondrial OS=Rattus norvegicus GN=Mdh2 PE=1 SV=2	LTLYDIAHTPGVAADLSHIETR
>	sp|P04636|MD	Malate dehydrogenase, mitochondrial OS=Rattus norvegicus GN=Mdh2 PE=1 SV=2	LTLYDIAHTPGVAADLSHIETR
>	sp|P04636|MD	Malate dehydrogenase, mitochondrial OS=Rattus norvegicus GN=Mdh2 PE=1 SV=2	LTLYDIAHTPGVAADLSHIETR

>	sp|P04636|MD	Malate dehydrogenase, mitochondrial OS=Rattus norvegicus GN=Mdh2 PE=1 SV=2	LTLYDIAHTPGVAADLSHIETR
>	sp|P04636|MD	Malate dehydrogenase, mitochondrial OS=Rattus norvegicus GN=Mdh2 PE=1 SV=2	LTLYDIAHTPGVAADLSHIETR
>	sp|P04636|MD	Malate dehydrogenase, mitochondrial OS=Rattus norvegicus GN=Mdh2 PE=1 SV=2	LTLYDIAHTPGVAADLSHIETR
>	sp|P04636|MD	Malate dehydrogenase, mitochondrial OS=Rattus norvegicus GN=Mdh2 PE=1 SV=2	LTLYDIAHTPGVAADLSHIETR
>	sp|P04636|MD	Malate dehydrogenase, mitochondrial OS=Rattus norvegicus GN=Mdh2 PE=1 SV=2	LTLYDIAHTPGVAADLSHIETR
>	sp|P04636|MD	Malate dehydrogenase, mitochondrial OS=Rattus norvegicus GN=Mdh2 PE=1 SV=2	LTLYDIAHTPGVAADLSHIETR
>	sp|P04636|MD	Malate dehydrogenase, mitochondrial OS=Rattus norvegicus GN=Mdh2 PE=1 SV=2	VAVLGASGGIGQPLSLLLK
>	sp|P04636|MD	Malate dehydrogenase, mitochondrial OS=Rattus norvegicus GN=Mdh2 PE=1 SV=2	VAVLGASGGIGQPLSLLLK
>	sp|P04636|MD	Malate dehydrogenase, mitochondrial OS=Rattus norvegicus GN=Mdh2 PE=1 SV=2	VAVLGASGGIGQPLSLLLK
>	sp|P04636|MD	Malate dehydrogenase, mitochondrial OS=Rattus norvegicus GN=Mdh2 PE=1 SV=2	VAVLGASGGIGQPLSLLLK
>	sp|P04636|MD	Malate dehydrogenase, mitochondrial OS=Rattus norvegicus GN=Mdh2 PE=1 SV=2	VAVLGASGGIGQPLSLLLK
>	sp|P04636|MD	Malate dehydrogenase, mitochondrial OS=Rattus norvegicus GN=Mdh2 PE=1 SV=2	VAVLGASGGIGQPLSLLLK
>	sp|P04636|MD	Malate dehydrogenase, mitochondrial OS=Rattus norvegicus GN=Mdh2 PE=1 SV=2	VAVLGASGGIGQPLSLLLK
>	sp|P04636|MD	Malate dehydrogenase, mitochondrial OS=Rattus norvegicus GN=Mdh2 PE=1 SV=2	VAVLGASGGIGQPLSLLLK
>	sp|P04636|MD	Malate dehydrogenase, mitochondrial OS=Rattus norvegicus GN=Mdh2 PE=1 SV=2	VAVLGASGGIGQPLSLLLK
>	sp|P04636|MD	Malate dehydrogenase, mitochondrial OS=Rattus norvegicus GN=Mdh2 PE=1 SV=2	VAVLGASGGIGQPLSLLLK
>	sp|P04636|MD	Malate dehydrogenase, mitochondrial OS=Rattus norvegicus GN=Mdh2 PE=1 SV=2	VAVLGASGGIGQPLSLLLK
>	sp|P04636|MD	Malate dehydrogenase, mitochondrial OS=Rattus norvegicus GN=Mdh2 PE=1 SV=2	VDFPQDQLATLTGR
>	sp|P04636|MD	Malate dehydrogenase, mitochondrial OS=Rattus norvegicus GN=Mdh2 PE=1 SV=2	VNVPVIGGHAGK
>	sp|P04636|MD	Malate dehydrogenase, mitochondrial OS=Rattus norvegicus GN=Mdh2 PE=1 SV=2	FVFSLVDAMNGK
>	sp|P04636|MD	Malate dehydrogenase, mitochondrial OS=Rattus norvegicus GN=Mdh2 PE=1 SV=2	DDLFNTNATIVATLTAACAQHCPEAMICIISNPVNSTIPITAEVFKK
>	sp|P04636|MD	Malate dehydrogenase, mitochondrial OS=Rattus norvegicus GN=Mdh2 PE=1 SV=2	FVFSLVDAMNGK
>	sp|P04636|MD	Malate dehydrogenase, mitochondrial OS=Rattus norvegicus GN=Mdh2 PE=1 SV=2	ETECTYFSTPLLLGK
>	sp|P04636|MD	Malate dehydrogenase, mitochondrial OS=Rattus norvegicus GN=Mdh2 PE=1 SV=2	VAVLGASGGIGQPLSLLLK
>	sp|P04636|MD	Malate dehydrogenase, mitochondrial OS=Rattus norvegicus GN=Mdh2 PE=1 SV=2	VNVPVIGGHAGK
>	sp|P04636|MD	Malate dehydrogenase, mitochondrial OS=Rattus norvegicus GN=Mdh2 PE=1 SV=2	AGAGSATLSMAYAGAR
>	sp|P04636|MD	Malate dehydrogenase, mitochondrial OS=Rattus norvegicus GN=Mdh2 PE=1 SV=2	AGAGSATLSMAYAGAR
>	sp|P04636|MD	Malate dehydrogenase, mitochondrial OS=Rattus norvegicus GN=Mdh2 PE=1 SV=2	FVFSLVDAMNGK
>	sp|P04636|MD	Malate dehydrogenase, mitochondrial OS=Rattus norvegicus GN=Mdh2 PE=1 SV=2	MIAEAIPELK
>	sp|P04636|MD	Malate dehydrogenase, mitochondrial OS=Rattus norvegicus GN=Mdh2 PE=1 SV=2	AGAGSATLSMAYAGAR
>	sp|P04636|MD	Malate dehydrogenase, mitochondrial OS=Rattus norvegicus GN=Mdh2 PE=1 SV=2	GYLGPEQLPDCLK
>	sp|P04636|MD	Malate dehydrogenase, mitochondrial OS=Rattus norvegicus GN=Mdh2 PE=1 SV=2	FVFSLVDAMNGK
>	sp|P04636|MD	Malate dehydrogenase, mitochondrial OS=Rattus norvegicus GN=Mdh2 PE=1 SV=2	IQEAGTEVVK
>	sp|P04636|MD	Malate dehydrogenase, mitochondrial OS=Rattus norvegicus GN=Mdh2 PE=1 SV=2	DDLFNTNATIVATLTAACAQHCPEAMICIISNPVNSTIPITAEVFKK
>	sp|P04636|MD	Malate dehydrogenase, mitochondrial OS=Rattus norvegicus GN=Mdh2 PE=1 SV=2	VSRLTLYDIAHTPGVAADLSHIETR
>	sp|P04636|MD	Malate dehydrogenase, mitochondrial OS=Rattus norvegicus GN=Mdh2 PE=1 SV=2	AGAGSATLSMAYAGAR
>	sp|P04636|MD	Malate dehydrogenase, mitochondrial OS=Rattus norvegicus GN=Mdh2 PE=1 SV=2	VNVPVIGGHAGK
>	sp|P04636|MD	Malate dehydrogenase, mitochondrial OS=Rattus norvegicus GN=Mdh2 PE=1 SV=2	ANTFVAELK
>	sp|P38650|DYH	Cytoplasmic dynein 1 heavy chain 1 OS=Rattus norvegicus GN=Dync1h1 PE=1 SV=1	FNALFVRPHIR
>	sp|P38650|DYH	Cytoplasmic dynein 1 heavy chain 1 OS=Rattus norvegicus GN=Dync1h1 PE=1 SV=1	HYLDFINHYANLFHEK
>	sp|P38650|DYH	Cytoplasmic dynein 1 heavy chain 1 OS=Rattus norvegicus GN=Dync1h1 PE=1 SV=1	IKNVVHELR
>	sp|P38650|DYH	Cytoplasmic dynein 1 heavy chain 1 OS=Rattus norvegicus GN=Dync1h1 PE=1 SV=1	ILDDDTIITTLENLKR
>	sp|P38650|DYH	Cytoplasmic dynein 1 heavy chain 1 OS=Rattus norvegicus GN=Dync1h1 PE=1 SV=1	INEWLTLVEK
>	sp|P38650|DYH	Cytoplasmic dynein 1 heavy chain 1 OS=Rattus norvegicus GN=Dync1h1 PE=1 SV=1	KIAELEMGLLHLQQNIEIPEISLPIHPIITNVAK
>	sp|P38650|DYH	Cytoplasmic dynein 1 heavy chain 1 OS=Rattus norvegicus GN=Dync1h1 PE=1 SV=1	KLEHLITELVHQR
>	sp|P38650|DYH	Cytoplasmic dynein 1 heavy chain 1 OS=Rattus norvegicus GN=Dync1h1 PE=1 SV=1	KLVPLLLEDGGDAPAALEAALEEK
>	sp|P38650|DYH	Cytoplasmic dynein 1 heavy chain 1 OS=Rattus norvegicus GN=Dync1h1 PE=1 SV=1	LEHIMDLTR
>	sp|P38650|DYH	Cytoplasmic dynein 1 heavy chain 1 OS=Rattus norvegicus GN=Dync1h1 PE=1 SV=1	RSELEEQQMHLNVGLR
>	sp|P38650|DYH	Cytoplasmic dynein 1 heavy chain 1 OS=Rattus norvegicus GN=Dync1h1 PE=1 SV=1	NVHLAPGWLMQLEK
>	sp|P38650|DYH	Cytoplasmic dynein 1 heavy chain 1 OS=Rattus norvegicus GN=Dync1h1 PE=1 SV=1	AHQANQLYPFAISLIESVR
>	sp|P38650|DYH	Cytoplasmic dynein 1 heavy chain 1 OS=Rattus norvegicus GN=Dync1h1 PE=1 SV=1	INEWLTLVEK
>	sp|P38650|DYH	Cytoplasmic dynein 1 heavy chain 1 OS=Rattus norvegicus GN=Dync1h1 PE=1 SV=1	KLEHLITELVHQR
>	sp|P38650|DYH	Cytoplasmic dynein 1 heavy chain 1 OS=Rattus norvegicus GN=Dync1h1 PE=1 SV=1	KLEHLITELVHQR
>	sp|P38650|DYH	Cytoplasmic dynein 1 heavy chain 1 OS=Rattus norvegicus GN=Dync1h1 PE=1 SV=1	KLVPLLLEDGGDAPAALEAALEEK
>	sp|P38650|DYH	Cytoplasmic dynein 1 heavy chain 1 OS=Rattus norvegicus GN=Dync1h1 PE=1 SV=1	LEHIMDLTR
>	sp|P38650|DYH	Cytoplasmic dynein 1 heavy chain 1 OS=Rattus norvegicus GN=Dync1h1 PE=1 SV=1	NVHLAPGWLMQLEK
>	sp|P38650|DYH	Cytoplasmic dynein 1 heavy chain 1 OS=Rattus norvegicus GN=Dync1h1 PE=1 SV=1	IFVFEPPPGVK
>	sp|P38650|DYH	Cytoplasmic dynein 1 heavy chain 1 OS=Rattus norvegicus GN=Dync1h1 PE=1 SV=1	RSELEEQQMHLNVGLR
>	sp|P38650|DYH	Cytoplasmic dynein 1 heavy chain 1 OS=Rattus norvegicus GN=Dync1h1 PE=1 SV=1	RSELEEQQMHLNVGLR
>	sp|P38650|DYH	Cytoplasmic dynein 1 heavy chain 1 OS=Rattus norvegicus GN=Dync1h1 PE=1 SV=1	LEHIMDLTR
>	sp|P38650|DYH	Cytoplasmic dynein 1 heavy chain 1 OS=Rattus norvegicus GN=Dync1h1 PE=1 SV=1	VAAPDVVVPTLDTVR
>	sp|P38650|DYH	Cytoplasmic dynein 1 heavy chain 1 OS=Rattus norvegicus GN=Dync1h1 PE=1 SV=1	AHQANQLYPFAISLIESVR
>	sp|P38650|DYH	Cytoplasmic dynein 1 heavy chain 1 OS=Rattus norvegicus GN=Dync1h1 PE=1 SV=1	VAAPDVVVPTLDTVRHEALLYTWLAEHKPLVLCGPPGSGK
>	sp|P38650|DYH	Cytoplasmic dynein 1 heavy chain 1 OS=Rattus norvegicus GN=Dync1h1 PE=1 SV=1	LEGVEGVAHIIDPK
>	sp|P38650|DYH	Cytoplasmic dynein 1 heavy chain 1 OS=Rattus norvegicus GN=Dync1h1 PE=1 SV=1	INEWLTLVEK
>	sp|P38650|DYH	Cytoplasmic dynein 1 heavy chain 1 OS=Rattus norvegicus GN=Dync1h1 PE=1 SV=1	NVHLAPGWLMQLEK
>	sp|P38650|DYH	Cytoplasmic dynein 1 heavy chain 1 OS=Rattus norvegicus GN=Dync1h1 PE=1 SV=1	NVHLAPGWLMQLEK
>	sp|P38650|DYH	Cytoplasmic dynein 1 heavy chain 1 OS=Rattus norvegicus GN=Dync1h1 PE=1 SV=1	IKNVVHELR
>	sp|P38650|DYH	Cytoplasmic dynein 1 heavy chain 1 OS=Rattus norvegicus GN=Dync1h1 PE=1 SV=1	KIAELEMGLLHLQQNIEIPEISLPIHPIITNVAK
>	sp|P38650|DYH	Cytoplasmic dynein 1 heavy chain 1 OS=Rattus norvegicus GN=Dync1h1 PE=1 SV=1	TLHTTASNWLHLIPQTLSPLKR
>	sp|P05708|HXK	Hexokinase-1 OS=Rattus norvegicus GN=Hk1 PE=1 SV=4	AAQLCGAGMAAVVEK
>	sp|P05708|HXK	Hexokinase-1 OS=Rattus norvegicus GN=Hk1 PE=1 SV=4	GDFIALDLGGSSFR
>	sp|P05708|HXK	Hexokinase-1 OS=Rattus norvegicus GN=Hk1 PE=1 SV=4	GKFNTSDVSAIEKDKEGIQNAK
>	sp|P05708|HXK	Hexokinase-1 OS=Rattus norvegicus GN=Hk1 PE=1 SV=4	HIDLVEGDEGR

>	sp|P05708|HXK	Hexokinase-1 OS=Rattus norvegicus GN=Hk1 PE=1 SV=4	KLPVGFTFSFPCR
>	sp|P05708|HXK	Hexokinase-1 OS=Rattus norvegicus GN=Hk1 PE=1 SV=4	LRTEMEMGLR
>	sp|P05708|HXK	Hexokinase-1 OS=Rattus norvegicus GN=Hk1 PE=1 SV=4	QIEETLAHFR
>	sp|P05708|HXK	Hexokinase-1 OS=Rattus norvegicus GN=Hk1 PE=1 SV=4	SANLVAATLGAILNR
>	sp|P05708|HXK	Hexokinase-1 OS=Rattus norvegicus GN=Hk1 PE=1 SV=4	SANLVAATLGAILNRLRDNKGTPR
>	sp|P05708|HXK	Hexokinase-1 OS=Rattus norvegicus GN=Hk1 PE=1 SV=4	IDEAVLITWTK
>	sp|P05708|HXK	Hexokinase-1 OS=Rattus norvegicus GN=Hk1 PE=1 SV=4	ATDCEGHDVASLLR
>	sp|P05708|HXK	Hexokinase-1 OS=Rattus norvegicus GN=Hk1 PE=1 SV=4	GAAMVTAVAYR
>	sp|P05708|HXK	Hexokinase-1 OS=Rattus norvegicus GN=Hk1 PE=1 SV=4	NQNVSMESEIYDTPENIVHGSGTQLFDHVADCLGDFMEK
>	sp|P05708|HXK	Hexokinase-1 OS=Rattus norvegicus GN=Hk1 PE=1 SV=4	LGVEPSDVDCVSVQHICTIVSFR
>	sp|P05708|HXK	Hexokinase-1 OS=Rattus norvegicus GN=Hk1 PE=1 SV=4	GLDHLNVTVGVDGTLYK
>	sp|P05708|HXK	Hexokinase-1 OS=Rattus norvegicus GN=Hk1 PE=1 SV=4	AAQLCGAGMAAVVEK
>	sp|P05708|HXK	Hexokinase-1 OS=Rattus norvegicus GN=Hk1 PE=1 SV=4	AAQLCGAGMAAVVEK
>	sp|P05708|HXK	Hexokinase-1 OS=Rattus norvegicus GN=Hk1 PE=1 SV=4	ATDCEGHDVASLLR
>	sp|P05708|HXK	Hexokinase-1 OS=Rattus norvegicus GN=Hk1 PE=1 SV=4	ATDCEGHDVASLLR
>	sp|P05708|HXK	Hexokinase-1 OS=Rattus norvegicus GN=Hk1 PE=1 SV=4	GDFIALDLGGSSFR
>	sp|P05708|HXK	Hexokinase-1 OS=Rattus norvegicus GN=Hk1 PE=1 SV=4	GKFNTSDVSAIEKDKEGIQNAK
>	sp|P05708|HXK	Hexokinase-1 OS=Rattus norvegicus GN=Hk1 PE=1 SV=4	IDEAVLITWTK
>	sp|P05708|HXK	Hexokinase-1 OS=Rattus norvegicus GN=Hk1 PE=1 SV=4	KLPVGFTFSFPCR
>	sp|P05708|HXK	Hexokinase-1 OS=Rattus norvegicus GN=Hk1 PE=1 SV=4	KLPVGFTFSFPCR
>	sp|P05708|HXK	Hexokinase-1 OS=Rattus norvegicus GN=Hk1 PE=1 SV=4	KLPVGFTFSFPCR
>	sp|P05708|HXK	Hexokinase-1 OS=Rattus norvegicus GN=Hk1 PE=1 SV=4	KLPVGFTFSFPCR
>	sp|P05708|HXK	Hexokinase-1 OS=Rattus norvegicus GN=Hk1 PE=1 SV=4	KLPVGFTFSFPCR
>	sp|P05708|HXK	Hexokinase-1 OS=Rattus norvegicus GN=Hk1 PE=1 SV=4	LGVEPSDVDCVSVQHICTIVSFR
>	sp|P05708|HXK	Hexokinase-1 OS=Rattus norvegicus GN=Hk1 PE=1 SV=4	LGVEPSDVDCVSVQHICTIVSFR
>	sp|P05708|HXK	Hexokinase-1 OS=Rattus norvegicus GN=Hk1 PE=1 SV=4	QIEETLAHFR
>	sp|P05708|HXK	Hexokinase-1 OS=Rattus norvegicus GN=Hk1 PE=1 SV=4	QIEETLAHFR
>	sp|P05708|HXK	Hexokinase-1 OS=Rattus norvegicus GN=Hk1 PE=1 SV=4	QIEETLAHFR
>	sp|P05708|HXK	Hexokinase-1 OS=Rattus norvegicus GN=Hk1 PE=1 SV=4	QIEETLAHFR
>	sp|P05708|HXK	Hexokinase-1 OS=Rattus norvegicus GN=Hk1 PE=1 SV=4	GKFNTSDVSAIEKDKEGIQNAK
>	sp|P05708|HXK	Hexokinase-1 OS=Rattus norvegicus GN=Hk1 PE=1 SV=4	HIDLVEGDEGR
>	sp|P05708|HXK	Hexokinase-1 OS=Rattus norvegicus GN=Hk1 PE=1 SV=4	IDEAVLITWTK
>	sp|P05708|HXK	Hexokinase-1 OS=Rattus norvegicus GN=Hk1 PE=1 SV=4	GLDHLNVTVGVDGTLYK
>	sp|P05708|HXK	Hexokinase-1 OS=Rattus norvegicus GN=Hk1 PE=1 SV=4	QIEETLAHFR
>	sp|P05708|HXK	Hexokinase-1 OS=Rattus norvegicus GN=Hk1 PE=1 SV=4	GAALITAVGVR
>	sp|P05708|HXK	Hexokinase-1 OS=Rattus norvegicus GN=Hk1 PE=1 SV=4	ITPELLTR
>	sp|P05708|HXK	Hexokinase-1 OS=Rattus norvegicus GN=Hk1 PE=1 SV=4	GDFIALDLGGSSFR
>	sp|P05708|HXK	Hexokinase-1 OS=Rattus norvegicus GN=Hk1 PE=1 SV=4	NQNVSMESEIYDTPENIVHGSGTQLFDHVADCLGDFMEK
>	sp|P05708|HXK	Hexokinase-1 OS=Rattus norvegicus GN=Hk1 PE=1 SV=4	KLPVGFTFSFPCR
>	sp|P05708|HXK	Hexokinase-1 OS=Rattus norvegicus GN=Hk1 PE=1 SV=4	LRTEMEMGLR
>	sp|P05708|HXK	Hexokinase-1 OS=Rattus norvegicus GN=Hk1 PE=1 SV=4	GLDHLNVTVGVDGTLYK
>	sp|P05708|HXK	Hexokinase-1 OS=Rattus norvegicus GN=Hk1 PE=1 SV=4	NQNVSMESEIYDTPENIVHGSGTQLFDHVADCLGDFMEK
>	sp|P05708|HXK	Hexokinase-1 OS=Rattus norvegicus GN=Hk1 PE=1 SV=4	NQNVSMESEIYDTPENIVHGSGTQLFDHVADCLGDFMEK
>	sp|P05708|HXK	Hexokinase-1 OS=Rattus norvegicus GN=Hk1 PE=1 SV=4	NQNVSMESEIYDTPENIVHGSGTQLFDHVADCLGDFMEK
>	sp|P05708|HXK	Hexokinase-1 OS=Rattus norvegicus GN=Hk1 PE=1 SV=4	TVEMHNK
>	sp|P42123|LDH	L-lactate dehydrogenase B chain OS=Rattus norvegicus GN=Ldhb PE=1 SV=2	GMYGIENEVFLSLPCILNAR
>	sp|P42123|LDH	L-lactate dehydrogenase B chain OS=Rattus norvegicus GN=Ldhb PE=1 SV=2	ITVVGVGQVGMACAISILGK
>	sp|P42123|LDH	L-lactate dehydrogenase B chain OS=Rattus norvegicus GN=Ldhb PE=1 SV=2	IVVVTAGVR
>	sp|P42123|LDH	L-lactate dehydrogenase B chain OS=Rattus norvegicus GN=Ldhb PE=1 SV=2	LIAPVADDETAVPNNK
>	sp|P42123|LDH	L-lactate dehydrogenase B chain OS=Rattus norvegicus GN=Ldhb PE=1 SV=2	LKDDEVAQLR
>	sp|P42123|LDH	L-lactate dehydrogenase B chain OS=Rattus norvegicus GN=Ldhb PE=1 SV=2	LKGEMMDLQHGSLFLQTPK
>	sp|P42123|LDH	L-lactate dehydrogenase B chain OS=Rattus norvegicus GN=Ldhb PE=1 SV=2	LKGYTNWAIGLSVADLIESMLKNLSR
>	sp|P42123|LDH	L-lactate dehydrogenase B chain OS=Rattus norvegicus GN=Ldhb PE=1 SV=2	SLADELALVDVLEDK
>	sp|P42123|LDH	L-lactate dehydrogenase B chain OS=Rattus norvegicus GN=Ldhb PE=1 SV=2	SLADELALVDVLEDKLK
>	sp|P42123|LDH	L-lactate dehydrogenase B chain OS=Rattus norvegicus GN=Ldhb PE=1 SV=2	SLADELALVDVLEDKLKGEMMDLQHGSLFLQTPK
>	sp|P42123|LDH	L-lactate dehydrogenase B chain OS=Rattus norvegicus GN=Ldhb PE=1 SV=2	YSPDCTIIVVSNPVDILTYVTWK
>	sp|P42123|LDH	L-lactate dehydrogenase B chain OS=Rattus norvegicus GN=Ldhb PE=1 SV=2	IHPVSTMVK
>	sp|P42123|LDH	L-lactate dehydrogenase B chain OS=Rattus norvegicus GN=Ldhb PE=1 SV=2	GMYGIENEVFLSLPCILNAR
>	sp|P42123|LDH	L-lactate dehydrogenase B chain OS=Rattus norvegicus GN=Ldhb PE=1 SV=2	ITVVGVGQVGMACAISILGK
>	sp|P42123|LDH	L-lactate dehydrogenase B chain OS=Rattus norvegicus GN=Ldhb PE=1 SV=2	ITVVGVGQVGMACAISILGK
>	sp|P42123|LDH	L-lactate dehydrogenase B chain OS=Rattus norvegicus GN=Ldhb PE=1 SV=2	ITVVGVGQVGMACAISILGK
>	sp|P42123|LDH	L-lactate dehydrogenase B chain OS=Rattus norvegicus GN=Ldhb PE=1 SV=2	ITVVGVGQVGMACAISILGK
>	sp|P42123|LDH	L-lactate dehydrogenase B chain OS=Rattus norvegicus GN=Ldhb PE=1 SV=2	ITVVGVGQVGMACAISILGK
>	sp|P42123|LDH	L-lactate dehydrogenase B chain OS=Rattus norvegicus GN=Ldhb PE=1 SV=2	ITVVGVGQVGMACAISILGK
>	sp|P42123|LDH	L-lactate dehydrogenase B chain OS=Rattus norvegicus GN=Ldhb PE=1 SV=2	ITVVGVGQVGMACAISILGK
>	sp|P42123|LDH	L-lactate dehydrogenase B chain OS=Rattus norvegicus GN=Ldhb PE=1 SV=2	ITVVGVGQVGMACAISILGK
>	sp|P42123|LDH	L-lactate dehydrogenase B chain OS=Rattus norvegicus GN=Ldhb PE=1 SV=2	ITVVGVGQVGMACAISILGK
>	sp|P42123|LDH	L-lactate dehydrogenase B chain OS=Rattus norvegicus GN=Ldhb PE=1 SV=2	IVVVTAGVR
>	sp|P42123|LDH	L-lactate dehydrogenase B chain OS=Rattus norvegicus GN=Ldhb PE=1 SV=2	IVVVTAGVR
>	sp|P42123|LDH	L-lactate dehydrogenase B chain OS=Rattus norvegicus GN=Ldhb PE=1 SV=2	IVVVTAGVR
>	sp|P42123|LDH	L-lactate dehydrogenase B chain OS=Rattus norvegicus GN=Ldhb PE=1 SV=2	LIAPVADDETAVPNNK
>	sp|P42123|LDH	L-lactate dehydrogenase B chain OS=Rattus norvegicus GN=Ldhb PE=1 SV=2	LIAPVADDETAVPNNK
>	sp|P42123|LDH	L-lactate dehydrogenase B chain OS=Rattus norvegicus GN=Ldhb PE=1 SV=2	LKGEMMDLQHGSLFLQTPK
>	sp|P42123|LDH	L-lactate dehydrogenase B chain OS=Rattus norvegicus GN=Ldhb PE=1 SV=2	LKGYTNWAIGLSVADLIESMLKNLSR

>	sp|P42123|LDH L-lactate dehydrogenase B chain OS=Rattus norvegicus GN=Ldhb PE=1 SV=2	SLADELALVDVLEDK
>	sp|P42123|LDH L-lactate dehydrogenase B chain OS=Rattus norvegicus GN=Ldhb PE=1 SV=2	SLADELALVDVLEDK
>	sp|P42123|LDH L-lactate dehydrogenase B chain OS=Rattus norvegicus GN=Ldhb PE=1 SV=2	SLADELALVDVLEDK
>	sp|P42123|LDH L-lactate dehydrogenase B chain OS=Rattus norvegicus GN=Ldhb PE=1 SV=2	SLADELALVDVLEDK
>	sp|P42123|LDH L-lactate dehydrogenase B chain OS=Rattus norvegicus GN=Ldhb PE=1 SV=2	SLADELALVDVLEDK
>	sp|P42123|LDH L-lactate dehydrogenase B chain OS=Rattus norvegicus GN=Ldhb PE=1 SV=2	SLADELALVDVLEDKLK
>	sp|P42123|LDH L-lactate dehydrogenase B chain OS=Rattus norvegicus GN=Ldhb PE=1 SV=2	SLADELALVDVLEDKLK
>	sp|P42123|LDH L-lactate dehydrogenase B chain OS=Rattus norvegicus GN=Ldhb PE=1 SV=2	SLADELALVDVLEDKLK
>	sp|P42123|LDH L-lactate dehydrogenase B chain OS=Rattus norvegicus GN=Ldhb PE=1 SV=2	SLADELALVDVLEDKLK
>	sp|P42123|LDH L-lactate dehydrogenase B chain OS=Rattus norvegicus GN=Ldhb PE=1 SV=2	SLADELALVDVLEDKLK
>	sp|P42123|LDH L-lactate dehydrogenase B chain OS=Rattus norvegicus GN=Ldhb PE=1 SV=2	SLADELALVDVLEDKLK
>	sp|P42123|LDH L-lactate dehydrogenase B chain OS=Rattus norvegicus GN=Ldhb PE=1 SV=2	SLADELALVDVLEDKLK
>	sp|P42123|LDH L-lactate dehydrogenase B chain OS=Rattus norvegicus GN=Ldhb PE=1 SV=2	SLADELALVDVLEDKLK
>	sp|P42123|LDH L-lactate dehydrogenase B chain OS=Rattus norvegicus GN=Ldhb PE=1 SV=2	SLADELALVDVLEDKLK
>	sp|P42123|LDH L-lactate dehydrogenase B chain OS=Rattus norvegicus GN=Ldhb PE=1 SV=2	SLADELALVDVLEDKLK
>	sp|P42123|LDH L-lactate dehydrogenase B chain OS=Rattus norvegicus GN=Ldhb PE=1 SV=2	SLADELALVDVLEDKLK
>	sp|P42123|LDH L-lactate dehydrogenase B chain OS=Rattus norvegicus GN=Ldhb PE=1 SV=2	SLADELALVDVLEDKLK
>	sp|P42123|LDH L-lactate dehydrogenase B chain OS=Rattus norvegicus GN=Ldhb PE=1 SV=2	SLADELALVDVLEDKLK
>	sp|P42123|LDH L-lactate dehydrogenase B chain OS=Rattus norvegicus GN=Ldhb PE=1 SV=2	SLADELALVDVLEDKLK
>	sp|P42123|LDH L-lactate dehydrogenase B chain OS=Rattus norvegicus GN=Ldhb PE=1 SV=2	SLADELALVDVLEDKLKGEMMDLQHGSLFLQTPK
>	sp|P42123|LDH L-lactate dehydrogenase B chain OS=Rattus norvegicus GN=Ldhb PE=1 SV=2	SLADELALVDVLEDKLKGEMMDLQHGSLFLQTPK
>	sp|P42123|LDH L-lactate dehydrogenase B chain OS=Rattus norvegicus GN=Ldhb PE=1 SV=2	YSPDCTIIVVSNPVDILTYVTWK
>	sp|P42123|LDH L-lactate dehydrogenase B chain OS=Rattus norvegicus GN=Ldhb PE=1 SV=2	YSPDCTIIVVSNPVDILTYVTWK
>	sp|P42123|LDH L-lactate dehydrogenase B chain OS=Rattus norvegicus GN=Ldhb PE=1 SV=2	YSPDCTIIVVSNPVDILTYVTWK
>	sp|P42123|LDH L-lactate dehydrogenase B chain OS=Rattus norvegicus GN=Ldhb PE=1 SV=2	IVVVTAGVR
>	sp|P42123|LDH L-lactate dehydrogenase B chain OS=Rattus norvegicus GN=Ldhb PE=1 SV=2	SLADELALVDVLEDK
>	sp|P42123|LDH L-lactate dehydrogenase B chain OS=Rattus norvegicus GN=Ldhb PE=1 SV=2	FIIPQIVK
>	sp|P42123|LDH L-lactate dehydrogenase B chain OS=Rattus norvegicus GN=Ldhb PE=1 SV=2	SLADELALVDVLEDK
>	sp|P42123|LDH L-lactate dehydrogenase B chain OS=Rattus norvegicus GN=Ldhb PE=1 SV=2	YSPDCTIIVVSNPVDILTYVTWK
>	sp|P42123|LDH L-lactate dehydrogenase B chain OS=Rattus norvegicus GN=Ldhb PE=1 SV=2	YSPDCTIIVVSNPVDILTYVTWKLSGLPK
>	sp|P42123|LDH L-lactate dehydrogenase B chain OS=Rattus norvegicus GN=Ldhb PE=1 SV=2	GLTSVINQK
>	sp|P42123|LDH L-lactate dehydrogenase B chain OS=Rattus norvegicus GN=Ldhb PE=1 SV=2	FIIPQIVK
>	sp|P42123|LDH L-lactate dehydrogenase B chain OS=Rattus norvegicus GN=Ldhb PE=1 SV=2	MVVDSAYEVIK
>	sp|P42123|LDH L-lactate dehydrogenase B chain OS=Rattus norvegicus GN=Ldhb PE=1 SV=2	YSPDCTIIVVSNPVDILTYVTWK
>	sp|P42123|LDH L-lactate dehydrogenase B chain OS=Rattus norvegicus GN=Ldhb PE=1 SV=2	SLADELALVDVLEDKLK
>	sp|P42123|LDH L-lactate dehydrogenase B chain OS=Rattus norvegicus GN=Ldhb PE=1 SV=2	IVADKDYSVTANSK
>	sp|P42123|LDH L-lactate dehydrogenase B chain OS=Rattus norvegicus GN=Ldhb PE=1 SV=2	YSPDCTIIVVSNPVDILTYVTWK
>	sp|P42123|LDH L-lactate dehydrogenase B chain OS=Rattus norvegicus GN=Ldhb PE=1 SV=2	SADTLWDIQK
>	sp|P42123|LDH L-lactate dehydrogenase B chain OS=Rattus norvegicus GN=Ldhb PE=1 SV=2	SLADELALVDVLEDKLK
>	sp|P42123|LDH L-lactate dehydrogenase B chain OS=Rattus norvegicus GN=Ldhb PE=1 SV=2	SLADELALVDVLEDKLK
>	sp|P42123|LDH L-lactate dehydrogenase B chain OS=Rattus norvegicus GN=Ldhb PE=1 SV=2	SADTLWDIQK
>	sp|P42123|LDH L-lactate dehydrogenase B chain OS=Rattus norvegicus GN=Ldhb PE=1 SV=2	FIIPQIVK
>	sp|P15146|MA Microtubule-associated protein 2 OS=Rattus norvegicus GN=Map2 PE=1 SV=3	DLATDLSLIEVK
>	sp|P15146|MA Microtubule-associated protein 2 OS=Rattus norvegicus GN=Map2 PE=1 SV=3	GLSSVPEVAEVETTTK
>	sp|P15146|MA Microtubule-associated protein 2 OS=Rattus norvegicus GN=Map2 PE=1 SV=3	GVVESVVTIEDDFITVVQTTTDEGELGSHSVR
>	sp|P15146|MA Microtubule-associated protein 2 OS=Rattus norvegicus GN=Map2 PE=1 SV=3	IVQVVTAEAVAVLK
>	sp|P15146|MA Microtubule-associated protein 2 OS=Rattus norvegicus GN=Map2 PE=1 SV=3	IVQVVTAEAVAVLKGEQEK
>	sp|P15146|MA Microtubule-associated protein 2 OS=Rattus norvegicus GN=Map2 PE=1 SV=3	KDEWGLAAPISPGPLTPMR
>	sp|P15146|MA Microtubule-associated protein 2 OS=Rattus norvegicus GN=Map2 PE=1 SV=3	KTTATSGESAQAPSAFK
>	sp|P15146|MA Microtubule-associated protein 2 OS=Rattus norvegicus GN=Map2 PE=1 SV=3	SDTLQITDLLVPGSR
>	sp|P15146|MA Microtubule-associated protein 2 OS=Rattus norvegicus GN=Map2 PE=1 SV=3	VTSEPEAVSEKR
>	sp|P15146|MA Microtubule-associated protein 2 OS=Rattus norvegicus GN=Map2 PE=1 SV=3	MQSKPGEDFEHAALVPQPDTSK
>	sp|P15146|MA Microtubule-associated protein 2 OS=Rattus norvegicus GN=Map2 PE=1 SV=3	RLSNVSSSGSINLLESPQLATLAEDVTAALAK
>	sp|P15146|MA Microtubule-associated protein 2 OS=Rattus norvegicus GN=Map2 PE=1 SV=3	DLATDLSLIEVK
>	sp|P15146|MA Microtubule-associated protein 2 OS=Rattus norvegicus GN=Map2 PE=1 SV=3	DLATDLSLIEVK
>	sp|P15146|MA Microtubule-associated protein 2 OS=Rattus norvegicus GN=Map2 PE=1 SV=3	DLATDLSLIEVK
>	sp|P15146|MA Microtubule-associated protein 2 OS=Rattus norvegicus GN=Map2 PE=1 SV=3	DLATDLSLIEVK
>	sp|P15146|MA Microtubule-associated protein 2 OS=Rattus norvegicus GN=Map2 PE=1 SV=3	DLATDLSLIEVK
>	sp|P15146|MA Microtubule-associated protein 2 OS=Rattus norvegicus GN=Map2 PE=1 SV=3	GVVESVVTIEDDFITVVQTTTDEGELGSHSVR
>	sp|P15146|MA Microtubule-associated protein 2 OS=Rattus norvegicus GN=Map2 PE=1 SV=3	IVQVVTAEAVAVLK
>	sp|P15146|MA Microtubule-associated protein 2 OS=Rattus norvegicus GN=Map2 PE=1 SV=3	IVQVVTAEAVAVLK
>	sp|P15146|MA Microtubule-associated protein 2 OS=Rattus norvegicus GN=Map2 PE=1 SV=3	IVQVVTAEAVAVLKGEQEK
>	sp|P15146|MA Microtubule-associated protein 2 OS=Rattus norvegicus GN=Map2 PE=1 SV=3	IVQVVTAEAVAVLKGEQEK
>	sp|P15146|MA Microtubule-associated protein 2 OS=Rattus norvegicus GN=Map2 PE=1 SV=3	KDEWGLAAPISPGPLTPMR
>	sp|P15146|MA Microtubule-associated protein 2 OS=Rattus norvegicus GN=Map2 PE=1 SV=3	KDEWGLAAPISPGPLTPMR
>	sp|P15146|MA Microtubule-associated protein 2 OS=Rattus norvegicus GN=Map2 PE=1 SV=3	KDEWGLAAPISPGPLTPMR
>	sp|P15146|MA Microtubule-associated protein 2 OS=Rattus norvegicus GN=Map2 PE=1 SV=3	KTTATSGESAQAPSAFK
>	sp|P15146|MA Microtubule-associated protein 2 OS=Rattus norvegicus GN=Map2 PE=1 SV=3	MQSKPGEDFEHAALVPQPDTSK
>	sp|P15146|MA Microtubule-associated protein 2 OS=Rattus norvegicus GN=Map2 PE=1 SV=3	VDHGAEIITQSPSR
>	sp|P15146|MA Microtubule-associated protein 2 OS=Rattus norvegicus GN=Map2 PE=1 SV=3	YFETSALKEDVTR
>	sp|P15146|MA Microtubule-associated protein 2 OS=Rattus norvegicus GN=Map2 PE=1 SV=3	VDHGAEIITQSPSR
>	sp|P15146|MA Microtubule-associated protein 2 OS=Rattus norvegicus GN=Map2 PE=1 SV=3	DLATDLSLIEVK
>	sp|P15146|MA Microtubule-associated protein 2 OS=Rattus norvegicus GN=Map2 PE=1 SV=3	VTSEPEAVSEK
>	sp|P15146|MA Microtubule-associated protein 2 OS=Rattus norvegicus GN=Map2 PE=1 SV=3	VDHGAEIITQSPSR

>	sp|P15146|MA Microtubule-associated protein 2 OS=Rattus norvegicus GN=Map2 PE=1 SV=3	VDHGAEIITQSPSR
>	sp|P15146|MA Microtubule-associated protein 2 OS=Rattus norvegicus GN=Map2 PE=1 SV=3	IGSTDNIK
>	sp|P15146|MA Microtubule-associated protein 2 OS=Rattus norvegicus GN=Map2 PE=1 SV=3	VADVPVSEATTVLGDVHSPAVEGFVGENISGEEK
>	sp|P15146|MA Microtubule-associated protein 2 OS=Rattus norvegicus GN=Map2 PE=1 SV=3	KIDLSHVTSK
>	sp|P15146|MA Microtubule-associated protein 2 OS=Rattus norvegicus GN=Map2 PE=1 SV=3	FAAPVQPEEER
>	sp|P15146|MA Microtubule-associated protein 2 OS=Rattus norvegicus GN=Map2 PE=1 SV=3	ESEEVGDKVELFGLGVTYEQTSAK
>	sp|P15146|MA Microtubule-associated protein 2 OS=Rattus norvegicus GN=Map2 PE=1 SV=3	GVSGDREENSFSLNSSISSAR
>	sp|P15146|MA Microtubule-associated protein 2 OS=Rattus norvegicus GN=Map2 PE=1 SV=3	DLATDLSLIEVK
>	sp|P15146|MA Microtubule-associated protein 2 OS=Rattus norvegicus GN=Map2 PE=1 SV=3	RLSNVSSSGSINLLESPQLATLAEDVTAALAK
>	sp|P15146|MA Microtubule-associated protein 2 OS=Rattus norvegicus GN=Map2 PE=1 SV=3	LINQPLPDLK
>	sp|P15146|MA Microtubule-associated protein 2 OS=Rattus norvegicus GN=Map2 PE=1 SV=3	RGVSGDREENSFSLNSSISSAR
>	sp|P06685|AT1 Sodium/potassium-transporting ATPase subunit alpha-1 OS=Rattus norvegicus GN=Atp1a1 PE=1 SV=1	AVAGDASESALLK
>	sp|P06685|AT1 Sodium/potassium-transporting ATPase subunit alpha-1 OS=Rattus norvegicus GN=Atp1a1 PE=1 SV=1	EQPLDEELKDAFQNAYLELGGLGER
>	sp|P06685|AT1 Sodium/potassium-transporting ATPase subunit alpha-1 OS=Rattus norvegicus GN=Atp1a1 PE=1 SV=1	KADIGVAMGIVGSDVSK
>	sp|P06685|AT1 Sodium/potassium-transporting ATPase subunit alpha-1 OS=Rattus norvegicus GN=Atp1a1 PE=1 SV=1	LNIPVNQVNPR
>	sp|P06685|AT1 Sodium/potassium-transporting ATPase subunit alpha-1 OS=Rattus norvegicus GN=Atp1a1 PE=1 SV=1	LSLDELHR
>	sp|P06685|AT1 Sodium/potassium-transporting ATPase subunit alpha-1 OS=Rattus norvegicus GN=Atp1a1 PE=1 SV=1	MSINAEDVVVGDLVEVK
>	sp|P06685|AT1 Sodium/potassium-transporting ATPase subunit alpha-1 OS=Rattus norvegicus GN=Atp1a1 PE=1 SV=1	SPDFTNENPLETR
>	sp|P06685|AT1 Sodium/potassium-transporting ATPase subunit alpha-1 OS=Rattus norvegicus GN=Atp1a1 PE=1 SV=1	YHTEIVFAR
>	sp|P06685|AT1 Sodium/potassium-transporting ATPase subunit alpha-1 OS=Rattus norvegicus GN=Atp1a1 PE=1 SV=1	HLLVMK
>	sp|P06685|AT1 Sodium/potassium-transporting ATPase subunit alpha-1 OS=Rattus norvegicus GN=Atp1a1 PE=1 SV=1	LSLDELHRK
>	sp|P06685|AT1 Sodium/potassium-transporting ATPase subunit alpha-1 OS=Rattus norvegicus GN=Atp1a1 PE=1 SV=1	ADIGVAMGIVGSDVSK
>	sp|P06685|AT1 Sodium/potassium-transporting ATPase subunit alpha-1 OS=Rattus norvegicus GN=Atp1a1 PE=1 SV=1	GGDRIPADLR
>	sp|P06685|AT1 Sodium/potassium-transporting ATPase subunit alpha-1 OS=Rattus norvegicus GN=Atp1a1 PE=1 SV=1	MTVAHMWFDNQIHEADTTENQSGVSFDK
>	sp|P06685|AT1 Sodium/potassium-transporting ATPase subunit alpha-1 OS=Rattus norvegicus GN=Atp1a1 PE=1 SV=1	AAVPDAVGK
>	sp|P06685|AT1 Sodium/potassium-transporting ATPase subunit alpha-1 OS=Rattus norvegicus GN=Atp1a1 PE=1 SV=1	AAVPDAVGK
>	sp|P06685|AT1 Sodium/potassium-transporting ATPase subunit alpha-1 OS=Rattus norvegicus GN=Atp1a1 PE=1 SV=1	AAVPDAVGK
>	sp|P06685|AT1 Sodium/potassium-transporting ATPase subunit alpha-1 OS=Rattus norvegicus GN=Atp1a1 PE=1 SV=1	ADIGVAMGIVGSDVSK
>	sp|P06685|AT1 Sodium/potassium-transporting ATPase subunit alpha-1 OS=Rattus norvegicus GN=Atp1a1 PE=1 SV=1	EQPLDEELKDAFQNAYLELGGLGER
>	sp|P06685|AT1 Sodium/potassium-transporting ATPase subunit alpha-1 OS=Rattus norvegicus GN=Atp1a1 PE=1 SV=1	EQPLDEELKDAFQNAYLELGGLGER
>	sp|P06685|AT1 Sodium/potassium-transporting ATPase subunit alpha-1 OS=Rattus norvegicus GN=Atp1a1 PE=1 SV=1	EQPLDEELKDAFQNAYLELGGLGER
>	sp|P06685|AT1 Sodium/potassium-transporting ATPase subunit alpha-1 OS=Rattus norvegicus GN=Atp1a1 PE=1 SV=1	EQPLDEELKDAFQNAYLELGGLGER
>	sp|P06685|AT1 Sodium/potassium-transporting ATPase subunit alpha-1 OS=Rattus norvegicus GN=Atp1a1 PE=1 SV=1	EQPLDEELKDAFQNAYLELGGLGER
>	sp|P06685|AT1 Sodium/potassium-transporting ATPase subunit alpha-1 OS=Rattus norvegicus GN=Atp1a1 PE=1 SV=1	EQPLDEELKDAFQNAYLELGGLGER
>	sp|P06685|AT1 Sodium/potassium-transporting ATPase subunit alpha-1 OS=Rattus norvegicus GN=Atp1a1 PE=1 SV=1	EQPLDEELKDAFQNAYLELGGLGER
>	sp|P06685|AT1 Sodium/potassium-transporting ATPase subunit alpha-1 OS=Rattus norvegicus GN=Atp1a1 PE=1 SV=1	EQPLDEELKDAFQNAYLELGGLGER
>	sp|P06685|AT1 Sodium/potassium-transporting ATPase subunit alpha-1 OS=Rattus norvegicus GN=Atp1a1 PE=1 SV=1	EQPLDEELKDAFQNAYLELGGLGER
>	sp|P06685|AT1 Sodium/potassium-transporting ATPase subunit alpha-1 OS=Rattus norvegicus GN=Atp1a1 PE=1 SV=1	EQPLDEELKDAFQNAYLELGGLGER
>	sp|P06685|AT1 Sodium/potassium-transporting ATPase subunit alpha-1 OS=Rattus norvegicus GN=Atp1a1 PE=1 SV=1	EVSMDDHK
>	sp|P06685|AT1 Sodium/potassium-transporting ATPase subunit alpha-1 OS=Rattus norvegicus GN=Atp1a1 PE=1 SV=1	GGDRIPADLR
>	sp|P06685|AT1 Sodium/potassium-transporting ATPase subunit alpha-1 OS=Rattus norvegicus GN=Atp1a1 PE=1 SV=1	GVGIISEGNETVEDIAAR
>	sp|P06685|AT1 Sodium/potassium-transporting ATPase subunit alpha-1 OS=Rattus norvegicus GN=Atp1a1 PE=1 SV=1	GVGIISEGNETVEDIAAR
>	sp|P06685|AT1 Sodium/potassium-transporting ATPase subunit alpha-1 OS=Rattus norvegicus GN=Atp1a1 PE=1 SV=1	GVGIISEGNETVEDIAAR
>	sp|P06685|AT1 Sodium/potassium-transporting ATPase subunit alpha-1 OS=Rattus norvegicus GN=Atp1a1 PE=1 SV=1	GVGIISEGNETVEDIAAR
>	sp|P06685|AT1 Sodium/potassium-transporting ATPase subunit alpha-1 OS=Rattus norvegicus GN=Atp1a1 PE=1 SV=1	GVGIISEGNETVEDIAAR
>	sp|P06685|AT1 Sodium/potassium-transporting ATPase subunit alpha-1 OS=Rattus norvegicus GN=Atp1a1 PE=1 SV=1	GVGIISEGNETVEDIAAR
>	sp|P06685|AT1 Sodium/potassium-transporting ATPase subunit alpha-1 OS=Rattus norvegicus GN=Atp1a1 PE=1 SV=1	KADIGVAMGIVGSDVSK
>	sp|P06685|AT1 Sodium/potassium-transporting ATPase subunit alpha-1 OS=Rattus norvegicus GN=Atp1a1 PE=1 SV=1	KADIGVAMGIVGSDVSK
>	sp|P06685|AT1 Sodium/potassium-transporting ATPase subunit alpha-1 OS=Rattus norvegicus GN=Atp1a1 PE=1 SV=1	KADIGVAMGIVGSDVSK
>	sp|P06685|AT1 Sodium/potassium-transporting ATPase subunit alpha-1 OS=Rattus norvegicus GN=Atp1a1 PE=1 SV=1	KADIGVAMGIVGSDVSK
>	sp|P06685|AT1 Sodium/potassium-transporting ATPase subunit alpha-1 OS=Rattus norvegicus GN=Atp1a1 PE=1 SV=1	KADIGVAMGIVGSDVSK
>	sp|P06685|AT1 Sodium/potassium-transporting ATPase subunit alpha-1 OS=Rattus norvegicus GN=Atp1a1 PE=1 SV=1	KADIGVAMGIVGSDVSK
>	sp|P06685|AT1 Sodium/potassium-transporting ATPase subunit alpha-1 OS=Rattus norvegicus GN=Atp1a1 PE=1 SV=1	KADIGVAMGIVGSDVSK
>	sp|P06685|AT1 Sodium/potassium-transporting ATPase subunit alpha-1 OS=Rattus norvegicus GN=Atp1a1 PE=1 SV=1	KADIGVAMGIVGSDVSK
>	sp|P06685|AT1 Sodium/potassium-transporting ATPase subunit alpha-1 OS=Rattus norvegicus GN=Atp1a1 PE=1 SV=1	KADIGVAMGIVGSDVSK
>	sp|P06685|AT1 Sodium/potassium-transporting ATPase subunit alpha-1 OS=Rattus norvegicus GN=Atp1a1 PE=1 SV=1	KADIGVAMGIVGSDVSK
>	sp|P06685|AT1 Sodium/potassium-transporting ATPase subunit alpha-1 OS=Rattus norvegicus GN=Atp1a1 PE=1 SV=1	LIFDNLKK
>	sp|P06685|AT1 Sodium/potassium-transporting ATPase subunit alpha-1 OS=Rattus norvegicus GN=Atp1a1 PE=1 SV=1	LIIVEGCQR
>	sp|P06685|AT1 Sodium/potassium-transporting ATPase subunit alpha-1 OS=Rattus norvegicus GN=Atp1a1 PE=1 SV=1	LNIPVNQVNPR
>	sp|P06685|AT1 Sodium/potassium-transporting ATPase subunit alpha-1 OS=Rattus norvegicus GN=Atp1a1 PE=1 SV=1	LSLDELHR
>	sp|P06685|AT1 Sodium/potassium-transporting ATPase subunit alpha-1 OS=Rattus norvegicus GN=Atp1a1 PE=1 SV=1	MSINAEDVVVGDLVEVK
>	sp|P06685|AT1 Sodium/potassium-transporting ATPase subunit alpha-1 OS=Rattus norvegicus GN=Atp1a1 PE=1 SV=1	MTVAHMWFDNQIHEADTTENQSGVSFDK
>	sp|P06685|AT1 Sodium/potassium-transporting ATPase subunit alpha-1 OS=Rattus norvegicus GN=Atp1a1 PE=1 SV=1	MTVAHMWFDNQIHEADTTENQSGVSFDK
>	sp|P06685|AT1 Sodium/potassium-transporting ATPase subunit alpha-1 OS=Rattus norvegicus GN=Atp1a1 PE=1 SV=1	NLEAVETLGSTSTICSDK
>	sp|P06685|AT1 Sodium/potassium-transporting ATPase subunit alpha-1 OS=Rattus norvegicus GN=Atp1a1 PE=1 SV=1	NLEAVETLGSTSTICSDK
>	sp|P06685|AT1 Sodium/potassium-transporting ATPase subunit alpha-1 OS=Rattus norvegicus GN=Atp1a1 PE=1 SV=1	NLEAVETLGSTSTICSDK
>	sp|P06685|AT1 Sodium/potassium-transporting ATPase subunit alpha-1 OS=Rattus norvegicus GN=Atp1a1 PE=1 SV=1	NLEAVETLGSTSTICSDK
>	sp|P06685|AT1 Sodium/potassium-transporting ATPase subunit alpha-1 OS=Rattus norvegicus GN=Atp1a1 PE=1 SV=1	NLEAVETLGSTSTICSDK
>	sp|P06685|AT1 Sodium/potassium-transporting ATPase subunit alpha-1 OS=Rattus norvegicus GN=Atp1a1 PE=1 SV=1	NLEAVETLGSTSTICSDKTGTLTQNR
>	sp|P06685|AT1 Sodium/potassium-transporting ATPase subunit alpha-1 OS=Rattus norvegicus GN=Atp1a1 PE=1 SV=1	NLEAVETLGSTSTICSDKTGTLTQNR
>	sp|P06685|AT1 Sodium/potassium-transporting ATPase subunit alpha-1 OS=Rattus norvegicus GN=Atp1a1 PE=1 SV=1	NLEAVETLGSTSTICSDKTGTLTQNR
>	sp|P06685|AT1 Sodium/potassium-transporting ATPase subunit alpha-1 OS=Rattus norvegicus GN=Atp1a1 PE=1 SV=1	NLEAVETLGSTSTICSDKTGTLTQNR
>	sp|P06685|AT1 Sodium/potassium-transporting ATPase subunit alpha-1 OS=Rattus norvegicus GN=Atp1a1 PE=1 SV=1	NMVPQQALVIR
>	sp|P06685|AT1 Sodium/potassium-transporting ATPase subunit alpha-1 OS=Rattus norvegicus GN=Atp1a1 PE=1 SV=1	NMVPQQALVIR

> sp|P06685|AT1 Sodium/potassium-transporting ATPase subunit alpha-1 OS=Rattus norvegicus GN=Atp1a1 PE=1 SV=1 NMVPQQALVIR
> sp|P06685|AT1 Sodium/potassium-transporting ATPase subunit alpha-1 OS=Rattus norvegicus GN=Atp1a1 PE=1 SV=1 QAADMILLDDNFASIVTGVEEGR
> sp|P06685|AT1 Sodium/potassium-transporting ATPase subunit alpha-1 OS=Rattus norvegicus GN=Atp1a1 PE=1 SV=1 QAADMILLDDNFASIVTGVEEGR
> sp|P06685|AT1 Sodium/potassium-transporting ATPase subunit alpha-1 OS=Rattus norvegicus GN=Atp1a1 PE=1 SV=1 QAADMILLDDNFASIVTGVEEGR
> sp|P06685|AT1 Sodium/potassium-transporting ATPase subunit alpha-1 OS=Rattus norvegicus GN=Atp1a1 PE=1 SV=1 QGAIVAVTGDGVNDSPALK
> sp|P06685|AT1 Sodium/potassium-transporting ATPase subunit alpha-1 OS=Rattus norvegicus GN=Atp1a1 PE=1 SV=1 QGAIVAVTGDGVNDSPALK
> sp|P06685|AT1 Sodium/potassium-transporting ATPase subunit alpha-1 OS=Rattus norvegicus GN=Atp1a1 PE=1 SV=1 QGAIVAVTGDGVNDSPALK
> sp|P06685|AT1 Sodium/potassium-transporting ATPase subunit alpha-1 OS=Rattus norvegicus GN=Atp1a1 PE=1 SV=1 QGAIVAVTGDGVNDSPALK
> sp|P06685|AT1 Sodium/potassium-transporting ATPase subunit alpha-1 OS=Rattus norvegicus GN=Atp1a1 PE=1 SV=1 QGAIVAVTGDGVNDSPALK
> sp|P06685|AT1 Sodium/potassium-transporting ATPase subunit alpha-1 OS=Rattus norvegicus GN=Atp1a1 PE=1 SV=1 RNSVFQQGMK
> sp|P06685|AT1 Sodium/potassium-transporting ATPase subunit alpha-1 OS=Rattus norvegicus GN=Atp1a1 PE=1 SV=1 TDKLVNER
> sp|P06685|AT1 Sodium/potassium-transporting ATPase subunit alpha-1 OS=Rattus norvegicus GN=Atp1a1 PE=1 SV=1 VDNSSLTGESEPQTR
> sp|P06685|AT1 Sodium/potassium-transporting ATPase subunit alpha-1 OS=Rattus norvegicus GN=Atp1a1 PE=1 SV=1 VDNSSLTGESEPQTR
> sp|P06685|AT1 Sodium/potassium-transporting ATPase subunit alpha-1 OS=Rattus norvegicus GN=Atp1a1 PE=1 SV=1 VDNSSLTGESEPQTR
> sp|P06685|AT1 Sodium/potassium-transporting ATPase subunit alpha-1 OS=Rattus norvegicus GN=Atp1a1 PE=1 SV=1 VDNSSLTGESEPQTR
> sp|P06685|AT1 Sodium/potassium-transporting ATPase subunit alpha-1 OS=Rattus norvegicus GN=Atp1a1 PE=1 SV=1 VIMVTGDHPITAK
> sp|P06685|AT1 Sodium/potassium-transporting ATPase subunit alpha-1 OS=Rattus norvegicus GN=Atp1a1 PE=1 SV=1 VIMVTGDHPITAK
> sp|P06685|AT1 Sodium/potassium-transporting ATPase subunit alpha-1 OS=Rattus norvegicus GN=Atp1a1 PE=1 SV=1 VIMVTGDHPITAK
> sp|P06685|AT1 Sodium/potassium-transporting ATPase subunit alpha-1 OS=Rattus norvegicus GN=Atp1a1 PE=1 SV=1 VIMVTGDHPITAK
> sp|P06685|AT1 Sodium/potassium-transporting ATPase subunit alpha-1 OS=Rattus norvegicus GN=Atp1a1 PE=1 SV=1 KADIGVAMGIVGSDVSK
> sp|P06685|AT1 Sodium/potassium-transporting ATPase subunit alpha-1 OS=Rattus norvegicus GN=Atp1a1 PE=1 SV=1 MTVAHMWFDNQIHEADTTENQSGVSFDK
> sp|P06685|AT1 Sodium/potassium-transporting ATPase subunit alpha-1 OS=Rattus norvegicus GN=Atp1a1 PE=1 SV=1 NLEAVETLGSTSTICSDK
> sp|P06685|AT1 Sodium/potassium-transporting ATPase subunit alpha-1 OS=Rattus norvegicus GN=Atp1a1 PE=1 SV=1 IVEIPFNSTNK
> sp|P06685|AT1 Sodium/potassium-transporting ATPase subunit alpha-1 OS=Rattus norvegicus GN=Atp1a1 PE=1 SV=1 AVFQANQENLPILK
> sp|P06685|AT1 Sodium/potassium-transporting ATPase subunit alpha-1 OS=Rattus norvegicus GN=Atp1a1 PE=1 SV=1 NLEAVETLGSTSTICSDK
> sp|P06685|AT1 Sodium/potassium-transporting ATPase subunit alpha-1 OS=Rattus norvegicus GN=Atp1a1 PE=1 SV=1 MTVAHMWFDNQIHEADTTENQSGVSFDK
> sp|P06685|AT1 Sodium/potassium-transporting ATPase subunit alpha-1 OS=Rattus norvegicus GN=Atp1a1 PE=1 SV=1 AVFQANQENLPILK
> sp|P06685|AT1 Sodium/potassium-transporting ATPase subunit alpha-1 OS=Rattus norvegicus GN=Atp1a1 PE=1 SV=1 SPDFTNENPLETR
> sp|P06685|AT1 Sodium/potassium-transporting ATPase subunit alpha-1 OS=Rattus norvegicus GN=Atp1a1 PE=1 SV=1 NIAFFSTNCVEGTAR
> sp|P06685|AT1 Sodium/potassium-transporting ATPase subunit alpha-1 OS=Rattus norvegicus GN=Atp1a1 PE=1 SV=1 LNIPVNQVNPR
> sp|P06685|AT1 Sodium/potassium-transporting ATPase subunit alpha-1 OS=Rattus norvegicus GN=Atp1a1 PE=1 SV=1 NMVPQQALVIR
> sp|P06685|AT1 Sodium/potassium-transporting ATPase subunit alpha-1 OS=Rattus norvegicus GN=Atp1a1 PE=1 SV=1 DGPNALTPPPTTPEWVK
> sp|P06685|AT1 Sodium/potassium-transporting ATPase subunit alpha-1 OS=Rattus norvegicus GN=Atp1a1 PE=1 SV=1 MTVAHMWFDNQIHEADTTENQSGVSFDK
> sp|P06685|AT1 Sodium/potassium-transporting ATPase subunit alpha-1 OS=Rattus norvegicus GN=Atp1a1 PE=1 SV=1 EQPLDEELKDAFQNAYLELGGLGER
> sp|P06685|AT1 Sodium/potassium-transporting ATPase subunit alpha-1 OS=Rattus norvegicus GN=Atp1a1 PE=1 SV=1 ACVVHGSDLKDMTSEELDDILR
> sp|P06685|AT1 Sodium/potassium-transporting ATPase subunit alpha-1 OS=Rattus norvegicus GN=Atp1a1 PE=1 SV=1 EVSMDDHK
> sp|P06685|AT1 Sodium/potassium-transporting ATPase subunit alpha-1 OS=Rattus norvegicus GN=Atp1a1 PE=1 SV=1 NMVPQQALVIR
> sp|P06685|AT1 Sodium/potassium-transporting ATPase subunit alpha-1 OS=Rattus norvegicus GN=Atp1a1 PE=1 SV=1 KADIGVAMGIVGSDVSK
> sp|P06685|AT1 Sodium/potassium-transporting ATPase subunit alpha-1 OS=Rattus norvegicus GN=Atp1a1 PE=1 SV=1 AVFQANQENLPILK
> sp|P06685|AT1 Sodium/potassium-transporting ATPase subunit alpha-1 OS=Rattus norvegicus GN=Atp1a1 PE=1 SV=1 GIVVYTGDR
> sp|P06685|AT1 Sodium/potassium-transporting ATPase subunit alpha-1 OS=Rattus norvegicus GN=Atp1a1 PE=1 SV=1 NIAFFSTNCVEGTAR
> sp|P06685|AT1 Sodium/potassium-transporting ATPase subunit alpha-1 OS=Rattus norvegicus GN=Atp1a1 PE=1 SV=1 NMVPQQALVIR
> sp|P06685|AT1 Sodium/potassium-transporting ATPase subunit alpha-1 OS=Rattus norvegicus GN=Atp1a1 PE=1 SV=1 NSVFQQGMK
> sp|P06685|AT1 Sodium/potassium-transporting ATPase subunit alpha-1 OS=Rattus norvegicus GN=Atp1a1 PE=1 SV=1 LIFDNLK
> sp|P06685|AT1 Sodium/potassium-transporting ATPase subunit alpha-1 OS=Rattus norvegicus GN=Atp1a1 PE=1 SV=1 VIMVTGDHPITAK
> sp|P06685|AT1 Sodium/potassium-transporting ATPase subunit alpha-1 OS=Rattus norvegicus GN=Atp1a1 PE=1 SV=1 HLLVMK
> sp|P06685|AT1 Sodium/potassium-transporting ATPase subunit alpha-1 OS=Rattus norvegicus GN=Atp1a1 PE=1 SV=1 NLEAVETLGSTSTICSDK
> sp|P06685|AT1 Sodium/potassium-transporting ATPase subunit alpha-1 OS=Rattus norvegicus GN=Atp1a1 PE=1 SV=1 MVTGDHPITAK
> sp|P06685|AT1 Sodium/potassium-transporting ATPase subunit alpha-1 OS=Rattus norvegicus GN=Atp1a1 PE=1 SV=1 AAEILARDGPNALTPPPTTPEWVK
> sp|P06685|AT1 Sodium/potassium-transporting ATPase subunit alpha-1 OS=Rattus norvegicus GN=Atp1a1 PE=1 SV=1 VIMVTGDHPITAK
> sp|P06685|AT1 Sodium/potassium-transporting ATPase subunit alpha-1 OS=Rattus norvegicus GN=Atp1a1 PE=1 SV=1 LIIVEGCQR
> sp|P06685|AT1 Sodium/potassium-transporting ATPase subunit alpha-1 OS=Rattus norvegicus GN=Atp1a1 PE=1 SV=1 KNCLVK
> sp|P06685|AT1 Sodium/potassium-transporting ATPase subunit alpha-1 OS=Rattus norvegicus GN=Atp1a1 PE=1 SV=1 LSLDELHRK
> sp|P06685|AT1 Sodium/potassium-transporting ATPase subunit alpha-1 OS=Rattus norvegicus GN=Atp1a1 PE=1 SV=1 MTVAHMWFDNQIHEADTTENQSGVSFDK
> sp|P06685|AT1 Sodium/potassium-transporting ATPase subunit alpha-1 OS=Rattus norvegicus GN=Atp1a1 PE=1 SV=1 KADIGVAMGIVGSDVSK
> sp|P06685|AT1 Sodium/potassium-transporting ATPase subunit alpha-1 OS=Rattus norvegicus GN=Atp1a1 PE=1 SV=1 NLEAVETLGSTSTICSDK
> sp|P06685|AT1 Sodium/potassium-transporting ATPase subunit alpha-1 OS=Rattus norvegicus GN=Atp1a1 PE=1 SV=1 IVEIPFNSTNK
> sp|P06685|AT1 Sodium/potassium-transporting ATPase subunit alpha-1 OS=Rattus norvegicus GN=Atp1a1 PE=1 SV=1 HLLVMK
> sp|P06685|AT1 Sodium/potassium-transporting ATPase subunit alpha-1 OS=Rattus norvegicus GN=Atp1a1 PE=1 SV=1 AVFQANQENLPILK
> sp|P06685|AT1 Sodium/potassium-transporting ATPase subunit alpha-1 OS=Rattus norvegicus GN=Atp1a1 PE=1 SV=1 AVAGDASESALLK
> sp|P06685|AT1 Sodium/potassium-transporting ATPase subunit alpha-1 OS=Rattus norvegicus GN=Atp1a1 PE=1 SV=1 EQPLDEELKDAFQNAYLELGGLGER
> sp|P06685|AT1 Sodium/potassium-transporting ATPase subunit alpha-1 OS=Rattus norvegicus GN=Atp1a1 PE=1 SV=1 IVEIPFNSTNK
> sp|P06685|AT1 Sodium/potassium-transporting ATPase subunit alpha-1 OS=Rattus norvegicus GN=Atp1a1 PE=1 SV=1 KNCLVK
> sp|P06685|AT1 Sodium/potassium-transporting ATPase subunit alpha-1 OS=Rattus norvegicus GN=Atp1a1 PE=1 SV=1 AVFQANQENLPILK
> sp|P06685|AT1 Sodium/potassium-transporting ATPase subunit alpha-1 OS=Rattus norvegicus GN=Atp1a1 PE=1 SV=1 NPNASEPK
> sp|P23565|AIN Alpha-internexin OS=Rattus norvegicus GN=Ina PE=1 SV=2 ALEAELAALR
> sp|P23565|AIN Alpha-internexin OS=Rattus norvegicus GN=Ina PE=1 SV=2 AQLEEASSAR
> sp|P23565|AIN Alpha-internexin OS=Rattus norvegicus GN=Ina PE=1 SV=2 DGLAEEVQR
> sp|P23565|AIN Alpha-internexin OS=Rattus norvegicus GN=Ina PE=1 SV=2 HSAEVAGYQDSIGQLESDLR
> sp|P23565|AIN Alpha-internexin OS=Rattus norvegicus GN=Ina PE=1 SV=2 KVESLLDELAFVR
> sp|P23565|AIN Alpha-internexin OS=Rattus norvegicus GN=Ina PE=1 SV=2 NLQSAEEWYK
> sp|P23565|AIN Alpha-internexin OS=Rattus norvegicus GN=Ina PE=1 SV=2 QVHDEEVAELLATLQASSQAAAEVDVAVAKPDLTSALR
> sp|P23565|AIN Alpha-internexin OS=Rattus norvegicus GN=Ina PE=1 SV=2 RLPASDGLDLSQAAAR
> sp|P23565|AIN Alpha-internexin OS=Rattus norvegicus GN=Ina PE=1 SV=2 TIEIEGLR

>	sp|P23565|AIN Alpha-internexin OS=Rattus norvegicus GN=Ina PE=1 SV=2	KEEEEEEEEEEGASK
>	sp|P23565|AIN Alpha-internexin OS=Rattus norvegicus GN=Ina PE=1 SV=2	VGESFEETLEETVVSTK
>	sp|P23565|AIN Alpha-internexin OS=Rattus norvegicus GN=Ina PE=1 SV=2	STIEEITTSSSQK
>	sp|P23565|AIN Alpha-internexin OS=Rattus norvegicus GN=Ina PE=1 SV=2	AQQRDVDGATLARLDLEKKVESLLDELAFVR
>	sp|P23565|AIN Alpha-internexin OS=Rattus norvegicus GN=Ina PE=1 SV=2	ALEAELAALR
>	sp|P23565|AIN Alpha-internexin OS=Rattus norvegicus GN=Ina PE=1 SV=2	ALEAELAALR
>	sp|P23565|AIN Alpha-internexin OS=Rattus norvegicus GN=Ina PE=1 SV=2	ALEAELAALR
>	sp|P23565|AIN Alpha-internexin OS=Rattus norvegicus GN=Ina PE=1 SV=2	AQLEEASSAR
>	sp|P23565|AIN Alpha-internexin OS=Rattus norvegicus GN=Ina PE=1 SV=2	AQLEEASSAR
>	sp|P23565|AIN Alpha-internexin OS=Rattus norvegicus GN=Ina PE=1 SV=2	DGLAEEVQR
>	sp|P23565|AIN Alpha-internexin OS=Rattus norvegicus GN=Ina PE=1 SV=2	HSAEVAGYQDSIGQLESDLR
>	sp|P23565|AIN Alpha-internexin OS=Rattus norvegicus GN=Ina PE=1 SV=2	HSAEVAGYQDSIGQLESDLR
>	sp|P23565|AIN Alpha-internexin OS=Rattus norvegicus GN=Ina PE=1 SV=2	KEEEEEEEEEEGASK
>	sp|P23565|AIN Alpha-internexin OS=Rattus norvegicus GN=Ina PE=1 SV=2	KEEEEEEEEEEGASK
>	sp|P23565|AIN Alpha-internexin OS=Rattus norvegicus GN=Ina PE=1 SV=2	KEEEEEEEEEEGASK
>	sp|P23565|AIN Alpha-internexin OS=Rattus norvegicus GN=Ina PE=1 SV=2	KEEEEEEEEEEGASK
>	sp|P23565|AIN Alpha-internexin OS=Rattus norvegicus GN=Ina PE=1 SV=2	KLLEGEETR
>	sp|P23565|AIN Alpha-internexin OS=Rattus norvegicus GN=Ina PE=1 SV=2	KLLEGEETR
>	sp|P23565|AIN Alpha-internexin OS=Rattus norvegicus GN=Ina PE=1 SV=2	KLLEGEETR
>	sp|P23565|AIN Alpha-internexin OS=Rattus norvegicus GN=Ina PE=1 SV=2	KLLEGEETR
>	sp|P23565|AIN Alpha-internexin OS=Rattus norvegicus GN=Ina PE=1 SV=2	KLLEGEETR
>	sp|P23565|AIN Alpha-internexin OS=Rattus norvegicus GN=Ina PE=1 SV=2	KVESLLDELAFVR
>	sp|P23565|AIN Alpha-internexin OS=Rattus norvegicus GN=Ina PE=1 SV=2	KVESLLDELAFVR
>	sp|P23565|AIN Alpha-internexin OS=Rattus norvegicus GN=Ina PE=1 SV=2	KVESLLDELAFVR
>	sp|P23565|AIN Alpha-internexin OS=Rattus norvegicus GN=Ina PE=1 SV=2	KVESLLDELAFVR
>	sp|P23565|AIN Alpha-internexin OS=Rattus norvegicus GN=Ina PE=1 SV=2	KVESLLDELAFVR
>	sp|P23565|AIN Alpha-internexin OS=Rattus norvegicus GN=Ina PE=1 SV=2	KVESLLDELAFVR
>	sp|P23565|AIN Alpha-internexin OS=Rattus norvegicus GN=Ina PE=1 SV=2	KVESLLDELAFVR
>	sp|P23565|AIN Alpha-internexin OS=Rattus norvegicus GN=Ina PE=1 SV=2	KVESLLDELAFVR
>	sp|P23565|AIN Alpha-internexin OS=Rattus norvegicus GN=Ina PE=1 SV=2	KVESLLDELAFVR
>	sp|P23565|AIN Alpha-internexin OS=Rattus norvegicus GN=Ina PE=1 SV=2	KVESLLDELAFVR
>	sp|P23565|AIN Alpha-internexin OS=Rattus norvegicus GN=Ina PE=1 SV=2	KVESLLDELAFVR
>	sp|P23565|AIN Alpha-internexin OS=Rattus norvegicus GN=Ina PE=1 SV=2	KVESLLDELAFVR
>	sp|P23565|AIN Alpha-internexin OS=Rattus norvegicus GN=Ina PE=1 SV=2	KVESLLDELAFVR
>	sp|P23565|AIN Alpha-internexin OS=Rattus norvegicus GN=Ina PE=1 SV=2	KVESLLDELAFVR
>	sp|P23565|AIN Alpha-internexin OS=Rattus norvegicus GN=Ina PE=1 SV=2	KVESLLDELAFVR
>	sp|P23565|AIN Alpha-internexin OS=Rattus norvegicus GN=Ina PE=1 SV=2	LLEGEETR
>	sp|P23565|AIN Alpha-internexin OS=Rattus norvegicus GN=Ina PE=1 SV=2	LLEGEETR
>	sp|P23565|AIN Alpha-internexin OS=Rattus norvegicus GN=Ina PE=1 SV=2	MALDIEIAAYR
>	sp|P23565|AIN Alpha-internexin OS=Rattus norvegicus GN=Ina PE=1 SV=2	NLQSAEEWYK
>	sp|P23565|AIN Alpha-internexin OS=Rattus norvegicus GN=Ina PE=1 SV=2	QVHDEEVAELLATLQASSQAAAEVDVAVAKPDLTSALR
>	sp|P23565|AIN Alpha-internexin OS=Rattus norvegicus GN=Ina PE=1 SV=2	QVHDEEVAELLATLQASSQAAAEVDVAVAKPDLTSALR
>	sp|P23565|AIN Alpha-internexin OS=Rattus norvegicus GN=Ina PE=1 SV=2	QVHDEEVAELLATLQASSQAAAEVDVAVAKPDLTSALR
>	sp|P23565|AIN Alpha-internexin OS=Rattus norvegicus GN=Ina PE=1 SV=2	QVHDEEVAELLATLQASSQAAAEVDVAVAKPDLTSALR
>	sp|P23565|AIN Alpha-internexin OS=Rattus norvegicus GN=Ina PE=1 SV=2	QVHDEEVAELLATLQASSQAAAEVDVAVAKPDLTSALR
>	sp|P23565|AIN Alpha-internexin OS=Rattus norvegicus GN=Ina PE=1 SV=2	QVHDEEVAELLATLQASSQAAAEVDVAVAKPDLTSALR
>	sp|P23565|AIN Alpha-internexin OS=Rattus norvegicus GN=Ina PE=1 SV=2	QVHDEEVAELLATLQASSQAAAEVDVAVAKPDLTSALR
>	sp|P23565|AIN Alpha-internexin OS=Rattus norvegicus GN=Ina PE=1 SV=2	QVHDEEVAELLATLQASSQAAAEVDVAVAKPDLTSALR
>	sp|P23565|AIN Alpha-internexin OS=Rattus norvegicus GN=Ina PE=1 SV=2	QVHDEEVAELLATLQASSQAAAEVDVAVAKPDLTSALR
>	sp|P23565|AIN Alpha-internexin OS=Rattus norvegicus GN=Ina PE=1 SV=2	QVHDEEVAELLATLQASSQAAAEVDVAVAKPDLTSALR
>	sp|P23565|AIN Alpha-internexin OS=Rattus norvegicus GN=Ina PE=1 SV=2	QVHDEEVAELLATLQASSQAAAEVDVAVAKPDLTSALR
>	sp|P23565|AIN Alpha-internexin OS=Rattus norvegicus GN=Ina PE=1 SV=2	QVHDEEVAELLATLQASSQAAAEVDVAVAKPDLTSALR
>	sp|P23565|AIN Alpha-internexin OS=Rattus norvegicus GN=Ina PE=1 SV=2	QVHDEEVAELLATLQASSQAAAEVDVAVAKPDLTSALR
>	sp|P23565|AIN Alpha-internexin OS=Rattus norvegicus GN=Ina PE=1 SV=2	QVHDEEVAELLATLQASSQAAAEVDVAVAKPDLTSALR
>	sp|P23565|AIN Alpha-internexin OS=Rattus norvegicus GN=Ina PE=1 SV=2	QVHDEEVAELLATLQASSQAAAEVDVAVAKPDLTSALR
>	sp|P23565|AIN Alpha-internexin OS=Rattus norvegicus GN=Ina PE=1 SV=2	QVHDEEVAELLATLQASSQAAAEVDVAVAKPDLTSALR
>	sp|P23565|AIN Alpha-internexin OS=Rattus norvegicus GN=Ina PE=1 SV=2	QVHDEEVAELLATLQASSQAAAEVDVAVAKPDLTSALR
>	sp|P23565|AIN Alpha-internexin OS=Rattus norvegicus GN=Ina PE=1 SV=2	QVHDEEVAELLATLQASSQAAAEVDVAVAKPDLTSALR
>	sp|P23565|AIN Alpha-internexin OS=Rattus norvegicus GN=Ina PE=1 SV=2	QVHDEEVAELLATLQASSQAAAEVDVAVAKPDLTSALR
>	sp|P23565|AIN Alpha-internexin OS=Rattus norvegicus GN=Ina PE=1 SV=2	QVHDEEVAELLATLQASSQAAAEVDVAVAKPDLTSALR
>	sp|P23565|AIN Alpha-internexin OS=Rattus norvegicus GN=Ina PE=1 SV=2	QVHDEEVAELLATLQASSQAAAEVDVAVAKPDLTSALR
>	sp|P23565|AIN Alpha-internexin OS=Rattus norvegicus GN=Ina PE=1 SV=2	QVHDEEVAELLATLQASSQAAAEVDVAVAKPDLTSALR
>	sp|P23565|AIN Alpha-internexin OS=Rattus norvegicus GN=Ina PE=1 SV=2	QVHDEEVAELLATLQASSQAAAEVDVAVAKPDLTSALR
>	sp|P23565|AIN Alpha-internexin OS=Rattus norvegicus GN=Ina PE=1 SV=2	QVHDEEVAELLATLQASSQAAAEVDVAVAKPDLTSALR
>	sp|P23565|AIN Alpha-internexin OS=Rattus norvegicus GN=Ina PE=1 SV=2	QVHDEEVAELLATLQASSQAAAEVDVAVAKPDLTSALR
>	sp|P23565|AIN Alpha-internexin OS=Rattus norvegicus GN=Ina PE=1 SV=2	QVHDEEVAELLATLQASSQAAAEVDVAVAKPDLTSALR
>	sp|P23565|AIN Alpha-internexin OS=Rattus norvegicus GN=Ina PE=1 SV=2	QVHDEEVAELLATLQASSQAAAEVDVAVAKPDLTSALR
>	sp|P23565|AIN Alpha-internexin OS=Rattus norvegicus GN=Ina PE=1 SV=2	QVHDEEVAELLATLQASSQAAAEVDVAVAKPDLTSALR
>	sp|P23565|AIN Alpha-internexin OS=Rattus norvegicus GN=Ina PE=1 SV=2	QVHDEEVAELLATLQASSQAAAEVDVAVAKPDLTSALR
>	sp|P23565|AIN Alpha-internexin OS=Rattus norvegicus GN=Ina PE=1 SV=2	RLPASDGLDLSQAAAR
>	sp|P23565|AIN Alpha-internexin OS=Rattus norvegicus GN=Ina PE=1 SV=2	RLPASDGLDLSQAAAR
>	sp|P23565|AIN Alpha-internexin OS=Rattus norvegicus GN=Ina PE=1 SV=2	RLPASDGLDLSQAAAR
>	sp|P23565|AIN Alpha-internexin OS=Rattus norvegicus GN=Ina PE=1 SV=2	RLPASDGLDLSQAAAR
>	sp|P23565|AIN Alpha-internexin OS=Rattus norvegicus GN=Ina PE=1 SV=2	RLPASDGLDLSQAAAR

>	sp|P23565|AIN Alpha-internexin OS=Rattus norvegicus GN=Ina PE=1 SV=2	STIEEITTSSSQK
>	sp|P23565|AIN Alpha-internexin OS=Rattus norvegicus GN=Ina PE=1 SV=2	TIEIEGLR
>	sp|P23565|AIN Alpha-internexin OS=Rattus norvegicus GN=Ina PE=1 SV=2	TNEKEQLQGLNDR
>	sp|P23565|AIN Alpha-internexin OS=Rattus norvegicus GN=Ina PE=1 SV=2	TNEKEQLQGLNDR
>	sp|P23565|AIN Alpha-internexin OS=Rattus norvegicus GN=Ina PE=1 SV=2	VGESFEETLEETVVSTK
>	sp|P23565|AIN Alpha-internexin OS=Rattus norvegicus GN=Ina PE=1 SV=2	DGLAEEVQR
>	sp|P23565|AIN Alpha-internexin OS=Rattus norvegicus GN=Ina PE=1 SV=2	FSTSGLSISGLNPLPNPSYLLPPR
>	sp|P23565|AIN Alpha-internexin OS=Rattus norvegicus GN=Ina PE=1 SV=2	QVHDEEVAELLATLQASSQAAAEVDVAVAKPDLTSALR
>	sp|P23565|AIN Alpha-internexin OS=Rattus norvegicus GN=Ina PE=1 SV=2	EYQDLLNVK
>	sp|P23565|AIN Alpha-internexin OS=Rattus norvegicus GN=Ina PE=1 SV=2	TNEKEQLQGLNDR
>	sp|P23565|AIN Alpha-internexin OS=Rattus norvegicus GN=Ina PE=1 SV=2	LLEGEETR
>	sp|P23565|AIN Alpha-internexin OS=Rattus norvegicus GN=Ina PE=1 SV=2	DGLAEEVQR
>	sp|P23565|AIN Alpha-internexin OS=Rattus norvegicus GN=Ina PE=1 SV=2	STIEEITTSSSQK
>	sp|P23565|AIN Alpha-internexin OS=Rattus norvegicus GN=Ina PE=1 SV=2	HSAEVAGYQDSIGQLESDLR
>	sp|P23565|AIN Alpha-internexin OS=Rattus norvegicus GN=Ina PE=1 SV=2	VGESFEETLEETVVSTK
>	sp|P23565|AIN Alpha-internexin OS=Rattus norvegicus GN=Ina PE=1 SV=2	LLEGEETR
>	sp|P23565|AIN Alpha-internexin OS=Rattus norvegicus GN=Ina PE=1 SV=2	TIEIEGLR
>	sp|P23565|AIN Alpha-internexin OS=Rattus norvegicus GN=Ina PE=1 SV=2	LLEGEETR
>	sp|P23565|AIN Alpha-internexin OS=Rattus norvegicus GN=Ina PE=1 SV=2	FAVFIEK
>	sp|P23565|AIN Alpha-internexin OS=Rattus norvegicus GN=Ina PE=1 SV=2	FANLNEQAAR
>	sp|P23565|AIN Alpha-internexin OS=Rattus norvegicus GN=Ina PE=1 SV=2	VGESFEETLEETVVSTK
>	sp|P23565|AIN Alpha-internexin OS=Rattus norvegicus GN=Ina PE=1 SV=2	TIEIEGLR
>	sp|P23565|AIN Alpha-internexin OS=Rattus norvegicus GN=Ina PE=1 SV=2	LLEGEETR
>	sp|P23565|AIN Alpha-internexin OS=Rattus norvegicus GN=Ina PE=1 SV=2	VGESFEETLEETVVSTK
>	sp|P23565|AIN Alpha-internexin OS=Rattus norvegicus GN=Ina PE=1 SV=2	ALEAELAALR
>	sp|P23565|AIN Alpha-internexin OS=Rattus norvegicus GN=Ina PE=1 SV=2	QVHDEEVAELLATLQASSQAAAEVDVAVAKPDLTSALR
>	sp|P23565|AIN Alpha-internexin OS=Rattus norvegicus GN=Ina PE=1 SV=2	VSSAGLSLKKEEEEEEEEEGASK
>	sp|P23565|AIN Alpha-internexin OS=Rattus norvegicus GN=Ina PE=1 SV=2	TNEKEQLQGLNDR
>	sp|P23565|AIN Alpha-internexin OS=Rattus norvegicus GN=Ina PE=1 SV=2	NLQSAEEWYK
>	sp|P23565|AIN Alpha-internexin OS=Rattus norvegicus GN=Ina PE=1 SV=2	TNEKEQLQGLNDR
>	sp|P23565|AIN Alpha-internexin OS=Rattus norvegicus GN=Ina PE=1 SV=2	VGELFQR
>	sp|P23565|AIN Alpha-internexin OS=Rattus norvegicus GN=Ina PE=1 SV=2	DGLAEEVQR
>	sp|P23565|AIN Alpha-internexin OS=Rattus norvegicus GN=Ina PE=1 SV=2	FAVFIEK
>	sp|P23565|AIN Alpha-internexin OS=Rattus norvegicus GN=Ina PE=1 SV=2	LLEGEETR
>	sp|P23565|AIN Alpha-internexin OS=Rattus norvegicus GN=Ina PE=1 SV=2	MALDIEIAAYR
>	sp|P63102|143 14-3-3 protein zeta/delta OS=Rattus norvegicus GN=Ywhaz PE=1 SV=1	DICNDVLSLLEK
>	sp|P63102|143 14-3-3 protein zeta/delta OS=Rattus norvegicus GN=Ywhaz PE=1 SV=1	DSTLIMQLLR
>	sp|P63102|143 14-3-3 protein zeta/delta OS=Rattus norvegicus GN=Ywhaz PE=1 SV=1	EKIETELR
>	sp|P63102|143 14-3-3 protein zeta/delta OS=Rattus norvegicus GN=Ywhaz PE=1 SV=1	GIVDQSQQAYQEAFEISK
>	sp|P63102|143 14-3-3 protein zeta/delta OS=Rattus norvegicus GN=Ywhaz PE=1 SV=1	IETELRDICNDVLSLLEK
>	sp|P63102|143 14-3-3 protein zeta/delta OS=Rattus norvegicus GN=Ywhaz PE=1 SV=1	KGIVDQSQQAYQEAFEISK
>	sp|P63102|143 14-3-3 protein zeta/delta OS=Rattus norvegicus GN=Ywhaz PE=1 SV=1	NLLSVAYK
>	sp|P63102|143 14-3-3 protein zeta/delta OS=Rattus norvegicus GN=Ywhaz PE=1 SV=1	SVTEQGAELSNEER
>	sp|P63102|143 14-3-3 protein zeta/delta OS=Rattus norvegicus GN=Ywhaz PE=1 SV=1	TAFDEAIAELDTLSEESYK
>	sp|P63102|143 14-3-3 protein zeta/delta OS=Rattus norvegicus GN=Ywhaz PE=1 SV=1	VVSSIEQK
>	sp|P63102|143 14-3-3 protein zeta/delta OS=Rattus norvegicus GN=Ywhaz PE=1 SV=1	YLAEVAAGDDKK
>	sp|P63102|143 14-3-3 protein zeta/delta OS=Rattus norvegicus GN=Ywhaz PE=1 SV=1	TAFDEAIAELDTLSEESYKDSTLIMQLLR
>	sp|P63102|143 14-3-3 protein zeta/delta OS=Rattus norvegicus GN=Ywhaz PE=1 SV=1	DICNDVLSLLEK
>	sp|P63102|143 14-3-3 protein zeta/delta OS=Rattus norvegicus GN=Ywhaz PE=1 SV=1	DICNDVLSLLEK
>	sp|P63102|143 14-3-3 protein zeta/delta OS=Rattus norvegicus GN=Ywhaz PE=1 SV=1	GIVDQSQQAYQEAFEISK
>	sp|P63102|143 14-3-3 protein zeta/delta OS=Rattus norvegicus GN=Ywhaz PE=1 SV=1	GIVDQSQQAYQEAFEISK
>	sp|P63102|143 14-3-3 protein zeta/delta OS=Rattus norvegicus GN=Ywhaz PE=1 SV=1	KGIVDQSQQAYQEAFEISK
>	sp|P63102|143 14-3-3 protein zeta/delta OS=Rattus norvegicus GN=Ywhaz PE=1 SV=1	KGIVDQSQQAYQEAFEISK
>	sp|P63102|143 14-3-3 protein zeta/delta OS=Rattus norvegicus GN=Ywhaz PE=1 SV=1	KGIVDQSQQAYQEAFEISK
>	sp|P63102|143 14-3-3 protein zeta/delta OS=Rattus norvegicus GN=Ywhaz PE=1 SV=1	KGIVDQSQQAYQEAFEISK
>	sp|P63102|143 14-3-3 protein zeta/delta OS=Rattus norvegicus GN=Ywhaz PE=1 SV=1	KGIVDQSQQAYQEAFEISK
>	sp|P63102|143 14-3-3 protein zeta/delta OS=Rattus norvegicus GN=Ywhaz PE=1 SV=1	NLLSVAYK
>	sp|P63102|143 14-3-3 protein zeta/delta OS=Rattus norvegicus GN=Ywhaz PE=1 SV=1	NLLSVAYK
>	sp|P63102|143 14-3-3 protein zeta/delta OS=Rattus norvegicus GN=Ywhaz PE=1 SV=1	NLLSVAYK
>	sp|P63102|143 14-3-3 protein zeta/delta OS=Rattus norvegicus GN=Ywhaz PE=1 SV=1	NLLSVAYK
>	sp|P63102|143 14-3-3 protein zeta/delta OS=Rattus norvegicus GN=Ywhaz PE=1 SV=1	SVTEQGAELSNEER
>	sp|P63102|143 14-3-3 protein zeta/delta OS=Rattus norvegicus GN=Ywhaz PE=1 SV=1	SVTEQGAELSNEER
>	sp|P63102|143 14-3-3 protein zeta/delta OS=Rattus norvegicus GN=Ywhaz PE=1 SV=1	SVTEQGAELSNEER
>	sp|P63102|143 14-3-3 protein zeta/delta OS=Rattus norvegicus GN=Ywhaz PE=1 SV=1	SVTEQGAELSNEER
>	sp|P63102|143 14-3-3 protein zeta/delta OS=Rattus norvegicus GN=Ywhaz PE=1 SV=1	SVTEQGAELSNEER
>	sp|P63102|143 14-3-3 protein zeta/delta OS=Rattus norvegicus GN=Ywhaz PE=1 SV=1	TAFDEAIAELDTLSEESYK
>	sp|P63102|143 14-3-3 protein zeta/delta OS=Rattus norvegicus GN=Ywhaz PE=1 SV=1	TAFDEAIAELDTLSEESYK
>	sp|P63102|143 14-3-3 protein zeta/delta OS=Rattus norvegicus GN=Ywhaz PE=1 SV=1	TAFDEAIAELDTLSEESYK
>	sp|P63102|143 14-3-3 protein zeta/delta OS=Rattus norvegicus GN=Ywhaz PE=1 SV=1	YLAEVAAGDDKK
>	sp|P63102|143 14-3-3 protein zeta/delta OS=Rattus norvegicus GN=Ywhaz PE=1 SV=1	YLAEVAAGDDKK
>	sp|P63102|143 14-3-3 protein zeta/delta OS=Rattus norvegicus GN=Ywhaz PE=1 SV=1	YLAEVAAGDDKK
>	sp|P63102|143 14-3-3 protein zeta/delta OS=Rattus norvegicus GN=Ywhaz PE=1 SV=1	NLLSVAYK
>	sp|P63102|143 14-3-3 protein zeta/delta OS=Rattus norvegicus GN=Ywhaz PE=1 SV=1	EMQPTHPIR
>	sp|P63102|143 14-3-3 protein zeta/delta OS=Rattus norvegicus GN=Ywhaz PE=1 SV=1	DICNDVLSLLEK

>	sp|P63102|143	14-3-3 protein zeta/delta OS=Rattus norvegicus GN=Ywhaz PE=1 SV=1	KGIVDQSQQAYQEAFEISK
>	sp|P63102|143	14-3-3 protein zeta/delta OS=Rattus norvegicus GN=Ywhaz PE=1 SV=1	FLIPNASQPESK
>	sp|P63102|143	14-3-3 protein zeta/delta OS=Rattus norvegicus GN=Ywhaz PE=1 SV=1	GIVDQSQQAYQEAFEISK
>	sp|P63102|143	14-3-3 protein zeta/delta OS=Rattus norvegicus GN=Ywhaz PE=1 SV=1	YLAEVAAGDDKK
>	sp|P63102|143	14-3-3 protein zeta/delta OS=Rattus norvegicus GN=Ywhaz PE=1 SV=1	YLAEVAAGDDKK
>	sp|P63102|143	14-3-3 protein zeta/delta OS=Rattus norvegicus GN=Ywhaz PE=1 SV=1	YDDMAACMK
>	sp|P63102|143	14-3-3 protein zeta/delta OS=Rattus norvegicus GN=Ywhaz PE=1 SV=1	NLLSVAYK
>	sp|P63102|143	14-3-3 protein zeta/delta OS=Rattus norvegicus GN=Ywhaz PE=1 SV=1	YDDMAACMK
>	sp|P63102|143	14-3-3 protein zeta/delta OS=Rattus norvegicus GN=Ywhaz PE=1 SV=1	NLLSVAYK
>	sp|P63102|143	14-3-3 protein zeta/delta OS=Rattus norvegicus GN=Ywhaz PE=1 SV=1	NLLSVAYK
>	sp|P63102|143	14-3-3 protein zeta/delta OS=Rattus norvegicus GN=Ywhaz PE=1 SV=1	TAFDEAIAELDTLSEESYKDSTLIMQLLR
>	sp|P63102|143	14-3-3 protein zeta/delta OS=Rattus norvegicus GN=Ywhaz PE=1 SV=1	TAFDEAIAELDTLSEESYKDSTLIMQLLR
>	sp|P63102|143	14-3-3 protein zeta/delta OS=Rattus norvegicus GN=Ywhaz PE=1 SV=1	GIVDQSQQAYQEAFEISK
>	sp|P63102|143	14-3-3 protein zeta/delta OS=Rattus norvegicus GN=Ywhaz PE=1 SV=1	EKIETELRDICNDVLSLLEK
>	sp|P63102|143	14-3-3 protein zeta/delta OS=Rattus norvegicus GN=Ywhaz PE=1 SV=1	TAFDEAIAELDTLSEESYK
>	sp|P63102|143	14-3-3 protein zeta/delta OS=Rattus norvegicus GN=Ywhaz PE=1 SV=1	YDDMAACMK
>	sp|P63102|143	14-3-3 protein zeta/delta OS=Rattus norvegicus GN=Ywhaz PE=1 SV=1	KGIVDQSQQAYQEAFEISK
>	sp|P63102|143	14-3-3 protein zeta/delta OS=Rattus norvegicus GN=Ywhaz PE=1 SV=1	KGIVDQSQQAYQEAFEISK
>	sp|P63102|143	14-3-3 protein zeta/delta OS=Rattus norvegicus GN=Ywhaz PE=1 SV=1	YLAEVAAGDDK
>	sp|Q9QWN8|SI	Spectrin beta chain, brain 2 OS=Rattus norvegicus GN=Sptbn2 PE=1 SV=2	DALLLWCQMK
>	sp|Q9QWN8|SI	Spectrin beta chain, brain 2 OS=Rattus norvegicus GN=Sptbn2 PE=1 SV=2	DQADPQCLFLR
>	sp|Q9QWN8|SI	Spectrin beta chain, brain 2 OS=Rattus norvegicus GN=Sptbn2 PE=1 SV=2	EQQHLLASAETGR
>	sp|Q9QWN8|SI	Spectrin beta chain, brain 2 OS=Rattus norvegicus GN=Sptbn2 PE=1 SV=2	GNLEVLLFTIQSK
>	sp|Q9QWN8|SI	Spectrin beta chain, brain 2 OS=Rattus norvegicus GN=Sptbn2 PE=1 SV=2	HQAFMAELAANKDWLDKVDKEGR
>	sp|Q9QWN8|SI	Spectrin beta chain, brain 2 OS=Rattus norvegicus GN=Sptbn2 PE=1 SV=2	TVEKPPKFTEK
>	sp|Q9QWN8|SI	Spectrin beta chain, brain 2 OS=Rattus norvegicus GN=Sptbn2 PE=1 SV=2	VDSANALANGLIAGGHAAR
>	sp|Q9QWN8|SI	Spectrin beta chain, brain 2 OS=Rattus norvegicus GN=Sptbn2 PE=1 SV=2	VQAVDAVAAELAAEHYHDIK
>	sp|Q9QWN8|SI	Spectrin beta chain, brain 2 OS=Rattus norvegicus GN=Sptbn2 PE=1 SV=2	LAQAHGFQGFLR
>	sp|Q9QWN8|SI	Spectrin beta chain, brain 2 OS=Rattus norvegicus GN=Sptbn2 PE=1 SV=2	LEKAEHER
>	sp|Q9QWN8|SI	Spectrin beta chain, brain 2 OS=Rattus norvegicus GN=Sptbn2 PE=1 SV=2	HLAGVEDLLQLHELVEADIAVQAER
>	sp|Q9QWN8|SI	Spectrin beta chain, brain 2 OS=Rattus norvegicus GN=Sptbn2 PE=1 SV=2	VTAVSDIAEQLLK
>	sp|Q9QWN8|SI	Spectrin beta chain, brain 2 OS=Rattus norvegicus GN=Sptbn2 PE=1 SV=2	DVEDEILWVTER
>	sp|Q9QWN8|SI	Spectrin beta chain, brain 2 OS=Rattus norvegicus GN=Sptbn2 PE=1 SV=2	EQQHLLASAETGR
>	sp|Q9QWN8|SI	Spectrin beta chain, brain 2 OS=Rattus norvegicus GN=Sptbn2 PE=1 SV=2	GNLEVLLFTIQSK
>	sp|Q9QWN8|SI	Spectrin beta chain, brain 2 OS=Rattus norvegicus GN=Sptbn2 PE=1 SV=2	LEKAEHER
>	sp|Q9QWN8|SI	Spectrin beta chain, brain 2 OS=Rattus norvegicus GN=Sptbn2 PE=1 SV=2	EQQHLLASAETGR
>	sp|Q9QWN8|SI	Spectrin beta chain, brain 2 OS=Rattus norvegicus GN=Sptbn2 PE=1 SV=2	DALLLWCQMK
>	sp|Q9QWN8|SI	Spectrin beta chain, brain 2 OS=Rattus norvegicus GN=Sptbn2 PE=1 SV=2	HLAGVEDLLQLHELVEADIAVQAER
>	sp|Q9QWN8|SI	Spectrin beta chain, brain 2 OS=Rattus norvegicus GN=Sptbn2 PE=1 SV=2	DALLLWCQMK
>	sp|Q9QWN8|SI	Spectrin beta chain, brain 2 OS=Rattus norvegicus GN=Sptbn2 PE=1 SV=2	LSHELELR
>	sp|Q9QWN8|SI	Spectrin beta chain, brain 2 OS=Rattus norvegicus GN=Sptbn2 PE=1 SV=2	HQAFMAELAANKDWLDKVDKEGR
>	sp|Q9QWN8|SI	Spectrin beta chain, brain 2 OS=Rattus norvegicus GN=Sptbn2 PE=1 SV=2	LAQAHGFQGFLR
>	sp|Q9QWN8|SI	Spectrin beta chain, brain 2 OS=Rattus norvegicus GN=Sptbn2 PE=1 SV=2	DALLLWCQMK
>	sp|Q9QWN8|SI	Spectrin beta chain, brain 2 OS=Rattus norvegicus GN=Sptbn2 PE=1 SV=2	DALLLWCQMK
>	sp|Q9QWN8|SI	Spectrin beta chain, brain 2 OS=Rattus norvegicus GN=Sptbn2 PE=1 SV=2	RLQDFLR
>	sp|Q9QWN8|SI	Spectrin beta chain, brain 2 OS=Rattus norvegicus GN=Sptbn2 PE=1 SV=2	AVREK
>	sp|Q9QWN8|SI	Spectrin beta chain, brain 2 OS=Rattus norvegicus GN=Sptbn2 PE=1 SV=2	LAQAHGFQGFLR
>	sp|Q9QWN8|SI	Spectrin beta chain, brain 2 OS=Rattus norvegicus GN=Sptbn2 PE=1 SV=2	LISDINK
>	sp|Q9QWN8|SI	Spectrin beta chain, brain 2 OS=Rattus norvegicus GN=Sptbn2 PE=1 SV=2	VHLENMGSHDIVDGNHR
>	sp|Q9QWN8|SI	Spectrin beta chain, brain 2 OS=Rattus norvegicus GN=Sptbn2 PE=1 SV=2	AATRRARLEESRR
>	sp|P16884|NFH	Neurofilament heavy polypeptide OS=Rattus norvegicus GN=Nefh PE=1 SV=4	AKEEDKGLPQEPSKPK
>	sp|P16884|NFH	Neurofilament heavy polypeptide OS=Rattus norvegicus GN=Nefh PE=1 SV=4	AQLEGHTVQSTLQSEEWFR
>	sp|P16884|NFH	Neurofilament heavy polypeptide OS=Rattus norvegicus GN=Nefh PE=1 SV=4	HHQEEVGELLGQIQGCGAAQAQAQAEAR
>	sp|P16884|NFH	Neurofilament heavy polypeptide OS=Rattus norvegicus GN=Nefh PE=1 SV=4	LEQEHLLEDIAHVR
>	sp|P16884|NFH	Neurofilament heavy polypeptide OS=Rattus norvegicus GN=Nefh PE=1 SV=4	QREEAEAAAR
>	sp|P16884|NFH	Neurofilament heavy polypeptide OS=Rattus norvegicus GN=Nefh PE=1 SV=4	RHHQEEVGELLGQIQGCGAAQAQAQAEAR
>	sp|P16884|NFH	Neurofilament heavy polypeptide OS=Rattus norvegicus GN=Nefh PE=1 SV=4	SEKEQLQALNDR
>	sp|P16884|NFH	Neurofilament heavy polypeptide OS=Rattus norvegicus GN=Nefh PE=1 SV=4	SSSTDQKDSQPSEKAPEDK
>	sp|P16884|NFH	Neurofilament heavy polypeptide OS=Rattus norvegicus GN=Nefh PE=1 SV=4	HHQEEVGELLGQIQGCGAAQAQAQAEAR
>	sp|P16884|NFH	Neurofilament heavy polypeptide OS=Rattus norvegicus GN=Nefh PE=1 SV=4	HHQEEVGELLGQIQGCGAAQAQAQAEAR
>	sp|P16884|NFH	Neurofilament heavy polypeptide OS=Rattus norvegicus GN=Nefh PE=1 SV=4	LEQEHLLEDIAHVR
>	sp|P16884|NFH	Neurofilament heavy polypeptide OS=Rattus norvegicus GN=Nefh PE=1 SV=4	LEQEHLLEDIAHVR
>	sp|P16884|NFH	Neurofilament heavy polypeptide OS=Rattus norvegicus GN=Nefh PE=1 SV=4	LEQEHLLEDIAHVR
>	sp|P16884|NFH	Neurofilament heavy polypeptide OS=Rattus norvegicus GN=Nefh PE=1 SV=4	LEQEHLLEDIAHVR
>	sp|P16884|NFH	Neurofilament heavy polypeptide OS=Rattus norvegicus GN=Nefh PE=1 SV=4	LEQEHLLEDIAHVR
>	sp|P16884|NFH	Neurofilament heavy polypeptide OS=Rattus norvegicus GN=Nefh PE=1 SV=4	LEQEHLLEDIAHVR
>	sp|P16884|NFH	Neurofilament heavy polypeptide OS=Rattus norvegicus GN=Nefh PE=1 SV=4	LEQEHLLEDIAHVR
>	sp|P16884|NFH	Neurofilament heavy polypeptide OS=Rattus norvegicus GN=Nefh PE=1 SV=4	LEQEHLLEDIAHVR
>	sp|P16884|NFH	Neurofilament heavy polypeptide OS=Rattus norvegicus GN=Nefh PE=1 SV=4	LEQEHLLEDIAHVR
>	sp|P16884|NFH	Neurofilament heavy polypeptide OS=Rattus norvegicus GN=Nefh PE=1 SV=4	LEQEHLLEDIAHVR
>	sp|P16884|NFH	Neurofilament heavy polypeptide OS=Rattus norvegicus GN=Nefh PE=1 SV=4	LEQEHLLEDIAHVR
>	sp|P16884|NFH	Neurofilament heavy polypeptide OS=Rattus norvegicus GN=Nefh PE=1 SV=4	LEQEHLLEDIAHVR
>	sp|P16884|NFH	Neurofilament heavy polypeptide OS=Rattus norvegicus GN=Nefh PE=1 SV=4	MALDIEIAAYR
>	sp|P16884|NFH	Neurofilament heavy polypeptide OS=Rattus norvegicus GN=Nefh PE=1 SV=4	EYQDLLNVK

>	sp|P16884|NFH Neurofilament heavy polypeptide OS=Rattus norvegicus GN=Nefh PE=1 SV=4	LEQEHLLEDIAHVR
>	sp|P16884|NFH Neurofilament heavy polypeptide OS=Rattus norvegicus GN=Nefh PE=1 SV=4	SPAEAK
>	sp|P16884|NFH Neurofilament heavy polypeptide OS=Rattus norvegicus GN=Nefh PE=1 SV=4	LEQEHLLEDIAHVR
>	sp|P16884|NFH Neurofilament heavy polypeptide OS=Rattus norvegicus GN=Nefh PE=1 SV=4	LEQEHLLEDIAHVR
>	sp|P16884|NFH Neurofilament heavy polypeptide OS=Rattus norvegicus GN=Nefh PE=1 SV=4	KEEVPAAPEKK
>	sp|P16884|NFH Neurofilament heavy polypeptide OS=Rattus norvegicus GN=Nefh PE=1 SV=4	LEQEHLLEDIAHVR
>	sp|P16884|NFH Neurofilament heavy polypeptide OS=Rattus norvegicus GN=Nefh PE=1 SV=4	LEQEHLLEDIAHVR
>	sp|P16884|NFH Neurofilament heavy polypeptide OS=Rattus norvegicus GN=Nefh PE=1 SV=4	KAAAPEEETPAK
>	sp|P16884|NFH Neurofilament heavy polypeptide OS=Rattus norvegicus GN=Nefh PE=1 SV=4	FAQEAEAAR
>	sp|P16884|NFH Neurofilament heavy polypeptide OS=Rattus norvegicus GN=Nefh PE=1 SV=4	KEEVPAAPEKK
>	sp|P16884|NFH Neurofilament heavy polypeptide OS=Rattus norvegicus GN=Nefh PE=1 SV=4	LEQEHLLEDIAHVR
>	sp|P16884|NFH Neurofilament heavy polypeptide OS=Rattus norvegicus GN=Nefh PE=1 SV=4	LEQEHLLEDIAHVR
>	sp|P16884|NFH Neurofilament heavy polypeptide OS=Rattus norvegicus GN=Nefh PE=1 SV=4	LEQEHLLEDIAHVR
>	sp|P16884|NFH Neurofilament heavy polypeptide OS=Rattus norvegicus GN=Nefh PE=1 SV=4	AEEKEPLTEKPKDSPGEAK
>	sp|P16884|NFH Neurofilament heavy polypeptide OS=Rattus norvegicus GN=Nefh PE=1 SV=4	LEQEHLLEDIAHVR
>	sp|P16884|NFH Neurofilament heavy polypeptide OS=Rattus norvegicus GN=Nefh PE=1 SV=4	HQVDMASYQDAIQQLDNELR
>	sp|P16884|NFH Neurofilament heavy polypeptide OS=Rattus norvegicus GN=Nefh PE=1 SV=4	LGVKEEAKPK
>	sp|P16884|NFH Neurofilament heavy polypeptide OS=Rattus norvegicus GN=Nefh PE=1 SV=4	AKEPSKPSEK
>	sp|P16884|NFH Neurofilament heavy polypeptide OS=Rattus norvegicus GN=Nefh PE=1 SV=4	LEQEHLLEDIAHVR
>	sp|P16884|NFH Neurofilament heavy polypeptide OS=Rattus norvegicus GN=Nefh PE=1 SV=4	MALDIEIAAYR
>	sp|P34926|MA Microtubule-associated protein 1A OS=Rattus norvegicus GN=Map1a PE=1 SV=1	DTDLQQTQATEPR
>	sp|P34926|MA Microtubule-associated protein 1A OS=Rattus norvegicus GN=Map1a PE=1 SV=1	DTGLGEKPLPADATEQGHPSAAIQVTQPSGPVLEGEHVER
>	sp|P34926|MA Microtubule-associated protein 1A OS=Rattus norvegicus GN=Map1a PE=1 SV=1	HLDRIDSVLLTHIGADNLPGINGLLQR
>	sp|P34926|MA Microtubule-associated protein 1A OS=Rattus norvegicus GN=Map1a PE=1 SV=1	HSPGVSKEDSEEQTVKPGPEEGTSEEGKGPPTR
>	sp|P34926|MA Microtubule-associated protein 1A OS=Rattus norvegicus GN=Map1a PE=1 SV=1	NLISPELGVVFFNVPDKLR
>	sp|P34926|MA Microtubule-associated protein 1A OS=Rattus norvegicus GN=Map1a PE=1 SV=1	SIEEACLTLQHLNR
>	sp|P34926|MA Microtubule-associated protein 1A OS=Rattus norvegicus GN=Map1a PE=1 SV=1	TAEQKDETLKEDKTQGQK
>	sp|P34926|MA Microtubule-associated protein 1A OS=Rattus norvegicus GN=Map1a PE=1 SV=1	VAVVEQDLIIHQK
>	sp|P34926|MA Microtubule-associated protein 1A OS=Rattus norvegicus GN=Map1a PE=1 SV=1	VVSNTIEPLTLFHK
>	sp|P34926|MA Microtubule-associated protein 1A OS=Rattus norvegicus GN=Map1a PE=1 SV=1	TSTEEATEPQKDEVLR
>	sp|P34926|MA Microtubule-associated protein 1A OS=Rattus norvegicus GN=Map1a PE=1 SV=1	LSGQYAAVFGAPGHTLPPGEPALGEVEER
>	sp|P34926|MA Microtubule-associated protein 1A OS=Rattus norvegicus GN=Map1a PE=1 SV=1	GAAPPAAVSGHR
>	sp|P34926|MA Microtubule-associated protein 1A OS=Rattus norvegicus GN=Map1a PE=1 SV=1	DTGLGEKPLPADATEQGHPSAAIQVTQPSGPVLEGEHVER
>	sp|P34926|MA Microtubule-associated protein 1A OS=Rattus norvegicus GN=Map1a PE=1 SV=1	DTGLGEKPLPADATEQGHPSAAIQVTQPSGPVLEGEHVER
>	sp|P34926|MA Microtubule-associated protein 1A OS=Rattus norvegicus GN=Map1a PE=1 SV=1	DTGLGEKPLPADATEQGHPSAAIQVTQPSGPVLEGEHVER
>	sp|P34926|MA Microtubule-associated protein 1A OS=Rattus norvegicus GN=Map1a PE=1 SV=1	DTGLGEKPLPADATEQGHPSAAIQVTQPSGPVLEGEHVER
>	sp|P34926|MA Microtubule-associated protein 1A OS=Rattus norvegicus GN=Map1a PE=1 SV=1	DTGLGEKPLPADATEQGHPSAAIQVTQPSGPVLEGEHVER
>	sp|P34926|MA Microtubule-associated protein 1A OS=Rattus norvegicus GN=Map1a PE=1 SV=1	DTGLGEKPLPADATEQGHPSAAIQVTQPSGPVLEGEHVER
>	sp|P34926|MA Microtubule-associated protein 1A OS=Rattus norvegicus GN=Map1a PE=1 SV=1	HSPGVSKEDSEEQTVKPGPEEGTSEEGKGPPTR
>	sp|P34926|MA Microtubule-associated protein 1A OS=Rattus norvegicus GN=Map1a PE=1 SV=1	HSPGVSKEDSEEQTVKPGPEEGTSEEGKGPPTR
>	sp|P34926|MA Microtubule-associated protein 1A OS=Rattus norvegicus GN=Map1a PE=1 SV=1	HSPGVSKEDSEEQTVKPGPEEGTSEEGKGPPTR
>	sp|P34926|MA Microtubule-associated protein 1A OS=Rattus norvegicus GN=Map1a PE=1 SV=1	HSPGVSKEDSEEQTVKPGPEEGTSEEGKGPPTR
>	sp|P34926|MA Microtubule-associated protein 1A OS=Rattus norvegicus GN=Map1a PE=1 SV=1	NLISPELGVVFFNVPDKLR
>	sp|P34926|MA Microtubule-associated protein 1A OS=Rattus norvegicus GN=Map1a PE=1 SV=1	NLISPELGVVFFNVPDKLR
>	sp|P34926|MA Microtubule-associated protein 1A OS=Rattus norvegicus GN=Map1a PE=1 SV=1	NLISPELGVVFFNVPDKLR
>	sp|P34926|MA Microtubule-associated protein 1A OS=Rattus norvegicus GN=Map1a PE=1 SV=1	NLISPELGVVFFNVPDKLR
>	sp|P34926|MA Microtubule-associated protein 1A OS=Rattus norvegicus GN=Map1a PE=1 SV=1	NLISPELGVVFFNVPDKLR
>	sp|P34926|MA Microtubule-associated protein 1A OS=Rattus norvegicus GN=Map1a PE=1 SV=1	NLISPELGVVFFNVPDKLR
>	sp|P34926|MA Microtubule-associated protein 1A OS=Rattus norvegicus GN=Map1a PE=1 SV=1	NLISPELGVVFFNVPDKLR
>	sp|P34926|MA Microtubule-associated protein 1A OS=Rattus norvegicus GN=Map1a PE=1 SV=1	VAVVEQDLIIHQK
>	sp|P34926|MA Microtubule-associated protein 1A OS=Rattus norvegicus GN=Map1a PE=1 SV=1	ESTFLDEGPDEQEITPLQHTPR
>	sp|P34926|MA Microtubule-associated protein 1A OS=Rattus norvegicus GN=Map1a PE=1 SV=1	AQWGENLQVTLIPTHDTEVTR
>	sp|P34926|MA Microtubule-associated protein 1A OS=Rattus norvegicus GN=Map1a PE=1 SV=1	TSTEEATEPQKDEVLR
>	sp|P34926|MA Microtubule-associated protein 1A OS=Rattus norvegicus GN=Map1a PE=1 SV=1	ESTFLDEGPDEQEITPLQHTPR
>	sp|P34926|MA Microtubule-associated protein 1A OS=Rattus norvegicus GN=Map1a PE=1 SV=1	RSIEEACLTLQHLNR
>	sp|P34926|MA Microtubule-associated protein 1A OS=Rattus norvegicus GN=Map1a PE=1 SV=1	VVSNTIEPLTLFHK
>	sp|P34926|MA Microtubule-associated protein 1A OS=Rattus norvegicus GN=Map1a PE=1 SV=1	VAVVEQDLIIHQK
>	sp|P34926|MA Microtubule-associated protein 1A OS=Rattus norvegicus GN=Map1a PE=1 SV=1	NLISPELGVVFFNVPDKLR
>	sp|P34926|MA Microtubule-associated protein 1A OS=Rattus norvegicus GN=Map1a PE=1 SV=1	SIEEACLTLQHLNR
>	sp|P34926|MA Microtubule-associated protein 1A OS=Rattus norvegicus GN=Map1a PE=1 SV=1	DTGLGEKPLPADATEQGHPSAAIQVTQPSGPVLEGEHVER
>	sp|P34926|MA Microtubule-associated protein 1A OS=Rattus norvegicus GN=Map1a PE=1 SV=1	VAVVEQDLIIHQK
>	sp|P34926|MA Microtubule-associated protein 1A OS=Rattus norvegicus GN=Map1a PE=1 SV=1	DTGLGEKPLPADATEQGHPSAAIQVTQPSGPVLEGEHVER
>	sp|P21707|SYT Synaptotagmin-1 OS=Rattus norvegicus GN=Syt1 PE=1 SV=3	DLQSAEKEEQEK
>	sp|P21707|SYT Synaptotagmin-1 OS=Rattus norvegicus GN=Syt1 PE=1 SV=3	DQALKDDDAETGLTDGEEKEEPKEEEK
>	sp|P21707|SYT Synaptotagmin-1 OS=Rattus norvegicus GN=Syt1 PE=1 SV=3	HDIIGEFK
>	sp|P21707|SYT Synaptotagmin-1 OS=Rattus norvegicus GN=Syt1 PE=1 SV=3	RPIAQWHTLQVEEEVDAMLAVK
>	sp|P21707|SYT Synaptotagmin-1 OS=Rattus norvegicus GN=Syt1 PE=1 SV=3	RPIAQWHTLQVEEEVDAMLAVKK
>	sp|P21707|SYT Synaptotagmin-1 OS=Rattus norvegicus GN=Syt1 PE=1 SV=3	TLNPVFNEQFTFK
>	sp|P21707|SYT Synaptotagmin-1 OS=Rattus norvegicus GN=Syt1 PE=1 SV=3	TLVMAVYDFDR
>	sp|P21707|SYT Synaptotagmin-1 OS=Rattus norvegicus GN=Syt1 PE=1 SV=3	TMKDQALKDDDAETGLTDGEEKEEPKEEEK
>	sp|P21707|SYT Synaptotagmin-1 OS=Rattus norvegicus GN=Syt1 PE=1 SV=3	VQVVVTVLDYDKIGKNDAIGK
>	sp|P21707|SYT Synaptotagmin-1 OS=Rattus norvegicus GN=Syt1 PE=1 SV=3	HDIIGEFKVPMNTVDFGHVTEEWR
>	sp|P21707|SYT Synaptotagmin-1 OS=Rattus norvegicus GN=Syt1 PE=1 SV=3	VQVVVTVLDYDK
>	sp|P21707|SYT Synaptotagmin-1 OS=Rattus norvegicus GN=Syt1 PE=1 SV=3	VPMNTVDFGHVTEEWR

>	sp|P21707|SYT Synaptotagmin-1 OS=Rattus norvegicus GN=Syt1 PE=1 SV=3	LQYSLDYDFQNNQLLVGIIQAAELPALDMGGTSDPYVK
>	sp|P21707|SYT Synaptotagmin-1 OS=Rattus norvegicus GN=Syt1 PE=1 SV=3	LTVVILEAK
>	sp|P21707|SYT Synaptotagmin-1 OS=Rattus norvegicus GN=Syt1 PE=1 SV=3	DQALKDDDAETGLTDGEEKEEPKEEEK
>	sp|P21707|SYT Synaptotagmin-1 OS=Rattus norvegicus GN=Syt1 PE=1 SV=3	DQALKDDDAETGLTDGEEKEEPKEEEK
>	sp|P21707|SYT Synaptotagmin-1 OS=Rattus norvegicus GN=Syt1 PE=1 SV=3	HDIIGEFKVPMNTVDFGHVTEEWR
>	sp|P21707|SYT Synaptotagmin-1 OS=Rattus norvegicus GN=Syt1 PE=1 SV=3	HDIIGEFKVPMNTVDFGHVTEEWR
>	sp|P21707|SYT Synaptotagmin-1 OS=Rattus norvegicus GN=Syt1 PE=1 SV=3	RPIAQWHTLQVEEEVDAMLAVK
>	sp|P21707|SYT Synaptotagmin-1 OS=Rattus norvegicus GN=Syt1 PE=1 SV=3	RPIAQWHTLQVEEEVDAMLAVK
>	sp|P21707|SYT Synaptotagmin-1 OS=Rattus norvegicus GN=Syt1 PE=1 SV=3	RPIAQWHTLQVEEEVDAMLAVK
>	sp|P21707|SYT Synaptotagmin-1 OS=Rattus norvegicus GN=Syt1 PE=1 SV=3	RPIAQWHTLQVEEEVDAMLAVK
>	sp|P21707|SYT Synaptotagmin-1 OS=Rattus norvegicus GN=Syt1 PE=1 SV=3	RPIAQWHTLQVEEEVDAMLAVKK
>	sp|P21707|SYT Synaptotagmin-1 OS=Rattus norvegicus GN=Syt1 PE=1 SV=3	TLNPVFNEQFTFK
>	sp|P21707|SYT Synaptotagmin-1 OS=Rattus norvegicus GN=Syt1 PE=1 SV=3	TLVMAVYDFDR
>	sp|P21707|SYT Synaptotagmin-1 OS=Rattus norvegicus GN=Syt1 PE=1 SV=3	TMKDQALKDDDAETGLTDGEEKEEPKEEEK
>	sp|P21707|SYT Synaptotagmin-1 OS=Rattus norvegicus GN=Syt1 PE=1 SV=3	VQVVVTVLDYDK
>	sp|P21707|SYT Synaptotagmin-1 OS=Rattus norvegicus GN=Syt1 PE=1 SV=3	VQVVVTVLDYDK
>	sp|P21707|SYT Synaptotagmin-1 OS=Rattus norvegicus GN=Syt1 PE=1 SV=3	HDIIGEFKVPMNTVDFGHVTEEWR
>	sp|P21707|SYT Synaptotagmin-1 OS=Rattus norvegicus GN=Syt1 PE=1 SV=3	RPIAQWHTLQVEEEVDAMLAVK
>	sp|P21707|SYT Synaptotagmin-1 OS=Rattus norvegicus GN=Syt1 PE=1 SV=3	HDIIGEFKVPMNTVDFGHVTEEWR
>	sp|P21707|SYT Synaptotagmin-1 OS=Rattus norvegicus GN=Syt1 PE=1 SV=3	VQVVVTVLDYDK
>	sp|P21707|SYT Synaptotagmin-1 OS=Rattus norvegicus GN=Syt1 PE=1 SV=3	LTVVILEAK
>	sp|P21707|SYT Synaptotagmin-1 OS=Rattus norvegicus GN=Syt1 PE=1 SV=3	TLNPVFNEQFTFK
>	sp|P21707|SYT Synaptotagmin-1 OS=Rattus norvegicus GN=Syt1 PE=1 SV=3	DLQSAEKEEQEK
>	sp|P21707|SYT Synaptotagmin-1 OS=Rattus norvegicus GN=Syt1 PE=1 SV=3	LTVVILEAK
>	sp|P21707|SYT Synaptotagmin-1 OS=Rattus norvegicus GN=Syt1 PE=1 SV=3	DLQSAEKEEQEK
>	sp|P21707|SYT Synaptotagmin-1 OS=Rattus norvegicus GN=Syt1 PE=1 SV=3	TLNPVFNEQFTFK
>	sp|P21707|SYT Synaptotagmin-1 OS=Rattus norvegicus GN=Syt1 PE=1 SV=3	LQYSLDYDFQNNQLLVGIIQAAELPALDMGGTSDPYVK
>	sp|P21707|SYT Synaptotagmin-1 OS=Rattus norvegicus GN=Syt1 PE=1 SV=3	LGDICFSLR
>	sp|P21707|SYT Synaptotagmin-1 OS=Rattus norvegicus GN=Syt1 PE=1 SV=3	LGDICFSLR
>	sp|P21707|SYT Synaptotagmin-1 OS=Rattus norvegicus GN=Syt1 PE=1 SV=3	HDIIGEFKVPMNTVDFGHVTEEWR
>	sp|P21707|SYT Synaptotagmin-1 OS=Rattus norvegicus GN=Syt1 PE=1 SV=3	NTLNPYYNESFSFEVPFEQIQK
>	sp|P21707|SYT Synaptotagmin-1 OS=Rattus norvegicus GN=Syt1 PE=1 SV=3	VFVGYNSTGAELR
>	sp|P21707|SYT Synaptotagmin-1 OS=Rattus norvegicus GN=Syt1 PE=1 SV=3	NDAIGK
>	sp|P21707|SYT Synaptotagmin-1 OS=Rattus norvegicus GN=Syt1 PE=1 SV=3	VQVVVTVLDYDKIGKNDAIGK
>	sp|P48500|TPIS Triosephosphate isomerase OS=Rattus norvegicus GN=Tpi1 PE=1 SV=2	DLGATWVVLGHSER
>	sp|P48500|TPIS Triosephosphate isomerase OS=Rattus norvegicus GN=Tpi1 PE=1 SV=2	ELASQPDVDGFLVGGASLKPEFVDIINAK
>	sp|P48500|TPIS Triosephosphate isomerase OS=Rattus norvegicus GN=Tpi1 PE=1 SV=2	IIYGGSVTGATCK
>	sp|P48500|TPIS Triosephosphate isomerase OS=Rattus norvegicus GN=Tpi1 PE=1 SV=2	LPADTEVVCAPPTAYIDFAR
>	sp|P48500|TPIS Triosephosphate isomerase OS=Rattus norvegicus GN=Tpi1 PE=1 SV=2	RHIFGESDELIGQK
>	sp|P48500|TPIS Triosephosphate isomerase OS=Rattus norvegicus GN=Tpi1 PE=1 SV=2	TATPQQAQEVHEK
>	sp|P48500|TPIS Triosephosphate isomerase OS=Rattus norvegicus GN=Tpi1 PE=1 SV=2	VNHALSEGLGVIACIGEK
>	sp|P48500|TPIS Triosephosphate isomerase OS=Rattus norvegicus GN=Tpi1 PE=1 SV=2	VVLAYEPVWAIGTGK
>	sp|P48500|TPIS Triosephosphate isomerase OS=Rattus norvegicus GN=Tpi1 PE=1 SV=2	VTNGAFTGEISPGMIK
>	sp|P48500|TPIS Triosephosphate isomerase OS=Rattus norvegicus GN=Tpi1 PE=1 SV=2	ELASQPDVDGFLVGGASLKPEFVDIINAKQ
>	sp|P48500|TPIS Triosephosphate isomerase OS=Rattus norvegicus GN=Tpi1 PE=1 SV=2	DLGATWVVLGHSER
>	sp|P48500|TPIS Triosephosphate isomerase OS=Rattus norvegicus GN=Tpi1 PE=1 SV=2	DLGATWVVLGHSER
>	sp|P48500|TPIS Triosephosphate isomerase OS=Rattus norvegicus GN=Tpi1 PE=1 SV=2	DLGATWVVLGHSER
>	sp|P48500|TPIS Triosephosphate isomerase OS=Rattus norvegicus GN=Tpi1 PE=1 SV=2	DLGATWVVLGHSER
>	sp|P48500|TPIS Triosephosphate isomerase OS=Rattus norvegicus GN=Tpi1 PE=1 SV=2	DLGATWVVLGHSER
>	sp|P48500|TPIS Triosephosphate isomerase OS=Rattus norvegicus GN=Tpi1 PE=1 SV=2	DLGATWVVLGHSER
>	sp|P48500|TPIS Triosephosphate isomerase OS=Rattus norvegicus GN=Tpi1 PE=1 SV=2	ELASQPDVDGFLVGGASLKPEFVDIINAK
>	sp|P48500|TPIS Triosephosphate isomerase OS=Rattus norvegicus GN=Tpi1 PE=1 SV=2	ELASQPDVDGFLVGGASLKPEFVDIINAK
>	sp|P48500|TPIS Triosephosphate isomerase OS=Rattus norvegicus GN=Tpi1 PE=1 SV=2	ELASQPDVDGFLVGGASLKPEFVDIINAK
>	sp|P48500|TPIS Triosephosphate isomerase OS=Rattus norvegicus GN=Tpi1 PE=1 SV=2	ELASQPDVDGFLVGGASLKPEFVDIINAK
>	sp|P48500|TPIS Triosephosphate isomerase OS=Rattus norvegicus GN=Tpi1 PE=1 SV=2	ELASQPDVDGFLVGGASLKPEFVDIINAK
>	sp|P48500|TPIS Triosephosphate isomerase OS=Rattus norvegicus GN=Tpi1 PE=1 SV=2	ELASQPDVDGFLVGGASLKPEFVDIINAK
>	sp|P48500|TPIS Triosephosphate isomerase OS=Rattus norvegicus GN=Tpi1 PE=1 SV=2	IIYGGSVTGATCK
>	sp|P48500|TPIS Triosephosphate isomerase OS=Rattus norvegicus GN=Tpi1 PE=1 SV=2	IIYGGSVTGATCK
>	sp|P48500|TPIS Triosephosphate isomerase OS=Rattus norvegicus GN=Tpi1 PE=1 SV=2	LPADTEVVCAPPTAYIDFAR
>	sp|P48500|TPIS Triosephosphate isomerase OS=Rattus norvegicus GN=Tpi1 PE=1 SV=2	LPADTEVVCAPPTAYIDFAR
>	sp|P48500|TPIS Triosephosphate isomerase OS=Rattus norvegicus GN=Tpi1 PE=1 SV=2	RHIFGESDELIGQK
>	sp|P48500|TPIS Triosephosphate isomerase OS=Rattus norvegicus GN=Tpi1 PE=1 SV=2	TATPQQAQEVHEK
>	sp|P48500|TPIS Triosephosphate isomerase OS=Rattus norvegicus GN=Tpi1 PE=1 SV=2	TATPQQAQEVHEK
>	sp|P48500|TPIS Triosephosphate isomerase OS=Rattus norvegicus GN=Tpi1 PE=1 SV=2	TATPQQAQEVHEK
>	sp|P48500|TPIS Triosephosphate isomerase OS=Rattus norvegicus GN=Tpi1 PE=1 SV=2	VNHALSEGLGVIACIGEK
>	sp|P48500|TPIS Triosephosphate isomerase OS=Rattus norvegicus GN=Tpi1 PE=1 SV=2	VNHALSEGLGVIACIGEK
>	sp|P48500|TPIS Triosephosphate isomerase OS=Rattus norvegicus GN=Tpi1 PE=1 SV=2	VNHALSEGLGVIACIGEK
>	sp|P48500|TPIS Triosephosphate isomerase OS=Rattus norvegicus GN=Tpi1 PE=1 SV=2	VNHALSEGLGVIACIGEK
>	sp|P48500|TPIS Triosephosphate isomerase OS=Rattus norvegicus GN=Tpi1 PE=1 SV=2	VNHALSEGLGVIACIGEK
>	sp|P48500|TPIS Triosephosphate isomerase OS=Rattus norvegicus GN=Tpi1 PE=1 SV=2	VNHALSEGLGVIACIGEK
>	sp|P48500|TPIS Triosephosphate isomerase OS=Rattus norvegicus GN=Tpi1 PE=1 SV=2	VNHALSEGLGVIACIGEK
>	sp|P48500|TPIS Triosephosphate isomerase OS=Rattus norvegicus GN=Tpi1 PE=1 SV=2	VNHALSEGLGVIACIGEK
>	sp|P48500|TPIS Triosephosphate isomerase OS=Rattus norvegicus GN=Tpi1 PE=1 SV=2	VNHALSEGLGVIACIGEK
>	sp|P48500|TPIS Triosephosphate isomerase OS=Rattus norvegicus GN=Tpi1 PE=1 SV=2	VNHALSEGLGVIACIGEK

>	sp|P48500|TPIS	Triosephosphate isomerase OS=Rattus norvegicus GN=Tpi1 PE=1 SV=2	VNHALSEGLGVIACIGEK
>	sp|P48500|TPIS	Triosephosphate isomerase OS=Rattus norvegicus GN=Tpi1 PE=1 SV=2	VNHALSEGLGVIACIGEK
>	sp|P48500|TPIS	Triosephosphate isomerase OS=Rattus norvegicus GN=Tpi1 PE=1 SV=2	VNHALSEGLGVIACIGEK
>	sp|P48500|TPIS	Triosephosphate isomerase OS=Rattus norvegicus GN=Tpi1 PE=1 SV=2	VNHALSEGLGVIACIGEK
>	sp|P48500|TPIS	Triosephosphate isomerase OS=Rattus norvegicus GN=Tpi1 PE=1 SV=2	VNHALSEGLGVIACIGEK
>	sp|P48500|TPIS	Triosephosphate isomerase OS=Rattus norvegicus GN=Tpi1 PE=1 SV=2	VNHALSEGLGVIACIGEK
>	sp|P48500|TPIS	Triosephosphate isomerase OS=Rattus norvegicus GN=Tpi1 PE=1 SV=2	VNHALSEGLGVIACIGEK
>	sp|P48500|TPIS	Triosephosphate isomerase OS=Rattus norvegicus GN=Tpi1 PE=1 SV=2	VNHALSEGLGVIACIGEK
>	sp|P48500|TPIS	Triosephosphate isomerase OS=Rattus norvegicus GN=Tpi1 PE=1 SV=2	VNHALSEGLGVIACIGEK
>	sp|P48500|TPIS	Triosephosphate isomerase OS=Rattus norvegicus GN=Tpi1 PE=1 SV=2	VNHALSEGLGVIACIGEK
>	sp|P48500|TPIS	Triosephosphate isomerase OS=Rattus norvegicus GN=Tpi1 PE=1 SV=2	VNHALSEGLGVIACIGEK
>	sp|P48500|TPIS	Triosephosphate isomerase OS=Rattus norvegicus GN=Tpi1 PE=1 SV=2	VNHALSEGLGVIACIGEK
>	sp|P48500|TPIS	Triosephosphate isomerase OS=Rattus norvegicus GN=Tpi1 PE=1 SV=2	VNHALSEGLGVIACIGEK
>	sp|P48500|TPIS	Triosephosphate isomerase OS=Rattus norvegicus GN=Tpi1 PE=1 SV=2	VNHALSEGLGVIACIGEK
>	sp|P48500|TPIS	Triosephosphate isomerase OS=Rattus norvegicus GN=Tpi1 PE=1 SV=2	VNHALSEGLGVIACIGEK
>	sp|P48500|TPIS	Triosephosphate isomerase OS=Rattus norvegicus GN=Tpi1 PE=1 SV=2	VNHALSEGLGVIACIGEK
>	sp|P48500|TPIS	Triosephosphate isomerase OS=Rattus norvegicus GN=Tpi1 PE=1 SV=2	VNHALSEGLGVIACIGEK
>	sp|P48500|TPIS	Triosephosphate isomerase OS=Rattus norvegicus GN=Tpi1 PE=1 SV=2	VNHALSEGLGVIACIGEK
>	sp|P48500|TPIS	Triosephosphate isomerase OS=Rattus norvegicus GN=Tpi1 PE=1 SV=2	VVLAYEPVWAIGTGK
>	sp|P48500|TPIS	Triosephosphate isomerase OS=Rattus norvegicus GN=Tpi1 PE=1 SV=2	VVLAYEPVWAIGTGK
>	sp|P48500|TPIS	Triosephosphate isomerase OS=Rattus norvegicus GN=Tpi1 PE=1 SV=2	VVLAYEPVWAIGTGK
>	sp|P48500|TPIS	Triosephosphate isomerase OS=Rattus norvegicus GN=Tpi1 PE=1 SV=2	VVLAYEPVWAIGTGK
>	sp|P48500|TPIS	Triosephosphate isomerase OS=Rattus norvegicus GN=Tpi1 PE=1 SV=2	VVLAYEPVWAIGTGK
>	sp|P48500|TPIS	Triosephosphate isomerase OS=Rattus norvegicus GN=Tpi1 PE=1 SV=2	VVLAYEPVWAIGTGK
>	sp|P48500|TPIS	Triosephosphate isomerase OS=Rattus norvegicus GN=Tpi1 PE=1 SV=2	VVLAYEPVWAIGTGK
>	sp|P48500|TPIS	Triosephosphate isomerase OS=Rattus norvegicus GN=Tpi1 PE=1 SV=2	VVLAYEPVWAIGTGK
>	sp|P48500|TPIS	Triosephosphate isomerase OS=Rattus norvegicus GN=Tpi1 PE=1 SV=2	VVLAYEPVWAIGTGK
>	sp|P48500|TPIS	Triosephosphate isomerase OS=Rattus norvegicus GN=Tpi1 PE=1 SV=2	VVLAYEPVWAIGTGK
>	sp|P48500|TPIS	Triosephosphate isomerase OS=Rattus norvegicus GN=Tpi1 PE=1 SV=2	TATPQQAQEVHEK
>	sp|P48500|TPIS	Triosephosphate isomerase OS=Rattus norvegicus GN=Tpi1 PE=1 SV=2	VVLAYEPVWAIGTGK
>	sp|P48500|TPIS	Triosephosphate isomerase OS=Rattus norvegicus GN=Tpi1 PE=1 SV=2	VNHALSEGLGVIACIGEK
>	sp|P48500|TPIS	Triosephosphate isomerase OS=Rattus norvegicus GN=Tpi1 PE=1 SV=2	ELASQPDVDGFLVGGASLKPEFVDIINAK
>	sp|P48500|TPIS	Triosephosphate isomerase OS=Rattus norvegicus GN=Tpi1 PE=1 SV=2	HIFGESDELIGQK
>	sp|P48500|TPIS	Triosephosphate isomerase OS=Rattus norvegicus GN=Tpi1 PE=1 SV=2	VTNGAFTGEISPGMIK
>	sp|P48500|TPIS	Triosephosphate isomerase OS=Rattus norvegicus GN=Tpi1 PE=1 SV=2	VTNGAFTGEISPGMIK
>	sp|P48500|TPIS	Triosephosphate isomerase OS=Rattus norvegicus GN=Tpi1 PE=1 SV=2	ELASQPDVDGFLVGGASLKPEFVDIINAK
>	sp|P48500|TPIS	Triosephosphate isomerase OS=Rattus norvegicus GN=Tpi1 PE=1 SV=2	IIYGGSVTGATCK
>	sp|P48500|TPIS	Triosephosphate isomerase OS=Rattus norvegicus GN=Tpi1 PE=1 SV=2	TATPQQAQEVHEK
>	sp|P48500|TPIS	Triosephosphate isomerase OS=Rattus norvegicus GN=Tpi1 PE=1 SV=2	ELASQPDVDGFLVGGASLKPEFVDIINAK
>	sp|P48500|TPIS	Triosephosphate isomerase OS=Rattus norvegicus GN=Tpi1 PE=1 SV=2	VVLAYEPVWAIGTGK
>	sp|P48500|TPIS	Triosephosphate isomerase OS=Rattus norvegicus GN=Tpi1 PE=1 SV=2	VNHALSEGLGVIACIGEK
>	sp|P48500|TPIS	Triosephosphate isomerase OS=Rattus norvegicus GN=Tpi1 PE=1 SV=2	FFVGGNWK
>	sp|P48500|TPIS	Triosephosphate isomerase OS=Rattus norvegicus GN=Tpi1 PE=1 SV=2	ELASQPDVDGFLVGGASLKPEFVDIINAK
>	sp|P48500|TPIS	Triosephosphate isomerase OS=Rattus norvegicus GN=Tpi1 PE=1 SV=2	ELASQPDVDGFLVGGASLKPEFVDIINAK
>	sp|P48500|TPIS	Triosephosphate isomerase OS=Rattus norvegicus GN=Tpi1 PE=1 SV=2	VVLAYEPVWAIGTGK
>	sp|P48500|TPIS	Triosephosphate isomerase OS=Rattus norvegicus GN=Tpi1 PE=1 SV=2	DLGATWVVLGHSER
>	sp|P48500|TPIS	Triosephosphate isomerase OS=Rattus norvegicus GN=Tpi1 PE=1 SV=2	VVLAYEPVWAIGTGK
>	sp|P48500|TPIS	Triosephosphate isomerase OS=Rattus norvegicus GN=Tpi1 PE=1 SV=2	ELASQPDVDGFLVGGASLKPEFVDIINAK
>	sp|P48500|TPIS	Triosephosphate isomerase OS=Rattus norvegicus GN=Tpi1 PE=1 SV=2	ELASQPDVDGFLVGGASLKPEFVDIINAK
>	sp|P48500|TPIS	Triosephosphate isomerase OS=Rattus norvegicus GN=Tpi1 PE=1 SV=2	VTNGAFTGEISPGMIK
>	sp|P48500|TPIS	Triosephosphate isomerase OS=Rattus norvegicus GN=Tpi1 PE=1 SV=2	DLGATWVVLGHSER
>	sp|P48500|TPIS	Triosephosphate isomerase OS=Rattus norvegicus GN=Tpi1 PE=1 SV=2	ELASQPDVDGFLVGGASLKPEFVDIINAK
>	sp|P48500|TPIS	Triosephosphate isomerase OS=Rattus norvegicus GN=Tpi1 PE=1 SV=2	VVLAYEPVWAIGTGK
>	sp|P48500|TPIS	Triosephosphate isomerase OS=Rattus norvegicus GN=Tpi1 PE=1 SV=2	DLGATWVVLGHSER
>	sp|P48500|TPIS	Triosephosphate isomerase OS=Rattus norvegicus GN=Tpi1 PE=1 SV=2	TATPQQAQEVHEK
>	sp|P48500|TPIS	Triosephosphate isomerase OS=Rattus norvegicus GN=Tpi1 PE=1 SV=2	TATPQQAQEVHEK
>	sp|P04764|ENO	Alpha-enolase OS=Rattus norvegicus GN=Eno1 PE=1 SV=4	FTATAGIQVVGDDLTVTNPK
>	sp|P04764|ENO	Alpha-enolase OS=Rattus norvegicus GN=Eno1 PE=1 SV=4	HIADLAGNPEVILPVPAFNVINGGSHAGNK
>	sp|P04764|ENO	Alpha-enolase OS=Rattus norvegicus GN=Eno1 PE=1 SV=4	KLNVVEQEK
>	sp|P04764|ENO	Alpha-enolase OS=Rattus norvegicus GN=Eno1 PE=1 SV=4	KLNVVEQEKIDQLMIEMDGTENK
>	sp|P04764|ENO	Alpha-enolase OS=Rattus norvegicus GN=Eno1 PE=1 SV=4	LAQSNGWGVMVSHR
>	sp|P04764|ENO	Alpha-enolase OS=Rattus norvegicus GN=Eno1 PE=1 SV=4	SFIKDYPVVSIEDPFDQDDWDAWQK
>	sp|P04764|ENO	Alpha-enolase OS=Rattus norvegicus GN=Eno1 PE=1 SV=4	VNQIGSVTESLQACK
>	sp|P04764|ENO	Alpha-enolase OS=Rattus norvegicus GN=Eno1 PE=1 SV=4	YITPDQLADLYK
>	sp|P04764|ENO	Alpha-enolase OS=Rattus norvegicus GN=Eno1 PE=1 SV=4	LAMQEFMILPVGASSFR
>	sp|P04764|ENO	Alpha-enolase OS=Rattus norvegicus GN=Eno1 PE=1 SV=4	IDQLMIEMDGTENK
>	sp|P04764|ENO	Alpha-enolase OS=Rattus norvegicus GN=Eno1 PE=1 SV=4	TIAPALVSK
>	sp|P04764|ENO	Alpha-enolase OS=Rattus norvegicus GN=Eno1 PE=1 SV=4	AAVPSGASTGIYEALELR
>	sp|P04764|ENO	Alpha-enolase OS=Rattus norvegicus GN=Eno1 PE=1 SV=4	AAVPSGASTGIYEALELR
>	sp|P04764|ENO	Alpha-enolase OS=Rattus norvegicus GN=Eno1 PE=1 SV=4	AAVPSGASTGIYEALELR
>	sp|P04764|ENO	Alpha-enolase OS=Rattus norvegicus GN=Eno1 PE=1 SV=4	AAVPSGASTGIYEALELR
>	sp|P04764|ENO	Alpha-enolase OS=Rattus norvegicus GN=Eno1 PE=1 SV=4	AAVPSGASTGIYEALELR
>	sp|P04764|ENO	Alpha-enolase OS=Rattus norvegicus GN=Eno1 PE=1 SV=4	AAVPSGASTGIYEALELR
>	sp|P04764|ENO	Alpha-enolase OS=Rattus norvegicus GN=Eno1 PE=1 SV=4	AAVPSGASTGIYEALELR

<table>
<tr><td>></td><td>sp|P04764|EN(Alpha-enolase OS=Rattus norvegicus GN=Eno1 PE=1 SV=4</td><td>AAVPSGASTGIYEALELR</td></tr>
<tr><td>></td><td>sp|P04764|EN(Alpha-enolase OS=Rattus norvegicus GN=Eno1 PE=1 SV=4</td><td>FGANAILGVSLAVCK</td></tr>
<tr><td>></td><td>sp|P04764|EN(Alpha-enolase OS=Rattus norvegicus GN=Eno1 PE=1 SV=4</td><td>FTATAGIQVVGDDLTVTNPK</td></tr>
<tr><td>></td><td>sp|P04764|EN(Alpha-enolase OS=Rattus norvegicus GN=Eno1 PE=1 SV=4</td><td>FTATAGIQVVGDDLTVTNPK</td></tr>
<tr><td>></td><td>sp|P04764|EN(Alpha-enolase OS=Rattus norvegicus GN=Eno1 PE=1 SV=4</td><td>FTATAGIQVVGDDLTVTNPK</td></tr>
<tr><td>></td><td>sp|P04764|EN(Alpha-enolase OS=Rattus norvegicus GN=Eno1 PE=1 SV=4</td><td>HIADLAGNPEVILPVPAFNVINGGSHAGNK</td></tr>
<tr><td>></td><td>sp|P04764|EN(Alpha-enolase OS=Rattus norvegicus GN=Eno1 PE=1 SV=4</td><td>HIADLAGNPEVILPVPAFNVINGGSHAGNK</td></tr>
<tr><td>></td><td>sp|P04764|EN(Alpha-enolase OS=Rattus norvegicus GN=Eno1 PE=1 SV=4</td><td>HIADLAGNPEVILPVPAFNVINGGSHAGNK</td></tr>
<tr><td>></td><td>sp|P04764|EN(Alpha-enolase OS=Rattus norvegicus GN=Eno1 PE=1 SV=4</td><td>HIADLAGNPEVILPVPAFNVINGGSHAGNK</td></tr>
<tr><td>></td><td>sp|P04764|EN(Alpha-enolase OS=Rattus norvegicus GN=Eno1 PE=1 SV=4</td><td>HIADLAGNPEVILPVPAFNVINGGSHAGNK</td></tr>
<tr><td>></td><td>sp|P04764|EN(Alpha-enolase OS=Rattus norvegicus GN=Eno1 PE=1 SV=4</td><td>HIADLAGNPEVILPVPAFNVINGGSHAGNK</td></tr>
<tr><td>></td><td>sp|P04764|EN(Alpha-enolase OS=Rattus norvegicus GN=Eno1 PE=1 SV=4</td><td>HIADLAGNPEVILPVPAFNVINGGSHAGNK</td></tr>
<tr><td>></td><td>sp|P04764|EN(Alpha-enolase OS=Rattus norvegicus GN=Eno1 PE=1 SV=4</td><td>HIADLAGNPEVILPVPAFNVINGGSHAGNK</td></tr>
<tr><td>></td><td>sp|P04764|EN(Alpha-enolase OS=Rattus norvegicus GN=Eno1 PE=1 SV=4</td><td>HIADLAGNPEVILPVPAFNVINGGSHAGNK</td></tr>
<tr><td>></td><td>sp|P04764|EN(Alpha-enolase OS=Rattus norvegicus GN=Eno1 PE=1 SV=4</td><td>HIADLAGNPEVILPVPAFNVINGGSHAGNK</td></tr>
<tr><td>></td><td>sp|P04764|EN(Alpha-enolase OS=Rattus norvegicus GN=Eno1 PE=1 SV=4</td><td>HIADLAGNPEVILPVPAFNVINGGSHAGNK</td></tr>
<tr><td>></td><td>sp|P04764|EN(Alpha-enolase OS=Rattus norvegicus GN=Eno1 PE=1 SV=4</td><td>HIADLAGNPEVILPVPAFNVINGGSHAGNK</td></tr>
<tr><td>></td><td>sp|P04764|EN(Alpha-enolase OS=Rattus norvegicus GN=Eno1 PE=1 SV=4</td><td>HIADLAGNPEVILPVPAFNVINGGSHAGNK</td></tr>
<tr><td>></td><td>sp|P04764|EN(Alpha-enolase OS=Rattus norvegicus GN=Eno1 PE=1 SV=4</td><td>HIADLAGNPEVILPVPAFNVINGGSHAGNK</td></tr>
<tr><td>></td><td>sp|P04764|EN(Alpha-enolase OS=Rattus norvegicus GN=Eno1 PE=1 SV=4</td><td>HIADLAGNPEVILPVPAFNVINGGSHAGNK</td></tr>
<tr><td>></td><td>sp|P04764|EN(Alpha-enolase OS=Rattus norvegicus GN=Eno1 PE=1 SV=4</td><td>HIADLAGNPEVILPVPAFNVINGGSHAGNK</td></tr>
<tr><td>></td><td>sp|P04764|EN(Alpha-enolase OS=Rattus norvegicus GN=Eno1 PE=1 SV=4</td><td>HIADLAGNPEVILPVPAFNVINGGSHAGNK</td></tr>
<tr><td>></td><td>sp|P04764|EN(Alpha-enolase OS=Rattus norvegicus GN=Eno1 PE=1 SV=4</td><td>HIADLAGNPEVILPVPAFNVINGGSHAGNK</td></tr>
<tr><td>></td><td>sp|P04764|EN(Alpha-enolase OS=Rattus norvegicus GN=Eno1 PE=1 SV=4</td><td>HIADLAGNPEVILPVPAFNVINGGSHAGNK</td></tr>
<tr><td>></td><td>sp|P04764|EN(Alpha-enolase OS=Rattus norvegicus GN=Eno1 PE=1 SV=4</td><td>HIADLAGNPEVILPVPAFNVINGGSHAGNK</td></tr>
<tr><td>></td><td>sp|P04764|EN(Alpha-enolase OS=Rattus norvegicus GN=Eno1 PE=1 SV=4</td><td>HIADLAGNPEVILPVPAFNVINGGSHAGNK</td></tr>
<tr><td>></td><td>sp|P04764|EN(Alpha-enolase OS=Rattus norvegicus GN=Eno1 PE=1 SV=4</td><td>HIADLAGNPEVILPVPAFNVINGGSHAGNK</td></tr>
<tr><td>></td><td>sp|P04764|EN(Alpha-enolase OS=Rattus norvegicus GN=Eno1 PE=1 SV=4</td><td>HIADLAGNPEVILPVPAFNVINGGSHAGNK</td></tr>
<tr><td>></td><td>sp|P04764|EN(Alpha-enolase OS=Rattus norvegicus GN=Eno1 PE=1 SV=4</td><td>HIADLAGNPEVILPVPAFNVINGGSHAGNK</td></tr>
<tr><td>></td><td>sp|P04764|EN(Alpha-enolase OS=Rattus norvegicus GN=Eno1 PE=1 SV=4</td><td>HIADLAGNPEVILPVPAFNVINGGSHAGNK</td></tr>
<tr><td>></td><td>sp|P04764|EN(Alpha-enolase OS=Rattus norvegicus GN=Eno1 PE=1 SV=4</td><td>HIADLAGNPEVILPVPAFNVINGGSHAGNK</td></tr>
<tr><td>></td><td>sp|P04764|EN(Alpha-enolase OS=Rattus norvegicus GN=Eno1 PE=1 SV=4</td><td>HIADLAGNPEVILPVPAFNVINGGSHAGNK</td></tr>
<tr><td>></td><td>sp|P04764|EN(Alpha-enolase OS=Rattus norvegicus GN=Eno1 PE=1 SV=4</td><td>HIADLAGNPEVILPVPAFNVINGGSHAGNK</td></tr>
<tr><td>></td><td>sp|P04764|EN(Alpha-enolase OS=Rattus norvegicus GN=Eno1 PE=1 SV=4</td><td>HIADLAGNPEVILPVPAFNVINGGSHAGNK</td></tr>
<tr><td>></td><td>sp|P04764|EN(Alpha-enolase OS=Rattus norvegicus GN=Eno1 PE=1 SV=4</td><td>HIADLAGNPEVILPVPAFNVINGGSHAGNK</td></tr>
<tr><td>></td><td>sp|P04764|EN(Alpha-enolase OS=Rattus norvegicus GN=Eno1 PE=1 SV=4</td><td>HIADLAGNPEVILPVPAFNVINGGSHAGNK</td></tr>
<tr><td>></td><td>sp|P04764|EN(Alpha-enolase OS=Rattus norvegicus GN=Eno1 PE=1 SV=4</td><td>HIADLAGNPEVILPVPAFNVINGGSHAGNK</td></tr>
<tr><td>></td><td>sp|P04764|EN(Alpha-enolase OS=Rattus norvegicus GN=Eno1 PE=1 SV=4</td><td>HIADLAGNPEVILPVPAFNVINGGSHAGNK</td></tr>
<tr><td>></td><td>sp|P04764|EN(Alpha-enolase OS=Rattus norvegicus GN=Eno1 PE=1 SV=4</td><td>HIADLAGNPEVILPVPAFNVINGGSHAGNK</td></tr>
<tr><td>></td><td>sp|P04764|EN(Alpha-enolase OS=Rattus norvegicus GN=Eno1 PE=1 SV=4</td><td>HIADLAGNPEVILPVPAFNVINGGSHAGNK</td></tr>
<tr><td>></td><td>sp|P04764|EN(Alpha-enolase OS=Rattus norvegicus GN=Eno1 PE=1 SV=4</td><td>HIADLAGNPEVILPVPAFNVINGGSHAGNK</td></tr>
<tr><td>></td><td>sp|P04764|EN(Alpha-enolase OS=Rattus norvegicus GN=Eno1 PE=1 SV=4</td><td>HIADLAGNPEVILPVPAFNVINGGSHAGNK</td></tr>
<tr><td>></td><td>sp|P04764|EN(Alpha-enolase OS=Rattus norvegicus GN=Eno1 PE=1 SV=4</td><td>HIADLAGNPEVILPVPAFNVINGGSHAGNK</td></tr>
<tr><td>></td><td>sp|P04764|EN(Alpha-enolase OS=Rattus norvegicus GN=Eno1 PE=1 SV=4</td><td>HIADLAGNPEVILPVPAFNVINGGSHAGNK</td></tr>
<tr><td>></td><td>sp|P04764|EN(Alpha-enolase OS=Rattus norvegicus GN=Eno1 PE=1 SV=4</td><td>HIADLAGNPEVILPVPAFNVINGGSHAGNK</td></tr>
<tr><td>></td><td>sp|P04764|EN(Alpha-enolase OS=Rattus norvegicus GN=Eno1 PE=1 SV=4</td><td>HIADLAGNPEVILPVPAFNVINGGSHAGNK</td></tr>
<tr><td>></td><td>sp|P04764|EN(Alpha-enolase OS=Rattus norvegicus GN=Eno1 PE=1 SV=4</td><td>HIADLAGNPEVILPVPAFNVINGGSHAGNK</td></tr>
<tr><td>></td><td>sp|P04764|EN(Alpha-enolase OS=Rattus norvegicus GN=Eno1 PE=1 SV=4</td><td>HIADLAGNPEVILPVPAFNVINGGSHAGNK</td></tr>
<tr><td>></td><td>sp|P04764|EN(Alpha-enolase OS=Rattus norvegicus GN=Eno1 PE=1 SV=4</td><td>HIADLAGNPEVILPVPAFNVINGGSHAGNK</td></tr>
<tr><td>></td><td>sp|P04764|EN(Alpha-enolase OS=Rattus norvegicus GN=Eno1 PE=1 SV=4</td><td>HIADLAGNPEVILPVPAFNVINGGSHAGNK</td></tr>
<tr><td>></td><td>sp|P04764|EN(Alpha-enolase OS=Rattus norvegicus GN=Eno1 PE=1 SV=4</td><td>HIADLAGNPEVILPVPAFNVINGGSHAGNK</td></tr>
<tr><td>></td><td>sp|P04764|EN(Alpha-enolase OS=Rattus norvegicus GN=Eno1 PE=1 SV=4</td><td>HIADLAGNPEVILPVPAFNVINGGSHAGNK</td></tr>
<tr><td>></td><td>sp|P04764|EN(Alpha-enolase OS=Rattus norvegicus GN=Eno1 PE=1 SV=4</td><td>HIADLAGNPEVILPVPAFNVINGGSHAGNK</td></tr>
<tr><td>></td><td>sp|P04764|EN(Alpha-enolase OS=Rattus norvegicus GN=Eno1 PE=1 SV=4</td><td>HIADLAGNPEVILPVPAFNVINGGSHAGNK</td></tr>
<tr><td>></td><td>sp|P04764|EN(Alpha-enolase OS=Rattus norvegicus GN=Eno1 PE=1 SV=4</td><td>HIADLAGNPEVILPVPAFNVINGGSHAGNK</td></tr>
<tr><td>></td><td>sp|P04764|EN(Alpha-enolase OS=Rattus norvegicus GN=Eno1 PE=1 SV=4</td><td>HIADLAGNPEVILPVPAFNVINGGSHAGNK</td></tr>
<tr><td>></td><td>sp|P04764|EN(Alpha-enolase OS=Rattus norvegicus GN=Eno1 PE=1 SV=4</td><td>HIADLAGNPEVILPVPAFNVINGGSHAGNK</td></tr>
<tr><td>></td><td>sp|P04764|EN(Alpha-enolase OS=Rattus norvegicus GN=Eno1 PE=1 SV=4</td><td>HIADLAGNPEVILPVPAFNVINGGSHAGNK</td></tr>
<tr><td>></td><td>sp|P04764|EN(Alpha-enolase OS=Rattus norvegicus GN=Eno1 PE=1 SV=4</td><td>KLNVVEQEK</td></tr>
<tr><td>></td><td>sp|P04764|EN(Alpha-enolase OS=Rattus norvegicus GN=Eno1 PE=1 SV=4</td><td>KLNVVEQEKIDQLMIEMDGTENK</td></tr>
<tr><td>></td><td>sp|P04764|EN(Alpha-enolase OS=Rattus norvegicus GN=Eno1 PE=1 SV=4</td><td>KLNVVEQEKIDQLMIEMDGTENK</td></tr>
<tr><td>></td><td>sp|P04764|EN(Alpha-enolase OS=Rattus norvegicus GN=Eno1 PE=1 SV=4</td><td>LAQSNGWGVMVSHR</td></tr>
<tr><td>></td><td>sp|P04764|EN(Alpha-enolase OS=Rattus norvegicus GN=Eno1 PE=1 SV=4</td><td>PVPAFNVINGGSHAGNK</td></tr>
<tr><td>></td><td>sp|P04764|EN(Alpha-enolase OS=Rattus norvegicus GN=Eno1 PE=1 SV=4</td><td>SFIKDYPVVSIEDPFDQDDWDAWQK</td></tr>
<tr><td>></td><td>sp|P04764|EN(Alpha-enolase OS=Rattus norvegicus GN=Eno1 PE=1 SV=4</td><td>SFIKDYPVVSIEDPFDQDDWDAWQK</td></tr>
<tr><td>></td><td>sp|P04764|EN(Alpha-enolase OS=Rattus norvegicus GN=Eno1 PE=1 SV=4</td><td>SFIKDYPVVSIEDPFDQDDWDAWQK</td></tr>
<tr><td>></td><td>sp|P04764|EN(Alpha-enolase OS=Rattus norvegicus GN=Eno1 PE=1 SV=4</td><td>SFIKDYPVVSIEDPFDQDDWDAWQK</td></tr>
<tr><td>></td><td>sp|P04764|EN(Alpha-enolase OS=Rattus norvegicus GN=Eno1 PE=1 SV=4</td><td>SGETEDTFIADLVVGLCTGQIK</td></tr>
<tr><td>></td><td>sp|P04764|EN(Alpha-enolase OS=Rattus norvegicus GN=Eno1 PE=1 SV=4</td><td>SGETEDTFIADLVVGLCTGQIK</td></tr>
<tr><td>></td><td>sp|P04764|EN(Alpha-enolase OS=Rattus norvegicus GN=Eno1 PE=1 SV=4</td><td>SGETEDTFIADLVVGLCTGQIK</td></tr>
<tr><td>></td><td>sp|P04764|EN(Alpha-enolase OS=Rattus norvegicus GN=Eno1 PE=1 SV=4</td><td>SGETEDTFIADLVVGLCTGQIK</td></tr>
<tr><td>></td><td>sp|P04764|EN(Alpha-enolase OS=Rattus norvegicus GN=Eno1 PE=1 SV=4</td><td>SGETEDTFIADLVVGLCTGQIK</td></tr>
<tr><td>></td><td>sp|P04764|EN(Alpha-enolase OS=Rattus norvegicus GN=Eno1 PE=1 SV=4</td><td>SGETEDTFIADLVVGLCTGQIK</td></tr>
<tr><td>></td><td>sp|P04764|EN(Alpha-enolase OS=Rattus norvegicus GN=Eno1 PE=1 SV=4</td><td>VNQIGSVTESLQACK</td></tr>
</table>

>	sp|P04764|EN(Alpha-enolase OS=Rattus norvegicus GN=Eno1 PE=1 SV=4	FGANAILGVSLAVCK
>	sp|P04764|EN(Alpha-enolase OS=Rattus norvegicus GN=Eno1 PE=1 SV=4	HIADLAGNPEVILPVPAFNVINGGSHAGNK
>	sp|P04764|EN(Alpha-enolase OS=Rattus norvegicus GN=Eno1 PE=1 SV=4	YITPDQLADLYK
>	sp|P04764|EN(Alpha-enolase OS=Rattus norvegicus GN=Eno1 PE=1 SV=4	IEEELGSK
>	sp|P04764|EN(Alpha-enolase OS=Rattus norvegicus GN=Eno1 PE=1 SV=4	AVEHINK
>	sp|P04764|EN(Alpha-enolase OS=Rattus norvegicus GN=Eno1 PE=1 SV=4	HIADLAGNPEVILPVPAFNVINGGSHAGNK
>	sp|P04764|EN(Alpha-enolase OS=Rattus norvegicus GN=Eno1 PE=1 SV=4	LAQSNGWGVMVSHR
>	sp|P04764|EN(Alpha-enolase OS=Rattus norvegicus GN=Eno1 PE=1 SV=4	HIADLAGNPEVILPVPAFNVINGGSHAGNK
>	sp|P04764|EN(Alpha-enolase OS=Rattus norvegicus GN=Eno1 PE=1 SV=4	FGANAILGVSLAVCK
>	sp|P04764|EN(Alpha-enolase OS=Rattus norvegicus GN=Eno1 PE=1 SV=4	SFIKDYPVVSIEDPFDQDDWDAWQK
>	sp|P04764|EN(Alpha-enolase OS=Rattus norvegicus GN=Eno1 PE=1 SV=4	HIADLAGNPEVILPVPAFNVINGGSHAGNK
>	sp|P04764|EN(Alpha-enolase OS=Rattus norvegicus GN=Eno1 PE=1 SV=4	AVEHINK
>	sp|P04764|EN(Alpha-enolase OS=Rattus norvegicus GN=Eno1 PE=1 SV=4	FGANAILGVSLAVCK
>	sp|P04764|EN(Alpha-enolase OS=Rattus norvegicus GN=Eno1 PE=1 SV=4	FRAAVPSGASTGIYEALELR
>	sp|P04764|EN(Alpha-enolase OS=Rattus norvegicus GN=Eno1 PE=1 SV=4	SFIKDYPVVSIEDPFDQDDWDAWQK
>	sp|P04764|EN(Alpha-enolase OS=Rattus norvegicus GN=Eno1 PE=1 SV=4	AAVPSGASTGIYEALELR
>	sp|P04764|EN(Alpha-enolase OS=Rattus norvegicus GN=Eno1 PE=1 SV=4	HIADLAGNPEVILPVPAFNVINGGSHAGNK
>	sp|P04764|EN(Alpha-enolase OS=Rattus norvegicus GN=Eno1 PE=1 SV=4	HIADLAGNPEVILPVPAFNVINGGSHAGNK
>	sp|P04764|EN(Alpha-enolase OS=Rattus norvegicus GN=Eno1 PE=1 SV=4	IEEELGSK
>	sp|P04764|EN(Alpha-enolase OS=Rattus norvegicus GN=Eno1 PE=1 SV=4	AVEHINK
>	sp|P04764|EN(Alpha-enolase OS=Rattus norvegicus GN=Eno1 PE=1 SV=4	GNPTVEVDLYTAK
>	sp|P04764|EN(Alpha-enolase OS=Rattus norvegicus GN=Eno1 PE=1 SV=4	HIADLAGNPEVILPVPAFNVINGGSHAGNK
>	sp|P04764|EN(Alpha-enolase OS=Rattus norvegicus GN=Eno1 PE=1 SV=4	HIADLAGNPEVILPVPAFNVINGGSHAGNK
>	sp|P04764|EN(Alpha-enolase OS=Rattus norvegicus GN=Eno1 PE=1 SV=4	IEEELGSK
>	sp|P04764|EN(Alpha-enolase OS=Rattus norvegicus GN=Eno1 PE=1 SV=4	FTATAGIQVVGDDLTVTNPK
>	sp|P04764|EN(Alpha-enolase OS=Rattus norvegicus GN=Eno1 PE=1 SV=4	PSGASTGIYEALELR
>	sp|P04764|EN(Alpha-enolase OS=Rattus norvegicus GN=Eno1 PE=1 SV=4	KLNVVEQEKIDQLMIEMDGTENK
>	sp|P04764|EN(Alpha-enolase OS=Rattus norvegicus GN=Eno1 PE=1 SV=4	LAQSNGWGVMVSHR
>	sp|P04764|EN(Alpha-enolase OS=Rattus norvegicus GN=Eno1 PE=1 SV=4	TIAPALVSK
>	sp|P04764|EN(Alpha-enolase OS=Rattus norvegicus GN=Eno1 PE=1 SV=4	SFIKDYPVVSIEDPFDQDDWDAWQK
>	sp|P04764|EN(Alpha-enolase OS=Rattus norvegicus GN=Eno1 PE=1 SV=4	IEEELGSK
>	sp|P04764|EN(Alpha-enolase OS=Rattus norvegicus GN=Eno1 PE=1 SV=4	HIADLAGNPEVILPVPAFNVINGGSHAGNKLAMQE
>	sp|P04764|EN(Alpha-enolase OS=Rattus norvegicus GN=Eno1 PE=1 SV=4	HIADLAGNPEVILPVPAFNVINGGSHAGNK
>	sp|P04764|EN(Alpha-enolase OS=Rattus norvegicus GN=Eno1 PE=1 SV=4	KLNVVEQEKIDQLMIEMDGTENK
>	sp|P04764|EN(Alpha-enolase OS=Rattus norvegicus GN=Eno1 PE=1 SV=4	LAQSNGWGVMVSHR
>	sp|P04764|EN(Alpha-enolase OS=Rattus norvegicus GN=Eno1 PE=1 SV=4	LAQSNGWGVMVSHR
>	sp|P10860|DHI Glutamate dehydrogenase 1, mitochondrial OS=Rattus norvegicus GN=Glud1 PE=1 SV=2	ALASLMTYK
>	sp|P10860|DHI Glutamate dehydrogenase 1, mitochondrial OS=Rattus norvegicus GN=Glud1 PE=1 SV=2	DIVHSGLAYTMER
>	sp|P10860|DHI Glutamate dehydrogenase 1, mitochondrial OS=Rattus norvegicus GN=Glud1 PE=1 SV=2	IIAEGANGPTTPEADKIFLER
>	sp|P10860|DHI Glutamate dehydrogenase 1, mitochondrial OS=Rattus norvegicus GN=Glud1 PE=1 SV=2	KGFIGPGIDVPAPDMSTGER
>	sp|P10860|DHI Glutamate dehydrogenase 1, mitochondrial OS=Rattus norvegicus GN=Glud1 PE=1 SV=2	TFVVQGFGNVGLHSMR
>	sp|P10860|DHI Glutamate dehydrogenase 1, mitochondrial OS=Rattus norvegicus GN=Glud1 PE=1 SV=2	TRENEEQKR
>	sp|P10860|DHI Glutamate dehydrogenase 1, mitochondrial OS=Rattus norvegicus GN=Glud1 PE=1 SV=2	VYEGSILEADCDILIPAASEK
>	sp|P10860|DHI Glutamate dehydrogenase 1, mitochondrial OS=Rattus norvegicus GN=Glud1 PE=1 SV=2	HGGTIPVVPTAEFQDR
>	sp|P10860|DHI Glutamate dehydrogenase 1, mitochondrial OS=Rattus norvegicus GN=Glud1 PE=1 SV=2	DSNYHLLMSVQESLER
>	sp|P10860|DHI Glutamate dehydrogenase 1, mitochondrial OS=Rattus norvegicus GN=Glud1 PE=1 SV=2	YNLGLDLR
>	sp|P10860|DHI Glutamate dehydrogenase 1, mitochondrial OS=Rattus norvegicus GN=Glud1 PE=1 SV=2	DDGSWEVIEGYR
>	sp|P10860|DHI Glutamate dehydrogenase 1, mitochondrial OS=Rattus norvegicus GN=Glud1 PE=1 SV=2	DSNYHLLMSVQESLER
>	sp|P10860|DHI Glutamate dehydrogenase 1, mitochondrial OS=Rattus norvegicus GN=Glud1 PE=1 SV=2	DSNYHLLMSVQESLER
>	sp|P10860|DHI Glutamate dehydrogenase 1, mitochondrial OS=Rattus norvegicus GN=Glud1 PE=1 SV=2	HGGTIPVVPTAEFQDR
>	sp|P10860|DHI Glutamate dehydrogenase 1, mitochondrial OS=Rattus norvegicus GN=Glud1 PE=1 SV=2	HGGTIPVVPTAEFQDR
>	sp|P10860|DHI Glutamate dehydrogenase 1, mitochondrial OS=Rattus norvegicus GN=Glud1 PE=1 SV=2	HGGTIPVVPTAEFQDR
>	sp|P10860|DHI Glutamate dehydrogenase 1, mitochondrial OS=Rattus norvegicus GN=Glud1 PE=1 SV=2	IIAEGANGPTTPEADKIFLER
>	sp|P10860|DHI Glutamate dehydrogenase 1, mitochondrial OS=Rattus norvegicus GN=Glud1 PE=1 SV=2	KGFIGPGIDVPAPDMSTGER
>	sp|P10860|DHI Glutamate dehydrogenase 1, mitochondrial OS=Rattus norvegicus GN=Glud1 PE=1 SV=2	VYEGSILEADCDILIPAASEK
>	sp|P10860|DHI Glutamate dehydrogenase 1, mitochondrial OS=Rattus norvegicus GN=Glud1 PE=1 SV=2	VYEGSILEADCDILIPAASEK
>	sp|P10860|DHI Glutamate dehydrogenase 1, mitochondrial OS=Rattus norvegicus GN=Glud1 PE=1 SV=2	GASIVEDKLVEDLK
>	sp|P10860|DHI Glutamate dehydrogenase 1, mitochondrial OS=Rattus norvegicus GN=Glud1 PE=1 SV=2	IIAEGANGPTTPEADKIFLER
>	sp|P10860|DHI Glutamate dehydrogenase 1, mitochondrial OS=Rattus norvegicus GN=Glud1 PE=1 SV=2	VYEGSILEADCDILIPAASEK
>	sp|P10860|DHI Glutamate dehydrogenase 1, mitochondrial OS=Rattus norvegicus GN=Glud1 PE=1 SV=2	YNLGLDLR
>	sp|P10860|DHI Glutamate dehydrogenase 1, mitochondrial OS=Rattus norvegicus GN=Glud1 PE=1 SV=2	DIVHSGLAYTMER
>	sp|P10860|DHI Glutamate dehydrogenase 1, mitochondrial OS=Rattus norvegicus GN=Glud1 PE=1 SV=2	IIAEGANGPTTPEADKIFLER
>	sp|P10860|DHI Glutamate dehydrogenase 1, mitochondrial OS=Rattus norvegicus GN=Glud1 PE=1 SV=2	CAVVDVPFGGAK
>	sp|P10860|DHI Glutamate dehydrogenase 1, mitochondrial OS=Rattus norvegicus GN=Glud1 PE=1 SV=2	DIVHSGLAYTMER
>	sp|P10860|DHI Glutamate dehydrogenase 1, mitochondrial OS=Rattus norvegicus GN=Glud1 PE=1 SV=2	DIVHSGLAYTMER
>	sp|P10860|DHI Glutamate dehydrogenase 1, mitochondrial OS=Rattus norvegicus GN=Glud1 PE=1 SV=2	GASIVEDKLVEDLK
>	sp|P10860|DHI Glutamate dehydrogenase 1, mitochondrial OS=Rattus norvegicus GN=Glud1 PE=1 SV=2	DSNYHLLMSVQESLER
>	sp|P10860|DHI Glutamate dehydrogenase 1, mitochondrial OS=Rattus norvegicus GN=Glud1 PE=1 SV=2	IIKPCNHVLSLSFPIR
>	sp|P10860|DHI Glutamate dehydrogenase 1, mitochondrial OS=Rattus norvegicus GN=Glud1 PE=1 SV=2	IIKPCNHVLSLSFPIRR
>	sp|P10860|DHI Glutamate dehydrogenase 1, mitochondrial OS=Rattus norvegicus GN=Glud1 PE=1 SV=2	HGGTIPVVPTAEFQDR
>	sp|Q6P6V0|G6 Glucose-6-phosphate isomerase OS=Rattus norvegicus GN=Gpi PE=1 SV=1	EVLHMLVDLAK
>	sp|Q6P6V0|G6 Glucose-6-phosphate isomerase OS=Rattus norvegicus GN=Gpi PE=1 SV=1	ILLANFLAQTEALMK
>	sp|Q6P6V0|G6 Glucose-6-phosphate isomerase OS=Rattus norvegicus GN=Gpi PE=1 SV=1	MIPCDFLIPVQTQHPIR
>	sp|Q6P6V0|G6 Glucose-6-phosphate isomerase OS=Rattus norvegicus GN=Gpi PE=1 SV=1	TFTTQETITNAETAK

>	sp|Q6P6V0|G6 Glucose-6-phosphate isomerase OS=Rattus norvegicus GN=Gpi PE=1 SV=1	TFTTQETITNAETAKEWFLQAAK
>	sp|Q6P6V0|G6 Glucose-6-phosphate isomerase OS=Rattus norvegicus GN=Gpi PE=1 SV=1	TLANLNPESSLFIIASK
>	sp|Q6P6V0|G6 Glucose-6-phosphate isomerase OS=Rattus norvegicus GN=Gpi PE=1 SV=1	VDYQTGPIVWGEPGTNGQHAFYQLIHQGTK
>	sp|Q6P6V0|G6 Glucose-6-phosphate isomerase OS=Rattus norvegicus GN=Gpi PE=1 SV=1	KELQAAGK
>	sp|Q6P6V0|G6 Glucose-6-phosphate isomerase OS=Rattus norvegicus GN=Gpi PE=1 SV=1	AITDIINIGIGGSDLGPLMVTEALKPYSK
>	sp|Q6P6V0|G6 Glucose-6-phosphate isomerase OS=Rattus norvegicus GN=Gpi PE=1 SV=1	NLVNKEVLHMLVDLAK
>	sp|Q6P6V0|G6 Glucose-6-phosphate isomerase OS=Rattus norvegicus GN=Gpi PE=1 SV=1	TFTTQETITNAETAKEWFLQAAKDPSAVAK
>	sp|Q6P6V0|G6 Glucose-6-phosphate isomerase OS=Rattus norvegicus GN=Gpi PE=1 SV=1	AITDIINIGIGGSDLGPLMVTEALKPYSK
>	sp|Q6P6V0|G6 Glucose-6-phosphate isomerase OS=Rattus norvegicus GN=Gpi PE=1 SV=1	AITDIINIGIGGSDLGPLMVTEALKPYSK
>	sp|Q6P6V0|G6 Glucose-6-phosphate isomerase OS=Rattus norvegicus GN=Gpi PE=1 SV=1	EVLHMLVDLAK
>	sp|Q6P6V0|G6 Glucose-6-phosphate isomerase OS=Rattus norvegicus GN=Gpi PE=1 SV=1	EVLHMLVDLAK
>	sp|Q6P6V0|G6 Glucose-6-phosphate isomerase OS=Rattus norvegicus GN=Gpi PE=1 SV=1	EVLHMLVDLAK
>	sp|Q6P6V0|G6 Glucose-6-phosphate isomerase OS=Rattus norvegicus GN=Gpi PE=1 SV=1	EVLHMLVDLAK
>	sp|Q6P6V0|G6 Glucose-6-phosphate isomerase OS=Rattus norvegicus GN=Gpi PE=1 SV=1	EVLHMLVDLAK
>	sp|Q6P6V0|G6 Glucose-6-phosphate isomerase OS=Rattus norvegicus GN=Gpi PE=1 SV=1	ILLANFLAQTEALMK
>	sp|Q6P6V0|G6 Glucose-6-phosphate isomerase OS=Rattus norvegicus GN=Gpi PE=1 SV=1	ILLANFLAQTEALMK
>	sp|Q6P6V0|G6 Glucose-6-phosphate isomerase OS=Rattus norvegicus GN=Gpi PE=1 SV=1	MIPCDFLIPVQTQHPIR
>	sp|Q6P6V0|G6 Glucose-6-phosphate isomerase OS=Rattus norvegicus GN=Gpi PE=1 SV=1	TLANLNPESSLFIIASK
>	sp|Q6P6V0|G6 Glucose-6-phosphate isomerase OS=Rattus norvegicus GN=Gpi PE=1 SV=1	VDYQTGPIVWGEPGTNGQHAFYQLIHQGTK
>	sp|Q6P6V0|G6 Glucose-6-phosphate isomerase OS=Rattus norvegicus GN=Gpi PE=1 SV=1	AVLHVALR
>	sp|Q6P6V0|G6 Glucose-6-phosphate isomerase OS=Rattus norvegicus GN=Gpi PE=1 SV=1	MIPCDFLIPVQTQHPIR
>	sp|Q6P6V0|G6 Glucose-6-phosphate isomerase OS=Rattus norvegicus GN=Gpi PE=1 SV=1	HFVALSTNTDKVK
>	sp|Q6P6V0|G6 Glucose-6-phosphate isomerase OS=Rattus norvegicus GN=Gpi PE=1 SV=1	AITDIINIGIGGSDLGPLMVTEALKPYSK
>	sp|Q6P6V0|G6 Glucose-6-phosphate isomerase OS=Rattus norvegicus GN=Gpi PE=1 SV=1	MIPCDFLIPVQTQHPIR
>	sp|Q6P6V0|G6 Glucose-6-phosphate isomerase OS=Rattus norvegicus GN=Gpi PE=1 SV=1	MIPCDFLIPVQTQHPIR
>	sp|Q6P6V0|G6 Glucose-6-phosphate isomerase OS=Rattus norvegicus GN=Gpi PE=1 SV=1	AVLHVALR
>	sp|Q6P6V0|G6 Glucose-6-phosphate isomerase OS=Rattus norvegicus GN=Gpi PE=1 SV=1	VDYQTGPIVWGEPGTNGQHAFYQLIHQGTK
>	sp|Q6P6V0|G6 Glucose-6-phosphate isomerase OS=Rattus norvegicus GN=Gpi PE=1 SV=1	AITDIINIGIGGSDLGPLMVTEALKPYSK
>	sp|Q6P6V0|G6 Glucose-6-phosphate isomerase OS=Rattus norvegicus GN=Gpi PE=1 SV=1	HFVALSTNTDKVK
>	sp|Q6P6V0|G6 Glucose-6-phosphate isomerase OS=Rattus norvegicus GN=Gpi PE=1 SV=1	AITDIINIGIGGSDLGPLMVTEALKPYSK
>	sp|Q6P6V0|G6 Glucose-6-phosphate isomerase OS=Rattus norvegicus GN=Gpi PE=1 SV=1	VDYQTGPIVWGEPGTNGQHAFYQLIHQGTK
>	sp|Q6P6V0|G6 Glucose-6-phosphate isomerase OS=Rattus norvegicus GN=Gpi PE=1 SV=1	AITDIINIGIGGSDLGPLMVTEALKPYSK
>	sp|Q6P6V0|G6 Glucose-6-phosphate isomerase OS=Rattus norvegicus GN=Gpi PE=1 SV=1	MIPCDFLIPVQTQHPIR
>	sp|Q6P6V0|G6 Glucose-6-phosphate isomerase OS=Rattus norvegicus GN=Gpi PE=1 SV=1	NLVNKEVLHMLVDLAK
>	sp|Q6P6V0|G6 Glucose-6-phosphate isomerase OS=Rattus norvegicus GN=Gpi PE=1 SV=1	VDYQTGPIVWGEPGTNGQHAFYQLIHQGTK
>	sp|P07936|NEU Neuromodulin OS=Rattus norvegicus GN=Gap43 PE=1 SV=1	AEEPSKAGDAPSEEK
>	sp|P07936|NEU Neuromodulin OS=Rattus norvegicus GN=Gap43 PE=1 SV=1	EKDDAPVADGVEK
>	sp|P07936|NEU Neuromodulin OS=Rattus norvegicus GN=Gap43 PE=1 SV=1	KGDAPAAEAEAK
>	sp|P07936|NEU Neuromodulin OS=Rattus norvegicus GN=Gap43 PE=1 SV=1	KGDAPAAEAEAKEKDDAPVADGVEKK
>	sp|P07936|NEU Neuromodulin OS=Rattus norvegicus GN=Gap43 PE=1 SV=1	KGEGDAAPSEEK
>	sp|P07936|NEU Neuromodulin OS=Rattus norvegicus GN=Gap43 PE=1 SV=1	QDEGKEDPEADQEHA
>	sp|P07936|NEU Neuromodulin OS=Rattus norvegicus GN=Gap43 PE=1 SV=1	QVEKNDEDQKIEQDGVKPEDK
>	sp|P07936|NEU Neuromodulin OS=Rattus norvegicus GN=Gap43 PE=1 SV=1	TKQVEKNDEDQKIEQDGVKPEDK
>	sp|P07936|NEU Neuromodulin OS=Rattus norvegicus GN=Gap43 PE=1 SV=1	QADVPAAVTDAAATTPAAEDAAK
>	sp|P07936|NEU Neuromodulin OS=Rattus norvegicus GN=Gap43 PE=1 SV=1	AEEPSKAGDAPSEEKKGEGDAAPSEEK
>	sp|P07936|NEU Neuromodulin OS=Rattus norvegicus GN=Gap43 PE=1 SV=1	ESARQDEGKEDPEADQEHA
>	sp|P07936|NEU Neuromodulin OS=Rattus norvegicus GN=Gap43 PE=1 SV=1	AEEPSKAGDAPSEEK
>	sp|P07936|NEU Neuromodulin OS=Rattus norvegicus GN=Gap43 PE=1 SV=1	AEEPSKAGDAPSEEK
>	sp|P07936|NEU Neuromodulin OS=Rattus norvegicus GN=Gap43 PE=1 SV=1	AEEPSKAGDAPSEEK
>	sp|P07936|NEU Neuromodulin OS=Rattus norvegicus GN=Gap43 PE=1 SV=1	ESARQDEGKEDPEADQEHA
>	sp|P07936|NEU Neuromodulin OS=Rattus norvegicus GN=Gap43 PE=1 SV=1	KGDAPAAEAEAK
>	sp|P07936|NEU Neuromodulin OS=Rattus norvegicus GN=Gap43 PE=1 SV=1	KGDAPAAEAEAKEKDDAPVADGVEKK
>	sp|P07936|NEU Neuromodulin OS=Rattus norvegicus GN=Gap43 PE=1 SV=1	QVEKNDEDQKIEQDGVKPEDK
>	sp|P07936|NEU Neuromodulin OS=Rattus norvegicus GN=Gap43 PE=1 SV=1	QVEKNDEDQKIEQDGVKPEDK
>	sp|P07936|NEU Neuromodulin OS=Rattus norvegicus GN=Gap43 PE=1 SV=1	QVEKNDEDQKIEQDGVKPEDK
>	sp|P07936|NEU Neuromodulin OS=Rattus norvegicus GN=Gap43 PE=1 SV=1	ESARQDEGKEDPEADQEHA
>	sp|P07936|NEU Neuromodulin OS=Rattus norvegicus GN=Gap43 PE=1 SV=1	AGSAETESAAK
>	sp|P07936|NEU Neuromodulin OS=Rattus norvegicus GN=Gap43 PE=1 SV=1	AGSAETESAAK
>	sp|P07936|NEU Neuromodulin OS=Rattus norvegicus GN=Gap43 PE=1 SV=1	KGEGDAAPSEEK
>	sp|P07936|NEU Neuromodulin OS=Rattus norvegicus GN=Gap43 PE=1 SV=1	ATTDNSPSSK
>	sp|P07936|NEU Neuromodulin OS=Rattus norvegicus GN=Gap43 PE=1 SV=1	KGEGDAAPSEEK
>	sp|P07936|NEU Neuromodulin OS=Rattus norvegicus GN=Gap43 PE=1 SV=1	AAQPPTETAESSQAEEEKEAVDEAKPK
>	sp|P07936|NEU Neuromodulin OS=Rattus norvegicus GN=Gap43 PE=1 SV=1	QVEKNDEDQKIEQDGVKPEDK
>	sp|P07936|NEU Neuromodulin OS=Rattus norvegicus GN=Gap43 PE=1 SV=1	AEEPSKAGDAPSEEK
>	sp|P07936|NEU Neuromodulin OS=Rattus norvegicus GN=Gap43 PE=1 SV=1	QDEGKEDPEADQEHA
>	sp|P07936|NEU Neuromodulin OS=Rattus norvegicus GN=Gap43 PE=1 SV=1	QVEKNDEDQKIEQDGVKPEDK
>	sp|P02688|MB Myelin basic protein S OS=Rattus norvegicus GN=Mbp PE=1 SV=3	DTGILDSIGR
>	sp|P02688|MB Myelin basic protein S OS=Rattus norvegicus GN=Mbp PE=1 SV=3	FSWGAEGQKPGFGYGGR
>	sp|P02688|MB Myelin basic protein S OS=Rattus norvegicus GN=Mbp PE=1 SV=3	GAYDAQGTLSK
>	sp|P02688|MB Myelin basic protein S OS=Rattus norvegicus GN=Mbp PE=1 SV=3	HRDTGILDSIGR
>	sp|P02688|MB Myelin basic protein S OS=Rattus norvegicus GN=Mbp PE=1 SV=3	NIVTPR
>	sp|P02688|MB Myelin basic protein S OS=Rattus norvegicus GN=Mbp PE=1 SV=3	TPPPSQGK
>	sp|P02688|MB Myelin basic protein S OS=Rattus norvegicus GN=Mbp PE=1 SV=3	TQDENPVVHFFK
>	sp|P02688|MB Myelin basic protein S OS=Rattus norvegicus GN=Mbp PE=1 SV=3	TTHYGSLPQK

>	sp|P02688|MB Myelin basic protein S OS=Rattus norvegicus GN=Mbp PE=1 SV=3	YLATASTMDHAR
>	sp|P02688|MB Myelin basic protein S OS=Rattus norvegicus GN=Mbp PE=1 SV=3	DENPVVHFFK
>	sp|P02688|MB Myelin basic protein S OS=Rattus norvegicus GN=Mbp PE=1 SV=3	DENPVVHFFK
>	sp|P02688|MB Myelin basic protein S OS=Rattus norvegicus GN=Mbp PE=1 SV=3	DTGILDSIGR
>	sp|P02688|MB Myelin basic protein S OS=Rattus norvegicus GN=Mbp PE=1 SV=3	DTGILDSIGR
>	sp|P02688|MB Myelin basic protein S OS=Rattus norvegicus GN=Mbp PE=1 SV=3	DTGILDSIGR
>	sp|P02688|MB Myelin basic protein S OS=Rattus norvegicus GN=Mbp PE=1 SV=3	DTGILDSIGR
>	sp|P02688|MB Myelin basic protein S OS=Rattus norvegicus GN=Mbp PE=1 SV=3	DTGILDSIGR
>	sp|P02688|MB Myelin basic protein S OS=Rattus norvegicus GN=Mbp PE=1 SV=3	DTGILDSIGR
>	sp|P02688|MB Myelin basic protein S OS=Rattus norvegicus GN=Mbp PE=1 SV=3	DTGILDSIGR
>	sp|P02688|MB Myelin basic protein S OS=Rattus norvegicus GN=Mbp PE=1 SV=3	DTGILDSIGR
>	sp|P02688|MB Myelin basic protein S OS=Rattus norvegicus GN=Mbp PE=1 SV=3	FSWGAEGQKPGFGYGGR
>	sp|P02688|MB Myelin basic protein S OS=Rattus norvegicus GN=Mbp PE=1 SV=3	FSWGAEGQKPGFGYGGR
>	sp|P02688|MB Myelin basic protein S OS=Rattus norvegicus GN=Mbp PE=1 SV=3	FSWGAEGQKPGFGYGGR
>	sp|P02688|MB Myelin basic protein S OS=Rattus norvegicus GN=Mbp PE=1 SV=3	FSWGAEGQKPGFGYGGR
>	sp|P02688|MB Myelin basic protein S OS=Rattus norvegicus GN=Mbp PE=1 SV=3	GAYDAQGTLSK
>	sp|P02688|MB Myelin basic protein S OS=Rattus norvegicus GN=Mbp PE=1 SV=3	GAYDAQGTLSK
>	sp|P02688|MB Myelin basic protein S OS=Rattus norvegicus GN=Mbp PE=1 SV=3	HRDTGILDSIGR
>	sp|P02688|MB Myelin basic protein S OS=Rattus norvegicus GN=Mbp PE=1 SV=3	HRDTGILDSIGR
>	sp|P02688|MB Myelin basic protein S OS=Rattus norvegicus GN=Mbp PE=1 SV=3	TPPPSQGK
>	sp|P02688|MB Myelin basic protein S OS=Rattus norvegicus GN=Mbp PE=1 SV=3	TQDENPVVHFFK
>	sp|P02688|MB Myelin basic protein S OS=Rattus norvegicus GN=Mbp PE=1 SV=3	TQDENPVVHFFK
>	sp|P02688|MB Myelin basic protein S OS=Rattus norvegicus GN=Mbp PE=1 SV=3	TQDENPVVHFFK
>	sp|P02688|MB Myelin basic protein S OS=Rattus norvegicus GN=Mbp PE=1 SV=3	TQDENPVVHFFK
>	sp|P02688|MB Myelin basic protein S OS=Rattus norvegicus GN=Mbp PE=1 SV=3	TQDENPVVHFFK
>	sp|P02688|MB Myelin basic protein S OS=Rattus norvegicus GN=Mbp PE=1 SV=3	TQDENPVVHFFK
>	sp|P02688|MB Myelin basic protein S OS=Rattus norvegicus GN=Mbp PE=1 SV=3	TQDENPVVHFFK
>	sp|P02688|MB Myelin basic protein S OS=Rattus norvegicus GN=Mbp PE=1 SV=3	TQDENPVVHFFK
>	sp|P02688|MB Myelin basic protein S OS=Rattus norvegicus GN=Mbp PE=1 SV=3	TQDENPVVHFFK
>	sp|P02688|MB Myelin basic protein S OS=Rattus norvegicus GN=Mbp PE=1 SV=3	TQDENPVVHFFK
>	sp|P02688|MB Myelin basic protein S OS=Rattus norvegicus GN=Mbp PE=1 SV=3	TQDENPVVHFFK
>	sp|P02688|MB Myelin basic protein S OS=Rattus norvegicus GN=Mbp PE=1 SV=3	TQDENPVVHFFK
>	sp|P02688|MB Myelin basic protein S OS=Rattus norvegicus GN=Mbp PE=1 SV=3	TQDENPVVHFFK
>	sp|P02688|MB Myelin basic protein S OS=Rattus norvegicus GN=Mbp PE=1 SV=3	TQDENPVVHFFK
>	sp|P02688|MB Myelin basic protein S OS=Rattus norvegicus GN=Mbp PE=1 SV=3	TQDENPVVHFFK
>	sp|P02688|MB Myelin basic protein S OS=Rattus norvegicus GN=Mbp PE=1 SV=3	TQDENPVVHFFK
>	sp|P02688|MB Myelin basic protein S OS=Rattus norvegicus GN=Mbp PE=1 SV=3	TQDENPVVHFFK
>	sp|P02688|MB Myelin basic protein S OS=Rattus norvegicus GN=Mbp PE=1 SV=3	TTHYGSLPQK
>	sp|P02688|MB Myelin basic protein S OS=Rattus norvegicus GN=Mbp PE=1 SV=3	YLATASTMDHAR
>	sp|P02688|MB Myelin basic protein S OS=Rattus norvegicus GN=Mbp PE=1 SV=3	YLATASTMDHAR
>	sp|P02688|MB Myelin basic protein S OS=Rattus norvegicus GN=Mbp PE=1 SV=3	YLATASTMDHAR
>	sp|P02688|MB Myelin basic protein S OS=Rattus norvegicus GN=Mbp PE=1 SV=3	YLATASTMDHAR
>	sp|P02688|MB Myelin basic protein S OS=Rattus norvegicus GN=Mbp PE=1 SV=3	YLATASTMDHAR
>	sp|P02688|MB Myelin basic protein S OS=Rattus norvegicus GN=Mbp PE=1 SV=3	YLATASTMDHAR
>	sp|P02688|MB Myelin basic protein S OS=Rattus norvegicus GN=Mbp PE=1 SV=3	YLATASTMDHAR
>	sp|P02688|MB Myelin basic protein S OS=Rattus norvegicus GN=Mbp PE=1 SV=3	YLATASTMDHAR
>	sp|P02688|MB Myelin basic protein S OS=Rattus norvegicus GN=Mbp PE=1 SV=3	FSWGAEGQKPGFGYGGR
>	sp|P02688|MB Myelin basic protein S OS=Rattus norvegicus GN=Mbp PE=1 SV=3	YLATASTMDHAR
>	sp|P02688|MB Myelin basic protein S OS=Rattus norvegicus GN=Mbp PE=1 SV=3	YLATASTMDHAR
>	sp|P02688|MB Myelin basic protein S OS=Rattus norvegicus GN=Mbp PE=1 SV=3	NIVTPRTPPPSQGK
>	sp|P02688|MB Myelin basic protein S OS=Rattus norvegicus GN=Mbp PE=1 SV=3	HRDTGILDSIGR
>	sp|P02688|MB Myelin basic protein S OS=Rattus norvegicus GN=Mbp PE=1 SV=3	TPPPSQGK
>	sp|P02688|MB Myelin basic protein S OS=Rattus norvegicus GN=Mbp PE=1 SV=3	DENPVVHFFK
>	sp|P02688|MB Myelin basic protein S OS=Rattus norvegicus GN=Mbp PE=1 SV=3	TQDENPVVHFFK
>	sp|P02688|MB Myelin basic protein S OS=Rattus norvegicus GN=Mbp PE=1 SV=3	FSWGAEGQKPGFGYGGR
>	sp|P02688|MB Myelin basic protein S OS=Rattus norvegicus GN=Mbp PE=1 SV=3	TPPPSQGK
>	sp|P02688|MB Myelin basic protein S OS=Rattus norvegicus GN=Mbp PE=1 SV=3	TPPPSQGK
>	sp|P02688|MB Myelin basic protein S OS=Rattus norvegicus GN=Mbp PE=1 SV=3	GAYDAQGTLSK
>	sp|P02688|MB Myelin basic protein S OS=Rattus norvegicus GN=Mbp PE=1 SV=3	HRDTGILDSIGR
>	sp|P02688|MB Myelin basic protein S OS=Rattus norvegicus GN=Mbp PE=1 SV=3	YLATASTMDHAR
>	sp|P02688|MB Myelin basic protein S OS=Rattus norvegicus GN=Mbp PE=1 SV=3	TQDENPVVHFFK
>	sp|P02688|MB Myelin basic protein S OS=Rattus norvegicus GN=Mbp PE=1 SV=3	SQRTQDENPVVHFFK
>	sp|P02688|MB Myelin basic protein S OS=Rattus norvegicus GN=Mbp PE=1 SV=3	TQDENPVVHFFK
>	sp|P02688|MB Myelin basic protein S OS=Rattus norvegicus GN=Mbp PE=1 SV=3	TQDENPVVHFFK
>	sp|P02688|MB Myelin basic protein S OS=Rattus norvegicus GN=Mbp PE=1 SV=3	GLSLSR
>	sp|P02688|MB Myelin basic protein S OS=Rattus norvegicus GN=Mbp PE=1 SV=3	TQDENPVVHFFK
>	sp|P02688|MB Myelin basic protein S OS=Rattus norvegicus GN=Mbp PE=1 SV=3	TTHYGSLPQK
>	sp|P02688|MB Myelin basic protein S OS=Rattus norvegicus GN=Mbp PE=1 SV=3	HRDTGILDSIGR
>	sp|P02688|MB Myelin basic protein S OS=Rattus norvegicus GN=Mbp PE=1 SV=3	HRDTGILDSIGR
>	sp|P02688|MB Myelin basic protein S OS=Rattus norvegicus GN=Mbp PE=1 SV=3	FSWGAEGQKPGFGYGGR
>	sp|P02688|MB Myelin basic protein S OS=Rattus norvegicus GN=Mbp PE=1 SV=3	SQRTQDENPVVHFFK
>	sp|P02688|MB Myelin basic protein S OS=Rattus norvegicus GN=Mbp PE=1 SV=3	TQDENPVVHFFK
>	sp|P02688|MB Myelin basic protein S OS=Rattus norvegicus GN=Mbp PE=1 SV=3	ATASTMDHAR
>	sp|P02688|MB Myelin basic protein S OS=Rattus norvegicus GN=Mbp PE=1 SV=3	DTGILDSIGR

>	sp|P02688|MB Myelin basic protein S OS=Rattus norvegicus GN=Mbp PE=1 SV=3	DENPVVHFFK
>	sp|P02688|MB Myelin basic protein S OS=Rattus norvegicus GN=Mbp PE=1 SV=3	TPPPSQGK
>	sp|P02688|MB Myelin basic protein S OS=Rattus norvegicus GN=Mbp PE=1 SV=3	HRDTGILDSIGR
>	sp|P02688|MB Myelin basic protein S OS=Rattus norvegicus GN=Mbp PE=1 SV=3	TTHYGSLPQK
>	sp|P02688|MB Myelin basic protein S OS=Rattus norvegicus GN=Mbp PE=1 SV=3	TPPPSQGK
>	sp|P02688|MB Myelin basic protein S OS=Rattus norvegicus GN=Mbp PE=1 SV=3	YLATASTMDHAR
>	sp|P02688|MB Myelin basic protein S OS=Rattus norvegicus GN=Mbp PE=1 SV=3	HRDTGILDSIGR
>	sp|P02688|MB Myelin basic protein S OS=Rattus norvegicus GN=Mbp PE=1 SV=3	TTHYGSLPQK
>	sp|P02688|MB Myelin basic protein S OS=Rattus norvegicus GN=Mbp PE=1 SV=3	YLATASTMDHAR
>	sp|P31596|EAA Excitatory amino acid transporter 2 OS=Rattus norvegicus GN=Slc1a2 PE=1 SV=2	CLEDNLGIDKR
>	sp|P31596|EAA Excitatory amino acid transporter 2 OS=Rattus norvegicus GN=Slc1a2 PE=1 SV=2	KNDEVSSLDAFLDLIR
>	sp|P31596|EAA Excitatory amino acid transporter 2 OS=Rattus norvegicus GN=Slc1a2 PE=1 SV=2	KQLGPGKK
>	sp|P31596|EAA Excitatory amino acid transporter 2 OS=Rattus norvegicus GN=Slc1a2 PE=1 SV=2	NDEVSSLDAFLDLIR
>	sp|P31596|EAA Excitatory amino acid transporter 2 OS=Rattus norvegicus GN=Slc1a2 PE=1 SV=2	NLFPENLVQACFQQIQTVTK
>	sp|P31596|EAA Excitatory amino acid transporter 2 OS=Rattus norvegicus GN=Slc1a2 PE=1 SV=2	NLFPENLVQACFQQIQTVTKK
>	sp|P31596|EAA Excitatory amino acid transporter 2 OS=Rattus norvegicus GN=Slc1a2 PE=1 SV=2	SADCSVEEEPWKR
>	sp|P31596|EAA Excitatory amino acid transporter 2 OS=Rattus norvegicus GN=Slc1a2 PE=1 SV=2	SELDTIDSQHR
>	sp|P31596|EAA Excitatory amino acid transporter 2 OS=Rattus norvegicus GN=Slc1a2 PE=1 SV=2	KNDEVSSLDAFLDLIR
>	sp|P31596|EAA Excitatory amino acid transporter 2 OS=Rattus norvegicus GN=Slc1a2 PE=1 SV=2	KNDEVSSLDAFLDLIR
>	sp|P31596|EAA Excitatory amino acid transporter 2 OS=Rattus norvegicus GN=Slc1a2 PE=1 SV=2	KNDEVSSLDAFLDLIR
>	sp|P31596|EAA Excitatory amino acid transporter 2 OS=Rattus norvegicus GN=Slc1a2 PE=1 SV=2	KNDEVSSLDAFLDLIR
>	sp|P31596|EAA Excitatory amino acid transporter 2 OS=Rattus norvegicus GN=Slc1a2 PE=1 SV=2	KNDEVSSLDAFLDLIR
>	sp|P31596|EAA Excitatory amino acid transporter 2 OS=Rattus norvegicus GN=Slc1a2 PE=1 SV=2	KNDEVSSLDAFLDLIR
>	sp|P31596|EAA Excitatory amino acid transporter 2 OS=Rattus norvegicus GN=Slc1a2 PE=1 SV=2	KNDEVSSLDAFLDLIR
>	sp|P31596|EAA Excitatory amino acid transporter 2 OS=Rattus norvegicus GN=Slc1a2 PE=1 SV=2	KNDEVSSLDAFLDLIR
>	sp|P31596|EAA Excitatory amino acid transporter 2 OS=Rattus norvegicus GN=Slc1a2 PE=1 SV=2	KNDEVSSLDAFLDLIR
>	sp|P31596|EAA Excitatory amino acid transporter 2 OS=Rattus norvegicus GN=Slc1a2 PE=1 SV=2	NDEVSSLDAFLDLIR
>	sp|P31596|EAA Excitatory amino acid transporter 2 OS=Rattus norvegicus GN=Slc1a2 PE=1 SV=2	NDEVSSLDAFLDLIR
>	sp|P31596|EAA Excitatory amino acid transporter 2 OS=Rattus norvegicus GN=Slc1a2 PE=1 SV=2	NLFPENLVQACFQQIQTVTK
>	sp|P31596|EAA Excitatory amino acid transporter 2 OS=Rattus norvegicus GN=Slc1a2 PE=1 SV=2	NLFPENLVQACFQQIQTVTK
>	sp|P31596|EAA Excitatory amino acid transporter 2 OS=Rattus norvegicus GN=Slc1a2 PE=1 SV=2	NLFPENLVQACFQQIQTVTK
>	sp|P31596|EAA Excitatory amino acid transporter 2 OS=Rattus norvegicus GN=Slc1a2 PE=1 SV=2	NLFPENLVQACFQQIQTVTKK
>	sp|P31596|EAA Excitatory amino acid transporter 2 OS=Rattus norvegicus GN=Slc1a2 PE=1 SV=2	SADCSVEEEPWKR
>	sp|P31596|EAA Excitatory amino acid transporter 2 OS=Rattus norvegicus GN=Slc1a2 PE=1 SV=2	SADCSVEEEPWKR
>	sp|P31596|EAA Excitatory amino acid transporter 2 OS=Rattus norvegicus GN=Slc1a2 PE=1 SV=2	KNDEVSSLDAFLDLIR
>	sp|P31596|EAA Excitatory amino acid transporter 2 OS=Rattus norvegicus GN=Slc1a2 PE=1 SV=2	NLFPENLVQACFQQIQTVTK
>	sp|P31596|EAA Excitatory amino acid transporter 2 OS=Rattus norvegicus GN=Slc1a2 PE=1 SV=2	KNDEVSSLDAFLDLIR
>	sp|P31596|EAA Excitatory amino acid transporter 2 OS=Rattus norvegicus GN=Slc1a2 PE=1 SV=2	TQSVYDDTK
>	sp|P31596|EAA Excitatory amino acid transporter 2 OS=Rattus norvegicus GN=Slc1a2 PE=1 SV=2	SELDTIDSQHR
>	sp|P31596|EAA Excitatory amino acid transporter 2 OS=Rattus norvegicus GN=Slc1a2 PE=1 SV=2	MHEDIEMTK
>	sp|P31596|EAA Excitatory amino acid transporter 2 OS=Rattus norvegicus GN=Slc1a2 PE=1 SV=2	SELDTIDSQHR
>	sp|P31596|EAA Excitatory amino acid transporter 2 OS=Rattus norvegicus GN=Slc1a2 PE=1 SV=2	NLFPENLVQACFQQIQTVTKK
>	sp|P31596|EAA Excitatory amino acid transporter 2 OS=Rattus norvegicus GN=Slc1a2 PE=1 SV=2	QLGPGKKNDEVSSLDAFLDLIR
>	sp|P31596|EAA Excitatory amino acid transporter 2 OS=Rattus norvegicus GN=Slc1a2 PE=1 SV=2	NLFPENLVQACFQQIQTVTKK
>	sp|P31596|EAA Excitatory amino acid transporter 2 OS=Rattus norvegicus GN=Slc1a2 PE=1 SV=2	KNDEVSSLDAFLDLIR
>	sp|P31596|EAA Excitatory amino acid transporter 2 OS=Rattus norvegicus GN=Slc1a2 PE=1 SV=2	CLEDNLGIDKR
>	sp|P31596|EAA Excitatory amino acid transporter 2 OS=Rattus norvegicus GN=Slc1a2 PE=1 SV=2	NLFPENLVQACFQQIQTVTK
>	sp|P31596|EAA Excitatory amino acid transporter 2 OS=Rattus norvegicus GN=Slc1a2 PE=1 SV=2	MHEDIEMTK
>	sp|P31596|EAA Excitatory amino acid transporter 2 OS=Rattus norvegicus GN=Slc1a2 PE=1 SV=2	TQSVYDDTK
>	sp|P31596|EAA Excitatory amino acid transporter 2 OS=Rattus norvegicus GN=Slc1a2 PE=1 SV=2	MLILPLIISS
>	sp|P31596|EAA Excitatory amino acid transporter 2 OS=Rattus norvegicus GN=Slc1a2 PE=1 SV=2	NLFPENLVQACFQQIQTVTK
>	sp|P31596|EAA Excitatory amino acid transporter 2 OS=Rattus norvegicus GN=Slc1a2 PE=1 SV=2	SADCSVEEEPWKR
>	sp|Q63537|SYN Synapsin-2 OS=Rattus norvegicus GN=Syn2 PE=1 SV=1	DGKDYIFEVMDCSMPLIGEHQVEDR
>	sp|Q63537|SYN Synapsin-2 OS=Rattus norvegicus GN=Syn2 PE=1 SV=1	QHAFGMAENEDFR
>	sp|Q63537|SYN Synapsin-2 OS=Rattus norvegicus GN=Syn2 PE=1 SV=1	QTAASAGLVDAPAPSAASR
>	sp|Q63537|SYN Synapsin-2 OS=Rattus norvegicus GN=Syn2 PE=1 SV=1	RPPPAQAPAPQPAPQPAPTPSVGSSFFSSLSQAVK
>	sp|Q63537|SYN Synapsin-2 OS=Rattus norvegicus GN=Syn2 PE=1 SV=1	SFRPDFVLIR
>	sp|Q63537|SYN Synapsin-2 OS=Rattus norvegicus GN=Syn2 PE=1 SV=1	TPALSPQRPLTTQQPQSGTLKEPDSSK
>	sp|Q63537|SYN Synapsin-2 OS=Rattus norvegicus GN=Syn2 PE=1 SV=1	VEQAEFSELNLVAHADGTYAVDMQVLR
>	sp|Q63537|SYN Synapsin-2 OS=Rattus norvegicus GN=Syn2 PE=1 SV=1	VKVENHYDFQDIASVVALTQTYATAEPFIDAK
>	sp|Q63537|SYN Synapsin-2 OS=Rattus norvegicus GN=Syn2 PE=1 SV=1	VLLVVDEPHTDWAK
>	sp|Q63537|SYN Synapsin-2 OS=Rattus norvegicus GN=Syn2 PE=1 SV=1	ILGDYDIKVEQAEFSELNLVAHADGTYAVDMQVLR
>	sp|Q63537|SYN Synapsin-2 OS=Rattus norvegicus GN=Syn2 PE=1 SV=1	EMLTLPTFPVVVK
>	sp|Q63537|SYN Synapsin-2 OS=Rattus norvegicus GN=Syn2 PE=1 SV=1	DGKDYIFEVMDCSMPLIGEHQVEDR
>	sp|Q63537|SYN Synapsin-2 OS=Rattus norvegicus GN=Syn2 PE=1 SV=1	DGKDYIFEVMDCSMPLIGEHQVEDR
>	sp|Q63537|SYN Synapsin-2 OS=Rattus norvegicus GN=Syn2 PE=1 SV=1	DGKDYIFEVMDCSMPLIGEHQVEDR
>	sp|Q63537|SYN Synapsin-2 OS=Rattus norvegicus GN=Syn2 PE=1 SV=1	DGKDYIFEVMDCSMPLIGEHQVEDR
>	sp|Q63537|SYN Synapsin-2 OS=Rattus norvegicus GN=Syn2 PE=1 SV=1	DGKDYIFEVMDCSMPLIGEHQVEDR
>	sp|Q63537|SYN Synapsin-2 OS=Rattus norvegicus GN=Syn2 PE=1 SV=1	DGKDYIFEVMDCSMPLIGEHQVEDR
>	sp|Q63537|SYN Synapsin-2 OS=Rattus norvegicus GN=Syn2 PE=1 SV=1	DGKDYIFEVMDCSMPLIGEHQVEDR
>	sp|Q63537|SYN Synapsin-2 OS=Rattus norvegicus GN=Syn2 PE=1 SV=1	DGKDYIFEVMDCSMPLIGEHQVEDR
>	sp|Q63537|SYN Synapsin-2 OS=Rattus norvegicus GN=Syn2 PE=1 SV=1	DGKDYIFEVMDCSMPLIGEHQVEDR
>	sp|Q63537|SYN Synapsin-2 OS=Rattus norvegicus GN=Syn2 PE=1 SV=1	DGKDYIFEVMDCSMPLIGEHQVEDR
>	sp|Q63537|SYN Synapsin-2 OS=Rattus norvegicus GN=Syn2 PE=1 SV=1	QHAFGMAENEDFR

>	sp|Q63537|SYI	Synapsin-2 OS=Rattus norvegicus GN=Syn2 PE=1 SV=1	QHAFGMAENEDFR
>	sp|Q63537|SYI	Synapsin-2 OS=Rattus norvegicus GN=Syn2 PE=1 SV=1	QTAASAGLVDAPAPSAASR
>	sp|Q63537|SYI	Synapsin-2 OS=Rattus norvegicus GN=Syn2 PE=1 SV=1	SFRPDFVLIR
>	sp|Q63537|SYI	Synapsin-2 OS=Rattus norvegicus GN=Syn2 PE=1 SV=1	SFRPDFVLIR
>	sp|Q63537|SYI	Synapsin-2 OS=Rattus norvegicus GN=Syn2 PE=1 SV=1	SFRPDFVLIR
>	sp|Q63537|SYI	Synapsin-2 OS=Rattus norvegicus GN=Syn2 PE=1 SV=1	SFRPDFVLIR
>	sp|Q63537|SYI	Synapsin-2 OS=Rattus norvegicus GN=Syn2 PE=1 SV=1	SFRPDFVLIR
>	sp|Q63537|SYI	Synapsin-2 OS=Rattus norvegicus GN=Syn2 PE=1 SV=1	SFRPDFVLIR
>	sp|Q63537|SYI	Synapsin-2 OS=Rattus norvegicus GN=Syn2 PE=1 SV=1	TNTGSAMLEQIAMSDR
>	sp|Q63537|SYI	Synapsin-2 OS=Rattus norvegicus GN=Syn2 PE=1 SV=1	TNTGSAMLEQIAMSDR
>	sp|Q63537|SYI	Synapsin-2 OS=Rattus norvegicus GN=Syn2 PE=1 SV=1	TNTGSAMLEQIAMSDR
>	sp|Q63537|SYI	Synapsin-2 OS=Rattus norvegicus GN=Syn2 PE=1 SV=1	TNTGSAMLEQIAMSDR
>	sp|Q63537|SYI	Synapsin-2 OS=Rattus norvegicus GN=Syn2 PE=1 SV=1	TPALSPQRPLTTQQPQSGTLKEPDSSK
>	sp|Q63537|SYI	Synapsin-2 OS=Rattus norvegicus GN=Syn2 PE=1 SV=1	VEQAEFSELNLVAHADGTYAVDMQVLR
>	sp|Q63537|SYI	Synapsin-2 OS=Rattus norvegicus GN=Syn2 PE=1 SV=1	VLLVVDEPHTDWAK
>	sp|Q63537|SYI	Synapsin-2 OS=Rattus norvegicus GN=Syn2 PE=1 SV=1	VLLVVDEPHTDWAK
>	sp|Q63537|SYI	Synapsin-2 OS=Rattus norvegicus GN=Syn2 PE=1 SV=1	VLLVVDEPHTDWAK
>	sp|Q63537|SYI	Synapsin-2 OS=Rattus norvegicus GN=Syn2 PE=1 SV=1	VLLVVDEPHTDWAK
>	sp|Q63537|SYI	Synapsin-2 OS=Rattus norvegicus GN=Syn2 PE=1 SV=1	VKVENHYDFQDIASVVALTQTYATAEPFIDAK
>	sp|Q63537|SYI	Synapsin-2 OS=Rattus norvegicus GN=Syn2 PE=1 SV=1	RPPPAQAPAPQPAPQPAPTPSVGSSFFSSLSQAVK
>	sp|Q63537|SYI	Synapsin-2 OS=Rattus norvegicus GN=Syn2 PE=1 SV=1	QLITDLVISK
>	sp|Q63537|SYI	Synapsin-2 OS=Rattus norvegicus GN=Syn2 PE=1 SV=1	TNTGSAMLEQIAMSDR
>	sp|Q63537|SYI	Synapsin-2 OS=Rattus norvegicus GN=Syn2 PE=1 SV=1	RPPPAQAPAPQPAPQPAPTPSVGSSFFSSLSQAVK
>	sp|Q63537|SYI	Synapsin-2 OS=Rattus norvegicus GN=Syn2 PE=1 SV=1	EMLTLPTFPVVVK
>	sp|Q63537|SYI	Synapsin-2 OS=Rattus norvegicus GN=Syn2 PE=1 SV=1	RPPPAQAPAPQPAPQPAPTPSVGSSFFSSLSQAVK
>	sp|Q63537|SYI	Synapsin-2 OS=Rattus norvegicus GN=Syn2 PE=1 SV=1	SFRPDFVLIR
>	sp|Q63537|SYI	Synapsin-2 OS=Rattus norvegicus GN=Syn2 PE=1 SV=1	VLLVVDEPHTDWAK
>	sp|Q63537|SYI	Synapsin-2 OS=Rattus norvegicus GN=Syn2 PE=1 SV=1	SFRPDFVLIR
>	sp|Q63537|SYI	Synapsin-2 OS=Rattus norvegicus GN=Syn2 PE=1 SV=1	SFRPDFVLIR
>	sp|Q63537|SYI	Synapsin-2 OS=Rattus norvegicus GN=Syn2 PE=1 SV=1	VKVENHYDFQDIASVVALTQTYATAEPFIDAK
>	sp|Q63537|SYI	Synapsin-2 OS=Rattus norvegicus GN=Syn2 PE=1 SV=1	QLITDLVISK
>	sp|Q63537|SYI	Synapsin-2 OS=Rattus norvegicus GN=Syn2 PE=1 SV=1	EMLTLPTFPVVVK
>	sp|Q63537|SYI	Synapsin-2 OS=Rattus norvegicus GN=Syn2 PE=1 SV=1	VKVENHYDFQDIASVVALTQTYATAEPFIDAK
>	sp|Q63537|SYI	Synapsin-2 OS=Rattus norvegicus GN=Syn2 PE=1 SV=1	QHAFGMAENEDFR
>	sp|Q63754|SYl	Beta-synuclein OS=Rattus norvegicus GN=Sncb PE=1 SV=1	EGVVAAAEK
>	sp|Q63754|SYl	Beta-synuclein OS=Rattus norvegicus GN=Sncb PE=1 SV=1	EGVVQGVASVAEK
>	sp|Q63754|SYl	Beta-synuclein OS=Rattus norvegicus GN=Sncb PE=1 SV=1	EQASHLGGAVFSGAGNIAAATGLVK
>	sp|Q63754|SYl	Beta-synuclein OS=Rattus norvegicus GN=Sncb PE=1 SV=1	KEEFPTDLKPEEVAQEAAEEPLIEPLMEPEGESYEDSPQEEYQEYEPEAKGP
>	sp|Q63754|SYl	Beta-synuclein OS=Rattus norvegicus GN=Sncb PE=1 SV=1	TKEGVLYVGSK
>	sp|Q63754|SYl	Beta-synuclein OS=Rattus norvegicus GN=Sncb PE=1 SV=1	TKEQASHLGGAVFSGAGNIAAATGLVK
>	sp|Q63754|SYl	Beta-synuclein OS=Rattus norvegicus GN=Sncb PE=1 SV=1	TKQGVTEAAEK
>	sp|Q63754|SYl	Beta-synuclein OS=Rattus norvegicus GN=Sncb PE=1 SV=1	KEEFPTDLKPEEVAQEAAEEPLIEPLMEPEGESYEDSPQEEYQEYEPEAK
>	sp|Q63754|SYl	Beta-synuclein OS=Rattus norvegicus GN=Sncb PE=1 SV=1	AKEGVVAAAEK
>	sp|Q63754|SYl	Beta-synuclein OS=Rattus norvegicus GN=Sncb PE=1 SV=1	KEEFPTDLKPEEVAQEAAEEPLIEPLMEPEGESYEDSPQEEYQEYEPEA
>	sp|Q63754|SYl	Beta-synuclein OS=Rattus norvegicus GN=Sncb PE=1 SV=1	AKEGVVAAAEK
>	sp|Q63754|SYl	Beta-synuclein OS=Rattus norvegicus GN=Sncb PE=1 SV=1	EGVVAAAEK
>	sp|Q63754|SYl	Beta-synuclein OS=Rattus norvegicus GN=Sncb PE=1 SV=1	EGVVAAAEK
>	sp|Q63754|SYl	Beta-synuclein OS=Rattus norvegicus GN=Sncb PE=1 SV=1	EGVVAAAEK
>	sp|Q63754|SYl	Beta-synuclein OS=Rattus norvegicus GN=Sncb PE=1 SV=1	EGVVQGVASVAEK
>	sp|Q63754|SYl	Beta-synuclein OS=Rattus norvegicus GN=Sncb PE=1 SV=1	EGVVQGVASVAEK
>	sp|Q63754|SYl	Beta-synuclein OS=Rattus norvegicus GN=Sncb PE=1 SV=1	EQASHLGGAVFSGAGNIAAATGLVK
>	sp|Q63754|SYl	Beta-synuclein OS=Rattus norvegicus GN=Sncb PE=1 SV=1	EQASHLGGAVFSGAGNIAAATGLVK
>	sp|Q63754|SYl	Beta-synuclein OS=Rattus norvegicus GN=Sncb PE=1 SV=1	EQASHLGGAVFSGAGNIAAATGLVK
>	sp|Q63754|SYl	Beta-synuclein OS=Rattus norvegicus GN=Sncb PE=1 SV=1	EQASHLGGAVFSGAGNIAAATGLVK
>	sp|Q63754|SYl	Beta-synuclein OS=Rattus norvegicus GN=Sncb PE=1 SV=1	KEEFPTDLKPEEVAQEAAEEPLIEPLMEPEGESYEDSPQEEYQEYEPEA
>	sp|Q63754|SYl	Beta-synuclein OS=Rattus norvegicus GN=Sncb PE=1 SV=1	KEEFPTDLKPEEVAQEAAEEPLIEPLMEPEGESYEDSPQEEYQEYEPEA
>	sp|Q63754|SYl	Beta-synuclein OS=Rattus norvegicus GN=Sncb PE=1 SV=1	KEEFPTDLKPEEVAQEAAEEPLIEPLMEPEGESYEDSPQEEYQEYEPEA
>	sp|Q63754|SYl	Beta-synuclein OS=Rattus norvegicus GN=Sncb PE=1 SV=1	KEEFPTDLKPEEVAQEAAEEPLIEPLMEPEGESYEDSPQEEYQEYEPEA
>	sp|Q63754|SYl	Beta-synuclein OS=Rattus norvegicus GN=Sncb PE=1 SV=1	KEEFPTDLKPEEVAQEAAEEPLIEPLMEPEGESYEDSPQEEYQEYEPEAK
>	sp|Q63754|SYl	Beta-synuclein OS=Rattus norvegicus GN=Sncb PE=1 SV=1	KEEFPTDLKPEEVAQEAAEEPLIEPLMEPEGESYEDSPQEEYQEYEPEAK
>	sp|Q63754|SYl	Beta-synuclein OS=Rattus norvegicus GN=Sncb PE=1 SV=1	KEEFPTDLKPEEVAQEAAEEPLIEPLMEPEGESYEDSPQEEYQEYEPEAKGP
>	sp|Q63754|SYl	Beta-synuclein OS=Rattus norvegicus GN=Sncb PE=1 SV=1	TKEGVLYVGSK
>	sp|Q63754|SYl	Beta-synuclein OS=Rattus norvegicus GN=Sncb PE=1 SV=1	TKEQASHLGGAVFSGAGNIAAATGLVK
>	sp|Q63754|SYl	Beta-synuclein OS=Rattus norvegicus GN=Sncb PE=1 SV=1	TKEQASHLGGAVFSGAGNIAAATGLVK
>	sp|Q63754|SYl	Beta-synuclein OS=Rattus norvegicus GN=Sncb PE=1 SV=1	TKEQASHLGGAVFSGAGNIAAATGLVK
>	sp|Q63754|SYl	Beta-synuclein OS=Rattus norvegicus GN=Sncb PE=1 SV=1	TKEQASHLGGAVFSGAGNIAAATGLVK
>	sp|Q63754|SYl	Beta-synuclein OS=Rattus norvegicus GN=Sncb PE=1 SV=1	KEEFPTDLKPEEVAQEAAEEPLIEPLMEPEGESYEDSPQEEYQEYEPEAKGP
>	sp|Q63754|SYl	Beta-synuclein OS=Rattus norvegicus GN=Sncb PE=1 SV=1	EGVVAAAEK
>	sp|Q63754|SYl	Beta-synuclein OS=Rattus norvegicus GN=Sncb PE=1 SV=1	TKEGVVQGVASVAEK
>	sp|Q63754|SYl	Beta-synuclein OS=Rattus norvegicus GN=Sncb PE=1 SV=1	TKEGVVQGVASVAEK
>	sp|Q63754|SYl	Beta-synuclein OS=Rattus norvegicus GN=Sncb PE=1 SV=1	EGVVQGVASVAEK
>	sp|Q63754|SYl	Beta-synuclein OS=Rattus norvegicus GN=Sncb PE=1 SV=1	QGVTEAAEK
>	sp|Q63754|SYl	Beta-synuclein OS=Rattus norvegicus GN=Sncb PE=1 SV=1	TKEQASHLGGAVFSGAGNIAAATGLVK
>	sp|Q63754|SYl	Beta-synuclein OS=Rattus norvegicus GN=Sncb PE=1 SV=1	TKEGVVQGVASVAEK

>	sp|Q63754|SYU Beta-synuclein OS=Rattus norvegicus GN=Sncb PE=1 SV=1	TKEQASHLGGAVFSGAGNIAAATGLVK
>	sp|Q63754|SYU Beta-synuclein OS=Rattus norvegicus GN=Sncb PE=1 SV=1	KEEFPTDLKPEEVAQEAAEEPLIEPLMEPEGESYEDSPQEEYQEYEPEAK
>	sp|Q63754|SYU Beta-synuclein OS=Rattus norvegicus GN=Sncb PE=1 SV=1	TKQGVTEAAEK
>	sp|Q63754|SYU Beta-synuclein OS=Rattus norvegicus GN=Sncb PE=1 SV=1	KEEFPTDLKPEEVAQEAAEEPLIEPLMEPEGESYEDSPQEEYQEYEPEAK
>	sp|Q63754|SYU Beta-synuclein OS=Rattus norvegicus GN=Sncb PE=1 SV=1	KEEFPTDLKPEEVAQEAAEEPLIEPLMEPEGESYEDSPQEEYQEYEPEAK
>	sp|Q63754|SYU Beta-synuclein OS=Rattus norvegicus GN=Sncb PE=1 SV=1	KEEFPTDLKPEEVAQEAAEEPLIEPLMEPEGESYEDSPQEEYQEYEPEA
>	sp|Q63754|SYU Beta-synuclein OS=Rattus norvegicus GN=Sncb PE=1 SV=1	TKQGVTEAAEK
>	sp|P63329|PP2 Serine/threonine-protein phosphatase 2B catalytic subunit alpha isoform OS=Rattus norvegicus GN=Ppp3ca PE=1 SV=1	AHEAQDAGYR
>	sp|P63329|PP2 Serine/threonine-protein phosphatase 2B catalytic subunit alpha isoform OS=Rattus norvegicus GN=Ppp3ca PE=1 SV=1	GLTPTGMLPSGVLSGGK
>	sp|P63329|PP2 Serine/threonine-protein phosphatase 2B catalytic subunit alpha isoform OS=Rattus norvegicus GN=Ppp3ca PE=1 SV=1	IITEGASILR
>	sp|P63329|PP2 Serine/threonine-protein phosphatase 2B catalytic subunit alpha isoform OS=Rattus norvegicus GN=Ppp3ca PE=1 SV=1	NLLDIDAPVTVCGDIHGQFFDLMK
>	sp|P63329|PP2 Serine/threonine-protein phosphatase 2B catalytic subunit alpha isoform OS=Rattus norvegicus GN=Ppp3ca PE=1 SV=1	QTLQSATVEAIEADEAIK
>	sp|P63329|PP2 Serine/threonine-protein phosphatase 2B catalytic subunit alpha isoform OS=Rattus norvegicus GN=Ppp3ca PE=1 SV=1	VFSVLREESESVLTLK
>	sp|P63329|PP2 Serine/threonine-protein phosphatase 2B catalytic subunit alpha isoform OS=Rattus norvegicus GN=Ppp3ca PE=1 SV=1	VYDACMDAFDCLPLAALMNQQFLCVHGGLSPEINTLDDIR
>	sp|P63329|PP2 Serine/threonine-protein phosphatase 2B catalytic subunit alpha isoform OS=Rattus norvegicus GN=Ppp3ca PE=1 SV=1	FKEPPAYGPMCDILWSDPLEDFGNEK
>	sp|P63329|PP2 Serine/threonine-protein phosphatase 2B catalytic subunit alpha isoform OS=Rattus norvegicus GN=Ppp3ca PE=1 SV=1	EGRLEESVALR
>	sp|P63329|PP2 Serine/threonine-protein phosphatase 2B catalytic subunit alpha isoform OS=Rattus norvegicus GN=Ppp3ca PE=1 SV=1	EGRLEESVALR
>	sp|P63329|PP2 Serine/threonine-protein phosphatase 2B catalytic subunit alpha isoform OS=Rattus norvegicus GN=Ppp3ca PE=1 SV=1	EGRLEESVALR
>	sp|P63329|PP2 Serine/threonine-protein phosphatase 2B catalytic subunit alpha isoform OS=Rattus norvegicus GN=Ppp3ca PE=1 SV=1	EGRLEESVALR
>	sp|P63329|PP2 Serine/threonine-protein phosphatase 2B catalytic subunit alpha isoform OS=Rattus norvegicus GN=Ppp3ca PE=1 SV=1	EGRLEESVALR
>	sp|P63329|PP2 Serine/threonine-protein phosphatase 2B catalytic subunit alpha isoform OS=Rattus norvegicus GN=Ppp3ca PE=1 SV=1	EGRLEESVALR
>	sp|P63329|PP2 Serine/threonine-protein phosphatase 2B catalytic subunit alpha isoform OS=Rattus norvegicus GN=Ppp3ca PE=1 SV=1	FKEPPAYGPMCDILWSDPLEDFGNEK
>	sp|P63329|PP2 Serine/threonine-protein phosphatase 2B catalytic subunit alpha isoform OS=Rattus norvegicus GN=Ppp3ca PE=1 SV=1	GLTPTGMLPSGVLSGGK
>	sp|P63329|PP2 Serine/threonine-protein phosphatase 2B catalytic subunit alpha isoform OS=Rattus norvegicus GN=Ppp3ca PE=1 SV=1	NLLDIDAPVTVCGDIHGQFFDLMK
>	sp|P63329|PP2 Serine/threonine-protein phosphatase 2B catalytic subunit alpha isoform OS=Rattus norvegicus GN=Ppp3ca PE=1 SV=1	NLLDIDAPVTVCGDIHGQFFDLMK
>	sp|P63329|PP2 Serine/threonine-protein phosphatase 2B catalytic subunit alpha isoform OS=Rattus norvegicus GN=Ppp3ca PE=1 SV=1	NLLDIDAPVTVCGDIHGQFFDLMK
>	sp|P63329|PP2 Serine/threonine-protein phosphatase 2B catalytic subunit alpha isoform OS=Rattus norvegicus GN=Ppp3ca PE=1 SV=1	NLLDIDAPVTVCGDIHGQFFDLMK
>	sp|P63329|PP2 Serine/threonine-protein phosphatase 2B catalytic subunit alpha isoform OS=Rattus norvegicus GN=Ppp3ca PE=1 SV=1	NLLDIDAPVTVCGDIHGQFFDLMK
>	sp|P63329|PP2 Serine/threonine-protein phosphatase 2B catalytic subunit alpha isoform OS=Rattus norvegicus GN=Ppp3ca PE=1 SV=1	NLLDIDAPVTVCGDIHGQFFDLMK
>	sp|P63329|PP2 Serine/threonine-protein phosphatase 2B catalytic subunit alpha isoform OS=Rattus norvegicus GN=Ppp3ca PE=1 SV=1	NLLDIDAPVTVCGDIHGQFFDLMK
>	sp|P63329|PP2 Serine/threonine-protein phosphatase 2B catalytic subunit alpha isoform OS=Rattus norvegicus GN=Ppp3ca PE=1 SV=1	NLLDIDAPVTVCGDIHGQFFDLMK
>	sp|P63329|PP2 Serine/threonine-protein phosphatase 2B catalytic subunit alpha isoform OS=Rattus norvegicus GN=Ppp3ca PE=1 SV=1	NLLDIDAPVTVCGDIHGQFFDLMK
>	sp|P63329|PP2 Serine/threonine-protein phosphatase 2B catalytic subunit alpha isoform OS=Rattus norvegicus GN=Ppp3ca PE=1 SV=1	QTLQSATVEAIEADEAIK
>	sp|P63329|PP2 Serine/threonine-protein phosphatase 2B catalytic subunit alpha isoform OS=Rattus norvegicus GN=Ppp3ca PE=1 SV=1	QTLQSATVEAIEADEAIK
>	sp|P63329|PP2 Serine/threonine-protein phosphatase 2B catalytic subunit alpha isoform OS=Rattus norvegicus GN=Ppp3ca PE=1 SV=1	VFSVLREESESVLTLK
>	sp|P63329|PP2 Serine/threonine-protein phosphatase 2B catalytic subunit alpha isoform OS=Rattus norvegicus GN=Ppp3ca PE=1 SV=1	VFSVLREESESVLTLK
>	sp|P63329|PP2 Serine/threonine-protein phosphatase 2B catalytic subunit alpha isoform OS=Rattus norvegicus GN=Ppp3ca PE=1 SV=1	VYDACMDAFDCLPLAALMNQQFLCVHGGLSPEINTLDDIR
>	sp|P63329|PP2 Serine/threonine-protein phosphatase 2B catalytic subunit alpha isoform OS=Rattus norvegicus GN=Ppp3ca PE=1 SV=1	VYDACMDAFDCLPLAALMNQQFLCVHGGLSPEINTLDDIR
>	sp|P63329|PP2 Serine/threonine-protein phosphatase 2B catalytic subunit alpha isoform OS=Rattus norvegicus GN=Ppp3ca PE=1 SV=1	ITSFEEAK
>	sp|P63329|PP2 Serine/threonine-protein phosphatase 2B catalytic subunit alpha isoform OS=Rattus norvegicus GN=Ppp3ca PE=1 SV=1	VFSVLREESESVLTLK
>	sp|P63329|PP2 Serine/threonine-protein phosphatase 2B catalytic subunit alpha isoform OS=Rattus norvegicus GN=Ppp3ca PE=1 SV=1	LFEVGGSPANTR
>	sp|P63329|PP2 Serine/threonine-protein phosphatase 2B catalytic subunit alpha isoform OS=Rattus norvegicus GN=Ppp3ca PE=1 SV=1	IITEGASILR
>	sp|P63329|PP2 Serine/threonine-protein phosphatase 2B catalytic subunit alpha isoform OS=Rattus norvegicus GN=Ppp3ca PE=1 SV=1	VYDACMDAFDCLPLAALMNQQFLCVHGGLSPEINTLDDIR
>	sp|P63329|PP2 Serine/threonine-protein phosphatase 2B catalytic subunit alpha isoform OS=Rattus norvegicus GN=Ppp3ca PE=1 SV=1	LEESVALR
>	sp|P63329|PP2 Serine/threonine-protein phosphatase 2B catalytic subunit alpha isoform OS=Rattus norvegicus GN=Ppp3ca PE=1 SV=1	NLLDIDAPVTVCGDIHGQFFDLMK
>	sp|P63329|PP2 Serine/threonine-protein phosphatase 2B catalytic subunit alpha isoform OS=Rattus norvegicus GN=Ppp3ca PE=1 SV=1	VFSVLREESESVLTLK
>	sp|P63329|PP2 Serine/threonine-protein phosphatase 2B catalytic subunit alpha isoform OS=Rattus norvegicus GN=Ppp3ca PE=1 SV=1	FKEPPAYGPMCDILWSDPLEDFGNEK
>	sp|P63329|PP2 Serine/threonine-protein phosphatase 2B catalytic subunit alpha isoform OS=Rattus norvegicus GN=Ppp3ca PE=1 SV=1	AHEAQDAGYR
>	sp|P63329|PP2 Serine/threonine-protein phosphatase 2B catalytic subunit alpha isoform OS=Rattus norvegicus GN=Ppp3ca PE=1 SV=1	AHEAQDAGYR
>	sp|P63329|PP2 Serine/threonine-protein phosphatase 2B catalytic subunit alpha isoform OS=Rattus norvegicus GN=Ppp3ca PE=1 SV=1	GLTPTGMLPSGVLSGGK
>	sp|P63329|PP2 Serine/threonine-protein phosphatase 2B catalytic subunit alpha isoform OS=Rattus norvegicus GN=Ppp3ca PE=1 SV=1	FKEPPAYGPMCDILWSDPLEDFGNEK
>	sp|P63329|PP2 Serine/threonine-protein phosphatase 2B catalytic subunit alpha isoform OS=Rattus norvegicus GN=Ppp3ca PE=1 SV=1	EESESVLTLK
>	sp|P63329|PP2 Serine/threonine-protein phosphatase 2B catalytic subunit alpha isoform OS=Rattus norvegicus GN=Ppp3ca PE=1 SV=1	LEESVALR
>	sp|P63329|PP2 Serine/threonine-protein phosphatase 2B catalytic subunit alpha isoform OS=Rattus norvegicus GN=Ppp3ca PE=1 SV=1	KSQTTGFPSLITIFSAPNYLDVYNNK
>	sp|Q4QRB4|TB Tubulin beta-3 chain OS=Rattus norvegicus GN=Tubb3 PE=1 SV=1	ALTVPELTQQMFDAK
>	sp|Q4QRB4|TB Tubulin beta-3 chain OS=Rattus norvegicus GN=Tubb3 PE=1 SV=1	EVDEQMLAIQSK
>	sp|Q4QRB4|TB Tubulin beta-3 chain OS=Rattus norvegicus GN=Tubb3 PE=1 SV=1	FWEVISDEHGIDPSGNYVGDSDLQLER
>	sp|Q4QRB4|TB Tubulin beta-3 chain OS=Rattus norvegicus GN=Tubb3 PE=1 SV=1	IMNTFSVVPSPK
>	sp|Q4QRB4|TB Tubulin beta-3 chain OS=Rattus norvegicus GN=Tubb3 PE=1 SV=1	LATPTYGDLNHLVSATMSGVTTSLR
>	sp|Q4QRB4|TB Tubulin beta-3 chain OS=Rattus norvegicus GN=Tubb3 PE=1 SV=1	LHFFMPGFAPLTAR
>	sp|Q4QRB4|TB Tubulin beta-3 chain OS=Rattus norvegicus GN=Tubb3 PE=1 SV=1	SGAFGHLFRPDNFIFGQSGAGNNWAK
>	sp|Q4QRB4|TB Tubulin beta-3 chain OS=Rattus norvegicus GN=Tubb3 PE=1 SV=1	SGAFGHLFRPDNFIFGQSGAGNNWAKGHYT
>	sp|Q4QRB4|TB Tubulin beta-3 chain OS=Rattus norvegicus GN=Tubb3 PE=1 SV=1	VSDTVVEPYNATLSIHQLVENTDETYCIDNEALYDICFR
>	sp|Q4QRB4|TB Tubulin beta-3 chain OS=Rattus norvegicus GN=Tubb3 PE=1 SV=1	PELTQQMFDAK
>	sp|Q4QRB4|TB Tubulin beta-3 chain OS=Rattus norvegicus GN=Tubb3 PE=1 SV=1	SVVPSPKVSDTVVEPYNATLSIHQLVENTDETYCIDNEALYDICFR
>	sp|Q4QRB4|TB Tubulin beta-3 chain OS=Rattus norvegicus GN=Tubb3 PE=1 SV=1	AILVDLEPGTMDSVR
>	sp|Q4QRB4|TB Tubulin beta-3 chain OS=Rattus norvegicus GN=Tubb3 PE=1 SV=1	AILVDLEPGTMDSVR
>	sp|Q4QRB4|TB Tubulin beta-3 chain OS=Rattus norvegicus GN=Tubb3 PE=1 SV=1	AILVDLEPGTMDSVR
>	sp|Q4QRB4|TB Tubulin beta-3 chain OS=Rattus norvegicus GN=Tubb3 PE=1 SV=1	AILVDLEPGTMDSVR
>	sp|Q4QRB4|TB Tubulin beta-3 chain OS=Rattus norvegicus GN=Tubb3 PE=1 SV=1	AILVDLEPGTMDSVR
>	sp|Q4QRB4|TB Tubulin beta-3 chain OS=Rattus norvegicus GN=Tubb3 PE=1 SV=1	AILVDLEPGTMDSVR
>	sp|Q4QRB4|TB Tubulin beta-3 chain OS=Rattus norvegicus GN=Tubb3 PE=1 SV=1	AILVDLEPGTMDSVR
>	sp|Q4QRB4|TB Tubulin beta-3 chain OS=Rattus norvegicus GN=Tubb3 PE=1 SV=1	AILVDLEPGTMDSVR
>	sp|Q4QRB4|TB Tubulin beta-3 chain OS=Rattus norvegicus GN=Tubb3 PE=1 SV=1	AILVDLEPGTMDSVR

>	sp|Q4QRB4|TB Tubulin beta-3 chain OS=Rattus norvegicus GN=Tubb3 PE=1 SV=1	AILVDLEPGTMDSVR
>	sp|Q4QRB4|TB Tubulin beta-3 chain OS=Rattus norvegicus GN=Tubb3 PE=1 SV=1	AILVDLEPGTMDSVR
>	sp|Q4QRB4|TB Tubulin beta-3 chain OS=Rattus norvegicus GN=Tubb3 PE=1 SV=1	ALTVPELTQQMFDAK
>	sp|Q4QRB4|TB Tubulin beta-3 chain OS=Rattus norvegicus GN=Tubb3 PE=1 SV=1	ALTVPELTQQMFDAK
>	sp|Q4QRB4|TB Tubulin beta-3 chain OS=Rattus norvegicus GN=Tubb3 PE=1 SV=1	ALTVPELTQQMFDAK
>	sp|Q4QRB4|TB Tubulin beta-3 chain OS=Rattus norvegicus GN=Tubb3 PE=1 SV=1	ALTVPELTQQMFDAK
>	sp|Q4QRB4|TB Tubulin beta-3 chain OS=Rattus norvegicus GN=Tubb3 PE=1 SV=1	ALTVPELTQQMFDAK
>	sp|Q4QRB4|TB Tubulin beta-3 chain OS=Rattus norvegicus GN=Tubb3 PE=1 SV=1	EIVHIQAGQCGNQIGAK
>	sp|Q4QRB4|TB Tubulin beta-3 chain OS=Rattus norvegicus GN=Tubb3 PE=1 SV=1	EIVHIQAGQCGNQIGAK
>	sp|Q4QRB4|TB Tubulin beta-3 chain OS=Rattus norvegicus GN=Tubb3 PE=1 SV=1	EIVHIQAGQCGNQIGAK
>	sp|Q4QRB4|TB Tubulin beta-3 chain OS=Rattus norvegicus GN=Tubb3 PE=1 SV=1	EIVHIQAGQCGNQIGAK
>	sp|Q4QRB4|TB Tubulin beta-3 chain OS=Rattus norvegicus GN=Tubb3 PE=1 SV=1	EIVHIQAGQCGNQIGAK
>	sp|Q4QRB4|TB Tubulin beta-3 chain OS=Rattus norvegicus GN=Tubb3 PE=1 SV=1	EIVHIQAGQCGNQIGAK
>	sp|Q4QRB4|TB Tubulin beta-3 chain OS=Rattus norvegicus GN=Tubb3 PE=1 SV=1	EIVHIQAGQCGNQIGAK
>	sp|Q4QRB4|TB Tubulin beta-3 chain OS=Rattus norvegicus GN=Tubb3 PE=1 SV=1	EIVHIQAGQCGNQIGAK
>	sp|Q4QRB4|TB Tubulin beta-3 chain OS=Rattus norvegicus GN=Tubb3 PE=1 SV=1	EIVHIQAGQCGNQIGAK
>	sp|Q4QRB4|TB Tubulin beta-3 chain OS=Rattus norvegicus GN=Tubb3 PE=1 SV=1	EIVHIQAGQCGNQIGAK
>	sp|Q4QRB4|TB Tubulin beta-3 chain OS=Rattus norvegicus GN=Tubb3 PE=1 SV=1	EIVHIQAGQCGNQIGAK
>	sp|Q4QRB4|TB Tubulin beta-3 chain OS=Rattus norvegicus GN=Tubb3 PE=1 SV=1	EIVHIQAGQCGNQIGAK
>	sp|Q4QRB4|TB Tubulin beta-3 chain OS=Rattus norvegicus GN=Tubb3 PE=1 SV=1	EIVHIQAGQCGNQIGAK
>	sp|Q4QRB4|TB Tubulin beta-3 chain OS=Rattus norvegicus GN=Tubb3 PE=1 SV=1	EIVHIQAGQCGNQIGAK
>	sp|Q4QRB4|TB Tubulin beta-3 chain OS=Rattus norvegicus GN=Tubb3 PE=1 SV=1	EIVHIQAGQCGNQIGAK
>	sp|Q4QRB4|TB Tubulin beta-3 chain OS=Rattus norvegicus GN=Tubb3 PE=1 SV=1	EIVHIQAGQCGNQIGAK
>	sp|Q4QRB4|TB Tubulin beta-3 chain OS=Rattus norvegicus GN=Tubb3 PE=1 SV=1	EIVHIQAGQCGNQIGAK
>	sp|Q4QRB4|TB Tubulin beta-3 chain OS=Rattus norvegicus GN=Tubb3 PE=1 SV=1	EIVHIQAGQCGNQIGAK
>	sp|Q4QRB4|TB Tubulin beta-3 chain OS=Rattus norvegicus GN=Tubb3 PE=1 SV=1	EIVHIQAGQCGNQIGAK
>	sp|Q4QRB4|TB Tubulin beta-3 chain OS=Rattus norvegicus GN=Tubb3 PE=1 SV=1	EIVHIQAGQCGNQIGAK
>	sp|Q4QRB4|TB Tubulin beta-3 chain OS=Rattus norvegicus GN=Tubb3 PE=1 SV=1	EIVHIQAGQCGNQIGAK
>	sp|Q4QRB4|TB Tubulin beta-3 chain OS=Rattus norvegicus GN=Tubb3 PE=1 SV=1	EIVHIQAGQCGNQIGAK
>	sp|Q4QRB4|TB Tubulin beta-3 chain OS=Rattus norvegicus GN=Tubb3 PE=1 SV=1	EIVHIQAGQCGNQIGAK
>	sp|Q4QRB4|TB Tubulin beta-3 chain OS=Rattus norvegicus GN=Tubb3 PE=1 SV=1	EIVHIQAGQCGNQIGAK
>	sp|Q4QRB4|TB Tubulin beta-3 chain OS=Rattus norvegicus GN=Tubb3 PE=1 SV=1	EIVHIQAGQCGNQIGAK
>	sp|Q4QRB4|TB Tubulin beta-3 chain OS=Rattus norvegicus GN=Tubb3 PE=1 SV=1	EIVHIQAGQCGNQIGAK
>	sp|Q4QRB4|TB Tubulin beta-3 chain OS=Rattus norvegicus GN=Tubb3 PE=1 SV=1	EIVHIQAGQCGNQIGAK
>	sp|Q4QRB4|TB Tubulin beta-3 chain OS=Rattus norvegicus GN=Tubb3 PE=1 SV=1	EVDEQMLAIQSK
>	sp|Q4QRB4|TB Tubulin beta-3 chain OS=Rattus norvegicus GN=Tubb3 PE=1 SV=1	EVDEQMLAIQSK
>	sp|Q4QRB4|TB Tubulin beta-3 chain OS=Rattus norvegicus GN=Tubb3 PE=1 SV=1	EVDEQMLAIQSK
>	sp|Q4QRB4|TB Tubulin beta-3 chain OS=Rattus norvegicus GN=Tubb3 PE=1 SV=1	EVDEQMLAIQSK
>	sp|Q4QRB4|TB Tubulin beta-3 chain OS=Rattus norvegicus GN=Tubb3 PE=1 SV=1	FPGQLNADLR
>	sp|Q4QRB4|TB Tubulin beta-3 chain OS=Rattus norvegicus GN=Tubb3 PE=1 SV=1	FPGQLNADLR
>	sp|Q4QRB4|TB Tubulin beta-3 chain OS=Rattus norvegicus GN=Tubb3 PE=1 SV=1	FPGQLNADLR
>	sp|Q4QRB4|TB Tubulin beta-3 chain OS=Rattus norvegicus GN=Tubb3 PE=1 SV=1	FPGQLNADLR
>	sp|Q4QRB4|TB Tubulin beta-3 chain OS=Rattus norvegicus GN=Tubb3 PE=1 SV=1	FPGQLNADLR
>	sp|Q4QRB4|TB Tubulin beta-3 chain OS=Rattus norvegicus GN=Tubb3 PE=1 SV=1	FPGQLNADLR
>	sp|Q4QRB4|TB Tubulin beta-3 chain OS=Rattus norvegicus GN=Tubb3 PE=1 SV=1	FPGQLNADLRK
>	sp|Q4QRB4|TB Tubulin beta-3 chain OS=Rattus norvegicus GN=Tubb3 PE=1 SV=1	FWEVISDEHGIDPSGNYVGDSDLQLER
>	sp|Q4QRB4|TB Tubulin beta-3 chain OS=Rattus norvegicus GN=Tubb3 PE=1 SV=1	FWEVISDEHGIDPSGNYVGDSDLQLER
>	sp|Q4QRB4|TB Tubulin beta-3 chain OS=Rattus norvegicus GN=Tubb3 PE=1 SV=1	FWEVISDEHGIDPSGNYVGDSDLQLER
>	sp|Q4QRB4|TB Tubulin beta-3 chain OS=Rattus norvegicus GN=Tubb3 PE=1 SV=1	FWEVISDEHGIDPSGNYVGDSDLQLER
>	sp|Q4QRB4|TB Tubulin beta-3 chain OS=Rattus norvegicus GN=Tubb3 PE=1 SV=1	FWEVISDEHGIDPSGNYVGDSDLQLER
>	sp|Q4QRB4|TB Tubulin beta-3 chain OS=Rattus norvegicus GN=Tubb3 PE=1 SV=1	FWEVISDEHGIDPSGNYVGDSDLQLER
>	sp|Q4QRB4|TB Tubulin beta-3 chain OS=Rattus norvegicus GN=Tubb3 PE=1 SV=1	FWEVISDEHGIDPSGNYVGDSDLQLER
>	sp|Q4QRB4|TB Tubulin beta-3 chain OS=Rattus norvegicus GN=Tubb3 PE=1 SV=1	FWEVISDEHGIDPSGNYVGDSDLQLER
>	sp|Q4QRB4|TB Tubulin beta-3 chain OS=Rattus norvegicus GN=Tubb3 PE=1 SV=1	FWEVISDEHGIDPSGNYVGDSDLQLER
>	sp|Q4QRB4|TB Tubulin beta-3 chain OS=Rattus norvegicus GN=Tubb3 PE=1 SV=1	FWEVISDEHGIDPSGNYVGDSDLQLER
>	sp|Q4QRB4|TB Tubulin beta-3 chain OS=Rattus norvegicus GN=Tubb3 PE=1 SV=1	FWEVISDEHGIDPSGNYVGDSDLQLER
>	sp|Q4QRB4|TB Tubulin beta-3 chain OS=Rattus norvegicus GN=Tubb3 PE=1 SV=1	GHYTEGAELVDSVLDVVR
>	sp|Q4QRB4|TB Tubulin beta-3 chain OS=Rattus norvegicus GN=Tubb3 PE=1 SV=1	GHYTEGAELVDSVLDVVR
>	sp|Q4QRB4|TB Tubulin beta-3 chain OS=Rattus norvegicus GN=Tubb3 PE=1 SV=1	GHYTEGAELVDSVLDVVR
>	sp|Q4QRB4|TB Tubulin beta-3 chain OS=Rattus norvegicus GN=Tubb3 PE=1 SV=1	GHYTEGAELVDSVLDVVR
>	sp|Q4QRB4|TB Tubulin beta-3 chain OS=Rattus norvegicus GN=Tubb3 PE=1 SV=1	GHYTEGAELVDSVLDVVR
>	sp|Q4QRB4|TB Tubulin beta-3 chain OS=Rattus norvegicus GN=Tubb3 PE=1 SV=1	GHYTEGAELVDSVLDVVR
>	sp|Q4QRB4|TB Tubulin beta-3 chain OS=Rattus norvegicus GN=Tubb3 PE=1 SV=1	GHYTEGAELVDSVLDVVR
>	sp|Q4QRB4|TB Tubulin beta-3 chain OS=Rattus norvegicus GN=Tubb3 PE=1 SV=1	GHYTEGAELVDSVLDVVR
>	sp|Q4QRB4|TB Tubulin beta-3 chain OS=Rattus norvegicus GN=Tubb3 PE=1 SV=1	GHYTEGAELVDSVLDVVR
>	sp|Q4QRB4|TB Tubulin beta-3 chain OS=Rattus norvegicus GN=Tubb3 PE=1 SV=1	GHYTEGAELVDSVLDVVR
>	sp|Q4QRB4|TB Tubulin beta-3 chain OS=Rattus norvegicus GN=Tubb3 PE=1 SV=1	GHYTEGAELVDSVLDVVR
>	sp|Q4QRB4|TB Tubulin beta-3 chain OS=Rattus norvegicus GN=Tubb3 PE=1 SV=1	GHYTEGAELVDSVLDVVR
>	sp|Q4QRB4|TB Tubulin beta-3 chain OS=Rattus norvegicus GN=Tubb3 PE=1 SV=1	GHYTEGAELVDSVLDVVR
>	sp|Q4QRB4|TB Tubulin beta-3 chain OS=Rattus norvegicus GN=Tubb3 PE=1 SV=1	GHYTEGAELVDSVLDVVR
>	sp|Q4QRB4|TB Tubulin beta-3 chain OS=Rattus norvegicus GN=Tubb3 PE=1 SV=1	GHYTEGAELVDSVLDVVR
>	sp|Q4QRB4|TB Tubulin beta-3 chain OS=Rattus norvegicus GN=Tubb3 PE=1 SV=1	GHYTEGAELVDSVLDVVR
>	sp|Q4QRB4|TB Tubulin beta-3 chain OS=Rattus norvegicus GN=Tubb3 PE=1 SV=1	GHYTEGAELVDSVLDVVR
>	sp|Q4QRB4|TB Tubulin beta-3 chain OS=Rattus norvegicus GN=Tubb3 PE=1 SV=1	GHYTEGAELVDSVLDVVR

>	sp|Q4QRB4|TB Tubulin beta-3 chain OS=Rattus norvegicus GN=Tubb3 PE=1 SV=1	GHYTEGAELVDSVLDVVR
>	sp|Q4QRB4|TB Tubulin beta-3 chain OS=Rattus norvegicus GN=Tubb3 PE=1 SV=1	GHYTEGAELVDSVLDVVR
>	sp|Q4QRB4|TB Tubulin beta-3 chain OS=Rattus norvegicus GN=Tubb3 PE=1 SV=1	GHYTEGAELVDSVLDVVR
>	sp|Q4QRB4|TB Tubulin beta-3 chain OS=Rattus norvegicus GN=Tubb3 PE=1 SV=1	GHYTEGAELVDSVLDVVR
>	sp|Q4QRB4|TB Tubulin beta-3 chain OS=Rattus norvegicus GN=Tubb3 PE=1 SV=1	GHYTEGAELVDSVLDVVR
>	sp|Q4QRB4|TB Tubulin beta-3 chain OS=Rattus norvegicus GN=Tubb3 PE=1 SV=1	GHYTEGAELVDSVLDVVR
>	sp|Q4QRB4|TB Tubulin beta-3 chain OS=Rattus norvegicus GN=Tubb3 PE=1 SV=1	GHYTEGAELVDSVLDVVR
>	sp|Q4QRB4|TB Tubulin beta-3 chain OS=Rattus norvegicus GN=Tubb3 PE=1 SV=1	GHYTEGAELVDSVLDVVR
>	sp|Q4QRB4|TB Tubulin beta-3 chain OS=Rattus norvegicus GN=Tubb3 PE=1 SV=1	GHYTEGAELVDSVLDVVR
>	sp|Q4QRB4|TB Tubulin beta-3 chain OS=Rattus norvegicus GN=Tubb3 PE=1 SV=1	GHYTEGAELVDSVLDVVR
>	sp|Q4QRB4|TB Tubulin beta-3 chain OS=Rattus norvegicus GN=Tubb3 PE=1 SV=1	GHYTEGAELVDSVLDVVR
>	sp|Q4QRB4|TB Tubulin beta-3 chain OS=Rattus norvegicus GN=Tubb3 PE=1 SV=1	GHYTEGAELVDSVLDVVR
>	sp|Q4QRB4|TB Tubulin beta-3 chain OS=Rattus norvegicus GN=Tubb3 PE=1 SV=1	GHYTEGAELVDSVLDVVR
>	sp|Q4QRB4|TB Tubulin beta-3 chain OS=Rattus norvegicus GN=Tubb3 PE=1 SV=1	GHYTEGAELVDSVLDVVR
>	sp|Q4QRB4|TB Tubulin beta-3 chain OS=Rattus norvegicus GN=Tubb3 PE=1 SV=1	GHYTEGAELVDSVLDVVRK
>	sp|Q4QRB4|TB Tubulin beta-3 chain OS=Rattus norvegicus GN=Tubb3 PE=1 SV=1	IMNTFSVVPSPK
>	sp|Q4QRB4|TB Tubulin beta-3 chain OS=Rattus norvegicus GN=Tubb3 PE=1 SV=1	IMNTFSVVPSPK
>	sp|Q4QRB4|TB Tubulin beta-3 chain OS=Rattus norvegicus GN=Tubb3 PE=1 SV=1	KLAVNMVPFPR
>	sp|Q4QRB4|TB Tubulin beta-3 chain OS=Rattus norvegicus GN=Tubb3 PE=1 SV=1	KLAVNMVPFPR
>	sp|Q4QRB4|TB Tubulin beta-3 chain OS=Rattus norvegicus GN=Tubb3 PE=1 SV=1	KLAVNMVPFPR
>	sp|Q4QRB4|TB Tubulin beta-3 chain OS=Rattus norvegicus GN=Tubb3 PE=1 SV=1	KLAVNMVPFPR
>	sp|Q4QRB4|TB Tubulin beta-3 chain OS=Rattus norvegicus GN=Tubb3 PE=1 SV=1	KLAVNMVPFPR
>	sp|Q4QRB4|TB Tubulin beta-3 chain OS=Rattus norvegicus GN=Tubb3 PE=1 SV=1	KLAVNMVPFPR
>	sp|Q4QRB4|TB Tubulin beta-3 chain OS=Rattus norvegicus GN=Tubb3 PE=1 SV=1	KLAVNMVPFPR
>	sp|Q4QRB4|TB Tubulin beta-3 chain OS=Rattus norvegicus GN=Tubb3 PE=1 SV=1	KLAVNMVPFPR
>	sp|Q4QRB4|TB Tubulin beta-3 chain OS=Rattus norvegicus GN=Tubb3 PE=1 SV=1	KLAVNMVPFPR
>	sp|Q4QRB4|TB Tubulin beta-3 chain OS=Rattus norvegicus GN=Tubb3 PE=1 SV=1	KLAVNMVPFPR
>	sp|Q4QRB4|TB Tubulin beta-3 chain OS=Rattus norvegicus GN=Tubb3 PE=1 SV=1	KLAVNMVPFPR
>	sp|Q4QRB4|TB Tubulin beta-3 chain OS=Rattus norvegicus GN=Tubb3 PE=1 SV=1	KLAVNMVPFPR
>	sp|Q4QRB4|TB Tubulin beta-3 chain OS=Rattus norvegicus GN=Tubb3 PE=1 SV=1	KLAVNMVPFPR
>	sp|Q4QRB4|TB Tubulin beta-3 chain OS=Rattus norvegicus GN=Tubb3 PE=1 SV=1	KLAVNMVPFPR
>	sp|Q4QRB4|TB Tubulin beta-3 chain OS=Rattus norvegicus GN=Tubb3 PE=1 SV=1	LATPTYGDLNHLVSATMSGVTTSLR
>	sp|Q4QRB4|TB Tubulin beta-3 chain OS=Rattus norvegicus GN=Tubb3 PE=1 SV=1	LATPTYGDLNHLVSATMSGVTTSLR
>	sp|Q4QRB4|TB Tubulin beta-3 chain OS=Rattus norvegicus GN=Tubb3 PE=1 SV=1	LATPTYGDLNHLVSATMSGVTTSLR
>	sp|Q4QRB4|TB Tubulin beta-3 chain OS=Rattus norvegicus GN=Tubb3 PE=1 SV=1	LAVNMVPFPR
>	sp|Q4QRB4|TB Tubulin beta-3 chain OS=Rattus norvegicus GN=Tubb3 PE=1 SV=1	LAVNMVPFPR
>	sp|Q4QRB4|TB Tubulin beta-3 chain OS=Rattus norvegicus GN=Tubb3 PE=1 SV=1	LAVNMVPFPR
>	sp|Q4QRB4|TB Tubulin beta-3 chain OS=Rattus norvegicus GN=Tubb3 PE=1 SV=1	LAVNMVPFPR
>	sp|Q4QRB4|TB Tubulin beta-3 chain OS=Rattus norvegicus GN=Tubb3 PE=1 SV=1	LAVNMVPFPR
>	sp|Q4QRB4|TB Tubulin beta-3 chain OS=Rattus norvegicus GN=Tubb3 PE=1 SV=1	LAVNMVPFPR
>	sp|Q4QRB4|TB Tubulin beta-3 chain OS=Rattus norvegicus GN=Tubb3 PE=1 SV=1	LAVNMVPFPR
>	sp|Q4QRB4|TB Tubulin beta-3 chain OS=Rattus norvegicus GN=Tubb3 PE=1 SV=1	LAVNMVPFPR
>	sp|Q4QRB4|TB Tubulin beta-3 chain OS=Rattus norvegicus GN=Tubb3 PE=1 SV=1	LHFFMPGFAPLTAR
>	sp|Q4QRB4|TB Tubulin beta-3 chain OS=Rattus norvegicus GN=Tubb3 PE=1 SV=1	LHFFMPGFAPLTAR
>	sp|Q4QRB4|TB Tubulin beta-3 chain OS=Rattus norvegicus GN=Tubb3 PE=1 SV=1	LHFFMPGFAPLTAR
>	sp|Q4QRB4|TB Tubulin beta-3 chain OS=Rattus norvegicus GN=Tubb3 PE=1 SV=1	LHFFMPGFAPLTAR
>	sp|Q4QRB4|TB Tubulin beta-3 chain OS=Rattus norvegicus GN=Tubb3 PE=1 SV=1	LHFFMPGFAPLTAR
>	sp|Q4QRB4|TB Tubulin beta-3 chain OS=Rattus norvegicus GN=Tubb3 PE=1 SV=1	LHFFMPGFAPLTAR
>	sp|Q4QRB4|TB Tubulin beta-3 chain OS=Rattus norvegicus GN=Tubb3 PE=1 SV=1	LHFFMPGFAPLTAR
>	sp|Q4QRB4|TB Tubulin beta-3 chain OS=Rattus norvegicus GN=Tubb3 PE=1 SV=1	LHFFMPGFAPLTAR
>	sp|Q4QRB4|TB Tubulin beta-3 chain OS=Rattus norvegicus GN=Tubb3 PE=1 SV=1	LHFFMPGFAPLTAR
>	sp|Q4QRB4|TB Tubulin beta-3 chain OS=Rattus norvegicus GN=Tubb3 PE=1 SV=1	LHFFMPGFAPLTAR
>	sp|Q4QRB4|TB Tubulin beta-3 chain OS=Rattus norvegicus GN=Tubb3 PE=1 SV=1	LHFFMPGFAPLTAR
>	sp|Q4QRB4|TB Tubulin beta-3 chain OS=Rattus norvegicus GN=Tubb3 PE=1 SV=1	LHFFMPGFAPLTAR
>	sp|Q4QRB4|TB Tubulin beta-3 chain OS=Rattus norvegicus GN=Tubb3 PE=1 SV=1	LHFFMPGFAPLTAR
>	sp|Q4QRB4|TB Tubulin beta-3 chain OS=Rattus norvegicus GN=Tubb3 PE=1 SV=1	LHFFMPGFAPLTAR
>	sp|Q4QRB4|TB Tubulin beta-3 chain OS=Rattus norvegicus GN=Tubb3 PE=1 SV=1	LHFFMPGFAPLTAR
>	sp|Q4QRB4|TB Tubulin beta-3 chain OS=Rattus norvegicus GN=Tubb3 PE=1 SV=1	LHFFMPGFAPLTAR
>	sp|Q4QRB4|TB Tubulin beta-3 chain OS=Rattus norvegicus GN=Tubb3 PE=1 SV=1	LHFFMPGFAPLTAR
>	sp|Q4QRB4|TB Tubulin beta-3 chain OS=Rattus norvegicus GN=Tubb3 PE=1 SV=1	LHFFMPGFAPLTAR
>	sp|Q4QRB4|TB Tubulin beta-3 chain OS=Rattus norvegicus GN=Tubb3 PE=1 SV=1	LHFFMPGFAPLTAR
>	sp|Q4QRB4|TB Tubulin beta-3 chain OS=Rattus norvegicus GN=Tubb3 PE=1 SV=1	LHFFMPGFAPLTAR
>	sp|Q4QRB4|TB Tubulin beta-3 chain OS=Rattus norvegicus GN=Tubb3 PE=1 SV=1	LHFFMPGFAPLTAR
>	sp|Q4QRB4|TB Tubulin beta-3 chain OS=Rattus norvegicus GN=Tubb3 PE=1 SV=1	LHFFMPGFAPLTAR
>	sp|Q4QRB4|TB Tubulin beta-3 chain OS=Rattus norvegicus GN=Tubb3 PE=1 SV=1	LHFFMPGFAPLTAR
>	sp|Q4QRB4|TB Tubulin beta-3 chain OS=Rattus norvegicus GN=Tubb3 PE=1 SV=1	NSSYFVEWIPNNVK
>	sp|Q4QRB4|TB Tubulin beta-3 chain OS=Rattus norvegicus GN=Tubb3 PE=1 SV=1	NSSYFVEWIPNNVK
>	sp|Q4QRB4|TB Tubulin beta-3 chain OS=Rattus norvegicus GN=Tubb3 PE=1 SV=1	NSSYFVEWIPNNVK
>	sp|Q4QRB4|TB Tubulin beta-3 chain OS=Rattus norvegicus GN=Tubb3 PE=1 SV=1	NSSYFVEWIPNNVK
>	sp|Q4QRB4|TB Tubulin beta-3 chain OS=Rattus norvegicus GN=Tubb3 PE=1 SV=1	PGQLNADLR
>	sp|Q4QRB4|TB Tubulin beta-3 chain OS=Rattus norvegicus GN=Tubb3 PE=1 SV=1	PGQLNADLRK
>	sp|Q4QRB4|TB Tubulin beta-3 chain OS=Rattus norvegicus GN=Tubb3 PE=1 SV=1	PGTMDSVR
>	sp|Q4QRB4|TB Tubulin beta-3 chain OS=Rattus norvegicus GN=Tubb3 PE=1 SV=1	PGTMDSVR
>	sp|Q4QRB4|TB Tubulin beta-3 chain OS=Rattus norvegicus GN=Tubb3 PE=1 SV=1	SGAFGHLFRPDNFIFGQSGAGNNWAK

>	sp|Q4QRB4|TB Tubulin beta-3 chain OS=Rattus norvegicus GN=Tubb3 PE=1 SV=1	VSDTVVEPYNATLSIHQLVENTDETYCIDNEALYDICFR
>	sp|Q4QRB4|TB Tubulin beta-3 chain OS=Rattus norvegicus GN=Tubb3 PE=1 SV=1	VSDTVVEPYNATLSIHQLVENTDETYCIDNEALYDICFR
>	sp|Q4QRB4|TB Tubulin beta-3 chain OS=Rattus norvegicus GN=Tubb3 PE=1 SV=1	VSDTVVEPYNATLSIHQLVENTDETYCIDNEALYDICFR
>	sp|Q4QRB4|TB Tubulin beta-3 chain OS=Rattus norvegicus GN=Tubb3 PE=1 SV=1	VSDTVVEPYNATLSIHQLVENTDETYCIDNEALYDICFR
>	sp|Q4QRB4|TB Tubulin beta-3 chain OS=Rattus norvegicus GN=Tubb3 PE=1 SV=1	VSDTVVEPYNATLSIHQLVENTDETYCIDNEALYDICFR
>	sp|Q4QRB4|TB Tubulin beta-3 chain OS=Rattus norvegicus GN=Tubb3 PE=1 SV=1	VSDTVVEPYNATLSIHQLVENTDETYCIDNEALYDICFR
>	sp|Q4QRB4|TB Tubulin beta-3 chain OS=Rattus norvegicus GN=Tubb3 PE=1 SV=1	VSDTVVEPYNATLSIHQLVENTDETYCIDNEALYDICFR
>	sp|Q4QRB4|TB Tubulin beta-3 chain OS=Rattus norvegicus GN=Tubb3 PE=1 SV=1	VSDTVVEPYNATLSIHQLVENTDETYCIDNEALYDICFR
>	sp|Q4QRB4|TB Tubulin beta-3 chain OS=Rattus norvegicus GN=Tubb3 PE=1 SV=1	VSDTVVEPYNATLSIHQLVENTDETYCIDNEALYDICFR
>	sp|Q4QRB4|TB Tubulin beta-3 chain OS=Rattus norvegicus GN=Tubb3 PE=1 SV=1	VSDTVVEPYNATLSIHQLVENTDETYCIDNEALYDICFR
>	sp|Q4QRB4|TB Tubulin beta-3 chain OS=Rattus norvegicus GN=Tubb3 PE=1 SV=1	VSDTVVEPYNATLSIHQLVENTDETYCIDNEALYDICFR
>	sp|Q4QRB4|TB Tubulin beta-3 chain OS=Rattus norvegicus GN=Tubb3 PE=1 SV=1	VSDTVVEPYNATLSIHQLVENTDETYCIDNEALYDICFR
>	sp|Q4QRB4|TB Tubulin beta-3 chain OS=Rattus norvegicus GN=Tubb3 PE=1 SV=1	VSDTVVEPYNATLSIHQLVENTDETYCIDNEALYDICFR
>	sp|Q4QRB4|TB Tubulin beta-3 chain OS=Rattus norvegicus GN=Tubb3 PE=1 SV=1	VSDTVVEPYNATLSIHQLVENTDETYCIDNEALYDICFR
>	sp|Q4QRB4|TB Tubulin beta-3 chain OS=Rattus norvegicus GN=Tubb3 PE=1 SV=1	VSDTVVEPYNATLSIHQLVENTDETYCIDNEALYDICFR
>	sp|Q4QRB4|TB Tubulin beta-3 chain OS=Rattus norvegicus GN=Tubb3 PE=1 SV=1	VSDTVVEPYNATLSIHQLVENTDETYCIDNEALYDICFR
>	sp|Q4QRB4|TB Tubulin beta-3 chain OS=Rattus norvegicus GN=Tubb3 PE=1 SV=1	VSDTVVEPYNATLSIHQLVENTDETYCIDNEALYDICFR
>	sp|Q4QRB4|TB Tubulin beta-3 chain OS=Rattus norvegicus GN=Tubb3 PE=1 SV=1	VSDTVVEPYNATLSIHQLVENTDETYCIDNEALYDICFR
>	sp|Q4QRB4|TB Tubulin beta-3 chain OS=Rattus norvegicus GN=Tubb3 PE=1 SV=1	VSDTVVEPYNATLSIHQLVENTDETYCIDNEALYDICFR
>	sp|Q4QRB4|TB Tubulin beta-3 chain OS=Rattus norvegicus GN=Tubb3 PE=1 SV=1	VSDTVVEPYNATLSIHQLVENTDETYCIDNEALYDICFR
>	sp|Q4QRB4|TB Tubulin beta-3 chain OS=Rattus norvegicus GN=Tubb3 PE=1 SV=1	VSDTVVEPYNATLSIHQLVENTDETYCIDNEALYDICFR
>	sp|Q4QRB4|TB Tubulin beta-3 chain OS=Rattus norvegicus GN=Tubb3 PE=1 SV=1	VSDTVVEPYNATLSIHQLVENTDETYCIDNEALYDICFR
>	sp|Q4QRB4|TB Tubulin beta-3 chain OS=Rattus norvegicus GN=Tubb3 PE=1 SV=1	VSDTVVEPYNATLSIHQLVENTDETYCIDNEALYDICFR
>	sp|Q4QRB4|TB Tubulin beta-3 chain OS=Rattus norvegicus GN=Tubb3 PE=1 SV=1	VSDTVVEPYNATLSIHQLVENTDETYCIDNEALYDICFR
>	sp|Q4QRB4|TB Tubulin beta-3 chain OS=Rattus norvegicus GN=Tubb3 PE=1 SV=1	VSDTVVEPYNATLSIHQLVENTDETYCIDNEALYDICFR
>	sp|Q4QRB4|TB Tubulin beta-3 chain OS=Rattus norvegicus GN=Tubb3 PE=1 SV=1	LHFFMPGFAPLTAR
>	sp|Q4QRB4|TB Tubulin beta-3 chain OS=Rattus norvegicus GN=Tubb3 PE=1 SV=1	NSSYFVEWIPNNVK
>	sp|Q4QRB4|TB Tubulin beta-3 chain OS=Rattus norvegicus GN=Tubb3 PE=1 SV=1	VSDTVVEPYNATLSIHQLVENTDETYCIDNEALYDICFR
>	sp|Q4QRB4|TB Tubulin beta-3 chain OS=Rattus norvegicus GN=Tubb3 PE=1 SV=1	EVDEQMLAIQSK
>	sp|Q4QRB4|TB Tubulin beta-3 chain OS=Rattus norvegicus GN=Tubb3 PE=1 SV=1	EIVHIQAGQCGNQIGAK
>	sp|Q4QRB4|TB Tubulin beta-3 chain OS=Rattus norvegicus GN=Tubb3 PE=1 SV=1	LHFFMPGFAPLTAR
>	sp|Q4QRB4|TB Tubulin beta-3 chain OS=Rattus norvegicus GN=Tubb3 PE=1 SV=1	EIVHIQAGQCGNQIGAK
>	sp|Q4QRB4|TB Tubulin beta-3 chain OS=Rattus norvegicus GN=Tubb3 PE=1 SV=1	DNFIFGQSGAGNNWAKGHYTEGAELVDSVLDVVR
>	sp|Q4QRB4|TB Tubulin beta-3 chain OS=Rattus norvegicus GN=Tubb3 PE=1 SV=1	YLTVATVFR
>	sp|Q4QRB4|TB Tubulin beta-3 chain OS=Rattus norvegicus GN=Tubb3 PE=1 SV=1	FPGQLNADLR
>	sp|Q4QRB4|TB Tubulin beta-3 chain OS=Rattus norvegicus GN=Tubb3 PE=1 SV=1	KLAVNMVPFPR
>	sp|Q4QRB4|TB Tubulin beta-3 chain OS=Rattus norvegicus GN=Tubb3 PE=1 SV=1	NSSYFVEWIPNNVK
>	sp|Q4QRB4|TB Tubulin beta-3 chain OS=Rattus norvegicus GN=Tubb3 PE=1 SV=1	NSSYFVEWIPNNVK
>	sp|Q4QRB4|TB Tubulin beta-3 chain OS=Rattus norvegicus GN=Tubb3 PE=1 SV=1	NSSYFVEWIPNNVK
>	sp|Q4QRB4|TB Tubulin beta-3 chain OS=Rattus norvegicus GN=Tubb3 PE=1 SV=1	VSDTVVEPYNATLSIHQLVENTDETYCIDNEALYDICFR
>	sp|Q4QRB4|TB Tubulin beta-3 chain OS=Rattus norvegicus GN=Tubb3 PE=1 SV=1	LHFFMPGFAPLTAR
>	sp|Q4QRB4|TB Tubulin beta-3 chain OS=Rattus norvegicus GN=Tubb3 PE=1 SV=1	GHYTEGAELVDSVLDVVR
>	sp|Q4QRB4|TB Tubulin beta-3 chain OS=Rattus norvegicus GN=Tubb3 PE=1 SV=1	EIVHIQAGQCGNQIGAK
>	sp|Q4QRB4|TB Tubulin beta-3 chain OS=Rattus norvegicus GN=Tubb3 PE=1 SV=1	GHYTEGAELVDSVLDVVR
>	sp|Q4QRB4|TB Tubulin beta-3 chain OS=Rattus norvegicus GN=Tubb3 PE=1 SV=1	GHYTEGAELVDSVLDVVR
>	sp|Q4QRB4|TB Tubulin beta-3 chain OS=Rattus norvegicus GN=Tubb3 PE=1 SV=1	GHYTEGAELVDSVLDVVR
>	sp|Q4QRB4|TB Tubulin beta-3 chain OS=Rattus norvegicus GN=Tubb3 PE=1 SV=1	FWEVISDEHGIDPSGNYVGDSDLQLER
>	sp|Q4QRB4|TB Tubulin beta-3 chain OS=Rattus norvegicus GN=Tubb3 PE=1 SV=1	VSDTVVEPYNATLSIHQLVENTDETYCIDNEALYDICFR
>	sp|Q4QRB4|TB Tubulin beta-3 chain OS=Rattus norvegicus GN=Tubb3 PE=1 SV=1	AILVDLEPGTMDSVR
>	sp|Q4QRB4|TB Tubulin beta-3 chain OS=Rattus norvegicus GN=Tubb3 PE=1 SV=1	KLAVNMVPFPRLHFFMPGFAPLTAR
>	sp|Q4QRB4|TB Tubulin beta-3 chain OS=Rattus norvegicus GN=Tubb3 PE=1 SV=1	EIVHIQAGQCGNQIGAK
>	sp|Q4QRB4|TB Tubulin beta-3 chain OS=Rattus norvegicus GN=Tubb3 PE=1 SV=1	KLAVNMVPFPR
>	sp|Q4QRB4|TB Tubulin beta-3 chain OS=Rattus norvegicus GN=Tubb3 PE=1 SV=1	LHFFMPGFAPLTAR
>	sp|Q4QRB4|TB Tubulin beta-3 chain OS=Rattus norvegicus GN=Tubb3 PE=1 SV=1	AILVDLEPGTMDSVR
>	sp|Q4QRB4|TB Tubulin beta-3 chain OS=Rattus norvegicus GN=Tubb3 PE=1 SV=1	EIVHIQAGQCGNQIGAK
>	sp|Q4QRB4|TB Tubulin beta-3 chain OS=Rattus norvegicus GN=Tubb3 PE=1 SV=1	EIVHIQAGQCGNQIGAK
>	sp|Q4QRB4|TB Tubulin beta-3 chain OS=Rattus norvegicus GN=Tubb3 PE=1 SV=1	LAVNMVPFPR
>	sp|Q4QRB4|TB Tubulin beta-3 chain OS=Rattus norvegicus GN=Tubb3 PE=1 SV=1	KLAVNMVPFPR
>	sp|Q4QRB4|TB Tubulin beta-3 chain OS=Rattus norvegicus GN=Tubb3 PE=1 SV=1	VSDTVVEPYNATLSIHQLVENTDETYCIDNEALYDICFR
>	sp|Q4QRB4|TB Tubulin beta-3 chain OS=Rattus norvegicus GN=Tubb3 PE=1 SV=1	PGTMDSVR
>	sp|Q4QRB4|TB Tubulin beta-3 chain OS=Rattus norvegicus GN=Tubb3 PE=1 SV=1	EVDEQMLAIQSK
>	sp|Q4QRB4|TB Tubulin beta-3 chain OS=Rattus norvegicus GN=Tubb3 PE=1 SV=1	VSDTVVEPYNATLSIHQLVENTDETYCIDNEALYDICFR
>	sp|Q4QRB4|TB Tubulin beta-3 chain OS=Rattus norvegicus GN=Tubb3 PE=1 SV=1	GHYTEGAELVDSVLDVVR
>	sp|Q4QRB4|TB Tubulin beta-3 chain OS=Rattus norvegicus GN=Tubb3 PE=1 SV=1	AILVDLEPGTMDSVR
>	sp|Q4QRB4|TB Tubulin beta-3 chain OS=Rattus norvegicus GN=Tubb3 PE=1 SV=1	AILVDLEPGTMDSVR
>	sp|Q4QRB4|TB Tubulin beta-3 chain OS=Rattus norvegicus GN=Tubb3 PE=1 SV=1	TFSVVPSPKVSDTVVEPYNATLSIHQLVENTDETYCIDNEALYDICFR
>	sp|Q4QRB4|TB Tubulin beta-3 chain OS=Rattus norvegicus GN=Tubb3 PE=1 SV=1	EIVHIQAGQCGNQIGAK
>	sp|Q4QRB4|TB Tubulin beta-3 chain OS=Rattus norvegicus GN=Tubb3 PE=1 SV=1	VSDTVVEPYNATLSIHQLVENTDETYCIDNEALYDICFR
>	sp|Q4QRB4|TB Tubulin beta-3 chain OS=Rattus norvegicus GN=Tubb3 PE=1 SV=1	KLAVNMVPFPR
>	sp|Q4QRB4|TB Tubulin beta-3 chain OS=Rattus norvegicus GN=Tubb3 PE=1 SV=1	VSDTVVEPYNATLSIHQLVENTDETYCIDNEALYDICFR
>	sp|Q4QRB4|TB Tubulin beta-3 chain OS=Rattus norvegicus GN=Tubb3 PE=1 SV=1	LHFFMPGFAPLTAR
>	sp|Q4QRB4|TB Tubulin beta-3 chain OS=Rattus norvegicus GN=Tubb3 PE=1 SV=1	SGAFGHLFRPDNFIFGQSGAGNNWAK
>	sp|Q4QRB4|TB Tubulin beta-3 chain OS=Rattus norvegicus GN=Tubb3 PE=1 SV=1	FPGQLNADLRK
>	sp|Q4QRB4|TB Tubulin beta-3 chain OS=Rattus norvegicus GN=Tubb3 PE=1 SV=1	VSDTVVEPYNATLSIHQLVENTDETYCIDNEALYDICFR

>	sp|Q4QRB4|TB Tubulin beta-3 chain OS=Rattus norvegicus GN=Tubb3 PE=1 SV=1	VSDTVVEPYNATLSIHQLVENTDETYCIDNEALYDICFR
>	sp|Q4QRB4|TB Tubulin beta-3 chain OS=Rattus norvegicus GN=Tubb3 PE=1 SV=1	LHFFMPGFAPLTAR
>	sp|Q4QRB4|TB Tubulin beta-3 chain OS=Rattus norvegicus GN=Tubb3 PE=1 SV=1	KLAVNMVPFPR
>	sp|Q4QRB4|TB Tubulin beta-3 chain OS=Rattus norvegicus GN=Tubb3 PE=1 SV=1	KLAVNMVPFPR
>	sp|Q4QRB4|TB Tubulin beta-3 chain OS=Rattus norvegicus GN=Tubb3 PE=1 SV=1	LAVNMVPFPR
>	sp|Q4QRB4|TB Tubulin beta-3 chain OS=Rattus norvegicus GN=Tubb3 PE=1 SV=1	KLAVNMVPFPR
>	sp|Q4QRB4|TB Tubulin beta-3 chain OS=Rattus norvegicus GN=Tubb3 PE=1 SV=1	KLAVNMVPFPR
>	sp|Q4QRB4|TB Tubulin beta-3 chain OS=Rattus norvegicus GN=Tubb3 PE=1 SV=1	GHYTEGAELVDSVLDVVR
>	sp|Q4QRB4|TB Tubulin beta-3 chain OS=Rattus norvegicus GN=Tubb3 PE=1 SV=1	KLAVNMVPFPR
>	sp|Q4QRB4|TB Tubulin beta-3 chain OS=Rattus norvegicus GN=Tubb3 PE=1 SV=1	LAVNMVPFPR
>	sp|Q4QRB4|TB Tubulin beta-3 chain OS=Rattus norvegicus GN=Tubb3 PE=1 SV=1	GHYTEGAELVDSVLDVVR
>	sp|Q4QRB4|TB Tubulin beta-3 chain OS=Rattus norvegicus GN=Tubb3 PE=1 SV=1	LHFFMPGFAPLTAR
>	sp|Q4QRB4|TB Tubulin beta-3 chain OS=Rattus norvegicus GN=Tubb3 PE=1 SV=1	EIVHIQAGQCGNQIGAK
>	sp|Q4QRB4|TB Tubulin beta-3 chain OS=Rattus norvegicus GN=Tubb3 PE=1 SV=1	VSDTVVEPYNATLSIHQLVENTDETYCIDNEALYDICFR
>	sp|Q4QRB4|TB Tubulin beta-3 chain OS=Rattus norvegicus GN=Tubb3 PE=1 SV=1	VSDTVVEPYNATLSIHQLVENTDETYCIDNEALYDICFR
>	sp|Q4QRB4|TB Tubulin beta-3 chain OS=Rattus norvegicus GN=Tubb3 PE=1 SV=1	KLAVNMVPFPR
>	sp|Q4QRB4|TB Tubulin beta-3 chain OS=Rattus norvegicus GN=Tubb3 PE=1 SV=1	VSDTVVEPYNATLSIHQLVENTDETYCIDNEALYDICFR
>	sp|Q4QRB4|TB Tubulin beta-3 chain OS=Rattus norvegicus GN=Tubb3 PE=1 SV=1	FWEVISDEHGIDPSGNYVGDSDLQLER
>	sp|Q4QRB4|TB Tubulin beta-3 chain OS=Rattus norvegicus GN=Tubb3 PE=1 SV=1	EIVHIQAGQCGNQIGAK
>	sp|Q4QRB4|TB Tubulin beta-3 chain OS=Rattus norvegicus GN=Tubb3 PE=1 SV=1	ALTVPELTQQMFDAK
>	sp|Q4QRB4|TB Tubulin beta-3 chain OS=Rattus norvegicus GN=Tubb3 PE=1 SV=1	FPGQLNADLR
>	sp|Q4QRB4|TB Tubulin beta-3 chain OS=Rattus norvegicus GN=Tubb3 PE=1 SV=1	AILVDLE
>	sp|Q4QRB4|TB Tubulin beta-3 chain OS=Rattus norvegicus GN=Tubb3 PE=1 SV=1	IMNTFSVVPSPK
>	sp|Q4QRB4|TB Tubulin beta-3 chain OS=Rattus norvegicus GN=Tubb3 PE=1 SV=1	VSDTVVEPYNATLSIHQLVENTDETYCIDNEALYDICFR
>	sp|Q4QRB4|TB Tubulin beta-3 chain OS=Rattus norvegicus GN=Tubb3 PE=1 SV=1	KLAVNMVPFPR
>	sp|Q4QRB4|TB Tubulin beta-3 chain OS=Rattus norvegicus GN=Tubb3 PE=1 SV=1	EIVHIQAGQCGNQIGAK
>	sp|Q4QRB4|TB Tubulin beta-3 chain OS=Rattus norvegicus GN=Tubb3 PE=1 SV=1	NSSYFVEWIPNNVK
>	sp|Q4QRB4|TB Tubulin beta-3 chain OS=Rattus norvegicus GN=Tubb3 PE=1 SV=1	IMNTFSVVPSPK
>	sp|Q4QRB4|TB Tubulin beta-3 chain OS=Rattus norvegicus GN=Tubb3 PE=1 SV=1	NSSYFVEWIPNNVK
>	sp|Q4QRB4|TB Tubulin beta-3 chain OS=Rattus norvegicus GN=Tubb3 PE=1 SV=1	FWEVISDEHGIDPSGNYVGDSDLQLER
>	sp|Q4QRB4|TB Tubulin beta-3 chain OS=Rattus norvegicus GN=Tubb3 PE=1 SV=1	NMMAACDPR
>	sp|Q4QRB4|TB Tubulin beta-3 chain OS=Rattus norvegicus GN=Tubb3 PE=1 SV=1	LHFFMPGFAPLTAR
>	sp|Q4QRB4|TB Tubulin beta-3 chain OS=Rattus norvegicus GN=Tubb3 PE=1 SV=1	VSDTVVEPYNATLSIHQLVENTDETYCIDNEALYDICFR
>	sp|Q4QRB4|TB Tubulin beta-3 chain OS=Rattus norvegicus GN=Tubb3 PE=1 SV=1	FWEVISDEHGIDPSGNYVGDSDLQLER
>	sp|Q4QRB4|TB Tubulin beta-3 chain OS=Rattus norvegicus GN=Tubb3 PE=1 SV=1	NSSYFVEWIPNNVK
>	sp|Q4QRB4|TB Tubulin beta-3 chain OS=Rattus norvegicus GN=Tubb3 PE=1 SV=1	VSDTVVEPYNATLSIHQLVENTDETYCIDNEALYDICFR
>	sp|Q4QRB4|TB Tubulin beta-3 chain OS=Rattus norvegicus GN=Tubb3 PE=1 SV=1	VSDTVVEPYNATLSIHQLVENTDETYCIDNEALYDICFR
>	sp|Q4QRB4|TB Tubulin beta-3 chain OS=Rattus norvegicus GN=Tubb3 PE=1 SV=1	LHFFMPGFAPLTAR
>	sp|Q4QRB4|TB Tubulin beta-3 chain OS=Rattus norvegicus GN=Tubb3 PE=1 SV=1	KLAVNMVPFPR
>	sp|Q4QRB4|TB Tubulin beta-3 chain OS=Rattus norvegicus GN=Tubb3 PE=1 SV=1	ISEQFTAMFR
>	sp|Q4QRB4|TB Tubulin beta-3 chain OS=Rattus norvegicus GN=Tubb3 PE=1 SV=1	LHFFMPGFAPLTAR
>	sp|Q4QRB4|TB Tubulin beta-3 chain OS=Rattus norvegicus GN=Tubb3 PE=1 SV=1	LHFFMPGFAPLTAR
>	sp|Q4QRB4|TB Tubulin beta-3 chain OS=Rattus norvegicus GN=Tubb3 PE=1 SV=1	LHFFMPGFAPLTAR
>	sp|Q4QRB4|TB Tubulin beta-3 chain OS=Rattus norvegicus GN=Tubb3 PE=1 SV=1	EIVHIQAGQCGNQIGAK
>	sp|Q4QRB4|TB Tubulin beta-3 chain OS=Rattus norvegicus GN=Tubb3 PE=1 SV=1	FWEVISDEHGIDPSGNYVGDSDLQLER
>	sp|Q4QRB4|TB Tubulin beta-3 chain OS=Rattus norvegicus GN=Tubb3 PE=1 SV=1	FPGQLNADLR
>	sp|Q4QRB4|TB Tubulin beta-3 chain OS=Rattus norvegicus GN=Tubb3 PE=1 SV=1	VSDTVVEPYNATLSIHQLVENTDETYCIDNEALYDICFR
>	sp|Q4QRB4|TB Tubulin beta-3 chain OS=Rattus norvegicus GN=Tubb3 PE=1 SV=1	VREEYPDR
>	sp|Q4QRB4|TB Tubulin beta-3 chain OS=Rattus norvegicus GN=Tubb3 PE=1 SV=1	KLAVNMVPFPR
>	sp|Q4QRB4|TB Tubulin beta-3 chain OS=Rattus norvegicus GN=Tubb3 PE=1 SV=1	LHFFMPGFAPLTAR
>	sp|Q4QRB4|TB Tubulin beta-3 chain OS=Rattus norvegicus GN=Tubb3 PE=1 SV=1	VSDTVVEPYNATLSIHQLVENTDETYCIDNEALYDICFR
>	sp|Q4QRB4|TB Tubulin beta-3 chain OS=Rattus norvegicus GN=Tubb3 PE=1 SV=1	VSDTVVEPYNATLSIHQLVENTDETYCIDNEALYDICFR
>	sp|Q4QRB4|TB Tubulin beta-3 chain OS=Rattus norvegicus GN=Tubb3 PE=1 SV=1	LATPTYGDLNHLVSATMSGVTTSLR
>	sp|Q4QRB4|TB Tubulin beta-3 chain OS=Rattus norvegicus GN=Tubb3 PE=1 SV=1	EIVHIQAGQCGNQIGAK
>	sp|Q4QRB4|TB Tubulin beta-3 chain OS=Rattus norvegicus GN=Tubb3 PE=1 SV=1	VSDTVVEPYNATLSIHQLVENTDETYCIDNEALYDICFR
>	sp|Q4QRB4|TB Tubulin beta-3 chain OS=Rattus norvegicus GN=Tubb3 PE=1 SV=1	LHFFMPGFAPLTAR
>	sp|Q4QRB4|TB Tubulin beta-3 chain OS=Rattus norvegicus GN=Tubb3 PE=1 SV=1	VSDTVVEPYNATLSIHQLVENTDETYCIDNEALYDICFR
>	sp|Q4QRB4|TB Tubulin beta-3 chain OS=Rattus norvegicus GN=Tubb3 PE=1 SV=1	EIVHIQAGQCGNQIGAK
>	sp|Q4QRB4|TB Tubulin beta-3 chain OS=Rattus norvegicus GN=Tubb3 PE=1 SV=1	KLAVNMVPFPR
>	sp|Q4QRB4|TB Tubulin beta-3 chain OS=Rattus norvegicus GN=Tubb3 PE=1 SV=1	SGAFGHLFRPDNFIFGQSGAGNNWAK
>	sp|Q4QRB4|TB Tubulin beta-3 chain OS=Rattus norvegicus GN=Tubb3 PE=1 SV=1	EIVHIQAGQCGNQIGAK
>	sp|Q4QRB4|TB Tubulin beta-3 chain OS=Rattus norvegicus GN=Tubb3 PE=1 SV=1	LHFFMPGFAPLTAR
>	sp|Q4QRB4|TB Tubulin beta-3 chain OS=Rattus norvegicus GN=Tubb3 PE=1 SV=1	LHFFMPGFAPLTAR
>	sp|Q4QRB4|TB Tubulin beta-3 chain OS=Rattus norvegicus GN=Tubb3 PE=1 SV=1	VSDTVVEPYNATLSIHQLVENTDETYCIDNEALYDICFR
>	sp|Q4QRB4|TB Tubulin beta-3 chain OS=Rattus norvegicus GN=Tubb3 PE=1 SV=1	VSDTVVEPYNATLSIHQLVENTDETYCIDNEALYDICFR
>	sp|Q4QRB4|TB Tubulin beta-3 chain OS=Rattus norvegicus GN=Tubb3 PE=1 SV=1	ALTVPELTQQMFDAK
>	sp|Q4QRB4|TB Tubulin beta-3 chain OS=Rattus norvegicus GN=Tubb3 PE=1 SV=1	FSVVPSPKVSDTVVEPYNATLSIHQLVENTDETYCIDNEALYDICFR
>	sp|Q4QRB4|TB Tubulin beta-3 chain OS=Rattus norvegicus GN=Tubb3 PE=1 SV=1	EIVHIQAGQCGNQIGAK
>	sp|Q4QRB4|TB Tubulin beta-3 chain OS=Rattus norvegicus GN=Tubb3 PE=1 SV=1	VSDTVVEPYNATLSIHQLVENTDETYCIDNEALYDICFR
>	sp|Q4QRB4|TB Tubulin beta-3 chain OS=Rattus norvegicus GN=Tubb3 PE=1 SV=1	FPGQLNADLR
>	sp|Q4QRB4|TB Tubulin beta-3 chain OS=Rattus norvegicus GN=Tubb3 PE=1 SV=1	LHFFMPGFAPLTAR
>	sp|Q4QRB4|TB Tubulin beta-3 chain OS=Rattus norvegicus GN=Tubb3 PE=1 SV=1	KLAVNMVPFPR
>	sp|Q4QRB4|TB Tubulin beta-3 chain OS=Rattus norvegicus GN=Tubb3 PE=1 SV=1	LATPTYGDLNHLVSATMSGVTTSLR
>	sp|Q4QRB4|TB Tubulin beta-3 chain OS=Rattus norvegicus GN=Tubb3 PE=1 SV=1	NSSYFVEWIPNNVK

>	sp|Q4QRB4|TB Tubulin beta-3 chain OS=Rattus norvegicus GN=Tubb3 PE=1 SV=1	KLAVNMVPFPR
>	sp|Q4QRB4|TB Tubulin beta-3 chain OS=Rattus norvegicus GN=Tubb3 PE=1 SV=1	KLAVNMVPFPR
>	sp|Q4QRB4|TB Tubulin beta-3 chain OS=Rattus norvegicus GN=Tubb3 PE=1 SV=1	KLAVNMVPFPR
>	sp|Q4QRB4|TB Tubulin beta-3 chain OS=Rattus norvegicus GN=Tubb3 PE=1 SV=1	LHFFMPGFAPLTAR
>	sp|Q4QRB4|TB Tubulin beta-3 chain OS=Rattus norvegicus GN=Tubb3 PE=1 SV=1	GHYTEGAELVDSVLDVVR
>	sp|Q4QRB4|TB Tubulin beta-3 chain OS=Rattus norvegicus GN=Tubb3 PE=1 SV=1	VSDTVVEPYNATLSIHQLVENTDETYCIDNEALYDICFR
>	sp|P04642|LDH L-lactate dehydrogenase A chain OS=Rattus norvegicus GN=Ldha PE=1 SV=1	DLADELALVDVIEDKLKGEMMDLQHGSLFLK
>	sp|P04642|LDH L-lactate dehydrogenase A chain OS=Rattus norvegicus GN=Ldha PE=1 SV=1	LKGEMMDLQHGSLFLK
>	sp|P04642|LDH L-lactate dehydrogenase A chain OS=Rattus norvegicus GN=Ldha PE=1 SV=1	LKGYTSWAIGLSVADLAESIMKNLR
>	sp|P04642|LDH L-lactate dehydrogenase A chain OS=Rattus norvegicus GN=Ldha PE=1 SV=1	LLIVSNPVDILTYVAWK
>	sp|P04642|LDH L-lactate dehydrogenase A chain OS=Rattus norvegicus GN=Ldha PE=1 SV=1	VTLTPDEEAR
>	sp|P04642|LDH L-lactate dehydrogenase A chain OS=Rattus norvegicus GN=Ldha PE=1 SV=1	LKGYTSWAIGLSVADLAESIMK
>	sp|P04642|LDH L-lactate dehydrogenase A chain OS=Rattus norvegicus GN=Ldha PE=1 SV=1	LKGEMMDLQHGSLFLKTPK
>	sp|P04642|LDH L-lactate dehydrogenase A chain OS=Rattus norvegicus GN=Ldha PE=1 SV=1	SLNPQLGTDADKEQWKDVHK
>	sp|P04642|LDH L-lactate dehydrogenase A chain OS=Rattus norvegicus GN=Ldha PE=1 SV=1	GLYGIKEDVFLSVPCILGQNGISDVVK
>	sp|P04642|LDH L-lactate dehydrogenase A chain OS=Rattus norvegicus GN=Ldha PE=1 SV=1	GYTSWAIGLSVADLAESIMK
>	sp|P04642|LDH L-lactate dehydrogenase A chain OS=Rattus norvegicus GN=Ldha PE=1 SV=1	DLADELALVDVIEDKLKGEMMDLQHGSLFLK
>	sp|P04642|LDH L-lactate dehydrogenase A chain OS=Rattus norvegicus GN=Ldha PE=1 SV=1	GLYGIKEDVFLSVPCILGQNGISDVVK
>	sp|P04642|LDH L-lactate dehydrogenase A chain OS=Rattus norvegicus GN=Ldha PE=1 SV=1	LKGEMMDLQHGSLFLK
>	sp|P04642|LDH L-lactate dehydrogenase A chain OS=Rattus norvegicus GN=Ldha PE=1 SV=1	LKGEMMDLQHGSLFLKTPK
>	sp|P04642|LDH L-lactate dehydrogenase A chain OS=Rattus norvegicus GN=Ldha PE=1 SV=1	LKGEMMDLQHGSLFLKTPK
>	sp|P04642|LDH L-lactate dehydrogenase A chain OS=Rattus norvegicus GN=Ldha PE=1 SV=1	LKGYTSWAIGLSVADLAESIMK
>	sp|P04642|LDH L-lactate dehydrogenase A chain OS=Rattus norvegicus GN=Ldha PE=1 SV=1	LKGYTSWAIGLSVADLAESIMK
>	sp|P04642|LDH L-lactate dehydrogenase A chain OS=Rattus norvegicus GN=Ldha PE=1 SV=1	LLIVSNPVDILTYVAWK
>	sp|P04642|LDH L-lactate dehydrogenase A chain OS=Rattus norvegicus GN=Ldha PE=1 SV=1	LLIVSNPVDILTYVAWK
>	sp|P04642|LDH L-lactate dehydrogenase A chain OS=Rattus norvegicus GN=Ldha PE=1 SV=1	LLIVSNPVDILTYVAWK
>	sp|P04642|LDH L-lactate dehydrogenase A chain OS=Rattus norvegicus GN=Ldha PE=1 SV=1	LLIVSNPVDILTYVAWK
>	sp|P04642|LDH L-lactate dehydrogenase A chain OS=Rattus norvegicus GN=Ldha PE=1 SV=1	LLIVSNPVDILTYVAWK
>	sp|P04642|LDH L-lactate dehydrogenase A chain OS=Rattus norvegicus GN=Ldha PE=1 SV=1	FIIPNVVK
>	sp|P04642|LDH L-lactate dehydrogenase A chain OS=Rattus norvegicus GN=Ldha PE=1 SV=1	VTLTPDEEAR
>	sp|P04642|LDH L-lactate dehydrogenase A chain OS=Rattus norvegicus GN=Ldha PE=1 SV=1	SADTLWGIQK
>	sp|P04642|LDH L-lactate dehydrogenase A chain OS=Rattus norvegicus GN=Ldha PE=1 SV=1	FIIPNVVK
>	sp|P04642|LDH L-lactate dehydrogenase A chain OS=Rattus norvegicus GN=Ldha PE=1 SV=1	FIIPNVVK
>	sp|P04642|LDH L-lactate dehydrogenase A chain OS=Rattus norvegicus GN=Ldha PE=1 SV=1	LVIITAGAR
>	sp|P04642|LDH L-lactate dehydrogenase A chain OS=Rattus norvegicus GN=Ldha PE=1 SV=1	QVVDSAYEVIK
>	sp|P04642|LDH L-lactate dehydrogenase A chain OS=Rattus norvegicus GN=Ldha PE=1 SV=1	FIIPNVVK
>	sp|P25113|PGA Phosphoglycerate mutase 1 OS=Rattus norvegicus GN=Pgam1 PE=1 SV=4	ALPFWNEEIVPQIK
>	sp|P25113|PGA Phosphoglycerate mutase 1 OS=Rattus norvegicus GN=Pgam1 PE=1 SV=4	FSGWYDADLSPAGHEEAK
>	sp|P25113|PGA Phosphoglycerate mutase 1 OS=Rattus norvegicus GN=Pgam1 PE=1 SV=4	HGESAWNLENR
>	sp|P25113|PGA Phosphoglycerate mutase 1 OS=Rattus norvegicus GN=Pgam1 PE=1 SV=4	HLEGLSEEAIMELNLPTGIPIVYELDK
>	sp|P25113|PGA Phosphoglycerate mutase 1 OS=Rattus norvegicus GN=Pgam1 PE=1 SV=4	HLEGLSEEAIMELNLPTGIPIVYELDKNLKPIKPMQFLGDEETVR
>	sp|P25113|PGA Phosphoglycerate mutase 1 OS=Rattus norvegicus GN=Pgam1 PE=1 SV=4	YADLTEDQLPSCESLKDTIAR
>	sp|P25113|PGA Phosphoglycerate mutase 1 OS=Rattus norvegicus GN=Pgam1 PE=1 SV=4	AMEAVAAQGK
>	sp|P25113|PGA Phosphoglycerate mutase 1 OS=Rattus norvegicus GN=Pgam1 PE=1 SV=4	VLIAAHGNSLR
>	sp|P25113|PGA Phosphoglycerate mutase 1 OS=Rattus norvegicus GN=Pgam1 PE=1 SV=4	NLKPIKPMQFLGDEETVRK
>	sp|P25113|PGA Phosphoglycerate mutase 1 OS=Rattus norvegicus GN=Pgam1 PE=1 SV=4	ALPFWNEEIVPQIK
>	sp|P25113|PGA Phosphoglycerate mutase 1 OS=Rattus norvegicus GN=Pgam1 PE=1 SV=4	ALPFWNEEIVPQIK
>	sp|P25113|PGA Phosphoglycerate mutase 1 OS=Rattus norvegicus GN=Pgam1 PE=1 SV=4	FSGWYDADLSPAGHEEAK
>	sp|P25113|PGA Phosphoglycerate mutase 1 OS=Rattus norvegicus GN=Pgam1 PE=1 SV=4	HGESAWNLENR
>	sp|P25113|PGA Phosphoglycerate mutase 1 OS=Rattus norvegicus GN=Pgam1 PE=1 SV=4	HGESAWNLENR
>	sp|P25113|PGA Phosphoglycerate mutase 1 OS=Rattus norvegicus GN=Pgam1 PE=1 SV=4	HLEGLSEEAIMELNLPTGIPIVYELDK
>	sp|P25113|PGA Phosphoglycerate mutase 1 OS=Rattus norvegicus GN=Pgam1 PE=1 SV=4	HLEGLSEEAIMELNLPTGIPIVYELDK
>	sp|P25113|PGA Phosphoglycerate mutase 1 OS=Rattus norvegicus GN=Pgam1 PE=1 SV=4	HLEGLSEEAIMELNLPTGIPIVYELDK
>	sp|P25113|PGA Phosphoglycerate mutase 1 OS=Rattus norvegicus GN=Pgam1 PE=1 SV=4	HLEGLSEEAIMELNLPTGIPIVYELDK
>	sp|P25113|PGA Phosphoglycerate mutase 1 OS=Rattus norvegicus GN=Pgam1 PE=1 SV=4	HLEGLSEEAIMELNLPTGIPIVYELDK
>	sp|P25113|PGA Phosphoglycerate mutase 1 OS=Rattus norvegicus GN=Pgam1 PE=1 SV=4	HLEGLSEEAIMELNLPTGIPIVYELDK
>	sp|P25113|PGA Phosphoglycerate mutase 1 OS=Rattus norvegicus GN=Pgam1 PE=1 SV=4	HLEGLSEEAIMELNLPTGIPIVYELDK
>	sp|P25113|PGA Phosphoglycerate mutase 1 OS=Rattus norvegicus GN=Pgam1 PE=1 SV=4	HLEGLSEEAIMELNLPTGIPIVYELDKNLKPIKPMQFLGDEETVR
>	sp|P25113|PGA Phosphoglycerate mutase 1 OS=Rattus norvegicus GN=Pgam1 PE=1 SV=4	HLEGLSEEAIMELNLPTGIPIVYELDKNLKPIKPMQFLGDEETVR
>	sp|P25113|PGA Phosphoglycerate mutase 1 OS=Rattus norvegicus GN=Pgam1 PE=1 SV=4	HLEGLSEEAIMELNLPTGIPIVYELDKNLKPIKPMQFLGDEETVR
>	sp|P25113|PGA Phosphoglycerate mutase 1 OS=Rattus norvegicus GN=Pgam1 PE=1 SV=4	HLEGLSEEAIMELNLPTGIPIVYELDKNLKPIKPMQFLGDEETVR
>	sp|P25113|PGA Phosphoglycerate mutase 1 OS=Rattus norvegicus GN=Pgam1 PE=1 SV=4	HLEGLSEEAIMELNLPTGIPIVYELDKNLKPIKPMQFLGDEETVR
>	sp|P25113|PGA Phosphoglycerate mutase 1 OS=Rattus norvegicus GN=Pgam1 PE=1 SV=4	HLEGLSEEAIMELNLPTGIPIVYELDKNLKPIKPMQFLGDEETVR
>	sp|P25113|PGA Phosphoglycerate mutase 1 OS=Rattus norvegicus GN=Pgam1 PE=1 SV=4	HLEGLSEEAIMELNLPTGIPIVYELDKNLKPIKPMQFLGDEETVR
>	sp|P25113|PGA Phosphoglycerate mutase 1 OS=Rattus norvegicus GN=Pgam1 PE=1 SV=4	HLEGLSEEAIMELNLPTGIPIVYELDKNLKPIKPMQFLGDEETVR
>	sp|P25113|PGA Phosphoglycerate mutase 1 OS=Rattus norvegicus GN=Pgam1 PE=1 SV=4	YADLTEDQLPSCESLKDTIAR
>	sp|P25113|PGA Phosphoglycerate mutase 1 OS=Rattus norvegicus GN=Pgam1 PE=1 SV=4	YADLTEDQLPSCESLKDTIAR
>	sp|P25113|PGA Phosphoglycerate mutase 1 OS=Rattus norvegicus GN=Pgam1 PE=1 SV=4	YADLTEDQLPSCESLKDTIAR
>	sp|P25113|PGA Phosphoglycerate mutase 1 OS=Rattus norvegicus GN=Pgam1 PE=1 SV=4	YADLTEDQLPSCESLKDTIAR
>	sp|P25113|PGA Phosphoglycerate mutase 1 OS=Rattus norvegicus GN=Pgam1 PE=1 SV=4	HLEGLSEEAIMELNLPTGIPIVYELDK
>	sp|P25113|PGA Phosphoglycerate mutase 1 OS=Rattus norvegicus GN=Pgam1 PE=1 SV=4	YADLTEDQLPSCESLKDTIAR
>	sp|P25113|PGA Phosphoglycerate mutase 1 OS=Rattus norvegicus GN=Pgam1 PE=1 SV=4	VLIAAHGNSLR
>	sp|P25113|PGA Phosphoglycerate mutase 1 OS=Rattus norvegicus GN=Pgam1 PE=1 SV=4	HGESAWNLENR
>	sp|P25113|PGA Phosphoglycerate mutase 1 OS=Rattus norvegicus GN=Pgam1 PE=1 SV=4	ALPFWNEEIVPQIK

>	sp|P25113|PG/	Phosphoglycerate mutase 1 OS=Rattus norvegicus GN=Pgam1 PE=1 SV=4	HLEGLSEEAIMELNLPTGIPIVYELDK
>	sp|P25113|PG/	Phosphoglycerate mutase 1 OS=Rattus norvegicus GN=Pgam1 PE=1 SV=4	KAMEAVAAQGK
>	sp|P25113|PG/	Phosphoglycerate mutase 1 OS=Rattus norvegicus GN=Pgam1 PE=1 SV=4	ALPFWNEEIVPQIK
>	sp|P25113|PG/	Phosphoglycerate mutase 1 OS=Rattus norvegicus GN=Pgam1 PE=1 SV=4	HLEGLSEEAIMELNLPTGIPIVYELDK
>	sp|P25113|PG/	Phosphoglycerate mutase 1 OS=Rattus norvegicus GN=Pgam1 PE=1 SV=4	LVLIRHGESAWNLENR
>	sp|P25113|PG/	Phosphoglycerate mutase 1 OS=Rattus norvegicus GN=Pgam1 PE=1 SV=4	YADLTEDQLPSCESLKDTIAR
>	sp|P25113|PG/	Phosphoglycerate mutase 1 OS=Rattus norvegicus GN=Pgam1 PE=1 SV=4	YADLTEDQLPSCESLKDTIAR
>	sp|P25113|PG/	Phosphoglycerate mutase 1 OS=Rattus norvegicus GN=Pgam1 PE=1 SV=4	VLIAAHGNSLR
>	sp|P25113|PG/	Phosphoglycerate mutase 1 OS=Rattus norvegicus GN=Pgam1 PE=1 SV=4	HLEGLSEEAIMELNLPTGIPIVYELDK
>	sp|P25113|PG/	Phosphoglycerate mutase 1 OS=Rattus norvegicus GN=Pgam1 PE=1 SV=4	DAGYEFDICFTSVQK
>	sp|P25113|PG/	Phosphoglycerate mutase 1 OS=Rattus norvegicus GN=Pgam1 PE=1 SV=4	AETAAK
>	sp|P25113|PG/	Phosphoglycerate mutase 1 OS=Rattus norvegicus GN=Pgam1 PE=1 SV=4	NLKPIKPMQFLGDEETVRK
>	sp|P25113|PG/	Phosphoglycerate mutase 1 OS=Rattus norvegicus GN=Pgam1 PE=1 SV=4	NLKPIKPMQFLGDEETVRK
>	sp|P25113|PG/	Phosphoglycerate mutase 1 OS=Rattus norvegicus GN=Pgam1 PE=1 SV=4	KAMEAVAAQGK
>	sp|P47860|K6F	6-phosphofructokinase type C OS=Rattus norvegicus GN=Pfkp PE=1 SV=2	DLLFKPVAELR
>	sp|P47860|K6F	6-phosphofructokinase type C OS=Rattus norvegicus GN=Pfkp PE=1 SV=2	EWSGLLEELAK
>	sp|P47860|K6F	6-phosphofructokinase type C OS=Rattus norvegicus GN=Pfkp PE=1 SV=2	HAYLNVVGMVGSIDNDFCGTDMTIGTDSALHR
>	sp|P47860|K6F	6-phosphofructokinase type C OS=Rattus norvegicus GN=Pfkp PE=1 SV=2	IIEVVDAIMTTAQSHQR
>	sp|P47860|K6F	6-phosphofructokinase type C OS=Rattus norvegicus GN=Pfkp PE=1 SV=2	KEWSGLLEELAK
>	sp|P47860|K6F	6-phosphofructokinase type C OS=Rattus norvegicus GN=Pfkp PE=1 SV=2	MGVEAVLALLEATPETPACVVSLR
>	sp|P47860|K6F	6-phosphofructokinase type C OS=Rattus norvegicus GN=Pfkp PE=1 SV=2	NVLGHMQQGGAPSPFDR
>	sp|P47860|K6F	6-phosphofructokinase type C OS=Rattus norvegicus GN=Pfkp PE=1 SV=2	IKELVVTNLGFDTR
>	sp|P47860|K6F	6-phosphofructokinase type C OS=Rattus norvegicus GN=Pfkp PE=1 SV=2	KFVSDDSICVLGIQK
>	sp|P47860|K6F	6-phosphofructokinase type C OS=Rattus norvegicus GN=Pfkp PE=1 SV=2	KEWSGLLEELAKNGEIDSDTVKK
>	sp|P47860|K6F	6-phosphofructokinase type C OS=Rattus norvegicus GN=Pfkp PE=1 SV=2	EWSGLLEELAK
>	sp|P47860|K6F	6-phosphofructokinase type C OS=Rattus norvegicus GN=Pfkp PE=1 SV=2	IIEVVDAIMTTAQSHQR
>	sp|P47860|K6F	6-phosphofructokinase type C OS=Rattus norvegicus GN=Pfkp PE=1 SV=2	IIEVVDAIMTTAQSHQR
>	sp|P47860|K6F	6-phosphofructokinase type C OS=Rattus norvegicus GN=Pfkp PE=1 SV=2	IIEVVDAIMTTAQSHQR
>	sp|P47860|K6F	6-phosphofructokinase type C OS=Rattus norvegicus GN=Pfkp PE=1 SV=2	IIEVVDAIMTTAQSHQR
>	sp|P47860|K6F	6-phosphofructokinase type C OS=Rattus norvegicus GN=Pfkp PE=1 SV=2	IKELVVTNLGFDTR
>	sp|P47860|K6F	6-phosphofructokinase type C OS=Rattus norvegicus GN=Pfkp PE=1 SV=2	MGVEAVLALLEATPETPACVVSLR
>	sp|P47860|K6F	6-phosphofructokinase type C OS=Rattus norvegicus GN=Pfkp PE=1 SV=2	KFVSDDSICVLGIQK
>	sp|P47860|K6F	6-phosphofructokinase type C OS=Rattus norvegicus GN=Pfkp PE=1 SV=2	IIEVVDAIMTTAQSHQR
>	sp|P47860|K6F	6-phosphofructokinase type C OS=Rattus norvegicus GN=Pfkp PE=1 SV=2	VTILGHVQR
>	sp|P47860|K6F	6-phosphofructokinase type C OS=Rattus norvegicus GN=Pfkp PE=1 SV=2	EWSGLLEELAK
>	sp|P47860|K6F	6-phosphofructokinase type C OS=Rattus norvegicus GN=Pfkp PE=1 SV=2	EWSGLLEELAK
>	sp|P47860|K6F	6-phosphofructokinase type C OS=Rattus norvegicus GN=Pfkp PE=1 SV=2	KNVLGHMQQGGAPSPFDR
>	sp|P47860|K6F	6-phosphofructokinase type C OS=Rattus norvegicus GN=Pfkp PE=1 SV=2	LAIKEPDDKIPK
>	sp|P47860|K6F	6-phosphofructokinase type C OS=Rattus norvegicus GN=Pfkp PE=1 SV=2	VTILGHVQR
>	sp|P60881|SNF	Synaptosomal-associated protein 25 OS=Rattus norvegicus GN=Snap25 PE=1 SV=1	AWGNNQDGVVASQPAR
>	sp|P60881|SNF	Synaptosomal-associated protein 25 OS=Rattus norvegicus GN=Snap25 PE=1 SV=1	EQMAISGGFIR
>	sp|P60881|SNF	Synaptosomal-associated protein 25 OS=Rattus norvegicus GN=Snap25 PE=1 SV=1	HMALDMGNEIDTQNR
>	sp|P60881|SNF	Synaptosomal-associated protein 25 OS=Rattus norvegicus GN=Snap25 PE=1 SV=1	KAWGNNQDGVVASQPAR
>	sp|P60881|SNF	Synaptosomal-associated protein 25 OS=Rattus norvegicus GN=Snap25 PE=1 SV=1	MLQLVEESK
>	sp|P60881|SNF	Synaptosomal-associated protein 25 OS=Rattus norvegicus GN=Snap25 PE=1 SV=1	RADQLADESLESTR
>	sp|P60881|SNF	Synaptosomal-associated protein 25 OS=Rattus norvegicus GN=Snap25 PE=1 SV=1	TLVMLDEQGEQLER
>	sp|P60881|SNF	Synaptosomal-associated protein 25 OS=Rattus norvegicus GN=Snap25 PE=1 SV=1	VVDEREQMAISGGFIR
>	sp|P60881|SNF	Synaptosomal-associated protein 25 OS=Rattus norvegicus GN=Snap25 PE=1 SV=1	RADQLADESLESTRR
>	sp|P60881|SNF	Synaptosomal-associated protein 25 OS=Rattus norvegicus GN=Snap25 PE=1 SV=1	RVTNDARENEMDENLEQVSGIIGNLR
>	sp|P60881|SNF	Synaptosomal-associated protein 25 OS=Rattus norvegicus GN=Snap25 PE=1 SV=1	AWGNNQDGVVASQPAR
>	sp|P60881|SNF	Synaptosomal-associated protein 25 OS=Rattus norvegicus GN=Snap25 PE=1 SV=1	AWGNNQDGVVASQPAR
>	sp|P60881|SNF	Synaptosomal-associated protein 25 OS=Rattus norvegicus GN=Snap25 PE=1 SV=1	HMALDMGNEIDTQNR
>	sp|P60881|SNF	Synaptosomal-associated protein 25 OS=Rattus norvegicus GN=Snap25 PE=1 SV=1	HMALDMGNEIDTQNR
>	sp|P60881|SNF	Synaptosomal-associated protein 25 OS=Rattus norvegicus GN=Snap25 PE=1 SV=1	HMALDMGNEIDTQNR
>	sp|P60881|SNF	Synaptosomal-associated protein 25 OS=Rattus norvegicus GN=Snap25 PE=1 SV=1	HMALDMGNEIDTQNR
>	sp|P60881|SNF	Synaptosomal-associated protein 25 OS=Rattus norvegicus GN=Snap25 PE=1 SV=1	HMALDMGNEIDTQNR
>	sp|P60881|SNF	Synaptosomal-associated protein 25 OS=Rattus norvegicus GN=Snap25 PE=1 SV=1	HMALDMGNEIDTQNR
>	sp|P60881|SNF	Synaptosomal-associated protein 25 OS=Rattus norvegicus GN=Snap25 PE=1 SV=1	HMALDMGNEIDTQNR
>	sp|P60881|SNF	Synaptosomal-associated protein 25 OS=Rattus norvegicus GN=Snap25 PE=1 SV=1	KAWGNNQDGVVASQPAR
>	sp|P60881|SNF	Synaptosomal-associated protein 25 OS=Rattus norvegicus GN=Snap25 PE=1 SV=1	KAWGNNQDGVVASQPAR
>	sp|P60881|SNF	Synaptosomal-associated protein 25 OS=Rattus norvegicus GN=Snap25 PE=1 SV=1	RADQLADESLESTR
>	sp|P60881|SNF	Synaptosomal-associated protein 25 OS=Rattus norvegicus GN=Snap25 PE=1 SV=1	RADQLADESLESTR
>	sp|P60881|SNF	Synaptosomal-associated protein 25 OS=Rattus norvegicus GN=Snap25 PE=1 SV=1	TLVMLDEQGEQLER
>	sp|P60881|SNF	Synaptosomal-associated protein 25 OS=Rattus norvegicus GN=Snap25 PE=1 SV=1	TLVMLDEQGEQLER
>	sp|P60881|SNF	Synaptosomal-associated protein 25 OS=Rattus norvegicus GN=Snap25 PE=1 SV=1	TLVMLDEQGEQLER
>	sp|P60881|SNF	Synaptosomal-associated protein 25 OS=Rattus norvegicus GN=Snap25 PE=1 SV=1	TLVMLDEQGEQLER
>	sp|P60881|SNF	Synaptosomal-associated protein 25 OS=Rattus norvegicus GN=Snap25 PE=1 SV=1	RVTNDARENEMDENLEQVSGIIGNLR
>	sp|P60881|SNF	Synaptosomal-associated protein 25 OS=Rattus norvegicus GN=Snap25 PE=1 SV=1	RADQLADESLESTR
>	sp|P60881|SNF	Synaptosomal-associated protein 25 OS=Rattus norvegicus GN=Snap25 PE=1 SV=1	MLQLVEESK
>	sp|P60881|SNF	Synaptosomal-associated protein 25 OS=Rattus norvegicus GN=Snap25 PE=1 SV=1	AWGNNQDGVVASQPAR
>	sp|P60881|SNF	Synaptosomal-associated protein 25 OS=Rattus norvegicus GN=Snap25 PE=1 SV=1	EQMAISGGFIR
>	sp|P60881|SNF	Synaptosomal-associated protein 25 OS=Rattus norvegicus GN=Snap25 PE=1 SV=1	KAWGNNQDGVVASQPAR
>	sp|P60881|SNF	Synaptosomal-associated protein 25 OS=Rattus norvegicus GN=Snap25 PE=1 SV=1	MLQLVEESKDAGIR
>	sp|P60881|SNF	Synaptosomal-associated protein 25 OS=Rattus norvegicus GN=Snap25 PE=1 SV=1	KAWGNNQDGVVASQPAR

>	sp|P62815|VA1	V-type proton ATPase subunit B, brain isoform OS=Rattus norvegicus GN=Atp6v1b2 PE=1 SV=1	AVVGEEALTSDDLLYLEFLQK
>	sp|P62815|VA1	V-type proton ATPase subunit B, brain isoform OS=Rattus norvegicus GN=Atp6v1b2 PE=1 SV=1	GPVVLAEDFLDIMGQPINPQCR
>	sp|P62815|VA1	V-type proton ATPase subunit B, brain isoform OS=Rattus norvegicus GN=Atp6v1b2 PE=1 SV=1	HVLVILTDMSSYAEALR
>	sp|P62815|VA1	V-type proton ATPase subunit B, brain isoform OS=Rattus norvegicus GN=Atp6v1b2 PE=1 SV=1	LALTTAEFLAYQCEK
>	sp|P62815|VA1	V-type proton ATPase subunit B, brain isoform OS=Rattus norvegicus GN=Atp6v1b2 PE=1 SV=1	KTSCEFTGDILR
>	sp|P62815|VA1	V-type proton ATPase subunit B, brain isoform OS=Rattus norvegicus GN=Atp6v1b2 PE=1 SV=1	SGQVLEVSGSK
>	sp|P62815|VA1	V-type proton ATPase subunit B, brain isoform OS=Rattus norvegicus GN=Atp6v1b2 PE=1 SV=1	IPIFSAAGLPHNEIAAQICR
>	sp|P62815|VA1	V-type proton ATPase subunit B, brain isoform OS=Rattus norvegicus GN=Atp6v1b2 PE=1 SV=1	YAEIVHLTLPDGTKR
>	sp|P62815|VA1	V-type proton ATPase subunit B, brain isoform OS=Rattus norvegicus GN=Atp6v1b2 PE=1 SV=1	TVSGVNGPLVILDHVK
>	sp|P62815|VA1	V-type proton ATPase subunit B, brain isoform OS=Rattus norvegicus GN=Atp6v1b2 PE=1 SV=1	AVVGEEALTSDDLLYLEFLQK
>	sp|P62815|VA1	V-type proton ATPase subunit B, brain isoform OS=Rattus norvegicus GN=Atp6v1b2 PE=1 SV=1	GPVVLAEDFLDIMGQPINPQCR
>	sp|P62815|VA1	V-type proton ATPase subunit B, brain isoform OS=Rattus norvegicus GN=Atp6v1b2 PE=1 SV=1	IPIFSAAGLPHNEIAAQICR
>	sp|P62815|VA1	V-type proton ATPase subunit B, brain isoform OS=Rattus norvegicus GN=Atp6v1b2 PE=1 SV=1	LALTTAEFLAYQCEK
>	sp|P62815|VA1	V-type proton ATPase subunit B, brain isoform OS=Rattus norvegicus GN=Atp6v1b2 PE=1 SV=1	YAEIVHLTLPDGTKR
>	sp|P62815|VA1	V-type proton ATPase subunit B, brain isoform OS=Rattus norvegicus GN=Atp6v1b2 PE=1 SV=1	YAEIVHLTLPDGTKR
>	sp|P62815|VA1	V-type proton ATPase subunit B, brain isoform OS=Rattus norvegicus GN=Atp6v1b2 PE=1 SV=1	AVVGEEALTSDDLLYLEFLQK
>	sp|P62815|VA1	V-type proton ATPase subunit B, brain isoform OS=Rattus norvegicus GN=Atp6v1b2 PE=1 SV=1	GIVNGAAPELPVPTGGPMAGAR
>	sp|P62815|VA1	V-type proton ATPase subunit B, brain isoform OS=Rattus norvegicus GN=Atp6v1b2 PE=1 SV=1	YAEIVHLTLPDGTKR
>	sp|P62815|VA1	V-type proton ATPase subunit B, brain isoform OS=Rattus norvegicus GN=Atp6v1b2 PE=1 SV=1	SGQVLEVSGSK
>	sp|P62815|VA1	V-type proton ATPase subunit B, brain isoform OS=Rattus norvegicus GN=Atp6v1b2 PE=1 SV=1	YAEIVHLTLPDGTK
>	sp|P62815|VA1	V-type proton ATPase subunit B, brain isoform OS=Rattus norvegicus GN=Atp6v1b2 PE=1 SV=1	LALTTAEFLAYQCEK
>	sp|P62815|VA1	V-type proton ATPase subunit B, brain isoform OS=Rattus norvegicus GN=Atp6v1b2 PE=1 SV=1	KTSCEFTGDILR
>	sp|P62815|VA1	V-type proton ATPase subunit B, brain isoform OS=Rattus norvegicus GN=Atp6v1b2 PE=1 SV=1	QIYPPINVLPSLSR
>	sp|P62815|VA1	V-type proton ATPase subunit B, brain isoform OS=Rattus norvegicus GN=Atp6v1b2 PE=1 SV=1	TVSGVNGPLVILDHVK
>	sp|P62815|VA1	V-type proton ATPase subunit B, brain isoform OS=Rattus norvegicus GN=Atp6v1b2 PE=1 SV=1	IPIFSAAGLPHNEIAAQICR
>	sp|P62815|VA1	V-type proton ATPase subunit B, brain isoform OS=Rattus norvegicus GN=Atp6v1b2 PE=1 SV=1	VFNGSGKPIDR
>	sp|P62815|VA1	V-type proton ATPase subunit B, brain isoform OS=Rattus norvegicus GN=Atp6v1b2 PE=1 SV=1	LALTTAEFLAYQCEK
>	sp|P62815|VA1	V-type proton ATPase subunit B, brain isoform OS=Rattus norvegicus GN=Atp6v1b2 PE=1 SV=1	VFNGSGKPIDR
>	sp|P02091|HBE	Hemoglobin subunit beta-1 OS=Rattus norvegicus GN=Hbb PE=1 SV=3	LLGNMIVIVLGHHLGK
>	sp|P02091|HBE	Hemoglobin subunit beta-1 OS=Rattus norvegicus GN=Hbb PE=1 SV=3	LLVVYPWTQR
>	sp|P02091|HBE	Hemoglobin subunit beta-1 OS=Rattus norvegicus GN=Hbb PE=1 SV=3	VHLTDAEK
>	sp|P02091|HBE	Hemoglobin subunit beta-1 OS=Rattus norvegicus GN=Hbb PE=1 SV=3	VNPDDVGGEALGR
>	sp|P02091|HBE	Hemoglobin subunit beta-1 OS=Rattus norvegicus GN=Hbb PE=1 SV=3	VVAGVASALAHKYH
>	sp|P02091|HBE	Hemoglobin subunit beta-1 OS=Rattus norvegicus GN=Hbb PE=1 SV=3	YFDSFGDLSSASAIMGNPK
>	sp|P02091|HBE	Hemoglobin subunit beta-1 OS=Rattus norvegicus GN=Hbb PE=1 SV=3	VINAFNDGLK
>	sp|P02091|HBE	Hemoglobin subunit beta-1 OS=Rattus norvegicus GN=Hbb PE=1 SV=3	GTFAHLSELHCDKLHVDPENFR
>	sp|P02091|HBE	Hemoglobin subunit beta-1 OS=Rattus norvegicus GN=Hbb PE=1 SV=3	GTFAHLSELHCDKLHVDPENFR
>	sp|P02091|HBE	Hemoglobin subunit beta-1 OS=Rattus norvegicus GN=Hbb PE=1 SV=3	LLVVYPWTQR
>	sp|P02091|HBE	Hemoglobin subunit beta-1 OS=Rattus norvegicus GN=Hbb PE=1 SV=3	VHLTDAEK
>	sp|P02091|HBE	Hemoglobin subunit beta-1 OS=Rattus norvegicus GN=Hbb PE=1 SV=3	VHLTDAEK
>	sp|P02091|HBE	Hemoglobin subunit beta-1 OS=Rattus norvegicus GN=Hbb PE=1 SV=3	VHLTDAEK
>	sp|P02091|HBE	Hemoglobin subunit beta-1 OS=Rattus norvegicus GN=Hbb PE=1 SV=3	VHLTDAEK
>	sp|P02091|HBE	Hemoglobin subunit beta-1 OS=Rattus norvegicus GN=Hbb PE=1 SV=3	VNPDDVGGEALGR
>	sp|P02091|HBE	Hemoglobin subunit beta-1 OS=Rattus norvegicus GN=Hbb PE=1 SV=3	VNPDDVGGEALGR
>	sp|P02091|HBE	Hemoglobin subunit beta-1 OS=Rattus norvegicus GN=Hbb PE=1 SV=3	VVAGVASALAHKYH
>	sp|P02091|HBE	Hemoglobin subunit beta-1 OS=Rattus norvegicus GN=Hbb PE=1 SV=3	YFDSFGDLSSASAIMGNPK
>	sp|P02091|HBE	Hemoglobin subunit beta-1 OS=Rattus norvegicus GN=Hbb PE=1 SV=3	YFDSFGDLSSASAIMGNPK
>	sp|P02091|HBE	Hemoglobin subunit beta-1 OS=Rattus norvegicus GN=Hbb PE=1 SV=3	YFDSFGDLSSASAIMGNPK
>	sp|P02091|HBE	Hemoglobin subunit beta-1 OS=Rattus norvegicus GN=Hbb PE=1 SV=3	YFDSFGDLSSASAIMGNPK
>	sp|P02091|HBE	Hemoglobin subunit beta-1 OS=Rattus norvegicus GN=Hbb PE=1 SV=3	YFDSFGDLSSASAIMGNPK
>	sp|P02091|HBE	Hemoglobin subunit beta-1 OS=Rattus norvegicus GN=Hbb PE=1 SV=3	VINAFNDGLK
>	sp|P02091|HBE	Hemoglobin subunit beta-1 OS=Rattus norvegicus GN=Hbb PE=1 SV=3	VINAFNDGLK
>	sp|P02091|HBE	Hemoglobin subunit beta-1 OS=Rattus norvegicus GN=Hbb PE=1 SV=3	HLDNLK
>	sp|P02091|HBE	Hemoglobin subunit beta-1 OS=Rattus norvegicus GN=Hbb PE=1 SV=3	AAVNGLWGK
>	sp|P02091|HBE	Hemoglobin subunit beta-1 OS=Rattus norvegicus GN=Hbb PE=1 SV=3	AAVNGLWGK
>	sp|P02091|HBE	Hemoglobin subunit beta-1 OS=Rattus norvegicus GN=Hbb PE=1 SV=3	VHLTDAEK
>	sp|P02091|HBE	Hemoglobin subunit beta-1 OS=Rattus norvegicus GN=Hbb PE=1 SV=3	AAVNGLWGK
>	sp|P02091|HBE	Hemoglobin subunit beta-1 OS=Rattus norvegicus GN=Hbb PE=1 SV=3	LLGNMIVIVLGHHLGKEFTPCAQAAFQK
>	sp|P02091|HBE	Hemoglobin subunit beta-1 OS=Rattus norvegicus GN=Hbb PE=1 SV=3	LLVVYPWTQR
>	sp|P02091|HBE	Hemoglobin subunit beta-1 OS=Rattus norvegicus GN=Hbb PE=1 SV=3	KVINAFNDGLK
>	sp|P02091|HBE	Hemoglobin subunit beta-1 OS=Rattus norvegicus GN=Hbb PE=1 SV=3	GTFAHLSELHCDKLHVDPENFR
>	sp|P02091|HBE	Hemoglobin subunit beta-1 OS=Rattus norvegicus GN=Hbb PE=1 SV=3	AAVNGLWGK
>	sp|P02091|HBE	Hemoglobin subunit beta-1 OS=Rattus norvegicus GN=Hbb PE=1 SV=3	LLVVYPWTQR
>	sp|P02091|HBE	Hemoglobin subunit beta-1 OS=Rattus norvegicus GN=Hbb PE=1 SV=3	LLVVYPWTQR
>	sp|P02091|HBE	Hemoglobin subunit beta-1 OS=Rattus norvegicus GN=Hbb PE=1 SV=3	AAVNGLWGK
>	sp|P02091|HBE	Hemoglobin subunit beta-1 OS=Rattus norvegicus GN=Hbb PE=1 SV=3	AAVNGLWGK
>	sp|P02091|HBE	Hemoglobin subunit beta-1 OS=Rattus norvegicus GN=Hbb PE=1 SV=3	AAVNGLWGK
>	sp|Q9Z2L0|VDA	Voltage-dependent anion-selective channel protein 1 OS=Rattus norvegicus GN=Vdac1 PE=1 SV=4	EHINLGCDVDFDIAGPSIR
>	sp|Q9Z2L0|VDA	Voltage-dependent anion-selective channel protein 1 OS=Rattus norvegicus GN=Vdac1 PE=1 SV=4	NVNAGGHK
>	sp|Q9Z2L0|VDA	Voltage-dependent anion-selective channel protein 1 OS=Rattus norvegicus GN=Vdac1 PE=1 SV=4	TDEFQLHTNVNDGTEFGGSIYQK
>	sp|Q9Z2L0|VDA	Voltage-dependent anion-selective channel protein 1 OS=Rattus norvegicus GN=Vdac1 PE=1 SV=4	VNNSSLIGLGYTQTLKPGIK
>	sp|Q9Z2L0|VDA	Voltage-dependent anion-selective channel protein 1 OS=Rattus norvegicus GN=Vdac1 PE=1 SV=4	WNTDNTLGTEITVEDQLAR
>	sp|Q9Z2L0|VDA	Voltage-dependent anion-selective channel protein 1 OS=Rattus norvegicus GN=Vdac1 PE=1 SV=4	TKSENGLEFTSSGSANTETTK
>	sp|Q9Z2L0|VDA	Voltage-dependent anion-selective channel protein 1 OS=Rattus norvegicus GN=Vdac1 PE=1 SV=4	VTQSNFAVGYK

>	sp|Q9Z2L0|VD	Voltage-dependent anion-selective channel protein 1 OS=Rattus norvegicus GN=Vdac1 PE=1 SV=4	KLETAVNLAWTAGNSNTR
>	sp|Q9Z2L0|VD	Voltage-dependent anion-selective channel protein 1 OS=Rattus norvegicus GN=Vdac1 PE=1 SV=4	LTLSALLDGK
>	sp|Q9Z2L0|VD	Voltage-dependent anion-selective channel protein 1 OS=Rattus norvegicus GN=Vdac1 PE=1 SV=4	EHINLGCDVDFDIAGPSIR
>	sp|Q9Z2L0|VD	Voltage-dependent anion-selective channel protein 1 OS=Rattus norvegicus GN=Vdac1 PE=1 SV=4	EHINLGCDVDFDIAGPSIR
>	sp|Q9Z2L0|VD	Voltage-dependent anion-selective channel protein 1 OS=Rattus norvegicus GN=Vdac1 PE=1 SV=4	EHINLGCDVDFDIAGPSIR
>	sp|Q9Z2L0|VD	Voltage-dependent anion-selective channel protein 1 OS=Rattus norvegicus GN=Vdac1 PE=1 SV=4	EHINLGCDVDFDIAGPSIR
>	sp|Q9Z2L0|VD	Voltage-dependent anion-selective channel protein 1 OS=Rattus norvegicus GN=Vdac1 PE=1 SV=4	EHINLGCDVDFDIAGPSIR
>	sp|Q9Z2L0|VD	Voltage-dependent anion-selective channel protein 1 OS=Rattus norvegicus GN=Vdac1 PE=1 SV=4	KLETAVNLAWTAGNSNTR
>	sp|Q9Z2L0|VD	Voltage-dependent anion-selective channel protein 1 OS=Rattus norvegicus GN=Vdac1 PE=1 SV=4	KLETAVNLAWTAGNSNTR
>	sp|Q9Z2L0|VD	Voltage-dependent anion-selective channel protein 1 OS=Rattus norvegicus GN=Vdac1 PE=1 SV=4	NVNAGGHK
>	sp|Q9Z2L0|VD	Voltage-dependent anion-selective channel protein 1 OS=Rattus norvegicus GN=Vdac1 PE=1 SV=4	NVNAGGHK
>	sp|Q9Z2L0|VD	Voltage-dependent anion-selective channel protein 1 OS=Rattus norvegicus GN=Vdac1 PE=1 SV=4	NVNAGGHK
>	sp|Q9Z2L0|VD	Voltage-dependent anion-selective channel protein 1 OS=Rattus norvegicus GN=Vdac1 PE=1 SV=4	TDEFQLHTNVNDGTEFGGSIYQK
>	sp|Q9Z2L0|VD	Voltage-dependent anion-selective channel protein 1 OS=Rattus norvegicus GN=Vdac1 PE=1 SV=4	VNNSSLIGLGYTQTLKPGIK
>	sp|Q9Z2L0|VD	Voltage-dependent anion-selective channel protein 1 OS=Rattus norvegicus GN=Vdac1 PE=1 SV=4	WNTDNTLGTEITVEDQLAR
>	sp|Q9Z2L0|VD	Voltage-dependent anion-selective channel protein 1 OS=Rattus norvegicus GN=Vdac1 PE=1 SV=4	VNNSSLIGLGYTQTLKPGIK
>	sp|Q9Z2L0|VD	Voltage-dependent anion-selective channel protein 1 OS=Rattus norvegicus GN=Vdac1 PE=1 SV=4	KLETAVNLAWTAGNSNTR
>	sp|Q9Z2L0|VD	Voltage-dependent anion-selective channel protein 1 OS=Rattus norvegicus GN=Vdac1 PE=1 SV=4	NVNAGGHK
>	sp|Q9Z2L0|VD	Voltage-dependent anion-selective channel protein 1 OS=Rattus norvegicus GN=Vdac1 PE=1 SV=4	LTLSALLDGK
>	sp|Q9Z2L0|VD	Voltage-dependent anion-selective channel protein 1 OS=Rattus norvegicus GN=Vdac1 PE=1 SV=4	VTQSNFAVGYK
>	sp|Q9Z2L0|VD	Voltage-dependent anion-selective channel protein 1 OS=Rattus norvegicus GN=Vdac1 PE=1 SV=4	VNNSSLIGLGYTQTLKPGIK
>	sp|Q9Z2L0|VD	Voltage-dependent anion-selective channel protein 1 OS=Rattus norvegicus GN=Vdac1 PE=1 SV=4	NVNAGGHK
>	sp|Q9Z2L0|VD	Voltage-dependent anion-selective channel protein 1 OS=Rattus norvegicus GN=Vdac1 PE=1 SV=4	WTEYGLTFTEK
>	sp|Q9Z2L0|VD	Voltage-dependent anion-selective channel protein 1 OS=Rattus norvegicus GN=Vdac1 PE=1 SV=4	VNNSSLIGLGYTQTLKPGIK
>	sp|Q9Z2L0|VD	Voltage-dependent anion-selective channel protein 1 OS=Rattus norvegicus GN=Vdac1 PE=1 SV=4	GALVLGYEGWLAGYQMNFETSK
>	sp|Q9Z2L0|VD	Voltage-dependent anion-selective channel protein 1 OS=Rattus norvegicus GN=Vdac1 PE=1 SV=4	LTLSALLDGK
>	sp|Q9Z2L0|VD	Voltage-dependent anion-selective channel protein 1 OS=Rattus norvegicus GN=Vdac1 PE=1 SV=4	GALVLGYEGWLAGYQMNFETSK
>	sp|Q9Z2L0|VD	Voltage-dependent anion-selective channel protein 1 OS=Rattus norvegicus GN=Vdac1 PE=1 SV=4	YQVDPDACFSAK
>	sp|Q9Z2L0|VD	Voltage-dependent anion-selective channel protein 1 OS=Rattus norvegicus GN=Vdac1 PE=1 SV=4	KLETAVNLAWTAGNSNTR
>	sp|Q9Z2L0|VD	Voltage-dependent anion-selective channel protein 1 OS=Rattus norvegicus GN=Vdac1 PE=1 SV=4	YQVDPDACFSAK
>	sp|Q9Z2L0|VD	Voltage-dependent anion-selective channel protein 1 OS=Rattus norvegicus GN=Vdac1 PE=1 SV=4	LTLSALLDGK
>	sp|Q9Z2L0|VD	Voltage-dependent anion-selective channel protein 1 OS=Rattus norvegicus GN=Vdac1 PE=1 SV=4	VTQSNFAVGYK
>	sp|Q9Z2L0|VD	Voltage-dependent anion-selective channel protein 1 OS=Rattus norvegicus GN=Vdac1 PE=1 SV=4	GYGFGLIK
>	sp|Q9Z2L0|VD	Voltage-dependent anion-selective channel protein 1 OS=Rattus norvegicus GN=Vdac1 PE=1 SV=4	KLETAVNLAWTAGNSNTR
>	sp|Q9Z2L0|VD	Voltage-dependent anion-selective channel protein 1 OS=Rattus norvegicus GN=Vdac1 PE=1 SV=4	VNNSSLIGLGYTQTLKPGIK
>	sp|Q9Z2L0|VD	Voltage-dependent anion-selective channel protein 1 OS=Rattus norvegicus GN=Vdac1 PE=1 SV=4	WTEYGLTFTEK
>	sp|Q9Z2L0|VD	Voltage-dependent anion-selective channel protein 1 OS=Rattus norvegicus GN=Vdac1 PE=1 SV=4	VNNSSLIGLGYTQTLKPGIK
>	sp|P10111|PPI	Peptidyl-prolyl cis-trans isomerase A OS=Rattus norvegicus GN=Ppia PE=1 SV=2	HTGPGILSMANAGPNTNGSQFFICTAK
>	sp|P10111|PPI	Peptidyl-prolyl cis-trans isomerase A OS=Rattus norvegicus GN=Ppia PE=1 SV=2	IIPGFMCQGGDFTR
>	sp|P10111|PPI	Peptidyl-prolyl cis-trans isomerase A OS=Rattus norvegicus GN=Ppia PE=1 SV=2	VCFELFADK
>	sp|P10111|PPI	Peptidyl-prolyl cis-trans isomerase A OS=Rattus norvegicus GN=Ppia PE=1 SV=2	VKEGMSIVEAMER
>	sp|P10111|PPI	Peptidyl-prolyl cis-trans isomerase A OS=Rattus norvegicus GN=Ppia PE=1 SV=2	VNPTVFFDITADGEPLGR
>	sp|P10111|PPI	Peptidyl-prolyl cis-trans isomerase A OS=Rattus norvegicus GN=Ppia PE=1 SV=2	VCFELFADKVPK
>	sp|P10111|PPI	Peptidyl-prolyl cis-trans isomerase A OS=Rattus norvegicus GN=Ppia PE=1 SV=2	HTGPGILSMANAGPNTNGSQFFICTAK
>	sp|P10111|PPI	Peptidyl-prolyl cis-trans isomerase A OS=Rattus norvegicus GN=Ppia PE=1 SV=2	HTGPGILSMANAGPNTNGSQFFICTAK
>	sp|P10111|PPI	Peptidyl-prolyl cis-trans isomerase A OS=Rattus norvegicus GN=Ppia PE=1 SV=2	HTGPGILSMANAGPNTNGSQFFICTAK
>	sp|P10111|PPI	Peptidyl-prolyl cis-trans isomerase A OS=Rattus norvegicus GN=Ppia PE=1 SV=2	HTGPGILSMANAGPNTNGSQFFICTAK
>	sp|P10111|PPI	Peptidyl-prolyl cis-trans isomerase A OS=Rattus norvegicus GN=Ppia PE=1 SV=2	HTGPGILSMANAGPNTNGSQFFICTAK
>	sp|P10111|PPI	Peptidyl-prolyl cis-trans isomerase A OS=Rattus norvegicus GN=Ppia PE=1 SV=2	HTGPGILSMANAGPNTNGSQFFICTAK
>	sp|P10111|PPI	Peptidyl-prolyl cis-trans isomerase A OS=Rattus norvegicus GN=Ppia PE=1 SV=2	HTGPGILSMANAGPNTNGSQFFICTAK
>	sp|P10111|PPI	Peptidyl-prolyl cis-trans isomerase A OS=Rattus norvegicus GN=Ppia PE=1 SV=2	HTGPGILSMANAGPNTNGSQFFICTAK
>	sp|P10111|PPI	Peptidyl-prolyl cis-trans isomerase A OS=Rattus norvegicus GN=Ppia PE=1 SV=2	HTGPGILSMANAGPNTNGSQFFICTAK
>	sp|P10111|PPI	Peptidyl-prolyl cis-trans isomerase A OS=Rattus norvegicus GN=Ppia PE=1 SV=2	HTGPGILSMANAGPNTNGSQFFICTAK
>	sp|P10111|PPI	Peptidyl-prolyl cis-trans isomerase A OS=Rattus norvegicus GN=Ppia PE=1 SV=2	HTGPGILSMANAGPNTNGSQFFICTAK
>	sp|P10111|PPI	Peptidyl-prolyl cis-trans isomerase A OS=Rattus norvegicus GN=Ppia PE=1 SV=2	HTGPGILSMANAGPNTNGSQFFICTAK
>	sp|P10111|PPI	Peptidyl-prolyl cis-trans isomerase A OS=Rattus norvegicus GN=Ppia PE=1 SV=2	HTGPGILSMANAGPNTNGSQFFICTAK
>	sp|P10111|PPI	Peptidyl-prolyl cis-trans isomerase A OS=Rattus norvegicus GN=Ppia PE=1 SV=2	HTGPGILSMANAGPNTNGSQFFICTAK
>	sp|P10111|PPI	Peptidyl-prolyl cis-trans isomerase A OS=Rattus norvegicus GN=Ppia PE=1 SV=2	HTGPGILSMANAGPNTNGSQFFICTAK
>	sp|P10111|PPI	Peptidyl-prolyl cis-trans isomerase A OS=Rattus norvegicus GN=Ppia PE=1 SV=2	HTGPGILSMANAGPNTNGSQFFICTAK
>	sp|P10111|PPI	Peptidyl-prolyl cis-trans isomerase A OS=Rattus norvegicus GN=Ppia PE=1 SV=2	HTGPGILSMANAGPNTNGSQFFICTAK
>	sp|P10111|PPI	Peptidyl-prolyl cis-trans isomerase A OS=Rattus norvegicus GN=Ppia PE=1 SV=2	HTGPGILSMANAGPNTNGSQFFICTAK
>	sp|P10111|PPI	Peptidyl-prolyl cis-trans isomerase A OS=Rattus norvegicus GN=Ppia PE=1 SV=2	IIPGFMCQGGDFTR
>	sp|P10111|PPI	Peptidyl-prolyl cis-trans isomerase A OS=Rattus norvegicus GN=Ppia PE=1 SV=2	IIPGFMCQGGDFTR
>	sp|P10111|PPI	Peptidyl-prolyl cis-trans isomerase A OS=Rattus norvegicus GN=Ppia PE=1 SV=2	IIPGFMCQGGDFTR
>	sp|P10111|PPI	Peptidyl-prolyl cis-trans isomerase A OS=Rattus norvegicus GN=Ppia PE=1 SV=2	VCFELFADKVPK
>	sp|P10111|PPI	Peptidyl-prolyl cis-trans isomerase A OS=Rattus norvegicus GN=Ppia PE=1 SV=2	VKEGMSIVEAMER
>	sp|P10111|PPI	Peptidyl-prolyl cis-trans isomerase A OS=Rattus norvegicus GN=Ppia PE=1 SV=2	VKEGMSIVEAMER
>	sp|P10111|PPI	Peptidyl-prolyl cis-trans isomerase A OS=Rattus norvegicus GN=Ppia PE=1 SV=2	VKEGMSIVEAMER
>	sp|P10111|PPI	Peptidyl-prolyl cis-trans isomerase A OS=Rattus norvegicus GN=Ppia PE=1 SV=2	VKEGMSIVEAMER
>	sp|P10111|PPI	Peptidyl-prolyl cis-trans isomerase A OS=Rattus norvegicus GN=Ppia PE=1 SV=2	VKEGMSIVEAMER
>	sp|P10111|PPI	Peptidyl-prolyl cis-trans isomerase A OS=Rattus norvegicus GN=Ppia PE=1 SV=2	VKEGMSIVEAMER
>	sp|P10111|PPI	Peptidyl-prolyl cis-trans isomerase A OS=Rattus norvegicus GN=Ppia PE=1 SV=2	VKEGMSIVEAMER
>	sp|P10111|PPI	Peptidyl-prolyl cis-trans isomerase A OS=Rattus norvegicus GN=Ppia PE=1 SV=2	VKEGMSIVEAMER
>	sp|P10111|PPI	Peptidyl-prolyl cis-trans isomerase A OS=Rattus norvegicus GN=Ppia PE=1 SV=2	VKEGMSIVEAMER

>	sp|P10111|PPI	Peptidyl-prolyl cis-trans isomerase A OS=Rattus norvegicus GN=Ppia PE=1 SV=2	VKEGMSIVEAMER
>	sp|P10111|PPI	Peptidyl-prolyl cis-trans isomerase A OS=Rattus norvegicus GN=Ppia PE=1 SV=2	VNPTVFFDITADGEPLGR
>	sp|P10111|PPI	Peptidyl-prolyl cis-trans isomerase A OS=Rattus norvegicus GN=Ppia PE=1 SV=2	VNPTVFFDITADGEPLGR
>	sp|P10111|PPI	Peptidyl-prolyl cis-trans isomerase A OS=Rattus norvegicus GN=Ppia PE=1 SV=2	VCFELFADKVPK
>	sp|P10111|PPI	Peptidyl-prolyl cis-trans isomerase A OS=Rattus norvegicus GN=Ppia PE=1 SV=2	EGMSIVEAMER
>	sp|P10111|PPI	Peptidyl-prolyl cis-trans isomerase A OS=Rattus norvegicus GN=Ppia PE=1 SV=2	IIPGFMCQGGDFTR
>	sp|P10111|PPI	Peptidyl-prolyl cis-trans isomerase A OS=Rattus norvegicus GN=Ppia PE=1 SV=2	VNPTVFFDITADGEPLGR
>	sp|P10111|PPI	Peptidyl-prolyl cis-trans isomerase A OS=Rattus norvegicus GN=Ppia PE=1 SV=2	IIPGFMCQGGDFTR
>	sp|P10111|PPI	Peptidyl-prolyl cis-trans isomerase A OS=Rattus norvegicus GN=Ppia PE=1 SV=2	HTGPGILSMANAGPNTNGSQFFICTAK
>	sp|P10111|PPI	Peptidyl-prolyl cis-trans isomerase A OS=Rattus norvegicus GN=Ppia PE=1 SV=2	HTGPGILSMANAGPNTNGSQFFICTAK
>	sp|P10111|PPI	Peptidyl-prolyl cis-trans isomerase A OS=Rattus norvegicus GN=Ppia PE=1 SV=2	HTGPGILSMANAGPNTNGSQFFICTAK
>	sp|P10111|PPI	Peptidyl-prolyl cis-trans isomerase A OS=Rattus norvegicus GN=Ppia PE=1 SV=2	SIYGEKFEDENFILK
>	sp|P10111|PPI	Peptidyl-prolyl cis-trans isomerase A OS=Rattus norvegicus GN=Ppia PE=1 SV=2	TEWLDGK
>	sp|P10111|PPI	Peptidyl-prolyl cis-trans isomerase A OS=Rattus norvegicus GN=Ppia PE=1 SV=2	HTGPGILSMANAGPNTNGSQFFICTAK
>	sp|P10111|PPI	Peptidyl-prolyl cis-trans isomerase A OS=Rattus norvegicus GN=Ppia PE=1 SV=2	KITISDCGQL
>	sp|P10111|PPI	Peptidyl-prolyl cis-trans isomerase A OS=Rattus norvegicus GN=Ppia PE=1 SV=2	TEWLDGK
>	sp|P10111|PPI	Peptidyl-prolyl cis-trans isomerase A OS=Rattus norvegicus GN=Ppia PE=1 SV=2	VKEGMSIVEAMER
>	sp|P10111|PPI	Peptidyl-prolyl cis-trans isomerase A OS=Rattus norvegicus GN=Ppia PE=1 SV=2	VCFELFADK
>	sp|P10111|PPI	Peptidyl-prolyl cis-trans isomerase A OS=Rattus norvegicus GN=Ppia PE=1 SV=2	HTGPGILSMANAGPNTNGSQFFICTAK
>	sp|P10111|PPI	Peptidyl-prolyl cis-trans isomerase A OS=Rattus norvegicus GN=Ppia PE=1 SV=2	VKEGMSIVEAMER
>	sp|P10111|PPI	Peptidyl-prolyl cis-trans isomerase A OS=Rattus norvegicus GN=Ppia PE=1 SV=2	HTGPGILSMANAGPNTNGSQFFICTAK
>	sp|P10111|PPI	Peptidyl-prolyl cis-trans isomerase A OS=Rattus norvegicus GN=Ppia PE=1 SV=2	VCFELFADKVPK
>	sp|P10111|PPI	Peptidyl-prolyl cis-trans isomerase A OS=Rattus norvegicus GN=Ppia PE=1 SV=2	VCFELFADK
>	sp|P10111|PPI	Peptidyl-prolyl cis-trans isomerase A OS=Rattus norvegicus GN=Ppia PE=1 SV=2	FEDENFILK
>	sp|P10111|PPI	Peptidyl-prolyl cis-trans isomerase A OS=Rattus norvegicus GN=Ppia PE=1 SV=2	KITISDCGQL
>	sp|P10111|PPI	Peptidyl-prolyl cis-trans isomerase A OS=Rattus norvegicus GN=Ppia PE=1 SV=2	VCFELFADKVPK
>	sp|P10111|PPI	Peptidyl-prolyl cis-trans isomerase A OS=Rattus norvegicus GN=Ppia PE=1 SV=2	FEDENFILK
>	sp|P10111|PPI	Peptidyl-prolyl cis-trans isomerase A OS=Rattus norvegicus GN=Ppia PE=1 SV=2	HTGPGILSMANAGPNTNGSQFFICTAK
>	sp|P10111|PPI	Peptidyl-prolyl cis-trans isomerase A OS=Rattus norvegicus GN=Ppia PE=1 SV=2	IIPGFMCQGGDFTR
>	sp|P10111|PPI	Peptidyl-prolyl cis-trans isomerase A OS=Rattus norvegicus GN=Ppia PE=1 SV=2	EGMSIVEAMER
>	sp|P10111|PPI	Peptidyl-prolyl cis-trans isomerase A OS=Rattus norvegicus GN=Ppia PE=1 SV=2	HTGPGILSMANAGPNTNGSQFFICTAK
>	sp|P10111|PPI	Peptidyl-prolyl cis-trans isomerase A OS=Rattus norvegicus GN=Ppia PE=1 SV=2	VCFELFADK
>	sp|P10111|PPI	Peptidyl-prolyl cis-trans isomerase A OS=Rattus norvegicus GN=Ppia PE=1 SV=2	VCFELFADKVPK
>	sp|P10111|PPI	Peptidyl-prolyl cis-trans isomerase A OS=Rattus norvegicus GN=Ppia PE=1 SV=2	VCFELFADK
>	sp|P10111|PPI	Peptidyl-prolyl cis-trans isomerase A OS=Rattus norvegicus GN=Ppia PE=1 SV=2	TEWLDGK
>	sp|P10111|PPI	Peptidyl-prolyl cis-trans isomerase A OS=Rattus norvegicus GN=Ppia PE=1 SV=2	VCFELFADK
>	sp|P10111|PPI	Peptidyl-prolyl cis-trans isomerase A OS=Rattus norvegicus GN=Ppia PE=1 SV=2	VNPTVFFDITADGEPLGR
>	sp|P13221|AAT	Aspartate aminotransferase, cytoplasmic OS=Rattus norvegicus GN=Got1 PE=1 SV=3	APPSFFAQVPQAPPVLVFK
>	sp|P13221|AAT	Aspartate aminotransferase, cytoplasmic OS=Rattus norvegicus GN=Got1 PE=1 SV=3	IANDHSLNHEYLPILGLAEFR
>	sp|P13221|AAT	Aspartate aminotransferase, cytoplasmic OS=Rattus norvegicus GN=Got1 PE=1 SV=3	NLDYVATSINEAVTK
>	sp|P13221|AAT	Aspartate aminotransferase, cytoplasmic OS=Rattus norvegicus GN=Got1 PE=1 SV=3	SCASQLVLGDNSPALR
>	sp|P13221|AAT	Aspartate aminotransferase, cytoplasmic OS=Rattus norvegicus GN=Got1 PE=1 SV=3	TDDSQPWVLPVVR
>	sp|P13221|AAT	Aspartate aminotransferase, cytoplasmic OS=Rattus norvegicus GN=Got1 PE=1 SV=3	VGGVQSLGGTGALR
>	sp|P13221|AAT	Aspartate aminotransferase, cytoplasmic OS=Rattus norvegicus GN=Got1 PE=1 SV=3	VGNLTVVGK
>	sp|P13221|AAT	Aspartate aminotransferase, cytoplasmic OS=Rattus norvegicus GN=Got1 PE=1 SV=3	APPSFFAQVPQAPPVLVFK
>	sp|P13221|AAT	Aspartate aminotransferase, cytoplasmic OS=Rattus norvegicus GN=Got1 PE=1 SV=3	IANDHSLNHEYLPILGLAEFR
>	sp|P13221|AAT	Aspartate aminotransferase, cytoplasmic OS=Rattus norvegicus GN=Got1 PE=1 SV=3	IANDHSLNHEYLPILGLAEFR
>	sp|P13221|AAT	Aspartate aminotransferase, cytoplasmic OS=Rattus norvegicus GN=Got1 PE=1 SV=3	IANDHSLNHEYLPILGLAEFR
>	sp|P13221|AAT	Aspartate aminotransferase, cytoplasmic OS=Rattus norvegicus GN=Got1 PE=1 SV=3	IANDHSLNHEYLPILGLAEFR
>	sp|P13221|AAT	Aspartate aminotransferase, cytoplasmic OS=Rattus norvegicus GN=Got1 PE=1 SV=3	IANDHSLNHEYLPILGLAEFR
>	sp|P13221|AAT	Aspartate aminotransferase, cytoplasmic OS=Rattus norvegicus GN=Got1 PE=1 SV=3	IANDHSLNHEYLPILGLAEFR
>	sp|P13221|AAT	Aspartate aminotransferase, cytoplasmic OS=Rattus norvegicus GN=Got1 PE=1 SV=3	SCASQLVLGDNSPALR
>	sp|P13221|AAT	Aspartate aminotransferase, cytoplasmic OS=Rattus norvegicus GN=Got1 PE=1 SV=3	SCASQLVLGDNSPALR
>	sp|P13221|AAT	Aspartate aminotransferase, cytoplasmic OS=Rattus norvegicus GN=Got1 PE=1 SV=3	SCASQLVLGDNSPALR
>	sp|P13221|AAT	Aspartate aminotransferase, cytoplasmic OS=Rattus norvegicus GN=Got1 PE=1 SV=3	SCASQLVLGDNSPALR
>	sp|P13221|AAT	Aspartate aminotransferase, cytoplasmic OS=Rattus norvegicus GN=Got1 PE=1 SV=3	TDDSQPWVLPVVR
>	sp|P13221|AAT	Aspartate aminotransferase, cytoplasmic OS=Rattus norvegicus GN=Got1 PE=1 SV=3	TDDSQPWVLPVVR
>	sp|P13221|AAT	Aspartate aminotransferase, cytoplasmic OS=Rattus norvegicus GN=Got1 PE=1 SV=3	VGGVQSLGGTGALR
>	sp|P13221|AAT	Aspartate aminotransferase, cytoplasmic OS=Rattus norvegicus GN=Got1 PE=1 SV=3	IANDHSLNHEYLPILGLAEFR
>	sp|P13221|AAT	Aspartate aminotransferase, cytoplasmic OS=Rattus norvegicus GN=Got1 PE=1 SV=3	TDDSQPWVLPVVR
>	sp|P13221|AAT	Aspartate aminotransferase, cytoplasmic OS=Rattus norvegicus GN=Got1 PE=1 SV=3	SCASQLVLGDNSPALR
>	sp|P13221|AAT	Aspartate aminotransferase, cytoplasmic OS=Rattus norvegicus GN=Got1 PE=1 SV=3	VGGVQSLGGTGALR
>	sp|P13221|AAT	Aspartate aminotransferase, cytoplasmic OS=Rattus norvegicus GN=Got1 PE=1 SV=3	TDDSQPWVLPVVR
>	sp|P13221|AAT	Aspartate aminotransferase, cytoplasmic OS=Rattus norvegicus GN=Got1 PE=1 SV=3	IANDHSLNHEYLPILGLAEFR
>	sp|P13221|AAT	Aspartate aminotransferase, cytoplasmic OS=Rattus norvegicus GN=Got1 PE=1 SV=3	LIADFRDDPDPR
>	sp|P13221|AAT	Aspartate aminotransferase, cytoplasmic OS=Rattus norvegicus GN=Got1 PE=1 SV=3	YFVSEGFELFCAQSFSK
>	sp|P13221|AAT	Aspartate aminotransferase, cytoplasmic OS=Rattus norvegicus GN=Got1 PE=1 SV=3	YFVSEGFELFCAQSFSK
>	sp|P13221|AAT	Aspartate aminotransferase, cytoplasmic OS=Rattus norvegicus GN=Got1 PE=1 SV=3	QIAAVMK
>	sp|P13221|AAT	Aspartate aminotransferase, cytoplasmic OS=Rattus norvegicus GN=Got1 PE=1 SV=3	ENRVGGVQSLGGTGALR
>	sp|P13221|AAT	Aspartate aminotransferase, cytoplasmic OS=Rattus norvegicus GN=Got1 PE=1 SV=3	VGGVQSLGGTGALR
>	sp|P13221|AAT	Aspartate aminotransferase, cytoplasmic OS=Rattus norvegicus GN=Got1 PE=1 SV=3	VEQKIANDHSLNHEYLPILGLAEFR
>	sp|P13221|AAT	Aspartate aminotransferase, cytoplasmic OS=Rattus norvegicus GN=Got1 PE=1 SV=3	VGGVQSLGGTGALR
>	sp|P13221|AAT	Aspartate aminotransferase, cytoplasmic OS=Rattus norvegicus GN=Got1 PE=1 SV=3	VEQKIANDHSLNHEYLPILGLAEFR
>	sp|P13221|AAT	Aspartate aminotransferase, cytoplasmic OS=Rattus norvegicus GN=Got1 PE=1 SV=3	SCASQLVLGDNSPALR

>	sp|P13221|AAT	Aspartate aminotransferase, cytoplasmic OS=Rattus norvegicus GN=Got1 PE=1 SV=3	LIADFRDDPDPR
>	sp|P13221|AAT	Aspartate aminotransferase, cytoplasmic OS=Rattus norvegicus GN=Got1 PE=1 SV=3	VGGVQSLGGTGALR
>	sp|P13221|AAT	Aspartate aminotransferase, cytoplasmic OS=Rattus norvegicus GN=Got1 PE=1 SV=3	VGGVQSLGGTGALR
>	sp|P18484|AP2	AP-2 complex subunit alpha-2 OS=Rattus norvegicus GN=Ap2a2 PE=1 SV=3	ACNQLGQFLQHR
>	sp|P18484|AP2	AP-2 complex subunit alpha-2 OS=Rattus norvegicus GN=Ap2a2 PE=1 SV=3	LTECLETILNK
>	sp|P18484|AP2	AP-2 complex subunit alpha-2 OS=Rattus norvegicus GN=Ap2a2 PE=1 SV=3	NAVLFEAISLIIHHDSEPNLLVR
>	sp|P18484|AP2	AP-2 complex subunit alpha-2 OS=Rattus norvegicus GN=Ap2a2 PE=1 SV=3	VGGYILGEFGNLIAGDPR
>	sp|P18484|AP2	AP-2 complex subunit alpha-2 OS=Rattus norvegicus GN=Ap2a2 PE=1 SV=3	VVHLLNDQHLGVVTAATSLITTLAQK
>	sp|P18484|AP2	AP-2 complex subunit alpha-2 OS=Rattus norvegicus GN=Ap2a2 PE=1 SV=3	VVHLLNDQHLGVVTAATSLITTLAQKNPEEFK
>	sp|P18484|AP2	AP-2 complex subunit alpha-2 OS=Rattus norvegicus GN=Ap2a2 PE=1 SV=3	ALLLSTYIK
>	sp|P18484|AP2	AP-2 complex subunit alpha-2 OS=Rattus norvegicus GN=Ap2a2 PE=1 SV=3	ACNQLGQFLQHR
>	sp|P18484|AP2	AP-2 complex subunit alpha-2 OS=Rattus norvegicus GN=Ap2a2 PE=1 SV=3	ACNQLGQFLQHR
>	sp|P18484|AP2	AP-2 complex subunit alpha-2 OS=Rattus norvegicus GN=Ap2a2 PE=1 SV=3	NAVLFEAISLIIHHDSEPNLLVR
>	sp|P18484|AP2	AP-2 complex subunit alpha-2 OS=Rattus norvegicus GN=Ap2a2 PE=1 SV=3	NAVLFEAISLIIHHDSEPNLLVR
>	sp|P18484|AP2	AP-2 complex subunit alpha-2 OS=Rattus norvegicus GN=Ap2a2 PE=1 SV=3	NAVLFEAISLIIHHDSEPNLLVR
>	sp|P18484|AP2	AP-2 complex subunit alpha-2 OS=Rattus norvegicus GN=Ap2a2 PE=1 SV=3	VGGYILGEFGNLIAGDPR
>	sp|P18484|AP2	AP-2 complex subunit alpha-2 OS=Rattus norvegicus GN=Ap2a2 PE=1 SV=3	VVHLLNDQHLGVVTAATSLITTLAQK
>	sp|P18484|AP2	AP-2 complex subunit alpha-2 OS=Rattus norvegicus GN=Ap2a2 PE=1 SV=3	VVHLLNDQHLGVVTAATSLITTLAQK
>	sp|P18484|AP2	AP-2 complex subunit alpha-2 OS=Rattus norvegicus GN=Ap2a2 PE=1 SV=3	VVHLLNDQHLGVVTAATSLITTLAQK
>	sp|P18484|AP2	AP-2 complex subunit alpha-2 OS=Rattus norvegicus GN=Ap2a2 PE=1 SV=3	VVHLLNDQHLGVVTAATSLITTLAQKNPEEFK
>	sp|P18484|AP2	AP-2 complex subunit alpha-2 OS=Rattus norvegicus GN=Ap2a2 PE=1 SV=3	VGGYILGEFGNLIAGDPR
>	sp|P18484|AP2	AP-2 complex subunit alpha-2 OS=Rattus norvegicus GN=Ap2a2 PE=1 SV=3	NPTFMGLALHCIANVGSR
>	sp|P18484|AP2	AP-2 complex subunit alpha-2 OS=Rattus norvegicus GN=Ap2a2 PE=1 SV=3	LTECLETILNK
>	sp|P18484|AP2	AP-2 complex subunit alpha-2 OS=Rattus norvegicus GN=Ap2a2 PE=1 SV=3	ALLLSTYIK
>	sp|P18484|AP2	AP-2 complex subunit alpha-2 OS=Rattus norvegicus GN=Ap2a2 PE=1 SV=3	LTECLETILNK
>	sp|P18484|AP2	AP-2 complex subunit alpha-2 OS=Rattus norvegicus GN=Ap2a2 PE=1 SV=3	LINNAIK
>	sp|P18484|AP2	AP-2 complex subunit alpha-2 OS=Rattus norvegicus GN=Ap2a2 PE=1 SV=3	AKHPMDTEITK
>	sp|P18484|AP2	AP-2 complex subunit alpha-2 OS=Rattus norvegicus GN=Ap2a2 PE=1 SV=3	KVQHSNAK
>	sp|P18484|AP2	AP-2 complex subunit alpha-2 OS=Rattus norvegicus GN=Ap2a2 PE=1 SV=3	AKHPMDTEITK
>	sp|P18484|AP2	AP-2 complex subunit alpha-2 OS=Rattus norvegicus GN=Ap2a2 PE=1 SV=3	AKHPMDTEITK
>	sp|P18484|AP2	AP-2 complex subunit alpha-2 OS=Rattus norvegicus GN=Ap2a2 PE=1 SV=3	IIGFGSALLEEVDPNPANFVGAGIIHTK
>	sp|P16617|PGK	Phosphoglycerate kinase 1 OS=Rattus norvegicus GN=Pgk1 PE=1 SV=2	ALESPERPFLAILGGAK
>	sp|P16617|PGK	Phosphoglycerate kinase 1 OS=Rattus norvegicus GN=Pgk1 PE=1 SV=2	GCITIIGGGDTATCCAK
>	sp|P16617|PGK	Phosphoglycerate kinase 1 OS=Rattus norvegicus GN=Pgk1 PE=1 SV=2	ITLPVDFVTADKFDENAK
>	sp|P16617|PGK	Phosphoglycerate kinase 1 OS=Rattus norvegicus GN=Pgk1 PE=1 SV=2	LGDVYVNDAFGTAHR
>	sp|P16617|PGK	Phosphoglycerate kinase 1 OS=Rattus norvegicus GN=Pgk1 PE=1 SV=2	SLLGKDVLFLK
>	sp|P16617|PGK	Phosphoglycerate kinase 1 OS=Rattus norvegicus GN=Pgk1 PE=1 SV=2	TGQATVASGIPAGWMGLDCGTESSKK
>	sp|P16617|PGK	Phosphoglycerate kinase 1 OS=Rattus norvegicus GN=Pgk1 PE=1 SV=2	SVVLMSHLGRPDGVPMPDKYSLEPVAAELK
>	sp|P16617|PGK	Phosphoglycerate kinase 1 OS=Rattus norvegicus GN=Pgk1 PE=1 SV=2	ALESPERPFLAILGGAK
>	sp|P16617|PGK	Phosphoglycerate kinase 1 OS=Rattus norvegicus GN=Pgk1 PE=1 SV=2	ALESPERPFLAILGGAK
>	sp|P16617|PGK	Phosphoglycerate kinase 1 OS=Rattus norvegicus GN=Pgk1 PE=1 SV=2	ALESPERPFLAILGGAK
>	sp|P16617|PGK	Phosphoglycerate kinase 1 OS=Rattus norvegicus GN=Pgk1 PE=1 SV=2	ALESPERPFLAILGGAK
>	sp|P16617|PGK	Phosphoglycerate kinase 1 OS=Rattus norvegicus GN=Pgk1 PE=1 SV=2	ALESPERPFLAILGGAK
>	sp|P16617|PGK	Phosphoglycerate kinase 1 OS=Rattus norvegicus GN=Pgk1 PE=1 SV=2	GCITIIGGGDTATCCAK
>	sp|P16617|PGK	Phosphoglycerate kinase 1 OS=Rattus norvegicus GN=Pgk1 PE=1 SV=2	ITLPVDFVTADKFDENAK
>	sp|P16617|PGK	Phosphoglycerate kinase 1 OS=Rattus norvegicus GN=Pgk1 PE=1 SV=2	ITLPVDFVTADKFDENAK
>	sp|P16617|PGK	Phosphoglycerate kinase 1 OS=Rattus norvegicus GN=Pgk1 PE=1 SV=2	ITLPVDFVTADKFDENAK
>	sp|P16617|PGK	Phosphoglycerate kinase 1 OS=Rattus norvegicus GN=Pgk1 PE=1 SV=2	ITLPVDFVTADKFDENAK
>	sp|P16617|PGK	Phosphoglycerate kinase 1 OS=Rattus norvegicus GN=Pgk1 PE=1 SV=2	ITLPVDFVTADKFDENAK
>	sp|P16617|PGK	Phosphoglycerate kinase 1 OS=Rattus norvegicus GN=Pgk1 PE=1 SV=2	SLLGKDVLFLK
>	sp|P16617|PGK	Phosphoglycerate kinase 1 OS=Rattus norvegicus GN=Pgk1 PE=1 SV=2	TGQATVASGIPAGWMGLDCGTESSKK
>	sp|P16617|PGK	Phosphoglycerate kinase 1 OS=Rattus norvegicus GN=Pgk1 PE=1 SV=2	ALESPERPFLAILGGAK
>	sp|P16617|PGK	Phosphoglycerate kinase 1 OS=Rattus norvegicus GN=Pgk1 PE=1 SV=2	ITLPVDFVTADKFDENAK
>	sp|P16617|PGK	Phosphoglycerate kinase 1 OS=Rattus norvegicus GN=Pgk1 PE=1 SV=2	ITLPVDFVTADKFDENAK
>	sp|P16617|PGK	Phosphoglycerate kinase 1 OS=Rattus norvegicus GN=Pgk1 PE=1 SV=2	ITLPVDFVTADKFDENAK
>	sp|P16617|PGK	Phosphoglycerate kinase 1 OS=Rattus norvegicus GN=Pgk1 PE=1 SV=2	ITLPVDFVTADKFDENAK
>	sp|P16617|PGK	Phosphoglycerate kinase 1 OS=Rattus norvegicus GN=Pgk1 PE=1 SV=2	SLLGKDVLFLK
>	sp|P16617|PGK	Phosphoglycerate kinase 1 OS=Rattus norvegicus GN=Pgk1 PE=1 SV=2	VDFNVPMK
>	sp|P16617|PGK	Phosphoglycerate kinase 1 OS=Rattus norvegicus GN=Pgk1 PE=1 SV=2	SLMDEVVK
>	sp|P16617|PGK	Phosphoglycerate kinase 1 OS=Rattus norvegicus GN=Pgk1 PE=1 SV=2	SLMDEVVK
>	sp|P16617|PGK	Phosphoglycerate kinase 1 OS=Rattus norvegicus GN=Pgk1 PE=1 SV=2	ITLPVDFVTADKFDENAK
>	sp|P16617|PGK	Phosphoglycerate kinase 1 OS=Rattus norvegicus GN=Pgk1 PE=1 SV=2	SLMDEVVK
>	sp|P16617|PGK	Phosphoglycerate kinase 1 OS=Rattus norvegicus GN=Pgk1 PE=1 SV=2	SVVLMSHLGRPDGVPMPDKYSLEPVAAELK
>	sp|P16617|PGK	Phosphoglycerate kinase 1 OS=Rattus norvegicus GN=Pgk1 PE=1 SV=2	ITLPVDFVTADKFDENAK
>	sp|P16617|PGK	Phosphoglycerate kinase 1 OS=Rattus norvegicus GN=Pgk1 PE=1 SV=2	SVVLMSHLGRPDGVPMPDKYSLEPVAAELK
>	sp|P16617|PGK	Phosphoglycerate kinase 1 OS=Rattus norvegicus GN=Pgk1 PE=1 SV=2	YAEAVAR
>	sp|P16617|PGK	Phosphoglycerate kinase 1 OS=Rattus norvegicus GN=Pgk1 PE=1 SV=2	LGDVYVNDAFGTAHR
>	sp|P16617|PGK	Phosphoglycerate kinase 1 OS=Rattus norvegicus GN=Pgk1 PE=1 SV=2	AGGFLMK
>	sp|P16617|PGK	Phosphoglycerate kinase 1 OS=Rattus norvegicus GN=Pgk1 PE=1 SV=2	ITLPVDFVTADKFDENAK
>	sp|P16617|PGK	Phosphoglycerate kinase 1 OS=Rattus norvegicus GN=Pgk1 PE=1 SV=2	ITLPVDFVTADKFDENAK
>	sp|P16617|PGK	Phosphoglycerate kinase 1 OS=Rattus norvegicus GN=Pgk1 PE=1 SV=2	LGDVYVNDAFGTAHR
>	sp|P16617|PGK	Phosphoglycerate kinase 1 OS=Rattus norvegicus GN=Pgk1 PE=1 SV=2	AHSSMVGVNLPQK
>	sp|P59215|GNA	Guanine nucleotide-binding protein G(o) subunit alpha OS=Rattus norvegicus GN=Gnao1 PE=1 SV=2	EIYCHMTCATDTNNIQVVFDAVTDIIIANNLR
>	sp|P59215|GNA	Guanine nucleotide-binding protein G(o) subunit alpha OS=Rattus norvegicus GN=Gnao1 PE=1 SV=2	IGAADYQPTEQDILR

>	sp|P59215|GN	Guanine nucleotide-binding protein G(o) subunit alpha OS=Rattus norvegicus GN=Gnao1 PE=1 SV=2	LLLLGAGESGK
>	sp|P59215|GN	Guanine nucleotide-binding protein G(o) subunit alpha OS=Rattus norvegicus GN=Gnao1 PE=1 SV=2	MEDTEPFSAELLSAMMR
>	sp|P59215|GN	Guanine nucleotide-binding protein G(o) subunit alpha OS=Rattus norvegicus GN=Gnao1 PE=1 SV=2	TTGIVETHFTFK
>	sp|P59215|GN	Guanine nucleotide-binding protein G(o) subunit alpha OS=Rattus norvegicus GN=Gnao1 PE=1 SV=2	IIHEDGFSGEDVK
>	sp|P59215|GN	Guanine nucleotide-binding protein G(o) subunit alpha OS=Rattus norvegicus GN=Gnao1 PE=1 SV=2	SPLTICFPEYPGSNTYEDAAAYIQTQFESK
>	sp|P59215|GN	Guanine nucleotide-binding protein G(o) subunit alpha OS=Rattus norvegicus GN=Gnao1 PE=1 SV=2	KSPLTICFPEYPGSNTYEDAAAYIQTQFESK
>	sp|P59215|GN	Guanine nucleotide-binding protein G(o) subunit alpha OS=Rattus norvegicus GN=Gnao1 PE=1 SV=2	AMDTLGVEYGDKER
>	sp|P59215|GN	Guanine nucleotide-binding protein G(o) subunit alpha OS=Rattus norvegicus GN=Gnao1 PE=1 SV=2	QYKPVVYSNTIQSLAAIVR
>	sp|P59215|GN	Guanine nucleotide-binding protein G(o) subunit alpha OS=Rattus norvegicus GN=Gnao1 PE=1 SV=2	AMDTLGVEYGDKER
>	sp|P59215|GN	Guanine nucleotide-binding protein G(o) subunit alpha OS=Rattus norvegicus GN=Gnao1 PE=1 SV=2	EIYCHMTCATDTNNIQVVFDAVTDIIIANNLR
>	sp|P59215|GN	Guanine nucleotide-binding protein G(o) subunit alpha OS=Rattus norvegicus GN=Gnao1 PE=1 SV=2	EIYCHMTCATDTNNIQVVFDAVTDIIIANNLR
>	sp|P59215|GN	Guanine nucleotide-binding protein G(o) subunit alpha OS=Rattus norvegicus GN=Gnao1 PE=1 SV=2	EIYCHMTCATDTNNIQVVFDAVTDIIIANNLR
>	sp|P59215|GN	Guanine nucleotide-binding protein G(o) subunit alpha OS=Rattus norvegicus GN=Gnao1 PE=1 SV=2	IGAADYQPTEQDILR
>	sp|P59215|GN	Guanine nucleotide-binding protein G(o) subunit alpha OS=Rattus norvegicus GN=Gnao1 PE=1 SV=2	KSPLTICFPEYPGSNTYEDAAAYIQTQFESK
>	sp|P59215|GN	Guanine nucleotide-binding protein G(o) subunit alpha OS=Rattus norvegicus GN=Gnao1 PE=1 SV=2	LLLLGAGESGK
>	sp|P59215|GN	Guanine nucleotide-binding protein G(o) subunit alpha OS=Rattus norvegicus GN=Gnao1 PE=1 SV=2	LLLLGAGESGK
>	sp|P59215|GN	Guanine nucleotide-binding protein G(o) subunit alpha OS=Rattus norvegicus GN=Gnao1 PE=1 SV=2	LLLLGAGESGK
>	sp|P59215|GN	Guanine nucleotide-binding protein G(o) subunit alpha OS=Rattus norvegicus GN=Gnao1 PE=1 SV=2	MEDTEPFSAELLSAMMR
>	sp|P59215|GN	Guanine nucleotide-binding protein G(o) subunit alpha OS=Rattus norvegicus GN=Gnao1 PE=1 SV=2	MEDTEPFSAELLSAMMR
>	sp|P59215|GN	Guanine nucleotide-binding protein G(o) subunit alpha OS=Rattus norvegicus GN=Gnao1 PE=1 SV=2	QYKPVVYSNTIQSLAAIVR
>	sp|P59215|GN	Guanine nucleotide-binding protein G(o) subunit alpha OS=Rattus norvegicus GN=Gnao1 PE=1 SV=2	QYKPVVYSNTIQSLAAIVR
>	sp|P59215|GN	Guanine nucleotide-binding protein G(o) subunit alpha OS=Rattus norvegicus GN=Gnao1 PE=1 SV=2	SPLTICFPEYPGSNTYEDAAAYIQTQFESK
>	sp|P59215|GN	Guanine nucleotide-binding protein G(o) subunit alpha OS=Rattus norvegicus GN=Gnao1 PE=1 SV=2	LLLLGAGESGK
>	sp|P59215|GN	Guanine nucleotide-binding protein G(o) subunit alpha OS=Rattus norvegicus GN=Gnao1 PE=1 SV=2	QYKPVVYSNTIQSLAAIVR
>	sp|P59215|GN	Guanine nucleotide-binding protein G(o) subunit alpha OS=Rattus norvegicus GN=Gnao1 PE=1 SV=2	QYKPVVYSNTIQSLAAIVR
>	sp|P59215|GN	Guanine nucleotide-binding protein G(o) subunit alpha OS=Rattus norvegicus GN=Gnao1 PE=1 SV=2	AMDTLGVEYGDKER
>	sp|P59215|GN	Guanine nucleotide-binding protein G(o) subunit alpha OS=Rattus norvegicus GN=Gnao1 PE=1 SV=2	QYKPVVYSNTIQSLAAIVR
>	sp|P59215|GN	Guanine nucleotide-binding protein G(o) subunit alpha OS=Rattus norvegicus GN=Gnao1 PE=1 SV=2	SPLTICFPEYPGSNTYEDAAAYIQTQFESK
>	sp|P59215|GN	Guanine nucleotide-binding protein G(o) subunit alpha OS=Rattus norvegicus GN=Gnao1 PE=1 SV=2	LFDVGGQR
>	sp|P59215|GN	Guanine nucleotide-binding protein G(o) subunit alpha OS=Rattus norvegicus GN=Gnao1 PE=1 SV=2	EYQLNDSAK
>	sp|P59215|GN	Guanine nucleotide-binding protein G(o) subunit alpha OS=Rattus norvegicus GN=Gnao1 PE=1 SV=2	AMDTLGVEYGDKER
>	sp|P59215|GN	Guanine nucleotide-binding protein G(o) subunit alpha OS=Rattus norvegicus GN=Gnao1 PE=1 SV=2	YYLDSLDR
>	sp|P53534|PYG	Glycogen phosphorylase, brain form (Fragment) OS=Rattus norvegicus GN=Pygb PE=1 SV=3	HLDHVAALFPGDVDR
>	sp|P53534|PYG	Glycogen phosphorylase, brain form (Fragment) OS=Rattus norvegicus GN=Pygb PE=1 SV=3	HLEIIYAINQR
>	sp|P53534|PYG	Glycogen phosphorylase, brain form (Fragment) OS=Rattus norvegicus GN=Pygb PE=1 SV=3	LKDFNVGDYIEAVLDR
>	sp|P53534|PYG	Glycogen phosphorylase, brain form (Fragment) OS=Rattus norvegicus GN=Pygb PE=1 SV=3	LKQEYFVVAATLQDIIR
>	sp|P53534|PYG	Glycogen phosphorylase, brain form (Fragment) OS=Rattus norvegicus GN=Pygb PE=1 SV=3	LKQEYFVVAATLQDIIRRFKSSK
>	sp|P53534|PYG	Glycogen phosphorylase, brain form (Fragment) OS=Rattus norvegicus GN=Pygb PE=1 SV=3	QAVDQISSGFFSPKDPDCFKDVVNMLMYHDR
>	sp|P53534|PYG	Glycogen phosphorylase, brain form (Fragment) OS=Rattus norvegicus GN=Pygb PE=1 SV=3	INMAHLCVIGSHAVNGVAR
>	sp|P53534|PYG	Glycogen phosphorylase, brain form (Fragment) OS=Rattus norvegicus GN=Pygb PE=1 SV=3	GIAGLGDVAEVR
>	sp|P53534|PYG	Glycogen phosphorylase, brain form (Fragment) OS=Rattus norvegicus GN=Pygb PE=1 SV=3	HLDHVAALFPGDVDR
>	sp|P53534|PYG	Glycogen phosphorylase, brain form (Fragment) OS=Rattus norvegicus GN=Pygb PE=1 SV=3	HLEIIYAINQR
>	sp|P53534|PYG	Glycogen phosphorylase, brain form (Fragment) OS=Rattus norvegicus GN=Pygb PE=1 SV=3	HLEIIYAINQR
>	sp|P53534|PYG	Glycogen phosphorylase, brain form (Fragment) OS=Rattus norvegicus GN=Pygb PE=1 SV=3	INMAHLCVIGSHAVNGVAR
>	sp|P53534|PYG	Glycogen phosphorylase, brain form (Fragment) OS=Rattus norvegicus GN=Pygb PE=1 SV=3	LKDFNVGDYIEAVLDR
>	sp|P53534|PYG	Glycogen phosphorylase, brain form (Fragment) OS=Rattus norvegicus GN=Pygb PE=1 SV=3	LKDFNVGDYIEAVLDR
>	sp|P53534|PYG	Glycogen phosphorylase, brain form (Fragment) OS=Rattus norvegicus GN=Pygb PE=1 SV=3	LKQEYFVVAATLQDIIR
>	sp|P53534|PYG	Glycogen phosphorylase, brain form (Fragment) OS=Rattus norvegicus GN=Pygb PE=1 SV=3	LKQEYFVVAATLQDIIR
>	sp|P53534|PYG	Glycogen phosphorylase, brain form (Fragment) OS=Rattus norvegicus GN=Pygb PE=1 SV=3	LKQEYFVVAATLQDIIR
>	sp|P53534|PYG	Glycogen phosphorylase, brain form (Fragment) OS=Rattus norvegicus GN=Pygb PE=1 SV=3	LKQEYFVVAATLQDIIR
>	sp|P53534|PYG	Glycogen phosphorylase, brain form (Fragment) OS=Rattus norvegicus GN=Pygb PE=1 SV=3	LKQEYFVVAATLQDIIR
>	sp|P53534|PYG	Glycogen phosphorylase, brain form (Fragment) OS=Rattus norvegicus GN=Pygb PE=1 SV=3	LKQEYFVVAATLQDIIR
>	sp|P53534|PYG	Glycogen phosphorylase, brain form (Fragment) OS=Rattus norvegicus GN=Pygb PE=1 SV=3	LKQEYFVVAATLQDIIR
>	sp|P53534|PYG	Glycogen phosphorylase, brain form (Fragment) OS=Rattus norvegicus GN=Pygb PE=1 SV=3	LKQEYFVVAATLQDIIRRFKSSK
>	sp|P53534|PYG	Glycogen phosphorylase, brain form (Fragment) OS=Rattus norvegicus GN=Pygb PE=1 SV=3	VIFLENYR
>	sp|P53534|PYG	Glycogen phosphorylase, brain form (Fragment) OS=Rattus norvegicus GN=Pygb PE=1 SV=3	INMAHLCVIGSHAVNGVAR
>	sp|P53534|PYG	Glycogen phosphorylase, brain form (Fragment) OS=Rattus norvegicus GN=Pygb PE=1 SV=3	INMAHLCVIGSHAVNGVAR
>	sp|P53534|PYG	Glycogen phosphorylase, brain form (Fragment) OS=Rattus norvegicus GN=Pygb PE=1 SV=3	VDWDKAWEITK
>	sp|P53534|PYG	Glycogen phosphorylase, brain form (Fragment) OS=Rattus norvegicus GN=Pygb PE=1 SV=3	INMAHLCVIGSHAVNGVAR
>	sp|P53534|PYG	Glycogen phosphorylase, brain form (Fragment) OS=Rattus norvegicus GN=Pygb PE=1 SV=3	QLLNCLHIITLYNR
>	sp|P53534|PYG	Glycogen phosphorylase, brain form (Fragment) OS=Rattus norvegicus GN=Pygb PE=1 SV=3	INMAHLCVIGSHAVNGVAR
>	sp|P53534|PYG	Glycogen phosphorylase, brain form (Fragment) OS=Rattus norvegicus GN=Pygb PE=1 SV=3	INMAHLCVIGSHAVNGVAR
>	sp|P53534|PYG	Glycogen phosphorylase, brain form (Fragment) OS=Rattus norvegicus GN=Pygb PE=1 SV=3	HLHFTLVK
>	sp|P53534|PYG	Glycogen phosphorylase, brain form (Fragment) OS=Rattus norvegicus GN=Pygb PE=1 SV=3	INMAHLCVIGSHAVNGVAR
>	sp|P09117|ALD	Fructose-bisphosphate aldolase C OS=Rattus norvegicus GN=Aldoc PE=1 SV=3	DNAGAATEEFIK
>	sp|P09117|ALD	Fructose-bisphosphate aldolase C OS=Rattus norvegicus GN=Aldoc PE=1 SV=3	GILAADESVGSMAK
>	sp|P09117|ALD	Fructose-bisphosphate aldolase C OS=Rattus norvegicus GN=Aldoc PE=1 SV=3	GVVPLAGTDGETTTQGLDGLLER
>	sp|P09117|ALD	Fructose-bisphosphate aldolase C OS=Rattus norvegicus GN=Aldoc PE=1 SV=3	ISDRTPSALAILENANVLAR
>	sp|P09117|ALD	Fructose-bisphosphate aldolase C OS=Rattus norvegicus GN=Aldoc PE=1 SV=3	TPSALAILENANVLAR
>	sp|P09117|ALD	Fructose-bisphosphate aldolase C OS=Rattus norvegicus GN=Aldoc PE=1 SV=3	VDKGVVPLAGTDGETTTQGLDGLLER
>	sp|P09117|ALD	Fructose-bisphosphate aldolase C OS=Rattus norvegicus GN=Aldoc PE=1 SV=3	YSPEEIAMATVTALR
>	sp|P09117|ALD	Fructose-bisphosphate aldolase C OS=Rattus norvegicus GN=Aldoc PE=1 SV=3	ALQASALSAWR
>	sp|P09117|ALD	Fructose-bisphosphate aldolase C OS=Rattus norvegicus GN=Aldoc PE=1 SV=3	GILAADESVGSMAK
>	sp|P09117|ALD	Fructose-bisphosphate aldolase C OS=Rattus norvegicus GN=Aldoc PE=1 SV=3	GILAADESVGSMAK

>	sp|P09117|ALD Fructose-bisphosphate aldolase C OS=Rattus norvegicus GN=Aldoc PE=1 SV=3	GILAADESVGSMAK
>	sp|P09117|ALD Fructose-bisphosphate aldolase C OS=Rattus norvegicus GN=Aldoc PE=1 SV=3	ISDRTPSALAILENANVLAR
>	sp|P09117|ALD Fructose-bisphosphate aldolase C OS=Rattus norvegicus GN=Aldoc PE=1 SV=3	ISDRTPSALAILENANVLAR
>	sp|P09117|ALD Fructose-bisphosphate aldolase C OS=Rattus norvegicus GN=Aldoc PE=1 SV=3	ISDRTPSALAILENANVLAR
>	sp|P09117|ALD Fructose-bisphosphate aldolase C OS=Rattus norvegicus GN=Aldoc PE=1 SV=3	ISDRTPSALAILENANVLAR
>	sp|P09117|ALD Fructose-bisphosphate aldolase C OS=Rattus norvegicus GN=Aldoc PE=1 SV=3	ISDRTPSALAILENANVLAR
>	sp|P09117|ALD Fructose-bisphosphate aldolase C OS=Rattus norvegicus GN=Aldoc PE=1 SV=3	VDKGVVPLAGTDGETTTQGLDGLLER
>	sp|P09117|ALD Fructose-bisphosphate aldolase C OS=Rattus norvegicus GN=Aldoc PE=1 SV=3	VDKGVVPLAGTDGETTTQGLDGLLER
>	sp|P09117|ALD Fructose-bisphosphate aldolase C OS=Rattus norvegicus GN=Aldoc PE=1 SV=3	VDKGVVPLAGTDGETTTQGLDGLLER
>	sp|P09117|ALD Fructose-bisphosphate aldolase C OS=Rattus norvegicus GN=Aldoc PE=1 SV=3	VDKGVVPLAGTDGETTTQGLDGLLER
>	sp|P09117|ALD Fructose-bisphosphate aldolase C OS=Rattus norvegicus GN=Aldoc PE=1 SV=3	YASICQQNGIVPIVEPEILPDGDHDLK
>	sp|P09117|ALD Fructose-bisphosphate aldolase C OS=Rattus norvegicus GN=Aldoc PE=1 SV=3	YASICQQNGIVPIVEPEILPDGDHDLK
>	sp|P09117|ALD Fructose-bisphosphate aldolase C OS=Rattus norvegicus GN=Aldoc PE=1 SV=3	YASICQQNGIVPIVEPEILPDGDHDLK
>	sp|P09117|ALD Fructose-bisphosphate aldolase C OS=Rattus norvegicus GN=Aldoc PE=1 SV=3	YASICQQNGIVPIVEPEILPDGDHDLK
>	sp|P09117|ALD Fructose-bisphosphate aldolase C OS=Rattus norvegicus GN=Aldoc PE=1 SV=3	YASICQQNGIVPIVEPEILPDGDHDLK
>	sp|P09117|ALD Fructose-bisphosphate aldolase C OS=Rattus norvegicus GN=Aldoc PE=1 SV=3	YASICQQNGIVPIVEPEILPDGDHDLKR
>	sp|P09117|ALD Fructose-bisphosphate aldolase C OS=Rattus norvegicus GN=Aldoc PE=1 SV=3	YASICQQNGIVPIVEPEILPDGDHDLKR
>	sp|P09117|ALD Fructose-bisphosphate aldolase C OS=Rattus norvegicus GN=Aldoc PE=1 SV=3	YASICQQNGIVPIVEPEILPDGDHDLKR
>	sp|P09117|ALD Fructose-bisphosphate aldolase C OS=Rattus norvegicus GN=Aldoc PE=1 SV=3	YASICQQNGIVPIVEPEILPDGDHDLKR
>	sp|P09117|ALD Fructose-bisphosphate aldolase C OS=Rattus norvegicus GN=Aldoc PE=1 SV=3	YASICQQNGIVPIVEPEILPDGDHDLKR
>	sp|P09117|ALD Fructose-bisphosphate aldolase C OS=Rattus norvegicus GN=Aldoc PE=1 SV=3	YASICQQNGIVPIVEPEILPDGDHDLKR
>	sp|P09117|ALD Fructose-bisphosphate aldolase C OS=Rattus norvegicus GN=Aldoc PE=1 SV=3	YASICQQNGIVPIVEPEILPDGDHDLKR
>	sp|P09117|ALD Fructose-bisphosphate aldolase C OS=Rattus norvegicus GN=Aldoc PE=1 SV=3	YASICQQNGIVPIVEPEILPDGDHDLKR
>	sp|P09117|ALD Fructose-bisphosphate aldolase C OS=Rattus norvegicus GN=Aldoc PE=1 SV=3	YASICQQNGIVPIVEPEILPDGDHDLKR
>	sp|P09117|ALD Fructose-bisphosphate aldolase C OS=Rattus norvegicus GN=Aldoc PE=1 SV=3	YASICQQNGIVPIVEPEILPDGDHDLKR
>	sp|P09117|ALD Fructose-bisphosphate aldolase C OS=Rattus norvegicus GN=Aldoc PE=1 SV=3	YASICQQNGIVPIVEPEILPDGDHDLKR
>	sp|P09117|ALD Fructose-bisphosphate aldolase C OS=Rattus norvegicus GN=Aldoc PE=1 SV=3	YASICQQNGIVPIVEPEILPDGDHDLKR
>	sp|P09117|ALD Fructose-bisphosphate aldolase C OS=Rattus norvegicus GN=Aldoc PE=1 SV=3	YASICQQNGIVPIVEPEILPDGDHDLKR
>	sp|P09117|ALD Fructose-bisphosphate aldolase C OS=Rattus norvegicus GN=Aldoc PE=1 SV=3	YASICQQNGIVPIVEPEILPDGDHDLKR
>	sp|P09117|ALD Fructose-bisphosphate aldolase C OS=Rattus norvegicus GN=Aldoc PE=1 SV=3	YASICQQNGIVPIVEPEILPDGDHDLKR
>	sp|P09117|ALD Fructose-bisphosphate aldolase C OS=Rattus norvegicus GN=Aldoc PE=1 SV=3	YASICQQNGIVPIVEPEILPDGDHDLKR
>	sp|P09117|ALD Fructose-bisphosphate aldolase C OS=Rattus norvegicus GN=Aldoc PE=1 SV=3	YASICQQNGIVPIVEPEILPDGDHDLKR
>	sp|P09117|ALD Fructose-bisphosphate aldolase C OS=Rattus norvegicus GN=Aldoc PE=1 SV=3	YASICQQNGIVPIVEPEILPDGDHDLKR
>	sp|P09117|ALD Fructose-bisphosphate aldolase C OS=Rattus norvegicus GN=Aldoc PE=1 SV=3	YASICQQNGIVPIVEPEILPDGDHDLKR
>	sp|P09117|ALD Fructose-bisphosphate aldolase C OS=Rattus norvegicus GN=Aldoc PE=1 SV=3	YASICQQNGIVPIVEPEILPDGDHDLKR
>	sp|P09117|ALD Fructose-bisphosphate aldolase C OS=Rattus norvegicus GN=Aldoc PE=1 SV=3	YASICQQNGIVPIVEPEILPDGDHDLKR
>	sp|P09117|ALD Fructose-bisphosphate aldolase C OS=Rattus norvegicus GN=Aldoc PE=1 SV=3	YASICQQNGIVPIVEPEILPDGDHDLKR
>	sp|P09117|ALD Fructose-bisphosphate aldolase C OS=Rattus norvegicus GN=Aldoc PE=1 SV=3	YASICQQNGIVPIVEPEILPDGDHDLKR
>	sp|P09117|ALD Fructose-bisphosphate aldolase C OS=Rattus norvegicus GN=Aldoc PE=1 SV=3	YASICQQNGIVPIVEPEILPDGDHDLKR
>	sp|P09117|ALD Fructose-bisphosphate aldolase C OS=Rattus norvegicus GN=Aldoc PE=1 SV=3	YASICQQNGIVPIVEPEILPDGDHDLKR
>	sp|P09117|ALD Fructose-bisphosphate aldolase C OS=Rattus norvegicus GN=Aldoc PE=1 SV=3	YASICQQNGIVPIVEPEILPDGDHDLKR
>	sp|P09117|ALD Fructose-bisphosphate aldolase C OS=Rattus norvegicus GN=Aldoc PE=1 SV=3	YASICQQNGIVPIVEPEILPDGDHDLKR
>	sp|P09117|ALD Fructose-bisphosphate aldolase C OS=Rattus norvegicus GN=Aldoc PE=1 SV=3	YASICQQNGIVPIVEPEILPDGDHDLKR
>	sp|P09117|ALD Fructose-bisphosphate aldolase C OS=Rattus norvegicus GN=Aldoc PE=1 SV=3	YASICQQNGIVPIVEPEILPDGDHDLKR
>	sp|P09117|ALD Fructose-bisphosphate aldolase C OS=Rattus norvegicus GN=Aldoc PE=1 SV=3	YASICQQNGIVPIVEPEILPDGDHDLKR
>	sp|P09117|ALD Fructose-bisphosphate aldolase C OS=Rattus norvegicus GN=Aldoc PE=1 SV=3	YSPEEIAMATVTALR
>	sp|P09117|ALD Fructose-bisphosphate aldolase C OS=Rattus norvegicus GN=Aldoc PE=1 SV=3	YSPEEIAMATVTALR
>	sp|P09117|ALD Fructose-bisphosphate aldolase C OS=Rattus norvegicus GN=Aldoc PE=1 SV=3	YSPEEIAMATVTALR
>	sp|P09117|ALD Fructose-bisphosphate aldolase C OS=Rattus norvegicus GN=Aldoc PE=1 SV=3	YSPEEIAMATVTALR
>	sp|P09117|ALD Fructose-bisphosphate aldolase C OS=Rattus norvegicus GN=Aldoc PE=1 SV=3	YSPEEIAMATVTALR
>	sp|P09117|ALD Fructose-bisphosphate aldolase C OS=Rattus norvegicus GN=Aldoc PE=1 SV=3	GILAADESVGSMAK
>	sp|P09117|ALD Fructose-bisphosphate aldolase C OS=Rattus norvegicus GN=Aldoc PE=1 SV=3	DNAGAATEEFIK
>	sp|P09117|ALD Fructose-bisphosphate aldolase C OS=Rattus norvegicus GN=Aldoc PE=1 SV=3	YSPEEIAMATVTALR
>	sp|P09117|ALD Fructose-bisphosphate aldolase C OS=Rattus norvegicus GN=Aldoc PE=1 SV=3	ISDRTPSALAILENANVLAR
>	sp|P09117|ALD Fructose-bisphosphate aldolase C OS=Rattus norvegicus GN=Aldoc PE=1 SV=3	GILAADESVGSMAK
>	sp|P09117|ALD Fructose-bisphosphate aldolase C OS=Rattus norvegicus GN=Aldoc PE=1 SV=3	IVAPGK
>	sp|P09117|ALD Fructose-bisphosphate aldolase C OS=Rattus norvegicus GN=Aldoc PE=1 SV=3	YASICQQNGIVPIVEPEILPDGDHDLKR
>	sp|P09117|ALD Fructose-bisphosphate aldolase C OS=Rattus norvegicus GN=Aldoc PE=1 SV=3	YASICQQNGIVPIVEPEILPDGDHDLKR
>	sp|P09117|ALD Fructose-bisphosphate aldolase C OS=Rattus norvegicus GN=Aldoc PE=1 SV=3	DGADFAK
>	sp|P09117|ALD Fructose-bisphosphate aldolase C OS=Rattus norvegicus GN=Aldoc PE=1 SV=3	IVAPGK
>	sp|P09117|ALD Fructose-bisphosphate aldolase C OS=Rattus norvegicus GN=Aldoc PE=1 SV=3	GILAADESVGSMAK
>	sp|P09117|ALD Fructose-bisphosphate aldolase C OS=Rattus norvegicus GN=Aldoc PE=1 SV=3	ALSDHHVYLEGTLLKPNMVTPGHACPIK
>	sp|P09117|ALD Fructose-bisphosphate aldolase C OS=Rattus norvegicus GN=Aldoc PE=1 SV=3	IVAPGK
>	sp|P09117|ALD Fructose-bisphosphate aldolase C OS=Rattus norvegicus GN=Aldoc PE=1 SV=3	YASICQQNGIVPIVEPEILPDGDHDLKR
>	sp|P09117|ALD Fructose-bisphosphate aldolase C OS=Rattus norvegicus GN=Aldoc PE=1 SV=3	IVAPGK
>	sp|P09117|ALD Fructose-bisphosphate aldolase C OS=Rattus norvegicus GN=Aldoc PE=1 SV=3	YSPEEIAMATVTALRR
>	sp|P09117|ALD Fructose-bisphosphate aldolase C OS=Rattus norvegicus GN=Aldoc PE=1 SV=3	TVPPAVPGVTFLSGGQSEEEASLNLNAINRCSLPRPWALTFSYGRALQASALS
>	sp|P09117|ALD Fructose-bisphosphate aldolase C OS=Rattus norvegicus GN=Aldoc PE=1 SV=3	VLAAVYK
>	sp|P09117|ALD Fructose-bisphosphate aldolase C OS=Rattus norvegicus GN=Aldoc PE=1 SV=3	ALSDHHVYLEGTLLKPNMVTPGHACPIK
>	sp|P09117|ALD Fructose-bisphosphate aldolase C OS=Rattus norvegicus GN=Aldoc PE=1 SV=3	IVAPGK
>	sp|P09117|ALD Fructose-bisphosphate aldolase C OS=Rattus norvegicus GN=Aldoc PE=1 SV=3	YASICQQNGIVPIVEPEILPDGDHDLK
>	sp|P09117|ALD Fructose-bisphosphate aldolase C OS=Rattus norvegicus GN=Aldoc PE=1 SV=3	YASICQQNGIVPIVEPEILPDGDHDLKR
>	sp|P09117|ALD Fructose-bisphosphate aldolase C OS=Rattus norvegicus GN=Aldoc PE=1 SV=3	YASICQQNGIVPIVEPEILPDGDHDLKR
>	sp|P09117|ALD Fructose-bisphosphate aldolase C OS=Rattus norvegicus GN=Aldoc PE=1 SV=3	IVAPGK

>	sp|P09117|ALD	Fructose-bisphosphate aldolase C OS=Rattus norvegicus GN=Aldoc PE=1 SV=3	YASICQQNGIVPIVEPEILPDGDHDLKR
>	sp|P09117|ALD	Fructose-bisphosphate aldolase C OS=Rattus norvegicus GN=Aldoc PE=1 SV=3	VLAAVYK
>	sp|P09117|ALD	Fructose-bisphosphate aldolase C OS=Rattus norvegicus GN=Aldoc PE=1 SV=3	YASICQQNGIVPIVEPEILPDGDHDLKR
>	sp|P68370|TBA	Tubulin alpha-1A chain OS=Rattus norvegicus GN=Tuba1a PE=1 SV=1	AVFVDLEPTVIDEVR
>	sp|P68370|TBA	Tubulin alpha-1A chain OS=Rattus norvegicus GN=Tuba1a PE=1 SV=1	DVNAAIATIK
>	sp|P68370|TBA	Tubulin alpha-1A chain OS=Rattus norvegicus GN=Tuba1a PE=1 SV=1	ECISIHVGQAGVQIGNACWELYCLEHGIQPDGQMPSDK
>	sp|P68370|TBA	Tubulin alpha-1A chain OS=Rattus norvegicus GN=Tuba1a PE=1 SV=1	ECISIHVGQAGVQIGNACWELYCLEHGIQPDGQMPSDKTIGGGDDSFNTFFSETGAGK
>	sp|P68370|TBA	Tubulin alpha-1A chain OS=Rattus norvegicus GN=Tuba1a PE=1 SV=1	NLDIERPTYTNLNRLIGQIVSSITASLR
>	sp|P68370|TBA	Tubulin alpha-1A chain OS=Rattus norvegicus GN=Tuba1a PE=1 SV=1	NLDIERPTYTNLNRLIGQIVSSITASLRFDGALNVDLTEFQTNLVPYPR
>	sp|P68370|TBA	Tubulin alpha-1A chain OS=Rattus norvegicus GN=Tuba1a PE=1 SV=1	TIGGGDDSFNTFFSETGAGK
>	sp|P68370|TBA	Tubulin alpha-1A chain OS=Rattus norvegicus GN=Tuba1a PE=1 SV=1	MRECISIHVGQAGVQIGNACWELYCLEHGIQPDGQMPSDK
>	sp|P68370|TBA	Tubulin alpha-1A chain OS=Rattus norvegicus GN=Tuba1a PE=1 SV=1	RECISIHVGQAGVQIGNACWELYCLEHGIQPDGQMPSDKTIGGGDDSFNTFFSETGAGK
>	sp|P68370|TBA	Tubulin alpha-1A chain OS=Rattus norvegicus GN=Tuba1a PE=1 SV=1	DGQMPSDKTIGGGDDSFNTFFSETGAGK
>	sp|P68370|TBA	Tubulin alpha-1A chain OS=Rattus norvegicus GN=Tuba1a PE=1 SV=1	CISIHVGQAGVQIGNACWELYCLEHGIQPDGQMPSDKTIGGGDDSFNTFFSETGAGK
>	sp|P68370|TBA	Tubulin alpha-1A chain OS=Rattus norvegicus GN=Tuba1a PE=1 SV=1	AFVHWYVGEGMEEGEFSEAR
>	sp|P68370|TBA	Tubulin alpha-1A chain OS=Rattus norvegicus GN=Tuba1a PE=1 SV=1	AFVHWYVGEGMEEGEFSEAR
>	sp|P68370|TBA	Tubulin alpha-1A chain OS=Rattus norvegicus GN=Tuba1a PE=1 SV=1	AFVHWYVGEGMEEGEFSEAR
>	sp|P68370|TBA	Tubulin alpha-1A chain OS=Rattus norvegicus GN=Tuba1a PE=1 SV=1	AFVHWYVGEGMEEGEFSEAR
>	sp|P68370|TBA	Tubulin alpha-1A chain OS=Rattus norvegicus GN=Tuba1a PE=1 SV=1	AFVHWYVGEGMEEGEFSEAR
>	sp|P68370|TBA	Tubulin alpha-1A chain OS=Rattus norvegicus GN=Tuba1a PE=1 SV=1	AFVHWYVGEGMEEGEFSEAR
>	sp|P68370|TBA	Tubulin alpha-1A chain OS=Rattus norvegicus GN=Tuba1a PE=1 SV=1	AFVHWYVGEGMEEGEFSEAR
>	sp|P68370|TBA	Tubulin alpha-1A chain OS=Rattus norvegicus GN=Tuba1a PE=1 SV=1	AFVHWYVGEGMEEGEFSEAR
>	sp|P68370|TBA	Tubulin alpha-1A chain OS=Rattus norvegicus GN=Tuba1a PE=1 SV=1	AFVHWYVGEGMEEGEFSEAR
>	sp|P68370|TBA	Tubulin alpha-1A chain OS=Rattus norvegicus GN=Tuba1a PE=1 SV=1	AFVHWYVGEGMEEGEFSEAR
>	sp|P68370|TBA	Tubulin alpha-1A chain OS=Rattus norvegicus GN=Tuba1a PE=1 SV=1	AFVHWYVGEGMEEGEFSEAR
>	sp|P68370|TBA	Tubulin alpha-1A chain OS=Rattus norvegicus GN=Tuba1a PE=1 SV=1	AFVHWYVGEGMEEGEFSEAR
>	sp|P68370|TBA	Tubulin alpha-1A chain OS=Rattus norvegicus GN=Tuba1a PE=1 SV=1	AFVHWYVGEGMEEGEFSEAR
>	sp|P68370|TBA	Tubulin alpha-1A chain OS=Rattus norvegicus GN=Tuba1a PE=1 SV=1	AFVHWYVGEGMEEGEFSEAR
>	sp|P68370|TBA	Tubulin alpha-1A chain OS=Rattus norvegicus GN=Tuba1a PE=1 SV=1	AFVHWYVGEGMEEGEFSEAREDMAALEK
>	sp|P68370|TBA	Tubulin alpha-1A chain OS=Rattus norvegicus GN=Tuba1a PE=1 SV=1	AFVHWYVGEGMEEGEFSEAREDMAALEK
>	sp|P68370|TBA	Tubulin alpha-1A chain OS=Rattus norvegicus GN=Tuba1a PE=1 SV=1	AFVHWYVGEGMEEGEFSEAREDMAALEK
>	sp|P68370|TBA	Tubulin alpha-1A chain OS=Rattus norvegicus GN=Tuba1a PE=1 SV=1	AFVHWYVGEGMEEGEFSEAREDMAALEK
>	sp|P68370|TBA	Tubulin alpha-1A chain OS=Rattus norvegicus GN=Tuba1a PE=1 SV=1	AVCMLSNTTAIAEAWAR
>	sp|P68370|TBA	Tubulin alpha-1A chain OS=Rattus norvegicus GN=Tuba1a PE=1 SV=1	AVCMLSNTTAIAEAWAR
>	sp|P68370|TBA	Tubulin alpha-1A chain OS=Rattus norvegicus GN=Tuba1a PE=1 SV=1	AVCMLSNTTAIAEAWAR
>	sp|P68370|TBA	Tubulin alpha-1A chain OS=Rattus norvegicus GN=Tuba1a PE=1 SV=1	AVCMLSNTTAIAEAWAR
>	sp|P68370|TBA	Tubulin alpha-1A chain OS=Rattus norvegicus GN=Tuba1a PE=1 SV=1	AVCMLSNTTAIAEAWAR
>	sp|P68370|TBA	Tubulin alpha-1A chain OS=Rattus norvegicus GN=Tuba1a PE=1 SV=1	AVCMLSNTTAIAEAWAR
>	sp|P68370|TBA	Tubulin alpha-1A chain OS=Rattus norvegicus GN=Tuba1a PE=1 SV=1	AVCMLSNTTAIAEAWAR
>	sp|P68370|TBA	Tubulin alpha-1A chain OS=Rattus norvegicus GN=Tuba1a PE=1 SV=1	AVCMLSNTTAIAEAWAR
>	sp|P68370|TBA	Tubulin alpha-1A chain OS=Rattus norvegicus GN=Tuba1a PE=1 SV=1	AVCMLSNTTAIAEAWAR
>	sp|P68370|TBA	Tubulin alpha-1A chain OS=Rattus norvegicus GN=Tuba1a PE=1 SV=1	AVCMLSNTTAIAEAWAR
>	sp|P68370|TBA	Tubulin alpha-1A chain OS=Rattus norvegicus GN=Tuba1a PE=1 SV=1	AVCMLSNTTAIAEAWAR
>	sp|P68370|TBA	Tubulin alpha-1A chain OS=Rattus norvegicus GN=Tuba1a PE=1 SV=1	AVCMLSNTTAIAEAWAR
>	sp|P68370|TBA	Tubulin alpha-1A chain OS=Rattus norvegicus GN=Tuba1a PE=1 SV=1	AVCMLSNTTAIAEAWAR
>	sp|P68370|TBA	Tubulin alpha-1A chain OS=Rattus norvegicus GN=Tuba1a PE=1 SV=1	AVCMLSNTTAIAEAWAR
>	sp|P68370|TBA	Tubulin alpha-1A chain OS=Rattus norvegicus GN=Tuba1a PE=1 SV=1	AVCMLSNTTAIAEAWAR
>	sp|P68370|TBA	Tubulin alpha-1A chain OS=Rattus norvegicus GN=Tuba1a PE=1 SV=1	AVCMLSNTTAIAEAWAR
>	sp|P68370|TBA	Tubulin alpha-1A chain OS=Rattus norvegicus GN=Tuba1a PE=1 SV=1	AVFVDLEPTVIDEVR
>	sp|P68370|TBA	Tubulin alpha-1A chain OS=Rattus norvegicus GN=Tuba1a PE=1 SV=1	AVFVDLEPTVIDEVR
>	sp|P68370|TBA	Tubulin alpha-1A chain OS=Rattus norvegicus GN=Tuba1a PE=1 SV=1	AVFVDLEPTVIDEVR
>	sp|P68370|TBA	Tubulin alpha-1A chain OS=Rattus norvegicus GN=Tuba1a PE=1 SV=1	AVFVDLEPTVIDEVR
>	sp|P68370|TBA	Tubulin alpha-1A chain OS=Rattus norvegicus GN=Tuba1a PE=1 SV=1	AVFVDLEPTVIDEVR
>	sp|P68370|TBA	Tubulin alpha-1A chain OS=Rattus norvegicus GN=Tuba1a PE=1 SV=1	AVFVDLEPTVIDEVR
>	sp|P68370|TBA	Tubulin alpha-1A chain OS=Rattus norvegicus GN=Tuba1a PE=1 SV=1	AVFVDLEPTVIDEVR
>	sp|P68370|TBA	Tubulin alpha-1A chain OS=Rattus norvegicus GN=Tuba1a PE=1 SV=1	AYHEQLSVAEITN
>	sp|P68370|TBA	Tubulin alpha-1A chain OS=Rattus norvegicus GN=Tuba1a PE=1 SV=1	AYHEQLSVAEITNACFEPANQMVK
>	sp|P68370|TBA	Tubulin alpha-1A chain OS=Rattus norvegicus GN=Tuba1a PE=1 SV=1	AYHEQLSVAEITNACFEPANQMVK
>	sp|P68370|TBA	Tubulin alpha-1A chain OS=Rattus norvegicus GN=Tuba1a PE=1 SV=1	AYHEQLSVAEITNACFEPANQMVK
>	sp|P68370|TBA	Tubulin alpha-1A chain OS=Rattus norvegicus GN=Tuba1a PE=1 SV=1	AYHEQLSVAEITNACFEPANQMVK
>	sp|P68370|TBA	Tubulin alpha-1A chain OS=Rattus norvegicus GN=Tuba1a PE=1 SV=1	AYHEQLSVAEITNACFEPANQMVK
>	sp|P68370|TBA	Tubulin alpha-1A chain OS=Rattus norvegicus GN=Tuba1a PE=1 SV=1	AYHEQLSVAEITNACFEPANQMVK
>	sp|P68370|TBA	Tubulin alpha-1A chain OS=Rattus norvegicus GN=Tuba1a PE=1 SV=1	AYHEQLSVAEITNACFEPANQMVK
>	sp|P68370|TBA	Tubulin alpha-1A chain OS=Rattus norvegicus GN=Tuba1a PE=1 SV=1	AYHEQLSVAEITNACFEPANQMVK
>	sp|P68370|TBA	Tubulin alpha-1A chain OS=Rattus norvegicus GN=Tuba1a PE=1 SV=1	AYHEQLSVAEITNACFEPANQMVK
>	sp|P68370|TBA	Tubulin alpha-1A chain OS=Rattus norvegicus GN=Tuba1a PE=1 SV=1	AYHEQLSVAEITNACFEPANQMVK
>	sp|P68370|TBA	Tubulin alpha-1A chain OS=Rattus norvegicus GN=Tuba1a PE=1 SV=1	AYHEQLSVAEITNACFEPANQMVK
>	sp|P68370|TBA	Tubulin alpha-1A chain OS=Rattus norvegicus GN=Tuba1a PE=1 SV=1	AYHEQLSVAEITNACFEPANQMVK
>	sp|P68370|TBA	Tubulin alpha-1A chain OS=Rattus norvegicus GN=Tuba1a PE=1 SV=1	AYHEQLSVAEITNACFEPANQMVK
>	sp|P68370|TBA	Tubulin alpha-1A chain OS=Rattus norvegicus GN=Tuba1a PE=1 SV=1	AYHEQLSVAEITNACFEPANQMVK
>	sp|P68370|TBA	Tubulin alpha-1A chain OS=Rattus norvegicus GN=Tuba1a PE=1 SV=1	AYHEQLSVAEITNACFEPANQMVK
>	sp|P68370|TBA	Tubulin alpha-1A chain OS=Rattus norvegicus GN=Tuba1a PE=1 SV=1	AYHEQLSVAEITNACFEPANQMVK
>	sp|P68370|TBA	Tubulin alpha-1A chain OS=Rattus norvegicus GN=Tuba1a PE=1 SV=1	AYHEQLSVAEITNACFEPANQMVK
>	sp|P68370|TBA	Tubulin alpha-1A chain OS=Rattus norvegicus GN=Tuba1a PE=1 SV=1	AYHEQLSVAEITNACFEPANQMVK

>	sp|P68370|TBA Tubulin alpha-1A chain OS=Rattus norvegicus GN=Tuba1a PE=1 SV=1	AYHEQLSVAEITNACFEPANQMVK
>	sp|P68370|TBA Tubulin alpha-1A chain OS=Rattus norvegicus GN=Tuba1a PE=1 SV=1	AYHEQLSVAEITNACFEPANQMVK
>	sp|P68370|TBA Tubulin alpha-1A chain OS=Rattus norvegicus GN=Tuba1a PE=1 SV=1	AYHEQLSVAEITNACFEPANQMVK
>	sp|P68370|TBA Tubulin alpha-1A chain OS=Rattus norvegicus GN=Tuba1a PE=1 SV=1	AYHEQLSVAEITNACFEPANQMVK
>	sp|P68370|TBA Tubulin alpha-1A chain OS=Rattus norvegicus GN=Tuba1a PE=1 SV=1	AYHEQLSVAEITNACFEPANQMVK
>	sp|P68370|TBA Tubulin alpha-1A chain OS=Rattus norvegicus GN=Tuba1a PE=1 SV=1	AYHEQLSVAEITNACFEPANQMVK
>	sp|P68370|TBA Tubulin alpha-1A chain OS=Rattus norvegicus GN=Tuba1a PE=1 SV=1	AYHEQLSVAEITNACFEPANQMVK
>	sp|P68370|TBA Tubulin alpha-1A chain OS=Rattus norvegicus GN=Tuba1a PE=1 SV=1	AYHEQLSVAEITNACFEPANQMVK
>	sp|P68370|TBA Tubulin alpha-1A chain OS=Rattus norvegicus GN=Tuba1a PE=1 SV=1	AYHEQLSVAEITNACFEPANQMVK
>	sp|P68370|TBA Tubulin alpha-1A chain OS=Rattus norvegicus GN=Tuba1a PE=1 SV=1	AYHEQLSVAEITNACFEPANQMVK
>	sp|P68370|TBA Tubulin alpha-1A chain OS=Rattus norvegicus GN=Tuba1a PE=1 SV=1	AYHEQLSVAEITNACFEPANQMVK
>	sp|P68370|TBA Tubulin alpha-1A chain OS=Rattus norvegicus GN=Tuba1a PE=1 SV=1	AYHEQLSVAEITNACFEPANQMVK
>	sp|P68370|TBA Tubulin alpha-1A chain OS=Rattus norvegicus GN=Tuba1a PE=1 SV=1	AYHEQLSVAEITNACFEPANQMVK
>	sp|P68370|TBA Tubulin alpha-1A chain OS=Rattus norvegicus GN=Tuba1a PE=1 SV=1	AYHEQLSVAEITNACFEPANQMVK
>	sp|P68370|TBA Tubulin alpha-1A chain OS=Rattus norvegicus GN=Tuba1a PE=1 SV=1	AYHEQLSVAEITNACFEPANQMVK
>	sp|P68370|TBA Tubulin alpha-1A chain OS=Rattus norvegicus GN=Tuba1a PE=1 SV=1	AYHEQLSVAEITNACFEPANQMVK
>	sp|P68370|TBA Tubulin alpha-1A chain OS=Rattus norvegicus GN=Tuba1a PE=1 SV=1	AYHEQLSVAEITNACFEPANQMVK
>	sp|P68370|TBA Tubulin alpha-1A chain OS=Rattus norvegicus GN=Tuba1a PE=1 SV=1	AYHEQLSVAEITNACFEPANQMVK
>	sp|P68370|TBA Tubulin alpha-1A chain OS=Rattus norvegicus GN=Tuba1a PE=1 SV=1	AYHEQLSVAEITNACFEPANQMVK
>	sp|P68370|TBA Tubulin alpha-1A chain OS=Rattus norvegicus GN=Tuba1a PE=1 SV=1	AYHEQLSVAEITNACFEPANQMVK
>	sp|P68370|TBA Tubulin alpha-1A chain OS=Rattus norvegicus GN=Tuba1a PE=1 SV=1	AYHEQLSVAEITNACFEPANQMVK
>	sp|P68370|TBA Tubulin alpha-1A chain OS=Rattus norvegicus GN=Tuba1a PE=1 SV=1	AYHEQLSVAEITNACFEPANQMVK
>	sp|P68370|TBA Tubulin alpha-1A chain OS=Rattus norvegicus GN=Tuba1a PE=1 SV=1	AYHEQLSVAEITNACFEPANQMVK
>	sp|P68370|TBA Tubulin alpha-1A chain OS=Rattus norvegicus GN=Tuba1a PE=1 SV=1	AYHEQLSVAEITNACFEPANQMVK
>	sp|P68370|TBA Tubulin alpha-1A chain OS=Rattus norvegicus GN=Tuba1a PE=1 SV=1	AYHEQLSVAEITNACFEPANQMVK
>	sp|P68370|TBA Tubulin alpha-1A chain OS=Rattus norvegicus GN=Tuba1a PE=1 SV=1	DVNAAIATIK
>	sp|P68370|TBA Tubulin alpha-1A chain OS=Rattus norvegicus GN=Tuba1a PE=1 SV=1	DVNAAIATIK
>	sp|P68370|TBA Tubulin alpha-1A chain OS=Rattus norvegicus GN=Tuba1a PE=1 SV=1	DVNAAIATIK
>	sp|P68370|TBA Tubulin alpha-1A chain OS=Rattus norvegicus GN=Tuba1a PE=1 SV=1	DVNAAIATIK
>	sp|P68370|TBA Tubulin alpha-1A chain OS=Rattus norvegicus GN=Tuba1a PE=1 SV=1	DVNAAIATIK
>	sp|P68370|TBA Tubulin alpha-1A chain OS=Rattus norvegicus GN=Tuba1a PE=1 SV=1	ECISIHVGQAGVQIGNACWELYCLEHGIQPDGQMPSDK
>	sp|P68370|TBA Tubulin alpha-1A chain OS=Rattus norvegicus GN=Tuba1a PE=1 SV=1	ECISIHVGQAGVQIGNACWELYCLEHGIQPDGQMPSDK
>	sp|P68370|TBA Tubulin alpha-1A chain OS=Rattus norvegicus GN=Tuba1a PE=1 SV=1	ECISIHVGQAGVQIGNACWELYCLEHGIQPDGQMPSDK
>	sp|P68370|TBA Tubulin alpha-1A chain OS=Rattus norvegicus GN=Tuba1a PE=1 SV=1	ECISIHVGQAGVQIGNACWELYCLEHGIQPDGQMPSDK
>	sp|P68370|TBA Tubulin alpha-1A chain OS=Rattus norvegicus GN=Tuba1a PE=1 SV=1	ECISIHVGQAGVQIGNACWELYCLEHGIQPDGQMPSDK
>	sp|P68370|TBA Tubulin alpha-1A chain OS=Rattus norvegicus GN=Tuba1a PE=1 SV=1	ECISIHVGQAGVQIGNACWELYCLEHGIQPDGQMPSDK
>	sp|P68370|TBA Tubulin alpha-1A chain OS=Rattus norvegicus GN=Tuba1a PE=1 SV=1	ECISIHVGQAGVQIGNACWELYCLEHGIQPDGQMPSDK
>	sp|P68370|TBA Tubulin alpha-1A chain OS=Rattus norvegicus GN=Tuba1a PE=1 SV=1	ECISIHVGQAGVQIGNACWELYCLEHGIQPDGQMPSDK
>	sp|P68370|TBA Tubulin alpha-1A chain OS=Rattus norvegicus GN=Tuba1a PE=1 SV=1	ECISIHVGQAGVQIGNACWELYCLEHGIQPDGQMPSDK
>	sp|P68370|TBA Tubulin alpha-1A chain OS=Rattus norvegicus GN=Tuba1a PE=1 SV=1	ECISIHVGQAGVQIGNACWELYCLEHGIQPDGQMPSDK
>	sp|P68370|TBA Tubulin alpha-1A chain OS=Rattus norvegicus GN=Tuba1a PE=1 SV=1	ECISIHVGQAGVQIGNACWELYCLEHGIQPDGQMPSDK
>	sp|P68370|TBA Tubulin alpha-1A chain OS=Rattus norvegicus GN=Tuba1a PE=1 SV=1	ECISIHVGQAGVQIGNACWELYCLEHGIQPDGQMPSDK
>	sp|P68370|TBA Tubulin alpha-1A chain OS=Rattus norvegicus GN=Tuba1a PE=1 SV=1	ECISIHVGQAGVQIGNACWELYCLEHGIQPDGQMPSDK
>	sp|P68370|TBA Tubulin alpha-1A chain OS=Rattus norvegicus GN=Tuba1a PE=1 SV=1	ECISIHVGQAGVQIGNACWELYCLEHGIQPDGQMPSDK
>	sp|P68370|TBA Tubulin alpha-1A chain OS=Rattus norvegicus GN=Tuba1a PE=1 SV=1	ECISIHVGQAGVQIGNACWELYCLEHGIQPDGQMPSDK
>	sp|P68370|TBA Tubulin alpha-1A chain OS=Rattus norvegicus GN=Tuba1a PE=1 SV=1	ECISIHVGQAGVQIGNACWELYCLEHGIQPDGQMPSDK
>	sp|P68370|TBA Tubulin alpha-1A chain OS=Rattus norvegicus GN=Tuba1a PE=1 SV=1	ECISIHVGQAGVQIGNACWELYCLEHGIQPDGQMPSDK
>	sp|P68370|TBA Tubulin alpha-1A chain OS=Rattus norvegicus GN=Tuba1a PE=1 SV=1	ECISIHVGQAGVQIGNACWELYCLEHGIQPDGQMPSDK
>	sp|P68370|TBA Tubulin alpha-1A chain OS=Rattus norvegicus GN=Tuba1a PE=1 SV=1	ECISIHVGQAGVQIGNACWELYCLEHGIQPDGQMPSDK
>	sp|P68370|TBA Tubulin alpha-1A chain OS=Rattus norvegicus GN=Tuba1a PE=1 SV=1	ECISIHVGQAGVQIGNACWELYCLEHGIQPDGQMPSDK
>	sp|P68370|TBA Tubulin alpha-1A chain OS=Rattus norvegicus GN=Tuba1a PE=1 SV=1	ECISIHVGQAGVQIGNACWELYCLEHGIQPDGQMPSDK
>	sp|P68370|TBA Tubulin alpha-1A chain OS=Rattus norvegicus GN=Tuba1a PE=1 SV=1	ECISIHVGQAGVQIGNACWELYCLEHGIQPDGQMPSDK
>	sp|P68370|TBA Tubulin alpha-1A chain OS=Rattus norvegicus GN=Tuba1a PE=1 SV=1	ECISIHVGQAGVQIGNACWELYCLEHGIQPDGQMPSDK
>	sp|P68370|TBA Tubulin alpha-1A chain OS=Rattus norvegicus GN=Tuba1a PE=1 SV=1	ECISIHVGQAGVQIGNACWELYCLEHGIQPDGQMPSDK
>	sp|P68370|TBA Tubulin alpha-1A chain OS=Rattus norvegicus GN=Tuba1a PE=1 SV=1	ECISIHVGQAGVQIGNACWELYCLEHGIQPDGQMPSDK
>	sp|P68370|TBA Tubulin alpha-1A chain OS=Rattus norvegicus GN=Tuba1a PE=1 SV=1	ECISIHVGQAGVQIGNACWELYCLEHGIQPDGQMPSDK
>	sp|P68370|TBA Tubulin alpha-1A chain OS=Rattus norvegicus GN=Tuba1a PE=1 SV=1	ECISIHVGQAGVQIGNACWELYCLEHGIQPDGQMPSDK
>	sp|P68370|TBA Tubulin alpha-1A chain OS=Rattus norvegicus GN=Tuba1a PE=1 SV=1	ECISIHVGQAGVQIGNACWELYCLEHGIQPDGQMPSDK
>	sp|P68370|TBA Tubulin alpha-1A chain OS=Rattus norvegicus GN=Tuba1a PE=1 SV=1	ECISIHVGQAGVQIGNACWELYCLEHGIQPDGQMPSDK
>	sp|P68370|TBA Tubulin alpha-1A chain OS=Rattus norvegicus GN=Tuba1a PE=1 SV=1	ECISIHVGQAGVQIGNACWELYCLEHGIQPDGQMPSDK
>	sp|P68370|TBA Tubulin alpha-1A chain OS=Rattus norvegicus GN=Tuba1a PE=1 SV=1	ECISIHVGQAGVQIGNACWELYCLEHGIQPDGQMPSDK
>	sp|P68370|TBA Tubulin alpha-1A chain OS=Rattus norvegicus GN=Tuba1a PE=1 SV=1	ECISIHVGQAGVQIGNACWELYCLEHGIQPDGQMPSDK
>	sp|P68370|TBA Tubulin alpha-1A chain OS=Rattus norvegicus GN=Tuba1a PE=1 SV=1	ECISIHVGQAGVQIGNACWELYCLEHGIQPDGQMPSDK
>	sp|P68370|TBA Tubulin alpha-1A chain OS=Rattus norvegicus GN=Tuba1a PE=1 SV=1	ECISIHVGQAGVQIGNACWELYCLEHGIQPDGQMPSDK
>	sp|P68370|TBA Tubulin alpha-1A chain OS=Rattus norvegicus GN=Tuba1a PE=1 SV=1	ECISIHVGQAGVQIGNACWELYCLEHGIQPDGQMPSDK
>	sp|P68370|TBA Tubulin alpha-1A chain OS=Rattus norvegicus GN=Tuba1a PE=1 SV=1	ECISIHVGQAGVQIGNACWELYCLEHGIQPDGQMPSDK
>	sp|P68370|TBA Tubulin alpha-1A chain OS=Rattus norvegicus GN=Tuba1a PE=1 SV=1	ECISIHVGQAGVQIGNACWELYCLEHGIQPDGQMPSDK
>	sp|P68370|TBA Tubulin alpha-1A chain OS=Rattus norvegicus GN=Tuba1a PE=1 SV=1	ECISIHVGQAGVQIGNACWELYCLEHGIQPDGQMPSDK
>	sp|P68370|TBA Tubulin alpha-1A chain OS=Rattus norvegicus GN=Tuba1a PE=1 SV=1	ECISIHVGQAGVQIGNACWELYCLEHGIQPDGQMPSDK
>	sp|P68370|TBA Tubulin alpha-1A chain OS=Rattus norvegicus GN=Tuba1a PE=1 SV=1	ECISIHVGQAGVQIGNACWELYCLEHGIQPDGQMPSDK
>	sp|P68370|TBA Tubulin alpha-1A chain OS=Rattus norvegicus GN=Tuba1a PE=1 SV=1	ECISIHVGQAGVQIGNACWELYCLEHGIQPDGQMPSDK
>	sp|P68370|TBA Tubulin alpha-1A chain OS=Rattus norvegicus GN=Tuba1a PE=1 SV=1	ECISIHVGQAGVQIGNACWELYCLEHGIQPDGQMPSDK
>	sp|P68370|TBA Tubulin alpha-1A chain OS=Rattus norvegicus GN=Tuba1a PE=1 SV=1	ECISIHVGQAGVQIGNACWELYCLEHGIQPDGQMPSDK
>	sp|P68370|TBA Tubulin alpha-1A chain OS=Rattus norvegicus GN=Tuba1a PE=1 SV=1	ECISIHVGQAGVQIGNACWELYCLEHGIQPDGQMPSDKTIGGGDDSFNTFFSETGAGK

<table>
<tr><td>></td><td>sp|P68370|TBA</td><td>Tubulin alpha-1A chain OS=Rattus norvegicus GN=Tuba1a PE=1 SV=1</td><td>ECISIHVGQAGVQIGNACWELYCLEHGIQPDGQMPSDKTIGGGDDSFNTFFSETGAGK</td></tr>
<tr><td>></td><td>sp|P68370|TBA</td><td>Tubulin alpha-1A chain OS=Rattus norvegicus GN=Tuba1a PE=1 SV=1</td><td>ECISIHVGQAGVQIGNACWELYCLEHGIQPDGQMPSDKTIGGGDDSFNTFFSETGAGK</td></tr>
<tr><td>></td><td>sp|P68370|TBA</td><td>Tubulin alpha-1A chain OS=Rattus norvegicus GN=Tuba1a PE=1 SV=1</td><td>ECISIHVGQAGVQIGNACWELYCLEHGIQPDGQMPSDKTIGGGDDSFNTFFSETGAGK</td></tr>
<tr><td>></td><td>sp|P68370|TBA</td><td>Tubulin alpha-1A chain OS=Rattus norvegicus GN=Tuba1a PE=1 SV=1</td><td>ECISIHVGQAGVQIGNACWELYCLEHGIQPDGQMPSDKTIGGGDDSFNTFFSETGAGK</td></tr>
<tr><td>></td><td>sp|P68370|TBA</td><td>Tubulin alpha-1A chain OS=Rattus norvegicus GN=Tuba1a PE=1 SV=1</td><td>ECISIHVGQAGVQIGNACWELYCLEHGIQPDGQMPSDKTIGGGDDSFNTFFSETGAGK</td></tr>
<tr><td>></td><td>sp|P68370|TBA</td><td>Tubulin alpha-1A chain OS=Rattus norvegicus GN=Tuba1a PE=1 SV=1</td><td>ECISIHVGQAGVQIGNACWELYCLEHGIQPDGQMPSDKTIGGGDDSFNTFFSETGAGK</td></tr>
<tr><td>></td><td>sp|P68370|TBA</td><td>Tubulin alpha-1A chain OS=Rattus norvegicus GN=Tuba1a PE=1 SV=1</td><td>ECISIHVGQAGVQIGNACWELYCLEHGIQPDGQMPSDKTIGGGDDSFNTFFSETGAGK</td></tr>
<tr><td>></td><td>sp|P68370|TBA</td><td>Tubulin alpha-1A chain OS=Rattus norvegicus GN=Tuba1a PE=1 SV=1</td><td>ECISIHVGQAGVQIGNACWELYCLEHGIQPDGQMPSDKTIGGGDDSFNTFFSETGAGK</td></tr>
<tr><td>></td><td>sp|P68370|TBA</td><td>Tubulin alpha-1A chain OS=Rattus norvegicus GN=Tuba1a PE=1 SV=1</td><td>ECISIHVGQAGVQIGNACWELYCLEHGIQPDGQMPSDKTIGGGDDSFNTFFSETGAGK</td></tr>
<tr><td>></td><td>sp|P68370|TBA</td><td>Tubulin alpha-1A chain OS=Rattus norvegicus GN=Tuba1a PE=1 SV=1</td><td>ECISIHVGQAGVQIGNACWELYCLEHGIQPDGQMPSDKTIGGGDDSFNTFFSETGAGK</td></tr>
<tr><td>></td><td>sp|P68370|TBA</td><td>Tubulin alpha-1A chain OS=Rattus norvegicus GN=Tuba1a PE=1 SV=1</td><td>ECISIHVGQAGVQIGNACWELYCLEHGIQPDGQMPSDKTIGGGDDSFNTFFSETGAGK</td></tr>
<tr><td>></td><td>sp|P68370|TBA</td><td>Tubulin alpha-1A chain OS=Rattus norvegicus GN=Tuba1a PE=1 SV=1</td><td>ECISIHVGQAGVQIGNACWELYCLEHGIQPDGQMPSDKTIGGGDDSFNTFFSETGAGK</td></tr>
<tr><td>></td><td>sp|P68370|TBA</td><td>Tubulin alpha-1A chain OS=Rattus norvegicus GN=Tuba1a PE=1 SV=1</td><td>ECISIHVGQAGVQIGNACWELYCLEHGIQPDGQMPSDKTIGGGDDSFNTFFSETGAGK</td></tr>
<tr><td>></td><td>sp|P68370|TBA</td><td>Tubulin alpha-1A chain OS=Rattus norvegicus GN=Tuba1a PE=1 SV=1</td><td>ECISIHVGQAGVQIGNACWELYCLEHGIQPDGQMPSDKTIGGGDDSFNTFFSETGAGK</td></tr>
<tr><td>></td><td>sp|P68370|TBA</td><td>Tubulin alpha-1A chain OS=Rattus norvegicus GN=Tuba1a PE=1 SV=1</td><td>ECISIHVGQAGVQIGNACWELYCLEHGIQPDGQMPSDKTIGGGDDSFNTFFSETGAGK</td></tr>
<tr><td>></td><td>sp|P68370|TBA</td><td>Tubulin alpha-1A chain OS=Rattus norvegicus GN=Tuba1a PE=1 SV=1</td><td>ECISIHVGQAGVQIGNACWELYCLEHGIQPDGQMPSDKTIGGGDDSFNTFFSETGAGK</td></tr>
<tr><td>></td><td>sp|P68370|TBA</td><td>Tubulin alpha-1A chain OS=Rattus norvegicus GN=Tuba1a PE=1 SV=1</td><td>ECISIHVGQAGVQIGNACWELYCLEHGIQPDGQMPSDKTIGGGDDSFNTFFSETGAGK</td></tr>
<tr><td>></td><td>sp|P68370|TBA</td><td>Tubulin alpha-1A chain OS=Rattus norvegicus GN=Tuba1a PE=1 SV=1</td><td>EDAANNYAR</td></tr>
<tr><td>></td><td>sp|P68370|TBA</td><td>Tubulin alpha-1A chain OS=Rattus norvegicus GN=Tuba1a PE=1 SV=1</td><td>FDGALNVDLTEFQTNLVPYPR</td></tr>
<tr><td>></td><td>sp|P68370|TBA</td><td>Tubulin alpha-1A chain OS=Rattus norvegicus GN=Tuba1a PE=1 SV=1</td><td>FDGALNVDLTEFQTNLVPYPR</td></tr>
<tr><td>></td><td>sp|P68370|TBA</td><td>Tubulin alpha-1A chain OS=Rattus norvegicus GN=Tuba1a PE=1 SV=1</td><td>FDGALNVDLTEFQTNLVPYPR</td></tr>
<tr><td>></td><td>sp|P68370|TBA</td><td>Tubulin alpha-1A chain OS=Rattus norvegicus GN=Tuba1a PE=1 SV=1</td><td>FDGALNVDLTEFQTNLVPYPR</td></tr>
<tr><td>></td><td>sp|P68370|TBA</td><td>Tubulin alpha-1A chain OS=Rattus norvegicus GN=Tuba1a PE=1 SV=1</td><td>FDGALNVDLTEFQTNLVPYPR</td></tr>
<tr><td>></td><td>sp|P68370|TBA</td><td>Tubulin alpha-1A chain OS=Rattus norvegicus GN=Tuba1a PE=1 SV=1</td><td>FDGALNVDLTEFQTNLVPYPR</td></tr>
<tr><td>></td><td>sp|P68370|TBA</td><td>Tubulin alpha-1A chain OS=Rattus norvegicus GN=Tuba1a PE=1 SV=1</td><td>IHFPLATYAPVIS</td></tr>
<tr><td>></td><td>sp|P68370|TBA</td><td>Tubulin alpha-1A chain OS=Rattus norvegicus GN=Tuba1a PE=1 SV=1</td><td>IHFPLATYAPVISAEK</td></tr>
<tr><td>></td><td>sp|P68370|TBA</td><td>Tubulin alpha-1A chain OS=Rattus norvegicus GN=Tuba1a PE=1 SV=1</td><td>IHFPLATYAPVISAEK</td></tr>
<tr><td>></td><td>sp|P68370|TBA</td><td>Tubulin alpha-1A chain OS=Rattus norvegicus GN=Tuba1a PE=1 SV=1</td><td>IHFPLATYAPVISAEK</td></tr>
<tr><td>></td><td>sp|P68370|TBA</td><td>Tubulin alpha-1A chain OS=Rattus norvegicus GN=Tuba1a PE=1 SV=1</td><td>IHFPLATYAPVISAEK</td></tr>
<tr><td>></td><td>sp|P68370|TBA</td><td>Tubulin alpha-1A chain OS=Rattus norvegicus GN=Tuba1a PE=1 SV=1</td><td>IHFPLATYAPVISAEK</td></tr>
<tr><td>></td><td>sp|P68370|TBA</td><td>Tubulin alpha-1A chain OS=Rattus norvegicus GN=Tuba1a PE=1 SV=1</td><td>IHFPLATYAPVISAEK</td></tr>
<tr><td>></td><td>sp|P68370|TBA</td><td>Tubulin alpha-1A chain OS=Rattus norvegicus GN=Tuba1a PE=1 SV=1</td><td>IHFPLATYAPVISAEK</td></tr>
<tr><td>></td><td>sp|P68370|TBA</td><td>Tubulin alpha-1A chain OS=Rattus norvegicus GN=Tuba1a PE=1 SV=1</td><td>IHFPLATYAPVISAEK</td></tr>
<tr><td>></td><td>sp|P68370|TBA</td><td>Tubulin alpha-1A chain OS=Rattus norvegicus GN=Tuba1a PE=1 SV=1</td><td>IHFPLATYAPVISAEK</td></tr>
<tr><td>></td><td>sp|P68370|TBA</td><td>Tubulin alpha-1A chain OS=Rattus norvegicus GN=Tuba1a PE=1 SV=1</td><td>IHFPLATYAPVISAEK</td></tr>
<tr><td>></td><td>sp|P68370|TBA</td><td>Tubulin alpha-1A chain OS=Rattus norvegicus GN=Tuba1a PE=1 SV=1</td><td>IHFPLATYAPVISAEK</td></tr>
<tr><td>></td><td>sp|P68370|TBA</td><td>Tubulin alpha-1A chain OS=Rattus norvegicus GN=Tuba1a PE=1 SV=1</td><td>IHFPLATYAPVISAEK</td></tr>
<tr><td>></td><td>sp|P68370|TBA</td><td>Tubulin alpha-1A chain OS=Rattus norvegicus GN=Tuba1a PE=1 SV=1</td><td>IHFPLATYAPVISAEK</td></tr>
<tr><td>></td><td>sp|P68370|TBA</td><td>Tubulin alpha-1A chain OS=Rattus norvegicus GN=Tuba1a PE=1 SV=1</td><td>IHFPLATYAPVISAEK</td></tr>
<tr><td>></td><td>sp|P68370|TBA</td><td>Tubulin alpha-1A chain OS=Rattus norvegicus GN=Tuba1a PE=1 SV=1</td><td>IHFPLATYAPVISAEK</td></tr>
<tr><td>></td><td>sp|P68370|TBA</td><td>Tubulin alpha-1A chain OS=Rattus norvegicus GN=Tuba1a PE=1 SV=1</td><td>IHFPLATYAPVISAEK</td></tr>
<tr><td>></td><td>sp|P68370|TBA</td><td>Tubulin alpha-1A chain OS=Rattus norvegicus GN=Tuba1a PE=1 SV=1</td><td>IHFPLATYAPVISAEK</td></tr>
<tr><td>></td><td>sp|P68370|TBA</td><td>Tubulin alpha-1A chain OS=Rattus norvegicus GN=Tuba1a PE=1 SV=1</td><td>IHFPLATYAPVISAEK</td></tr>
<tr><td>></td><td>sp|P68370|TBA</td><td>Tubulin alpha-1A chain OS=Rattus norvegicus GN=Tuba1a PE=1 SV=1</td><td>IHFPLATYAPVISAEK</td></tr>
<tr><td>></td><td>sp|P68370|TBA</td><td>Tubulin alpha-1A chain OS=Rattus norvegicus GN=Tuba1a PE=1 SV=1</td><td>IHFPLATYAPVISAEK</td></tr>
<tr><td>></td><td>sp|P68370|TBA</td><td>Tubulin alpha-1A chain OS=Rattus norvegicus GN=Tuba1a PE=1 SV=1</td><td>IHFPLATYAPVISAEK</td></tr>
<tr><td>></td><td>sp|P68370|TBA</td><td>Tubulin alpha-1A chain OS=Rattus norvegicus GN=Tuba1a PE=1 SV=1</td><td>IHFPLATYAPVISAEK</td></tr>
<tr><td>></td><td>sp|P68370|TBA</td><td>Tubulin alpha-1A chain OS=Rattus norvegicus GN=Tuba1a PE=1 SV=1</td><td>IHFPLATYAPVISAEK</td></tr>
<tr><td>></td><td>sp|P68370|TBA</td><td>Tubulin alpha-1A chain OS=Rattus norvegicus GN=Tuba1a PE=1 SV=1</td><td>IHFPLATYAPVISAEK</td></tr>
<tr><td>></td><td>sp|P68370|TBA</td><td>Tubulin alpha-1A chain OS=Rattus norvegicus GN=Tuba1a PE=1 SV=1</td><td>IHFPLATYAPVISAEK</td></tr>
<tr><td>></td><td>sp|P68370|TBA</td><td>Tubulin alpha-1A chain OS=Rattus norvegicus GN=Tuba1a PE=1 SV=1</td><td>IHFPLATYAPVISAEK</td></tr>
<tr><td>></td><td>sp|P68370|TBA</td><td>Tubulin alpha-1A chain OS=Rattus norvegicus GN=Tuba1a PE=1 SV=1</td><td>IHFPLATYAPVISAEK</td></tr>
<tr><td>></td><td>sp|P68370|TBA</td><td>Tubulin alpha-1A chain OS=Rattus norvegicus GN=Tuba1a PE=1 SV=1</td><td>IHFPLATYAPVISAEK</td></tr>
<tr><td>></td><td>sp|P68370|TBA</td><td>Tubulin alpha-1A chain OS=Rattus norvegicus GN=Tuba1a PE=1 SV=1</td><td>IHFPLATYAPVISAEK</td></tr>
<tr><td>></td><td>sp|P68370|TBA</td><td>Tubulin alpha-1A chain OS=Rattus norvegicus GN=Tuba1a PE=1 SV=1</td><td>IHFPLATYAPVISAEK</td></tr>
<tr><td>></td><td>sp|P68370|TBA</td><td>Tubulin alpha-1A chain OS=Rattus norvegicus GN=Tuba1a PE=1 SV=1</td><td>IHFPLATYAPVISAEK</td></tr>
<tr><td>></td><td>sp|P68370|TBA</td><td>Tubulin alpha-1A chain OS=Rattus norvegicus GN=Tuba1a PE=1 SV=1</td><td>IHFPLATYAPVISAEK</td></tr>
<tr><td>></td><td>sp|P68370|TBA</td><td>Tubulin alpha-1A chain OS=Rattus norvegicus GN=Tuba1a PE=1 SV=1</td><td>IHFPLATYAPVISAEK</td></tr>
<tr><td>></td><td>sp|P68370|TBA</td><td>Tubulin alpha-1A chain OS=Rattus norvegicus GN=Tuba1a PE=1 SV=1</td><td>IHFPLATYAPVISAEK</td></tr>
<tr><td>></td><td>sp|P68370|TBA</td><td>Tubulin alpha-1A chain OS=Rattus norvegicus GN=Tuba1a PE=1 SV=1</td><td>IHFPLATYAPVISAEK</td></tr>
<tr><td>></td><td>sp|P68370|TBA</td><td>Tubulin alpha-1A chain OS=Rattus norvegicus GN=Tuba1a PE=1 SV=1</td><td>IHFPLATYAPVISAEK</td></tr>
<tr><td>></td><td>sp|P68370|TBA</td><td>Tubulin alpha-1A chain OS=Rattus norvegicus GN=Tuba1a PE=1 SV=1</td><td>IHFPLATYAPVISAEK</td></tr>
<tr><td>></td><td>sp|P68370|TBA</td><td>Tubulin alpha-1A chain OS=Rattus norvegicus GN=Tuba1a PE=1 SV=1</td><td>IHFPLATYAPVISAEK</td></tr>
<tr><td>></td><td>sp|P68370|TBA</td><td>Tubulin alpha-1A chain OS=Rattus norvegicus GN=Tuba1a PE=1 SV=1</td><td>IHFPLATYAPVISAEK</td></tr>
<tr><td>></td><td>sp|P68370|TBA</td><td>Tubulin alpha-1A chain OS=Rattus norvegicus GN=Tuba1a PE=1 SV=1</td><td>IHFPLATYAPVISAEK</td></tr>
<tr><td>></td><td>sp|P68370|TBA</td><td>Tubulin alpha-1A chain OS=Rattus norvegicus GN=Tuba1a PE=1 SV=1</td><td>IHFPLATYAPVISAEK</td></tr>
<tr><td>></td><td>sp|P68370|TBA</td><td>Tubulin alpha-1A chain OS=Rattus norvegicus GN=Tuba1a PE=1 SV=1</td><td>IHFPLATYAPVISAEK</td></tr>
<tr><td>></td><td>sp|P68370|TBA</td><td>Tubulin alpha-1A chain OS=Rattus norvegicus GN=Tuba1a PE=1 SV=1</td><td>IHFPLATYAPVISAEK</td></tr>
<tr><td>></td><td>sp|P68370|TBA</td><td>Tubulin alpha-1A chain OS=Rattus norvegicus GN=Tuba1a PE=1 SV=1</td><td>IHFPLATYAPVISAEK</td></tr>
<tr><td>></td><td>sp|P68370|TBA</td><td>Tubulin alpha-1A chain OS=Rattus norvegicus GN=Tuba1a PE=1 SV=1</td><td>IHFPLATYAPVISAEK</td></tr>
<tr><td>></td><td>sp|P68370|TBA</td><td>Tubulin alpha-1A chain OS=Rattus norvegicus GN=Tuba1a PE=1 SV=1</td><td>IHFPLATYAPVISAEK</td></tr>
<tr><td>></td><td>sp|P68370|TBA</td><td>Tubulin alpha-1A chain OS=Rattus norvegicus GN=Tuba1a PE=1 SV=1</td><td>IHFPLATYAPVISAEK</td></tr>
<tr><td>></td><td>sp|P68370|TBA</td><td>Tubulin alpha-1A chain OS=Rattus norvegicus GN=Tuba1a PE=1 SV=1</td><td>IHFPLATYAPVISAEK</td></tr>
<tr><td>></td><td>sp|P68370|TBA</td><td>Tubulin alpha-1A chain OS=Rattus norvegicus GN=Tuba1a PE=1 SV=1</td><td>IHFPLATYAPVISAEK</td></tr>
</table>

>	sp|P68370|TBA Tubulin alpha-1A chain OS=Rattus norvegicus GN=Tuba1a PE=1 SV=1	IHFPLATYAPVISAEK
>	sp|P68370|TBA Tubulin alpha-1A chain OS=Rattus norvegicus GN=Tuba1a PE=1 SV=1	IHFPLATYAPVISAEK
>	sp|P68370|TBA Tubulin alpha-1A chain OS=Rattus norvegicus GN=Tuba1a PE=1 SV=1	IHFPLATYAPVISAEK
>	sp|P68370|TBA Tubulin alpha-1A chain OS=Rattus norvegicus GN=Tuba1a PE=1 SV=1	IHFPLATYAPVISAEK
>	sp|P68370|TBA Tubulin alpha-1A chain OS=Rattus norvegicus GN=Tuba1a PE=1 SV=1	IHFPLATYAPVISAEK
>	sp|P68370|TBA Tubulin alpha-1A chain OS=Rattus norvegicus GN=Tuba1a PE=1 SV=1	IHFPLATYAPVISAEK
>	sp|P68370|TBA Tubulin alpha-1A chain OS=Rattus norvegicus GN=Tuba1a PE=1 SV=1	IHFPLATYAPVISAEK
>	sp|P68370|TBA Tubulin alpha-1A chain OS=Rattus norvegicus GN=Tuba1a PE=1 SV=1	IHFPLATYAPVISAEK
>	sp|P68370|TBA Tubulin alpha-1A chain OS=Rattus norvegicus GN=Tuba1a PE=1 SV=1	IHFPLATYAPVISAEK
>	sp|P68370|TBA Tubulin alpha-1A chain OS=Rattus norvegicus GN=Tuba1a PE=1 SV=1	IHFPLATYAPVISAEK
>	sp|P68370|TBA Tubulin alpha-1A chain OS=Rattus norvegicus GN=Tuba1a PE=1 SV=1	IHFPLATYAPVISAEK
>	sp|P68370|TBA Tubulin alpha-1A chain OS=Rattus norvegicus GN=Tuba1a PE=1 SV=1	IHFPLATYAPVISAEK
>	sp|P68370|TBA Tubulin alpha-1A chain OS=Rattus norvegicus GN=Tuba1a PE=1 SV=1	KLADQCTGLQGFLVFHSFGGGTGSGFTSLLMER
>	sp|P68370|TBA Tubulin alpha-1A chain OS=Rattus norvegicus GN=Tuba1a PE=1 SV=1	LDHKFDLMYAK
>	sp|P68370|TBA Tubulin alpha-1A chain OS=Rattus norvegicus GN=Tuba1a PE=1 SV=1	LDHKFDLMYAK
>	sp|P68370|TBA Tubulin alpha-1A chain OS=Rattus norvegicus GN=Tuba1a PE=1 SV=1	LDHKFDLMYAK
>	sp|P68370|TBA Tubulin alpha-1A chain OS=Rattus norvegicus GN=Tuba1a PE=1 SV=1	LEFSIYPAPQVSTAVVEPYNSILTTHTTLEHSDCAFMVDNEAIYDICR
>	sp|P68370|TBA Tubulin alpha-1A chain OS=Rattus norvegicus GN=Tuba1a PE=1 SV=1	LEFSIYPAPQVSTAVVEPYNSILTTHTTLEHSDCAFMVDNEAIYDICR
>	sp|P68370|TBA Tubulin alpha-1A chain OS=Rattus norvegicus GN=Tuba1a PE=1 SV=1	LEFSIYPAPQVSTAVVEPYNSILTTHTTLEHSDCAFMVDNEAIYDICR
>	sp|P68370|TBA Tubulin alpha-1A chain OS=Rattus norvegicus GN=Tuba1a PE=1 SV=1	LEFSIYPAPQVSTAVVEPYNSILTTHTTLEHSDCAFMVDNEAIYDICR
>	sp|P68370|TBA Tubulin alpha-1A chain OS=Rattus norvegicus GN=Tuba1a PE=1 SV=1	LEFSIYPAPQVSTAVVEPYNSILTTHTTLEHSDCAFMVDNEAIYDICR
>	sp|P68370|TBA Tubulin alpha-1A chain OS=Rattus norvegicus GN=Tuba1a PE=1 SV=1	LEFSIYPAPQVSTAVVEPYNSILTTHTTLEHSDCAFMVDNEAIYDICR
>	sp|P68370|TBA Tubulin alpha-1A chain OS=Rattus norvegicus GN=Tuba1a PE=1 SV=1	LEFSIYPAPQVSTAVVEPYNSILTTHTTLEHSDCAFMVDNEAIYDICR
>	sp|P68370|TBA Tubulin alpha-1A chain OS=Rattus norvegicus GN=Tuba1a PE=1 SV=1	LEFSIYPAPQVSTAVVEPYNSILTTHTTLEHSDCAFMVDNEAIYDICR
>	sp|P68370|TBA Tubulin alpha-1A chain OS=Rattus norvegicus GN=Tuba1a PE=1 SV=1	LEFSIYPAPQVSTAVVEPYNSILTTHTTLEHSDCAFMVDNEAIYDICR
>	sp|P68370|TBA Tubulin alpha-1A chain OS=Rattus norvegicus GN=Tuba1a PE=1 SV=1	LEFSIYPAPQVSTAVVEPYNSILTTHTTLEHSDCAFMVDNEAIYDICR
>	sp|P68370|TBA Tubulin alpha-1A chain OS=Rattus norvegicus GN=Tuba1a PE=1 SV=1	LEFSIYPAPQVSTAVVEPYNSILTTHTTLEHSDCAFMVDNEAIYDICR
>	sp|P68370|TBA Tubulin alpha-1A chain OS=Rattus norvegicus GN=Tuba1a PE=1 SV=1	LEFSIYPAPQVSTAVVEPYNSILTTHTTLEHSDCAFMVDNEAIYDICR
>	sp|P68370|TBA Tubulin alpha-1A chain OS=Rattus norvegicus GN=Tuba1a PE=1 SV=1	LEFSIYPAPQVSTAVVEPYNSILTTHTTLEHSDCAFMVDNEAIYDICR
>	sp|P68370|TBA Tubulin alpha-1A chain OS=Rattus norvegicus GN=Tuba1a PE=1 SV=1	LEFSIYPAPQVSTAVVEPYNSILTTHTTLEHSDCAFMVDNEAIYDICR
>	sp|P68370|TBA Tubulin alpha-1A chain OS=Rattus norvegicus GN=Tuba1a PE=1 SV=1	LEFSIYPAPQVSTAVVEPYNSILTTHTTLEHSDCAFMVDNEAIYDICR
>	sp|P68370|TBA Tubulin alpha-1A chain OS=Rattus norvegicus GN=Tuba1a PE=1 SV=1	LEFSIYPAPQVSTAVVEPYNSILTTHTTLEHSDCAFMVDNEAIYDICR
>	sp|P68370|TBA Tubulin alpha-1A chain OS=Rattus norvegicus GN=Tuba1a PE=1 SV=1	LEFSIYPAPQVSTAVVEPYNSILTTHTTLEHSDCAFMVDNEAIYDICR
>	sp|P68370|TBA Tubulin alpha-1A chain OS=Rattus norvegicus GN=Tuba1a PE=1 SV=1	LEFSIYPAPQVSTAVVEPYNSILTTHTTLEHSDCAFMVDNEAIYDICR
>	sp|P68370|TBA Tubulin alpha-1A chain OS=Rattus norvegicus GN=Tuba1a PE=1 SV=1	LEFSIYPAPQVSTAVVEPYNSILTTHTTLEHSDCAFMVDNEAIYDICR
>	sp|P68370|TBA Tubulin alpha-1A chain OS=Rattus norvegicus GN=Tuba1a PE=1 SV=1	LEFSIYPAPQVSTAVVEPYNSILTTHTTLEHSDCAFMVDNEAIYDICR
>	sp|P68370|TBA Tubulin alpha-1A chain OS=Rattus norvegicus GN=Tuba1a PE=1 SV=1	LEFSIYPAPQVSTAVVEPYNSILTTHTTLEHSDCAFMVDNEAIYDICR
>	sp|P68370|TBA Tubulin alpha-1A chain OS=Rattus norvegicus GN=Tuba1a PE=1 SV=1	LEFSIYPAPQVSTAVVEPYNSILTTHTTLEHSDCAFMVDNEAIYDICR
>	sp|P68370|TBA Tubulin alpha-1A chain OS=Rattus norvegicus GN=Tuba1a PE=1 SV=1	MRECISIHVGQAGVQIGNACWELYCLEHGIQPDGQMPSDK
>	sp|P68370|TBA Tubulin alpha-1A chain OS=Rattus norvegicus GN=Tuba1a PE=1 SV=1	NLDIERPTYTNLNR
>	sp|P68370|TBA Tubulin alpha-1A chain OS=Rattus norvegicus GN=Tuba1a PE=1 SV=1	NLDIERPTYTNLNRLIGQIVSSITASLR
>	sp|P68370|TBA Tubulin alpha-1A chain OS=Rattus norvegicus GN=Tuba1a PE=1 SV=1	NLDIERPTYTNLNRLIGQIVSSITASLRFDGALNVDLTEFQTNLVPYPR
>	sp|P68370|TBA Tubulin alpha-1A chain OS=Rattus norvegicus GN=Tuba1a PE=1 SV=1	PGGDLAK
>	sp|P68370|TBA Tubulin alpha-1A chain OS=Rattus norvegicus GN=Tuba1a PE=1 SV=1	PPTVVPGGDLAK
>	sp|P68370|TBA Tubulin alpha-1A chain OS=Rattus norvegicus GN=Tuba1a PE=1 SV=1	QLFHPEQLITGK
>	sp|P68370|TBA Tubulin alpha-1A chain OS=Rattus norvegicus GN=Tuba1a PE=1 SV=1	QLFHPEQLITGK
>	sp|P68370|TBA Tubulin alpha-1A chain OS=Rattus norvegicus GN=Tuba1a PE=1 SV=1	QLFHPEQLITGK
>	sp|P68370|TBA Tubulin alpha-1A chain OS=Rattus norvegicus GN=Tuba1a PE=1 SV=1	QLFHPEQLITGK
>	sp|P68370|TBA Tubulin alpha-1A chain OS=Rattus norvegicus GN=Tuba1a PE=1 SV=1	QLFHPEQLITGK
>	sp|P68370|TBA Tubulin alpha-1A chain OS=Rattus norvegicus GN=Tuba1a PE=1 SV=1	QLFHPEQLITGKEDAANNYAR
>	sp|P68370|TBA Tubulin alpha-1A chain OS=Rattus norvegicus GN=Tuba1a PE=1 SV=1	QLFHPEQLITGKEDAANNYAR
>	sp|P68370|TBA Tubulin alpha-1A chain OS=Rattus norvegicus GN=Tuba1a PE=1 SV=1	QLFHPEQLITGKEDAANNYAR
>	sp|P68370|TBA Tubulin alpha-1A chain OS=Rattus norvegicus GN=Tuba1a PE=1 SV=1	QLFHPEQLITGKEDAANNYAR
>	sp|P68370|TBA Tubulin alpha-1A chain OS=Rattus norvegicus GN=Tuba1a PE=1 SV=1	QLFHPEQLITGKEDAANNYAR
>	sp|P68370|TBA Tubulin alpha-1A chain OS=Rattus norvegicus GN=Tuba1a PE=1 SV=1	QLFHPEQLITGKEDAANNYAR
>	sp|P68370|TBA Tubulin alpha-1A chain OS=Rattus norvegicus GN=Tuba1a PE=1 SV=1	QLFHPEQLITGKEDAANNYAR
>	sp|P68370|TBA Tubulin alpha-1A chain OS=Rattus norvegicus GN=Tuba1a PE=1 SV=1	QLFHPEQLITGKEDAANNYAR
>	sp|P68370|TBA Tubulin alpha-1A chain OS=Rattus norvegicus GN=Tuba1a PE=1 SV=1	QLFHPEQLITGKEDAANNYAR
>	sp|P68370|TBA Tubulin alpha-1A chain OS=Rattus norvegicus GN=Tuba1a PE=1 SV=1	QLFHPEQLITGKEDAANNYAR
>	sp|P68370|TBA Tubulin alpha-1A chain OS=Rattus norvegicus GN=Tuba1a PE=1 SV=1	QLFHPEQLITGKEDAANNYAR
>	sp|P68370|TBA Tubulin alpha-1A chain OS=Rattus norvegicus GN=Tuba1a PE=1 SV=1	QLFHPEQLITGKEDAANNYAR
>	sp|P68370|TBA Tubulin alpha-1A chain OS=Rattus norvegicus GN=Tuba1a PE=1 SV=1	QLFHPEQLITGKEDAANNYAR
>	sp|P68370|TBA Tubulin alpha-1A chain OS=Rattus norvegicus GN=Tuba1a PE=1 SV=1	QLFHPEQLITGKEDAANNYAR
>	sp|P68370|TBA Tubulin alpha-1A chain OS=Rattus norvegicus GN=Tuba1a PE=1 SV=1	QLFHPEQLITGKEDAANNYAR
>	sp|P68370|TBA Tubulin alpha-1A chain OS=Rattus norvegicus GN=Tuba1a PE=1 SV=1	QLFHPEQLITGKEDAANNYAR
>	sp|P68370|TBA Tubulin alpha-1A chain OS=Rattus norvegicus GN=Tuba1a PE=1 SV=1	QLFHPEQLITGKEDAANNYAR
>	sp|P68370|TBA Tubulin alpha-1A chain OS=Rattus norvegicus GN=Tuba1a PE=1 SV=1	QLFHPEQLITGKEDAANNYAR
>	sp|P68370|TBA Tubulin alpha-1A chain OS=Rattus norvegicus GN=Tuba1a PE=1 SV=1	QLFHPEQLITGKEDAANNYAR
>	sp|P68370|TBA Tubulin alpha-1A chain OS=Rattus norvegicus GN=Tuba1a PE=1 SV=1	QLFHPEQLITGKEDAANNYAR
>	sp|P68370|TBA Tubulin alpha-1A chain OS=Rattus norvegicus GN=Tuba1a PE=1 SV=1	QLFHPEQLITGKEDAANNYAR
>	sp|P68370|TBA Tubulin alpha-1A chain OS=Rattus norvegicus GN=Tuba1a PE=1 SV=1	QLFHPEQLITGKEDAANNYAR
>	sp|P68370|TBA Tubulin alpha-1A chain OS=Rattus norvegicus GN=Tuba1a PE=1 SV=1	QLFHPEQLITGKEDAANNYAR
>	sp|P68370|TBA Tubulin alpha-1A chain OS=Rattus norvegicus GN=Tuba1a PE=1 SV=1	QLFHPEQLITGKEDAANNYAR
>	sp|P68370|TBA Tubulin alpha-1A chain OS=Rattus norvegicus GN=Tuba1a PE=1 SV=1	QLFHPEQLITGKEDAANNYAR

>	sp|P68370|TBA	Tubulin alpha-1A chain OS=Rattus norvegicus GN=Tuba1a PE=1 SV=1	QLFHPEQLITGKEDAANNYAR
>	sp|P68370|TBA	Tubulin alpha-1A chain OS=Rattus norvegicus GN=Tuba1a PE=1 SV=1	QLFHPEQLITGKEDAANNYAR
>	sp|P68370|TBA	Tubulin alpha-1A chain OS=Rattus norvegicus GN=Tuba1a PE=1 SV=1	QLFHPEQLITGKEDAANNYAR
>	sp|P68370|TBA	Tubulin alpha-1A chain OS=Rattus norvegicus GN=Tuba1a PE=1 SV=1	QLFHPEQLITGKEDAANNYAR
>	sp|P68370|TBA	Tubulin alpha-1A chain OS=Rattus norvegicus GN=Tuba1a PE=1 SV=1	QLFHPEQLITGKEDAANNYAR
>	sp|P68370|TBA	Tubulin alpha-1A chain OS=Rattus norvegicus GN=Tuba1a PE=1 SV=1	RAFVHWYVGEGMEEGEFSEAR
>	sp|P68370|TBA	Tubulin alpha-1A chain OS=Rattus norvegicus GN=Tuba1a PE=1 SV=1	RAFVHWYVGEGMEEGEFSEAR
>	sp|P68370|TBA	Tubulin alpha-1A chain OS=Rattus norvegicus GN=Tuba1a PE=1 SV=1	RAFVHWYVGEGMEEGEFSEAR
>	sp|P68370|TBA	Tubulin alpha-1A chain OS=Rattus norvegicus GN=Tuba1a PE=1 SV=1	RAFVHWYVGEGMEEGEFSEAR
>	sp|P68370|TBA	Tubulin alpha-1A chain OS=Rattus norvegicus GN=Tuba1a PE=1 SV=1	RECISIHVGQAGVQIGNACWELYCLEHGIQPDGQMPSDKTIGGGDDSFNTFFSETGAGK
>	sp|P68370|TBA	Tubulin alpha-1A chain OS=Rattus norvegicus GN=Tuba1a PE=1 SV=1	SKLEFSIYPAPQVSTAVVEPYNSILTTHTTLEHSDCAFMVDNEAIYDICR
>	sp|P68370|TBA	Tubulin alpha-1A chain OS=Rattus norvegicus GN=Tuba1a PE=1 SV=1	SKLEFSIYPAPQVSTAVVEPYNSILTTHTTLEHSDCAFMVDNEAIYDICR
>	sp|P68370|TBA	Tubulin alpha-1A chain OS=Rattus norvegicus GN=Tuba1a PE=1 SV=1	SKLEFSIYPAPQVSTAVVEPYNSILTTHTTLEHSDCAFMVDNEAIYDICR
>	sp|P68370|TBA	Tubulin alpha-1A chain OS=Rattus norvegicus GN=Tuba1a PE=1 SV=1	SKLEFSIYPAPQVSTAVVEPYNSILTTHTTLEHSDCAFMVDNEAIYDICR
>	sp|P68370|TBA	Tubulin alpha-1A chain OS=Rattus norvegicus GN=Tuba1a PE=1 SV=1	SKLEFSIYPAPQVSTAVVEPYNSILTTHTTLEHSDCAFMVDNEAIYDICR
>	sp|P68370|TBA	Tubulin alpha-1A chain OS=Rattus norvegicus GN=Tuba1a PE=1 SV=1	SKLEFSIYPAPQVSTAVVEPYNSILTTHTTLEHSDCAFMVDNEAIYDICR
>	sp|P68370|TBA	Tubulin alpha-1A chain OS=Rattus norvegicus GN=Tuba1a PE=1 SV=1	SKLEFSIYPAPQVSTAVVEPYNSILTTHTTLEHSDCAFMVDNEAIYDICR
>	sp|P68370|TBA	Tubulin alpha-1A chain OS=Rattus norvegicus GN=Tuba1a PE=1 SV=1	SKLEFSIYPAPQVSTAVVEPYNSILTTHTTLEHSDCAFMVDNEAIYDICR
>	sp|P68370|TBA	Tubulin alpha-1A chain OS=Rattus norvegicus GN=Tuba1a PE=1 SV=1	SKLEFSIYPAPQVSTAVVEPYNSILTTHTTLEHSDCAFMVDNEAIYDICR
>	sp|P68370|TBA	Tubulin alpha-1A chain OS=Rattus norvegicus GN=Tuba1a PE=1 SV=1	SKLEFSIYPAPQVSTAVVEPYNSILTTHTTLEHSDCAFMVDNEAIYDICR
>	sp|P68370|TBA	Tubulin alpha-1A chain OS=Rattus norvegicus GN=Tuba1a PE=1 SV=1	SKLEFSIYPAPQVSTAVVEPYNSILTTHTTLEHSDCAFMVDNEAIYDICR
>	sp|P68370|TBA	Tubulin alpha-1A chain OS=Rattus norvegicus GN=Tuba1a PE=1 SV=1	SKLEFSIYPAPQVSTAVVEPYNSILTTHTTLEHSDCAFMVDNEAIYDICR
>	sp|P68370|TBA	Tubulin alpha-1A chain OS=Rattus norvegicus GN=Tuba1a PE=1 SV=1	SKLEFSIYPAPQVSTAVVEPYNSILTTHTTLEHSDCAFMVDNEAIYDICR
>	sp|P68370|TBA	Tubulin alpha-1A chain OS=Rattus norvegicus GN=Tuba1a PE=1 SV=1	SKLEFSIYPAPQVSTAVVEPYNSILTTHTTLEHSDCAFMVDNEAIYDICR
>	sp|P68370|TBA	Tubulin alpha-1A chain OS=Rattus norvegicus GN=Tuba1a PE=1 SV=1	SKLEFSIYPAPQVSTAVVEPYNSILTTHTTLEHSDCAFMVDNEAIYDICR
>	sp|P68370|TBA	Tubulin alpha-1A chain OS=Rattus norvegicus GN=Tuba1a PE=1 SV=1	SKLEFSIYPAPQVSTAVVEPYNSILTTHTTLEHSDCAFMVDNEAIYDICR
>	sp|P68370|TBA	Tubulin alpha-1A chain OS=Rattus norvegicus GN=Tuba1a PE=1 SV=1	SKLEFSIYPAPQVSTAVVEPYNSILTTHTTLEHSDCAFMVDNEAIYDICR
>	sp|P68370|TBA	Tubulin alpha-1A chain OS=Rattus norvegicus GN=Tuba1a PE=1 SV=1	SKLEFSIYPAPQVSTAVVEPYNSILTTHTTLEHSDCAFMVDNEAIYDICR
>	sp|P68370|TBA	Tubulin alpha-1A chain OS=Rattus norvegicus GN=Tuba1a PE=1 SV=1	SKLEFSIYPAPQVSTAVVEPYNSILTTHTTLEHSDCAFMVDNEAIYDICR
>	sp|P68370|TBA	Tubulin alpha-1A chain OS=Rattus norvegicus GN=Tuba1a PE=1 SV=1	SKLEFSIYPAPQVSTAVVEPYNSILTTHTTLEHSDCAFMVDNEAIYDICR
>	sp|P68370|TBA	Tubulin alpha-1A chain OS=Rattus norvegicus GN=Tuba1a PE=1 SV=1	SKLEFSIYPAPQVSTAVVEPYNSILTTHTTLEHSDCAFMVDNEAIYDICR
>	sp|P68370|TBA	Tubulin alpha-1A chain OS=Rattus norvegicus GN=Tuba1a PE=1 SV=1	SKLEFSIYPAPQVSTAVVEPYNSILTTHTTLEHSDCAFMVDNEAIYDICR
>	sp|P68370|TBA	Tubulin alpha-1A chain OS=Rattus norvegicus GN=Tuba1a PE=1 SV=1	SKLEFSIYPAPQVSTAVVEPYNSILTTHTTLEHSDCAFMVDNEAIYDICR
>	sp|P68370|TBA	Tubulin alpha-1A chain OS=Rattus norvegicus GN=Tuba1a PE=1 SV=1	SKLEFSIYPAPQVSTAVVEPYNSILTTHTTLEHSDCAFMVDNEAIYDICR
>	sp|P68370|TBA	Tubulin alpha-1A chain OS=Rattus norvegicus GN=Tuba1a PE=1 SV=1	SKLEFSIYPAPQVSTAVVEPYNSILTTHTTLEHSDCAFMVDNEAIYDICR
>	sp|P68370|TBA	Tubulin alpha-1A chain OS=Rattus norvegicus GN=Tuba1a PE=1 SV=1	SKLEFSIYPAPQVSTAVVEPYNSILTTHTTLEHSDCAFMVDNEAIYDICR
>	sp|P68370|TBA	Tubulin alpha-1A chain OS=Rattus norvegicus GN=Tuba1a PE=1 SV=1	SKLEFSIYPAPQVSTAVVEPYNSILTTHTTLEHSDCAFMVDNEAIYDICR
>	sp|P68370|TBA	Tubulin alpha-1A chain OS=Rattus norvegicus GN=Tuba1a PE=1 SV=1	SKLEFSIYPAPQVSTAVVEPYNSILTTHTTLEHSDCAFMVDNEAIYDICR
>	sp|P68370|TBA	Tubulin alpha-1A chain OS=Rattus norvegicus GN=Tuba1a PE=1 SV=1	SKLEFSIYPAPQVSTAVVEPYNSILTTHTTLEHSDCAFMVDNEAIYDICR
>	sp|P68370|TBA	Tubulin alpha-1A chain OS=Rattus norvegicus GN=Tuba1a PE=1 SV=1	SKLEFSIYPAPQVSTAVVEPYNSILTTHTTLEHSDCAFMVDNEAIYDICR
>	sp|P68370|TBA	Tubulin alpha-1A chain OS=Rattus norvegicus GN=Tuba1a PE=1 SV=1	SKLEFSIYPAPQVSTAVVEPYNSILTTHTTLEHSDCAFMVDNEAIYDICR
>	sp|P68370|TBA	Tubulin alpha-1A chain OS=Rattus norvegicus GN=Tuba1a PE=1 SV=1	SKLEFSIYPAPQVSTAVVEPYNSILTTHTTLEHSDCAFMVDNEAIYDICR
>	sp|P68370|TBA	Tubulin alpha-1A chain OS=Rattus norvegicus GN=Tuba1a PE=1 SV=1	SKLEFSIYPAPQVSTAVVEPYNSILTTHTTLEHSDCAFMVDNEAIYDICR
>	sp|P68370|TBA	Tubulin alpha-1A chain OS=Rattus norvegicus GN=Tuba1a PE=1 SV=1	SKLEFSIYPAPQVSTAVVEPYNSILTTHTTLEHSDCAFMVDNEAIYDICR
>	sp|P68370|TBA	Tubulin alpha-1A chain OS=Rattus norvegicus GN=Tuba1a PE=1 SV=1	SKLEFSIYPAPQVSTAVVEPYNSILTTHTTLEHSDCAFMVDNEAIYDICR
>	sp|P68370|TBA	Tubulin alpha-1A chain OS=Rattus norvegicus GN=Tuba1a PE=1 SV=1	SKLEFSIYPAPQVSTAVVEPYNSILTTHTTLEHSDCAFMVDNEAIYDICR
>	sp|P68370|TBA	Tubulin alpha-1A chain OS=Rattus norvegicus GN=Tuba1a PE=1 SV=1	SKLEFSIYPAPQVSTAVVEPYNSILTTHTTLEHSDCAFMVDNEAIYDICR
>	sp|P68370|TBA	Tubulin alpha-1A chain OS=Rattus norvegicus GN=Tuba1a PE=1 SV=1	SKLEFSIYPAPQVSTAVVEPYNSILTTHTTLEHSDCAFMVDNEAIYDICR
>	sp|P68370|TBA	Tubulin alpha-1A chain OS=Rattus norvegicus GN=Tuba1a PE=1 SV=1	SKLEFSIYPAPQVSTAVVEPYNSILTTHTTLEHSDCAFMVDNEAIYDICR
>	sp|P68370|TBA	Tubulin alpha-1A chain OS=Rattus norvegicus GN=Tuba1a PE=1 SV=1	SKLEFSIYPAPQVSTAVVEPYNSILTTHTTLEHSDCAFMVDNEAIYDICR
>	sp|P68370|TBA	Tubulin alpha-1A chain OS=Rattus norvegicus GN=Tuba1a PE=1 SV=1	TIGGGDDSFNTFFSETGAGK
>	sp|P68370|TBA	Tubulin alpha-1A chain OS=Rattus norvegicus GN=Tuba1a PE=1 SV=1	TIGGGDDSFNTFFSETGAGK
>	sp|P68370|TBA	Tubulin alpha-1A chain OS=Rattus norvegicus GN=Tuba1a PE=1 SV=1	TIGGGDDSFNTFFSETGAGK
>	sp|P68370|TBA	Tubulin alpha-1A chain OS=Rattus norvegicus GN=Tuba1a PE=1 SV=1	TIGGGDDSFNTFFSETGAGK
>	sp|P68370|TBA	Tubulin alpha-1A chain OS=Rattus norvegicus GN=Tuba1a PE=1 SV=1	TIGGGDDSFNTFFSETGAGK
>	sp|P68370|TBA	Tubulin alpha-1A chain OS=Rattus norvegicus GN=Tuba1a PE=1 SV=1	TIQFVDWCPTGFK
>	sp|P68370|TBA	Tubulin alpha-1A chain OS=Rattus norvegicus GN=Tuba1a PE=1 SV=1	TIQFVDWCPTGFK
>	sp|P68370|TBA	Tubulin alpha-1A chain OS=Rattus norvegicus GN=Tuba1a PE=1 SV=1	TIQFVDWCPTGFK
>	sp|P68370|TBA	Tubulin alpha-1A chain OS=Rattus norvegicus GN=Tuba1a PE=1 SV=1	TIQFVDWCPTGFK
>	sp|P68370|TBA	Tubulin alpha-1A chain OS=Rattus norvegicus GN=Tuba1a PE=1 SV=1	VGINYQPPTVVPGGDLAK
>	sp|P68370|TBA	Tubulin alpha-1A chain OS=Rattus norvegicus GN=Tuba1a PE=1 SV=1	VGINYQPPTVVPGGDLAK
>	sp|P68370|TBA	Tubulin alpha-1A chain OS=Rattus norvegicus GN=Tuba1a PE=1 SV=1	VGINYQPPTVVPGGDLAK
>	sp|P68370|TBA	Tubulin alpha-1A chain OS=Rattus norvegicus GN=Tuba1a PE=1 SV=1	VGINYQPPTVVPGGDLAK
>	sp|P68370|TBA	Tubulin alpha-1A chain OS=Rattus norvegicus GN=Tuba1a PE=1 SV=1	VGINYQPPTVVPGGDLAK
>	sp|P68370|TBA	Tubulin alpha-1A chain OS=Rattus norvegicus GN=Tuba1a PE=1 SV=1	VGINYQPPTVVPGGDLAK
>	sp|P68370|TBA	Tubulin alpha-1A chain OS=Rattus norvegicus GN=Tuba1a PE=1 SV=1	VGINYQPPTVVPGGDLAK
>	sp|P68370|TBA	Tubulin alpha-1A chain OS=Rattus norvegicus GN=Tuba1a PE=1 SV=1	VGINYQPPTVVPGGDLAK
>	sp|P68370|TBA	Tubulin alpha-1A chain OS=Rattus norvegicus GN=Tuba1a PE=1 SV=1	VGINYQPPTVVPGGDLAK
>	sp|P68370|TBA	Tubulin alpha-1A chain OS=Rattus norvegicus GN=Tuba1a PE=1 SV=1	VGINYQPPTVVPGGDLAK
>	sp|P68370|TBA	Tubulin alpha-1A chain OS=Rattus norvegicus GN=Tuba1a PE=1 SV=1	VGINYQPPTVVPGGDLAK
>	sp|P68370|TBA	Tubulin alpha-1A chain OS=Rattus norvegicus GN=Tuba1a PE=1 SV=1	VGINYQPPTVVPGGDLAK
>	sp|P68370|TBA	Tubulin alpha-1A chain OS=Rattus norvegicus GN=Tuba1a PE=1 SV=1	VGINYQPPTVVPGGDLAK
>	sp|P68370|TBA	Tubulin alpha-1A chain OS=Rattus norvegicus GN=Tuba1a PE=1 SV=1	YMACCLLYR
>	sp|P68370|TBA	Tubulin alpha-1A chain OS=Rattus norvegicus GN=Tuba1a PE=1 SV=1	YMACCLLYR

<table>
<tr><td>></td><td>sp|P68370|TBA</td><td>Tubulin alpha-1A chain OS=Rattus norvegicus GN=Tuba1a PE=1 SV=1</td><td>DVNAAIATIK</td></tr>
<tr><td>></td><td>sp|P68370|TBA</td><td>Tubulin alpha-1A chain OS=Rattus norvegicus GN=Tuba1a PE=1 SV=1</td><td>IHFPLATYAPVISAEK</td></tr>
<tr><td>></td><td>sp|P68370|TBA</td><td>Tubulin alpha-1A chain OS=Rattus norvegicus GN=Tuba1a PE=1 SV=1</td><td>AYHEQLSVAEITNACFEPANQMVK</td></tr>
<tr><td>></td><td>sp|P68370|TBA</td><td>Tubulin alpha-1A chain OS=Rattus norvegicus GN=Tuba1a PE=1 SV=1</td><td>ECISIHVGQAGVQIGNACWELYCLEHGIQPDGQMPSDK</td></tr>
<tr><td>></td><td>sp|P68370|TBA</td><td>Tubulin alpha-1A chain OS=Rattus norvegicus GN=Tuba1a PE=1 SV=1</td><td>IHFPLATYAPVISAEK</td></tr>
<tr><td>></td><td>sp|P68370|TBA</td><td>Tubulin alpha-1A chain OS=Rattus norvegicus GN=Tuba1a PE=1 SV=1</td><td>QLFHPEQLITGKEDAANNYAR</td></tr>
<tr><td>></td><td>sp|P68370|TBA</td><td>Tubulin alpha-1A chain OS=Rattus norvegicus GN=Tuba1a PE=1 SV=1</td><td>LEFSIYPAPQVSTAVVEPYNSILTTHTTLEHSDCAFMVDNEAIYDICR</td></tr>
<tr><td>></td><td>sp|P68370|TBA</td><td>Tubulin alpha-1A chain OS=Rattus norvegicus GN=Tuba1a PE=1 SV=1</td><td>SKLEFSIYPAPQVSTAVVEPYNSILTTHTTLEHSDCAFMVDNEAIYDICR</td></tr>
<tr><td>></td><td>sp|P68370|TBA</td><td>Tubulin alpha-1A chain OS=Rattus norvegicus GN=Tuba1a PE=1 SV=1</td><td>QLFHPEQLITGKEDAANNYAR</td></tr>
<tr><td>></td><td>sp|P68370|TBA</td><td>Tubulin alpha-1A chain OS=Rattus norvegicus GN=Tuba1a PE=1 SV=1</td><td>LSVDYGK</td></tr>
<tr><td>></td><td>sp|P68370|TBA</td><td>Tubulin alpha-1A chain OS=Rattus norvegicus GN=Tuba1a PE=1 SV=1</td><td>TIGGGDDSFNTFFSETGAGK</td></tr>
<tr><td>></td><td>sp|P68370|TBA</td><td>Tubulin alpha-1A chain OS=Rattus norvegicus GN=Tuba1a PE=1 SV=1</td><td>IHFPLATYAPVISAEK</td></tr>
<tr><td>></td><td>sp|P68370|TBA</td><td>Tubulin alpha-1A chain OS=Rattus norvegicus GN=Tuba1a PE=1 SV=1</td><td>TIQFVDWCPTGFK</td></tr>
<tr><td>></td><td>sp|P68370|TBA</td><td>Tubulin alpha-1A chain OS=Rattus norvegicus GN=Tuba1a PE=1 SV=1</td><td>NLDIERPTYTNLNRLIGQIVSSITASLR</td></tr>
<tr><td>></td><td>sp|P68370|TBA</td><td>Tubulin alpha-1A chain OS=Rattus norvegicus GN=Tuba1a PE=1 SV=1</td><td>QLFHPEQLITGKEDAANNYAR</td></tr>
<tr><td>></td><td>sp|P68370|TBA</td><td>Tubulin alpha-1A chain OS=Rattus norvegicus GN=Tuba1a PE=1 SV=1</td><td>VGINYQPPTVVPGGDLAK</td></tr>
<tr><td>></td><td>sp|P68370|TBA</td><td>Tubulin alpha-1A chain OS=Rattus norvegicus GN=Tuba1a PE=1 SV=1</td><td>IHFPLATYAPVISAE</td></tr>
<tr><td>></td><td>sp|P68370|TBA</td><td>Tubulin alpha-1A chain OS=Rattus norvegicus GN=Tuba1a PE=1 SV=1</td><td>IHFPLATYAPVISAEK</td></tr>
<tr><td>></td><td>sp|P68370|TBA</td><td>Tubulin alpha-1A chain OS=Rattus norvegicus GN=Tuba1a PE=1 SV=1</td><td>QLFHPEQLITGKEDAANNYAR</td></tr>
<tr><td>></td><td>sp|P68370|TBA</td><td>Tubulin alpha-1A chain OS=Rattus norvegicus GN=Tuba1a PE=1 SV=1</td><td>SKLEFSIYPAPQVSTAVVEPYNSILTTHTTLEHSDCAFMVDNEAIYDICR</td></tr>
<tr><td>></td><td>sp|P68370|TBA</td><td>Tubulin alpha-1A chain OS=Rattus norvegicus GN=Tuba1a PE=1 SV=1</td><td>KFDLMYAKRAFVHWYVGEGMEEGEFSEAR</td></tr>
<tr><td>></td><td>sp|P68370|TBA</td><td>Tubulin alpha-1A chain OS=Rattus norvegicus GN=Tuba1a PE=1 SV=1</td><td>KFDLMYAKRAFVHWYVGEGMEEGEFSEAR</td></tr>
<tr><td>></td><td>sp|P68370|TBA</td><td>Tubulin alpha-1A chain OS=Rattus norvegicus GN=Tuba1a PE=1 SV=1</td><td>IHFPLATYAPVISAEK</td></tr>
<tr><td>></td><td>sp|P68370|TBA</td><td>Tubulin alpha-1A chain OS=Rattus norvegicus GN=Tuba1a PE=1 SV=1</td><td>AVCMLSNTTAIAEAWAR</td></tr>
<tr><td>></td><td>sp|P68370|TBA</td><td>Tubulin alpha-1A chain OS=Rattus norvegicus GN=Tuba1a PE=1 SV=1</td><td>AYHEQLSVAEITNACFEPANQMVK</td></tr>
<tr><td>></td><td>sp|P68370|TBA</td><td>Tubulin alpha-1A chain OS=Rattus norvegicus GN=Tuba1a PE=1 SV=1</td><td>VGINYQPPTVVPGGDLAK</td></tr>
<tr><td>></td><td>sp|P68370|TBA</td><td>Tubulin alpha-1A chain OS=Rattus norvegicus GN=Tuba1a PE=1 SV=1</td><td>SKLEFSIYPAPQVSTAVVEPYNSILTTHTTLEHSDCAFMVDNEAIYDICR</td></tr>
<tr><td>></td><td>sp|P68370|TBA</td><td>Tubulin alpha-1A chain OS=Rattus norvegicus GN=Tuba1a PE=1 SV=1</td><td>SKLEFSIYPAPQVSTAVVEPYNSILTTHTTLEHSDCAFMVDNEAIYDICR</td></tr>
<tr><td>></td><td>sp|P68370|TBA</td><td>Tubulin alpha-1A chain OS=Rattus norvegicus GN=Tuba1a PE=1 SV=1</td><td>VGINYQPPTVVPGGDLAK</td></tr>
<tr><td>></td><td>sp|P68370|TBA</td><td>Tubulin alpha-1A chain OS=Rattus norvegicus GN=Tuba1a PE=1 SV=1</td><td>AYHEQLSVAEITNACFEPANQMVK</td></tr>
<tr><td>></td><td>sp|P68370|TBA</td><td>Tubulin alpha-1A chain OS=Rattus norvegicus GN=Tuba1a PE=1 SV=1</td><td>VGINYQPPTVVPGGDLAK</td></tr>
<tr><td>></td><td>sp|P68370|TBA</td><td>Tubulin alpha-1A chain OS=Rattus norvegicus GN=Tuba1a PE=1 SV=1</td><td>QLFHPEQLITGK</td></tr>
<tr><td>></td><td>sp|P68370|TBA</td><td>Tubulin alpha-1A chain OS=Rattus norvegicus GN=Tuba1a PE=1 SV=1</td><td>TIGGGDDSFNTFFSETGAGK</td></tr>
<tr><td>></td><td>sp|P68370|TBA</td><td>Tubulin alpha-1A chain OS=Rattus norvegicus GN=Tuba1a PE=1 SV=1</td><td>YMACCLLYR</td></tr>
<tr><td>></td><td>sp|P68370|TBA</td><td>Tubulin alpha-1A chain OS=Rattus norvegicus GN=Tuba1a PE=1 SV=1</td><td>AYHEQLSVAEITNACFEPANQMVK</td></tr>
<tr><td>></td><td>sp|P68370|TBA</td><td>Tubulin alpha-1A chain OS=Rattus norvegicus GN=Tuba1a PE=1 SV=1</td><td>ECISIHVGQAGVQIGNACWELYCLEHGIQPDGQMPSDK</td></tr>
<tr><td>></td><td>sp|P68370|TBA</td><td>Tubulin alpha-1A chain OS=Rattus norvegicus GN=Tuba1a PE=1 SV=1</td><td>QLFHPEQLITGKEDAANNYAR</td></tr>
<tr><td>></td><td>sp|P68370|TBA</td><td>Tubulin alpha-1A chain OS=Rattus norvegicus GN=Tuba1a PE=1 SV=1</td><td>QLFHPEQLITGKEDAANNYAR</td></tr>
<tr><td>></td><td>sp|P68370|TBA</td><td>Tubulin alpha-1A chain OS=Rattus norvegicus GN=Tuba1a PE=1 SV=1</td><td>QLFHPEQLITGKEDAANNYAR</td></tr>
<tr><td>></td><td>sp|P68370|TBA</td><td>Tubulin alpha-1A chain OS=Rattus norvegicus GN=Tuba1a PE=1 SV=1</td><td>AYHEQLSVAEITNACFEPANQMVK</td></tr>
<tr><td>></td><td>sp|P68370|TBA</td><td>Tubulin alpha-1A chain OS=Rattus norvegicus GN=Tuba1a PE=1 SV=1</td><td>LEFSIYPAPQVSTAVVEPYNSILTTHTTLEHSDCAFMVDNEAIYDICR</td></tr>
<tr><td>></td><td>sp|P68370|TBA</td><td>Tubulin alpha-1A chain OS=Rattus norvegicus GN=Tuba1a PE=1 SV=1</td><td>RECISIHVGQAGVQIGNACWELYCLEHGIQPDGQMPSDKTIGGGDDSFNTFFSETGAGK</td></tr>
<tr><td>></td><td>sp|P68370|TBA</td><td>Tubulin alpha-1A chain OS=Rattus norvegicus GN=Tuba1a PE=1 SV=1</td><td>FDGALNVDLTEFQTNLVPYPR</td></tr>
<tr><td>></td><td>sp|P68370|TBA</td><td>Tubulin alpha-1A chain OS=Rattus norvegicus GN=Tuba1a PE=1 SV=1</td><td>QLFHPEQLITGKEDAANNYAR</td></tr>
<tr><td>></td><td>sp|P68370|TBA</td><td>Tubulin alpha-1A chain OS=Rattus norvegicus GN=Tuba1a PE=1 SV=1</td><td>RECISIHVGQAGVQIGNACWELYCLEHGIQPDGQMPSDKTIGGGDDSFNTFFSETGAGK</td></tr>
<tr><td>></td><td>sp|P68370|TBA</td><td>Tubulin alpha-1A chain OS=Rattus norvegicus GN=Tuba1a PE=1 SV=1</td><td>QLFHPEQLITGKEDAANNYAR</td></tr>
<tr><td>></td><td>sp|P68370|TBA</td><td>Tubulin alpha-1A chain OS=Rattus norvegicus GN=Tuba1a PE=1 SV=1</td><td>YMACCLLYR</td></tr>
<tr><td>></td><td>sp|P68370|TBA</td><td>Tubulin alpha-1A chain OS=Rattus norvegicus GN=Tuba1a PE=1 SV=1</td><td>TIQFVDWCPTGFK</td></tr>
<tr><td>></td><td>sp|P68370|TBA</td><td>Tubulin alpha-1A chain OS=Rattus norvegicus GN=Tuba1a PE=1 SV=1</td><td>AVFVDLEPTVIDEVR</td></tr>
<tr><td>></td><td>sp|P68370|TBA</td><td>Tubulin alpha-1A chain OS=Rattus norvegicus GN=Tuba1a PE=1 SV=1</td><td>DVNAAIATIK</td></tr>
<tr><td>></td><td>sp|P68370|TBA</td><td>Tubulin alpha-1A chain OS=Rattus norvegicus GN=Tuba1a PE=1 SV=1</td><td>PPTVVPGGDLAK</td></tr>
<tr><td>></td><td>sp|P68370|TBA</td><td>Tubulin alpha-1A chain OS=Rattus norvegicus GN=Tuba1a PE=1 SV=1</td><td>VGINYQPPTVVPGGDLAK</td></tr>
<tr><td>></td><td>sp|P68370|TBA</td><td>Tubulin alpha-1A chain OS=Rattus norvegicus GN=Tuba1a PE=1 SV=1</td><td>AVFVDLE</td></tr>
<tr><td>></td><td>sp|P68370|TBA</td><td>Tubulin alpha-1A chain OS=Rattus norvegicus GN=Tuba1a PE=1 SV=1</td><td>RECISIHVGQAGVQIGNACWELYCLEHGIQPDGQMPSDKTIGGGDDSFNTFFSETGAGK</td></tr>
<tr><td>></td><td>sp|P68370|TBA</td><td>Tubulin alpha-1A chain OS=Rattus norvegicus GN=Tuba1a PE=1 SV=1</td><td>AVFVDLEPTVIDEVR</td></tr>
<tr><td>></td><td>sp|P68370|TBA</td><td>Tubulin alpha-1A chain OS=Rattus norvegicus GN=Tuba1a PE=1 SV=1</td><td>AVCMLSNTTAIAEAWAR</td></tr>
<tr><td>></td><td>sp|P68370|TBA</td><td>Tubulin alpha-1A chain OS=Rattus norvegicus GN=Tuba1a PE=1 SV=1</td><td>YMACCLLYR</td></tr>
<tr><td>></td><td>sp|P68370|TBA</td><td>Tubulin alpha-1A chain OS=Rattus norvegicus GN=Tuba1a PE=1 SV=1</td><td>AYHEQLSVAEITNACFEPANQMVK</td></tr>
<tr><td>></td><td>sp|P68370|TBA</td><td>Tubulin alpha-1A chain OS=Rattus norvegicus GN=Tuba1a PE=1 SV=1</td><td>AFVHWYVGEGMEEGEFSEAREDMAALEK</td></tr>
<tr><td>></td><td>sp|P68370|TBA</td><td>Tubulin alpha-1A chain OS=Rattus norvegicus GN=Tuba1a PE=1 SV=1</td><td>YMACCLLYR</td></tr>
<tr><td>></td><td>sp|P68370|TBA</td><td>Tubulin alpha-1A chain OS=Rattus norvegicus GN=Tuba1a PE=1 SV=1</td><td>FDGALNVDLTEFQTNLVPYPR</td></tr>
<tr><td>></td><td>sp|P68370|TBA</td><td>Tubulin alpha-1A chain OS=Rattus norvegicus GN=Tuba1a PE=1 SV=1</td><td>AFVHWYVGEGMEEGEFSEAR</td></tr>
<tr><td>></td><td>sp|P68370|TBA</td><td>Tubulin alpha-1A chain OS=Rattus norvegicus GN=Tuba1a PE=1 SV=1</td><td>MRECISIHVGQAGVQIGNACWELYCLEHGIQPDGQMPSDKTIGGGDDSFNTFFSETGAGK</td></tr>
<tr><td>></td><td>sp|P68370|TBA</td><td>Tubulin alpha-1A chain OS=Rattus norvegicus GN=Tuba1a PE=1 SV=1</td><td>AYHEQLSVAEITNACFEPANQMVK</td></tr>
<tr><td>></td><td>sp|P68370|TBA</td><td>Tubulin alpha-1A chain OS=Rattus norvegicus GN=Tuba1a PE=1 SV=1</td><td>AYHEQLSVAEITNACFEPANQMVK</td></tr>
<tr><td>></td><td>sp|P68370|TBA</td><td>Tubulin alpha-1A chain OS=Rattus norvegicus GN=Tuba1a PE=1 SV=1</td><td>IHFPLATYAPVISAEK</td></tr>
<tr><td>></td><td>sp|P68370|TBA</td><td>Tubulin alpha-1A chain OS=Rattus norvegicus GN=Tuba1a PE=1 SV=1</td><td>GDVVPK</td></tr>
<tr><td>></td><td>sp|P68370|TBA</td><td>Tubulin alpha-1A chain OS=Rattus norvegicus GN=Tuba1a PE=1 SV=1</td><td>AVFVDLEPTVIDEVR</td></tr>
<tr><td>></td><td>sp|P68370|TBA</td><td>Tubulin alpha-1A chain OS=Rattus norvegicus GN=Tuba1a PE=1 SV=1</td><td>AYHEQLSVAEITNACFEPANQMVK</td></tr>
<tr><td>></td><td>sp|P68370|TBA</td><td>Tubulin alpha-1A chain OS=Rattus norvegicus GN=Tuba1a PE=1 SV=1</td><td>YMACCLLYR</td></tr>
<tr><td>></td><td>sp|P68370|TBA</td><td>Tubulin alpha-1A chain OS=Rattus norvegicus GN=Tuba1a PE=1 SV=1</td><td>LEFSIYPAPQVSTAVVEPYNSILTTHTTLEHSDCAFMVDNEAIYDICR</td></tr>
<tr><td>></td><td>sp|P68370|TBA</td><td>Tubulin alpha-1A chain OS=Rattus norvegicus GN=Tuba1a PE=1 SV=1</td><td>LEFSIYPAPQVSTAVVEPYNSILTTHTTLEHSDCAFMVDNEAIYDICR</td></tr>
<tr><td>></td><td>sp|P68370|TBA</td><td>Tubulin alpha-1A chain OS=Rattus norvegicus GN=Tuba1a PE=1 SV=1</td><td>FDGALNVDLTEFQTNLVPYPR</td></tr>
<tr><td>></td><td>sp|P68370|TBA</td><td>Tubulin alpha-1A chain OS=Rattus norvegicus GN=Tuba1a PE=1 SV=1</td><td>ECISIHVGQAGVQIGNACWELYCLEHGIQPDGQMPSDK</td></tr>
</table>

>	sp|P68370|TBA	Tubulin alpha-1A chain OS=Rattus norvegicus GN=Tuba1a PE=1 SV=1	LDHKFDLMYAK
>	sp|P68370|TBA	Tubulin alpha-1A chain OS=Rattus norvegicus GN=Tuba1a PE=1 SV=1	QLFHPEQLITGKEDAANNYAR
>	sp|P68370|TBA	Tubulin alpha-1A chain OS=Rattus norvegicus GN=Tuba1a PE=1 SV=1	FDGALNVDLTEFQTNLVPYPR
>	sp|P68370|TBA	Tubulin alpha-1A chain OS=Rattus norvegicus GN=Tuba1a PE=1 SV=1	SKLEFSIYPAPQVSTAVVEPYNSILTTHTTLEHSDCAFMVDNEAIYDICR
>	sp|P68370|TBA	Tubulin alpha-1A chain OS=Rattus norvegicus GN=Tuba1a PE=1 SV=1	AVFVDLEPTVIDEVR
>	sp|P68370|TBA	Tubulin alpha-1A chain OS=Rattus norvegicus GN=Tuba1a PE=1 SV=1	PGGDLAK
>	sp|P68370|TBA	Tubulin alpha-1A chain OS=Rattus norvegicus GN=Tuba1a PE=1 SV=1	SKLEFSIYPAPQVSTAVVEPYNSILTTHTTLEHSDCAFMVDNEAIYDICR
>	sp|P68370|TBA	Tubulin alpha-1A chain OS=Rattus norvegicus GN=Tuba1a PE=1 SV=1	AYHEQLSVAEITNACFEPANQMVK
>	sp|P68370|TBA	Tubulin alpha-1A chain OS=Rattus norvegicus GN=Tuba1a PE=1 SV=1	QLFHPEQLITGKEDAANNYAR
>	sp|P68370|TBA	Tubulin alpha-1A chain OS=Rattus norvegicus GN=Tuba1a PE=1 SV=1	QLFHPEQLITGK
>	sp|P68370|TBA	Tubulin alpha-1A chain OS=Rattus norvegicus GN=Tuba1a PE=1 SV=1	AYHEQLSVAEITN
>	sp|P68370|TBA	Tubulin alpha-1A chain OS=Rattus norvegicus GN=Tuba1a PE=1 SV=1	ECISIHVGQAGVQIGNACWELYCLEHGIQPDGQMPSDK
>	sp|P68370|TBA	Tubulin alpha-1A chain OS=Rattus norvegicus GN=Tuba1a PE=1 SV=1	AYHEQLSVAEITNACFEPANQMVK
>	sp|P68370|TBA	Tubulin alpha-1A chain OS=Rattus norvegicus GN=Tuba1a PE=1 SV=1	IHFPLATYAPVIS
>	sp|P68370|TBA	Tubulin alpha-1A chain OS=Rattus norvegicus GN=Tuba1a PE=1 SV=1	AYHEQLSVAEITNACFEPANQMVK
>	sp|P68370|TBA	Tubulin alpha-1A chain OS=Rattus norvegicus GN=Tuba1a PE=1 SV=1	VGINYQPPTVVPGGDLAK
>	sp|P68370|TBA	Tubulin alpha-1A chain OS=Rattus norvegicus GN=Tuba1a PE=1 SV=1	AFVHWYVGEGMEEGEFSEAR
>	sp|P68370|TBA	Tubulin alpha-1A chain OS=Rattus norvegicus GN=Tuba1a PE=1 SV=1	VGINYQPPTVVPGGDLAK
>	sp|P68370|TBA	Tubulin alpha-1A chain OS=Rattus norvegicus GN=Tuba1a PE=1 SV=1	SKLEFSIYPAPQVSTAVVEPYNSILTTHTTLEHSDCAFMVDNEAIYDICR
>	sp|P68370|TBA	Tubulin alpha-1A chain OS=Rattus norvegicus GN=Tuba1a PE=1 SV=1	AVFVDLEPTVIDEVR
>	sp|P68370|TBA	Tubulin alpha-1A chain OS=Rattus norvegicus GN=Tuba1a PE=1 SV=1	TIGGGDDSFNTFFSETGAGK
>	sp|P68370|TBA	Tubulin alpha-1A chain OS=Rattus norvegicus GN=Tuba1a PE=1 SV=1	DVNAAIATIK
>	sp|P68370|TBA	Tubulin alpha-1A chain OS=Rattus norvegicus GN=Tuba1a PE=1 SV=1	TIGGGDDSFNTFFSETGAGK
>	sp|P68370|TBA	Tubulin alpha-1A chain OS=Rattus norvegicus GN=Tuba1a PE=1 SV=1	YMACCLLYR
>	sp|P68370|TBA	Tubulin alpha-1A chain OS=Rattus norvegicus GN=Tuba1a PE=1 SV=1	ECISIHVGQAGVQIGNACWELYCLEHGIQPDGQMPSDK
>	sp|P68370|TBA	Tubulin alpha-1A chain OS=Rattus norvegicus GN=Tuba1a PE=1 SV=1	AYHEQLSVAEITNACFEPANQMVK
>	sp|P68370|TBA	Tubulin alpha-1A chain OS=Rattus norvegicus GN=Tuba1a PE=1 SV=1	LSVDYGK
>	sp|P68370|TBA	Tubulin alpha-1A chain OS=Rattus norvegicus GN=Tuba1a PE=1 SV=1	SKLEFSIYPAPQVSTAVVEPYNSILTTHTTLEHSDCAFMVDNEAIYDICR
>	sp|P68370|TBA	Tubulin alpha-1A chain OS=Rattus norvegicus GN=Tuba1a PE=1 SV=1	AYHEQLSVAEITNACFEPANQMVK
>	sp|P68370|TBA	Tubulin alpha-1A chain OS=Rattus norvegicus GN=Tuba1a PE=1 SV=1	ECISIHVGQAGVQIGNACWELYCLEHGIQPDGQMPSDK
>	sp|P68370|TBA	Tubulin alpha-1A chain OS=Rattus norvegicus GN=Tuba1a PE=1 SV=1	QLFHPEQLITGKEDAANNYAR
>	sp|P68370|TBA	Tubulin alpha-1A chain OS=Rattus norvegicus GN=Tuba1a PE=1 SV=1	SKLEFSIYPAPQVSTAVVEPYNSILTTHTTLEHSDCAFMVDNEAIYDICR
>	sp|P68370|TBA	Tubulin alpha-1A chain OS=Rattus norvegicus GN=Tuba1a PE=1 SV=1	SKLEFSIYPAPQVSTAVVEPYNSILTTHTTLEHSDCAFMVDNEAIYDICR
>	sp|P68370|TBA	Tubulin alpha-1A chain OS=Rattus norvegicus GN=Tuba1a PE=1 SV=1	AVFVDLEPTVIDEVR
>	sp|P68370|TBA	Tubulin alpha-1A chain OS=Rattus norvegicus GN=Tuba1a PE=1 SV=1	AYHEQLSVAEITNACFEPANQMVK
>	sp|P68370|TBA	Tubulin alpha-1A chain OS=Rattus norvegicus GN=Tuba1a PE=1 SV=1	SKLEFSIYPAPQVSTAVVEPYNSILTTHTTLEHSDCAFMVDNEAIYDICR
>	sp|P68370|TBA	Tubulin alpha-1A chain OS=Rattus norvegicus GN=Tuba1a PE=1 SV=1	IHFPLATYAPVISA
>	sp|P68370|TBA	Tubulin alpha-1A chain OS=Rattus norvegicus GN=Tuba1a PE=1 SV=1	QLFHPEQLITGKEDAANNYAR
>	sp|P68370|TBA	Tubulin alpha-1A chain OS=Rattus norvegicus GN=Tuba1a PE=1 SV=1	ECISIHVGQAGVQIGNACWELYCLEHGIQPDGQMPSDK
>	sp|P68370|TBA	Tubulin alpha-1A chain OS=Rattus norvegicus GN=Tuba1a PE=1 SV=1	AFVHWYVGEGMEEGEFSEAR
>	sp|P68370|TBA	Tubulin alpha-1A chain OS=Rattus norvegicus GN=Tuba1a PE=1 SV=1	LSVDYGKK
>	sp|P68370|TBA	Tubulin alpha-1A chain OS=Rattus norvegicus GN=Tuba1a PE=1 SV=1	VGINYQPPTVVPGGDLAK
>	sp|P68370|TBA	Tubulin alpha-1A chain OS=Rattus norvegicus GN=Tuba1a PE=1 SV=1	LDHKFDLMYAK
>	sp|P68370|TBA	Tubulin alpha-1A chain OS=Rattus norvegicus GN=Tuba1a PE=1 SV=1	IHFPLATYAPVISA
>	sp|P68370|TBA	Tubulin alpha-1A chain OS=Rattus norvegicus GN=Tuba1a PE=1 SV=1	QLFHPEQLITGKEDAANNYAR
>	sp|P68370|TBA	Tubulin alpha-1A chain OS=Rattus norvegicus GN=Tuba1a PE=1 SV=1	AYHEQLSVAEITNACFEPANQMVK
>	sp|P68370|TBA	Tubulin alpha-1A chain OS=Rattus norvegicus GN=Tuba1a PE=1 SV=1	IHFPLATYAP
>	sp|P68370|TBA	Tubulin alpha-1A chain OS=Rattus norvegicus GN=Tuba1a PE=1 SV=1	ECISIHVGQAGVQIGNACWELYCLEHGIQPDGQMPSDK
>	sp|P68370|TBA	Tubulin alpha-1A chain OS=Rattus norvegicus GN=Tuba1a PE=1 SV=1	VGINYQPPTVVPGGDLAK
>	sp|P68370|TBA	Tubulin alpha-1A chain OS=Rattus norvegicus GN=Tuba1a PE=1 SV=1	IHFPLATYAPVISAEK
>	sp|P68370|TBA	Tubulin alpha-1A chain OS=Rattus norvegicus GN=Tuba1a PE=1 SV=1	AFVHWYVGEGMEEGEFSEAREDMAALEK
>	sp|P68370|TBA	Tubulin alpha-1A chain OS=Rattus norvegicus GN=Tuba1a PE=1 SV=1	QLFHPEQLITGKEDAANNYAR
>	sp|P68370|TBA	Tubulin alpha-1A chain OS=Rattus norvegicus GN=Tuba1a PE=1 SV=1	AVFVDLEPTVIDEVR
>	sp|P68370|TBA	Tubulin alpha-1A chain OS=Rattus norvegicus GN=Tuba1a PE=1 SV=1	MRECISIHVGQAGVQIGNACWELYCLEHGIQPDGQMPSDKTIGGGDDSFNTFFSETGAGK
>	sp|P68370|TBA	Tubulin alpha-1A chain OS=Rattus norvegicus GN=Tuba1a PE=1 SV=1	QLFHPEQLITGKEDAANNYAR
>	sp|P68370|TBA	Tubulin alpha-1A chain OS=Rattus norvegicus GN=Tuba1a PE=1 SV=1	AYHEQLSVAEITNACFEPANQMVK
>	sp|P68370|TBA	Tubulin alpha-1A chain OS=Rattus norvegicus GN=Tuba1a PE=1 SV=1	LEFSIYPAPQVSTAVVEPYNSILTTHTTLEHSDCAFMVDNEAIYDICR
>	sp|P68370|TBA	Tubulin alpha-1A chain OS=Rattus norvegicus GN=Tuba1a PE=1 SV=1	SKLEFSIYPAPQVSTAVVEPYNSILTTHTTLEHSDCAFMVDNEAIYDICR
>	sp|P68370|TBA	Tubulin alpha-1A chain OS=Rattus norvegicus GN=Tuba1a PE=1 SV=1	IHFPLATYAP
>	sp|P68370|TBA	Tubulin alpha-1A chain OS=Rattus norvegicus GN=Tuba1a PE=1 SV=1	TIQFVDWCPTGFK
>	sp|P68370|TBA	Tubulin alpha-1A chain OS=Rattus norvegicus GN=Tuba1a PE=1 SV=1	AVCMLSNTTAIAEAWAR
>	sp|P68370|TBA	Tubulin alpha-1A chain OS=Rattus norvegicus GN=Tuba1a PE=1 SV=1	SKLEFSIYPAPQVSTAVVEPYNSILTTHTTLEHSDCAFMVDNEAIYDICR
>	sp|P68370|TBA	Tubulin alpha-1A chain OS=Rattus norvegicus GN=Tuba1a PE=1 SV=1	RECISIHVGQAGVQIGNACWELYCLEHGIQPDGQMPSDKTIGGGDDSFNTFFSETGAGK
>	sp|P68370|TBA	Tubulin alpha-1A chain OS=Rattus norvegicus GN=Tuba1a PE=1 SV=1	QLFHPEQLITGKEDAANNYAR
>	sp|P68370|TBA	Tubulin alpha-1A chain OS=Rattus norvegicus GN=Tuba1a PE=1 SV=1	RECISIHVGQAGVQIGNACWELYCLEHGIQPDGQMPSDKTIGGGDDSFNTFFSETGAGK
>	sp|P68370|TBA	Tubulin alpha-1A chain OS=Rattus norvegicus GN=Tuba1a PE=1 SV=1	LEFSIYPAPQVSTAVVEPYNSILTTHTTLEHSDCAFMVDNEAIYDICR
>	sp|P68370|TBA	Tubulin alpha-1A chain OS=Rattus norvegicus GN=Tuba1a PE=1 SV=1	MRECISIHVGQAGVQIGNACWELYCLEHGIQPDGQMPSDKTIGGGDDSFNTFFSETGAGK
>	sp|P68370|TBA	Tubulin alpha-1A chain OS=Rattus norvegicus GN=Tuba1a PE=1 SV=1	ECISIHVGQAGVQIGNACWELYCLEHGIQPDGQMPSDKTIGGGDDSFNTFFSETGAGK
>	sp|P68370|TBA	Tubulin alpha-1A chain OS=Rattus norvegicus GN=Tuba1a PE=1 SV=1	ECISIHVGQAGVQIGNACWELYCLEHGIQPDGQMPSDK
>	sp|P68370|TBA	Tubulin alpha-1A chain OS=Rattus norvegicus GN=Tuba1a PE=1 SV=1	ECISIHVGQAGVQIGNACWELYCLEHGIQPDGQMPSDKTIGGGDDSFNTFFSETGAGK
>	sp|P68370|TBA	Tubulin alpha-1A chain OS=Rattus norvegicus GN=Tuba1a PE=1 SV=1	LDHKFDLMYAK
>	sp|P68370|TBA	Tubulin alpha-1A chain OS=Rattus norvegicus GN=Tuba1a PE=1 SV=1	AVCMLSNTTAIAEAWAR
>	sp|P68370|TBA	Tubulin alpha-1A chain OS=Rattus norvegicus GN=Tuba1a PE=1 SV=1	LEFSIYPAPQVSTAVVEPYNSILTTHTTLEHSDCAFMVDNEAIYDICR
>	sp|Q6P6R2|DLI	Dihydrolipoyl dehydrogenase, mitochondrial OS=Rattus norvegicus GN=Dld PE=1 SV=1	EANLAASFGKPINF

>	sp|Q6P6R2|DLI Dihydrolipoyl dehydrogenase, mitochondrial OS=Rattus norvegicus GN=Dld PE=1 SV=1	ILGAHILGPGAGEMVNEAALALEYGASCEDVAR
>	sp|Q6P6R2|DLI Dihydrolipoyl dehydrogenase, mitochondrial OS=Rattus norvegicus GN=Dld PE=1 SV=1	IPNIFAIGDVVAGPMLAHK
>	sp|Q6P6R2|DLI Dihydrolipoyl dehydrogenase, mitochondrial OS=Rattus norvegicus GN=Dld PE=1 SV=1	KSDGKIDVSVEAASGGK
>	sp|Q6P6R2|DLI Dihydrolipoyl dehydrogenase, mitochondrial OS=Rattus norvegicus GN=Dld PE=1 SV=1	SEEQLKEEGVEFK
>	sp|Q6P6R2|DLI Dihydrolipoyl dehydrogenase, mitochondrial OS=Rattus norvegicus GN=Dld PE=1 SV=1	RPFTQNLGLEELGIELDPK
>	sp|Q6P6R2|DLI Dihydrolipoyl dehydrogenase, mitochondrial OS=Rattus norvegicus GN=Dld PE=1 SV=1	VVHVNGFGK
>	sp|Q6P6R2|DLI Dihydrolipoyl dehydrogenase, mitochondrial OS=Rattus norvegicus GN=Dld PE=1 SV=1	SDQPIDADVTVIGSGPGGYVAAIK
>	sp|Q6P6R2|DLI Dihydrolipoyl dehydrogenase, mitochondrial OS=Rattus norvegicus GN=Dld PE=1 SV=1	ALTGGIAHLFK
>	sp|Q6P6R2|DLI Dihydrolipoyl dehydrogenase, mitochondrial OS=Rattus norvegicus GN=Dld PE=1 SV=1	AEDEGIICVEGMAGGAVHIDYNCVPSVIYTHPEVAWVGK
>	sp|Q6P6R2|DLI Dihydrolipoyl dehydrogenase, mitochondrial OS=Rattus norvegicus GN=Dld PE=1 SV=1	KVPEKLVVIGAGVIGVELGSVWQR
>	sp|Q6P6R2|DLI Dihydrolipoyl dehydrogenase, mitochondrial OS=Rattus norvegicus GN=Dld PE=1 SV=1	AEDEGIICVEGMAGGAVHIDYNCVPSVIYTHPEVAWVGK
>	sp|Q6P6R2|DLI Dihydrolipoyl dehydrogenase, mitochondrial OS=Rattus norvegicus GN=Dld PE=1 SV=1	AEDEGIICVEGMAGGAVHIDYNCVPSVIYTHPEVAWVGK
>	sp|Q6P6R2|DLI Dihydrolipoyl dehydrogenase, mitochondrial OS=Rattus norvegicus GN=Dld PE=1 SV=1	ALTGGIAHLFK
>	sp|Q6P6R2|DLI Dihydrolipoyl dehydrogenase, mitochondrial OS=Rattus norvegicus GN=Dld PE=1 SV=1	ALTGGIAHLFK
>	sp|Q6P6R2|DLI Dihydrolipoyl dehydrogenase, mitochondrial OS=Rattus norvegicus GN=Dld PE=1 SV=1	ALTGGIAHLFK
>	sp|Q6P6R2|DLI Dihydrolipoyl dehydrogenase, mitochondrial OS=Rattus norvegicus GN=Dld PE=1 SV=1	ILGAHILGPGAGEMVNEAALALEYGASCEDVAR
>	sp|Q6P6R2|DLI Dihydrolipoyl dehydrogenase, mitochondrial OS=Rattus norvegicus GN=Dld PE=1 SV=1	ILGAHILGPGAGEMVNEAALALEYGASCEDVAR
>	sp|Q6P6R2|DLI Dihydrolipoyl dehydrogenase, mitochondrial OS=Rattus norvegicus GN=Dld PE=1 SV=1	ILGAHILGPGAGEMVNEAALALEYGASCEDVAR
>	sp|Q6P6R2|DLI Dihydrolipoyl dehydrogenase, mitochondrial OS=Rattus norvegicus GN=Dld PE=1 SV=1	ILGAHILGPGAGEMVNEAALALEYGASCEDVAR
>	sp|Q6P6R2|DLI Dihydrolipoyl dehydrogenase, mitochondrial OS=Rattus norvegicus GN=Dld PE=1 SV=1	ILGAHILGPGAGEMVNEAALALEYGASCEDVAR
>	sp|Q6P6R2|DLI Dihydrolipoyl dehydrogenase, mitochondrial OS=Rattus norvegicus GN=Dld PE=1 SV=1	ILGAHILGPGAGEMVNEAALALEYGASCEDVAR
>	sp|Q6P6R2|DLI Dihydrolipoyl dehydrogenase, mitochondrial OS=Rattus norvegicus GN=Dld PE=1 SV=1	ILGAHILGPGAGEMVNEAALALEYGASCEDVAR
>	sp|Q6P6R2|DLI Dihydrolipoyl dehydrogenase, mitochondrial OS=Rattus norvegicus GN=Dld PE=1 SV=1	ILGAHILGPGAGEMVNEAALALEYGASCEDVAR
>	sp|Q6P6R2|DLI Dihydrolipoyl dehydrogenase, mitochondrial OS=Rattus norvegicus GN=Dld PE=1 SV=1	ILGAHILGPGAGEMVNEAALALEYGASCEDVAR
>	sp|Q6P6R2|DLI Dihydrolipoyl dehydrogenase, mitochondrial OS=Rattus norvegicus GN=Dld PE=1 SV=1	IPNIFAIGDVVAGPMLAHK
>	sp|Q6P6R2|DLI Dihydrolipoyl dehydrogenase, mitochondrial OS=Rattus norvegicus GN=Dld PE=1 SV=1	IPNIFAIGDVVAGPMLAHK
>	sp|Q6P6R2|DLI Dihydrolipoyl dehydrogenase, mitochondrial OS=Rattus norvegicus GN=Dld PE=1 SV=1	IPNIFAIGDVVAGPMLAHK
>	sp|Q6P6R2|DLI Dihydrolipoyl dehydrogenase, mitochondrial OS=Rattus norvegicus GN=Dld PE=1 SV=1	IPNIFAIGDVVAGPMLAHK
>	sp|Q6P6R2|DLI Dihydrolipoyl dehydrogenase, mitochondrial OS=Rattus norvegicus GN=Dld PE=1 SV=1	IPNIFAIGDVVAGPMLAHK
>	sp|Q6P6R2|DLI Dihydrolipoyl dehydrogenase, mitochondrial OS=Rattus norvegicus GN=Dld PE=1 SV=1	IPNIFAIGDVVAGPMLAHK
>	sp|Q6P6R2|DLI Dihydrolipoyl dehydrogenase, mitochondrial OS=Rattus norvegicus GN=Dld PE=1 SV=1	IPNIFAIGDVVAGPMLAHK
>	sp|Q6P6R2|DLI Dihydrolipoyl dehydrogenase, mitochondrial OS=Rattus norvegicus GN=Dld PE=1 SV=1	IPNIFAIGDVVAGPMLAHK
>	sp|Q6P6R2|DLI Dihydrolipoyl dehydrogenase, mitochondrial OS=Rattus norvegicus GN=Dld PE=1 SV=1	IPNIFAIGDVVAGPMLAHK
>	sp|Q6P6R2|DLI Dihydrolipoyl dehydrogenase, mitochondrial OS=Rattus norvegicus GN=Dld PE=1 SV=1	RPFTQNLGLEELGIELDPK
>	sp|Q6P6R2|DLI Dihydrolipoyl dehydrogenase, mitochondrial OS=Rattus norvegicus GN=Dld PE=1 SV=1	RPFTQNLGLEELGIELDPK
>	sp|Q6P6R2|DLI Dihydrolipoyl dehydrogenase, mitochondrial OS=Rattus norvegicus GN=Dld PE=1 SV=1	RPFTQNLGLEELGIELDPK
>	sp|Q6P6R2|DLI Dihydrolipoyl dehydrogenase, mitochondrial OS=Rattus norvegicus GN=Dld PE=1 SV=1	RPFTQNLGLEELGIELDPK
>	sp|Q6P6R2|DLI Dihydrolipoyl dehydrogenase, mitochondrial OS=Rattus norvegicus GN=Dld PE=1 SV=1	IPNIFAIGDVVAGPMLAHK
>	sp|Q6P6R2|DLI Dihydrolipoyl dehydrogenase, mitochondrial OS=Rattus norvegicus GN=Dld PE=1 SV=1	GPMLAHK
>	sp|Q6P6R2|DLI Dihydrolipoyl dehydrogenase, mitochondrial OS=Rattus norvegicus GN=Dld PE=1 SV=1	SEEQLKEEGVEFK
>	sp|Q6P6R2|DLI Dihydrolipoyl dehydrogenase, mitochondrial OS=Rattus norvegicus GN=Dld PE=1 SV=1	AEDEGIICVEGMAGGAVHIDYNCVPSVIYTHPEVAWVGK
>	sp|Q6P6R2|DLI Dihydrolipoyl dehydrogenase, mitochondrial OS=Rattus norvegicus GN=Dld PE=1 SV=1	EANLAASFGKPINF
>	sp|P45592|COI Cofilin-1 OS=Rattus norvegicus GN=Cfl1 PE=1 SV=3	AVLFCLSEDKK
>	sp|P45592|COI Cofilin-1 OS=Rattus norvegicus GN=Cfl1 PE=1 SV=3	KEDLVFIFWAPESAPLK
>	sp|P45592|COI Cofilin-1 OS=Rattus norvegicus GN=Cfl1 PE=1 SV=3	KLTGIKHELQANCYEEVKDR
>	sp|P45592|COI Cofilin-1 OS=Rattus norvegicus GN=Cfl1 PE=1 SV=3	LGGSAVISLEGKPL
>	sp|P45592|COI Cofilin-1 OS=Rattus norvegicus GN=Cfl1 PE=1 SV=3	NIILEEGKEILVGDVGQTVDDPYTTFVK
>	sp|P45592|COI Cofilin-1 OS=Rattus norvegicus GN=Cfl1 PE=1 SV=3	KAVLFCLSEDKK
>	sp|P45592|COI Cofilin-1 OS=Rattus norvegicus GN=Cfl1 PE=1 SV=3	ESKKEDLVFIFWAPESAPLK
>	sp|P45592|COI Cofilin-1 OS=Rattus norvegicus GN=Cfl1 PE=1 SV=3	AVLFCLSEDKK
>	sp|P45592|COI Cofilin-1 OS=Rattus norvegicus GN=Cfl1 PE=1 SV=3	AVLFCLSEDKK
>	sp|P45592|COI Cofilin-1 OS=Rattus norvegicus GN=Cfl1 PE=1 SV=3	ESKKEDLVFIFWAPESAPLK
>	sp|P45592|COI Cofilin-1 OS=Rattus norvegicus GN=Cfl1 PE=1 SV=3	ESKKEDLVFIFWAPESAPLK
>	sp|P45592|COI Cofilin-1 OS=Rattus norvegicus GN=Cfl1 PE=1 SV=3	KEDLVFIFWAPESAPLK
>	sp|P45592|COI Cofilin-1 OS=Rattus norvegicus GN=Cfl1 PE=1 SV=3	KEDLVFIFWAPESAPLK
>	sp|P45592|COI Cofilin-1 OS=Rattus norvegicus GN=Cfl1 PE=1 SV=3	KEDLVFIFWAPESAPLK
>	sp|P45592|COI Cofilin-1 OS=Rattus norvegicus GN=Cfl1 PE=1 SV=3	KEDLVFIFWAPESAPLK
>	sp|P45592|COI Cofilin-1 OS=Rattus norvegicus GN=Cfl1 PE=1 SV=3	KEDLVFIFWAPESAPLK
>	sp|P45592|COI Cofilin-1 OS=Rattus norvegicus GN=Cfl1 PE=1 SV=3	LGGSAVISLEGKPL
>	sp|P45592|COI Cofilin-1 OS=Rattus norvegicus GN=Cfl1 PE=1 SV=3	NIILEEGKEILVGDVGQTVDDPYTTFVK
>	sp|P45592|COI Cofilin-1 OS=Rattus norvegicus GN=Cfl1 PE=1 SV=3	NIILEEGKEILVGDVGQTVDDPYTTFVK
>	sp|P45592|COI Cofilin-1 OS=Rattus norvegicus GN=Cfl1 PE=1 SV=3	NIILEEGKEILVGDVGQTVDDPYTTFVK
>	sp|P45592|COI Cofilin-1 OS=Rattus norvegicus GN=Cfl1 PE=1 SV=3	NIILEEGKEILVGDVGQTVDDPYTTFVK
>	sp|P45592|COI Cofilin-1 OS=Rattus norvegicus GN=Cfl1 PE=1 SV=3	NIILEEGKEILVGDVGQTVDDPYTTFVK
>	sp|P45592|COI Cofilin-1 OS=Rattus norvegicus GN=Cfl1 PE=1 SV=3	NIILEEGKEILVGDVGQTVDDPYTTFVK
>	sp|P45592|COI Cofilin-1 OS=Rattus norvegicus GN=Cfl1 PE=1 SV=3	NIILEEGKEILVGDVGQTVDDPYTTFVK
>	sp|P45592|COI Cofilin-1 OS=Rattus norvegicus GN=Cfl1 PE=1 SV=3	NIILEEGKEILVGDVGQTVDDPYTTFVK
>	sp|P45592|COI Cofilin-1 OS=Rattus norvegicus GN=Cfl1 PE=1 SV=3	NIILEEGKEILVGDVGQTVDDPYTTFVK
>	sp|P45592|COI Cofilin-1 OS=Rattus norvegicus GN=Cfl1 PE=1 SV=3	KEDLVFIFWAPESAPLK
>	sp|P45592|COI Cofilin-1 OS=Rattus norvegicus GN=Cfl1 PE=1 SV=3	AVLFCLSEDKK
>	sp|P45592|COI Cofilin-1 OS=Rattus norvegicus GN=Cfl1 PE=1 SV=3	PESAPLK
>	sp|P45592|COI Cofilin-1 OS=Rattus norvegicus GN=Cfl1 PE=1 SV=3	LGGSAVISLEGKPL
>	sp|P45592|COI Cofilin-1 OS=Rattus norvegicus GN=Cfl1 PE=1 SV=3	ESKKEDLVFIFWAPESAPLK
>	sp|P45592|COI Cofilin-1 OS=Rattus norvegicus GN=Cfl1 PE=1 SV=3	AVLFCLSEDKK

> sp|P45592|COI Cofilin-1 OS=Rattus norvegicus GN=Cfl1 PE=1 SV=3 KAVLFCLSEDKK
> sp|P45592|COI Cofilin-1 OS=Rattus norvegicus GN=Cfl1 PE=1 SV=3 APESAPLK
> sp|P45592|COI Cofilin-1 OS=Rattus norvegicus GN=Cfl1 PE=1 SV=3 NIILEEGKEILVGDVGQTVDDPYTTFVK
> sp|P45592|COI Cofilin-1 OS=Rattus norvegicus GN=Cfl1 PE=1 SV=3 LGGSAVISLEGKPL
> sp|P45592|COI Cofilin-1 OS=Rattus norvegicus GN=Cfl1 PE=1 SV=3 KSSTPEEVK
> sp|P45592|COI Cofilin-1 OS=Rattus norvegicus GN=Cfl1 PE=1 SV=3 SSTPEEVK
> sp|P45592|COI Cofilin-1 OS=Rattus norvegicus GN=Cfl1 PE=1 SV=3 AVLFCLSEDKK
> sp|P45592|COI Cofilin-1 OS=Rattus norvegicus GN=Cfl1 PE=1 SV=3 NIILEEGKEILVGDVGQTVDDPYTTFVK
> sp|P45592|COI Cofilin-1 OS=Rattus norvegicus GN=Cfl1 PE=1 SV=3 PESAPLK
> sp|P45592|COI Cofilin-1 OS=Rattus norvegicus GN=Cfl1 PE=1 SV=3 NIILEEGKEILVGDVGQTVDDPYTTFVK
> sp|P45592|COI Cofilin-1 OS=Rattus norvegicus GN=Cfl1 PE=1 SV=3 ESKKEDLVFIFWAPESAPLK
> sp|P45592|COI Cofilin-1 OS=Rattus norvegicus GN=Cfl1 PE=1 SV=3 KEDLVFIFWAPESAPLK
> sp|P45592|COI Cofilin-1 OS=Rattus norvegicus GN=Cfl1 PE=1 SV=3 KSSTPEEVKK
> sp|P47819|GFA Glial fibrillary acidic protein OS=Rattus norvegicus GN=Gfap PE=1 SV=2 ITIPVQTFSNLQIR
> sp|P47819|GFA Glial fibrillary acidic protein OS=Rattus norvegicus GN=Gfap PE=1 SV=2 KIHEEEVRELQEQLAQQQVHVEMDVAKPDLTAALR
> sp|P47819|GFA Glial fibrillary acidic protein OS=Rattus norvegicus GN=Gfap PE=1 SV=2 KLLEGEENR
> sp|P47819|GFA Glial fibrillary acidic protein OS=Rattus norvegicus GN=Gfap PE=1 SV=2 KVESLEEEIQFLR
> sp|P47819|GFA Glial fibrillary acidic protein OS=Rattus norvegicus GN=Gfap PE=1 SV=2 LEVERDNLTQDLGTLR
> sp|P47819|GFA Glial fibrillary acidic protein OS=Rattus norvegicus GN=Gfap PE=1 SV=2 QKLQDETNLR
> sp|P47819|GFA Glial fibrillary acidic protein OS=Rattus norvegicus GN=Gfap PE=1 SV=2 KIHEEEVRELQEQLAQQQVHVEMDVAKPDLTAALR
> sp|P47819|GFA Glial fibrillary acidic protein OS=Rattus norvegicus GN=Gfap PE=1 SV=2 KIHEEEVRELQEQLAQQQVHVEMDVAKPDLTAALR
> sp|P47819|GFA Glial fibrillary acidic protein OS=Rattus norvegicus GN=Gfap PE=1 SV=2 KVESLEEEIQFLR
> sp|P47819|GFA Glial fibrillary acidic protein OS=Rattus norvegicus GN=Gfap PE=1 SV=2 KVESLEEEIQFLR
> sp|P47819|GFA Glial fibrillary acidic protein OS=Rattus norvegicus GN=Gfap PE=1 SV=2 KVESLEEEIQFLR
> sp|P47819|GFA Glial fibrillary acidic protein OS=Rattus norvegicus GN=Gfap PE=1 SV=2 KVESLEEEIQFLR
> sp|P47819|GFA Glial fibrillary acidic protein OS=Rattus norvegicus GN=Gfap PE=1 SV=2 EYQDLLNVK
> sp|P47819|GFA Glial fibrillary acidic protein OS=Rattus norvegicus GN=Gfap PE=1 SV=2 KVESLEEEIQFLR
> sp|P47819|GFA Glial fibrillary acidic protein OS=Rattus norvegicus GN=Gfap PE=1 SV=2 KVESLEEEIQFLR
> sp|P47819|GFA Glial fibrillary acidic protein OS=Rattus norvegicus GN=Gfap PE=1 SV=2 LALDIEIATYR
> sp|P47819|GFA Glial fibrillary acidic protein OS=Rattus norvegicus GN=Gfap PE=1 SV=2 LADVYQAELR
> sp|P47819|GFA Glial fibrillary acidic protein OS=Rattus norvegicus GN=Gfap PE=1 SV=2 KIHEEEVRELQEQLAQQQVHVEMDVAKPDLTAALR
> sp|P47819|GFA Glial fibrillary acidic protein OS=Rattus norvegicus GN=Gfap PE=1 SV=2 KVESLEEEIQFLR
> sp|P47819|GFA Glial fibrillary acidic protein OS=Rattus norvegicus GN=Gfap PE=1 SV=2 ALAAELNQLR
> sp|P47819|GFA Glial fibrillary acidic protein OS=Rattus norvegicus GN=Gfap PE=1 SV=2 LEEEGQSLKEEMAR
> sp|P11507|AT2 Sarcoplasmic/endoplasmic reticulum calcium ATPase 2 OS=Rattus norvegicus GN=Atp2a2 PE=1 SV=1 AKDIVPGDIVEIAVGDKVPADIR
> sp|P11507|AT2 Sarcoplasmic/endoplasmic reticulum calcium ATPase 2 OS=Rattus norvegicus GN=Atp2a2 PE=1 SV=1 CLALATHDNPLR
> sp|P11507|AT2 Sarcoplasmic/endoplasmic reticulum calcium ATPase 2 OS=Rattus norvegicus GN=Atp2a2 PE=1 SV=1 CLALATHDNPLRR
> sp|P11507|AT2 Sarcoplasmic/endoplasmic reticulum calcium ATPase 2 OS=Rattus norvegicus GN=Atp2a2 PE=1 SV=1 DIVPGDIVEIAVGDKVPADIR
> sp|P11507|AT2 Sarcoplasmic/endoplasmic reticulum calcium ATPase 2 OS=Rattus norvegicus GN=Atp2a2 PE=1 SV=1 IVEFLQSFDEITAMTGDGVNDAPALK
> sp|P11507|AT2 Sarcoplasmic/endoplasmic reticulum calcium ATPase 2 OS=Rattus norvegicus GN=Atp2a2 PE=1 SV=1 TVEEVLGHFGVNESTGLSLEQVK
> sp|P11507|AT2 Sarcoplasmic/endoplasmic reticulum calcium ATPase 2 OS=Rattus norvegicus GN=Atp2a2 PE=1 SV=1 VGEATETALTCLVEK
> sp|P11507|AT2 Sarcoplasmic/endoplasmic reticulum calcium ATPase 2 OS=Rattus norvegicus GN=Atp2a2 PE=1 SV=1 AKDIVPGDIVEIAVGDKVPADIR
> sp|P11507|AT2 Sarcoplasmic/endoplasmic reticulum calcium ATPase 2 OS=Rattus norvegicus GN=Atp2a2 PE=1 SV=1 AKDIVPGDIVEIAVGDKVPADIR
> sp|P11507|AT2 Sarcoplasmic/endoplasmic reticulum calcium ATPase 2 OS=Rattus norvegicus GN=Atp2a2 PE=1 SV=1 AKDIVPGDIVEIAVGDKVPADIR
> sp|P11507|AT2 Sarcoplasmic/endoplasmic reticulum calcium ATPase 2 OS=Rattus norvegicus GN=Atp2a2 PE=1 SV=1 AKDIVPGDIVEIAVGDKVPADIR
> sp|P11507|AT2 Sarcoplasmic/endoplasmic reticulum calcium ATPase 2 OS=Rattus norvegicus GN=Atp2a2 PE=1 SV=1 AKDIVPGDIVEIAVGDKVPADIR
> sp|P11507|AT2 Sarcoplasmic/endoplasmic reticulum calcium ATPase 2 OS=Rattus norvegicus GN=Atp2a2 PE=1 SV=1 DIVPGDIVEIAVGDKVPADIR
> sp|P11507|AT2 Sarcoplasmic/endoplasmic reticulum calcium ATPase 2 OS=Rattus norvegicus GN=Atp2a2 PE=1 SV=1 DIVPGDIVEIAVGDKVPADIR
> sp|P11507|AT2 Sarcoplasmic/endoplasmic reticulum calcium ATPase 2 OS=Rattus norvegicus GN=Atp2a2 PE=1 SV=1 DIVPGDIVEIAVGDKVPADIR
> sp|P11507|AT2 Sarcoplasmic/endoplasmic reticulum calcium ATPase 2 OS=Rattus norvegicus GN=Atp2a2 PE=1 SV=1 VDQSILTGESVSVIK
> sp|P11507|AT2 Sarcoplasmic/endoplasmic reticulum calcium ATPase 2 OS=Rattus norvegicus GN=Atp2a2 PE=1 SV=1 AKDIVPGDIVEIAVGDKVPADIR
> sp|P11507|AT2 Sarcoplasmic/endoplasmic reticulum calcium ATPase 2 OS=Rattus norvegicus GN=Atp2a2 PE=1 SV=1 AKDIVPGDIVEIAVGDKVPADIR
> sp|P11507|AT2 Sarcoplasmic/endoplasmic reticulum calcium ATPase 2 OS=Rattus norvegicus GN=Atp2a2 PE=1 SV=1 KSEIGIAMGSGTAVAK
> sp|P11507|AT2 Sarcoplasmic/endoplasmic reticulum calcium ATPase 2 OS=Rattus norvegicus GN=Atp2a2 PE=1 SV=1 IVEFLQSFDEITAMTGDGVNDAPALKK
> sp|P11507|AT2 Sarcoplasmic/endoplasmic reticulum calcium ATPase 2 OS=Rattus norvegicus GN=Atp2a2 PE=1 SV=1 MFILDKVEGDTCSLNEFTITGSTYAPIGEVQKDDKPVK
> sp|P11507|AT2 Sarcoplasmic/endoplasmic reticulum calcium ATPase 2 OS=Rattus norvegicus GN=Atp2a2 PE=1 SV=1 MFILDKVEGDTCSLNEFTITGSTYAPIGEVQKDDKPVK
> sp|P11507|AT2 Sarcoplasmic/endoplasmic reticulum calcium ATPase 2 OS=Rattus norvegicus GN=Atp2a2 PE=1 SV=1 MFILDKVEGDTCSLNEFTITGSTYAPIGEVQKDDKPVK
> sp|P11507|AT2 Sarcoplasmic/endoplasmic reticulum calcium ATPase 2 OS=Rattus norvegicus GN=Atp2a2 PE=1 SV=1 VDQSILTGESVSVIK
> sp|P62161|CAL Calmodulin OS=Rattus norvegicus GN=Calm1 PE=1 SV=2 ADQLTEEQIAEFKEAFSLFDKDGDGTITTK
> sp|P62161|CAL Calmodulin OS=Rattus norvegicus GN=Calm1 PE=1 SV=2 DTDSEEEIR
> sp|P62161|CAL Calmodulin OS=Rattus norvegicus GN=Calm1 PE=1 SV=2 EADIDGDGQVNYEEFVQMMTAK
> sp|P62161|CAL Calmodulin OS=Rattus norvegicus GN=Calm1 PE=1 SV=2 HVMTNLGEKLTDEEVDEMIREADIDGDGQVNYEEFVQMMTAK
> sp|P62161|CAL Calmodulin OS=Rattus norvegicus GN=Calm1 PE=1 SV=2 KMKDTDSEEEIREAFR
> sp|P62161|CAL Calmodulin OS=Rattus norvegicus GN=Calm1 PE=1 SV=2 MKDTDSEEEIR
> sp|P62161|CAL Calmodulin OS=Rattus norvegicus GN=Calm1 PE=1 SV=2 VFDKDGNGYISAAELR
> sp|P62161|CAL Calmodulin OS=Rattus norvegicus GN=Calm1 PE=1 SV=2 EAFSLFDKDGDGTITTK
> sp|P62161|CAL Calmodulin OS=Rattus norvegicus GN=Calm1 PE=1 SV=2 MKDTDSEEEIREAFR
> sp|P62161|CAL Calmodulin OS=Rattus norvegicus GN=Calm1 PE=1 SV=2 DTDSEEEIR
> sp|P62161|CAL Calmodulin OS=Rattus norvegicus GN=Calm1 PE=1 SV=2 EADIDGDGQVNYEEFVQMMTAK
> sp|P62161|CAL Calmodulin OS=Rattus norvegicus GN=Calm1 PE=1 SV=2 EADIDGDGQVNYEEFVQMMTAK
> sp|P62161|CAL Calmodulin OS=Rattus norvegicus GN=Calm1 PE=1 SV=2 EADIDGDGQVNYEEFVQMMTAK
> sp|P62161|CAL Calmodulin OS=Rattus norvegicus GN=Calm1 PE=1 SV=2 EAFSLFDKDGDGTITTK
> sp|P62161|CAL Calmodulin OS=Rattus norvegicus GN=Calm1 PE=1 SV=2 KMKDTDSEEEIR
> sp|P62161|CAL Calmodulin OS=Rattus norvegicus GN=Calm1 PE=1 SV=2 MKDTDSEEEIR

>	sp|P62161|CAL	Calmodulin OS=Rattus norvegicus GN=Calm1 PE=1 SV=2	VFDKDGNGYISAAELR
>	sp|P62161|CAL	Calmodulin OS=Rattus norvegicus GN=Calm1 PE=1 SV=2	VFDKDGNGYISAAELR
>	sp|P62161|CAL	Calmodulin OS=Rattus norvegicus GN=Calm1 PE=1 SV=2	VFDKDGNGYISAAELR
>	sp|P62161|CAL	Calmodulin OS=Rattus norvegicus GN=Calm1 PE=1 SV=2	VFDKDGNGYISAAELR
>	sp|P62161|CAL	Calmodulin OS=Rattus norvegicus GN=Calm1 PE=1 SV=2	VFDKDGNGYISAAELR
>	sp|P62161|CAL	Calmodulin OS=Rattus norvegicus GN=Calm1 PE=1 SV=2	VFDKDGNGYISAAELR
>	sp|P62161|CAL	Calmodulin OS=Rattus norvegicus GN=Calm1 PE=1 SV=2	VFDKDGNGYISAAELR
>	sp|P62161|CAL	Calmodulin OS=Rattus norvegicus GN=Calm1 PE=1 SV=2	VFDKDGNGYISAAELR
>	sp|P62161|CAL	Calmodulin OS=Rattus norvegicus GN=Calm1 PE=1 SV=2	VFDKDGNGYISAAELR
>	sp|P62161|CAL	Calmodulin OS=Rattus norvegicus GN=Calm1 PE=1 SV=2	VFDKDGNGYISAAELR
>	sp|P62161|CAL	Calmodulin OS=Rattus norvegicus GN=Calm1 PE=1 SV=2	VFDKDGNGYISAAELR
>	sp|P62161|CAL	Calmodulin OS=Rattus norvegicus GN=Calm1 PE=1 SV=2	VFDKDGNGYISAAELR
>	sp|P62161|CAL	Calmodulin OS=Rattus norvegicus GN=Calm1 PE=1 SV=2	VFDKDGNGYISAAELR
>	sp|P62161|CAL	Calmodulin OS=Rattus norvegicus GN=Calm1 PE=1 SV=2	VFDKDGNGYISAAELR
>	sp|P62161|CAL	Calmodulin OS=Rattus norvegicus GN=Calm1 PE=1 SV=2	VFDKDGNGYISAAELR
>	sp|P62161|CAL	Calmodulin OS=Rattus norvegicus GN=Calm1 PE=1 SV=2	MKDTDSEEEIR
>	sp|P62161|CAL	Calmodulin OS=Rattus norvegicus GN=Calm1 PE=1 SV=2	MKDTDSEEEIREAFR
>	sp|P62161|CAL	Calmodulin OS=Rattus norvegicus GN=Calm1 PE=1 SV=2	EAFSLFDKDGDGTITTK
>	sp|P62161|CAL	Calmodulin OS=Rattus norvegicus GN=Calm1 PE=1 SV=2	VFDKDGNGYISAAELR
>	sp|P62161|CAL	Calmodulin OS=Rattus norvegicus GN=Calm1 PE=1 SV=2	EAFSLFDKDGDGTITTK
>	sp|P62161|CAL	Calmodulin OS=Rattus norvegicus GN=Calm1 PE=1 SV=2	EAFSLFDKDGDGTITTK
>	sp|P62161|CAL	Calmodulin OS=Rattus norvegicus GN=Calm1 PE=1 SV=2	VFDKDGNGYISAAELR
>	sp|P62161|CAL	Calmodulin OS=Rattus norvegicus GN=Calm1 PE=1 SV=2	EAFSLFDKDGDGTITTK
>	sp|P62161|CAL	Calmodulin OS=Rattus norvegicus GN=Calm1 PE=1 SV=2	MKDTDSEEEIREAFR
>	sp|P62161|CAL	Calmodulin OS=Rattus norvegicus GN=Calm1 PE=1 SV=2	VFDKDGNGYISAAELR
>	sp|P62161|CAL	Calmodulin OS=Rattus norvegicus GN=Calm1 PE=1 SV=2	EADIDGDGQVNYEEFVQMMTAK
>	sp|P62161|CAL	Calmodulin OS=Rattus norvegicus GN=Calm1 PE=1 SV=2	MKDTDSEEEIREAFR
>	sp|P62161|CAL	Calmodulin OS=Rattus norvegicus GN=Calm1 PE=1 SV=2	HVMTNLGEKLTDEEVDEMIREADIDGDGQVNYEEFVQMMTAK
>	sp|P62161|CAL	Calmodulin OS=Rattus norvegicus GN=Calm1 PE=1 SV=2	EADIDGDGQVNYEEFVQMMTAK
>	sp|P62161|CAL	Calmodulin OS=Rattus norvegicus GN=Calm1 PE=1 SV=2	EADIDGDGQVNYEEFVQMMTAK
>	sp|P62161|CAL	Calmodulin OS=Rattus norvegicus GN=Calm1 PE=1 SV=2	EAFSLFDKDGDGTITTK
>	sp|P62161|CAL	Calmodulin OS=Rattus norvegicus GN=Calm1 PE=1 SV=2	KMKDTDSEEEIR
>	sp|P62161|CAL	Calmodulin OS=Rattus norvegicus GN=Calm1 PE=1 SV=2	HVMTNLGEKLTDEEVDEMIREADIDGDGQVNYEEFVQMMTAK
>	sp|P62161|CAL	Calmodulin OS=Rattus norvegicus GN=Calm1 PE=1 SV=2	EADIDGDGQVNYEEFVQMMTAK
>	sp|P62161|CAL	Calmodulin OS=Rattus norvegicus GN=Calm1 PE=1 SV=2	VFDKDGNGYISAAELR
>	sp|P62161|CAL	Calmodulin OS=Rattus norvegicus GN=Calm1 PE=1 SV=2	EAFSLFDKDGDGTITTK
>	sp|P62161|CAL	Calmodulin OS=Rattus norvegicus GN=Calm1 PE=1 SV=2	HVMTNLGEKLTDEEVDEMIREADIDGDGQVNYEEFVQMMTAK
>	sp|P62161|CAL	Calmodulin OS=Rattus norvegicus GN=Calm1 PE=1 SV=2	VFDKDGNGYISAAELR
>	sp|P62161|CAL	Calmodulin OS=Rattus norvegicus GN=Calm1 PE=1 SV=2	VFDKDGNGYISAAELR
>	sp|P54311|GB	Guanine nucleotide-binding protein G(I)/G(S)/G(T) subunit beta-1 OS=Rattus norvegicus GN=Gnb1 PE=1 SV=4	ADQELMTYSHDNIICGITSVSFSK
>	sp|P54311|GB	Guanine nucleotide-binding protein G(I)/G(S)/G(T) subunit beta-1 OS=Rattus norvegicus GN=Gnb1 PE=1 SV=4	KACADATLSQITNNIDPVGR
>	sp|P54311|GB	Guanine nucleotide-binding protein G(I)/G(S)/G(T) subunit beta-1 OS=Rattus norvegicus GN=Gnb1 PE=1 SV=4	LFVSGACDASAK
>	sp|P54311|GB	Guanine nucleotide-binding protein G(I)/G(S)/G(T) subunit beta-1 OS=Rattus norvegicus GN=Gnb1 PE=1 SV=4	LLVSASQDGK
>	sp|P54311|GB	Guanine nucleotide-binding protein G(I)/G(S)/G(T) subunit beta-1 OS=Rattus norvegicus GN=Gnb1 PE=1 SV=4	VSCLGVTDDGMAVATGSWDSFLK
>	sp|P54311|GB	Guanine nucleotide-binding protein G(I)/G(S)/G(T) subunit beta-1 OS=Rattus norvegicus GN=Gnb1 PE=1 SV=4	LLLAGYDDFNCNVWDALK
>	sp|P54311|GB	Guanine nucleotide-binding protein G(I)/G(S)/G(T) subunit beta-1 OS=Rattus norvegicus GN=Gnb1 PE=1 SV=4	AGVLAGHDNR
>	sp|P54311|GB	Guanine nucleotide-binding protein G(I)/G(S)/G(T) subunit beta-1 OS=Rattus norvegicus GN=Gnb1 PE=1 SV=4	QTFTGHESDINAICFFPNGNAFATGSDDATCR
>	sp|P54311|GB	Guanine nucleotide-binding protein G(I)/G(S)/G(T) subunit beta-1 OS=Rattus norvegicus GN=Gnb1 PE=1 SV=4	ADQELMTYSHDNIICGITSVSFSK
>	sp|P54311|GB	Guanine nucleotide-binding protein G(I)/G(S)/G(T) subunit beta-1 OS=Rattus norvegicus GN=Gnb1 PE=1 SV=4	ADQELMTYSHDNIICGITSVSFSK
>	sp|P54311|GB	Guanine nucleotide-binding protein G(I)/G(S)/G(T) subunit beta-1 OS=Rattus norvegicus GN=Gnb1 PE=1 SV=4	AGVLAGHDNR
>	sp|P54311|GB	Guanine nucleotide-binding protein G(I)/G(S)/G(T) subunit beta-1 OS=Rattus norvegicus GN=Gnb1 PE=1 SV=4	AGVLAGHDNR
>	sp|P54311|GB	Guanine nucleotide-binding protein G(I)/G(S)/G(T) subunit beta-1 OS=Rattus norvegicus GN=Gnb1 PE=1 SV=4	AGVLAGHDNR
>	sp|P54311|GB	Guanine nucleotide-binding protein G(I)/G(S)/G(T) subunit beta-1 OS=Rattus norvegicus GN=Gnb1 PE=1 SV=4	AGVLAGHDNR
>	sp|P54311|GB	Guanine nucleotide-binding protein G(I)/G(S)/G(T) subunit beta-1 OS=Rattus norvegicus GN=Gnb1 PE=1 SV=4	KACADATLSQITNNIDPVGR
>	sp|P54311|GB	Guanine nucleotide-binding protein G(I)/G(S)/G(T) subunit beta-1 OS=Rattus norvegicus GN=Gnb1 PE=1 SV=4	KACADATLSQITNNIDPVGR
>	sp|P54311|GB	Guanine nucleotide-binding protein G(I)/G(S)/G(T) subunit beta-1 OS=Rattus norvegicus GN=Gnb1 PE=1 SV=4	KACADATLSQITNNIDPVGR
>	sp|P54311|GB	Guanine nucleotide-binding protein G(I)/G(S)/G(T) subunit beta-1 OS=Rattus norvegicus GN=Gnb1 PE=1 SV=4	KACADATLSQITNNIDPVGR
>	sp|P54311|GB	Guanine nucleotide-binding protein G(I)/G(S)/G(T) subunit beta-1 OS=Rattus norvegicus GN=Gnb1 PE=1 SV=4	LFVSGACDASAK
>	sp|P54311|GB	Guanine nucleotide-binding protein G(I)/G(S)/G(T) subunit beta-1 OS=Rattus norvegicus GN=Gnb1 PE=1 SV=4	LFVSGACDASAK
>	sp|P54311|GB	Guanine nucleotide-binding protein G(I)/G(S)/G(T) subunit beta-1 OS=Rattus norvegicus GN=Gnb1 PE=1 SV=4	LLLAGYDDFNCNVWDALK
>	sp|P54311|GB	Guanine nucleotide-binding protein G(I)/G(S)/G(T) subunit beta-1 OS=Rattus norvegicus GN=Gnb1 PE=1 SV=4	LLVSASQDGK
>	sp|P54311|GB	Guanine nucleotide-binding protein G(I)/G(S)/G(T) subunit beta-1 OS=Rattus norvegicus GN=Gnb1 PE=1 SV=4	QTFTGHESDINAICFFPNGNAFATGSDDATCR
>	sp|P54311|GB	Guanine nucleotide-binding protein G(I)/G(S)/G(T) subunit beta-1 OS=Rattus norvegicus GN=Gnb1 PE=1 SV=4	VSCLGVTDDGMAVATGSWDSFLK
>	sp|P54311|GB	Guanine nucleotide-binding protein G(I)/G(S)/G(T) subunit beta-1 OS=Rattus norvegicus GN=Gnb1 PE=1 SV=4	KACADATLSQITNNIDPVGR
>	sp|P54311|GB	Guanine nucleotide-binding protein G(I)/G(S)/G(T) subunit beta-1 OS=Rattus norvegicus GN=Gnb1 PE=1 SV=4	LLLAGYDDFNCNVWDALK
>	sp|P54311|GB	Guanine nucleotide-binding protein G(I)/G(S)/G(T) subunit beta-1 OS=Rattus norvegicus GN=Gnb1 PE=1 SV=4	VSCLGVTDDGMAVATGSWDSFLK
>	sp|P54311|GB	Guanine nucleotide-binding protein G(I)/G(S)/G(T) subunit beta-1 OS=Rattus norvegicus GN=Gnb1 PE=1 SV=4	LLVSASQDGK
>	sp|P54311|GB	Guanine nucleotide-binding protein G(I)/G(S)/G(T) subunit beta-1 OS=Rattus norvegicus GN=Gnb1 PE=1 SV=4	FLDDNQIVTSSGDTTCALWDIETGQQTTTFTGHTGDVMSLSLAPDTR
>	sp|P54311|GB	Guanine nucleotide-binding protein G(I)/G(S)/G(T) subunit beta-1 OS=Rattus norvegicus GN=Gnb1 PE=1 SV=4	VSCLGVTDDGMAVATGSWDSFLK
>	sp|P54311|GB	Guanine nucleotide-binding protein G(I)/G(S)/G(T) subunit beta-1 OS=Rattus norvegicus GN=Gnb1 PE=1 SV=4	LLVSASQDGK
>	sp|P54311|GB	Guanine nucleotide-binding protein G(I)/G(S)/G(T) subunit beta-1 OS=Rattus norvegicus GN=Gnb1 PE=1 SV=4	IYAMHWGTDSR
>	sp|P54311|GB	Guanine nucleotide-binding protein G(I)/G(S)/G(T) subunit beta-1 OS=Rattus norvegicus GN=Gnb1 PE=1 SV=4	KACADATLSQITNNIDPVGR
>	sp|P54311|GB	Guanine nucleotide-binding protein G(I)/G(S)/G(T) subunit beta-1 OS=Rattus norvegicus GN=Gnb1 PE=1 SV=4	LFVSGACDASAK
>	sp|P54311|GB	Guanine nucleotide-binding protein G(I)/G(S)/G(T) subunit beta-1 OS=Rattus norvegicus GN=Gnb1 PE=1 SV=4	FLDDNQIVTSSGDTTCALWDIETGQQTTTFTGHTGDVMSLSLAPDTR

>	sp|P54311|GBB Guanine nucleotide-binding protein G(I)/G(S)/G(T) subunit beta-1 OS=Rattus norvegicus GN=Gnb1 PE=1 SV=4	LFDLR
>	sp|P54311|GBB Guanine nucleotide-binding protein G(I)/G(S)/G(T) subunit beta-1 OS=Rattus norvegicus GN=Gnb1 PE=1 SV=4	LIIWDSYTTNKVHAIPLR
>	sp|P54311|GBB Guanine nucleotide-binding protein G(I)/G(S)/G(T) subunit beta-1 OS=Rattus norvegicus GN=Gnb1 PE=1 SV=4	QEAEQLK
>	sp|P25286|VPP V-type proton ATPase 116 kDa subunit a isoform 1 OS=Rattus norvegicus GN=Atp6v0a1 PE=2 SV=1	FLPFSFEHIR
>	sp|P25286|VPP V-type proton ATPase 116 kDa subunit a isoform 1 OS=Rattus norvegicus GN=Atp6v0a1 PE=2 SV=1	HLGTLNFGGIR
>	sp|P25286|VPP V-type proton ATPase 116 kDa subunit a isoform 1 OS=Rattus norvegicus GN=Atp6v0a1 PE=2 SV=1	KANIPIMDTGENPEVPFPR
>	sp|P25286|VPP V-type proton ATPase 116 kDa subunit a isoform 1 OS=Rattus norvegicus GN=Atp6v0a1 PE=2 SV=1	KHLGTLNFGGIR
>	sp|P25286|VPP V-type proton ATPase 116 kDa subunit a isoform 1 OS=Rattus norvegicus GN=Atp6v0a1 PE=2 SV=1	SVFIIFFQGDQLK
>	sp|P25286|VPP V-type proton ATPase 116 kDa subunit a isoform 1 OS=Rattus norvegicus GN=Atp6v0a1 PE=2 SV=1	NFLELTELK
>	sp|P25286|VPP V-type proton ATPase 116 kDa subunit a isoform 1 OS=Rattus norvegicus GN=Atp6v0a1 PE=2 SV=1	IENELKEINTNQEALKR
>	sp|P25286|VPP V-type proton ATPase 116 kDa subunit a isoform 1 OS=Rattus norvegicus GN=Atp6v0a1 PE=2 SV=1	AIYHTLNLCNIDVTQK
>	sp|P25286|VPP V-type proton ATPase 116 kDa subunit a isoform 1 OS=Rattus norvegicus GN=Atp6v0a1 PE=2 SV=1	IDDLQMVLNQTEDHR
>	sp|P25286|VPP V-type proton ATPase 116 kDa subunit a isoform 1 OS=Rattus norvegicus GN=Atp6v0a1 PE=2 SV=1	FLPFSFEHIR
>	sp|P25286|VPP V-type proton ATPase 116 kDa subunit a isoform 1 OS=Rattus norvegicus GN=Atp6v0a1 PE=2 SV=1	HLGTLNFGGIR
>	sp|P25286|VPP V-type proton ATPase 116 kDa subunit a isoform 1 OS=Rattus norvegicus GN=Atp6v0a1 PE=2 SV=1	HLGTLNFGGIR
>	sp|P25286|VPP V-type proton ATPase 116 kDa subunit a isoform 1 OS=Rattus norvegicus GN=Atp6v0a1 PE=2 SV=1	HLGTLNFGGIR
>	sp|P25286|VPP V-type proton ATPase 116 kDa subunit a isoform 1 OS=Rattus norvegicus GN=Atp6v0a1 PE=2 SV=1	KANIPIMDTGENPEVPFPR
>	sp|P25286|VPP V-type proton ATPase 116 kDa subunit a isoform 1 OS=Rattus norvegicus GN=Atp6v0a1 PE=2 SV=1	KANIPIMDTGENPEVPFPR
>	sp|P25286|VPP V-type proton ATPase 116 kDa subunit a isoform 1 OS=Rattus norvegicus GN=Atp6v0a1 PE=2 SV=1	KANIPIMDTGENPEVPFPR
>	sp|P25286|VPP V-type proton ATPase 116 kDa subunit a isoform 1 OS=Rattus norvegicus GN=Atp6v0a1 PE=2 SV=1	KANIPIMDTGENPEVPFPR
>	sp|P25286|VPP V-type proton ATPase 116 kDa subunit a isoform 1 OS=Rattus norvegicus GN=Atp6v0a1 PE=2 SV=1	IENELKEINTNQEALKR
>	sp|P25286|VPP V-type proton ATPase 116 kDa subunit a isoform 1 OS=Rattus norvegicus GN=Atp6v0a1 PE=2 SV=1	NFLELTELK
>	sp|P25286|VPP V-type proton ATPase 116 kDa subunit a isoform 1 OS=Rattus norvegicus GN=Atp6v0a1 PE=2 SV=1	IDDLQMVLNQTEDHR
>	sp|P25286|VPP V-type proton ATPase 116 kDa subunit a isoform 1 OS=Rattus norvegicus GN=Atp6v0a1 PE=2 SV=1	IDDLQMVLNQTEDHR
>	sp|P25286|VPP V-type proton ATPase 116 kDa subunit a isoform 1 OS=Rattus norvegicus GN=Atp6v0a1 PE=2 SV=1	HLGTLNFGGIR
>	sp|P25286|VPP V-type proton ATPase 116 kDa subunit a isoform 1 OS=Rattus norvegicus GN=Atp6v0a1 PE=2 SV=1	HLGTLNFGGIR
>	sp|P25286|VPP V-type proton ATPase 116 kDa subunit a isoform 1 OS=Rattus norvegicus GN=Atp6v0a1 PE=2 SV=1	KANIPIMDTGENPEVPFPR
>	sp|P25286|VPP V-type proton ATPase 116 kDa subunit a isoform 1 OS=Rattus norvegicus GN=Atp6v0a1 PE=2 SV=1	FLPFSFEHIR
>	sp|P25286|VPP V-type proton ATPase 116 kDa subunit a isoform 1 OS=Rattus norvegicus GN=Atp6v0a1 PE=2 SV=1	NFLELTELK
>	sp|P25286|VPP V-type proton ATPase 116 kDa subunit a isoform 1 OS=Rattus norvegicus GN=Atp6v0a1 PE=2 SV=1	HLGTLNFGGIR
>	sp|P25286|VPP V-type proton ATPase 116 kDa subunit a isoform 1 OS=Rattus norvegicus GN=Atp6v0a1 PE=2 SV=1	AIYHTLNLCNIDVTQK
>	sp|P25286|VPP V-type proton ATPase 116 kDa subunit a isoform 1 OS=Rattus norvegicus GN=Atp6v0a1 PE=2 SV=1	HLGTLNFGGIR
>	sp|P06686|AT1 Sodium/potassium-transporting ATPase subunit alpha-2 OS=Rattus norvegicus GN=Atp1a2 PE=1 SV=1	DMTSEQLDEILRDHTEIVFAR
>	sp|P06686|AT1 Sodium/potassium-transporting ATPase subunit alpha-2 OS=Rattus norvegicus GN=Atp1a2 PE=1 SV=1	DTAGDASESALLK
>	sp|P06686|AT1 Sodium/potassium-transporting ATPase subunit alpha-2 OS=Rattus norvegicus GN=Atp1a2 PE=1 SV=1	GFKFDTDELNFPTEK
>	sp|P06686|AT1 Sodium/potassium-transporting ATPase subunit alpha-2 OS=Rattus norvegicus GN=Atp1a2 PE=1 SV=1	MTVAHMWFDNQIHEADTTEDQSGATFDKR
>	sp|P06686|AT1 Sodium/potassium-transporting ATPase subunit alpha-2 OS=Rattus norvegicus GN=Atp1a2 PE=1 SV=1	SPEFTHENPLETR
>	sp|P06686|AT1 Sodium/potassium-transporting ATPase subunit alpha-2 OS=Rattus norvegicus GN=Atp1a2 PE=1 SV=1	VLGFCQLNLPSGKFPR
>	sp|P06686|AT1 Sodium/potassium-transporting ATPase subunit alpha-2 OS=Rattus norvegicus GN=Atp1a2 PE=1 SV=1	MQINAEEVVVGDLVEVK
>	sp|P06686|AT1 Sodium/potassium-transporting ATPase subunit alpha-2 OS=Rattus norvegicus GN=Atp1a2 PE=1 SV=1	AAVPDAVGK
>	sp|P06686|AT1 Sodium/potassium-transporting ATPase subunit alpha-2 OS=Rattus norvegicus GN=Atp1a2 PE=1 SV=1	AAVPDAVGK
>	sp|P06686|AT1 Sodium/potassium-transporting ATPase subunit alpha-2 OS=Rattus norvegicus GN=Atp1a2 PE=1 SV=1	AAVPDAVGK
>	sp|P06686|AT1 Sodium/potassium-transporting ATPase subunit alpha-2 OS=Rattus norvegicus GN=Atp1a2 PE=1 SV=1	DMTSEQLDEILRDHTEIVFAR
>	sp|P06686|AT1 Sodium/potassium-transporting ATPase subunit alpha-2 OS=Rattus norvegicus GN=Atp1a2 PE=1 SV=1	EIPLDKEMQDAFQNAYMELGGLGER
>	sp|P06686|AT1 Sodium/potassium-transporting ATPase subunit alpha-2 OS=Rattus norvegicus GN=Atp1a2 PE=1 SV=1	EIPLDKEMQDAFQNAYMELGGLGER
>	sp|P06686|AT1 Sodium/potassium-transporting ATPase subunit alpha-2 OS=Rattus norvegicus GN=Atp1a2 PE=1 SV=1	EVAMDDHK
>	sp|P06686|AT1 Sodium/potassium-transporting ATPase subunit alpha-2 OS=Rattus norvegicus GN=Atp1a2 PE=1 SV=1	GFKFDTDELNFPTEK
>	sp|P06686|AT1 Sodium/potassium-transporting ATPase subunit alpha-2 OS=Rattus norvegicus GN=Atp1a2 PE=1 SV=1	GFKFDTDELNFPTEK
>	sp|P06686|AT1 Sodium/potassium-transporting ATPase subunit alpha-2 OS=Rattus norvegicus GN=Atp1a2 PE=1 SV=1	GFKFDTDELNFPTEK
>	sp|P06686|AT1 Sodium/potassium-transporting ATPase subunit alpha-2 OS=Rattus norvegicus GN=Atp1a2 PE=1 SV=1	GVGIISEGNETVEDIAAR
>	sp|P06686|AT1 Sodium/potassium-transporting ATPase subunit alpha-2 OS=Rattus norvegicus GN=Atp1a2 PE=1 SV=1	GVGIISEGNETVEDIAAR
>	sp|P06686|AT1 Sodium/potassium-transporting ATPase subunit alpha-2 OS=Rattus norvegicus GN=Atp1a2 PE=1 SV=1	GVGIISEGNETVEDIAAR
>	sp|P06686|AT1 Sodium/potassium-transporting ATPase subunit alpha-2 OS=Rattus norvegicus GN=Atp1a2 PE=1 SV=1	GVGIISEGNETVEDIAAR
>	sp|P06686|AT1 Sodium/potassium-transporting ATPase subunit alpha-2 OS=Rattus norvegicus GN=Atp1a2 PE=1 SV=1	GVGIISEGNETVEDIAAR
>	sp|P06686|AT1 Sodium/potassium-transporting ATPase subunit alpha-2 OS=Rattus norvegicus GN=Atp1a2 PE=1 SV=1	GVGIISEGNETVEDIAAR
>	sp|P06686|AT1 Sodium/potassium-transporting ATPase subunit alpha-2 OS=Rattus norvegicus GN=Atp1a2 PE=1 SV=1	LCFVGLMSMIDPPR
>	sp|P06686|AT1 Sodium/potassium-transporting ATPase subunit alpha-2 OS=Rattus norvegicus GN=Atp1a2 PE=1 SV=1	LCFVGLMSMIDPPR
>	sp|P06686|AT1 Sodium/potassium-transporting ATPase subunit alpha-2 OS=Rattus norvegicus GN=Atp1a2 PE=1 SV=1	LIFDNLKK
>	sp|P06686|AT1 Sodium/potassium-transporting ATPase subunit alpha-2 OS=Rattus norvegicus GN=Atp1a2 PE=1 SV=1	LIIVEGCQR
>	sp|P06686|AT1 Sodium/potassium-transporting ATPase subunit alpha-2 OS=Rattus norvegicus GN=Atp1a2 PE=1 SV=1	LNIPVSQVNPR
>	sp|P06686|AT1 Sodium/potassium-transporting ATPase subunit alpha-2 OS=Rattus norvegicus GN=Atp1a2 PE=1 SV=1	MTVAHMWFDNQIHEADTTEDQSGATFDK
>	sp|P06686|AT1 Sodium/potassium-transporting ATPase subunit alpha-2 OS=Rattus norvegicus GN=Atp1a2 PE=1 SV=1	MTVAHMWFDNQIHEADTTEDQSGATFDKR
>	sp|P06686|AT1 Sodium/potassium-transporting ATPase subunit alpha-2 OS=Rattus norvegicus GN=Atp1a2 PE=1 SV=1	MTVAHMWFDNQIHEADTTEDQSGATFDKR
>	sp|P06686|AT1 Sodium/potassium-transporting ATPase subunit alpha-2 OS=Rattus norvegicus GN=Atp1a2 PE=1 SV=1	MTVAHMWFDNQIHEADTTEDQSGATFDKR
>	sp|P06686|AT1 Sodium/potassium-transporting ATPase subunit alpha-2 OS=Rattus norvegicus GN=Atp1a2 PE=1 SV=1	NLEAVETLGSTSTICSDK
>	sp|P06686|AT1 Sodium/potassium-transporting ATPase subunit alpha-2 OS=Rattus norvegicus GN=Atp1a2 PE=1 SV=1	NLEAVETLGSTSTICSDK
>	sp|P06686|AT1 Sodium/potassium-transporting ATPase subunit alpha-2 OS=Rattus norvegicus GN=Atp1a2 PE=1 SV=1	NLEAVETLGSTSTICSDK
>	sp|P06686|AT1 Sodium/potassium-transporting ATPase subunit alpha-2 OS=Rattus norvegicus GN=Atp1a2 PE=1 SV=1	NLEAVETLGSTSTICSDK
>	sp|P06686|AT1 Sodium/potassium-transporting ATPase subunit alpha-2 OS=Rattus norvegicus GN=Atp1a2 PE=1 SV=1	NLEAVETLGSTSTICSDK
>	sp|P06686|AT1 Sodium/potassium-transporting ATPase subunit alpha-2 OS=Rattus norvegicus GN=Atp1a2 PE=1 SV=1	NLEAVETLGSTSTICSDKTGTLTQNR
>	sp|P06686|AT1 Sodium/potassium-transporting ATPase subunit alpha-2 OS=Rattus norvegicus GN=Atp1a2 PE=1 SV=1	NLEAVETLGSTSTICSDKTGTLTQNR
>	sp|P06686|AT1 Sodium/potassium-transporting ATPase subunit alpha-2 OS=Rattus norvegicus GN=Atp1a2 PE=1 SV=1	NLEAVETLGSTSTICSDKTGTLTQNR
>	sp|P06686|AT1 Sodium/potassium-transporting ATPase subunit alpha-2 OS=Rattus norvegicus GN=Atp1a2 PE=1 SV=1	NLEAVETLGSTSTICSDKTGTLTQNR
>	sp|P06686|AT1 Sodium/potassium-transporting ATPase subunit alpha-2 OS=Rattus norvegicus GN=Atp1a2 PE=1 SV=1	NMVPQQALVIR

>	sp|P06686|AT1	Sodium/potassium-transporting ATPase subunit alpha-2 OS=Rattus norvegicus GN=Atp1a2 PE=1 SV=1	NMVPQQALVIR
>	sp|P06686|AT1	Sodium/potassium-transporting ATPase subunit alpha-2 OS=Rattus norvegicus GN=Atp1a2 PE=1 SV=1	NMVPQQALVIR
>	sp|P06686|AT1	Sodium/potassium-transporting ATPase subunit alpha-2 OS=Rattus norvegicus GN=Atp1a2 PE=1 SV=1	QAADMILLDDNFASIVTGVEEGR
>	sp|P06686|AT1	Sodium/potassium-transporting ATPase subunit alpha-2 OS=Rattus norvegicus GN=Atp1a2 PE=1 SV=1	QAADMILLDDNFASIVTGVEEGR
>	sp|P06686|AT1	Sodium/potassium-transporting ATPase subunit alpha-2 OS=Rattus norvegicus GN=Atp1a2 PE=1 SV=1	QAADMILLDDNFASIVTGVEEGR
>	sp|P06686|AT1	Sodium/potassium-transporting ATPase subunit alpha-2 OS=Rattus norvegicus GN=Atp1a2 PE=1 SV=1	QGAIVAVTGDGVNDSPALK
>	sp|P06686|AT1	Sodium/potassium-transporting ATPase subunit alpha-2 OS=Rattus norvegicus GN=Atp1a2 PE=1 SV=1	QGAIVAVTGDGVNDSPALK
>	sp|P06686|AT1	Sodium/potassium-transporting ATPase subunit alpha-2 OS=Rattus norvegicus GN=Atp1a2 PE=1 SV=1	QGAIVAVTGDGVNDSPALK
>	sp|P06686|AT1	Sodium/potassium-transporting ATPase subunit alpha-2 OS=Rattus norvegicus GN=Atp1a2 PE=1 SV=1	QGAIVAVTGDGVNDSPALK
>	sp|P06686|AT1	Sodium/potassium-transporting ATPase subunit alpha-2 OS=Rattus norvegicus GN=Atp1a2 PE=1 SV=1	QGAIVAVTGDGVNDSPALK
>	sp|P06686|AT1	Sodium/potassium-transporting ATPase subunit alpha-2 OS=Rattus norvegicus GN=Atp1a2 PE=1 SV=1	RNSVFQQGMK
>	sp|P06686|AT1	Sodium/potassium-transporting ATPase subunit alpha-2 OS=Rattus norvegicus GN=Atp1a2 PE=1 SV=1	SPEFTHENPLETR
>	sp|P06686|AT1	Sodium/potassium-transporting ATPase subunit alpha-2 OS=Rattus norvegicus GN=Atp1a2 PE=1 SV=1	SPEFTHENPLETR
>	sp|P06686|AT1	Sodium/potassium-transporting ATPase subunit alpha-2 OS=Rattus norvegicus GN=Atp1a2 PE=1 SV=1	TDKLVNER
>	sp|P06686|AT1	Sodium/potassium-transporting ATPase subunit alpha-2 OS=Rattus norvegicus GN=Atp1a2 PE=1 SV=1	VAEIPFNSTNK
>	sp|P06686|AT1	Sodium/potassium-transporting ATPase subunit alpha-2 OS=Rattus norvegicus GN=Atp1a2 PE=1 SV=1	VAEIPFNSTNK
>	sp|P06686|AT1	Sodium/potassium-transporting ATPase subunit alpha-2 OS=Rattus norvegicus GN=Atp1a2 PE=1 SV=1	VAEIPFNSTNK
>	sp|P06686|AT1	Sodium/potassium-transporting ATPase subunit alpha-2 OS=Rattus norvegicus GN=Atp1a2 PE=1 SV=1	VDNSSLTGESEPQTR
>	sp|P06686|AT1	Sodium/potassium-transporting ATPase subunit alpha-2 OS=Rattus norvegicus GN=Atp1a2 PE=1 SV=1	VDNSSLTGESEPQTR
>	sp|P06686|AT1	Sodium/potassium-transporting ATPase subunit alpha-2 OS=Rattus norvegicus GN=Atp1a2 PE=1 SV=1	VDNSSLTGESEPQTR
>	sp|P06686|AT1	Sodium/potassium-transporting ATPase subunit alpha-2 OS=Rattus norvegicus GN=Atp1a2 PE=1 SV=1	VDNSSLTGESEPQTR
>	sp|P06686|AT1	Sodium/potassium-transporting ATPase subunit alpha-2 OS=Rattus norvegicus GN=Atp1a2 PE=1 SV=1	VIMVTGDHPITAK
>	sp|P06686|AT1	Sodium/potassium-transporting ATPase subunit alpha-2 OS=Rattus norvegicus GN=Atp1a2 PE=1 SV=1	VIMVTGDHPITAK
>	sp|P06686|AT1	Sodium/potassium-transporting ATPase subunit alpha-2 OS=Rattus norvegicus GN=Atp1a2 PE=1 SV=1	VIMVTGDHPITAK
>	sp|P06686|AT1	Sodium/potassium-transporting ATPase subunit alpha-2 OS=Rattus norvegicus GN=Atp1a2 PE=1 SV=1	VIMVTGDHPITAK
>	sp|P06686|AT1	Sodium/potassium-transporting ATPase subunit alpha-2 OS=Rattus norvegicus GN=Atp1a2 PE=1 SV=1	GFKFDTDELNFPTEK
>	sp|P06686|AT1	Sodium/potassium-transporting ATPase subunit alpha-2 OS=Rattus norvegicus GN=Atp1a2 PE=1 SV=1	NLEAVETLGSTSTICSDK
>	sp|P06686|AT1	Sodium/potassium-transporting ATPase subunit alpha-2 OS=Rattus norvegicus GN=Atp1a2 PE=1 SV=1	NLEAVETLGSTSTICSDK
>	sp|P06686|AT1	Sodium/potassium-transporting ATPase subunit alpha-2 OS=Rattus norvegicus GN=Atp1a2 PE=1 SV=1	ACVVHGSDLKDMTSEQLDEILRDHTEIVFAR
>	sp|P06686|AT1	Sodium/potassium-transporting ATPase subunit alpha-2 OS=Rattus norvegicus GN=Atp1a2 PE=1 SV=1	MQINAEEVVVGDLVEVK
>	sp|P06686|AT1	Sodium/potassium-transporting ATPase subunit alpha-2 OS=Rattus norvegicus GN=Atp1a2 PE=1 SV=1	NSQTDKLVNER
>	sp|P06686|AT1	Sodium/potassium-transporting ATPase subunit alpha-2 OS=Rattus norvegicus GN=Atp1a2 PE=1 SV=1	SPEFTHENPLETR
>	sp|P06686|AT1	Sodium/potassium-transporting ATPase subunit alpha-2 OS=Rattus norvegicus GN=Atp1a2 PE=1 SV=1	DHTEIVFAR
>	sp|P06686|AT1	Sodium/potassium-transporting ATPase subunit alpha-2 OS=Rattus norvegicus GN=Atp1a2 PE=1 SV=1	VLGFCQLNLPSGKFPR
>	sp|P06686|AT1	Sodium/potassium-transporting ATPase subunit alpha-2 OS=Rattus norvegicus GN=Atp1a2 PE=1 SV=1	MTVAHMWFDNQIHEADTTEDQSGATFDKR
>	sp|P06686|AT1	Sodium/potassium-transporting ATPase subunit alpha-2 OS=Rattus norvegicus GN=Atp1a2 PE=1 SV=1	LNIPVSQVNPR
>	sp|P06686|AT1	Sodium/potassium-transporting ATPase subunit alpha-2 OS=Rattus norvegicus GN=Atp1a2 PE=1 SV=1	NMVPQQALVIR
>	sp|P06686|AT1	Sodium/potassium-transporting ATPase subunit alpha-2 OS=Rattus norvegicus GN=Atp1a2 PE=1 SV=1	DHTEIVFAR
>	sp|P06686|AT1	Sodium/potassium-transporting ATPase subunit alpha-2 OS=Rattus norvegicus GN=Atp1a2 PE=1 SV=1	ACVVHGSDLKDMTSEQLDEILRDHTEIVFAR
>	sp|P06686|AT1	Sodium/potassium-transporting ATPase subunit alpha-2 OS=Rattus norvegicus GN=Atp1a2 PE=1 SV=1	DGPNALTPPPTTPEWVK
>	sp|P06686|AT1	Sodium/potassium-transporting ATPase subunit alpha-2 OS=Rattus norvegicus GN=Atp1a2 PE=1 SV=1	NMVPQQALVIR
>	sp|P06686|AT1	Sodium/potassium-transporting ATPase subunit alpha-2 OS=Rattus norvegicus GN=Atp1a2 PE=1 SV=1	LNIPVSQVNPR
>	sp|P06686|AT1	Sodium/potassium-transporting ATPase subunit alpha-2 OS=Rattus norvegicus GN=Atp1a2 PE=1 SV=1	MQINAEEVVVGDLVEVK
>	sp|P06686|AT1	Sodium/potassium-transporting ATPase subunit alpha-2 OS=Rattus norvegicus GN=Atp1a2 PE=1 SV=1	MQINAEEVVVGDLVEVK
>	sp|P06686|AT1	Sodium/potassium-transporting ATPase subunit alpha-2 OS=Rattus norvegicus GN=Atp1a2 PE=1 SV=1	NMVPQQALVIR
>	sp|P06686|AT1	Sodium/potassium-transporting ATPase subunit alpha-2 OS=Rattus norvegicus GN=Atp1a2 PE=1 SV=1	NSVFQQGMK
>	sp|P06686|AT1	Sodium/potassium-transporting ATPase subunit alpha-2 OS=Rattus norvegicus GN=Atp1a2 PE=1 SV=1	LNIPVSQVNPR
>	sp|P06686|AT1	Sodium/potassium-transporting ATPase subunit alpha-2 OS=Rattus norvegicus GN=Atp1a2 PE=1 SV=1	LIFDNLK
>	sp|P06686|AT1	Sodium/potassium-transporting ATPase subunit alpha-2 OS=Rattus norvegicus GN=Atp1a2 PE=1 SV=1	VIMVTGDHPITAK
>	sp|P06686|AT1	Sodium/potassium-transporting ATPase subunit alpha-2 OS=Rattus norvegicus GN=Atp1a2 PE=1 SV=1	EDSPQSHVLVMK
>	sp|P06686|AT1	Sodium/potassium-transporting ATPase subunit alpha-2 OS=Rattus norvegicus GN=Atp1a2 PE=1 SV=1	NLEAVETLGSTSTICSDK
>	sp|P06686|AT1	Sodium/potassium-transporting ATPase subunit alpha-2 OS=Rattus norvegicus GN=Atp1a2 PE=1 SV=1	EVAMDDHK
>	sp|P06686|AT1	Sodium/potassium-transporting ATPase subunit alpha-2 OS=Rattus norvegicus GN=Atp1a2 PE=1 SV=1	MVTGDHPITAK
>	sp|P06686|AT1	Sodium/potassium-transporting ATPase subunit alpha-2 OS=Rattus norvegicus GN=Atp1a2 PE=1 SV=1	VIMVTGDHPITAK
>	sp|P06686|AT1	Sodium/potassium-transporting ATPase subunit alpha-2 OS=Rattus norvegicus GN=Atp1a2 PE=1 SV=1	LIIVEGCQR
>	sp|P06686|AT1	Sodium/potassium-transporting ATPase subunit alpha-2 OS=Rattus norvegicus GN=Atp1a2 PE=1 SV=1	KNCLVK
>	sp|P06686|AT1	Sodium/potassium-transporting ATPase subunit alpha-2 OS=Rattus norvegicus GN=Atp1a2 PE=1 SV=1	NSQTDKLVNER
>	sp|P06686|AT1	Sodium/potassium-transporting ATPase subunit alpha-2 OS=Rattus norvegicus GN=Atp1a2 PE=1 SV=1	LNIPVSQVNPR
>	sp|P06686|AT1	Sodium/potassium-transporting ATPase subunit alpha-2 OS=Rattus norvegicus GN=Atp1a2 PE=1 SV=1	NLEAVETLGSTSTICSDK
>	sp|P06686|AT1	Sodium/potassium-transporting ATPase subunit alpha-2 OS=Rattus norvegicus GN=Atp1a2 PE=1 SV=1	LSLDELGR
>	sp|P06686|AT1	Sodium/potassium-transporting ATPase subunit alpha-2 OS=Rattus norvegicus GN=Atp1a2 PE=1 SV=1	LSLDELGR
>	sp|P06686|AT1	Sodium/potassium-transporting ATPase subunit alpha-2 OS=Rattus norvegicus GN=Atp1a2 PE=1 SV=1	KNCLVK
>	sp|P01946|HBA	Hemoglobin subunit alpha-1/2 OS=Rattus norvegicus GN=Hba1 PE=1 SV=3	AADHVEDLPGALSTLSDLHAHK
>	sp|P01946|HBA	Hemoglobin subunit alpha-1/2 OS=Rattus norvegicus GN=Hba1 PE=1 SV=3	DALAKAADHVEDLPGALSTLSDLHAHK
>	sp|P01946|HBA	Hemoglobin subunit alpha-1/2 OS=Rattus norvegicus GN=Hba1 PE=1 SV=3	IGGHGGEYGEEALQR
>	sp|P01946|HBA	Hemoglobin subunit alpha-1/2 OS=Rattus norvegicus GN=Hba1 PE=1 SV=3	PGALSTLSDLHAHK
>	sp|P01946|HBA	Hemoglobin subunit alpha-1/2 OS=Rattus norvegicus GN=Hba1 PE=1 SV=3	SDLHAHK
>	sp|P01946|HBA	Hemoglobin subunit alpha-1/2 OS=Rattus norvegicus GN=Hba1 PE=1 SV=3	FLSHCLLVTLACHHPGDFTPAMHASLDKFLASVSTVLTSK
>	sp|P01946|HBA	Hemoglobin subunit alpha-1/2 OS=Rattus norvegicus GN=Hba1 PE=1 SV=3	KVADALAK
>	sp|P01946|HBA	Hemoglobin subunit alpha-1/2 OS=Rattus norvegicus GN=Hba1 PE=1 SV=3	TYFSHIDVSPGSAQVK
>	sp|P01946|HBA	Hemoglobin subunit alpha-1/2 OS=Rattus norvegicus GN=Hba1 PE=1 SV=3	AADHVEDLPGALSTLSDLHAHK
>	sp|P01946|HBA	Hemoglobin subunit alpha-1/2 OS=Rattus norvegicus GN=Hba1 PE=1 SV=3	AADHVEDLPGALSTLSDLHAHK
>	sp|P01946|HBA	Hemoglobin subunit alpha-1/2 OS=Rattus norvegicus GN=Hba1 PE=1 SV=3	AADHVEDLPGALSTLSDLHAHK
>	sp|P01946|HBA	Hemoglobin subunit alpha-1/2 OS=Rattus norvegicus GN=Hba1 PE=1 SV=3	AADHVEDLPGALSTLSDLHAHK

>	sp|P01946|HBA	Hemoglobin subunit alpha-1/2 OS=Rattus norvegicus GN=Hba1 PE=1 SV=3	AADHVEDLPGALSTLSDLHAHK
>	sp|P01946|HBA	Hemoglobin subunit alpha-1/2 OS=Rattus norvegicus GN=Hba1 PE=1 SV=3	AADHVEDLPGALSTLSDLHAHK
>	sp|P01946|HBA	Hemoglobin subunit alpha-1/2 OS=Rattus norvegicus GN=Hba1 PE=1 SV=3	AADHVEDLPGALSTLSDLHAHK
>	sp|P01946|HBA	Hemoglobin subunit alpha-1/2 OS=Rattus norvegicus GN=Hba1 PE=1 SV=3	AADHVEDLPGALSTLSDLHAHK
>	sp|P01946|HBA	Hemoglobin subunit alpha-1/2 OS=Rattus norvegicus GN=Hba1 PE=1 SV=3	AADHVEDLPGALSTLSDLHAHK
>	sp|P01946|HBA	Hemoglobin subunit alpha-1/2 OS=Rattus norvegicus GN=Hba1 PE=1 SV=3	AADHVEDLPGALSTLSDLHAHK
>	sp|P01946|HBA	Hemoglobin subunit alpha-1/2 OS=Rattus norvegicus GN=Hba1 PE=1 SV=3	AADHVEDLPGALSTLSDLHAHK
>	sp|P01946|HBA	Hemoglobin subunit alpha-1/2 OS=Rattus norvegicus GN=Hba1 PE=1 SV=3	AADHVEDLPGALSTLSDLHAHK
>	sp|P01946|HBA	Hemoglobin subunit alpha-1/2 OS=Rattus norvegicus GN=Hba1 PE=1 SV=3	AADHVEDLPGALSTLSDLHAHK
>	sp|P01946|HBA	Hemoglobin subunit alpha-1/2 OS=Rattus norvegicus GN=Hba1 PE=1 SV=3	AADHVEDLPGALSTLSDLHAHK
>	sp|P01946|HBA	Hemoglobin subunit alpha-1/2 OS=Rattus norvegicus GN=Hba1 PE=1 SV=3	AADHVEDLPGALSTLSDLHAHK
>	sp|P01946|HBA	Hemoglobin subunit alpha-1/2 OS=Rattus norvegicus GN=Hba1 PE=1 SV=3	AADHVEDLPGALSTLSDLHAHK
>	sp|P01946|HBA	Hemoglobin subunit alpha-1/2 OS=Rattus norvegicus GN=Hba1 PE=1 SV=3	AADHVEDLPGALSTLSDLHAHK
>	sp|P01946|HBA	Hemoglobin subunit alpha-1/2 OS=Rattus norvegicus GN=Hba1 PE=1 SV=3	AADHVEDLPGALSTLSDLHAHK
>	sp|P01946|HBA	Hemoglobin subunit alpha-1/2 OS=Rattus norvegicus GN=Hba1 PE=1 SV=3	AADHVEDLPGALSTLSDLHAHK
>	sp|P01946|HBA	Hemoglobin subunit alpha-1/2 OS=Rattus norvegicus GN=Hba1 PE=1 SV=3	AADHVEDLPGALSTLSDLHAHK
>	sp|P01946|HBA	Hemoglobin subunit alpha-1/2 OS=Rattus norvegicus GN=Hba1 PE=1 SV=3	AADHVEDLPGALSTLSDLHAHK
>	sp|P01946|HBA	Hemoglobin subunit alpha-1/2 OS=Rattus norvegicus GN=Hba1 PE=1 SV=3	AADHVEDLPGALSTLSDLHAHK
>	sp|P01946|HBA	Hemoglobin subunit alpha-1/2 OS=Rattus norvegicus GN=Hba1 PE=1 SV=3	AADHVEDLPGALSTLSDLHAHK
>	sp|P01946|HBA	Hemoglobin subunit alpha-1/2 OS=Rattus norvegicus GN=Hba1 PE=1 SV=3	AADHVEDLPGALSTLSDLHAHK
>	sp|P01946|HBA	Hemoglobin subunit alpha-1/2 OS=Rattus norvegicus GN=Hba1 PE=1 SV=3	AADHVEDLPGALSTLSDLHAHK
>	sp|P01946|HBA	Hemoglobin subunit alpha-1/2 OS=Rattus norvegicus GN=Hba1 PE=1 SV=3	AADHVEDLPGALSTLSDLHAHK
>	sp|P01946|HBA	Hemoglobin subunit alpha-1/2 OS=Rattus norvegicus GN=Hba1 PE=1 SV=3	AADHVEDLPGALSTLSDLHAHK
>	sp|P01946|HBA	Hemoglobin subunit alpha-1/2 OS=Rattus norvegicus GN=Hba1 PE=1 SV=3	AADHVEDLPGALSTLSDLHAHK
>	sp|P01946|HBA	Hemoglobin subunit alpha-1/2 OS=Rattus norvegicus GN=Hba1 PE=1 SV=3	IGGHGGEYGEEALQR
>	sp|P01946|HBA	Hemoglobin subunit alpha-1/2 OS=Rattus norvegicus GN=Hba1 PE=1 SV=3	IGGHGGEYGEEALQR
>	sp|P01946|HBA	Hemoglobin subunit alpha-1/2 OS=Rattus norvegicus GN=Hba1 PE=1 SV=3	IGGHGGEYGEEALQR
>	sp|P01946|HBA	Hemoglobin subunit alpha-1/2 OS=Rattus norvegicus GN=Hba1 PE=1 SV=3	IGGHGGEYGEEALQR
>	sp|P01946|HBA	Hemoglobin subunit alpha-1/2 OS=Rattus norvegicus GN=Hba1 PE=1 SV=3	IGGHGGEYGEEALQR
>	sp|P01946|HBA	Hemoglobin subunit alpha-1/2 OS=Rattus norvegicus GN=Hba1 PE=1 SV=3	IGGHGGEYGEEALQR
>	sp|P01946|HBA	Hemoglobin subunit alpha-1/2 OS=Rattus norvegicus GN=Hba1 PE=1 SV=3	IGGHGGEYGEEALQR
>	sp|P01946|HBA	Hemoglobin subunit alpha-1/2 OS=Rattus norvegicus GN=Hba1 PE=1 SV=3	IGGHGGEYGEEALQR
>	sp|P01946|HBA	Hemoglobin subunit alpha-1/2 OS=Rattus norvegicus GN=Hba1 PE=1 SV=3	IGGHGGEYGEEALQR
>	sp|P01946|HBA	Hemoglobin subunit alpha-1/2 OS=Rattus norvegicus GN=Hba1 PE=1 SV=3	IGGHGGEYGEEALQR
>	sp|P01946|HBA	Hemoglobin subunit alpha-1/2 OS=Rattus norvegicus GN=Hba1 PE=1 SV=3	IGGHGGEYGEEALQR
>	sp|P01946|HBA	Hemoglobin subunit alpha-1/2 OS=Rattus norvegicus GN=Hba1 PE=1 SV=3	IGGHGGEYGEEALQR
>	sp|P01946|HBA	Hemoglobin subunit alpha-1/2 OS=Rattus norvegicus GN=Hba1 PE=1 SV=3	IGGHGGEYGEEALQR
>	sp|P01946|HBA	Hemoglobin subunit alpha-1/2 OS=Rattus norvegicus GN=Hba1 PE=1 SV=3	IGGHGGEYGEEALQR
>	sp|P01946|HBA	Hemoglobin subunit alpha-1/2 OS=Rattus norvegicus GN=Hba1 PE=1 SV=3	IGGHGGEYGEEALQR
>	sp|P01946|HBA	Hemoglobin subunit alpha-1/2 OS=Rattus norvegicus GN=Hba1 PE=1 SV=3	IGGHGGEYGEEALQR
>	sp|P01946|HBA	Hemoglobin subunit alpha-1/2 OS=Rattus norvegicus GN=Hba1 PE=1 SV=3	IGGHGGEYGEEALQR
>	sp|P01946|HBA	Hemoglobin subunit alpha-1/2 OS=Rattus norvegicus GN=Hba1 PE=1 SV=3	IGGHGGEYGEEALQR
>	sp|P01946|HBA	Hemoglobin subunit alpha-1/2 OS=Rattus norvegicus GN=Hba1 PE=1 SV=3	KVADALAK
>	sp|P01946|HBA	Hemoglobin subunit alpha-1/2 OS=Rattus norvegicus GN=Hba1 PE=1 SV=3	IGGHGGEYGEEALQR
>	sp|P01946|HBA	Hemoglobin subunit alpha-1/2 OS=Rattus norvegicus GN=Hba1 PE=1 SV=3	IGGHGGEYGEEALQR
>	sp|P01946|HBA	Hemoglobin subunit alpha-1/2 OS=Rattus norvegicus GN=Hba1 PE=1 SV=3	STLSDLHAHK
>	sp|P01946|HBA	Hemoglobin subunit alpha-1/2 OS=Rattus norvegicus GN=Hba1 PE=1 SV=3	AADHVEDLPGALSTLSDLHAHK
>	sp|P01946|HBA	Hemoglobin subunit alpha-1/2 OS=Rattus norvegicus GN=Hba1 PE=1 SV=3	AADHVEDLPGALSTLSDLHAHK
>	sp|P01946|HBA	Hemoglobin subunit alpha-1/2 OS=Rattus norvegicus GN=Hba1 PE=1 SV=3	FLSHCLLVTLACHHPGDFTPAMHASLDKFLASVSTVLTSK
>	sp|P01946|HBA	Hemoglobin subunit alpha-1/2 OS=Rattus norvegicus GN=Hba1 PE=1 SV=3	IGGHGGEYGEEALQR
>	sp|P01946|HBA	Hemoglobin subunit alpha-1/2 OS=Rattus norvegicus GN=Hba1 PE=1 SV=3	VADALAK
>	sp|P01946|HBA	Hemoglobin subunit alpha-1/2 OS=Rattus norvegicus GN=Hba1 PE=1 SV=3	AADHVEDLPGALSTLSDLHAHK
>	sp|P01946|HBA	Hemoglobin subunit alpha-1/2 OS=Rattus norvegicus GN=Hba1 PE=1 SV=3	MFAAFPTTK
>	sp|P01946|HBA	Hemoglobin subunit alpha-1/2 OS=Rattus norvegicus GN=Hba1 PE=1 SV=3	VADALAK
>	sp|P01946|HBA	Hemoglobin subunit alpha-1/2 OS=Rattus norvegicus GN=Hba1 PE=1 SV=3	KVADALAK
>	sp|P01946|HBA	Hemoglobin subunit alpha-1/2 OS=Rattus norvegicus GN=Hba1 PE=1 SV=3	TYFSHIDVSPGSAQVK
>	sp|P01946|HBA	Hemoglobin subunit alpha-1/2 OS=Rattus norvegicus GN=Hba1 PE=1 SV=3	IGGHGGEYGEEALQR
>	sp|P01946|HBA	Hemoglobin subunit alpha-1/2 OS=Rattus norvegicus GN=Hba1 PE=1 SV=3	MFAAFPTTK
>	sp|P01946|HBA	Hemoglobin subunit alpha-1/2 OS=Rattus norvegicus GN=Hba1 PE=1 SV=3	AADHVEDLPGALSTLSDLHAHK
>	sp|P01946|HBA	Hemoglobin subunit alpha-1/2 OS=Rattus norvegicus GN=Hba1 PE=1 SV=3	AADHVEDLPGALSTLSDLHAHK
>	sp|P01946|HBA	Hemoglobin subunit alpha-1/2 OS=Rattus norvegicus GN=Hba1 PE=1 SV=3	AADHVEDLPGALSTLSDLHAHKLR
>	sp|P01946|HBA	Hemoglobin subunit alpha-1/2 OS=Rattus norvegicus GN=Hba1 PE=1 SV=3	AADHVEDLPGALSTLSDLHAHK
>	sp|P01946|HBA	Hemoglobin subunit alpha-1/2 OS=Rattus norvegicus GN=Hba1 PE=1 SV=3	AADHVEDLPGALSTLSDLHAHK
>	sp|P09606|GLN	Glutamine synthetase OS=Rattus norvegicus GN=Glul PE=1 SV=3	IQLMYIWVDGTGEGLR
>	sp|P09606|GLN	Glutamine synthetase OS=Rattus norvegicus GN=Glul PE=1 SV=3	MGDHLWVAR
>	sp|P09606|GLN	Glutamine synthetase OS=Rattus norvegicus GN=Glul PE=1 SV=3	RLTGFHETSNINDFSAGVANR
>	sp|P09606|GLN	Glutamine synthetase OS=Rattus norvegicus GN=Glul PE=1 SV=3	RPSANCDPYAVTEAIVR
>	sp|P09606|GLN	Glutamine synthetase OS=Rattus norvegicus GN=Glul PE=1 SV=3	RDPNKLVFCEVFK
>	sp|P09606|GLN	Glutamine synthetase OS=Rattus norvegicus GN=Glul PE=1 SV=3	LTGFHETSNINDFSAGVANR
>	sp|P09606|GLN	Glutamine synthetase OS=Rattus norvegicus GN=Glul PE=1 SV=3	IQLMYIWVDGTGEGLR
>	sp|P09606|GLN	Glutamine synthetase OS=Rattus norvegicus GN=Glul PE=1 SV=3	LTGFHETSNINDFSAGVANR
>	sp|P09606|GLN	Glutamine synthetase OS=Rattus norvegicus GN=Glul PE=1 SV=3	MGDHLWVAR
>	sp|P09606|GLN	Glutamine synthetase OS=Rattus norvegicus GN=Glul PE=1 SV=3	RLTGFHETSNINDFSAGVANR
>	sp|P09606|GLN	Glutamine synthetase OS=Rattus norvegicus GN=Glul PE=1 SV=3	RLTGFHETSNINDFSAGVANR

>	sp|P09606|GLN	Glutamine synthetase OS=Rattus norvegicus GN=Glul PE=1 SV=3	RPSANCDPYAVTEAIVR
>	sp|P09606|GLN	Glutamine synthetase OS=Rattus norvegicus GN=Glul PE=1 SV=3	RPSANCDPYAVTEAIVR
>	sp|P09606|GLN	Glutamine synthetase OS=Rattus norvegicus GN=Glul PE=1 SV=3	RPSANCDPYAVTEAIVR
>	sp|P09606|GLN	Glutamine synthetase OS=Rattus norvegicus GN=Glul PE=1 SV=3	RPSANCDPYAVTEAIVR
>	sp|P09606|GLN	Glutamine synthetase OS=Rattus norvegicus GN=Glul PE=1 SV=3	RPSANCDPYAVTEAIVR
>	sp|P09606|GLN	Glutamine synthetase OS=Rattus norvegicus GN=Glul PE=1 SV=3	TCLLNETGDEPFQYKN
>	sp|P09606|GLN	Glutamine synthetase OS=Rattus norvegicus GN=Glul PE=1 SV=3	VCEDFGVIATFDPKPIPGNWNGAGCHTNFSTK
>	sp|P09606|GLN	Glutamine synthetase OS=Rattus norvegicus GN=Glul PE=1 SV=3	LVFCEVFK
>	sp|P09606|GLN	Glutamine synthetase OS=Rattus norvegicus GN=Glul PE=1 SV=3	DIVEAHYR
>	sp|P09606|GLN	Glutamine synthetase OS=Rattus norvegicus GN=Glul PE=1 SV=3	RPSANCDPYAVTEAIVR
>	sp|P09606|GLN	Glutamine synthetase OS=Rattus norvegicus GN=Glul PE=1 SV=3	VCEDFGVIATFDPKPIPGNWNGAGCHTNFSTK
>	sp|P09606|GLN	Glutamine synthetase OS=Rattus norvegicus GN=Glul PE=1 SV=3	LVFCEVFK
>	sp|P09606|GLN	Glutamine synthetase OS=Rattus norvegicus GN=Glul PE=1 SV=3	ACLYAGIK
>	sp|P09606|GLN	Glutamine synthetase OS=Rattus norvegicus GN=Glul PE=1 SV=3	DIVEAHYR
>	sp|P09606|GLN	Glutamine synthetase OS=Rattus norvegicus GN=Glul PE=1 SV=3	LTGFHETSNINDFSAGVANR
>	sp|P09606|GLN	Glutamine synthetase OS=Rattus norvegicus GN=Glul PE=1 SV=3	RPSANCDPYAVTEAIVR
>	sp|P09606|GLN	Glutamine synthetase OS=Rattus norvegicus GN=Glul PE=1 SV=3	DIVEAHYR
>	sp|Q9R063|PRI	Peroxiredoxin-5, mitochondrial OS=Rattus norvegicus GN=Prdx5 PE=1 SV=1	ETDLLLDDSLVSLFGNR
>	sp|Q9R063|PRI	Peroxiredoxin-5, mitochondrial OS=Rattus norvegicus GN=Prdx5 PE=1 SV=1	GVLFGVPGAFTPGCSK
>	sp|Q9R063|PRI	Peroxiredoxin-5, mitochondrial OS=Rattus norvegicus GN=Prdx5 PE=1 SV=1	KVNLAELFK
>	sp|Q9R063|PRI	Peroxiredoxin-5, mitochondrial OS=Rattus norvegicus GN=Prdx5 PE=1 SV=1	THLPGFVEQAGALK
>	sp|Q9R063|PRI	Peroxiredoxin-5, mitochondrial OS=Rattus norvegicus GN=Prdx5 PE=1 SV=1	VNLAELFK
>	sp|Q9R063|PRI	Peroxiredoxin-5, mitochondrial OS=Rattus norvegicus GN=Prdx5 PE=1 SV=1	VQLLADPTGAFGK
>	sp|Q9R063|PRI	Peroxiredoxin-5, mitochondrial OS=Rattus norvegicus GN=Prdx5 PE=1 SV=1	ETDLLLDDSLVSLFGNR
>	sp|Q9R063|PRI	Peroxiredoxin-5, mitochondrial OS=Rattus norvegicus GN=Prdx5 PE=1 SV=1	THLPGFVEQAGALK
>	sp|Q9R063|PRI	Peroxiredoxin-5, mitochondrial OS=Rattus norvegicus GN=Prdx5 PE=1 SV=1	THLPGFVEQAGALK
>	sp|Q9R063|PRI	Peroxiredoxin-5, mitochondrial OS=Rattus norvegicus GN=Prdx5 PE=1 SV=1	VNLAELFK
>	sp|Q9R063|PRI	Peroxiredoxin-5, mitochondrial OS=Rattus norvegicus GN=Prdx5 PE=1 SV=1	KGVLFGVPGAFTPGCSK
>	sp|Q9R063|PRI	Peroxiredoxin-5, mitochondrial OS=Rattus norvegicus GN=Prdx5 PE=1 SV=1	KVNLAELFK
>	sp|Q9R063|PRI	Peroxiredoxin-5, mitochondrial OS=Rattus norvegicus GN=Prdx5 PE=1 SV=1	VNLAELFK
>	sp|Q9R063|PRI	Peroxiredoxin-5, mitochondrial OS=Rattus norvegicus GN=Prdx5 PE=1 SV=1	KVNLAELFK
>	sp|Q9R063|PRI	Peroxiredoxin-5, mitochondrial OS=Rattus norvegicus GN=Prdx5 PE=1 SV=1	ETDLLLDDSLVSLFGNR
>	sp|Q9R063|PRI	Peroxiredoxin-5, mitochondrial OS=Rattus norvegicus GN=Prdx5 PE=1 SV=1	GVLFGVPGAFTPGCSK
>	sp|Q9R063|PRI	Peroxiredoxin-5, mitochondrial OS=Rattus norvegicus GN=Prdx5 PE=1 SV=1	THLPGFVEQAGALK
>	sp|Q68FY0|QCI	Cytochrome b-c1 complex subunit 1, mitochondrial OS=Rattus norvegicus GN=Uqcrc1 PE=1 SV=1	EMQENDASMQNVVFDYLHATAFQGTPLAQAVEGPSENVR
>	sp|Q68FY0|QCI	Cytochrome b-c1 complex subunit 1, mitochondrial OS=Rattus norvegicus GN=Uqcrc1 PE=1 SV=1	HQQLLDLAQDHFSSVSQVYEEDAVPSITPCR
>	sp|Q68FY0|QCI	Cytochrome b-c1 complex subunit 1, mitochondrial OS=Rattus norvegicus GN=Uqcrc1 PE=1 SV=1	MVLAAAGGVK
>	sp|Q68FY0|QCI	Cytochrome b-c1 complex subunit 1, mitochondrial OS=Rattus norvegicus GN=Uqcrc1 PE=1 SV=1	PSITPCR
>	sp|Q68FY0|QCI	Cytochrome b-c1 complex subunit 1, mitochondrial OS=Rattus norvegicus GN=Uqcrc1 PE=1 SV=1	RIPLAEWESR
>	sp|Q68FY0|QCI	Cytochrome b-c1 complex subunit 1, mitochondrial OS=Rattus norvegicus GN=Uqcrc1 PE=1 SV=1	VVELLADIVQNISLEDSQIEKER
>	sp|Q68FY0|QCI	Cytochrome b-c1 complex subunit 1, mitochondrial OS=Rattus norvegicus GN=Uqcrc1 PE=1 SV=1	NALISHLDGTTPVCEDIGR
>	sp|Q68FY0|QCI	Cytochrome b-c1 complex subunit 1, mitochondrial OS=Rattus norvegicus GN=Uqcrc1 PE=1 SV=1	EMQENDASMQNVVFDYLHATAFQGTPLAQAVEGPSENVR
>	sp|Q68FY0|QCI	Cytochrome b-c1 complex subunit 1, mitochondrial OS=Rattus norvegicus GN=Uqcrc1 PE=1 SV=1	HQQLLDLAQDHFSSVSQVYEEDAVPSITPCR
>	sp|Q68FY0|QCI	Cytochrome b-c1 complex subunit 1, mitochondrial OS=Rattus norvegicus GN=Uqcrc1 PE=1 SV=1	HQQLLDLAQDHFSSVSQVYEEDAVPSITPCR
>	sp|Q68FY0|QCI	Cytochrome b-c1 complex subunit 1, mitochondrial OS=Rattus norvegicus GN=Uqcrc1 PE=1 SV=1	HQQLLDLAQDHFSSVSQVYEEDAVPSITPCR
>	sp|Q68FY0|QCI	Cytochrome b-c1 complex subunit 1, mitochondrial OS=Rattus norvegicus GN=Uqcrc1 PE=1 SV=1	MVLAAAGGVK
>	sp|Q68FY0|QCI	Cytochrome b-c1 complex subunit 1, mitochondrial OS=Rattus norvegicus GN=Uqcrc1 PE=1 SV=1	NALISHLDGTTPVCEDIGR
>	sp|Q68FY0|QCI	Cytochrome b-c1 complex subunit 1, mitochondrial OS=Rattus norvegicus GN=Uqcrc1 PE=1 SV=1	NALISHLDGTTPVCEDIGR
>	sp|Q68FY0|QCI	Cytochrome b-c1 complex subunit 1, mitochondrial OS=Rattus norvegicus GN=Uqcrc1 PE=1 SV=1	NALISHLDGTTPVCEDIGR
>	sp|Q68FY0|QCI	Cytochrome b-c1 complex subunit 1, mitochondrial OS=Rattus norvegicus GN=Uqcrc1 PE=1 SV=1	RIPLAEWESR
>	sp|Q68FY0|QCI	Cytochrome b-c1 complex subunit 1, mitochondrial OS=Rattus norvegicus GN=Uqcrc1 PE=1 SV=1	RIPLAEWESR
>	sp|Q68FY0|QCI	Cytochrome b-c1 complex subunit 1, mitochondrial OS=Rattus norvegicus GN=Uqcrc1 PE=1 SV=1	MVLAAAGGVK
>	sp|Q68FY0|QCI	Cytochrome b-c1 complex subunit 1, mitochondrial OS=Rattus norvegicus GN=Uqcrc1 PE=1 SV=1	MVLAAAGGVK
>	sp|Q68FY0|QCI	Cytochrome b-c1 complex subunit 1, mitochondrial OS=Rattus norvegicus GN=Uqcrc1 PE=1 SV=1	RIPLAEWESR
>	sp|Q68FY0|QCI	Cytochrome b-c1 complex subunit 1, mitochondrial OS=Rattus norvegicus GN=Uqcrc1 PE=1 SV=1	RIPLAEWESR
>	sp|P61206|ARF	ADP-ribosylation factor 3 OS=Rattus norvegicus GN=Arf3 PE=1 SV=2	ILMVGLDAAGK
>	sp|P61206|ARF	ADP-ribosylation factor 3 OS=Rattus norvegicus GN=Arf3 PE=1 SV=2	LGEIVTTIPTIGFNVETVEYK
>	sp|P61206|ARF	ADP-ribosylation factor 3 OS=Rattus norvegicus GN=Arf3 PE=1 SV=2	MLAEDELRDAVLLVFANK
>	sp|P61206|ARF	ADP-ribosylation factor 3 OS=Rattus norvegicus GN=Arf3 PE=1 SV=2	MLAEDELRDAVLLVFANKQDLPNAMNAAEITDKLGLHSLR
>	sp|P61206|ARF	ADP-ribosylation factor 3 OS=Rattus norvegicus GN=Arf3 PE=1 SV=2	QDLPNAMNAAEITDK
>	sp|P61206|ARF	ADP-ribosylation factor 3 OS=Rattus norvegicus GN=Arf3 PE=1 SV=2	QDLPNAMNAAEITDKLGLHSLR
>	sp|P61206|ARF	ADP-ribosylation factor 3 OS=Rattus norvegicus GN=Arf3 PE=1 SV=2	VNEAREELMR
>	sp|P61206|ARF	ADP-ribosylation factor 3 OS=Rattus norvegicus GN=Arf3 PE=1 SV=2	MLAEDELRDAVLLVFANKQDLPNAMNAAEITDK
>	sp|P61206|ARF	ADP-ribosylation factor 3 OS=Rattus norvegicus GN=Arf3 PE=1 SV=2	ILMVGLDAAGK
>	sp|P61206|ARF	ADP-ribosylation factor 3 OS=Rattus norvegicus GN=Arf3 PE=1 SV=2	ILMVGLDAAGK
>	sp|P61206|ARF	ADP-ribosylation factor 3 OS=Rattus norvegicus GN=Arf3 PE=1 SV=2	ILMVGLDAAGK
>	sp|P61206|ARF	ADP-ribosylation factor 3 OS=Rattus norvegicus GN=Arf3 PE=1 SV=2	ILMVGLDAAGK
>	sp|P61206|ARF	ADP-ribosylation factor 3 OS=Rattus norvegicus GN=Arf3 PE=1 SV=2	ILMVGLDAAGK
>	sp|P61206|ARF	ADP-ribosylation factor 3 OS=Rattus norvegicus GN=Arf3 PE=1 SV=2	ILMVGLDAAGK
>	sp|P61206|ARF	ADP-ribosylation factor 3 OS=Rattus norvegicus GN=Arf3 PE=1 SV=2	LGEIVTTIPTIGFNVETVEYK
>	sp|P61206|ARF	ADP-ribosylation factor 3 OS=Rattus norvegicus GN=Arf3 PE=1 SV=2	LGEIVTTIPTIGFNVETVEYK
>	sp|P61206|ARF	ADP-ribosylation factor 3 OS=Rattus norvegicus GN=Arf3 PE=1 SV=2	LGEIVTTIPTIGFNVETVEYK
>	sp|P61206|ARF	ADP-ribosylation factor 3 OS=Rattus norvegicus GN=Arf3 PE=1 SV=2	LGEIVTTIPTIGFNVETVEYK
>	sp|P61206|ARF	ADP-ribosylation factor 3 OS=Rattus norvegicus GN=Arf3 PE=1 SV=2	MLAEDELRDAVLLVFANK

>	sp|P61206|ARF ADP-ribosylation factor 3 OS=Rattus norvegicus GN=Arf3 PE=1 SV=2	MLAEDELRDAVLLVFANK
>	sp|P61206|ARF ADP-ribosylation factor 3 OS=Rattus norvegicus GN=Arf3 PE=1 SV=2	MLAEDELRDAVLLVFANK
>	sp|P61206|ARF ADP-ribosylation factor 3 OS=Rattus norvegicus GN=Arf3 PE=1 SV=2	MLAEDELRDAVLLVFANKQDLPNAMNAAEITDKLGLHSLR
>	sp|P61206|ARF ADP-ribosylation factor 3 OS=Rattus norvegicus GN=Arf3 PE=1 SV=2	MLAEDELRDAVLLVFANKQDLPNAMNAAEITDKLGLHSLR
>	sp|P61206|ARF ADP-ribosylation factor 3 OS=Rattus norvegicus GN=Arf3 PE=1 SV=2	QDLPNAMNAAEITDKLGLHSLR
>	sp|P61206|ARF ADP-ribosylation factor 3 OS=Rattus norvegicus GN=Arf3 PE=1 SV=2	VNEAREELMR
>	sp|P61206|ARF ADP-ribosylation factor 3 OS=Rattus norvegicus GN=Arf3 PE=1 SV=2	VNEAREELMR
>	sp|P61206|ARF ADP-ribosylation factor 3 OS=Rattus norvegicus GN=Arf3 PE=1 SV=2	LGEIVTTIPTIGFNVETVEYK
>	sp|P61206|ARF ADP-ribosylation factor 3 OS=Rattus norvegicus GN=Arf3 PE=1 SV=2	MLAEDELRDAVLLVFANKQDLPNAMNAAEITDK
>	sp|P61206|ARF ADP-ribosylation factor 3 OS=Rattus norvegicus GN=Arf3 PE=1 SV=2	MLAEDELRDAVLLVFANKQDLPNAMNAAEITDK
>	sp|P61206|ARF ADP-ribosylation factor 3 OS=Rattus norvegicus GN=Arf3 PE=1 SV=2	ILMVGLDAAGK
>	sp|P61206|ARF ADP-ribosylation factor 3 OS=Rattus norvegicus GN=Arf3 PE=1 SV=2	MLAEDELR
>	sp|P61206|ARF ADP-ribosylation factor 3 OS=Rattus norvegicus GN=Arf3 PE=1 SV=2	LGEIVTTIPTIGFNVETVEYK
>	sp|P61206|ARF ADP-ribosylation factor 3 OS=Rattus norvegicus GN=Arf3 PE=1 SV=2	LGEIVTTIPTIGFNVETVEYK
>	sp|P61206|ARF ADP-ribosylation factor 3 OS=Rattus norvegicus GN=Arf3 PE=1 SV=2	VNEAREELMR
>	sp|P61206|ARF ADP-ribosylation factor 3 OS=Rattus norvegicus GN=Arf3 PE=1 SV=2	MLAEDELRDAVLLVFANKQDLPNAMNAAEITDKLGLHSLR
>	sp|P61206|ARF ADP-ribosylation factor 3 OS=Rattus norvegicus GN=Arf3 PE=1 SV=2	LKLGEIVTTIPTIGFNVETVEYK
>	sp|P61206|ARF ADP-ribosylation factor 3 OS=Rattus norvegicus GN=Arf3 PE=1 SV=2	QDLPNAMNAAEITDKLGLHSLR
>	sp|P61206|ARF ADP-ribosylation factor 3 OS=Rattus norvegicus GN=Arf3 PE=1 SV=2	MLAEDELRDAVLLVFANKQDLPNAMNAAEITDK
>	sp|P61206|ARF ADP-ribosylation factor 3 OS=Rattus norvegicus GN=Arf3 PE=1 SV=2	MLAEDELRDAVLLVFANK
>	sp|P61206|ARF ADP-ribosylation factor 3 OS=Rattus norvegicus GN=Arf3 PE=1 SV=2	LKLGEIVTTIPTIGFNVETVEYK
>	sp|P61206|ARF ADP-ribosylation factor 3 OS=Rattus norvegicus GN=Arf3 PE=1 SV=2	LGEIVTTIPTIGFNVETVEYK
>	sp|P08461|ODI Dihydrolipoyllysine-residue acetyltransferase component of pyruvate dehydrogenase complex, mitochondrial OS=Rattus norvegicus GN=Dlat PE=1 SV=3	DVPLGTPLCIIVEK
>	sp|P08461|ODI Dihydrolipoyllysine-residue acetyltransferase component of pyruvate dehydrogenase complex, mitochondrial OS=Rattus norvegicus GN=Dlat PE=1 SV=3	DVPVGSIICITVEKPQDIEAFK
>	sp|P08461|ODI Dihydrolipoyllysine-residue acetyltransferase component of pyruvate dehydrogenase complex, mitochondrial OS=Rattus norvegicus GN=Dlat PE=1 SV=3	LQPHEFQGGTFTISNLGMFGIK
>	sp|P08461|ODI Dihydrolipoyllysine-residue acetyltransferase component of pyruvate dehydrogenase complex, mitochondrial OS=Rattus norvegicus GN=Dlat PE=1 SV=3	VAPTPAGVFIDIPISNIR
>	sp|P08461|ODI Dihydrolipoyllysine-residue acetyltransferase component of pyruvate dehydrogenase complex, mitochondrial OS=Rattus norvegicus GN=Dlat PE=1 SV=3	VVDGAVGAQWLAEFK
>	sp|P08461|ODI Dihydrolipoyllysine-residue acetyltransferase component of pyruvate dehydrogenase complex, mitochondrial OS=Rattus norvegicus GN=Dlat PE=1 SV=3	GLETIASDVVSLASK
>	sp|P08461|ODI Dihydrolipoyllysine-residue acetyltransferase component of pyruvate dehydrogenase complex, mitochondrial OS=Rattus norvegicus GN=Dlat PE=1 SV=3	GLETIASDVVSLASK
>	sp|P08461|ODI Dihydrolipoyllysine-residue acetyltransferase component of pyruvate dehydrogenase complex, mitochondrial OS=Rattus norvegicus GN=Dlat PE=1 SV=3	GLETIASDVVSLASK
>	sp|P08461|ODI Dihydrolipoyllysine-residue acetyltransferase component of pyruvate dehydrogenase complex, mitochondrial OS=Rattus norvegicus GN=Dlat PE=1 SV=3	GLETIASDVVSLASK
>	sp|P08461|ODI Dihydrolipoyllysine-residue acetyltransferase component of pyruvate dehydrogenase complex, mitochondrial OS=Rattus norvegicus GN=Dlat PE=1 SV=3	GLETIASDVVSLASK
>	sp|P08461|ODI Dihydrolipoyllysine-residue acetyltransferase component of pyruvate dehydrogenase complex, mitochondrial OS=Rattus norvegicus GN=Dlat PE=1 SV=3	LQPHEFQGGTFTISNLGMFGIK
>	sp|P08461|ODI Dihydrolipoyllysine-residue acetyltransferase component of pyruvate dehydrogenase complex, mitochondrial OS=Rattus norvegicus GN=Dlat PE=1 SV=3	NFSAIINPPQACILAIGASEDKLIPADNEK
>	sp|P08461|ODI Dihydrolipoyllysine-residue acetyltransferase component of pyruvate dehydrogenase complex, mitochondrial OS=Rattus norvegicus GN=Dlat PE=1 SV=3	DVPLGTPLCIIVEK
>	sp|P08461|ODI Dihydrolipoyllysine-residue acetyltransferase component of pyruvate dehydrogenase complex, mitochondrial OS=Rattus norvegicus GN=Dlat PE=1 SV=3	DVPVGSIICITVEKPQDIEAFK
>	sp|P08461|ODI Dihydrolipoyllysine-residue acetyltransferase component of pyruvate dehydrogenase complex, mitochondrial OS=Rattus norvegicus GN=Dlat PE=1 SV=3	ILVPEGTR
>	sp|P08461|ODI Dihydrolipoyllysine-residue acetyltransferase component of pyruvate dehydrogenase complex, mitochondrial OS=Rattus norvegicus GN=Dlat PE=1 SV=3	LQPHEFQGGTFTISNLGMFGIK
>	sp|P08461|ODI Dihydrolipoyllysine-residue acetyltransferase component of pyruvate dehydrogenase complex, mitochondrial OS=Rattus norvegicus GN=Dlat PE=1 SV=3	DVPVGSIICITVEKPQDIEAFK
>	sp|P08461|ODI Dihydrolipoyllysine-residue acetyltransferase component of pyruvate dehydrogenase complex, mitochondrial OS=Rattus norvegicus GN=Dlat PE=1 SV=3	DVPVGSIICITVEKPQDIEAFK
>	sp|P08461|ODI Dihydrolipoyllysine-residue acetyltransferase component of pyruvate dehydrogenase complex, mitochondrial OS=Rattus norvegicus GN=Dlat PE=1 SV=3	DVPVGSIICITVEKPQDIEAFK
>	sp|P32551|QCI Cytochrome b-c1 complex subunit 2, mitochondrial OS=Rattus norvegicus GN=Uqcrc2 PE=1 SV=2	AVAFQNPQTR
>	sp|P32551|QCI Cytochrome b-c1 complex subunit 2, mitochondrial OS=Rattus norvegicus GN=Uqcrc2 PE=1 SV=2	AVAQGNLSSADVQAAK
>	sp|P32551|QCI Cytochrome b-c1 complex subunit 2, mitochondrial OS=Rattus norvegicus GN=Uqcrc2 PE=1 SV=2	EQNGDNLVHAAIVAESAAIGNAEANAFSVLQHLLGAGPHIK
>	sp|P32551|QCI Cytochrome b-c1 complex subunit 2, mitochondrial OS=Rattus norvegicus GN=Uqcrc2 PE=1 SV=2	EQNGDNLVHAAIVAESAAIGNAEANAFSVLQHLLGAGPHIKR
>	sp|P32551|QCI Cytochrome b-c1 complex subunit 2, mitochondrial OS=Rattus norvegicus GN=Uqcrc2 PE=1 SV=2	GGEIREQNGDNLVHAAIVAESAAIGNAEANAFSVLQHLLGAGPHIK
>	sp|P32551|QCI Cytochrome b-c1 complex subunit 2, mitochondrial OS=Rattus norvegicus GN=Uqcrc2 PE=1 SV=2	MALVGLGVSHSILK
>	sp|P32551|QCI Cytochrome b-c1 complex subunit 2, mitochondrial OS=Rattus norvegicus GN=Uqcrc2 PE=1 SV=2	LPNGLVIASLENYAPLSR
>	sp|P32551|QCI Cytochrome b-c1 complex subunit 2, mitochondrial OS=Rattus norvegicus GN=Uqcrc2 PE=1 SV=2	EQNGDNLVHAAIVAESAAIGNAEANAFSVLQHLLGAGPHIK
>	sp|P32551|QCI Cytochrome b-c1 complex subunit 2, mitochondrial OS=Rattus norvegicus GN=Uqcrc2 PE=1 SV=2	EQNGDNLVHAAIVAESAAIGNAEANAFSVLQHLLGAGPHIK
>	sp|P32551|QCI Cytochrome b-c1 complex subunit 2, mitochondrial OS=Rattus norvegicus GN=Uqcrc2 PE=1 SV=2	EQNGDNLVHAAIVAESAAIGNAEANAFSVLQHLLGAGPHIK
>	sp|P32551|QCI Cytochrome b-c1 complex subunit 2, mitochondrial OS=Rattus norvegicus GN=Uqcrc2 PE=1 SV=2	EQNGDNLVHAAIVAESAAIGNAEANAFSVLQHLLGAGPHIK
>	sp|P32551|QCI Cytochrome b-c1 complex subunit 2, mitochondrial OS=Rattus norvegicus GN=Uqcrc2 PE=1 SV=2	EQNGDNLVHAAIVAESAAIGNAEANAFSVLQHLLGAGPHIK
>	sp|P32551|QCI Cytochrome b-c1 complex subunit 2, mitochondrial OS=Rattus norvegicus GN=Uqcrc2 PE=1 SV=2	EQNGDNLVHAAIVAESAAIGNAEANAFSVLQHLLGAGPHIKR
>	sp|P32551|QCI Cytochrome b-c1 complex subunit 2, mitochondrial OS=Rattus norvegicus GN=Uqcrc2 PE=1 SV=2	GGEIREQNGDNLVHAAIVAESAAIGNAEANAFSVLQHLLGAGPHIK
>	sp|P32551|QCI Cytochrome b-c1 complex subunit 2, mitochondrial OS=Rattus norvegicus GN=Uqcrc2 PE=1 SV=2	GGEIREQNGDNLVHAAIVAESAAIGNAEANAFSVLQHLLGAGPHIK
>	sp|P32551|QCI Cytochrome b-c1 complex subunit 2, mitochondrial OS=Rattus norvegicus GN=Uqcrc2 PE=1 SV=2	EQNGDNLVHAAIVAESAAIGNAEANAFSVLQHLLGAGPHIK
>	sp|P32551|QCI Cytochrome b-c1 complex subunit 2, mitochondrial OS=Rattus norvegicus GN=Uqcrc2 PE=1 SV=2	EQNGDNLVHAAIVAESAAIGNAEANAFSVLQHLLGAGPHIK
>	sp|P32551|QCI Cytochrome b-c1 complex subunit 2, mitochondrial OS=Rattus norvegicus GN=Uqcrc2 PE=1 SV=2	EQNGDNLVHAAIVAESAAIGNAEANAFSVLQHLLGAGPHIK
>	sp|P32551|QCI Cytochrome b-c1 complex subunit 2, mitochondrial OS=Rattus norvegicus GN=Uqcrc2 PE=1 SV=2	EQNGDNLVHAAIVAESAAIGNAEANAFSVLQHLLGAGPHIK
>	sp|P32551|QCI Cytochrome b-c1 complex subunit 2, mitochondrial OS=Rattus norvegicus GN=Uqcrc2 PE=1 SV=2	AVAQGNLSSADVQAAK
>	sp|P07340|AT1 Sodium/potassium-transporting ATPase subunit beta-1 OS=Rattus norvegicus GN=Atp1b1 PE=1 SV=1	AYGENIGYSEK
>	sp|P07340|AT1 Sodium/potassium-transporting ATPase subunit beta-1 OS=Rattus norvegicus GN=Atp1b1 PE=1 SV=1	VAPPGLTQIPQIQK
>	sp|P07340|AT1 Sodium/potassium-transporting ATPase subunit beta-1 OS=Rattus norvegicus GN=Atp1b1 PE=1 SV=1	YKDSAQKDDMIFEDCGSMPSEPKER
>	sp|P07340|AT1 Sodium/potassium-transporting ATPase subunit beta-1 OS=Rattus norvegicus GN=Atp1b1 PE=1 SV=1	YNPNVLPVQCTGK
>	sp|P07340|AT1 Sodium/potassium-transporting ATPase subunit beta-1 OS=Rattus norvegicus GN=Atp1b1 PE=1 SV=1	SYEAYVLNIIR
>	sp|P07340|AT1 Sodium/potassium-transporting ATPase subunit beta-1 OS=Rattus norvegicus GN=Atp1b1 PE=1 SV=1	TEISFRPNDPK
>	sp|P07340|AT1 Sodium/potassium-transporting ATPase subunit beta-1 OS=Rattus norvegicus GN=Atp1b1 PE=1 SV=1	AYGENIGYSEK
>	sp|P07340|AT1 Sodium/potassium-transporting ATPase subunit beta-1 OS=Rattus norvegicus GN=Atp1b1 PE=1 SV=1	SYEAYVLNIIR
>	sp|P07340|AT1 Sodium/potassium-transporting ATPase subunit beta-1 OS=Rattus norvegicus GN=Atp1b1 PE=1 SV=1	SYEAYVLNIIR
>	sp|P07340|AT1 Sodium/potassium-transporting ATPase subunit beta-1 OS=Rattus norvegicus GN=Atp1b1 PE=1 SV=1	SYEAYVLNIIR
>	sp|P07340|AT1 Sodium/potassium-transporting ATPase subunit beta-1 OS=Rattus norvegicus GN=Atp1b1 PE=1 SV=1	SYEAYVLNIIR
>	sp|P07340|AT1 Sodium/potassium-transporting ATPase subunit beta-1 OS=Rattus norvegicus GN=Atp1b1 PE=1 SV=1	SYEAYVLNIIR
>	sp|P07340|AT1 Sodium/potassium-transporting ATPase subunit beta-1 OS=Rattus norvegicus GN=Atp1b1 PE=1 SV=1	SYEAYVLNIIR

>	sp|P07340|AT1 Sodium/potassium-transporting ATPase subunit beta-1 OS=Rattus norvegicus GN=Atp1b1 PE=1 SV=1	SYEAYVLNIIR
>	sp|P07340|AT1 Sodium/potassium-transporting ATPase subunit beta-1 OS=Rattus norvegicus GN=Atp1b1 PE=1 SV=1	YKDSAQKDDMIFEDCGSMPSEPKER
>	sp|P07340|AT1 Sodium/potassium-transporting ATPase subunit beta-1 OS=Rattus norvegicus GN=Atp1b1 PE=1 SV=1	YKDSAQKDDMIFEDCGSMPSEPKER
>	sp|P07340|AT1 Sodium/potassium-transporting ATPase subunit beta-1 OS=Rattus norvegicus GN=Atp1b1 PE=1 SV=1	YKDSAQKDDMIFEDCGSMPSEPKER
>	sp|P07340|AT1 Sodium/potassium-transporting ATPase subunit beta-1 OS=Rattus norvegicus GN=Atp1b1 PE=1 SV=1	YKDSAQKDDMIFEDCGSMPSEPKER
>	sp|P07340|AT1 Sodium/potassium-transporting ATPase subunit beta-1 OS=Rattus norvegicus GN=Atp1b1 PE=1 SV=1	YKDSAQKDDMIFEDCGSMPSEPKER
>	sp|P07340|AT1 Sodium/potassium-transporting ATPase subunit beta-1 OS=Rattus norvegicus GN=Atp1b1 PE=1 SV=1	AKEEGSWK
>	sp|P07340|AT1 Sodium/potassium-transporting ATPase subunit beta-1 OS=Rattus norvegicus GN=Atp1b1 PE=1 SV=1	VAPPGLTQIPQIQK
>	sp|P07340|AT1 Sodium/potassium-transporting ATPase subunit beta-1 OS=Rattus norvegicus GN=Atp1b1 PE=1 SV=1	YNPNVLPVQCTGK
>	sp|P07340|AT1 Sodium/potassium-transporting ATPase subunit beta-1 OS=Rattus norvegicus GN=Atp1b1 PE=1 SV=1	SYEAYVLNIIR
>	sp|P07340|AT1 Sodium/potassium-transporting ATPase subunit beta-1 OS=Rattus norvegicus GN=Atp1b1 PE=1 SV=1	VGNIEYFGMGGFYGFPLQYYPYYGK
>	sp|P07340|AT1 Sodium/potassium-transporting ATPase subunit beta-1 OS=Rattus norvegicus GN=Atp1b1 PE=1 SV=1	SYEAYVLNIIR
>	sp|P07340|AT1 Sodium/potassium-transporting ATPase subunit beta-1 OS=Rattus norvegicus GN=Atp1b1 PE=1 SV=1	VAPPGLTQIPQIQK
>	sp|P07340|AT1 Sodium/potassium-transporting ATPase subunit beta-1 OS=Rattus norvegicus GN=Atp1b1 PE=1 SV=1	YNPNVLPVQCTGK
>	sp|P07340|AT1 Sodium/potassium-transporting ATPase subunit beta-1 OS=Rattus norvegicus GN=Atp1b1 PE=1 SV=1	YNPNVLPVQCTGK
>	sp|P07340|AT1 Sodium/potassium-transporting ATPase subunit beta-1 OS=Rattus norvegicus GN=Atp1b1 PE=1 SV=1	AYGENIGYSEK
>	sp|P07340|AT1 Sodium/potassium-transporting ATPase subunit beta-1 OS=Rattus norvegicus GN=Atp1b1 PE=1 SV=1	VAPPGLTQIPQIQK
>	sp|P26284|ODI Pyruvate dehydrogenase E1 component subunit alpha, somatic form, mitochondrial OS=Rattus norvegicus GN=Pdha1 PE=1 SV=2	GFCHLCDGQEACCVGLEAGINPTDHLITAYR
>	sp|P26284|ODI Pyruvate dehydrogenase E1 component subunit alpha, somatic form, mitochondrial OS=Rattus norvegicus GN=Pdha1 PE=1 SV=2	GPILMELQTYR
>	sp|P26284|ODI Pyruvate dehydrogenase E1 component subunit alpha, somatic form, mitochondrial OS=Rattus norvegicus GN=Pdha1 PE=1 SV=2	LEEGPPVTTVLTR
>	sp|P26284|ODI Pyruvate dehydrogenase E1 component subunit alpha, somatic form, mitochondrial OS=Rattus norvegicus GN=Pdha1 PE=1 SV=2	MVNSNLASVEELKEIDVEVR
>	sp|P26284|ODI Pyruvate dehydrogenase E1 component subunit alpha, somatic form, mitochondrial OS=Rattus norvegicus GN=Pdha1 PE=1 SV=2	KEIEDAAQFATADPEPPLEELGYHIYSSDPPFEVR
>	sp|P26284|ODI Pyruvate dehydrogenase E1 component subunit alpha, somatic form, mitochondrial OS=Rattus norvegicus GN=Pdha1 PE=1 SV=2	GFCHLCDGQEACCVGLEAGINPTDHLITAYR
>	sp|P26284|ODI Pyruvate dehydrogenase E1 component subunit alpha, somatic form, mitochondrial OS=Rattus norvegicus GN=Pdha1 PE=1 SV=2	GFCHLCDGQEACCVGLEAGINPTDHLITAYR
>	sp|P26284|ODI Pyruvate dehydrogenase E1 component subunit alpha, somatic form, mitochondrial OS=Rattus norvegicus GN=Pdha1 PE=1 SV=2	LEEGPPVTTVLTR
>	sp|P26284|ODI Pyruvate dehydrogenase E1 component subunit alpha, somatic form, mitochondrial OS=Rattus norvegicus GN=Pdha1 PE=1 SV=2	AILAELTGR
>	sp|P26284|ODI Pyruvate dehydrogenase E1 component subunit alpha, somatic form, mitochondrial OS=Rattus norvegicus GN=Pdha1 PE=1 SV=2	RGDFIPGLR
>	sp|P26284|ODI Pyruvate dehydrogenase E1 component subunit alpha, somatic form, mitochondrial OS=Rattus norvegicus GN=Pdha1 PE=1 SV=2	AILAELTGR
>	sp|P26284|ODI Pyruvate dehydrogenase E1 component subunit alpha, somatic form, mitochondrial OS=Rattus norvegicus GN=Pdha1 PE=1 SV=2	MVNSNLASVEELKEIDVEVR
>	sp|P26284|ODI Pyruvate dehydrogenase E1 component subunit alpha, somatic form, mitochondrial OS=Rattus norvegicus GN=Pdha1 PE=1 SV=2	AILAELTGR
>	sp|P26284|ODI Pyruvate dehydrogenase E1 component subunit alpha, somatic form, mitochondrial OS=Rattus norvegicus GN=Pdha1 PE=1 SV=2	RGDFIPGLR
>	sp|P26284|ODI Pyruvate dehydrogenase E1 component subunit alpha, somatic form, mitochondrial OS=Rattus norvegicus GN=Pdha1 PE=1 SV=2	EIEDAAQFATADPEPPLEELGYHIYSSDPPFEVR
>	sp|P26284|ODI Pyruvate dehydrogenase E1 component subunit alpha, somatic form, mitochondrial OS=Rattus norvegicus GN=Pdha1 PE=1 SV=2	KEIEDAAQFATADPEPPLEELGYHIYSSDPPFEVR
>	sp|P26284|ODI Pyruvate dehydrogenase E1 component subunit alpha, somatic form, mitochondrial OS=Rattus norvegicus GN=Pdha1 PE=1 SV=2	LPCIFICENNR
>	sp|P26284|ODI Pyruvate dehydrogenase E1 component subunit alpha, somatic form, mitochondrial OS=Rattus norvegicus GN=Pdha1 PE=1 SV=2	KEIEDAAQFATADPEPPLEELGYHIYSSDPP
>	sp|P26284|ODI Pyruvate dehydrogenase E1 component subunit alpha, somatic form, mitochondrial OS=Rattus norvegicus GN=Pdha1 PE=1 SV=2	AILAELTGR
>	sp|P26284|ODI Pyruvate dehydrogenase E1 component subunit alpha, somatic form, mitochondrial OS=Rattus norvegicus GN=Pdha1 PE=1 SV=2	KEIEDAAQFATADPEPPLEELGYHIYSSDPPFEVR
>	sp|P26284|ODI Pyruvate dehydrogenase E1 component subunit alpha, somatic form, mitochondrial OS=Rattus norvegicus GN=Pdha1 PE=1 SV=2	KEIEDAAQFATADPEPPLEELGYHIYSSDPPFEVR
>	sp|O88767|PAI Protein DJ-1 OS=Rattus norvegicus GN=Park7 PE=1 SV=1	GLIAAICAGPTALLAHEVGFGCK
>	sp|O88767|PAI Protein DJ-1 OS=Rattus norvegicus GN=Park7 PE=1 SV=1	GPGTSFEFALAIVEALSGK
>	sp|O88767|PAI Protein DJ-1 OS=Rattus norvegicus GN=Park7 PE=1 SV=1	GPGTSFEFALAIVEALSGKDMANQVK
>	sp|O88767|PAI Protein DJ-1 OS=Rattus norvegicus GN=Park7 PE=1 SV=1	KGLIAAICAGPTALLAHEVGFGCK
>	sp|O88767|PAI Protein DJ-1 OS=Rattus norvegicus GN=Park7 PE=1 SV=1	ALVILAK
>	sp|O88767|PAI Protein DJ-1 OS=Rattus norvegicus GN=Park7 PE=1 SV=1	GLIAAICAGPTALLAHEVGFGCK
>	sp|O88767|PAI Protein DJ-1 OS=Rattus norvegicus GN=Park7 PE=1 SV=1	KGLIAAICAGPTALLAHEVGFGCK
>	sp|O88767|PAI Protein DJ-1 OS=Rattus norvegicus GN=Park7 PE=1 SV=1	KGLIAAICAGPTALLAHEVGFGCK
>	sp|O88767|PAI Protein DJ-1 OS=Rattus norvegicus GN=Park7 PE=1 SV=1	KGLIAAICAGPTALLAHEVGFGCK
>	sp|O88767|PAI Protein DJ-1 OS=Rattus norvegicus GN=Park7 PE=1 SV=1	KGLIAAICAGPTALLAHEVGFGCK
>	sp|O88767|PAI Protein DJ-1 OS=Rattus norvegicus GN=Park7 PE=1 SV=1	KGLIAAICAGPTALLAHEVGFGCK
>	sp|O88767|PAI Protein DJ-1 OS=Rattus norvegicus GN=Park7 PE=1 SV=1	KGLIAAICAGPTALLAHEVGFGCK
>	sp|O88767|PAI Protein DJ-1 OS=Rattus norvegicus GN=Park7 PE=1 SV=1	KGLIAAICAGPTALLAHEVGFGCK
>	sp|O88767|PAI Protein DJ-1 OS=Rattus norvegicus GN=Park7 PE=1 SV=1	KGLIAAICAGPTALLAHEVGFGCK
>	sp|O88767|PAI Protein DJ-1 OS=Rattus norvegicus GN=Park7 PE=1 SV=1	KGLIAAICAGPTALLAHEVGFGCK
>	sp|O88767|PAI Protein DJ-1 OS=Rattus norvegicus GN=Park7 PE=1 SV=1	KGLIAAICAGPTALLAHEVGFGCK
>	sp|O88767|PAI Protein DJ-1 OS=Rattus norvegicus GN=Park7 PE=1 SV=1	KGLIAAICAGPTALLAHEVGFGCK
>	sp|O88767|PAI Protein DJ-1 OS=Rattus norvegicus GN=Park7 PE=1 SV=1	VTVAGLAGKDPVQCSR
>	sp|O88767|PAI Protein DJ-1 OS=Rattus norvegicus GN=Park7 PE=1 SV=1	VTSHPLAK
>	sp|O88767|PAI Protein DJ-1 OS=Rattus norvegicus GN=Park7 PE=1 SV=1	KGLIAAICAGPTALLAHEVGFGCK
>	sp|O88767|PAI Protein DJ-1 OS=Rattus norvegicus GN=Park7 PE=1 SV=1	VTVAGLAGKDPVQCSR
>	sp|O88767|PAI Protein DJ-1 OS=Rattus norvegicus GN=Park7 PE=1 SV=1	KGLIAAICAGPTALLAHEVGFGCK
>	sp|O88767|PAI Protein DJ-1 OS=Rattus norvegicus GN=Park7 PE=1 SV=1	VTSHPLAK
>	sp|O88767|PAI Protein DJ-1 OS=Rattus norvegicus GN=Park7 PE=1 SV=1	VTSHPLAK
>	sp|P35435|ATF ATP synthase subunit gamma, mitochondrial OS=Rattus norvegicus GN=Atp5c1 PE=1 SV=2	NASDMIDKLTLTFNR
>	sp|P35435|ATF ATP synthase subunit gamma, mitochondrial OS=Rattus norvegicus GN=Atp5c1 PE=1 SV=2	VYGTGSLALYEK
>	sp|P35435|ATF ATP synthase subunit gamma, mitochondrial OS=Rattus norvegicus GN=Atp5c1 PE=1 SV=2	HLIIGVSSDR
>	sp|P35435|ATF ATP synthase subunit gamma, mitochondrial OS=Rattus norvegicus GN=Atp5c1 PE=1 SV=2	EVMIVGIGEK
>	sp|P35435|ATF ATP synthase subunit gamma, mitochondrial OS=Rattus norvegicus GN=Atp5c1 PE=1 SV=2	THSDQFLVSFK
>	sp|P35435|ATF ATP synthase subunit gamma, mitochondrial OS=Rattus norvegicus GN=Atp5c1 PE=1 SV=2	KPPTFGDASVIALELLNSGYEFDEGSIIFNQFK
>	sp|P35435|ATF ATP synthase subunit gamma, mitochondrial OS=Rattus norvegicus GN=Atp5c1 PE=1 SV=2	KPPTFGDASVIALELLNSGYEFDEGSIIFNQFK
>	sp|P35435|ATF ATP synthase subunit gamma, mitochondrial OS=Rattus norvegicus GN=Atp5c1 PE=1 SV=2	KPPTFGDASVIALELLNSGYEFDEGSIIFNQFK
>	sp|P35435|ATF ATP synthase subunit gamma, mitochondrial OS=Rattus norvegicus GN=Atp5c1 PE=1 SV=2	NASDMIDKLTLTFNR
>	sp|P35435|ATF ATP synthase subunit gamma, mitochondrial OS=Rattus norvegicus GN=Atp5c1 PE=1 SV=2	NASDMIDKLTLTFNR
>	sp|P35435|ATF ATP synthase subunit gamma, mitochondrial OS=Rattus norvegicus GN=Atp5c1 PE=1 SV=2	NASDMIDKLTLTFNR
>	sp|P35435|ATF ATP synthase subunit gamma, mitochondrial OS=Rattus norvegicus GN=Atp5c1 PE=1 SV=2	EVMIVGIGEK

>	sp|P35435|ATF	ATP synthase subunit gamma, mitochondrial OS=Rattus norvegicus GN=Atp5c1 PE=1 SV=2	EVMIVGIGEK
>	sp|P35435|ATF	ATP synthase subunit gamma, mitochondrial OS=Rattus norvegicus GN=Atp5c1 PE=1 SV=2	NDMAALTAAGK
>	sp|P35435|ATF	ATP synthase subunit gamma, mitochondrial OS=Rattus norvegicus GN=Atp5c1 PE=1 SV=2	KPPTFGDASVIALELLNSGYEFDEGSIIFNQFK
>	sp|P35435|ATF	ATP synthase subunit gamma, mitochondrial OS=Rattus norvegicus GN=Atp5c1 PE=1 SV=2	THSDQFLVSFK
>	sp|P35435|ATF	ATP synthase subunit gamma, mitochondrial OS=Rattus norvegicus GN=Atp5c1 PE=1 SV=2	KPPTFGDASVIALELLNSGYEFDEGSIIFNQFK
>	sp|P35435|ATF	ATP synthase subunit gamma, mitochondrial OS=Rattus norvegicus GN=Atp5c1 PE=1 SV=2	MVAAAK
>	sp|P35435|ATF	ATP synthase subunit gamma, mitochondrial OS=Rattus norvegicus GN=Atp5c1 PE=1 SV=2	HLIIGVSSDR
>	sp|Q63560|MA	Microtubule-associated protein 6 OS=Rattus norvegicus GN=Map6 PE=1 SV=1	AKDQSFPAPAPTPLKDPGPVIPEPEKDGAPMVPER
>	sp|Q63560|MA	Microtubule-associated protein 6 OS=Rattus norvegicus GN=Map6 PE=1 SV=1	ATGPAPGPSGDRETAAAPGR
>	sp|Q63560|MA	Microtubule-associated protein 6 OS=Rattus norvegicus GN=Map6 PE=1 SV=1	AVAIETQPAQGESDAVAR
>	sp|Q63560|MA	Microtubule-associated protein 6 OS=Rattus norvegicus GN=Map6 PE=1 SV=1	RGDHPWIPKPVQIPATSQPSPPVLGMPK
>	sp|Q63560|MA	Microtubule-associated protein 6 OS=Rattus norvegicus GN=Map6 PE=1 SV=1	DPEGAGGAGVPAAGK
>	sp|Q63560|MA	Microtubule-associated protein 6 OS=Rattus norvegicus GN=Map6 PE=1 SV=1	TEGHEEKPLPPAQSQTQEGGPAAGK
>	sp|Q63560|MA	Microtubule-associated protein 6 OS=Rattus norvegicus GN=Map6 PE=1 SV=1	AKDQSFPAPAPTPLKDPGPVIPEPEKDGAPMVPER
>	sp|Q63560|MA	Microtubule-associated protein 6 OS=Rattus norvegicus GN=Map6 PE=1 SV=1	AKDQSFPAPAPTPLKDPGPVIPEPEKDGAPMVPER
>	sp|Q63560|MA	Microtubule-associated protein 6 OS=Rattus norvegicus GN=Map6 PE=1 SV=1	RGDHPWIPKPVQIPATSQPSPPVLGMPK
>	sp|Q63560|MA	Microtubule-associated protein 6 OS=Rattus norvegicus GN=Map6 PE=1 SV=1	TEGHEEKPLPPAQSQTQEGGPAAGK
>	sp|Q63560|MA	Microtubule-associated protein 6 OS=Rattus norvegicus GN=Map6 PE=1 SV=1	TEGHEEKPLPPAQSQTQEGGPAAGK
>	sp|Q63560|MA	Microtubule-associated protein 6 OS=Rattus norvegicus GN=Map6 PE=1 SV=1	TEGHEEKPLPPAQSQTQEGGPAAGK
>	sp|Q63560|MA	Microtubule-associated protein 6 OS=Rattus norvegicus GN=Map6 PE=1 SV=1	KAGPAWMVTR
>	sp|Q63560|MA	Microtubule-associated protein 6 OS=Rattus norvegicus GN=Map6 PE=1 SV=1	AKDQSFPAPAPTPLKDPGPVIPEPEKDGAPMVPER
>	sp|Q63560|MA	Microtubule-associated protein 6 OS=Rattus norvegicus GN=Map6 PE=1 SV=1	DTGTDLKGHGSVFVAPVK
>	sp|Q63560|MA	Microtubule-associated protein 6 OS=Rattus norvegicus GN=Map6 PE=1 SV=1	AKDQSFPAPAPTPLKDPGPVIPEPEKDGAPMVPER
>	sp|Q63560|MA	Microtubule-associated protein 6 OS=Rattus norvegicus GN=Map6 PE=1 SV=1	AKDQSFPAPAPTPLKDPGPVIPEPEKDGAPMVPER
>	sp|Q63560|MA	Microtubule-associated protein 6 OS=Rattus norvegicus GN=Map6 PE=1 SV=1	RGDHPWIPKPVQIPATSQPSPPVLGMPK
>	sp|Q63560|MA	Microtubule-associated protein 6 OS=Rattus norvegicus GN=Map6 PE=1 SV=1	AKPQYKPPDDK
>	sp|Q63560|MA	Microtubule-associated protein 6 OS=Rattus norvegicus GN=Map6 PE=1 SV=1	RGDHPWIPKPVQIPATSQPSPPVLGMPK
>	sp|O35244|PRI	Peroxiredoxin-6 OS=Rattus norvegicus GN=Prdx6 PE=1 SV=3	DFTPVCTTELGR
>	sp|O35244|PRI	Peroxiredoxin-6 OS=Rattus norvegicus GN=Prdx6 PE=1 SV=3	FHDFLGDSWGILFSHPR
>	sp|O35244|PRI	Peroxiredoxin-6 OS=Rattus norvegicus GN=Prdx6 PE=1 SV=3	KGESVMVLPTLPEEEAK
>	sp|O35244|PRI	Peroxiredoxin-6 OS=Rattus norvegicus GN=Prdx6 PE=1 SV=3	LIALSIDSVEDHFAWSK
>	sp|O35244|PRI	Peroxiredoxin-6 OS=Rattus norvegicus GN=Prdx6 PE=1 SV=3	LPFPIIDDKDRDLAILLGMLDPAEKDEK
>	sp|O35244|PRI	Peroxiredoxin-6 OS=Rattus norvegicus GN=Prdx6 PE=1 SV=3	VVDSLQLTASNPVATPVDWK
>	sp|O35244|PRI	Peroxiredoxin-6 OS=Rattus norvegicus GN=Prdx6 PE=1 SV=3	FHDFLGDSWGILFSHPR
>	sp|O35244|PRI	Peroxiredoxin-6 OS=Rattus norvegicus GN=Prdx6 PE=1 SV=3	KGESVMVLPTLPEEEAK
>	sp|O35244|PRI	Peroxiredoxin-6 OS=Rattus norvegicus GN=Prdx6 PE=1 SV=3	KGESVMVLPTLPEEEAK
>	sp|O35244|PRI	Peroxiredoxin-6 OS=Rattus norvegicus GN=Prdx6 PE=1 SV=3	KGESVMVLPTLPEEEAK
>	sp|O35244|PRI	Peroxiredoxin-6 OS=Rattus norvegicus GN=Prdx6 PE=1 SV=3	LIALSIDSVEDHFAWSK
>	sp|O35244|PRI	Peroxiredoxin-6 OS=Rattus norvegicus GN=Prdx6 PE=1 SV=3	LPFPIIDDKDRDLAILLGMLDPAEKDEK
>	sp|O35244|PRI	Peroxiredoxin-6 OS=Rattus norvegicus GN=Prdx6 PE=1 SV=3	LPFPIIDDKDRDLAILLGMLDPAEKDEK
>	sp|O35244|PRI	Peroxiredoxin-6 OS=Rattus norvegicus GN=Prdx6 PE=1 SV=3	LPFPIIDDKDRDLAILLGMLDPAEKDEK
>	sp|O35244|PRI	Peroxiredoxin-6 OS=Rattus norvegicus GN=Prdx6 PE=1 SV=3	LPFPIIDDKDRDLAILLGMLDPAEKDEK
>	sp|O35244|PRI	Peroxiredoxin-6 OS=Rattus norvegicus GN=Prdx6 PE=1 SV=3	LPFPIIDDKDRDLAILLGMLDPAEKDEK
>	sp|O35244|PRI	Peroxiredoxin-6 OS=Rattus norvegicus GN=Prdx6 PE=1 SV=3	LPFPIIDDKDRDLAILLGMLDPAEKDEK
>	sp|O35244|PRI	Peroxiredoxin-6 OS=Rattus norvegicus GN=Prdx6 PE=1 SV=3	LPFPIIDDKDRDLAILLGMLDPAEKDEK
>	sp|O35244|PRI	Peroxiredoxin-6 OS=Rattus norvegicus GN=Prdx6 PE=1 SV=3	LPFPIIDDKDRDLAILLGMLDPAEKDEK
>	sp|O35244|PRI	Peroxiredoxin-6 OS=Rattus norvegicus GN=Prdx6 PE=1 SV=3	LPFPIIDDKDRDLAILLGMLDPAEKDEK
>	sp|O35244|PRI	Peroxiredoxin-6 OS=Rattus norvegicus GN=Prdx6 PE=1 SV=3	LPFPIIDDKDRDLAILLGMLDPAEKDEK
>	sp|O35244|PRI	Peroxiredoxin-6 OS=Rattus norvegicus GN=Prdx6 PE=1 SV=3	LPFPIIDDKDRDLAILLGMLDPAEKDEK
>	sp|O35244|PRI	Peroxiredoxin-6 OS=Rattus norvegicus GN=Prdx6 PE=1 SV=3	VVDSLQLTASNPVATPVDWK
>	sp|O35244|PRI	Peroxiredoxin-6 OS=Rattus norvegicus GN=Prdx6 PE=1 SV=3	VVDSLQLTASNPVATPVDWK
>	sp|O35244|PRI	Peroxiredoxin-6 OS=Rattus norvegicus GN=Prdx6 PE=1 SV=3	LPFPIIDDKDRDLAILLGMLDPAEKDEK
>	sp|O35244|PRI	Peroxiredoxin-6 OS=Rattus norvegicus GN=Prdx6 PE=1 SV=3	LSILYPATTGR
>	sp|O35244|PRI	Peroxiredoxin-6 OS=Rattus norvegicus GN=Prdx6 PE=1 SV=3	VVDSLQLTASNPVATPVDWK
>	sp|O35244|PRI	Peroxiredoxin-6 OS=Rattus norvegicus GN=Prdx6 PE=1 SV=3	LPFPIIDDKDRDLAILLGMLDPAEKDEK
>	sp|P46462|TER	Transitional endoplasmic reticulum ATPase OS=Rattus norvegicus GN=Vcp PE=1 SV=3	KNVFIIGATNRPDIIDPAILRPGR
>	sp|P46462|TER	Transitional endoplasmic reticulum ATPase OS=Rattus norvegicus GN=Vcp PE=1 SV=3	LADDVDLEQVANETHGHVGADLAALCSEAALQAIR
>	sp|P46462|TER	Transitional endoplasmic reticulum ATPase OS=Rattus norvegicus GN=Vcp PE=1 SV=3	NAPAIIFIDELDAIAPK
>	sp|P46462|TER	Transitional endoplasmic reticulum ATPase OS=Rattus norvegicus GN=Vcp PE=1 SV=3	NVFIIGATNRPDIIDPAILRPGR
>	sp|P46462|TER	Transitional endoplasmic reticulum ATPase OS=Rattus norvegicus GN=Vcp PE=1 SV=3	VVETDPSPYCIVAPDTVIHCEGEPIKREDEEESLNEVGYDDIGGCR
>	sp|P46462|TER	Transitional endoplasmic reticulum ATPase OS=Rattus norvegicus GN=Vcp PE=1 SV=3	EMVELPLRHPALFK
>	sp|P46462|TER	Transitional endoplasmic reticulum ATPase OS=Rattus norvegicus GN=Vcp PE=1 SV=3	KNVFIIGATNRPDIIDPAILRPGR
>	sp|P46462|TER	Transitional endoplasmic reticulum ATPase OS=Rattus norvegicus GN=Vcp PE=1 SV=3	LADDVDLEQVANETHGHVGADLAALCSEAALQAIR
>	sp|P46462|TER	Transitional endoplasmic reticulum ATPase OS=Rattus norvegicus GN=Vcp PE=1 SV=3	LADDVDLEQVANETHGHVGADLAALCSEAALQAIR
>	sp|P46462|TER	Transitional endoplasmic reticulum ATPase OS=Rattus norvegicus GN=Vcp PE=1 SV=3	LADDVDLEQVANETHGHVGADLAALCSEAALQAIR
>	sp|P46462|TER	Transitional endoplasmic reticulum ATPase OS=Rattus norvegicus GN=Vcp PE=1 SV=3	LADDVDLEQVANETHGHVGADLAALCSEAALQAIR
>	sp|P46462|TER	Transitional endoplasmic reticulum ATPase OS=Rattus norvegicus GN=Vcp PE=1 SV=3	NAPAIIFIDELDAIAPK
>	sp|P46462|TER	Transitional endoplasmic reticulum ATPase OS=Rattus norvegicus GN=Vcp PE=1 SV=3	NAPAIIFIDELDAIAPK
>	sp|P46462|TER	Transitional endoplasmic reticulum ATPase OS=Rattus norvegicus GN=Vcp PE=1 SV=3	QAAPCVLFFDELDSIAK
>	sp|P46462|TER	Transitional endoplasmic reticulum ATPase OS=Rattus norvegicus GN=Vcp PE=1 SV=3	KNVFIIGATNRPDIIDPAILRPGR
>	sp|P46462|TER	Transitional endoplasmic reticulum ATPase OS=Rattus norvegicus GN=Vcp PE=1 SV=3	LADDVDLEQVANETHGHVGADLAALCSEAALQAIR
>	sp|P46462|TER	Transitional endoplasmic reticulum ATPase OS=Rattus norvegicus GN=Vcp PE=1 SV=3	NVFIIGATNRPDIIDPAILRPGR
>	sp|P46462|TER	Transitional endoplasmic reticulum ATPase OS=Rattus norvegicus GN=Vcp PE=1 SV=3	VVETDPSPYCIVAPDTVIHCEGEPIKREDEEESLNEVGYDDIGGCR
>	sp|P46462|TER	Transitional endoplasmic reticulum ATPase OS=Rattus norvegicus GN=Vcp PE=1 SV=3	LADDVDLEQVANETHGHVGADLAALCSEAALQAIR

>	sp|P46462|TER Transitional endoplasmic reticulum ATPase OS=Rattus norvegicus GN=Vcp PE=1 SV=3	ELQELVQYPVEHPDKFLK
>	sp|P62630|EF1 Elongation factor 1-alpha 1 OS=Rattus norvegicus GN=Eef1a1 PE=1 SV=1	DGSASGTTLLEALDCILPPTRPTDKPLRLPLQDVYK
>	sp|P62630|EF1 Elongation factor 1-alpha 1 OS=Rattus norvegicus GN=Eef1a1 PE=1 SV=1	EHALLAYTLGVK
>	sp|P62630|EF1 Elongation factor 1-alpha 1 OS=Rattus norvegicus GN=Eef1a1 PE=1 SV=1	KDGSASGTTLLEALDCILPPTRPTDKPLR
>	sp|P62630|EF1 Elongation factor 1-alpha 1 OS=Rattus norvegicus GN=Eef1a1 PE=1 SV=1	KDGSASGTTLLEALDCILPPTRPTDKPLRLPLQDVYK
>	sp|P62630|EF1 Elongation factor 1-alpha 1 OS=Rattus norvegicus GN=Eef1a1 PE=1 SV=1	VETGVLKPGMVVTFAPVNVTTEVK
>	sp|P62630|EF1 Elongation factor 1-alpha 1 OS=Rattus norvegicus GN=Eef1a1 PE=1 SV=1	IGGIGTVPVGR
>	sp|P62630|EF1 Elongation factor 1-alpha 1 OS=Rattus norvegicus GN=Eef1a1 PE=1 SV=1	GSFKYAWVLDKLK
>	sp|P62630|EF1 Elongation factor 1-alpha 1 OS=Rattus norvegicus GN=Eef1a1 PE=1 SV=1	DGSASGTTLLEALDCILPPTRPTDKPLRLPLQDVYK
>	sp|P62630|EF1 Elongation factor 1-alpha 1 OS=Rattus norvegicus GN=Eef1a1 PE=1 SV=1	DGSASGTTLLEALDCILPPTRPTDKPLRLPLQDVYK
>	sp|P62630|EF1 Elongation factor 1-alpha 1 OS=Rattus norvegicus GN=Eef1a1 PE=1 SV=1	DGSASGTTLLEALDCILPPTRPTDKPLRLPLQDVYK
>	sp|P62630|EF1 Elongation factor 1-alpha 1 OS=Rattus norvegicus GN=Eef1a1 PE=1 SV=1	DGSASGTTLLEALDCILPPTRPTDKPLRLPLQDVYK
>	sp|P62630|EF1 Elongation factor 1-alpha 1 OS=Rattus norvegicus GN=Eef1a1 PE=1 SV=1	DGSASGTTLLEALDCILPPTRPTDKPLRLPLQDVYK
>	sp|P62630|EF1 Elongation factor 1-alpha 1 OS=Rattus norvegicus GN=Eef1a1 PE=1 SV=1	DGSASGTTLLEALDCILPPTRPTDKPLRLPLQDVYK
>	sp|P62630|EF1 Elongation factor 1-alpha 1 OS=Rattus norvegicus GN=Eef1a1 PE=1 SV=1	EHALLAYTLGVK
>	sp|P62630|EF1 Elongation factor 1-alpha 1 OS=Rattus norvegicus GN=Eef1a1 PE=1 SV=1	EHALLAYTLGVK
>	sp|P62630|EF1 Elongation factor 1-alpha 1 OS=Rattus norvegicus GN=Eef1a1 PE=1 SV=1	GSFKYAWVLDKLK
>	sp|P62630|EF1 Elongation factor 1-alpha 1 OS=Rattus norvegicus GN=Eef1a1 PE=1 SV=1	KDGSASGTTLLEALDCILPPTRPTDKPLR
>	sp|P62630|EF1 Elongation factor 1-alpha 1 OS=Rattus norvegicus GN=Eef1a1 PE=1 SV=1	KDGSASGTTLLEALDCILPPTRPTDKPLR
>	sp|P62630|EF1 Elongation factor 1-alpha 1 OS=Rattus norvegicus GN=Eef1a1 PE=1 SV=1	KDGSASGTTLLEALDCILPPTRPTDKPLR
>	sp|P62630|EF1 Elongation factor 1-alpha 1 OS=Rattus norvegicus GN=Eef1a1 PE=1 SV=1	KDGSASGTTLLEALDCILPPTRPTDKPLR
>	sp|P62630|EF1 Elongation factor 1-alpha 1 OS=Rattus norvegicus GN=Eef1a1 PE=1 SV=1	KDGSASGTTLLEALDCILPPTRPTDKPLR
>	sp|P62630|EF1 Elongation factor 1-alpha 1 OS=Rattus norvegicus GN=Eef1a1 PE=1 SV=1	KDGSASGTTLLEALDCILPPTRPTDKPLR
>	sp|P62630|EF1 Elongation factor 1-alpha 1 OS=Rattus norvegicus GN=Eef1a1 PE=1 SV=1	KDGSASGTTLLEALDCILPPTRPTDKPLR
>	sp|P62630|EF1 Elongation factor 1-alpha 1 OS=Rattus norvegicus GN=Eef1a1 PE=1 SV=1	KDGSASGTTLLEALDCILPPTRPTDKPLRLPLQDVYK
>	sp|P62630|EF1 Elongation factor 1-alpha 1 OS=Rattus norvegicus GN=Eef1a1 PE=1 SV=1	KDGSASGTTLLEALDCILPPTRPTDKPLRLPLQDVYK
>	sp|P62630|EF1 Elongation factor 1-alpha 1 OS=Rattus norvegicus GN=Eef1a1 PE=1 SV=1	KDGSASGTTLLEALDCILPPTRPTDKPLRLPLQDVYK
>	sp|P62630|EF1 Elongation factor 1-alpha 1 OS=Rattus norvegicus GN=Eef1a1 PE=1 SV=1	KDGSASGTTLLEALDCILPPTRPTDKPLRLPLQDVYK
>	sp|P62630|EF1 Elongation factor 1-alpha 1 OS=Rattus norvegicus GN=Eef1a1 PE=1 SV=1	KDGSASGTTLLEALDCILPPTRPTDKPLRLPLQDVYK
>	sp|P62630|EF1 Elongation factor 1-alpha 1 OS=Rattus norvegicus GN=Eef1a1 PE=1 SV=1	KDGSASGTTLLEALDCILPPTRPTDKPLRLPLQDVYK
>	sp|P62630|EF1 Elongation factor 1-alpha 1 OS=Rattus norvegicus GN=Eef1a1 PE=1 SV=1	KDGSASGTTLLEALDCILPPTRPTDKPLRLPLQDVYK
>	sp|P62630|EF1 Elongation factor 1-alpha 1 OS=Rattus norvegicus GN=Eef1a1 PE=1 SV=1	KDGSASGTTLLEALDCILPPTRPTDKPLRLPLQDVYK
>	sp|P62630|EF1 Elongation factor 1-alpha 1 OS=Rattus norvegicus GN=Eef1a1 PE=1 SV=1	VETGVLKPGMVVTFAPVNVTTEVK
>	sp|P62630|EF1 Elongation factor 1-alpha 1 OS=Rattus norvegicus GN=Eef1a1 PE=1 SV=1	VETGVLKPGMVVTFAPVNVTTEVK
>	sp|P62630|EF1 Elongation factor 1-alpha 1 OS=Rattus norvegicus GN=Eef1a1 PE=1 SV=1	VETGVLKPGMVVTFAPVNVTTEVK
>	sp|P62630|EF1 Elongation factor 1-alpha 1 OS=Rattus norvegicus GN=Eef1a1 PE=1 SV=1	IGGIGTVPVGR
>	sp|P62630|EF1 Elongation factor 1-alpha 1 OS=Rattus norvegicus GN=Eef1a1 PE=1 SV=1	GSFKYAWVLDKLK
>	sp|P62630|EF1 Elongation factor 1-alpha 1 OS=Rattus norvegicus GN=Eef1a1 PE=1 SV=1	DGSASGTTLLEALDCILPPTRPTDKPLRLPLQDVYK
>	sp|P62630|EF1 Elongation factor 1-alpha 1 OS=Rattus norvegicus GN=Eef1a1 PE=1 SV=1	KDGSASGTTLLEALDCILPPTRPTDKPLRLPLQDVYK
>	sp|P62630|EF1 Elongation factor 1-alpha 1 OS=Rattus norvegicus GN=Eef1a1 PE=1 SV=1	IGGIGTVPVGR
>	sp|P62630|EF1 Elongation factor 1-alpha 1 OS=Rattus norvegicus GN=Eef1a1 PE=1 SV=1	SGDAAIVDMVPGKPMCVESFSDYPPLGR
>	sp|P62630|EF1 Elongation factor 1-alpha 1 OS=Rattus norvegicus GN=Eef1a1 PE=1 SV=1	VETGVLKPGMVVTFAPVNVTTEVK
>	sp|P62630|EF1 Elongation factor 1-alpha 1 OS=Rattus norvegicus GN=Eef1a1 PE=1 SV=1	NDPPMEAAGFTAQVIILNHPGQISAGYAPVLDCHTAHIACK
>	sp|P62630|EF1 Elongation factor 1-alpha 1 OS=Rattus norvegicus GN=Eef1a1 PE=1 SV=1	EHALLAYTLGVK
>	sp|P62630|EF1 Elongation factor 1-alpha 1 OS=Rattus norvegicus GN=Eef1a1 PE=1 SV=1	DGSASGTTLLEALDCILPPTRPTDKPLRLPLQDVYK
>	sp|P62630|EF1 Elongation factor 1-alpha 1 OS=Rattus norvegicus GN=Eef1a1 PE=1 SV=1	IGGIGTVPVGR
>	sp|P62630|EF1 Elongation factor 1-alpha 1 OS=Rattus norvegicus GN=Eef1a1 PE=1 SV=1	KLEDGPK
>	sp|P62630|EF1 Elongation factor 1-alpha 1 OS=Rattus norvegicus GN=Eef1a1 PE=1 SV=1	KDGSASGTTLLEALDCILPPTRPTDKPLR
>	sp|P62630|EF1 Elongation factor 1-alpha 1 OS=Rattus norvegicus GN=Eef1a1 PE=1 SV=1	KLEDGPK
>	sp|P62630|EF1 Elongation factor 1-alpha 1 OS=Rattus norvegicus GN=Eef1a1 PE=1 SV=1	NDPPMEAAGFTAQVIILNHPGQISAGYAPVLDCHTAHIACK
>	sp|P62630|EF1 Elongation factor 1-alpha 1 OS=Rattus norvegicus GN=Eef1a1 PE=1 SV=1	NDPPMEAAGFTAQVIILNHPGQISAGYAPVLDCHTAHIACK
>	sp|P62630|EF1 Elongation factor 1-alpha 1 OS=Rattus norvegicus GN=Eef1a1 PE=1 SV=1	DGSASGTTLLEALDCILPPTRPTDKPLRLPLQDVYK
>	sp|P62630|EF1 Elongation factor 1-alpha 1 OS=Rattus norvegicus GN=Eef1a1 PE=1 SV=1	NDPPMEAAGFTAQVIILNHPGQISAGYAPVLDCHTAHIACK
>	sp|P62260|143 14-3-3 protein epsilon OS=Rattus norvegicus GN=Ywhae PE=1 SV=1	AAFDDAIAELDTLSEESYK
>	sp|P62260|143 14-3-3 protein epsilon OS=Rattus norvegicus GN=Ywhae PE=1 SV=1	KVAGMDVELTVEER
>	sp|P62260|143 14-3-3 protein epsilon OS=Rattus norvegicus GN=Ywhae PE=1 SV=1	LICCDILDVLDK
>	sp|P62260|143 14-3-3 protein epsilon OS=Rattus norvegicus GN=Ywhae PE=1 SV=1	QMVETELK
>	sp|P62260|143 14-3-3 protein epsilon OS=Rattus norvegicus GN=Ywhae PE=1 SV=1	EAAENSLVAYK
>	sp|P62260|143 14-3-3 protein epsilon OS=Rattus norvegicus GN=Ywhae PE=1 SV=1	LICCDILDVLDKHLIPAANTGESK
>	sp|P62260|143 14-3-3 protein epsilon OS=Rattus norvegicus GN=Ywhae PE=1 SV=1	AAFDDAIAELDTLSEESYK
>	sp|P62260|143 14-3-3 protein epsilon OS=Rattus norvegicus GN=Ywhae PE=1 SV=1	DSTLIMQLLR
>	sp|P62260|143 14-3-3 protein epsilon OS=Rattus norvegicus GN=Ywhae PE=1 SV=1	EAAENSLVAYK
>	sp|P62260|143 14-3-3 protein epsilon OS=Rattus norvegicus GN=Ywhae PE=1 SV=1	KVAGMDVELTVEER
>	sp|P62260|143 14-3-3 protein epsilon OS=Rattus norvegicus GN=Ywhae PE=1 SV=1	KVAGMDVELTVEER
>	sp|P62260|143 14-3-3 protein epsilon OS=Rattus norvegicus GN=Ywhae PE=1 SV=1	KVAGMDVELTVEER
>	sp|P62260|143 14-3-3 protein epsilon OS=Rattus norvegicus GN=Ywhae PE=1 SV=1	LICCDILDVLDK
>	sp|P62260|143 14-3-3 protein epsilon OS=Rattus norvegicus GN=Ywhae PE=1 SV=1	LICCDILDVLDK
>	sp|P62260|143 14-3-3 protein epsilon OS=Rattus norvegicus GN=Ywhae PE=1 SV=1	LICCDILDVLDK
>	sp|P62260|143 14-3-3 protein epsilon OS=Rattus norvegicus GN=Ywhae PE=1 SV=1	LICCDILDVLDK
>	sp|P62260|143 14-3-3 protein epsilon OS=Rattus norvegicus GN=Ywhae PE=1 SV=1	LICCDILDVLDK
>	sp|P62260|143 14-3-3 protein epsilon OS=Rattus norvegicus GN=Ywhae PE=1 SV=1	LICCDILDVLDKHLIPAANTGESK
>	sp|P62260|143 14-3-3 protein epsilon OS=Rattus norvegicus GN=Ywhae PE=1 SV=1	LICCDILDVLDKHLIPAANTGESK
>	sp|P62260|143 14-3-3 protein epsilon OS=Rattus norvegicus GN=Ywhae PE=1 SV=1	LICCDILDVLDKHLIPAANTGESK
>	sp|P62260|143 14-3-3 protein epsilon OS=Rattus norvegicus GN=Ywhae PE=1 SV=1	LICCDILDVLDKHLIPAANTGESK

>	sp|P62260|143 14-3-3 protein epsilon OS=Rattus norvegicus GN=Ywhae PE=1 SV=1	NLLSVAYK
>	sp|P62260|143 14-3-3 protein epsilon OS=Rattus norvegicus GN=Ywhae PE=1 SV=1	NLLSVAYK
>	sp|P62260|143 14-3-3 protein epsilon OS=Rattus norvegicus GN=Ywhae PE=1 SV=1	NLLSVAYK
>	sp|P62260|143 14-3-3 protein epsilon OS=Rattus norvegicus GN=Ywhae PE=1 SV=1	NLLSVAYK
>	sp|P62260|143 14-3-3 protein epsilon OS=Rattus norvegicus GN=Ywhae PE=1 SV=1	NLLSVAYK
>	sp|P62260|143 14-3-3 protein epsilon OS=Rattus norvegicus GN=Ywhae PE=1 SV=1	AAFDDAIAELDTLSEESYKDSTLIMQLLR
>	sp|P62260|143 14-3-3 protein epsilon OS=Rattus norvegicus GN=Ywhae PE=1 SV=1	LICCDILDVLDK
>	sp|P62260|143 14-3-3 protein epsilon OS=Rattus norvegicus GN=Ywhae PE=1 SV=1	NLLSVAYK
>	sp|P62260|143 14-3-3 protein epsilon OS=Rattus norvegicus GN=Ywhae PE=1 SV=1	LICCDILDVLDK
>	sp|P62260|143 14-3-3 protein epsilon OS=Rattus norvegicus GN=Ywhae PE=1 SV=1	NLLSVAYK
>	sp|P62260|143 14-3-3 protein epsilon OS=Rattus norvegicus GN=Ywhae PE=1 SV=1	LICCDILDVLDK
>	sp|P62260|143 14-3-3 protein epsilon OS=Rattus norvegicus GN=Ywhae PE=1 SV=1	NLLSVAYK
>	sp|P62260|143 14-3-3 protein epsilon OS=Rattus norvegicus GN=Ywhae PE=1 SV=1	LICCDILDVLDKHLIPAANTGESK
>	sp|P62260|143 14-3-3 protein epsilon OS=Rattus norvegicus GN=Ywhae PE=1 SV=1	NLLSVAYK
>	sp|P62260|143 14-3-3 protein epsilon OS=Rattus norvegicus GN=Ywhae PE=1 SV=1	LICCDILDVLDKHLIPAANTGESK
>	sp|P62260|143 14-3-3 protein epsilon OS=Rattus norvegicus GN=Ywhae PE=1 SV=1	KVAGMDVELTVEER
>	sp|P62260|143 14-3-3 protein epsilon OS=Rattus norvegicus GN=Ywhae PE=1 SV=1	AAFDDAIAELDTLSEESYK
>	sp|P62260|143 14-3-3 protein epsilon OS=Rattus norvegicus GN=Ywhae PE=1 SV=1	YDEMVESMK
>	sp|P62260|143 14-3-3 protein epsilon OS=Rattus norvegicus GN=Ywhae PE=1 SV=1	YLAEFATGNDRK
>	sp|P62260|143 14-3-3 protein epsilon OS=Rattus norvegicus GN=Ywhae PE=1 SV=1	QMVETELK
>	sp|P62260|143 14-3-3 protein epsilon OS=Rattus norvegicus GN=Ywhae PE=1 SV=1	KVAGMDVELTVEER
>	sp|P62260|143 14-3-3 protein epsilon OS=Rattus norvegicus GN=Ywhae PE=1 SV=1	LICCDILDVLDKHLIPAANTGESK
>	sp|P60203|MY Myelin proteolipid protein OS=Rattus norvegicus GN=Plp1 PE=1 SV=2	GLSATVTGGQK
>	sp|P60203|MY Myelin proteolipid protein OS=Rattus norvegicus GN=Plp1 PE=1 SV=2	LIETYFSK
>	sp|P60203|MY Myelin proteolipid protein OS=Rattus norvegicus GN=Plp1 PE=1 SV=2	MYGVLPWNAFPGK
>	sp|P60203|MY Myelin proteolipid protein OS=Rattus norvegicus GN=Plp1 PE=1 SV=2	TSASIGSLCADAR
>	sp|P60203|MY Myelin proteolipid protein OS=Rattus norvegicus GN=Plp1 PE=1 SV=2	VCGSNLLSICK
>	sp|P60203|MY Myelin proteolipid protein OS=Rattus norvegicus GN=Plp1 PE=1 SV=2	QIFGDYK
>	sp|P60203|MY Myelin proteolipid protein OS=Rattus norvegicus GN=Plp1 PE=1 SV=2	GLSATVTGGQK
>	sp|P60203|MY Myelin proteolipid protein OS=Rattus norvegicus GN=Plp1 PE=1 SV=2	LIETYFSK
>	sp|P60203|MY Myelin proteolipid protein OS=Rattus norvegicus GN=Plp1 PE=1 SV=2	LIETYFSK
>	sp|P60203|MY Myelin proteolipid protein OS=Rattus norvegicus GN=Plp1 PE=1 SV=2	LIETYFSK
>	sp|P60203|MY Myelin proteolipid protein OS=Rattus norvegicus GN=Plp1 PE=1 SV=2	MYGVLPWNAFPGK
>	sp|P60203|MY Myelin proteolipid protein OS=Rattus norvegicus GN=Plp1 PE=1 SV=2	MYGVLPWNAFPGK
>	sp|P60203|MY Myelin proteolipid protein OS=Rattus norvegicus GN=Plp1 PE=1 SV=2	MYGVLPWNAFPGK
>	sp|P60203|MY Myelin proteolipid protein OS=Rattus norvegicus GN=Plp1 PE=1 SV=2	MYGVLPWNAFPGK
>	sp|P60203|MY Myelin proteolipid protein OS=Rattus norvegicus GN=Plp1 PE=1 SV=2	MYGVLPWNAFPGK
>	sp|P60203|MY Myelin proteolipid protein OS=Rattus norvegicus GN=Plp1 PE=1 SV=2	MYGVLPWNAFPGK
>	sp|P60203|MY Myelin proteolipid protein OS=Rattus norvegicus GN=Plp1 PE=1 SV=2	MYGVLPWNAFPGK
>	sp|P60203|MY Myelin proteolipid protein OS=Rattus norvegicus GN=Plp1 PE=1 SV=2	TSASIGSLCADAR
>	sp|P60203|MY Myelin proteolipid protein OS=Rattus norvegicus GN=Plp1 PE=1 SV=2	TSASIGSLCADAR
>	sp|P60203|MY Myelin proteolipid protein OS=Rattus norvegicus GN=Plp1 PE=1 SV=2	TSASIGSLCADAR
>	sp|P60203|MY Myelin proteolipid protein OS=Rattus norvegicus GN=Plp1 PE=1 SV=2	TSASIGSLCADAR
>	sp|P60203|MY Myelin proteolipid protein OS=Rattus norvegicus GN=Plp1 PE=1 SV=2	TSASIGSLCADAR
>	sp|P60203|MY Myelin proteolipid protein OS=Rattus norvegicus GN=Plp1 PE=1 SV=2	TSASIGSLCADAR
>	sp|P60203|MY Myelin proteolipid protein OS=Rattus norvegicus GN=Plp1 PE=1 SV=2	TSASIGSLCADAR
>	sp|P60203|MY Myelin proteolipid protein OS=Rattus norvegicus GN=Plp1 PE=1 SV=2	TSASIGSLCADAR
>	sp|P60203|MY Myelin proteolipid protein OS=Rattus norvegicus GN=Plp1 PE=1 SV=2	VCGSNLLSICK
>	sp|P60203|MY Myelin proteolipid protein OS=Rattus norvegicus GN=Plp1 PE=1 SV=2	MYGVLPWNAFPGK
>	sp|P60203|MY Myelin proteolipid protein OS=Rattus norvegicus GN=Plp1 PE=1 SV=2	VCGSNLLSICK
>	sp|P60203|MY Myelin proteolipid protein OS=Rattus norvegicus GN=Plp1 PE=1 SV=2	LIETYFSK
>	sp|P60203|MY Myelin proteolipid protein OS=Rattus norvegicus GN=Plp1 PE=1 SV=2	LIETYFSK
>	sp|P60203|MY Myelin proteolipid protein OS=Rattus norvegicus GN=Plp1 PE=1 SV=2	TSASIGSLCADAR
>	sp|P60203|MY Myelin proteolipid protein OS=Rattus norvegicus GN=Plp1 PE=1 SV=2	MYGVLPWNAFPGK
>	sp|P60203|MY Myelin proteolipid protein OS=Rattus norvegicus GN=Plp1 PE=1 SV=2	MYGVLPWNAFPGK
>	sp|P60203|MY Myelin proteolipid protein OS=Rattus norvegicus GN=Plp1 PE=1 SV=2	LIETYFSK
>	sp|P60203|MY Myelin proteolipid protein OS=Rattus norvegicus GN=Plp1 PE=1 SV=2	VCGSNLLSICK
>	sp|P60203|MY Myelin proteolipid protein OS=Rattus norvegicus GN=Plp1 PE=1 SV=2	LIETYFSK
>	sp|P60203|MY Myelin proteolipid protein OS=Rattus norvegicus GN=Plp1 PE=1 SV=2	GLSATVTGGQK
>	sp|P60203|MY Myelin proteolipid protein OS=Rattus norvegicus GN=Plp1 PE=1 SV=2	LIETYFSK
>	sp|P60203|MY Myelin proteolipid protein OS=Rattus norvegicus GN=Plp1 PE=1 SV=2	MYGVLPWNAFPGK
>	sp|P60203|MY Myelin proteolipid protein OS=Rattus norvegicus GN=Plp1 PE=1 SV=2	MYGVLPWNAFPGK
>	sp|P60203|MY Myelin proteolipid protein OS=Rattus norvegicus GN=Plp1 PE=1 SV=2	LIETYFSK
>	sp|P60203|MY Myelin proteolipid protein OS=Rattus norvegicus GN=Plp1 PE=1 SV=2	GLSATVTGGQK
>	sp|P60203|MY Myelin proteolipid protein OS=Rattus norvegicus GN=Plp1 PE=1 SV=2	VCGSNLLSICK
>	sp|P60203|MY Myelin proteolipid protein OS=Rattus norvegicus GN=Plp1 PE=1 SV=2	QIFGDYK
>	sp|P60203|MY Myelin proteolipid protein OS=Rattus norvegicus GN=Plp1 PE=1 SV=2	PWNAFPGK
>	sp|P60203|MY Myelin proteolipid protein OS=Rattus norvegicus GN=Plp1 PE=1 SV=2	MYGVLPWNAFPGK
>	sp|P60203|MY Myelin proteolipid protein OS=Rattus norvegicus GN=Plp1 PE=1 SV=2	MYGVLPWNAFPGK
>	sp|P60203|MY Myelin proteolipid protein OS=Rattus norvegicus GN=Plp1 PE=1 SV=2	VCGSNLLSICK
>	sp|P60203|MY Myelin proteolipid protein OS=Rattus norvegicus GN=Plp1 PE=1 SV=2	PWNAFPGK
>	sp|P60203|MY Myelin proteolipid protein OS=Rattus norvegicus GN=Plp1 PE=1 SV=2	GLSATVTGGQK
>	sp|P60203|MY Myelin proteolipid protein OS=Rattus norvegicus GN=Plp1 PE=1 SV=2	GQHQAHSLER
>	sp|P60203|MY Myelin proteolipid protein OS=Rattus norvegicus GN=Plp1 PE=1 SV=2	MYGVLPWNAFPGK

<table>
<tr><td>></td><td>sp|P60203|MY</td><td>Myelin proteolipid protein OS=Rattus norvegicus GN=Plp1 PE=1 SV=2</td><td>GLSATVTGGQK</td></tr>
<tr><td>></td><td>sp|P60203|MY</td><td>Myelin proteolipid protein OS=Rattus norvegicus GN=Plp1 PE=1 SV=2</td><td>MYGVLPWNAFPGK</td></tr>
<tr><td>></td><td>sp|P60203|MY</td><td>Myelin proteolipid protein OS=Rattus norvegicus GN=Plp1 PE=1 SV=2</td><td>LIETYFSK</td></tr>
<tr><td>></td><td>sp|P61265|STX</td><td>Syntaxin-1B OS=Rattus norvegicus GN=Stx1b PE=1 SV=1</td><td>AIEQSIEQEEGLNR</td></tr>
<tr><td>></td><td>sp|P61265|STX</td><td>Syntaxin-1B OS=Rattus norvegicus GN=Stx1b PE=1 SV=1</td><td>IEYNVEHSVDYVER</td></tr>
<tr><td>></td><td>sp|P61265|STX</td><td>Syntaxin-1B OS=Rattus norvegicus GN=Stx1b PE=1 SV=1</td><td>KQHSAILAAPNPDEK</td></tr>
<tr><td>></td><td>sp|P61265|STX</td><td>Syntaxin-1B OS=Rattus norvegicus GN=Stx1b PE=1 SV=1</td><td>QHSAILAAPNPDEK</td></tr>
<tr><td>></td><td>sp|P61265|STX</td><td>Syntaxin-1B OS=Rattus norvegicus GN=Stx1b PE=1 SV=1</td><td>TKQELEDLTADIK</td></tr>
<tr><td>></td><td>sp|P61265|STX</td><td>Syntaxin-1B OS=Rattus norvegicus GN=Stx1b PE=1 SV=1</td><td>SAKDSDDEEEVVHVDRDHFMDEFFEQVEEIR</td></tr>
<tr><td>></td><td>sp|P61265|STX</td><td>Syntaxin-1B OS=Rattus norvegicus GN=Stx1b PE=1 SV=1</td><td>TTTNEELEDMLESGK</td></tr>
<tr><td>></td><td>sp|P61265|STX</td><td>Syntaxin-1B OS=Rattus norvegicus GN=Stx1b PE=1 SV=1</td><td>TTTNEELEDMLESGK</td></tr>
<tr><td>></td><td>sp|P61265|STX</td><td>Syntaxin-1B OS=Rattus norvegicus GN=Stx1b PE=1 SV=1</td><td>AIEQSIEQEEGLNR</td></tr>
<tr><td>></td><td>sp|P61265|STX</td><td>Syntaxin-1B OS=Rattus norvegicus GN=Stx1b PE=1 SV=1</td><td>LAIFTDDIK</td></tr>
<tr><td>></td><td>sp|P61265|STX</td><td>Syntaxin-1B OS=Rattus norvegicus GN=Stx1b PE=1 SV=1</td><td>AIEQSIEQEEGLNR</td></tr>
<tr><td>></td><td>sp|P61265|STX</td><td>Syntaxin-1B OS=Rattus norvegicus GN=Stx1b PE=1 SV=1</td><td>AIEQSIEQEEGLNR</td></tr>
<tr><td>></td><td>sp|P61265|STX</td><td>Syntaxin-1B OS=Rattus norvegicus GN=Stx1b PE=1 SV=1</td><td>AIEQSIEQEEGLNR</td></tr>
<tr><td>></td><td>sp|P61265|STX</td><td>Syntaxin-1B OS=Rattus norvegicus GN=Stx1b PE=1 SV=1</td><td>IEYNVEHSVDYVER</td></tr>
<tr><td>></td><td>sp|P61265|STX</td><td>Syntaxin-1B OS=Rattus norvegicus GN=Stx1b PE=1 SV=1</td><td>TKQELEDLTADIK</td></tr>
<tr><td>></td><td>sp|P61265|STX</td><td>Syntaxin-1B OS=Rattus norvegicus GN=Stx1b PE=1 SV=1</td><td>IEYNVEHSVDYVER</td></tr>
<tr><td>></td><td>sp|P61265|STX</td><td>Syntaxin-1B OS=Rattus norvegicus GN=Stx1b PE=1 SV=1</td><td>LAIFTDDIK</td></tr>
<tr><td>></td><td>sp|P61265|STX</td><td>Syntaxin-1B OS=Rattus norvegicus GN=Stx1b PE=1 SV=1</td><td>QHSAILAAPNPDEK</td></tr>
<tr><td>></td><td>sp|P61265|STX</td><td>Syntaxin-1B OS=Rattus norvegicus GN=Stx1b PE=1 SV=1</td><td>SAKDSDDEEEVVHVDRDHFMDEFFEQVEEIR</td></tr>
<tr><td>></td><td>sp|P12369|KAP</td><td>cAMP-dependent protein kinase type II-beta regulatory subunit OS=Rattus norvegicus GN=Prkar2b PE=1 SV=3</td><td>AATITATSPGALWGLDR</td></tr>
<tr><td>></td><td>sp|P12369|KAP</td><td>cAMP-dependent protein kinase type II-beta regulatory subunit OS=Rattus norvegicus GN=Prkar2b PE=1 SV=3</td><td>GSFGELALMYNTPR</td></tr>
<tr><td>></td><td>sp|P12369|KAP</td><td>cAMP-dependent protein kinase type II-beta regulatory subunit OS=Rattus norvegicus GN=Prkar2b PE=1 SV=3</td><td>HQPADLLEFALQHFTR</td></tr>
<tr><td>></td><td>sp|P12369|KAP</td><td>cAMP-dependent protein kinase type II-beta regulatory subunit OS=Rattus norvegicus GN=Prkar2b PE=1 SV=3</td><td>NLDPEQMSQVLDAMFEK</td></tr>
<tr><td>></td><td>sp|P12369|KAP</td><td>cAMP-dependent protein kinase type II-beta regulatory subunit OS=Rattus norvegicus GN=Prkar2b PE=1 SV=3</td><td>TWGDAGAAAGGGTPSK</td></tr>
<tr><td>></td><td>sp|P12369|KAP</td><td>cAMP-dependent protein kinase type II-beta regulatory subunit OS=Rattus norvegicus GN=Prkar2b PE=1 SV=3</td><td>EGEHVIDQGDDGDNFYVIDR</td></tr>
<tr><td>></td><td>sp|P12369|KAP</td><td>cAMP-dependent protein kinase type II-beta regulatory subunit OS=Rattus norvegicus GN=Prkar2b PE=1 SV=3</td><td>GQYFGELALVTNKPR</td></tr>
<tr><td>></td><td>sp|P12369|KAP</td><td>cAMP-dependent protein kinase type II-beta regulatory subunit OS=Rattus norvegicus GN=Prkar2b PE=1 SV=3</td><td>EGEHVIDQGDDGDNFYVIDR</td></tr>
<tr><td>></td><td>sp|P12369|KAP</td><td>cAMP-dependent protein kinase type II-beta regulatory subunit OS=Rattus norvegicus GN=Prkar2b PE=1 SV=3</td><td>HQPADLLEFALQHFTR</td></tr>
<tr><td>></td><td>sp|P12369|KAP</td><td>cAMP-dependent protein kinase type II-beta regulatory subunit OS=Rattus norvegicus GN=Prkar2b PE=1 SV=3</td><td>HQPADLLEFALQHFTR</td></tr>
<tr><td>></td><td>sp|P12369|KAP</td><td>cAMP-dependent protein kinase type II-beta regulatory subunit OS=Rattus norvegicus GN=Prkar2b PE=1 SV=3</td><td>HQPADLLEFALQHFTR</td></tr>
<tr><td>></td><td>sp|P12369|KAP</td><td>cAMP-dependent protein kinase type II-beta regulatory subunit OS=Rattus norvegicus GN=Prkar2b PE=1 SV=3</td><td>HQPADLLEFALQHFTR</td></tr>
<tr><td>></td><td>sp|P12369|KAP</td><td>cAMP-dependent protein kinase type II-beta regulatory subunit OS=Rattus norvegicus GN=Prkar2b PE=1 SV=3</td><td>NLDPEQMSQVLDAMFEK</td></tr>
<tr><td>></td><td>sp|P12369|KAP</td><td>cAMP-dependent protein kinase type II-beta regulatory subunit OS=Rattus norvegicus GN=Prkar2b PE=1 SV=3</td><td>GSFGELALMYNTPR</td></tr>
<tr><td>></td><td>sp|P31016|DLG</td><td>Disks large homolog 4 OS=Rattus norvegicus GN=Dlg4 PE=1 SV=1</td><td>ATKLEQEFTECFSAIVEGDSFEEIYHK</td></tr>
<tr><td>></td><td>sp|P31016|DLG</td><td>Disks large homolog 4 OS=Rattus norvegicus GN=Dlg4 PE=1 SV=1</td><td>HCILDVSANAVR</td></tr>
<tr><td>></td><td>sp|P31016|DLG</td><td>Disks large homolog 4 OS=Rattus norvegicus GN=Dlg4 PE=1 SV=1</td><td>LQAAHLHPIAIFIRPR</td></tr>
<tr><td>></td><td>sp|P31016|DLG</td><td>Disks large homolog 4 OS=Rattus norvegicus GN=Dlg4 PE=1 SV=1</td><td>NASHEQAAIALK</td></tr>
<tr><td>></td><td>sp|P31016|DLG</td><td>Disks large homolog 4 OS=Rattus norvegicus GN=Dlg4 PE=1 SV=1</td><td>ILAVNSVGLEDVMHEDAVAALK</td></tr>
<tr><td>></td><td>sp|P31016|DLG</td><td>Disks large homolog 4 OS=Rattus norvegicus GN=Dlg4 PE=1 SV=1</td><td>VIEDLSGPYIWVPAR</td></tr>
<tr><td>></td><td>sp|P31016|DLG</td><td>Disks large homolog 4 OS=Rattus norvegicus GN=Dlg4 PE=1 SV=1</td><td>ATKLEQEFTECFSAIVEGDSFEEIYHK</td></tr>
<tr><td>></td><td>sp|P31016|DLG</td><td>Disks large homolog 4 OS=Rattus norvegicus GN=Dlg4 PE=1 SV=1</td><td>ATKLEQEFTECFSAIVEGDSFEEIYHK</td></tr>
<tr><td>></td><td>sp|P31016|DLG</td><td>Disks large homolog 4 OS=Rattus norvegicus GN=Dlg4 PE=1 SV=1</td><td>HCILDVSANAVR</td></tr>
<tr><td>></td><td>sp|P31016|DLG</td><td>Disks large homolog 4 OS=Rattus norvegicus GN=Dlg4 PE=1 SV=1</td><td>DLLGEEDIPREPR</td></tr>
<tr><td>></td><td>sp|P31016|DLG</td><td>Disks large homolog 4 OS=Rattus norvegicus GN=Dlg4 PE=1 SV=1</td><td>HCILDVSANAVR</td></tr>
<tr><td>></td><td>sp|Q63633|S12</td><td>Solute carrier family 12 member 5 OS=Rattus norvegicus GN=Slc12a5 PE=1 SV=2</td><td>GLTIVGSVLEGTFLDNHPQAQR</td></tr>
<tr><td>></td><td>sp|Q63633|S12</td><td>Solute carrier family 12 member 5 OS=Rattus norvegicus GN=Slc12a5 PE=1 SV=2</td><td>KDLTTFLYHLR</td></tr>
<tr><td>></td><td>sp|Q63633|S12</td><td>Solute carrier family 12 member 5 OS=Rattus norvegicus GN=Slc12a5 PE=1 SV=2</td><td>LVLLNMPGPPR</td></tr>
<tr><td>></td><td>sp|Q63633|S12</td><td>Solute carrier family 12 member 5 OS=Rattus norvegicus GN=Slc12a5 PE=1 SV=2</td><td>DGIVPFLQVFGHGK</td></tr>
<tr><td>></td><td>sp|Q63633|S12</td><td>Solute carrier family 12 member 5 OS=Rattus norvegicus GN=Slc12a5 PE=1 SV=2</td><td>DLTTFLYHLR</td></tr>
<tr><td>></td><td>sp|Q63633|S12</td><td>Solute carrier family 12 member 5 OS=Rattus norvegicus GN=Slc12a5 PE=1 SV=2</td><td>GLTIVGSVLEGTFLDNHPQAQR</td></tr>
<tr><td>></td><td>sp|Q63633|S12</td><td>Solute carrier family 12 member 5 OS=Rattus norvegicus GN=Slc12a5 PE=1 SV=2</td><td>NKGPSPVSSEGIKDFFSMKPEWENLNQSNVR</td></tr>
<tr><td>></td><td>sp|Q63633|S12</td><td>Solute carrier family 12 member 5 OS=Rattus norvegicus GN=Slc12a5 PE=1 SV=2</td><td>LNEVIVNK</td></tr>
<tr><td>></td><td>sp|Q63633|S12</td><td>Solute carrier family 12 member 5 OS=Rattus norvegicus GN=Slc12a5 PE=1 SV=2</td><td>NKGPSPVSSEGIKDFFSMKPEWENLNQSNVR</td></tr>
<tr><td>></td><td>sp|Q63633|S12</td><td>Solute carrier family 12 member 5 OS=Rattus norvegicus GN=Slc12a5 PE=1 SV=2</td><td>LNEVIVNK</td></tr>
<tr><td>></td><td>sp|Q63633|S12</td><td>Solute carrier family 12 member 5 OS=Rattus norvegicus GN=Slc12a5 PE=1 SV=2</td><td>KKPVQAPR</td></tr>
<tr><td>></td><td>sp|P68511|143</td><td>14-3-3 protein eta OS=Rattus norvegicus GN=Ywhah PE=1 SV=2</td><td>AVTELNEPLSNEDR</td></tr>
<tr><td>></td><td>sp|P68511|143</td><td>14-3-3 protein eta OS=Rattus norvegicus GN=Ywhah PE=1 SV=2</td><td>EKIEKELETVCNDVLALLDK</td></tr>
<tr><td>></td><td>sp|P68511|143</td><td>14-3-3 protein eta OS=Rattus norvegicus GN=Ywhah PE=1 SV=2</td><td>EKIEKELETVCNDVLALLDKFLIK</td></tr>
<tr><td>></td><td>sp|P68511|143</td><td>14-3-3 protein eta OS=Rattus norvegicus GN=Ywhah PE=1 SV=2</td><td>IEKELETVCNDVLALLDK</td></tr>
<tr><td>></td><td>sp|P68511|143</td><td>14-3-3 protein eta OS=Rattus norvegicus GN=Ywhah PE=1 SV=2</td><td>IEKELETVCNDVLALLDKFLIK</td></tr>
<tr><td>></td><td>sp|P68511|143</td><td>14-3-3 protein eta OS=Rattus norvegicus GN=Ywhah PE=1 SV=2</td><td>AVTELNEPLSNEDR</td></tr>
<tr><td>></td><td>sp|P68511|143</td><td>14-3-3 protein eta OS=Rattus norvegicus GN=Ywhah PE=1 SV=2</td><td>AVTELNEPLSNEDR</td></tr>
<tr><td>></td><td>sp|P68511|143</td><td>14-3-3 protein eta OS=Rattus norvegicus GN=Ywhah PE=1 SV=2</td><td>AVTELNEPLSNEDR</td></tr>
<tr><td>></td><td>sp|P68511|143</td><td>14-3-3 protein eta OS=Rattus norvegicus GN=Ywhah PE=1 SV=2</td><td>DSTLIMQLLR</td></tr>
<tr><td>></td><td>sp|P68511|143</td><td>14-3-3 protein eta OS=Rattus norvegicus GN=Ywhah PE=1 SV=2</td><td>EKIEKELETVCNDVLALLDKFLIK</td></tr>
<tr><td>></td><td>sp|P68511|143</td><td>14-3-3 protein eta OS=Rattus norvegicus GN=Ywhah PE=1 SV=2</td><td>IEKELETVCNDVLALLDK</td></tr>
<tr><td>></td><td>sp|P68511|143</td><td>14-3-3 protein eta OS=Rattus norvegicus GN=Ywhah PE=1 SV=2</td><td>NLLSVAYK</td></tr>
<tr><td>></td><td>sp|P68511|143</td><td>14-3-3 protein eta OS=Rattus norvegicus GN=Ywhah PE=1 SV=2</td><td>NLLSVAYK</td></tr>
<tr><td>></td><td>sp|P68511|143</td><td>14-3-3 protein eta OS=Rattus norvegicus GN=Ywhah PE=1 SV=2</td><td>NLLSVAYK</td></tr>
<tr><td>></td><td>sp|P68511|143</td><td>14-3-3 protein eta OS=Rattus norvegicus GN=Ywhah PE=1 SV=2</td><td>NLLSVAYK</td></tr>
<tr><td>></td><td>sp|P68511|143</td><td>14-3-3 protein eta OS=Rattus norvegicus GN=Ywhah PE=1 SV=2</td><td>NLLSVAYK</td></tr>
</table>

>	sp|P68511|143 14-3-3 protein eta OS=Rattus norvegicus GN=Ywhah PE=1 SV=2	VISSIEQK
>	sp|P68511|143 14-3-3 protein eta OS=Rattus norvegicus GN=Ywhah PE=1 SV=2	NLLSVAYK
>	sp|P68511|143 14-3-3 protein eta OS=Rattus norvegicus GN=Ywhah PE=1 SV=2	VISSIEQK
>	sp|P68511|143 14-3-3 protein eta OS=Rattus norvegicus GN=Ywhah PE=1 SV=2	NLLSVAYK
>	sp|P68511|143 14-3-3 protein eta OS=Rattus norvegicus GN=Ywhah PE=1 SV=2	NLLSVAYK
>	sp|P68511|143 14-3-3 protein eta OS=Rattus norvegicus GN=Ywhah PE=1 SV=2	NLLSVAYK
>	sp|P68511|143 14-3-3 protein eta OS=Rattus norvegicus GN=Ywhah PE=1 SV=2	VISSIEQK
>	sp|P68511|143 14-3-3 protein eta OS=Rattus norvegicus GN=Ywhah PE=1 SV=2	IEKELETVCNDVLALLDK
>	sp|P50398|GDI Rab GDP dissociation inhibitor alpha OS=Rattus norvegicus GN=Gdi1 PE=1 SV=1	IYKVPSTETEALASNLMGMFEK
>	sp|P50398|GDI Rab GDP dissociation inhibitor alpha OS=Rattus norvegicus GN=Gdi1 PE=1 SV=1	KFDLGQDVIDFTGHALALYR
>	sp|P50398|GDI Rab GDP dissociation inhibitor alpha OS=Rattus norvegicus GN=Gdi1 PE=1 SV=1	TDDYLDQPCLETINR
>	sp|P50398|GDI Rab GDP dissociation inhibitor alpha OS=Rattus norvegicus GN=Gdi1 PE=1 SV=1	TFEGVDPQTTSMR
>	sp|P50398|GDI Rab GDP dissociation inhibitor alpha OS=Rattus norvegicus GN=Gdi1 PE=1 SV=1	YIAIASTTVETAEPEKEVEPALELLEPIDQK
>	sp|P50398|GDI Rab GDP dissociation inhibitor alpha OS=Rattus norvegicus GN=Gdi1 PE=1 SV=1	FQLLEGPPESMGR
>	sp|P50398|GDI Rab GDP dissociation inhibitor alpha OS=Rattus norvegicus GN=Gdi1 PE=1 SV=1	FQLLEGPPESMGR
>	sp|P50398|GDI Rab GDP dissociation inhibitor alpha OS=Rattus norvegicus GN=Gdi1 PE=1 SV=1	IYKVPSTETEALASNLMGMFEK
>	sp|P50398|GDI Rab GDP dissociation inhibitor alpha OS=Rattus norvegicus GN=Gdi1 PE=1 SV=1	IYKVPSTETEALASNLMGMFEK
>	sp|P50398|GDI Rab GDP dissociation inhibitor alpha OS=Rattus norvegicus GN=Gdi1 PE=1 SV=1	IYKVPSTETEALASNLMGMFEK
>	sp|P50398|GDI Rab GDP dissociation inhibitor alpha OS=Rattus norvegicus GN=Gdi1 PE=1 SV=1	IYKVPSTETEALASNLMGMFEK
>	sp|P50398|GDI Rab GDP dissociation inhibitor alpha OS=Rattus norvegicus GN=Gdi1 PE=1 SV=1	IYKVPSTETEALASNLMGMFEK
>	sp|P50398|GDI Rab GDP dissociation inhibitor alpha OS=Rattus norvegicus GN=Gdi1 PE=1 SV=1	KFDLGQDVIDFTGHALALYR
>	sp|P50398|GDI Rab GDP dissociation inhibitor alpha OS=Rattus norvegicus GN=Gdi1 PE=1 SV=1	KFDLGQDVIDFTGHALALYR
>	sp|P50398|GDI Rab GDP dissociation inhibitor alpha OS=Rattus norvegicus GN=Gdi1 PE=1 SV=1	KFDLGQDVIDFTGHALALYR
>	sp|P50398|GDI Rab GDP dissociation inhibitor alpha OS=Rattus norvegicus GN=Gdi1 PE=1 SV=1	KFDLGQDVIDFTGHALALYR
>	sp|P50398|GDI Rab GDP dissociation inhibitor alpha OS=Rattus norvegicus GN=Gdi1 PE=1 SV=1	KFDLGQDVIDFTGHALALYR
>	sp|P50398|GDI Rab GDP dissociation inhibitor alpha OS=Rattus norvegicus GN=Gdi1 PE=1 SV=1	KFDLGQDVIDFTGHALALYR
>	sp|P50398|GDI Rab GDP dissociation inhibitor alpha OS=Rattus norvegicus GN=Gdi1 PE=1 SV=1	KFDLGQDVIDFTGHALALYR
>	sp|P50398|GDI Rab GDP dissociation inhibitor alpha OS=Rattus norvegicus GN=Gdi1 PE=1 SV=1	KFDLGQDVIDFTGHALALYR
>	sp|P50398|GDI Rab GDP dissociation inhibitor alpha OS=Rattus norvegicus GN=Gdi1 PE=1 SV=1	KFDLGQDVIDFTGHALALYR
>	sp|P50398|GDI Rab GDP dissociation inhibitor alpha OS=Rattus norvegicus GN=Gdi1 PE=1 SV=1	KFDLGQDVIDFTGHALALYR
>	sp|P50398|GDI Rab GDP dissociation inhibitor alpha OS=Rattus norvegicus GN=Gdi1 PE=1 SV=1	TDDYLDQPCLETINR
>	sp|P50398|GDI Rab GDP dissociation inhibitor alpha OS=Rattus norvegicus GN=Gdi1 PE=1 SV=1	TDDYLDQPCLETINR
>	sp|P50398|GDI Rab GDP dissociation inhibitor alpha OS=Rattus norvegicus GN=Gdi1 PE=1 SV=1	TDDYLDQPCLETINR
>	sp|P50398|GDI Rab GDP dissociation inhibitor alpha OS=Rattus norvegicus GN=Gdi1 PE=1 SV=1	TDDYLDQPCLETINR
>	sp|P50398|GDI Rab GDP dissociation inhibitor alpha OS=Rattus norvegicus GN=Gdi1 PE=1 SV=1	YIAIASTTVETAEPEKEVEPALELLEPIDQK
>	sp|P50398|GDI Rab GDP dissociation inhibitor alpha OS=Rattus norvegicus GN=Gdi1 PE=1 SV=1	YIAIASTTVETAEPEKEVEPALELLEPIDQK
>	sp|P50398|GDI Rab GDP dissociation inhibitor alpha OS=Rattus norvegicus GN=Gdi1 PE=1 SV=1	YIAIASTTVETAEPEKEVEPALELLEPIDQK
>	sp|P50398|GDI Rab GDP dissociation inhibitor alpha OS=Rattus norvegicus GN=Gdi1 PE=1 SV=1	YIAIASTTVETAEPEKEVEPALELLEPIDQK
>	sp|P50398|GDI Rab GDP dissociation inhibitor alpha OS=Rattus norvegicus GN=Gdi1 PE=1 SV=1	YIAIASTTVETAEPEKEVEPALELLEPIDQK
>	sp|P50398|GDI Rab GDP dissociation inhibitor alpha OS=Rattus norvegicus GN=Gdi1 PE=1 SV=1	KQNDVFGEADQ
>	sp|P50398|GDI Rab GDP dissociation inhibitor alpha OS=Rattus norvegicus GN=Gdi1 PE=1 SV=1	ICILSHPIK
>	sp|P50398|GDI Rab GDP dissociation inhibitor alpha OS=Rattus norvegicus GN=Gdi1 PE=1 SV=1	IICILSHPIKNTNDANSCQIIIPQNQVNR
>	sp|P50398|GDI Rab GDP dissociation inhibitor alpha OS=Rattus norvegicus GN=Gdi1 PE=1 SV=1	KFDLGQDVIDFTGHALALYR
>	sp|P50398|GDI Rab GDP dissociation inhibitor alpha OS=Rattus norvegicus GN=Gdi1 PE=1 SV=1	MLLYTEVTR
>	sp|P50398|GDI Rab GDP dissociation inhibitor alpha OS=Rattus norvegicus GN=Gdi1 PE=1 SV=1	IYKVPSTETEALASNLMGMFEK
>	sp|P67779|PHB Prohibitin OS=Rattus norvegicus GN=Phb PE=1 SV=1	AAELIANSLATAGDGLIELR
>	sp|P67779|PHB Prohibitin OS=Rattus norvegicus GN=Phb PE=1 SV=1	AATFGLILDDVSLTHLTFGK
>	sp|P67779|PHB Prohibitin OS=Rattus norvegicus GN=Phb PE=1 SV=1	GVQDIVVGEGTHFLIPWVQKPIIFDCR
>	sp|P67779|PHB Prohibitin OS=Rattus norvegicus GN=Phb PE=1 SV=1	KLEAAEDIAYQLSR
>	sp|P67779|PHB Prohibitin OS=Rattus norvegicus GN=Phb PE=1 SV=1	VFESIGKFGLALAVAGGVVNSALYNVDAGHR
>	sp|P67779|PHB Prohibitin OS=Rattus norvegicus GN=Phb PE=1 SV=1	FGLALAVAGGVVNSALYNVDAGHR
>	sp|P67779|PHB Prohibitin OS=Rattus norvegicus GN=Phb PE=1 SV=1	GVQDIVVGEGTHFLIPWVQKPIIFDCR
>	sp|P67779|PHB Prohibitin OS=Rattus norvegicus GN=Phb PE=1 SV=1	GVQDIVVGEGTHFLIPWVQKPIIFDCR
>	sp|P67779|PHB Prohibitin OS=Rattus norvegicus GN=Phb PE=1 SV=1	GVQDIVVGEGTHFLIPWVQKPIIFDCR
>	sp|P67779|PHB Prohibitin OS=Rattus norvegicus GN=Phb PE=1 SV=1	KLEAAEDIAYQLSR
>	sp|P67779|PHB Prohibitin OS=Rattus norvegicus GN=Phb PE=1 SV=1	VFESIGKFGLALAVAGGVVNSALYNVDAGHR
>	sp|P67779|PHB Prohibitin OS=Rattus norvegicus GN=Phb PE=1 SV=1	VLPSITTEILK
>	sp|P67779|PHB Prohibitin OS=Rattus norvegicus GN=Phb PE=1 SV=1	VLPSITTEILK
>	sp|P49432|ODI Pyruvate dehydrogenase E1 component subunit beta, mitochondrial OS=Rattus norvegicus GN=Pdhb PE=1 SV=2	EGIECEVINLR
>	sp|P49432|ODI Pyruvate dehydrogenase E1 component subunit beta, mitochondrial OS=Rattus norvegicus GN=Pdhb PE=1 SV=2	IMEGPAFNFLDAPAVR
>	sp|P49432|ODI Pyruvate dehydrogenase E1 component subunit beta, mitochondrial OS=Rattus norvegicus GN=Pdhb PE=1 SV=2	SAIRDDNPVVMLENELMYGVAFELPTEAQSK
>	sp|P49432|ODI Pyruvate dehydrogenase E1 component subunit beta, mitochondrial OS=Rattus norvegicus GN=Pdhb PE=1 SV=2	VFLLGEEVAQYDGAYK
>	sp|P49432|ODI Pyruvate dehydrogenase E1 component subunit beta, mitochondrial OS=Rattus norvegicus GN=Pdhb PE=1 SV=2	TIRPMDIEAIEASVMK
>	sp|P49432|ODI Pyruvate dehydrogenase E1 component subunit beta, mitochondrial OS=Rattus norvegicus GN=Pdhb PE=1 SV=2	IMEGPAFNFLDAPAVR
>	sp|P49432|ODI Pyruvate dehydrogenase E1 component subunit beta, mitochondrial OS=Rattus norvegicus GN=Pdhb PE=1 SV=2	IMEGPAFNFLDAPAVR
>	sp|P49432|ODI Pyruvate dehydrogenase E1 component subunit beta, mitochondrial OS=Rattus norvegicus GN=Pdhb PE=1 SV=2	IMEGPAFNFLDAPAVR
>	sp|P49432|ODI Pyruvate dehydrogenase E1 component subunit beta, mitochondrial OS=Rattus norvegicus GN=Pdhb PE=1 SV=2	IMEGPAFNFLDAPAVR
>	sp|P49432|ODI Pyruvate dehydrogenase E1 component subunit beta, mitochondrial OS=Rattus norvegicus GN=Pdhb PE=1 SV=2	SAIRDDNPVVMLENELMYGVAFELPTEAQSK
>	sp|P49432|ODI Pyruvate dehydrogenase E1 component subunit beta, mitochondrial OS=Rattus norvegicus GN=Pdhb PE=1 SV=2	SAIRDDNPVVMLENELMYGVAFELPTEAQSK
>	sp|P49432|ODI Pyruvate dehydrogenase E1 component subunit beta, mitochondrial OS=Rattus norvegicus GN=Pdhb PE=1 SV=2	SAIRDDNPVVMLENELMYGVAFELPTEAQSK
>	sp|P49432|ODI Pyruvate dehydrogenase E1 component subunit beta, mitochondrial OS=Rattus norvegicus GN=Pdhb PE=1 SV=2	VFLLGEEVAQYDGAYK
>	sp|P49432|ODI Pyruvate dehydrogenase E1 component subunit beta, mitochondrial OS=Rattus norvegicus GN=Pdhb PE=1 SV=2	VFLLGEEVAQYDGAYK
>	sp|P49432|ODI Pyruvate dehydrogenase E1 component subunit beta, mitochondrial OS=Rattus norvegicus GN=Pdhb PE=1 SV=2	VFLLGEEVAQYDGAYK
>	sp|P49432|ODI Pyruvate dehydrogenase E1 component subunit beta, mitochondrial OS=Rattus norvegicus GN=Pdhb PE=1 SV=2	VFLLGEEVAQYDGAYK

>	sp|P49432|ODI	Pyruvate dehydrogenase E1 component subunit beta, mitochondrial OS=Rattus norvegicus GN=Pdhb PE=1 SV=2	DFLIPIGK
>	sp|P49432|ODI	Pyruvate dehydrogenase E1 component subunit beta, mitochondrial OS=Rattus norvegicus GN=Pdhb PE=1 SV=2	DIIFAIK
>	sp|P49432|ODI	Pyruvate dehydrogenase E1 component subunit beta, mitochondrial OS=Rattus norvegicus GN=Pdhb PE=1 SV=2	DIIFAIK
>	sp|P49432|ODI	Pyruvate dehydrogenase E1 component subunit beta, mitochondrial OS=Rattus norvegicus GN=Pdhb PE=1 SV=2	DIIFAIK
>	sp|P49432|ODI	Pyruvate dehydrogenase E1 component subunit beta, mitochondrial OS=Rattus norvegicus GN=Pdhb PE=1 SV=2	TIRPMDIEAIEASVMK
>	sp|O88989|MD	Malate dehydrogenase, cytoplasmic OS=Rattus norvegicus GN=Mdh1 PE=1 SV=3	ELTEEKETAFEFLSSA
>	sp|O88989|MD	Malate dehydrogenase, cytoplasmic OS=Rattus norvegicus GN=Mdh1 PE=1 SV=3	EVGVYEALKDDSWLK
>	sp|O88989|MD	Malate dehydrogenase, cytoplasmic OS=Rattus norvegicus GN=Mdh1 PE=1 SV=3	GEFITTVQQR
>	sp|O88989|MD	Malate dehydrogenase, cytoplasmic OS=Rattus norvegicus GN=Mdh1 PE=1 SV=3	LGVTADDVK
>	sp|O88989|MD	Malate dehydrogenase, cytoplasmic OS=Rattus norvegicus GN=Mdh1 PE=1 SV=3	VIVVGNPANTNCLTASK
>	sp|O88989|MD	Malate dehydrogenase, cytoplasmic OS=Rattus norvegicus GN=Mdh1 PE=1 SV=3	ELTEEKETAFEFLSSA
>	sp|O88989|MD	Malate dehydrogenase, cytoplasmic OS=Rattus norvegicus GN=Mdh1 PE=1 SV=3	ELTEEKETAFEFLSSA
>	sp|O88989|MD	Malate dehydrogenase, cytoplasmic OS=Rattus norvegicus GN=Mdh1 PE=1 SV=3	EVGVYEALKDDSWLK
>	sp|O88989|MD	Malate dehydrogenase, cytoplasmic OS=Rattus norvegicus GN=Mdh1 PE=1 SV=3	EVGVYEALKDDSWLK
>	sp|O88989|MD	Malate dehydrogenase, cytoplasmic OS=Rattus norvegicus GN=Mdh1 PE=1 SV=3	EVGVYEALKDDSWLK
>	sp|O88989|MD	Malate dehydrogenase, cytoplasmic OS=Rattus norvegicus GN=Mdh1 PE=1 SV=3	EVGVYEALKDDSWLK
>	sp|O88989|MD	Malate dehydrogenase, cytoplasmic OS=Rattus norvegicus GN=Mdh1 PE=1 SV=3	GEFITTVQQR
>	sp|O88989|MD	Malate dehydrogenase, cytoplasmic OS=Rattus norvegicus GN=Mdh1 PE=1 SV=3	GEFITTVQQR
>	sp|O88989|MD	Malate dehydrogenase, cytoplasmic OS=Rattus norvegicus GN=Mdh1 PE=1 SV=3	LGVTADDVK
>	sp|O88989|MD	Malate dehydrogenase, cytoplasmic OS=Rattus norvegicus GN=Mdh1 PE=1 SV=3	VIVVGNPANTNCLTASK
>	sp|O88989|MD	Malate dehydrogenase, cytoplasmic OS=Rattus norvegicus GN=Mdh1 PE=1 SV=3	VIVVGNPANTNCLTASK
>	sp|O88989|MD	Malate dehydrogenase, cytoplasmic OS=Rattus norvegicus GN=Mdh1 PE=1 SV=3	VIVVGNPANTNCLTASK
>	sp|O88989|MD	Malate dehydrogenase, cytoplasmic OS=Rattus norvegicus GN=Mdh1 PE=1 SV=3	DIWFGTPEGEFVSMGVISDGNSYGVPDDLLYSFPVVIK
>	sp|O88989|MD	Malate dehydrogenase, cytoplasmic OS=Rattus norvegicus GN=Mdh1 PE=1 SV=3	GEFITTVQQR
>	sp|O88989|MD	Malate dehydrogenase, cytoplasmic OS=Rattus norvegicus GN=Mdh1 PE=1 SV=3	VIVVGNPANTNCLTASK
>	sp|O88989|MD	Malate dehydrogenase, cytoplasmic OS=Rattus norvegicus GN=Mdh1 PE=1 SV=3	VIVVGNPANTNCLTASK
>	sp|O88989|MD	Malate dehydrogenase, cytoplasmic OS=Rattus norvegicus GN=Mdh1 PE=1 SV=3	GEFITTVQQR
>	sp|O88989|MD	Malate dehydrogenase, cytoplasmic OS=Rattus norvegicus GN=Mdh1 PE=1 SV=3	GEFITTVQQR
>	sp|O88989|MD	Malate dehydrogenase, cytoplasmic OS=Rattus norvegicus GN=Mdh1 PE=1 SV=3	VIVVGNPANTNCLTASK
>	sp|O88989|MD	Malate dehydrogenase, cytoplasmic OS=Rattus norvegicus GN=Mdh1 PE=1 SV=3	EVGVYEALKDDSWLK
>	sp|O88989|MD	Malate dehydrogenase, cytoplasmic OS=Rattus norvegicus GN=Mdh1 PE=1 SV=3	EVGVYEALKDDSWLK
>	sp|O88989|MD	Malate dehydrogenase, cytoplasmic OS=Rattus norvegicus GN=Mdh1 PE=1 SV=3	FVEGLPINDFSR
>	sp|O88989|MD	Malate dehydrogenase, cytoplasmic OS=Rattus norvegicus GN=Mdh1 PE=1 SV=3	FVEGLPINDFSR
>	sp|O88989|MD	Malate dehydrogenase, cytoplasmic OS=Rattus norvegicus GN=Mdh1 PE=1 SV=3	GEFITTVQQR
>	sp|O88989|MD	Malate dehydrogenase, cytoplasmic OS=Rattus norvegicus GN=Mdh1 PE=1 SV=3	LGVTADDVK
>	sp|O88989|MD	Malate dehydrogenase, cytoplasmic OS=Rattus norvegicus GN=Mdh1 PE=1 SV=3	VIVVGNPANTNCLTASK
>	sp|O88989|MD	Malate dehydrogenase, cytoplasmic OS=Rattus norvegicus GN=Mdh1 PE=1 SV=3	GEFITTVQQR
>	sp|Q8VHF5|CIS	Citrate synthase, mitochondrial OS=Rattus norvegicus GN=Cs PE=1 SV=1	AALPSHVVTMLDNFPTNLHPMSQLSAAITALNSESNFAR
>	sp|Q8VHF5|CIS	Citrate synthase, mitochondrial OS=Rattus norvegicus GN=Cs PE=1 SV=1	GLVYETSVLDPDEGIR
>	sp|Q8VHF5|CIS	Citrate synthase, mitochondrial OS=Rattus norvegicus GN=Cs PE=1 SV=1	IVPNILLEQGK
>	sp|Q8VHF5|CIS	Citrate synthase, mitochondrial OS=Rattus norvegicus GN=Cs PE=1 SV=1	RAALPSHVVTMLDNFPTNLHPMSQLSAAITALNSESNFAR
>	sp|Q8VHF5|CIS	Citrate synthase, mitochondrial OS=Rattus norvegicus GN=Cs PE=1 SV=1	AALPSHVVTMLDNFPTNLHPMSQLSAAITALNSESNFARAYA
>	sp|Q8VHF5|CIS	Citrate synthase, mitochondrial OS=Rattus norvegicus GN=Cs PE=1 SV=1	TKYWELIYEDCMDLIAK
>	sp|Q8VHF5|CIS	Citrate synthase, mitochondrial OS=Rattus norvegicus GN=Cs PE=1 SV=1	AALPSHVVTMLDNFPTNLHPMSQLSAAITALNSESNFAR
>	sp|Q8VHF5|CIS	Citrate synthase, mitochondrial OS=Rattus norvegicus GN=Cs PE=1 SV=1	AALPSHVVTMLDNFPTNLHPMSQLSAAITALNSESNFAR
>	sp|Q8VHF5|CIS	Citrate synthase, mitochondrial OS=Rattus norvegicus GN=Cs PE=1 SV=1	AALPSHVVTMLDNFPTNLHPMSQLSAAITALNSESNFAR
>	sp|Q8VHF5|CIS	Citrate synthase, mitochondrial OS=Rattus norvegicus GN=Cs PE=1 SV=1	AALPSHVVTMLDNFPTNLHPMSQLSAAITALNSESNFAR
>	sp|Q8VHF5|CIS	Citrate synthase, mitochondrial OS=Rattus norvegicus GN=Cs PE=1 SV=1	GLVYETSVLDPDEGIR
>	sp|Q8VHF5|CIS	Citrate synthase, mitochondrial OS=Rattus norvegicus GN=Cs PE=1 SV=1	GLVYETSVLDPDEGIR
>	sp|Q8VHF5|CIS	Citrate synthase, mitochondrial OS=Rattus norvegicus GN=Cs PE=1 SV=1	GLVYETSVLDPDEGIR
>	sp|Q8VHF5|CIS	Citrate synthase, mitochondrial OS=Rattus norvegicus GN=Cs PE=1 SV=1	IVPNILLEQGK
>	sp|Q8VHF5|CIS	Citrate synthase, mitochondrial OS=Rattus norvegicus GN=Cs PE=1 SV=1	TKYWELIYEDCMDLIAK
>	sp|Q8VHF5|CIS	Citrate synthase, mitochondrial OS=Rattus norvegicus GN=Cs PE=1 SV=1	ALGFPLERPK
>	sp|Q8VHF5|CIS	Citrate synthase, mitochondrial OS=Rattus norvegicus GN=Cs PE=1 SV=1	ALGFPLERPK
>	sp|Q8VHF5|CIS	Citrate synthase, mitochondrial OS=Rattus norvegicus GN=Cs PE=1 SV=1	AALPSHVVTMLDNFPTNLHPMSQLSAAITALNSESNFAR
>	sp|Q8VHF5|CIS	Citrate synthase, mitochondrial OS=Rattus norvegicus GN=Cs PE=1 SV=1	ALGFPLERPK
>	sp|Q8VHF5|CIS	Citrate synthase, mitochondrial OS=Rattus norvegicus GN=Cs PE=1 SV=1	GLVYETSVLDPDEGIR
>	sp|Q8VHF5|CIS	Citrate synthase, mitochondrial OS=Rattus norvegicus GN=Cs PE=1 SV=1	TVVGQITVDMMYGGMR
>	sp|Q8VHF5|CIS	Citrate synthase, mitochondrial OS=Rattus norvegicus GN=Cs PE=1 SV=1	HLPKDPMFK
>	sp|Q8VHF5|CIS	Citrate synthase, mitochondrial OS=Rattus norvegicus GN=Cs PE=1 SV=1	EGSSIGAIDSK
>	sp|Q8VHF5|CIS	Citrate synthase, mitochondrial OS=Rattus norvegicus GN=Cs PE=1 SV=1	LVAQLYK
>	sp|Q8VHF5|CIS	Citrate synthase, mitochondrial OS=Rattus norvegicus GN=Cs PE=1 SV=1	ALGFPLERPK
>	sp|Q8VHF5|CIS	Citrate synthase, mitochondrial OS=Rattus norvegicus GN=Cs PE=1 SV=1	RAALPSHVVTMLDNFPTNLHPMSQLSAAITALNSESNFAR
>	sp|Q8VHF5|CIS	Citrate synthase, mitochondrial OS=Rattus norvegicus GN=Cs PE=1 SV=1	IVPNILLEQGK
>	sp|Q8VHF5|CIS	Citrate synthase, mitochondrial OS=Rattus norvegicus GN=Cs PE=1 SV=1	RAALPSHVVTMLDNFPTNLHPMSQLSAAITALNSESNFAR
>	sp|Q8VHF5|CIS	Citrate synthase, mitochondrial OS=Rattus norvegicus GN=Cs PE=1 SV=1	RAALPSHVVTMLDNFPTNLHPMSQLSAAITALNSESNFAR
>	sp|Q8VHF5|CIS	Citrate synthase, mitochondrial OS=Rattus norvegicus GN=Cs PE=1 SV=1	HLPKDPMFK
>	sp|P25809|KCF	Creatine kinase U-type, mitochondrial OS=Rattus norvegicus GN=Ckmt1 PE=1 SV=1	LSEMTEAEQQQLIDDHFLFDKPVSPLLTAAGMAR
>	sp|P25809|KCF	Creatine kinase U-type, mitochondrial OS=Rattus norvegicus GN=Ckmt1 PE=1 SV=1	TVGMVAGDEETYEVFAELFDPVIQER
>	sp|P25809|KCF	Creatine kinase U-type, mitochondrial OS=Rattus norvegicus GN=Ckmt1 PE=1 SV=1	VVVDALSGLK
>	sp|P25809|KCF	Creatine kinase U-type, mitochondrial OS=Rattus norvegicus GN=Ckmt1 PE=1 SV=1	LCDKTTPTGWTLDQCIQTGVDNPGHPFIK
>	sp|P25809|KCF	Creatine kinase U-type, mitochondrial OS=Rattus norvegicus GN=Ckmt1 PE=1 SV=1	LGYILTCPSNLGTGLR
>	sp|P25809|KCF	Creatine kinase U-type, mitochondrial OS=Rattus norvegicus GN=Ckmt1 PE=1 SV=1	LGYILTCPSNLGTGLR
>	sp|P25809|KCF	Creatine kinase U-type, mitochondrial OS=Rattus norvegicus GN=Ckmt1 PE=1 SV=1	LGYILTCPSNLGTGLR

>	sp|P25809|KCF Creatine kinase U-type, mitochondrial OS=Rattus norvegicus GN=Ckmt1 PE=1 SV=1	LSEMTEAEQQQLIDDHFLFDKPVSPLLTAAGMAR
>	sp|P25809|KCF Creatine kinase U-type, mitochondrial OS=Rattus norvegicus GN=Ckmt1 PE=1 SV=1	LSEMTEAEQQQLIDDHFLFDKPVSPLLTAAGMAR
>	sp|P25809|KCF Creatine kinase U-type, mitochondrial OS=Rattus norvegicus GN=Ckmt1 PE=1 SV=1	LSEMTEAEQQQLIDDHFLFDKPVSPLLTAAGMAR
>	sp|P25809|KCF Creatine kinase U-type, mitochondrial OS=Rattus norvegicus GN=Ckmt1 PE=1 SV=1	LSEMTEAEQQQLIDDHFLFDKPVSPLLTAAGMAR
>	sp|P25809|KCF Creatine kinase U-type, mitochondrial OS=Rattus norvegicus GN=Ckmt1 PE=1 SV=1	TVGMVAGDEETYEVFAELFDPVIQER
>	sp|P25809|KCF Creatine kinase U-type, mitochondrial OS=Rattus norvegicus GN=Ckmt1 PE=1 SV=1	TVGMVAGDEETYEVFAELFDPVIQER
>	sp|P25809|KCF Creatine kinase U-type, mitochondrial OS=Rattus norvegicus GN=Ckmt1 PE=1 SV=1	VVVDALSGLK
>	sp|P25809|KCF Creatine kinase U-type, mitochondrial OS=Rattus norvegicus GN=Ckmt1 PE=1 SV=1	VVVDALSGLK
>	sp|P25809|KCF Creatine kinase U-type, mitochondrial OS=Rattus norvegicus GN=Ckmt1 PE=1 SV=1	VVVDALSGLK
>	sp|P25809|KCF Creatine kinase U-type, mitochondrial OS=Rattus norvegicus GN=Ckmt1 PE=1 SV=1	VVVDALSGLK
>	sp|P25809|KCF Creatine kinase U-type, mitochondrial OS=Rattus norvegicus GN=Ckmt1 PE=1 SV=1	VVVDALSGLK
>	sp|P25809|KCF Creatine kinase U-type, mitochondrial OS=Rattus norvegicus GN=Ckmt1 PE=1 SV=1	SFLIWVNEEDHTR
>	sp|P25809|KCF Creatine kinase U-type, mitochondrial OS=Rattus norvegicus GN=Ckmt1 PE=1 SV=1	LCDKTTPTGWTLDQCIQTGVDNPGHPFIK
>	sp|P25809|KCF Creatine kinase U-type, mitochondrial OS=Rattus norvegicus GN=Ckmt1 PE=1 SV=1	VVVDALSGLK
>	sp|P25809|KCF Creatine kinase U-type, mitochondrial OS=Rattus norvegicus GN=Ckmt1 PE=1 SV=1	LPLLSK
>	sp|P25809|KCF Creatine kinase U-type, mitochondrial OS=Rattus norvegicus GN=Ckmt1 PE=1 SV=1	LPLLSK
>	sp|P25809|KCF Creatine kinase U-type, mitochondrial OS=Rattus norvegicus GN=Ckmt1 PE=1 SV=1	AGVHVK
>	sp|P25809|KCF Creatine kinase U-type, mitochondrial OS=Rattus norvegicus GN=Ckmt1 PE=1 SV=1	GLSLPPACTR
>	sp|P25809|KCF Creatine kinase U-type, mitochondrial OS=Rattus norvegicus GN=Ckmt1 PE=1 SV=1	LPLLSK
>	sp|P25809|KCF Creatine kinase U-type, mitochondrial OS=Rattus norvegicus GN=Ckmt1 PE=1 SV=1	DWPDAR
>	sp|P25809|KCF Creatine kinase U-type, mitochondrial OS=Rattus norvegicus GN=Ckmt1 PE=1 SV=1	LPLLSK
>	sp|P25809|KCF Creatine kinase U-type, mitochondrial OS=Rattus norvegicus GN=Ckmt1 PE=1 SV=1	LSEMTEAEQQQLIDDHFLFDKPVSPLLTAAGMAR
>	sp|P25809|KCF Creatine kinase U-type, mitochondrial OS=Rattus norvegicus GN=Ckmt1 PE=1 SV=1	LSEMTEAEQQQLIDDHFLFDKPVSPLLTAAGMAR
>	sp|P25809|KCF Creatine kinase U-type, mitochondrial OS=Rattus norvegicus GN=Ckmt1 PE=1 SV=1	SFLIWVNEEDHTR
>	sp|P25809|KCF Creatine kinase U-type, mitochondrial OS=Rattus norvegicus GN=Ckmt1 PE=1 SV=1	SFLIWVNEEDHTR
>	sp|P25809|KCF Creatine kinase U-type, mitochondrial OS=Rattus norvegicus GN=Ckmt1 PE=1 SV=1	LGKSEVELVQLVIDGVNYLIDCER
>	sp|P19332|TAL Microtubule-associated protein tau OS=Rattus norvegicus GN=Mapt PE=1 SV=3	LQTAPVPMPDLK
>	sp|P19332|TAL Microtubule-associated protein tau OS=Rattus norvegicus GN=Mapt PE=1 SV=3	TDHGAEIVYK
>	sp|P19332|TAL Microtubule-associated protein tau OS=Rattus norvegicus GN=Mapt PE=1 SV=3	GAATPGQK
>	sp|P19332|TAL Microtubule-associated protein tau OS=Rattus norvegicus GN=Mapt PE=1 SV=3	TPPKSPSASK
>	sp|P19332|TAL Microtubule-associated protein tau OS=Rattus norvegicus GN=Mapt PE=1 SV=3	KLDLSNVQSK
>	sp|P19332|TAL Microtubule-associated protein tau OS=Rattus norvegicus GN=Mapt PE=1 SV=3	TDHGAEIVYK
>	sp|P19332|TAL Microtubule-associated protein tau OS=Rattus norvegicus GN=Mapt PE=1 SV=3	LQTAPVPMPDLK
>	sp|P19332|TAL Microtubule-associated protein tau OS=Rattus norvegicus GN=Mapt PE=1 SV=3	IGSTENLK
>	sp|P19332|TAL Microtubule-associated protein tau OS=Rattus norvegicus GN=Mapt PE=1 SV=3	AKTDHGAEIVYK
>	sp|P19332|TAL Microtubule-associated protein tau OS=Rattus norvegicus GN=Mapt PE=1 SV=3	AKTDHGAEIVYK
>	sp|P19332|TAL Microtubule-associated protein tau OS=Rattus norvegicus GN=Mapt PE=1 SV=3	AKTDHGAEIVYK
>	sp|P19332|TAL Microtubule-associated protein tau OS=Rattus norvegicus GN=Mapt PE=1 SV=3	IGSLDNITHVPGGGNK
>	sp|P19332|TAL Microtubule-associated protein tau OS=Rattus norvegicus GN=Mapt PE=1 SV=3	AKTDHGAEIVYK
>	sp|Q9Z1P2|AC Alpha-actinin-1 OS=Rattus norvegicus GN=Actn1 PE=1 SV=1	ATLPDADKER
>	sp|Q9Z1P2|AC Alpha-actinin-1 OS=Rattus norvegicus GN=Actn1 PE=1 SV=1	IDQLEGDHQLIQEALIFDNK
>	sp|Q9Z1P2|AC Alpha-actinin-1 OS=Rattus norvegicus GN=Actn1 PE=1 SV=1	RDQALTEEHSR
>	sp|Q9Z1P2|AC Alpha-actinin-1 OS=Rattus norvegicus GN=Actn1 PE=1 SV=1	VGWEQLLTTIAR
>	sp|Q9Z1P2|AC Alpha-actinin-1 OS=Rattus norvegicus GN=Actn1 PE=1 SV=1	LLETIDQLYLEYAK
>	sp|Q9Z1P2|AC Alpha-actinin-1 OS=Rattus norvegicus GN=Actn1 PE=1 SV=1	DGLGFCALIHR
>	sp|Q9Z1P2|AC Alpha-actinin-1 OS=Rattus norvegicus GN=Actn1 PE=1 SV=1	IDQLEGDHQLIQEALIFDNK
>	sp|Q9Z1P2|AC Alpha-actinin-1 OS=Rattus norvegicus GN=Actn1 PE=1 SV=1	IDQLEGDHQLIQEALIFDNK
>	sp|Q9Z1P2|AC Alpha-actinin-1 OS=Rattus norvegicus GN=Actn1 PE=1 SV=1	IDQLEGDHQLIQEALIFDNK
>	sp|Q9Z1P2|AC Alpha-actinin-1 OS=Rattus norvegicus GN=Actn1 PE=1 SV=1	IDQLEGDHQLIQEALIFDNK
>	sp|Q9Z1P2|AC Alpha-actinin-1 OS=Rattus norvegicus GN=Actn1 PE=1 SV=1	IDQLEGDHQLIQEALIFDNK
>	sp|Q9Z1P2|AC Alpha-actinin-1 OS=Rattus norvegicus GN=Actn1 PE=1 SV=1	IDQLEGDHQLIQEALIFDNK
>	sp|Q9Z1P2|AC Alpha-actinin-1 OS=Rattus norvegicus GN=Actn1 PE=1 SV=1	IDQLEGDHQLIQEALIFDNK
>	sp|Q9Z1P2|AC Alpha-actinin-1 OS=Rattus norvegicus GN=Actn1 PE=1 SV=1	IDQLEGDHQLIQEALIFDNK
>	sp|Q9Z1P2|AC Alpha-actinin-1 OS=Rattus norvegicus GN=Actn1 PE=1 SV=1	IDQLEGDHQLIQEALIFDNK
>	sp|Q9Z1P2|AC Alpha-actinin-1 OS=Rattus norvegicus GN=Actn1 PE=1 SV=1	VGWEQLLTTIAR
>	sp|Q9Z1P2|AC Alpha-actinin-1 OS=Rattus norvegicus GN=Actn1 PE=1 SV=1	KDDPLTNLNTAFDVAER
>	sp|Q9Z1P2|AC Alpha-actinin-1 OS=Rattus norvegicus GN=Actn1 PE=1 SV=1	IDQLEGDHQLIQEALIFDNK
>	sp|Q9Z1P2|AC Alpha-actinin-1 OS=Rattus norvegicus GN=Actn1 PE=1 SV=1	IDQLEGDHQLIQEALIFDNK
>	sp|Q9Z1P2|AC Alpha-actinin-1 OS=Rattus norvegicus GN=Actn1 PE=1 SV=1	ASIHEAWTDGK
>	sp|Q9Z1P2|AC Alpha-actinin-1 OS=Rattus norvegicus GN=Actn1 PE=1 SV=1	RDQALTEEHSR
>	sp|Q9Z1P2|AC Alpha-actinin-1 OS=Rattus norvegicus GN=Actn1 PE=1 SV=1	ISIEMHGTLEDQLSHLR
>	sp|Q9Z1P2|AC Alpha-actinin-1 OS=Rattus norvegicus GN=Actn1 PE=1 SV=1	LDHLAEK
>	sp|P13596|NC Neural cell adhesion molecule 1 OS=Rattus norvegicus GN=Ncam1 PE=1 SV=1	EANMEGIVTIMGLKPETR
>	sp|P13596|NC Neural cell adhesion molecule 1 OS=Rattus norvegicus GN=Ncam1 PE=1 SV=1	FFLCQVAGDAK
>	sp|P13596|NC Neural cell adhesion molecule 1 OS=Rattus norvegicus GN=Ncam1 PE=1 SV=1	IGQESLEFILVQADTPSSPSIDRVEPYSSTAQVQFDEPEATGGVPILK
>	sp|P13596|NC Neural cell adhesion molecule 1 OS=Rattus norvegicus GN=Ncam1 PE=1 SV=1	NVDKNDEAEYVCIAENK
>	sp|P13596|NC Neural cell adhesion molecule 1 OS=Rattus norvegicus GN=Ncam1 PE=1 SV=1	SEPQESEAKPAPTEVK
>	sp|P13596|NC Neural cell adhesion molecule 1 OS=Rattus norvegicus GN=Ncam1 PE=1 SV=1	GLGEISAATEFK
>	sp|P13596|NC Neural cell adhesion molecule 1 OS=Rattus norvegicus GN=Ncam1 PE=1 SV=1	QETLDGHMVVR
>	sp|P13596|NC Neural cell adhesion molecule 1 OS=Rattus norvegicus GN=Ncam1 PE=1 SV=1	DGEPIENEEEDDEKHIFSDDSSELTIR
>	sp|P13596|NC Neural cell adhesion molecule 1 OS=Rattus norvegicus GN=Ncam1 PE=1 SV=1	EANMEGIVTIMGLKPETR
>	sp|P13596|NC Neural cell adhesion molecule 1 OS=Rattus norvegicus GN=Ncam1 PE=1 SV=1	EANMEGIVTIMGLKPETR
>	sp|P13596|NC Neural cell adhesion molecule 1 OS=Rattus norvegicus GN=Ncam1 PE=1 SV=1	EANMEGIVTIMGLKPETR
>	sp|P13596|NC Neural cell adhesion molecule 1 OS=Rattus norvegicus GN=Ncam1 PE=1 SV=1	GLGEISAATEFK

>	sp|P13596|NC Neural cell adhesion molecule 1 OS=Rattus norvegicus GN=Ncam1 PE=1 SV=1	GLGEISAATEFK
>	sp|P13596|NC Neural cell adhesion molecule 1 OS=Rattus norvegicus GN=Ncam1 PE=1 SV=1	NVDKNDEAEYVCIAENK
>	sp|P13596|NC Neural cell adhesion molecule 1 OS=Rattus norvegicus GN=Ncam1 PE=1 SV=1	FFLCQVAGDAK
>	sp|P13596|NC Neural cell adhesion molecule 1 OS=Rattus norvegicus GN=Ncam1 PE=1 SV=1	EANMEGIVTIMGLKPETR
>	sp|P13596|NC Neural cell adhesion molecule 1 OS=Rattus norvegicus GN=Ncam1 PE=1 SV=1	FFLCQVAGDAK
>	sp|P13596|NC Neural cell adhesion molecule 1 OS=Rattus norvegicus GN=Ncam1 PE=1 SV=1	GLGEISAATEFK
>	sp|P13596|NC Neural cell adhesion molecule 1 OS=Rattus norvegicus GN=Ncam1 PE=1 SV=1	QETLDGHMVVR
>	sp|P13596|NC Neural cell adhesion molecule 1 OS=Rattus norvegicus GN=Ncam1 PE=1 SV=1	TQPVREPSAPK
>	sp|P13596|NC Neural cell adhesion molecule 1 OS=Rattus norvegicus GN=Ncam1 PE=1 SV=1	DKDISWFSPNGEK
>	sp|P13596|NC Neural cell adhesion molecule 1 OS=Rattus norvegicus GN=Ncam1 PE=1 SV=1	DGEPIENEEEDDEKHIFSDDSSELTIR
>	sp|P13596|NC Neural cell adhesion molecule 1 OS=Rattus norvegicus GN=Ncam1 PE=1 SV=1	DGEPIENEEEDDEKHIFSDDSSELTIR
>	sp|P15205|MA Microtubule-associated protein 1B OS=Rattus norvegicus GN=Map1b PE=1 SV=2	FYLLVVVGETVTEEHLR
>	sp|P15205|MA Microtubule-associated protein 1B OS=Rattus norvegicus GN=Map1b PE=1 SV=2	HLDRVDSILLTHIGDDNLPGINSMLQR
>	sp|P15205|MA Microtubule-associated protein 1B OS=Rattus norvegicus GN=Map1b PE=1 SV=2	HSPTEDEEIAKAEADVHIK
>	sp|P15205|MA Microtubule-associated protein 1B OS=Rattus norvegicus GN=Map1b PE=1 SV=2	SSMLFDTMQHHLALSR
>	sp|P15205|MA Microtubule-associated protein 1B OS=Rattus norvegicus GN=Map1b PE=1 SV=2	FYLLVVVGETVTEEHLR
>	sp|P15205|MA Microtubule-associated protein 1B OS=Rattus norvegicus GN=Map1b PE=1 SV=2	NLISPDLGVVFLNVPENLKNPEPNIK
>	sp|P15205|MA Microtubule-associated protein 1B OS=Rattus norvegicus GN=Map1b PE=1 SV=2	DLTGQVSTPPVK
>	sp|P15205|MA Microtubule-associated protein 1B OS=Rattus norvegicus GN=Map1b PE=1 SV=2	NLISPDLGVVFLNVPENLKNPEPNIK
>	sp|P15205|MA Microtubule-associated protein 1B OS=Rattus norvegicus GN=Map1b PE=1 SV=2	HSPTEDEEIAKAEADVHIK
>	sp|P15205|MA Microtubule-associated protein 1B OS=Rattus norvegicus GN=Map1b PE=1 SV=2	KESKEEAPEATK
>	sp|P15205|MA Microtubule-associated protein 1B OS=Rattus norvegicus GN=Map1b PE=1 SV=2	KESKEEAPEATK
>	sp|P47858|K6P 6-phosphofructokinase, muscle type OS=Rattus norvegicus GN=Pfkm PE=2 SV=3	GITNLCVIGGDGSLTGADTFR
>	sp|P47858|K6P 6-phosphofructokinase, muscle type OS=Rattus norvegicus GN=Pfkm PE=2 SV=3	IFANTPDSGCVLGMR
>	sp|P47858|K6P 6-phosphofructokinase, muscle type OS=Rattus norvegicus GN=Pfkm PE=2 SV=3	IVEIVDAITTTAQSHQR
>	sp|P47858|K6P 6-phosphofructokinase, muscle type OS=Rattus norvegicus GN=Pfkm PE=2 SV=3	SEWSDLLNDLQK
>	sp|P47858|K6P 6-phosphofructokinase, muscle type OS=Rattus norvegicus GN=Pfkm PE=2 SV=3	VLVVHDGFEGLAK
>	sp|P47858|K6P 6-phosphofructokinase, muscle type OS=Rattus norvegicus GN=Pfkm PE=2 SV=3	IVEIVDAITTTAQSHQR
>	sp|P47858|K6P 6-phosphofructokinase, muscle type OS=Rattus norvegicus GN=Pfkm PE=2 SV=3	IVEIVDAITTTAQSHQR
>	sp|P47858|K6P 6-phosphofructokinase, muscle type OS=Rattus norvegicus GN=Pfkm PE=2 SV=3	IVEIVDAITTTAQSHQR
>	sp|P47858|K6P 6-phosphofructokinase, muscle type OS=Rattus norvegicus GN=Pfkm PE=2 SV=3	IVEIVDAITTTAQSHQR
>	sp|P47858|K6P 6-phosphofructokinase, muscle type OS=Rattus norvegicus GN=Pfkm PE=2 SV=3	IVEIVDAITTTAQSHQR
>	sp|P47858|K6P 6-phosphofructokinase, muscle type OS=Rattus norvegicus GN=Pfkm PE=2 SV=3	IVEIVDAITTTAQSHQR
>	sp|P47858|K6P 6-phosphofructokinase, muscle type OS=Rattus norvegicus GN=Pfkm PE=2 SV=3	IVEIVDAITTTAQSHQR
>	sp|P47858|K6P 6-phosphofructokinase, muscle type OS=Rattus norvegicus GN=Pfkm PE=2 SV=3	IVEIVDAITTTAQSHQR
>	sp|P47858|K6P 6-phosphofructokinase, muscle type OS=Rattus norvegicus GN=Pfkm PE=2 SV=3	IVEIVDAITTTAQSHQR
>	sp|P47858|K6P 6-phosphofructokinase, muscle type OS=Rattus norvegicus GN=Pfkm PE=2 SV=3	IVEIVDAITTTAQSHQR
>	sp|P47858|K6P 6-phosphofructokinase, muscle type OS=Rattus norvegicus GN=Pfkm PE=2 SV=3	IVEIVDAITTTAQSHQR
>	sp|P47858|K6P 6-phosphofructokinase, muscle type OS=Rattus norvegicus GN=Pfkm PE=2 SV=3	IVEIVDAITTTAQSHQR
>	sp|P47858|K6P 6-phosphofructokinase, muscle type OS=Rattus norvegicus GN=Pfkm PE=2 SV=3	VLVVHDGFEGLAK
>	sp|P47858|K6P 6-phosphofructokinase, muscle type OS=Rattus norvegicus GN=Pfkm PE=2 SV=3	VLVVHDGFEGLAK
>	sp|P47858|K6P 6-phosphofructokinase, muscle type OS=Rattus norvegicus GN=Pfkm PE=2 SV=3	VLVVHDGFEGLAK
>	sp|P47858|K6P 6-phosphofructokinase, muscle type OS=Rattus norvegicus GN=Pfkm PE=2 SV=3	VLVVHDGFEGLAK
>	sp|P47858|K6P 6-phosphofructokinase, muscle type OS=Rattus norvegicus GN=Pfkm PE=2 SV=3	SEWSDLLNDLQK
>	sp|P47858|K6P 6-phosphofructokinase, muscle type OS=Rattus norvegicus GN=Pfkm PE=2 SV=3	IVEIVDAITTTAQSHQR
>	sp|P47858|K6P 6-phosphofructokinase, muscle type OS=Rattus norvegicus GN=Pfkm PE=2 SV=3	VTVLGHVQR
>	sp|Q63198|CN Contactin-1 OS=Rattus norvegicus GN=Cntn1 PE=1 SV=2	IKTDGAAPNVAPSDVGGGGGTNR
>	sp|Q63198|CN Contactin-1 OS=Rattus norvegicus GN=Cntn1 PE=1 SV=2	ILALAPTFEMNPMKK
>	sp|Q63198|CN Contactin-1 OS=Rattus norvegicus GN=Cntn1 PE=1 SV=2	WLLNEFPVFITMDKR
>	sp|Q63198|CN Contactin-1 OS=Rattus norvegicus GN=Cntn1 PE=1 SV=2	ILALAPTFEMNPMK
>	sp|Q63198|CN Contactin-1 OS=Rattus norvegicus GN=Cntn1 PE=1 SV=2	AVDLIPWMEYEFR
>	sp|Q63198|CN Contactin-1 OS=Rattus norvegicus GN=Cntn1 PE=1 SV=2	FIPLIPIPER
>	sp|Q63198|CN Contactin-1 OS=Rattus norvegicus GN=Cntn1 PE=1 SV=2	FIPLIPIPER
>	sp|Q63198|CN Contactin-1 OS=Rattus norvegicus GN=Cntn1 PE=1 SV=2	IKTDGAAPNVAPSDVGGGGGTNR
>	sp|Q63198|CN Contactin-1 OS=Rattus norvegicus GN=Cntn1 PE=1 SV=2	WLLNEFPVFITMDKR
>	sp|Q63198|CN Contactin-1 OS=Rattus norvegicus GN=Cntn1 PE=1 SV=2	AHSDGGDGVVSQVK
>	sp|Q63198|CN Contactin-1 OS=Rattus norvegicus GN=Cntn1 PE=1 SV=2	AHSDGGDGVVSQVK
>	sp|Q63198|CN Contactin-1 OS=Rattus norvegicus GN=Cntn1 PE=1 SV=2	AHSDGGDGVVSQVK
>	sp|Q63198|CN Contactin-1 OS=Rattus norvegicus GN=Cntn1 PE=1 SV=2	AHSDGGDGVVSQVK
>	sp|Q63198|CN Contactin-1 OS=Rattus norvegicus GN=Cntn1 PE=1 SV=2	ELTITWAPLSR
>	sp|A7VJC2|RO Heterogeneous nuclear ribonucleoproteins A2/B1 OS=Rattus norvegicus GN=Hnrnpa2b1 PE=1 SV=1	GFGFVTFSSMAEVDAAMAARPHSIDGR
>	sp|A7VJC2|RO Heterogeneous nuclear ribonucleoproteins A2/B1 OS=Rattus norvegicus GN=Hnrnpa2b1 PE=1 SV=1	KLFIGGLSFETTEESLR
>	sp|A7VJC2|RO Heterogeneous nuclear ribonucleoproteins A2/B1 OS=Rattus norvegicus GN=Hnrnpa2b1 PE=1 SV=1	YHTINGHNAEVR
>	sp|A7VJC2|RO Heterogeneous nuclear ribonucleoproteins A2/B1 OS=Rattus norvegicus GN=Hnrnpa2b1 PE=1 SV=1	GFGFVTFDDHDPVDK
>	sp|A7VJC2|RO Heterogeneous nuclear ribonucleoproteins A2/B1 OS=Rattus norvegicus GN=Hnrnpa2b1 PE=1 SV=1	GFGFVTFSSMAEVDAAMAARPHSIDGR
>	sp|A7VJC2|RO Heterogeneous nuclear ribonucleoproteins A2/B1 OS=Rattus norvegicus GN=Hnrnpa2b1 PE=1 SV=1	GFGFVTFSSMAEVDAAMAARPHSIDGR
>	sp|A7VJC2|RO Heterogeneous nuclear ribonucleoproteins A2/B1 OS=Rattus norvegicus GN=Hnrnpa2b1 PE=1 SV=1	GFGFVTFSSMAEVDAAMAARPHSIDGR
>	sp|A7VJC2|RO Heterogeneous nuclear ribonucleoproteins A2/B1 OS=Rattus norvegicus GN=Hnrnpa2b1 PE=1 SV=1	KLFIGGLSFETTEESLR
>	sp|A7VJC2|RO Heterogeneous nuclear ribonucleoproteins A2/B1 OS=Rattus norvegicus GN=Hnrnpa2b1 PE=1 SV=1	KLFIGGLSFETTEESLR
>	sp|A7VJC2|RO Heterogeneous nuclear ribonucleoproteins A2/B1 OS=Rattus norvegicus GN=Hnrnpa2b1 PE=1 SV=1	KLFIGGLSFETTEESLR
>	sp|A7VJC2|RO Heterogeneous nuclear ribonucleoproteins A2/B1 OS=Rattus norvegicus GN=Hnrnpa2b1 PE=1 SV=1	YHTINGHNAEVR
>	sp|A7VJC2|RO Heterogeneous nuclear ribonucleoproteins A2/B1 OS=Rattus norvegicus GN=Hnrnpa2b1 PE=1 SV=1	EESGKPGAHVTVK
>	sp|A7VJC2|RO Heterogeneous nuclear ribonucleoproteins A2/B1 OS=Rattus norvegicus GN=Hnrnpa2b1 PE=1 SV=1	IDTIEIITDR
>	sp|A7VJC2|RO Heterogeneous nuclear ribonucleoproteins A2/B1 OS=Rattus norvegicus GN=Hnrnpa2b1 PE=1 SV=1	KLFVGGIKEDTEEHHLR

>	sp|A7VJC2|ROA	Heterogeneous nuclear ribonucleoproteins A2/B1 OS=Rattus norvegicus GN=Hnrnpa2b1 PE=1 SV=1	KLFIGGLSFETTEESLR
>	sp|P34058|HS9	Heat shock protein HSP 90-beta OS=Rattus norvegicus GN=Hsp90ab1 PE=1 SV=4	HLEINPDHPIVETLR
>	sp|P34058|HS9	Heat shock protein HSP 90-beta OS=Rattus norvegicus GN=Hsp90ab1 PE=1 SV=4	KCLELFSELAEDKENYKK
>	sp|P34058|HS9	Heat shock protein HSP 90-beta OS=Rattus norvegicus GN=Hsp90ab1 PE=1 SV=4	RAPFDLFENK
>	sp|P34058|HS9	Heat shock protein HSP 90-beta OS=Rattus norvegicus GN=Hsp90ab1 PE=1 SV=4	QKAEADKNDKAVKDLVVLLFETALLSSGFSLEDPQTHSNR
>	sp|P34058|HS9	Heat shock protein HSP 90-beta OS=Rattus norvegicus GN=Hsp90ab1 PE=1 SV=4	ADLINNLGTIAK
>	sp|P34058|HS9	Heat shock protein HSP 90-beta OS=Rattus norvegicus GN=Hsp90ab1 PE=1 SV=4	GVVDSEDLPLNISR
>	sp|P34058|HS9	Heat shock protein HSP 90-beta OS=Rattus norvegicus GN=Hsp90ab1 PE=1 SV=4	GVVDSEDLPLNISR
>	sp|P34058|HS9	Heat shock protein HSP 90-beta OS=Rattus norvegicus GN=Hsp90ab1 PE=1 SV=4	GVVDSEDLPLNISR
>	sp|P34058|HS9	Heat shock protein HSP 90-beta OS=Rattus norvegicus GN=Hsp90ab1 PE=1 SV=4	HNDDEQYAWESSAGGSFTVR
>	sp|P34058|HS9	Heat shock protein HSP 90-beta OS=Rattus norvegicus GN=Hsp90ab1 PE=1 SV=4	HNDDEQYAWESSAGGSFTVR
>	sp|P34058|HS9	Heat shock protein HSP 90-beta OS=Rattus norvegicus GN=Hsp90ab1 PE=1 SV=4	HNDDEQYAWESSAGGSFTVR
>	sp|P34058|HS9	Heat shock protein HSP 90-beta OS=Rattus norvegicus GN=Hsp90ab1 PE=1 SV=4	HNDDEQYAWESSAGGSFTVR
>	sp|P34058|HS9	Heat shock protein HSP 90-beta OS=Rattus norvegicus GN=Hsp90ab1 PE=1 SV=4	TLTLVDTGIGMTK
>	sp|P34058|HS9	Heat shock protein HSP 90-beta OS=Rattus norvegicus GN=Hsp90ab1 PE=1 SV=4	VILHLKEDQTEYLEER
>	sp|P34058|HS9	Heat shock protein HSP 90-beta OS=Rattus norvegicus GN=Hsp90ab1 PE=1 SV=4	VILHLKEDQTEYLEER
>	sp|P34058|HS9	Heat shock protein HSP 90-beta OS=Rattus norvegicus GN=Hsp90ab1 PE=1 SV=4	AEADKNDKAVKDLVVLLFETALLSSGFSLEDPQTHSNR
>	sp|P34058|HS9	Heat shock protein HSP 90-beta OS=Rattus norvegicus GN=Hsp90ab1 PE=1 SV=4	ADLINNLGTIAK
>	sp|P34058|HS9	Heat shock protein HSP 90-beta OS=Rattus norvegicus GN=Hsp90ab1 PE=1 SV=4	ADLINNLGTIAK
>	sp|P34058|HS9	Heat shock protein HSP 90-beta OS=Rattus norvegicus GN=Hsp90ab1 PE=1 SV=4	HLEINPDHPIVETLR
>	sp|P34058|HS9	Heat shock protein HSP 90-beta OS=Rattus norvegicus GN=Hsp90ab1 PE=1 SV=4	VILHLKEDQTEYLEER
>	sp|P34058|HS9	Heat shock protein HSP 90-beta OS=Rattus norvegicus GN=Hsp90ab1 PE=1 SV=4	GTKVILHLKEDQTEYLEER
>	sp|P34058|HS9	Heat shock protein HSP 90-beta OS=Rattus norvegicus GN=Hsp90ab1 PE=1 SV=4	RAPFDLFENKK
>	sp|P34058|HS9	Heat shock protein HSP 90-beta OS=Rattus norvegicus GN=Hsp90ab1 PE=1 SV=4	YIDQEELNK
>	sp|P34058|HS9	Heat shock protein HSP 90-beta OS=Rattus norvegicus GN=Hsp90ab1 PE=1 SV=4	ADLINNLGTIAK
>	sp|P34058|HS9	Heat shock protein HSP 90-beta OS=Rattus norvegicus GN=Hsp90ab1 PE=1 SV=4	YIDQEELNK
>	sp|P34058|HS9	Heat shock protein HSP 90-beta OS=Rattus norvegicus GN=Hsp90ab1 PE=1 SV=4	HLEINPDHPIVETLR
>	sp|P34058|HS9	Heat shock protein HSP 90-beta OS=Rattus norvegicus GN=Hsp90ab1 PE=1 SV=4	RAPFDLFENKK
>	sp|P34058|HS9	Heat shock protein HSP 90-beta OS=Rattus norvegicus GN=Hsp90ab1 PE=1 SV=4	LSELLR
>	sp|P34058|HS9	Heat shock protein HSP 90-beta OS=Rattus norvegicus GN=Hsp90ab1 PE=1 SV=4	HLEINPDHPIVETLR
>	sp|P34058|HS9	Heat shock protein HSP 90-beta OS=Rattus norvegicus GN=Hsp90ab1 PE=1 SV=4	YIDQEELNK
>	sp|P34058|HS9	Heat shock protein HSP 90-beta OS=Rattus norvegicus GN=Hsp90ab1 PE=1 SV=4	CLELFSELAEDKENYKK
>	sp|P34058|HS9	Heat shock protein HSP 90-beta OS=Rattus norvegicus GN=Hsp90ab1 PE=1 SV=4	YIDQEELNK
>	sp|P34058|HS9	Heat shock protein HSP 90-beta OS=Rattus norvegicus GN=Hsp90ab1 PE=1 SV=4	YIDQEELNK
>	sp|Q5RJQ4|SIR	NAD-dependent deacetylase sirtuin-2 OS=Rattus norvegicus GN=Sirt2 PE=1 SV=1	EHANIDAQSGSQASNPSATVSPR
>	sp|Q5RJQ4|SIR	NAD-dependent deacetylase sirtuin-2 OS=Rattus norvegicus GN=Sirt2 PE=1 SV=1	NLFTQTLGLGSQK
>	sp|Q5RJQ4|SIR	NAD-dependent deacetylase sirtuin-2 OS=Rattus norvegicus GN=Sirt2 PE=1 SV=1	REHANIDAQSGSQASNPSATVSPR
>	sp|Q5RJQ4|SIR	NAD-dependent deacetylase sirtuin-2 OS=Rattus norvegicus GN=Sirt2 PE=1 SV=1	VICLVGAGISTSAGIPDFR
>	sp|Q5RJQ4|SIR	NAD-dependent deacetylase sirtuin-2 OS=Rattus norvegicus GN=Sirt2 PE=1 SV=1	YHLPYPEAIFEISYFK
>	sp|Q5RJQ4|SIR	NAD-dependent deacetylase sirtuin-2 OS=Rattus norvegicus GN=Sirt2 PE=1 SV=1	LLDELTLEGVTR
>	sp|Q5RJQ4|SIR	NAD-dependent deacetylase sirtuin-2 OS=Rattus norvegicus GN=Sirt2 PE=1 SV=1	EHANIDAQSGSQASNPSATVSPR
>	sp|Q5RJQ4|SIR	NAD-dependent deacetylase sirtuin-2 OS=Rattus norvegicus GN=Sirt2 PE=1 SV=1	LLDELTLEGVTR
>	sp|Q5RJQ4|SIR	NAD-dependent deacetylase sirtuin-2 OS=Rattus norvegicus GN=Sirt2 PE=1 SV=1	LLDELTLEGVTR
>	sp|Q5RJQ4|SIR	NAD-dependent deacetylase sirtuin-2 OS=Rattus norvegicus GN=Sirt2 PE=1 SV=1	NLFTQTLGLGSQK
>	sp|Q5RJQ4|SIR	NAD-dependent deacetylase sirtuin-2 OS=Rattus norvegicus GN=Sirt2 PE=1 SV=1	VICLVGAGISTSAGIPDFR
>	sp|Q5RJQ4|SIR	NAD-dependent deacetylase sirtuin-2 OS=Rattus norvegicus GN=Sirt2 PE=1 SV=1	YHLPYPEAIFEISYFK
>	sp|Q5RJQ4|SIR	NAD-dependent deacetylase sirtuin-2 OS=Rattus norvegicus GN=Sirt2 PE=1 SV=1	YHLPYPEAIFEISYFK
>	sp|Q5RJQ4|SIR	NAD-dependent deacetylase sirtuin-2 OS=Rattus norvegicus GN=Sirt2 PE=1 SV=1	YHLPYPEAIFEISYFK
>	sp|Q5RJQ4|SIR	NAD-dependent deacetylase sirtuin-2 OS=Rattus norvegicus GN=Sirt2 PE=1 SV=1	YHLPYPEAIFEISYFK
>	sp|Q5RJQ4|SIR	NAD-dependent deacetylase sirtuin-2 OS=Rattus norvegicus GN=Sirt2 PE=1 SV=1	YHLPYPEAIFEISYFK
>	sp|Q5RJQ4|SIR	NAD-dependent deacetylase sirtuin-2 OS=Rattus norvegicus GN=Sirt2 PE=1 SV=1	EHANIDAQSGSQASNPSATVSPR
>	sp|Q5RJQ4|SIR	NAD-dependent deacetylase sirtuin-2 OS=Rattus norvegicus GN=Sirt2 PE=1 SV=1	NLFTQTLGLGSQK
>	sp|Q5RJQ4|SIR	NAD-dependent deacetylase sirtuin-2 OS=Rattus norvegicus GN=Sirt2 PE=1 SV=1	YHLPYPEAIFEISYFK
>	sp|Q5RJQ4|SIR	NAD-dependent deacetylase sirtuin-2 OS=Rattus norvegicus GN=Sirt2 PE=1 SV=1	YHLPYPEAIFEISYFK
>	sp|Q5RJQ4|SIR	NAD-dependent deacetylase sirtuin-2 OS=Rattus norvegicus GN=Sirt2 PE=1 SV=1	EHANIDAQSGSQASNPSATVSPR
>	sp|Q5RJQ4|SIR	NAD-dependent deacetylase sirtuin-2 OS=Rattus norvegicus GN=Sirt2 PE=1 SV=1	YHLPYPEAIFEISYFK
>	sp|Q64548|RTN	Reticulon-1 OS=Rattus norvegicus GN=Rtn1 PE=2 SV=1	HQAQVDQYLGLVR
>	sp|Q64548|RTN	Reticulon-1 OS=Rattus norvegicus GN=Rtn1 PE=2 SV=1	HQAQVDQYLGLVRTHINTVVAKIQAKIPGAK
>	sp|Q64548|RTN	Reticulon-1 OS=Rattus norvegicus GN=Rtn1 PE=2 SV=1	LIKDNLFEESTFAPYIDELSDEQHR
>	sp|Q64548|RTN	Reticulon-1 OS=Rattus norvegicus GN=Rtn1 PE=2 SV=1	THINTVVAK
>	sp|Q64548|RTN	Reticulon-1 OS=Rattus norvegicus GN=Rtn1 PE=2 SV=1	HQAQVDQYLGLVRTHINTVVAKIQAKIPGAKR
>	sp|Q64548|RTN	Reticulon-1 OS=Rattus norvegicus GN=Rtn1 PE=2 SV=1	HQAQVDQYLGLVR
>	sp|Q64548|RTN	Reticulon-1 OS=Rattus norvegicus GN=Rtn1 PE=2 SV=1	HQAQVDQYLGLVR
>	sp|Q64548|RTN	Reticulon-1 OS=Rattus norvegicus GN=Rtn1 PE=2 SV=1	HQAQVDQYLGLVR
>	sp|Q64548|RTN	Reticulon-1 OS=Rattus norvegicus GN=Rtn1 PE=2 SV=1	HQAQVDQYLGLVR
>	sp|Q64548|RTN	Reticulon-1 OS=Rattus norvegicus GN=Rtn1 PE=2 SV=1	HQAQVDQYLGLVRTHINTVVAKIQAKIPGAK
>	sp|Q64548|RTN	Reticulon-1 OS=Rattus norvegicus GN=Rtn1 PE=2 SV=1	HQAQVDQYLGLVRTHINTVVAKIQAKIPGAK
>	sp|Q64548|RTN	Reticulon-1 OS=Rattus norvegicus GN=Rtn1 PE=2 SV=1	THINTVVAK
>	sp|Q64548|RTN	Reticulon-1 OS=Rattus norvegicus GN=Rtn1 PE=2 SV=1	THINTVVAK
>	sp|Q64548|RTN	Reticulon-1 OS=Rattus norvegicus GN=Rtn1 PE=2 SV=1	HQAQVDQYLGLVRTHINTVVAKIQAKIPGAKR
>	sp|Q64548|RTN	Reticulon-1 OS=Rattus norvegicus GN=Rtn1 PE=2 SV=1	HQAQVDQYLGLVRTHINTVVAKIQAKIPGAKR
>	sp|Q64548|RTN	Reticulon-1 OS=Rattus norvegicus GN=Rtn1 PE=2 SV=1	HQAQVDQYLGLVRTHINTVVAKIQAKIPGAKR
>	sp|Q64548|RTN	Reticulon-1 OS=Rattus norvegicus GN=Rtn1 PE=2 SV=1	THINTVVAK
>	sp|Q64548|RTN	Reticulon-1 OS=Rattus norvegicus GN=Rtn1 PE=2 SV=1	HQAQVDQYLGLVRTHINTVVAKIQAKIPGAK

>	sp|Q64548|RTN	Reticulon-1 OS=Rattus norvegicus GN=Rtn1 PE=2 SV=1	HQAQVDQYLGLVR
>	sp|Q64548|RTN	Reticulon-1 OS=Rattus norvegicus GN=Rtn1 PE=2 SV=1	HQAQVDQYLGLVR
>	sp|P63100|CAN	Calcineurin subunit B type 1 OS=Rattus norvegicus GN=Ppp3r1 PE=1 SV=2	ISFEEFCAVVGGLDIHK
>	sp|P63100|CAN	Calcineurin subunit B type 1 OS=Rattus norvegicus GN=Ppp3r1 PE=1 SV=2	IYDMDKDGYISNGELFQVLK
>	sp|P63100|CAN	Calcineurin subunit B type 1 OS=Rattus norvegicus GN=Ppp3r1 PE=1 SV=2	KLDLDNSGSLSVEEFMSLPELQQNPLVQR
>	sp|P63100|CAN	Calcineurin subunit B type 1 OS=Rattus norvegicus GN=Ppp3r1 PE=1 SV=2	VIDIFDTDGNGEVDFKEFIEGVSQFSVKGDKEQK
>	sp|P63100|CAN	Calcineurin subunit B type 1 OS=Rattus norvegicus GN=Ppp3r1 PE=1 SV=2	ISFEEFCAVVGGLDIHKK
>	sp|P63100|CAN	Calcineurin subunit B type 1 OS=Rattus norvegicus GN=Ppp3r1 PE=1 SV=2	ISFEEFCAVVGGLDIHK
>	sp|P63100|CAN	Calcineurin subunit B type 1 OS=Rattus norvegicus GN=Ppp3r1 PE=1 SV=2	ISFEEFCAVVGGLDIHK
>	sp|P63100|CAN	Calcineurin subunit B type 1 OS=Rattus norvegicus GN=Ppp3r1 PE=1 SV=2	ISFEEFCAVVGGLDIHK
>	sp|P63100|CAN	Calcineurin subunit B type 1 OS=Rattus norvegicus GN=Ppp3r1 PE=1 SV=2	ISFEEFCAVVGGLDIHK
>	sp|P63100|CAN	Calcineurin subunit B type 1 OS=Rattus norvegicus GN=Ppp3r1 PE=1 SV=2	ISFEEFCAVVGGLDIHK
>	sp|P63100|CAN	Calcineurin subunit B type 1 OS=Rattus norvegicus GN=Ppp3r1 PE=1 SV=2	ISFEEFCAVVGGLDIHK
>	sp|P63100|CAN	Calcineurin subunit B type 1 OS=Rattus norvegicus GN=Ppp3r1 PE=1 SV=2	ISFEEFCAVVGGLDIHK
>	sp|P63100|CAN	Calcineurin subunit B type 1 OS=Rattus norvegicus GN=Ppp3r1 PE=1 SV=2	ISFEEFCAVVGGLDIHKK
>	sp|P63100|CAN	Calcineurin subunit B type 1 OS=Rattus norvegicus GN=Ppp3r1 PE=1 SV=2	ISFEEFCAVVGGLDIHKK
>	sp|P63100|CAN	Calcineurin subunit B type 1 OS=Rattus norvegicus GN=Ppp3r1 PE=1 SV=2	ISFEEFCAVVGGLDIHK
>	sp|P63100|CAN	Calcineurin subunit B type 1 OS=Rattus norvegicus GN=Ppp3r1 PE=1 SV=2	KLDLDNSGSLSVEEFMSLPELQQNPLVQR
>	sp|P63100|CAN	Calcineurin subunit B type 1 OS=Rattus norvegicus GN=Ppp3r1 PE=1 SV=2	IYDMDKDGYISNGELFQVLK
>	sp|P63100|CAN	Calcineurin subunit B type 1 OS=Rattus norvegicus GN=Ppp3r1 PE=1 SV=2	TIINADKDGDGR
>	sp|P63100|CAN	Calcineurin subunit B type 1 OS=Rattus norvegicus GN=Ppp3r1 PE=1 SV=2	DTQLQQIVDK
>	sp|P63100|CAN	Calcineurin subunit B type 1 OS=Rattus norvegicus GN=Ppp3r1 PE=1 SV=2	VIDIFDTDGNGEVDFKEFIEGVSQFSVKGDKEQK
>	sp|P63100|CAN	Calcineurin subunit B type 1 OS=Rattus norvegicus GN=Ppp3r1 PE=1 SV=2	VIDIFDTDGNGEVDFKEFIEGVSQFSVKGDKEQK
>	sp|P63100|CAN	Calcineurin subunit B type 1 OS=Rattus norvegicus GN=Ppp3r1 PE=1 SV=2	GGLDIHKK
>	sp|Q66HA8|HS	Heat shock protein 105 kDa OS=Rattus norvegicus GN=Hsph1 PE=1 SV=1	GCALQCAILSPAFK
>	sp|Q66HA8|HS	Heat shock protein 105 kDa OS=Rattus norvegicus GN=Hsph1 PE=1 SV=1	IEVPLHLLMEQTHLK
>	sp|Q66HA8|HS	Heat shock protein 105 kDa OS=Rattus norvegicus GN=Hsph1 PE=1 SV=1	VLGTAFDPFLGGK
>	sp|Q66HA8|HS	Heat shock protein 105 kDa OS=Rattus norvegicus GN=Hsph1 PE=1 SV=1	SNLGADAPHQNGECHPNEK
>	sp|Q66HA8|HS	Heat shock protein 105 kDa OS=Rattus norvegicus GN=Hsph1 PE=1 SV=1	QAYIDKLEELMK
>	sp|Q66HA8|HS	Heat shock protein 105 kDa OS=Rattus norvegicus GN=Hsph1 PE=1 SV=1	IEVPLHLLMEQTHLK
>	sp|Q66HA8|HS	Heat shock protein 105 kDa OS=Rattus norvegicus GN=Hsph1 PE=1 SV=1	IEVPLHLLMEQTHLK
>	sp|Q66HA8|HS	Heat shock protein 105 kDa OS=Rattus norvegicus GN=Hsph1 PE=1 SV=1	IEVPLHLLMEQTHLK
>	sp|Q66HA8|HS	Heat shock protein 105 kDa OS=Rattus norvegicus GN=Hsph1 PE=1 SV=1	IEVPLHLLMEQTHLK
>	sp|Q66HA8|HS	Heat shock protein 105 kDa OS=Rattus norvegicus GN=Hsph1 PE=1 SV=1	NHAAPFSK
>	sp|Q66HA8|HS	Heat shock protein 105 kDa OS=Rattus norvegicus GN=Hsph1 PE=1 SV=1	VLGTAFDPFLGGK
>	sp|Q66HA8|HS	Heat shock protein 105 kDa OS=Rattus norvegicus GN=Hsph1 PE=1 SV=1	LVEHFCAEFK
>	sp|O35179|SH3	Endophilin-A1 OS=Rattus norvegicus GN=Sh3gl2 PE=1 SV=2	ELGDDCNFGPALGEVGEAMR
>	sp|O35179|SH3	Endophilin-A1 OS=Rattus norvegicus GN=Sh3gl2 PE=1 SV=2	GPGYPQAEALLAEAMLK
>	sp|O35179|SH3	Endophilin-A1 OS=Rattus norvegicus GN=Sh3gl2 PE=1 SV=2	QAVQILQQVTVR
>	sp|O35179|SH3	Endophilin-A1 OS=Rattus norvegicus GN=Sh3gl2 PE=1 SV=2	MSLEFATGDGTQPNGGLSHTGTPKPAGVQMDQPCCR
>	sp|O35179|SH3	Endophilin-A1 OS=Rattus norvegicus GN=Sh3gl2 PE=1 SV=2	VGGAEGTKLDDDFKEMER
>	sp|O35179|SH3	Endophilin-A1 OS=Rattus norvegicus GN=Sh3gl2 PE=1 SV=2	GPGYPQAEALLAEAMLK
>	sp|O35179|SH3	Endophilin-A1 OS=Rattus norvegicus GN=Sh3gl2 PE=1 SV=2	GPGYPQAEALLAEAMLK
>	sp|O35179|SH3	Endophilin-A1 OS=Rattus norvegicus GN=Sh3gl2 PE=1 SV=2	GPGYPQAEALLAEAMLK
>	sp|O35179|SH3	Endophilin-A1 OS=Rattus norvegicus GN=Sh3gl2 PE=1 SV=2	GPGYPQAEALLAEAMLK
>	sp|O35179|SH3	Endophilin-A1 OS=Rattus norvegicus GN=Sh3gl2 PE=1 SV=2	GPGYPQAEALLAEAMLK
>	sp|O35179|SH3	Endophilin-A1 OS=Rattus norvegicus GN=Sh3gl2 PE=1 SV=2	GPGYPQAEALLAEAMLK
>	sp|O35179|SH3	Endophilin-A1 OS=Rattus norvegicus GN=Sh3gl2 PE=1 SV=2	REYQPKPR
>	sp|O35179|SH3	Endophilin-A1 OS=Rattus norvegicus GN=Sh3gl2 PE=1 SV=2	QNFIDPLQNLHDKDLR
>	sp|O35179|SH3	Endophilin-A1 OS=Rattus norvegicus GN=Sh3gl2 PE=1 SV=2	GPGYPQAEALLAEAMLK
>	sp|O35179|SH3	Endophilin-A1 OS=Rattus norvegicus GN=Sh3gl2 PE=1 SV=2	QNFIDPLQNLHDKDLR
>	sp|O35179|SH3	Endophilin-A1 OS=Rattus norvegicus GN=Sh3gl2 PE=1 SV=2	QNFIDPLQNLHDKDLR
>	sp|P08413|KCC	Calcium/calmodulin-dependent protein kinase type II subunit beta OS=Rattus norvegicus GN=Camk2b PE=1 SV=1	ICDPGLTSFEPEALGNLVEGMDFHR
>	sp|P08413|KCC	Calcium/calmodulin-dependent protein kinase type II subunit beta OS=Rattus norvegicus GN=Camk2b PE=1 SV=1	NLINQMLTINPAK
>	sp|P08413|KCC	Calcium/calmodulin-dependent protein kinase type II subunit beta OS=Rattus norvegicus GN=Camk2b PE=1 SV=1	NLINQMLTINPAKR
>	sp|P08413|KCC	Calcium/calmodulin-dependent protein kinase type II subunit beta OS=Rattus norvegicus GN=Camk2b PE=1 SV=1	LHDSISEEGFHYLVFDLVTGGELFEDIVAR
>	sp|P08413|KCC	Calcium/calmodulin-dependent protein kinase type II subunit beta OS=Rattus norvegicus GN=Camk2b PE=1 SV=1	WQNVHFHCSGAPVAPLQ
>	sp|P08413|KCC	Calcium/calmodulin-dependent protein kinase type II subunit beta OS=Rattus norvegicus GN=Camk2b PE=1 SV=1	AGAYDFPSPEWDTVTPEAK
>	sp|P08413|KCC	Calcium/calmodulin-dependent protein kinase type II subunit beta OS=Rattus norvegicus GN=Camk2b PE=1 SV=1	AGAYDFPSPEWDTVTPEAK
>	sp|P08413|KCC	Calcium/calmodulin-dependent protein kinase type II subunit beta OS=Rattus norvegicus GN=Camk2b PE=1 SV=1	AGAYDFPSPEWDTVTPEAK
>	sp|P08413|KCC	Calcium/calmodulin-dependent protein kinase type II subunit beta OS=Rattus norvegicus GN=Camk2b PE=1 SV=1	AGAYDFPSPEWDTVTPEAK
>	sp|P08413|KCC	Calcium/calmodulin-dependent protein kinase type II subunit beta OS=Rattus norvegicus GN=Camk2b PE=1 SV=1	DLKPENLLLASK
>	sp|P08413|KCC	Calcium/calmodulin-dependent protein kinase type II subunit beta OS=Rattus norvegicus GN=Camk2b PE=1 SV=1	EYYSEADASHCIQQILEAVLHCHQMGVVHR
>	sp|P08413|KCC	Calcium/calmodulin-dependent protein kinase type II subunit beta OS=Rattus norvegicus GN=Camk2b PE=1 SV=1	EYYSEADASHCIQQILEAVLHCHQMGVVHR
>	sp|P08413|KCC	Calcium/calmodulin-dependent protein kinase type II subunit beta OS=Rattus norvegicus GN=Camk2b PE=1 SV=1	EYYSEADASHCIQQILEAVLHCHQMGVVHR
>	sp|P08413|KCC	Calcium/calmodulin-dependent protein kinase type II subunit beta OS=Rattus norvegicus GN=Camk2b PE=1 SV=1	EYYSEADASHCIQQILEAVLHCHQMGVVHR
>	sp|P08413|KCC	Calcium/calmodulin-dependent protein kinase type II subunit beta OS=Rattus norvegicus GN=Camk2b PE=1 SV=1	EYYSEADASHCIQQILEAVLHCHQMGVVHR
>	sp|P08413|KCC	Calcium/calmodulin-dependent protein kinase type II subunit beta OS=Rattus norvegicus GN=Camk2b PE=1 SV=1	EYYSEADASHCIQQILEAVLHCHQMGVVHR
>	sp|P08413|KCC	Calcium/calmodulin-dependent protein kinase type II subunit beta OS=Rattus norvegicus GN=Camk2b PE=1 SV=1	EYYSEADASHCIQQILEAVLHCHQMGVVHR
>	sp|P08413|KCC	Calcium/calmodulin-dependent protein kinase type II subunit beta OS=Rattus norvegicus GN=Camk2b PE=1 SV=1	EYYSEADASHCIQQILEAVLHCHQMGVVHR
>	sp|P08413|KCC	Calcium/calmodulin-dependent protein kinase type II subunit beta OS=Rattus norvegicus GN=Camk2b PE=1 SV=1	EYYSEADASHCIQQILEAVLHCHQMGVVHR
>	sp|P08413|KCC	Calcium/calmodulin-dependent protein kinase type II subunit beta OS=Rattus norvegicus GN=Camk2b PE=1 SV=1	EYYSEADASHCIQQILEAVLHCHQMGVVHR
>	sp|P08413|KCC	Calcium/calmodulin-dependent protein kinase type II subunit beta OS=Rattus norvegicus GN=Camk2b PE=1 SV=1	EYYSEADASHCIQQILEAVLHCHQMGVVHR
>	sp|P08413|KCC	Calcium/calmodulin-dependent protein kinase type II subunit beta OS=Rattus norvegicus GN=Camk2b PE=1 SV=1	EYYSEADASHCIQQILEAVLHCHQMGVVHR

>	sp|P08413|KCC Calcium/calmodulin-dependent protein kinase type II subunit beta OS=Rattus norvegicus GN=Camk2b PE=1 SV=1	EYYSEADASHCIQQILEAVLHCHQMGVVHR
>	sp|P08413|KCC Calcium/calmodulin-dependent protein kinase type II subunit beta OS=Rattus norvegicus GN=Camk2b PE=1 SV=1	EYYSEADASHCIQQILEAVLHCHQMGVVHR
>	sp|P08413|KCC Calcium/calmodulin-dependent protein kinase type II subunit beta OS=Rattus norvegicus GN=Camk2b PE=1 SV=1	EYYSEADASHCIQQILEAVLHCHQMGVVHR
>	sp|P08413|KCC Calcium/calmodulin-dependent protein kinase type II subunit beta OS=Rattus norvegicus GN=Camk2b PE=1 SV=1	EYYSEADASHCIQQILEAVLHCHQMGVVHR
>	sp|P08413|KCC Calcium/calmodulin-dependent protein kinase type II subunit beta OS=Rattus norvegicus GN=Camk2b PE=1 SV=1	EYYSEADASHCIQQILEAVLHCHQMGVVHR
>	sp|P08413|KCC Calcium/calmodulin-dependent protein kinase type II subunit beta OS=Rattus norvegicus GN=Camk2b PE=1 SV=1	EYYSEADASHCIQQILEAVLHCHQMGVVHR
>	sp|P08413|KCC Calcium/calmodulin-dependent protein kinase type II subunit beta OS=Rattus norvegicus GN=Camk2b PE=1 SV=1	EYYSEADASHCIQQILEAVLHCHQMGVVHR
>	sp|P08413|KCC Calcium/calmodulin-dependent protein kinase type II subunit beta OS=Rattus norvegicus GN=Camk2b PE=1 SV=1	EYYSEADASHCIQQILEAVLHCHQMGVVHR
>	sp|P08413|KCC Calcium/calmodulin-dependent protein kinase type II subunit beta OS=Rattus norvegicus GN=Camk2b PE=1 SV=1	EYYSEADASHCIQQILEAVLHCHQMGVVHR
>	sp|P08413|KCC Calcium/calmodulin-dependent protein kinase type II subunit beta OS=Rattus norvegicus GN=Camk2b PE=1 SV=1	EYYSEADASHCIQQILEAVLHCHQMGVVHR
>	sp|P08413|KCC Calcium/calmodulin-dependent protein kinase type II subunit beta OS=Rattus norvegicus GN=Camk2b PE=1 SV=1	EYYSEADASHCIQQILEAVLHCHQMGVVHR
>	sp|P08413|KCC Calcium/calmodulin-dependent protein kinase type II subunit beta OS=Rattus norvegicus GN=Camk2b PE=1 SV=1	EYYSEADASHCIQQILEAVLHCHQMGVVHR
>	sp|P08413|KCC Calcium/calmodulin-dependent protein kinase type II subunit beta OS=Rattus norvegicus GN=Camk2b PE=1 SV=1	EYYSEADASHCIQQILEAVLHCHQMGVVHR
>	sp|P08413|KCC Calcium/calmodulin-dependent protein kinase type II subunit beta OS=Rattus norvegicus GN=Camk2b PE=1 SV=1	EYYSEADASHCIQQILEAVLHCHQMGVVHR
>	sp|P08413|KCC Calcium/calmodulin-dependent protein kinase type II subunit beta OS=Rattus norvegicus GN=Camk2b PE=1 SV=1	EYYSEADASHCIQQILEAVLHCHQMGVVHR
>	sp|P08413|KCC Calcium/calmodulin-dependent protein kinase type II subunit beta OS=Rattus norvegicus GN=Camk2b PE=1 SV=1	EYYSEADASHCIQQILEAVLHCHQMGVVHR
>	sp|P08413|KCC Calcium/calmodulin-dependent protein kinase type II subunit beta OS=Rattus norvegicus GN=Camk2b PE=1 SV=1	EYYSEADASHCIQQILEAVLHCHQMGVVHR
>	sp|P08413|KCC Calcium/calmodulin-dependent protein kinase type II subunit beta OS=Rattus norvegicus GN=Camk2b PE=1 SV=1	EYYSEADASHCIQQILEAVLHCHQMGVVHR
>	sp|P08413|KCC Calcium/calmodulin-dependent protein kinase type II subunit beta OS=Rattus norvegicus GN=Camk2b PE=1 SV=1	EYYSEADASHCIQQILEAVLHCHQMGVVHR
>	sp|P08413|KCC Calcium/calmodulin-dependent protein kinase type II subunit beta OS=Rattus norvegicus GN=Camk2b PE=1 SV=1	EYYSEADASHCIQQILEAVLHCHQMGVVHR
>	sp|P08413|KCC Calcium/calmodulin-dependent protein kinase type II subunit beta OS=Rattus norvegicus GN=Camk2b PE=1 SV=1	EYYSEADASHCIQQILEAVLHCHQMGVVHR
>	sp|P08413|KCC Calcium/calmodulin-dependent protein kinase type II subunit beta OS=Rattus norvegicus GN=Camk2b PE=1 SV=1	EYYSEADASHCIQQILEAVLHCHQMGVVHR
>	sp|P08413|KCC Calcium/calmodulin-dependent protein kinase type II subunit beta OS=Rattus norvegicus GN=Camk2b PE=1 SV=1	EYYSEADASHCIQQILEAVLHCHQMGVVHR
>	sp|P08413|KCC Calcium/calmodulin-dependent protein kinase type II subunit beta OS=Rattus norvegicus GN=Camk2b PE=1 SV=1	EYYSEADASHCIQQILEAVLHCHQMGVVHR
>	sp|P08413|KCC Calcium/calmodulin-dependent protein kinase type II subunit beta OS=Rattus norvegicus GN=Camk2b PE=1 SV=1	EYYSEADASHCIQQILEAVLHCHQMGVVHR
>	sp|P08413|KCC Calcium/calmodulin-dependent protein kinase type II subunit beta OS=Rattus norvegicus GN=Camk2b PE=1 SV=1	EYYSEADASHCIQQILEAVLHCHQMGVVHR
>	sp|P08413|KCC Calcium/calmodulin-dependent protein kinase type II subunit beta OS=Rattus norvegicus GN=Camk2b PE=1 SV=1	EYYSEADASHCIQQILEAVLHCHQMGVVHR
>	sp|P08413|KCC Calcium/calmodulin-dependent protein kinase type II subunit beta OS=Rattus norvegicus GN=Camk2b PE=1 SV=1	EYYSEADASHCIQQILEAVLHCHQMGVVHR
>	sp|P08413|KCC Calcium/calmodulin-dependent protein kinase type II subunit beta OS=Rattus norvegicus GN=Camk2b PE=1 SV=1	EYYSEADASHCIQQILEAVLHCHQMGVVHR
>	sp|P08413|KCC Calcium/calmodulin-dependent protein kinase type II subunit beta OS=Rattus norvegicus GN=Camk2b PE=1 SV=1	EYYSEADASHCIQQILEAVLHCHQMGVVHR
>	sp|P08413|KCC Calcium/calmodulin-dependent protein kinase type II subunit beta OS=Rattus norvegicus GN=Camk2b PE=1 SV=1	GAILTTMLATR
>	sp|P08413|KCC Calcium/calmodulin-dependent protein kinase type II subunit beta OS=Rattus norvegicus GN=Camk2b PE=1 SV=1	GAILTTMLATR
>	sp|P08413|KCC Calcium/calmodulin-dependent protein kinase type II subunit beta OS=Rattus norvegicus GN=Camk2b PE=1 SV=1	GAILTTMLATR
>	sp|P08413|KCC Calcium/calmodulin-dependent protein kinase type II subunit beta OS=Rattus norvegicus GN=Camk2b PE=1 SV=1	ICDPGLTSFEPEALGNLVEGMDFHR
>	sp|P08413|KCC Calcium/calmodulin-dependent protein kinase type II subunit beta OS=Rattus norvegicus GN=Camk2b PE=1 SV=1	LEAVLHCHQMGVVHR
>	sp|P08413|KCC Calcium/calmodulin-dependent protein kinase type II subunit beta OS=Rattus norvegicus GN=Camk2b PE=1 SV=1	LEAVLHCHQMGVVHR
>	sp|P08413|KCC Calcium/calmodulin-dependent protein kinase type II subunit beta OS=Rattus norvegicus GN=Camk2b PE=1 SV=1	LEAVLHCHQMGVVHR
>	sp|P08413|KCC Calcium/calmodulin-dependent protein kinase type II subunit beta OS=Rattus norvegicus GN=Camk2b PE=1 SV=1	LHDSISEEGFHYLVFDLVTGGELFEDIVAR
>	sp|P08413|KCC Calcium/calmodulin-dependent protein kinase type II subunit beta OS=Rattus norvegicus GN=Camk2b PE=1 SV=1	LKGAILTTMLATR
>	sp|P08413|KCC Calcium/calmodulin-dependent protein kinase type II subunit beta OS=Rattus norvegicus GN=Camk2b PE=1 SV=1	NLINQMLTINPAK
>	sp|P08413|KCC Calcium/calmodulin-dependent protein kinase type II subunit beta OS=Rattus norvegicus GN=Camk2b PE=1 SV=1	NLINQMLTINPAKR
>	sp|P08413|KCC Calcium/calmodulin-dependent protein kinase type II subunit beta OS=Rattus norvegicus GN=Camk2b PE=1 SV=1	NLINQMLTINPAKR
>	sp|P08413|KCC Calcium/calmodulin-dependent protein kinase type II subunit beta OS=Rattus norvegicus GN=Camk2b PE=1 SV=1	NLINQMLTINPAKR
>	sp|P08413|KCC Calcium/calmodulin-dependent protein kinase type II subunit beta OS=Rattus norvegicus GN=Camk2b PE=1 SV=1	VFDLVTGGELFEDIVAREYYSEADASHCIQQILEAVLHCHQMGVVHR
>	sp|P08413|KCC Calcium/calmodulin-dependent protein kinase type II subunit beta OS=Rattus norvegicus GN=Camk2b PE=1 SV=1	NLINQMLTINPAKR
>	sp|P08413|KCC Calcium/calmodulin-dependent protein kinase type II subunit beta OS=Rattus norvegicus GN=Camk2b PE=1 SV=1	EYYSEADASHCIQQILEAVLHCHQMGVVHR
>	sp|P08413|KCC Calcium/calmodulin-dependent protein kinase type II subunit beta OS=Rattus norvegicus GN=Camk2b PE=1 SV=1	EYYSEADASHCIQQILEAVLHCHQMGVVHR
>	sp|P08413|KCC Calcium/calmodulin-dependent protein kinase type II subunit beta OS=Rattus norvegicus GN=Camk2b PE=1 SV=1	ITAHEALK
>	sp|P08413|KCC Calcium/calmodulin-dependent protein kinase type II subunit beta OS=Rattus norvegicus GN=Camk2b PE=1 SV=1	ITAHEALK
>	sp|P08413|KCC Calcium/calmodulin-dependent protein kinase type II subunit beta OS=Rattus norvegicus GN=Camk2b PE=1 SV=1	AGAYDFPSPEWDTVTPEAK
>	sp|P08413|KCC Calcium/calmodulin-dependent protein kinase type II subunit beta OS=Rattus norvegicus GN=Camk2b PE=1 SV=1	DLKPENLLLASK
>	sp|P08413|KCC Calcium/calmodulin-dependent protein kinase type II subunit beta OS=Rattus norvegicus GN=Camk2b PE=1 SV=1	EYYSEADASHCIQQILEAVLHCHQMGVVHR
>	sp|P08413|KCC Calcium/calmodulin-dependent protein kinase type II subunit beta OS=Rattus norvegicus GN=Camk2b PE=1 SV=1	EYYSEADASHCIQQILEAVLHCHQMGVVHR
>	sp|P08413|KCC Calcium/calmodulin-dependent protein kinase type II subunit beta OS=Rattus norvegicus GN=Camk2b PE=1 SV=1	EYYSEADASHCIQQILEAVLHCHQMGVVHR
>	sp|P08413|KCC Calcium/calmodulin-dependent protein kinase type II subunit beta OS=Rattus norvegicus GN=Camk2b PE=1 SV=1	NLINQMLTINPAK
>	sp|P08413|KCC Calcium/calmodulin-dependent protein kinase type II subunit beta OS=Rattus norvegicus GN=Camk2b PE=1 SV=1	FYFENLLAK
>	sp|P08413|KCC Calcium/calmodulin-dependent protein kinase type II subunit beta OS=Rattus norvegicus GN=Camk2b PE=1 SV=1	LTQYIDGQGRPR
>	sp|P08413|KCC Calcium/calmodulin-dependent protein kinase type II subunit beta OS=Rattus norvegicus GN=Camk2b PE=1 SV=1	ITAHEALK
>	sp|P08413|KCC Calcium/calmodulin-dependent protein kinase type II subunit beta OS=Rattus norvegicus GN=Camk2b PE=1 SV=1	EYYSEADASHCIQQILEAVLHCHQMGVVHR
>	sp|P08413|KCC Calcium/calmodulin-dependent protein kinase type II subunit beta OS=Rattus norvegicus GN=Camk2b PE=1 SV=1	LTQYIDGQGRPR
>	sp|P08413|KCC Calcium/calmodulin-dependent protein kinase type II subunit beta OS=Rattus norvegicus GN=Camk2b PE=1 SV=1	EYYSEADASHCIQQILEAVLHCHQMGVVHR
>	sp|P08413|KCC Calcium/calmodulin-dependent protein kinase type II subunit beta OS=Rattus norvegicus GN=Camk2b PE=1 SV=1	GGELFEDIVAREYYSEADASHCIQQILEAVLHCHQMGVVHR
>	sp|P08413|KCC Calcium/calmodulin-dependent protein kinase type II subunit beta OS=Rattus norvegicus GN=Camk2b PE=1 SV=1	DLKPENLLLASK
>	sp|P08413|KCC Calcium/calmodulin-dependent protein kinase type II subunit beta OS=Rattus norvegicus GN=Camk2b PE=1 SV=1	EYYSEADASHCIQQILEAVLHCHQMGVVHR
>	sp|P08413|KCC Calcium/calmodulin-dependent protein kinase type II subunit beta OS=Rattus norvegicus GN=Camk2b PE=1 SV=1	GGELFEDIVAREYYSEADASHCIQQILEAVLHCHQMGVVHR
>	sp|P08413|KCC Calcium/calmodulin-dependent protein kinase type II subunit beta OS=Rattus norvegicus GN=Camk2b PE=1 SV=1	LTQYIDGQGRPR
>	sp|P08413|KCC Calcium/calmodulin-dependent protein kinase type II subunit beta OS=Rattus norvegicus GN=Camk2b PE=1 SV=1	EYYSEADASHCIQQILEAVLHCHQMGVVHR
>	sp|P08413|KCC Calcium/calmodulin-dependent protein kinase type II subunit beta OS=Rattus norvegicus GN=Camk2b PE=1 SV=1	EYYSEADASHCIQQILEAVLHCHQMGVVHR
>	sp|P08413|KCC Calcium/calmodulin-dependent protein kinase type II subunit beta OS=Rattus norvegicus GN=Camk2b PE=1 SV=1	TTEQLIEAVNNGDFEAYAK
>	sp|P08413|KCC Calcium/calmodulin-dependent protein kinase type II subunit beta OS=Rattus norvegicus GN=Camk2b PE=1 SV=1	FYFENLLAK
>	sp|P08413|KCC Calcium/calmodulin-dependent protein kinase type II subunit beta OS=Rattus norvegicus GN=Camk2b PE=1 SV=1	EYYSEADASHCIQQILEAVLHCHQMGVVHR
>	sp|P08413|KCC Calcium/calmodulin-dependent protein kinase type II subunit beta OS=Rattus norvegicus GN=Camk2b PE=1 SV=1	FYFENLLAK
>	sp|P08413|KCC Calcium/calmodulin-dependent protein kinase type II subunit beta OS=Rattus norvegicus GN=Camk2b PE=1 SV=1	KADGVKPQTNSTK
>	sp|P08413|KCC Calcium/calmodulin-dependent protein kinase type II subunit beta OS=Rattus norvegicus GN=Camk2b PE=1 SV=1	DLKPENLLLASK

>	sp|P08413|KCC Calcium/calmodulin-dependent protein kinase type II subunit beta OS=Rattus norvegicus GN=Camk2b PE=1 SV=1	DLKPENLLLASK
>	sp|P08413|KCC Calcium/calmodulin-dependent protein kinase type II subunit beta OS=Rattus norvegicus GN=Camk2b PE=1 SV=1	EYYSEADASHCIQQILEAVLHCHQMGVVHR
>	sp|P08413|KCC Calcium/calmodulin-dependent protein kinase type II subunit beta OS=Rattus norvegicus GN=Camk2b PE=1 SV=1	KADGVKPQTNSTK
>	sp|P08413|KCC Calcium/calmodulin-dependent protein kinase type II subunit beta OS=Rattus norvegicus GN=Camk2b PE=1 SV=1	DLKPENLLLASK
>	sp|P62804|H4_ Histone H4 OS=Rattus norvegicus GN=Hist1h4b PE=1 SV=2	DAVTYTEHAK
>	sp|P62804|H4_ Histone H4 OS=Rattus norvegicus GN=Hist1h4b PE=1 SV=2	TVTAMDVVYALK
>	sp|P62804|H4_ Histone H4 OS=Rattus norvegicus GN=Hist1h4b PE=1 SV=2	TVTAMDVVYALKR
>	sp|P62804|H4_ Histone H4 OS=Rattus norvegicus GN=Hist1h4b PE=1 SV=2	VFLENVIR
>	sp|P62804|H4_ Histone H4 OS=Rattus norvegicus GN=Hist1h4b PE=1 SV=2	KTVTAMDVVYALK
>	sp|P62804|H4_ Histone H4 OS=Rattus norvegicus GN=Hist1h4b PE=1 SV=2	DAVTYTEHAK
>	sp|P62804|H4_ Histone H4 OS=Rattus norvegicus GN=Hist1h4b PE=1 SV=2	KTVTAMDVVYALK
>	sp|P62804|H4_ Histone H4 OS=Rattus norvegicus GN=Hist1h4b PE=1 SV=2	KTVTAMDVVYALK
>	sp|P62804|H4_ Histone H4 OS=Rattus norvegicus GN=Hist1h4b PE=1 SV=2	KTVTAMDVVYALK
>	sp|P62804|H4_ Histone H4 OS=Rattus norvegicus GN=Hist1h4b PE=1 SV=2	KTVTAMDVVYALK
>	sp|P62804|H4_ Histone H4 OS=Rattus norvegicus GN=Hist1h4b PE=1 SV=2	TVTAMDVVYALK
>	sp|P62804|H4_ Histone H4 OS=Rattus norvegicus GN=Hist1h4b PE=1 SV=2	TVTAMDVVYALK
>	sp|P62804|H4_ Histone H4 OS=Rattus norvegicus GN=Hist1h4b PE=1 SV=2	TVTAMDVVYALK
>	sp|P62804|H4_ Histone H4 OS=Rattus norvegicus GN=Hist1h4b PE=1 SV=2	TVTAMDVVYALK
>	sp|P62804|H4_ Histone H4 OS=Rattus norvegicus GN=Hist1h4b PE=1 SV=2	TVTAMDVVYALKR
>	sp|P62804|H4_ Histone H4 OS=Rattus norvegicus GN=Hist1h4b PE=1 SV=2	TVTAMDVVYALKR
>	sp|P62804|H4_ Histone H4 OS=Rattus norvegicus GN=Hist1h4b PE=1 SV=2	TVTAMDVVYALKR
>	sp|P62804|H4_ Histone H4 OS=Rattus norvegicus GN=Hist1h4b PE=1 SV=2	TVTAMDVVYALKR
>	sp|P62804|H4_ Histone H4 OS=Rattus norvegicus GN=Hist1h4b PE=1 SV=2	VFLENVIR
>	sp|P62804|H4_ Histone H4 OS=Rattus norvegicus GN=Hist1h4b PE=1 SV=2	TVTAMDVVYALKR
>	sp|P62804|H4_ Histone H4 OS=Rattus norvegicus GN=Hist1h4b PE=1 SV=2	KTVTAMDVVYALK
>	sp|P62804|H4_ Histone H4 OS=Rattus norvegicus GN=Hist1h4b PE=1 SV=2	KTVTAMDVVYALK
>	sp|P62804|H4_ Histone H4 OS=Rattus norvegicus GN=Hist1h4b PE=1 SV=2	DAVTYTEHAK
>	sp|P62804|H4_ Histone H4 OS=Rattus norvegicus GN=Hist1h4b PE=1 SV=2	TVTAMDVVYALK
>	sp|P62804|H4_ Histone H4 OS=Rattus norvegicus GN=Hist1h4b PE=1 SV=2	DAVTYTEHAK
>	sp|P62804|H4_ Histone H4 OS=Rattus norvegicus GN=Hist1h4b PE=1 SV=2	ISGLIYEETR
>	sp|P62804|H4_ Histone H4 OS=Rattus norvegicus GN=Hist1h4b PE=1 SV=2	ISGLIYEETR
>	sp|P62804|H4_ Histone H4 OS=Rattus norvegicus GN=Hist1h4b PE=1 SV=2	TVTAMDVVYALKR
>	sp|P62804|H4_ Histone H4 OS=Rattus norvegicus GN=Hist1h4b PE=1 SV=2	DNIQGITKPAIR
>	sp|P62804|H4_ Histone H4 OS=Rattus norvegicus GN=Hist1h4b PE=1 SV=2	VFLENVIR
>	sp|Q62950|DP' Dihydropyrimidinase-related protein 1 OS=Rattus norvegicus GN=Crmp1 PE=1 SV=1	AALAGGTTMIIDHVVPEPGSSLLTSFEK
>	sp|Q62950|DP' Dihydropyrimidinase-related protein 1 OS=Rattus norvegicus GN=Crmp1 PE=1 SV=1	AVGKDNFTLIPEGVNGIEER
>	sp|Q62950|DP' Dihydropyrimidinase-related protein 1 OS=Rattus norvegicus GN=Crmp1 PE=1 SV=1	ILEMGITGPEGHALSRPEELEAEAVFR
>	sp|Q62950|DP' Dihydropyrimidinase-related protein 1 OS=Rattus norvegicus GN=Crmp1 PE=1 SV=1	VFGLHSVSR
>	sp|Q62950|DP' Dihydropyrimidinase-related protein 1 OS=Rattus norvegicus GN=Crmp1 PE=1 SV=1	IAVGSDADVVIWDPDKMK
>	sp|Q62950|DP' Dihydropyrimidinase-related protein 1 OS=Rattus norvegicus GN=Crmp1 PE=1 SV=1	AALAGGTTMIIDHVVPEPGSSLLTSFEK
>	sp|Q62950|DP' Dihydropyrimidinase-related protein 1 OS=Rattus norvegicus GN=Crmp1 PE=1 SV=1	AALAGGTTMIIDHVVPEPGSSLLTSFEK
>	sp|Q62950|DP' Dihydropyrimidinase-related protein 1 OS=Rattus norvegicus GN=Crmp1 PE=1 SV=1	GSPLVVISQGK
>	sp|Q62950|DP' Dihydropyrimidinase-related protein 1 OS=Rattus norvegicus GN=Crmp1 PE=1 SV=1	GSPLVVISQGK
>	sp|Q62950|DP' Dihydropyrimidinase-related protein 1 OS=Rattus norvegicus GN=Crmp1 PE=1 SV=1	ILEMGITGPEGHALSRPEELEAEAVFR
>	sp|Q62950|DP' Dihydropyrimidinase-related protein 1 OS=Rattus norvegicus GN=Crmp1 PE=1 SV=1	ILEMGITGPEGHALSRPEELEAEAVFR
>	sp|Q62950|DP' Dihydropyrimidinase-related protein 1 OS=Rattus norvegicus GN=Crmp1 PE=1 SV=1	ILEMGITGPEGHALSRPEELEAEAVFR
>	sp|Q62950|DP' Dihydropyrimidinase-related protein 1 OS=Rattus norvegicus GN=Crmp1 PE=1 SV=1	ILEMGITGPEGHALSRPEELEAEAVFR
>	sp|Q62950|DP' Dihydropyrimidinase-related protein 1 OS=Rattus norvegicus GN=Crmp1 PE=1 SV=1	ILEMGITGPEGHALSRPEELEAEAVFR
>	sp|Q62950|DP' Dihydropyrimidinase-related protein 1 OS=Rattus norvegicus GN=Crmp1 PE=1 SV=1	MDENQFVAVTSTNAAK
>	sp|Q62950|DP' Dihydropyrimidinase-related protein 1 OS=Rattus norvegicus GN=Crmp1 PE=1 SV=1	MDENQFVAVTSTNAAK
>	sp|Q62950|DP' Dihydropyrimidinase-related protein 1 OS=Rattus norvegicus GN=Crmp1 PE=1 SV=1	MDENQFVAVTSTNAAK
>	sp|Q62950|DP' Dihydropyrimidinase-related protein 1 OS=Rattus norvegicus GN=Crmp1 PE=1 SV=1	MDENQFVAVTSTNAAK
>	sp|Q62950|DP' Dihydropyrimidinase-related protein 1 OS=Rattus norvegicus GN=Crmp1 PE=1 SV=1	QIGENLIVPGGVK
>	sp|Q62950|DP' Dihydropyrimidinase-related protein 1 OS=Rattus norvegicus GN=Crmp1 PE=1 SV=1	QIGENLIVPGGVK
>	sp|Q62950|DP' Dihydropyrimidinase-related protein 1 OS=Rattus norvegicus GN=Crmp1 PE=1 SV=1	QIGENLIVPGGVK
>	sp|Q62950|DP' Dihydropyrimidinase-related protein 1 OS=Rattus norvegicus GN=Crmp1 PE=1 SV=1	AAAFVTSPPLSPDPTTPDYLTSLLACGDLQVTGSGHCPYSTAQKAVGK
>	sp|Q62950|DP' Dihydropyrimidinase-related protein 1 OS=Rattus norvegicus GN=Crmp1 PE=1 SV=1	QIGENLIVPGGVK
>	sp|Q62950|DP' Dihydropyrimidinase-related protein 1 OS=Rattus norvegicus GN=Crmp1 PE=1 SV=1	QIGENLIVPGGVK
>	sp|Q62950|DP' Dihydropyrimidinase-related protein 1 OS=Rattus norvegicus GN=Crmp1 PE=1 SV=1	GSPLVVISQGK
>	sp|Q62950|DP' Dihydropyrimidinase-related protein 1 OS=Rattus norvegicus GN=Crmp1 PE=1 SV=1	MDENQFVAVTSTNAAK
>	sp|Q62950|DP' Dihydropyrimidinase-related protein 1 OS=Rattus norvegicus GN=Crmp1 PE=1 SV=1	MDENQFVAVTSTNAAK
>	sp|Q62950|DP' Dihydropyrimidinase-related protein 1 OS=Rattus norvegicus GN=Crmp1 PE=1 SV=1	GSPLVVISQGK
>	sp|Q62950|DP' Dihydropyrimidinase-related protein 1 OS=Rattus norvegicus GN=Crmp1 PE=1 SV=1	IVAPPGGR
>	sp|Q62950|DP' Dihydropyrimidinase-related protein 1 OS=Rattus norvegicus GN=Crmp1 PE=1 SV=1	MVIPGGIDVNTYLQKPSQGMTSADDFFQGTR
>	sp|Q62950|DP' Dihydropyrimidinase-related protein 1 OS=Rattus norvegicus GN=Crmp1 PE=1 SV=1	IVAPPGGR
>	sp|Q62950|DP' Dihydropyrimidinase-related protein 1 OS=Rattus norvegicus GN=Crmp1 PE=1 SV=1	GSPLVVISQGK
>	sp|P31044|PEB Phosphatidylethanolamine-binding protein 1 OS=Rattus norvegicus GN=Pebp1 PE=1 SV=3	VLTPTQVMNRPSSISWDGLDPGKLYTLVLTDPDAPSR
>	sp|P31044|PEB Phosphatidylethanolamine-binding protein 1 OS=Rattus norvegicus GN=Pebp1 PE=1 SV=3	VLTPTQVMNRPSSISWDGLDPGKLYTLVLTDPDAPSRKDPK
>	sp|P31044|PEB Phosphatidylethanolamine-binding protein 1 OS=Rattus norvegicus GN=Pebp1 PE=1 SV=3	YVWLVYEQEQPLNCDEPILSNK
>	sp|P31044|PEB Phosphatidylethanolamine-binding protein 1 OS=Rattus norvegicus GN=Pebp1 PE=1 SV=3	VDYGGVTVDELGK
>	sp|P31044|PEB Phosphatidylethanolamine-binding protein 1 OS=Rattus norvegicus GN=Pebp1 PE=1 SV=3	VLTPTQVMNRPSSISWDGLDPGK
>	sp|P31044|PEB Phosphatidylethanolamine-binding protein 1 OS=Rattus norvegicus GN=Pebp1 PE=1 SV=3	KYHLGAPVAGTCFQAEWDDSVPK
>	sp|P31044|PEB Phosphatidylethanolamine-binding protein 1 OS=Rattus norvegicus GN=Pebp1 PE=1 SV=3	LYTLVLTDPDAPSR
>	sp|P31044|PEB Phosphatidylethanolamine-binding protein 1 OS=Rattus norvegicus GN=Pebp1 PE=1 SV=3	LYTLVLTDPDAPSR

>	sp|P31044|PEB	Phosphatidylethanolamine-binding protein 1 OS=Rattus norvegicus GN=Pebp1 PE=1 SV=3	LYTLVLTDPDAPSR
>	sp|P31044|PEB	Phosphatidylethanolamine-binding protein 1 OS=Rattus norvegicus GN=Pebp1 PE=1 SV=3	VDYGGVTVDELGK
>	sp|P31044|PEB	Phosphatidylethanolamine-binding protein 1 OS=Rattus norvegicus GN=Pebp1 PE=1 SV=3	VDYGGVTVDELGK
>	sp|P31044|PEB	Phosphatidylethanolamine-binding protein 1 OS=Rattus norvegicus GN=Pebp1 PE=1 SV=3	VDYGGVTVDELGK
>	sp|P31044|PEB	Phosphatidylethanolamine-binding protein 1 OS=Rattus norvegicus GN=Pebp1 PE=1 SV=3	VLTPTQVMNRPSSISWDGLDPGKLYTLVLTDPDAPSR
>	sp|P31044|PEB	Phosphatidylethanolamine-binding protein 1 OS=Rattus norvegicus GN=Pebp1 PE=1 SV=3	VLTPTQVMNRPSSISWDGLDPGKLYTLVLTDPDAPSR
>	sp|P31044|PEB	Phosphatidylethanolamine-binding protein 1 OS=Rattus norvegicus GN=Pebp1 PE=1 SV=3	VLTPTQVMNRPSSISWDGLDPGKLYTLVLTDPDAPSRKDPK
>	sp|P31044|PEB	Phosphatidylethanolamine-binding protein 1 OS=Rattus norvegicus GN=Pebp1 PE=1 SV=3	VLTPTQVMNRPSSISWDGLDPGKLYTLVLTDPDAPSRKDPK
>	sp|P31044|PEB	Phosphatidylethanolamine-binding protein 1 OS=Rattus norvegicus GN=Pebp1 PE=1 SV=3	VLTPTQVMNRPSSISWDGLDPGKLYTLVLTDPDAPSRKDPK
>	sp|P31044|PEB	Phosphatidylethanolamine-binding protein 1 OS=Rattus norvegicus GN=Pebp1 PE=1 SV=3	VLTPTQVMNRPSSISWDGLDPGKLYTLVLTDPDAPSRKDPK
>	sp|P31044|PEB	Phosphatidylethanolamine-binding protein 1 OS=Rattus norvegicus GN=Pebp1 PE=1 SV=3	YVWLVYEQEQPLNCDEPILSNK
>	sp|P31044|PEB	Phosphatidylethanolamine-binding protein 1 OS=Rattus norvegicus GN=Pebp1 PE=1 SV=3	LHDQLAGK
>	sp|P31044|PEB	Phosphatidylethanolamine-binding protein 1 OS=Rattus norvegicus GN=Pebp1 PE=1 SV=3	LYTLVLTDPDAPSR
>	sp|P31044|PEB	Phosphatidylethanolamine-binding protein 1 OS=Rattus norvegicus GN=Pebp1 PE=1 SV=3	VLTPTQVMNRPSSISWDGLDPGKLYTLVLTDPDAPSRKDPK
>	sp|P31044|PEB	Phosphatidylethanolamine-binding protein 1 OS=Rattus norvegicus GN=Pebp1 PE=1 SV=3	EWHHFLVVNMK
>	sp|P31044|PEB	Phosphatidylethanolamine-binding protein 1 OS=Rattus norvegicus GN=Pebp1 PE=1 SV=3	VLTPTQVMNRPSSISWDGLDPGKLYTLVLTDPDAPSRKDPK
>	sp|P31044|PEB	Phosphatidylethanolamine-binding protein 1 OS=Rattus norvegicus GN=Pebp1 PE=1 SV=3	VDYGGVTVDELGK
>	sp|P31044|PEB	Phosphatidylethanolamine-binding protein 1 OS=Rattus norvegicus GN=Pebp1 PE=1 SV=3	YVWLVYEQEQPLNCDEPILSNK
>	sp|P31044|PEB	Phosphatidylethanolamine-binding protein 1 OS=Rattus norvegicus GN=Pebp1 PE=1 SV=3	VDYGGVTVDELGK
>	sp|P31044|PEB	Phosphatidylethanolamine-binding protein 1 OS=Rattus norvegicus GN=Pebp1 PE=1 SV=3	FREWHHFLVVNMK
>	sp|P31044|PEB	Phosphatidylethanolamine-binding protein 1 OS=Rattus norvegicus GN=Pebp1 PE=1 SV=3	VLTPTQVMNRPSSISWDGLDPGKLYTLVLTDPDAPSR
>	sp|P31044|PEB	Phosphatidylethanolamine-binding protein 1 OS=Rattus norvegicus GN=Pebp1 PE=1 SV=3	FKVESFR
>	sp|P31044|PEB	Phosphatidylethanolamine-binding protein 1 OS=Rattus norvegicus GN=Pebp1 PE=1 SV=3	GNDISSGTVLSEYVGSGPPKDTGLHR
>	sp|P31044|PEB	Phosphatidylethanolamine-binding protein 1 OS=Rattus norvegicus GN=Pebp1 PE=1 SV=3	LHDQLAGK
>	sp|P31044|PEB	Phosphatidylethanolamine-binding protein 1 OS=Rattus norvegicus GN=Pebp1 PE=1 SV=3	LYTLVLTDPDAPSR
>	sp|P31044|PEB	Phosphatidylethanolamine-binding protein 1 OS=Rattus norvegicus GN=Pebp1 PE=1 SV=3	LYTLVLTDPDAPSR
>	sp|P31044|PEB	Phosphatidylethanolamine-binding protein 1 OS=Rattus norvegicus GN=Pebp1 PE=1 SV=3	VLTPTQVMNRPSSISWDGLDPGKLYTLVLTDPDAPSR
>	sp|P31044|PEB	Phosphatidylethanolamine-binding protein 1 OS=Rattus norvegicus GN=Pebp1 PE=1 SV=3	LHDQLAGK
>	sp|P31044|PEB	Phosphatidylethanolamine-binding protein 1 OS=Rattus norvegicus GN=Pebp1 PE=1 SV=3	VLTPTQVMNRPSSISWDGLDPGKLYTLVLTDPDAPSR
>	sp|P48721|GRP	Stress-70 protein, mitochondrial OS=Rattus norvegicus GN=Hspa9 PE=1 SV=3	AQFEGIVTDLIK
>	sp|P48721|GRP	Stress-70 protein, mitochondrial OS=Rattus norvegicus GN=Hspa9 PE=1 SV=3	AQFEGIVTDLIKR
>	sp|P48721|GRP	Stress-70 protein, mitochondrial OS=Rattus norvegicus GN=Hspa9 PE=1 SV=3	EGSGSSSTGEQKEDQKEEKQ
>	sp|P48721|GRP	Stress-70 protein, mitochondrial OS=Rattus norvegicus GN=Hspa9 PE=1 SV=3	KDSETGENIR
>	sp|P48721|GRP	Stress-70 protein, mitochondrial OS=Rattus norvegicus GN=Hspa9 PE=1 SV=3	AQFEGIVTDLIK
>	sp|P48721|GRP	Stress-70 protein, mitochondrial OS=Rattus norvegicus GN=Hspa9 PE=1 SV=3	AQFEGIVTDLIK
>	sp|P48721|GRP	Stress-70 protein, mitochondrial OS=Rattus norvegicus GN=Hspa9 PE=1 SV=3	EGSGSSSTGEQKEDQKEEKQ
>	sp|P48721|GRP	Stress-70 protein, mitochondrial OS=Rattus norvegicus GN=Hspa9 PE=1 SV=3	EQQIVIQSSGGLSKDDIENMVK
>	sp|P48721|GRP	Stress-70 protein, mitochondrial OS=Rattus norvegicus GN=Hspa9 PE=1 SV=3	KDSETGENIR
>	sp|P13086|SUC	Succinyl-CoA ligase [GDP-forming] subunit alpha, mitochondrial OS=Rattus norvegicus GN=Suclg1 PE=2 SV=2	AKPVVSFIAGITAPPGR
>	sp|P13086|SUC	Succinyl-CoA ligase [GDP-forming] subunit alpha, mitochondrial OS=Rattus norvegicus GN=Suclg1 PE=2 SV=2	AKPVVSFIAGITAPPGRR
>	sp|P13086|SUC	Succinyl-CoA ligase [GDP-forming] subunit alpha, mitochondrial OS=Rattus norvegicus GN=Suclg1 PE=2 SV=2	LIGPNCPGIINPGECK
>	sp|P13086|SUC	Succinyl-CoA ligase [GDP-forming] subunit alpha, mitochondrial OS=Rattus norvegicus GN=Suclg1 PE=2 SV=2	KHLGLPVFNTVK
>	sp|P13086|SUC	Succinyl-CoA ligase [GDP-forming] subunit alpha, mitochondrial OS=Rattus norvegicus GN=Suclg1 PE=2 SV=2	MGHAGAIIAGGK
>	sp|P13086|SUC	Succinyl-CoA ligase [GDP-forming] subunit alpha, mitochondrial OS=Rattus norvegicus GN=Suclg1 PE=2 SV=2	AKPVVSFIAGITAPPGR
>	sp|P13086|SUC	Succinyl-CoA ligase [GDP-forming] subunit alpha, mitochondrial OS=Rattus norvegicus GN=Suclg1 PE=2 SV=2	AKPVVSFIAGITAPPGR
>	sp|P13086|SUC	Succinyl-CoA ligase [GDP-forming] subunit alpha, mitochondrial OS=Rattus norvegicus GN=Suclg1 PE=2 SV=2	AKPVVSFIAGITAPPGR
>	sp|P13086|SUC	Succinyl-CoA ligase [GDP-forming] subunit alpha, mitochondrial OS=Rattus norvegicus GN=Suclg1 PE=2 SV=2	AKPVVSFIAGITAPPGR
>	sp|P13086|SUC	Succinyl-CoA ligase [GDP-forming] subunit alpha, mitochondrial OS=Rattus norvegicus GN=Suclg1 PE=2 SV=2	AKPVVSFIAGITAPPGR
>	sp|P13086|SUC	Succinyl-CoA ligase [GDP-forming] subunit alpha, mitochondrial OS=Rattus norvegicus GN=Suclg1 PE=2 SV=2	AKPVVSFIAGITAPPGR
>	sp|P13086|SUC	Succinyl-CoA ligase [GDP-forming] subunit alpha, mitochondrial OS=Rattus norvegicus GN=Suclg1 PE=2 SV=2	AKPVVSFIAGITAPPGR
>	sp|P13086|SUC	Succinyl-CoA ligase [GDP-forming] subunit alpha, mitochondrial OS=Rattus norvegicus GN=Suclg1 PE=2 SV=2	KHLGLPVFNTVK
>	sp|P13086|SUC	Succinyl-CoA ligase [GDP-forming] subunit alpha, mitochondrial OS=Rattus norvegicus GN=Suclg1 PE=2 SV=2	VICQGFTGK
>	sp|P13086|SUC	Succinyl-CoA ligase [GDP-forming] subunit alpha, mitochondrial OS=Rattus norvegicus GN=Suclg1 PE=2 SV=2	IGIMPGHIHK
>	sp|P13086|SUC	Succinyl-CoA ligase [GDP-forming] subunit alpha, mitochondrial OS=Rattus norvegicus GN=Suclg1 PE=2 SV=2	MGHAGAIIAGGK
>	sp|Q3KR86|IMM	Mitochondrial inner membrane protein (Fragment) OS=Rattus norvegicus GN=Immt PE=1 SV=1	GIEQAVQSHAVAEEEAR
>	sp|Q3KR86|IMM	Mitochondrial inner membrane protein (Fragment) OS=Rattus norvegicus GN=Immt PE=1 SV=1	KAHQLWLSVEALK
>	sp|Q3KR86|IMM	Mitochondrial inner membrane protein (Fragment) OS=Rattus norvegicus GN=Immt PE=1 SV=1	LSEQELEFHR
>	sp|Q3KR86|IMM	Mitochondrial inner membrane protein (Fragment) OS=Rattus norvegicus GN=Immt PE=1 SV=1	QHIELALER
>	sp|Q3KR86|IMM	Mitochondrial inner membrane protein (Fragment) OS=Rattus norvegicus GN=Immt PE=1 SV=1	GMSISDLAGTLSTDDLNALIAHAHR
>	sp|Q3KR86|IMM	Mitochondrial inner membrane protein (Fragment) OS=Rattus norvegicus GN=Immt PE=1 SV=1	GIEQAVQSHAVAEEEAR
>	sp|Q3KR86|IMM	Mitochondrial inner membrane protein (Fragment) OS=Rattus norvegicus GN=Immt PE=1 SV=1	GMSISDLAGTLSTDDLNALIAHAHR
>	sp|Q3KR86|IMM	Mitochondrial inner membrane protein (Fragment) OS=Rattus norvegicus GN=Immt PE=1 SV=1	QHIELALER
>	sp|Q3KR86|IMM	Mitochondrial inner membrane protein (Fragment) OS=Rattus norvegicus GN=Immt PE=1 SV=1	QHIELALER
>	sp|P04631|S10	Protein S100-B OS=Rattus norvegicus GN=S100b PE=1 SV=2	AMVALIDVFHQYSGR
>	sp|P04631|S10	Protein S100-B OS=Rattus norvegicus GN=S100b PE=1 SV=2	ELINNELSHFLEEIKEQEVVDK
>	sp|P04631|S10	Protein S100-B OS=Rattus norvegicus GN=S100b PE=1 SV=2	SELKELINNELSHFLEEIKEQEVVDK
>	sp|P04631|S10	Protein S100-B OS=Rattus norvegicus GN=S100b PE=1 SV=2	ELINNELSHFLEEIK
>	sp|P04631|S10	Protein S100-B OS=Rattus norvegicus GN=S100b PE=1 SV=2	EGDKHK
>	sp|P04631|S10	Protein S100-B OS=Rattus norvegicus GN=S100b PE=1 SV=2	AMVALIDVFHQYSGR
>	sp|P04631|S10	Protein S100-B OS=Rattus norvegicus GN=S100b PE=1 SV=2	AMVALIDVFHQYSGR
>	sp|P04631|S10	Protein S100-B OS=Rattus norvegicus GN=S100b PE=1 SV=2	AMVALIDVFHQYSGR
>	sp|P04631|S10	Protein S100-B OS=Rattus norvegicus GN=S100b PE=1 SV=2	AMVALIDVFHQYSGR
>	sp|P04631|S10	Protein S100-B OS=Rattus norvegicus GN=S100b PE=1 SV=2	AMVALIDVFHQYSGR
>	sp|P04631|S10	Protein S100-B OS=Rattus norvegicus GN=S100b PE=1 SV=2	AMVALIDVFHQYSGR

>	sp|P04631|S10	Protein S100-B OS=Rattus norvegicus GN=S100b PE=1 SV=2	AMVALIDVFHQYSGR
>	sp|P04631|S10	Protein S100-B OS=Rattus norvegicus GN=S100b PE=1 SV=2	AMVALIDVFHQYSGR
>	sp|P04631|S10	Protein S100-B OS=Rattus norvegicus GN=S100b PE=1 SV=2	AMVALIDVFHQYSGR
>	sp|P04631|S10	Protein S100-B OS=Rattus norvegicus GN=S100b PE=1 SV=2	AMVALIDVFHQYSGR
>	sp|P04631|S10	Protein S100-B OS=Rattus norvegicus GN=S100b PE=1 SV=2	AMVALIDVFHQYSGR
>	sp|P04631|S10	Protein S100-B OS=Rattus norvegicus GN=S100b PE=1 SV=2	AMVALIDVFHQYSGR
>	sp|P04631|S10	Protein S100-B OS=Rattus norvegicus GN=S100b PE=1 SV=2	AMVALIDVFHQYSGR
>	sp|P04631|S10	Protein S100-B OS=Rattus norvegicus GN=S100b PE=1 SV=2	AMVALIDVFHQYSGR
>	sp|P04631|S10	Protein S100-B OS=Rattus norvegicus GN=S100b PE=1 SV=2	ELINNELSHFLEEIKEQEVVDK
>	sp|P04631|S10	Protein S100-B OS=Rattus norvegicus GN=S100b PE=1 SV=2	ELINNELSHFLEEIKEQEVVDK
>	sp|P04631|S10	Protein S100-B OS=Rattus norvegicus GN=S100b PE=1 SV=2	SELKELINNELSHFLEEIKEQEVVDK
>	sp|P04631|S10	Protein S100-B OS=Rattus norvegicus GN=S100b PE=1 SV=2	SELKELINNELSHFLEEIKEQEVVDK
>	sp|P04631|S10	Protein S100-B OS=Rattus norvegicus GN=S100b PE=1 SV=2	ELINNELSHFLEEIKEQEVVDK
>	sp|P04631|S10	Protein S100-B OS=Rattus norvegicus GN=S100b PE=1 SV=2	EGDKHK
>	sp|Q6P9T8|TBI	Tubulin beta-2C chain OS=Rattus norvegicus GN=Tubb2c PE=1 SV=1	AVLVDLEPGTMDSVR
>	sp|Q6P9T8|TBI	Tubulin beta-2C chain OS=Rattus norvegicus GN=Tubb2c PE=1 SV=1	FWEVISDEHGIDPTGTYHGDSDLQLER
>	sp|Q6P9T8|TBI	Tubulin beta-2C chain OS=Rattus norvegicus GN=Tubb2c PE=1 SV=1	KEAESCDCLQGFQLTHSLGGGTGSGMGTLLISK
>	sp|Q6P9T8|TBI	Tubulin beta-2C chain OS=Rattus norvegicus GN=Tubb2c PE=1 SV=1	VSDTVVEPYNATLSVHQLVENTDETYCIDNEALYDICFR
>	sp|Q6P9T8|TBI	Tubulin beta-2C chain OS=Rattus norvegicus GN=Tubb2c PE=1 SV=1	YLTVAAVFR
>	sp|Q6P9T8|TBI	Tubulin beta-2C chain OS=Rattus norvegicus GN=Tubb2c PE=1 SV=1	ALTVPELTQQMFDAK
>	sp|Q6P9T8|TBI	Tubulin beta-2C chain OS=Rattus norvegicus GN=Tubb2c PE=1 SV=1	ALTVPELTQQMFDAK
>	sp|Q6P9T8|TBI	Tubulin beta-2C chain OS=Rattus norvegicus GN=Tubb2c PE=1 SV=1	ALTVPELTQQMFDAK
>	sp|Q6P9T8|TBI	Tubulin beta-2C chain OS=Rattus norvegicus GN=Tubb2c PE=1 SV=1	ALTVPELTQQMFDAK
>	sp|Q6P9T8|TBI	Tubulin beta-2C chain OS=Rattus norvegicus GN=Tubb2c PE=1 SV=1	ALTVPELTQQMFDAK
>	sp|Q6P9T8|TBI	Tubulin beta-2C chain OS=Rattus norvegicus GN=Tubb2c PE=1 SV=1	ALTVPELTQQMFDAK
>	sp|Q6P9T8|TBI	Tubulin beta-2C chain OS=Rattus norvegicus GN=Tubb2c PE=1 SV=1	AVLVDLEPGTMDSVR
>	sp|Q6P9T8|TBI	Tubulin beta-2C chain OS=Rattus norvegicus GN=Tubb2c PE=1 SV=1	AVLVDLEPGTMDSVR
>	sp|Q6P9T8|TBI	Tubulin beta-2C chain OS=Rattus norvegicus GN=Tubb2c PE=1 SV=1	EIVHLQAGQCGNQIGAK
>	sp|Q6P9T8|TBI	Tubulin beta-2C chain OS=Rattus norvegicus GN=Tubb2c PE=1 SV=1	EIVHLQAGQCGNQIGAK
>	sp|Q6P9T8|TBI	Tubulin beta-2C chain OS=Rattus norvegicus GN=Tubb2c PE=1 SV=1	EIVHLQAGQCGNQIGAK
>	sp|Q6P9T8|TBI	Tubulin beta-2C chain OS=Rattus norvegicus GN=Tubb2c PE=1 SV=1	EIVHLQAGQCGNQIGAK
>	sp|Q6P9T8|TBI	Tubulin beta-2C chain OS=Rattus norvegicus GN=Tubb2c PE=1 SV=1	EIVHLQAGQCGNQIGAK
>	sp|Q6P9T8|TBI	Tubulin beta-2C chain OS=Rattus norvegicus GN=Tubb2c PE=1 SV=1	EIVHLQAGQCGNQIGAK
>	sp|Q6P9T8|TBI	Tubulin beta-2C chain OS=Rattus norvegicus GN=Tubb2c PE=1 SV=1	EIVHLQAGQCGNQIGAK
>	sp|Q6P9T8|TBI	Tubulin beta-2C chain OS=Rattus norvegicus GN=Tubb2c PE=1 SV=1	EIVHLQAGQCGNQIGAK
>	sp|Q6P9T8|TBI	Tubulin beta-2C chain OS=Rattus norvegicus GN=Tubb2c PE=1 SV=1	EIVHLQAGQCGNQIGAK
>	sp|Q6P9T8|TBI	Tubulin beta-2C chain OS=Rattus norvegicus GN=Tubb2c PE=1 SV=1	EIVHLQAGQCGNQIGAK
>	sp|Q6P9T8|TBI	Tubulin beta-2C chain OS=Rattus norvegicus GN=Tubb2c PE=1 SV=1	EIVHLQAGQCGNQIGAK
>	sp|Q6P9T8|TBI	Tubulin beta-2C chain OS=Rattus norvegicus GN=Tubb2c PE=1 SV=1	EIVHLQAGQCGNQIGAK
>	sp|Q6P9T8|TBI	Tubulin beta-2C chain OS=Rattus norvegicus GN=Tubb2c PE=1 SV=1	EIVHLQAGQCGNQIGAK
>	sp|Q6P9T8|TBI	Tubulin beta-2C chain OS=Rattus norvegicus GN=Tubb2c PE=1 SV=1	EIVHLQAGQCGNQIGAK
>	sp|Q6P9T8|TBI	Tubulin beta-2C chain OS=Rattus norvegicus GN=Tubb2c PE=1 SV=1	EIVHLQAGQCGNQIGAK
>	sp|Q6P9T8|TBI	Tubulin beta-2C chain OS=Rattus norvegicus GN=Tubb2c PE=1 SV=1	EIVHLQAGQCGNQIGAK
>	sp|Q6P9T8|TBI	Tubulin beta-2C chain OS=Rattus norvegicus GN=Tubb2c PE=1 SV=1	EIVHLQAGQCGNQIGAK
>	sp|Q6P9T8|TBI	Tubulin beta-2C chain OS=Rattus norvegicus GN=Tubb2c PE=1 SV=1	EIVHLQAGQCGNQIGAK
>	sp|Q6P9T8|TBI	Tubulin beta-2C chain OS=Rattus norvegicus GN=Tubb2c PE=1 SV=1	EIVHLQAGQCGNQIGAK
>	sp|Q6P9T8|TBI	Tubulin beta-2C chain OS=Rattus norvegicus GN=Tubb2c PE=1 SV=1	EIVHLQAGQCGNQIGAK
>	sp|Q6P9T8|TBI	Tubulin beta-2C chain OS=Rattus norvegicus GN=Tubb2c PE=1 SV=1	EIVHLQAGQCGNQIGAK
>	sp|Q6P9T8|TBI	Tubulin beta-2C chain OS=Rattus norvegicus GN=Tubb2c PE=1 SV=1	EIVHLQAGQCGNQIGAK
>	sp|Q6P9T8|TBI	Tubulin beta-2C chain OS=Rattus norvegicus GN=Tubb2c PE=1 SV=1	EIVHLQAGQCGNQIGAK
>	sp|Q6P9T8|TBI	Tubulin beta-2C chain OS=Rattus norvegicus GN=Tubb2c PE=1 SV=1	EIVHLQAGQCGNQIGAK
>	sp|Q6P9T8|TBI	Tubulin beta-2C chain OS=Rattus norvegicus GN=Tubb2c PE=1 SV=1	EVDEQMLNVQNK
>	sp|Q6P9T8|TBI	Tubulin beta-2C chain OS=Rattus norvegicus GN=Tubb2c PE=1 SV=1	EVDEQMLNVQNK
>	sp|Q6P9T8|TBI	Tubulin beta-2C chain OS=Rattus norvegicus GN=Tubb2c PE=1 SV=1	EVDEQMLNVQNK
>	sp|Q6P9T8|TBI	Tubulin beta-2C chain OS=Rattus norvegicus GN=Tubb2c PE=1 SV=1	EVDEQMLNVQNK
>	sp|Q6P9T8|TBI	Tubulin beta-2C chain OS=Rattus norvegicus GN=Tubb2c PE=1 SV=1	FPGQLNADLR
>	sp|Q6P9T8|TBI	Tubulin beta-2C chain OS=Rattus norvegicus GN=Tubb2c PE=1 SV=1	FPGQLNADLR
>	sp|Q6P9T8|TBI	Tubulin beta-2C chain OS=Rattus norvegicus GN=Tubb2c PE=1 SV=1	FPGQLNADLR
>	sp|Q6P9T8|TBI	Tubulin beta-2C chain OS=Rattus norvegicus GN=Tubb2c PE=1 SV=1	FPGQLNADLR
>	sp|Q6P9T8|TBI	Tubulin beta-2C chain OS=Rattus norvegicus GN=Tubb2c PE=1 SV=1	FPGQLNADLR
>	sp|Q6P9T8|TBI	Tubulin beta-2C chain OS=Rattus norvegicus GN=Tubb2c PE=1 SV=1	FPGQLNADLR
>	sp|Q6P9T8|TBI	Tubulin beta-2C chain OS=Rattus norvegicus GN=Tubb2c PE=1 SV=1	FPGQLNADLRK
>	sp|Q6P9T8|TBI	Tubulin beta-2C chain OS=Rattus norvegicus GN=Tubb2c PE=1 SV=1	FWEVISDEHGIDPTGTYHGDSDLQLER
>	sp|Q6P9T8|TBI	Tubulin beta-2C chain OS=Rattus norvegicus GN=Tubb2c PE=1 SV=1	FWEVISDEHGIDPTGTYHGDSDLQLER
>	sp|Q6P9T8|TBI	Tubulin beta-2C chain OS=Rattus norvegicus GN=Tubb2c PE=1 SV=1	FWEVISDEHGIDPTGTYHGDSDLQLER
>	sp|Q6P9T8|TBI	Tubulin beta-2C chain OS=Rattus norvegicus GN=Tubb2c PE=1 SV=1	FWEVISDEHGIDPTGTYHGDSDLQLER
>	sp|Q6P9T8|TBI	Tubulin beta-2C chain OS=Rattus norvegicus GN=Tubb2c PE=1 SV=1	FWEVISDEHGIDPTGTYHGDSDLQLER
>	sp|Q6P9T8|TBI	Tubulin beta-2C chain OS=Rattus norvegicus GN=Tubb2c PE=1 SV=1	GHYTEGAELVDSVLDVVR
>	sp|Q6P9T8|TBI	Tubulin beta-2C chain OS=Rattus norvegicus GN=Tubb2c PE=1 SV=1	GHYTEGAELVDSVLDVVR
>	sp|Q6P9T8|TBI	Tubulin beta-2C chain OS=Rattus norvegicus GN=Tubb2c PE=1 SV=1	GHYTEGAELVDSVLDVVR
>	sp|Q6P9T8|TBI	Tubulin beta-2C chain OS=Rattus norvegicus GN=Tubb2c PE=1 SV=1	GHYTEGAELVDSVLDVVR
>	sp|Q6P9T8|TBI	Tubulin beta-2C chain OS=Rattus norvegicus GN=Tubb2c PE=1 SV=1	GHYTEGAELVDSVLDVVR
>	sp|Q6P9T8|TBI	Tubulin beta-2C chain OS=Rattus norvegicus GN=Tubb2c PE=1 SV=1	GHYTEGAELVDSVLDVVR
>	sp|Q6P9T8|TBI	Tubulin beta-2C chain OS=Rattus norvegicus GN=Tubb2c PE=1 SV=1	GHYTEGAELVDSVLDVVR

>	sp|Q6P9T8|TB	Tubulin beta-2C chain OS=Rattus norvegicus GN=Tubb2c PE=1 SV=1	GHYTEGAELVDSVLDVVR
>	sp|Q6P9T8|TB	Tubulin beta-2C chain OS=Rattus norvegicus GN=Tubb2c PE=1 SV=1	GHYTEGAELVDSVLDVVR
>	sp|Q6P9T8|TB	Tubulin beta-2C chain OS=Rattus norvegicus GN=Tubb2c PE=1 SV=1	GHYTEGAELVDSVLDVVR
>	sp|Q6P9T8|TB	Tubulin beta-2C chain OS=Rattus norvegicus GN=Tubb2c PE=1 SV=1	GHYTEGAELVDSVLDVVR
>	sp|Q6P9T8|TB	Tubulin beta-2C chain OS=Rattus norvegicus GN=Tubb2c PE=1 SV=1	GHYTEGAELVDSVLDVVR
>	sp|Q6P9T8|TB	Tubulin beta-2C chain OS=Rattus norvegicus GN=Tubb2c PE=1 SV=1	GHYTEGAELVDSVLDVVR
>	sp|Q6P9T8|TB	Tubulin beta-2C chain OS=Rattus norvegicus GN=Tubb2c PE=1 SV=1	GHYTEGAELVDSVLDVVR
>	sp|Q6P9T8|TB	Tubulin beta-2C chain OS=Rattus norvegicus GN=Tubb2c PE=1 SV=1	GHYTEGAELVDSVLDVVR
>	sp|Q6P9T8|TB	Tubulin beta-2C chain OS=Rattus norvegicus GN=Tubb2c PE=1 SV=1	GHYTEGAELVDSVLDVVR
>	sp|Q6P9T8|TB	Tubulin beta-2C chain OS=Rattus norvegicus GN=Tubb2c PE=1 SV=1	GHYTEGAELVDSVLDVVR
>	sp|Q6P9T8|TB	Tubulin beta-2C chain OS=Rattus norvegicus GN=Tubb2c PE=1 SV=1	GHYTEGAELVDSVLDVVR
>	sp|Q6P9T8|TB	Tubulin beta-2C chain OS=Rattus norvegicus GN=Tubb2c PE=1 SV=1	GHYTEGAELVDSVLDVVR
>	sp|Q6P9T8|TB	Tubulin beta-2C chain OS=Rattus norvegicus GN=Tubb2c PE=1 SV=1	GHYTEGAELVDSVLDVVR
>	sp|Q6P9T8|TB	Tubulin beta-2C chain OS=Rattus norvegicus GN=Tubb2c PE=1 SV=1	GHYTEGAELVDSVLDVVR
>	sp|Q6P9T8|TB	Tubulin beta-2C chain OS=Rattus norvegicus GN=Tubb2c PE=1 SV=1	GHYTEGAELVDSVLDVVR
>	sp|Q6P9T8|TB	Tubulin beta-2C chain OS=Rattus norvegicus GN=Tubb2c PE=1 SV=1	GHYTEGAELVDSVLDVVR
>	sp|Q6P9T8|TB	Tubulin beta-2C chain OS=Rattus norvegicus GN=Tubb2c PE=1 SV=1	GHYTEGAELVDSVLDVVR
>	sp|Q6P9T8|TB	Tubulin beta-2C chain OS=Rattus norvegicus GN=Tubb2c PE=1 SV=1	GHYTEGAELVDSVLDVVR
>	sp|Q6P9T8|TB	Tubulin beta-2C chain OS=Rattus norvegicus GN=Tubb2c PE=1 SV=1	GHYTEGAELVDSVLDVVR
>	sp|Q6P9T8|TB	Tubulin beta-2C chain OS=Rattus norvegicus GN=Tubb2c PE=1 SV=1	GHYTEGAELVDSVLDVVR
>	sp|Q6P9T8|TB	Tubulin beta-2C chain OS=Rattus norvegicus GN=Tubb2c PE=1 SV=1	GHYTEGAELVDSVLDVVR
>	sp|Q6P9T8|TB	Tubulin beta-2C chain OS=Rattus norvegicus GN=Tubb2c PE=1 SV=1	GHYTEGAELVDSVLDVVR
>	sp|Q6P9T8|TB	Tubulin beta-2C chain OS=Rattus norvegicus GN=Tubb2c PE=1 SV=1	GHYTEGAELVDSVLDVVR
>	sp|Q6P9T8|TB	Tubulin beta-2C chain OS=Rattus norvegicus GN=Tubb2c PE=1 SV=1	GHYTEGAELVDSVLDVVR
>	sp|Q6P9T8|TB	Tubulin beta-2C chain OS=Rattus norvegicus GN=Tubb2c PE=1 SV=1	GHYTEGAELVDSVLDVVR
>	sp|Q6P9T8|TB	Tubulin beta-2C chain OS=Rattus norvegicus GN=Tubb2c PE=1 SV=1	GHYTEGAELVDSVLDVVRK
>	sp|Q6P9T8|TB	Tubulin beta-2C chain OS=Rattus norvegicus GN=Tubb2c PE=1 SV=1	IMNTFSVVPSPK
>	sp|Q6P9T8|TB	Tubulin beta-2C chain OS=Rattus norvegicus GN=Tubb2c PE=1 SV=1	IMNTFSVVPSPK
>	sp|Q6P9T8|TB	Tubulin beta-2C chain OS=Rattus norvegicus GN=Tubb2c PE=1 SV=1	IMNTFSVVPSPK
>	sp|Q6P9T8|TB	Tubulin beta-2C chain OS=Rattus norvegicus GN=Tubb2c PE=1 SV=1	IREEYPDR
>	sp|Q6P9T8|TB	Tubulin beta-2C chain OS=Rattus norvegicus GN=Tubb2c PE=1 SV=1	KEAESCDCLQGFQLTHSLGGGTGSGMGTLLISK
>	sp|Q6P9T8|TB	Tubulin beta-2C chain OS=Rattus norvegicus GN=Tubb2c PE=1 SV=1	KEAESCDCLQGFQLTHSLGGGTGSGMGTLLISK
>	sp|Q6P9T8|TB	Tubulin beta-2C chain OS=Rattus norvegicus GN=Tubb2c PE=1 SV=1	KEAESCDCLQGFQLTHSLGGGTGSGMGTLLISK
>	sp|Q6P9T8|TB	Tubulin beta-2C chain OS=Rattus norvegicus GN=Tubb2c PE=1 SV=1	KEAESCDCLQGFQLTHSLGGGTGSGMGTLLISK
>	sp|Q6P9T8|TB	Tubulin beta-2C chain OS=Rattus norvegicus GN=Tubb2c PE=1 SV=1	KLAVNMVPFPR
>	sp|Q6P9T8|TB	Tubulin beta-2C chain OS=Rattus norvegicus GN=Tubb2c PE=1 SV=1	KLAVNMVPFPR
>	sp|Q6P9T8|TB	Tubulin beta-2C chain OS=Rattus norvegicus GN=Tubb2c PE=1 SV=1	KLAVNMVPFPR
>	sp|Q6P9T8|TB	Tubulin beta-2C chain OS=Rattus norvegicus GN=Tubb2c PE=1 SV=1	KLAVNMVPFPR
>	sp|Q6P9T8|TB	Tubulin beta-2C chain OS=Rattus norvegicus GN=Tubb2c PE=1 SV=1	KLAVNMVPFPR
>	sp|Q6P9T8|TB	Tubulin beta-2C chain OS=Rattus norvegicus GN=Tubb2c PE=1 SV=1	KLAVNMVPFPR
>	sp|Q6P9T8|TB	Tubulin beta-2C chain OS=Rattus norvegicus GN=Tubb2c PE=1 SV=1	KLAVNMVPFPR
>	sp|Q6P9T8|TB	Tubulin beta-2C chain OS=Rattus norvegicus GN=Tubb2c PE=1 SV=1	KLAVNMVPFPR
>	sp|Q6P9T8|TB	Tubulin beta-2C chain OS=Rattus norvegicus GN=Tubb2c PE=1 SV=1	KLAVNMVPFPR
>	sp|Q6P9T8|TB	Tubulin beta-2C chain OS=Rattus norvegicus GN=Tubb2c PE=1 SV=1	KLAVNMVPFPR
>	sp|Q6P9T8|TB	Tubulin beta-2C chain OS=Rattus norvegicus GN=Tubb2c PE=1 SV=1	KLAVNMVPFPR
>	sp|Q6P9T8|TB	Tubulin beta-2C chain OS=Rattus norvegicus GN=Tubb2c PE=1 SV=1	KLAVNMVPFPR
>	sp|Q6P9T8|TB	Tubulin beta-2C chain OS=Rattus norvegicus GN=Tubb2c PE=1 SV=1	KLAVNMVPFPR
>	sp|Q6P9T8|TB	Tubulin beta-2C chain OS=Rattus norvegicus GN=Tubb2c PE=1 SV=1	KLAVNMVPFPR
>	sp|Q6P9T8|TB	Tubulin beta-2C chain OS=Rattus norvegicus GN=Tubb2c PE=1 SV=1	KLAVNMVPFPRLHFFMPGFAPLTSR
>	sp|Q6P9T8|TB	Tubulin beta-2C chain OS=Rattus norvegicus GN=Tubb2c PE=1 SV=1	KLAVNMVPFPRLHFFMPGFAPLTSR
>	sp|Q6P9T8|TB	Tubulin beta-2C chain OS=Rattus norvegicus GN=Tubb2c PE=1 SV=1	KLAVNMVPFPRLHFFMPGFAPLTSR
>	sp|Q6P9T8|TB	Tubulin beta-2C chain OS=Rattus norvegicus GN=Tubb2c PE=1 SV=1	LAVNMVPFPR
>	sp|Q6P9T8|TB	Tubulin beta-2C chain OS=Rattus norvegicus GN=Tubb2c PE=1 SV=1	LAVNMVPFPR
>	sp|Q6P9T8|TB	Tubulin beta-2C chain OS=Rattus norvegicus GN=Tubb2c PE=1 SV=1	LAVNMVPFPR
>	sp|Q6P9T8|TB	Tubulin beta-2C chain OS=Rattus norvegicus GN=Tubb2c PE=1 SV=1	LAVNMVPFPR
>	sp|Q6P9T8|TB	Tubulin beta-2C chain OS=Rattus norvegicus GN=Tubb2c PE=1 SV=1	LAVNMVPFPR
>	sp|Q6P9T8|TB	Tubulin beta-2C chain OS=Rattus norvegicus GN=Tubb2c PE=1 SV=1	LAVNMVPFPR
>	sp|Q6P9T8|TB	Tubulin beta-2C chain OS=Rattus norvegicus GN=Tubb2c PE=1 SV=1	LAVNMVPFPR
>	sp|Q6P9T8|TB	Tubulin beta-2C chain OS=Rattus norvegicus GN=Tubb2c PE=1 SV=1	LAVNMVPFPR
>	sp|Q6P9T8|TB	Tubulin beta-2C chain OS=Rattus norvegicus GN=Tubb2c PE=1 SV=1	LHFFMPGFAPLTSR
>	sp|Q6P9T8|TB	Tubulin beta-2C chain OS=Rattus norvegicus GN=Tubb2c PE=1 SV=1	LHFFMPGFAPLTSR
>	sp|Q6P9T8|TB	Tubulin beta-2C chain OS=Rattus norvegicus GN=Tubb2c PE=1 SV=1	LHFFMPGFAPLTSR
>	sp|Q6P9T8|TB	Tubulin beta-2C chain OS=Rattus norvegicus GN=Tubb2c PE=1 SV=1	LHFFMPGFAPLTSR
>	sp|Q6P9T8|TB	Tubulin beta-2C chain OS=Rattus norvegicus GN=Tubb2c PE=1 SV=1	LHFFMPGFAPLTSR
>	sp|Q6P9T8|TB	Tubulin beta-2C chain OS=Rattus norvegicus GN=Tubb2c PE=1 SV=1	LHFFMPGFAPLTSR
>	sp|Q6P9T8|TB	Tubulin beta-2C chain OS=Rattus norvegicus GN=Tubb2c PE=1 SV=1	LHFFMPGFAPLTSR
>	sp|Q6P9T8|TB	Tubulin beta-2C chain OS=Rattus norvegicus GN=Tubb2c PE=1 SV=1	LHFFMPGFAPLTSR
>	sp|Q6P9T8|TB	Tubulin beta-2C chain OS=Rattus norvegicus GN=Tubb2c PE=1 SV=1	LHFFMPGFAPLTSR
>	sp|Q6P9T8|TB	Tubulin beta-2C chain OS=Rattus norvegicus GN=Tubb2c PE=1 SV=1	LHFFMPGFAPLTSR
>	sp|Q6P9T8|TB	Tubulin beta-2C chain OS=Rattus norvegicus GN=Tubb2c PE=1 SV=1	LHFFMPGFAPLTSR
>	sp|Q6P9T8|TB	Tubulin beta-2C chain OS=Rattus norvegicus GN=Tubb2c PE=1 SV=1	LHFFMPGFAPLTSR
>	sp|Q6P9T8|TB	Tubulin beta-2C chain OS=Rattus norvegicus GN=Tubb2c PE=1 SV=1	LHFFMPGFAPLTSR
>	sp|Q6P9T8|TB	Tubulin beta-2C chain OS=Rattus norvegicus GN=Tubb2c PE=1 SV=1	LHFFMPGFAPLTSR
>	sp|Q6P9T8|TB	Tubulin beta-2C chain OS=Rattus norvegicus GN=Tubb2c PE=1 SV=1	LHFFMPGFAPLTSR

>	sp|Q6P9T8|TB	Tubulin beta-2C chain OS=Rattus norvegicus GN=Tubb2c PE=1 SV=1	LHFFMPGFAPLTSR
>	sp|Q6P9T8|TB	Tubulin beta-2C chain OS=Rattus norvegicus GN=Tubb2c PE=1 SV=1	LHFFMPGFAPLTSR
>	sp|Q6P9T8|TB	Tubulin beta-2C chain OS=Rattus norvegicus GN=Tubb2c PE=1 SV=1	LHFFMPGFAPLTSR
>	sp|Q6P9T8|TB	Tubulin beta-2C chain OS=Rattus norvegicus GN=Tubb2c PE=1 SV=1	LHFFMPGFAPLTSR
>	sp|Q6P9T8|TB	Tubulin beta-2C chain OS=Rattus norvegicus GN=Tubb2c PE=1 SV=1	LHFFMPGFAPLTSR
>	sp|Q6P9T8|TB	Tubulin beta-2C chain OS=Rattus norvegicus GN=Tubb2c PE=1 SV=1	LHFFMPGFAPLTSR
>	sp|Q6P9T8|TB	Tubulin beta-2C chain OS=Rattus norvegicus GN=Tubb2c PE=1 SV=1	LHFFMPGFAPLTSR
>	sp|Q6P9T8|TB	Tubulin beta-2C chain OS=Rattus norvegicus GN=Tubb2c PE=1 SV=1	LHFFMPGFAPLTSR
>	sp|Q6P9T8|TB	Tubulin beta-2C chain OS=Rattus norvegicus GN=Tubb2c PE=1 SV=1	LHFFMPGFAPLTSR
>	sp|Q6P9T8|TB	Tubulin beta-2C chain OS=Rattus norvegicus GN=Tubb2c PE=1 SV=1	LHFFMPGFAPLTSR
>	sp|Q6P9T8|TB	Tubulin beta-2C chain OS=Rattus norvegicus GN=Tubb2c PE=1 SV=1	LHFFMPGFAPLTSR
>	sp|Q6P9T8|TB	Tubulin beta-2C chain OS=Rattus norvegicus GN=Tubb2c PE=1 SV=1	LHFFMPGFAPLTSR
>	sp|Q6P9T8|TB	Tubulin beta-2C chain OS=Rattus norvegicus GN=Tubb2c PE=1 SV=1	LHFFMPGFAPLTSR
>	sp|Q6P9T8|TB	Tubulin beta-2C chain OS=Rattus norvegicus GN=Tubb2c PE=1 SV=1	LHFFMPGFAPLTSR
>	sp|Q6P9T8|TB	Tubulin beta-2C chain OS=Rattus norvegicus GN=Tubb2c PE=1 SV=1	LHFFMPGFAPLTSR
>	sp|Q6P9T8|TB	Tubulin beta-2C chain OS=Rattus norvegicus GN=Tubb2c PE=1 SV=1	LHFFMPGFAPLTSR
>	sp|Q6P9T8|TB	Tubulin beta-2C chain OS=Rattus norvegicus GN=Tubb2c PE=1 SV=1	LHFFMPGFAPLTSR
>	sp|Q6P9T8|TB	Tubulin beta-2C chain OS=Rattus norvegicus GN=Tubb2c PE=1 SV=1	LHFFMPGFAPLTSR
>	sp|Q6P9T8|TB	Tubulin beta-2C chain OS=Rattus norvegicus GN=Tubb2c PE=1 SV=1	LHFFMPGFAPLTSR
>	sp|Q6P9T8|TB	Tubulin beta-2C chain OS=Rattus norvegicus GN=Tubb2c PE=1 SV=1	LHFFMPGFAPLTSR
>	sp|Q6P9T8|TB	Tubulin beta-2C chain OS=Rattus norvegicus GN=Tubb2c PE=1 SV=1	LHFFMPGFAPLTSR
>	sp|Q6P9T8|TB	Tubulin beta-2C chain OS=Rattus norvegicus GN=Tubb2c PE=1 SV=1	LHFFMPGFAPLTSR
>	sp|Q6P9T8|TB	Tubulin beta-2C chain OS=Rattus norvegicus GN=Tubb2c PE=1 SV=1	LHFFMPGFAPLTSR
>	sp|Q6P9T8|TB	Tubulin beta-2C chain OS=Rattus norvegicus GN=Tubb2c PE=1 SV=1	LHFFMPGFAPLTSR
>	sp|Q6P9T8|TB	Tubulin beta-2C chain OS=Rattus norvegicus GN=Tubb2c PE=1 SV=1	LHFFMPGFAPLTSR
>	sp|Q6P9T8|TB	Tubulin beta-2C chain OS=Rattus norvegicus GN=Tubb2c PE=1 SV=1	LHFFMPGFAPLTSR
>	sp|Q6P9T8|TB	Tubulin beta-2C chain OS=Rattus norvegicus GN=Tubb2c PE=1 SV=1	LHFFMPGFAPLTSR
>	sp|Q6P9T8|TB	Tubulin beta-2C chain OS=Rattus norvegicus GN=Tubb2c PE=1 SV=1	LHFFMPGFAPLTSR
>	sp|Q6P9T8|TB	Tubulin beta-2C chain OS=Rattus norvegicus GN=Tubb2c PE=1 SV=1	LHFFMPGFAPLTSR
>	sp|Q6P9T8|TB	Tubulin beta-2C chain OS=Rattus norvegicus GN=Tubb2c PE=1 SV=1	LHFFMPGFAPLTSR
>	sp|Q6P9T8|TB	Tubulin beta-2C chain OS=Rattus norvegicus GN=Tubb2c PE=1 SV=1	MSATFIGNSTAIQELFKRISEQFTAMFR
>	sp|Q6P9T8|TB	Tubulin beta-2C chain OS=Rattus norvegicus GN=Tubb2c PE=1 SV=1	MSMKEVDEQMLNVQNK
>	sp|Q6P9T8|TB	Tubulin beta-2C chain OS=Rattus norvegicus GN=Tubb2c PE=1 SV=1	MSMKEVDEQMLNVQNK
>	sp|Q6P9T8|TB	Tubulin beta-2C chain OS=Rattus norvegicus GN=Tubb2c PE=1 SV=1	NSSYFVEWIPNNVK
>	sp|Q6P9T8|TB	Tubulin beta-2C chain OS=Rattus norvegicus GN=Tubb2c PE=1 SV=1	NSSYFVEWIPNNVK
>	sp|Q6P9T8|TB	Tubulin beta-2C chain OS=Rattus norvegicus GN=Tubb2c PE=1 SV=1	NSSYFVEWIPNNVK
>	sp|Q6P9T8|TB	Tubulin beta-2C chain OS=Rattus norvegicus GN=Tubb2c PE=1 SV=1	NSSYFVEWIPNNVK
>	sp|Q6P9T8|TB	Tubulin beta-2C chain OS=Rattus norvegicus GN=Tubb2c PE=1 SV=1	PELTQQMFDAK
>	sp|Q6P9T8|TB	Tubulin beta-2C chain OS=Rattus norvegicus GN=Tubb2c PE=1 SV=1	PGFAPLTSR
>	sp|Q6P9T8|TB	Tubulin beta-2C chain OS=Rattus norvegicus GN=Tubb2c PE=1 SV=1	PGQLNADLR
>	sp|Q6P9T8|TB	Tubulin beta-2C chain OS=Rattus norvegicus GN=Tubb2c PE=1 SV=1	PGQLNADLRK
>	sp|Q6P9T8|TB	Tubulin beta-2C chain OS=Rattus norvegicus GN=Tubb2c PE=1 SV=1	PGTMDSVR
>	sp|Q6P9T8|TB	Tubulin beta-2C chain OS=Rattus norvegicus GN=Tubb2c PE=1 SV=1	PGTMDSVR
>	sp|Q6P9T8|TB	Tubulin beta-2C chain OS=Rattus norvegicus GN=Tubb2c PE=1 SV=1	SGPFGQIFRPDNFVFGQSGAGNNWAK
>	sp|Q6P9T8|TB	Tubulin beta-2C chain OS=Rattus norvegicus GN=Tubb2c PE=1 SV=1	SGPFGQIFRPDNFVFGQSGAGNNWAK
>	sp|Q6P9T8|TB	Tubulin beta-2C chain OS=Rattus norvegicus GN=Tubb2c PE=1 SV=1	SGPFGQIFRPDNFVFGQSGAGNNWAK
>	sp|Q6P9T8|TB	Tubulin beta-2C chain OS=Rattus norvegicus GN=Tubb2c PE=1 SV=1	SGPFGQIFRPDNFVFGQSGAGNNWAK
>	sp|Q6P9T8|TB	Tubulin beta-2C chain OS=Rattus norvegicus GN=Tubb2c PE=1 SV=1	SGPFGQIFRPDNFVFGQSGAGNNWAK
>	sp|Q6P9T8|TB	Tubulin beta-2C chain OS=Rattus norvegicus GN=Tubb2c PE=1 SV=1	SGPFGQIFRPDNFVFGQSGAGNNWAK
>	sp|Q6P9T8|TB	Tubulin beta-2C chain OS=Rattus norvegicus GN=Tubb2c PE=1 SV=1	SGPFGQIFRPDNFVFGQSGAGNNWAK
>	sp|Q6P9T8|TB	Tubulin beta-2C chain OS=Rattus norvegicus GN=Tubb2c PE=1 SV=1	SGPFGQIFRPDNFVFGQSGAGNNWAK
>	sp|Q6P9T8|TB	Tubulin beta-2C chain OS=Rattus norvegicus GN=Tubb2c PE=1 SV=1	SGPFGQIFRPDNFVFGQSGAGNNWAK
>	sp|Q6P9T8|TB	Tubulin beta-2C chain OS=Rattus norvegicus GN=Tubb2c PE=1 SV=1	SGPFGQIFRPDNFVFGQSGAGNNWAK
>	sp|Q6P9T8|TB	Tubulin beta-2C chain OS=Rattus norvegicus GN=Tubb2c PE=1 SV=1	SGPFGQIFRPDNFVFGQSGAGNNWAK
>	sp|Q6P9T8|TB	Tubulin beta-2C chain OS=Rattus norvegicus GN=Tubb2c PE=1 SV=1	SGPFGQIFRPDNFVFGQSGAGNNWAK
>	sp|Q6P9T8|TB	Tubulin beta-2C chain OS=Rattus norvegicus GN=Tubb2c PE=1 SV=1	SGPFGQIFRPDNFVFGQSGAGNNWAK
>	sp|Q6P9T8|TB	Tubulin beta-2C chain OS=Rattus norvegicus GN=Tubb2c PE=1 SV=1	SGPFGQIFRPDNFVFGQSGAGNNWAK
>	sp|Q6P9T8|TB	Tubulin beta-2C chain OS=Rattus norvegicus GN=Tubb2c PE=1 SV=1	SGPFGQIFRPDNFVFGQSGAGNNWAK
>	sp|Q6P9T8|TB	Tubulin beta-2C chain OS=Rattus norvegicus GN=Tubb2c PE=1 SV=1	SGPFGQIFRPDNFVFGQSGAGNNWAK
>	sp|Q6P9T8|TB	Tubulin beta-2C chain OS=Rattus norvegicus GN=Tubb2c PE=1 SV=1	SGPFGQIFRPDNFVFGQSGAGNNWAK
>	sp|Q6P9T8|TB	Tubulin beta-2C chain OS=Rattus norvegicus GN=Tubb2c PE=1 SV=1	TAVCDIPPR
>	sp|Q6P9T8|TB	Tubulin beta-2C chain OS=Rattus norvegicus GN=Tubb2c PE=1 SV=1	TAVCDIPPR
>	sp|Q6P9T8|TB	Tubulin beta-2C chain OS=Rattus norvegicus GN=Tubb2c PE=1 SV=1	TAVCDIPPR
>	sp|Q6P9T8|TB	Tubulin beta-2C chain OS=Rattus norvegicus GN=Tubb2c PE=1 SV=1	TAVCDIPPR
>	sp|Q6P9T8|TB	Tubulin beta-2C chain OS=Rattus norvegicus GN=Tubb2c PE=1 SV=1	TAVCDIPPR
>	sp|Q6P9T8|TB	Tubulin beta-2C chain OS=Rattus norvegicus GN=Tubb2c PE=1 SV=1	TAVCDIPPR
>	sp|Q6P9T8|TB	Tubulin beta-2C chain OS=Rattus norvegicus GN=Tubb2c PE=1 SV=1	TAVCDIPPR
>	sp|Q6P9T8|TB	Tubulin beta-2C chain OS=Rattus norvegicus GN=Tubb2c PE=1 SV=1	TAVCDIPPR
>	sp|Q6P9T8|TB	Tubulin beta-2C chain OS=Rattus norvegicus GN=Tubb2c PE=1 SV=1	TAVCDIPPR
>	sp|Q6P9T8|TB	Tubulin beta-2C chain OS=Rattus norvegicus GN=Tubb2c PE=1 SV=1	VSDTVVEPYNATLSVHQLVENTDETYCIDNEALYDICFR
>	sp|Q6P9T8|TB	Tubulin beta-2C chain OS=Rattus norvegicus GN=Tubb2c PE=1 SV=1	VSDTVVEPYNATLSVHQLVENTDETYCIDNEALYDICFR
>	sp|Q6P9T8|TB	Tubulin beta-2C chain OS=Rattus norvegicus GN=Tubb2c PE=1 SV=1	VSDTVVEPYNATLSVHQLVENTDETYCIDNEALYDICFR
>	sp|Q6P9T8|TB	Tubulin beta-2C chain OS=Rattus norvegicus GN=Tubb2c PE=1 SV=1	VSDTVVEPYNATLSVHQLVENTDETYCIDNEALYDICFR
>	sp|Q6P9T8|TB	Tubulin beta-2C chain OS=Rattus norvegicus GN=Tubb2c PE=1 SV=1	VSDTVVEPYNATLSVHQLVENTDETYCIDNEALYDICFR

>	sp|Q6P9T8|TBI Tubulin beta-2C chain OS=Rattus norvegicus GN=Tubb2c PE=1 SV=1	VSDTVVEPYNATLSVHQLVENTDETYCIDNEALYDICFR
>	sp|Q6P9T8|TBI Tubulin beta-2C chain OS=Rattus norvegicus GN=Tubb2c PE=1 SV=1	VSDTVVEPYNATLSVHQLVENTDETYCIDNEALYDICFR
>	sp|Q6P9T8|TBI Tubulin beta-2C chain OS=Rattus norvegicus GN=Tubb2c PE=1 SV=1	VSDTVVEPYNATLSVHQLVENTDETYCIDNEALYDICFR
>	sp|Q6P9T8|TBI Tubulin beta-2C chain OS=Rattus norvegicus GN=Tubb2c PE=1 SV=1	VSDTVVEPYNATLSVHQLVENTDETYCIDNEALYDICFR
>	sp|Q6P9T8|TBI Tubulin beta-2C chain OS=Rattus norvegicus GN=Tubb2c PE=1 SV=1	VSDTVVEPYNATLSVHQLVENTDETYCIDNEALYDICFR
>	sp|Q6P9T8|TBI Tubulin beta-2C chain OS=Rattus norvegicus GN=Tubb2c PE=1 SV=1	VSDTVVEPYNATLSVHQLVENTDETYCIDNEALYDICFR
>	sp|Q6P9T8|TBI Tubulin beta-2C chain OS=Rattus norvegicus GN=Tubb2c PE=1 SV=1	VSDTVVEPYNATLSVHQLVENTDETYCIDNEALYDICFR
>	sp|Q6P9T8|TBI Tubulin beta-2C chain OS=Rattus norvegicus GN=Tubb2c PE=1 SV=1	VSDTVVEPYNATLSVHQLVENTDETYCIDNEALYDICFR
>	sp|Q6P9T8|TBI Tubulin beta-2C chain OS=Rattus norvegicus GN=Tubb2c PE=1 SV=1	VSDTVVEPYNATLSVHQLVENTDETYCIDNEALYDICFR
>	sp|Q6P9T8|TBI Tubulin beta-2C chain OS=Rattus norvegicus GN=Tubb2c PE=1 SV=1	VSDTVVEPYNATLSVHQLVENTDETYCIDNEALYDICFR
>	sp|Q6P9T8|TBI Tubulin beta-2C chain OS=Rattus norvegicus GN=Tubb2c PE=1 SV=1	VSDTVVEPYNATLSVHQLVENTDETYCIDNEALYDICFR
>	sp|Q6P9T8|TBI Tubulin beta-2C chain OS=Rattus norvegicus GN=Tubb2c PE=1 SV=1	VSDTVVEPYNATLSVHQLVENTDETYCIDNEALYDICFR
>	sp|Q6P9T8|TBI Tubulin beta-2C chain OS=Rattus norvegicus GN=Tubb2c PE=1 SV=1	VSDTVVEPYNATLSVHQLVENTDETYCIDNEALYDICFR
>	sp|Q6P9T8|TBI Tubulin beta-2C chain OS=Rattus norvegicus GN=Tubb2c PE=1 SV=1	VSDTVVEPYNATLSVHQLVENTDETYCIDNEALYDICFR
>	sp|Q6P9T8|TBI Tubulin beta-2C chain OS=Rattus norvegicus GN=Tubb2c PE=1 SV=1	VSDTVVEPYNATLSVHQLVENTDETYCIDNEALYDICFR
>	sp|Q6P9T8|TBI Tubulin beta-2C chain OS=Rattus norvegicus GN=Tubb2c PE=1 SV=1	VSDTVVEPYNATLSVHQLVENTDETYCIDNEALYDICFR
>	sp|Q6P9T8|TBI Tubulin beta-2C chain OS=Rattus norvegicus GN=Tubb2c PE=1 SV=1	VSDTVVEPYNATLSVHQLVENTDETYCIDNEALYDICFR
>	sp|Q6P9T8|TBI Tubulin beta-2C chain OS=Rattus norvegicus GN=Tubb2c PE=1 SV=1	VSDTVVEPYNATLSVHQLVENTDETYCIDNEALYDICFR
>	sp|Q6P9T8|TBI Tubulin beta-2C chain OS=Rattus norvegicus GN=Tubb2c PE=1 SV=1	VSDTVVEPYNATLSVHQLVENTDETYCIDNEALYDICFR
>	sp|Q6P9T8|TBI Tubulin beta-2C chain OS=Rattus norvegicus GN=Tubb2c PE=1 SV=1	VSDTVVEPYNATLSVHQLVENTDETYCIDNEALYDICFR
>	sp|Q6P9T8|TBI Tubulin beta-2C chain OS=Rattus norvegicus GN=Tubb2c PE=1 SV=1	VSDTVVEPYNATLSVHQLVENTDETYCIDNEALYDICFR
>	sp|Q6P9T8|TBI Tubulin beta-2C chain OS=Rattus norvegicus GN=Tubb2c PE=1 SV=1	VSDTVVEPYNATLSVHQLVENTDETYCIDNEALYDICFR
>	sp|Q6P9T8|TBI Tubulin beta-2C chain OS=Rattus norvegicus GN=Tubb2c PE=1 SV=1	VSDTVVEPYNATLSVHQLVENTDETYCIDNEALYDICFR
>	sp|Q6P9T8|TBI Tubulin beta-2C chain OS=Rattus norvegicus GN=Tubb2c PE=1 SV=1	VSDTVVEPYNATLSVHQLVENTDETYCIDNEALYDICFR
>	sp|Q6P9T8|TBI Tubulin beta-2C chain OS=Rattus norvegicus GN=Tubb2c PE=1 SV=1	VSDTVVEPYNATLSVHQLVENTDETYCIDNEALYDICFR
>	sp|Q6P9T8|TBI Tubulin beta-2C chain OS=Rattus norvegicus GN=Tubb2c PE=1 SV=1	VSDTVVEPYNATLSVHQLVENTDETYCIDNEALYDICFR
>	sp|Q6P9T8|TBI Tubulin beta-2C chain OS=Rattus norvegicus GN=Tubb2c PE=1 SV=1	VSDTVVEPYNATLSVHQLVENTDETYCIDNEALYDICFR
>	sp|Q6P9T8|TBI Tubulin beta-2C chain OS=Rattus norvegicus GN=Tubb2c PE=1 SV=1	VSDTVVEPYNATLSVHQLVENTDETYCIDNEALYDICFR
>	sp|Q6P9T8|TBI Tubulin beta-2C chain OS=Rattus norvegicus GN=Tubb2c PE=1 SV=1	VSDTVVEPYNATLSVHQLVENTDETYCIDNEALYDICFR
>	sp|Q6P9T8|TBI Tubulin beta-2C chain OS=Rattus norvegicus GN=Tubb2c PE=1 SV=1	VSDTVVEPYNATLSVHQLVENTDETYCIDNEALYDICFR
>	sp|Q6P9T8|TBI Tubulin beta-2C chain OS=Rattus norvegicus GN=Tubb2c PE=1 SV=1	VSDTVVEPYNATLSVHQLVENTDETYCIDNEALYDICFR
>	sp|Q6P9T8|TBI Tubulin beta-2C chain OS=Rattus norvegicus GN=Tubb2c PE=1 SV=1	VSDTVVEPYNATLSVHQLVENTDETYCIDNEALYDICFR
>	sp|Q6P9T8|TBI Tubulin beta-2C chain OS=Rattus norvegicus GN=Tubb2c PE=1 SV=1	VSDTVVEPYNATLSVHQLVENTDETYCIDNEALYDICFR
>	sp|Q6P9T8|TBI Tubulin beta-2C chain OS=Rattus norvegicus GN=Tubb2c PE=1 SV=1	VSDTVVEPYNATLSVHQLVENTDETYCIDNEALYDICFR
>	sp|Q6P9T8|TBI Tubulin beta-2C chain OS=Rattus norvegicus GN=Tubb2c PE=1 SV=1	VSDTVVEPYNATLSVHQLVENTDETYCIDNEALYDICFR
>	sp|Q6P9T8|TBI Tubulin beta-2C chain OS=Rattus norvegicus GN=Tubb2c PE=1 SV=1	VSDTVVEPYNATLSVHQLVENTDETYCIDNEALYDICFR
>	sp|Q6P9T8|TBI Tubulin beta-2C chain OS=Rattus norvegicus GN=Tubb2c PE=1 SV=1	VSDTVVEPYNATLSVHQLVENTDETYCIDNEALYDICFR
>	sp|Q6P9T8|TBI Tubulin beta-2C chain OS=Rattus norvegicus GN=Tubb2c PE=1 SV=1	VSDTVVEPYNATLSVHQLVENTDETYCIDNEALYDICFR
>	sp|Q6P9T8|TBI Tubulin beta-2C chain OS=Rattus norvegicus GN=Tubb2c PE=1 SV=1	VSDTVVEPYNATLSVHQLVENTDETYCIDNEALYDICFR
>	sp|Q6P9T8|TBI Tubulin beta-2C chain OS=Rattus norvegicus GN=Tubb2c PE=1 SV=1	VSDTVVEPYNATLSVHQLVENTDETYCIDNEALYDICFR
>	sp|Q6P9T8|TBI Tubulin beta-2C chain OS=Rattus norvegicus GN=Tubb2c PE=1 SV=1	VSDTVVEPYNATLSVHQLVENTDETYCIDNEALYDICFR
>	sp|Q6P9T8|TBI Tubulin beta-2C chain OS=Rattus norvegicus GN=Tubb2c PE=1 SV=1	VSDTVVEPYNATLSVHQLVENTDETYCIDNEALYDICFR
>	sp|Q6P9T8|TBI Tubulin beta-2C chain OS=Rattus norvegicus GN=Tubb2c PE=1 SV=1	VSDTVVEPYNATLSVHQLVENTDETYCIDNEALYDICFR
>	sp|Q6P9T8|TBI Tubulin beta-2C chain OS=Rattus norvegicus GN=Tubb2c PE=1 SV=1	VSDTVVEPYNATLSVHQLVENTDETYCIDNEALYDICFR
>	sp|Q6P9T8|TBI Tubulin beta-2C chain OS=Rattus norvegicus GN=Tubb2c PE=1 SV=1	VSDTVVEPYNATLSVHQLVENTDETYCIDNEALYDICFR
>	sp|Q6P9T8|TBI Tubulin beta-2C chain OS=Rattus norvegicus GN=Tubb2c PE=1 SV=1	VSDTVVEPYNATLSVHQLVENTDETYCIDNEALYDICFR
>	sp|Q6P9T8|TBI Tubulin beta-2C chain OS=Rattus norvegicus GN=Tubb2c PE=1 SV=1	VSDTVVEPYNATLSVHQLVENTDETYCIDNEALYDICFR
>	sp|Q6P9T8|TBI Tubulin beta-2C chain OS=Rattus norvegicus GN=Tubb2c PE=1 SV=1	VSDTVVEPYNATLSVHQLVENTDETYCIDNEALYDICFR
>	sp|Q6P9T8|TBI Tubulin beta-2C chain OS=Rattus norvegicus GN=Tubb2c PE=1 SV=1	VSDTVVEPYNATLSVHQLVENTDETYCIDNEALYDICFR
>	sp|Q6P9T8|TBI Tubulin beta-2C chain OS=Rattus norvegicus GN=Tubb2c PE=1 SV=1	VSDTVVEPYNATLSVHQLVENTDETYCIDNEALYDICFR
>	sp|Q6P9T8|TBI Tubulin beta-2C chain OS=Rattus norvegicus GN=Tubb2c PE=1 SV=1	VSDTVVEPYNATLSVHQLVENTDETYCIDNEALYDICFR
>	sp|Q6P9T8|TBI Tubulin beta-2C chain OS=Rattus norvegicus GN=Tubb2c PE=1 SV=1	VSDTVVEPYNATLSVHQLVENTDETYCIDNEALYDICFR
>	sp|Q6P9T8|TBI Tubulin beta-2C chain OS=Rattus norvegicus GN=Tubb2c PE=1 SV=1	VSDTVVEPYNATLSVHQLVENTDETYCIDNEALYDICFR
>	sp|Q6P9T8|TBI Tubulin beta-2C chain OS=Rattus norvegicus GN=Tubb2c PE=1 SV=1	VSDTVVEPYNATLSVHQLVENTDETYCIDNEALYDICFR
>	sp|Q6P9T8|TBI Tubulin beta-2C chain OS=Rattus norvegicus GN=Tubb2c PE=1 SV=1	VSDTVVEPYNATLSVHQLVENTDETYCIDNEALYDICFR
>	sp|Q6P9T8|TBI Tubulin beta-2C chain OS=Rattus norvegicus GN=Tubb2c PE=1 SV=1	VSDTVVEPYNATLSVHQLVENTDETYCIDNEALYDICFR
>	sp|Q6P9T8|TBI Tubulin beta-2C chain OS=Rattus norvegicus GN=Tubb2c PE=1 SV=1	YLTVAAVFR
>	sp|Q6P9T8|TBI Tubulin beta-2C chain OS=Rattus norvegicus GN=Tubb2c PE=1 SV=1	YLTVAAVFR
>	sp|Q6P9T8|TBI Tubulin beta-2C chain OS=Rattus norvegicus GN=Tubb2c PE=1 SV=1	YLTVAAVFR
>	sp|Q6P9T8|TBI Tubulin beta-2C chain OS=Rattus norvegicus GN=Tubb2c PE=1 SV=1	TAVCDIPPR
>	sp|Q6P9T8|TBI Tubulin beta-2C chain OS=Rattus norvegicus GN=Tubb2c PE=1 SV=1	NSSYFVEWIPNNVK
>	sp|Q6P9T8|TBI Tubulin beta-2C chain OS=Rattus norvegicus GN=Tubb2c PE=1 SV=1	TAVCDIPPR
>	sp|Q6P9T8|TBI Tubulin beta-2C chain OS=Rattus norvegicus GN=Tubb2c PE=1 SV=1	EIVHLQAGQCGNQIGAK
>	sp|Q6P9T8|TBI Tubulin beta-2C chain OS=Rattus norvegicus GN=Tubb2c PE=1 SV=1	LHFFMPGFAPLTSR
>	sp|Q6P9T8|TBI Tubulin beta-2C chain OS=Rattus norvegicus GN=Tubb2c PE=1 SV=1	VSDTVVEPYNATLSVHQLVENTDETYCIDNEALYDICFR
>	sp|Q6P9T8|TBI Tubulin beta-2C chain OS=Rattus norvegicus GN=Tubb2c PE=1 SV=1	TAVCDIPPR
>	sp|Q6P9T8|TBI Tubulin beta-2C chain OS=Rattus norvegicus GN=Tubb2c PE=1 SV=1	VSDTVVEPYNATLSVHQLVENTDETYCIDNEALYDICFR
>	sp|Q6P9T8|TBI Tubulin beta-2C chain OS=Rattus norvegicus GN=Tubb2c PE=1 SV=1	VSDTVVEPYNATLSVHQLVENTDETYCIDNEALYDICFR
>	sp|Q6P9T8|TBI Tubulin beta-2C chain OS=Rattus norvegicus GN=Tubb2c PE=1 SV=1	FPGQLNADLR
>	sp|Q6P9T8|TBI Tubulin beta-2C chain OS=Rattus norvegicus GN=Tubb2c PE=1 SV=1	KLAVNMVPFPR
>	sp|Q6P9T8|TBI Tubulin beta-2C chain OS=Rattus norvegicus GN=Tubb2c PE=1 SV=1	NSSYFVEWIPNNVK
>	sp|Q6P9T8|TBI Tubulin beta-2C chain OS=Rattus norvegicus GN=Tubb2c PE=1 SV=1	NSSYFVEWIPNNVK
>	sp|Q6P9T8|TBI Tubulin beta-2C chain OS=Rattus norvegicus GN=Tubb2c PE=1 SV=1	LHFFMPGFAPLTSR
>	sp|Q6P9T8|TBI Tubulin beta-2C chain OS=Rattus norvegicus GN=Tubb2c PE=1 SV=1	NSSYFVEWIPNNVK

>	sp|Q6P9T8|TBB	Tubulin beta-2C chain OS=Rattus norvegicus GN=Tubb2c PE=1 SV=1	LHFFMPGFAPLTSR
>	sp|Q6P9T8|TBB	Tubulin beta-2C chain OS=Rattus norvegicus GN=Tubb2c PE=1 SV=1	GHYTEGAELVDSVLDVVR
>	sp|Q6P9T8|TBB	Tubulin beta-2C chain OS=Rattus norvegicus GN=Tubb2c PE=1 SV=1	LHFFMPGFAPLTSR
>	sp|Q6P9T8|TBB	Tubulin beta-2C chain OS=Rattus norvegicus GN=Tubb2c PE=1 SV=1	VSDTVVEPYNATLSVHQLVENTDETYCIDNEALYDICFR
>	sp|Q6P9T8|TBB	Tubulin beta-2C chain OS=Rattus norvegicus GN=Tubb2c PE=1 SV=1	VSDTVVEPYNATLSVHQLVENTDETYCIDNEALYDICFR
>	sp|Q6P9T8|TBB	Tubulin beta-2C chain OS=Rattus norvegicus GN=Tubb2c PE=1 SV=1	EIVHLQAGQCGNQIGAK
>	sp|Q6P9T8|TBB	Tubulin beta-2C chain OS=Rattus norvegicus GN=Tubb2c PE=1 SV=1	GHYTEGAELVDSVLDVVR
>	sp|Q6P9T8|TBB	Tubulin beta-2C chain OS=Rattus norvegicus GN=Tubb2c PE=1 SV=1	GHYTEGAELVDSVLDVVR
>	sp|Q6P9T8|TBB	Tubulin beta-2C chain OS=Rattus norvegicus GN=Tubb2c PE=1 SV=1	VSDTVVEPYNATLSVHQLVENTDETYCIDNEALYDICFR
>	sp|Q6P9T8|TBB	Tubulin beta-2C chain OS=Rattus norvegicus GN=Tubb2c PE=1 SV=1	GHYTEGAELVDSVLDVVR
>	sp|Q6P9T8|TBB	Tubulin beta-2C chain OS=Rattus norvegicus GN=Tubb2c PE=1 SV=1	VSDTVVEPYNATLSVHQLVENTDETYCIDNEALYDICFR
>	sp|Q6P9T8|TBB	Tubulin beta-2C chain OS=Rattus norvegicus GN=Tubb2c PE=1 SV=1	VSDTVVEPYNATLSVHQLVENTDETYCIDNEALYDICFR
>	sp|Q6P9T8|TBB	Tubulin beta-2C chain OS=Rattus norvegicus GN=Tubb2c PE=1 SV=1	VSDTVVEPYNATLSVHQLVENTDETYCIDNEALYDICFR
>	sp|Q6P9T8|TBB	Tubulin beta-2C chain OS=Rattus norvegicus GN=Tubb2c PE=1 SV=1	EIVHLQAGQCGNQIGAK
>	sp|Q6P9T8|TBB	Tubulin beta-2C chain OS=Rattus norvegicus GN=Tubb2c PE=1 SV=1	EVDEQMLNVQNK
>	sp|Q6P9T8|TBB	Tubulin beta-2C chain OS=Rattus norvegicus GN=Tubb2c PE=1 SV=1	KLAVNMVPFPR
>	sp|Q6P9T8|TBB	Tubulin beta-2C chain OS=Rattus norvegicus GN=Tubb2c PE=1 SV=1	EAESCDCLQGFQLTHSLGGGTGSGMGTLLISK
>	sp|Q6P9T8|TBB	Tubulin beta-2C chain OS=Rattus norvegicus GN=Tubb2c PE=1 SV=1	EIVHLQAGQCGNQIGAK
>	sp|Q6P9T8|TBB	Tubulin beta-2C chain OS=Rattus norvegicus GN=Tubb2c PE=1 SV=1	EIVHLQAGQCGNQIGAK
>	sp|Q6P9T8|TBB	Tubulin beta-2C chain OS=Rattus norvegicus GN=Tubb2c PE=1 SV=1	LHFFMPGFAPLTSR
>	sp|Q6P9T8|TBB	Tubulin beta-2C chain OS=Rattus norvegicus GN=Tubb2c PE=1 SV=1	LAVNMVPFPR
>	sp|Q6P9T8|TBB	Tubulin beta-2C chain OS=Rattus norvegicus GN=Tubb2c PE=1 SV=1	KLAVNMVPFPR
>	sp|Q6P9T8|TBB	Tubulin beta-2C chain OS=Rattus norvegicus GN=Tubb2c PE=1 SV=1	PGTMDSVR
>	sp|Q6P9T8|TBB	Tubulin beta-2C chain OS=Rattus norvegicus GN=Tubb2c PE=1 SV=1	VSDTVVEPYNATLSVHQLVENTDETYCIDNEALYDICFR
>	sp|Q6P9T8|TBB	Tubulin beta-2C chain OS=Rattus norvegicus GN=Tubb2c PE=1 SV=1	GHYTEGAELVDSVLDVVR
>	sp|Q6P9T8|TBB	Tubulin beta-2C chain OS=Rattus norvegicus GN=Tubb2c PE=1 SV=1	LTTPTYGDLNHLVSATMSGVTACLR
>	sp|Q6P9T8|TBB	Tubulin beta-2C chain OS=Rattus norvegicus GN=Tubb2c PE=1 SV=1	VSDTVVEPYNATLSVHQLVENTDETYCIDNEALYDICFR
>	sp|Q6P9T8|TBB	Tubulin beta-2C chain OS=Rattus norvegicus GN=Tubb2c PE=1 SV=1	SGPFGQIFRPDNFVFGQSGAGNNWAK
>	sp|Q6P9T8|TBB	Tubulin beta-2C chain OS=Rattus norvegicus GN=Tubb2c PE=1 SV=1	SGPFGQIFRPDNFVFGQSGAGNNWAK
>	sp|Q6P9T8|TBB	Tubulin beta-2C chain OS=Rattus norvegicus GN=Tubb2c PE=1 SV=1	EIVHLQAGQCGNQIGAK
>	sp|Q6P9T8|TBB	Tubulin beta-2C chain OS=Rattus norvegicus GN=Tubb2c PE=1 SV=1	KLAVNMVPFPR
>	sp|Q6P9T8|TBB	Tubulin beta-2C chain OS=Rattus norvegicus GN=Tubb2c PE=1 SV=1	LHFFMPGFAPLTSR
>	sp|Q6P9T8|TBB	Tubulin beta-2C chain OS=Rattus norvegicus GN=Tubb2c PE=1 SV=1	KLAVNMVPFPRLHFFMPGFAPLTSR
>	sp|Q6P9T8|TBB	Tubulin beta-2C chain OS=Rattus norvegicus GN=Tubb2c PE=1 SV=1	FPGQLNADLRK
>	sp|Q6P9T8|TBB	Tubulin beta-2C chain OS=Rattus norvegicus GN=Tubb2c PE=1 SV=1	KLAVNMVPFPR
>	sp|Q6P9T8|TBB	Tubulin beta-2C chain OS=Rattus norvegicus GN=Tubb2c PE=1 SV=1	KLAVNMVPFPR
>	sp|Q6P9T8|TBB	Tubulin beta-2C chain OS=Rattus norvegicus GN=Tubb2c PE=1 SV=1	LAVNMVPFPR
>	sp|Q6P9T8|TBB	Tubulin beta-2C chain OS=Rattus norvegicus GN=Tubb2c PE=1 SV=1	KLAVNMVPFPR
>	sp|Q6P9T8|TBB	Tubulin beta-2C chain OS=Rattus norvegicus GN=Tubb2c PE=1 SV=1	KLAVNMVPFPR
>	sp|Q6P9T8|TBB	Tubulin beta-2C chain OS=Rattus norvegicus GN=Tubb2c PE=1 SV=1	GHYTEGAELVDSVLDVVR
>	sp|Q6P9T8|TBB	Tubulin beta-2C chain OS=Rattus norvegicus GN=Tubb2c PE=1 SV=1	INVYYNEATGGK
>	sp|Q6P9T8|TBB	Tubulin beta-2C chain OS=Rattus norvegicus GN=Tubb2c PE=1 SV=1	KLAVNMVPFPR
>	sp|Q6P9T8|TBB	Tubulin beta-2C chain OS=Rattus norvegicus GN=Tubb2c PE=1 SV=1	LAVNMVPFPR
>	sp|Q6P9T8|TBB	Tubulin beta-2C chain OS=Rattus norvegicus GN=Tubb2c PE=1 SV=1	VSDTVVEPYNATLSVHQLVENTDETYCIDNEALYDICFR
>	sp|Q6P9T8|TBB	Tubulin beta-2C chain OS=Rattus norvegicus GN=Tubb2c PE=1 SV=1	SGPFGQIFRPDNFVFGQSGAGNNWAK
>	sp|Q6P9T8|TBB	Tubulin beta-2C chain OS=Rattus norvegicus GN=Tubb2c PE=1 SV=1	AVLVDLEPGTMDSVR
>	sp|Q6P9T8|TBB	Tubulin beta-2C chain OS=Rattus norvegicus GN=Tubb2c PE=1 SV=1	VSDTVVEPYNATLSVHQLVENTDETYCIDNEALYDICFR
>	sp|Q6P9T8|TBB	Tubulin beta-2C chain OS=Rattus norvegicus GN=Tubb2c PE=1 SV=1	VSDTVVEPYNATLSVHQLVENTDETYCIDNEALYDICFR
>	sp|Q6P9T8|TBB	Tubulin beta-2C chain OS=Rattus norvegicus GN=Tubb2c PE=1 SV=1	LHFFMPGFAPLTSR
>	sp|Q6P9T8|TBB	Tubulin beta-2C chain OS=Rattus norvegicus GN=Tubb2c PE=1 SV=1	GHYTEGAELVDSVLDVVR
>	sp|Q6P9T8|TBB	Tubulin beta-2C chain OS=Rattus norvegicus GN=Tubb2c PE=1 SV=1	EIVHLQAGQCGNQIGAK
>	sp|Q6P9T8|TBB	Tubulin beta-2C chain OS=Rattus norvegicus GN=Tubb2c PE=1 SV=1	KLAVNMVPFPR
>	sp|Q6P9T8|TBB	Tubulin beta-2C chain OS=Rattus norvegicus GN=Tubb2c PE=1 SV=1	KLAVNMVPFPRLHFFMPGFAPLTSR
>	sp|Q6P9T8|TBB	Tubulin beta-2C chain OS=Rattus norvegicus GN=Tubb2c PE=1 SV=1	TAVCDIPPR
>	sp|Q6P9T8|TBB	Tubulin beta-2C chain OS=Rattus norvegicus GN=Tubb2c PE=1 SV=1	EIVHLQAGQCGNQIGAK
>	sp|Q6P9T8|TBB	Tubulin beta-2C chain OS=Rattus norvegicus GN=Tubb2c PE=1 SV=1	LHFFMPGFAPLTSR
>	sp|Q6P9T8|TBB	Tubulin beta-2C chain OS=Rattus norvegicus GN=Tubb2c PE=1 SV=1	VSDTVVEPYNATLSVHQLVENTDETYCIDNEALYDICFR
>	sp|Q6P9T8|TBB	Tubulin beta-2C chain OS=Rattus norvegicus GN=Tubb2c PE=1 SV=1	ALTVPELTQQMFDAK
>	sp|Q6P9T8|TBB	Tubulin beta-2C chain OS=Rattus norvegicus GN=Tubb2c PE=1 SV=1	FPGQLNADLR
>	sp|Q6P9T8|TBB	Tubulin beta-2C chain OS=Rattus norvegicus GN=Tubb2c PE=1 SV=1	VSDTVVEPYNATLSVHQLVENTDETYCIDNEALYDICFR
>	sp|Q6P9T8|TBB	Tubulin beta-2C chain OS=Rattus norvegicus GN=Tubb2c PE=1 SV=1	IMNTFSVVPSPK
>	sp|Q6P9T8|TBB	Tubulin beta-2C chain OS=Rattus norvegicus GN=Tubb2c PE=1 SV=1	VSDTVVEPYNATLSVHQLVENTDETYCIDNEALYDICFR
>	sp|Q6P9T8|TBB	Tubulin beta-2C chain OS=Rattus norvegicus GN=Tubb2c PE=1 SV=1	YLTVAAVFR
>	sp|Q6P9T8|TBB	Tubulin beta-2C chain OS=Rattus norvegicus GN=Tubb2c PE=1 SV=1	KLAVNMVPFPR
>	sp|Q6P9T8|TBB	Tubulin beta-2C chain OS=Rattus norvegicus GN=Tubb2c PE=1 SV=1	EIVHLQAGQCGNQIGAK
>	sp|Q6P9T8|TBB	Tubulin beta-2C chain OS=Rattus norvegicus GN=Tubb2c PE=1 SV=1	NSSYFVEWIPNNVK
>	sp|Q6P9T8|TBB	Tubulin beta-2C chain OS=Rattus norvegicus GN=Tubb2c PE=1 SV=1	VSDTVVEPYNATLSVHQLVENTDETYCIDNEALYDICFR
>	sp|Q6P9T8|TBB	Tubulin beta-2C chain OS=Rattus norvegicus GN=Tubb2c PE=1 SV=1	VSDTVVEPYNATLSVHQLVENTDETYCIDNEALYDICFR
>	sp|Q6P9T8|TBB	Tubulin beta-2C chain OS=Rattus norvegicus GN=Tubb2c PE=1 SV=1	IMNTFSVVPSPK
>	sp|Q6P9T8|TBB	Tubulin beta-2C chain OS=Rattus norvegicus GN=Tubb2c PE=1 SV=1	NSSYFVEWIPNNVK
>	sp|Q6P9T8|TBB	Tubulin beta-2C chain OS=Rattus norvegicus GN=Tubb2c PE=1 SV=1	NMMAACDPR
>	sp|Q6P9T8|TBB	Tubulin beta-2C chain OS=Rattus norvegicus GN=Tubb2c PE=1 SV=1	KEAESCDCLQGFQLTHSLGGGTGSGMGTLLISK
>	sp|Q6P9T8|TBB	Tubulin beta-2C chain OS=Rattus norvegicus GN=Tubb2c PE=1 SV=1	NSSYFVEWIPNNVK
>	sp|Q6P9T8|TBB	Tubulin beta-2C chain OS=Rattus norvegicus GN=Tubb2c PE=1 SV=1	KLAVNMVPFPR

>	sp	Q6P9T8	TBI Tubulin beta-2C chain OS=Rattus norvegicus GN=Tubb2c PE=1 SV=1	ISEQFTAMFR
>	sp	Q6P9T8	TBI Tubulin beta-2C chain OS=Rattus norvegicus GN=Tubb2c PE=1 SV=1	VSDTVVEPYNATLSVHQLVENTDETYCIDNEALYDICFR
>	sp	Q6P9T8	TBI Tubulin beta-2C chain OS=Rattus norvegicus GN=Tubb2c PE=1 SV=1	TAVCDIPPR
>	sp	Q6P9T8	TBI Tubulin beta-2C chain OS=Rattus norvegicus GN=Tubb2c PE=1 SV=1	VSDTVVEPYNATLSVHQLVENTDETYCIDNEALYDICFR
>	sp	Q6P9T8	TBI Tubulin beta-2C chain OS=Rattus norvegicus GN=Tubb2c PE=1 SV=1	AVLVDLEPGTMDSVR
>	sp	Q6P9T8	TBI Tubulin beta-2C chain OS=Rattus norvegicus GN=Tubb2c PE=1 SV=1	SGPFGQIFRPDNFVFGQSGAGNNWAK
>	sp	Q6P9T8	TBI Tubulin beta-2C chain OS=Rattus norvegicus GN=Tubb2c PE=1 SV=1	FPGQLNADLR
>	sp	Q6P9T8	TBI Tubulin beta-2C chain OS=Rattus norvegicus GN=Tubb2c PE=1 SV=1	KLAVNMVPFPR
>	sp	Q6P9T8	TBI Tubulin beta-2C chain OS=Rattus norvegicus GN=Tubb2c PE=1 SV=1	LTTPTYGDLNHLVSATMSGVTACLRF
>	sp	Q6P9T8	TBI Tubulin beta-2C chain OS=Rattus norvegicus GN=Tubb2c PE=1 SV=1	TAVCDIPPR
>	sp	Q6P9T8	TBI Tubulin beta-2C chain OS=Rattus norvegicus GN=Tubb2c PE=1 SV=1	EIVHLQAGQCGNQIGAK
>	sp	Q6P9T8	TBI Tubulin beta-2C chain OS=Rattus norvegicus GN=Tubb2c PE=1 SV=1	INVYYNEATGGK
>	sp	Q6P9T8	TBI Tubulin beta-2C chain OS=Rattus norvegicus GN=Tubb2c PE=1 SV=1	EIVHLQAGQCGNQIGAK
>	sp	Q6P9T8	TBI Tubulin beta-2C chain OS=Rattus norvegicus GN=Tubb2c PE=1 SV=1	KLAVNMVPFPR
>	sp	Q6P9T8	TBI Tubulin beta-2C chain OS=Rattus norvegicus GN=Tubb2c PE=1 SV=1	EIVHLQAGQCGNQIGAK
>	sp	Q6P9T8	TBI Tubulin beta-2C chain OS=Rattus norvegicus GN=Tubb2c PE=1 SV=1	EVDEQMLNVQNK
>	sp	Q6P9T8	TBI Tubulin beta-2C chain OS=Rattus norvegicus GN=Tubb2c PE=1 SV=1	VSDTVVEPYNATLSVHQLVENTDETYCIDNEALYDICFR
>	sp	Q6P9T8	TBI Tubulin beta-2C chain OS=Rattus norvegicus GN=Tubb2c PE=1 SV=1	VSDTVVEPYNATLSVHQLVENTDETYCIDNEALYDICFR
>	sp	Q6P9T8	TBI Tubulin beta-2C chain OS=Rattus norvegicus GN=Tubb2c PE=1 SV=1	TAVCDIPPR
>	sp	Q6P9T8	TBI Tubulin beta-2C chain OS=Rattus norvegicus GN=Tubb2c PE=1 SV=1	ALTVPELTQQMFDAK
>	sp	Q6P9T8	TBI Tubulin beta-2C chain OS=Rattus norvegicus GN=Tubb2c PE=1 SV=1	EIVHLQAGQCGNQIGAK
>	sp	Q6P9T8	TBI Tubulin beta-2C chain OS=Rattus norvegicus GN=Tubb2c PE=1 SV=1	VSDTVVEPYNATLSVHQLVENTDETYCIDNEALYDICFR
>	sp	Q6P9T8	TBI Tubulin beta-2C chain OS=Rattus norvegicus GN=Tubb2c PE=1 SV=1	FPGQLNADLR
>	sp	Q6P9T8	TBI Tubulin beta-2C chain OS=Rattus norvegicus GN=Tubb2c PE=1 SV=1	KLAVNMVPFPR
>	sp	Q6P9T8	TBI Tubulin beta-2C chain OS=Rattus norvegicus GN=Tubb2c PE=1 SV=1	YLTVAAVFR
>	sp	Q6P9T8	TBI Tubulin beta-2C chain OS=Rattus norvegicus GN=Tubb2c PE=1 SV=1	MSMKEVDEQMLNVQNK
>	sp	Q6P9T8	TBI Tubulin beta-2C chain OS=Rattus norvegicus GN=Tubb2c PE=1 SV=1	NSSYFVEWIPNNVK
>	sp	Q6P9T8	TBI Tubulin beta-2C chain OS=Rattus norvegicus GN=Tubb2c PE=1 SV=1	KLAVNMVPFPR
>	sp	Q6P9T8	TBI Tubulin beta-2C chain OS=Rattus norvegicus GN=Tubb2c PE=1 SV=1	KLAVNMVPFPR
>	sp	Q6P9T8	TBI Tubulin beta-2C chain OS=Rattus norvegicus GN=Tubb2c PE=1 SV=1	KLAVNMVPFPR
>	sp	Q6P9T8	TBI Tubulin beta-2C chain OS=Rattus norvegicus GN=Tubb2c PE=1 SV=1	EVDEQMLNVQNK
>	sp	Q6P9T8	TBI Tubulin beta-2C chain OS=Rattus norvegicus GN=Tubb2c PE=1 SV=1	VSDTVVEPYNATLSVHQLVENTDETYCIDNEALYDICFR
>	sp	Q6P9T8	TBI Tubulin beta-2C chain OS=Rattus norvegicus GN=Tubb2c PE=1 SV=1	GHYTEGAELVDSVLDVVR
>	sp	Q6P9T8	TBI Tubulin beta-2C chain OS=Rattus norvegicus GN=Tubb2c PE=1 SV=1	FWEVISDEHGIDPTGTYHGDSDLQLER
>	sp	Q6P9T8	TBI Tubulin beta-2C chain OS=Rattus norvegicus GN=Tubb2c PE=1 SV=1	VVPSPKVSDTVVEPYNATLSVHQLVENTDETYCIDNEALYDICFR
>	sp	Q6P9T8	TBI Tubulin beta-2C chain OS=Rattus norvegicus GN=Tubb2c PE=1 SV=1	FWEVISDEHGIDPTGTYHGDSDLQLER
>	sp	P62632	EF1 Elongation factor 1-alpha 2 OS=Rattus norvegicus GN=Eef1a2 PE=2 SV=1	ADPPQEAAQFTSQVIILNHPGQISAGYSPVIDCHTAHIACK
>	sp	P62632	EF1 Elongation factor 1-alpha 2 OS=Rattus norvegicus GN=Eef1a2 PE=2 SV=1	EGNASGVSLLEALDTILPPTRPTDKPLRLPLQDVYK
>	sp	P62632	EF1 Elongation factor 1-alpha 2 OS=Rattus norvegicus GN=Eef1a2 PE=2 SV=1	KEGNASGVSLLEALDTILPPTRPTDKPLR
>	sp	P62632	EF1 Elongation factor 1-alpha 2 OS=Rattus norvegicus GN=Eef1a2 PE=2 SV=1	KEGNASGVSLLEALDTILPPTRPTDKPLRLPLQDVYK
>	sp	P62632	EF1 Elongation factor 1-alpha 2 OS=Rattus norvegicus GN=Eef1a2 PE=2 SV=1	VETGILRPGMVVTFAPVNITTEVK
>	sp	P62632	EF1 Elongation factor 1-alpha 2 OS=Rattus norvegicus GN=Eef1a2 PE=2 SV=1	ADPPQEAAQFTSQVIILNHPGQISAGYSPVIDCHTAHIACK
>	sp	P62632	EF1 Elongation factor 1-alpha 2 OS=Rattus norvegicus GN=Eef1a2 PE=2 SV=1	ADPPQEAAQFTSQVIILNHPGQISAGYSPVIDCHTAHIACK
>	sp	P62632	EF1 Elongation factor 1-alpha 2 OS=Rattus norvegicus GN=Eef1a2 PE=2 SV=1	ADPPQEAAQFTSQVIILNHPGQISAGYSPVIDCHTAHIACK
>	sp	P62632	EF1 Elongation factor 1-alpha 2 OS=Rattus norvegicus GN=Eef1a2 PE=2 SV=1	ADPPQEAAQFTSQVIILNHPGQISAGYSPVIDCHTAHIACK
>	sp	P62632	EF1 Elongation factor 1-alpha 2 OS=Rattus norvegicus GN=Eef1a2 PE=2 SV=1	ADPPQEAAQFTSQVIILNHPGQISAGYSPVIDCHTAHIACK
>	sp	P62632	EF1 Elongation factor 1-alpha 2 OS=Rattus norvegicus GN=Eef1a2 PE=2 SV=1	ADPPQEAAQFTSQVIILNHPGQISAGYSPVIDCHTAHIACK
>	sp	P62632	EF1 Elongation factor 1-alpha 2 OS=Rattus norvegicus GN=Eef1a2 PE=2 SV=1	ADPPQEAAQFTSQVIILNHPGQISAGYSPVIDCHTAHIACK
>	sp	P62632	EF1 Elongation factor 1-alpha 2 OS=Rattus norvegicus GN=Eef1a2 PE=2 SV=1	ADPPQEAAQFTSQVIILNHPGQISAGYSPVIDCHTAHIACK
>	sp	P62632	EF1 Elongation factor 1-alpha 2 OS=Rattus norvegicus GN=Eef1a2 PE=2 SV=1	EGNASGVSLLEALDTILPPTRPTDKPLRLPLQDVYK
>	sp	P62632	EF1 Elongation factor 1-alpha 2 OS=Rattus norvegicus GN=Eef1a2 PE=2 SV=1	EGNASGVSLLEALDTILPPTRPTDKPLRLPLQDVYK
>	sp	P62632	EF1 Elongation factor 1-alpha 2 OS=Rattus norvegicus GN=Eef1a2 PE=2 SV=1	EGNASGVSLLEALDTILPPTRPTDKPLRLPLQDVYK
>	sp	P62632	EF1 Elongation factor 1-alpha 2 OS=Rattus norvegicus GN=Eef1a2 PE=2 SV=1	EGNASGVSLLEALDTILPPTRPTDKPLRLPLQDVYK
>	sp	P62632	EF1 Elongation factor 1-alpha 2 OS=Rattus norvegicus GN=Eef1a2 PE=2 SV=1	EGNASGVSLLEALDTILPPTRPTDKPLRLPLQDVYK
>	sp	P62632	EF1 Elongation factor 1-alpha 2 OS=Rattus norvegicus GN=Eef1a2 PE=2 SV=1	EGNASGVSLLEALDTILPPTRPTDKPLRLPLQDVYK
>	sp	P62632	EF1 Elongation factor 1-alpha 2 OS=Rattus norvegicus GN=Eef1a2 PE=2 SV=1	EGNASGVSLLEALDTILPPTRPTDKPLRLPLQDVYK
>	sp	P62632	EF1 Elongation factor 1-alpha 2 OS=Rattus norvegicus GN=Eef1a2 PE=2 SV=1	EGNASGVSLLEALDTILPPTRPTDKPLRLPLQDVYK
>	sp	P62632	EF1 Elongation factor 1-alpha 2 OS=Rattus norvegicus GN=Eef1a2 PE=2 SV=1	EGNASGVSLLEALDTILPPTRPTDKPLRLPLQDVYK
>	sp	P62632	EF1 Elongation factor 1-alpha 2 OS=Rattus norvegicus GN=Eef1a2 PE=2 SV=1	EGNASGVSLLEALDTILPPTRPTDKPLRLPLQDVYK
>	sp	P62632	EF1 Elongation factor 1-alpha 2 OS=Rattus norvegicus GN=Eef1a2 PE=2 SV=1	EGNASGVSLLEALDTILPPTRPTDKPLRLPLQDVYK
>	sp	P62632	EF1 Elongation factor 1-alpha 2 OS=Rattus norvegicus GN=Eef1a2 PE=2 SV=1	EHALLAYTLGVK
>	sp	P62632	EF1 Elongation factor 1-alpha 2 OS=Rattus norvegicus GN=Eef1a2 PE=2 SV=1	EHALLAYTLGVK
>	sp	P62632	EF1 Elongation factor 1-alpha 2 OS=Rattus norvegicus GN=Eef1a2 PE=2 SV=1	EHALLAYTLGVK
>	sp	P62632	EF1 Elongation factor 1-alpha 2 OS=Rattus norvegicus GN=Eef1a2 PE=2 SV=1	GSFKYAWVLDKLK
>	sp	P62632	EF1 Elongation factor 1-alpha 2 OS=Rattus norvegicus GN=Eef1a2 PE=2 SV=1	GSFKYAWVLDKLK
>	sp	P62632	EF1 Elongation factor 1-alpha 2 OS=Rattus norvegicus GN=Eef1a2 PE=2 SV=1	IGGIGTVPVGR
>	sp	P62632	EF1 Elongation factor 1-alpha 2 OS=Rattus norvegicus GN=Eef1a2 PE=2 SV=1	KEGNASGVSLLEALDTILPPTRPTDKPLR
>	sp	P62632	EF1 Elongation factor 1-alpha 2 OS=Rattus norvegicus GN=Eef1a2 PE=2 SV=1	KEGNASGVSLLEALDTILPPTRPTDKPLR
>	sp	P62632	EF1 Elongation factor 1-alpha 2 OS=Rattus norvegicus GN=Eef1a2 PE=2 SV=1	KEGNASGVSLLEALDTILPPTRPTDKPLR
>	sp	P62632	EF1 Elongation factor 1-alpha 2 OS=Rattus norvegicus GN=Eef1a2 PE=2 SV=1	KEGNASGVSLLEALDTILPPTRPTDKPLR
>	sp	P62632	EF1 Elongation factor 1-alpha 2 OS=Rattus norvegicus GN=Eef1a2 PE=2 SV=1	KEGNASGVSLLEALDTILPPTRPTDKPLRLPLQDVYK
>	sp	P62632	EF1 Elongation factor 1-alpha 2 OS=Rattus norvegicus GN=Eef1a2 PE=2 SV=1	KEGNASGVSLLEALDTILPPTRPTDKPLRLPLQDVYK
>	sp	P62632	EF1 Elongation factor 1-alpha 2 OS=Rattus norvegicus GN=Eef1a2 PE=2 SV=1	KEGNASGVSLLEALDTILPPTRPTDKPLRLPLQDVYK
>	sp	P62632	EF1 Elongation factor 1-alpha 2 OS=Rattus norvegicus GN=Eef1a2 PE=2 SV=1	KEGNASGVSLLEALDTILPPTRPTDKPLRLPLQDVYK

>	sp|P62632|EF1	Elongation factor 1-alpha 2 OS=Rattus norvegicus GN=Eef1a2 PE=2 SV=1	KEGNASGVSLLEALDTILPPTRPTDKPLRLPLQDVYK
>	sp|P62632|EF1	Elongation factor 1-alpha 2 OS=Rattus norvegicus GN=Eef1a2 PE=2 SV=1	KEGNASGVSLLEALDTILPPTRPTDKPLRLPLQDVYK
>	sp|P62632|EF1	Elongation factor 1-alpha 2 OS=Rattus norvegicus GN=Eef1a2 PE=2 SV=1	KEGNASGVSLLEALDTILPPTRPTDKPLRLPLQDVYK
>	sp|P62632|EF1	Elongation factor 1-alpha 2 OS=Rattus norvegicus GN=Eef1a2 PE=2 SV=1	KEGNASGVSLLEALDTILPPTRPTDKPLRLPLQDVYK
>	sp|P62632|EF1	Elongation factor 1-alpha 2 OS=Rattus norvegicus GN=Eef1a2 PE=2 SV=1	KEGNASGVSLLEALDTILPPTRPTDKPLRLPLQDVYK
>	sp|P62632|EF1	Elongation factor 1-alpha 2 OS=Rattus norvegicus GN=Eef1a2 PE=2 SV=1	KEGNASGVSLLEALDTILPPTRPTDKPLRLPLQDVYK
>	sp|P62632|EF1	Elongation factor 1-alpha 2 OS=Rattus norvegicus GN=Eef1a2 PE=2 SV=1	KEGNASGVSLLEALDTILPPTRPTDKPLRLPLQDVYK
>	sp|P62632|EF1	Elongation factor 1-alpha 2 OS=Rattus norvegicus GN=Eef1a2 PE=2 SV=1	KEGNASGVSLLEALDTILPPTRPTDKPLRLPLQDVYK
>	sp|P62632|EF1	Elongation factor 1-alpha 2 OS=Rattus norvegicus GN=Eef1a2 PE=2 SV=1	VETGILRPGMVVTFAPVNITTEVK
>	sp|P62632|EF1	Elongation factor 1-alpha 2 OS=Rattus norvegicus GN=Eef1a2 PE=2 SV=1	VETGILRPGMVVTFAPVNITTEVK
>	sp|P62632|EF1	Elongation factor 1-alpha 2 OS=Rattus norvegicus GN=Eef1a2 PE=2 SV=1	VETGILRPGMVVTFAPVNITTEVK
>	sp|P62632|EF1	Elongation factor 1-alpha 2 OS=Rattus norvegicus GN=Eef1a2 PE=2 SV=1	VETGILRPGMVVTFAPVNITTEVK
>	sp|P62632|EF1	Elongation factor 1-alpha 2 OS=Rattus norvegicus GN=Eef1a2 PE=2 SV=1	VETGILRPGMVVTFAPVNITTEVK
>	sp|P62632|EF1	Elongation factor 1-alpha 2 OS=Rattus norvegicus GN=Eef1a2 PE=2 SV=1	IGGIGTVPVGR
>	sp|P62632|EF1	Elongation factor 1-alpha 2 OS=Rattus norvegicus GN=Eef1a2 PE=2 SV=1	KEGNASGVSLLEALDTILPPTRPTDKPLRLPLQDVYK
>	sp|P62632|EF1	Elongation factor 1-alpha 2 OS=Rattus norvegicus GN=Eef1a2 PE=2 SV=1	IGYNPATVPFVPISGWHGDNMLEPSPNMPWFK
>	sp|P62632|EF1	Elongation factor 1-alpha 2 OS=Rattus norvegicus GN=Eef1a2 PE=2 SV=1	VETGILRPGMVVTFAPVNITTEVK
>	sp|P62632|EF1	Elongation factor 1-alpha 2 OS=Rattus norvegicus GN=Eef1a2 PE=2 SV=1	VETGILRPGMVVTFAPVNITTEVK
>	sp|P62632|EF1	Elongation factor 1-alpha 2 OS=Rattus norvegicus GN=Eef1a2 PE=2 SV=1	GSFKYAWVLDKLK
>	sp|P62632|EF1	Elongation factor 1-alpha 2 OS=Rattus norvegicus GN=Eef1a2 PE=2 SV=1	EGNASGVSLLEALDTILPPTRPTDKPLRLPLQDVYK
>	sp|P62632|EF1	Elongation factor 1-alpha 2 OS=Rattus norvegicus GN=Eef1a2 PE=2 SV=1	IGGIGTVPVGR
>	sp|P62632|EF1	Elongation factor 1-alpha 2 OS=Rattus norvegicus GN=Eef1a2 PE=2 SV=1	ADPPQEAAQFTSQVIILNHPGQISAGYSPVIDCHTAHIACK
>	sp|P62632|EF1	Elongation factor 1-alpha 2 OS=Rattus norvegicus GN=Eef1a2 PE=2 SV=1	KEGNASGVSLLEALDTILPPTRPTDKPLRLPLQDVYK
>	sp|P62632|EF1	Elongation factor 1-alpha 2 OS=Rattus norvegicus GN=Eef1a2 PE=2 SV=1	EHALLAYTLGVK
>	sp|P62632|EF1	Elongation factor 1-alpha 2 OS=Rattus norvegicus GN=Eef1a2 PE=2 SV=1	VETGILRPGMVVTFAPVNITTEVK
>	sp|P62632|EF1	Elongation factor 1-alpha 2 OS=Rattus norvegicus GN=Eef1a2 PE=2 SV=1	IGGIGTVPVGR
>	sp|P62632|EF1	Elongation factor 1-alpha 2 OS=Rattus norvegicus GN=Eef1a2 PE=2 SV=1	KEGNASGVSLLEALDTILPPTRPTDKPLR
>	sp|P62632|EF1	Elongation factor 1-alpha 2 OS=Rattus norvegicus GN=Eef1a2 PE=2 SV=1	KEGNASGVSLLEALDTILPPTRPTDKPLRLPLQDVYK
>	sp|Q00981|UC	Ubiquitin carboxyl-terminal hydrolase isozyme L1 OS=Rattus norvegicus GN=Uchl1 PE=1 SV=2	MQLKPMEINPEMLNK
>	sp|Q00981|UC	Ubiquitin carboxyl-terminal hydrolase isozyme L1 OS=Rattus norvegicus GN=Uchl1 PE=1 SV=2	MQLKPMEINPEMLNKVLAKLGVAGQWR
>	sp|Q00981|UC	Ubiquitin carboxyl-terminal hydrolase isozyme L1 OS=Rattus norvegicus GN=Uchl1 PE=1 SV=2	VDDKVNFHFILFNNVDGHLYELDGR
>	sp|Q00981|UC	Ubiquitin carboxyl-terminal hydrolase isozyme L1 OS=Rattus norvegicus GN=Uchl1 PE=1 SV=2	NEAIQAAHDSVAQEGQCR
>	sp|Q00981|UC	Ubiquitin carboxyl-terminal hydrolase isozyme L1 OS=Rattus norvegicus GN=Uchl1 PE=1 SV=2	QIEELKGQEVSPK
>	sp|Q00981|UC	Ubiquitin carboxyl-terminal hydrolase isozyme L1 OS=Rattus norvegicus GN=Uchl1 PE=1 SV=2	MQLKPMEINPEMLNK
>	sp|Q00981|UC	Ubiquitin carboxyl-terminal hydrolase isozyme L1 OS=Rattus norvegicus GN=Uchl1 PE=1 SV=2	MQLKPMEINPEMLNKVLAKLGVAGQWR
>	sp|Q00981|UC	Ubiquitin carboxyl-terminal hydrolase isozyme L1 OS=Rattus norvegicus GN=Uchl1 PE=1 SV=2	MQLKPMEINPEMLNKVLAKLGVAGQWR
>	sp|Q00981|UC	Ubiquitin carboxyl-terminal hydrolase isozyme L1 OS=Rattus norvegicus GN=Uchl1 PE=1 SV=2	NEAIQAAHDSVAQEGQCR
>	sp|Q00981|UC	Ubiquitin carboxyl-terminal hydrolase isozyme L1 OS=Rattus norvegicus GN=Uchl1 PE=1 SV=2	NEAIQAAHDSVAQEGQCR
>	sp|Q00981|UC	Ubiquitin carboxyl-terminal hydrolase isozyme L1 OS=Rattus norvegicus GN=Uchl1 PE=1 SV=2	VDDKVNFHFILFNNVDGHLYELDGR
>	sp|Q00981|UC	Ubiquitin carboxyl-terminal hydrolase isozyme L1 OS=Rattus norvegicus GN=Uchl1 PE=1 SV=2	VDDKVNFHFILFNNVDGHLYELDGR
>	sp|Q00981|UC	Ubiquitin carboxyl-terminal hydrolase isozyme L1 OS=Rattus norvegicus GN=Uchl1 PE=1 SV=2	VDDKVNFHFILFNNVDGHLYELDGR
>	sp|Q00981|UC	Ubiquitin carboxyl-terminal hydrolase isozyme L1 OS=Rattus norvegicus GN=Uchl1 PE=1 SV=2	VDDKVNFHFILFNNVDGHLYELDGR
>	sp|Q00981|UC	Ubiquitin carboxyl-terminal hydrolase isozyme L1 OS=Rattus norvegicus GN=Uchl1 PE=1 SV=2	VDDKVNFHFILFNNVDGHLYELDGR
>	sp|Q00981|UC	Ubiquitin carboxyl-terminal hydrolase isozyme L1 OS=Rattus norvegicus GN=Uchl1 PE=1 SV=2	VDDKVNFHFILFNNVDGHLYELDGR
>	sp|Q00981|UC	Ubiquitin carboxyl-terminal hydrolase isozyme L1 OS=Rattus norvegicus GN=Uchl1 PE=1 SV=2	VDDKVNFHFILFNNVDGHLYELDGR
>	sp|Q00981|UC	Ubiquitin carboxyl-terminal hydrolase isozyme L1 OS=Rattus norvegicus GN=Uchl1 PE=1 SV=2	VDDKVNFHFILFNNVDGHLYELDGR
>	sp|Q00981|UC	Ubiquitin carboxyl-terminal hydrolase isozyme L1 OS=Rattus norvegicus GN=Uchl1 PE=1 SV=2	VDDKVNFHFILFNNVDGHLYELDGR
>	sp|Q00981|UC	Ubiquitin carboxyl-terminal hydrolase isozyme L1 OS=Rattus norvegicus GN=Uchl1 PE=1 SV=2	VDDKVNFHFILFNNVDGHLYELDGR
>	sp|Q00981|UC	Ubiquitin carboxyl-terminal hydrolase isozyme L1 OS=Rattus norvegicus GN=Uchl1 PE=1 SV=2	VDDKVNFHFILFNNVDGHLYELDGR
>	sp|Q00981|UC	Ubiquitin carboxyl-terminal hydrolase isozyme L1 OS=Rattus norvegicus GN=Uchl1 PE=1 SV=2	VDDKVNFHFILFNNVDGHLYELDGR
>	sp|Q00981|UC	Ubiquitin carboxyl-terminal hydrolase isozyme L1 OS=Rattus norvegicus GN=Uchl1 PE=1 SV=2	VDDKVNFHFILFNNVDGHLYELDGR
>	sp|Q00981|UC	Ubiquitin carboxyl-terminal hydrolase isozyme L1 OS=Rattus norvegicus GN=Uchl1 PE=1 SV=2	VDDKVNFHFILFNNVDGHLYELDGR
>	sp|Q00981|UC	Ubiquitin carboxyl-terminal hydrolase isozyme L1 OS=Rattus norvegicus GN=Uchl1 PE=1 SV=2	VDDKVNFHFILFNNVDGHLYELDGR
>	sp|Q00981|UC	Ubiquitin carboxyl-terminal hydrolase isozyme L1 OS=Rattus norvegicus GN=Uchl1 PE=1 SV=2	VDDKVNFHFILFNNVDGHLYELDGR
>	sp|Q00981|UC	Ubiquitin carboxyl-terminal hydrolase isozyme L1 OS=Rattus norvegicus GN=Uchl1 PE=1 SV=2	VDDKVNFHFILFNNVDGHLYELDGR
>	sp|Q00981|UC	Ubiquitin carboxyl-terminal hydrolase isozyme L1 OS=Rattus norvegicus GN=Uchl1 PE=1 SV=2	VDDKVNFHFILFNNVDGHLYELDGR
>	sp|Q00981|UC	Ubiquitin carboxyl-terminal hydrolase isozyme L1 OS=Rattus norvegicus GN=Uchl1 PE=1 SV=2	VDDKVNFHFILFNNVDGHLYELDGR
>	sp|Q00981|UC	Ubiquitin carboxyl-terminal hydrolase isozyme L1 OS=Rattus norvegicus GN=Uchl1 PE=1 SV=2	VDDKVNFHFILFNNVDGHLYELDGR
>	sp|Q00981|UC	Ubiquitin carboxyl-terminal hydrolase isozyme L1 OS=Rattus norvegicus GN=Uchl1 PE=1 SV=2	VDDKVNFHFILFNNVDGHLYELDGR
>	sp|Q00981|UC	Ubiquitin carboxyl-terminal hydrolase isozyme L1 OS=Rattus norvegicus GN=Uchl1 PE=1 SV=2	VDDKVNFHFILFNNVDGHLYELDGR
>	sp|Q00981|UC	Ubiquitin carboxyl-terminal hydrolase isozyme L1 OS=Rattus norvegicus GN=Uchl1 PE=1 SV=2	MQLKPMEINPEMLNK
>	sp|Q00981|UC	Ubiquitin carboxyl-terminal hydrolase isozyme L1 OS=Rattus norvegicus GN=Uchl1 PE=1 SV=2	MQLKPMEINPEMLNK
>	sp|Q00981|UC	Ubiquitin carboxyl-terminal hydrolase isozyme L1 OS=Rattus norvegicus GN=Uchl1 PE=1 SV=2	MPFPVNHGASSEDSLLQDAAK
>	sp|Q00981|UC	Ubiquitin carboxyl-terminal hydrolase isozyme L1 OS=Rattus norvegicus GN=Uchl1 PE=1 SV=2	MQLKPMEINPEMLNK
>	sp|Q00981|UC	Ubiquitin carboxyl-terminal hydrolase isozyme L1 OS=Rattus norvegicus GN=Uchl1 PE=1 SV=2	MQLKPMEINPEMLNKVLAKLGVAGQWR
>	sp|Q00981|UC	Ubiquitin carboxyl-terminal hydrolase isozyme L1 OS=Rattus norvegicus GN=Uchl1 PE=1 SV=2	FSAVALCK
>	sp|Q00981|UC	Ubiquitin carboxyl-terminal hydrolase isozyme L1 OS=Rattus norvegicus GN=Uchl1 PE=1 SV=2	FSAVALCK
>	sp|Q00981|UC	Ubiquitin carboxyl-terminal hydrolase isozyme L1 OS=Rattus norvegicus GN=Uchl1 PE=1 SV=2	MPFPVNHGASSEDSLLQDAAK
>	sp|Q00981|UC	Ubiquitin carboxyl-terminal hydrolase isozyme L1 OS=Rattus norvegicus GN=Uchl1 PE=1 SV=2	MQLKPMEINPEMLNKVLAKLGVAGQWR
>	sp|Q00981|UC	Ubiquitin carboxyl-terminal hydrolase isozyme L1 OS=Rattus norvegicus GN=Uchl1 PE=1 SV=2	MPFPVNHGASSEDSLLQDAAK
>	sp|Q66HF1|ND	NADH-ubiquinone oxidoreductase 75 kDa subunit, mitochondrial OS=Rattus norvegicus GN=Ndufs1 PE=1 SV=1	ALSEIAGITLPYDTLDQVR
>	sp|Q66HF1|ND	NADH-ubiquinone oxidoreductase 75 kDa subunit, mitochondrial OS=Rattus norvegicus GN=Ndufs1 PE=1 SV=1	DDGAAILAAVSSIAQK
>	sp|Q66HF1|ND	NADH-ubiquinone oxidoreductase 75 kDa subunit, mitochondrial OS=Rattus norvegicus GN=Ndufs1 PE=1 SV=1	KPMVVLGSSALQR
>	sp|Q66HF1|ND	NADH-ubiquinone oxidoreductase 75 kDa subunit, mitochondrial OS=Rattus norvegicus GN=Ndufs1 PE=1 SV=1	LLFLLGADGGCITR

>	sp|Q66HF1|ND	NADH-ubiquinone oxidoreductase 75 kDa subunit, mitochondrial OS=Rattus norvegicus GN=Ndufs1 PE=1 SV=1	ALSEIAGITLPYDTLDQVR
>	sp|Q66HF1|ND	NADH-ubiquinone oxidoreductase 75 kDa subunit, mitochondrial OS=Rattus norvegicus GN=Ndufs1 PE=1 SV=1	ALSEIAGITLPYDTLDQVR
>	sp|Q66HF1|ND	NADH-ubiquinone oxidoreductase 75 kDa subunit, mitochondrial OS=Rattus norvegicus GN=Ndufs1 PE=1 SV=1	DDGAAILAAVSSIAQK
>	sp|Q66HF1|ND	NADH-ubiquinone oxidoreductase 75 kDa subunit, mitochondrial OS=Rattus norvegicus GN=Ndufs1 PE=1 SV=1	DDGAAILAAVSSIAQK
>	sp|Q66HF1|ND	NADH-ubiquinone oxidoreductase 75 kDa subunit, mitochondrial OS=Rattus norvegicus GN=Ndufs1 PE=1 SV=1	DDGAAILAAVSSIAQK
>	sp|Q66HF1|ND	NADH-ubiquinone oxidoreductase 75 kDa subunit, mitochondrial OS=Rattus norvegicus GN=Ndufs1 PE=1 SV=1	MFMSELSGNIIDICPVGALTSKPYAFTARPWETR
>	sp|Q66HF1|ND	NADH-ubiquinone oxidoreductase 75 kDa subunit, mitochondrial OS=Rattus norvegicus GN=Ndufs1 PE=1 SV=1	DDGAAILAAVSSIAQK
>	sp|Q66HF1|ND	NADH-ubiquinone oxidoreductase 75 kDa subunit, mitochondrial OS=Rattus norvegicus GN=Ndufs1 PE=1 SV=1	DDGAAILAAVSSIAQK
>	sp|Q66HF1|ND	NADH-ubiquinone oxidoreductase 75 kDa subunit, mitochondrial OS=Rattus norvegicus GN=Ndufs1 PE=1 SV=1	IASQVAALDLGYKPGVEAIR
>	sp|Q63610|TPM	Tropomyosin alpha-3 chain OS=Rattus norvegicus GN=Tpm3 PE=1 SV=2	KIQVLQQQADDAEER
>	sp|Q63610|TPM	Tropomyosin alpha-3 chain OS=Rattus norvegicus GN=Tpm3 PE=1 SV=2	KLVIIEGDLER
>	sp|Q63610|TPM	Tropomyosin alpha-3 chain OS=Rattus norvegicus GN=Tpm3 PE=1 SV=2	RIQLVEEELDR
>	sp|Q63610|TPM	Tropomyosin alpha-3 chain OS=Rattus norvegicus GN=Tpm3 PE=1 SV=2	LATALQKLEEAEK
>	sp|Q63610|TPM	Tropomyosin alpha-3 chain OS=Rattus norvegicus GN=Tpm3 PE=1 SV=2	KIQVLQQQADDAEER
>	sp|Q63610|TPM	Tropomyosin alpha-3 chain OS=Rattus norvegicus GN=Tpm3 PE=1 SV=2	KIQVLQQQADDAEER
>	sp|Q63610|TPM	Tropomyosin alpha-3 chain OS=Rattus norvegicus GN=Tpm3 PE=1 SV=2	KLVIIEGDLER
>	sp|Q63610|TPM	Tropomyosin alpha-3 chain OS=Rattus norvegicus GN=Tpm3 PE=1 SV=2	KLVIIEGDLER
>	sp|Q63610|TPM	Tropomyosin alpha-3 chain OS=Rattus norvegicus GN=Tpm3 PE=1 SV=2	KLVIIEGDLER
>	sp|Q63610|TPM	Tropomyosin alpha-3 chain OS=Rattus norvegicus GN=Tpm3 PE=1 SV=2	TIDDLEDKLK
>	sp|Q63610|TPM	Tropomyosin alpha-3 chain OS=Rattus norvegicus GN=Tpm3 PE=1 SV=2	KLVIIEGDLER
>	sp|Q63610|TPM	Tropomyosin alpha-3 chain OS=Rattus norvegicus GN=Tpm3 PE=1 SV=2	KLVIIEGDLER
>	sp|Q63610|TPM	Tropomyosin alpha-3 chain OS=Rattus norvegicus GN=Tpm3 PE=1 SV=2	MELQEIQLK
>	sp|P32851|STX	Syntaxin-1A OS=Rattus norvegicus GN=Stx1a PE=1 SV=1	SIEQSIEQEEGLNR
>	sp|P32851|STX	Syntaxin-1A OS=Rattus norvegicus GN=Stx1a PE=1 SV=1	TAKDSDDDDDVTVTVDRDR
>	sp|P32851|STX	Syntaxin-1A OS=Rattus norvegicus GN=Stx1a PE=1 SV=1	TKEELEELMSDIKK
>	sp|P32851|STX	Syntaxin-1A OS=Rattus norvegicus GN=Stx1a PE=1 SV=1	KHSAILASPNPDEK
>	sp|P32851|STX	Syntaxin-1A OS=Rattus norvegicus GN=Stx1a PE=1 SV=1	TAKDSDDDDDVTVTVDRDR
>	sp|P32851|STX	Syntaxin-1A OS=Rattus norvegicus GN=Stx1a PE=1 SV=1	TAKDSDDDDDVTVTVDRDR
>	sp|P32851|STX	Syntaxin-1A OS=Rattus norvegicus GN=Stx1a PE=1 SV=1	KFVEVMSEYNATQSDYR
>	sp|P32851|STX	Syntaxin-1A OS=Rattus norvegicus GN=Stx1a PE=1 SV=1	TKEELEELMSDIK
>	sp|P32851|STX	Syntaxin-1A OS=Rattus norvegicus GN=Stx1a PE=1 SV=1	TKEELEELMSDIK
>	sp|P32851|STX	Syntaxin-1A OS=Rattus norvegicus GN=Stx1a PE=1 SV=1	TTTSEELEDMLESGNPAIFASGIIMDSSISK
>	sp|P32851|STX	Syntaxin-1A OS=Rattus norvegicus GN=Stx1a PE=1 SV=1	KHSAILASPNPDEK
>	sp|P32851|STX	Syntaxin-1A OS=Rattus norvegicus GN=Stx1a PE=1 SV=1	IEYNVEHAVDYVER
>	sp|Q9WVC0|SE	Septin-7 OS=Rattus norvegicus GN=Sept7 PE=1 SV=1	LKDSEAELQR
>	sp|Q9WVC0|SE	Septin-7 OS=Rattus norvegicus GN=Sept7 PE=1 SV=1	QYPWGVAEVENGEHCDFTILR
>	sp|Q9WVC0|SE	Septin-7 OS=Rattus norvegicus GN=Sept7 PE=1 SV=1	MEMEMEQVFEMK
>	sp|Q9WVC0|SE	Septin-7 OS=Rattus norvegicus GN=Sept7 PE=1 SV=1	NLEAQHKELEEK
>	sp|Q9WVC0|SE	Septin-7 OS=Rattus norvegicus GN=Sept7 PE=1 SV=1	VNIIPLIAK
>	sp|Q9WVC0|SE	Septin-7 OS=Rattus norvegicus GN=Sept7 PE=1 SV=1	VNIIPLIAK
>	sp|Q9WVC0|SE	Septin-7 OS=Rattus norvegicus GN=Sept7 PE=1 SV=1	LKDSEAELQR
>	sp|Q9WVC0|SE	Septin-7 OS=Rattus norvegicus GN=Sept7 PE=1 SV=1	NLEAQHK
>	sp|Q9WVC0|SE	Septin-7 OS=Rattus norvegicus GN=Sept7 PE=1 SV=1	IYEFPETDDEEENKLVK
>	sp|Q9WVC0|SE	Septin-7 OS=Rattus norvegicus GN=Sept7 PE=1 SV=1	KLAAVTYNGVDNNKNKGQLTK
>	sp|P84092|AP2	AP-2 complex subunit mu OS=Rattus norvegicus GN=Ap2m1 PE=1 SV=1	NELFLDVLESVNLLMSPQGQVLSAHVSGR
>	sp|P84092|AP2	AP-2 complex subunit mu OS=Rattus norvegicus GN=Ap2m1 PE=1 SV=1	QSIAIDDCTFHQCVR
>	sp|P84092|AP2	AP-2 complex subunit mu OS=Rattus norvegicus GN=Ap2m1 PE=1 SV=1	RNELFLDVLESVNLLMSPQGQVLSAHVSGR
>	sp|P84092|AP2	AP-2 complex subunit mu OS=Rattus norvegicus GN=Ap2m1 PE=1 SV=1	WARPPISMNFEVPFAPSGLK
>	sp|P84092|AP2	AP-2 complex subunit mu OS=Rattus norvegicus GN=Ap2m1 PE=1 SV=1	NELFLDVLESVNLLMSPQGQVLSAHVSGR
>	sp|P84092|AP2	AP-2 complex subunit mu OS=Rattus norvegicus GN=Ap2m1 PE=1 SV=1	NELFLDVLESVNLLMSPQGQVLSAHVSGR
>	sp|P84092|AP2	AP-2 complex subunit mu OS=Rattus norvegicus GN=Ap2m1 PE=1 SV=1	NELFLDVLESVNLLMSPQGQVLSAHVSGR
>	sp|P84092|AP2	AP-2 complex subunit mu OS=Rattus norvegicus GN=Ap2m1 PE=1 SV=1	RNELFLDVLESVNLLMSPQGQVLSAHVSGR
>	sp|P84092|AP2	AP-2 complex subunit mu OS=Rattus norvegicus GN=Ap2m1 PE=1 SV=1	RNELFLDVLESVNLLMSPQGQVLSAHVSGR
>	sp|P84092|AP2	AP-2 complex subunit mu OS=Rattus norvegicus GN=Ap2m1 PE=1 SV=1	RNELFLDVLESVNLLMSPQGQVLSAHVSGR
>	sp|P84092|AP2	AP-2 complex subunit mu OS=Rattus norvegicus GN=Ap2m1 PE=1 SV=1	RNELFLDVLESVNLLMSPQGQVLSAHVSGR
>	sp|P84092|AP2	AP-2 complex subunit mu OS=Rattus norvegicus GN=Ap2m1 PE=1 SV=1	RNELFLDVLESVNLLMSPQGQVLSAHVSGR
>	sp|P84092|AP2	AP-2 complex subunit mu OS=Rattus norvegicus GN=Ap2m1 PE=1 SV=1	WARPPISMNFEVPFAPSGLK
>	sp|P06761|GRP	78 kDa glucose-regulated protein OS=Rattus norvegicus GN=Hspa5 PE=1 SV=1	KSDIDEIVLVGGSTR
>	sp|P06761|GRP	78 kDa glucose-regulated protein OS=Rattus norvegicus GN=Hspa5 PE=1 SV=1	TFAPEEISAMVLTK
>	sp|P06761|GRP	78 kDa glucose-regulated protein OS=Rattus norvegicus GN=Hspa5 PE=1 SV=1	AKFEELNMDLFR
>	sp|P06761|GRP	78 kDa glucose-regulated protein OS=Rattus norvegicus GN=Hspa5 PE=1 SV=1	VTHAVVTVPAYFNDAQR
>	sp|P06761|GRP	78 kDa glucose-regulated protein OS=Rattus norvegicus GN=Hspa5 PE=1 SV=1	AKFEELNMDLFR
>	sp|P06761|GRP	78 kDa glucose-regulated protein OS=Rattus norvegicus GN=Hspa5 PE=1 SV=1	AKFEELNMDLFR
>	sp|P06761|GRP	78 kDa glucose-regulated protein OS=Rattus norvegicus GN=Hspa5 PE=1 SV=1	IINEPTAAAIAYGLDK
>	sp|P06761|GRP	78 kDa glucose-regulated protein OS=Rattus norvegicus GN=Hspa5 PE=1 SV=1	VEIIANDQGNR
>	sp|P06761|GRP	78 kDa glucose-regulated protein OS=Rattus norvegicus GN=Hspa5 PE=1 SV=1	VEIIANDQGNR
>	sp|P06761|GRP	78 kDa glucose-regulated protein OS=Rattus norvegicus GN=Hspa5 PE=1 SV=1	VEIIANDQGNR
>	sp|P06761|GRP	78 kDa glucose-regulated protein OS=Rattus norvegicus GN=Hspa5 PE=1 SV=1	TFAPEEISAMVLTK
>	sp|P06761|GRP	78 kDa glucose-regulated protein OS=Rattus norvegicus GN=Hspa5 PE=1 SV=1	IINEPTAAAIAYGLDK
>	sp|P06761|GRP	78 kDa glucose-regulated protein OS=Rattus norvegicus GN=Hspa5 PE=1 SV=1	IINEPTAAAIAYGLDKR
>	sp|P06761|GRP	78 kDa glucose-regulated protein OS=Rattus norvegicus GN=Hspa5 PE=1 SV=1	IINEPTAAAIAYGLDK
>	sp|P06761|GRP	78 kDa glucose-regulated protein OS=Rattus norvegicus GN=Hspa5 PE=1 SV=1	VEIIANDQGNR
>	sp|P06761|GRP	78 kDa glucose-regulated protein OS=Rattus norvegicus GN=Hspa5 PE=1 SV=1	LIGDAAK
>	sp|P06761|GRP	78 kDa glucose-regulated protein OS=Rattus norvegicus GN=Hspa5 PE=1 SV=1	AKFEELNMDLFR

>	sp|P06761|GRI	78 kDa glucose-regulated protein OS=Rattus norvegicus GN=Hspa5 PE=1 SV=1	VMEHFIK
>	sp|P06761|GRI	78 kDa glucose-regulated protein OS=Rattus norvegicus GN=Hspa5 PE=1 SV=1	LIGDAAK
>	sp|P06761|GRI	78 kDa glucose-regulated protein OS=Rattus norvegicus GN=Hspa5 PE=1 SV=1	VTHAVVTV
>	sp|P06761|GRI	78 kDa glucose-regulated protein OS=Rattus norvegicus GN=Hspa5 PE=1 SV=1	MKETAEAYLGKK
>	sp|P22062|PIN	Protein-L-isoaspartate(D-aspartate) O-methyltransferase OS=Rattus norvegicus GN=Pcmt1 PE=1 SV=2	MGFAEEAPYDAIHVGAAAPVVPQALIDQLKPGGR
>	sp|P22062|PIN	Protein-L-isoaspartate(D-aspartate) O-methyltransferase OS=Rattus norvegicus GN=Pcmt1 PE=1 SV=2	TDKVFEVMLATDR
>	sp|P22062|PIN	Protein-L-isoaspartate(D-aspartate) O-methyltransferase OS=Rattus norvegicus GN=Pcmt1 PE=1 SV=2	VIGIDHIK
>	sp|P22062|PIN	Protein-L-isoaspartate(D-aspartate) O-methyltransferase OS=Rattus norvegicus GN=Pcmt1 PE=1 SV=2	ALDVGSGSGILTACFAR
>	sp|P22062|PIN	Protein-L-isoaspartate(D-aspartate) O-methyltransferase OS=Rattus norvegicus GN=Pcmt1 PE=1 SV=2	MKPLMGVIYVPLTDKEK
>	sp|P22062|PIN	Protein-L-isoaspartate(D-aspartate) O-methyltransferase OS=Rattus norvegicus GN=Pcmt1 PE=1 SV=2	MGFAEEAPYDAIHVGAAAPVVPQALIDQLKPGGR
>	sp|P22062|PIN	Protein-L-isoaspartate(D-aspartate) O-methyltransferase OS=Rattus norvegicus GN=Pcmt1 PE=1 SV=2	MGFAEEAPYDAIHVGAAAPVVPQALIDQLKPGGR
>	sp|P22062|PIN	Protein-L-isoaspartate(D-aspartate) O-methyltransferase OS=Rattus norvegicus GN=Pcmt1 PE=1 SV=2	MGFAEEAPYDAIHVGAAAPVVPQALIDQLKPGGR
>	sp|P22062|PIN	Protein-L-isoaspartate(D-aspartate) O-methyltransferase OS=Rattus norvegicus GN=Pcmt1 PE=1 SV=2	MGFAEEAPYDAIHVGAAAPVVPQALIDQLKPGGR
>	sp|P22062|PIN	Protein-L-isoaspartate(D-aspartate) O-methyltransferase OS=Rattus norvegicus GN=Pcmt1 PE=1 SV=2	MKPLMGVIYVPLTDKEK
>	sp|P22062|PIN	Protein-L-isoaspartate(D-aspartate) O-methyltransferase OS=Rattus norvegicus GN=Pcmt1 PE=1 SV=2	MKPLMGVIYVPLTDKEK
>	sp|P22062|PIN	Protein-L-isoaspartate(D-aspartate) O-methyltransferase OS=Rattus norvegicus GN=Pcmt1 PE=1 SV=2	TDKVFEVMLATDR
>	sp|P22062|PIN	Protein-L-isoaspartate(D-aspartate) O-methyltransferase OS=Rattus norvegicus GN=Pcmt1 PE=1 SV=2	VIGIDHIK
>	sp|P22062|PIN	Protein-L-isoaspartate(D-aspartate) O-methyltransferase OS=Rattus norvegicus GN=Pcmt1 PE=1 SV=2	VIGIDHIKELVDDSITNVK
>	sp|P22062|PIN	Protein-L-isoaspartate(D-aspartate) O-methyltransferase OS=Rattus norvegicus GN=Pcmt1 PE=1 SV=2	MKPLMGVIYVPLTDKEK
>	sp|P22062|PIN	Protein-L-isoaspartate(D-aspartate) O-methyltransferase OS=Rattus norvegicus GN=Pcmt1 PE=1 SV=2	VIGIDHIK
>	sp|P22062|PIN	Protein-L-isoaspartate(D-aspartate) O-methyltransferase OS=Rattus norvegicus GN=Pcmt1 PE=1 SV=2	MGFAEEAPYDAIHVGAAAPVVPQALIDQLKPGGR
>	sp|P22062|PIN	Protein-L-isoaspartate(D-aspartate) O-methyltransferase OS=Rattus norvegicus GN=Pcmt1 PE=1 SV=2	MGFAEEAPYDAIHVGAAAPVVPQALIDQLKPGGR
>	sp|P22062|PIN	Protein-L-isoaspartate(D-aspartate) O-methyltransferase OS=Rattus norvegicus GN=Pcmt1 PE=1 SV=2	MGFAEEAPYDAIHVGAAAPVVPQALIDQLKPGGR
>	sp|B2RYG6|OT	Ubiquitin thioesterase OTUB1 OS=Rattus norvegicus GN=Otub1 PE=1 SV=1	AFGFSHLEALLDDSK
>	sp|B2RYG6|OT	Ubiquitin thioesterase OTUB1 OS=Rattus norvegicus GN=Otub1 PE=1 SV=1	AFGFSHLEALLDDSKELQR
>	sp|B2RYG6|OT	Ubiquitin thioesterase OTUB1 OS=Rattus norvegicus GN=Otub1 PE=1 SV=1	FFEHFIEGGR
>	sp|B2RYG6|OT	Ubiquitin thioesterase OTUB1 OS=Rattus norvegicus GN=Otub1 PE=1 SV=1	IQQEIAVQNPLVSER
>	sp|B2RYG6|OT	Ubiquitin thioesterase OTUB1 OS=Rattus norvegicus GN=Otub1 PE=1 SV=1	AFGFSHLEALLDDSK
>	sp|B2RYG6|OT	Ubiquitin thioesterase OTUB1 OS=Rattus norvegicus GN=Otub1 PE=1 SV=1	AFGFSHLEALLDDSKELQR
>	sp|B2RYG6|OT	Ubiquitin thioesterase OTUB1 OS=Rattus norvegicus GN=Otub1 PE=1 SV=1	AFGFSHLEALLDDSKELQR
>	sp|B2RYG6|OT	Ubiquitin thioesterase OTUB1 OS=Rattus norvegicus GN=Otub1 PE=1 SV=1	FFEHFIEGGR
>	sp|B2RYG6|OT	Ubiquitin thioesterase OTUB1 OS=Rattus norvegicus GN=Otub1 PE=1 SV=1	AFGFSHLEALLDDSKELQR
>	sp|B2RYG6|OT	Ubiquitin thioesterase OTUB1 OS=Rattus norvegicus GN=Otub1 PE=1 SV=1	FFEHFIEGGR
>	sp|B2RYG6|OT	Ubiquitin thioesterase OTUB1 OS=Rattus norvegicus GN=Otub1 PE=1 SV=1	AFGFSHLEALLDDSK
>	sp|B2RYG6|OT	Ubiquitin thioesterase OTUB1 OS=Rattus norvegicus GN=Otub1 PE=1 SV=1	FFEHFIEGGR
>	sp|Q02563|SV	Synaptic vesicle glycoprotein 2A OS=Rattus norvegicus GN=Sv2a PE=1 SV=2	AAPILFASAALALGSSLALK
>	sp|Q02563|SV	Synaptic vesicle glycoprotein 2A OS=Rattus norvegicus GN=Sv2a PE=1 SV=2	AAPILFASAALALGSSLALKLPETR
>	sp|Q02563|SV	Synaptic vesicle glycoprotein 2A OS=Rattus norvegicus GN=Sv2a PE=1 SV=2	TIHQEDELIEIQSDTGTWYQR
>	sp|Q02563|SV	Synaptic vesicle glycoprotein 2A OS=Rattus norvegicus GN=Sv2a PE=1 SV=2	HLQAVDYAAR
>	sp|Q02563|SV	Synaptic vesicle glycoprotein 2A OS=Rattus norvegicus GN=Sv2a PE=1 SV=2	AAPILFASAALALGSSLALKLPETR
>	sp|Q02563|SV	Synaptic vesicle glycoprotein 2A OS=Rattus norvegicus GN=Sv2a PE=1 SV=2	AAPILFASAALALGSSLALKLPETR
>	sp|Q02563|SV	Synaptic vesicle glycoprotein 2A OS=Rattus norvegicus GN=Sv2a PE=1 SV=2	AAPILFASAALALGSSLALKLPETR
>	sp|Q02563|SV	Synaptic vesicle glycoprotein 2A OS=Rattus norvegicus GN=Sv2a PE=1 SV=2	AAPILFASAALALGSSLALKLPETR
>	sp|Q02563|SV	Synaptic vesicle glycoprotein 2A OS=Rattus norvegicus GN=Sv2a PE=1 SV=2	AAPILFASAALALGSSLALKLPETR
>	sp|Q02563|SV	Synaptic vesicle glycoprotein 2A OS=Rattus norvegicus GN=Sv2a PE=1 SV=2	AAPILFASAALALGSSLALKLPETR
>	sp|Q02563|SV	Synaptic vesicle glycoprotein 2A OS=Rattus norvegicus GN=Sv2a PE=1 SV=2	AAPILFASAALALGSSLALKLPETR
>	sp|Q02563|SV	Synaptic vesicle glycoprotein 2A OS=Rattus norvegicus GN=Sv2a PE=1 SV=2	AAPILFASAALALGSSLALKLPETR
>	sp|Q02563|SV	Synaptic vesicle glycoprotein 2A OS=Rattus norvegicus GN=Sv2a PE=1 SV=2	TIHQEDELIEIQSDTGTWYQR
>	sp|O35303|DN	Dynamin-1-like protein OS=Rattus norvegicus GN=Dnm1l PE=1 SV=1	AVMHFLVNHVKDTLQSELVGQLYK
>	sp|O35303|DN	Dynamin-1-like protein OS=Rattus norvegicus GN=Dnm1l PE=1 SV=1	GHAVNLLDVPVPVAR
>	sp|O35303|DN	Dynamin-1-like protein OS=Rattus norvegicus GN=Dnm1l PE=1 SV=1	LHDAIVEVVTCLLR
>	sp|O35303|DN	Dynamin-1-like protein OS=Rattus norvegicus GN=Dnm1l PE=1 SV=1	TSKAEELLAEEK
>	sp|O35303|DN	Dynamin-1-like protein OS=Rattus norvegicus GN=Dnm1l PE=1 SV=1	AVMHFLVNHVKDTLQSELVGQLYK
>	sp|O35303|DN	Dynamin-1-like protein OS=Rattus norvegicus GN=Dnm1l PE=1 SV=1	AVMHFLVNHVKDTLQSELVGQLYK
>	sp|O35303|DN	Dynamin-1-like protein OS=Rattus norvegicus GN=Dnm1l PE=1 SV=1	AVMHFLVNHVKDTLQSELVGQLYK
>	sp|O35303|DN	Dynamin-1-like protein OS=Rattus norvegicus GN=Dnm1l PE=1 SV=1	AVMHFLVNHVKDTLQSELVGQLYK
>	sp|O35303|DN	Dynamin-1-like protein OS=Rattus norvegicus GN=Dnm1l PE=1 SV=1	AVMHFLVNHVKDTLQSELVGQLYK
>	sp|O35303|DN	Dynamin-1-like protein OS=Rattus norvegicus GN=Dnm1l PE=1 SV=1	GHAVNLLDVPVPVAR
>	sp|O35303|DN	Dynamin-1-like protein OS=Rattus norvegicus GN=Dnm1l PE=1 SV=1	GHAVNLLDVPVPVAR
>	sp|O35303|DN	Dynamin-1-like protein OS=Rattus norvegicus GN=Dnm1l PE=1 SV=1	PGMTK
>	sp|O35303|DN	Dynamin-1-like protein OS=Rattus norvegicus GN=Dnm1l PE=1 SV=1	AVMHFLVNHVKDTLQSELVGQLYK
>	sp|O35303|DN	Dynamin-1-like protein OS=Rattus norvegicus GN=Dnm1l PE=1 SV=1	IAEIR
>	sp|Q6PCU2|VA	V-type proton ATPase subunit E 1 OS=Rattus norvegicus GN=Atp6v1e1 PE=1 SV=1	ARDDLITDLLNEAK
>	sp|Q6PCU2|VA	V-type proton ATPase subunit E 1 OS=Rattus norvegicus GN=Atp6v1e1 PE=1 SV=1	HMMAFIEQEANEKAEEIDAKAEEEFNIEK
>	sp|Q6PCU2|VA	V-type proton ATPase subunit E 1 OS=Rattus norvegicus GN=Atp6v1e1 PE=1 SV=1	LDLIAQQMMPEVR
>	sp|Q6PCU2|VA	V-type proton ATPase subunit E 1 OS=Rattus norvegicus GN=Atp6v1e1 PE=1 SV=1	HMMAFIEQEANEKAEEIDAK
>	sp|Q6PCU2|VA	V-type proton ATPase subunit E 1 OS=Rattus norvegicus GN=Atp6v1e1 PE=1 SV=1	ARDDLITDLLNEAK
>	sp|Q6PCU2|VA	V-type proton ATPase subunit E 1 OS=Rattus norvegicus GN=Atp6v1e1 PE=1 SV=1	ARDDLITDLLNEAK
>	sp|Q6PCU2|VA	V-type proton ATPase subunit E 1 OS=Rattus norvegicus GN=Atp6v1e1 PE=1 SV=1	ARDDLITDLLNEAK
>	sp|Q6PCU2|VA	V-type proton ATPase subunit E 1 OS=Rattus norvegicus GN=Atp6v1e1 PE=1 SV=1	HMMAFIEQEANEKAEEIDAK
>	sp|Q6PCU2|VA	V-type proton ATPase subunit E 1 OS=Rattus norvegicus GN=Atp6v1e1 PE=1 SV=1	HMMAFIEQEANEKAEEIDAK
>	sp|Q6PCU2|VA	V-type proton ATPase subunit E 1 OS=Rattus norvegicus GN=Atp6v1e1 PE=1 SV=1	HMMAFIEQEANEKAEEIDAKAEEEFNIEK
>	sp|Q6PCU2|VA	V-type proton ATPase subunit E 1 OS=Rattus norvegicus GN=Atp6v1e1 PE=1 SV=1	HMMAFIEQEANEKAEEIDAKAEEEFNIEK
>	sp|Q6PCU2|VA	V-type proton ATPase subunit E 1 OS=Rattus norvegicus GN=Atp6v1e1 PE=1 SV=1	HMMAFIEQEANEKAEEIDAKAEEEFNIEK

>	sp|Q6PCU2|VA	V-type proton ATPase subunit E 1 OS=Rattus norvegicus GN=Atp6v1e1 PE=1 SV=1	HMMAFIEQEANEKAEEIDAKAEEEFNIEK
>	sp|Q6PCU2|VA	V-type proton ATPase subunit E 1 OS=Rattus norvegicus GN=Atp6v1e1 PE=1 SV=1	HMMAFIEQEANEKAEEIDAKAEEEFNIEK
>	sp|Q6PCU2|VA	V-type proton ATPase subunit E 1 OS=Rattus norvegicus GN=Atp6v1e1 PE=1 SV=1	HMMAFIEQEANEKAEEIDAKAEEEFNIEK
>	sp|Q6PCU2|VA	V-type proton ATPase subunit E 1 OS=Rattus norvegicus GN=Atp6v1e1 PE=1 SV=1	LDLIAQQMMPEVR
>	sp|Q6PCU2|VA	V-type proton ATPase subunit E 1 OS=Rattus norvegicus GN=Atp6v1e1 PE=1 SV=1	LDLIAQQMMPEVR
>	sp|Q6PCU2|VA	V-type proton ATPase subunit E 1 OS=Rattus norvegicus GN=Atp6v1e1 PE=1 SV=1	LDLIAQQMMPEVR
>	sp|Q6PCU2|VA	V-type proton ATPase subunit E 1 OS=Rattus norvegicus GN=Atp6v1e1 PE=1 SV=1	ARDDLITDLLNEAK
>	sp|Q6PCU2|VA	V-type proton ATPase subunit E 1 OS=Rattus norvegicus GN=Atp6v1e1 PE=1 SV=1	ARDDLITDLLNEAK
>	sp|Q6PCU2|VA	V-type proton ATPase subunit E 1 OS=Rattus norvegicus GN=Atp6v1e1 PE=1 SV=1	HMMAFIEQEANEKAEEIDAK
>	sp|Q6PCU2|VA	V-type proton ATPase subunit E 1 OS=Rattus norvegicus GN=Atp6v1e1 PE=1 SV=1	ARDDLITDLLNEAK
>	sp|Q9EPC6|PR(	Profilin-2 OS=Rattus norvegicus GN=Pfn2 PE=1 SV=3	EGVHGGGLNKK
>	sp|Q9EPC6|PR(	Profilin-2 OS=Rattus norvegicus GN=Pfn2 PE=1 SV=3	YVWAATAGGVFQSITPAEIDVIIGK
>	sp|Q9EPC6|PR(	Profilin-2 OS=Rattus norvegicus GN=Pfn2 PE=1 SV=3	VLVFVMGK
>	sp|Q9EPC6|PR(	Profilin-2 OS=Rattus norvegicus GN=Pfn2 PE=1 SV=3	DREGFFTNGLTLGGK
>	sp|Q9EPC6|PR(	Profilin-2 OS=Rattus norvegicus GN=Pfn2 PE=1 SV=3	VLVFVMGK
>	sp|Q9EPC6|PR(	Profilin-2 OS=Rattus norvegicus GN=Pfn2 PE=1 SV=3	VLVFVMGK
>	sp|Q9EPC6|PR(	Profilin-2 OS=Rattus norvegicus GN=Pfn2 PE=1 SV=3	VLVFVMGK
>	sp|Q9EPC6|PR(	Profilin-2 OS=Rattus norvegicus GN=Pfn2 PE=1 SV=3	EGVHGGGLNK
>	sp|P85845|FSC	Fascin OS=Rattus norvegicus GN=Fscn1 PE=1 SV=2	FLVVAHDDGR
>	sp|P85845|FSC	Fascin OS=Rattus norvegicus GN=Fscn1 PE=1 SV=2	KVTGTLDANR
>	sp|P85845|FSC	Fascin OS=Rattus norvegicus GN=Fscn1 PE=1 SV=2	VGKDELFALEQSCAQVVLQAANER
>	sp|P85845|FSC	Fascin OS=Rattus norvegicus GN=Fscn1 PE=1 SV=2	FLVVAHDDGR
>	sp|P85845|FSC	Fascin OS=Rattus norvegicus GN=Fscn1 PE=1 SV=2	FLVVAHDDGR
>	sp|P85845|FSC	Fascin OS=Rattus norvegicus GN=Fscn1 PE=1 SV=2	FLVVAHDDGR
>	sp|P85845|FSC	Fascin OS=Rattus norvegicus GN=Fscn1 PE=1 SV=2	FLVVAHDDGR
>	sp|P85845|FSC	Fascin OS=Rattus norvegicus GN=Fscn1 PE=1 SV=2	VGKDELFALEQSCAQVVLQAANER
>	sp|P85845|FSC	Fascin OS=Rattus norvegicus GN=Fscn1 PE=1 SV=2	VGKDELFALEQSCAQVVLQAANER
>	sp|P85845|FSC	Fascin OS=Rattus norvegicus GN=Fscn1 PE=1 SV=2	VGKDELFALEQSCAQVVLQAANER
>	sp|P85845|FSC	Fascin OS=Rattus norvegicus GN=Fscn1 PE=1 SV=2	VGKDELFALEQSCAQVVLQAANER
>	sp|P85845|FSC	Fascin OS=Rattus norvegicus GN=Fscn1 PE=1 SV=2	VGKDELFALEQSCAQVVLQAANER
>	sp|P85845|FSC	Fascin OS=Rattus norvegicus GN=Fscn1 PE=1 SV=2	VGKDELFALEQSCAQVVLQAANER
>	sp|P85845|FSC	Fascin OS=Rattus norvegicus GN=Fscn1 PE=1 SV=2	VGKDELFALEQSCAQVVLQAANER
>	sp|P85845|FSC	Fascin OS=Rattus norvegicus GN=Fscn1 PE=1 SV=2	VGKDELFALEQSCAQVVLQAANER
>	sp|P85845|FSC	Fascin OS=Rattus norvegicus GN=Fscn1 PE=1 SV=2	VGKDELFALEQSCAQVVLQAANER
>	sp|P85845|FSC	Fascin OS=Rattus norvegicus GN=Fscn1 PE=1 SV=2	LVARPEPATGFTLEFR
>	sp|P85845|FSC	Fascin OS=Rattus norvegicus GN=Fscn1 PE=1 SV=2	FLVVAHDDGR
>	sp|P61983|143	14-3-3 protein gamma OS=Rattus norvegicus GN=Ywhag PE=1 SV=2	EKIEKELEAVCQDVLSLLDNYLIK
>	sp|P61983|143	14-3-3 protein gamma OS=Rattus norvegicus GN=Ywhag PE=1 SV=2	NVTELNEPLSNEER
>	sp|P61983|143	14-3-3 protein gamma OS=Rattus norvegicus GN=Ywhag PE=1 SV=2	YDDMAAAMK
>	sp|P61983|143	14-3-3 protein gamma OS=Rattus norvegicus GN=Ywhag PE=1 SV=2	TAFDDAIAELDTLNEDSYKDSTLIMQLLR
>	sp|P61983|143	14-3-3 protein gamma OS=Rattus norvegicus GN=Ywhag PE=1 SV=2	LGLALNYSVFYYEIQNAPEQACHLAK
>	sp|P61983|143	14-3-3 protein gamma OS=Rattus norvegicus GN=Ywhag PE=1 SV=2	DSTLIMQLLR
>	sp|P61983|143	14-3-3 protein gamma OS=Rattus norvegicus GN=Ywhag PE=1 SV=2	EKIEKELEAVCQDVLSLLDNYLIK
>	sp|P61983|143	14-3-3 protein gamma OS=Rattus norvegicus GN=Ywhag PE=1 SV=2	EKIEKELEAVCQDVLSLLDNYLIK
>	sp|P61983|143	14-3-3 protein gamma OS=Rattus norvegicus GN=Ywhag PE=1 SV=2	LGLALNYSVFYYEIQNAPEQACHLAK
>	sp|P61983|143	14-3-3 protein gamma OS=Rattus norvegicus GN=Ywhag PE=1 SV=2	LGLALNYSVFYYEIQNAPEQACHLAK
>	sp|P61983|143	14-3-3 protein gamma OS=Rattus norvegicus GN=Ywhag PE=1 SV=2	LGLALNYSVFYYEIQNAPEQACHLAK
>	sp|P61983|143	14-3-3 protein gamma OS=Rattus norvegicus GN=Ywhag PE=1 SV=2	LGLALNYSVFYYEIQNAPEQACHLAK
>	sp|P61983|143	14-3-3 protein gamma OS=Rattus norvegicus GN=Ywhag PE=1 SV=2	NLLSVAYK
>	sp|P61983|143	14-3-3 protein gamma OS=Rattus norvegicus GN=Ywhag PE=1 SV=2	NLLSVAYK
>	sp|P61983|143	14-3-3 protein gamma OS=Rattus norvegicus GN=Ywhag PE=1 SV=2	NLLSVAYK
>	sp|P61983|143	14-3-3 protein gamma OS=Rattus norvegicus GN=Ywhag PE=1 SV=2	NLLSVAYK
>	sp|P61983|143	14-3-3 protein gamma OS=Rattus norvegicus GN=Ywhag PE=1 SV=2	NLLSVAYK
>	sp|P61983|143	14-3-3 protein gamma OS=Rattus norvegicus GN=Ywhag PE=1 SV=2	NVTELNEPLSNEER
>	sp|P61983|143	14-3-3 protein gamma OS=Rattus norvegicus GN=Ywhag PE=1 SV=2	NVTELNEPLSNEER
>	sp|P61983|143	14-3-3 protein gamma OS=Rattus norvegicus GN=Ywhag PE=1 SV=2	NVTELNEPLSNEER
>	sp|P61983|143	14-3-3 protein gamma OS=Rattus norvegicus GN=Ywhag PE=1 SV=2	TAFDDAIAELDTLNEDSYKDSTLIMQLLR
>	sp|P61983|143	14-3-3 protein gamma OS=Rattus norvegicus GN=Ywhag PE=1 SV=2	VISSIEQK
>	sp|P61983|143	14-3-3 protein gamma OS=Rattus norvegicus GN=Ywhag PE=1 SV=2	NLLSVAYK
>	sp|P61983|143	14-3-3 protein gamma OS=Rattus norvegicus GN=Ywhag PE=1 SV=2	ATVVESSEK
>	sp|P61983|143	14-3-3 protein gamma OS=Rattus norvegicus GN=Ywhag PE=1 SV=2	VISSIEQK
>	sp|P61983|143	14-3-3 protein gamma OS=Rattus norvegicus GN=Ywhag PE=1 SV=2	NLLSVAYK
>	sp|P61983|143	14-3-3 protein gamma OS=Rattus norvegicus GN=Ywhag PE=1 SV=2	NLLSVAYK
>	sp|P61983|143	14-3-3 protein gamma OS=Rattus norvegicus GN=Ywhag PE=1 SV=2	NLLSVAYK
>	sp|P61983|143	14-3-3 protein gamma OS=Rattus norvegicus GN=Ywhag PE=1 SV=2	LGLALNYSVFYYEIQNAPEQACHLAK
>	sp|P61983|143	14-3-3 protein gamma OS=Rattus norvegicus GN=Ywhag PE=1 SV=2	YDDMAAAMK
>	sp|P61983|143	14-3-3 protein gamma OS=Rattus norvegicus GN=Ywhag PE=1 SV=2	VISSIEQK
>	sp|P61983|143	14-3-3 protein gamma OS=Rattus norvegicus GN=Ywhag PE=1 SV=2	YDDMAAAMK
>	sp|P61983|143	14-3-3 protein gamma OS=Rattus norvegicus GN=Ywhag PE=1 SV=2	LGLALNYSVFYYEIQNAPEQACHLAK
>	sp|P61983|143	14-3-3 protein gamma OS=Rattus norvegicus GN=Ywhag PE=1 SV=2	LGLALNYSVFYYEIQNAPEQACHLAK
>	sp|Q63345|MC	Myelin-oligodendrocyte glycoprotein OS=Rattus norvegicus GN=Mog PE=1 SV=1	ALVGDEAELPCR
>	sp|Q63345|MC	Myelin-oligodendrocyte glycoprotein OS=Rattus norvegicus GN=Mog PE=1 SV=1	VIGPGHPIR
>	sp|Q63345|MC	Myelin-oligodendrocyte glycoprotein OS=Rattus norvegicus GN=Mog PE=1 SV=1	NGKDQDAEQAPEYR
>	sp|Q63345|MC	Myelin-oligodendrocyte glycoprotein OS=Rattus norvegicus GN=Mog PE=1 SV=1	ALVGDEAELPCR

>	sp|Q63345|MC	Myelin-oligodendrocyte glycoprotein OS=Rattus norvegicus GN=Mog PE=1 SV=1	ALVGDEAELPCR
>	sp|Q63345|MC	Myelin-oligodendrocyte glycoprotein OS=Rattus norvegicus GN=Mog PE=1 SV=1	ALVGDEAELPCR
>	sp|Q63345|MC	Myelin-oligodendrocyte glycoprotein OS=Rattus norvegicus GN=Mog PE=1 SV=1	ALVGDEAELPCR
>	sp|Q63345|MC	Myelin-oligodendrocyte glycoprotein OS=Rattus norvegicus GN=Mog PE=1 SV=1	ALVGDEAELPCR
>	sp|Q63345|MC	Myelin-oligodendrocyte glycoprotein OS=Rattus norvegicus GN=Mog PE=1 SV=1	ALVGDEAELPCR
>	sp|Q63345|MC	Myelin-oligodendrocyte glycoprotein OS=Rattus norvegicus GN=Mog PE=1 SV=1	NGKDQDAEQAPEYR
>	sp|Q63345|MC	Myelin-oligodendrocyte glycoprotein OS=Rattus norvegicus GN=Mog PE=1 SV=1	NGKDQDAEQAPEYR
>	sp|Q63345|MC	Myelin-oligodendrocyte glycoprotein OS=Rattus norvegicus GN=Mog PE=1 SV=1	FSDEGGYTCFFR
>	sp|Q63345|MC	Myelin-oligodendrocyte glycoprotein OS=Rattus norvegicus GN=Mog PE=1 SV=1	NGKDQDAEQAPEYR
>	sp|Q63345|MC	Myelin-oligodendrocyte glycoprotein OS=Rattus norvegicus GN=Mog PE=1 SV=1	NGKDQDAEQAPEYR
>	sp|Q63345|MC	Myelin-oligodendrocyte glycoprotein OS=Rattus norvegicus GN=Mog PE=1 SV=1	NGKDQDAEQAPEYR
>	sp|P11240|CO	Cytochrome c oxidase subunit 5A, mitochondrial OS=Rattus norvegicus GN=Cox5a PE=1 SV=1	WVTYFNKPDIDAWELR
>	sp|P11240|CO	Cytochrome c oxidase subunit 5A, mitochondrial OS=Rattus norvegicus GN=Cox5a PE=1 SV=1	WVTYFNKPDIDAWELRK
>	sp|P11240|CO	Cytochrome c oxidase subunit 5A, mitochondrial OS=Rattus norvegicus GN=Cox5a PE=1 SV=1	RLNDFASAVR
>	sp|P11240|CO	Cytochrome c oxidase subunit 5A, mitochondrial OS=Rattus norvegicus GN=Cox5a PE=1 SV=1	AGPHKEIYPYVIQELRPTLNELGISTPEELGLDKV
>	sp|P11240|CO	Cytochrome c oxidase subunit 5A, mitochondrial OS=Rattus norvegicus GN=Cox5a PE=1 SV=1	WVTYFNKPDIDAWELR
>	sp|P11240|CO	Cytochrome c oxidase subunit 5A, mitochondrial OS=Rattus norvegicus GN=Cox5a PE=1 SV=1	WVTYFNKPDIDAWELRK
>	sp|P11240|CO	Cytochrome c oxidase subunit 5A, mitochondrial OS=Rattus norvegicus GN=Cox5a PE=1 SV=1	WVTYFNKPDIDAWELRK
>	sp|P11240|CO	Cytochrome c oxidase subunit 5A, mitochondrial OS=Rattus norvegicus GN=Cox5a PE=1 SV=1	WVTYFNKPDIDAWELRK
>	sp|P11240|CO	Cytochrome c oxidase subunit 5A, mitochondrial OS=Rattus norvegicus GN=Cox5a PE=1 SV=1	WVTYFNKPDIDAWELR
>	sp|P11240|CO	Cytochrome c oxidase subunit 5A, mitochondrial OS=Rattus norvegicus GN=Cox5a PE=1 SV=1	ILEVVKDK
>	sp|P11240|CO	Cytochrome c oxidase subunit 5A, mitochondrial OS=Rattus norvegicus GN=Cox5a PE=1 SV=1	RLNDFASAVR
>	sp|P11240|CO	Cytochrome c oxidase subunit 5A, mitochondrial OS=Rattus norvegicus GN=Cox5a PE=1 SV=1	ILEVVKDK
>	sp|P11240|CO	Cytochrome c oxidase subunit 5A, mitochondrial OS=Rattus norvegicus GN=Cox5a PE=1 SV=1	ILEVVKDK
>	sp|P11240|CO	Cytochrome c oxidase subunit 5A, mitochondrial OS=Rattus norvegicus GN=Cox5a PE=1 SV=1	ILEVVKDK
>	sp|P11240|CO	Cytochrome c oxidase subunit 5A, mitochondrial OS=Rattus norvegicus GN=Cox5a PE=1 SV=1	AGPHKEIYPYVIQELRPTLNELGISTPEELGLDKV
>	sp|P11240|CO	Cytochrome c oxidase subunit 5A, mitochondrial OS=Rattus norvegicus GN=Cox5a PE=1 SV=1	ILEVVKDK
>	sp|P07632|SOI	Superoxide dismutase [Cu-Zn] OS=Rattus norvegicus GN=Sod1 PE=1 SV=2	DGVANVSIEDR
>	sp|P07632|SOI	Superoxide dismutase [Cu-Zn] OS=Rattus norvegicus GN=Sod1 PE=1 SV=2	GDGPVQGVIHFEQK
>	sp|P07632|SOI	Superoxide dismutase [Cu-Zn] OS=Rattus norvegicus GN=Sod1 PE=1 SV=2	KHGGPADEER
>	sp|P07632|SOI	Superoxide dismutase [Cu-Zn] OS=Rattus norvegicus GN=Sod1 PE=1 SV=2	ASGEPVVVSGQITGLTEGEHGFHVHQYGDNTQGCTTAGPHFNPHSK
>	sp|P07632|SOI	Superoxide dismutase [Cu-Zn] OS=Rattus norvegicus GN=Sod1 PE=1 SV=2	QDDLGKGGNEESTK
>	sp|P07632|SOI	Superoxide dismutase [Cu-Zn] OS=Rattus norvegicus GN=Sod1 PE=1 SV=2	GDGPVQGVIHFEQK
>	sp|P07632|SOI	Superoxide dismutase [Cu-Zn] OS=Rattus norvegicus GN=Sod1 PE=1 SV=2	GDGPVQGVIHFEQK
>	sp|P07632|SOI	Superoxide dismutase [Cu-Zn] OS=Rattus norvegicus GN=Sod1 PE=1 SV=2	GDGPVQGVIHFEQK
>	sp|P07632|SOI	Superoxide dismutase [Cu-Zn] OS=Rattus norvegicus GN=Sod1 PE=1 SV=2	KHGGPADEER
>	sp|P07632|SOI	Superoxide dismutase [Cu-Zn] OS=Rattus norvegicus GN=Sod1 PE=1 SV=2	KHGGPADEER
>	sp|P07632|SOI	Superoxide dismutase [Cu-Zn] OS=Rattus norvegicus GN=Sod1 PE=1 SV=2	QDDLGKGGNEESTK
>	sp|P07632|SOI	Superoxide dismutase [Cu-Zn] OS=Rattus norvegicus GN=Sod1 PE=1 SV=2	TMVVHEK
>	sp|P07632|SOI	Superoxide dismutase [Cu-Zn] OS=Rattus norvegicus GN=Sod1 PE=1 SV=2	QDDLGKGGNEESTK
>	sp|Q9JJM9|SEF	Septin-5 OS=Rattus norvegicus GN=Sept5 PE=1 SV=2	ADCLVPSEIR
>	sp|Q9JJM9|SEF	Septin-5 OS=Rattus norvegicus GN=Sept5 PE=1 SV=2	STLVHSLFLTDLYKDR
>	sp|Q9JJM9|SEF	Septin-5 OS=Rattus norvegicus GN=Sept5 PE=1 SV=2	VNIVPLIAK
>	sp|Q9JJM9|SEF	Septin-5 OS=Rattus norvegicus GN=Sept5 PE=1 SV=2	LYPWGIVEVENQAHCDFVK
>	sp|Q9JJM9|SEF	Septin-5 OS=Rattus norvegicus GN=Sept5 PE=1 SV=2	STLVHSLFLTDLYK
>	sp|Q9JJM9|SEF	Septin-5 OS=Rattus norvegicus GN=Sept5 PE=1 SV=2	STLVHSLFLTDLYK
>	sp|Q9JJM9|SEF	Septin-5 OS=Rattus norvegicus GN=Sept5 PE=1 SV=2	STLVHSLFLTDLYK
>	sp|Q9JJM9|SEF	Septin-5 OS=Rattus norvegicus GN=Sept5 PE=1 SV=2	MESPIPILPLPTPDSETEK
>	sp|Q9JJM9|SEF	Septin-5 OS=Rattus norvegicus GN=Sept5 PE=1 SV=2	KLLSAEER
>	sp|Q9JJM9|SEF	Septin-5 OS=Rattus norvegicus GN=Sept5 PE=1 SV=2	STLVHSLFLTDLYKDR
>	sp|P13264|GLS	Glutaminase kidney isoform, mitochondrial OS=Rattus norvegicus GN=Gls PE=1 SV=2	GIHFCHDLVSLCNFHNYDNLR
>	sp|P13264|GLS	Glutaminase kidney isoform, mitochondrial OS=Rattus norvegicus GN=Gls PE=1 SV=2	TALHVAAAEGHVEVVK
>	sp|P13264|GLS	Glutaminase kidney isoform, mitochondrial OS=Rattus norvegicus GN=Gls PE=1 SV=2	VKSVINLLFAAYTGDVSALR
>	sp|P13264|GLS	Glutaminase kidney isoform, mitochondrial OS=Rattus norvegicus GN=Gls PE=1 SV=2	FSPDLWGVSVCTVDGQR
>	sp|P13264|GLS	Glutaminase kidney isoform, mitochondrial OS=Rattus norvegicus GN=Gls PE=1 SV=2	GIHFCHDLVSLCNFHNYDNLR
>	sp|P13264|GLS	Glutaminase kidney isoform, mitochondrial OS=Rattus norvegicus GN=Gls PE=1 SV=2	TALHVAAAEGHVEVVK
>	sp|P13264|GLS	Glutaminase kidney isoform, mitochondrial OS=Rattus norvegicus GN=Gls PE=1 SV=2	TALHVAAAEGHVEVVK
>	sp|P13264|GLS	Glutaminase kidney isoform, mitochondrial OS=Rattus norvegicus GN=Gls PE=1 SV=2	GIHFCHDLVSLCNFHNYDNLR
>	sp|O35095|NC	Neurochondrin OS=Rattus norvegicus GN=Ncdn PE=1 SV=2	ILGAWLAEETSSLRK
>	sp|O35095|NC	Neurochondrin OS=Rattus norvegicus GN=Ncdn PE=1 SV=2	EAPDGCPDHVLR
>	sp|O35095|NC	Neurochondrin OS=Rattus norvegicus GN=Ncdn PE=1 SV=2	RIFDAVGFTFPNR
>	sp|O35095|NC	Neurochondrin OS=Rattus norvegicus GN=Ncdn PE=1 SV=2	LLLPGWCHLTVEDGPR
>	sp|O35095|NC	Neurochondrin OS=Rattus norvegicus GN=Ncdn PE=1 SV=2	EAPDGCPDHVLR
>	sp|O35095|NC	Neurochondrin OS=Rattus norvegicus GN=Ncdn PE=1 SV=2	DLQAGLAR
>	sp|Q561S0|ND	NADH dehydrogenase [ubiquinone] 1 alpha subcomplex subunit 10, mitochondrial OS=Rattus norvegicus GN=Ndufa10 PE=1 SV=1	LLQYSDALEHLLSTGQGVVLER
>	sp|Q561S0|ND	NADH dehydrogenase [ubiquinone] 1 alpha subcomplex subunit 10, mitochondrial OS=Rattus norvegicus GN=Ndufa10 PE=1 SV=1	YGLLASILGDK
>	sp|Q561S0|ND	NADH dehydrogenase [ubiquinone] 1 alpha subcomplex subunit 10, mitochondrial OS=Rattus norvegicus GN=Ndufa10 PE=1 SV=1	VITVDGNICSGK
>	sp|Q561S0|ND	NADH dehydrogenase [ubiquinone] 1 alpha subcomplex subunit 10, mitochondrial OS=Rattus norvegicus GN=Ndufa10 PE=1 SV=1	YGLLASILGDKTTK
>	sp|Q561S0|ND	NADH dehydrogenase [ubiquinone] 1 alpha subcomplex subunit 10, mitochondrial OS=Rattus norvegicus GN=Ndufa10 PE=1 SV=1	LLQYSDALEHLLSTGQGVVLER
>	sp|Q561S0|ND	NADH dehydrogenase [ubiquinone] 1 alpha subcomplex subunit 10, mitochondrial OS=Rattus norvegicus GN=Ndufa10 PE=1 SV=1	LLQYSDALEHLLSTGQGVVLER
>	sp|Q561S0|ND	NADH dehydrogenase [ubiquinone] 1 alpha subcomplex subunit 10, mitochondrial OS=Rattus norvegicus GN=Ndufa10 PE=1 SV=1	LLQYSDALEHLLSTGQGVVLER
>	sp|Q561S0|ND	NADH dehydrogenase [ubiquinone] 1 alpha subcomplex subunit 10, mitochondrial OS=Rattus norvegicus GN=Ndufa10 PE=1 SV=1	YGLLASILGDK
>	sp|Q561S0|ND	NADH dehydrogenase [ubiquinone] 1 alpha subcomplex subunit 10, mitochondrial OS=Rattus norvegicus GN=Ndufa10 PE=1 SV=1	YGLLASILGDK
>	sp|Q561S0|ND	NADH dehydrogenase [ubiquinone] 1 alpha subcomplex subunit 10, mitochondrial OS=Rattus norvegicus GN=Ndufa10 PE=1 SV=1	LLQYSDALEHLLSTGQGVVLER

>	sp|Q561S0|ND	NADH dehydrogenase [ubiquinone] 1 alpha subcomplex subunit 10, mitochondrial OS=Rattus norvegicus GN=Ndufa10 PE=1 SV=1	LLQYSDALEHLLSTGQGVVLER
>	sp|Q561S0|ND	NADH dehydrogenase [ubiquinone] 1 alpha subcomplex subunit 10, mitochondrial OS=Rattus norvegicus GN=Ndufa10 PE=1 SV=1	LVPASASARGLAAG
>	sp|P63012|RAE	Ras-related protein Rab-3A OS=Rattus norvegicus GN=Rab3a PE=1 SV=1	LQIWDTAGQER
>	sp|P63012|RAE	Ras-related protein Rab-3A OS=Rattus norvegicus GN=Rab3a PE=1 SV=1	MSESLDTADPAVTGAK
>	sp|P63012|RAE	Ras-related protein Rab-3A OS=Rattus norvegicus GN=Rab3a PE=1 SV=1	LVDVICEK
>	sp|P63012|RAE	Ras-related protein Rab-3A OS=Rattus norvegicus GN=Rab3a PE=1 SV=1	QLADHLGFEFFEASAK
>	sp|P63012|RAE	Ras-related protein Rab-3A OS=Rattus norvegicus GN=Rab3a PE=1 SV=1	LQIWDTAGQER
>	sp|P63012|RAE	Ras-related protein Rab-3A OS=Rattus norvegicus GN=Rab3a PE=1 SV=1	MSESLDTADPAVTGAK
>	sp|P63012|RAE	Ras-related protein Rab-3A OS=Rattus norvegicus GN=Rab3a PE=1 SV=1	MSESLDTADPAVTGAK
>	sp|P63012|RAE	Ras-related protein Rab-3A OS=Rattus norvegicus GN=Rab3a PE=1 SV=1	QLADHLGFEFFEASAK
>	sp|P63012|RAE	Ras-related protein Rab-3A OS=Rattus norvegicus GN=Rab3a PE=1 SV=1	QLADHLGFEFFEASAK
>	sp|P63012|RAE	Ras-related protein Rab-3A OS=Rattus norvegicus GN=Rab3a PE=1 SV=1	QLADHLGFEFFEASAK
>	sp|P63012|RAE	Ras-related protein Rab-3A OS=Rattus norvegicus GN=Rab3a PE=1 SV=1	QLADHLGFEFFEASAK
>	sp|P63012|RAE	Ras-related protein Rab-3A OS=Rattus norvegicus GN=Rab3a PE=1 SV=1	QLADHLGFEFFEASAK
>	sp|P63012|RAE	Ras-related protein Rab-3A OS=Rattus norvegicus GN=Rab3a PE=1 SV=1	QLADHLGFEFFEASAK
>	sp|P63012|RAE	Ras-related protein Rab-3A OS=Rattus norvegicus GN=Rab3a PE=1 SV=1	QLADHLGFEFFEASAK
>	sp|P63012|RAE	Ras-related protein Rab-3A OS=Rattus norvegicus GN=Rab3a PE=1 SV=1	LQIWDTAGQER
>	sp|P63012|RAE	Ras-related protein Rab-3A OS=Rattus norvegicus GN=Rab3a PE=1 SV=1	QLADHLGFEFFEASAK
>	sp|P63012|RAE	Ras-related protein Rab-3A OS=Rattus norvegicus GN=Rab3a PE=1 SV=1	TYSWDNAQVLLVGNKCDMEDER
>	sp|P63012|RAE	Ras-related protein Rab-3A OS=Rattus norvegicus GN=Rab3a PE=1 SV=1	TYSWDNAQVLLVGNK
>	sp|P63012|RAE	Ras-related protein Rab-3A OS=Rattus norvegicus GN=Rab3a PE=1 SV=1	LVDVICEK
>	sp|P63012|RAE	Ras-related protein Rab-3A OS=Rattus norvegicus GN=Rab3a PE=1 SV=1	TYSWDNAQVLLVGNK
>	sp|P63012|RAE	Ras-related protein Rab-3A OS=Rattus norvegicus GN=Rab3a PE=1 SV=1	QLADHLGFEFFEASAK
>	sp|P63012|RAE	Ras-related protein Rab-3A OS=Rattus norvegicus GN=Rab3a PE=1 SV=1	LQIWDTAGQER
>	sp|P63012|RAE	Ras-related protein Rab-3A OS=Rattus norvegicus GN=Rab3a PE=1 SV=1	LVDVICEK
>	sp|Q06647|ATI	ATP synthase subunit O, mitochondrial OS=Rattus norvegicus GN=Atp5o PE=1 SV=1	LDQVEKELLR
>	sp|Q06647|ATI	ATP synthase subunit O, mitochondrial OS=Rattus norvegicus GN=Atp5o PE=1 SV=1	LVRPPVQVYGIEGR
>	sp|Q06647|ATI	ATP synthase subunit O, mitochondrial OS=Rattus norvegicus GN=Atp5o PE=1 SV=1	VSLAVLNPYIK
>	sp|Q06647|ATI	ATP synthase subunit O, mitochondrial OS=Rattus norvegicus GN=Atp5o PE=1 SV=1	LVRPPVQVYGIEGR
>	sp|Q06647|ATI	ATP synthase subunit O, mitochondrial OS=Rattus norvegicus GN=Atp5o PE=1 SV=1	LVRPPVQVYGIEGR
>	sp|Q06647|ATI	ATP synthase subunit O, mitochondrial OS=Rattus norvegicus GN=Atp5o PE=1 SV=1	LVRPPVQVYGIEGR
>	sp|Q06647|ATI	ATP synthase subunit O, mitochondrial OS=Rattus norvegicus GN=Atp5o PE=1 SV=1	LVRPPVQVYGIEGR
>	sp|Q06647|ATI	ATP synthase subunit O, mitochondrial OS=Rattus norvegicus GN=Atp5o PE=1 SV=1	LVRPPVQVYGIEGR
>	sp|Q06647|ATI	ATP synthase subunit O, mitochondrial OS=Rattus norvegicus GN=Atp5o PE=1 SV=1	VSLAVLNPYIK
>	sp|Q06647|ATI	ATP synthase subunit O, mitochondrial OS=Rattus norvegicus GN=Atp5o PE=1 SV=1	VSLAVLNPYIK
>	sp|Q06647|ATI	ATP synthase subunit O, mitochondrial OS=Rattus norvegicus GN=Atp5o PE=1 SV=1	VSLAVLNPYIK
>	sp|Q06647|ATI	ATP synthase subunit O, mitochondrial OS=Rattus norvegicus GN=Atp5o PE=1 SV=1	VSLAVLNPYIK
>	sp|Q06647|ATI	ATP synthase subunit O, mitochondrial OS=Rattus norvegicus GN=Atp5o PE=1 SV=1	LVRPPVQVYGIEGR
>	sp|Q06647|ATI	ATP synthase subunit O, mitochondrial OS=Rattus norvegicus GN=Atp5o PE=1 SV=1	TDPSIMGGMIVR
>	sp|Q06647|ATI	ATP synthase subunit O, mitochondrial OS=Rattus norvegicus GN=Atp5o PE=1 SV=1	VSLAVLNPYIK
>	sp|P62762|VISI	Visinin-like protein 1 OS=Rattus norvegicus GN=Vsnl1 PE=1 SV=2	MDKNKDDQITLDEFKEAAK
>	sp|P62762|VISI	Visinin-like protein 1 OS=Rattus norvegicus GN=Vsnl1 PE=1 SV=2	TFDKNGDGTIDFR
>	sp|P62762|VISI	Visinin-like protein 1 OS=Rattus norvegicus GN=Vsnl1 PE=1 SV=2	STEFNEHELK
>	sp|P62762|VISI	Visinin-like protein 1 OS=Rattus norvegicus GN=Vsnl1 PE=1 SV=2	SDPSIVLLLQCDIQK
>	sp|P62762|VISI	Visinin-like protein 1 OS=Rattus norvegicus GN=Vsnl1 PE=1 SV=2	LNLEEFQQLYVK
>	sp|P62762|VISI	Visinin-like protein 1 OS=Rattus norvegicus GN=Vsnl1 PE=1 SV=2	MDKNKDDQITLDEFKEAAK
>	sp|P62762|VISI	Visinin-like protein 1 OS=Rattus norvegicus GN=Vsnl1 PE=1 SV=2	MDKNKDDQITLDEFKEAAK
>	sp|P62762|VISI	Visinin-like protein 1 OS=Rattus norvegicus GN=Vsnl1 PE=1 SV=2	LNLEEFQQLYVK
>	sp|P62762|VISI	Visinin-like protein 1 OS=Rattus norvegicus GN=Vsnl1 PE=1 SV=2	LNLEEFQQLYVK
>	sp|P62762|VISI	Visinin-like protein 1 OS=Rattus norvegicus GN=Vsnl1 PE=1 SV=2	LNLEEFQQLYVK
>	sp|P62762|VISI	Visinin-like protein 1 OS=Rattus norvegicus GN=Vsnl1 PE=1 SV=2	TFDKNGDGTIDFR
>	sp|P62762|VISI	Visinin-like protein 1 OS=Rattus norvegicus GN=Vsnl1 PE=1 SV=2	EFICALSITSR
>	sp|P62762|VISI	Visinin-like protein 1 OS=Rattus norvegicus GN=Vsnl1 PE=1 SV=2	LAPEVMEDLVK
>	sp|P62762|VISI	Visinin-like protein 1 OS=Rattus norvegicus GN=Vsnl1 PE=1 SV=2	LNLEEFQQLYVK
>	sp|P62762|VISI	Visinin-like protein 1 OS=Rattus norvegicus GN=Vsnl1 PE=1 SV=2	SDPSIVLLLQCDIQK
>	sp|P62762|VISI	Visinin-like protein 1 OS=Rattus norvegicus GN=Vsnl1 PE=1 SV=2	SDPSIVLLLQCDIQK
>	sp|P62762|VISI	Visinin-like protein 1 OS=Rattus norvegicus GN=Vsnl1 PE=1 SV=2	SDPSIVLLLQCDIQK
>	sp|P62762|VISI	Visinin-like protein 1 OS=Rattus norvegicus GN=Vsnl1 PE=1 SV=2	SDPSIVLLLQCDIQK
>	sp|P62762|VISI	Visinin-like protein 1 OS=Rattus norvegicus GN=Vsnl1 PE=1 SV=2	LNLEEFQQLYVK
>	sp|P62762|VISI	Visinin-like protein 1 OS=Rattus norvegicus GN=Vsnl1 PE=1 SV=2	SDPSIVLLLQCDIQK
>	sp|P62762|VISI	Visinin-like protein 1 OS=Rattus norvegicus GN=Vsnl1 PE=1 SV=2	LAPEVMEDLVK
>	sp|P62762|VISI	Visinin-like protein 1 OS=Rattus norvegicus GN=Vsnl1 PE=1 SV=2	LNLEEFQQLYVK
>	sp|Q68FX0|IDH	Isocitrate dehydrogenase [NAD] subunit beta, mitochondrial OS=Rattus norvegicus GN=Idh3B PE=1 SV=1	HNNLDLVIIR
>	sp|Q68FX0|IDH	Isocitrate dehydrogenase [NAD] subunit beta, mitochondrial OS=Rattus norvegicus GN=Idh3B PE=1 SV=1	NIANPTAMLLSASNMLR
>	sp|Q68FX0|IDH	Isocitrate dehydrogenase [NAD] subunit beta, mitochondrial OS=Rattus norvegicus GN=Idh3B PE=1 SV=1	LGDGLFLQCCEEVAELYPK
>	sp|Q68FX0|IDH	Isocitrate dehydrogenase [NAD] subunit beta, mitochondrial OS=Rattus norvegicus GN=Idh3B PE=1 SV=1	VEGAFPVTMLPGDGVGPELMHAVK
>	sp|Q68FX0|IDH	Isocitrate dehydrogenase [NAD] subunit beta, mitochondrial OS=Rattus norvegicus GN=Idh3B PE=1 SV=1	SVIGHLHPHGG
>	sp|Q68FX0|IDH	Isocitrate dehydrogenase [NAD] subunit beta, mitochondrial OS=Rattus norvegicus GN=Idh3B PE=1 SV=1	VEGAFPVTMLPGDGVGPELMHAVK
>	sp|Q68FX0|IDH	Isocitrate dehydrogenase [NAD] subunit beta, mitochondrial OS=Rattus norvegicus GN=Idh3B PE=1 SV=1	VEGAFPVTMLPGDGVGPELMHAVK
>	sp|Q68FX0|IDH	Isocitrate dehydrogenase [NAD] subunit beta, mitochondrial OS=Rattus norvegicus GN=Idh3B PE=1 SV=1	VEGAFPVTMLPGDGVGPELMHAVK
>	sp|Q68FX0|IDH	Isocitrate dehydrogenase [NAD] subunit beta, mitochondrial OS=Rattus norvegicus GN=Idh3B PE=1 SV=1	VEGAFPVTMLPGDGVGPELMHAVK
>	sp|Q68FX0|IDH	Isocitrate dehydrogenase [NAD] subunit beta, mitochondrial OS=Rattus norvegicus GN=Idh3B PE=1 SV=1	VEGAFPVTMLPGDGVGPELMHAVK
>	sp|Q68FX0|IDH	Isocitrate dehydrogenase [NAD] subunit beta, mitochondrial OS=Rattus norvegicus GN=Idh3B PE=1 SV=1	VEGAFPVTMLPGDGVGPELMHAVK
>	sp|Q68FX0|IDH	Isocitrate dehydrogenase [NAD] subunit beta, mitochondrial OS=Rattus norvegicus GN=Idh3B PE=1 SV=1	KLDLFANVVHVK

>	sp|Q68FX0|IDH Isocitrate dehydrogenase [NAD] subunit beta, mitochondrial OS=Rattus norvegicus GN=Idh3B PE=1 SV=1	KLDLFANVVHVK
>	sp|Q68FX0|IDH Isocitrate dehydrogenase [NAD] subunit beta, mitochondrial OS=Rattus norvegicus GN=Idh3B PE=1 SV=1	VEGAFPVTMLPGDGVGPELMHAVK
>	sp|Q68FX0|IDH Isocitrate dehydrogenase [NAD] subunit beta, mitochondrial OS=Rattus norvegicus GN=Idh3B PE=1 SV=1	VEGAFPVTMLPGDGVGPELMHAVK
>	sp|Q68FX0|IDH Isocitrate dehydrogenase [NAD] subunit beta, mitochondrial OS=Rattus norvegicus GN=Idh3B PE=1 SV=1	VEGAFPVTMLPGDGVGPELMHAVK
>	sp|Q68FX0|IDH Isocitrate dehydrogenase [NAD] subunit beta, mitochondrial OS=Rattus norvegicus GN=Idh3B PE=1 SV=1	LGDGLFLQCCEEVAELYPK
>	sp|P54313|GBB Guanine nucleotide-binding protein G(I)/G(S)/G(T) subunit beta-2 OS=Rattus norvegicus GN=Gnb2 PE=1 SV=4	ADQELLMYSHDNIICGITSVAFSR
>	sp|P54313|GBB Guanine nucleotide-binding protein G(I)/G(S)/G(T) subunit beta-2 OS=Rattus norvegicus GN=Gnb2 PE=1 SV=4	QTFIGHESDINAVAFFPNGYAFTTGSDDATCR
>	sp|P54313|GBB Guanine nucleotide-binding protein G(I)/G(S)/G(T) subunit beta-2 OS=Rattus norvegicus GN=Gnb2 PE=1 SV=4	TFVSGACDASIK
>	sp|P54313|GBB Guanine nucleotide-binding protein G(I)/G(S)/G(T) subunit beta-2 OS=Rattus norvegicus GN=Gnb2 PE=1 SV=4	KACGDSTLTQITAGLDPVGR
>	sp|P54313|GBB Guanine nucleotide-binding protein G(I)/G(S)/G(T) subunit beta-2 OS=Rattus norvegicus GN=Gnb2 PE=1 SV=4	ADQELLMYSHDNIICGITSVAFSR
>	sp|P54313|GBB Guanine nucleotide-binding protein G(I)/G(S)/G(T) subunit beta-2 OS=Rattus norvegicus GN=Gnb2 PE=1 SV=4	ADQELLMYSHDNIICGITSVAFSR
>	sp|P54313|GBB Guanine nucleotide-binding protein G(I)/G(S)/G(T) subunit beta-2 OS=Rattus norvegicus GN=Gnb2 PE=1 SV=4	ADQELLMYSHDNIICGITSVAFSR
>	sp|P54313|GBB Guanine nucleotide-binding protein G(I)/G(S)/G(T) subunit beta-2 OS=Rattus norvegicus GN=Gnb2 PE=1 SV=4	ADQELLMYSHDNIICGITSVAFSR
>	sp|P54313|GBB Guanine nucleotide-binding protein G(I)/G(S)/G(T) subunit beta-2 OS=Rattus norvegicus GN=Gnb2 PE=1 SV=4	AGVLAGHDNR
>	sp|P54313|GBB Guanine nucleotide-binding protein G(I)/G(S)/G(T) subunit beta-2 OS=Rattus norvegicus GN=Gnb2 PE=1 SV=4	AGVLAGHDNR
>	sp|P54313|GBB Guanine nucleotide-binding protein G(I)/G(S)/G(T) subunit beta-2 OS=Rattus norvegicus GN=Gnb2 PE=1 SV=4	AGVLAGHDNR
>	sp|P54313|GBB Guanine nucleotide-binding protein G(I)/G(S)/G(T) subunit beta-2 OS=Rattus norvegicus GN=Gnb2 PE=1 SV=4	AGVLAGHDNR
>	sp|P54313|GBB Guanine nucleotide-binding protein G(I)/G(S)/G(T) subunit beta-2 OS=Rattus norvegicus GN=Gnb2 PE=1 SV=4	AGVLAGHDNR
>	sp|P54313|GBB Guanine nucleotide-binding protein G(I)/G(S)/G(T) subunit beta-2 OS=Rattus norvegicus GN=Gnb2 PE=1 SV=4	KACGDSTLTQITAGLDPVGR
>	sp|P54313|GBB Guanine nucleotide-binding protein G(I)/G(S)/G(T) subunit beta-2 OS=Rattus norvegicus GN=Gnb2 PE=1 SV=4	KACGDSTLTQITAGLDPVGR
>	sp|P54313|GBB Guanine nucleotide-binding protein G(I)/G(S)/G(T) subunit beta-2 OS=Rattus norvegicus GN=Gnb2 PE=1 SV=4	KACGDSTLTQITAGLDPVGR
>	sp|P54313|GBB Guanine nucleotide-binding protein G(I)/G(S)/G(T) subunit beta-2 OS=Rattus norvegicus GN=Gnb2 PE=1 SV=4	KACGDSTLTQITAGLDPVGR
>	sp|P54313|GBB Guanine nucleotide-binding protein G(I)/G(S)/G(T) subunit beta-2 OS=Rattus norvegicus GN=Gnb2 PE=1 SV=4	KACGDSTLTQITAGLDPVGR
>	sp|P54313|GBB Guanine nucleotide-binding protein G(I)/G(S)/G(T) subunit beta-2 OS=Rattus norvegicus GN=Gnb2 PE=1 SV=4	LLVSASQDGK
>	sp|P54313|GBB Guanine nucleotide-binding protein G(I)/G(S)/G(T) subunit beta-2 OS=Rattus norvegicus GN=Gnb2 PE=1 SV=4	LLVSASQDGK
>	sp|P54313|GBB Guanine nucleotide-binding protein G(I)/G(S)/G(T) subunit beta-2 OS=Rattus norvegicus GN=Gnb2 PE=1 SV=4	QTFIGHESDINAVAFFPNGYAFTTGSDDATCR
>	sp|P54313|GBB Guanine nucleotide-binding protein G(I)/G(S)/G(T) subunit beta-2 OS=Rattus norvegicus GN=Gnb2 PE=1 SV=4	VSCLGVTDDGMAVATGSWDSFLK
>	sp|P54313|GBB Guanine nucleotide-binding protein G(I)/G(S)/G(T) subunit beta-2 OS=Rattus norvegicus GN=Gnb2 PE=1 SV=4	VSCLGVTDDGMAVATGSWDSFLK
>	sp|P54313|GBB Guanine nucleotide-binding protein G(I)/G(S)/G(T) subunit beta-2 OS=Rattus norvegicus GN=Gnb2 PE=1 SV=4	TFVSGACDASIK
>	sp|P54313|GBB Guanine nucleotide-binding protein G(I)/G(S)/G(T) subunit beta-2 OS=Rattus norvegicus GN=Gnb2 PE=1 SV=4	VSCLGVTDDGMAVATGSWDSFLK
>	sp|P54313|GBB Guanine nucleotide-binding protein G(I)/G(S)/G(T) subunit beta-2 OS=Rattus norvegicus GN=Gnb2 PE=1 SV=4	LLVSASQDGK
>	sp|P54313|GBB Guanine nucleotide-binding protein G(I)/G(S)/G(T) subunit beta-2 OS=Rattus norvegicus GN=Gnb2 PE=1 SV=4	VSCLGVTDDGMAVATGSWDSFLK
>	sp|P54313|GBB Guanine nucleotide-binding protein G(I)/G(S)/G(T) subunit beta-2 OS=Rattus norvegicus GN=Gnb2 PE=1 SV=4	LLVSASQDGK
>	sp|P54313|GBB Guanine nucleotide-binding protein G(I)/G(S)/G(T) subunit beta-2 OS=Rattus norvegicus GN=Gnb2 PE=1 SV=4	IYAMHWGTDSR
>	sp|P54313|GBB Guanine nucleotide-binding protein G(I)/G(S)/G(T) subunit beta-2 OS=Rattus norvegicus GN=Gnb2 PE=1 SV=4	LFDLR
>	sp|P54313|GBB Guanine nucleotide-binding protein G(I)/G(S)/G(T) subunit beta-2 OS=Rattus norvegicus GN=Gnb2 PE=1 SV=4	LIIWDSYTTNKVHAIPLR
>	sp|P54313|GBB Guanine nucleotide-binding protein G(I)/G(S)/G(T) subunit beta-2 OS=Rattus norvegicus GN=Gnb2 PE=1 SV=4	QTFIGHESDINAVAFFPNGYAFTTGSDDATCR
>	sp|P11506|AT2 Plasma membrane calcium-transporting ATPase 2 OS=Rattus norvegicus GN=Atp2b2 PE=2 SV=2	AGQVVQIPVAEIVVGDIAQIK
>	sp|P11506|AT2 Plasma membrane calcium-transporting ATPase 2 OS=Rattus norvegicus GN=Atp2b2 PE=2 SV=2	LTQKEEIPEEELNEDVEEIDHAER
>	sp|P11506|AT2 Plasma membrane calcium-transporting ATPase 2 OS=Rattus norvegicus GN=Atp2b2 PE=2 SV=2	MVVTAVGVNSQTGIIFTLLGAGGEEEEKKDKK
>	sp|P11506|AT2 Plasma membrane calcium-transporting ATPase 2 OS=Rattus norvegicus GN=Atp2b2 PE=2 SV=2	QVGNKTECGLLGFVLDLR
>	sp|P11506|AT2 Plasma membrane calcium-transporting ATPase 2 OS=Rattus norvegicus GN=Atp2b2 PE=2 SV=2	TSIHNFMAHPEFR
>	sp|P11506|AT2 Plasma membrane calcium-transporting ATPase 2 OS=Rattus norvegicus GN=Atp2b2 PE=2 SV=2	AGQVVQIPVAEIVVGDIAQIK
>	sp|P11506|AT2 Plasma membrane calcium-transporting ATPase 2 OS=Rattus norvegicus GN=Atp2b2 PE=2 SV=2	AGQVVQIPVAEIVVGDIAQIK
>	sp|P11506|AT2 Plasma membrane calcium-transporting ATPase 2 OS=Rattus norvegicus GN=Atp2b2 PE=2 SV=2	CGIIHPGEDFLCLEGK
>	sp|P11506|AT2 Plasma membrane calcium-transporting ATPase 2 OS=Rattus norvegicus GN=Atp2b2 PE=2 SV=2	HLDACETMGNATAICSDK
>	sp|P11506|AT2 Plasma membrane calcium-transporting ATPase 2 OS=Rattus norvegicus GN=Atp2b2 PE=2 SV=2	KADVGFAMGIAGTDVAK
>	sp|P11506|AT2 Plasma membrane calcium-transporting ATPase 2 OS=Rattus norvegicus GN=Atp2b2 PE=2 SV=2	KADVGFAMGIAGTDVAK
>	sp|P11506|AT2 Plasma membrane calcium-transporting ATPase 2 OS=Rattus norvegicus GN=Atp2b2 PE=2 SV=2	KADVGFAMGIAGTDVAK
>	sp|P11506|AT2 Plasma membrane calcium-transporting ATPase 2 OS=Rattus norvegicus GN=Atp2b2 PE=2 SV=2	KADVGFAMGIAGTDVAK
>	sp|P11506|AT2 Plasma membrane calcium-transporting ATPase 2 OS=Rattus norvegicus GN=Atp2b2 PE=2 SV=2	KADVGFAMGIAGTDVAK
>	sp|P11506|AT2 Plasma membrane calcium-transporting ATPase 2 OS=Rattus norvegicus GN=Atp2b2 PE=2 SV=2	LTQKEEIPEEELNEDVEEIDHAER
>	sp|P11506|AT2 Plasma membrane calcium-transporting ATPase 2 OS=Rattus norvegicus GN=Atp2b2 PE=2 SV=2	MVTGDNINTAR
>	sp|P11506|AT2 Plasma membrane calcium-transporting ATPase 2 OS=Rattus norvegicus GN=Atp2b2 PE=2 SV=2	MVVTAVGVNSQTGIIFTLLGAGGEEEEKKDKK
>	sp|P11506|AT2 Plasma membrane calcium-transporting ATPase 2 OS=Rattus norvegicus GN=Atp2b2 PE=2 SV=2	NEKGEIEQER
>	sp|P11506|AT2 Plasma membrane calcium-transporting ATPase 2 OS=Rattus norvegicus GN=Atp2b2 PE=2 SV=2	NEKGEIEQER
>	sp|P11506|AT2 Plasma membrane calcium-transporting ATPase 2 OS=Rattus norvegicus GN=Atp2b2 PE=2 SV=2	QVVAVTGDGTNDGPALK
>	sp|P11506|AT2 Plasma membrane calcium-transporting ATPase 2 OS=Rattus norvegicus GN=Atp2b2 PE=2 SV=2	HLDACETMGNATAICSDK
>	sp|P11506|AT2 Plasma membrane calcium-transporting ATPase 2 OS=Rattus norvegicus GN=Atp2b2 PE=2 SV=2	ANMHKK
>	sp|P11506|AT2 Plasma membrane calcium-transporting ATPase 2 OS=Rattus norvegicus GN=Atp2b2 PE=2 SV=2	HLDACETMGNATAICSDK
>	sp|P11506|AT2 Plasma membrane calcium-transporting ATPase 2 OS=Rattus norvegicus GN=Atp2b2 PE=2 SV=2	MVVTAVGVNSQTGIIFTLLGAGGEEEEKKDKK
>	sp|P11506|AT2 Plasma membrane calcium-transporting ATPase 2 OS=Rattus norvegicus GN=Atp2b2 PE=2 SV=2	MVVTAVGVNSQTGIIFTLLGAGGEEEEKKDKK
>	sp|P11506|AT2 Plasma membrane calcium-transporting ATPase 2 OS=Rattus norvegicus GN=Atp2b2 PE=2 SV=2	YGDLLPADGLFIQGNDLK
>	sp|P11506|AT2 Plasma membrane calcium-transporting ATPase 2 OS=Rattus norvegicus GN=Atp2b2 PE=2 SV=2	RGQILWFR
>	sp|P11506|AT2 Plasma membrane calcium-transporting ATPase 2 OS=Rattus norvegicus GN=Atp2b2 PE=2 SV=2	HTLVK
>	sp|P11506|AT2 Plasma membrane calcium-transporting ATPase 2 OS=Rattus norvegicus GN=Atp2b2 PE=2 SV=2	LTQKEEIPEEELNEDVEEIDHAER
>	sp|P11506|AT2 Plasma membrane calcium-transporting ATPase 2 OS=Rattus norvegicus GN=Atp2b2 PE=2 SV=2	HLDACETMGNATAICSDK
>	sp|P20788|UCR Cytochrome b-c1 complex subunit Rieske, mitochondrial OS=Rattus norvegicus GN=Uqcrfs1 PE=1 SV=2	EIDQEAAVEVSQLRDPQHDLER
>	sp|P20788|UCR Cytochrome b-c1 complex subunit Rieske, mitochondrial OS=Rattus norvegicus GN=Uqcrfs1 PE=1 SV=2	TKKEIDQEAAVEVSQLRDPQHDLER
>	sp|P20788|UCR Cytochrome b-c1 complex subunit Rieske, mitochondrial OS=Rattus norvegicus GN=Uqcrfs1 PE=1 SV=2	NAVSQFVSSMSASADVLAMSK
>	sp|P20788|UCR Cytochrome b-c1 complex subunit Rieske, mitochondrial OS=Rattus norvegicus GN=Uqcrfs1 PE=1 SV=2	GVAGALRPLLQSAVPATSEPPVLDVK
>	sp|P20788|UCR Cytochrome b-c1 complex subunit Rieske, mitochondrial OS=Rattus norvegicus GN=Uqcrfs1 PE=1 SV=2	EIDQEAAVEVSQLRDPQHDLER
>	sp|P20788|UCR Cytochrome b-c1 complex subunit Rieske, mitochondrial OS=Rattus norvegicus GN=Uqcrfs1 PE=1 SV=2	EIDQEAAVEVSQLRDPQHDLER
>	sp|P20788|UCR Cytochrome b-c1 complex subunit Rieske, mitochondrial OS=Rattus norvegicus GN=Uqcrfs1 PE=1 SV=2	EIDQEAAVEVSQLRDPQHDLER

>	sp|P20788|UCF	Cytochrome b-c1 complex subunit Rieske, mitochondrial OS=Rattus norvegicus GN=Uqcrfs1 PE=1 SV=2	EIDQEAAVEVSQLRDPQHDLER
>	sp|P20788|UCF	Cytochrome b-c1 complex subunit Rieske, mitochondrial OS=Rattus norvegicus GN=Uqcrfs1 PE=1 SV=2	EIDQEAAVEVSQLRDPQHDLER
>	sp|P20788|UCF	Cytochrome b-c1 complex subunit Rieske, mitochondrial OS=Rattus norvegicus GN=Uqcrfs1 PE=1 SV=2	EIDQEAAVEVSQLRDPQHDLER
>	sp|P20788|UCF	Cytochrome b-c1 complex subunit Rieske, mitochondrial OS=Rattus norvegicus GN=Uqcrfs1 PE=1 SV=2	EIDQEAAVEVSQLRDPQHDLER
>	sp|P20788|UCF	Cytochrome b-c1 complex subunit Rieske, mitochondrial OS=Rattus norvegicus GN=Uqcrfs1 PE=1 SV=2	EIDQEAAVEVSQLRDPQHDLER
>	sp|P20788|UCF	Cytochrome b-c1 complex subunit Rieske, mitochondrial OS=Rattus norvegicus GN=Uqcrfs1 PE=1 SV=2	EIDQEAAVEVSQLRDPQHDLER
>	sp|P20788|UCF	Cytochrome b-c1 complex subunit Rieske, mitochondrial OS=Rattus norvegicus GN=Uqcrfs1 PE=1 SV=2	EIDQEAAVEVSQLRDPQHDLER
>	sp|P20788|UCF	Cytochrome b-c1 complex subunit Rieske, mitochondrial OS=Rattus norvegicus GN=Uqcrfs1 PE=1 SV=2	GVAGALRPLLQSAVPATSEPPVLDVK
>	sp|P20788|UCF	Cytochrome b-c1 complex subunit Rieske, mitochondrial OS=Rattus norvegicus GN=Uqcrfs1 PE=1 SV=2	GVAGALRPLLQSAVPATSEPPVLDVK
>	sp|P20788|UCF	Cytochrome b-c1 complex subunit Rieske, mitochondrial OS=Rattus norvegicus GN=Uqcrfs1 PE=1 SV=2	GVAGALRPLLQSAVPATSEPPVLDVK
>	sp|P20788|UCF	Cytochrome b-c1 complex subunit Rieske, mitochondrial OS=Rattus norvegicus GN=Uqcrfs1 PE=1 SV=2	GVAGALRPLLQSAVPATSEPPVLDVK
>	sp|P20788|UCF	Cytochrome b-c1 complex subunit Rieske, mitochondrial OS=Rattus norvegicus GN=Uqcrfs1 PE=1 SV=2	GVAGALRPLLQSAVPATSEPPVLDVK
>	sp|P20788|UCF	Cytochrome b-c1 complex subunit Rieske, mitochondrial OS=Rattus norvegicus GN=Uqcrfs1 PE=1 SV=2	GVAGALRPLLQSAVPATSEPPVLDVK
>	sp|P20788|UCF	Cytochrome b-c1 complex subunit Rieske, mitochondrial OS=Rattus norvegicus GN=Uqcrfs1 PE=1 SV=2	GVAGALRPLLQSAVPATSEPPVLDVK
>	sp|P20788|UCF	Cytochrome b-c1 complex subunit Rieske, mitochondrial OS=Rattus norvegicus GN=Uqcrfs1 PE=1 SV=2	GVAGALRPLLQSAVPATSEPPVLDVK
>	sp|P20788|UCF	Cytochrome b-c1 complex subunit Rieske, mitochondrial OS=Rattus norvegicus GN=Uqcrfs1 PE=1 SV=2	TKKEIDQEAAVEVSQLRDPQHDLER
>	sp|P20788|UCF	Cytochrome b-c1 complex subunit Rieske, mitochondrial OS=Rattus norvegicus GN=Uqcrfs1 PE=1 SV=2	GVAGALRPLLQSAVPATSEPPVLDVK
>	sp|P20788|UCF	Cytochrome b-c1 complex subunit Rieske, mitochondrial OS=Rattus norvegicus GN=Uqcrfs1 PE=1 SV=2	RAEVLDSTK
>	sp|P35704|PRD	Peroxiredoxin-2 OS=Rattus norvegicus GN=Prdx2 PE=1 SV=3	KEGGLGPLNIPLLADVTK
>	sp|P35704|PRD	Peroxiredoxin-2 OS=Rattus norvegicus GN=Prdx2 PE=1 SV=3	LVQAFQYTDEHGEVCPAGWKPGSDTIKPNVDDSKEYFSK
>	sp|P35704|PRD	Peroxiredoxin-2 OS=Rattus norvegicus GN=Prdx2 PE=1 SV=3	PLLADVTK
>	sp|P35704|PRD	Peroxiredoxin-2 OS=Rattus norvegicus GN=Prdx2 PE=1 SV=3	KLGCEVLGVSVDSQFTHLAWINTPR
>	sp|P35704|PRD	Peroxiredoxin-2 OS=Rattus norvegicus GN=Prdx2 PE=1 SV=3	KEGGLGPLNIPLLADVTK
>	sp|P35704|PRD	Peroxiredoxin-2 OS=Rattus norvegicus GN=Prdx2 PE=1 SV=3	KEGGLGPLNIPLLADVTK
>	sp|P35704|PRD	Peroxiredoxin-2 OS=Rattus norvegicus GN=Prdx2 PE=1 SV=3	KEGGLGPLNIPLLADVTK
>	sp|P35704|PRD	Peroxiredoxin-2 OS=Rattus norvegicus GN=Prdx2 PE=1 SV=3	KEGGLGPLNIPLLADVTK
>	sp|P35704|PRD	Peroxiredoxin-2 OS=Rattus norvegicus GN=Prdx2 PE=1 SV=3	KEGGLGPLNIPLLADVTK
>	sp|P35704|PRD	Peroxiredoxin-2 OS=Rattus norvegicus GN=Prdx2 PE=1 SV=3	KEGGLGPLNIPLLADVTK
>	sp|P35704|PRD	Peroxiredoxin-2 OS=Rattus norvegicus GN=Prdx2 PE=1 SV=3	KEGGLGPLNIPLLADVTK
>	sp|P35704|PRD	Peroxiredoxin-2 OS=Rattus norvegicus GN=Prdx2 PE=1 SV=3	KEGGLGPLNIPLLADVTK
>	sp|P35704|PRD	Peroxiredoxin-2 OS=Rattus norvegicus GN=Prdx2 PE=1 SV=3	KEGGLGPLNIPLLADVTK
>	sp|P35704|PRD	Peroxiredoxin-2 OS=Rattus norvegicus GN=Prdx2 PE=1 SV=3	KEGGLGPLNIPLLADVTK
>	sp|P35704|PRD	Peroxiredoxin-2 OS=Rattus norvegicus GN=Prdx2 PE=1 SV=3	KEGGLGPLNIPLLADVTK
>	sp|P35704|PRD	Peroxiredoxin-2 OS=Rattus norvegicus GN=Prdx2 PE=1 SV=3	KEGGLGPLNIPLLADVTK
>	sp|P35704|PRD	Peroxiredoxin-2 OS=Rattus norvegicus GN=Prdx2 PE=1 SV=3	KEGGLGPLNIPLLADVTK
>	sp|P35704|PRD	Peroxiredoxin-2 OS=Rattus norvegicus GN=Prdx2 PE=1 SV=3	KEGGLGPLNIPLLADVTK
>	sp|P35704|PRD	Peroxiredoxin-2 OS=Rattus norvegicus GN=Prdx2 PE=1 SV=3	KEGGLGPLNIPLLADVTK
>	sp|P35704|PRD	Peroxiredoxin-2 OS=Rattus norvegicus GN=Prdx2 PE=1 SV=3	KEGGLGPLNIPLLADVTK
>	sp|P35704|PRD	Peroxiredoxin-2 OS=Rattus norvegicus GN=Prdx2 PE=1 SV=3	LVQAFQYTDEHGEVCPAGWKPGSDTIKPNVDDSKEYFSK
>	sp|P35704|PRD	Peroxiredoxin-2 OS=Rattus norvegicus GN=Prdx2 PE=1 SV=3	LVQAFQYTDEHGEVCPAGWKPGSDTIKPNVDDSKEYFSK
>	sp|P35704|PRD	Peroxiredoxin-2 OS=Rattus norvegicus GN=Prdx2 PE=1 SV=3	KEGGLGPLNIPLLADVTK
>	sp|P35704|PRD	Peroxiredoxin-2 OS=Rattus norvegicus GN=Prdx2 PE=1 SV=3	GLFIIDAK
>	sp|P35704|PRD	Peroxiredoxin-2 OS=Rattus norvegicus GN=Prdx2 PE=1 SV=3	LVQAFQYTDEHGEVCPAGWKPGSDTIKPNVDDSKEYFSK
>	sp|P35704|PRD	Peroxiredoxin-2 OS=Rattus norvegicus GN=Prdx2 PE=1 SV=3	KEGGLGPLNIPLLADVTK
>	sp|P35704|PRD	Peroxiredoxin-2 OS=Rattus norvegicus GN=Prdx2 PE=1 SV=3	KEGGLGPLNIPLLADVTK
>	sp|P35704|PRD	Peroxiredoxin-2 OS=Rattus norvegicus GN=Prdx2 PE=1 SV=3	KEGGLGPLNIPLLADVTK
>	sp|P35704|PRD	Peroxiredoxin-2 OS=Rattus norvegicus GN=Prdx2 PE=1 SV=3	GLFIIDAK
>	sp|P35704|PRD	Peroxiredoxin-2 OS=Rattus norvegicus GN=Prdx2 PE=1 SV=3	LVQAFQYTDEHGEVCPAGWKPGSDTIKPNVDDSK
>	sp|P35704|PRD	Peroxiredoxin-2 OS=Rattus norvegicus GN=Prdx2 PE=1 SV=3	KEGGLGPLNIPLLADVTK
>	sp|P35704|PRD	Peroxiredoxin-2 OS=Rattus norvegicus GN=Prdx2 PE=1 SV=3	GLFIIDAK
>	sp|P35704|PRD	Peroxiredoxin-2 OS=Rattus norvegicus GN=Prdx2 PE=1 SV=3	KEGGLGPLNIPLLADVTK
>	sp|P35704|PRD	Peroxiredoxin-2 OS=Rattus norvegicus GN=Prdx2 PE=1 SV=3	KEGGLGPLNIPLLADVTK
>	sp|P35704|PRD	Peroxiredoxin-2 OS=Rattus norvegicus GN=Prdx2 PE=1 SV=3	KEGGLGPLNIPLLADVTK
>	sp|P35704|PRD	Peroxiredoxin-2 OS=Rattus norvegicus GN=Prdx2 PE=1 SV=3	LVQAFQYTDEHGEVCPAGWKPGSDTIKPNVDDSKEYFSK
>	sp|P35704|PRD	Peroxiredoxin-2 OS=Rattus norvegicus GN=Prdx2 PE=1 SV=3	KEGGLGPLNIPLLADVTK
>	sp|P35704|PRD	Peroxiredoxin-2 OS=Rattus norvegicus GN=Prdx2 PE=1 SV=3	KEGGLGPLNIPLLADVTK
>	sp|P35704|PRD	Peroxiredoxin-2 OS=Rattus norvegicus GN=Prdx2 PE=1 SV=3	KEGGLGPLNIPLLADVTK
>	sp|P35704|PRD	Peroxiredoxin-2 OS=Rattus norvegicus GN=Prdx2 PE=1 SV=3	KEGGLGPLNIPLLADVTK
>	sp|P35704|PRD	Peroxiredoxin-2 OS=Rattus norvegicus GN=Prdx2 PE=1 SV=3	GLFIIDAK
>	sp|P35704|PRD	Peroxiredoxin-2 OS=Rattus norvegicus GN=Prdx2 PE=1 SV=3	LVQAFQYTDEHGEVCPAGWKPGSDTIKPNVDDSKEYFSK
>	sp|P35704|PRD	Peroxiredoxin-2 OS=Rattus norvegicus GN=Prdx2 PE=1 SV=3	LVQAFQYTDEHGEVCPAGWKPGSDTIKPNVDDSK
>	sp|P85969|SNA	Beta-soluble NSF attachment protein OS=Rattus norvegicus GN=Napb PE=1 SV=1	AALCHFIVDELNAK
>	sp|P85969|SNA	Beta-soluble NSF attachment protein OS=Rattus norvegicus GN=Napb PE=1 SV=1	LALEKYEEMFPAFTDSR
>	sp|P85969|SNA	Beta-soluble NSF attachment protein OS=Rattus norvegicus GN=Napb PE=1 SV=1	LHMQLQSK
>	sp|P85969|SNA	Beta-soluble NSF attachment protein OS=Rattus norvegicus GN=Napb PE=1 SV=1	HHITIAEIYETELVDIEK
>	sp|P85969|SNA	Beta-soluble NSF attachment protein OS=Rattus norvegicus GN=Napb PE=1 SV=1	AALCHFIVDELNAK
>	sp|P85969|SNA	Beta-soluble NSF attachment protein OS=Rattus norvegicus GN=Napb PE=1 SV=1	AALCHFIVDELNAK
>	sp|P85969|SNA	Beta-soluble NSF attachment protein OS=Rattus norvegicus GN=Napb PE=1 SV=1	AALCHFIVDELNAK
>	sp|P85969|SNA	Beta-soluble NSF attachment protein OS=Rattus norvegicus GN=Napb PE=1 SV=1	AALCHFIVDELNAK
>	sp|P85969|SNA	Beta-soluble NSF attachment protein OS=Rattus norvegicus GN=Napb PE=1 SV=1	AALCHFIVDELNAK
>	sp|P85969|SNA	Beta-soluble NSF attachment protein OS=Rattus norvegicus GN=Napb PE=1 SV=1	AALCHFIVDELNAK
>	sp|P85969|SNA	Beta-soluble NSF attachment protein OS=Rattus norvegicus GN=Napb PE=1 SV=1	AALCHFIVDELNAK
>	sp|P85969|SNA	Beta-soluble NSF attachment protein OS=Rattus norvegicus GN=Napb PE=1 SV=1	AALCHFIVDELNAK
>	sp|P85969|SNA	Beta-soluble NSF attachment protein OS=Rattus norvegicus GN=Napb PE=1 SV=1	AALCHFIVDELNAK

>	sp|P85969|SN/	Beta-soluble NSF attachment protein OS=Rattus norvegicus GN=Napb PE=1 SV=1	AALCHFIVDELNAK
>	sp|P85969|SN/	Beta-soluble NSF attachment protein OS=Rattus norvegicus GN=Napb PE=1 SV=1	LALEKYEEMFPAFTDSR
>	sp|P85969|SN/	Beta-soluble NSF attachment protein OS=Rattus norvegicus GN=Napb PE=1 SV=1	HHITIAEIYETELVDIEK
>	sp|P85969|SN/	Beta-soluble NSF attachment protein OS=Rattus norvegicus GN=Napb PE=1 SV=1	AALCHFIVDELNAK
>	sp|P85969|SN/	Beta-soluble NSF attachment protein OS=Rattus norvegicus GN=Napb PE=1 SV=1	HHITIAEIYETELVDIEK
>	sp|P37377|SYL	Alpha-synuclein OS=Rattus norvegicus GN=Snca PE=1 SV=1	TKEGVVHGVTTVAEK
>	sp|P37377|SYL	Alpha-synuclein OS=Rattus norvegicus GN=Snca PE=1 SV=1	TKEQVTNVGGAVVTGVTAVAQK
>	sp|P37377|SYL	Alpha-synuclein OS=Rattus norvegicus GN=Snca PE=1 SV=1	TVEGAGNIAAATGFVK
>	sp|P37377|SYL	Alpha-synuclein OS=Rattus norvegicus GN=Snca PE=1 SV=1	AKEGVVAAAEK
>	sp|P37377|SYL	Alpha-synuclein OS=Rattus norvegicus GN=Snca PE=1 SV=1	AKEGVVAAAEK
>	sp|P37377|SYL	Alpha-synuclein OS=Rattus norvegicus GN=Snca PE=1 SV=1	EGVVAAAEK
>	sp|P37377|SYL	Alpha-synuclein OS=Rattus norvegicus GN=Snca PE=1 SV=1	EGVVAAAEK
>	sp|P37377|SYL	Alpha-synuclein OS=Rattus norvegicus GN=Snca PE=1 SV=1	EGVVAAAEK
>	sp|P37377|SYL	Alpha-synuclein OS=Rattus norvegicus GN=Snca PE=1 SV=1	EGVVAAAEK
>	sp|P37377|SYL	Alpha-synuclein OS=Rattus norvegicus GN=Snca PE=1 SV=1	TKEGVLYVGSK
>	sp|P37377|SYL	Alpha-synuclein OS=Rattus norvegicus GN=Snca PE=1 SV=1	TKEGVLYVGSK
>	sp|P37377|SYL	Alpha-synuclein OS=Rattus norvegicus GN=Snca PE=1 SV=1	TKEQVTNVGGAVVTGVTAVAQK
>	sp|P37377|SYL	Alpha-synuclein OS=Rattus norvegicus GN=Snca PE=1 SV=1	TKEQVTNVGGAVVTGVTAVAQK
>	sp|P37377|SYL	Alpha-synuclein OS=Rattus norvegicus GN=Snca PE=1 SV=1	TKEQVTNVGGAVVTGVTAVAQK
>	sp|P37377|SYL	Alpha-synuclein OS=Rattus norvegicus GN=Snca PE=1 SV=1	TKEQVTNVGGAVVTGVTAVAQK
>	sp|P37377|SYL	Alpha-synuclein OS=Rattus norvegicus GN=Snca PE=1 SV=1	TKEQVTNVGGAVVTGVTAVAQK
>	sp|P37377|SYL	Alpha-synuclein OS=Rattus norvegicus GN=Snca PE=1 SV=1	TKEQVTNVGGAVVTGVTAVAQK
>	sp|P37377|SYL	Alpha-synuclein OS=Rattus norvegicus GN=Snca PE=1 SV=1	TKEQVTNVGGAVVTGVTAVAQK
>	sp|P37377|SYL	Alpha-synuclein OS=Rattus norvegicus GN=Snca PE=1 SV=1	TKEQVTNVGGAVVTGVTAVAQK
>	sp|P37377|SYL	Alpha-synuclein OS=Rattus norvegicus GN=Snca PE=1 SV=1	TKEQVTNVGGAVVTGVTAVAQK
>	sp|P37377|SYL	Alpha-synuclein OS=Rattus norvegicus GN=Snca PE=1 SV=1	TKEQVTNVGGAVVTGVTAVAQK
>	sp|P37377|SYL	Alpha-synuclein OS=Rattus norvegicus GN=Snca PE=1 SV=1	TVEGAGNIAAATGFVK
>	sp|P37377|SYL	Alpha-synuclein OS=Rattus norvegicus GN=Snca PE=1 SV=1	TVEGAGNIAAATGFVK
>	sp|P37377|SYL	Alpha-synuclein OS=Rattus norvegicus GN=Snca PE=1 SV=1	TVEGAGNIAAATGFVK
>	sp|P37377|SYL	Alpha-synuclein OS=Rattus norvegicus GN=Snca PE=1 SV=1	TVEGAGNIAAATGFVK
>	sp|P37377|SYL	Alpha-synuclein OS=Rattus norvegicus GN=Snca PE=1 SV=1	EGVVAAAEK
>	sp|P37377|SYL	Alpha-synuclein OS=Rattus norvegicus GN=Snca PE=1 SV=1	TKEQVTNVGGAVVTGVTAVAQK
>	sp|P37377|SYL	Alpha-synuclein OS=Rattus norvegicus GN=Snca PE=1 SV=1	TKEQVTNVGGAVVTGVTAVAQK
>	sp|P37377|SYL	Alpha-synuclein OS=Rattus norvegicus GN=Snca PE=1 SV=1	TKEQVTNVGGAVVTGVTAVAQK
>	sp|P37377|SYL	Alpha-synuclein OS=Rattus norvegicus GN=Snca PE=1 SV=1	TKEQVTNVGGAVVTGVTAVAQK
>	sp|P37377|SYL	Alpha-synuclein OS=Rattus norvegicus GN=Snca PE=1 SV=1	TKEQVTNVGGAVVTGVTAVAQK
>	sp|P37377|SYL	Alpha-synuclein OS=Rattus norvegicus GN=Snca PE=1 SV=1	TKQGVAEAAGK
>	sp|P37377|SYL	Alpha-synuclein OS=Rattus norvegicus GN=Snca PE=1 SV=1	TKEGVVHGVTTVAEK
>	sp|Q00715|H2I	Histone H2B type 1 OS=Rattus norvegicus PE=1 SV=2	HAVSEGTK
>	sp|Q00715|H2I	Histone H2B type 1 OS=Rattus norvegicus PE=1 SV=2	LLLPGELAK
>	sp|Q00715|H2I	Histone H2B type 1 OS=Rattus norvegicus PE=1 SV=2	QVHPDTGISSK
>	sp|Q00715|H2I	Histone H2B type 1 OS=Rattus norvegicus PE=1 SV=2	HAVSEGTK
>	sp|Q00715|H2I	Histone H2B type 1 OS=Rattus norvegicus PE=1 SV=2	HAVSEGTK
>	sp|Q00715|H2I	Histone H2B type 1 OS=Rattus norvegicus PE=1 SV=2	HAVSEGTK
>	sp|Q00715|H2I	Histone H2B type 1 OS=Rattus norvegicus PE=1 SV=2	AMGIMNSFVNDIFER
>	sp|Q00715|H2I	Histone H2B type 1 OS=Rattus norvegicus PE=1 SV=2	LLLPGELAK
>	sp|Q00715|H2I	Histone H2B type 1 OS=Rattus norvegicus PE=1 SV=2	LLLPGELAK
>	sp|Q00715|H2I	Histone H2B type 1 OS=Rattus norvegicus PE=1 SV=2	QVHPDTGISSK
>	sp|Q9JLT0|MYI	Myosin-10 OS=Rattus norvegicus GN=Myh10 PE=1 SV=1	AGVLAHLEEER
>	sp|Q9JLT0|MYI	Myosin-10 OS=Rattus norvegicus GN=Myh10 PE=1 SV=1	KQELEEILHDLESR
>	sp|Q9JLT0|MYI	Myosin-10 OS=Rattus norvegicus GN=Myh10 PE=1 SV=1	LDPHLVLDQLR
>	sp|Q9JLT0|MYI	Myosin-10 OS=Rattus norvegicus GN=Myh10 PE=1 SV=1	KQELEEILHDLESR
>	sp|Q9JLT0|MYI	Myosin-10 OS=Rattus norvegicus GN=Myh10 PE=1 SV=1	LDPHLVLDQLR
>	sp|Q9JLT0|MYI	Myosin-10 OS=Rattus norvegicus GN=Myh10 PE=1 SV=1	LDPHLVLDQLR
>	sp|Q9JLT0|MYI	Myosin-10 OS=Rattus norvegicus GN=Myh10 PE=1 SV=1	HATALEELSEQLEQAK
>	sp|Q9JLT0|MYI	Myosin-10 OS=Rattus norvegicus GN=Myh10 PE=1 SV=1	KQELEEILHDLESR
>	sp|Q9JLT0|MYI	Myosin-10 OS=Rattus norvegicus GN=Myh10 PE=1 SV=1	LDPHLVLDQLR
>	sp|Q9JLT0|MYI	Myosin-10 OS=Rattus norvegicus GN=Myh10 PE=1 SV=1	AGKLDPHLVLDQLR
>	sp|Q9JLT0|MYI	Myosin-10 OS=Rattus norvegicus GN=Myh10 PE=1 SV=1	YLFVDR
>	sp|Q9JLT0|MYI	Myosin-10 OS=Rattus norvegicus GN=Myh10 PE=1 SV=1	AGVLAHLEEER
>	sp|Q9JLT0|MYI	Myosin-10 OS=Rattus norvegicus GN=Myh10 PE=1 SV=1	KHQQLLEEK
>	sp|Q9JLT0|MYI	Myosin-10 OS=Rattus norvegicus GN=Myh10 PE=1 SV=1	KDHNIPGELER
>	sp|Q99NA5|IDI	Isocitrate dehydrogenase [NAD] subunit alpha, mitochondrial OS=Rattus norvegicus GN=Idh3a PE=1 SV=1	IAEFAFEYAR
>	sp|Q99NA5|IDI	Isocitrate dehydrogenase [NAD] subunit alpha, mitochondrial OS=Rattus norvegicus GN=Idh3a PE=1 SV=1	AGGVQTVTLIPGDGIGPEISASVMK
>	sp|Q99NA5|IDI	Isocitrate dehydrogenase [NAD] subunit alpha, mitochondrial OS=Rattus norvegicus GN=Idh3a PE=1 SV=1	IEAACFATIKDGK
>	sp|Q99NA5|IDI	Isocitrate dehydrogenase [NAD] subunit alpha, mitochondrial OS=Rattus norvegicus GN=Idh3a PE=1 SV=1	IAEFAFEYAR
>	sp|Q99NA5|IDI	Isocitrate dehydrogenase [NAD] subunit alpha, mitochondrial OS=Rattus norvegicus GN=Idh3a PE=1 SV=1	APIQWEER
>	sp|Q99NA5|IDI	Isocitrate dehydrogenase [NAD] subunit alpha, mitochondrial OS=Rattus norvegicus GN=Idh3a PE=1 SV=1	IAEFAFEYAR
>	sp|Q99NA5|IDI	Isocitrate dehydrogenase [NAD] subunit alpha, mitochondrial OS=Rattus norvegicus GN=Idh3a PE=1 SV=1	IFDAAK
>	sp|Q99NA5|IDI	Isocitrate dehydrogenase [NAD] subunit alpha, mitochondrial OS=Rattus norvegicus GN=Idh3a PE=1 SV=1	IEAACFATIK
>	sp|Q99NA5|IDI	Isocitrate dehydrogenase [NAD] subunit alpha, mitochondrial OS=Rattus norvegicus GN=Idh3a PE=1 SV=1	WMIPPEAK
>	sp|Q99NA5|IDI	Isocitrate dehydrogenase [NAD] subunit alpha, mitochondrial OS=Rattus norvegicus GN=Idh3a PE=1 SV=1	TPYTDVNIVTIR
>	sp|Q99NA5|IDI	Isocitrate dehydrogenase [NAD] subunit alpha, mitochondrial OS=Rattus norvegicus GN=Idh3a PE=1 SV=1	APIQWEER
>	sp|Q5M9I5|QC	Cytochrome b-c1 complex subunit 6, mitochondrial OS=Rattus norvegicus GN=Uqcrh PE=2 SV=1	DHCVAHK

>	sp|Q5M9I5|QC	Cytochrome b-c1 complex subunit 6, mitochondrial OS=Rattus norvegicus GN=Uqcrh PE=2 SV=1	SQTEEDCTEELFDFLHAR
>	sp|Q5M9I5|QC	Cytochrome b-c1 complex subunit 6, mitochondrial OS=Rattus norvegicus GN=Uqcrh PE=2 SV=1	GDPKEEEEEELVDPLTTVR
>	sp|Q5M9I5|QC	Cytochrome b-c1 complex subunit 6, mitochondrial OS=Rattus norvegicus GN=Uqcrh PE=2 SV=1	GDPKEEEEEELVDPLTTVR
>	sp|Q5M9I5|QC	Cytochrome b-c1 complex subunit 6, mitochondrial OS=Rattus norvegicus GN=Uqcrh PE=2 SV=1	GDPKEEEEEELVDPLTTVR
>	sp|Q5M9I5|QC	Cytochrome b-c1 complex subunit 6, mitochondrial OS=Rattus norvegicus GN=Uqcrh PE=2 SV=1	SQTEEDCTEELFDFLHAR
>	sp|Q5M9I5|QC	Cytochrome b-c1 complex subunit 6, mitochondrial OS=Rattus norvegicus GN=Uqcrh PE=2 SV=1	SQTEEDCTEELFDFLHAR
>	sp|Q5M9I5|QC	Cytochrome b-c1 complex subunit 6, mitochondrial OS=Rattus norvegicus GN=Uqcrh PE=2 SV=1	EHCEQLEK
>	sp|Q5M9I5|QC	Cytochrome b-c1 complex subunit 6, mitochondrial OS=Rattus norvegicus GN=Uqcrh PE=2 SV=1	EHCEQLEK
>	sp|P97536|CAN	Cullin-associated NEDD8-dissociated protein 1 OS=Rattus norvegicus GN=Cand1 PE=1 SV=1	ALTLIAGSPLKIDLRPVLGEGVPILASFLRK
>	sp|P97536|CAN	Cullin-associated NEDD8-dissociated protein 1 OS=Rattus norvegicus GN=Cand1 PE=1 SV=1	ITSEALLVTQQLVK
>	sp|P97536|CAN	Cullin-associated NEDD8-dissociated protein 1 OS=Rattus norvegicus GN=Cand1 PE=1 SV=1	QGGLLVNFHPSILTCLLPQLTSPR
>	sp|P97536|CAN	Cullin-associated NEDD8-dissociated protein 1 OS=Rattus norvegicus GN=Cand1 PE=1 SV=1	ITSEALLVTQQLVK
>	sp|P97536|CAN	Cullin-associated NEDD8-dissociated protein 1 OS=Rattus norvegicus GN=Cand1 PE=1 SV=1	QGGLLVNFHPSILTCLLPQLTSPR
>	sp|P97536|CAN	Cullin-associated NEDD8-dissociated protein 1 OS=Rattus norvegicus GN=Cand1 PE=1 SV=1	QGGLLVNFHPSILTCLLPQLTSPR
>	sp|P97536|CAN	Cullin-associated NEDD8-dissociated protein 1 OS=Rattus norvegicus GN=Cand1 PE=1 SV=1	QGGLLVNFHPSILTCLLPQLTSPR
>	sp|P97536|CAN	Cullin-associated NEDD8-dissociated protein 1 OS=Rattus norvegicus GN=Cand1 PE=1 SV=1	AVAALLTIPEAEK
>	sp|P97536|CAN	Cullin-associated NEDD8-dissociated protein 1 OS=Rattus norvegicus GN=Cand1 PE=1 SV=1	HTVDDGLDIR
>	sp|P97536|CAN	Cullin-associated NEDD8-dissociated protein 1 OS=Rattus norvegicus GN=Cand1 PE=1 SV=1	ALTLIAGSPLKIDLRPVLGEGVPILASFLRK
>	sp|P14408|FUN	Fumarate hydratase, mitochondrial OS=Rattus norvegicus GN=Fh PE=1 SV=1	KPVHPNDHVNK
>	sp|P14408|FUN	Fumarate hydratase, mitochondrial OS=Rattus norvegicus GN=Fh PE=1 SV=1	TAIELGYLTAEQFDEWVKPK
>	sp|P14408|FUN	Fumarate hydratase, mitochondrial OS=Rattus norvegicus GN=Fh PE=1 SV=1	THTQDAVPLTLGQEFSGYVQQVQYAMER
>	sp|P14408|FUN	Fumarate hydratase, mitochondrial OS=Rattus norvegicus GN=Fh PE=1 SV=1	LMNESLMLVTALNPHIGYDK
>	sp|P14408|FUN	Fumarate hydratase, mitochondrial OS=Rattus norvegicus GN=Fh PE=1 SV=1	KPVHPNDHVNK
>	sp|P14408|FUN	Fumarate hydratase, mitochondrial OS=Rattus norvegicus GN=Fh PE=1 SV=1	TAIELGYLTAEQFDEWVKPK
>	sp|P14408|FUN	Fumarate hydratase, mitochondrial OS=Rattus norvegicus GN=Fh PE=1 SV=1	TAIELGYLTAEQFDEWVKPK
>	sp|P14408|FUN	Fumarate hydratase, mitochondrial OS=Rattus norvegicus GN=Fh PE=1 SV=1	THTQDAVPLTLGQEFSGYVQQVQYAMER
>	sp|P14408|FUN	Fumarate hydratase, mitochondrial OS=Rattus norvegicus GN=Fh PE=1 SV=1	KTAIELGYLTAEQFDEWVKPK
>	sp|P14408|FUN	Fumarate hydratase, mitochondrial OS=Rattus norvegicus GN=Fh PE=1 SV=1	AIEMLGGELGSK
>	sp|Q5FVI4|CEN	Cell cycle exit and neuronal differentiation protein 1 OS=Rattus norvegicus GN=Cend1 PE=1 SV=1	ATPAADGK
>	sp|Q5FVI4|CEN	Cell cycle exit and neuronal differentiation protein 1 OS=Rattus norvegicus GN=Cend1 PE=1 SV=1	DAQAEKQEQPAAPGPATTK
>	sp|Q5FVI4|CEN	Cell cycle exit and neuronal differentiation protein 1 OS=Rattus norvegicus GN=Cend1 PE=1 SV=1	SASSPKPDTKVPQATAEAK
>	sp|Q5FVI4|CEN	Cell cycle exit and neuronal differentiation protein 1 OS=Rattus norvegicus GN=Cend1 PE=1 SV=1	ATPAADGK
>	sp|Q5FVI4|CEN	Cell cycle exit and neuronal differentiation protein 1 OS=Rattus norvegicus GN=Cend1 PE=1 SV=1	DAQAEKQEQPAAPGPATTK
>	sp|Q5FVI4|CEN	Cell cycle exit and neuronal differentiation protein 1 OS=Rattus norvegicus GN=Cend1 PE=1 SV=1	DAQAEKQEQPAAPGPATTK
>	sp|Q5FVI4|CEN	Cell cycle exit and neuronal differentiation protein 1 OS=Rattus norvegicus GN=Cend1 PE=1 SV=1	DAQAEKQEQPAAPGPATTK
>	sp|Q5FVI4|CEN	Cell cycle exit and neuronal differentiation protein 1 OS=Rattus norvegicus GN=Cend1 PE=1 SV=1	DAQAEKQEQPAAPGPATTK
>	sp|Q5FVI4|CEN	Cell cycle exit and neuronal differentiation protein 1 OS=Rattus norvegicus GN=Cend1 PE=1 SV=1	DAQAEKQEQPAAPGPATTK
>	sp|Q5FVI4|CEN	Cell cycle exit and neuronal differentiation protein 1 OS=Rattus norvegicus GN=Cend1 PE=1 SV=1	SASSPKPDTK
>	sp|Q5FVI4|CEN	Cell cycle exit and neuronal differentiation protein 1 OS=Rattus norvegicus GN=Cend1 PE=1 SV=1	DAQAEKQEQPAAPGPATTK
>	sp|O35077|GPD	Glycerol-3-phosphate dehydrogenase [NAD+], cytoplasmic OS=Rattus norvegicus GN=Gpd1 PE=1 SV=4	GLVDKFPLFTAVYK
>	sp|O35077|GPD	Glycerol-3-phosphate dehydrogenase [NAD+], cytoplasmic OS=Rattus norvegicus GN=Gpd1 PE=1 SV=4	LGLMEMIAFAK
>	sp|O35077|GPD	Glycerol-3-phosphate dehydrogenase [NAD+], cytoplasmic OS=Rattus norvegicus GN=Gpd1 PE=1 SV=4	LPPNVVAVPDVVQAATGADILVFVVPHQFIGK
>	sp|O35077|GPD	Glycerol-3-phosphate dehydrogenase [NAD+], cytoplasmic OS=Rattus norvegicus GN=Gpd1 PE=1 SV=4	LPPNVVAVPDVVQAATGADILVFVVPHQFIGK
>	sp|O35077|GPD	Glycerol-3-phosphate dehydrogenase [NAD+], cytoplasmic OS=Rattus norvegicus GN=Gpd1 PE=1 SV=4	ELHSILQHK
>	sp|P16036|MP	Phosphate carrier protein, mitochondrial OS=Rattus norvegicus GN=Slc25a3 PE=1 SV=1	GSTASQVLQR
>	sp|P16036|MP	Phosphate carrier protein, mitochondrial OS=Rattus norvegicus GN=Slc25a3 PE=1 SV=1	YYALCGFGGVLSCGLTHTAVVPLDLVK
>	sp|P16036|MP	Phosphate carrier protein, mitochondrial OS=Rattus norvegicus GN=Slc25a3 PE=1 SV=1	GIFNGFSITLK
>	sp|P16036|MP	Phosphate carrier protein, mitochondrial OS=Rattus norvegicus GN=Slc25a3 PE=1 SV=1	YYALCGFGGVLSCGLTHTAVVPLDLVK
>	sp|P16036|MP	Phosphate carrier protein, mitochondrial OS=Rattus norvegicus GN=Slc25a3 PE=1 SV=1	KLGLTE
>	sp|P16036|MP	Phosphate carrier protein, mitochondrial OS=Rattus norvegicus GN=Slc25a3 PE=1 SV=1	TVEALYK
>	sp|P16036|MP	Phosphate carrier protein, mitochondrial OS=Rattus norvegicus GN=Slc25a3 PE=1 SV=1	IQTQPGYANTLR
>	sp|P62982|RS2	Ubiquitin-40S ribosomal protein S27a OS=Rattus norvegicus GN=Rps27a PE=1 SV=2	IQDKEGIPPDQQR
>	sp|P62982|RS2	Ubiquitin-40S ribosomal protein S27a OS=Rattus norvegicus GN=Rps27a PE=1 SV=2	TITLEVEPSDTIENVK
>	sp|P62982|RS2	Ubiquitin-40S ribosomal protein S27a OS=Rattus norvegicus GN=Rps27a PE=1 SV=2	ESTLHLVLR
>	sp|P62982|RS2	Ubiquitin-40S ribosomal protein S27a OS=Rattus norvegicus GN=Rps27a PE=1 SV=2	TITLEVEPSDTIENVK
>	sp|P62982|RS2	Ubiquitin-40S ribosomal protein S27a OS=Rattus norvegicus GN=Rps27a PE=1 SV=2	TITLEVEPSDTIENVK
>	sp|P62982|RS2	Ubiquitin-40S ribosomal protein S27a OS=Rattus norvegicus GN=Rps27a PE=1 SV=2	TITLEVEPSDTIENVK
>	sp|P62982|RS2	Ubiquitin-40S ribosomal protein S27a OS=Rattus norvegicus GN=Rps27a PE=1 SV=2	ESTLHLVLR
>	sp|P62982|RS2	Ubiquitin-40S ribosomal protein S27a OS=Rattus norvegicus GN=Rps27a PE=1 SV=2	ESTLHLVLR
>	sp|P62982|RS2	Ubiquitin-40S ribosomal protein S27a OS=Rattus norvegicus GN=Rps27a PE=1 SV=2	IQDKEGIPPDQQR
>	sp|P62982|RS2	Ubiquitin-40S ribosomal protein S27a OS=Rattus norvegicus GN=Rps27a PE=1 SV=2	TITLEVEPSDTIENVK
>	sp|P62982|RS2	Ubiquitin-40S ribosomal protein S27a OS=Rattus norvegicus GN=Rps27a PE=1 SV=2	ESTLHLVLR
>	sp|P62982|RS2	Ubiquitin-40S ribosomal protein S27a OS=Rattus norvegicus GN=Rps27a PE=1 SV=2	TLSDYNIQK
>	sp|P12785|FAS	Fatty acid synthase OS=Rattus norvegicus GN=Fasn PE=1 SV=3	AEAVVAVLLTK
>	sp|P12785|FAS	Fatty acid synthase OS=Rattus norvegicus GN=Fasn PE=1 SV=3	EHDLVLPIR
>	sp|P12785|FAS	Fatty acid synthase OS=Rattus norvegicus GN=Fasn PE=1 SV=3	GLESIINIIHSSLAEPR
>	sp|P12785|FAS	Fatty acid synthase OS=Rattus norvegicus GN=Fasn PE=1 SV=3	HFQLEQDKPEEQTAHAFVNVLTR
>	sp|P12785|FAS	Fatty acid synthase OS=Rattus norvegicus GN=Fasn PE=1 SV=3	AEAVVAVLLTK
>	sp|P12785|FAS	Fatty acid synthase OS=Rattus norvegicus GN=Fasn PE=1 SV=3	GLESIINIIHSSLAEPR
>	sp|P12785|FAS	Fatty acid synthase OS=Rattus norvegicus GN=Fasn PE=1 SV=3	GLESIINIIHSSLAEPR
>	sp|P12785|FAS	Fatty acid synthase OS=Rattus norvegicus GN=Fasn PE=1 SV=3	GLESIINIIHSSLAEPR
>	sp|Q6URK4|RO	Heterogeneous nuclear ribonucleoprotein A3 OS=Rattus norvegicus GN=Hnrnpa3 PE=1 SV=1	KLFIGGLSFETTDDSLREHFEK
>	sp|Q6URK4|RO	Heterogeneous nuclear ribonucleoprotein A3 OS=Rattus norvegicus GN=Hnrnpa3 PE=1 SV=1	LFIGGLSFETTDDSLREHFEK
>	sp|Q6URK4|RO	Heterogeneous nuclear ribonucleoprotein A3 OS=Rattus norvegicus GN=Hnrnpa3 PE=1 SV=1	WGTLTDCVVMRDPQTK

>	sp|Q6URK4|RO	Heterogeneous nuclear ribonucleoprotein A3 OS=Rattus norvegicus GN=Hnrnpa3 PE=1 SV=1	KLFIGGLSFETTDDSLREHFEK
>	sp|Q6URK4|RO	Heterogeneous nuclear ribonucleoprotein A3 OS=Rattus norvegicus GN=Hnrnpa3 PE=1 SV=1	KLFIGGLSFETTDDSLREHFEK
>	sp|Q6URK4|RO	Heterogeneous nuclear ribonucleoprotein A3 OS=Rattus norvegicus GN=Hnrnpa3 PE=1 SV=1	KLFIGGLSFETTDDSLREHFEK
>	sp|Q9QYF3|MY	Myosin-Va OS=Rattus norvegicus GN=Myo5a PE=2 SV=1	KIGELEVGQMENISPGQIIDEPIRPVNIPR
>	sp|Q9QYF3|MY	Myosin-Va OS=Rattus norvegicus GN=Myo5a PE=2 SV=1	YDDLKEEMTLMLNVPKPGHK
>	sp|Q9QYF3|MY	Myosin-Va OS=Rattus norvegicus GN=Myo5a PE=2 SV=1	VLLLHLEEGKDLEYR
>	sp|Q9QYF3|MY	Myosin-Va OS=Rattus norvegicus GN=Myo5a PE=2 SV=1	KIGELEVGQMENISPGQIIDEPIRPVNIPR
>	sp|Q9QYF3|MY	Myosin-Va OS=Rattus norvegicus GN=Myo5a PE=2 SV=1	YDDLKEEMTLMLNVPKPGHK
>	sp|Q9QYF3|MY	Myosin-Va OS=Rattus norvegicus GN=Myo5a PE=2 SV=1	KVPLDMSLFLK
>	sp|Q66HD0|EN	Endoplasmin OS=Rattus norvegicus GN=Hsp90b1 PE=1 SV=2	RVKEDEDDKTVMDLAVVLFETATLR
>	sp|Q66HD0|EN	Endoplasmin OS=Rattus norvegicus GN=Hsp90b1 PE=1 SV=2	TETVEEPLEEDETAQEEKEEADDEAAVEEEEEEKKPK
>	sp|Q66HD0|EN	Endoplasmin OS=Rattus norvegicus GN=Hsp90b1 PE=1 SV=2	VKEDEDDKTVMDLAVVLFETATLR
>	sp|Q66HD0|EN	Endoplasmin OS=Rattus norvegicus GN=Hsp90b1 PE=1 SV=2	RVKEDEDDKTVMDLAVVLFETATLR
>	sp|Q66HD0|EN	Endoplasmin OS=Rattus norvegicus GN=Hsp90b1 PE=1 SV=2	TETVEEPLEEDETAQEEKEEADDEAAVEEEEEEKKPK
>	sp|Q66HD0|EN	Endoplasmin OS=Rattus norvegicus GN=Hsp90b1 PE=1 SV=2	TETVEEPLEEDETAQEEKEEADDEAAVEEEEEEKKPK
>	sp|Q66HD0|EN	Endoplasmin OS=Rattus norvegicus GN=Hsp90b1 PE=1 SV=2	TETVEEPLEEDETAQEEKEEADDEAAVEEEEEEKKPK
>	sp|Q66HD0|EN	Endoplasmin OS=Rattus norvegicus GN=Hsp90b1 PE=1 SV=2	TETVEEPLEEDETAQEEKEEADDEAAVEEEEEEKKPK
>	sp|Q66HD0|EN	Endoplasmin OS=Rattus norvegicus GN=Hsp90b1 PE=1 SV=2	TETVEEPLEEDETAQEEKEEADDEAAVEEEEEEKKPK
>	sp|P56571|ES1	ES1 protein homolog, mitochondrial OS=Rattus norvegicus PE=1 SV=2	GGAEVHIFAPDVPQMHVIDHTK
>	sp|P56571|ES1	ES1 protein homolog, mitochondrial OS=Rattus norvegicus PE=1 SV=2	GGAEVHIFAPDVPQMHVIDHTKGEPSEK
>	sp|P56571|ES1	ES1 protein homolog, mitochondrial OS=Rattus norvegicus PE=1 SV=2	GVEVTVGHEQEEGGKWPYAGTAEAVK
>	sp|P56571|ES1	ES1 protein homolog, mitochondrial OS=Rattus norvegicus PE=1 SV=2	GVEVTVGHEQEEGGKWPYAGTAEAVK
>	sp|P56571|ES1	ES1 protein homolog, mitochondrial OS=Rattus norvegicus PE=1 SV=2	GGAEVHIFAPDVPQMHVIDHTK
>	sp|P56571|ES1	ES1 protein homolog, mitochondrial OS=Rattus norvegicus PE=1 SV=2	GGAEVHIFAPDVPQMHVIDHTK
>	sp|P56571|ES1	ES1 protein homolog, mitochondrial OS=Rattus norvegicus PE=1 SV=2	GGAEVHIFAPDVPQMHVIDHTK
>	sp|P15865|H12	Histone H1.2 OS=Rattus norvegicus GN=Hist1h1c PE=1 SV=3	SGVSLAALK
>	sp|P15865|H12	Histone H1.2 OS=Rattus norvegicus GN=Hist1h1c PE=1 SV=3	AKKPAGAAK
>	sp|P15865|H12	Histone H1.2 OS=Rattus norvegicus GN=Hist1h1c PE=1 SV=3	KPAAAAGAK
>	sp|P15865|H12	Histone H1.2 OS=Rattus norvegicus GN=Hist1h1c PE=1 SV=3	SGVSLAALK
>	sp|P15865|H12	Histone H1.2 OS=Rattus norvegicus GN=Hist1h1c PE=1 SV=3	ALAAAGYDVEK
>	sp|P15865|H12	Histone H1.2 OS=Rattus norvegicus GN=Hist1h1c PE=1 SV=3	AKKPAGAAK
>	sp|P15865|H12	Histone H1.2 OS=Rattus norvegicus GN=Hist1h1c PE=1 SV=3	SGVSLAALKK
>	sp|P37805|TAG	Transgelin-3 OS=Rattus norvegicus GN=Tagln3 PE=1 SV=2	LVDWIILQCAEDIEHPPPGR
>	sp|P37805|TAG	Transgelin-3 OS=Rattus norvegicus GN=Tagln3 PE=1 SV=2	TTDIFQTVDLWEGK
>	sp|P37805|TAG	Transgelin-3 OS=Rattus norvegicus GN=Tagln3 PE=1 SV=2	LVDWIILQCAEDIEHPPPGR
>	sp|P37805|TAG	Transgelin-3 OS=Rattus norvegicus GN=Tagln3 PE=1 SV=2	LVDWIILQCAEDIEHPPPGR
>	sp|P37805|TAG	Transgelin-3 OS=Rattus norvegicus GN=Tagln3 PE=1 SV=2	LVDWIILQCAEDIEHPPPGR
>	sp|P37805|TAG	Transgelin-3 OS=Rattus norvegicus GN=Tagln3 PE=1 SV=2	LVDWIILQCAEDIEHPPPGR
>	sp|P37805|TAG	Transgelin-3 OS=Rattus norvegicus GN=Tagln3 PE=1 SV=2	TTDIFQTVDLWEGK
>	sp|P37805|TAG	Transgelin-3 OS=Rattus norvegicus GN=Tagln3 PE=1 SV=2	LVDWIILQCAEDIEHPPPGR
>	sp|P37805|TAG	Transgelin-3 OS=Rattus norvegicus GN=Tagln3 PE=1 SV=2	TTDIFQTVDLWEGK
>	sp|P37805|TAG	Transgelin-3 OS=Rattus norvegicus GN=Tagln3 PE=1 SV=2	EVQEK
>	sp|P37805|TAG	Transgelin-3 OS=Rattus norvegicus GN=Tagln3 PE=1 SV=2	WLMDGTVLCK
>	sp|P37805|TAG	Transgelin-3 OS=Rattus norvegicus GN=Tagln3 PE=1 SV=2	RGFSEEQLR
>	sp|P62944|AP2	AP-2 complex subunit beta OS=Rattus norvegicus GN=Ap2b1 PE=1 SV=1	AAMIWIVGEYAER
>	sp|P62944|AP2	AP-2 complex subunit beta OS=Rattus norvegicus GN=Ap2b1 PE=1 SV=1	KPSETQELVQQVLSLATQDSDNPDLRDR
>	sp|P62944|AP2	AP-2 complex subunit beta OS=Rattus norvegicus GN=Ap2b1 PE=1 SV=1	LASQANIAQVLAELK
>	sp|P62944|AP2	AP-2 complex subunit beta OS=Rattus norvegicus GN=Ap2b1 PE=1 SV=1	AAMIWIVGEYAER
>	sp|P62944|AP2	AP-2 complex subunit beta OS=Rattus norvegicus GN=Ap2b1 PE=1 SV=1	AAMIWIVGEYAER
>	sp|P62944|AP2	AP-2 complex subunit beta OS=Rattus norvegicus GN=Ap2b1 PE=1 SV=1	ECHLNADTVSSK
>	sp|P62944|AP2	AP-2 complex subunit beta OS=Rattus norvegicus GN=Ap2b1 PE=1 SV=1	KTAAVCVAK
>	sp|P62944|AP2	AP-2 complex subunit beta OS=Rattus norvegicus GN=Ap2b1 PE=1 SV=1	ECHLNADTVSSK
>	sp|P62944|AP2	AP-2 complex subunit beta OS=Rattus norvegicus GN=Ap2b1 PE=1 SV=1	LAELR
>	sp|P63045|VAM	Vesicle-associated membrane protein 2 OS=Rattus norvegicus GN=Vamp2 PE=1 SV=2	LQQTQAQVDEVVDIMR
>	sp|P63045|VAM	Vesicle-associated membrane protein 2 OS=Rattus norvegicus GN=Vamp2 PE=1 SV=2	RLQQTQAQVDEVVDIMR
>	sp|P63045|VAM	Vesicle-associated membrane protein 2 OS=Rattus norvegicus GN=Vamp2 PE=1 SV=2	LSELDDRADALQAGASQFETSAAK
>	sp|P63045|VAM	Vesicle-associated membrane protein 2 OS=Rattus norvegicus GN=Vamp2 PE=1 SV=2	LQQTQAQVDEVVDIMR
>	sp|P63045|VAM	Vesicle-associated membrane protein 2 OS=Rattus norvegicus GN=Vamp2 PE=1 SV=2	LSELDDRADALQAGASQFETSAAK
>	sp|P63045|VAM	Vesicle-associated membrane protein 2 OS=Rattus norvegicus GN=Vamp2 PE=1 SV=2	LSELDDRADALQAGASQFETSAAK
>	sp|P63045|VAM	Vesicle-associated membrane protein 2 OS=Rattus norvegicus GN=Vamp2 PE=1 SV=2	LSELDDRADALQAGASQFETSAAK
>	sp|P63045|VAM	Vesicle-associated membrane protein 2 OS=Rattus norvegicus GN=Vamp2 PE=1 SV=2	LSELDDRADALQAGASQFETSAAK
>	sp|P63045|VAM	Vesicle-associated membrane protein 2 OS=Rattus norvegicus GN=Vamp2 PE=1 SV=2	RLQQTQAQVDEVVDIMR
>	sp|P63045|VAM	Vesicle-associated membrane protein 2 OS=Rattus norvegicus GN=Vamp2 PE=1 SV=2	RLQQTQAQVDEVVDIMR
>	sp|P63045|VAM	Vesicle-associated membrane protein 2 OS=Rattus norvegicus GN=Vamp2 PE=1 SV=2	RLQQTQAQVDEVVDIMR
>	sp|P63045|VAM	Vesicle-associated membrane protein 2 OS=Rattus norvegicus GN=Vamp2 PE=1 SV=2	RLQQTQAQVDEVVDIMR
>	sp|P63045|VAM	Vesicle-associated membrane protein 2 OS=Rattus norvegicus GN=Vamp2 PE=1 SV=2	RLQQTQAQVDEVVDIMR
>	sp|P63045|VAM	Vesicle-associated membrane protein 2 OS=Rattus norvegicus GN=Vamp2 PE=1 SV=2	RLQQTQAQVDEVVDIMR
>	sp|P63045|VAM	Vesicle-associated membrane protein 2 OS=Rattus norvegicus GN=Vamp2 PE=1 SV=2	RLQQTQAQVDEVVDIMR
>	sp|P63045|VAM	Vesicle-associated membrane protein 2 OS=Rattus norvegicus GN=Vamp2 PE=1 SV=2	RLQQTQAQVDEVVDIMR
>	sp|P63045|VAM	Vesicle-associated membrane protein 2 OS=Rattus norvegicus GN=Vamp2 PE=1 SV=2	RLQQTQAQVDEVVDIMR
>	sp|P63045|VAM	Vesicle-associated membrane protein 2 OS=Rattus norvegicus GN=Vamp2 PE=1 SV=2	LSELDDRADALQAGASQFETSAAK
>	sp|P63045|VAM	Vesicle-associated membrane protein 2 OS=Rattus norvegicus GN=Vamp2 PE=1 SV=2	LQQTQAQVDEVVDIMR
>	sp|P63045|VAM	Vesicle-associated membrane protein 2 OS=Rattus norvegicus GN=Vamp2 PE=1 SV=2	RLQQTQAQVDEVVDIMR
>	sp|P63045|VAM	Vesicle-associated membrane protein 2 OS=Rattus norvegicus GN=Vamp2 PE=1 SV=2	ADALQAGASQFETSAAK

>	sp|P63045|VAM	Vesicle-associated membrane protein 2 OS=Rattus norvegicus GN=Vamp2 PE=1 SV=2	RLQQTQAQVDEVVDIMR
>	sp|P63045|VAM	Vesicle-associated membrane protein 2 OS=Rattus norvegicus GN=Vamp2 PE=1 SV=2	LSELDDRADALQAGASQFETSAAK
>	sp|P63045|VAM	Vesicle-associated membrane protein 2 OS=Rattus norvegicus GN=Vamp2 PE=1 SV=2	LSELDDRADALQAGASQFETSAAK
>	sp|P63045|VAM	Vesicle-associated membrane protein 2 OS=Rattus norvegicus GN=Vamp2 PE=1 SV=2	LSELDDRADALQAGASQFETSAAK
>	sp|P63045|VAM	Vesicle-associated membrane protein 2 OS=Rattus norvegicus GN=Vamp2 PE=1 SV=2	LSELDDRADALQAGASQFETSAAK
>	sp|P63045|VAM	Vesicle-associated membrane protein 2 OS=Rattus norvegicus GN=Vamp2 PE=1 SV=2	ADALQAGASQFETSAAK
>	sp|P50554|GAB	4-aminobutyrate aminotransferase, mitochondrial OS=Rattus norvegicus GN=Abat PE=1 SV=3	FWAHEHWGLDDPADVMSFSK
>	sp|P50554|GAB	4-aminobutyrate aminotransferase, mitochondrial OS=Rattus norvegicus GN=Abat PE=1 SV=3	IDIPSFDWPIAPFPR
>	sp|P50554|GAB	4-aminobutyrate aminotransferase, mitochondrial OS=Rattus norvegicus GN=Abat PE=1 SV=3	GFSKEELETCMVNQSPGCPDYSILSFMGAFHGR
>	sp|P50554|GAB	4-aminobutyrate aminotransferase, mitochondrial OS=Rattus norvegicus GN=Abat PE=1 SV=3	KHGCAFLVDEVQTGGGCTGK
>	sp|P50554|GAB	4-aminobutyrate aminotransferase, mitochondrial OS=Rattus norvegicus GN=Abat PE=1 SV=3	FWAHEHWGLDDPADVMSFSK
>	sp|P50554|GAB	4-aminobutyrate aminotransferase, mitochondrial OS=Rattus norvegicus GN=Abat PE=1 SV=3	GFSKEELETCMVNQSPGCPDYSILSFMGAFHGR
>	sp|P50554|GAB	4-aminobutyrate aminotransferase, mitochondrial OS=Rattus norvegicus GN=Abat PE=1 SV=3	TVAGIIVEPIQSEGGDNHASDDFFR
>	sp|P50554|GAB	4-aminobutyrate aminotransferase, mitochondrial OS=Rattus norvegicus GN=Abat PE=1 SV=3	LVQQPQNASTFINRPALGILPPENFVDKLRESLMSVAPK
>	sp|P50554|GAB	4-aminobutyrate aminotransferase, mitochondrial OS=Rattus norvegicus GN=Abat PE=1 SV=3	GFSKEELETCMVNQSPGCPDYSILSFMGAFHGR
>	sp|P50554|GAB	4-aminobutyrate aminotransferase, mitochondrial OS=Rattus norvegicus GN=Abat PE=1 SV=3	LVQQPQNASTFINRPALGILPPENFVDKLRESLMSVAPK
>	sp|P50554|GAB	4-aminobutyrate aminotransferase, mitochondrial OS=Rattus norvegicus GN=Abat PE=1 SV=3	AIHKIDIPSFDWPIAPFPR
>	sp|P50554|GAB	4-aminobutyrate aminotransferase, mitochondrial OS=Rattus norvegicus GN=Abat PE=1 SV=3	NKGVVLGGCGDK
>	sp|P50554|GAB	4-aminobutyrate aminotransferase, mitochondrial OS=Rattus norvegicus GN=Abat PE=1 SV=3	AIHKIDIPSFDWPIAPFPR
>	sp|P50554|GAB	4-aminobutyrate aminotransferase, mitochondrial OS=Rattus norvegicus GN=Abat PE=1 SV=3	IDIPSFDWPIAPFPR
>	sp|Q5XIN6|LET	LETM1 and EF-hand domain-containing protein 1, mitochondrial OS=Rattus norvegicus GN=Letm1 PE=1 SV=1	LEATLQEEAAIQQEHLEELKR
>	sp|Q5XIN6|LET	LETM1 and EF-hand domain-containing protein 1, mitochondrial OS=Rattus norvegicus GN=Letm1 PE=1 SV=1	VIDLVNKEDVQISTTQVAEIVATLEKEEKVEEKEK
>	sp|Q5XIN6|LET	LETM1 and EF-hand domain-containing protein 1, mitochondrial OS=Rattus norvegicus GN=Letm1 PE=1 SV=1	VIDLVNKEDVQISTTQVAEIVATLEKEEKVEEKEK
>	sp|Q5XIN6|LET	LETM1 and EF-hand domain-containing protein 1, mitochondrial OS=Rattus norvegicus GN=Letm1 PE=1 SV=1	VIDLVNKEDVQISTTQVAEIVATLEKEEKVEEKEK
>	sp|Q5XIN6|LET	LETM1 and EF-hand domain-containing protein 1, mitochondrial OS=Rattus norvegicus GN=Letm1 PE=1 SV=1	MVDVIPPSEILKDTAPVLEGLKGEEITKEEIDILSDACSK
>	sp|Q5XIN6|LET	LETM1 and EF-hand domain-containing protein 1, mitochondrial OS=Rattus norvegicus GN=Letm1 PE=1 SV=1	VIDLVNKEDVQISTTQVAEIVATLEKEEK
>	sp|Q5XIN6|LET	LETM1 and EF-hand domain-containing protein 1, mitochondrial OS=Rattus norvegicus GN=Letm1 PE=1 SV=1	ARGMRALGVTEDR
>	sp|Q5XIN6|LET	LETM1 and EF-hand domain-containing protein 1, mitochondrial OS=Rattus norvegicus GN=Letm1 PE=1 SV=1	EKEELELLKEDVQDYSEDLQEIKK
>	sp|Q5XIN6|LET	LETM1 and EF-hand domain-containing protein 1, mitochondrial OS=Rattus norvegicus GN=Letm1 PE=1 SV=1	EKEELELLKEDVQDYSEDLQEIKK
>	sp|Q5XIN6|LET	LETM1 and EF-hand domain-containing protein 1, mitochondrial OS=Rattus norvegicus GN=Letm1 PE=1 SV=1	LFEDELTLDNLTRPQLVALCK
>	sp|Q9Z0W5|PA	Protein kinase C and casein kinase substrate in neurons protein 1 OS=Rattus norvegicus GN=Pacsin1 PE=1 SV=1	KAEGAALSNATGAVESTSQAGDR
>	sp|Q9Z0W5|PA	Protein kinase C and casein kinase substrate in neurons protein 1 OS=Rattus norvegicus GN=Pacsin1 PE=1 SV=1	TEQSVTPEQQK
>	sp|Q9Z0W5|PA	Protein kinase C and casein kinase substrate in neurons protein 1 OS=Rattus norvegicus GN=Pacsin1 PE=1 SV=1	AWGAMMTEADKVSELHQEVK
>	sp|Q9Z0W5|PA	Protein kinase C and casein kinase substrate in neurons protein 1 OS=Rattus norvegicus GN=Pacsin1 PE=1 SV=1	KAEGAALSNATGAVESTSQAGDR
>	sp|Q9Z0W5|PA	Protein kinase C and casein kinase substrate in neurons protein 1 OS=Rattus norvegicus GN=Pacsin1 PE=1 SV=1	KAEGAALSNATGAVESTSQAGDR
>	sp|Q9Z0W5|PA	Protein kinase C and casein kinase substrate in neurons protein 1 OS=Rattus norvegicus GN=Pacsin1 PE=1 SV=1	KAEGAALSNATGAVESTSQAGDR
>	sp|Q9Z0W5|PA	Protein kinase C and casein kinase substrate in neurons protein 1 OS=Rattus norvegicus GN=Pacsin1 PE=1 SV=1	KAEGAALSNATGAVESTSQAGDR
>	sp|Q9Z0W5|PA	Protein kinase C and casein kinase substrate in neurons protein 1 OS=Rattus norvegicus GN=Pacsin1 PE=1 SV=1	KAEGAALSNATGAVESTSQAGDR
>	sp|Q9Z0W5|PA	Protein kinase C and casein kinase substrate in neurons protein 1 OS=Rattus norvegicus GN=Pacsin1 PE=1 SV=1	KAEGAALSNATGAVESTSQAGDR
>	sp|Q9Z0W5|PA	Protein kinase C and casein kinase substrate in neurons protein 1 OS=Rattus norvegicus GN=Pacsin1 PE=1 SV=1	AYAQQLTDWAK
>	sp|Q9Z0W5|PA	Protein kinase C and casein kinase substrate in neurons protein 1 OS=Rattus norvegicus GN=Pacsin1 PE=1 SV=1	TEQSVTPEQQK
>	sp|Q9Z0W5|PA	Protein kinase C and casein kinase substrate in neurons protein 1 OS=Rattus norvegicus GN=Pacsin1 PE=1 SV=1	KAEGAALSNATGAVESTSQAGDR
>	sp|B2GV06|SCO	Succinyl-CoA:3-ketoacid-coenzyme A transferase 1, mitochondrial OS=Rattus norvegicus GN=Oxct1 PE=1 SV=1	AAGTTVVEVEEIVDIGSFAPEDIHIPK
>	sp|B2GV06|SCO	Succinyl-CoA:3-ketoacid-coenzyme A transferase 1, mitochondrial OS=Rattus norvegicus GN=Oxct1 PE=1 SV=1	GQHFILEEAITGDFALVK
>	sp|B2GV06|SCO	Succinyl-CoA:3-ketoacid-coenzyme A transferase 1, mitochondrial OS=Rattus norvegicus GN=Oxct1 PE=1 SV=1	NGLTLIELWEGLTVDDIRK
>	sp|B2GV06|SCO	Succinyl-CoA:3-ketoacid-coenzyme A transferase 1, mitochondrial OS=Rattus norvegicus GN=Oxct1 PE=1 SV=1	GQHFILEEAITGDFALVK
>	sp|B2GV06|SCO	Succinyl-CoA:3-ketoacid-coenzyme A transferase 1, mitochondrial OS=Rattus norvegicus GN=Oxct1 PE=1 SV=1	GQHFILEEAITGDFALVK
>	sp|B2GV06|SCO	Succinyl-CoA:3-ketoacid-coenzyme A transferase 1, mitochondrial OS=Rattus norvegicus GN=Oxct1 PE=1 SV=1	GQHFILEEAITGDFALVK
>	sp|B2GV06|SCO	Succinyl-CoA:3-ketoacid-coenzyme A transferase 1, mitochondrial OS=Rattus norvegicus GN=Oxct1 PE=1 SV=1	VVVTMEHSAK
>	sp|P63041|CPL	Complexin-1 OS=Rattus norvegicus GN=Cplx1 PE=1 SV=1	EAEAQAAMEANSEGSLTRPK
>	sp|P63041|CPL	Complexin-1 OS=Rattus norvegicus GN=Cplx1 PE=1 SV=1	MLGGDEEKDPDAAKKEEERQEALR
>	sp|P63041|CPL	Complexin-1 OS=Rattus norvegicus GN=Cplx1 PE=1 SV=1	YLPGPLQDMFK
>	sp|P63041|CPL	Complexin-1 OS=Rattus norvegicus GN=Cplx1 PE=1 SV=1	EAEAQAAMEANSEGSLTRPK
>	sp|P63041|CPL	Complexin-1 OS=Rattus norvegicus GN=Cplx1 PE=1 SV=1	EAEAQAAMEANSEGSLTRPK
>	sp|P63041|CPL	Complexin-1 OS=Rattus norvegicus GN=Cplx1 PE=1 SV=1	EAEAQAAMEANSEGSLTRPK
>	sp|P63041|CPL	Complexin-1 OS=Rattus norvegicus GN=Cplx1 PE=1 SV=1	EAEAQAAMEANSEGSLTRPK
>	sp|P63041|CPL	Complexin-1 OS=Rattus norvegicus GN=Cplx1 PE=1 SV=1	MLGGDEEKDPDAAKKEEERQEALR
>	sp|P63041|CPL	Complexin-1 OS=Rattus norvegicus GN=Cplx1 PE=1 SV=1	YLPGPLQDMFK
>	sp|P63041|CPL	Complexin-1 OS=Rattus norvegicus GN=Cplx1 PE=1 SV=1	YLPGPLQDMFK
>	sp|P63041|CPL	Complexin-1 OS=Rattus norvegicus GN=Cplx1 PE=1 SV=1	YLPGPLQDMFK
>	sp|P63041|CPL	Complexin-1 OS=Rattus norvegicus GN=Cplx1 PE=1 SV=1	YLPGPLQDMFK
>	sp|P63041|CPL	Complexin-1 OS=Rattus norvegicus GN=Cplx1 PE=1 SV=1	YLPGPLQDMFK
>	sp|P63041|CPL	Complexin-1 OS=Rattus norvegicus GN=Cplx1 PE=1 SV=1	YLPGPLQDMFK
>	sp|P63041|CPL	Complexin-1 OS=Rattus norvegicus GN=Cplx1 PE=1 SV=1	YLPGPLQDMFK
>	sp|P63041|CPL	Complexin-1 OS=Rattus norvegicus GN=Cplx1 PE=1 SV=1	MLGGDEEKDPDAAK
>	sp|P63041|CPL	Complexin-1 OS=Rattus norvegicus GN=Cplx1 PE=1 SV=1	EAEAQAAMEANSEGSLTRPK
>	sp|P63041|CPL	Complexin-1 OS=Rattus norvegicus GN=Cplx1 PE=1 SV=1	YLPGPLQDMFK
>	sp|P63041|CPL	Complexin-1 OS=Rattus norvegicus GN=Cplx1 PE=1 SV=1	YLPGPLQDMFK
>	sp|Q05546|TEN	Tenascin-R OS=Rattus norvegicus GN=Tnr PE=1 SV=1	GHEFSIPFVEMK
>	sp|Q05546|TEN	Tenascin-R OS=Rattus norvegicus GN=Tnr PE=1 SV=1	LILNYSPRDEEEEMMEVLLDATKR
>	sp|Q05546|TEN	Tenascin-R OS=Rattus norvegicus GN=Tnr PE=1 SV=1	LILNYSPRDEEEEMMEVLLDATKR
>	sp|Q05546|TEN	Tenascin-R OS=Rattus norvegicus GN=Tnr PE=1 SV=1	LILNYSPRDEEEEMMEVLLDATKR
>	sp|Q05546|TEN	Tenascin-R OS=Rattus norvegicus GN=Tnr PE=1 SV=1	GHEFSIPFVEMK
>	sp|Q05546|TEN	Tenascin-R OS=Rattus norvegicus GN=Tnr PE=1 SV=1	VDFILLK

>	sp|Q05546|TEN	Tenascin-R OS=Rattus norvegicus GN=Tnr PE=1 SV=1	LDSSVVPNTVTEFTITR
>	sp|Q9QXQ0|AC	Alpha-actinin-4 OS=Rattus norvegicus GN=Actn4 PE=1 SV=2	LMLLLEVISGERLPKPER
>	sp|Q9QXQ0|AC	Alpha-actinin-4 OS=Rattus norvegicus GN=Actn4 PE=1 SV=2	VLAGDKNFITAEELR
>	sp|Q9QXQ0|AC	Alpha-actinin-4 OS=Rattus norvegicus GN=Actn4 PE=1 SV=2	SIVDYKPNLDLLEQQHQLIQEALIFDNK
>	sp|Q9QXQ0|AC	Alpha-actinin-4 OS=Rattus norvegicus GN=Actn4 PE=1 SV=2	ICDQWDNLGSLTHSR
>	sp|Q9QXQ0|AC	Alpha-actinin-4 OS=Rattus norvegicus GN=Actn4 PE=1 SV=2	VGWEQLLTTIAR
>	sp|Q9QXQ0|AC	Alpha-actinin-4 OS=Rattus norvegicus GN=Actn4 PE=1 SV=2	VGWEQLLTTIAR
>	sp|Q9QXQ0|AC	Alpha-actinin-4 OS=Rattus norvegicus GN=Actn4 PE=1 SV=2	VLAGDKNFITAEELR
>	sp|Q9QXQ0|AC	Alpha-actinin-4 OS=Rattus norvegicus GN=Actn4 PE=1 SV=2	ASIHEAWTDGK
>	sp|Q9QXQ0|AC	Alpha-actinin-4 OS=Rattus norvegicus GN=Actn4 PE=1 SV=2	DGLAFNALIHR
>	sp|Q9QXQ0|AC	Alpha-actinin-4 OS=Rattus norvegicus GN=Actn4 PE=1 SV=2	VLAGDKNFITAEELR
>	sp|Q9QXQ0|AC	Alpha-actinin-4 OS=Rattus norvegicus GN=Actn4 PE=1 SV=2	RDHALLEEQSK
>	sp|Q9QXQ0|AC	Alpha-actinin-4 OS=Rattus norvegicus GN=Actn4 PE=1 SV=2	LDHLAEK
>	sp|P24942|EAA	Excitatory amino acid transporter 1 OS=Rattus norvegicus GN=Slc1a3 PE=1 SV=2	IVQVTAADAFLDLIR
>	sp|P24942|EAA	Excitatory amino acid transporter 1 OS=Rattus norvegicus GN=Slc1a3 PE=1 SV=2	KPYQLIAQDNEPEKPVADSETK
>	sp|P24942|EAA	Excitatory amino acid transporter 1 OS=Rattus norvegicus GN=Slc1a3 PE=1 SV=2	TKSNGEEPR
>	sp|P24942|EAA	Excitatory amino acid transporter 1 OS=Rattus norvegicus GN=Slc1a3 PE=1 SV=2	TTTNVLGDSLGAGIVEHLSR
>	sp|P24942|EAA	Excitatory amino acid transporter 1 OS=Rattus norvegicus GN=Slc1a3 PE=1 SV=2	IVQVTAADAFLDLIR
>	sp|P24942|EAA	Excitatory amino acid transporter 1 OS=Rattus norvegicus GN=Slc1a3 PE=1 SV=2	IVQVTAADAFLDLIR
>	sp|P24942|EAA	Excitatory amino acid transporter 1 OS=Rattus norvegicus GN=Slc1a3 PE=1 SV=2	TTTNVLGDSLGAGIVEHLSR
>	sp|P24942|EAA	Excitatory amino acid transporter 1 OS=Rattus norvegicus GN=Slc1a3 PE=1 SV=2	KPYQLIAQDNEPEKPVADSETK
>	sp|P24942|EAA	Excitatory amino acid transporter 1 OS=Rattus norvegicus GN=Slc1a3 PE=1 SV=2	KPYQLIAQDNEPEKPVADSETK
>	sp|P19804|NDI	Nucleoside diphosphate kinase B OS=Rattus norvegicus GN=Nme2 PE=1 SV=1	EIGLWFKPEELIDYK
>	sp|P19804|NDI	Nucleoside diphosphate kinase B OS=Rattus norvegicus GN=Nme2 PE=1 SV=1	TFIAIKPDGVQR
>	sp|P19804|NDI	Nucleoside diphosphate kinase B OS=Rattus norvegicus GN=Nme2 PE=1 SV=1	EIGLWFKPEELIDYK
>	sp|P19804|NDI	Nucleoside diphosphate kinase B OS=Rattus norvegicus GN=Nme2 PE=1 SV=1	TFIAIKPDGVQR
>	sp|P19804|NDI	Nucleoside diphosphate kinase B OS=Rattus norvegicus GN=Nme2 PE=1 SV=1	VMLGETNPADSKPGTIR
>	sp|P19804|NDI	Nucleoside diphosphate kinase B OS=Rattus norvegicus GN=Nme2 PE=1 SV=1	EIGLWFKPEELIDYK
>	sp|P19804|NDI	Nucleoside diphosphate kinase B OS=Rattus norvegicus GN=Nme2 PE=1 SV=1	TFIAIKPDGVQR
>	sp|P19804|NDI	Nucleoside diphosphate kinase B OS=Rattus norvegicus GN=Nme2 PE=1 SV=1	TFIAIKPDGVQR
>	sp|P19804|NDI	Nucleoside diphosphate kinase B OS=Rattus norvegicus GN=Nme2 PE=1 SV=1	VMLGETNPADSKPGTIR
>	sp|P19804|NDI	Nucleoside diphosphate kinase B OS=Rattus norvegicus GN=Nme2 PE=1 SV=1	EIGLWFKPEELIDYK
>	sp|P19804|NDI	Nucleoside diphosphate kinase B OS=Rattus norvegicus GN=Nme2 PE=1 SV=1	VMLGETNPADSKPGTIR
>	sp|P35213|143	14-3-3 protein beta/alpha OS=Rattus norvegicus GN=Ywhab PE=1 SV=3	AVTEQGHELSNEER
>	sp|P35213|143	14-3-3 protein beta/alpha OS=Rattus norvegicus GN=Ywhab PE=1 SV=3	EKIEAELQDICSDVLELLDK
>	sp|P35213|143	14-3-3 protein beta/alpha OS=Rattus norvegicus GN=Ywhab PE=1 SV=3	EKIEAELQDICSDVLELLDKYLILNATHAESK
>	sp|P35213|143	14-3-3 protein beta/alpha OS=Rattus norvegicus GN=Ywhab PE=1 SV=3	AVTEQGHELSNEER
>	sp|P35213|143	14-3-3 protein beta/alpha OS=Rattus norvegicus GN=Ywhab PE=1 SV=3	AVTEQGHELSNEER
>	sp|P35213|143	14-3-3 protein beta/alpha OS=Rattus norvegicus GN=Ywhab PE=1 SV=3	AVTEQGHELSNEER
>	sp|P35213|143	14-3-3 protein beta/alpha OS=Rattus norvegicus GN=Ywhab PE=1 SV=3	AVTEQGHELSNEER
>	sp|P35213|143	14-3-3 protein beta/alpha OS=Rattus norvegicus GN=Ywhab PE=1 SV=3	DSTLIMQLLR
>	sp|P35213|143	14-3-3 protein beta/alpha OS=Rattus norvegicus GN=Ywhab PE=1 SV=3	NLLSVAYK
>	sp|P35213|143	14-3-3 protein beta/alpha OS=Rattus norvegicus GN=Ywhab PE=1 SV=3	NLLSVAYK
>	sp|P35213|143	14-3-3 protein beta/alpha OS=Rattus norvegicus GN=Ywhab PE=1 SV=3	NLLSVAYK
>	sp|P35213|143	14-3-3 protein beta/alpha OS=Rattus norvegicus GN=Ywhab PE=1 SV=3	NLLSVAYK
>	sp|P35213|143	14-3-3 protein beta/alpha OS=Rattus norvegicus GN=Ywhab PE=1 SV=3	NLLSVAYK
>	sp|P35213|143	14-3-3 protein beta/alpha OS=Rattus norvegicus GN=Ywhab PE=1 SV=3	YDDMAAAMK
>	sp|P35213|143	14-3-3 protein beta/alpha OS=Rattus norvegicus GN=Ywhab PE=1 SV=3	VISSIEQK
>	sp|P35213|143	14-3-3 protein beta/alpha OS=Rattus norvegicus GN=Ywhab PE=1 SV=3	NLLSVAYK
>	sp|P35213|143	14-3-3 protein beta/alpha OS=Rattus norvegicus GN=Ywhab PE=1 SV=3	EMQPTHPIR
>	sp|P35213|143	14-3-3 protein beta/alpha OS=Rattus norvegicus GN=Ywhab PE=1 SV=3	AVTEQGHELSNEER
>	sp|P35213|143	14-3-3 protein beta/alpha OS=Rattus norvegicus GN=Ywhab PE=1 SV=3	VISSIEQK
>	sp|P35213|143	14-3-3 protein beta/alpha OS=Rattus norvegicus GN=Ywhab PE=1 SV=3	EKIEAELQDICSDVLELLDKYLILNATHAESK
>	sp|P35213|143	14-3-3 protein beta/alpha OS=Rattus norvegicus GN=Ywhab PE=1 SV=3	NLLSVAYK
>	sp|P35213|143	14-3-3 protein beta/alpha OS=Rattus norvegicus GN=Ywhab PE=1 SV=3	AVTEQGHELSNEER
>	sp|P35213|143	14-3-3 protein beta/alpha OS=Rattus norvegicus GN=Ywhab PE=1 SV=3	NLLSVAYK
>	sp|P35213|143	14-3-3 protein beta/alpha OS=Rattus norvegicus GN=Ywhab PE=1 SV=3	AVTEQGHELSNEER
>	sp|P35213|143	14-3-3 protein beta/alpha OS=Rattus norvegicus GN=Ywhab PE=1 SV=3	NLLSVAYK
>	sp|P35213|143	14-3-3 protein beta/alpha OS=Rattus norvegicus GN=Ywhab PE=1 SV=3	YDDMAAAMK
>	sp|P35213|143	14-3-3 protein beta/alpha OS=Rattus norvegicus GN=Ywhab PE=1 SV=3	TAFDEAIAELDTLNEESYKDSTLIMQLLR
>	sp|P35213|143	14-3-3 protein beta/alpha OS=Rattus norvegicus GN=Ywhab PE=1 SV=3	VISSIEQK
>	sp|P35213|143	14-3-3 protein beta/alpha OS=Rattus norvegicus GN=Ywhab PE=1 SV=3	YDDMAAAMK
>	sp|O08839|BIN	Myc box-dependent-interacting protein 1 OS=Rattus norvegicus GN=Bin1 PE=1 SV=1	LDLPPGFMFK
>	sp|O08839|BIN	Myc box-dependent-interacting protein 1 OS=Rattus norvegicus GN=Bin1 PE=1 SV=1	LQAHLVAQTNLLR
>	sp|O08839|BIN	Myc box-dependent-interacting protein 1 OS=Rattus norvegicus GN=Bin1 PE=1 SV=1	LDLPPGFMFK
>	sp|O08839|BIN	Myc box-dependent-interacting protein 1 OS=Rattus norvegicus GN=Bin1 PE=1 SV=1	LQAHLVAQTNLLR
>	sp|O08839|BIN	Myc box-dependent-interacting protein 1 OS=Rattus norvegicus GN=Bin1 PE=1 SV=1	LQAHLVAQTNLLR
>	sp|O08839|BIN	Myc box-dependent-interacting protein 1 OS=Rattus norvegicus GN=Bin1 PE=1 SV=1	LQAHLVAQTNLLR
>	sp|O08839|BIN	Myc box-dependent-interacting protein 1 OS=Rattus norvegicus GN=Bin1 PE=1 SV=1	IAKPVSLLEK
>	sp|O08839|BIN	Myc box-dependent-interacting protein 1 OS=Rattus norvegicus GN=Bin1 PE=1 SV=1	LQAHLVAQTNLLR
>	sp|O08839|BIN	Myc box-dependent-interacting protein 1 OS=Rattus norvegicus GN=Bin1 PE=1 SV=1	KLSECLQEVYEPEWPGRDEANK
>	sp|Q6AYH5|DC	Dynactin subunit 2 OS=Rattus norvegicus GN=Dctn2 PE=1 SV=1	LLHEVQELTTEVEK
>	sp|Q6AYH5|DC	Dynactin subunit 2 OS=Rattus norvegicus GN=Dctn2 PE=1 SV=1	VNALDLAVLDQVEAR
>	sp|Q6AYH5|DC	Dynactin subunit 2 OS=Rattus norvegicus GN=Dctn2 PE=1 SV=1	LLHEVQELTTEVEK

>	sp|Q6AYH5|DC Dynactin subunit 2 OS=Rattus norvegicus GN=Dctn2 PE=1 SV=1	LLHEVQELTTEVEK
>	sp|Q6AYH5|DC Dynactin subunit 2 OS=Rattus norvegicus GN=Dctn2 PE=1 SV=1	LLHEVQELTTEVEK
>	sp|Q6AYH5|DC Dynactin subunit 2 OS=Rattus norvegicus GN=Dctn2 PE=1 SV=1	LLLQLEATK
>	sp|Q6AYH5|DC Dynactin subunit 2 OS=Rattus norvegicus GN=Dctn2 PE=1 SV=1	LLLQLEATK
>	sp|P62898|CYC Cytochrome c, somatic OS=Rattus norvegicus GN=Cycs PE=1 SV=2	KTGQAAGFSYTDANK
>	sp|P62898|CYC Cytochrome c, somatic OS=Rattus norvegicus GN=Cycs PE=1 SV=2	TGPNLHGLFGR
>	sp|P62898|CYC Cytochrome c, somatic OS=Rattus norvegicus GN=Cycs PE=1 SV=2	ADLIAYLK
>	sp|P62898|CYC Cytochrome c, somatic OS=Rattus norvegicus GN=Cycs PE=1 SV=2	GITWGEDTLMEYLENPKK
>	sp|P62898|CYC Cytochrome c, somatic OS=Rattus norvegicus GN=Cycs PE=1 SV=2	ADLIAYLK
>	sp|P62898|CYC Cytochrome c, somatic OS=Rattus norvegicus GN=Cycs PE=1 SV=2	ADLIAYLK
>	sp|P62898|CYC Cytochrome c, somatic OS=Rattus norvegicus GN=Cycs PE=1 SV=2	ADLIAYLK
>	sp|P62898|CYC Cytochrome c, somatic OS=Rattus norvegicus GN=Cycs PE=1 SV=2	ADLIAYLK
>	sp|P62898|CYC Cytochrome c, somatic OS=Rattus norvegicus GN=Cycs PE=1 SV=2	GITWGEDTLMEYLENPKK
>	sp|P62898|CYC Cytochrome c, somatic OS=Rattus norvegicus GN=Cycs PE=1 SV=2	GITWGEDTLMEYLENPKK
>	sp|P62898|CYC Cytochrome c, somatic OS=Rattus norvegicus GN=Cycs PE=1 SV=2	TGPNLHGLFGR
>	sp|P62898|CYC Cytochrome c, somatic OS=Rattus norvegicus GN=Cycs PE=1 SV=2	TGPNLHGLFGR
>	sp|P62898|CYC Cytochrome c, somatic OS=Rattus norvegicus GN=Cycs PE=1 SV=2	GITWGEDTLMEYLENPKK
>	sp|P62898|CYC Cytochrome c, somatic OS=Rattus norvegicus GN=Cycs PE=1 SV=2	TGPNLHGLFGR
>	sp|P62898|CYC Cytochrome c, somatic OS=Rattus norvegicus GN=Cycs PE=1 SV=2	GITWGEDTLMEYLENPK
>	sp|P62898|CYC Cytochrome c, somatic OS=Rattus norvegicus GN=Cycs PE=1 SV=2	GITWGEDTLMEYLENPKK
>	sp|P62898|CYC Cytochrome c, somatic OS=Rattus norvegicus GN=Cycs PE=1 SV=2	GITWGEDTLMEYLENPKK
>	sp|P62898|CYC Cytochrome c, somatic OS=Rattus norvegicus GN=Cycs PE=1 SV=2	ADLIAYLK
>	sp|P62898|CYC Cytochrome c, somatic OS=Rattus norvegicus GN=Cycs PE=1 SV=2	TGPNLHGLFGR
>	sp|P62898|CYC Cytochrome c, somatic OS=Rattus norvegicus GN=Cycs PE=1 SV=2	ADLIAYLK
>	sp|P62898|CYC Cytochrome c, somatic OS=Rattus norvegicus GN=Cycs PE=1 SV=2	TGPNLHGLFGR
>	sp|P62898|CYC Cytochrome c, somatic OS=Rattus norvegicus GN=Cycs PE=1 SV=2	GITWGEDTLMEYLENPKK
>	sp|P62898|CYC Cytochrome c, somatic OS=Rattus norvegicus GN=Cycs PE=1 SV=2	MIFAGIK
>	sp|P62898|CYC Cytochrome c, somatic OS=Rattus norvegicus GN=Cycs PE=1 SV=2	GITWGEDTLMEYLENPKK
>	sp|P62898|CYC Cytochrome c, somatic OS=Rattus norvegicus GN=Cycs PE=1 SV=2	MIFAGIK
>	sp|P62898|CYC Cytochrome c, somatic OS=Rattus norvegicus GN=Cycs PE=1 SV=2	GITWGEDTLMEYLENPKK
>	sp|Q09073|AD ADP/ATP translocase 2 OS=Rattus norvegicus GN=Slc25a5 PE=1 SV=3	DFLAGGVAAAISK
>	sp|Q09073|AD ADP/ATP translocase 2 OS=Rattus norvegicus GN=Slc25a5 PE=1 SV=3	KGTDIMYTGTLDCWR
>	sp|Q09073|AD ADP/ATP translocase 2 OS=Rattus norvegicus GN=Slc25a5 PE=1 SV=3	DFLAGGVAAAISK
>	sp|Q09073|AD ADP/ATP translocase 2 OS=Rattus norvegicus GN=Slc25a5 PE=1 SV=3	DFLAGGVAAAISK
>	sp|Q09073|AD ADP/ATP translocase 2 OS=Rattus norvegicus GN=Slc25a5 PE=1 SV=3	DFLAGGVAAAISK
>	sp|Q09073|AD ADP/ATP translocase 2 OS=Rattus norvegicus GN=Slc25a5 PE=1 SV=3	GAWSNVLR
>	sp|Q09073|AD ADP/ATP translocase 2 OS=Rattus norvegicus GN=Slc25a5 PE=1 SV=3	GMGGAFVLVLYDEIKK
>	sp|Q09073|AD ADP/ATP translocase 2 OS=Rattus norvegicus GN=Slc25a5 PE=1 SV=3	GMGGAFVLVLYDEIKK
>	sp|Q09073|AD ADP/ATP translocase 2 OS=Rattus norvegicus GN=Slc25a5 PE=1 SV=3	GMGGAFVLVLYDEIKK
>	sp|Q09073|AD ADP/ATP translocase 2 OS=Rattus norvegicus GN=Slc25a5 PE=1 SV=3	GMGGAFVLVLYDEIKK
>	sp|Q09073|AD ADP/ATP translocase 2 OS=Rattus norvegicus GN=Slc25a5 PE=1 SV=3	GMGGAFVLVLYDEIKK
>	sp|Q09073|AD ADP/ATP translocase 2 OS=Rattus norvegicus GN=Slc25a5 PE=1 SV=3	GMGGAFVLVLYDEIKK
>	sp|Q09073|AD ADP/ATP translocase 2 OS=Rattus norvegicus GN=Slc25a5 PE=1 SV=3	GMGGAFVLVLYDEIKK
>	sp|Q09073|AD ADP/ATP translocase 2 OS=Rattus norvegicus GN=Slc25a5 PE=1 SV=3	KGTDIMYTGTLDCWR
>	sp|Q09073|AD ADP/ATP translocase 2 OS=Rattus norvegicus GN=Slc25a5 PE=1 SV=3	KGTDIMYTGTLDCWR
>	sp|Q09073|AD ADP/ATP translocase 2 OS=Rattus norvegicus GN=Slc25a5 PE=1 SV=3	KGTDIMYTGTLDCWR
>	sp|Q09073|AD ADP/ATP translocase 2 OS=Rattus norvegicus GN=Slc25a5 PE=1 SV=3	LLLQVQHASK
>	sp|Q09073|AD ADP/ATP translocase 2 OS=Rattus norvegicus GN=Slc25a5 PE=1 SV=3	TAVAPIER
>	sp|Q09073|AD ADP/ATP translocase 2 OS=Rattus norvegicus GN=Slc25a5 PE=1 SV=3	TAVAPIER
>	sp|Q09073|AD ADP/ATP translocase 2 OS=Rattus norvegicus GN=Slc25a5 PE=1 SV=3	TAVAPIER
>	sp|Q09073|AD ADP/ATP translocase 2 OS=Rattus norvegicus GN=Slc25a5 PE=1 SV=3	YFAGNLASGGAAGATSLCFVYPLDFAR
>	sp|Q09073|AD ADP/ATP translocase 2 OS=Rattus norvegicus GN=Slc25a5 PE=1 SV=3	YFAGNLASGGAAGATSLCFVYPLDFAR
>	sp|Q09073|AD ADP/ATP translocase 2 OS=Rattus norvegicus GN=Slc25a5 PE=1 SV=3	YFAGNLASGGAAGATSLCFVYPLDFAR
>	sp|Q09073|AD ADP/ATP translocase 2 OS=Rattus norvegicus GN=Slc25a5 PE=1 SV=3	YFAGNLASGGAAGATSLCFVYPLDFAR
>	sp|Q09073|AD ADP/ATP translocase 2 OS=Rattus norvegicus GN=Slc25a5 PE=1 SV=3	YFAGNLASGGAAGATSLCFVYPLDFAR
>	sp|Q09073|AD ADP/ATP translocase 2 OS=Rattus norvegicus GN=Slc25a5 PE=1 SV=3	YFAGNLASGGAAGATSLCFVYPLDFAR
>	sp|Q09073|AD ADP/ATP translocase 2 OS=Rattus norvegicus GN=Slc25a5 PE=1 SV=3	YFAGNLASGGAAGATSLCFVYPLDFAR
>	sp|Q09073|AD ADP/ATP translocase 2 OS=Rattus norvegicus GN=Slc25a5 PE=1 SV=3	YFAGNLASGGAAGATSLCFVYPLDFAR
>	sp|Q09073|AD ADP/ATP translocase 2 OS=Rattus norvegicus GN=Slc25a5 PE=1 SV=3	YFAGNLASGGAAGATSLCFVYPLDFAR
>	sp|Q09073|AD ADP/ATP translocase 2 OS=Rattus norvegicus GN=Slc25a5 PE=1 SV=3	YFAGNLASGGAAGATSLCFVYPLDFAR
>	sp|Q09073|AD ADP/ATP translocase 2 OS=Rattus norvegicus GN=Slc25a5 PE=1 SV=3	YFAGNLASGGAAGATSLCFVYPLDFAR
>	sp|Q09073|AD ADP/ATP translocase 2 OS=Rattus norvegicus GN=Slc25a5 PE=1 SV=3	YFAGNLASGGAAGATSLCFVYPLDFAR
>	sp|Q09073|AD ADP/ATP translocase 2 OS=Rattus norvegicus GN=Slc25a5 PE=1 SV=3	YFAGNLASGGAAGATSLCFVYPLDFAR
>	sp|Q09073|AD ADP/ATP translocase 2 OS=Rattus norvegicus GN=Slc25a5 PE=1 SV=3	YFAGNLASGGAAGATSLCFVYPLDFAR
>	sp|Q09073|AD ADP/ATP translocase 2 OS=Rattus norvegicus GN=Slc25a5 PE=1 SV=3	YFPTQALNFAFK
>	sp|Q09073|AD ADP/ATP translocase 2 OS=Rattus norvegicus GN=Slc25a5 PE=1 SV=3	YFPTQALNFAFK
>	sp|Q09073|AD ADP/ATP translocase 2 OS=Rattus norvegicus GN=Slc25a5 PE=1 SV=3	YFPTQALNFAFK
>	sp|Q09073|AD ADP/ATP translocase 2 OS=Rattus norvegicus GN=Slc25a5 PE=1 SV=3	GMGGAFVLVLYDEIKK
>	sp|Q09073|AD ADP/ATP translocase 2 OS=Rattus norvegicus GN=Slc25a5 PE=1 SV=3	TAVAPIER
>	sp|Q09073|AD ADP/ATP translocase 2 OS=Rattus norvegicus GN=Slc25a5 PE=1 SV=3	GMGGAFVLVLYDEIKK
>	sp|Q09073|AD ADP/ATP translocase 2 OS=Rattus norvegicus GN=Slc25a5 PE=1 SV=3	KGTDIMYTGTLDCWR
>	sp|Q09073|AD ADP/ATP translocase 2 OS=Rattus norvegicus GN=Slc25a5 PE=1 SV=3	QIFLGGVDKR
>	sp|Q09073|AD ADP/ATP translocase 2 OS=Rattus norvegicus GN=Slc25a5 PE=1 SV=3	GAWSNVLR
>	sp|Q09073|AD ADP/ATP translocase 2 OS=Rattus norvegicus GN=Slc25a5 PE=1 SV=3	GMLPDPK

>	sp|Q09073|AD	ADP/ATP translocase 2 OS=Rattus norvegicus GN=Slc25a5 PE=1 SV=3	LAADVGK
>	sp|Q09073|AD	ADP/ATP translocase 2 OS=Rattus norvegicus GN=Slc25a5 PE=1 SV=3	LLLQVQHASK
>	sp|Q05140|AP	Clathrin coat assembly protein AP180 OS=Rattus norvegicus GN=Snap91 PE=1 SV=1	ALVTTHHLMVHGNER
>	sp|Q05140|AP	Clathrin coat assembly protein AP180 OS=Rattus norvegicus GN=Snap91 PE=1 SV=1	IAAAQYSVTGSAVAR
>	sp|Q05140|AP	Clathrin coat assembly protein AP180 OS=Rattus norvegicus GN=Snap91 PE=1 SV=1	NTLFNLSNFLDK
>	sp|Q05140|AP	Clathrin coat assembly protein AP180 OS=Rattus norvegicus GN=Snap91 PE=1 SV=1	NTLFNLSNFLDK
>	sp|Q05140|AP	Clathrin coat assembly protein AP180 OS=Rattus norvegicus GN=Snap91 PE=1 SV=1	NTLFNLSNFLDK
>	sp|Q05140|AP	Clathrin coat assembly protein AP180 OS=Rattus norvegicus GN=Snap91 PE=1 SV=1	NTLFNLSNFLDK
>	sp|Q05140|AP	Clathrin coat assembly protein AP180 OS=Rattus norvegicus GN=Snap91 PE=1 SV=1	ATTHEVMGPK
>	sp|Q05140|AP	Clathrin coat assembly protein AP180 OS=Rattus norvegicus GN=Snap91 PE=1 SV=1	NTLFNLSNFLDK
>	sp|Q01986|MP	Dual specificity mitogen-activated protein kinase kinase 1 OS=Rattus norvegicus GN=Map2k1 PE=1 SV=2	KLIHLEIKPAIR
>	sp|Q01986|MP	Dual specificity mitogen-activated protein kinase kinase 1 OS=Rattus norvegicus GN=Map2k1 PE=1 SV=2	QLMVHAFIK
>	sp|Q01986|MP	Dual specificity mitogen-activated protein kinase kinase 1 OS=Rattus norvegicus GN=Map2k1 PE=1 SV=2	QLMVHAFIK
>	sp|Q01986|MP	Dual specificity mitogen-activated protein kinase kinase 1 OS=Rattus norvegicus GN=Map2k1 PE=1 SV=2	QLMVHAFIK
>	sp|Q01986|MP	Dual specificity mitogen-activated protein kinase kinase 1 OS=Rattus norvegicus GN=Map2k1 PE=1 SV=2	QLMVHAFIK
>	sp|Q01986|MP	Dual specificity mitogen-activated protein kinase kinase 1 OS=Rattus norvegicus GN=Map2k1 PE=1 SV=2	KLEELELDEQQR
>	sp|Q01986|MP	Dual specificity mitogen-activated protein kinase kinase 1 OS=Rattus norvegicus GN=Map2k1 PE=1 SV=2	KLEELELDEQQR
>	sp|Q01986|MP	Dual specificity mitogen-activated protein kinase kinase 1 OS=Rattus norvegicus GN=Map2k1 PE=1 SV=2	ELELLFGCQVEGDAAETPPRPR
>	sp|Q641Y2|ND	NADH dehydrogenase [ubiquinone] iron-sulfur protein 2, mitochondrial OS=Rattus norvegicus GN=Ndufs2 PE=1 SV=1	IKAPGFAHLAGLDK
>	sp|Q641Y2|ND	NADH dehydrogenase [ubiquinone] iron-sulfur protein 2, mitochondrial OS=Rattus norvegicus GN=Ndufs2 PE=1 SV=1	VVTNVTLNFGPQHPAAHGVLR
>	sp|Q641Y2|ND	NADH dehydrogenase [ubiquinone] iron-sulfur protein 2, mitochondrial OS=Rattus norvegicus GN=Ndufs2 PE=1 SV=1	MHAAYIRPGGVHQDLPLGLMDDIYEFSK
>	sp|Q641Y2|ND	NADH dehydrogenase [ubiquinone] iron-sulfur protein 2, mitochondrial OS=Rattus norvegicus GN=Ndufs2 PE=1 SV=1	MHAAYIRPGGVHQDLPLGLMDDIYEFSK
>	sp|Q641Y2|ND	NADH dehydrogenase [ubiquinone] iron-sulfur protein 2, mitochondrial OS=Rattus norvegicus GN=Ndufs2 PE=1 SV=1	VVTNVTLNFGPQHPAAHGVLR
>	sp|Q641Y2|ND	NADH dehydrogenase [ubiquinone] iron-sulfur protein 2, mitochondrial OS=Rattus norvegicus GN=Ndufs2 PE=1 SV=1	VVTNVTLNFGPQHPAAHGVLR
>	sp|Q641Y2|ND	NADH dehydrogenase [ubiquinone] iron-sulfur protein 2, mitochondrial OS=Rattus norvegicus GN=Ndufs2 PE=1 SV=1	VVTNVTLNFGPQHPAAHGVLR
>	sp|Q641Y2|ND	NADH dehydrogenase [ubiquinone] iron-sulfur protein 2, mitochondrial OS=Rattus norvegicus GN=Ndufs2 PE=1 SV=1	VVTNVTLNFGPQHPAAHGVLR
>	sp|Q641Y2|ND	NADH dehydrogenase [ubiquinone] iron-sulfur protein 2, mitochondrial OS=Rattus norvegicus GN=Ndufs2 PE=1 SV=1	VVTNVTLNFGPQHPAAHGVLR
>	sp|Q641Y2|ND	NADH dehydrogenase [ubiquinone] iron-sulfur protein 2, mitochondrial OS=Rattus norvegicus GN=Ndufs2 PE=1 SV=1	VVTNVTLNFGPQHPAAHGVLR
>	sp|Q641Y2|ND	NADH dehydrogenase [ubiquinone] iron-sulfur protein 2, mitochondrial OS=Rattus norvegicus GN=Ndufs2 PE=1 SV=1	VVTNVTLNFGPQHPAAHGVLR
>	sp|Q641Y2|ND	NADH dehydrogenase [ubiquinone] iron-sulfur protein 2, mitochondrial OS=Rattus norvegicus GN=Ndufs2 PE=1 SV=1	VSGARMHAAYIRPGGVHQDLPLGLMDDIYEFSK
>	sp|P27139|CA	Carbonic anhydrase 2 OS=Rattus norvegicus GN=Ca2 PE=1 SV=2	AVQHPDGLAVLGIFLK
>	sp|P27139|CA	Carbonic anhydrase 2 OS=Rattus norvegicus GN=Ca2 PE=1 SV=2	ITEALHSIK
>	sp|P27139|CA	Carbonic anhydrase 2 OS=Rattus norvegicus GN=Ca2 PE=1 SV=2	KLNFNSEGEAEELMVDNWRPAQPLK
>	sp|P27139|CA	Carbonic anhydrase 2 OS=Rattus norvegicus GN=Ca2 PE=1 SV=2	AVQHPDGLAVLGIFLK
>	sp|P27139|CA	Carbonic anhydrase 2 OS=Rattus norvegicus GN=Ca2 PE=1 SV=2	AVQHPDGLAVLGIFLK
>	sp|P27139|CA	Carbonic anhydrase 2 OS=Rattus norvegicus GN=Ca2 PE=1 SV=2	AVQHPDGLAVLGIFLK
>	sp|P27139|CA	Carbonic anhydrase 2 OS=Rattus norvegicus GN=Ca2 PE=1 SV=2	AVQHPDGLAVLGIFLK
>	sp|P27139|CA	Carbonic anhydrase 2 OS=Rattus norvegicus GN=Ca2 PE=1 SV=2	AVQHPDGLAVLGIFLK
>	sp|P27139|CA	Carbonic anhydrase 2 OS=Rattus norvegicus GN=Ca2 PE=1 SV=2	AVQHPDGLAVLGIFLK
>	sp|P27139|CA	Carbonic anhydrase 2 OS=Rattus norvegicus GN=Ca2 PE=1 SV=2	AVQHPDGLAVLGIFLK
>	sp|P27139|CA	Carbonic anhydrase 2 OS=Rattus norvegicus GN=Ca2 PE=1 SV=2	AVQHPDGLAVLGIFLK
>	sp|P27139|CA	Carbonic anhydrase 2 OS=Rattus norvegicus GN=Ca2 PE=1 SV=2	AVQHPDGLAVLGIFLK
>	sp|P27139|CA	Carbonic anhydrase 2 OS=Rattus norvegicus GN=Ca2 PE=1 SV=2	ITEALHSIK
>	sp|P27139|CA	Carbonic anhydrase 2 OS=Rattus norvegicus GN=Ca2 PE=1 SV=2	KLNFNSEGEAEELMVDNWRPAQPLK
>	sp|P27139|CA	Carbonic anhydrase 2 OS=Rattus norvegicus GN=Ca2 PE=1 SV=2	ITEALHSIK
>	sp|P27139|CA	Carbonic anhydrase 2 OS=Rattus norvegicus GN=Ca2 PE=1 SV=2	ITEALHSIK
>	sp|P27139|CA	Carbonic anhydrase 2 OS=Rattus norvegicus GN=Ca2 PE=1 SV=2	KLNFNSEGEAEELMVDNWRPAQPLK
>	sp|P27139|CA	Carbonic anhydrase 2 OS=Rattus norvegicus GN=Ca2 PE=1 SV=2	KLNFNSEGEAEELMVDNWRPAQPLK
>	sp|P27139|CA	Carbonic anhydrase 2 OS=Rattus norvegicus GN=Ca2 PE=1 SV=2	IGPASQGLQK
>	sp|Q5XI73|GDI	Rho GDP-dissociation inhibitor 1 OS=Rattus norvegicus GN=Arhgdia PE=1 SV=1	VAVSADPNVPNVIVTR
>	sp|Q5XI73|GDI	Rho GDP-dissociation inhibitor 1 OS=Rattus norvegicus GN=Arhgdia PE=1 SV=1	LTLVCSTAPGPLELDLTGDLESFKK
>	sp|Q5XI73|GDI	Rho GDP-dissociation inhibitor 1 OS=Rattus norvegicus GN=Arhgdia PE=1 SV=1	VAVSADPNVPNVIVTR
>	sp|Q5XI73|GDI	Rho GDP-dissociation inhibitor 1 OS=Rattus norvegicus GN=Arhgdia PE=1 SV=1	VAVSADPNVPNVIVTR
>	sp|Q5XI73|GDI	Rho GDP-dissociation inhibitor 1 OS=Rattus norvegicus GN=Arhgdia PE=1 SV=1	SIQEIQELDKDDESLR
>	sp|Q5XI73|GDI	Rho GDP-dissociation inhibitor 1 OS=Rattus norvegicus GN=Arhgdia PE=1 SV=1	LTLVCSTAPGPLELDLTGDLESFKK
>	sp|Q5XI73|GDI	Rho GDP-dissociation inhibitor 1 OS=Rattus norvegicus GN=Arhgdia PE=1 SV=1	LTLVCSTAPGPLELDLTGDLESFKK
>	sp|Q5XI73|GDI	Rho GDP-dissociation inhibitor 1 OS=Rattus norvegicus GN=Arhgdia PE=1 SV=1	LTLVCSTAPGPLELDLTGDLESFKK
>	sp|P84087|CPL	Complexin-2 OS=Rattus norvegicus GN=Cplx2 PE=1 SV=1	AALEQPCEGSLTRPK
>	sp|P84087|CPL	Complexin-2 OS=Rattus norvegicus GN=Cplx2 PE=1 SV=1	KAIPAGCGDEEEEEEESILDTVLK
>	sp|P84087|CPL	Complexin-2 OS=Rattus norvegicus GN=Cplx2 PE=1 SV=1	AALEQPCEGSLTRPK
>	sp|P84087|CPL	Complexin-2 OS=Rattus norvegicus GN=Cplx2 PE=1 SV=1	KAIPAGCGDEEEEEEESILDTVLK
>	sp|P84087|CPL	Complexin-2 OS=Rattus norvegicus GN=Cplx2 PE=1 SV=1	YLPGPLQDMFK
>	sp|P84087|CPL	Complexin-2 OS=Rattus norvegicus GN=Cplx2 PE=1 SV=1	YLPGPLQDMFK
>	sp|P84087|CPL	Complexin-2 OS=Rattus norvegicus GN=Cplx2 PE=1 SV=1	YLPGPLQDMFK
>	sp|P84087|CPL	Complexin-2 OS=Rattus norvegicus GN=Cplx2 PE=1 SV=1	YLPGPLQDMFK
>	sp|P84087|CPL	Complexin-2 OS=Rattus norvegicus GN=Cplx2 PE=1 SV=1	YLPGPLQDMFK
>	sp|P84087|CPL	Complexin-2 OS=Rattus norvegicus GN=Cplx2 PE=1 SV=1	YLPGPLQDMFK
>	sp|P84087|CPL	Complexin-2 OS=Rattus norvegicus GN=Cplx2 PE=1 SV=1	YLPGPLQDMFK
>	sp|P84087|CPL	Complexin-2 OS=Rattus norvegicus GN=Cplx2 PE=1 SV=1	YLPGPLQDMFK
>	sp|P84087|CPL	Complexin-2 OS=Rattus norvegicus GN=Cplx2 PE=1 SV=1	MLGGEEEKDPDAQKKEEERQEALR
>	sp|P84087|CPL	Complexin-2 OS=Rattus norvegicus GN=Cplx2 PE=1 SV=1	AALEQPCEGSLTRPK
>	sp|P84087|CPL	Complexin-2 OS=Rattus norvegicus GN=Cplx2 PE=1 SV=1	YLPGPLQDMFK
>	sp|P84087|CPL	Complexin-2 OS=Rattus norvegicus GN=Cplx2 PE=1 SV=1	YLPGPLQDMFK
>	sp|P84087|CPL	Complexin-2 OS=Rattus norvegicus GN=Cplx2 PE=1 SV=1	AALEQPCEGSLTRPK

>	sp|Q63564|SV2	Synaptic vesicle glycoprotein 2B OS=Rattus norvegicus GN=Sv2b PE=1 SV=1	VFTVSHIK
>	sp|Q63564|SV2	Synaptic vesicle glycoprotein 2B OS=Rattus norvegicus GN=Sv2b PE=1 SV=1	HVLFEDTFFDK
>	sp|Q63564|SV2	Synaptic vesicle glycoprotein 2B OS=Rattus norvegicus GN=Sv2b PE=1 SV=1	VFTVSHIK
>	sp|Q63564|SV2	Synaptic vesicle glycoprotein 2B OS=Rattus norvegicus GN=Sv2b PE=1 SV=1	FLLEMGK
>	sp|Q63564|SV2	Synaptic vesicle glycoprotein 2B OS=Rattus norvegicus GN=Sv2b PE=1 SV=1	VFTVSHIK
>	sp|Q63564|SV2	Synaptic vesicle glycoprotein 2B OS=Rattus norvegicus GN=Sv2b PE=1 SV=1	HVLFEDTFFDK
>	sp|Q63564|SV2	Synaptic vesicle glycoprotein 2B OS=Rattus norvegicus GN=Sv2b PE=1 SV=1	HVLFEDTFFDK
>	sp|Q63564|SV2	Synaptic vesicle glycoprotein 2B OS=Rattus norvegicus GN=Sv2b PE=1 SV=1	FLLEMGKHDEAWMILK
>	sp|P61589|RH	Transforming protein RhoA OS=Rattus norvegicus GN=Rhoa PE=1 SV=1	HFCPNVPIILVGNK
>	sp|P61589|RH	Transforming protein RhoA OS=Rattus norvegicus GN=Rhoa PE=1 SV=1	QVELALWDTAGQEDYDR
>	sp|P61589|RH	Transforming protein RhoA OS=Rattus norvegicus GN=Rhoa PE=1 SV=1	HFCPNVPIILVGNK
>	sp|P61589|RH	Transforming protein RhoA OS=Rattus norvegicus GN=Rhoa PE=1 SV=1	HFCPNVPIILVGNK
>	sp|P61589|RH	Transforming protein RhoA OS=Rattus norvegicus GN=Rhoa PE=1 SV=1	HFCPNVPIILVGNK
>	sp|P61589|RH	Transforming protein RhoA OS=Rattus norvegicus GN=Rhoa PE=1 SV=1	LRPLSYPDTDVILMCFSIDSPDSLENIPEK
>	sp|P61589|RH	Transforming protein RhoA OS=Rattus norvegicus GN=Rhoa PE=1 SV=1	HFCPNVPIILVGNK
>	sp|P61589|RH	Transforming protein RhoA OS=Rattus norvegicus GN=Rhoa PE=1 SV=1	HFCPNVPIILVGNK
>	sp|P61589|RH	Transforming protein RhoA OS=Rattus norvegicus GN=Rhoa PE=1 SV=1	LRPLSYPDTDVILMCFSIDSPDSLENIPEKWTPEVK
>	sp|P61589|RH	Transforming protein RhoA OS=Rattus norvegicus GN=Rhoa PE=1 SV=1	LVIVGDGACGK
>	sp|P61589|RH	Transforming protein RhoA OS=Rattus norvegicus GN=Rhoa PE=1 SV=1	LRPLSYPDTDVILMCFSIDSPDSLENIPEKWTPEVK
>	sp|Q68FR6|EF1	Elongation factor 1-gamma OS=Rattus norvegicus GN=Eef1g PE=1 SV=3	ILGLLDTHLK
>	sp|Q68FR6|EF1	Elongation factor 1-gamma OS=Rattus norvegicus GN=Eef1g PE=1 SV=3	VLSAPPHFHFGQTNR
>	sp|Q68FR6|EF1	Elongation factor 1-gamma OS=Rattus norvegicus GN=Eef1g PE=1 SV=3	GSTPEAAAQVVQWVSFADSDIVPPASTWVFPTLGIMHHNK
>	sp|Q68FR6|EF1	Elongation factor 1-gamma OS=Rattus norvegicus GN=Eef1g PE=1 SV=3	ILGLLDTHLK
>	sp|Q68FR6|EF1	Elongation factor 1-gamma OS=Rattus norvegicus GN=Eef1g PE=1 SV=3	ILGLLDTHLK
>	sp|Q68FR6|EF1	Elongation factor 1-gamma OS=Rattus norvegicus GN=Eef1g PE=1 SV=3	VLSAPPHFHFGQTNR
>	sp|Q68FR6|EF1	Elongation factor 1-gamma OS=Rattus norvegicus GN=Eef1g PE=1 SV=3	VLSAPPHFHFGQTNR
>	sp|Q68FR6|EF1	Elongation factor 1-gamma OS=Rattus norvegicus GN=Eef1g PE=1 SV=3	AKDPFAHLPK
>	sp|Q68FR6|EF1	Elongation factor 1-gamma OS=Rattus norvegicus GN=Eef1g PE=1 SV=3	VLSAPPHFHFGQTNR
>	sp|Q68FR6|EF1	Elongation factor 1-gamma OS=Rattus norvegicus GN=Eef1g PE=1 SV=3	VLSAPPHFHFGQTNR
>	sp|P07825|SYP	Synaptophysin OS=Rattus norvegicus GN=Syp PE=1 SV=1	MATDPENIIK
>	sp|P07825|SYP	Synaptophysin OS=Rattus norvegicus GN=Syp PE=1 SV=1	LHQVYFDAPSCVK
>	sp|P07825|SYP	Synaptophysin OS=Rattus norvegicus GN=Syp PE=1 SV=1	MATDPENIIK
>	sp|P07825|SYP	Synaptophysin OS=Rattus norvegicus GN=Syp PE=1 SV=1	LHQVYFDAPSCVK
>	sp|P07825|SYP	Synaptophysin OS=Rattus norvegicus GN=Syp PE=1 SV=1	GLSDVK
>	sp|P07825|SYP	Synaptophysin OS=Rattus norvegicus GN=Syp PE=1 SV=1	GLSDVK
>	sp|P07825|SYP	Synaptophysin OS=Rattus norvegicus GN=Syp PE=1 SV=1	MATDPENIIK
>	sp|P07825|SYP	Synaptophysin OS=Rattus norvegicus GN=Syp PE=1 SV=1	MATDPENIIK
>	sp|P07825|SYP	Synaptophysin OS=Rattus norvegicus GN=Syp PE=1 SV=1	GLSDVK
>	sp|P07825|SYP	Synaptophysin OS=Rattus norvegicus GN=Syp PE=1 SV=1	GLSDVK
>	sp|A0JPJ7|OLA	Obg-like ATPase 1 OS=Rattus norvegicus GN=Ola1 PE=2 SV=1	HLLLTSKPMVYLVNLSEKDYIR
>	sp|A0JPJ7|OLA	Obg-like ATPase 1 OS=Rattus norvegicus GN=Ola1 PE=2 SV=1	IPAFLNVVDIAGLVK
>	sp|A0JPJ7|OLA	Obg-like ATPase 1 OS=Rattus norvegicus GN=Ola1 PE=2 SV=1	IPAFLNVVDIAGLVK
>	sp|A0JPJ7|OLA	Obg-like ATPase 1 OS=Rattus norvegicus GN=Ola1 PE=2 SV=1	IPAFLNVVDIAGLVK
>	sp|A0JPJ7|OLA	Obg-like ATPase 1 OS=Rattus norvegicus GN=Ola1 PE=2 SV=1	IGIVGLPNVGK
>	sp|Q05683|DC	Glutamate decarboxylase 2 OS=Rattus norvegicus GN=Gad2 PE=1 SV=1	MMGVPLQCSALLVR
>	sp|Q05683|DC	Glutamate decarboxylase 2 OS=Rattus norvegicus GN=Gad2 PE=1 SV=1	VIDFHYPNELLQEYNWELADQPQNLEEILTHCQTTLK
>	sp|Q05683|DC	Glutamate decarboxylase 2 OS=Rattus norvegicus GN=Gad2 PE=1 SV=1	MVISNPAATHQDIDFLIEEIER
>	sp|Q05683|DC	Glutamate decarboxylase 2 OS=Rattus norvegicus GN=Gad2 PE=1 SV=1	VIDFHYPNELLQEYNWELADQPQNLEEILTHCQTTLK
>	sp|Q05683|DC	Glutamate decarboxylase 2 OS=Rattus norvegicus GN=Gad2 PE=1 SV=1	VIDFHYPNELLQEYNWELADQPQNLEEILTHCQTTLK
>	sp|Q05683|DC	Glutamate decarboxylase 2 OS=Rattus norvegicus GN=Gad2 PE=1 SV=1	VIDFHYPNELLQEYNWELADQPQNLEEILTHCQTTLK
>	sp|Q05683|DC	Glutamate decarboxylase 2 OS=Rattus norvegicus GN=Gad2 PE=1 SV=1	VIDFHYPNELLQEYNWELADQPQNLEEILTHCQTTLK
>	sp|Q05683|DC	Glutamate decarboxylase 2 OS=Rattus norvegicus GN=Gad2 PE=1 SV=1	MVISNPAATHQDIDFLIEEIER
>	sp|Q6P7Q4|LG	Lactoylglutathione lyase OS=Rattus norvegicus GN=Glo1 PE=1 SV=3	GFGHIGIAVPDVYEACK
>	sp|Q6P7Q4|LG	Lactoylglutathione lyase OS=Rattus norvegicus GN=Glo1 PE=1 SV=3	VLGLTLLQK
>	sp|Q6P7Q4|LG	Lactoylglutathione lyase OS=Rattus norvegicus GN=Glo1 PE=1 SV=3	VLGLTLLQK
>	sp|Q6P7Q4|LG	Lactoylglutathione lyase OS=Rattus norvegicus GN=Glo1 PE=1 SV=3	RFEELGVK
>	sp|Q6P7Q4|LG	Lactoylglutathione lyase OS=Rattus norvegicus GN=Glo1 PE=1 SV=3	RFEELGVK
>	sp|P11232|TH	Thioredoxin OS=Rattus norvegicus GN=Txn PE=1 SV=2	EAFQEALAAAGDK
>	sp|P11232|TH	Thioredoxin OS=Rattus norvegicus GN=Txn PE=1 SV=2	VGEFSGANK
>	sp|P11232|TH	Thioredoxin OS=Rattus norvegicus GN=Txn PE=1 SV=2	EAFQEALAAAGDK
>	sp|P11232|TH	Thioredoxin OS=Rattus norvegicus GN=Txn PE=1 SV=2	EAFQEALAAAGDK
>	sp|P11232|TH	Thioredoxin OS=Rattus norvegicus GN=Txn PE=1 SV=2	EAFQEALAAAGDK
>	sp|P11232|TH	Thioredoxin OS=Rattus norvegicus GN=Txn PE=1 SV=2	EAFQEALAAAGDK
>	sp|P11232|TH	Thioredoxin OS=Rattus norvegicus GN=Txn PE=1 SV=2	EAFQEALAAAGDK
>	sp|P11232|TH	Thioredoxin OS=Rattus norvegicus GN=Txn PE=1 SV=2	EAFQEALAAAGDK
>	sp|P11232|TH	Thioredoxin OS=Rattus norvegicus GN=Txn PE=1 SV=2	MIKPFFHSLCDK
>	sp|Q9WTT6|G	Guanine deaminase OS=Rattus norvegicus GN=Gda PE=1 SV=1	IVFLEESSQQEK
>	sp|Q9WTT6|G	Guanine deaminase OS=Rattus norvegicus GN=Gda PE=1 SV=1	LATLGGSQALGLDREIGNFEVGKDFDALLINPR
>	sp|Q9WTT6|G	Guanine deaminase OS=Rattus norvegicus GN=Gda PE=1 SV=1	EIGNFEVGKDFDALLINPR
>	sp|Q9WTT6|G	Guanine deaminase OS=Rattus norvegicus GN=Gda PE=1 SV=1	EIGNFEVGKDFDALLINPR
>	sp|Q9WTT6|G	Guanine deaminase OS=Rattus norvegicus GN=Gda PE=1 SV=1	EIGNFEVGKDFDALLINPR
>	sp|Q9WTT6|G	Guanine deaminase OS=Rattus norvegicus GN=Gda PE=1 SV=1	EIGNFEVGKDFDALLINPR
>	sp|P97546|NP	Neuroplastin OS=Rattus norvegicus GN=Nptn PE=1 SV=2	AAPDITGHKR
>	sp|P97546|NP	Neuroplastin OS=Rattus norvegicus GN=Nptn PE=1 SV=2	AAPDITGHK

>	sp|P97546|NPT	Neuroplastin OS=Rattus norvegicus GN=Nptn PE=1 SV=2	AQATISVLQKPR
>	sp|P97546|NPT	Neuroplastin OS=Rattus norvegicus GN=Nptn PE=1 SV=2	IVTSEEVIIR
>	sp|P97546|NPT	Neuroplastin OS=Rattus norvegicus GN=Nptn PE=1 SV=2	IVTSEEVIIR
>	sp|P09812|PYG	Glycogen phosphorylase, muscle form OS=Rattus norvegicus GN=Pygm PE=2 SV=5	ARPEFTLPVHFYGR
>	sp|P09812|PYG	Glycogen phosphorylase, muscle form OS=Rattus norvegicus GN=Pygm PE=2 SV=5	RWLVLCNPGLAEVIAER
>	sp|P09812|PYG	Glycogen phosphorylase, muscle form OS=Rattus norvegicus GN=Pygm PE=2 SV=5	LKQEYFVVAATLQDIIR
>	sp|P09812|PYG	Glycogen phosphorylase, muscle form OS=Rattus norvegicus GN=Pygm PE=2 SV=5	LKQEYFVVAATLQDIIR
>	sp|P09812|PYG	Glycogen phosphorylase, muscle form OS=Rattus norvegicus GN=Pygm PE=2 SV=5	LKQEYFVVAATLQDIIR
>	sp|P09812|PYG	Glycogen phosphorylase, muscle form OS=Rattus norvegicus GN=Pygm PE=2 SV=5	LKQEYFVVAATLQDIIR
>	sp|P09812|PYG	Glycogen phosphorylase, muscle form OS=Rattus norvegicus GN=Pygm PE=2 SV=5	LKQEYFVVAATLQDIIR
>	sp|P09812|PYG	Glycogen phosphorylase, muscle form OS=Rattus norvegicus GN=Pygm PE=2 SV=5	LKQEYFVVAATLQDIIR
>	sp|P09812|PYG	Glycogen phosphorylase, muscle form OS=Rattus norvegicus GN=Pygm PE=2 SV=5	LKQEYFVVAATLQDIIR
>	sp|P09812|PYG	Glycogen phosphorylase, muscle form OS=Rattus norvegicus GN=Pygm PE=2 SV=5	LKQEYFVVAATLQDIIR
>	sp|P09812|PYG	Glycogen phosphorylase, muscle form OS=Rattus norvegicus GN=Pygm PE=2 SV=5	LKQEYFVVAATLQDIIRRFKSSK
>	sp|P09812|PYG	Glycogen phosphorylase, muscle form OS=Rattus norvegicus GN=Pygm PE=2 SV=5	LKQEYFVVAATLQDIIRRFKSSK
>	sp|P09812|PYG	Glycogen phosphorylase, muscle form OS=Rattus norvegicus GN=Pygm PE=2 SV=5	RWLVLCNPGLAEVIAER
>	sp|P09812|PYG	Glycogen phosphorylase, muscle form OS=Rattus norvegicus GN=Pygm PE=2 SV=5	VIFLENYR
>	sp|P09812|PYG	Glycogen phosphorylase, muscle form OS=Rattus norvegicus GN=Pygm PE=2 SV=5	QLLNCLHIITLYNR
>	sp|P09812|PYG	Glycogen phosphorylase, muscle form OS=Rattus norvegicus GN=Pygm PE=2 SV=5	HLHFTLVK
>	sp|Q5M7U6|AI	Actin-related protein 2 OS=Rattus norvegicus GN=Actr2 PE=1 SV=1	HMVFLGGAVLADIMK
>	sp|Q5M7U6|AI	Actin-related protein 2 OS=Rattus norvegicus GN=Actr2 PE=1 SV=1	LALETTVLVESYTLPDGR
>	sp|Q5M7U6|AI	Actin-related protein 2 OS=Rattus norvegicus GN=Actr2 PE=1 SV=1	LDIAGR
>	sp|Q5M7U6|AI	Actin-related protein 2 OS=Rattus norvegicus GN=Actr2 PE=1 SV=1	ILLTEPPMNPTK
>	sp|Q5M7U6|AI	Actin-related protein 2 OS=Rattus norvegicus GN=Actr2 PE=1 SV=1	LDIAGR
>	sp|Q5M7U6|AI	Actin-related protein 2 OS=Rattus norvegicus GN=Actr2 PE=1 SV=1	LDIAGR
>	sp|Q9WU82|CT	Catenin beta-1 OS=Rattus norvegicus GN=Ctnnb1 PE=1 SV=1	HQEAEMAQNAVR
>	sp|Q9WU82|CT	Catenin beta-1 OS=Rattus norvegicus GN=Ctnnb1 PE=1 SV=1	VAAGVLCELAQDKEAAEAIEAEGATAPLTELLHSR
>	sp|Q9WU82|CT	Catenin beta-1 OS=Rattus norvegicus GN=Ctnnb1 PE=1 SV=1	VAAGVLCELAQDKEAAEAIEAEGATAPLTELLHSR
>	sp|Q9WU82|CT	Catenin beta-1 OS=Rattus norvegicus GN=Ctnnb1 PE=1 SV=1	HAVVNLINYQDDAELATR
>	sp|Q5XIH7|PHE	Prohibitin-2 OS=Rattus norvegicus GN=Phb2 PE=1 SV=1	AKDFSLILDDVAITELSFSR
>	sp|Q5XIH7|PHE	Prohibitin-2 OS=Rattus norvegicus GN=Phb2 PE=1 SV=1	IGGVQQDTILAEGLHFR
>	sp|Q5XIH7|PHE	Prohibitin-2 OS=Rattus norvegicus GN=Phb2 PE=1 SV=1	AKDFSLILDDVAITELSFSR
>	sp|O35331|PD	Pyridoxal kinase OS=Rattus norvegicus GN=Pdxk PE=1 SV=1	DKSFLGMVVDIVQELK
>	sp|O35331|PD	Pyridoxal kinase OS=Rattus norvegicus GN=Pdxk PE=1 SV=1	KIHSQEEAFAVMDVLHR
>	sp|Q3T1K5|CA	F-actin-capping protein subunit alpha-2 OS=Rattus norvegicus GN=Capza2 PE=1 SV=1	EGAAHAFAQYNLDQFTPVKIEGYEDQVLITEHGDLGNGK
>	sp|Q3T1K5|CA	F-actin-capping protein subunit alpha-2 OS=Rattus norvegicus GN=Capza2 PE=1 SV=1	FIIHAPPGEFNEVFNDVR
>	sp|Q3T1K5|CA	F-actin-capping protein subunit alpha-2 OS=Rattus norvegicus GN=Capza2 PE=1 SV=1	EGAAHAFAQYNLDQFTPVKIEGYEDQVLITEHGDLGNGK
>	sp|Q3T1K5|CA	F-actin-capping protein subunit alpha-2 OS=Rattus norvegicus GN=Capza2 PE=1 SV=1	EGAAHAFAQYNLDQFTPVKIEGYEDQVLITEHGDLGNGK
>	sp|Q3T1K5|CA	F-actin-capping protein subunit alpha-2 OS=Rattus norvegicus GN=Capza2 PE=1 SV=1	FIIHAPPGEFNEVFNDVR
>	sp|Q3T1K5|CA	F-actin-capping protein subunit alpha-2 OS=Rattus norvegicus GN=Capza2 PE=1 SV=1	FIIHAPPGEFNEVFNDVR
>	sp|Q3T1K5|CA	F-actin-capping protein subunit alpha-2 OS=Rattus norvegicus GN=Capza2 PE=1 SV=1	FIIHAPPGEFNEVFNDVR
>	sp|Q3T1K5|CA	F-actin-capping protein subunit alpha-2 OS=Rattus norvegicus GN=Capza2 PE=1 SV=1	FIIHAPPGEFNEVFNDVR
>	sp|Q3T1K5|CA	F-actin-capping protein subunit alpha-2 OS=Rattus norvegicus GN=Capza2 PE=1 SV=1	FIIHAPPGEFNEVFNDVR
>	sp|Q3T1K5|CA	F-actin-capping protein subunit alpha-2 OS=Rattus norvegicus GN=Capza2 PE=1 SV=1	FIIHAPPGEFNEVFNDVR
>	sp|Q3T1K5|CA	F-actin-capping protein subunit alpha-2 OS=Rattus norvegicus GN=Capza2 PE=1 SV=1	EGAAHAFAQYNLDQFTPVKIEGYEDQVLITEHGDLGNGK
>	sp|Q5XI78|OD	2-oxoglutarate dehydrogenase, mitochondrial OS=Rattus norvegicus GN=Ogdh PE=1 SV=1	FLDTAFDLDAFKK
>	sp|Q5XI78|OD	2-oxoglutarate dehydrogenase, mitochondrial OS=Rattus norvegicus GN=Ogdh PE=1 SV=1	IEQLSPFPFDLLLK
>	sp|Q5XI78|OD	2-oxoglutarate dehydrogenase, mitochondrial OS=Rattus norvegicus GN=Ogdh PE=1 SV=1	IEQLSPFPFDLLLK
>	sp|P14604|ECH	Enoyl-CoA hydratase, mitochondrial OS=Rattus norvegicus GN=Echs1 PE=1 SV=1	FLSHWDHITR
>	sp|P14604|ECH	Enoyl-CoA hydratase, mitochondrial OS=Rattus norvegicus GN=Echs1 PE=1 SV=1	IFPVETLVEEAIQCAEK
>	sp|P10687|PLC	1-phosphatidylinositol-4,5-bisphosphate phosphodiesterase beta-1 OS=Rattus norvegicus GN=Plcb1 PE=1 SV=1	ELLDVGNIGHLEQR
>	sp|P10687|PLC	1-phosphatidylinositol-4,5-bisphosphate phosphodiesterase beta-1 OS=Rattus norvegicus GN=Plcb1 PE=1 SV=1	RLPLEILEFVQEAMK
>	sp|P10687|PLC	1-phosphatidylinositol-4,5-bisphosphate phosphodiesterase beta-1 OS=Rattus norvegicus GN=Plcb1 PE=1 SV=1	ELLDVGNIGHLEQR
>	sp|P10687|PLC	1-phosphatidylinositol-4,5-bisphosphate phosphodiesterase beta-1 OS=Rattus norvegicus GN=Plcb1 PE=1 SV=1	RLPLEILEFVQEAMK
>	sp|P10687|PLC	1-phosphatidylinositol-4,5-bisphosphate phosphodiesterase beta-1 OS=Rattus norvegicus GN=Plcb1 PE=1 SV=1	RLPLEILEFVQEAMK
>	sp|P10687|PLC	1-phosphatidylinositol-4,5-bisphosphate phosphodiesterase beta-1 OS=Rattus norvegicus GN=Plcb1 PE=1 SV=1	RLPLEILEFVQEAMK
>	sp|P62142|PP1	Serine/threonine-protein phosphatase PP1-beta catalytic subunit OS=Rattus norvegicus GN=Ppp1cb PE=1 SV=3	FLNRHDLDLICR
>	sp|P62142|PP1	Serine/threonine-protein phosphatase PP1-beta catalytic subunit OS=Rattus norvegicus GN=Ppp1cb PE=1 SV=3	EIFLSQPILLELEAPLK
>	sp|P62142|PP1	Serine/threonine-protein phosphatase PP1-beta catalytic subunit OS=Rattus norvegicus GN=Ppp1cb PE=1 SV=3	AHQVVEDGYEFFAK
>	sp|P62142|PP1	Serine/threonine-protein phosphatase PP1-beta catalytic subunit OS=Rattus norvegicus GN=Ppp1cb PE=1 SV=3	IMRPTDVPDTGLLCDLLWSDPDKDVQGWGENDR
>	sp|Q64568|AT	Plasma membrane calcium-transporting ATPase 3 OS=Rattus norvegicus GN=Atp2b3 PE=2 SV=2	EVPHVGGFGCTLAELR
>	sp|Q64568|AT	Plasma membrane calcium-transporting ATPase 3 OS=Rattus norvegicus GN=Atp2b3 PE=2 SV=2	ILDLLVHAISINSAYTTK
>	sp|Q64568|AT	Plasma membrane calcium-transporting ATPase 3 OS=Rattus norvegicus GN=Atp2b3 PE=2 SV=2	HLDACETMGNATAICSDK
>	sp|Q64568|AT	Plasma membrane calcium-transporting ATPase 3 OS=Rattus norvegicus GN=Atp2b3 PE=2 SV=2	ILDLLVHAISINSAYTTK
>	sp|Q64568|AT	Plasma membrane calcium-transporting ATPase 3 OS=Rattus norvegicus GN=Atp2b3 PE=2 SV=2	ILDLLVHAISINSAYTTK
>	sp|Q64568|AT	Plasma membrane calcium-transporting ATPase 3 OS=Rattus norvegicus GN=Atp2b3 PE=2 SV=2	KADVGFAMGIAGTDVAK
>	sp|Q64568|AT	Plasma membrane calcium-transporting ATPase 3 OS=Rattus norvegicus GN=Atp2b3 PE=2 SV=2	KADVGFAMGIAGTDVAK
>	sp|Q64568|AT	Plasma membrane calcium-transporting ATPase 3 OS=Rattus norvegicus GN=Atp2b3 PE=2 SV=2	KADVGFAMGIAGTDVAK
>	sp|Q64568|AT	Plasma membrane calcium-transporting ATPase 3 OS=Rattus norvegicus GN=Atp2b3 PE=2 SV=2	KADVGFAMGIAGTDVAK
>	sp|Q64568|AT	Plasma membrane calcium-transporting ATPase 3 OS=Rattus norvegicus GN=Atp2b3 PE=2 SV=2	KADVGFAMGIAGTDVAK
>	sp|Q64568|AT	Plasma membrane calcium-transporting ATPase 3 OS=Rattus norvegicus GN=Atp2b3 PE=2 SV=2	MVTGDNINTAR
>	sp|Q64568|AT	Plasma membrane calcium-transporting ATPase 3 OS=Rattus norvegicus GN=Atp2b3 PE=2 SV=2	MVVTAVGVNSQTGIIFTLLGAGGEEEEKKDKK
>	sp|Q64568|AT	Plasma membrane calcium-transporting ATPase 3 OS=Rattus norvegicus GN=Atp2b3 PE=2 SV=2	MVVTAVGVNSQTGIIFTLLGAGGEEEEKKDKK
>	sp|Q64568|AT	Plasma membrane calcium-transporting ATPase 3 OS=Rattus norvegicus GN=Atp2b3 PE=2 SV=2	NEKGEIEQER

>	sp|Q64568|AT	Plasma membrane calcium-transporting ATPase 3 OS=Rattus norvegicus GN=Atp2b3 PE=2 SV=2	NEKGEIEQER
>	sp|Q64568|AT	Plasma membrane calcium-transporting ATPase 3 OS=Rattus norvegicus GN=Atp2b3 PE=2 SV=2	QVVAVTGDGTNDGPALK
>	sp|Q64568|AT	Plasma membrane calcium-transporting ATPase 3 OS=Rattus norvegicus GN=Atp2b3 PE=2 SV=2	TECALLGFILDLKR
>	sp|Q64568|AT	Plasma membrane calcium-transporting ATPase 3 OS=Rattus norvegicus GN=Atp2b3 PE=2 SV=2	HLDACETMGNATAICSDK
>	sp|Q64568|AT	Plasma membrane calcium-transporting ATPase 3 OS=Rattus norvegicus GN=Atp2b3 PE=2 SV=2	HLDACETMGNATAICSDK
>	sp|Q64568|AT	Plasma membrane calcium-transporting ATPase 3 OS=Rattus norvegicus GN=Atp2b3 PE=2 SV=2	MVVTAVGVNSQTGIIFTLLGAGGEEEEKKDKK
>	sp|Q64568|AT	Plasma membrane calcium-transporting ATPase 3 OS=Rattus norvegicus GN=Atp2b3 PE=2 SV=2	MVVTAVGVNSQTGIIFTLLGAGGEEEEKKDKK
>	sp|Q64568|AT	Plasma membrane calcium-transporting ATPase 3 OS=Rattus norvegicus GN=Atp2b3 PE=2 SV=2	RGQILWFR
>	sp|Q64568|AT	Plasma membrane calcium-transporting ATPase 3 OS=Rattus norvegicus GN=Atp2b3 PE=2 SV=2	HTLVK
>	sp|Q64568|AT	Plasma membrane calcium-transporting ATPase 3 OS=Rattus norvegicus GN=Atp2b3 PE=2 SV=2	HLDACETMGNATAICSDK
>	sp|Q64568|AT	Plasma membrane calcium-transporting ATPase 3 OS=Rattus norvegicus GN=Atp2b3 PE=2 SV=2	LAELR
>	sp|P15791|KCC	Calcium/calmodulin-dependent protein kinase type II subunit delta OS=Rattus norvegicus GN=Camk2d PE=1 SV=1	ICDPGLTAFEPEALGNLVEGMDFHR
>	sp|P15791|KCC	Calcium/calmodulin-dependent protein kinase type II subunit delta OS=Rattus norvegicus GN=Camk2d PE=1 SV=1	INKPIHTIILNPHVHLVGDDAACIAYIR
>	sp|P15791|KCC	Calcium/calmodulin-dependent protein kinase type II subunit delta OS=Rattus norvegicus GN=Camk2d PE=1 SV=1	AGAYDFPSPEWDTVTPEAK
>	sp|P15791|KCC	Calcium/calmodulin-dependent protein kinase type II subunit delta OS=Rattus norvegicus GN=Camk2d PE=1 SV=1	AGAYDFPSPEWDTVTPEAK
>	sp|P15791|KCC	Calcium/calmodulin-dependent protein kinase type II subunit delta OS=Rattus norvegicus GN=Camk2d PE=1 SV=1	AGAYDFPSPEWDTVTPEAK
>	sp|P15791|KCC	Calcium/calmodulin-dependent protein kinase type II subunit delta OS=Rattus norvegicus GN=Camk2d PE=1 SV=1	AGAYDFPSPEWDTVTPEAK
>	sp|P15791|KCC	Calcium/calmodulin-dependent protein kinase type II subunit delta OS=Rattus norvegicus GN=Camk2d PE=1 SV=1	DLKPENLLLASK
>	sp|P15791|KCC	Calcium/calmodulin-dependent protein kinase type II subunit delta OS=Rattus norvegicus GN=Camk2d PE=1 SV=1	EYYSEADASHCIQQILESVNHCHLNGIVHR
>	sp|P15791|KCC	Calcium/calmodulin-dependent protein kinase type II subunit delta OS=Rattus norvegicus GN=Camk2d PE=1 SV=1	EYYSEADASHCIQQILESVNHCHLNGIVHR
>	sp|P15791|KCC	Calcium/calmodulin-dependent protein kinase type II subunit delta OS=Rattus norvegicus GN=Camk2d PE=1 SV=1	GAILTTMLATR
>	sp|P15791|KCC	Calcium/calmodulin-dependent protein kinase type II subunit delta OS=Rattus norvegicus GN=Camk2d PE=1 SV=1	GAILTTMLATR
>	sp|P15791|KCC	Calcium/calmodulin-dependent protein kinase type II subunit delta OS=Rattus norvegicus GN=Camk2d PE=1 SV=1	GAILTTMLATR
>	sp|P15791|KCC	Calcium/calmodulin-dependent protein kinase type II subunit delta OS=Rattus norvegicus GN=Camk2d PE=1 SV=1	INKPIHTIILNPHVHLVGDDAACIAYIR
>	sp|P15791|KCC	Calcium/calmodulin-dependent protein kinase type II subunit delta OS=Rattus norvegicus GN=Camk2d PE=1 SV=1	LHDSISEEGFHYLVFDLVTGGELFEDIVAR
>	sp|P15791|KCC	Calcium/calmodulin-dependent protein kinase type II subunit delta OS=Rattus norvegicus GN=Camk2d PE=1 SV=1	LHDSISEEGFHYLVFDLVTGGELFEDIVAR
>	sp|P15791|KCC	Calcium/calmodulin-dependent protein kinase type II subunit delta OS=Rattus norvegicus GN=Camk2d PE=1 SV=1	LKGAILTTMLATR
>	sp|P15791|KCC	Calcium/calmodulin-dependent protein kinase type II subunit delta OS=Rattus norvegicus GN=Camk2d PE=1 SV=1	QETVDCLKK
>	sp|P15791|KCC	Calcium/calmodulin-dependent protein kinase type II subunit delta OS=Rattus norvegicus GN=Camk2d PE=1 SV=1	AGAYDFPSPEWDTVTPEAK
>	sp|P15791|KCC	Calcium/calmodulin-dependent protein kinase type II subunit delta OS=Rattus norvegicus GN=Camk2d PE=1 SV=1	DLKPENLLLASK
>	sp|P15791|KCC	Calcium/calmodulin-dependent protein kinase type II subunit delta OS=Rattus norvegicus GN=Camk2d PE=1 SV=1	EYYSEADASHCIQQILESVNHCHLNGIVHR
>	sp|P15791|KCC	Calcium/calmodulin-dependent protein kinase type II subunit delta OS=Rattus norvegicus GN=Camk2d PE=1 SV=1	DLKPENLLLASK
>	sp|P15791|KCC	Calcium/calmodulin-dependent protein kinase type II subunit delta OS=Rattus norvegicus GN=Camk2d PE=1 SV=1	INKPIHTIILNPHVHLVGDDAACIAYIR
>	sp|P15791|KCC	Calcium/calmodulin-dependent protein kinase type II subunit delta OS=Rattus norvegicus GN=Camk2d PE=1 SV=1	LLKHPNIVR
>	sp|P15791|KCC	Calcium/calmodulin-dependent protein kinase type II subunit delta OS=Rattus norvegicus GN=Camk2d PE=1 SV=1	ICDPGLTAFEPEALGNLVEGMDFHR
>	sp|P15791|KCC	Calcium/calmodulin-dependent protein kinase type II subunit delta OS=Rattus norvegicus GN=Camk2d PE=1 SV=1	EYYSEADASHCIQQILESVNHCHLNGIVHR
>	sp|P15791|KCC	Calcium/calmodulin-dependent protein kinase type II subunit delta OS=Rattus norvegicus GN=Camk2d PE=1 SV=1	EYYSEADASHCIQQILESVNHCHLNGIVHR
>	sp|P15791|KCC	Calcium/calmodulin-dependent protein kinase type II subunit delta OS=Rattus norvegicus GN=Camk2d PE=1 SV=1	EYYSEADASHCIQQILESVNHCHLNGIVHR
>	sp|P15791|KCC	Calcium/calmodulin-dependent protein kinase type II subunit delta OS=Rattus norvegicus GN=Camk2d PE=1 SV=1	EYYSEADASHCIQQILESVNHCHLNGIVHR
>	sp|P15791|KCC	Calcium/calmodulin-dependent protein kinase type II subunit delta OS=Rattus norvegicus GN=Camk2d PE=1 SV=1	DLKPENLLLASK
>	sp|P15791|KCC	Calcium/calmodulin-dependent protein kinase type II subunit delta OS=Rattus norvegicus GN=Camk2d PE=1 SV=1	DLKPENLLLASK
>	sp|P15791|KCC	Calcium/calmodulin-dependent protein kinase type II subunit delta OS=Rattus norvegicus GN=Camk2d PE=1 SV=1	EYYSEADASHCIQQILESVNHCHLNGIVHR
>	sp|P15791|KCC	Calcium/calmodulin-dependent protein kinase type II subunit delta OS=Rattus norvegicus GN=Camk2d PE=1 SV=1	EYYSEADASHCIQQILESVNHCHLNGIVHR
>	sp|P15791|KCC	Calcium/calmodulin-dependent protein kinase type II subunit delta OS=Rattus norvegicus GN=Camk2d PE=1 SV=1	LLKHPNIVR
>	sp|P15791|KCC	Calcium/calmodulin-dependent protein kinase type II subunit delta OS=Rattus norvegicus GN=Camk2d PE=1 SV=1	DLKPENLLLASK
>	sp|P17220|PSA	Proteasome subunit alpha type-2 OS=Rattus norvegicus GN=Psma2 PE=1 SV=3	LVQIEYALAAVAGGAPSVGIK
>	sp|P17220|PSA	Proteasome subunit alpha type-2 OS=Rattus norvegicus GN=Psma2 PE=1 SV=3	RYNEDLELEDAIHTAILTLK
>	sp|P17220|PSA	Proteasome subunit alpha type-2 OS=Rattus norvegicus GN=Psma2 PE=1 SV=3	LVQIEYALAAVAGGAPSVGIK
>	sp|P17220|PSA	Proteasome subunit alpha type-2 OS=Rattus norvegicus GN=Psma2 PE=1 SV=3	LVQIEYALAAVAGGAPSVGIK
>	sp|P31000|VIM	Vimentin OS=Rattus norvegicus GN=Vim PE=1 SV=2	ILLAELEQLK
>	sp|P31000|VIM	Vimentin OS=Rattus norvegicus GN=Vim PE=1 SV=2	KLHDEEIQELQAQIQEQHVQIDVDVSKPDLTAALR
>	sp|P31000|VIM	Vimentin OS=Rattus norvegicus GN=Vim PE=1 SV=2	KLHDEEIQELQAQIQEQHVQIDVDVSKPDLTAALR
>	sp|P31000|VIM	Vimentin OS=Rattus norvegicus GN=Vim PE=1 SV=2	KLHDEEIQELQAQIQEQHVQIDVDVSKPDLTAALR
>	sp|P31000|VIM	Vimentin OS=Rattus norvegicus GN=Vim PE=1 SV=2	KLHDEEIQELQAQIQEQHVQIDVDVSKPDLTAALR
>	sp|P31000|VIM	Vimentin OS=Rattus norvegicus GN=Vim PE=1 SV=2	KLHDEEIQELQAQIQEQHVQIDVDVSKPDLTAALR
>	sp|P31000|VIM	Vimentin OS=Rattus norvegicus GN=Vim PE=1 SV=2	KLHDEEIQELQAQIQEQHVQIDVDVSKPDLTAALR
>	sp|P31000|VIM	Vimentin OS=Rattus norvegicus GN=Vim PE=1 SV=2	KLHDEEIQELQAQIQEQHVQIDVDVSKPDLTAALR
>	sp|P31000|VIM	Vimentin OS=Rattus norvegicus GN=Vim PE=1 SV=2	KLHDEEIQELQAQIQEQHVQIDVDVSKPDLTAALR
>	sp|P31000|VIM	Vimentin OS=Rattus norvegicus GN=Vim PE=1 SV=2	KLHDEEIQELQAQIQEQHVQIDVDVSKPDLTAALR
>	sp|P31000|VIM	Vimentin OS=Rattus norvegicus GN=Vim PE=1 SV=2	EYQDLLNVK
>	sp|P31000|VIM	Vimentin OS=Rattus norvegicus GN=Vim PE=1 SV=2	DGQVINETSQHHDDLE
>	sp|P31000|VIM	Vimentin OS=Rattus norvegicus GN=Vim PE=1 SV=2	KLLEGEESR
>	sp|P31000|VIM	Vimentin OS=Rattus norvegicus GN=Vim PE=1 SV=2	KLLEGEESR
>	sp|P97700|M2	Mitochondrial 2-oxoglutarate/malate carrier protein OS=Rattus norvegicus GN=Slc25a11 PE=2 SV=3	IAREEGVPTLWR
>	sp|P97700|M2	Mitochondrial 2-oxoglutarate/malate carrier protein OS=Rattus norvegicus GN=Slc25a11 PE=2 SV=3	LGPHTVLTFIFLEQMNK
>	sp|P97700|M2	Mitochondrial 2-oxoglutarate/malate carrier protein OS=Rattus norvegicus GN=Slc25a11 PE=2 SV=3	IAREEGVPTLWR
>	sp|P97700|M2	Mitochondrial 2-oxoglutarate/malate carrier protein OS=Rattus norvegicus GN=Slc25a11 PE=2 SV=3	LGPHTVLTFIFLEQMNK
>	sp|P97700|M2	Mitochondrial 2-oxoglutarate/malate carrier protein OS=Rattus norvegicus GN=Slc25a11 PE=2 SV=3	LGPHTVLTFIFLEQMNK
>	sp|P97700|M2	Mitochondrial 2-oxoglutarate/malate carrier protein OS=Rattus norvegicus GN=Slc25a11 PE=2 SV=3	LGPHTVLTFIFLEQMNK
>	sp|P97700|M2	Mitochondrial 2-oxoglutarate/malate carrier protein OS=Rattus norvegicus GN=Slc25a11 PE=2 SV=3	LGPHTVLTFIFLEQMNK
>	sp|P97700|M2	Mitochondrial 2-oxoglutarate/malate carrier protein OS=Rattus norvegicus GN=Slc25a11 PE=2 SV=3	LGPHTVLTFIFLEQMNK
>	sp|Q9JHU0|DP	Dihydropyrimidinase-related protein 5 OS=Rattus norvegicus GN=Dpysl5 PE=1 SV=1	EALDLGITGPEGIEISHPEELEAEATHR
>	sp|Q9JHU0|DP	Dihydropyrimidinase-related protein 5 OS=Rattus norvegicus GN=Dpysl5 PE=1 SV=1	AALVGGTTMIIGHVLPDKETSLVEAYEK
>	sp|Q9JHU0|DP	Dihydropyrimidinase-related protein 5 OS=Rattus norvegicus GN=Dpysl5 PE=1 SV=1	THCPIYLVNVSSISAGDVIAAAK

>	sp|Q9JHU0|DP	Dihydropyrimidinase-related protein 5 OS=Rattus norvegicus GN=Dpysl5 PE=1 SV=1	EALDLGITGPEGIEISHPEELEAEATHR
>	sp|Q9JHU0|DP	Dihydropyrimidinase-related protein 5 OS=Rattus norvegicus GN=Dpysl5 PE=1 SV=1	EALDLGITGPEGIEISHPEELEAEATHR
>	sp|Q9JHU0|DP	Dihydropyrimidinase-related protein 5 OS=Rattus norvegicus GN=Dpysl5 PE=1 SV=1	EALDLGITGPEGIEISHPEELEAEATHR
>	sp|Q9JHU0|DP	Dihydropyrimidinase-related protein 5 OS=Rattus norvegicus GN=Dpysl5 PE=1 SV=1	THCPIYLVNVSSISAGDVIAAAK
>	sp|Q9JHU0|DP	Dihydropyrimidinase-related protein 5 OS=Rattus norvegicus GN=Dpysl5 PE=1 SV=1	IIPGADADVVVWDPEATK
>	sp|Q9JHU0|DP	Dihydropyrimidinase-related protein 5 OS=Rattus norvegicus GN=Dpysl5 PE=1 SV=1	VHAENGELVAEGAK
>	sp|Q9JHU0|DP	Dihydropyrimidinase-related protein 5 OS=Rattus norvegicus GN=Dpysl5 PE=1 SV=1	AALVGGTTMIIGHVLPDKETSLVEAYEK
>	sp|Q9JHU0|DP	Dihydropyrimidinase-related protein 5 OS=Rattus norvegicus GN=Dpysl5 PE=1 SV=1	DSELYQVFHACR
>	sp|Q9JHU0|DP	Dihydropyrimidinase-related protein 5 OS=Rattus norvegicus GN=Dpysl5 PE=1 SV=1	VHAENGELVAEGAK
>	sp|Q62658|FKB	Peptidyl-prolyl cis-trans isomerase FKBP1A OS=Rattus norvegicus GN=Fkbp1a PE=2 SV=3	GWEEGVAQMSVGQR
>	sp|Q62658|FKB	Peptidyl-prolyl cis-trans isomerase FKBP1A OS=Rattus norvegicus GN=Fkbp1a PE=2 SV=3	GVQVETISSGDGR
>	sp|Q62658|FKB	Peptidyl-prolyl cis-trans isomerase FKBP1A OS=Rattus norvegicus GN=Fkbp1a PE=2 SV=3	GVQVETISSGDGR
>	sp|Q62658|FKB	Peptidyl-prolyl cis-trans isomerase FKBP1A OS=Rattus norvegicus GN=Fkbp1a PE=2 SV=3	GWEEGVAQMSVGQR
>	sp|Q62658|FKB	Peptidyl-prolyl cis-trans isomerase FKBP1A OS=Rattus norvegicus GN=Fkbp1a PE=2 SV=3	GWEEGVAQMSVGQR
>	sp|Q62658|FKB	Peptidyl-prolyl cis-trans isomerase FKBP1A OS=Rattus norvegicus GN=Fkbp1a PE=2 SV=3	GWEEGVAQMSVGQR
>	sp|Q62658|FKB	Peptidyl-prolyl cis-trans isomerase FKBP1A OS=Rattus norvegicus GN=Fkbp1a PE=2 SV=3	GVQVETISSGDGR
>	sp|P85834|EFT	Elongation factor Tu, mitochondrial OS=Rattus norvegicus GN=Tufm PE=1 SV=1	DKPHVNVGTIGHVDHGK
>	sp|P85834|EFT	Elongation factor Tu, mitochondrial OS=Rattus norvegicus GN=Tufm PE=1 SV=1	DLEKPFLLPVESVYSIPGR
>	sp|P85834|EFT	Elongation factor Tu, mitochondrial OS=Rattus norvegicus GN=Tufm PE=1 SV=1	ELLTEFGYKGEETPVIVGSALCALEQR
>	sp|P85834|EFT	Elongation factor Tu, mitochondrial OS=Rattus norvegicus GN=Tufm PE=1 SV=1	DLEKPFLLPVESVYSIPGR
>	sp|P85834|EFT	Elongation factor Tu, mitochondrial OS=Rattus norvegicus GN=Tufm PE=1 SV=1	DLEKPFLLPVESVYSIPGR
>	sp|P85834|EFT	Elongation factor Tu, mitochondrial OS=Rattus norvegicus GN=Tufm PE=1 SV=1	ELLTEFGYKGEETPVIVGSALCALEQR
>	sp|P85834|EFT	Elongation factor Tu, mitochondrial OS=Rattus norvegicus GN=Tufm PE=1 SV=1	LLDAVDTYIPVPTR
>	sp|P85834|EFT	Elongation factor Tu, mitochondrial OS=Rattus norvegicus GN=Tufm PE=1 SV=1	KGDECELLGHNK
>	sp|P85834|EFT	Elongation factor Tu, mitochondrial OS=Rattus norvegicus GN=Tufm PE=1 SV=1	KGDECELLGHNK
>	sp|P85834|EFT	Elongation factor Tu, mitochondrial OS=Rattus norvegicus GN=Tufm PE=1 SV=1	KGDECELLGHNK
>	sp|P62828|RAN	GTP-binding nuclear protein Ran OS=Rattus norvegicus GN=Ran PE=1 SV=3	KLIGDPNLEFVAMPALAPPEVVMDPALAAQYEHDLEVAQTTALPDEDDDL
>	sp|P62828|RAN	GTP-binding nuclear protein Ran OS=Rattus norvegicus GN=Ran PE=1 SV=3	LVLVGDGGTGK
>	sp|P62828|RAN	GTP-binding nuclear protein Ran OS=Rattus norvegicus GN=Ran PE=1 SV=3	KLIGDPNLEFVAMPALAPPEVVMDPALAAQYEHDLEVAQTTALPDEDDDL
>	sp|P62828|RAN	GTP-binding nuclear protein Ran OS=Rattus norvegicus GN=Ran PE=1 SV=3	KLIGDPNLEFVAMPALAPPEVVMDPALAAQYEHDLEVAQTTALPDEDDDL
>	sp|P10824|GN	Guanine nucleotide-binding protein G(i) subunit alpha-1 OS=Rattus norvegicus GN=Gnai1 PE=1 SV=3	NVQFVFDAVTDVIIK
>	sp|P10824|GN	Guanine nucleotide-binding protein G(i) subunit alpha-1 OS=Rattus norvegicus GN=Gnai1 PE=1 SV=3	LLLLGAGESGK
>	sp|P10824|GN	Guanine nucleotide-binding protein G(i) subunit alpha-1 OS=Rattus norvegicus GN=Gnai1 PE=1 SV=3	LLLLGAGESGK
>	sp|P10824|GN	Guanine nucleotide-binding protein G(i) subunit alpha-1 OS=Rattus norvegicus GN=Gnai1 PE=1 SV=3	LLLLGAGESGK
>	sp|P10824|GN	Guanine nucleotide-binding protein G(i) subunit alpha-1 OS=Rattus norvegicus GN=Gnai1 PE=1 SV=3	LLLLGAGESGK
>	sp|P10824|GN	Guanine nucleotide-binding protein G(i) subunit alpha-1 OS=Rattus norvegicus GN=Gnai1 PE=1 SV=3	TTGIVETHFTFK
>	sp|P10824|GN	Guanine nucleotide-binding protein G(i) subunit alpha-1 OS=Rattus norvegicus GN=Gnai1 PE=1 SV=3	LLLLGAGESGK
>	sp|P10824|GN	Guanine nucleotide-binding protein G(i) subunit alpha-1 OS=Rattus norvegicus GN=Gnai1 PE=1 SV=3	LKIDFGDAAR
>	sp|P10824|GN	Guanine nucleotide-binding protein G(i) subunit alpha-1 OS=Rattus norvegicus GN=Gnai1 PE=1 SV=3	IAQPNYIPTQQDVLR
>	sp|P10824|GN	Guanine nucleotide-binding protein G(i) subunit alpha-1 OS=Rattus norvegicus GN=Gnai1 PE=1 SV=3	LFDSICNNKWFTDTSIILFLNKKDLFEEK
>	sp|P10824|GN	Guanine nucleotide-binding protein G(i) subunit alpha-1 OS=Rattus norvegicus GN=Gnai1 PE=1 SV=3	AVVYSNTIQSIIAIIRAMGR
>	sp|Q62910|SYN	Synaptojanin-1 OS=Rattus norvegicus GN=Synj1 PE=1 SV=3	LAGIQEFQDKR
>	sp|Q62910|SYN	Synaptojanin-1 OS=Rattus norvegicus GN=Synj1 PE=1 SV=3	ERNEDFVEIAR
>	sp|Q62910|SYN	Synaptojanin-1 OS=Rattus norvegicus GN=Synj1 PE=1 SV=3	LHSVLKPQVQK
>	sp|Q71UE8|NE	NEDD8 OS=Rattus norvegicus GN=Nedd8 PE=1 SV=1	ILGGSVLHLVLALR
>	sp|Q71UE8|NE	NEDD8 OS=Rattus norvegicus GN=Nedd8 PE=1 SV=1	ILGGSVLHLVLALRGG
>	sp|Q71UE8|NE	NEDD8 OS=Rattus norvegicus GN=Nedd8 PE=1 SV=1	ILGGSVLHLVLALR
>	sp|Q71UE8|NE	NEDD8 OS=Rattus norvegicus GN=Nedd8 PE=1 SV=1	ILGGSVLHLVLALR
>	sp|Q71UE8|NE	NEDD8 OS=Rattus norvegicus GN=Nedd8 PE=1 SV=1	TLTGKEIEIDIEPTDKVER
>	sp|P31399|ATP	ATP synthase subunit d, mitochondrial OS=Rattus norvegicus GN=Atp5h PE=1 SV=3	IPVPEDKYTALVDAEEKEDVKNCAQFVTGSQAR
>	sp|P31399|ATP	ATP synthase subunit d, mitochondrial OS=Rattus norvegicus GN=Atp5h PE=1 SV=3	NMIPFDQMTIDDLNEVFPETK
>	sp|P31399|ATP	ATP synthase subunit d, mitochondrial OS=Rattus norvegicus GN=Atp5h PE=1 SV=3	AIGNALK
>	sp|P31399|ATP	ATP synthase subunit d, mitochondrial OS=Rattus norvegicus GN=Atp5h PE=1 SV=3	TIDWVSFVEIMPQNQK
>	sp|P31399|ATP	ATP synthase subunit d, mitochondrial OS=Rattus norvegicus GN=Atp5h PE=1 SV=3	TIDWVSFVEIMPQNQK
>	sp|P24268|CAT	Cathepsin D OS=Rattus norvegicus GN=Ctsd PE=1 SV=1	LLDIACWVHHK
>	sp|P24268|CAT	Cathepsin D OS=Rattus norvegicus GN=Ctsd PE=1 SV=1	VSSLPIITFK
>	sp|P24268|CAT	Cathepsin D OS=Rattus norvegicus GN=Ctsd PE=1 SV=1	LLDIACWVHHK
>	sp|P63170|DYL	Dynein light chain 1, cytoplasmic OS=Rattus norvegicus GN=Dynll1 PE=1 SV=1	NADMSEEMQQDSVECATQALEK
>	sp|P63170|DYL	Dynein light chain 1, cytoplasmic OS=Rattus norvegicus GN=Dynll1 PE=1 SV=1	DIAAHIK
>	sp|P63170|DYL	Dynein light chain 1, cytoplasmic OS=Rattus norvegicus GN=Dynll1 PE=1 SV=1	YNPTWHCIVGR
>	sp|P63170|DYL	Dynein light chain 1, cytoplasmic OS=Rattus norvegicus GN=Dynll1 PE=1 SV=1	DIAAHIKK
>	sp|P63170|DYL	Dynein light chain 1, cytoplasmic OS=Rattus norvegicus GN=Dynll1 PE=1 SV=1	NADMSEEMQQDSVECATQALEK
>	sp|P63170|DYL	Dynein light chain 1, cytoplasmic OS=Rattus norvegicus GN=Dynll1 PE=1 SV=1	YNPTWHCIVGR
>	sp|P47709|RP3	Rabphilin-3A OS=Rattus norvegicus GN=Rph3a PE=1 SV=1	TKPQQPAGEPATQEQPTPESR
>	sp|P47709|RP3	Rabphilin-3A OS=Rattus norvegicus GN=Rph3a PE=1 SV=1	RAPGQKPGPDLTSAPGR
>	sp|P47709|RP3	Rabphilin-3A OS=Rattus norvegicus GN=Rph3a PE=1 SV=1	LVDRLETMR
>	sp|P01830|THY	Thy-1 membrane glycoprotein OS=Rattus norvegicus GN=Thy1 PE=1 SV=1	VISLTACLVNQNLR
>	sp|P01830|THY	Thy-1 membrane glycoprotein OS=Rattus norvegicus GN=Thy1 PE=1 SV=1	VNLFSDR
>	sp|P01830|THY	Thy-1 membrane glycoprotein OS=Rattus norvegicus GN=Thy1 PE=1 SV=1	VNLFSDR
>	sp|P01830|THY	Thy-1 membrane glycoprotein OS=Rattus norvegicus GN=Thy1 PE=1 SV=1	VNLFSDR
>	sp|P01830|THY	Thy-1 membrane glycoprotein OS=Rattus norvegicus GN=Thy1 PE=1 SV=1	KHVLSGTLGVPEHTYR
>	sp|P01830|THY	Thy-1 membrane glycoprotein OS=Rattus norvegicus GN=Thy1 PE=1 SV=1	VNLFSDR
>	sp|Q62951|DP	Dihydropyrimidinase-related protein 4 (Fragment) OS=Rattus norvegicus GN=Dpysl4 PE=1 SV=1	TIDAHGLMVLPGGVDVHTR
>	sp|Q62951|DP	Dihydropyrimidinase-related protein 4 (Fragment) OS=Rattus norvegicus GN=Dpysl4 PE=1 SV=1	GLYDGPVHEVMLPAKPGSGTQAR

<table>
<tr><td>></td><td>sp|Q62951|DP</td><td>Dihydropyrimidinase-related protein 4 (Fragment) OS=Rattus norvegicus GN=Dpysl4 PE=1 SV=1</td><td>TIDAHGLMVLPGGVDVHTR</td></tr>
<tr><td>></td><td>sp|Q62951|DP</td><td>Dihydropyrimidinase-related protein 4 (Fragment) OS=Rattus norvegicus GN=Dpysl4 PE=1 SV=1</td><td>TIDAHGLMVLPGGVDVHTR</td></tr>
<tr><td>></td><td>sp|Q62951|DP</td><td>Dihydropyrimidinase-related protein 4 (Fragment) OS=Rattus norvegicus GN=Dpysl4 PE=1 SV=1</td><td>TIDAHGLMVLPGGVDVHTR</td></tr>
<tr><td>></td><td>sp|Q62951|DP</td><td>Dihydropyrimidinase-related protein 4 (Fragment) OS=Rattus norvegicus GN=Dpysl4 PE=1 SV=1</td><td>LAEIHGVPR</td></tr>
<tr><td>></td><td>sp|Q62951|DP</td><td>Dihydropyrimidinase-related protein 4 (Fragment) OS=Rattus norvegicus GN=Dpysl4 PE=1 SV=1</td><td>AAAFVTSPPINPDPTTADHLTSLLSSGDLQVTGSAHCTFTTAQKAVGK</td></tr>
<tr><td>></td><td>sp|Q62951|DP</td><td>Dihydropyrimidinase-related protein 4 (Fragment) OS=Rattus norvegicus GN=Dpysl4 PE=1 SV=1</td><td>GAADMVAQAK</td></tr>
<tr><td>></td><td>sp|Q62951|DP</td><td>Dihydropyrimidinase-related protein 4 (Fragment) OS=Rattus norvegicus GN=Dpysl4 PE=1 SV=1</td><td>TIDAHGLMVLPGGVDVHTR</td></tr>
<tr><td>></td><td>sp|Q6RUV5|RA</td><td>Ras-related C3 botulinum toxin substrate 1 OS=Rattus norvegicus GN=Rac1 PE=1 SV=1</td><td>TVFDEAIR</td></tr>
<tr><td>></td><td>sp|Q6RUV5|RA</td><td>Ras-related C3 botulinum toxin substrate 1 OS=Rattus norvegicus GN=Rac1 PE=1 SV=1</td><td>TVFDEAIR</td></tr>
<tr><td>></td><td>sp|Q6RUV5|RA</td><td>Ras-related C3 botulinum toxin substrate 1 OS=Rattus norvegicus GN=Rac1 PE=1 SV=1</td><td>CVVVGDGAVGK</td></tr>
<tr><td>></td><td>sp|Q6RUV5|RA</td><td>Ras-related C3 botulinum toxin substrate 1 OS=Rattus norvegicus GN=Rac1 PE=1 SV=1</td><td>AVLCPPPVK</td></tr>
<tr><td>></td><td>sp|P48768|NA</td><td>Sodium/calcium exchanger 2 OS=Rattus norvegicus GN=Slc8a2 PE=2 SV=1</td><td>KLTAEEEEAQR</td></tr>
<tr><td>></td><td>sp|P48768|NA</td><td>Sodium/calcium exchanger 2 OS=Rattus norvegicus GN=Slc8a2 PE=2 SV=1</td><td>QKHPDKDLEQLVGIAK</td></tr>
<tr><td>></td><td>sp|P48768|NA</td><td>Sodium/calcium exchanger 2 OS=Rattus norvegicus GN=Slc8a2 PE=2 SV=1</td><td>LVAPLLATVTILDDDHAGIFSFQDR</td></tr>
<tr><td>></td><td>sp|P48768|NA</td><td>Sodium/calcium exchanger 2 OS=Rattus norvegicus GN=Slc8a2 PE=2 SV=1</td><td>IGIIDDDIFEEDEHFFVR</td></tr>
<tr><td>></td><td>sp|P30427|PLE</td><td>Plectin OS=Rattus norvegicus GN=Plec PE=1 SV=2</td><td>LLEAQIATGGIIDPVHSHR</td></tr>
<tr><td>></td><td>sp|P30427|PLE</td><td>Plectin OS=Rattus norvegicus GN=Plec PE=1 SV=2</td><td>LLEAQIATGGIIDPVHSHR</td></tr>
<tr><td>></td><td>sp|P30427|PLE</td><td>Plectin OS=Rattus norvegicus GN=Plec PE=1 SV=2</td><td>LHVAILER</td></tr>
<tr><td>></td><td>sp|P30427|PLE</td><td>Plectin OS=Rattus norvegicus GN=Plec PE=1 SV=2</td><td>LHVAILER</td></tr>
<tr><td>></td><td>sp|P30427|PLE</td><td>Plectin OS=Rattus norvegicus GN=Plec PE=1 SV=2</td><td>LTQGHTTVAELTQR</td></tr>
<tr><td>></td><td>sp|P30427|PLE</td><td>Plectin OS=Rattus norvegicus GN=Plec PE=1 SV=2</td><td>EAIAELER</td></tr>
<tr><td>></td><td>sp|P30427|PLE</td><td>Plectin OS=Rattus norvegicus GN=Plec PE=1 SV=2</td><td>LTQGHTTVAELTQR</td></tr>
<tr><td>></td><td>sp|P30427|PLE</td><td>Plectin OS=Rattus norvegicus GN=Plec PE=1 SV=2</td><td>VTQLLER</td></tr>
<tr><td>></td><td>sp|P30427|PLE</td><td>Plectin OS=Rattus norvegicus GN=Plec PE=1 SV=2</td><td>LTVDEAVR</td></tr>
<tr><td>></td><td>sp|P30427|PLE</td><td>Plectin OS=Rattus norvegicus GN=Plec PE=1 SV=2</td><td>KGLVGPELHDR</td></tr>
<tr><td>></td><td>sp|P26772|CH</td><td>10 kDa heat shock protein, mitochondrial OS=Rattus norvegicus GN=Hspe1 PE=1 SV=3</td><td>VLQATVVAVGSGGK</td></tr>
<tr><td>></td><td>sp|P26772|CH</td><td>10 kDa heat shock protein, mitochondrial OS=Rattus norvegicus GN=Hspe1 PE=1 SV=3</td><td>VVLDDKDYFLFR</td></tr>
<tr><td>></td><td>sp|P26772|CH</td><td>10 kDa heat shock protein, mitochondrial OS=Rattus norvegicus GN=Hspe1 PE=1 SV=3</td><td>VLQATVVAVGSGGK</td></tr>
<tr><td>></td><td>sp|P26772|CH</td><td>10 kDa heat shock protein, mitochondrial OS=Rattus norvegicus GN=Hspe1 PE=1 SV=3</td><td>VLQATVVAVGSGGK</td></tr>
<tr><td>></td><td>sp|P26772|CH</td><td>10 kDa heat shock protein, mitochondrial OS=Rattus norvegicus GN=Hspe1 PE=1 SV=3</td><td>KFLPLFDR</td></tr>
<tr><td>></td><td>sp|P26772|CH</td><td>10 kDa heat shock protein, mitochondrial OS=Rattus norvegicus GN=Hspe1 PE=1 SV=3</td><td>FLPLFDR</td></tr>
<tr><td>></td><td>sp|P63086|MK</td><td>Mitogen-activated protein kinase 1 OS=Rattus norvegicus GN=Mapk1 PE=1 SV=3</td><td>VADPDHDHTGFLTEYVATR</td></tr>
<tr><td>></td><td>sp|P63086|MK</td><td>Mitogen-activated protein kinase 1 OS=Rattus norvegicus GN=Mapk1 PE=1 SV=3</td><td>VADPDHDHTGFLTEYVATR</td></tr>
<tr><td>></td><td>sp|P63086|MK</td><td>Mitogen-activated protein kinase 1 OS=Rattus norvegicus GN=Mapk1 PE=1 SV=3</td><td>LKELIFEETAR</td></tr>
<tr><td>></td><td>sp|P63086|MK</td><td>Mitogen-activated protein kinase 1 OS=Rattus norvegicus GN=Mapk1 PE=1 SV=3</td><td>DLKPSNLLLNTTCDLK</td></tr>
<tr><td>></td><td>sp|P63086|MK</td><td>Mitogen-activated protein kinase 1 OS=Rattus norvegicus GN=Mapk1 PE=1 SV=3</td><td>VADPDHDHTGFLTEYVATR</td></tr>
<tr><td>></td><td>sp|P62836|RA</td><td>Ras-related protein Rap-1A OS=Rattus norvegicus GN=Rap1a PE=1 SV=1</td><td>SKINVNEIFYDLVR</td></tr>
<tr><td>></td><td>sp|P62836|RA</td><td>Ras-related protein Rap-1A OS=Rattus norvegicus GN=Rap1a PE=1 SV=1</td><td>LVVLGSGGVGK</td></tr>
<tr><td>></td><td>sp|P62836|RA</td><td>Ras-related protein Rap-1A OS=Rattus norvegicus GN=Rap1a PE=1 SV=1</td><td>VKDTEDVPMILVGNK</td></tr>
<tr><td>></td><td>sp|P56574|IDH</td><td>Isocitrate dehydrogenase [NADP], mitochondrial OS=Rattus norvegicus GN=Idh2 PE=1 SV=2</td><td>NILGGTVFREPIICK</td></tr>
<tr><td>></td><td>sp|P56574|IDH</td><td>Isocitrate dehydrogenase [NADP], mitochondrial OS=Rattus norvegicus GN=Idh2 PE=1 SV=2</td><td>LNEHFLNTTDFLDTIK</td></tr>
<tr><td>></td><td>sp|P56574|IDH</td><td>Isocitrate dehydrogenase [NADP], mitochondrial OS=Rattus norvegicus GN=Idh2 PE=1 SV=2</td><td>LNEHFLNTTDFLDTIK</td></tr>
<tr><td>></td><td>sp|P56574|IDH</td><td>Isocitrate dehydrogenase [NADP], mitochondrial OS=Rattus norvegicus GN=Idh2 PE=1 SV=2</td><td>LIDDMVAQVLK</td></tr>
<tr><td>></td><td>sp|P56574|IDH</td><td>Isocitrate dehydrogenase [NADP], mitochondrial OS=Rattus norvegicus GN=Idh2 PE=1 SV=2</td><td>NILGGTVFREPIICK</td></tr>
<tr><td>></td><td>sp|P56574|IDH</td><td>Isocitrate dehydrogenase [NADP], mitochondrial OS=Rattus norvegicus GN=Idh2 PE=1 SV=2</td><td>LNEHFLNTTDFLDTIK</td></tr>
<tr><td>></td><td>sp|Q2TA68|OP</td><td>Dynamin-like 120 kDa protein, mitochondrial OS=Rattus norvegicus GN=Opa1 PE=1 SV=1</td><td>HWEEILQQSLWER</td></tr>
<tr><td>></td><td>sp|Q2TA68|OP</td><td>Dynamin-like 120 kDa protein, mitochondrial OS=Rattus norvegicus GN=Opa1 PE=1 SV=1</td><td>LDAFIEALHQEK</td></tr>
<tr><td>></td><td>sp|Q2TA68|OP</td><td>Dynamin-like 120 kDa protein, mitochondrial OS=Rattus norvegicus GN=Opa1 PE=1 SV=1</td><td>HWEEILQQSLWER</td></tr>
<tr><td>></td><td>sp|Q2TA68|OP</td><td>Dynamin-like 120 kDa protein, mitochondrial OS=Rattus norvegicus GN=Opa1 PE=1 SV=1</td><td>HWEEILQQSLWER</td></tr>
<tr><td>></td><td>sp|Q2TA68|OP</td><td>Dynamin-like 120 kDa protein, mitochondrial OS=Rattus norvegicus GN=Opa1 PE=1 SV=1</td><td>LDAFIEALHQEK</td></tr>
<tr><td>></td><td>sp|Q2TA68|OP</td><td>Dynamin-like 120 kDa protein, mitochondrial OS=Rattus norvegicus GN=Opa1 PE=1 SV=1</td><td>IQRMLAITANTLR</td></tr>
<tr><td>></td><td>sp|Q2TA68|OP</td><td>Dynamin-like 120 kDa protein, mitochondrial OS=Rattus norvegicus GN=Opa1 PE=1 SV=1</td><td>LAARLLK</td></tr>
<tr><td>></td><td>sp|P62963|PR</td><td>Profilin-1 OS=Rattus norvegicus GN=Pfn1 PE=1 SV=2</td><td>TFVSITPAEVGVLVGK</td></tr>
<tr><td>></td><td>sp|P62963|PR</td><td>Profilin-1 OS=Rattus norvegicus GN=Pfn1 PE=1 SV=2</td><td>TFVSITPAEVGVLVGKDR</td></tr>
<tr><td>></td><td>sp|P62963|PR</td><td>Profilin-1 OS=Rattus norvegicus GN=Pfn1 PE=1 SV=2</td><td>TFVSITPAEVGVLVGK</td></tr>
<tr><td>></td><td>sp|P62963|PR</td><td>Profilin-1 OS=Rattus norvegicus GN=Pfn1 PE=1 SV=2</td><td>TFVSITPAEVGVLVGK</td></tr>
<tr><td>></td><td>sp|P62963|PR</td><td>Profilin-1 OS=Rattus norvegicus GN=Pfn1 PE=1 SV=2</td><td>TFVSITPAEVGVLVGKDR</td></tr>
<tr><td>></td><td>sp|P62963|PR</td><td>Profilin-1 OS=Rattus norvegicus GN=Pfn1 PE=1 SV=2</td><td>TLVLLMGK</td></tr>
<tr><td>></td><td>sp|P62963|PR</td><td>Profilin-1 OS=Rattus norvegicus GN=Pfn1 PE=1 SV=2</td><td>TLVLLMGK</td></tr>
<tr><td>></td><td>sp|P62963|PR</td><td>Profilin-1 OS=Rattus norvegicus GN=Pfn1 PE=1 SV=2</td><td>TFVSITPAEVGVLVGKDR</td></tr>
<tr><td>></td><td>sp|P62963|PR</td><td>Profilin-1 OS=Rattus norvegicus GN=Pfn1 PE=1 SV=2</td><td>TFVSITPAEVGVLVGKDR</td></tr>
<tr><td>></td><td>sp|P02262|H2</td><td>Histone H2A type 1 OS=Rattus norvegicus PE=1 SV=2</td><td>VTIAQGGVLPNIQAVLLPK</td></tr>
<tr><td>></td><td>sp|P02262|H2</td><td>Histone H2A type 1 OS=Rattus norvegicus PE=1 SV=2</td><td>VTIAQGGVLPNIQAVLLPK</td></tr>
<tr><td>></td><td>sp|P02262|H2</td><td>Histone H2A type 1 OS=Rattus norvegicus PE=1 SV=2</td><td>VTIAQGGVLPNIQAVLLPK</td></tr>
<tr><td>></td><td>sp|P02262|H2</td><td>Histone H2A type 1 OS=Rattus norvegicus PE=1 SV=2</td><td>VTIAQGGVLPNIQAVLLPK</td></tr>
<tr><td>></td><td>sp|P02262|H2</td><td>Histone H2A type 1 OS=Rattus norvegicus PE=1 SV=2</td><td>AGLQFPVGR</td></tr>
<tr><td>></td><td>sp|P02262|H2</td><td>Histone H2A type 1 OS=Rattus norvegicus PE=1 SV=2</td><td>AGLQFPVGR</td></tr>
<tr><td>></td><td>sp|P02262|H2</td><td>Histone H2A type 1 OS=Rattus norvegicus PE=1 SV=2</td><td>AGLQFPVGR</td></tr>
<tr><td>></td><td>sp|P35281|RA</td><td>Ras-related protein Rab-10 OS=Rattus norvegicus GN=Rab10 PE=2 SV=1</td><td>NIDQHANEDVER</td></tr>
<tr><td>></td><td>sp|P35281|RA</td><td>Ras-related protein Rab-10 OS=Rattus norvegicus GN=Rab10 PE=2 SV=1</td><td>LLLIGDSGVGK</td></tr>
<tr><td>></td><td>sp|P35281|RA</td><td>Ras-related protein Rab-10 OS=Rattus norvegicus GN=Rab10 PE=2 SV=1</td><td>LQIWDTAGQER</td></tr>
<tr><td>></td><td>sp|P35281|RA</td><td>Ras-related protein Rab-10 OS=Rattus norvegicus GN=Rab10 PE=2 SV=1</td><td>LQIWDTAGQER</td></tr>
<tr><td>></td><td>sp|P35281|RA</td><td>Ras-related protein Rab-10 OS=Rattus norvegicus GN=Rab10 PE=2 SV=1</td><td>NIDQHANEDVER</td></tr>
<tr><td>></td><td>sp|P35281|RA</td><td>Ras-related protein Rab-10 OS=Rattus norvegicus GN=Rab10 PE=2 SV=1</td><td>NIDQHANEDVER</td></tr>
</table>

>	sp|P35281|RAE	Ras-related protein Rab-10 OS=Rattus norvegicus GN=Rab10 PE=2 SV=1	NIDQHANEDVER
>	sp|P35281|RAE	Ras-related protein Rab-10 OS=Rattus norvegicus GN=Rab10 PE=2 SV=1	NIDQHANEDVER
>	sp|P35281|RAE	Ras-related protein Rab-10 OS=Rattus norvegicus GN=Rab10 PE=2 SV=1	LQIWDTAGQER
>	sp|P35281|RAE	Ras-related protein Rab-10 OS=Rattus norvegicus GN=Rab10 PE=2 SV=1	NIDQHANEDVER
>	sp|P35281|RAE	Ras-related protein Rab-10 OS=Rattus norvegicus GN=Rab10 PE=2 SV=1	NIDQHANEDVER
>	sp|P35281|RAE	Ras-related protein Rab-10 OS=Rattus norvegicus GN=Rab10 PE=2 SV=1	LQIWDTAGQER
>	sp|Q5U318|PE	Astrocytic phosphoprotein PEA-15 OS=Rattus norvegicus GN=Pea15 PE=1 SV=1	RPDLLTMVVDYR
>	sp|Q5U318|PE	Astrocytic phosphoprotein PEA-15 OS=Rattus norvegicus GN=Pea15 PE=1 SV=1	RPDLLTMVVDYR
>	sp|Q5U318|PE	Astrocytic phosphoprotein PEA-15 OS=Rattus norvegicus GN=Pea15 PE=1 SV=1	RPDLLTMVVDYR
>	sp|Q5U318|PE	Astrocytic phosphoprotein PEA-15 OS=Rattus norvegicus GN=Pea15 PE=1 SV=1	RPDLLTMVVDYR
>	sp|Q5U318|PE	Astrocytic phosphoprotein PEA-15 OS=Rattus norvegicus GN=Pea15 PE=1 SV=1	RPDLLTMVVDYR
>	sp|Q5U318|PE	Astrocytic phosphoprotein PEA-15 OS=Rattus norvegicus GN=Pea15 PE=1 SV=1	RPDLLTMVVDYR
>	sp|Q5U318|PE	Astrocytic phosphoprotein PEA-15 OS=Rattus norvegicus GN=Pea15 PE=1 SV=1	RPDLLTMVVDYR
>	sp|Q5U318|PE	Astrocytic phosphoprotein PEA-15 OS=Rattus norvegicus GN=Pea15 PE=1 SV=1	ISEEDELDTK
>	sp|Q5U318|PE	Astrocytic phosphoprotein PEA-15 OS=Rattus norvegicus GN=Pea15 PE=1 SV=1	RPDLLTMVVDYR
>	sp|P03994|HPL	Hyaluronan and proteoglycan link protein 1 OS=Rattus norvegicus GN=Hapln1 PE=1 SV=2	VGQIFAAWK
>	sp|P03994|HPL	Hyaluronan and proteoglycan link protein 1 OS=Rattus norvegicus GN=Hapln1 PE=1 SV=2	LLVEAEQAK
>	sp|P13668|STN	Stathmin OS=Rattus norvegicus GN=Stmn1 PE=1 SV=2	NKESKDPADETEAD
>	sp|P13668|STN	Stathmin OS=Rattus norvegicus GN=Stmn1 PE=1 SV=2	ASGQAFELILSPR
>	sp|P13668|STN	Stathmin OS=Rattus norvegicus GN=Stmn1 PE=1 SV=2	NKESKDPADETEAD
>	sp|P13668|STN	Stathmin OS=Rattus norvegicus GN=Stmn1 PE=1 SV=2	EHEKEVLQK
>	sp|P13668|STN	Stathmin OS=Rattus norvegicus GN=Stmn1 PE=1 SV=2	KLEAAEER
>	sp|P13668|STN	Stathmin OS=Rattus norvegicus GN=Stmn1 PE=1 SV=2	NKESKDPADETEAD
>	sp|P13668|STN	Stathmin OS=Rattus norvegicus GN=Stmn1 PE=1 SV=2	KNKESKDPADETEAD
>	sp|P13668|STN	Stathmin OS=Rattus norvegicus GN=Stmn1 PE=1 SV=2	SKESVPEFPLSPPKKK
>	sp|P13668|STN	Stathmin OS=Rattus norvegicus GN=Stmn1 PE=1 SV=2	KNKESKDPADETEAD
>	sp|Q07266|DRI	Drebrin OS=Rattus norvegicus GN=Dbn1 PE=2 SV=3	KSESEVEEAAAIIAQRPDNPR
>	sp|Q07266|DRI	Drebrin OS=Rattus norvegicus GN=Dbn1 PE=2 SV=3	KSESEVEEAAAIIAQRPDNPR
>	sp|Q07266|DRI	Drebrin OS=Rattus norvegicus GN=Dbn1 PE=2 SV=3	KSESEVEEAAAIIAQRPDNPR
>	sp|Q07266|DRI	Drebrin OS=Rattus norvegicus GN=Dbn1 PE=2 SV=3	LREDENAEPVGTTYQK
>	sp|Q07266|DRI	Drebrin OS=Rattus norvegicus GN=Dbn1 PE=2 SV=3	LKDQSIFGDQRDEEEESQMK
>	sp|Q6J4I0|PPR	Protein phosphatase 1 regulatory subunit 1B OS=Rattus norvegicus GN=Ppp1r1b PE=2 SV=1	RPTPALLFR
>	sp|Q6J4I0|PPR	Protein phosphatase 1 regulatory subunit 1B OS=Rattus norvegicus GN=Ppp1r1b PE=2 SV=1	IAESHLQTISNLSENQASEEEDELGELR
>	sp|P84245|H33	Histone H3.3 OS=Rattus norvegicus GN=H3f3b PE=1 SV=2	FQSAAIGALQEASEAYLVGLFEDTNLCAIHAK
>	sp|P84245|H33	Histone H3.3 OS=Rattus norvegicus GN=H3f3b PE=1 SV=2	FQSAAIGALQEASEAYLVGLFEDTNLCAIHAK
>	sp|P84245|H33	Histone H3.3 OS=Rattus norvegicus GN=H3f3b PE=1 SV=2	FQSAAIGALQEASEAYLVGLFEDTNLCAIHAK
>	sp|P84245|H33	Histone H3.3 OS=Rattus norvegicus GN=H3f3b PE=1 SV=2	FQSAAIGALQEASEAYLVGLFEDTNLCAIHAK
>	sp|P84245|H33	Histone H3.3 OS=Rattus norvegicus GN=H3f3b PE=1 SV=2	FQSAAIGALQEASEAYLVGLFEDTNLCAIHAK
>	sp|P84245|H33	Histone H3.3 OS=Rattus norvegicus GN=H3f3b PE=1 SV=2	EIAQDFK
>	sp|P84245|H33	Histone H3.3 OS=Rattus norvegicus GN=H3f3b PE=1 SV=2	YRPGTVALR
>	sp|P84245|H33	Histone H3.3 OS=Rattus norvegicus GN=H3f3b PE=1 SV=2	EIAQDFK
>	sp|P61980|HNI	Heterogeneous nuclear ribonucleoprotein K OS=Rattus norvegicus GN=Hnrnpk PE=1 SV=1	ILSISADIETIGEILKK
>	sp|P61980|HNI	Heterogeneous nuclear ribonucleoprotein K OS=Rattus norvegicus GN=Hnrnpk PE=1 SV=1	RPAEDMEEEQAFKR
>	sp|P61980|HNI	Heterogeneous nuclear ribonucleoprotein K OS=Rattus norvegicus GN=Hnrnpk PE=1 SV=1	ILSISADIETIGEILKK
>	sp|P61980|HNI	Heterogeneous nuclear ribonucleoprotein K OS=Rattus norvegicus GN=Hnrnpk PE=1 SV=1	ILSISADIETIGEILKK
>	sp|P61980|HNI	Heterogeneous nuclear ribonucleoprotein K OS=Rattus norvegicus GN=Hnrnpk PE=1 SV=1	LLIHQSLAGGIIGVK
>	sp|Q62813|LSA	Limbic system-associated membrane protein OS=Rattus norvegicus GN=Lsamp PE=1 SV=1	EFEGEEEYLEILGITR
>	sp|Q62813|LSA	Limbic system-associated membrane protein OS=Rattus norvegicus GN=Lsamp PE=1 SV=1	AANEVSSADVK
>	sp|Q62813|LSA	Limbic system-associated membrane protein OS=Rattus norvegicus GN=Lsamp PE=1 SV=1	EFEGEEEYLEILGITR
>	sp|Q07647|GTI	Solute carrier family 2, facilitated glucose transporter member 3 OS=Rattus norvegicus GN=Slc2a3 PE=1 SV=1	KEEDQATEILQR
>	sp|Q07647|GTI	Solute carrier family 2, facilitated glucose transporter member 3 OS=Rattus norvegicus GN=Slc2a3 PE=1 SV=1	LWGTPDVIQEIQEMKDESIR
>	sp|Q07647|GTI	Solute carrier family 2, facilitated glucose transporter member 3 OS=Rattus norvegicus GN=Slc2a3 PE=1 SV=1	LWGTPDVIQEIQEMKDESIR
>	sp|P63322|RAL	Ras-related protein Ral-A OS=Rattus norvegicus GN=Rala PE=1 SV=1	VKEDENVPFLLVGNK
>	sp|P63322|RAL	Ras-related protein Ral-A OS=Rattus norvegicus GN=Rala PE=1 SV=1	VKEDENVPFLLVGNK
>	sp|P63322|RAL	Ras-related protein Ral-A OS=Rattus norvegicus GN=Rala PE=1 SV=1	VKEDENVPFLLVGNK
>	sp|P63322|RAL	Ras-related protein Ral-A OS=Rattus norvegicus GN=Rala PE=1 SV=1	VKEDENVPFLLVGNK
>	sp|P63322|RAL	Ras-related protein Ral-A OS=Rattus norvegicus GN=Rala PE=1 SV=1	ANVDKVFFDLMR
>	sp|P63322|RAL	Ras-related protein Ral-A OS=Rattus norvegicus GN=Rala PE=1 SV=1	VKEDENVPFLLVGNK
>	sp|P63322|RAL	Ras-related protein Ral-A OS=Rattus norvegicus GN=Rala PE=1 SV=1	ANVDKVFFDLMR
>	sp|Q7M0E3|DE	Destrin OS=Rattus norvegicus GN=Dstn PE=1 SV=3	KEELMFFLWAPEQAPLK
>	sp|Q7M0E3|DE	Destrin OS=Rattus norvegicus GN=Dstn PE=1 SV=3	KEELMFFLWAPEQAPLK
>	sp|Q7M0E3|DE	Destrin OS=Rattus norvegicus GN=Dstn PE=1 SV=3	KEELMFFLWAPEQAPLK
>	sp|Q7M0E3|DE	Destrin OS=Rattus norvegicus GN=Dstn PE=1 SV=3	KEELMFFLWAPEQAPLK
>	sp|Q7M0E3|DE	Destrin OS=Rattus norvegicus GN=Dstn PE=1 SV=3	KEELMFFLWAPEQAPLK
>	sp|Q7M0E3|DE	Destrin OS=Rattus norvegicus GN=Dstn PE=1 SV=3	KEELMFFLWAPEQAPLK
>	sp|Q7M0E3|DE	Destrin OS=Rattus norvegicus GN=Dstn PE=1 SV=3	KEELMFFLWAPEQAPLK
>	sp|Q7M0E3|DE	Destrin OS=Rattus norvegicus GN=Dstn PE=1 SV=3	KEELMFFLWAPEQAPLK
>	sp|Q7M0E3|DE	Destrin OS=Rattus norvegicus GN=Dstn PE=1 SV=3	HEYQANGPEDLNR
>	sp|Q7M0E3|DE	Destrin OS=Rattus norvegicus GN=Dstn PE=1 SV=3	KEELMFFLWAPEQAPLK
>	sp|Q7M0E3|DE	Destrin OS=Rattus norvegicus GN=Dstn PE=1 SV=3	AVIFCLSADKK
>	sp|Q7M0E3|DE	Destrin OS=Rattus norvegicus GN=Dstn PE=1 SV=3	HEYQANGPEDLNR
>	sp|Q7M0E3|DE	Destrin OS=Rattus norvegicus GN=Dstn PE=1 SV=3	KEELMFFLWAPEQAPLKSK
>	sp|Q63716|PRI	Peroxiredoxin-1 OS=Rattus norvegicus GN=Prdx1 PE=1 SV=1	LVQAFQFTDK
>	sp|Q63716|PRI	Peroxiredoxin-1 OS=Rattus norvegicus GN=Prdx1 PE=1 SV=1	GLFIIDDK

>	sp|Q63716|PRI	Peroxiredoxin-1 OS=Rattus norvegicus GN=Prdx1 PE=1 SV=1	GLFIIDDK
>	sp|Q63716|PRI	Peroxiredoxin-1 OS=Rattus norvegicus GN=Prdx1 PE=1 SV=1	KQGGLGPMNIPLVSDPKR
>	sp|Q63716|PRI	Peroxiredoxin-1 OS=Rattus norvegicus GN=Prdx1 PE=1 SV=1	GLFIIDDK
>	sp|Q63716|PRI	Peroxiredoxin-1 OS=Rattus norvegicus GN=Prdx1 PE=1 SV=1	HGEVCPAGWKPGSDTIKPDVNK
>	sp|Q63716|PRI	Peroxiredoxin-1 OS=Rattus norvegicus GN=Prdx1 PE=1 SV=1	LVQAFQFTDK
>	sp|Q63716|PRI	Peroxiredoxin-1 OS=Rattus norvegicus GN=Prdx1 PE=1 SV=1	GLFIIDDK
>	sp|O35763|MC	Moesin OS=Rattus norvegicus GN=Msn PE=1 SV=3	RKPDTIEVQQMK
>	sp|O35763|MC	Moesin OS=Rattus norvegicus GN=Msn PE=1 SV=3	LFFLQVK
>	sp|P50878|RL4	60S ribosomal protein L4 OS=Rattus norvegicus GN=Rpl4 PE=1 SV=3	KLEAAAAALAAK
>	sp|P50878|RL4	60S ribosomal protein L4 OS=Rattus norvegicus GN=Rpl4 PE=1 SV=3	KLEAAAAALAAK
>	sp|P50878|RL4	60S ribosomal protein L4 OS=Rattus norvegicus GN=Rpl4 PE=1 SV=3	APIRPDIVNFVHTNLR
>	sp|P28480|TCP	T-complex protein 1 subunit alpha OS=Rattus norvegicus GN=Tcp1 PE=1 SV=1	WIGLDLVHGKPR
>	sp|P28480|TCP	T-complex protein 1 subunit alpha OS=Rattus norvegicus GN=Tcp1 PE=1 SV=1	WIGLDLVHGKPR
>	sp|P28480|TCP	T-complex protein 1 subunit alpha OS=Rattus norvegicus GN=Tcp1 PE=1 SV=1	WIGLDLVHGKPR
>	sp|P28480|TCP	T-complex protein 1 subunit alpha OS=Rattus norvegicus GN=Tcp1 PE=1 SV=1	LLEVEHPAAK
>	sp|P36876|2AE	Serine/threonine-protein phosphatase 2A 55 kDa regulatory subunit B alpha isoform OS=Rattus norvegicus GN=Ppp2r2a PE=2 SV=1	INLWHLEITDR
>	sp|P36876|2AE	Serine/threonine-protein phosphatase 2A 55 kDa regulatory subunit B alpha isoform OS=Rattus norvegicus GN=Ppp2r2a PE=2 SV=1	ILHTAWHPK
>	sp|P36876|2AE	Serine/threonine-protein phosphatase 2A 55 kDa regulatory subunit B alpha isoform OS=Rattus norvegicus GN=Ppp2r2a PE=2 SV=1	INLWHLEITDR
>	sp|P36876|2AE	Serine/threonine-protein phosphatase 2A 55 kDa regulatory subunit B alpha isoform OS=Rattus norvegicus GN=Ppp2r2a PE=2 SV=1	INLWHLEITDR
>	sp|Q1WIM2|CA	Cell adhesion molecule 2 OS=Rattus norvegicus GN=Cadm2 PE=1 SV=2	VDRSDDGVAVICR
>	sp|Q1WIM2|CA	Cell adhesion molecule 2 OS=Rattus norvegicus GN=Cadm2 PE=1 SV=2	AYLTVLGVPEKPQISGFSSPVMEGDLMQLTCK
>	sp|Q1WIM2|CA	Cell adhesion molecule 2 OS=Rattus norvegicus GN=Cadm2 PE=1 SV=2	AYLTVLGVPEKPQISGFSSPVMEGDLMQLTCK
>	sp|Q1WIM2|CA	Cell adhesion molecule 2 OS=Rattus norvegicus GN=Cadm2 PE=1 SV=2	AYLTVLGVPEKPQISGFSSPVMEGDLMQLTCK
>	sp|Q1WIM2|CA	Cell adhesion molecule 2 OS=Rattus norvegicus GN=Cadm2 PE=1 SV=2	IIPSTPFPQEGQALTLTCESK
>	sp|Q05764|AD	Beta-adducin OS=Rattus norvegicus GN=Add2 PE=2 SV=4	LGEHEFEALMR
>	sp|Q05764|AD	Beta-adducin OS=Rattus norvegicus GN=Add2 PE=2 SV=4	LGEHEFEALMR
>	sp|Q05764|AD	Beta-adducin OS=Rattus norvegicus GN=Add2 PE=2 SV=4	LGEHEFEALMR
>	sp|Q05764|AD	Beta-adducin OS=Rattus norvegicus GN=Add2 PE=2 SV=4	LGEHEFEALMR
>	sp|Q05764|AD	Beta-adducin OS=Rattus norvegicus GN=Add2 PE=2 SV=4	LGEHEFEALMR
>	sp|Q05764|AD	Beta-adducin OS=Rattus norvegicus GN=Add2 PE=2 SV=4	VNILGEVVEK
>	sp|Q05764|AD	Beta-adducin OS=Rattus norvegicus GN=Add2 PE=2 SV=4	LGEHEFEALMR
>	sp|Q05764|AD	Beta-adducin OS=Rattus norvegicus GN=Add2 PE=2 SV=4	LGEHEFEALMR
>	sp|Q05695|L1C	Neural cell adhesion molecule L1 OS=Rattus norvegicus GN=L1cam PE=2 SV=3	VLLHHLAVK
>	sp|Q05695|L1C	Neural cell adhesion molecule L1 OS=Rattus norvegicus GN=L1cam PE=2 SV=3	VLLHHLAVK
>	sp|Q05695|L1C	Neural cell adhesion molecule L1 OS=Rattus norvegicus GN=L1cam PE=2 SV=3	FHILFK
>	sp|Q05695|L1C	Neural cell adhesion molecule L1 OS=Rattus norvegicus GN=L1cam PE=2 SV=3	FHILFK
>	sp|Q9Z2F5|CTE	C-terminal-binding protein 1 OS=Rattus norvegicus GN=Ctbp1 PE=1 SV=3	IRGETLGIIGLGR
>	sp|Q9Z2F5|CTE	C-terminal-binding protein 1 OS=Rattus norvegicus GN=Ctbp1 PE=1 SV=3	VLNEAVGALMYHTITLTR
>	sp|Q9Z2F5|CTE	C-terminal-binding protein 1 OS=Rattus norvegicus GN=Ctbp1 PE=1 SV=3	VLNEAVGALMYHTITLTREDLEK
>	sp|Q9Z2F5|CTE	C-terminal-binding protein 1 OS=Rattus norvegicus GN=Ctbp1 PE=1 SV=3	VLNEAVGALMYHTITLTR
>	sp|Q9Z2F5|CTE	C-terminal-binding protein 1 OS=Rattus norvegicus GN=Ctbp1 PE=1 SV=3	VLNEAVGALMYHTITLTR
>	sp|P60892|PRF	Ribose-phosphate pyrophosphokinase 1 OS=Rattus norvegicus GN=Prps1 PE=1 SV=2	LNVDFALIHK
>	sp|P60892|PRF	Ribose-phosphate pyrophosphokinase 1 OS=Rattus norvegicus GN=Prps1 PE=1 SV=2	VAILVDDMADTCGTICHAADK
>	sp|P50137|TKT	Transketolase OS=Rattus norvegicus GN=Tkt PE=1 SV=1	SKDDQVTVIGAGVTLHEALAAAEMLK
>	sp|P50137|TKT	Transketolase OS=Rattus norvegicus GN=Tkt PE=1 SV=1	MFGIDKDAIVQAVK
>	sp|P50137|TKT	Transketolase OS=Rattus norvegicus GN=Tkt PE=1 SV=1	AVELAANTK
>	sp|P50137|TKT	Transketolase OS=Rattus norvegicus GN=Tkt PE=1 SV=1	ILATPPQEDAPSVDIANIR
>	sp|P0C5X8|TTY	Protein tweety homolog 1 OS=Rattus norvegicus GN=Ttyh1 PE=2 SV=1	SFHQLVALLHCR
>	sp|P0C5X8|TTY	Protein tweety homolog 1 OS=Rattus norvegicus GN=Ttyh1 PE=2 SV=1	ALASIHSQLQGLER
>	sp|P0C5X8|TTY	Protein tweety homolog 1 OS=Rattus norvegicus GN=Ttyh1 PE=2 SV=1	SFHQLVALLHCR
>	sp|P84076|HPC	Neuron-specific calcium-binding protein hippocalcin OS=Rattus norvegicus GN=Hpca PE=1 SV=2	LRPEMLQDLR
>	sp|P84076|HPC	Neuron-specific calcium-binding protein hippocalcin OS=Rattus norvegicus GN=Hpca PE=1 SV=2	LRPEMLQDLR
>	sp|P84076|HPC	Neuron-specific calcium-binding protein hippocalcin OS=Rattus norvegicus GN=Hpca PE=1 SV=2	LRPEMLQDLR
>	sp|P84076|HPC	Neuron-specific calcium-binding protein hippocalcin OS=Rattus norvegicus GN=Hpca PE=1 SV=2	LRPEMLQDLR
>	sp|P84076|HPC	Neuron-specific calcium-binding protein hippocalcin OS=Rattus norvegicus GN=Hpca PE=1 SV=2	LRPEMLQDLR
>	sp|P84076|HPC	Neuron-specific calcium-binding protein hippocalcin OS=Rattus norvegicus GN=Hpca PE=1 SV=2	LRPEMLQDLR
>	sp|P84076|HPC	Neuron-specific calcium-binding protein hippocalcin OS=Rattus norvegicus GN=Hpca PE=1 SV=2	FAEHVFR
>	sp|P10888|COX	Cytochrome c oxidase subunit 4 isoform 1, mitochondrial OS=Rattus norvegicus GN=Cox4i1 PE=1 SV=1	ADWSSLSR
>	sp|P10888|COX	Cytochrome c oxidase subunit 4 isoform 1, mitochondrial OS=Rattus norvegicus GN=Cox4i1 PE=1 SV=1	VNPIQGFSAK
>	sp|P10888|COX	Cytochrome c oxidase subunit 4 isoform 1, mitochondrial OS=Rattus norvegicus GN=Cox4i1 PE=1 SV=1	DYPLPDVAHVK
>	sp|P10888|COX	Cytochrome c oxidase subunit 4 isoform 1, mitochondrial OS=Rattus norvegicus GN=Cox4i1 PE=1 SV=1	DYPLPDVAHVK
>	sp|Q8CFN2|CD	Cell division control protein 42 homolog OS=Rattus norvegicus GN=Cdc42 PE=1 SV=2	NVFDEAILAALEPPEPK
>	sp|Q8CFN2|CD	Cell division control protein 42 homolog OS=Rattus norvegicus GN=Cdc42 PE=1 SV=2	LRPLSYPQTDVFLVCFSVVSPSSFENVKEK
>	sp|Q8CFN2|CD	Cell division control protein 42 homolog OS=Rattus norvegicus GN=Cdc42 PE=1 SV=2	LRPLSYPQTDVFLVCFSVVSPSSFENVKEK
>	sp|Q8CFN2|CD	Cell division control protein 42 homolog OS=Rattus norvegicus GN=Cdc42 PE=1 SV=2	LRPLSYPQTDVFLVCFSVVSPSSFENVKEK
>	sp|Q8CFN2|CD	Cell division control protein 42 homolog OS=Rattus norvegicus GN=Cdc42 PE=1 SV=2	NVFDEAILAALEPPEPK
>	sp|Q8CFN2|CD	Cell division control protein 42 homolog OS=Rattus norvegicus GN=Cdc42 PE=1 SV=2	LRPLSYPQTDVFLVCFSVVSPSSFENVKEK
>	sp|Q8CFN2|CD	Cell division control protein 42 homolog OS=Rattus norvegicus GN=Cdc42 PE=1 SV=2	NVFDEAILAALEPPEPK
>	sp|Q8CFN2|CD	Cell division control protein 42 homolog OS=Rattus norvegicus GN=Cdc42 PE=1 SV=2	CVVVGDGAVGK
>	sp|P00406|COX	Cytochrome c oxidase subunit 2 OS=Rattus norvegicus GN=Mtco2 PE=2 SV=2	VVLPMELPIR
>	sp|P00406|COX	Cytochrome c oxidase subunit 2 OS=Rattus norvegicus GN=Mtco2 PE=2 SV=2	VVLPMELPIR
>	sp|P00406|COX	Cytochrome c oxidase subunit 2 OS=Rattus norvegicus GN=Mtco2 PE=2 SV=2	VVLPMELPIR
>	sp|P00406|COX	Cytochrome c oxidase subunit 2 OS=Rattus norvegicus GN=Mtco2 PE=2 SV=2	MLISSEDVLHSWAIPSLGLK
>	sp|P48037|ANX	Annexin A6 OS=Rattus norvegicus GN=Anxa6 PE=1 SV=2	GFGSDKESILELITSR

>	sp|P48037|ANX	Annexin A6 OS=Rattus norvegicus GN=Anxa6 PE=1 SV=2	GFGSDKESILELITSR
>	sp|P48037|ANX	Annexin A6 OS=Rattus norvegicus GN=Anxa6 PE=1 SV=2	SEIDLLNIR
>	sp|P48037|ANX	Annexin A6 OS=Rattus norvegicus GN=Anxa6 PE=1 SV=2	DLMADLKSEISGDLAR
>	sp|P48037|ANX	Annexin A6 OS=Rattus norvegicus GN=Anxa6 PE=1 SV=2	GFGSDKESILELITSR
>	sp|Q6AY09|HN	Heterogeneous nuclear ribonucleoprotein H2 OS=Rattus norvegicus GN=Hnrnph2 PE=1 SV=1	VHIEIGPDGR
>	sp|Q6AY09|HN	Heterogeneous nuclear ribonucleoprotein H2 OS=Rattus norvegicus GN=Hnrnph2 PE=1 SV=1	DKANMQHR
>	sp|Q03344|ATI	ATPase inhibitor, mitochondrial OS=Rattus norvegicus GN=Atpif1 PE=2 SV=2	EQLAALK
>	sp|Q03344|ATI	ATPase inhibitor, mitochondrial OS=Rattus norvegicus GN=Atpif1 PE=2 SV=2	EAGGAFGK
>	sp|P30009|MA	Myristoylated alanine-rich C-kinase substrate OS=Rattus norvegicus GN=Marcks PE=1 SV=2	GEAAAERPGEAAVASSPSK
>	sp|P30009|MA	Myristoylated alanine-rich C-kinase substrate OS=Rattus norvegicus GN=Marcks PE=1 SV=2	GEAAAERPGEAAVASSPSK
>	sp|P30009|MA	Myristoylated alanine-rich C-kinase substrate OS=Rattus norvegicus GN=Marcks PE=1 SV=2	LSGFSFK
>	sp|Q07439|HSI	Heat shock 70 kDa protein 1A/1B OS=Rattus norvegicus GN=Hspa1a PE=2 SV=2	LVSHFVEEFKR
>	sp|Q07439|HSI	Heat shock 70 kDa protein 1A/1B OS=Rattus norvegicus GN=Hspa1a PE=2 SV=2	TTPSYVAFTDTER
>	sp|Q07439|HSI	Heat shock 70 kDa protein 1A/1B OS=Rattus norvegicus GN=Hspa1a PE=2 SV=2	VEIIANDQGNR
>	sp|Q07439|HSI	Heat shock 70 kDa protein 1A/1B OS=Rattus norvegicus GN=Hspa1a PE=2 SV=2	VEIIANDQGNR
>	sp|Q07439|HSI	Heat shock 70 kDa protein 1A/1B OS=Rattus norvegicus GN=Hspa1a PE=2 SV=2	VEIIANDQGNR
>	sp|Q07439|HSI	Heat shock 70 kDa protein 1A/1B OS=Rattus norvegicus GN=Hspa1a PE=2 SV=2	TTPSYVAFTDTER
>	sp|Q07439|HSI	Heat shock 70 kDa protein 1A/1B OS=Rattus norvegicus GN=Hspa1a PE=2 SV=2	QTQTFTTYSDNQPGVLIQVYEGER
>	sp|Q07439|HSI	Heat shock 70 kDa protein 1A/1B OS=Rattus norvegicus GN=Hspa1a PE=2 SV=2	VEIIANDQGNR
>	sp|Q07439|HSI	Heat shock 70 kDa protein 1A/1B OS=Rattus norvegicus GN=Hspa1a PE=2 SV=2	TTPSYVAFTDTER
>	sp|Q07439|HSI	Heat shock 70 kDa protein 1A/1B OS=Rattus norvegicus GN=Hspa1a PE=2 SV=2	LIGDAAK
>	sp|Q07439|HSI	Heat shock 70 kDa protein 1A/1B OS=Rattus norvegicus GN=Hspa1a PE=2 SV=2	TTPSYVAFTDTER
>	sp|Q07439|HSI	Heat shock 70 kDa protein 1A/1B OS=Rattus norvegicus GN=Hspa1a PE=2 SV=2	ATAGDTHLGGEDFDNR
>	sp|Q07439|HSI	Heat shock 70 kDa protein 1A/1B OS=Rattus norvegicus GN=Hspa1a PE=2 SV=2	LIGDAAK
>	sp|P19234|NDI	NADH dehydrogenase [ubiquinone] flavoprotein 2, mitochondrial OS=Rattus norvegicus GN=Ndufv2 PE=1 SV=2	AAAVLPVLDLAQR
>	sp|P19234|NDI	NADH dehydrogenase [ubiquinone] flavoprotein 2, mitochondrial OS=Rattus norvegicus GN=Ndufv2 PE=1 SV=2	GAGGALFVHR
>	sp|P19234|NDI	NADH dehydrogenase [ubiquinone] flavoprotein 2, mitochondrial OS=Rattus norvegicus GN=Ndufv2 PE=1 SV=2	YHIQVCTTTPCMLR
>	sp|P12075|COX	Cytochrome c oxidase subunit 5B, mitochondrial OS=Rattus norvegicus GN=Cox5b PE=1 SV=2	EDPNLVPSVSNKR
>	sp|P12075|COX	Cytochrome c oxidase subunit 5B, mitochondrial OS=Rattus norvegicus GN=Cox5b PE=1 SV=2	EIMIAAQR
>	sp|P12075|COX	Cytochrome c oxidase subunit 5B, mitochondrial OS=Rattus norvegicus GN=Cox5b PE=1 SV=2	LVPYQMVH
>	sp|P12075|COX	Cytochrome c oxidase subunit 5B, mitochondrial OS=Rattus norvegicus GN=Cox5b PE=1 SV=2	EDPNLVPSVSNK
>	sp|Q5BK63|ND	NADH dehydrogenase [ubiquinone] 1 alpha subcomplex subunit 9, mitochondrial OS=Rattus norvegicus GN=Ndufa9 PE=1 SV=2	NFDFEDVFVNIPR
>	sp|Q5BK63|ND	NADH dehydrogenase [ubiquinone] 1 alpha subcomplex subunit 9, mitochondrial OS=Rattus norvegicus GN=Ndufa9 PE=1 SV=2	KAVQHSNVVINLIGR
>	sp|Q5BK63|ND	NADH dehydrogenase [ubiquinone] 1 alpha subcomplex subunit 9, mitochondrial OS=Rattus norvegicus GN=Ndufa9 PE=1 SV=2	KAVQHSNVVINLIGR
>	sp|Q91Z79|LIP	Liprin-alpha-3 OS=Rattus norvegicus GN=Ppfia3 PE=1 SV=2	LLLEHLECLVSR
>	sp|P68255|143	14-3-3 protein theta OS=Rattus norvegicus GN=Ywhaq PE=1 SV=1	AVTEQGAELSNEER
>	sp|P68255|143	14-3-3 protein theta OS=Rattus norvegicus GN=Ywhaq PE=1 SV=1	SICTTVLELLDKYLIANATNPESK
>	sp|P68255|143	14-3-3 protein theta OS=Rattus norvegicus GN=Ywhaq PE=1 SV=1	AVTEQGAELSNEER
>	sp|P68255|143	14-3-3 protein theta OS=Rattus norvegicus GN=Ywhaq PE=1 SV=1	AVTEQGAELSNEER
>	sp|P68255|143	14-3-3 protein theta OS=Rattus norvegicus GN=Ywhaq PE=1 SV=1	AVTEQGAELSNEER
>	sp|P68255|143	14-3-3 protein theta OS=Rattus norvegicus GN=Ywhaq PE=1 SV=1	AVTEQGAELSNEER
>	sp|P68255|143	14-3-3 protein theta OS=Rattus norvegicus GN=Ywhaq PE=1 SV=1	DSTLIMQLLR
>	sp|P68255|143	14-3-3 protein theta OS=Rattus norvegicus GN=Ywhaq PE=1 SV=1	NLLSVAYK
>	sp|P68255|143	14-3-3 protein theta OS=Rattus norvegicus GN=Ywhaq PE=1 SV=1	NLLSVAYK
>	sp|P68255|143	14-3-3 protein theta OS=Rattus norvegicus GN=Ywhaq PE=1 SV=1	NLLSVAYK
>	sp|P68255|143	14-3-3 protein theta OS=Rattus norvegicus GN=Ywhaq PE=1 SV=1	NLLSVAYK
>	sp|P68255|143	14-3-3 protein theta OS=Rattus norvegicus GN=Ywhaq PE=1 SV=1	NLLSVAYK
>	sp|P68255|143	14-3-3 protein theta OS=Rattus norvegicus GN=Ywhaq PE=1 SV=1	VISSIEQK
>	sp|P68255|143	14-3-3 protein theta OS=Rattus norvegicus GN=Ywhaq PE=1 SV=1	NLLSVAYK
>	sp|P68255|143	14-3-3 protein theta OS=Rattus norvegicus GN=Ywhaq PE=1 SV=1	EMQPTHPIR
>	sp|P68255|143	14-3-3 protein theta OS=Rattus norvegicus GN=Ywhaq PE=1 SV=1	VISSIEQK
>	sp|P68255|143	14-3-3 protein theta OS=Rattus norvegicus GN=Ywhaq PE=1 SV=1	NLLSVAYK
>	sp|P68255|143	14-3-3 protein theta OS=Rattus norvegicus GN=Ywhaq PE=1 SV=1	NLLSVAYK
>	sp|P68255|143	14-3-3 protein theta OS=Rattus norvegicus GN=Ywhaq PE=1 SV=1	NLLSVAYK
>	sp|P68255|143	14-3-3 protein theta OS=Rattus norvegicus GN=Ywhaq PE=1 SV=1	AVTEQGAELSNEER
>	sp|P68255|143	14-3-3 protein theta OS=Rattus norvegicus GN=Ywhaq PE=1 SV=1	TAFDEAIAELDTLNEDSYKDSTLIMQLLR
>	sp|P68255|143	14-3-3 protein theta OS=Rattus norvegicus GN=Ywhaq PE=1 SV=1	VISSIEQK
>	sp|P07895|SOD	Superoxide dismutase [Mn], mitochondrial OS=Rattus norvegicus GN=Sod2 PE=1 SV=2	AIWNVINWENVSQR
>	sp|P07895|SOD	Superoxide dismutase [Mn], mitochondrial OS=Rattus norvegicus GN=Sod2 PE=1 SV=2	LQIAACSNQDPLQGTTGLIPLLGIDVWEHAYYLQYK
>	sp|P07895|SOD	Superoxide dismutase [Mn], mitochondrial OS=Rattus norvegicus GN=Sod2 PE=1 SV=2	GDVTTQVALQPALK
>	sp|P07895|SOD	Superoxide dismutase [Mn], mitochondrial OS=Rattus norvegicus GN=Sod2 PE=1 SV=2	AIWNVINWENVSQR
>	sp|P07895|SOD	Superoxide dismutase [Mn], mitochondrial OS=Rattus norvegicus GN=Sod2 PE=1 SV=2	GDVTTQVALQPALK
>	sp|P07895|SOD	Superoxide dismutase [Mn], mitochondrial OS=Rattus norvegicus GN=Sod2 PE=1 SV=2	KHSLPDLPYDYGALEPHINAQIMQLHHSK
>	sp|P07895|SOD	Superoxide dismutase [Mn], mitochondrial OS=Rattus norvegicus GN=Sod2 PE=1 SV=2	AIWNVINWENVSQR
>	sp|O35796|C1Q	Complement component 1 Q subcomponent-binding protein, mitochondrial OS=Rattus norvegicus GN=C1qbp PE=1 SV=2	AFVEFLTDEIKEEK
>	sp|O35796|C1Q	Complement component 1 Q subcomponent-binding protein, mitochondrial OS=Rattus norvegicus GN=C1qbp PE=1 SV=2	AFVEFLTDEIKEEK
>	sp|O35796|C1Q	Complement component 1 Q subcomponent-binding protein, mitochondrial OS=Rattus norvegicus GN=C1qbp PE=1 SV=2	AFVEFLTDEIKEEK
>	sp|O35796|C1Q	Complement component 1 Q subcomponent-binding protein, mitochondrial OS=Rattus norvegicus GN=C1qbp PE=1 SV=2	AFVEFLTDEIKEEK
>	sp|O35796|C1Q	Complement component 1 Q subcomponent-binding protein, mitochondrial OS=Rattus norvegicus GN=C1qbp PE=1 SV=2	AFVEFLTDEIKEEK
>	sp|O35796|C1Q	Complement component 1 Q subcomponent-binding protein, mitochondrial OS=Rattus norvegicus GN=C1qbp PE=1 SV=2	LHTEGDKAFVEFLTDEIKEEKK
>	sp|O35796|C1Q	Complement component 1 Q subcomponent-binding protein, mitochondrial OS=Rattus norvegicus GN=C1qbp PE=1 SV=2	LHTEGDKAFVEFLTDEIKEEKK
>	sp|Q9JHY2|SFX	Sideroflexin-3 OS=Rattus norvegicus GN=Sfxn3 PE=2 SV=1	ELQVGIPVTDEAGQR
>	sp|P62250|RS1	40S ribosomal protein S16 OS=Rattus norvegicus GN=Rps16 PE=1 SV=2	LLEPVLLLGK
>	sp|P62250|RS1	40S ribosomal protein S16 OS=Rattus norvegicus GN=Rps16 PE=1 SV=2	LLEPVLLLGK

>	sp|P62250|RS1 40S ribosomal protein S16 OS=Rattus norvegicus GN=Rps16 PE=1 SV=2	VNGRPLEMIEPR
>	sp|Q6AY56|TB/ Tubulin alpha-8 chain OS=Rattus norvegicus GN=Tuba8 PE=2 SV=1	RAFVHWYVGEGMEEGEFSEAREDLAALEK
>	sp|Q6AY56|TB/ Tubulin alpha-8 chain OS=Rattus norvegicus GN=Tuba8 PE=2 SV=1	AFVHWYVGEGMEEGEFSEAR
>	sp|Q6AY56|TB/ Tubulin alpha-8 chain OS=Rattus norvegicus GN=Tuba8 PE=2 SV=1	AFVHWYVGEGMEEGEFSEAR
>	sp|Q6AY56|TB/ Tubulin alpha-8 chain OS=Rattus norvegicus GN=Tuba8 PE=2 SV=1	AFVHWYVGEGMEEGEFSEAR
>	sp|Q6AY56|TB/ Tubulin alpha-8 chain OS=Rattus norvegicus GN=Tuba8 PE=2 SV=1	AFVHWYVGEGMEEGEFSEAR
>	sp|Q6AY56|TB/ Tubulin alpha-8 chain OS=Rattus norvegicus GN=Tuba8 PE=2 SV=1	AFVHWYVGEGMEEGEFSEAR
>	sp|Q6AY56|TB/ Tubulin alpha-8 chain OS=Rattus norvegicus GN=Tuba8 PE=2 SV=1	AFVHWYVGEGMEEGEFSEAR
>	sp|Q6AY56|TB/ Tubulin alpha-8 chain OS=Rattus norvegicus GN=Tuba8 PE=2 SV=1	AFVHWYVGEGMEEGEFSEAR
>	sp|Q6AY56|TB/ Tubulin alpha-8 chain OS=Rattus norvegicus GN=Tuba8 PE=2 SV=1	AFVHWYVGEGMEEGEFSEAR
>	sp|Q6AY56|TB/ Tubulin alpha-8 chain OS=Rattus norvegicus GN=Tuba8 PE=2 SV=1	AFVHWYVGEGMEEGEFSEAR
>	sp|Q6AY56|TB/ Tubulin alpha-8 chain OS=Rattus norvegicus GN=Tuba8 PE=2 SV=1	AFVHWYVGEGMEEGEFSEAR
>	sp|Q6AY56|TB/ Tubulin alpha-8 chain OS=Rattus norvegicus GN=Tuba8 PE=2 SV=1	AFVHWYVGEGMEEGEFSEAR
>	sp|Q6AY56|TB/ Tubulin alpha-8 chain OS=Rattus norvegicus GN=Tuba8 PE=2 SV=1	AFVHWYVGEGMEEGEFSEAR
>	sp|Q6AY56|TB/ Tubulin alpha-8 chain OS=Rattus norvegicus GN=Tuba8 PE=2 SV=1	AFVHWYVGEGMEEGEFSEAR
>	sp|Q6AY56|TB/ Tubulin alpha-8 chain OS=Rattus norvegicus GN=Tuba8 PE=2 SV=1	AFVHWYVGEGMEEGEFSEAR
>	sp|Q6AY56|TB/ Tubulin alpha-8 chain OS=Rattus norvegicus GN=Tuba8 PE=2 SV=1	AVCMLSNTTAIAEAWAR
>	sp|Q6AY56|TB/ Tubulin alpha-8 chain OS=Rattus norvegicus GN=Tuba8 PE=2 SV=1	AVCMLSNTTAIAEAWAR
>	sp|Q6AY56|TB/ Tubulin alpha-8 chain OS=Rattus norvegicus GN=Tuba8 PE=2 SV=1	AVCMLSNTTAIAEAWAR
>	sp|Q6AY56|TB/ Tubulin alpha-8 chain OS=Rattus norvegicus GN=Tuba8 PE=2 SV=1	AVCMLSNTTAIAEAWAR
>	sp|Q6AY56|TB/ Tubulin alpha-8 chain OS=Rattus norvegicus GN=Tuba8 PE=2 SV=1	AVCMLSNTTAIAEAWAR
>	sp|Q6AY56|TB/ Tubulin alpha-8 chain OS=Rattus norvegicus GN=Tuba8 PE=2 SV=1	AVCMLSNTTAIAEAWAR
>	sp|Q6AY56|TB/ Tubulin alpha-8 chain OS=Rattus norvegicus GN=Tuba8 PE=2 SV=1	AVCMLSNTTAIAEAWAR
>	sp|Q6AY56|TB/ Tubulin alpha-8 chain OS=Rattus norvegicus GN=Tuba8 PE=2 SV=1	AVCMLSNTTAIAEAWAR
>	sp|Q6AY56|TB/ Tubulin alpha-8 chain OS=Rattus norvegicus GN=Tuba8 PE=2 SV=1	AVCMLSNTTAIAEAWAR
>	sp|Q6AY56|TB/ Tubulin alpha-8 chain OS=Rattus norvegicus GN=Tuba8 PE=2 SV=1	AVCMLSNTTAIAEAWAR
>	sp|Q6AY56|TB/ Tubulin alpha-8 chain OS=Rattus norvegicus GN=Tuba8 PE=2 SV=1	AVCMLSNTTAIAEAWAR
>	sp|Q6AY56|TB/ Tubulin alpha-8 chain OS=Rattus norvegicus GN=Tuba8 PE=2 SV=1	AVCMLSNTTAIAEAWAR
>	sp|Q6AY56|TB/ Tubulin alpha-8 chain OS=Rattus norvegicus GN=Tuba8 PE=2 SV=1	AVCMLSNTTAIAEAWAR
>	sp|Q6AY56|TB/ Tubulin alpha-8 chain OS=Rattus norvegicus GN=Tuba8 PE=2 SV=1	AVCMLSNTTAIAEAWAR
>	sp|Q6AY56|TB/ Tubulin alpha-8 chain OS=Rattus norvegicus GN=Tuba8 PE=2 SV=1	AVCMLSNTTAIAEAWAR
>	sp|Q6AY56|TB/ Tubulin alpha-8 chain OS=Rattus norvegicus GN=Tuba8 PE=2 SV=1	AVCMLSNTTAIAEAWAR
>	sp|Q6AY56|TB/ Tubulin alpha-8 chain OS=Rattus norvegicus GN=Tuba8 PE=2 SV=1	EDAANNYAR
>	sp|Q6AY56|TB/ Tubulin alpha-8 chain OS=Rattus norvegicus GN=Tuba8 PE=2 SV=1	FDGALNVDLTEFQTNLVPYPR
>	sp|Q6AY56|TB/ Tubulin alpha-8 chain OS=Rattus norvegicus GN=Tuba8 PE=2 SV=1	FDGALNVDLTEFQTNLVPYPR
>	sp|Q6AY56|TB/ Tubulin alpha-8 chain OS=Rattus norvegicus GN=Tuba8 PE=2 SV=1	FDGALNVDLTEFQTNLVPYPR
>	sp|Q6AY56|TB/ Tubulin alpha-8 chain OS=Rattus norvegicus GN=Tuba8 PE=2 SV=1	FDGALNVDLTEFQTNLVPYPR
>	sp|Q6AY56|TB/ Tubulin alpha-8 chain OS=Rattus norvegicus GN=Tuba8 PE=2 SV=1	FDGALNVDLTEFQTNLVPYPR
>	sp|Q6AY56|TB/ Tubulin alpha-8 chain OS=Rattus norvegicus GN=Tuba8 PE=2 SV=1	FDGALNVDLTEFQTNLVPYPR
>	sp|Q6AY56|TB/ Tubulin alpha-8 chain OS=Rattus norvegicus GN=Tuba8 PE=2 SV=1	LDHKFDLMYAK
>	sp|Q6AY56|TB/ Tubulin alpha-8 chain OS=Rattus norvegicus GN=Tuba8 PE=2 SV=1	LDHKFDLMYAK
>	sp|Q6AY56|TB/ Tubulin alpha-8 chain OS=Rattus norvegicus GN=Tuba8 PE=2 SV=1	LDHKFDLMYAK
>	sp|Q6AY56|TB/ Tubulin alpha-8 chain OS=Rattus norvegicus GN=Tuba8 PE=2 SV=1	LEFAIYPAPQVSTAVVEPYNSILTTHTTLEHSDCAFMVDNEAIYDICR
>	sp|Q6AY56|TB/ Tubulin alpha-8 chain OS=Rattus norvegicus GN=Tuba8 PE=2 SV=1	NLDIERPTYTNLNR
>	sp|Q6AY56|TB/ Tubulin alpha-8 chain OS=Rattus norvegicus GN=Tuba8 PE=2 SV=1	NLDIERPTYTNLNRLISQIVSSITASLR
>	sp|Q6AY56|TB/ Tubulin alpha-8 chain OS=Rattus norvegicus GN=Tuba8 PE=2 SV=1	NLDIERPTYTNLNRLISQIVSSITASLRFDGALNVDLTEFQTNLVPYPR
>	sp|Q6AY56|TB/ Tubulin alpha-8 chain OS=Rattus norvegicus GN=Tuba8 PE=2 SV=1	NLDIERPTYTNLNRLISQIVSSITASLRFDGALNVDLTEFQTNLVPYPR
>	sp|Q6AY56|TB/ Tubulin alpha-8 chain OS=Rattus norvegicus GN=Tuba8 PE=2 SV=1	NLDIERPTYTNLNRLISQIVSSITASLRFDGALNVDLTEFQTNLVPYPR
>	sp|Q6AY56|TB/ Tubulin alpha-8 chain OS=Rattus norvegicus GN=Tuba8 PE=2 SV=1	NLDIERPTYTNLNRLISQIVSSITASLRFDGALNVDLTEFQTNLVPYPR
>	sp|Q6AY56|TB/ Tubulin alpha-8 chain OS=Rattus norvegicus GN=Tuba8 PE=2 SV=1	PGGDLAK
>	sp|Q6AY56|TB/ Tubulin alpha-8 chain OS=Rattus norvegicus GN=Tuba8 PE=2 SV=1	PPTVVPGGDLAK
>	sp|Q6AY56|TB/ Tubulin alpha-8 chain OS=Rattus norvegicus GN=Tuba8 PE=2 SV=1	QLFHPEQLITGK
>	sp|Q6AY56|TB/ Tubulin alpha-8 chain OS=Rattus norvegicus GN=Tuba8 PE=2 SV=1	QLFHPEQLITGK
>	sp|Q6AY56|TB/ Tubulin alpha-8 chain OS=Rattus norvegicus GN=Tuba8 PE=2 SV=1	QLFHPEQLITGK
>	sp|Q6AY56|TB/ Tubulin alpha-8 chain OS=Rattus norvegicus GN=Tuba8 PE=2 SV=1	QLFHPEQLITGK
>	sp|Q6AY56|TB/ Tubulin alpha-8 chain OS=Rattus norvegicus GN=Tuba8 PE=2 SV=1	QLFHPEQLITGK
>	sp|Q6AY56|TB/ Tubulin alpha-8 chain OS=Rattus norvegicus GN=Tuba8 PE=2 SV=1	QLFHPEQLITGKEDAANNYAR
>	sp|Q6AY56|TB/ Tubulin alpha-8 chain OS=Rattus norvegicus GN=Tuba8 PE=2 SV=1	QLFHPEQLITGKEDAANNYAR
>	sp|Q6AY56|TB/ Tubulin alpha-8 chain OS=Rattus norvegicus GN=Tuba8 PE=2 SV=1	QLFHPEQLITGKEDAANNYAR
>	sp|Q6AY56|TB/ Tubulin alpha-8 chain OS=Rattus norvegicus GN=Tuba8 PE=2 SV=1	QLFHPEQLITGKEDAANNYAR
>	sp|Q6AY56|TB/ Tubulin alpha-8 chain OS=Rattus norvegicus GN=Tuba8 PE=2 SV=1	QLFHPEQLITGKEDAANNYAR
>	sp|Q6AY56|TB/ Tubulin alpha-8 chain OS=Rattus norvegicus GN=Tuba8 PE=2 SV=1	QLFHPEQLITGKEDAANNYAR
>	sp|Q6AY56|TB/ Tubulin alpha-8 chain OS=Rattus norvegicus GN=Tuba8 PE=2 SV=1	QLFHPEQLITGKEDAANNYAR
>	sp|Q6AY56|TB/ Tubulin alpha-8 chain OS=Rattus norvegicus GN=Tuba8 PE=2 SV=1	QLFHPEQLITGKEDAANNYAR
>	sp|Q6AY56|TB/ Tubulin alpha-8 chain OS=Rattus norvegicus GN=Tuba8 PE=2 SV=1	QLFHPEQLITGKEDAANNYAR
>	sp|Q6AY56|TB/ Tubulin alpha-8 chain OS=Rattus norvegicus GN=Tuba8 PE=2 SV=1	QLFHPEQLITGKEDAANNYAR
>	sp|Q6AY56|TB/ Tubulin alpha-8 chain OS=Rattus norvegicus GN=Tuba8 PE=2 SV=1	QLFHPEQLITGKEDAANNYAR
>	sp|Q6AY56|TB/ Tubulin alpha-8 chain OS=Rattus norvegicus GN=Tuba8 PE=2 SV=1	QLFHPEQLITGKEDAANNYAR
>	sp|Q6AY56|TB/ Tubulin alpha-8 chain OS=Rattus norvegicus GN=Tuba8 PE=2 SV=1	QLFHPEQLITGKEDAANNYAR
>	sp|Q6AY56|TB/ Tubulin alpha-8 chain OS=Rattus norvegicus GN=Tuba8 PE=2 SV=1	QLFHPEQLITGKEDAANNYAR
>	sp|Q6AY56|TB/ Tubulin alpha-8 chain OS=Rattus norvegicus GN=Tuba8 PE=2 SV=1	QLFHPEQLITGKEDAANNYAR
>	sp|Q6AY56|TB/ Tubulin alpha-8 chain OS=Rattus norvegicus GN=Tuba8 PE=2 SV=1	QLFHPEQLITGKEDAANNYAR
>	sp|Q6AY56|TB/ Tubulin alpha-8 chain OS=Rattus norvegicus GN=Tuba8 PE=2 SV=1	QLFHPEQLITGKEDAANNYAR
>	sp|Q6AY56|TB/ Tubulin alpha-8 chain OS=Rattus norvegicus GN=Tuba8 PE=2 SV=1	QLFHPEQLITGKEDAANNYAR

>	sp|Q6AY56|TB Tubulin alpha-8 chain OS=Rattus norvegicus GN=Tuba8 PE=2 SV=1	QLFHPEQLITGKEDAANNYAR
>	sp|Q6AY56|TB Tubulin alpha-8 chain OS=Rattus norvegicus GN=Tuba8 PE=2 SV=1	QLFHPEQLITGKEDAANNYAR
>	sp|Q6AY56|TB Tubulin alpha-8 chain OS=Rattus norvegicus GN=Tuba8 PE=2 SV=1	QLFHPEQLITGKEDAANNYAR
>	sp|Q6AY56|TB Tubulin alpha-8 chain OS=Rattus norvegicus GN=Tuba8 PE=2 SV=1	QLFHPEQLITGKEDAANNYAR
>	sp|Q6AY56|TB Tubulin alpha-8 chain OS=Rattus norvegicus GN=Tuba8 PE=2 SV=1	QLFHPEQLITGKEDAANNYAR
>	sp|Q6AY56|TB Tubulin alpha-8 chain OS=Rattus norvegicus GN=Tuba8 PE=2 SV=1	QLFHPEQLITGKEDAANNYAR
>	sp|Q6AY56|TB Tubulin alpha-8 chain OS=Rattus norvegicus GN=Tuba8 PE=2 SV=1	QLFHPEQLITGKEDAANNYAR
>	sp|Q6AY56|TB Tubulin alpha-8 chain OS=Rattus norvegicus GN=Tuba8 PE=2 SV=1	QLFHPEQLITGKEDAANNYAR
>	sp|Q6AY56|TB Tubulin alpha-8 chain OS=Rattus norvegicus GN=Tuba8 PE=2 SV=1	QLFHPEQLITGKEDAANNYAR
>	sp|Q6AY56|TB Tubulin alpha-8 chain OS=Rattus norvegicus GN=Tuba8 PE=2 SV=1	QLFHPEQLITGKEDAANNYAR
>	sp|Q6AY56|TB Tubulin alpha-8 chain OS=Rattus norvegicus GN=Tuba8 PE=2 SV=1	QLFHPEQLITGKEDAANNYAR
>	sp|Q6AY56|TB Tubulin alpha-8 chain OS=Rattus norvegicus GN=Tuba8 PE=2 SV=1	QLFHPEQLITGKEDAANNYAR
>	sp|Q6AY56|TB Tubulin alpha-8 chain OS=Rattus norvegicus GN=Tuba8 PE=2 SV=1	RAFVHWYVGEGMEEGEFSEAR
>	sp|Q6AY56|TB Tubulin alpha-8 chain OS=Rattus norvegicus GN=Tuba8 PE=2 SV=1	RAFVHWYVGEGMEEGEFSEAR
>	sp|Q6AY56|TB Tubulin alpha-8 chain OS=Rattus norvegicus GN=Tuba8 PE=2 SV=1	RAFVHWYVGEGMEEGEFSEAR
>	sp|Q6AY56|TB Tubulin alpha-8 chain OS=Rattus norvegicus GN=Tuba8 PE=2 SV=1	RAFVHWYVGEGMEEGEFSEAR
>	sp|Q6AY56|TB Tubulin alpha-8 chain OS=Rattus norvegicus GN=Tuba8 PE=2 SV=1	SKLEFAIYPAPQVSTAVVEPYNSILTTHTTLEHSDCAFMVDNEAIYDICR
>	sp|Q6AY56|TB Tubulin alpha-8 chain OS=Rattus norvegicus GN=Tuba8 PE=2 SV=1	SKLEFAIYPAPQVSTAVVEPYNSILTTHTTLEHSDCAFMVDNEAIYDICR
>	sp|Q6AY56|TB Tubulin alpha-8 chain OS=Rattus norvegicus GN=Tuba8 PE=2 SV=1	SKLEFAIYPAPQVSTAVVEPYNSILTTHTTLEHSDCAFMVDNEAIYDICR
>	sp|Q6AY56|TB Tubulin alpha-8 chain OS=Rattus norvegicus GN=Tuba8 PE=2 SV=1	SKLEFAIYPAPQVSTAVVEPYNSILTTHTTLEHSDCAFMVDNEAIYDICR
>	sp|Q6AY56|TB Tubulin alpha-8 chain OS=Rattus norvegicus GN=Tuba8 PE=2 SV=1	TIQFVDWCPTGFK
>	sp|Q6AY56|TB Tubulin alpha-8 chain OS=Rattus norvegicus GN=Tuba8 PE=2 SV=1	TIQFVDWCPTGFK
>	sp|Q6AY56|TB Tubulin alpha-8 chain OS=Rattus norvegicus GN=Tuba8 PE=2 SV=1	TIQFVDWCPTGFK
>	sp|Q6AY56|TB Tubulin alpha-8 chain OS=Rattus norvegicus GN=Tuba8 PE=2 SV=1	TIQFVDWCPTGFK
>	sp|Q6AY56|TB Tubulin alpha-8 chain OS=Rattus norvegicus GN=Tuba8 PE=2 SV=1	VGINYQPPTVVPGGDLAK
>	sp|Q6AY56|TB Tubulin alpha-8 chain OS=Rattus norvegicus GN=Tuba8 PE=2 SV=1	VGINYQPPTVVPGGDLAK
>	sp|Q6AY56|TB Tubulin alpha-8 chain OS=Rattus norvegicus GN=Tuba8 PE=2 SV=1	VGINYQPPTVVPGGDLAK
>	sp|Q6AY56|TB Tubulin alpha-8 chain OS=Rattus norvegicus GN=Tuba8 PE=2 SV=1	VGINYQPPTVVPGGDLAK
>	sp|Q6AY56|TB Tubulin alpha-8 chain OS=Rattus norvegicus GN=Tuba8 PE=2 SV=1	VGINYQPPTVVPGGDLAK
>	sp|Q6AY56|TB Tubulin alpha-8 chain OS=Rattus norvegicus GN=Tuba8 PE=2 SV=1	VGINYQPPTVVPGGDLAK
>	sp|Q6AY56|TB Tubulin alpha-8 chain OS=Rattus norvegicus GN=Tuba8 PE=2 SV=1	VGINYQPPTVVPGGDLAK
>	sp|Q6AY56|TB Tubulin alpha-8 chain OS=Rattus norvegicus GN=Tuba8 PE=2 SV=1	VGINYQPPTVVPGGDLAK
>	sp|Q6AY56|TB Tubulin alpha-8 chain OS=Rattus norvegicus GN=Tuba8 PE=2 SV=1	VGINYQPPTVVPGGDLAK
>	sp|Q6AY56|TB Tubulin alpha-8 chain OS=Rattus norvegicus GN=Tuba8 PE=2 SV=1	VGINYQPPTVVPGGDLAK
>	sp|Q6AY56|TB Tubulin alpha-8 chain OS=Rattus norvegicus GN=Tuba8 PE=2 SV=1	VGINYQPPTVVPGGDLAK
>	sp|Q6AY56|TB Tubulin alpha-8 chain OS=Rattus norvegicus GN=Tuba8 PE=2 SV=1	VGINYQPPTVVPGGDLAK
>	sp|Q6AY56|TB Tubulin alpha-8 chain OS=Rattus norvegicus GN=Tuba8 PE=2 SV=1	VGINYQPPTVVPGGDLAK
>	sp|Q6AY56|TB Tubulin alpha-8 chain OS=Rattus norvegicus GN=Tuba8 PE=2 SV=1	QLFHPEQLITGKEDAANNYAR
>	sp|Q6AY56|TB Tubulin alpha-8 chain OS=Rattus norvegicus GN=Tuba8 PE=2 SV=1	QLFHPEQLITGKEDAANNYAR
>	sp|Q6AY56|TB Tubulin alpha-8 chain OS=Rattus norvegicus GN=Tuba8 PE=2 SV=1	TIQFVDWCPTGFK
>	sp|Q6AY56|TB Tubulin alpha-8 chain OS=Rattus norvegicus GN=Tuba8 PE=2 SV=1	QLFHPEQLITGKEDAANNYAR
>	sp|Q6AY56|TB Tubulin alpha-8 chain OS=Rattus norvegicus GN=Tuba8 PE=2 SV=1	VGINYQPPTVVPGGDLAK
>	sp|Q6AY56|TB Tubulin alpha-8 chain OS=Rattus norvegicus GN=Tuba8 PE=2 SV=1	QLFHPEQLITGKEDAANNYAR
>	sp|Q6AY56|TB Tubulin alpha-8 chain OS=Rattus norvegicus GN=Tuba8 PE=2 SV=1	KFDLMYAKRAFVHWYVGEGMEEGEFSEAR
>	sp|Q6AY56|TB Tubulin alpha-8 chain OS=Rattus norvegicus GN=Tuba8 PE=2 SV=1	KFDLMYAKRAFVHWYVGEGMEEGEFSEAR
>	sp|Q6AY56|TB Tubulin alpha-8 chain OS=Rattus norvegicus GN=Tuba8 PE=2 SV=1	AVCMLSNTTAIAEAWAR
>	sp|Q6AY56|TB Tubulin alpha-8 chain OS=Rattus norvegicus GN=Tuba8 PE=2 SV=1	VGINYQPPTVVPGGDLAK
>	sp|Q6AY56|TB Tubulin alpha-8 chain OS=Rattus norvegicus GN=Tuba8 PE=2 SV=1	VGINYQPPTVVPGGDLAK
>	sp|Q6AY56|TB Tubulin alpha-8 chain OS=Rattus norvegicus GN=Tuba8 PE=2 SV=1	VGINYQPPTVVPGGDLAK
>	sp|Q6AY56|TB Tubulin alpha-8 chain OS=Rattus norvegicus GN=Tuba8 PE=2 SV=1	QLFHPEQLITGK
>	sp|Q6AY56|TB Tubulin alpha-8 chain OS=Rattus norvegicus GN=Tuba8 PE=2 SV=1	LEFAIYPAPQVSTAVVEPYNSILTTHTTLEHSDCAFMVDNEAIYDICR
>	sp|Q6AY56|TB Tubulin alpha-8 chain OS=Rattus norvegicus GN=Tuba8 PE=2 SV=1	SKLEFAIYPAPQVSTAVVEPYNSILTTHTTLEHSDCAFMVDNEAIYDICR
>	sp|Q6AY56|TB Tubulin alpha-8 chain OS=Rattus norvegicus GN=Tuba8 PE=2 SV=1	SKLEFAIYPAPQVSTAVVEPYNSILTTHTTLEHSDCAFMVDNEAIYDICR
>	sp|Q6AY56|TB Tubulin alpha-8 chain OS=Rattus norvegicus GN=Tuba8 PE=2 SV=1	QLFHPEQLITGKEDAANNYAR
>	sp|Q6AY56|TB Tubulin alpha-8 chain OS=Rattus norvegicus GN=Tuba8 PE=2 SV=1	QLFHPEQLITGKEDAANNYAR
>	sp|Q6AY56|TB Tubulin alpha-8 chain OS=Rattus norvegicus GN=Tuba8 PE=2 SV=1	QLFHPEQLITGKEDAANNYAR
>	sp|Q6AY56|TB Tubulin alpha-8 chain OS=Rattus norvegicus GN=Tuba8 PE=2 SV=1	LEFAIYPAPQVSTAVVEPYNSILTTHTTLEHSDCAFMVDNEAIYDICR
>	sp|Q6AY56|TB Tubulin alpha-8 chain OS=Rattus norvegicus GN=Tuba8 PE=2 SV=1	FDGALNVDLTEFQTNLVPYPR
>	sp|Q6AY56|TB Tubulin alpha-8 chain OS=Rattus norvegicus GN=Tuba8 PE=2 SV=1	QLFHPEQLITGKEDAANNYAR
>	sp|Q6AY56|TB Tubulin alpha-8 chain OS=Rattus norvegicus GN=Tuba8 PE=2 SV=1	QLFHPEQLITGKEDAANNYAR
>	sp|Q6AY56|TB Tubulin alpha-8 chain OS=Rattus norvegicus GN=Tuba8 PE=2 SV=1	TIQFVDWCPTGFK
>	sp|Q6AY56|TB Tubulin alpha-8 chain OS=Rattus norvegicus GN=Tuba8 PE=2 SV=1	PPTVVPGGDLAK
>	sp|Q6AY56|TB Tubulin alpha-8 chain OS=Rattus norvegicus GN=Tuba8 PE=2 SV=1	AYHEQLSVAEITSSCFEPNSQMVK
>	sp|Q6AY56|TB Tubulin alpha-8 chain OS=Rattus norvegicus GN=Tuba8 PE=2 SV=1	VGINYQPPTVVPGGDLAK
>	sp|Q6AY56|TB Tubulin alpha-8 chain OS=Rattus norvegicus GN=Tuba8 PE=2 SV=1	SKLEFAIYPAPQVSTAVVEPYNSILTTHTTLEHSDCAFMVDNEAIYDICR
>	sp|Q6AY56|TB Tubulin alpha-8 chain OS=Rattus norvegicus GN=Tuba8 PE=2 SV=1	AVCMLSNTTAIAEAWAR
>	sp|Q6AY56|TB Tubulin alpha-8 chain OS=Rattus norvegicus GN=Tuba8 PE=2 SV=1	FDGALNVDLTEFQTNLVPYPR
>	sp|Q6AY56|TB Tubulin alpha-8 chain OS=Rattus norvegicus GN=Tuba8 PE=2 SV=1	AFVHWYVGEGMEEGEFSEAR
>	sp|Q6AY56|TB Tubulin alpha-8 chain OS=Rattus norvegicus GN=Tuba8 PE=2 SV=1	GDVVPK
>	sp|Q6AY56|TB Tubulin alpha-8 chain OS=Rattus norvegicus GN=Tuba8 PE=2 SV=1	FDGALNVDLTEFQTNLVPYPR
>	sp|Q6AY56|TB Tubulin alpha-8 chain OS=Rattus norvegicus GN=Tuba8 PE=2 SV=1	LDHKFDLMYAK
>	sp|Q6AY56|TB Tubulin alpha-8 chain OS=Rattus norvegicus GN=Tuba8 PE=2 SV=1	AFVHWYVGEGMEEGEFSEAREDLAALEK
>	sp|Q6AY56|TB Tubulin alpha-8 chain OS=Rattus norvegicus GN=Tuba8 PE=2 SV=1	QLFHPEQLITGKEDAANNYAR
>	sp|Q6AY56|TB Tubulin alpha-8 chain OS=Rattus norvegicus GN=Tuba8 PE=2 SV=1	FDGALNVDLTEFQTNLVPYPR

>	sp|Q6AY56|TB	Tubulin alpha-8 chain OS=Rattus norvegicus GN=Tuba8 PE=2 SV=1	PGGDLAK
>	sp|Q6AY56|TB	Tubulin alpha-8 chain OS=Rattus norvegicus GN=Tuba8 PE=2 SV=1	QLFHPEQLITGKEDAANNYAR
>	sp|Q6AY56|TB	Tubulin alpha-8 chain OS=Rattus norvegicus GN=Tuba8 PE=2 SV=1	QLFHPEQLITGK
>	sp|Q6AY56|TB	Tubulin alpha-8 chain OS=Rattus norvegicus GN=Tuba8 PE=2 SV=1	VGINYQPPTVVPGGDLAK
>	sp|Q6AY56|TB	Tubulin alpha-8 chain OS=Rattus norvegicus GN=Tuba8 PE=2 SV=1	AFVHWYVGEGMEEGEFSEAR
>	sp|Q6AY56|TB	Tubulin alpha-8 chain OS=Rattus norvegicus GN=Tuba8 PE=2 SV=1	VGINYQPPTVVPGGDLAK
>	sp|Q6AY56|TB	Tubulin alpha-8 chain OS=Rattus norvegicus GN=Tuba8 PE=2 SV=1	LEFAIYPAPQVSTAVVEPYNSILTTHTTLEHSDCAFMVDNEAIYDICR
>	sp|Q6AY56|TB	Tubulin alpha-8 chain OS=Rattus norvegicus GN=Tuba8 PE=2 SV=1	QLFHPEQLITGKEDAANNYAR
>	sp|Q6AY56|TB	Tubulin alpha-8 chain OS=Rattus norvegicus GN=Tuba8 PE=2 SV=1	QLFHPEQLITGKEDAANNYAR
>	sp|Q6AY56|TB	Tubulin alpha-8 chain OS=Rattus norvegicus GN=Tuba8 PE=2 SV=1	AFVHWYVGEGMEEGEFSEAR
>	sp|Q6AY56|TB	Tubulin alpha-8 chain OS=Rattus norvegicus GN=Tuba8 PE=2 SV=1	VGINYQPPTVVPGGDLAK
>	sp|Q6AY56|TB	Tubulin alpha-8 chain OS=Rattus norvegicus GN=Tuba8 PE=2 SV=1	LDHKFDLMYAK
>	sp|Q6AY56|TB	Tubulin alpha-8 chain OS=Rattus norvegicus GN=Tuba8 PE=2 SV=1	QLFHPEQLITGKEDAANNYAR
>	sp|Q6AY56|TB	Tubulin alpha-8 chain OS=Rattus norvegicus GN=Tuba8 PE=2 SV=1	VGINYQPPTVVPGGDLAK
>	sp|Q6AY56|TB	Tubulin alpha-8 chain OS=Rattus norvegicus GN=Tuba8 PE=2 SV=1	QLFHPEQLITGKEDAANNYAR
>	sp|Q6AY56|TB	Tubulin alpha-8 chain OS=Rattus norvegicus GN=Tuba8 PE=2 SV=1	QLFHPEQLITGKEDAANNYAR
>	sp|Q6AY56|TB	Tubulin alpha-8 chain OS=Rattus norvegicus GN=Tuba8 PE=2 SV=1	AYHEQLSVAEITSSCFEPNSQMVK
>	sp|Q6AY56|TB	Tubulin alpha-8 chain OS=Rattus norvegicus GN=Tuba8 PE=2 SV=1	TIQFVDWCPTGFK
>	sp|Q6AY56|TB	Tubulin alpha-8 chain OS=Rattus norvegicus GN=Tuba8 PE=2 SV=1	AVCMLSNTTAIAEAWAR
>	sp|Q6AY56|TB	Tubulin alpha-8 chain OS=Rattus norvegicus GN=Tuba8 PE=2 SV=1	QLFHPEQLITGKEDAANNYAR
>	sp|Q6AY56|TB	Tubulin alpha-8 chain OS=Rattus norvegicus GN=Tuba8 PE=2 SV=1	NLDIERPTYTNLNRLISQIVSSITASLR
>	sp|Q6AY56|TB	Tubulin alpha-8 chain OS=Rattus norvegicus GN=Tuba8 PE=2 SV=1	LDHKFDLMYAK
>	sp|Q6AY56|TB	Tubulin alpha-8 chain OS=Rattus norvegicus GN=Tuba8 PE=2 SV=1	AVCMLSNTTAIAEAWAR
>	sp|Q5RKI1|IF4	Eukaryotic initiation factor 4A-II OS=Rattus norvegicus GN=Eif4a2 PE=1 SV=1	LQAEAPHIVVGTPGR
>	sp|Q5RKI1|IF4	Eukaryotic initiation factor 4A-II OS=Rattus norvegicus GN=Eif4a2 PE=1 SV=1	LQAEAPHIVVGTPGR
>	sp|Q5RKI1|IF4	Eukaryotic initiation factor 4A-II OS=Rattus norvegicus GN=Eif4a2 PE=1 SV=1	LQAEAPHIVVGTPGR
>	sp|Q5RKI1|IF4	Eukaryotic initiation factor 4A-II OS=Rattus norvegicus GN=Eif4a2 PE=1 SV=1	LQAEAPHIVVGTPGR
>	sp|P63029|TCT	Translationally-controlled tumor protein OS=Rattus norvegicus GN=Tpt1 PE=1 SV=1	DLISHDELFSDIYK
>	sp|P63029|TCT	Translationally-controlled tumor protein OS=Rattus norvegicus GN=Tpt1 PE=1 SV=1	DLISHDELFSDIYK
>	sp|P63029|TCT	Translationally-controlled tumor protein OS=Rattus norvegicus GN=Tpt1 PE=1 SV=1	DLISHDELFSDIYK
>	sp|P62198|PRS	26S protease regulatory subunit 8 OS=Rattus norvegicus GN=Psmc5 PE=1 SV=1	IDILDSALLRPGR
>	sp|Q9ESW0|DD	DNA damage-binding protein 1 OS=Rattus norvegicus GN=Ddb1 PE=2 SV=1	KTEPATGFIDGDLIESFLDISRPK
>	sp|Q9ESW0|DD	DNA damage-binding protein 1 OS=Rattus norvegicus GN=Ddb1 PE=2 SV=1	KTEPATGFIDGDLIESFLDISRPK
>	sp|P04256|RO	Heterogeneous nuclear ribonucleoprotein A1 OS=Rattus norvegicus GN=Hnrnpa1 PE=1 SV=3	GFGFVTYATVEEVDAAMNARPHK
>	sp|P04256|RO	Heterogeneous nuclear ribonucleoprotein A1 OS=Rattus norvegicus GN=Hnrnpa1 PE=1 SV=3	KIFVGGIKEDTEEHHLR
>	sp|P04256|RO	Heterogeneous nuclear ribonucleoprotein A1 OS=Rattus norvegicus GN=Hnrnpa1 PE=1 SV=3	ASASSSQRGR
>	sp|P04256|RO	Heterogeneous nuclear ribonucleoprotein A1 OS=Rattus norvegicus GN=Hnrnpa1 PE=1 SV=3	GFAFVTFDDHDSVDK
>	sp|P70566|TM	Tropomodulin-2 OS=Rattus norvegicus GN=Tmod2 PE=1 SV=1	SNDPVALAFAEMLK
>	sp|Q920Q0|PA	Paralemmin-1 OS=Rattus norvegicus GN=Palm PE=1 SV=1	AELVVIEDSVTPR
>	sp|Q920Q0|PA	Paralemmin-1 OS=Rattus norvegicus GN=Palm PE=1 SV=1	AELVVIEDSVTPR
>	sp|P17074|RS1	40S ribosomal protein S19 OS=Rattus norvegicus GN=Rps19 PE=2 SV=3	VLQALEGLK
>	sp|Q03555|GE	Gephyrin OS=Rattus norvegicus GN=Gphn PE=1 SV=3	KGSQECFQFILPALPHAIDLLR
>	sp|P17764|THI	Acetyl-CoA acetyltransferase, mitochondrial OS=Rattus norvegicus GN=Acat1 PE=1 SV=1	IVVHLAHALK
>	sp|P17764|THI	Acetyl-CoA acetyltransferase, mitochondrial OS=Rattus norvegicus GN=Acat1 PE=1 SV=1	IVVHLAHALK
>	sp|P17764|THI	Acetyl-CoA acetyltransferase, mitochondrial OS=Rattus norvegicus GN=Acat1 PE=1 SV=1	GKPDVVVKEDEEYKR
>	sp|P40241|CD	CD9 antigen OS=Rattus norvegicus GN=Cd9 PE=1 SV=2	AIHMALNCCGIAGGVEQFISDICPK
>	sp|P40241|CD	CD9 antigen OS=Rattus norvegicus GN=Cd9 PE=1 SV=2	AIHMALNCCGIAGGVEQFISDICPK
>	sp|P40241|CD	CD9 antigen OS=Rattus norvegicus GN=Cd9 PE=1 SV=2	AIHMALNCCGIAGGVEQFISDICPK
>	sp|P40241|CD	CD9 antigen OS=Rattus norvegicus GN=Cd9 PE=1 SV=2	AIHMALNCCGIAGGVEQFISDICPK
>	sp|P40241|CD	CD9 antigen OS=Rattus norvegicus GN=Cd9 PE=1 SV=2	AIHMALNCCGIAGGVEQFISDICPK
>	sp|P40241|CD	CD9 antigen OS=Rattus norvegicus GN=Cd9 PE=1 SV=2	AIHMALNCCGIAGGVEQFISDICPK
>	sp|P69897|TB	Tubulin beta-5 chain OS=Rattus norvegicus GN=Tubb5 PE=1 SV=1	ALTVPELTQQVFDAK
>	sp|P69897|TB	Tubulin beta-5 chain OS=Rattus norvegicus GN=Tubb5 PE=1 SV=1	AILVDLEPGTMDSVR
>	sp|P69897|TB	Tubulin beta-5 chain OS=Rattus norvegicus GN=Tubb5 PE=1 SV=1	AILVDLEPGTMDSVR
>	sp|P69897|TB	Tubulin beta-5 chain OS=Rattus norvegicus GN=Tubb5 PE=1 SV=1	AILVDLEPGTMDSVR
>	sp|P69897|TB	Tubulin beta-5 chain OS=Rattus norvegicus GN=Tubb5 PE=1 SV=1	AILVDLEPGTMDSVR
>	sp|P69897|TB	Tubulin beta-5 chain OS=Rattus norvegicus GN=Tubb5 PE=1 SV=1	AILVDLEPGTMDSVR
>	sp|P69897|TB	Tubulin beta-5 chain OS=Rattus norvegicus GN=Tubb5 PE=1 SV=1	AILVDLEPGTMDSVR
>	sp|P69897|TB	Tubulin beta-5 chain OS=Rattus norvegicus GN=Tubb5 PE=1 SV=1	AILVDLEPGTMDSVR
>	sp|P69897|TB	Tubulin beta-5 chain OS=Rattus norvegicus GN=Tubb5 PE=1 SV=1	AILVDLEPGTMDSVR
>	sp|P69897|TB	Tubulin beta-5 chain OS=Rattus norvegicus GN=Tubb5 PE=1 SV=1	AILVDLEPGTMDSVR
>	sp|P69897|TB	Tubulin beta-5 chain OS=Rattus norvegicus GN=Tubb5 PE=1 SV=1	AILVDLEPGTMDSVR
>	sp|P69897|TB	Tubulin beta-5 chain OS=Rattus norvegicus GN=Tubb5 PE=1 SV=1	AILVDLEPGTMDSVR
>	sp|P69897|TB	Tubulin beta-5 chain OS=Rattus norvegicus GN=Tubb5 PE=1 SV=1	ALTVPELTQQVFDAK
>	sp|P69897|TB	Tubulin beta-5 chain OS=Rattus norvegicus GN=Tubb5 PE=1 SV=1	EIVHIQAGQCGNQIGAK
>	sp|P69897|TB	Tubulin beta-5 chain OS=Rattus norvegicus GN=Tubb5 PE=1 SV=1	EIVHIQAGQCGNQIGAK
>	sp|P69897|TB	Tubulin beta-5 chain OS=Rattus norvegicus GN=Tubb5 PE=1 SV=1	EIVHIQAGQCGNQIGAK
>	sp|P69897|TB	Tubulin beta-5 chain OS=Rattus norvegicus GN=Tubb5 PE=1 SV=1	EIVHIQAGQCGNQIGAK
>	sp|P69897|TB	Tubulin beta-5 chain OS=Rattus norvegicus GN=Tubb5 PE=1 SV=1	EIVHIQAGQCGNQIGAK
>	sp|P69897|TB	Tubulin beta-5 chain OS=Rattus norvegicus GN=Tubb5 PE=1 SV=1	EIVHIQAGQCGNQIGAK
>	sp|P69897|TB	Tubulin beta-5 chain OS=Rattus norvegicus GN=Tubb5 PE=1 SV=1	EIVHIQAGQCGNQIGAK
>	sp|P69897|TB	Tubulin beta-5 chain OS=Rattus norvegicus GN=Tubb5 PE=1 SV=1	EIVHIQAGQCGNQIGAK
>	sp|P69897|TB	Tubulin beta-5 chain OS=Rattus norvegicus GN=Tubb5 PE=1 SV=1	EIVHIQAGQCGNQIGAK
>	sp|P69897|TB	Tubulin beta-5 chain OS=Rattus norvegicus GN=Tubb5 PE=1 SV=1	EIVHIQAGQCGNQIGAK

<table>
<tr><td>></td><td>sp|P69897|TBE Tubulin beta-5 chain OS=Rattus norvegicus GN=Tubb5 PE=1 SV=1</td><td>EIVHIQAGQCGNQIGAK</td></tr>
<tr><td>></td><td>sp|P69897|TBE Tubulin beta-5 chain OS=Rattus norvegicus GN=Tubb5 PE=1 SV=1</td><td>EIVHIQAGQCGNQIGAK</td></tr>
<tr><td>></td><td>sp|P69897|TBE Tubulin beta-5 chain OS=Rattus norvegicus GN=Tubb5 PE=1 SV=1</td><td>EIVHIQAGQCGNQIGAK</td></tr>
<tr><td>></td><td>sp|P69897|TBE Tubulin beta-5 chain OS=Rattus norvegicus GN=Tubb5 PE=1 SV=1</td><td>EIVHIQAGQCGNQIGAK</td></tr>
<tr><td>></td><td>sp|P69897|TBE Tubulin beta-5 chain OS=Rattus norvegicus GN=Tubb5 PE=1 SV=1</td><td>EIVHIQAGQCGNQIGAK</td></tr>
<tr><td>></td><td>sp|P69897|TBE Tubulin beta-5 chain OS=Rattus norvegicus GN=Tubb5 PE=1 SV=1</td><td>EIVHIQAGQCGNQIGAK</td></tr>
<tr><td>></td><td>sp|P69897|TBE Tubulin beta-5 chain OS=Rattus norvegicus GN=Tubb5 PE=1 SV=1</td><td>EIVHIQAGQCGNQIGAK</td></tr>
<tr><td>></td><td>sp|P69897|TBE Tubulin beta-5 chain OS=Rattus norvegicus GN=Tubb5 PE=1 SV=1</td><td>EIVHIQAGQCGNQIGAK</td></tr>
<tr><td>></td><td>sp|P69897|TBE Tubulin beta-5 chain OS=Rattus norvegicus GN=Tubb5 PE=1 SV=1</td><td>EIVHIQAGQCGNQIGAK</td></tr>
<tr><td>></td><td>sp|P69897|TBE Tubulin beta-5 chain OS=Rattus norvegicus GN=Tubb5 PE=1 SV=1</td><td>EIVHIQAGQCGNQIGAK</td></tr>
<tr><td>></td><td>sp|P69897|TBE Tubulin beta-5 chain OS=Rattus norvegicus GN=Tubb5 PE=1 SV=1</td><td>EIVHIQAGQCGNQIGAK</td></tr>
<tr><td>></td><td>sp|P69897|TBE Tubulin beta-5 chain OS=Rattus norvegicus GN=Tubb5 PE=1 SV=1</td><td>EIVHIQAGQCGNQIGAK</td></tr>
<tr><td>></td><td>sp|P69897|TBE Tubulin beta-5 chain OS=Rattus norvegicus GN=Tubb5 PE=1 SV=1</td><td>EIVHIQAGQCGNQIGAK</td></tr>
<tr><td>></td><td>sp|P69897|TBE Tubulin beta-5 chain OS=Rattus norvegicus GN=Tubb5 PE=1 SV=1</td><td>EIVHIQAGQCGNQIGAK</td></tr>
<tr><td>></td><td>sp|P69897|TBE Tubulin beta-5 chain OS=Rattus norvegicus GN=Tubb5 PE=1 SV=1</td><td>EIVHIQAGQCGNQIGAK</td></tr>
<tr><td>></td><td>sp|P69897|TBE Tubulin beta-5 chain OS=Rattus norvegicus GN=Tubb5 PE=1 SV=1</td><td>EIVHIQAGQCGNQIGAK</td></tr>
<tr><td>></td><td>sp|P69897|TBE Tubulin beta-5 chain OS=Rattus norvegicus GN=Tubb5 PE=1 SV=1</td><td>EIVHIQAGQCGNQIGAK</td></tr>
<tr><td>></td><td>sp|P69897|TBE Tubulin beta-5 chain OS=Rattus norvegicus GN=Tubb5 PE=1 SV=1</td><td>EVDEQMLNVQNK</td></tr>
<tr><td>></td><td>sp|P69897|TBE Tubulin beta-5 chain OS=Rattus norvegicus GN=Tubb5 PE=1 SV=1</td><td>EVDEQMLNVQNK</td></tr>
<tr><td>></td><td>sp|P69897|TBE Tubulin beta-5 chain OS=Rattus norvegicus GN=Tubb5 PE=1 SV=1</td><td>EVDEQMLNVQNK</td></tr>
<tr><td>></td><td>sp|P69897|TBE Tubulin beta-5 chain OS=Rattus norvegicus GN=Tubb5 PE=1 SV=1</td><td>EVDEQMLNVQNK</td></tr>
<tr><td>></td><td>sp|P69897|TBE Tubulin beta-5 chain OS=Rattus norvegicus GN=Tubb5 PE=1 SV=1</td><td>FPGQLNADLR</td></tr>
<tr><td>></td><td>sp|P69897|TBE Tubulin beta-5 chain OS=Rattus norvegicus GN=Tubb5 PE=1 SV=1</td><td>FPGQLNADLR</td></tr>
<tr><td>></td><td>sp|P69897|TBE Tubulin beta-5 chain OS=Rattus norvegicus GN=Tubb5 PE=1 SV=1</td><td>FPGQLNADLR</td></tr>
<tr><td>></td><td>sp|P69897|TBE Tubulin beta-5 chain OS=Rattus norvegicus GN=Tubb5 PE=1 SV=1</td><td>FPGQLNADLR</td></tr>
<tr><td>></td><td>sp|P69897|TBE Tubulin beta-5 chain OS=Rattus norvegicus GN=Tubb5 PE=1 SV=1</td><td>FPGQLNADLR</td></tr>
<tr><td>></td><td>sp|P69897|TBE Tubulin beta-5 chain OS=Rattus norvegicus GN=Tubb5 PE=1 SV=1</td><td>FPGQLNADLR</td></tr>
<tr><td>></td><td>sp|P69897|TBE Tubulin beta-5 chain OS=Rattus norvegicus GN=Tubb5 PE=1 SV=1</td><td>FPGQLNADLRK</td></tr>
<tr><td>></td><td>sp|P69897|TBE Tubulin beta-5 chain OS=Rattus norvegicus GN=Tubb5 PE=1 SV=1</td><td>FWEVISDEHGIDPTGTYHGDSDLQLDR</td></tr>
<tr><td>></td><td>sp|P69897|TBE Tubulin beta-5 chain OS=Rattus norvegicus GN=Tubb5 PE=1 SV=1</td><td>GHYTEGAELVDSVLDVVR</td></tr>
<tr><td>></td><td>sp|P69897|TBE Tubulin beta-5 chain OS=Rattus norvegicus GN=Tubb5 PE=1 SV=1</td><td>GHYTEGAELVDSVLDVVR</td></tr>
<tr><td>></td><td>sp|P69897|TBE Tubulin beta-5 chain OS=Rattus norvegicus GN=Tubb5 PE=1 SV=1</td><td>GHYTEGAELVDSVLDVVR</td></tr>
<tr><td>></td><td>sp|P69897|TBE Tubulin beta-5 chain OS=Rattus norvegicus GN=Tubb5 PE=1 SV=1</td><td>GHYTEGAELVDSVLDVVR</td></tr>
<tr><td>></td><td>sp|P69897|TBE Tubulin beta-5 chain OS=Rattus norvegicus GN=Tubb5 PE=1 SV=1</td><td>GHYTEGAELVDSVLDVVR</td></tr>
<tr><td>></td><td>sp|P69897|TBE Tubulin beta-5 chain OS=Rattus norvegicus GN=Tubb5 PE=1 SV=1</td><td>GHYTEGAELVDSVLDVVR</td></tr>
<tr><td>></td><td>sp|P69897|TBE Tubulin beta-5 chain OS=Rattus norvegicus GN=Tubb5 PE=1 SV=1</td><td>GHYTEGAELVDSVLDVVR</td></tr>
<tr><td>></td><td>sp|P69897|TBE Tubulin beta-5 chain OS=Rattus norvegicus GN=Tubb5 PE=1 SV=1</td><td>GHYTEGAELVDSVLDVVR</td></tr>
<tr><td>></td><td>sp|P69897|TBE Tubulin beta-5 chain OS=Rattus norvegicus GN=Tubb5 PE=1 SV=1</td><td>GHYTEGAELVDSVLDVVR</td></tr>
<tr><td>></td><td>sp|P69897|TBE Tubulin beta-5 chain OS=Rattus norvegicus GN=Tubb5 PE=1 SV=1</td><td>GHYTEGAELVDSVLDVVR</td></tr>
<tr><td>></td><td>sp|P69897|TBE Tubulin beta-5 chain OS=Rattus norvegicus GN=Tubb5 PE=1 SV=1</td><td>GHYTEGAELVDSVLDVVR</td></tr>
<tr><td>></td><td>sp|P69897|TBE Tubulin beta-5 chain OS=Rattus norvegicus GN=Tubb5 PE=1 SV=1</td><td>GHYTEGAELVDSVLDVVR</td></tr>
<tr><td>></td><td>sp|P69897|TBE Tubulin beta-5 chain OS=Rattus norvegicus GN=Tubb5 PE=1 SV=1</td><td>GHYTEGAELVDSVLDVVR</td></tr>
<tr><td>></td><td>sp|P69897|TBE Tubulin beta-5 chain OS=Rattus norvegicus GN=Tubb5 PE=1 SV=1</td><td>GHYTEGAELVDSVLDVVR</td></tr>
<tr><td>></td><td>sp|P69897|TBE Tubulin beta-5 chain OS=Rattus norvegicus GN=Tubb5 PE=1 SV=1</td><td>GHYTEGAELVDSVLDVVR</td></tr>
<tr><td>></td><td>sp|P69897|TBE Tubulin beta-5 chain OS=Rattus norvegicus GN=Tubb5 PE=1 SV=1</td><td>GHYTEGAELVDSVLDVVR</td></tr>
<tr><td>></td><td>sp|P69897|TBE Tubulin beta-5 chain OS=Rattus norvegicus GN=Tubb5 PE=1 SV=1</td><td>GHYTEGAELVDSVLDVVR</td></tr>
<tr><td>></td><td>sp|P69897|TBE Tubulin beta-5 chain OS=Rattus norvegicus GN=Tubb5 PE=1 SV=1</td><td>GHYTEGAELVDSVLDVVR</td></tr>
<tr><td>></td><td>sp|P69897|TBE Tubulin beta-5 chain OS=Rattus norvegicus GN=Tubb5 PE=1 SV=1</td><td>GHYTEGAELVDSVLDVVR</td></tr>
<tr><td>></td><td>sp|P69897|TBE Tubulin beta-5 chain OS=Rattus norvegicus GN=Tubb5 PE=1 SV=1</td><td>GHYTEGAELVDSVLDVVR</td></tr>
<tr><td>></td><td>sp|P69897|TBE Tubulin beta-5 chain OS=Rattus norvegicus GN=Tubb5 PE=1 SV=1</td><td>GHYTEGAELVDSVLDVVR</td></tr>
<tr><td>></td><td>sp|P69897|TBE Tubulin beta-5 chain OS=Rattus norvegicus GN=Tubb5 PE=1 SV=1</td><td>GHYTEGAELVDSVLDVVR</td></tr>
<tr><td>></td><td>sp|P69897|TBE Tubulin beta-5 chain OS=Rattus norvegicus GN=Tubb5 PE=1 SV=1</td><td>GHYTEGAELVDSVLDVVR</td></tr>
<tr><td>></td><td>sp|P69897|TBE Tubulin beta-5 chain OS=Rattus norvegicus GN=Tubb5 PE=1 SV=1</td><td>GHYTEGAELVDSVLDVVR</td></tr>
<tr><td>></td><td>sp|P69897|TBE Tubulin beta-5 chain OS=Rattus norvegicus GN=Tubb5 PE=1 SV=1</td><td>GHYTEGAELVDSVLDVVR</td></tr>
<tr><td>></td><td>sp|P69897|TBE Tubulin beta-5 chain OS=Rattus norvegicus GN=Tubb5 PE=1 SV=1</td><td>GHYTEGAELVDSVLDVVR</td></tr>
<tr><td>></td><td>sp|P69897|TBE Tubulin beta-5 chain OS=Rattus norvegicus GN=Tubb5 PE=1 SV=1</td><td>GHYTEGAELVDSVLDVVR</td></tr>
<tr><td>></td><td>sp|P69897|TBE Tubulin beta-5 chain OS=Rattus norvegicus GN=Tubb5 PE=1 SV=1</td><td>GHYTEGAELVDSVLDVVR</td></tr>
<tr><td>></td><td>sp|P69897|TBE Tubulin beta-5 chain OS=Rattus norvegicus GN=Tubb5 PE=1 SV=1</td><td>GHYTEGAELVDSVLDVVR</td></tr>
<tr><td>></td><td>sp|P69897|TBE Tubulin beta-5 chain OS=Rattus norvegicus GN=Tubb5 PE=1 SV=1</td><td>GHYTEGAELVDSVLDVVR</td></tr>
<tr><td>></td><td>sp|P69897|TBE Tubulin beta-5 chain OS=Rattus norvegicus GN=Tubb5 PE=1 SV=1</td><td>GHYTEGAELVDSVLDVVR</td></tr>
<tr><td>></td><td>sp|P69897|TBE Tubulin beta-5 chain OS=Rattus norvegicus GN=Tubb5 PE=1 SV=1</td><td>GHYTEGAELVDSVLDVVR</td></tr>
<tr><td>></td><td>sp|P69897|TBE Tubulin beta-5 chain OS=Rattus norvegicus GN=Tubb5 PE=1 SV=1</td><td>GHYTEGAELVDSVLDVVRK</td></tr>
<tr><td>></td><td>sp|P69897|TBE Tubulin beta-5 chain OS=Rattus norvegicus GN=Tubb5 PE=1 SV=1</td><td>IMNTFSVVPSPK</td></tr>
<tr><td>></td><td>sp|P69897|TBE Tubulin beta-5 chain OS=Rattus norvegicus GN=Tubb5 PE=1 SV=1</td><td>IMNTFSVVPSPK</td></tr>
<tr><td>></td><td>sp|P69897|TBE Tubulin beta-5 chain OS=Rattus norvegicus GN=Tubb5 PE=1 SV=1</td><td>IMNTFSVVPSPK</td></tr>
<tr><td>></td><td>sp|P69897|TBE Tubulin beta-5 chain OS=Rattus norvegicus GN=Tubb5 PE=1 SV=1</td><td>IREEYPDR</td></tr>
<tr><td>></td><td>sp|P69897|TBE Tubulin beta-5 chain OS=Rattus norvegicus GN=Tubb5 PE=1 SV=1</td><td>KEAESCDCLQGFQLTHSLGGGTGSGMGTLLISK</td></tr>
<tr><td>></td><td>sp|P69897|TBE Tubulin beta-5 chain OS=Rattus norvegicus GN=Tubb5 PE=1 SV=1</td><td>KEAESCDCLQGFQLTHSLGGGTGSGMGTLLISK</td></tr>
<tr><td>></td><td>sp|P69897|TBE Tubulin beta-5 chain OS=Rattus norvegicus GN=Tubb5 PE=1 SV=1</td><td>KEAESCDCLQGFQLTHSLGGGTGSGMGTLLISK</td></tr>
<tr><td>></td><td>sp|P69897|TBE Tubulin beta-5 chain OS=Rattus norvegicus GN=Tubb5 PE=1 SV=1</td><td>KEAESCDCLQGFQLTHSLGGGTGSGMGTLLISK</td></tr>
<tr><td>></td><td>sp|P69897|TBE Tubulin beta-5 chain OS=Rattus norvegicus GN=Tubb5 PE=1 SV=1</td><td>KEAESCDCLQGFQLTHSLGGGTGSGMGTLLISK</td></tr>
<tr><td>></td><td>sp|P69897|TBE Tubulin beta-5 chain OS=Rattus norvegicus GN=Tubb5 PE=1 SV=1</td><td>KLAVNMVPFPR</td></tr>
<tr><td>></td><td>sp|P69897|TBE Tubulin beta-5 chain OS=Rattus norvegicus GN=Tubb5 PE=1 SV=1</td><td>KLAVNMVPFPR</td></tr>
<tr><td>></td><td>sp|P69897|TBE Tubulin beta-5 chain OS=Rattus norvegicus GN=Tubb5 PE=1 SV=1</td><td>KLAVNMVPFPR</td></tr>
</table>

>	sp|P69897|TBE Tubulin beta-5 chain OS=Rattus norvegicus GN=Tubb5 PE=1 SV=1	KLAVNMVPFPR
>	sp|P69897|TBE Tubulin beta-5 chain OS=Rattus norvegicus GN=Tubb5 PE=1 SV=1	KLAVNMVPFPR
>	sp|P69897|TBE Tubulin beta-5 chain OS=Rattus norvegicus GN=Tubb5 PE=1 SV=1	KLAVNMVPFPR
>	sp|P69897|TBE Tubulin beta-5 chain OS=Rattus norvegicus GN=Tubb5 PE=1 SV=1	KLAVNMVPFPR
>	sp|P69897|TBE Tubulin beta-5 chain OS=Rattus norvegicus GN=Tubb5 PE=1 SV=1	KLAVNMVPFPR
>	sp|P69897|TBE Tubulin beta-5 chain OS=Rattus norvegicus GN=Tubb5 PE=1 SV=1	KLAVNMVPFPR
>	sp|P69897|TBE Tubulin beta-5 chain OS=Rattus norvegicus GN=Tubb5 PE=1 SV=1	KLAVNMVPFPR
>	sp|P69897|TBE Tubulin beta-5 chain OS=Rattus norvegicus GN=Tubb5 PE=1 SV=1	KLAVNMVPFPR
>	sp|P69897|TBE Tubulin beta-5 chain OS=Rattus norvegicus GN=Tubb5 PE=1 SV=1	KLAVNMVPFPR
>	sp|P69897|TBE Tubulin beta-5 chain OS=Rattus norvegicus GN=Tubb5 PE=1 SV=1	KLAVNMVPFPR
>	sp|P69897|TBE Tubulin beta-5 chain OS=Rattus norvegicus GN=Tubb5 PE=1 SV=1	KLAVNMVPFPR
>	sp|P69897|TBE Tubulin beta-5 chain OS=Rattus norvegicus GN=Tubb5 PE=1 SV=1	KLAVNMVPFPRLHFFMPGFAPLTSR
>	sp|P69897|TBE Tubulin beta-5 chain OS=Rattus norvegicus GN=Tubb5 PE=1 SV=1	KLAVNMVPFPRLHFFMPGFAPLTSR
>	sp|P69897|TBE Tubulin beta-5 chain OS=Rattus norvegicus GN=Tubb5 PE=1 SV=1	KLAVNMVPFPRLHFFMPGFAPLTSR
>	sp|P69897|TBE Tubulin beta-5 chain OS=Rattus norvegicus GN=Tubb5 PE=1 SV=1	LAVNMVPFPR
>	sp|P69897|TBE Tubulin beta-5 chain OS=Rattus norvegicus GN=Tubb5 PE=1 SV=1	LAVNMVPFPR
>	sp|P69897|TBE Tubulin beta-5 chain OS=Rattus norvegicus GN=Tubb5 PE=1 SV=1	LAVNMVPFPR
>	sp|P69897|TBE Tubulin beta-5 chain OS=Rattus norvegicus GN=Tubb5 PE=1 SV=1	LAVNMVPFPR
>	sp|P69897|TBE Tubulin beta-5 chain OS=Rattus norvegicus GN=Tubb5 PE=1 SV=1	LAVNMVPFPR
>	sp|P69897|TBE Tubulin beta-5 chain OS=Rattus norvegicus GN=Tubb5 PE=1 SV=1	LAVNMVPFPR
>	sp|P69897|TBE Tubulin beta-5 chain OS=Rattus norvegicus GN=Tubb5 PE=1 SV=1	LAVNMVPFPR
>	sp|P69897|TBE Tubulin beta-5 chain OS=Rattus norvegicus GN=Tubb5 PE=1 SV=1	LAVNMVPFPR
>	sp|P69897|TBE Tubulin beta-5 chain OS=Rattus norvegicus GN=Tubb5 PE=1 SV=1	LHFFMPGFAPLTSR
>	sp|P69897|TBE Tubulin beta-5 chain OS=Rattus norvegicus GN=Tubb5 PE=1 SV=1	LHFFMPGFAPLTSR
>	sp|P69897|TBE Tubulin beta-5 chain OS=Rattus norvegicus GN=Tubb5 PE=1 SV=1	LHFFMPGFAPLTSR
>	sp|P69897|TBE Tubulin beta-5 chain OS=Rattus norvegicus GN=Tubb5 PE=1 SV=1	LHFFMPGFAPLTSR
>	sp|P69897|TBE Tubulin beta-5 chain OS=Rattus norvegicus GN=Tubb5 PE=1 SV=1	LHFFMPGFAPLTSR
>	sp|P69897|TBE Tubulin beta-5 chain OS=Rattus norvegicus GN=Tubb5 PE=1 SV=1	LHFFMPGFAPLTSR
>	sp|P69897|TBE Tubulin beta-5 chain OS=Rattus norvegicus GN=Tubb5 PE=1 SV=1	LHFFMPGFAPLTSR
>	sp|P69897|TBE Tubulin beta-5 chain OS=Rattus norvegicus GN=Tubb5 PE=1 SV=1	LHFFMPGFAPLTSR
>	sp|P69897|TBE Tubulin beta-5 chain OS=Rattus norvegicus GN=Tubb5 PE=1 SV=1	LHFFMPGFAPLTSR
>	sp|P69897|TBE Tubulin beta-5 chain OS=Rattus norvegicus GN=Tubb5 PE=1 SV=1	LHFFMPGFAPLTSR
>	sp|P69897|TBE Tubulin beta-5 chain OS=Rattus norvegicus GN=Tubb5 PE=1 SV=1	LHFFMPGFAPLTSR
>	sp|P69897|TBE Tubulin beta-5 chain OS=Rattus norvegicus GN=Tubb5 PE=1 SV=1	LHFFMPGFAPLTSR
>	sp|P69897|TBE Tubulin beta-5 chain OS=Rattus norvegicus GN=Tubb5 PE=1 SV=1	LHFFMPGFAPLTSR
>	sp|P69897|TBE Tubulin beta-5 chain OS=Rattus norvegicus GN=Tubb5 PE=1 SV=1	LHFFMPGFAPLTSR
>	sp|P69897|TBE Tubulin beta-5 chain OS=Rattus norvegicus GN=Tubb5 PE=1 SV=1	LHFFMPGFAPLTSR
>	sp|P69897|TBE Tubulin beta-5 chain OS=Rattus norvegicus GN=Tubb5 PE=1 SV=1	LHFFMPGFAPLTSR
>	sp|P69897|TBE Tubulin beta-5 chain OS=Rattus norvegicus GN=Tubb5 PE=1 SV=1	LHFFMPGFAPLTSR
>	sp|P69897|TBE Tubulin beta-5 chain OS=Rattus norvegicus GN=Tubb5 PE=1 SV=1	LHFFMPGFAPLTSR
>	sp|P69897|TBE Tubulin beta-5 chain OS=Rattus norvegicus GN=Tubb5 PE=1 SV=1	LHFFMPGFAPLTSR
>	sp|P69897|TBE Tubulin beta-5 chain OS=Rattus norvegicus GN=Tubb5 PE=1 SV=1	LHFFMPGFAPLTSR
>	sp|P69897|TBE Tubulin beta-5 chain OS=Rattus norvegicus GN=Tubb5 PE=1 SV=1	LHFFMPGFAPLTSR
>	sp|P69897|TBE Tubulin beta-5 chain OS=Rattus norvegicus GN=Tubb5 PE=1 SV=1	LHFFMPGFAPLTSR
>	sp|P69897|TBE Tubulin beta-5 chain OS=Rattus norvegicus GN=Tubb5 PE=1 SV=1	LHFFMPGFAPLTSR
>	sp|P69897|TBE Tubulin beta-5 chain OS=Rattus norvegicus GN=Tubb5 PE=1 SV=1	LHFFMPGFAPLTSR
>	sp|P69897|TBE Tubulin beta-5 chain OS=Rattus norvegicus GN=Tubb5 PE=1 SV=1	LHFFMPGFAPLTSR
>	sp|P69897|TBE Tubulin beta-5 chain OS=Rattus norvegicus GN=Tubb5 PE=1 SV=1	LHFFMPGFAPLTSR
>	sp|P69897|TBE Tubulin beta-5 chain OS=Rattus norvegicus GN=Tubb5 PE=1 SV=1	LHFFMPGFAPLTSR
>	sp|P69897|TBE Tubulin beta-5 chain OS=Rattus norvegicus GN=Tubb5 PE=1 SV=1	LHFFMPGFAPLTSR
>	sp|P69897|TBE Tubulin beta-5 chain OS=Rattus norvegicus GN=Tubb5 PE=1 SV=1	LHFFMPGFAPLTSR
>	sp|P69897|TBE Tubulin beta-5 chain OS=Rattus norvegicus GN=Tubb5 PE=1 SV=1	LHFFMPGFAPLTSR
>	sp|P69897|TBE Tubulin beta-5 chain OS=Rattus norvegicus GN=Tubb5 PE=1 SV=1	LHFFMPGFAPLTSR
>	sp|P69897|TBE Tubulin beta-5 chain OS=Rattus norvegicus GN=Tubb5 PE=1 SV=1	LHFFMPGFAPLTSR
>	sp|P69897|TBE Tubulin beta-5 chain OS=Rattus norvegicus GN=Tubb5 PE=1 SV=1	LHFFMPGFAPLTSR
>	sp|P69897|TBE Tubulin beta-5 chain OS=Rattus norvegicus GN=Tubb5 PE=1 SV=1	LHFFMPGFAPLTSR
>	sp|P69897|TBE Tubulin beta-5 chain OS=Rattus norvegicus GN=Tubb5 PE=1 SV=1	LHFFMPGFAPLTSR
>	sp|P69897|TBE Tubulin beta-5 chain OS=Rattus norvegicus GN=Tubb5 PE=1 SV=1	LHFFMPGFAPLTSR
>	sp|P69897|TBE Tubulin beta-5 chain OS=Rattus norvegicus GN=Tubb5 PE=1 SV=1	LHFFMPGFAPLTSR
>	sp|P69897|TBE Tubulin beta-5 chain OS=Rattus norvegicus GN=Tubb5 PE=1 SV=1	LHFFMPGFAPLTSR
>	sp|P69897|TBE Tubulin beta-5 chain OS=Rattus norvegicus GN=Tubb5 PE=1 SV=1	LHFFMPGFAPLTSR
>	sp|P69897|TBE Tubulin beta-5 chain OS=Rattus norvegicus GN=Tubb5 PE=1 SV=1	LHFFMPGFAPLTSR
>	sp|P69897|TBE Tubulin beta-5 chain OS=Rattus norvegicus GN=Tubb5 PE=1 SV=1	LHFFMPGFAPLTSR
>	sp|P69897|TBE Tubulin beta-5 chain OS=Rattus norvegicus GN=Tubb5 PE=1 SV=1	LHFFMPGFAPLTSR
>	sp|P69897|TBE Tubulin beta-5 chain OS=Rattus norvegicus GN=Tubb5 PE=1 SV=1	LHFFMPGFAPLTSR
>	sp|P69897|TBE Tubulin beta-5 chain OS=Rattus norvegicus GN=Tubb5 PE=1 SV=1	LHFFMPGFAPLTSR
>	sp|P69897|TBE Tubulin beta-5 chain OS=Rattus norvegicus GN=Tubb5 PE=1 SV=1	LHFFMPGFAPLTSR
>	sp|P69897|TBE Tubulin beta-5 chain OS=Rattus norvegicus GN=Tubb5 PE=1 SV=1	LTTPTYGDLNHLVSATMSGVTTCLR
>	sp|P69897|TBE Tubulin beta-5 chain OS=Rattus norvegicus GN=Tubb5 PE=1 SV=1	LTTPTYGDLNHLVSATMSGVTTCLR
>	sp|P69897|TBE Tubulin beta-5 chain OS=Rattus norvegicus GN=Tubb5 PE=1 SV=1	LTTPTYGDLNHLVSATMSGVTTCLR
>	sp|P69897|TBE Tubulin beta-5 chain OS=Rattus norvegicus GN=Tubb5 PE=1 SV=1	LTTPTYGDLNHLVSATMSGVTTCLR
>	sp|P69897|TBE Tubulin beta-5 chain OS=Rattus norvegicus GN=Tubb5 PE=1 SV=1	LTTPTYGDLNHLVSATMSGVTTCLR
>	sp|P69897|TBE Tubulin beta-5 chain OS=Rattus norvegicus GN=Tubb5 PE=1 SV=1	LTTPTYGDLNHLVSATMSGVTTCLR
>	sp|P69897|TBE Tubulin beta-5 chain OS=Rattus norvegicus GN=Tubb5 PE=1 SV=1	LTTPTYGDLNHLVSATMSGVTTCLR

>	sp|P69897|TBE Tubulin beta-5 chain OS=Rattus norvegicus GN=Tubb5 PE=1 SV=1	LTTPTYGDLNHLVSATMSGVTTCLR
>	sp|P69897|TBE Tubulin beta-5 chain OS=Rattus norvegicus GN=Tubb5 PE=1 SV=1	LTTPTYGDLNHLVSATMSGVTTCLR
>	sp|P69897|TBE Tubulin beta-5 chain OS=Rattus norvegicus GN=Tubb5 PE=1 SV=1	LTTPTYGDLNHLVSATMSGVTTCLR
>	sp|P69897|TBE Tubulin beta-5 chain OS=Rattus norvegicus GN=Tubb5 PE=1 SV=1	LTTPTYGDLNHLVSATMSGVTTCLR
>	sp|P69897|TBE Tubulin beta-5 chain OS=Rattus norvegicus GN=Tubb5 PE=1 SV=1	LTTPTYGDLNHLVSATMSGVTTCLR
>	sp|P69897|TBE Tubulin beta-5 chain OS=Rattus norvegicus GN=Tubb5 PE=1 SV=1	LTTPTYGDLNHLVSATMSGVTTCLR
>	sp|P69897|TBE Tubulin beta-5 chain OS=Rattus norvegicus GN=Tubb5 PE=1 SV=1	LTTPTYGDLNHLVSATMSGVTTCLR
>	sp|P69897|TBE Tubulin beta-5 chain OS=Rattus norvegicus GN=Tubb5 PE=1 SV=1	LTTPTYGDLNHLVSATMSGVTTCLR
>	sp|P69897|TBE Tubulin beta-5 chain OS=Rattus norvegicus GN=Tubb5 PE=1 SV=1	LTTPTYGDLNHLVSATMSGVTTCLR
>	sp|P69897|TBE Tubulin beta-5 chain OS=Rattus norvegicus GN=Tubb5 PE=1 SV=1	LTTPTYGDLNHLVSATMSGVTTCLR
>	sp|P69897|TBE Tubulin beta-5 chain OS=Rattus norvegicus GN=Tubb5 PE=1 SV=1	LTTPTYGDLNHLVSATMSGVTTCLR
>	sp|P69897|TBE Tubulin beta-5 chain OS=Rattus norvegicus GN=Tubb5 PE=1 SV=1	LTTPTYGDLNHLVSATMSGVTTCLR
>	sp|P69897|TBE Tubulin beta-5 chain OS=Rattus norvegicus GN=Tubb5 PE=1 SV=1	LTTPTYGDLNHLVSATMSGVTTCLR
>	sp|P69897|TBE Tubulin beta-5 chain OS=Rattus norvegicus GN=Tubb5 PE=1 SV=1	LTTPTYGDLNHLVSATMSGVTTCLR
>	sp|P69897|TBE Tubulin beta-5 chain OS=Rattus norvegicus GN=Tubb5 PE=1 SV=1	LTTPTYGDLNHLVSATMSGVTTCLR
>	sp|P69897|TBE Tubulin beta-5 chain OS=Rattus norvegicus GN=Tubb5 PE=1 SV=1	LTTPTYGDLNHLVSATMSGVTTCLR
>	sp|P69897|TBE Tubulin beta-5 chain OS=Rattus norvegicus GN=Tubb5 PE=1 SV=1	MSMKEVDEQMLNVQNK
>	sp|P69897|TBE Tubulin beta-5 chain OS=Rattus norvegicus GN=Tubb5 PE=1 SV=1	MSMKEVDEQMLNVQNK
>	sp|P69897|TBE Tubulin beta-5 chain OS=Rattus norvegicus GN=Tubb5 PE=1 SV=1	NSSYFVEWIPNNVK
>	sp|P69897|TBE Tubulin beta-5 chain OS=Rattus norvegicus GN=Tubb5 PE=1 SV=1	NSSYFVEWIPNNVK
>	sp|P69897|TBE Tubulin beta-5 chain OS=Rattus norvegicus GN=Tubb5 PE=1 SV=1	NSSYFVEWIPNNVK
>	sp|P69897|TBE Tubulin beta-5 chain OS=Rattus norvegicus GN=Tubb5 PE=1 SV=1	NSSYFVEWIPNNVK
>	sp|P69897|TBE Tubulin beta-5 chain OS=Rattus norvegicus GN=Tubb5 PE=1 SV=1	PGFAPLTSR
>	sp|P69897|TBE Tubulin beta-5 chain OS=Rattus norvegicus GN=Tubb5 PE=1 SV=1	PGQLNADLR
>	sp|P69897|TBE Tubulin beta-5 chain OS=Rattus norvegicus GN=Tubb5 PE=1 SV=1	PGQLNADLRK
>	sp|P69897|TBE Tubulin beta-5 chain OS=Rattus norvegicus GN=Tubb5 PE=1 SV=1	PGTMDSVR
>	sp|P69897|TBE Tubulin beta-5 chain OS=Rattus norvegicus GN=Tubb5 PE=1 SV=1	PGTMDSVR
>	sp|P69897|TBE Tubulin beta-5 chain OS=Rattus norvegicus GN=Tubb5 PE=1 SV=1	SGPFGQIFRPDNFVFGQSGAGNNWAK
>	sp|P69897|TBE Tubulin beta-5 chain OS=Rattus norvegicus GN=Tubb5 PE=1 SV=1	SGPFGQIFRPDNFVFGQSGAGNNWAK
>	sp|P69897|TBE Tubulin beta-5 chain OS=Rattus norvegicus GN=Tubb5 PE=1 SV=1	SGPFGQIFRPDNFVFGQSGAGNNWAK
>	sp|P69897|TBE Tubulin beta-5 chain OS=Rattus norvegicus GN=Tubb5 PE=1 SV=1	SGPFGQIFRPDNFVFGQSGAGNNWAK
>	sp|P69897|TBE Tubulin beta-5 chain OS=Rattus norvegicus GN=Tubb5 PE=1 SV=1	SGPFGQIFRPDNFVFGQSGAGNNWAK
>	sp|P69897|TBE Tubulin beta-5 chain OS=Rattus norvegicus GN=Tubb5 PE=1 SV=1	SGPFGQIFRPDNFVFGQSGAGNNWAK
>	sp|P69897|TBE Tubulin beta-5 chain OS=Rattus norvegicus GN=Tubb5 PE=1 SV=1	SGPFGQIFRPDNFVFGQSGAGNNWAK
>	sp|P69897|TBE Tubulin beta-5 chain OS=Rattus norvegicus GN=Tubb5 PE=1 SV=1	SGPFGQIFRPDNFVFGQSGAGNNWAK
>	sp|P69897|TBE Tubulin beta-5 chain OS=Rattus norvegicus GN=Tubb5 PE=1 SV=1	SGPFGQIFRPDNFVFGQSGAGNNWAK
>	sp|P69897|TBE Tubulin beta-5 chain OS=Rattus norvegicus GN=Tubb5 PE=1 SV=1	SGPFGQIFRPDNFVFGQSGAGNNWAK
>	sp|P69897|TBE Tubulin beta-5 chain OS=Rattus norvegicus GN=Tubb5 PE=1 SV=1	SGPFGQIFRPDNFVFGQSGAGNNWAK
>	sp|P69897|TBE Tubulin beta-5 chain OS=Rattus norvegicus GN=Tubb5 PE=1 SV=1	SGPFGQIFRPDNFVFGQSGAGNNWAK
>	sp|P69897|TBE Tubulin beta-5 chain OS=Rattus norvegicus GN=Tubb5 PE=1 SV=1	SGPFGQIFRPDNFVFGQSGAGNNWAK
>	sp|P69897|TBE Tubulin beta-5 chain OS=Rattus norvegicus GN=Tubb5 PE=1 SV=1	SGPFGQIFRPDNFVFGQSGAGNNWAK
>	sp|P69897|TBE Tubulin beta-5 chain OS=Rattus norvegicus GN=Tubb5 PE=1 SV=1	SGPFGQIFRPDNFVFGQSGAGNNWAK
>	sp|P69897|TBE Tubulin beta-5 chain OS=Rattus norvegicus GN=Tubb5 PE=1 SV=1	SGPFGQIFRPDNFVFGQSGAGNNWAK
>	sp|P69897|TBE Tubulin beta-5 chain OS=Rattus norvegicus GN=Tubb5 PE=1 SV=1	SGPFGQIFRPDNFVFGQSGAGNNWAK
>	sp|P69897|TBE Tubulin beta-5 chain OS=Rattus norvegicus GN=Tubb5 PE=1 SV=1	TAVCDIPPR
>	sp|P69897|TBE Tubulin beta-5 chain OS=Rattus norvegicus GN=Tubb5 PE=1 SV=1	TAVCDIPPR
>	sp|P69897|TBE Tubulin beta-5 chain OS=Rattus norvegicus GN=Tubb5 PE=1 SV=1	TAVCDIPPR
>	sp|P69897|TBE Tubulin beta-5 chain OS=Rattus norvegicus GN=Tubb5 PE=1 SV=1	TAVCDIPPR
>	sp|P69897|TBE Tubulin beta-5 chain OS=Rattus norvegicus GN=Tubb5 PE=1 SV=1	TAVCDIPPR
>	sp|P69897|TBE Tubulin beta-5 chain OS=Rattus norvegicus GN=Tubb5 PE=1 SV=1	TAVCDIPPR
>	sp|P69897|TBE Tubulin beta-5 chain OS=Rattus norvegicus GN=Tubb5 PE=1 SV=1	TAVCDIPPR
>	sp|P69897|TBE Tubulin beta-5 chain OS=Rattus norvegicus GN=Tubb5 PE=1 SV=1	TAVCDIPPR
>	sp|P69897|TBE Tubulin beta-5 chain OS=Rattus norvegicus GN=Tubb5 PE=1 SV=1	TAVCDIPPR
>	sp|P69897|TBE Tubulin beta-5 chain OS=Rattus norvegicus GN=Tubb5 PE=1 SV=1	VSDTVVEPYNATLSVHQLVENTDETYCIDNEALYDICFR
>	sp|P69897|TBE Tubulin beta-5 chain OS=Rattus norvegicus GN=Tubb5 PE=1 SV=1	VSDTVVEPYNATLSVHQLVENTDETYCIDNEALYDICFR
>	sp|P69897|TBE Tubulin beta-5 chain OS=Rattus norvegicus GN=Tubb5 PE=1 SV=1	VSDTVVEPYNATLSVHQLVENTDETYCIDNEALYDICFR
>	sp|P69897|TBE Tubulin beta-5 chain OS=Rattus norvegicus GN=Tubb5 PE=1 SV=1	VSDTVVEPYNATLSVHQLVENTDETYCIDNEALYDICFR
>	sp|P69897|TBE Tubulin beta-5 chain OS=Rattus norvegicus GN=Tubb5 PE=1 SV=1	VSDTVVEPYNATLSVHQLVENTDETYCIDNEALYDICFR
>	sp|P69897|TBE Tubulin beta-5 chain OS=Rattus norvegicus GN=Tubb5 PE=1 SV=1	VSDTVVEPYNATLSVHQLVENTDETYCIDNEALYDICFR
>	sp|P69897|TBE Tubulin beta-5 chain OS=Rattus norvegicus GN=Tubb5 PE=1 SV=1	VSDTVVEPYNATLSVHQLVENTDETYCIDNEALYDICFR
>	sp|P69897|TBE Tubulin beta-5 chain OS=Rattus norvegicus GN=Tubb5 PE=1 SV=1	VSDTVVEPYNATLSVHQLVENTDETYCIDNEALYDICFR
>	sp|P69897|TBE Tubulin beta-5 chain OS=Rattus norvegicus GN=Tubb5 PE=1 SV=1	VSDTVVEPYNATLSVHQLVENTDETYCIDNEALYDICFR
>	sp|P69897|TBE Tubulin beta-5 chain OS=Rattus norvegicus GN=Tubb5 PE=1 SV=1	VSDTVVEPYNATLSVHQLVENTDETYCIDNEALYDICFR
>	sp|P69897|TBE Tubulin beta-5 chain OS=Rattus norvegicus GN=Tubb5 PE=1 SV=1	VSDTVVEPYNATLSVHQLVENTDETYCIDNEALYDICFR
>	sp|P69897|TBE Tubulin beta-5 chain OS=Rattus norvegicus GN=Tubb5 PE=1 SV=1	VSDTVVEPYNATLSVHQLVENTDETYCIDNEALYDICFR
>	sp|P69897|TBE Tubulin beta-5 chain OS=Rattus norvegicus GN=Tubb5 PE=1 SV=1	VSDTVVEPYNATLSVHQLVENTDETYCIDNEALYDICFR
>	sp|P69897|TBE Tubulin beta-5 chain OS=Rattus norvegicus GN=Tubb5 PE=1 SV=1	VSDTVVEPYNATLSVHQLVENTDETYCIDNEALYDICFR
>	sp|P69897|TBE Tubulin beta-5 chain OS=Rattus norvegicus GN=Tubb5 PE=1 SV=1	VSDTVVEPYNATLSVHQLVENTDETYCIDNEALYDICFR
>	sp|P69897|TBE Tubulin beta-5 chain OS=Rattus norvegicus GN=Tubb5 PE=1 SV=1	VSDTVVEPYNATLSVHQLVENTDETYCIDNEALYDICFR
>	sp|P69897|TBE Tubulin beta-5 chain OS=Rattus norvegicus GN=Tubb5 PE=1 SV=1	VSDTVVEPYNATLSVHQLVENTDETYCIDNEALYDICFR
>	sp|P69897|TBE Tubulin beta-5 chain OS=Rattus norvegicus GN=Tubb5 PE=1 SV=1	VSDTVVEPYNATLSVHQLVENTDETYCIDNEALYDICFR
>	sp|P69897|TBE Tubulin beta-5 chain OS=Rattus norvegicus GN=Tubb5 PE=1 SV=1	VSDTVVEPYNATLSVHQLVENTDETYCIDNEALYDICFR
>	sp|P69897|TBE Tubulin beta-5 chain OS=Rattus norvegicus GN=Tubb5 PE=1 SV=1	VSDTVVEPYNATLSVHQLVENTDETYCIDNEALYDICFR
>	sp|P69897|TBE Tubulin beta-5 chain OS=Rattus norvegicus GN=Tubb5 PE=1 SV=1	VSDTVVEPYNATLSVHQLVENTDETYCIDNEALYDICFR

>	sp|P69897|TBE Tubulin beta-5 chain OS=Rattus norvegicus GN=Tubb5 PE=1 SV=1	VSDTVVEPYNATLSVHQLVENTDETYCIDNEALYDICFR
>	sp|P69897|TBE Tubulin beta-5 chain OS=Rattus norvegicus GN=Tubb5 PE=1 SV=1	VSDTVVEPYNATLSVHQLVENTDETYCIDNEALYDICFR
>	sp|P69897|TBE Tubulin beta-5 chain OS=Rattus norvegicus GN=Tubb5 PE=1 SV=1	VSDTVVEPYNATLSVHQLVENTDETYCIDNEALYDICFR
>	sp|P69897|TBE Tubulin beta-5 chain OS=Rattus norvegicus GN=Tubb5 PE=1 SV=1	VSDTVVEPYNATLSVHQLVENTDETYCIDNEALYDICFR
>	sp|P69897|TBE Tubulin beta-5 chain OS=Rattus norvegicus GN=Tubb5 PE=1 SV=1	VSDTVVEPYNATLSVHQLVENTDETYCIDNEALYDICFR
>	sp|P69897|TBE Tubulin beta-5 chain OS=Rattus norvegicus GN=Tubb5 PE=1 SV=1	VSDTVVEPYNATLSVHQLVENTDETYCIDNEALYDICFR
>	sp|P69897|TBE Tubulin beta-5 chain OS=Rattus norvegicus GN=Tubb5 PE=1 SV=1	VSDTVVEPYNATLSVHQLVENTDETYCIDNEALYDICFR
>	sp|P69897|TBE Tubulin beta-5 chain OS=Rattus norvegicus GN=Tubb5 PE=1 SV=1	VSDTVVEPYNATLSVHQLVENTDETYCIDNEALYDICFR
>	sp|P69897|TBE Tubulin beta-5 chain OS=Rattus norvegicus GN=Tubb5 PE=1 SV=1	VSDTVVEPYNATLSVHQLVENTDETYCIDNEALYDICFR
>	sp|P69897|TBE Tubulin beta-5 chain OS=Rattus norvegicus GN=Tubb5 PE=1 SV=1	VSDTVVEPYNATLSVHQLVENTDETYCIDNEALYDICFR
>	sp|P69897|TBE Tubulin beta-5 chain OS=Rattus norvegicus GN=Tubb5 PE=1 SV=1	VSDTVVEPYNATLSVHQLVENTDETYCIDNEALYDICFR
>	sp|P69897|TBE Tubulin beta-5 chain OS=Rattus norvegicus GN=Tubb5 PE=1 SV=1	VSDTVVEPYNATLSVHQLVENTDETYCIDNEALYDICFR
>	sp|P69897|TBE Tubulin beta-5 chain OS=Rattus norvegicus GN=Tubb5 PE=1 SV=1	VSDTVVEPYNATLSVHQLVENTDETYCIDNEALYDICFR
>	sp|P69897|TBE Tubulin beta-5 chain OS=Rattus norvegicus GN=Tubb5 PE=1 SV=1	VSDTVVEPYNATLSVHQLVENTDETYCIDNEALYDICFR
>	sp|P69897|TBE Tubulin beta-5 chain OS=Rattus norvegicus GN=Tubb5 PE=1 SV=1	VSDTVVEPYNATLSVHQLVENTDETYCIDNEALYDICFR
>	sp|P69897|TBE Tubulin beta-5 chain OS=Rattus norvegicus GN=Tubb5 PE=1 SV=1	VSDTVVEPYNATLSVHQLVENTDETYCIDNEALYDICFR
>	sp|P69897|TBE Tubulin beta-5 chain OS=Rattus norvegicus GN=Tubb5 PE=1 SV=1	VSDTVVEPYNATLSVHQLVENTDETYCIDNEALYDICFR
>	sp|P69897|TBE Tubulin beta-5 chain OS=Rattus norvegicus GN=Tubb5 PE=1 SV=1	VSDTVVEPYNATLSVHQLVENTDETYCIDNEALYDICFR
>	sp|P69897|TBE Tubulin beta-5 chain OS=Rattus norvegicus GN=Tubb5 PE=1 SV=1	VSDTVVEPYNATLSVHQLVENTDETYCIDNEALYDICFR
>	sp|P69897|TBE Tubulin beta-5 chain OS=Rattus norvegicus GN=Tubb5 PE=1 SV=1	VSDTVVEPYNATLSVHQLVENTDETYCIDNEALYDICFR
>	sp|P69897|TBE Tubulin beta-5 chain OS=Rattus norvegicus GN=Tubb5 PE=1 SV=1	VSDTVVEPYNATLSVHQLVENTDETYCIDNEALYDICFR
>	sp|P69897|TBE Tubulin beta-5 chain OS=Rattus norvegicus GN=Tubb5 PE=1 SV=1	VSDTVVEPYNATLSVHQLVENTDETYCIDNEALYDICFR
>	sp|P69897|TBE Tubulin beta-5 chain OS=Rattus norvegicus GN=Tubb5 PE=1 SV=1	VSDTVVEPYNATLSVHQLVENTDETYCIDNEALYDICFR
>	sp|P69897|TBE Tubulin beta-5 chain OS=Rattus norvegicus GN=Tubb5 PE=1 SV=1	VSDTVVEPYNATLSVHQLVENTDETYCIDNEALYDICFR
>	sp|P69897|TBE Tubulin beta-5 chain OS=Rattus norvegicus GN=Tubb5 PE=1 SV=1	VSDTVVEPYNATLSVHQLVENTDETYCIDNEALYDICFR
>	sp|P69897|TBE Tubulin beta-5 chain OS=Rattus norvegicus GN=Tubb5 PE=1 SV=1	VSDTVVEPYNATLSVHQLVENTDETYCIDNEALYDICFR
>	sp|P69897|TBE Tubulin beta-5 chain OS=Rattus norvegicus GN=Tubb5 PE=1 SV=1	VSDTVVEPYNATLSVHQLVENTDETYCIDNEALYDICFR
>	sp|P69897|TBE Tubulin beta-5 chain OS=Rattus norvegicus GN=Tubb5 PE=1 SV=1	VSDTVVEPYNATLSVHQLVENTDETYCIDNEALYDICFR
>	sp|P69897|TBE Tubulin beta-5 chain OS=Rattus norvegicus GN=Tubb5 PE=1 SV=1	VSDTVVEPYNATLSVHQLVENTDETYCIDNEALYDICFR
>	sp|P69897|TBE Tubulin beta-5 chain OS=Rattus norvegicus GN=Tubb5 PE=1 SV=1	VSDTVVEPYNATLSVHQLVENTDETYCIDNEALYDICFR
>	sp|P69897|TBE Tubulin beta-5 chain OS=Rattus norvegicus GN=Tubb5 PE=1 SV=1	VSDTVVEPYNATLSVHQLVENTDETYCIDNEALYDICFR
>	sp|P69897|TBE Tubulin beta-5 chain OS=Rattus norvegicus GN=Tubb5 PE=1 SV=1	VSDTVVEPYNATLSVHQLVENTDETYCIDNEALYDICFR
>	sp|P69897|TBE Tubulin beta-5 chain OS=Rattus norvegicus GN=Tubb5 PE=1 SV=1	VSDTVVEPYNATLSVHQLVENTDETYCIDNEALYDICFR
>	sp|P69897|TBE Tubulin beta-5 chain OS=Rattus norvegicus GN=Tubb5 PE=1 SV=1	VSDTVVEPYNATLSVHQLVENTDETYCIDNEALYDICFR
>	sp|P69897|TBE Tubulin beta-5 chain OS=Rattus norvegicus GN=Tubb5 PE=1 SV=1	VSDTVVEPYNATLSVHQLVENTDETYCIDNEALYDICFR
>	sp|P69897|TBE Tubulin beta-5 chain OS=Rattus norvegicus GN=Tubb5 PE=1 SV=1	VSDTVVEPYNATLSVHQLVENTDETYCIDNEALYDICFR
>	sp|P69897|TBE Tubulin beta-5 chain OS=Rattus norvegicus GN=Tubb5 PE=1 SV=1	VSDTVVEPYNATLSVHQLVENTDETYCIDNEALYDICFR
>	sp|P69897|TBE Tubulin beta-5 chain OS=Rattus norvegicus GN=Tubb5 PE=1 SV=1	VSDTVVEPYNATLSVHQLVENTDETYCIDNEALYDICFR
>	sp|P69897|TBE Tubulin beta-5 chain OS=Rattus norvegicus GN=Tubb5 PE=1 SV=1	VSDTVVEPYNATLSVHQLVENTDETYCIDNEALYDICFR
>	sp|P69897|TBE Tubulin beta-5 chain OS=Rattus norvegicus GN=Tubb5 PE=1 SV=1	VSDTVVEPYNATLSVHQLVENTDETYCIDNEALYDICFR
>	sp|P69897|TBE Tubulin beta-5 chain OS=Rattus norvegicus GN=Tubb5 PE=1 SV=1	VSDTVVEPYNATLSVHQLVENTDETYCIDNEALYDICFR
>	sp|P69897|TBE Tubulin beta-5 chain OS=Rattus norvegicus GN=Tubb5 PE=1 SV=1	YLTVAAVFR
>	sp|P69897|TBE Tubulin beta-5 chain OS=Rattus norvegicus GN=Tubb5 PE=1 SV=1	YLTVAAVFR
>	sp|P69897|TBE Tubulin beta-5 chain OS=Rattus norvegicus GN=Tubb5 PE=1 SV=1	YLTVAAVFR
>	sp|P69897|TBE Tubulin beta-5 chain OS=Rattus norvegicus GN=Tubb5 PE=1 SV=1	YLTVAAVFR
>	sp|P69897|TBE Tubulin beta-5 chain OS=Rattus norvegicus GN=Tubb5 PE=1 SV=1	TAVCDIPPR
>	sp|P69897|TBE Tubulin beta-5 chain OS=Rattus norvegicus GN=Tubb5 PE=1 SV=1	NSSYFVEWIPNNVK
>	sp|P69897|TBE Tubulin beta-5 chain OS=Rattus norvegicus GN=Tubb5 PE=1 SV=1	EIVHIQAGQCGNQIGAK
>	sp|P69897|TBE Tubulin beta-5 chain OS=Rattus norvegicus GN=Tubb5 PE=1 SV=1	TAVCDIPPR
>	sp|P69897|TBE Tubulin beta-5 chain OS=Rattus norvegicus GN=Tubb5 PE=1 SV=1	ALTVPELTQQVFDAK
>	sp|P69897|TBE Tubulin beta-5 chain OS=Rattus norvegicus GN=Tubb5 PE=1 SV=1	EIVHIQAGQCGNQIGAK
>	sp|P69897|TBE Tubulin beta-5 chain OS=Rattus norvegicus GN=Tubb5 PE=1 SV=1	LHFFMPGFAPLTSR
>	sp|P69897|TBE Tubulin beta-5 chain OS=Rattus norvegicus GN=Tubb5 PE=1 SV=1	VSDTVVEPYNATLSVHQLVENTDETYCIDNEALYDICFR
>	sp|P69897|TBE Tubulin beta-5 chain OS=Rattus norvegicus GN=Tubb5 PE=1 SV=1	ALTVPELTQQVFDAK
>	sp|P69897|TBE Tubulin beta-5 chain OS=Rattus norvegicus GN=Tubb5 PE=1 SV=1	TAVCDIPPR
>	sp|P69897|TBE Tubulin beta-5 chain OS=Rattus norvegicus GN=Tubb5 PE=1 SV=1	VSDTVVEPYNATLSVHQLVENTDETYCIDNEALYDICFR
>	sp|P69897|TBE Tubulin beta-5 chain OS=Rattus norvegicus GN=Tubb5 PE=1 SV=1	VSDTVVEPYNATLSVHQLVENTDETYCIDNEALYDICFR
>	sp|P69897|TBE Tubulin beta-5 chain OS=Rattus norvegicus GN=Tubb5 PE=1 SV=1	FPGQLNADLR
>	sp|P69897|TBE Tubulin beta-5 chain OS=Rattus norvegicus GN=Tubb5 PE=1 SV=1	KLAVNMVPFPR
>	sp|P69897|TBE Tubulin beta-5 chain OS=Rattus norvegicus GN=Tubb5 PE=1 SV=1	NSSYFVEWIPNNVK
>	sp|P69897|TBE Tubulin beta-5 chain OS=Rattus norvegicus GN=Tubb5 PE=1 SV=1	NSSYFVEWIPNNVK
>	sp|P69897|TBE Tubulin beta-5 chain OS=Rattus norvegicus GN=Tubb5 PE=1 SV=1	LHFFMPGFAPLTSR
>	sp|P69897|TBE Tubulin beta-5 chain OS=Rattus norvegicus GN=Tubb5 PE=1 SV=1	NSSYFVEWIPNNVK
>	sp|P69897|TBE Tubulin beta-5 chain OS=Rattus norvegicus GN=Tubb5 PE=1 SV=1	LHFFMPGFAPLTSR
>	sp|P69897|TBE Tubulin beta-5 chain OS=Rattus norvegicus GN=Tubb5 PE=1 SV=1	GHYTEGAELVDSVLDVVR
>	sp|P69897|TBE Tubulin beta-5 chain OS=Rattus norvegicus GN=Tubb5 PE=1 SV=1	LHFFMPGFAPLTSR
>	sp|P69897|TBE Tubulin beta-5 chain OS=Rattus norvegicus GN=Tubb5 PE=1 SV=1	VSDTVVEPYNATLSVHQLVENTDETYCIDNEALYDICFR
>	sp|P69897|TBE Tubulin beta-5 chain OS=Rattus norvegicus GN=Tubb5 PE=1 SV=1	VSDTVVEPYNATLSVHQLVENTDETYCIDNEALYDICFR
>	sp|P69897|TBE Tubulin beta-5 chain OS=Rattus norvegicus GN=Tubb5 PE=1 SV=1	EIVHIQAGQCGNQIGAK
>	sp|P69897|TBE Tubulin beta-5 chain OS=Rattus norvegicus GN=Tubb5 PE=1 SV=1	GHYTEGAELVDSVLDVVR
>	sp|P69897|TBE Tubulin beta-5 chain OS=Rattus norvegicus GN=Tubb5 PE=1 SV=1	GHYTEGAELVDSVLDVVR
>	sp|P69897|TBE Tubulin beta-5 chain OS=Rattus norvegicus GN=Tubb5 PE=1 SV=1	VSDTVVEPYNATLSVHQLVENTDETYCIDNEALYDICFR
>	sp|P69897|TBE Tubulin beta-5 chain OS=Rattus norvegicus GN=Tubb5 PE=1 SV=1	GHYTEGAELVDSVLDVVR
>	sp|P69897|TBE Tubulin beta-5 chain OS=Rattus norvegicus GN=Tubb5 PE=1 SV=1	VSDTVVEPYNATLSVHQLVENTDETYCIDNEALYDICFR

>	sp|P69897|TBE Tubulin beta-5 chain OS=Rattus norvegicus GN=Tubb5 PE=1 SV=1	VSDTVVEPYNATLSVHQLVENTDETYCIDNEALYDICFR
>	sp|P69897|TBE Tubulin beta-5 chain OS=Rattus norvegicus GN=Tubb5 PE=1 SV=1	AILVDLEPGTMDSVR
>	sp|P69897|TBE Tubulin beta-5 chain OS=Rattus norvegicus GN=Tubb5 PE=1 SV=1	VSDTVVEPYNATLSVHQLVENTDETYCIDNEALYDICFR
>	sp|P69897|TBE Tubulin beta-5 chain OS=Rattus norvegicus GN=Tubb5 PE=1 SV=1	EIVHIQAGQCGNQIGAK
>	sp|P69897|TBE Tubulin beta-5 chain OS=Rattus norvegicus GN=Tubb5 PE=1 SV=1	EVDEQMLNVQNK
>	sp|P69897|TBE Tubulin beta-5 chain OS=Rattus norvegicus GN=Tubb5 PE=1 SV=1	KLAVNMVPFPR
>	sp|P69897|TBE Tubulin beta-5 chain OS=Rattus norvegicus GN=Tubb5 PE=1 SV=1	EAESCDCLQGFQLTHSLGGGTGSGMGTLLISK
>	sp|P69897|TBE Tubulin beta-5 chain OS=Rattus norvegicus GN=Tubb5 PE=1 SV=1	AILVDLEPGTMDSVR
>	sp|P69897|TBE Tubulin beta-5 chain OS=Rattus norvegicus GN=Tubb5 PE=1 SV=1	EIVHIQAGQCGNQIGAK
>	sp|P69897|TBE Tubulin beta-5 chain OS=Rattus norvegicus GN=Tubb5 PE=1 SV=1	EIVHIQAGQCGNQIGAK
>	sp|P69897|TBE Tubulin beta-5 chain OS=Rattus norvegicus GN=Tubb5 PE=1 SV=1	LHFFMPGFAPLTSR
>	sp|P69897|TBE Tubulin beta-5 chain OS=Rattus norvegicus GN=Tubb5 PE=1 SV=1	LAVNMVPFPR
>	sp|P69897|TBE Tubulin beta-5 chain OS=Rattus norvegicus GN=Tubb5 PE=1 SV=1	KLAVNMVPFPR
>	sp|P69897|TBE Tubulin beta-5 chain OS=Rattus norvegicus GN=Tubb5 PE=1 SV=1	PGTMDSVR
>	sp|P69897|TBE Tubulin beta-5 chain OS=Rattus norvegicus GN=Tubb5 PE=1 SV=1	VSDTVVEPYNATLSVHQLVENTDETYCIDNEALYDICFR
>	sp|P69897|TBE Tubulin beta-5 chain OS=Rattus norvegicus GN=Tubb5 PE=1 SV=1	GHYTEGAELVDSVLDVVR
>	sp|P69897|TBE Tubulin beta-5 chain OS=Rattus norvegicus GN=Tubb5 PE=1 SV=1	LTTPTYGDLNHLVSATMSGVTTCLR
>	sp|P69897|TBE Tubulin beta-5 chain OS=Rattus norvegicus GN=Tubb5 PE=1 SV=1	AILVDLEPGTMDSVR
>	sp|P69897|TBE Tubulin beta-5 chain OS=Rattus norvegicus GN=Tubb5 PE=1 SV=1	AILVDLEPGTMDSVR
>	sp|P69897|TBE Tubulin beta-5 chain OS=Rattus norvegicus GN=Tubb5 PE=1 SV=1	VSDTVVEPYNATLSVHQLVENTDETYCIDNEALYDICFR
>	sp|P69897|TBE Tubulin beta-5 chain OS=Rattus norvegicus GN=Tubb5 PE=1 SV=1	SGPFGQIFRPDNFVFGQSGAGNNWAK
>	sp|P69897|TBE Tubulin beta-5 chain OS=Rattus norvegicus GN=Tubb5 PE=1 SV=1	SGPFGQIFRPDNFVFGQSGAGNNWAK
>	sp|P69897|TBE Tubulin beta-5 chain OS=Rattus norvegicus GN=Tubb5 PE=1 SV=1	EIVHIQAGQCGNQIGAK
>	sp|P69897|TBE Tubulin beta-5 chain OS=Rattus norvegicus GN=Tubb5 PE=1 SV=1	KLAVNMVPFPR
>	sp|P69897|TBE Tubulin beta-5 chain OS=Rattus norvegicus GN=Tubb5 PE=1 SV=1	LHFFMPGFAPLTSR
>	sp|P69897|TBE Tubulin beta-5 chain OS=Rattus norvegicus GN=Tubb5 PE=1 SV=1	KLAVNMVPFPRLHFFMPGFAPLTSR
>	sp|P69897|TBE Tubulin beta-5 chain OS=Rattus norvegicus GN=Tubb5 PE=1 SV=1	FPGQLNADLRK
>	sp|P69897|TBE Tubulin beta-5 chain OS=Rattus norvegicus GN=Tubb5 PE=1 SV=1	KLAVNMVPFPR
>	sp|P69897|TBE Tubulin beta-5 chain OS=Rattus norvegicus GN=Tubb5 PE=1 SV=1	KLAVNMVPFPR
>	sp|P69897|TBE Tubulin beta-5 chain OS=Rattus norvegicus GN=Tubb5 PE=1 SV=1	LAVNMVPFPR
>	sp|P69897|TBE Tubulin beta-5 chain OS=Rattus norvegicus GN=Tubb5 PE=1 SV=1	KLAVNMVPFPR
>	sp|P69897|TBE Tubulin beta-5 chain OS=Rattus norvegicus GN=Tubb5 PE=1 SV=1	KLAVNMVPFPR
>	sp|P69897|TBE Tubulin beta-5 chain OS=Rattus norvegicus GN=Tubb5 PE=1 SV=1	GHYTEGAELVDSVLDVVR
>	sp|P69897|TBE Tubulin beta-5 chain OS=Rattus norvegicus GN=Tubb5 PE=1 SV=1	LTTPTYGDLNHLVSATMSGVTTCLR
>	sp|P69897|TBE Tubulin beta-5 chain OS=Rattus norvegicus GN=Tubb5 PE=1 SV=1	KLAVNMVPFPR
>	sp|P69897|TBE Tubulin beta-5 chain OS=Rattus norvegicus GN=Tubb5 PE=1 SV=1	LAVNMVPFPR
>	sp|P69897|TBE Tubulin beta-5 chain OS=Rattus norvegicus GN=Tubb5 PE=1 SV=1	VSDTVVEPYNATLSVHQLVENTDETYCIDNEALYDICFR
>	sp|P69897|TBE Tubulin beta-5 chain OS=Rattus norvegicus GN=Tubb5 PE=1 SV=1	SGPFGQIFRPDNFVFGQSGAGNNWAK
>	sp|P69897|TBE Tubulin beta-5 chain OS=Rattus norvegicus GN=Tubb5 PE=1 SV=1	VSDTVVEPYNATLSVHQLVENTDETYCIDNEALYDICFR
>	sp|P69897|TBE Tubulin beta-5 chain OS=Rattus norvegicus GN=Tubb5 PE=1 SV=1	VSDTVVEPYNATLSVHQLVENTDETYCIDNEALYDICFR
>	sp|P69897|TBE Tubulin beta-5 chain OS=Rattus norvegicus GN=Tubb5 PE=1 SV=1	LHFFMPGFAPLTSR
>	sp|P69897|TBE Tubulin beta-5 chain OS=Rattus norvegicus GN=Tubb5 PE=1 SV=1	GHYTEGAELVDSVLDVVR
>	sp|P69897|TBE Tubulin beta-5 chain OS=Rattus norvegicus GN=Tubb5 PE=1 SV=1	EIVHIQAGQCGNQIGAK
>	sp|P69897|TBE Tubulin beta-5 chain OS=Rattus norvegicus GN=Tubb5 PE=1 SV=1	KLAVNMVPFPR
>	sp|P69897|TBE Tubulin beta-5 chain OS=Rattus norvegicus GN=Tubb5 PE=1 SV=1	KLAVNMVPFPRLHFFMPGFAPLTSR
>	sp|P69897|TBE Tubulin beta-5 chain OS=Rattus norvegicus GN=Tubb5 PE=1 SV=1	TAVCDIPPR
>	sp|P69897|TBE Tubulin beta-5 chain OS=Rattus norvegicus GN=Tubb5 PE=1 SV=1	EIVHIQAGQCGNQIGAK
>	sp|P69897|TBE Tubulin beta-5 chain OS=Rattus norvegicus GN=Tubb5 PE=1 SV=1	LHFFMPGFAPLTSR
>	sp|P69897|TBE Tubulin beta-5 chain OS=Rattus norvegicus GN=Tubb5 PE=1 SV=1	VSDTVVEPYNATLSVHQLVENTDETYCIDNEALYDICFR
>	sp|P69897|TBE Tubulin beta-5 chain OS=Rattus norvegicus GN=Tubb5 PE=1 SV=1	FPGQLNADLR
>	sp|P69897|TBE Tubulin beta-5 chain OS=Rattus norvegicus GN=Tubb5 PE=1 SV=1	AILVDLE
>	sp|P69897|TBE Tubulin beta-5 chain OS=Rattus norvegicus GN=Tubb5 PE=1 SV=1	VSDTVVEPYNATLSVHQLVENTDETYCIDNEALYDICFR
>	sp|P69897|TBE Tubulin beta-5 chain OS=Rattus norvegicus GN=Tubb5 PE=1 SV=1	IMNTFSVVPSPK
>	sp|P69897|TBE Tubulin beta-5 chain OS=Rattus norvegicus GN=Tubb5 PE=1 SV=1	VSDTVVEPYNATLSVHQLVENTDETYCIDNEALYDICFR
>	sp|P69897|TBE Tubulin beta-5 chain OS=Rattus norvegicus GN=Tubb5 PE=1 SV=1	LTTPTYGDLNHLVSATMSGVTTCLR
>	sp|P69897|TBE Tubulin beta-5 chain OS=Rattus norvegicus GN=Tubb5 PE=1 SV=1	YLTVAAVFR
>	sp|P69897|TBE Tubulin beta-5 chain OS=Rattus norvegicus GN=Tubb5 PE=1 SV=1	KLAVNMVPFPR
>	sp|P69897|TBE Tubulin beta-5 chain OS=Rattus norvegicus GN=Tubb5 PE=1 SV=1	EIVHIQAGQCGNQIGAK
>	sp|P69897|TBE Tubulin beta-5 chain OS=Rattus norvegicus GN=Tubb5 PE=1 SV=1	NSSYFVEWIPNNVK
>	sp|P69897|TBE Tubulin beta-5 chain OS=Rattus norvegicus GN=Tubb5 PE=1 SV=1	VSDTVVEPYNATLSVHQLVENTDETYCIDNEALYDICFR
>	sp|P69897|TBE Tubulin beta-5 chain OS=Rattus norvegicus GN=Tubb5 PE=1 SV=1	VSDTVVEPYNATLSVHQLVENTDETYCIDNEALYDICFR
>	sp|P69897|TBE Tubulin beta-5 chain OS=Rattus norvegicus GN=Tubb5 PE=1 SV=1	IMNTFSVVPSPK
>	sp|P69897|TBE Tubulin beta-5 chain OS=Rattus norvegicus GN=Tubb5 PE=1 SV=1	NSSYFVEWIPNNVK
>	sp|P69897|TBE Tubulin beta-5 chain OS=Rattus norvegicus GN=Tubb5 PE=1 SV=1	NMMAACDPR
>	sp|P69897|TBE Tubulin beta-5 chain OS=Rattus norvegicus GN=Tubb5 PE=1 SV=1	KEAESCDCLQGFQLTHSLGGGTGSGMGTLLISK
>	sp|P69897|TBE Tubulin beta-5 chain OS=Rattus norvegicus GN=Tubb5 PE=1 SV=1	NSSYFVEWIPNNVK
>	sp|P69897|TBE Tubulin beta-5 chain OS=Rattus norvegicus GN=Tubb5 PE=1 SV=1	KLAVNMVPFPR
>	sp|P69897|TBE Tubulin beta-5 chain OS=Rattus norvegicus GN=Tubb5 PE=1 SV=1	ISEQFTAMFR
>	sp|P69897|TBE Tubulin beta-5 chain OS=Rattus norvegicus GN=Tubb5 PE=1 SV=1	LTTPTYGDLNHLVSATMSGVTTCLR
>	sp|P69897|TBE Tubulin beta-5 chain OS=Rattus norvegicus GN=Tubb5 PE=1 SV=1	VSDTVVEPYNATLSVHQLVENTDETYCIDNEALYDICFR
>	sp|P69897|TBE Tubulin beta-5 chain OS=Rattus norvegicus GN=Tubb5 PE=1 SV=1	TAVCDIPPR
>	sp|P69897|TBE Tubulin beta-5 chain OS=Rattus norvegicus GN=Tubb5 PE=1 SV=1	VSDTVVEPYNATLSVHQLVENTDETYCIDNEALYDICFR
>	sp|P69897|TBE Tubulin beta-5 chain OS=Rattus norvegicus GN=Tubb5 PE=1 SV=1	SGPFGQIFRPDNFVFGQSGAGNNWAK
>	sp|P69897|TBE Tubulin beta-5 chain OS=Rattus norvegicus GN=Tubb5 PE=1 SV=1	EIVHIQAGQCGNQIGAK

>	sp|P69897|TBE	Tubulin beta-5 chain OS=Rattus norvegicus GN=Tubb5 PE=1 SV=1	FPGQLNADLR
>	sp|P69897|TBE	Tubulin beta-5 chain OS=Rattus norvegicus GN=Tubb5 PE=1 SV=1	KLAVNMVPFPR
>	sp|P69897|TBE	Tubulin beta-5 chain OS=Rattus norvegicus GN=Tubb5 PE=1 SV=1	LTTPTYGDLNHLVSATMSGVTTCLR
>	sp|P69897|TBE	Tubulin beta-5 chain OS=Rattus norvegicus GN=Tubb5 PE=1 SV=1	TAVCDIPPR
>	sp|P69897|TBE	Tubulin beta-5 chain OS=Rattus norvegicus GN=Tubb5 PE=1 SV=1	EIVHIQAGQCGNQIGAK
>	sp|P69897|TBE	Tubulin beta-5 chain OS=Rattus norvegicus GN=Tubb5 PE=1 SV=1	LTTPTYGDLNHLVSATMSGVTTCLR
>	sp|P69897|TBE	Tubulin beta-5 chain OS=Rattus norvegicus GN=Tubb5 PE=1 SV=1	EIVHIQAGQCGNQIGAK
>	sp|P69897|TBE	Tubulin beta-5 chain OS=Rattus norvegicus GN=Tubb5 PE=1 SV=1	KLAVNMVPFPR
>	sp|P69897|TBE	Tubulin beta-5 chain OS=Rattus norvegicus GN=Tubb5 PE=1 SV=1	EIVHIQAGQCGNQIGAK
>	sp|P69897|TBE	Tubulin beta-5 chain OS=Rattus norvegicus GN=Tubb5 PE=1 SV=1	EVDEQMLNVQNK
>	sp|P69897|TBE	Tubulin beta-5 chain OS=Rattus norvegicus GN=Tubb5 PE=1 SV=1	VSDTVVEPYNATLSVHQLVENTDETYCIDNEALYDICFR
>	sp|P69897|TBE	Tubulin beta-5 chain OS=Rattus norvegicus GN=Tubb5 PE=1 SV=1	VSDTVVEPYNATLSVHQLVENTDETYCIDNEALYDICFR
>	sp|P69897|TBE	Tubulin beta-5 chain OS=Rattus norvegicus GN=Tubb5 PE=1 SV=1	TAVCDIPPR
>	sp|P69897|TBE	Tubulin beta-5 chain OS=Rattus norvegicus GN=Tubb5 PE=1 SV=1	FWEVISDEHGIDPTGTYHGDSDLQLDR
>	sp|P69897|TBE	Tubulin beta-5 chain OS=Rattus norvegicus GN=Tubb5 PE=1 SV=1	LTTPTYGDLNHLVSATMSGVTTCLR
>	sp|P69897|TBE	Tubulin beta-5 chain OS=Rattus norvegicus GN=Tubb5 PE=1 SV=1	EIVHIQAGQCGNQIGAK
>	sp|P69897|TBE	Tubulin beta-5 chain OS=Rattus norvegicus GN=Tubb5 PE=1 SV=1	VSDTVVEPYNATLSVHQLVENTDETYCIDNEALYDICFR
>	sp|P69897|TBE	Tubulin beta-5 chain OS=Rattus norvegicus GN=Tubb5 PE=1 SV=1	FPGQLNADLR
>	sp|P69897|TBE	Tubulin beta-5 chain OS=Rattus norvegicus GN=Tubb5 PE=1 SV=1	KLAVNMVPFPR
>	sp|P69897|TBE	Tubulin beta-5 chain OS=Rattus norvegicus GN=Tubb5 PE=1 SV=1	YLTVAAVFR
>	sp|P69897|TBE	Tubulin beta-5 chain OS=Rattus norvegicus GN=Tubb5 PE=1 SV=1	MSMKEVDEQMLNVQNK
>	sp|P69897|TBE	Tubulin beta-5 chain OS=Rattus norvegicus GN=Tubb5 PE=1 SV=1	NSSYFVEWIPNNVK
>	sp|P69897|TBE	Tubulin beta-5 chain OS=Rattus norvegicus GN=Tubb5 PE=1 SV=1	KLAVNMVPFPR
>	sp|P69897|TBE	Tubulin beta-5 chain OS=Rattus norvegicus GN=Tubb5 PE=1 SV=1	KLAVNMVPFPR
>	sp|P69897|TBE	Tubulin beta-5 chain OS=Rattus norvegicus GN=Tubb5 PE=1 SV=1	KLAVNMVPFPR
>	sp|P69897|TBE	Tubulin beta-5 chain OS=Rattus norvegicus GN=Tubb5 PE=1 SV=1	EVDEQMLNVQNK
>	sp|P69897|TBE	Tubulin beta-5 chain OS=Rattus norvegicus GN=Tubb5 PE=1 SV=1	VSDTVVEPYNATLSVHQLVENTDETYCIDNEALYDICFR
>	sp|P69897|TBE	Tubulin beta-5 chain OS=Rattus norvegicus GN=Tubb5 PE=1 SV=1	GHYTEGAELVDSVLDVVR
>	sp|P69897|TBE	Tubulin beta-5 chain OS=Rattus norvegicus GN=Tubb5 PE=1 SV=1	LTTPTYGDLNHLVSATMSGVTTCLR
>	sp|P69897|TBE	Tubulin beta-5 chain OS=Rattus norvegicus GN=Tubb5 PE=1 SV=1	FWEVISDEHGIDPTGTYHGDSDLQLDR
>	sp|P69897|TBE	Tubulin beta-5 chain OS=Rattus norvegicus GN=Tubb5 PE=1 SV=1	VVPSPKVSDTVVEPYNATLSVHQLVENTDETYCIDNEALYDICFR
>	sp|P69897|TBE	Tubulin beta-5 chain OS=Rattus norvegicus GN=Tubb5 PE=1 SV=1	LTTPTYGDLNHLVSATMSGVTTCLR
>	sp|P69897|TBE	Tubulin beta-5 chain OS=Rattus norvegicus GN=Tubb5 PE=1 SV=1	ALTVPELTQQVFDAK
>	sp|Q5U2N3|PI	Membrane-associated phosphatidylinositol transfer protein 1 OS=Rattus norvegicus GN=Pitpnm1 PE=2 SV=1	VHFPEALGHVALR
>	sp|Q5U2N3|PI	Membrane-associated phosphatidylinositol transfer protein 1 OS=Rattus norvegicus GN=Pitpnm1 PE=2 SV=1	VHFPEALGHVALR
>	sp|Q5XHZ0|TR	Heat shock protein 75 kDa, mitochondrial OS=Rattus norvegicus GN=Trap1 PE=1 SV=1	GVVDSEDIPLNLSR
>	sp|Q5XHZ0|TR	Heat shock protein 75 kDa, mitochondrial OS=Rattus norvegicus GN=Trap1 PE=1 SV=1	GVVDSEDIPLNLSR
>	sp|Q5XHZ0|TR	Heat shock protein 75 kDa, mitochondrial OS=Rattus norvegicus GN=Trap1 PE=1 SV=1	GVVDSEDIPLNLSR
>	sp|Q5XHZ0|TR	Heat shock protein 75 kDa, mitochondrial OS=Rattus norvegicus GN=Trap1 PE=1 SV=1	GVVDSEDIPLNLSR
>	sp|P04906|GST	Glutathione S-transferase P OS=Rattus norvegicus GN=Gstp1 PE=1 SV=2	FEDGDLTLYQSNAILR
>	sp|P63088|PP1	Serine/threonine-protein phosphatase PP1-gamma catalytic subunit OS=Rattus norvegicus GN=Ppp1cc PE=1 SV=1	FLHKHDLDLICR
>	sp|P63088|PP1	Serine/threonine-protein phosphatase PP1-gamma catalytic subunit OS=Rattus norvegicus GN=Ppp1cc PE=1 SV=1	EIFLSQPILLELEAPLK
>	sp|P63088|PP1	Serine/threonine-protein phosphatase PP1-gamma catalytic subunit OS=Rattus norvegicus GN=Ppp1cc PE=1 SV=1	FLHKHDLDLICR
>	sp|P63088|PP1	Serine/threonine-protein phosphatase PP1-gamma catalytic subunit OS=Rattus norvegicus GN=Ppp1cc PE=1 SV=1	AHQVVEDGYEFFAK
>	sp|P41565|IDH	Isocitrate dehydrogenase [NAD] subunit gamma 1, mitochondrial OS=Rattus norvegicus GN=Idh3g PE=2 SV=2	KAVLASMDNENMHTPDIGGQGTTSQAIQDIIR
>	sp|P41565|IDH	Isocitrate dehydrogenase [NAD] subunit gamma 1, mitochondrial OS=Rattus norvegicus GN=Idh3g PE=2 SV=2	KAVLASMDNENMHTPDIGGQGTTSQAIQDIIR
>	sp|P41565|IDH	Isocitrate dehydrogenase [NAD] subunit gamma 1, mitochondrial OS=Rattus norvegicus GN=Idh3g PE=2 SV=2	KAVLASMDNENMHTPDIGGQGTTSQAIQDIIR
>	sp|P41565|IDH	Isocitrate dehydrogenase [NAD] subunit gamma 1, mitochondrial OS=Rattus norvegicus GN=Idh3g PE=2 SV=2	KAVLASMDNENMHTPDIGGQGTTSQAIQDIIR
>	sp|P41565|IDH	Isocitrate dehydrogenase [NAD] subunit gamma 1, mitochondrial OS=Rattus norvegicus GN=Idh3g PE=2 SV=2	HTVTMIPGDGIGPELMLHVK
>	sp|P28075|PSB	Proteasome subunit beta type-5 OS=Rattus norvegicus GN=Psmb5 PE=1 SV=3	FQHGVIVAADSR
>	sp|P28075|PSB	Proteasome subunit beta type-5 OS=Rattus norvegicus GN=Psmb5 PE=1 SV=3	FQHGVIVAADSR
>	sp|P30835|K6P	6-phosphofructokinase, liver type OS=Rattus norvegicus GN=Pfkl PE=2 SV=3	IMEVIDAITTTAQSHQR
>	sp|P30835|K6P	6-phosphofructokinase, liver type OS=Rattus norvegicus GN=Pfkl PE=2 SV=3	IMEVIDAITTTAQSHQR
>	sp|P30835|K6P	6-phosphofructokinase, liver type OS=Rattus norvegicus GN=Pfkl PE=2 SV=3	IMEVIDAITTTAQSHQR
>	sp|P30835|K6P	6-phosphofructokinase, liver type OS=Rattus norvegicus GN=Pfkl PE=2 SV=3	VTVLGHVQR
>	sp|P30835|K6P	6-phosphofructokinase, liver type OS=Rattus norvegicus GN=Pfkl PE=2 SV=3	IMEVIDAITTTAQSHQR
>	sp|P62744|AP2	AP-2 complex subunit sigma OS=Rattus norvegicus GN=Ap2s1 PE=2 SV=1	LIEEVHAVVTVR
>	sp|P62744|AP2	AP-2 complex subunit sigma OS=Rattus norvegicus GN=Ap2s1 PE=2 SV=1	LIEEVHAVVTVR
>	sp|P62744|AP2	AP-2 complex subunit sigma OS=Rattus norvegicus GN=Ap2s1 PE=2 SV=1	LIEEVHAVVTVR
>	sp|P62744|AP2	AP-2 complex subunit sigma OS=Rattus norvegicus GN=Ap2s1 PE=2 SV=1	LIEEVHAVVTVR
>	sp|P62744|AP2	AP-2 complex subunit sigma OS=Rattus norvegicus GN=Ap2s1 PE=2 SV=1	LIEEVHAVVTVR
>	sp|P62744|AP2	AP-2 complex subunit sigma OS=Rattus norvegicus GN=Ap2s1 PE=2 SV=1	LIEEVHAVVTVR
>	sp|P97685|NFA	Neurofascin OS=Rattus norvegicus GN=Nfasc PE=1 SV=2	TSGAPPESNPSDVKGEGTR
>	sp|P97685|NFA	Neurofascin OS=Rattus norvegicus GN=Nfasc PE=1 SV=2	TSGAPPESNPSDVKGEGTR
>	sp|O35952|GLO	Hydroxyacylglutathione hydrolase, mitochondrial OS=Rattus norvegicus GN=Hagh PE=1 SV=2	TVQQHAGETDPVTTMR
>	sp|Q9QXU9|PC	ProSAAS OS=Rattus norvegicus GN=Pcsk1n PE=1 SV=1	ALAHLLEAER
>	sp|Q9QXU9|PC	ProSAAS OS=Rattus norvegicus GN=Pcsk1n PE=1 SV=1	ALAHLLEAER
>	sp|Q9QXU9|PC	ProSAAS OS=Rattus norvegicus GN=Pcsk1n PE=1 SV=1	ALAHLLEAER
>	sp|Q9QXU9|PC	ProSAAS OS=Rattus norvegicus GN=Pcsk1n PE=1 SV=1	ALAHLLEAER
>	sp|Q9QXU9|PC	ProSAAS OS=Rattus norvegicus GN=Pcsk1n PE=1 SV=1	ALAHLLEAER
>	sp|Q9QXU9|PC	ProSAAS OS=Rattus norvegicus GN=Pcsk1n PE=1 SV=1	ALAHLLEAER
>	sp|Q64119|MY	Myosin light polypeptide 6 OS=Rattus norvegicus GN=Myl6 PE=1 SV=3	VLDFEHFLPMLQTVAK
>	sp|Q64119|MY	Myosin light polypeptide 6 OS=Rattus norvegicus GN=Myl6 PE=1 SV=3	VLDFEHFLPMLQTVAK
>	sp|Q64119|MY	Myosin light polypeptide 6 OS=Rattus norvegicus GN=Myl6 PE=1 SV=3	VLDFEHFLPMLQTVAK

>	sp|Q5M7A7|CN	CB1 cannabinoid receptor-interacting protein 1 OS=Rattus norvegicus GN=Cnrip1 PE=1 SV=1	IKPTTLQVENISIGGVLVPLELK
>	sp|Q5M7A7|CN	CB1 cannabinoid receptor-interacting protein 1 OS=Rattus norvegicus GN=Cnrip1 PE=1 SV=1	IKPTTLQVENISIGGVLVPLELK
>	sp|Q5M7A7|CN	CB1 cannabinoid receptor-interacting protein 1 OS=Rattus norvegicus GN=Cnrip1 PE=1 SV=1	IKPTTLQVENISIGGVLVPLELK
>	sp|Q5M7A7|CN	CB1 cannabinoid receptor-interacting protein 1 OS=Rattus norvegicus GN=Cnrip1 PE=1 SV=1	IKPTTLQVENISIGGVLVPLELK
>	sp|Q5M7A7|CN	CB1 cannabinoid receptor-interacting protein 1 OS=Rattus norvegicus GN=Cnrip1 PE=1 SV=1	IKPTTLQVENISIGGVLVPLELK
>	sp|Q5M7A7|CN	CB1 cannabinoid receptor-interacting protein 1 OS=Rattus norvegicus GN=Cnrip1 PE=1 SV=1	IKPTTLQVENISIGGVLVPLELK
>	sp|P0C0S7|H2A	Histone H2A.Z OS=Rattus norvegicus GN=H2afz PE=1 SV=2	ATIAGGGVIPHIHK
>	sp|P0C0S7|H2A	Histone H2A.Z OS=Rattus norvegicus GN=H2afz PE=1 SV=2	ATIAGGGVIPHIHK
>	sp|P0C0S7|H2A	Histone H2A.Z OS=Rattus norvegicus GN=H2afz PE=1 SV=2	ATIAGGGVIPHIHK
>	sp|P0C0S7|H2A	Histone H2A.Z OS=Rattus norvegicus GN=H2afz PE=1 SV=2	ATIAGGGVIPHIHK
>	sp|P0C0S7|H2A	Histone H2A.Z OS=Rattus norvegicus GN=H2afz PE=1 SV=2	AGLQFPVGR
>	sp|P0C0S7|H2A	Histone H2A.Z OS=Rattus norvegicus GN=H2afz PE=1 SV=2	AGLQFPVGR
>	sp|P0C0S7|H2A	Histone H2A.Z OS=Rattus norvegicus GN=H2afz PE=1 SV=2	AGLQFPVGR
>	sp|P05508|NU	NADH-ubiquinone oxidoreductase chain 4 OS=Rattus norvegicus GN=Mtnd4 PE=2 SV=2	ELTLMALHIIPLMLLTINPK
>	sp|P05508|NU	NADH-ubiquinone oxidoreductase chain 4 OS=Rattus norvegicus GN=Mtnd4 PE=2 SV=2	ELTLMALHIIPLMLLTINPK
>	sp|P05508|NU	NADH-ubiquinone oxidoreductase chain 4 OS=Rattus norvegicus GN=Mtnd4 PE=2 SV=2	ELTLMALHIIPLMLLTINPK
>	sp|P05508|NU	NADH-ubiquinone oxidoreductase chain 4 OS=Rattus norvegicus GN=Mtnd4 PE=2 SV=2	IHSRTMIMARGLQMIFP
>	sp|P27605|HPI	Hypoxanthine-guanine phosphoribosyltransferase OS=Rattus norvegicus GN=Hprt1 PE=1 SV=1	VFIPHGLIMDR
>	sp|P27605|HPI	Hypoxanthine-guanine phosphoribosyltransferase OS=Rattus norvegicus GN=Hprt1 PE=1 SV=1	VFIPHGLIMDR
>	sp|P27605|HPI	Hypoxanthine-guanine phosphoribosyltransferase OS=Rattus norvegicus GN=Hprt1 PE=1 SV=1	VFIPHGLIMDR
>	sp|P27605|HPI	Hypoxanthine-guanine phosphoribosyltransferase OS=Rattus norvegicus GN=Hprt1 PE=1 SV=1	VFIPHGLIMDR
>	sp|P27605|HPI	Hypoxanthine-guanine phosphoribosyltransferase OS=Rattus norvegicus GN=Hprt1 PE=1 SV=1	VFIPHGLIMDR
>	sp|P47875|CSR	Cysteine and glycine-rich protein 1 OS=Rattus norvegicus GN=Csrp1 PE=2 SV=2	GLESTTLADKDGEIYCK
>	sp|P62747|RH	Rho-related GTP-binding protein RhoB OS=Rattus norvegicus GN=Rhob PE=1 SV=1	HFCPNVPIILVANK
>	sp|P62747|RH	Rho-related GTP-binding protein RhoB OS=Rattus norvegicus GN=Rhob PE=1 SV=1	QVELALWDTAGQEDYDR
>	sp|O35964|SH	Endophilin-A2 OS=Rattus norvegicus GN=Sh3gl1 PE=1 SV=1	QAVQILEELADKLK
>	sp|O35964|SH	Endophilin-A2 OS=Rattus norvegicus GN=Sh3gl1 PE=1 SV=1	QAVQILEELADKLK
>	sp|Q08163|CAI	Adenylyl cyclase-associated protein 1 OS=Rattus norvegicus GN=Cap1 PE=1 SV=3	KEPALLELEGKK
>	sp|Q64428|ECI	Trifunctional enzyme subunit alpha, mitochondrial OS=Rattus norvegicus GN=Hadha PE=1 SV=2	ADMVIEAVFEDLAVK
>	sp|Q64428|ECI	Trifunctional enzyme subunit alpha, mitochondrial OS=Rattus norvegicus GN=Hadha PE=1 SV=2	ADMVIEAVFEDLAVK
>	sp|Q5GFD9|IM	Protein IMPACT OS=Rattus norvegicus GN=Impact PE=2 SV=1	ISESAPEAEELPPIAHGAPITDRR
>	sp|Q5GFD9|IM	Protein IMPACT OS=Rattus norvegicus GN=Impact PE=2 SV=1	ISESAPEAEELPPIAHGAPITDRR
>	sp|P85971|6PC	6-phosphogluconolactonase OS=Rattus norvegicus GN=Pgls PE=1 SV=1	VTLTLPVLNAAQSVIFVATGEGK
>	sp|Q5XI22|THI	Acetyl-CoA acetyltransferase, cytosolic OS=Rattus norvegicus GN=Acat2 PE=1 SV=1	ILVTLLHTLER
>	sp|Q5XI22|THI	Acetyl-CoA acetyltransferase, cytosolic OS=Rattus norvegicus GN=Acat2 PE=1 SV=1	ILVTLLHTLER
>	sp|Q5XI22|THI	Acetyl-CoA acetyltransferase, cytosolic OS=Rattus norvegicus GN=Acat2 PE=1 SV=1	ILVTLLHTLER
>	sp|Q5XI22|THI	Acetyl-CoA acetyltransferase, cytosolic OS=Rattus norvegicus GN=Acat2 PE=1 SV=1	ILVTLLHTLER
>	sp|Q8R431|MC	Monoglyceride lipase OS=Rattus norvegicus GN=Mgll PE=1 SV=1	ELPEVTNSVLHEINTWVSHR
>	sp|Q8R431|MC	Monoglyceride lipase OS=Rattus norvegicus GN=Mgll PE=1 SV=1	ELPEVTNSVLHEINTWVSHR
>	sp|Q8R431|MC	Monoglyceride lipase OS=Rattus norvegicus GN=Mgll PE=1 SV=1	ELPEVTNSVLHEINTWVSHR
>	sp|P12711|ADI	Alcohol dehydrogenase class-3 OS=Rattus norvegicus GN=Adh5 PE=1 SV=2	AAVAWEAGKPLSIEEIEVAPPQAHEVR
>	sp|P12711|ADI	Alcohol dehydrogenase class-3 OS=Rattus norvegicus GN=Adh5 PE=1 SV=2	AAVAWEAGKPLSIEEIEVAPPQAHEVR
>	sp|O35567|PUI	Bifunctional purine biosynthesis protein PURH OS=Rattus norvegicus GN=Atic PE=1 SV=2	TLHPAVHAGILAR
>	sp|O35567|PUI	Bifunctional purine biosynthesis protein PURH OS=Rattus norvegicus GN=Atic PE=1 SV=2	TLHPAVHAGILAR
>	sp|Q6DGG0|PF	Peptidyl-prolyl cis-trans isomerase D OS=Rattus norvegicus GN=Ppid PE=1 SV=3	IVLELFADIVPK
>	sp|Q6PDV7|RL	60S ribosomal protein L10 OS=Rattus norvegicus GN=Rpl10 PE=1 SV=3	VHIGQVIMSIR
>	sp|Q6PDV7|RL	60S ribosomal protein L10 OS=Rattus norvegicus GN=Rpl10 PE=1 SV=3	VHIGQVIMSIR
>	sp|Q6PDV7|RL	60S ribosomal protein L10 OS=Rattus norvegicus GN=Rpl10 PE=1 SV=3	VHIGQVIMSIR
>	sp|P23978|SC6	Sodium- and chloride-dependent GABA transporter 1 OS=Rattus norvegicus GN=Slc6a1 PE=1 SV=1	VADGQISTEVSEAPVASDKPK
>	sp|P23978|SC6	Sodium- and chloride-dependent GABA transporter 1 OS=Rattus norvegicus GN=Slc6a1 PE=1 SV=1	VADGQISTEVSEAPVASDKPK
>	sp|Q6AYQ8|FA	Fumarylacetoacetate hydrolase domain-containing protein 1 OS=Rattus norvegicus GN=Fahd1 PE=2 SV=1	NLHHEVELGVLLGR
>	sp|Q6AYQ8|FA	Fumarylacetoacetate hydrolase domain-containing protein 1 OS=Rattus norvegicus GN=Fahd1 PE=2 SV=1	NLHHEVELGVLLGR
>	sp|Q6AYQ8|FA	Fumarylacetoacetate hydrolase domain-containing protein 1 OS=Rattus norvegicus GN=Fahd1 PE=2 SV=1	NLHHEVELGVLLGR
>	sp|P18422|PSA	Proteasome subunit alpha type-3 OS=Rattus norvegicus GN=Psma3 PE=1 SV=3	HVGMAVAGLLADAR
>	sp|P18422|PSA	Proteasome subunit alpha type-3 OS=Rattus norvegicus GN=Psma3 PE=1 SV=3	HVGMAVAGLLADAR
>	sp|P18418|CAL	Calreticulin OS=Rattus norvegicus GN=Calr PE=1 SV=1	AKIDDPTDSKPEDWDKPEHIPDPDAK
>	sp|P18418|CAL	Calreticulin OS=Rattus norvegicus GN=Calr PE=1 SV=1	AKIDDPTDSKPEDWDKPEHIPDPDAK
>	sp|P00787|CAT	Cathepsin B OS=Rattus norvegicus GN=Ctsb PE=1 SV=2	HEAGDVMGGHAIR
>	sp|Q9ES40|PRA	PRA1 family protein 3 OS=Rattus norvegicus GN=Arl6ip5 PE=1 SV=1	KTPMGIILDALEQQEDSINK
>	sp|Q63362|ND	NADH dehydrogenase [ubiquinone] 1 alpha subcomplex subunit 5 OS=Rattus norvegicus GN=Ndufa5 PE=1 SV=3	KLENLLQGGEVEEVILQAEK
>	sp|Q63362|ND	NADH dehydrogenase [ubiquinone] 1 alpha subcomplex subunit 5 OS=Rattus norvegicus GN=Ndufa5 PE=1 SV=3	KLENLLQGGEVEEVILQAEK
>	sp|Q63362|ND	NADH dehydrogenase [ubiquinone] 1 alpha subcomplex subunit 5 OS=Rattus norvegicus GN=Ndufa5 PE=1 SV=3	KLENLLQGGEVEEVILQAEK
>	sp|Q63362|ND	NADH dehydrogenase [ubiquinone] 1 alpha subcomplex subunit 5 OS=Rattus norvegicus GN=Ndufa5 PE=1 SV=3	KLENLLQGGEVEEVILQAEK
>	sp|Q63362|ND	NADH dehydrogenase [ubiquinone] 1 alpha subcomplex subunit 5 OS=Rattus norvegicus GN=Ndufa5 PE=1 SV=3	KLENLLQGGEVEEVILQAEK
>	sp|Q63362|ND	NADH dehydrogenase [ubiquinone] 1 alpha subcomplex subunit 5 OS=Rattus norvegicus GN=Ndufa5 PE=1 SV=3	KLENLLQGGEVEEVILQAEK
>	sp|Q63362|ND	NADH dehydrogenase [ubiquinone] 1 alpha subcomplex subunit 5 OS=Rattus norvegicus GN=Ndufa5 PE=1 SV=3	KLENLLQGGEVEEVILQAEK
>	sp|Q63362|ND	NADH dehydrogenase [ubiquinone] 1 alpha subcomplex subunit 5 OS=Rattus norvegicus GN=Ndufa5 PE=1 SV=3	KLENLLQGGEVEEVILQAEK
>	sp|Q04940|NEI	Neurogranin OS=Rattus norvegicus GN=Nrgn PE=1 SV=1	KGPGPGGPGGAGGAR
>	sp|Q04940|NEI	Neurogranin OS=Rattus norvegicus GN=Nrgn PE=1 SV=1	KGPGPGGPGGAGGAR
>	sp|Q04940|NEI	Neurogranin OS=Rattus norvegicus GN=Nrgn PE=1 SV=1	KGPGPGGPGGAGGAR
>	sp|Q04940|NEI	Neurogranin OS=Rattus norvegicus GN=Nrgn PE=1 SV=1	KGPGPGGPGGAGGAR
>	sp|Q04940|NEI	Neurogranin OS=Rattus norvegicus GN=Nrgn PE=1 SV=1	KGPGPGGPGGAGGAR
>	sp|Q04940|NEI	Neurogranin OS=Rattus norvegicus GN=Nrgn PE=1 SV=1	KGPGPGGPGGAGGAR
>	sp|Q04940|NEI	Neurogranin OS=Rattus norvegicus GN=Nrgn PE=1 SV=1	KGPGPGGPGGAGGAR

>	sp|Q04940|NE	Neurogranin OS=Rattus norvegicus GN=Nrgn PE=1 SV=1	KGPGPGGPGGAGGAR
>	sp|Q04940|NE	Neurogranin OS=Rattus norvegicus GN=Nrgn PE=1 SV=1	KGPGPGGPGGAGGAR
>	sp|Q04940|NE	Neurogranin OS=Rattus norvegicus GN=Nrgn PE=1 SV=1	KGPGPGGPGGAGGAR
>	sp|Q04940|NE	Neurogranin OS=Rattus norvegicus GN=Nrgn PE=1 SV=1	KGPGPGGPGGAGGAR
>	sp|Q04940|NE	Neurogranin OS=Rattus norvegicus GN=Nrgn PE=1 SV=1	KGPGPGGPGGAGGAR
>	sp|Q04940|NE	Neurogranin OS=Rattus norvegicus GN=Nrgn PE=1 SV=1	KGPGPGGPGGAGGAR
>	sp|P07483|FAB	Fatty acid-binding protein, heart OS=Rattus norvegicus GN=Fabp3 PE=1 SV=2	LILTLTHGNVVSTR
>	sp|Q3T1J1|IF5	Eukaryotic translation initiation factor 5A-1 OS=Rattus norvegicus GN=Eif5a PE=1 SV=3	VHLVGIDIFTGK
>	sp|Q3T1J1|IF5	Eukaryotic translation initiation factor 5A-1 OS=Rattus norvegicus GN=Eif5a PE=1 SV=3	VHLVGIDIFTGK
>	sp|Q3T1J1|IF5	Eukaryotic translation initiation factor 5A-1 OS=Rattus norvegicus GN=Eif5a PE=1 SV=3	VHLVGIDIFTGK
>	sp|Q3T1J1|IF5	Eukaryotic translation initiation factor 5A-1 OS=Rattus norvegicus GN=Eif5a PE=1 SV=3	VHLVGIDIFTGK
>	sp|Q3T1J1|IF5	Eukaryotic translation initiation factor 5A-1 OS=Rattus norvegicus GN=Eif5a PE=1 SV=3	VHLVGIDIFTGK
>	sp|Q3T1J1|IF5	Eukaryotic translation initiation factor 5A-1 OS=Rattus norvegicus GN=Eif5a PE=1 SV=3	VHLVGIDIFTGK
>	sp|Q3T1J1|IF5	Eukaryotic translation initiation factor 5A-1 OS=Rattus norvegicus GN=Eif5a PE=1 SV=3	VHLVGIDIFTGK
>	sp|Q3T1J1|IF5	Eukaryotic translation initiation factor 5A-1 OS=Rattus norvegicus GN=Eif5a PE=1 SV=3	VHLVGIDIFTGK
>	sp|Q3T1J1|IF5	Eukaryotic translation initiation factor 5A-1 OS=Rattus norvegicus GN=Eif5a PE=1 SV=3	VHLVGIDIFTGK
>	sp|Q3T1J1|IF5	Eukaryotic translation initiation factor 5A-1 OS=Rattus norvegicus GN=Eif5a PE=1 SV=3	VHLVGIDIFTGK
>	sp|Q3T1J1|IF5	Eukaryotic translation initiation factor 5A-1 OS=Rattus norvegicus GN=Eif5a PE=1 SV=3	VHLVGIDIFTGK
>	sp|Q3T1J1|IF5	Eukaryotic translation initiation factor 5A-1 OS=Rattus norvegicus GN=Eif5a PE=1 SV=3	VHLVGIDIFTGK
>	sp|P00762|TRY	Anionic trypsin-1 OS=Rattus norvegicus GN=Prss1 PE=1 SV=1	LGEHNINVLEGDEQFINAAK
>	sp|P00762|TRY	Anionic trypsin-1 OS=Rattus norvegicus GN=Prss1 PE=1 SV=1	LGEHNINVLEGDEQFINAAK
>	sp|P00762|TRY	Anionic trypsin-1 OS=Rattus norvegicus GN=Prss1 PE=1 SV=1	LGEHNINVLEGDEQFINAAK
>	sp|P00762|TRY	Anionic trypsin-1 OS=Rattus norvegicus GN=Prss1 PE=1 SV=1	LGEHNINVLEGDEQFINAAK
>	sp|P00762|TRY	Anionic trypsin-1 OS=Rattus norvegicus GN=Prss1 PE=1 SV=1	LGEHNINVLEGDEQFINAAK
>	sp|P00762|TRY	Anionic trypsin-1 OS=Rattus norvegicus GN=Prss1 PE=1 SV=1	LGEHNINVLEGDEQFINAAK
>	sp|P00762|TRY	Anionic trypsin-1 OS=Rattus norvegicus GN=Prss1 PE=1 SV=1	LGEHNINVLEGDEQFINAAK
>	sp|P00762|TRY	Anionic trypsin-1 OS=Rattus norvegicus GN=Prss1 PE=1 SV=1	LGEHNINVLEGDEQFINAAK
>	sp|P00762|TRY	Anionic trypsin-1 OS=Rattus norvegicus GN=Prss1 PE=1 SV=1	LGEHNINVLEGDEQFINAAK
>	sp|P00762|TRY	Anionic trypsin-1 OS=Rattus norvegicus GN=Prss1 PE=1 SV=1	LGEHNINVLEGDEQFINAAK
>	sp|P00762|TRY	Anionic trypsin-1 OS=Rattus norvegicus GN=Prss1 PE=1 SV=1	LGEHNINVLEGDEQFINAAK
>	sp|P00762|TRY	Anionic trypsin-1 OS=Rattus norvegicus GN=Prss1 PE=1 SV=1	LGEHNINVLEGDEQFINAAK
>	sp|P00762|TRY	Anionic trypsin-1 OS=Rattus norvegicus GN=Prss1 PE=1 SV=1	LGEHNINVLEGDEQFINAAK
>	sp|P00762|TRY	Anionic trypsin-1 OS=Rattus norvegicus GN=Prss1 PE=1 SV=1	LGEHNINVLEGDEQFINAAK
>	sp|P00762|TRY	Anionic trypsin-1 OS=Rattus norvegicus GN=Prss1 PE=1 SV=1	LGEHNINVLEGDEQFINAAK
>	sp|P00762|TRY	Anionic trypsin-1 OS=Rattus norvegicus GN=Prss1 PE=1 SV=1	LGEHNINVLEGDEQFINAAK
>	sp|P00762|TRY	Anionic trypsin-1 OS=Rattus norvegicus GN=Prss1 PE=1 SV=1	LGEHNINVLEGDEQFINAAK
>	sp|P00762|TRY	Anionic trypsin-1 OS=Rattus norvegicus GN=Prss1 PE=1 SV=1	LGEHNINVLEGDEQFINAAK
>	sp|P00762|TRY	Anionic trypsin-1 OS=Rattus norvegicus GN=Prss1 PE=1 SV=1	LGEHNINVLEGDEQFINAAK
>	sp|P00762|TRY	Anionic trypsin-1 OS=Rattus norvegicus GN=Prss1 PE=1 SV=1	LGEHNINVLEGDEQFINAAK
>	sp|P00762|TRY	Anionic trypsin-1 OS=Rattus norvegicus GN=Prss1 PE=1 SV=1	LGEHNINVLEGDEQFINAAK
>	sp|P00762|TRY	Anionic trypsin-1 OS=Rattus norvegicus GN=Prss1 PE=1 SV=1	LGEHNINVLEGDEQFINAAK
>	sp|P00762|TRY	Anionic trypsin-1 OS=Rattus norvegicus GN=Prss1 PE=1 SV=1	LGEHNINVLEGDEQFINAAK
>	sp|P00762|TRY	Anionic trypsin-1 OS=Rattus norvegicus GN=Prss1 PE=1 SV=1	LGEHNINVLEGDEQFINAAK
>	sp|P00762|TRY	Anionic trypsin-1 OS=Rattus norvegicus GN=Prss1 PE=1 SV=1	LGEHNINVLEGDEQFINAAK
>	sp|P00762|TRY	Anionic trypsin-1 OS=Rattus norvegicus GN=Prss1 PE=1 SV=1	LGEHNINVLEGDEQFINAAK
>	sp|P00762|TRY	Anionic trypsin-1 OS=Rattus norvegicus GN=Prss1 PE=1 SV=1	LGEHNINVLEGDEQFINAAKIIKHPNYSSWTLNNDIMLIK
>	sp|P00762|TRY	Anionic trypsin-1 OS=Rattus norvegicus GN=Prss1 PE=1 SV=1	LGEHNINVLEGDEQFINAAKIIKHPNYSSWTLNNDIMLIK
>	sp|P00762|TRY	Anionic trypsin-1 OS=Rattus norvegicus GN=Prss1 PE=1 SV=1	LGEHNINVLEGDEQFINAAK
>	sp|P00762|TRY	Anionic trypsin-1 OS=Rattus norvegicus GN=Prss1 PE=1 SV=1	LGEHNINVLEGDEQFINAAK
>	sp|P19944|RLA	60S acidic ribosomal protein P1 OS=Rattus norvegicus GN=Rplp1 PE=3 SV=1	AAGVNVEPFWPGLFAK
>	sp|P19944|RLA	60S acidic ribosomal protein P1 OS=Rattus norvegicus GN=Rplp1 PE=3 SV=1	AAGVNVEPFWPGLFAK
>	sp|Q9WVA1|TI	Mitochondrial import inner membrane translocase subunit Tim8 A OS=Rattus norvegicus GN=Timm8a PE=1 SV=1	FQQLVHQMTELCWEK
>	sp|P61972|NTF	Nuclear transport factor 2 OS=Rattus norvegicus GN=Nutf2 PE=1 SV=1	ADEDPIMGFHQMFLLK
>	sp|P61972|NTF	Nuclear transport factor 2 OS=Rattus norvegicus GN=Nutf2 PE=1 SV=1	ADEDPIMGFHQMFLLK
>	sp|P41498|PPA	Low molecular weight phosphotyrosine protein phosphatase OS=Rattus norvegicus GN=Acp1 PE=1 SV=3	SVLFVCLGNICR
>	sp|Q01062|PD	cGMP-dependent 3',5'-cyclic phosphodiesterase OS=Rattus norvegicus GN=Pde2a PE=1 SV=2	VLGEEVSFPLTMGR
>	sp|P29101|SYT	Synaptotagmin-2 OS=Rattus norvegicus GN=Syt2 PE=1 SV=1	DLQGGEKEEPEKLGDICTSLR
>	sp|P29101|SYT	Synaptotagmin-2 OS=Rattus norvegicus GN=Syt2 PE=1 SV=1	VQVVVTVLDYDK
>	sp|P29101|SYT	Synaptotagmin-2 OS=Rattus norvegicus GN=Syt2 PE=1 SV=1	VQVVVTVLDYDK
>	sp|P29101|SYT	Synaptotagmin-2 OS=Rattus norvegicus GN=Syt2 PE=1 SV=1	VQVVVTVLDYDK
>	sp|P29101|SYT	Synaptotagmin-2 OS=Rattus norvegicus GN=Syt2 PE=1 SV=1	VQVVVTVLDYDK
>	sp|P39069|KA	Adenylate kinase isoenzyme 1 OS=Rattus norvegicus GN=Ak1 PE=1 SV=3	IIFVVGGPGSGK
>	sp|P39069|KA	Adenylate kinase isoenzyme 1 OS=Rattus norvegicus GN=Ak1 PE=1 SV=3	IIFVVGGPGSGK
>	sp|P39069|KA	Adenylate kinase isoenzyme 1 OS=Rattus norvegicus GN=Ak1 PE=1 SV=3	KVNAEGSVDTVFSQVCTYLDSLK
>	sp|P39069|KA	Adenylate kinase isoenzyme 1 OS=Rattus norvegicus GN=Ak1 PE=1 SV=3	KVNAEGSVDTVFSQVCTYLDSLK
>	sp|P39069|KA	Adenylate kinase isoenzyme 1 OS=Rattus norvegicus GN=Ak1 PE=1 SV=3	IIFVVGGPGSGK
>	sp|P39069|KA	Adenylate kinase isoenzyme 1 OS=Rattus norvegicus GN=Ak1 PE=1 SV=3	KVNAEGSVDTVFSQVCTYLDSLK
>	sp|P97710|SH	Tyrosine-protein phosphatase non-receptor type substrate 1 OS=Rattus norvegicus GN=Sirpa PE=1 SV=1	LSPEDIHSR
>	sp|Q63570|PR	26S protease regulatory subunit 6B OS=Rattus norvegicus GN=Psmc4 PE=1 SV=1	ENAPAIIFIDEIDAIATK
>	sp|P20651|PP2	Serine/threonine-protein phosphatase 2B catalytic subunit beta isoform OS=Rattus norvegicus GN=Ppp3cb PE=2 SV=1	GLTPTGMLPSGVLAGGR
>	sp|P20651|PP2	Serine/threonine-protein phosphatase 2B catalytic subunit beta isoform OS=Rattus norvegicus GN=Ppp3cb PE=2 SV=1	AHEAQDAGYR
>	sp|P20651|PP2	Serine/threonine-protein phosphatase 2B catalytic subunit beta isoform OS=Rattus norvegicus GN=Ppp3cb PE=2 SV=1	VFSVLREESESVLTLK
>	sp|P20651|PP2	Serine/threonine-protein phosphatase 2B catalytic subunit beta isoform OS=Rattus norvegicus GN=Ppp3cb PE=2 SV=1	VFSVLREESESVLTLK
>	sp|P20651|PP2	Serine/threonine-protein phosphatase 2B catalytic subunit beta isoform OS=Rattus norvegicus GN=Ppp3cb PE=2 SV=1	VFSVLREESESVLTLK

>	sp|P20651|PP2	Serine/threonine-protein phosphatase 2B catalytic subunit beta isoform OS=Rattus norvegicus GN=Ppp3cb PE=2 SV=1	VFSVLREESESVLTLK
>	sp|P20651|PP2	Serine/threonine-protein phosphatase 2B catalytic subunit beta isoform OS=Rattus norvegicus GN=Ppp3cb PE=2 SV=1	LFEVGGSPANTR
>	sp|P20651|PP2	Serine/threonine-protein phosphatase 2B catalytic subunit beta isoform OS=Rattus norvegicus GN=Ppp3cb PE=2 SV=1	VFSVLREESESVLTLK
>	sp|P20651|PP2	Serine/threonine-protein phosphatase 2B catalytic subunit beta isoform OS=Rattus norvegicus GN=Ppp3cb PE=2 SV=1	AHEAQDAGYR
>	sp|P20651|PP2	Serine/threonine-protein phosphatase 2B catalytic subunit beta isoform OS=Rattus norvegicus GN=Ppp3cb PE=2 SV=1	AHEAQDAGYR
>	sp|P20651|PP2	Serine/threonine-protein phosphatase 2B catalytic subunit beta isoform OS=Rattus norvegicus GN=Ppp3cb PE=2 SV=1	EESESVLTLK
>	sp|P20651|PP2	Serine/threonine-protein phosphatase 2B catalytic subunit beta isoform OS=Rattus norvegicus GN=Ppp3cb PE=2 SV=1	KSQTTGFPSLITIFSAPNYLDVYNNK
>	sp|P04905|GST	Glutathione S-transferase Mu 1 OS=Rattus norvegicus GN=Gstm1 PE=1 SV=2	KHHLCGETEEER
>	sp|P04905|GST	Glutathione S-transferase Mu 1 OS=Rattus norvegicus GN=Gstm1 PE=1 SV=2	KHHLCGETEEER
>	sp|Q8CFD0|SF	Sideroflexin-5 OS=Rattus norvegicus GN=Sfxn5 PE=2 SV=1	HALLETALTR
>	sp|Q8CFD0|SF	Sideroflexin-5 OS=Rattus norvegicus GN=Sfxn5 PE=2 SV=1	HALLETALTR
>	sp|Q8CFD0|SF	Sideroflexin-5 OS=Rattus norvegicus GN=Sfxn5 PE=2 SV=1	HALLETALTR
>	sp|Q6MG55|AI	Abhydrolase domain-containing protein 16A OS=Rattus norvegicus GN=Abhd16a PE=2 SV=1	GVALLRPEPLHR
>	sp|Q9Z0V6|PRI	Thioredoxin-dependent peroxide reductase, mitochondrial OS=Rattus norvegicus GN=Prdx3 PE=1 SV=2	AFQFVETHGEVCPANWTPESPTIKPSPTASK
>	sp|P62703|RS4	40S ribosomal protein S4, X isoform OS=Rattus norvegicus GN=Rps4x PE=2 SV=2	LRECLPLIIFLR
>	sp|P62703|RS4	40S ribosomal protein S4, X isoform OS=Rattus norvegicus GN=Rps4x PE=2 SV=2	LRECLPLIIFLR
>	RRRRRsp|P705(	REVERSED CDP-diacylglycerol--inositol 3-phosphatidyltransferase OS=Rattus norvegicus GN=Cdipt PE=1 SV=1	AATVLHIVSIISK
>	sp|P80254|DOI	D-dopachrome decarboxylase OS=Rattus norvegicus GN=Ddt PE=1 SV=3	LCAATATILDKPEDR
>	sp|P80254|DOI	D-dopachrome decarboxylase OS=Rattus norvegicus GN=Ddt PE=1 SV=3	FLTEELSLDQDR
>	sp|P80254|DOI	D-dopachrome decarboxylase OS=Rattus norvegicus GN=Ddt PE=1 SV=3	FFPLEPWQIGK
>	sp|P48679|LMI	Prelamin-A/C OS=Rattus norvegicus GN=Lmna PE=1 SV=1	LKDLEALLNSK
>	sp|Q8CGU4|AG	Arf-GAP with GTPase, ANK repeat and PH domain-containing protein 2 OS=Rattus norvegicus GN=Agap2 PE=1 SV=1	LGVLGDVRSGK
>	sp|P35465|PAI	Serine/threonine-protein kinase PAK 1 OS=Rattus norvegicus GN=Pak1 PE=1 SV=3	ECLQALEFLHSNQVIHR
>	sp|P35465|PAI	Serine/threonine-protein kinase PAK 1 OS=Rattus norvegicus GN=Pak1 PE=1 SV=3	ECLQALEFLHSNQVIHR
>	sp|P35465|PAI	Serine/threonine-protein kinase PAK 1 OS=Rattus norvegicus GN=Pak1 PE=1 SV=3	IAKPLSSLTPLIAAAK
>	sp|P63326|RS1	40S ribosomal protein S10 OS=Rattus norvegicus GN=Rps10 PE=2 SV=1	IAIYELLFK
>	sp|P35571|GPI	Glycerol-3-phosphate dehydrogenase, mitochondrial OS=Rattus norvegicus GN=Gpd2 PE=1 SV=1	VIFFLPWEK
>	sp|Q9JK11|RTN	Reticulon-4 OS=Rattus norvegicus GN=Rtn4 PE=1 SV=1	HQVQIDHYLGLANK
>	sp|Q9WVJ4|SY	Synaptojanin-2-binding protein OS=Rattus norvegicus GN=Synj2bp PE=1 SV=2	NLLHQDAVDLFR
>	sp|P04550|PTN	Parathymosin OS=Rattus norvegicus GN=Ptms PE=1 SV=2	SVEAAAELSAK
>	sp|Q78P75|DYI	Dynein light chain 2, cytoplasmic OS=Rattus norvegicus GN=Dynll2 PE=1 SV=1	NADMSEDMQQDAVDCATQAMEK
>	sp|Q78P75|DYI	Dynein light chain 2, cytoplasmic OS=Rattus norvegicus GN=Dynll2 PE=1 SV=1	YNPTWHCIVGR
>	sp|Q78P75|DYI	Dynein light chain 2, cytoplasmic OS=Rattus norvegicus GN=Dynll2 PE=1 SV=1	YNPTWHCIVGR
>	sp|Q5FVC2|ARI	Rho guanine nucleotide exchange factor 2 OS=Rattus norvegicus GN=Arhgef2 PE=2 SV=1	GHDRLDLPVTVR
>	sp|P60868|RS2	40S ribosomal protein S20 OS=Rattus norvegicus GN=Rps20 PE=3 SV=1	DTGKTPVEPEVAIHR
>	sp|P97686|NR(	Neuronal cell adhesion molecule OS=Rattus norvegicus GN=Nrcam PE=1 SV=2	FIIEYEDAMHEAGLWR
>	sp|P97686|NR(	Neuronal cell adhesion molecule OS=Rattus norvegicus GN=Nrcam PE=1 SV=2	NEVHLEIKDPTR
>	sp|P0C6S7|ANS	Ankyrin repeat and sterile alpha motif domain-containing protein 1B OS=Rattus norvegicus GN=Anks1b PE=1 SV=1	ILQAIQLLPK
>	sp|P0C6S7|ANS	Ankyrin repeat and sterile alpha motif domain-containing protein 1B OS=Rattus norvegicus GN=Anks1b PE=1 SV=1	LHEDPPQKPPR
>	sp|P0C6S7|ANS	Ankyrin repeat and sterile alpha motif domain-containing protein 1B OS=Rattus norvegicus GN=Anks1b PE=1 SV=1	NIIAEHEIR
>	sp|P05712|RAE	Ras-related protein Rab-2A OS=Rattus norvegicus GN=Rab2a PE=1 SV=1	SCLLLQFTDKR
>	sp|P05712|RAE	Ras-related protein Rab-2A OS=Rattus norvegicus GN=Rab2a PE=1 SV=1	LQIWDTAGQESFR
>	sp|P82471|GN	Guanine nucleotide-binding protein G(q) subunit alpha OS=Rattus norvegicus GN=Gnaq PE=1 SV=2	LLLLGTGESGK
>	sp|P82471|GN	Guanine nucleotide-binding protein G(q) subunit alpha OS=Rattus norvegicus GN=Gnaq PE=1 SV=2	VPTTGIIEYPFDLQSVIFR
>	sp|O88600|HSI	Heat shock 70 kDa protein 4 OS=Rattus norvegicus GN=Hspa4 PE=1 SV=1	GCALQCAILSPAFK
>	sp|O88600|HSI	Heat shock 70 kDa protein 4 OS=Rattus norvegicus GN=Hspa4 PE=1 SV=1	NHAAPFSK
>	sp|O88600|HSI	Heat shock 70 kDa protein 4 OS=Rattus norvegicus GN=Hspa4 PE=1 SV=1	LKKEDIYAVEIVGGATR
>	sp|O88600|HSI	Heat shock 70 kDa protein 4 OS=Rattus norvegicus GN=Hspa4 PE=1 SV=1	IKELTNICSPIISKPKPK
>	sp|O88600|HSI	Heat shock 70 kDa protein 4 OS=Rattus norvegicus GN=Hspa4 PE=1 SV=1	EFSITDVVPYPISLR
>	sp|O88600|HSI	Heat shock 70 kDa protein 4 OS=Rattus norvegicus GN=Hspa4 PE=1 SV=1	LKETAESVLK
>	sp|O88600|HSI	Heat shock 70 kDa protein 4 OS=Rattus norvegicus GN=Hspa4 PE=1 SV=1	IKELTNICSPIISKPKPK
>	sp|O88600|HSI	Heat shock 70 kDa protein 4 OS=Rattus norvegicus GN=Hspa4 PE=1 SV=1	IKELTNICSPIISKPKPK
>	sp|O88600|HSI	Heat shock 70 kDa protein 4 OS=Rattus norvegicus GN=Hspa4 PE=1 SV=1	LAELR
>	sp|P81377|KAI	cAMP-dependent protein kinase type I-beta regulatory subunit OS=Rattus norvegicus GN=Prkar1b PE=2 SV=2	NVLFSHLDDNER
>	sp|Q63228|GN	Glia maturation factor beta OS=Rattus norvegicus GN=Gmfb PE=1 SV=2	LVVLDEELEGVSPDELKDELPERQPR
>	sp|Q63228|GN	Glia maturation factor beta OS=Rattus norvegicus GN=Gmfb PE=1 SV=2	LVVLDEELEGVSPDELKDELPERQPR
>	sp|P38718|BR	Brain protein 44 OS=Rattus norvegicus GN=Brp44 PE=2 SV=1	LMDKVELLLPK
>	sp|P38718|BR	Brain protein 44 OS=Rattus norvegicus GN=Brp44 PE=2 SV=1	LMDKVELLLPK
>	sp|P38718|BR	Brain protein 44 OS=Rattus norvegicus GN=Brp44 PE=2 SV=1	LMDKVELLLPK
>	sp|P21913|DHS	Succinate dehydrogenase [ubiquinone] iron-sulfur subunit, mitochondrial OS=Rattus norvegicus GN=Sdhb PE=2 SV=2	IKNEIDSTLTFR
>	sp|P21913|DHS	Succinate dehydrogenase [ubiquinone] iron-sulfur subunit, mitochondrial OS=Rattus norvegicus GN=Sdhb PE=2 SV=2	IKNEIDSTLTFR
>	sp|P85973|PNI	Purine nucleoside phosphorylase OS=Rattus norvegicus GN=Pnp PE=1 SV=1	ASHQEVLEAGK
>	sp|Q5EB94|GC	GRINL1A complex locus protein 1 OS=Rattus norvegicus GN=Gcom1 PE=1 SV=1	TLVDVTLENSNIK
>	sp|P63319|KPC	Protein kinase C gamma type OS=Rattus norvegicus GN=Prkcg PE=1 SV=1	ATLNPVWNETFVFNLKPGDVER
>	sp|Q6GMN2|B	Brain-specific angiogenesis inhibitor 1-associated protein 2 OS=Rattus norvegicus GN=Baiap2 PE=1 SV=1	SNLVISDPIPGAKPLPVPPELAPFVGR
>	sp|Q6GMN2|B	Brain-specific angiogenesis inhibitor 1-associated protein 2 OS=Rattus norvegicus GN=Baiap2 PE=1 SV=1	SNLVISDPIPGAKPLPVPPELAPFVGR
>	sp|Q6GMN2|B	Brain-specific angiogenesis inhibitor 1-associated protein 2 OS=Rattus norvegicus GN=Baiap2 PE=1 SV=1	SNLVISDPIPGAKPLPVPPELAPFVGR
>	sp|Q91Y81|SEF	Septin-2 OS=Rattus norvegicus GN=Sept2 PE=1 SV=1	ILDEIEEHSIK
>	sp|Q99PF5|FUI	Far upstream element-binding protein 2 OS=Rattus norvegicus GN=Khsrp PE=1 SV=1	HSVGVVIGR
>	sp|P97829|CD	Leukocyte surface antigen CD47 OS=Rattus norvegicus GN=Cd47 PE=1 SV=1	VLNVEAQSTDEMFVK
>	sp|P97829|CD	Leukocyte surface antigen CD47 OS=Rattus norvegicus GN=Cd47 PE=1 SV=1	GILTLK
>	sp|P97829|CD	Leukocyte surface antigen CD47 OS=Rattus norvegicus GN=Cd47 PE=1 SV=1	GILTLK
>	sp|P97829|CD	Leukocyte surface antigen CD47 OS=Rattus norvegicus GN=Cd47 PE=1 SV=1	GILTLK
>	sp|P83732|RL2	60S ribosomal protein L24 OS=Rattus norvegicus GN=Rpl24 PE=1 SV=1	VFQFLNAK

>	sp|P11348|DHI	Dihydropteridine reductase OS=Rattus norvegicus GN=Qdpr PE=1 SV=1	GAVHQLCQSLAGK
>	sp|P11348|DHI	Dihydropteridine reductase OS=Rattus norvegicus GN=Qdpr PE=1 SV=1	GAVHQLCQSLAGK
>	sp|P11348|DHI	Dihydropteridine reductase OS=Rattus norvegicus GN=Qdpr PE=1 SV=1	GAVHQLCQSLAGK
>	sp|P11348|DHI	Dihydropteridine reductase OS=Rattus norvegicus GN=Qdpr PE=1 SV=1	GAVHQLCQSLAGK
>	sp|P21396|AOI	Amine oxidase [flavin-containing] A OS=Rattus norvegicus GN=Maoa PE=1 SV=1	DVPAIEITHTFLER
>	sp|P21396|AOI	Amine oxidase [flavin-containing] A OS=Rattus norvegicus GN=Maoa PE=1 SV=1	DVPAIEITHTFLER
>	sp|P21396|AOI	Amine oxidase [flavin-containing] A OS=Rattus norvegicus GN=Maoa PE=1 SV=1	IHFKPELPPER
>	sp|P12346|TRF	Serotransferrin OS=Rattus norvegicus GN=Tf PE=1 SV=3	VSTVLTAQK
>	sp|P12346|TRF	Serotransferrin OS=Rattus norvegicus GN=Tf PE=1 SV=3	ADRDQYELLCLDNTR
>	sp|P12346|TRF	Serotransferrin OS=Rattus norvegicus GN=Tf PE=1 SV=3	EGVCPEGSIDSAPVK
>	sp|P55068|PG	Brevican core protein OS=Rattus norvegicus GN=Bcan PE=1 SV=2	MGLVSCGPPPQLPLAQIFGRPR
>	sp|P55068|PG	Brevican core protein OS=Rattus norvegicus GN=Bcan PE=1 SV=2	GVLGGALAIPCHVHHLRPPPSR
>	sp|P55068|PG	Brevican core protein OS=Rattus norvegicus GN=Bcan PE=1 SV=2	MGLVSCGPPPQLPLAQIFGRPR
>	sp|P55068|PG	Brevican core protein OS=Rattus norvegicus GN=Bcan PE=1 SV=2	MGLVSCGPPPQLPLAQIFGRPR
>	sp|P55068|PG	Brevican core protein OS=Rattus norvegicus GN=Bcan PE=1 SV=2	MGLVSCGPPPQLPLAQIFGRPR
>	sp|P55068|PG	Brevican core protein OS=Rattus norvegicus GN=Bcan PE=1 SV=2	MGLVSCGPPPQLPLAQIFGRPR
>	sp|P55068|PG	Brevican core protein OS=Rattus norvegicus GN=Bcan PE=1 SV=2	MGLVSCGPPPQLPLAQIFGRPR
>	sp|P55068|PG	Brevican core protein OS=Rattus norvegicus GN=Bcan PE=1 SV=2	GVLGGALAIPCHVHHLRPPPSR
>	sp|P55068|PG	Brevican core protein OS=Rattus norvegicus GN=Bcan PE=1 SV=2	MGLVSCGPPPQLPLAQIFGRPR
>	sp|P55068|PG	Brevican core protein OS=Rattus norvegicus GN=Bcan PE=1 SV=2	YAVDTVLR
>	sp|P43425|GB	Guanine nucleotide-binding protein G(I)/G(S)/G(O) subunit gamma-7 OS=Rattus norvegicus GN=Gng7 PE=2 SV=2	KLVEQLR
>	sp|P05197|EF2	Elongation factor 2 OS=Rattus norvegicus GN=Eef2 PE=1 SV=4	ARPFPDGLAEDIDKGEVSAR
>	sp|P05197|EF2	Elongation factor 2 OS=Rattus norvegicus GN=Eef2 PE=1 SV=4	VFSGVVSTGLK
>	sp|P05197|EF2	Elongation factor 2 OS=Rattus norvegicus GN=Eef2 PE=1 SV=4	KEDLYLKPIQR
>	sp|Q7M767|UE	Ubiquitin-conjugating enzyme E2 variant 2 OS=Rattus norvegicus GN=Ube2v2 PE=1 SV=3	LLEELEEGQK
>	sp|Q7M767|UE	Ubiquitin-conjugating enzyme E2 variant 2 OS=Rattus norvegicus GN=Ube2v2 PE=1 SV=3	LLEELEEGQK
>	sp|Q6AY84|SCI	Secernin-1 OS=Rattus norvegicus GN=Scrn1 PE=1 SV=1	SIFKPFIFVDDVK
>	sp|Q9Z1N3|MY	Myosin-IXa OS=Rattus norvegicus GN=Myo9a PE=1 SV=1	TVTVGEK
>	sp|Q9Z1N3|MY	Myosin-IXa OS=Rattus norvegicus GN=Myo9a PE=1 SV=1	VLLSRQQFLHLR
>	sp|Q9EQX9|UB	Ubiquitin-conjugating enzyme E2 N OS=Rattus norvegicus GN=Ube2n PE=1 SV=1	LLAEPVPGIK
>	sp|Q9EQX9|UB	Ubiquitin-conjugating enzyme E2 N OS=Rattus norvegicus GN=Ube2n PE=1 SV=1	YFHVVIAGPQDSPFEGGTFK
>	sp|Q62717|CAI	Calcium-dependent secretion activator 1 OS=Rattus norvegicus GN=Cadps PE=1 SV=1	LFTESTGVLALEDKELGR
>	sp|Q3B7T8|CEI	Centrosomal protein of 44 kDa OS=Rattus norvegicus GN=Cep44 PE=2 SV=1	IPEAQAPEVK
>	sp|P49911|AN	Acidic leucine-rich nuclear phosphoprotein 32 family member A OS=Rattus norvegicus GN=Anp32a PE=2 SV=1	KREPDDEGQEDD
>	sp|O88269|MR	Multidrug resistance-associated protein 6 OS=Rattus norvegicus GN=Abcc6 PE=2 SV=1	LLGELLK
>	sp|O88269|MR	Multidrug resistance-associated protein 6 OS=Rattus norvegicus GN=Abcc6 PE=2 SV=1	LLGELLK
>	sp|P49242|RS3	40S ribosomal protein S3a OS=Rattus norvegicus GN=Rps3a PE=1 SV=2	NCLTNFHGMDLTR
>	sp|P63081|VA1	V-type proton ATPase 16 kDa proteolipid subunit OS=Rattus norvegicus GN=Atp6v0c PE=2 SV=1	SGTGIAAMSVMRPELIMK
>	sp|P18421|PSB	Proteasome subunit beta type-1 OS=Rattus norvegicus GN=Psmb1 PE=1 SV=3	NMQNVEHVPLTLDR
>	sp|P29314|RS9	40S ribosomal protein S9 OS=Rattus norvegicus GN=Rps9 PE=1 SV=4	MKLDYILGLK
>	sp|O35923|BR	Breast cancer type 2 susceptibility protein homolog OS=Rattus norvegicus GN=Brca2 PE=1 SV=1	SSTSGTVYINK
>	RRRRRsp|Q634	REVERSED Serine/threonine-protein kinase N1 OS=Rattus norvegicus GN=Pkn1 PE=1 SV=2	LQMRIIDIK
>	RRRRRsp|Q634	REVERSED Serine/threonine-protein kinase N1 OS=Rattus norvegicus GN=Pkn1 PE=1 SV=2	AGEAVAHEVR
>	RRRRRsp|Q9JJI	REVERSED Neuromedin-U receptor 1 OS=Rattus norvegicus GN=Nmur1 PE=1 SV=1	TGLGLTER
>	RRRRRsp|O702	REVERSED WAP four-disulfide core domain protein 1 OS=Rattus norvegicus GN=Wfdc1 PE=1 SV=1	SGTAAVEEAQSK
>	sp|P51635|AK1	Alcohol dehydrogenase [NADP+] OS=Rattus norvegicus GN=Akr1a1 PE=1 SV=2	AVPREELFVTSK
>	sp|P51635|AK1	Alcohol dehydrogenase [NADP+] OS=Rattus norvegicus GN=Akr1a1 PE=1 SV=2	ALEALVAK
>	sp|P51635|AK1	Alcohol dehydrogenase [NADP+] OS=Rattus norvegicus GN=Akr1a1 PE=1 SV=2	HPDEPVLLEEPVVLALAEK
>	sp|P35434|ATF	ATP synthase subunit delta, mitochondrial OS=Rattus norvegicus GN=Atp5d PE=1 SV=2	AQSELSGAADEAAR
>	sp|P35434|ATF	ATP synthase subunit delta, mitochondrial OS=Rattus norvegicus GN=Atp5d PE=1 SV=2	QVDVPTLTGAFGILASHVPTLQVLRPGLVMVHAEDGTTTK
>	sp|P21708|MK	Mitogen-activated protein kinase 3 OS=Rattus norvegicus GN=Mapk3 PE=1 SV=5	LKELIFQETAR
>	sp|P21708|MK	Mitogen-activated protein kinase 3 OS=Rattus norvegicus GN=Mapk3 PE=1 SV=5	LKELIFQETAR
>	sp|P21708|MK	Mitogen-activated protein kinase 3 OS=Rattus norvegicus GN=Mapk3 PE=1 SV=5	DLKPSNLLINTTCDLK
>	sp|P19945|RLA	60S acidic ribosomal protein P0 OS=Rattus norvegicus GN=Rplp0 PE=1 SV=2	IIQLLDDYPK
>	sp|P19945|RLA	60S acidic ribosomal protein P0 OS=Rattus norvegicus GN=Rplp0 PE=1 SV=2	IIQLLDDYPK
>	RRRRRsp|Q9R1	REVERSED Cyclin-L1 OS=Rattus norvegicus GN=Ccnl1 PE=1 SV=1	RPTHSRSR
>	sp|P19511|AT5	ATP synthase subunit b, mitochondrial OS=Rattus norvegicus GN=Atp5f1 PE=1 SV=1	AQQALVQK
>	sp|P19511|AT5	ATP synthase subunit b, mitochondrial OS=Rattus norvegicus GN=Atp5f1 PE=1 SV=1	AQQALVQK
>	sp|P19511|AT5	ATP synthase subunit b, mitochondrial OS=Rattus norvegicus GN=Atp5f1 PE=1 SV=1	AQQALVQK
>	sp|P24090|FET	Alpha-2-HS-glycoprotein OS=Rattus norvegicus GN=Ahsg PE=1 SV=2	QQAEHAVEGDCDFHILK
>	sp|Q920L2|DH	Succinate dehydrogenase [ubiquinone] flavoprotein subunit, mitochondrial OS=Rattus norvegicus GN=Sdha PE=1 SV=1	AFGGQSLK
>	sp|Q920L2|DH	Succinate dehydrogenase [ubiquinone] flavoprotein subunit, mitochondrial OS=Rattus norvegicus GN=Sdha PE=1 SV=1	MAGVGAVSRLLRGR
>	sp|P09527|RAE	Ras-related protein Rab-7a OS=Rattus norvegicus GN=Rab7a PE=1 SV=2	EAINVEQAFQTIAR
>	sp|P09527|RAE	Ras-related protein Rab-7a OS=Rattus norvegicus GN=Rab7a PE=1 SV=2	EAINVEQAFQTIAR
>	sp|P09527|RAE	Ras-related protein Rab-7a OS=Rattus norvegicus GN=Rab7a PE=1 SV=2	EAINVEQAFQTIAR
>	sp|P09527|RAE	Ras-related protein Rab-7a OS=Rattus norvegicus GN=Rab7a PE=1 SV=2	EAINVEQAFQTIAR
>	sp|P09527|RAE	Ras-related protein Rab-7a OS=Rattus norvegicus GN=Rab7a PE=1 SV=2	NALKQETEVELYNEFPEPIKLDKNER
>	sp|P15389|SCN	Sodium channel protein type 5 subunit alpha OS=Rattus norvegicus GN=Scn5a PE=2 SV=1	TISVISGLK
>	RRRRRsp|Q5BJ	REVERSED Copine-9 OS=Rattus norvegicus GN=Cpne9 PE=2 SV=1	VFDPNLTNDIVETR
>	sp|P28564|5H1	5-hydroxytryptamine receptor 1B OS=Rattus norvegicus GN=Htr1b PE=2 SV=1	LTRAQLITDSPGSTSSVTSINSR
>	sp|P02401|RLA	60S acidic ribosomal protein P2 OS=Rattus norvegicus GN=Rplp2 PE=1 SV=2	NIEDVIAQGVGK
>	sp|Q9QUH6|SY	Ras GTPase-activating protein SynGAP OS=Rattus norvegicus GN=Syngap1 PE=1 SV=2	LMLVEEELRR
>	sp|Q5FWT8|RC	REST corepressor 2 OS=Rattus norvegicus GN=Rcor2 PE=2 SV=1	GLDSQLISLK
>	RRRRRsp|Q923	REVERSED Cryptochrome-2 OS=Rattus norvegicus GN=Cry2 PE=2 SV=1	EVGAEK

>	RRRRRsp|Q9QV	REVERSED Spectrin beta chain, brain 2 OS=Rattus norvegicus GN=Sptbn2 PE=1 SV=2	GEVALAK
>	RRRRRsp|Q9QV	REVERSED Spectrin beta chain, brain 2 OS=Rattus norvegicus GN=Sptbn2 PE=1 SV=2	ILETRLALER
>	RRRRRsp|Q9QV	REVERSED Spectrin beta chain, brain 2 OS=Rattus norvegicus GN=Sptbn2 PE=1 SV=2	LAELR
>	RRRRRsp|Q9QV	REVERSED Spectrin beta chain, brain 2 OS=Rattus norvegicus GN=Sptbn2 PE=1 SV=2	KGEVALAK
>	sp|O35824|DN	DnaJ homolog subfamily A member 2 OS=Rattus norvegicus GN=Dnaja2 PE=2 SV=1	ILEVHVDK
>	sp|Q99NH3|R3	Guanine nucleotide exchange factor for Rab-3A OS=Rattus norvegicus GN=Rab3il1 PE=1 SV=1	LRSSSMEIREK
>	sp|P35565|CAL	Calnexin OS=Rattus norvegicus GN=Canx PE=1 SV=1	KIPNPDFFEDLEPFR
>	sp|P35565|CAL	Calnexin OS=Rattus norvegicus GN=Canx PE=1 SV=1	KIPNPDFFEDLEPFR
>	sp|P35565|CAL	Calnexin OS=Rattus norvegicus GN=Canx PE=1 SV=1	KIPNPDFFEDLEPFR
>	sp|P35565|CAL	Calnexin OS=Rattus norvegicus GN=Canx PE=1 SV=1	KIPNPDFFEDLEPFR
>	sp|P35565|CAL	Calnexin OS=Rattus norvegicus GN=Canx PE=1 SV=1	NKGDEEEEEEKLEEK
>	sp|Q9Z0J8|NEG	Neuronal growth regulator 1 OS=Rattus norvegicus GN=Negr1 PE=1 SV=1	VVVNFAPTIQEIK
>	RRRRRsp|Q700	REVERSED SCO-spondin OS=Rattus norvegicus GN=Sspo PE=2 SV=1	ATSHVRSATSAR
>	RRRRRsp|P5501	REVERSED Solute carrier family 12 member 3 OS=Rattus norvegicus GN=Slc12a3 PE=2 SV=2	GLGSAQMLIQVGSR
>	RRRRRsp|Q636	REVERSED ATP-sensitive inward rectifier potassium channel 8 OS=Rattus norvegicus GN=Kcnj8 PE=2 SV=1	VAIVAHRSFILTEARR
>	sp|B1WC68|HD	Histone deacetylase 8 OS=Rattus norvegicus GN=Hdac8 PE=2 SV=1	VMTVSLHK
>	sp|B1WC68|HD	Histone deacetylase 8 OS=Rattus norvegicus GN=Hdac8 PE=2 SV=1	VMTVSLHK
>	sp|P31422|GRI	Metabotropic glutamate receptor 3 OS=Rattus norvegicus GN=Grm3 PE=1 SV=1	LGVHILDTCSR
>	sp|P08081|CLC	Clathrin light chain A OS=Rattus norvegicus GN=Clta PE=1 SV=1	SVLISLK
>	sp|P70496|PLD	Phospholipase D1 OS=Rattus norvegicus GN=Pld1 PE=1 SV=3	LTDVGSVK
>	RRRRRsp|Q3KR	REVERSED ATPase family AAA domain-containing protein 3 OS=Rattus norvegicus GN=Atad3 PE=2 SV=1	GAVSTANK
>	RRRRRsp|Q3KR	REVERSED ATPase family AAA domain-containing protein 3 OS=Rattus norvegicus GN=Atad3 PE=2 SV=1	GLRAEIYR
>	sp|B2RZ78|VPS	Vacuolar protein sorting-associated protein 29 OS=Rattus norvegicus GN=Vps29 PE=1 SV=2	TLAGDVHIVR
>	RRRRRsp|P583	REVERSED Cadherin-23 OS=Rattus norvegicus GN=Cdh23 PE=2 SV=1	VTIVGTIADIK
>	RRRRRsp|Q99M	REVERSED WD40 repeat-containing protein SMU1 OS=Rattus norvegicus GN=Smu1 PE=2 SV=1	LAQGLLAMLR
>	RRRRRsp|Q627	REVERSED Secreted phosphoprotein 24 OS=Rattus norvegicus GN=Spp2 PE=2 SV=2	RQLNLFR
>	RRRRRsp|P128	REVERSED Neurofilament medium polypeptide OS=Rattus norvegicus GN=Nefm PE=1 SV=4	ESSGEK
>	sp|Q4V8G4|WI	WD repeat-containing protein 78 OS=Rattus norvegicus GN=Wdr78 PE=2 SV=1	RTTVTSSK
>	sp|Q3MJK5|CD	Cyclin-dependent kinase 12 OS=Rattus norvegicus GN=Cdk12 PE=1 SV=1	LADFGLAR
>	RRRRRsp|O549	REVERSED [Pyruvate dehydrogenase [lipoamide]] kinase isozyme 4, mitochondrial OS=Rattus norvegicus GN=Pdk4 PE=2 SV=1	GLVVTAEVPTLFPR
>	RRRRRsp|Q6RF	REVERSED Pleckstrin homology domain-containing family G member 5 OS=Rattus norvegicus GN=Plekhg5 PE=1 SV=1	VESATLTSNLLLTTR
>	sp|O08838|AM	Amphiphysin OS=Rattus norvegicus GN=Amph PE=1 SV=1	AFSIQGAPSDSGPLR
>	sp|Q9WVS2|OS	Probable tRNA threonylcarbamoyladenosine biosynthesis protein Osgep (Fragment) OS=Rattus norvegicus GN=Osgep PE=2 SV=1	DGTVLANPR
>	RRRRRsp|O704	REVERSED Serine/threonine-protein kinase pim-3 OS=Rattus norvegicus GN=Pim3 PE=1 SV=3	AGGAAGVK
>	sp|P68403|KPC	Protein kinase C beta type OS=Rattus norvegicus GN=Prkcb PE=1 SV=3	DIKEHAFFR
>	sp|P68403|KPC	Protein kinase C beta type OS=Rattus norvegicus GN=Prkcb PE=1 SV=3	DIKEHAFFR
>	sp|P82458|TRI	Midline-1 OS=Rattus norvegicus GN=Mid1 PE=2 SV=1	NVTLQNIIDR
>	sp|P82458|TRI	Midline-1 OS=Rattus norvegicus GN=Mid1 PE=2 SV=1	NVTLQNIIDR
>	sp|Q80ZF0|COI	Collagen alpha-1(XXVII) chain OS=Rattus norvegicus GN=Col27a1 PE=2 SV=1	LGSSQTPLVPAK
>	sp|P28840|NEC	Neuroendocrine convertase 1 OS=Rattus norvegicus GN=Pcsk1 PE=2 SV=1	FGFGLLNAK
>	RRRRRsp|Q99JI	REVERSED Mitochondrial 2-oxodicarboxylate carrier OS=Rattus norvegicus GN=Slc25a21 PE=2 SV=1	NVQLGVK
>	sp|P81155|VDA	Voltage-dependent anion-selective channel protein 2 OS=Rattus norvegicus GN=Vdac2 PE=1 SV=2	LTLSALVDGK
>	sp|P81155|VDA	Voltage-dependent anion-selective channel protein 2 OS=Rattus norvegicus GN=Vdac2 PE=1 SV=2	LTLSALVDGK
>	sp|P81155|VDA	Voltage-dependent anion-selective channel protein 2 OS=Rattus norvegicus GN=Vdac2 PE=1 SV=2	GFGFGLVK
>	sp|Q03346|MP	Mitochondrial-processing peptidase subunit beta OS=Rattus norvegicus GN=Pmpcb PE=1 SV=3	TLLPVAGRR
>	sp|Q9Z270|VAI	Vesicle-associated membrane protein-associated protein A OS=Rattus norvegicus GN=Vapa PE=1 SV=3	HEQILVLDPPSDLK
>	sp|Q9Z270|VAI	Vesicle-associated membrane protein-associated protein A OS=Rattus norvegicus GN=Vapa PE=1 SV=3	FKGPFTDVVTTNLK
>	RRRRRsp|O549	REVERSED Hepatitis A virus cellular receptor 1 homolog OS=Rattus norvegicus GN=Havcr1 PE=2 SV=1	TSITTPRTTTPR
>	sp|P54290|CA2	Voltage-dependent calcium channel subunit alpha-2/delta-1 OS=Rattus norvegicus GN=Cacna2d1 PE=1 SV=1	LMNTNLVFIMVESK
>	sp|P54290|CA2	Voltage-dependent calcium channel subunit alpha-2/delta-1 OS=Rattus norvegicus GN=Cacna2d1 PE=1 SV=1	LLKPAVVGIK
>	RRRRRsp|Q635	REVERSED Nuclear receptor subfamily 1 group D member 1 OS=Rattus norvegicus GN=Nr1d1 PE=2 SV=2	TINSTGK
>	sp|Q5M888|CG	UPF0415 protein C7orf25 homolog OS=Rattus norvegicus PE=2 SV=1	LVASSKINSR
>	sp|Q80ZA5|S4A	Sodium-driven chloride bicarbonate exchanger OS=Rattus norvegicus GN=Slc4a10 PE=2 SV=1	TLFIGVHVPLGGR
>	sp|Q9Z2L9|NDI	Protein NDRG4 OS=Rattus norvegicus GN=Ndrg4 PE=2 SV=1	LDPTTTTFLK
>	sp|Q1AAU6|AS	Arf-GAP with SH3 domain, ANK repeat and PH domain-containing protein 1 OS=Rattus norvegicus GN=Asap1 PE=2 SV=2	AIIEDVQR
>	RRRRRsp|Q4FZ	REVERSED Protein FAM65A OS=Rattus norvegicus GN=Fam65a PE=2 SV=2	TVTSAK
>	RRRRRsp|Q4FZ	REVERSED Protein FAM65A OS=Rattus norvegicus GN=Fam65a PE=2 SV=2	GLSLSR
>	sp|Q5PPJ9|SHL	Endophilin-B2 OS=Rattus norvegicus GN=Sh3glb2 PE=2 SV=2	TELDAHFENLLAR
>	sp|Q64559|BA	Cytosolic acyl coenzyme A thioester hydrolase OS=Rattus norvegicus GN=Acot7 PE=1 SV=4	IMRPDDANVAGNVHGGTILK
>	RRRRRsp|Q646	REVERSED Serine/threonine-protein phosphatase 6 catalytic subunit OS=Rattus norvegicus GN=Ppp6c PE=2 SV=2	LFYPTTTRPPIVR
>	sp|Q9ESV1|LU	Leucine zipper protein 1 OS=Rattus norvegicus GN=Luzp1 PE=2 SV=1	ELGNGDIEGEDAFLLGR
>	sp|Q4FZU8|FA	Protein FAM65A OS=Rattus norvegicus GN=Fam65a PE=2 SV=2	IESSGK
>	sp|Q04400|AD	Adenylate cyclase type 5 OS=Rattus norvegicus GN=Adcy5 PE=2 SV=2	GAAGGGSTR
>	sp|Q7TMA5|AP	Apolipoprotein B-100 OS=Rattus norvegicus GN=Apob PE=1 SV=1	DVLLIFR
>	sp|Q7TMA5|AP	Apolipoprotein B-100 OS=Rattus norvegicus GN=Apob PE=1 SV=1	TAILFQR
>	sp|Q4V8G7|CE	Centromere protein U OS=Rattus norvegicus GN=Mlf1ip PE=2 SV=1	IISNMEK
>	sp|Q9JHS2|HIF	Hypoxia-inducible factor 3-alpha OS=Rattus norvegicus GN=Hif3a PE=1 SV=1	DAARSR
>	sp|P50503|F10	Hsc70-interacting protein OS=Rattus norvegicus GN=St13 PE=1 SV=1	LLGHWEEAAR
>	sp|P30904|MIF	Macrophage migration inhibitory factor OS=Rattus norvegicus GN=Mif PE=1 SV=4	LLCGLLSDR
>	sp|P30904|MIF	Macrophage migration inhibitory factor OS=Rattus norvegicus GN=Mif PE=1 SV=4	LLCGLLSDR
>	sp|P84817|FIS1	Mitochondrial fission 1 protein OS=Rattus norvegicus GN=Fis1 PE=1 SV=1	GIVLLEELLPK
>	RRRRRsp|Q4KL	REVERSED Paraspeckle component 1 OS=Rattus norvegicus GN=Pspc1 PE=2 SV=1	AAEMEAELKEKAER
>	sp|Q5FVL7|KTU	Protein kintoun OS=Rattus norvegicus GN=Dnaaf2 PE=2 SV=2	LQSEAK
>	sp|Q62718|NTI	Neurotrimin OS=Rattus norvegicus GN=Ntm PE=1 SV=1	AVGFVSEDEYLEIQGITR

>	RRRRRsp|O886	REVERSED Actin-related protein 2/3 complex subunit 1B OS=Rattus norvegicus GN=Arpc1b PE=2 SV=3	KDLNQFR
>	sp|Q4QQU7|GI	Growth hormone-regulated TBC protein 1 OS=Rattus norvegicus GN=Grtp1 PE=2 SV=1	LLKGSGGVR
>	sp|P35171|CX7	Cytochrome c oxidase subunit 7A2, mitochondrial OS=Rattus norvegicus GN=Cox7a2 PE=1 SV=1	LFQEDNGMPVHLK
>	RRRRRsp|P047	REVERSED Glyceraldehyde-3-phosphate dehydrogenase OS=Rattus norvegicus GN=Gapdh PE=1 SV=3	AAGTSAPIINQAAGR
>	sp|Q5HZV9|PP	Protein phosphatase 1 regulatory subunit 7 OS=Rattus norvegicus GN=Ppp1r7 PE=1 SV=1	IENISTLQQLQMLELGSNR
>	sp|P13697|MA	NADP-dependent malic enzyme OS=Rattus norvegicus GN=Me1 PE=1 SV=2	IKPTALIGVAAIGGAFTEQILKDMAAFNERPIIFALSNPTSK
>	sp|Q925N3|PE	PEX5-related protein OS=Rattus norvegicus GN=Pex5l PE=1 SV=1	ALEIQPGFIRSR
>	RRRRRsp|Q630	REVERSED Runt-related transcription factor 1 OS=Rattus norvegicus GN=Runx1 PE=2 SV=1	ESFSLSGPK
>	RRRRRsp|Q9JJ7	REVERSED Cytoplasmic dynein 2 heavy chain 1 OS=Rattus norvegicus GN=Dync2h1 PE=1 SV=1	LTAVAWSR
>	RRRRRsp|Q9JJ7	REVERSED Cytoplasmic dynein 2 heavy chain 1 OS=Rattus norvegicus GN=Dync2h1 PE=1 SV=1	LLTER
>	RRRRRsp|Q9JJ7	REVERSED Cytoplasmic dynein 2 heavy chain 1 OS=Rattus norvegicus GN=Dync2h1 PE=1 SV=1	LGEPAASLYTIFAAALQAR
>	sp|Q9Z339|GST	Glutathione S-transferase omega-1 OS=Rattus norvegicus GN=Gsto1 PE=1 SV=2	VPSLVTSFIR
>	RRRRRsp|Q024	REVERSED Filensin (Fragment) OS=Rattus norvegicus GN=Bfsp1 PE=2 SV=2	LGAHRQQLARAR
>	sp|Q5BJN8|CA	UPF0667 protein C1orf55 homolog OS=Rattus norvegicus PE=2 SV=2	LFSVRGLSK
>	sp|Q5BJN8|CA	UPF0667 protein C1orf55 homolog OS=Rattus norvegicus PE=2 SV=2	DLSGRRLR
>	RRRRRsp|Q4V7	REVERSED Uncharacterized protein KIAA0895-like OS=Rattus norvegicus GN=Kiaa0895l PE=2 SV=1	GLEAEDLR
>	sp|Q68G31|PB	Phenazine biosynthesis-like domain-containing protein OS=Rattus norvegicus GN=Pbld PE=2 SV=1	SFLESLK
>	RRRRRsp|P0C5	REVERSED Microtubule-associated protein 1S OS=Rattus norvegicus GN=Map1s PE=1 SV=1	RTLPVRGPK
>	sp|P07722|MA	Myelin-associated glycoprotein OS=Rattus norvegicus GN=Mag PE=1 SV=1	LREDEGTWVQVSLLHFVPTR
>	sp|P29419|ATF	ATP synthase subunit e, mitochondrial OS=Rattus norvegicus GN=Atp5i PE=1 SV=3	ELAEAEDVSIFK
>	sp|Q9JI66|S4A	Electrogenic sodium bicarbonate cotransporter 1 OS=Rattus norvegicus GN=Slc4a4 PE=1 SV=1	EASSLPQLVEMIADHQIETGLLKPDLK
>	sp|Q9JI66|S4A	Electrogenic sodium bicarbonate cotransporter 1 OS=Rattus norvegicus GN=Slc4a4 PE=1 SV=1	EASSLPQLVEMIADHQIETGLLKPDLK
>	sp|Q9JI66|S4A	Electrogenic sodium bicarbonate cotransporter 1 OS=Rattus norvegicus GN=Slc4a4 PE=1 SV=1	EASSLPQLVEMIADHQIETGLLKPDLK
>	sp|Q9JI66|S4A	Electrogenic sodium bicarbonate cotransporter 1 OS=Rattus norvegicus GN=Slc4a4 PE=1 SV=1	LLWILK
>	sp|Q9JI66|S4A	Electrogenic sodium bicarbonate cotransporter 1 OS=Rattus norvegicus GN=Slc4a4 PE=1 SV=1	EASSLPQLVEMIADHQIETGLLKPDLKDKVTYTLLR
>	RRRRRsp|Q4V8	REVERSED Beta-catenin-like protein 1 OS=Rattus norvegicus GN=Ctnnbl1 PE=2 SV=1	LASSRSIK
>	sp|Q8VBU2|ND	Protein NDRG2 OS=Rattus norvegicus GN=Ndrg2 PE=1 SV=1	DLNFER
>	RRRRRsp|Q9Z1	REVERSED 3'(2'),5'-bisphosphate nucleotidase 1 OS=Rattus norvegicus GN=Bpnt1 PE=1 SV=1	DAKTQLDTASTK
>	RRRRRsp|O887	REVERSED Protein bassoon OS=Rattus norvegicus GN=Bsn PE=1 SV=3	AGGAPLEPRPPGGAGSAK
>	sp|Q5XIN7|PM	Protein O-linked-mannose beta-1,2-N-acetylglucosaminyltransferase 1 OS=Rattus norvegicus GN=Pomgnt1 PE=2 SV=1	GLWRLFR
>	sp|P62907|RL1	60S ribosomal protein L10a OS=Rattus norvegicus GN=Rpl10a PE=1 SV=2	AVDIPHMDIEALKK
>	RRRRRsp|Q9Z1	REVERSED Rap guanine nucleotide exchange factor 3 OS=Rattus norvegicus GN=Rapgef3 PE=2 SV=2	LLNSLRTDR
>	sp|P11598|PDI	Protein disulfide-isomerase A3 OS=Rattus norvegicus GN=Pdia3 PE=1 SV=2	FLQEYFDGNLKR
>	RRRRRsp|P313	REVERSED ATP synthase subunit d, mitochondrial OS=Rattus norvegicus GN=Atp5h PE=1 SV=3	DEPVPIK
>	sp|Q3SWS9|JK	Janus kinase and microtubule-interacting protein 1 OS=Rattus norvegicus GN=Jakmip1 PE=1 SV=1	AYALLQEQVGGTLDAER
>	RRRRRsp|O357	REVERSED Kinesin-like protein KIF1C OS=Rattus norvegicus GN=Kif1c PE=1 SV=1	LGGGRGGSGQLGQLR
>	RRRRRsp|Q66H	REVERSED Coiled-coil and C2 domain-containing protein 1A OS=Rattus norvegicus GN=Cc2d1a PE=2 SV=2	ARLAAVKYEHQLSQLR
>	RRRRRsp|Q5XI	REVERSED Uncharacterized protein C8orf45 homolog OS=Rattus norvegicus PE=2 SV=1	GPIFVTVSR
>	sp|Q62952|DP	Dihydropyrimidinase-related protein 3 OS=Rattus norvegicus GN=Dpysl3 PE=1 SV=2	MDENQFVAVTSTNAAK
>	sp|Q62952|DP	Dihydropyrimidinase-related protein 3 OS=Rattus norvegicus GN=Dpysl3 PE=1 SV=2	MDENQFVAVTSTNAAK
>	sp|Q62952|DP	Dihydropyrimidinase-related protein 3 OS=Rattus norvegicus GN=Dpysl3 PE=1 SV=2	MDENQFVAVTSTNAAK
>	sp|Q62952|DP	Dihydropyrimidinase-related protein 3 OS=Rattus norvegicus GN=Dpysl3 PE=1 SV=2	MDENQFVAVTSTNAAK
>	sp|Q62952|DP	Dihydropyrimidinase-related protein 3 OS=Rattus norvegicus GN=Dpysl3 PE=1 SV=2	IVNDDQSFYADIYMEDGLIK
>	sp|Q62952|DP	Dihydropyrimidinase-related protein 3 OS=Rattus norvegicus GN=Dpysl3 PE=1 SV=2	MDENQFVAVTSTNAAK
>	sp|Q62952|DP	Dihydropyrimidinase-related protein 3 OS=Rattus norvegicus GN=Dpysl3 PE=1 SV=2	IMLEDGNLHVTQGAGR
>	sp|Q62952|DP	Dihydropyrimidinase-related protein 3 OS=Rattus norvegicus GN=Dpysl3 PE=1 SV=2	MDENQFVAVTSTNAAK
>	sp|Q62952|DP	Dihydropyrimidinase-related protein 3 OS=Rattus norvegicus GN=Dpysl3 PE=1 SV=2	IVAPPGGR
>	sp|Q62952|DP	Dihydropyrimidinase-related protein 3 OS=Rattus norvegicus GN=Dpysl3 PE=1 SV=2	IVAPPGGR
>	sp|Q62952|DP	Dihydropyrimidinase-related protein 3 OS=Rattus norvegicus GN=Dpysl3 PE=1 SV=2	MADLHAVPR
>	sp|Q62952|DP	Dihydropyrimidinase-related protein 3 OS=Rattus norvegicus GN=Dpysl3 PE=1 SV=2	QIGDNLIVPGGVK
>	sp|Q62952|DP	Dihydropyrimidinase-related protein 3 OS=Rattus norvegicus GN=Dpysl3 PE=1 SV=2	KMADLHAVPR
>	sp|P83829|IFT	Intraflagellar transport protein 81 homolog OS=Rattus norvegicus GN=Ift81 PE=2 SV=1	EKLASLER
>	sp|Q4QQT4|2A	Serine/threonine-protein phosphatase 2A 65 kDa regulatory subunit A beta isoform OS=Rattus norvegicus GN=Ppp2r1b PE=2 SV=1	KLSTIALALGVER
>	sp|Q63347|PR	26S protease regulatory subunit 7 OS=Rattus norvegicus GN=Psmc2 PE=1 SV=3	LREVVETPLLHPER
>	sp|P23711|HM	Heme oxygenase 2 OS=Rattus norvegicus GN=Hmox2 PE=1 SV=1	ETLEDGLPVHDGKGDVR
>	sp|Q9JIL8|RAD	DNA repair protein RAD50 OS=Rattus norvegicus GN=Rad50 PE=1 SV=1	FQMELK
>	sp|Q9JIL8|RAD	DNA repair protein RAD50 OS=Rattus norvegicus GN=Rad50 PE=1 SV=1	ALDTLR
>	sp|Q9JIX5|CHD	Chromodomain-helicase-DNA-binding protein 8 OS=Rattus norvegicus GN=Chd8 PE=1 SV=2	TTGYPSSPATTTSGTALR
>	sp|P07171|CAL	Calbindin OS=Rattus norvegicus GN=Calb1 PE=1 SV=2	LLPVQENFLLK
>	sp|Q6TLK4|RH	Rho GTPase-activating protein 27 OS=Rattus norvegicus GN=Arhgap27 PE=1 SV=1	AAAPPR
>	sp|Q99P96|HD	Histone deacetylase 7 (Fragment) OS=Rattus norvegicus GN=Hdac7 PE=2 SV=1	LAEVILK
>	sp|Q9WU34|SE	Neuronal-specific septin-3 OS=Rattus norvegicus GN=Sept3 PE=1 SV=2	AIGHVIEEGGVK
>	sp|Q9JJH2|GP1	Probable G-protein coupled receptor 173 OS=Rattus norvegicus GN=Gpr173 PE=2 SV=1	GPMPPTLLGIRQNGHAASR
>	RRRRRsp|Q68F	REVERSED Threonyl-tRNA synthetase, mitochondrial OS=Rattus norvegicus GN=Tars2 PE=2 SV=1	IFAVLANYIR
>	sp|P08082|CLC	Clathrin light chain B OS=Rattus norvegicus GN=Cltb PE=1 SV=1	LQELDAASK
>	sp|P19491|GRI	Glutamate receptor 2 OS=Rattus norvegicus GN=Gria2 PE=1 SV=2	SLFQDLELKK
>	sp|Q68A21|PU	Transcriptional activator protein Pur-beta OS=Rattus norvegicus GN=Purb PE=1 SV=3	FYLDVK
>	sp|P62329|TYB	Thymosin beta-4 OS=Rattus norvegicus GN=Tmsb4x PE=1 SV=2	TETQEKNPLPSKETIEQEK
>	sp|P28023|DC	Dynactin subunit 1 OS=Rattus norvegicus GN=Dctn1 PE=2 SV=2	LVLTQEQLHQLHGR
>	sp|P21816|CD	Cysteine dioxygenase type 1 OS=Rattus norvegicus GN=Cdo1 PE=1 SV=1	LLQGNLK
>	RRRRRsp|Q66H	REVERSED Protein FAM48A OS=Rattus norvegicus GN=Fam48a PE=2 SV=1	EVDVESPVALNCPSR
>	RRRRRsp|O705	REVERSED Sterol O-acyltransferase 1 OS=Rattus norvegicus GN=Soat1 PE=2 SV=1	VTPTRPYSDR
>	sp|P12928|KPY	Pyruvate kinase isozymes R/L OS=Rattus norvegicus GN=Pklr PE=2 SV=2	GDLGIEIPAEK
>	sp|P12928|KPY	Pyruvate kinase isozymes R/L OS=Rattus norvegicus GN=Pklr PE=2 SV=2	GDLGIEIPAEK

>	sp|P12928|KPY	Pyruvate kinase isozymes R/L OS=Rattus norvegicus GN=Pklr PE=2 SV=2	GDLGIEIPAEK
>	sp|P12928|KPY	Pyruvate kinase isozymes R/L OS=Rattus norvegicus GN=Pklr PE=2 SV=2	VFLAQK
>	sp|P12928|KPY	Pyruvate kinase isozymes R/L OS=Rattus norvegicus GN=Pklr PE=2 SV=2	AAVIAVTRSAQAAR
>	sp|P12928|KPY	Pyruvate kinase isozymes R/L OS=Rattus norvegicus GN=Pklr PE=2 SV=2	GDLGIEIPAEK
>	sp|P12928|KPY	Pyruvate kinase isozymes R/L OS=Rattus norvegicus GN=Pklr PE=2 SV=2	VFLAQK
>	sp|P12928|KPY	Pyruvate kinase isozymes R/L OS=Rattus norvegicus GN=Pklr PE=2 SV=2	AAVIAVTRSAQAAR
>	RRRRRsp|Q9JIR	REVERSED Peripheral-type benzodiazepine receptor-associated protein 1 OS=Rattus norvegicus GN=Bzrap1 PE=1 SV=2	EVQEK
>	RRRRRsp|Q9JIR	REVERSED Peripheral-type benzodiazepine receptor-associated protein 1 OS=Rattus norvegicus GN=Bzrap1 PE=1 SV=2	AQLERCER
>	RRRRRsp|Q090	REVERSED ADP/ATP translocase 2 OS=Rattus norvegicus GN=Slc25a5 PE=1 SV=3	GGEDRAIK
>	RRRRRsp|Q629	REVERSED SH2B adapter protein 1 OS=Rattus norvegicus GN=Sh2b1 PE=1 SV=1	GVSRLSFR
>	sp|O88204|LRF	Low-density lipoprotein receptor-related protein 3 OS=Rattus norvegicus GN=Lrp3 PE=1 SV=1	LSSAVPALAACSGK
>	sp|Q1WIM3|C	Cell adhesion molecule 3 OS=Rattus norvegicus GN=Cadm3 PE=2 SV=1	IEVLYTPTAMIRPEPAHPR
>	RRRRRsp|Q9Z1	REVERSED Hamartin OS=Rattus norvegicus GN=Tsc1 PE=1 SV=1	LEAIMSRCDELK
>	RRRRRsp|Q9W	REVERSED SH3 and multiple ankyrin repeat domains protein 1 OS=Rattus norvegicus GN=Shank1 PE=1 SV=1	RAAPPSATLAK
>	sp|Q9Z0U5|AD	Aldehyde oxidase OS=Rattus norvegicus GN=Aox1 PE=2 SV=1	LEPIISK
>	sp|O35867|NE	Neurabin-1 OS=Rattus norvegicus GN=Ppp1r9a PE=1 SV=1	IRDLEAEVFR
>	RRRRRsp|O086	REVERSED Homeobox protein Hox-A1 OS=Rattus norvegicus GN=Hoxa1 PE=2 SV=1	VANPQGVYGYEGVK
>	sp|Q9ER30|KB	Kelch repeat and BTB domain-containing protein 10 OS=Rattus norvegicus GN=Kbtbd10 PE=1 SV=1	EWAGMLK
>	sp|P62718|RL1	60S ribosomal protein L18a OS=Rattus norvegicus GN=Rpl18a PE=2 SV=1	IKFPLPHR
>	sp|P63036|DN	DnaJ homolog subfamily A member 1 OS=Rattus norvegicus GN=Dnaja1 PE=2 SV=1	ILEVHIDK
>	RRRRRsp|P363	REVERSED Antigen peptide transporter 1 OS=Rattus norvegicus GN=Tap1 PE=1 SV=2	AGATVGLVGR
>	sp|Q63170|DY	Dynein heavy chain 7, axonemal OS=Rattus norvegicus GN=Dnah7 PE=2 SV=2	LDAAADK
>	RRRRRsp|Q9ER	REVERSED Myosin-XVI OS=Rattus norvegicus GN=Myo16 PE=1 SV=1	GVASRR
>	RRRRRsp|Q019	REVERSED Potassium voltage-gated channel subfamily C member 3 OS=Rattus norvegicus GN=Kcnc3 PE=2 SV=1	VGGVNIVIK
>	sp|P19490|GRI	Glutamate receptor 1 OS=Rattus norvegicus GN=Gria1 PE=1 SV=2	GLSVLQR
>	sp|Q9QYU4|CR	Mu-crystallin homolog OS=Rattus norvegicus GN=Crym PE=1 SV=1	SLGMAVEDLVAAK
>	RRRRRsp|Q6IG	REVERSED Keratin, type II cytoskeletal 72 OS=Rattus norvegicus GN=Krt72 PE=2 SV=2	LDLQR
>	sp|Q64627|NE	Sialidase-2 OS=Rattus norvegicus GN=Neu2 PE=2 SV=1	AGSLLVPAYAYR
>	sp|Q9JIS1|RIM	Regulating synaptic membrane exocytosis protein 2 OS=Rattus norvegicus GN=Rims2 PE=1 SV=1	KIIQAVMDR
>	RRRRRsp|Q6M	REVERSED Bromodomain-containing protein 2 OS=Rattus norvegicus GN=Brd2 PE=2 SV=1	IIDHYDHLGLASADVPK
>	sp|Q6AYB4|HS	Heat shock 70 kDa protein 14 OS=Rattus norvegicus GN=Hspa14 PE=2 SV=1	VTPAVVAYSER
>	sp|Q62685|NR	Oxysterols receptor LXR-alpha OS=Rattus norvegicus GN=Nr1h3 PE=2 SV=1	TSAIEVMLLETSRR
>	sp|P10362|SC	Secretogranin-2 OS=Rattus norvegicus GN=Scg2 PE=2 SV=1	DQLSEDASKVITYLR
>	sp|O89000|DP	Dihydropyrimidine dehydrogenase [NADP+] OS=Rattus norvegicus GN=Dpyd PE=2 SV=1	DFLPLVAK
>	RRRRRsp|O351	REVERSED Short transient receptor potential channel 4 OS=Rattus norvegicus GN=Trpc4 PE=1 SV=2	ESAIVASRPHILTTLDFLSLNKR
>	sp|P84903|STI	Stromal interaction molecule 1 OS=Rattus norvegicus GN=Stim1 PE=1 SV=1	QALSEVTAALR
>	RRRRRsp|Q633	REVERSED Neurexin-2-alpha OS=Rattus norvegicus GN=Nrxn2 PE=1 SV=3	LSVDEAETHMANPLMIKMYMSGDYSLVTAER
>	RRRRRsp|P355	REVERSED Insulin receptor substrate 1 OS=Rattus norvegicus GN=Irs1 PE=1 SV=1	APGGAESAAR
>	sp|Q01205|OD	Dihydrolipoyllysine-residue succinyltransferase component of 2-oxoglutarate dehydrogenase complex, mitochondrial OS=Rattus norvegicus GN=Dlst PE=1 SV=2	LGFMSAFVK
>	sp|Q01205|OD	Dihydrolipoyllysine-residue succinyltransferase component of 2-oxoglutarate dehydrogenase complex, mitochondrial OS=Rattus norvegicus GN=Dlst PE=1 SV=2	ASAFALQEQPVVNAVIDDATK
>	sp|Q01205|OD	Dihydrolipoyllysine-residue succinyltransferase component of 2-oxoglutarate dehydrogenase complex, mitochondrial OS=Rattus norvegicus GN=Dlst PE=1 SV=2	VEVRPMMYVALTYDHR
>	sp|Q01205|OD	Dihydrolipoyllysine-residue succinyltransferase component of 2-oxoglutarate dehydrogenase complex, mitochondrial OS=Rattus norvegicus GN=Dlst PE=1 SV=2	LGFMSAFVK
>	sp|Q01205|OD	Dihydrolipoyllysine-residue succinyltransferase component of 2-oxoglutarate dehydrogenase complex, mitochondrial OS=Rattus norvegicus GN=Dlst PE=1 SV=2	LGFMSAFVK
>	RRRRRsp|Q9JM	REVERSED Pre-mRNA-processing factor 19 OS=Rattus norvegicus GN=Prpf19 PE=2 SV=2	LTALAER
>	RRRRRsp|Q8R2	REVERSED NF-kappa-B inhibitor-like protein 1 OS=Rattus norvegicus GN=Nfkbil1 PE=2 SV=2	ARERFLR
>	sp|Q9Z286|AD	Adenylate cyclase type 10 OS=Rattus norvegicus GN=Adcy10 PE=1 SV=1	ILEQTVR
>	sp|Q5EXX3|ZB	Zinc finger and BTB domain-containing protein 38 OS=Rattus norvegicus GN=Zbtb38 PE=2 SV=1	HERIHLGVK
>	RRRRRsp|Q6M	REVERSED Estradiol 17-beta-dehydrogenase 8 OS=Rattus norvegicus GN=Hsd17b8 PE=2 SV=1	AATQTLGIVGAK
>	sp|Q4G056|TY	Tyrosyl-DNA phosphodiesterase 1 OS=Rattus norvegicus GN=Tdp1 PE=2 SV=1	LAWFLVTSANLSK
>	sp|Q9WTL3|SE	Semaphorin-6C OS=Rattus norvegicus GN=Sema6c PE=2 SV=1	LGVSGSR
>	RRRRRsp|Q5P	REVERSED Pleckstrin homology-like domain family A member 3 OS=Rattus norvegicus GN=Phlda3 PE=2 SV=1	VTQIAQQNK
>	sp|Q6XVN8|M	Microtubule-associated proteins 1A/1B light chain 3A OS=Rattus norvegicus GN=Map1lc3a PE=1 SV=1	QLPVLDK
>	sp|Q9EPI8|MT	Transcription termination factor, mitochondrial OS=Rattus norvegicus GN=Mterf PE=1 SV=1	AITRTPESLSK
>	sp|Q9EPI8|MT	Transcription termination factor, mitochondrial OS=Rattus norvegicus GN=Mterf PE=1 SV=1	LFDLR
>	sp|P50399|GDI	Rab GDP dissociation inhibitor beta OS=Rattus norvegicus GN=Gdi2 PE=1 SV=2	ICILSHPIK
>	sp|P50399|GDI	Rab GDP dissociation inhibitor beta OS=Rattus norvegicus GN=Gdi2 PE=1 SV=2	FLVYVANFDEKDPR
>	RRRRRsp|O359	REVERSED Breast cancer type 2 susceptibility protein homolog OS=Rattus norvegicus GN=Brca2 PE=1 SV=1	LSLVGHTNHVVSSER
>	RRRRRsp|O359	REVERSED Breast cancer type 2 susceptibility protein homolog OS=Rattus norvegicus GN=Brca2 PE=1 SV=1	QGVTLRGSK
>	sp|Q62826|HN	Heterogeneous nuclear ribonucleoprotein M OS=Rattus norvegicus GN=Hnrnpm PE=1 SV=4	ADILEDK
>	sp|B2GV50|RF	DNA-binding protein RFX2 OS=Rattus norvegicus GN=Rfx2 PE=2 SV=1	LGTRGNSK
>	sp|Q68FQ0|TC	T-complex protein 1 subunit epsilon OS=Rattus norvegicus GN=Cct5 PE=1 SV=1	IAIQHLDK
>	RRRRRsp|Q5RJ	REVERSED Zinc finger protein 467 OS=Rattus norvegicus GN=Znf467 PE=2 SV=1	AGSHARSHAVLNPR
>	sp|Q8R491|EH	EH domain-containing protein 3 OS=Rattus norvegicus GN=Ehd3 PE=1 SV=2	LADIDKDGMLDDEEFALANHLIK
>	sp|Q5XIF9|S25	Solute carrier family 25 member 34 OS=Rattus norvegicus GN=Slc25a34 PE=2 SV=2	VTVGSAAQLATFTSAK
>	RRRRRsp|Q8CI	REVERSED Dual oxidase 1 OS=Rattus norvegicus GN=Duox1 PE=1 SV=1	LAARINEVLAQR
>	RRRRRsp|O886	REVERSED Serine/threonine-protein kinase TAO1 OS=Rattus norvegicus GN=Taok1 PE=1 SV=1	IRQLFK
>	RRRRRsp|O886	REVERSED Serine/threonine-protein kinase TAO1 OS=Rattus norvegicus GN=Taok1 PE=1 SV=1	IRQLFK
>	sp|Q9QYP2|CE	Cadherin EGF LAG seven-pass G-type receptor 2 (Fragment) OS=Rattus norvegicus GN=Celsr2 PE=2 SV=1	ALTEVR
>	sp|Q2WEA5|T	Transient receptor potential cation channel subfamily M member 1 OS=Rattus norvegicus GN=Trpm1 PE=2 SV=1	SDLIQARSR
>	RRRRRsp|Q9Q	REVERSED Programmed cell death 6-interacting protein OS=Rattus norvegicus GN=Pdcd6ip PE=1 SV=2	VSGGFLSGK
>	RRRRRsp|Q633	REVERSED Neurexin-1-alpha OS=Rattus norvegicus GN=Nrxn1 PE=1 SV=3	TPPKGRR
>	RRRRRsp|Q626	REVERSED Inactive phospholipase C-like protein 1 OS=Rattus norvegicus GN=Plcl1 PE=1 SV=1	IISTRRPTAR
>	sp|Q75WE7|V	von Willebrand factor A domain-containing protein 5A OS=Rattus norvegicus GN=Vwa5a PE=2 SV=1	LDADLGGTEILTPLR
>	sp|O88626|GA	Galanin receptor type 3 OS=Rattus norvegicus GN=Galr3 PE=2 SV=1	GGGETRLTLSPR

>	sp|P49301|MN	Macrophage asialoglycoprotein-binding protein 1 OS=Rattus norvegicus GN=Mgl1 PE=1 SV=1	EQTLRTDLSEITDR
>	sp|B0K020|CISI	CDGSH iron-sulfur domain-containing protein 1 OS=Rattus norvegicus GN=Cisd1 PE=3 SV=1	VVHAFDMEDLGDK
>	sp|B0K020|CISI	CDGSH iron-sulfur domain-containing protein 1 OS=Rattus norvegicus GN=Cisd1 PE=3 SV=1	VVHAFDMEDLGDK
>	sp|Q5BJT0|ARC	Arginine and glutamate-rich protein 1 OS=Rattus norvegicus GN=Arglu1 PE=2 SV=1	QLLEELER
>	sp|Q5BJT0|ARC	Arginine and glutamate-rich protein 1 OS=Rattus norvegicus GN=Arglu1 PE=2 SV=1	QLLEELER
>	sp|Q5XI32|CAP	F-actin-capping protein subunit beta OS=Rattus norvegicus GN=Capzb PE=1 SV=1	EVQEK
>	sp|Q5XI32|CAP	F-actin-capping protein subunit beta OS=Rattus norvegicus GN=Capzb PE=1 SV=1	LVEDMENK
>	sp|Q2Q0I9|FNI	Fibronectin type III domain-containing protein 1 OS=Rattus norvegicus GN=Fndc1 PE=2 SV=2	LSVAWK
>	RRRRRsp|Q923	REVERSED Trace amine-associated receptor 8b OS=Rattus norvegicus GN=Taar8b PE=3 SV=1	TPYVLPETVAIYR
>	RRRRRsp|P551	REVERSED Nck-associated protein 1 OS=Rattus norvegicus GN=Nckap1 PE=2 SV=1	LLSSSALALFEKLR
>	sp|Q7TSP2|KIF	Kinesin-like protein KIF15 OS=Rattus norvegicus GN=Kif15 PE=2 SV=1	ALETVR
>	sp|Q62925|M3	Mitogen-activated protein kinase kinase kinase 1 OS=Rattus norvegicus GN=Map3k1 PE=1 SV=1	LSRRIYLSSAR
>	sp|Q499P3|C1	C1GALT1-specific chaperone 1 OS=Rattus norvegicus GN=C1galt1c1 PE=1 SV=1	MLSESSSFLK
>	sp|P19132|FRII	Ferritin heavy chain OS=Rattus norvegicus GN=Fth1 PE=1 SV=3	IFLQDIK
>	RRRRRsp|Q6AY	REVERSED Uncharacterized protein C12orf41 homolog OS=Rattus norvegicus PE=2 SV=1	QSSRTTHAQQAAGETALMR
>	sp|P31503|PO	POU domain, class 2, transcription factor 1 (Fragment) OS=Rattus norvegicus GN=Pou2f1 PE=2 SV=2	TIAATPIQTLPQSQTTPK
>	sp|Q6NYB7|RA	Ras-related protein Rab-1A OS=Rattus norvegicus GN=Rab1A PE=1 SV=3	LLLIGDSGVGK
>	sp|Q6NYB7|RA	Ras-related protein Rab-1A OS=Rattus norvegicus GN=Rab1A PE=1 SV=3	LQIWDTAGQER
>	sp|Q6NYB7|RA	Ras-related protein Rab-1A OS=Rattus norvegicus GN=Rab1A PE=1 SV=3	LQIWDTAGQER
>	sp|Q6NYB7|RA	Ras-related protein Rab-1A OS=Rattus norvegicus GN=Rab1A PE=1 SV=3	LQIWDTAGQER
>	sp|Q6NYB7|RA	Ras-related protein Rab-1A OS=Rattus norvegicus GN=Rab1A PE=1 SV=3	LQIWDTAGQER
>	sp|P48675|DES	Desmin OS=Rattus norvegicus GN=Des PE=2 SV=2	EYQDLLNVK
>	sp|P48675|DES	Desmin OS=Rattus norvegicus GN=Des PE=2 SV=2	KLLEGEESR
>	sp|P48675|DES	Desmin OS=Rattus norvegicus GN=Des PE=2 SV=2	GSEVHTK
>	sp|P48675|DES	Desmin OS=Rattus norvegicus GN=Des PE=2 SV=2	KLLEGEESR
>	sp|O88778|BSN	Protein bassoon OS=Rattus norvegicus GN=Bsn PE=1 SV=3	LQLEQIQQLQQQLQQQLEEQK
>	sp|Q6GV29|B3	UDP-GlcNAc:betaGal beta-1,3-N-acetylglucosaminyltransferase-like protein 1 OS=Rattus norvegicus GN=B3gntl1 PE=2 SV=1	GGGVFRVDHSLLLYR
>	sp|Q62814|E2F	Transcription factor E2F5 (Fragment) OS=Rattus norvegicus GN=E2f5 PE=2 SV=1	GVGAGCNTKEVIDRLR
>	sp|Q63787|P8	Phosphatidylinositol 3-kinase regulatory subunit alpha OS=Rattus norvegicus GN=Pik3r1 PE=1 SV=1	LLEAIEK
>	RRRRRsp|Q625	REVERSED Metastasis-associated protein MTA1 OS=Rattus norvegicus GN=Mta1 PE=1 SV=1	AEVNGNATK
>	sp|P31423|GRI	Metabotropic glutamate receptor 4 OS=Rattus norvegicus GN=Grm4 PE=1 SV=1	LSRHALK
>	sp|Q9Z250|LIN	Protein lin-7 homolog A OS=Rattus norvegicus GN=Lin7a PE=1 SV=2	AIELLEK
>	RRRRRsp|Q634	REVERSED Ras-related GTP-binding protein B OS=Rattus norvegicus GN=RragB PE=1 SV=1	DLITAGLRR
>	RRRRRsp|P120	REVERSED 60S ribosomal protein L18 OS=Rattus norvegicus GN=Rpl18 PE=2 SV=2	VTGRGK
>	sp|Q6MGD0|CI	Protein CutA OS=Rattus norvegicus GN=Cuta PE=1 SV=2	TQSSLVPALTEFVR
>	sp|P15429|EN	Beta-enolase OS=Rattus norvegicus GN=Eno3 PE=1 SV=3	HIADLAGNPDLVLPVPAFNVINGGSHAGNK
>	sp|P15429|EN	Beta-enolase OS=Rattus norvegicus GN=Eno3 PE=1 SV=3	AAVPSGASTGIYEALELR
>	sp|P15429|EN	Beta-enolase OS=Rattus norvegicus GN=Eno3 PE=1 SV=3	AAVPSGASTGIYEALELR
>	sp|P15429|EN	Beta-enolase OS=Rattus norvegicus GN=Eno3 PE=1 SV=3	AAVPSGASTGIYEALELR
>	sp|P15429|EN	Beta-enolase OS=Rattus norvegicus GN=Eno3 PE=1 SV=3	AAVPSGASTGIYEALELR
>	sp|P15429|EN	Beta-enolase OS=Rattus norvegicus GN=Eno3 PE=1 SV=3	AAVPSGASTGIYEALELR
>	sp|P15429|EN	Beta-enolase OS=Rattus norvegicus GN=Eno3 PE=1 SV=3	AAVPSGASTGIYEALELR
>	sp|P15429|EN	Beta-enolase OS=Rattus norvegicus GN=Eno3 PE=1 SV=3	AAVPSGASTGIYEALELR
>	sp|P15429|EN	Beta-enolase OS=Rattus norvegicus GN=Eno3 PE=1 SV=3	AAVPSGASTGIYEALELR
>	sp|P15429|EN	Beta-enolase OS=Rattus norvegicus GN=Eno3 PE=1 SV=3	FGANAILGVSLAVCK
>	sp|P15429|EN	Beta-enolase OS=Rattus norvegicus GN=Eno3 PE=1 SV=3	GNPTVEVDLHTAK
>	sp|P15429|EN	Beta-enolase OS=Rattus norvegicus GN=Eno3 PE=1 SV=3	HIADLAGNPDLVLPVPAFNVINGGSHAGNK
>	sp|P15429|EN	Beta-enolase OS=Rattus norvegicus GN=Eno3 PE=1 SV=3	HIADLAGNPDLVLPVPAFNVINGGSHAGNK
>	sp|P15429|EN	Beta-enolase OS=Rattus norvegicus GN=Eno3 PE=1 SV=3	HIADLAGNPDLVLPVPAFNVINGGSHAGNK
>	sp|P15429|EN	Beta-enolase OS=Rattus norvegicus GN=Eno3 PE=1 SV=3	HIADLAGNPDLVLPVPAFNVINGGSHAGNK
>	sp|P15429|EN	Beta-enolase OS=Rattus norvegicus GN=Eno3 PE=1 SV=3	HIADLAGNPDLVLPVPAFNVINGGSHAGNK
>	sp|P15429|EN	Beta-enolase OS=Rattus norvegicus GN=Eno3 PE=1 SV=3	LAQSNGWGVMVSHR
>	sp|P15429|EN	Beta-enolase OS=Rattus norvegicus GN=Eno3 PE=1 SV=3	LAQSNGWGVMVSHR
>	sp|P15429|EN	Beta-enolase OS=Rattus norvegicus GN=Eno3 PE=1 SV=3	PVPAFNVINGGSHAGNK
>	sp|P15429|EN	Beta-enolase OS=Rattus norvegicus GN=Eno3 PE=1 SV=3	SGETEDTFIADLVVGLCTGQIK
>	sp|P15429|EN	Beta-enolase OS=Rattus norvegicus GN=Eno3 PE=1 SV=3	SGETEDTFIADLVVGLCTGQIK
>	sp|P15429|EN	Beta-enolase OS=Rattus norvegicus GN=Eno3 PE=1 SV=3	SGETEDTFIADLVVGLCTGQIK
>	sp|P15429|EN	Beta-enolase OS=Rattus norvegicus GN=Eno3 PE=1 SV=3	SGETEDTFIADLVVGLCTGQIK
>	sp|P15429|EN	Beta-enolase OS=Rattus norvegicus GN=Eno3 PE=1 SV=3	SGETEDTFIADLVVGLCTGQIK
>	sp|P15429|EN	Beta-enolase OS=Rattus norvegicus GN=Eno3 PE=1 SV=3	SGETEDTFIADLVVGLCTGQIK
>	sp|P15429|EN	Beta-enolase OS=Rattus norvegicus GN=Eno3 PE=1 SV=3	VNQIGSVTESIQACK
>	sp|P15429|EN	Beta-enolase OS=Rattus norvegicus GN=Eno3 PE=1 SV=3	VNQIGSVTESIQACK
>	sp|P15429|EN	Beta-enolase OS=Rattus norvegicus GN=Eno3 PE=1 SV=3	HIADLAGNPDLVLPVPAFNVINGGSHAGNK
>	sp|P15429|EN	Beta-enolase OS=Rattus norvegicus GN=Eno3 PE=1 SV=3	HIADLAGNPDLVLPVPAFNVINGGSHAGNK
>	sp|P15429|EN	Beta-enolase OS=Rattus norvegicus GN=Eno3 PE=1 SV=3	FGANAILGVSLAVCK
>	sp|P15429|EN	Beta-enolase OS=Rattus norvegicus GN=Eno3 PE=1 SV=3	HIADLAGNPDLVLPVPAFNVINGGSHAGNK
>	sp|P15429|EN	Beta-enolase OS=Rattus norvegicus GN=Eno3 PE=1 SV=3	AVEHINK
>	sp|P15429|EN	Beta-enolase OS=Rattus norvegicus GN=Eno3 PE=1 SV=3	LAQSNGWGVMVSHR
>	sp|P15429|EN	Beta-enolase OS=Rattus norvegicus GN=Eno3 PE=1 SV=3	FGANAILGVSLAVCK
>	sp|P15429|EN	Beta-enolase OS=Rattus norvegicus GN=Eno3 PE=1 SV=3	GNPTVEVDLHTAK
>	sp|P15429|EN	Beta-enolase OS=Rattus norvegicus GN=Eno3 PE=1 SV=3	AVEHINK
>	sp|P15429|EN	Beta-enolase OS=Rattus norvegicus GN=Eno3 PE=1 SV=3	FGANAILGVSLAVCK
>	sp|P15429|EN	Beta-enolase OS=Rattus norvegicus GN=Eno3 PE=1 SV=3	FRAAVPSGASTGIYEALELR
>	sp|P15429|EN	Beta-enolase OS=Rattus norvegicus GN=Eno3 PE=1 SV=3	AAVPSGASTGIYEALELR

>	sp|P15429|ENO	Beta-enolase OS=Rattus norvegicus GN=Eno3 PE=1 SV=3	HIADLAGNPDLVLPVPAFNVINGGSHAGNK
>	sp|P15429|ENO	Beta-enolase OS=Rattus norvegicus GN=Eno3 PE=1 SV=3	HIADLAGNPDLVLPVPAFNVINGGSHAGNK
>	sp|P15429|ENO	Beta-enolase OS=Rattus norvegicus GN=Eno3 PE=1 SV=3	HIADLAGNPDLVLPVPAFNVINGGSHAGNK
>	sp|P15429|ENO	Beta-enolase OS=Rattus norvegicus GN=Eno3 PE=1 SV=3	HIADLAGNPDLVLPVPAFNVINGGSHAGNK
>	sp|P15429|ENO	Beta-enolase OS=Rattus norvegicus GN=Eno3 PE=1 SV=3	HIADLAGNPDLVLPVPAFNVINGGSHAGNK
>	sp|P15429|ENO	Beta-enolase OS=Rattus norvegicus GN=Eno3 PE=1 SV=3	HIADLAGNPDLVLPVPAFNVINGGSHAGNK
>	sp|P15429|ENO	Beta-enolase OS=Rattus norvegicus GN=Eno3 PE=1 SV=3	HIADLAGNPDLVLPVPAFNVINGGSHAGNK
>	sp|P15429|ENO	Beta-enolase OS=Rattus norvegicus GN=Eno3 PE=1 SV=3	HIADLAGNPDLVLPVPAFNVINGGSHAGNK
>	sp|P15429|ENO	Beta-enolase OS=Rattus norvegicus GN=Eno3 PE=1 SV=3	HIADLAGNPDLVLPVPAFNVINGGSHAGNK
>	sp|P15429|ENO	Beta-enolase OS=Rattus norvegicus GN=Eno3 PE=1 SV=3	AVEHINK
>	sp|P15429|ENO	Beta-enolase OS=Rattus norvegicus GN=Eno3 PE=1 SV=3	GNPTVEVDLHTAK
>	sp|P15429|ENO	Beta-enolase OS=Rattus norvegicus GN=Eno3 PE=1 SV=3	HIADLAGNPDLVLPVPAFNVINGGSHAGNK
>	sp|P15429|ENO	Beta-enolase OS=Rattus norvegicus GN=Eno3 PE=1 SV=3	HIADLAGNPDLVL
>	sp|P15429|ENO	Beta-enolase OS=Rattus norvegicus GN=Eno3 PE=1 SV=3	PSGASTGIYEALELR
>	sp|P15429|ENO	Beta-enolase OS=Rattus norvegicus GN=Eno3 PE=1 SV=3	LAQSNGWGVMVSHR
>	sp|P15429|ENO	Beta-enolase OS=Rattus norvegicus GN=Eno3 PE=1 SV=3	HIADLAGNPDLVLPVPAFNVINGGSHAGNK
>	sp|P15429|ENO	Beta-enolase OS=Rattus norvegicus GN=Eno3 PE=1 SV=3	NGKYDLDFK
>	sp|P15429|ENO	Beta-enolase OS=Rattus norvegicus GN=Eno3 PE=1 SV=3	LAQSNGWGVMVSHR
>	sp|P15429|ENO	Beta-enolase OS=Rattus norvegicus GN=Eno3 PE=1 SV=3	LAQSNGWGVMVSHR
>	sp|P11517|HBB	Hemoglobin subunit beta-2 OS=Rattus norvegicus PE=1 SV=2	GTFAHLSELHCDKLHVDPENFR
>	sp|P11517|HBB	Hemoglobin subunit beta-2 OS=Rattus norvegicus PE=1 SV=2	GTFAHLSELHCDKLHVDPENFR
>	sp|P11517|HBB	Hemoglobin subunit beta-2 OS=Rattus norvegicus PE=1 SV=2	LLGNMIVIVLGHHLGK
>	sp|P11517|HBB	Hemoglobin subunit beta-2 OS=Rattus norvegicus PE=1 SV=2	LLVVYPWTQR
>	sp|P11517|HBB	Hemoglobin subunit beta-2 OS=Rattus norvegicus PE=1 SV=2	LLVVYPWTQR
>	sp|P11517|HBB	Hemoglobin subunit beta-2 OS=Rattus norvegicus PE=1 SV=2	VHLTDAEK
>	sp|P11517|HBB	Hemoglobin subunit beta-2 OS=Rattus norvegicus PE=1 SV=2	VHLTDAEK
>	sp|P11517|HBB	Hemoglobin subunit beta-2 OS=Rattus norvegicus PE=1 SV=2	VHLTDAEK
>	sp|P11517|HBB	Hemoglobin subunit beta-2 OS=Rattus norvegicus PE=1 SV=2	VHLTDAEK
>	sp|P11517|HBB	Hemoglobin subunit beta-2 OS=Rattus norvegicus PE=1 SV=2	VHLTDAEK
>	sp|P11517|HBB	Hemoglobin subunit beta-2 OS=Rattus norvegicus PE=1 SV=2	VINAFNDGLK
>	sp|P11517|HBB	Hemoglobin subunit beta-2 OS=Rattus norvegicus PE=1 SV=2	VVAGVASALAHKYH
>	sp|P11517|HBB	Hemoglobin subunit beta-2 OS=Rattus norvegicus PE=1 SV=2	VVAGVASALAHKYH
>	sp|P11517|HBB	Hemoglobin subunit beta-2 OS=Rattus norvegicus PE=1 SV=2	VINAFNDGLK
>	sp|P11517|HBB	Hemoglobin subunit beta-2 OS=Rattus norvegicus PE=1 SV=2	VINAFNDGLK
>	sp|P11517|HBB	Hemoglobin subunit beta-2 OS=Rattus norvegicus PE=1 SV=2	HLDNLK
>	sp|P11517|HBB	Hemoglobin subunit beta-2 OS=Rattus norvegicus PE=1 SV=2	FGDLSSASAIMGNPQVK
>	sp|P11517|HBB	Hemoglobin subunit beta-2 OS=Rattus norvegicus PE=1 SV=2	VHLTDAEK
>	sp|P11517|HBB	Hemoglobin subunit beta-2 OS=Rattus norvegicus PE=1 SV=2	LLGNMIVIVLGHHLGKEFTPCAQAAFQK
>	sp|P11517|HBB	Hemoglobin subunit beta-2 OS=Rattus norvegicus PE=1 SV=2	LLVVYPWTQR
>	sp|P11517|HBB	Hemoglobin subunit beta-2 OS=Rattus norvegicus PE=1 SV=2	FGDLSSASAIMGNPQVK
>	sp|P11517|HBB	Hemoglobin subunit beta-2 OS=Rattus norvegicus PE=1 SV=2	KVINAFNDGLK
>	sp|P11517|HBB	Hemoglobin subunit beta-2 OS=Rattus norvegicus PE=1 SV=2	GTFAHLSELHCDKLHVDPENFR
>	sp|P11517|HBB	Hemoglobin subunit beta-2 OS=Rattus norvegicus PE=1 SV=2	LLVVYPWTQR
>	sp|P11517|HBB	Hemoglobin subunit beta-2 OS=Rattus norvegicus PE=1 SV=2	LLVVYPWTQR
>	sp|P11517|HBB	Hemoglobin subunit beta-2 OS=Rattus norvegicus PE=1 SV=2	ATVSGLWGK
>	sp|Q9WVB1|RA	Ras-related protein Rab-6A OS=Rattus norvegicus GN=Rab6a PE=2 SV=2	LQLWDTAGQER
>	sp|Q9WVB1|RA	Ras-related protein Rab-6A OS=Rattus norvegicus GN=Rab6a PE=2 SV=2	LQLWDTAGQER
>	sp|Q9WVB1|RA	Ras-related protein Rab-6A OS=Rattus norvegicus GN=Rab6a PE=2 SV=2	LQLWDTAGQER
>	sp|Q9WVB1|RA	Ras-related protein Rab-6A OS=Rattus norvegicus GN=Rab6a PE=2 SV=2	GSDVIIMLVGNK
>	sp|Q9WVB1|RA	Ras-related protein Rab-6A OS=Rattus norvegicus GN=Rab6a PE=2 SV=2	LQLWDTAGQER
>	RRRRRsp|Q9QZ	REVERSED PDZ domain-containing protein 2 OS=Rattus norvegicus GN=Pdzd2 PE=1 SV=1	ESSGEK
>	RRRRRsp|Q044	REVERSED Valyl-tRNA synthetase OS=Rattus norvegicus GN=Vars PE=2 SV=2	VAEALRSR
>	RRRRRsp|Q044	REVERSED Valyl-tRNA synthetase OS=Rattus norvegicus GN=Vars PE=2 SV=2	IAELR
>	sp|O88279|SLIT	Slit homolog 1 protein OS=Rattus norvegicus GN=Slit1 PE=1 SV=1	INLSNNK
>	sp|B1H234|FLR	Leucine-rich repeat transmembrane protein FLRT3 OS=Rattus norvegicus GN=Flrt3 PE=2 SV=1	ISTISSPSLHGLTSLK
>	RRRRRsp|P0868	REVERSED Sex hormone-binding globulin OS=Rattus norvegicus GN=Shbg PE=1 SV=1	LSAVEGK
>	sp|Q9JJ79|DYH	Cytoplasmic dynein 2 heavy chain 1 OS=Rattus norvegicus GN=Dync2h1 PE=1 SV=1	SDTNLPK
>	sp|P84100|RL1	60S ribosomal protein L19 OS=Rattus norvegicus GN=Rpl19 PE=1 SV=1	ILMEHIHK
>	sp|Q66H44|TM	Transmembrane protein 39B OS=Rattus norvegicus GN=Tmem39b PE=2 SV=1	EAVSGLARSR
>	RRRRRsp|Q562	REVERSED Serine/threonine-protein phosphatase PGAM5, mitochondrial OS=Rattus norvegicus GN=Pgam5 PE=2 SV=1	SIIDTTEVARTMSSHVIK
>	RRRRRsp|D3ZH	REVERSED Microtubule-actin cross-linking factor 1 OS=Rattus norvegicus GN=Macf1 PE=1 SV=1	EVQQK
>	sp|Q9JLU4|SHA	SH3 and multiple ankyrin repeat domains protein 3 OS=Rattus norvegicus GN=Shank3 PE=1 SV=2	LLGTGATTGR
>	RRRRRsp|Q9WT	REVERSED Glutamate receptor-interacting protein 2 OS=Rattus norvegicus GN=Grip2 PE=1 SV=1	LSVSNIALIR
>	sp|P58405|STR	Striatin-3 OS=Rattus norvegicus GN=Strn3 PE=2 SV=2	AEEAEPITFPSGGGK
>	RRRRRsp|P3543	REVERSED Glutamate [NMDA] receptor subunit zeta-1 OS=Rattus norvegicus GN=Grin1 PE=1 SV=1	NAQNVAER
>	RRRRRsp|Q4V8	REVERSED Dual specificity tyrosine-phosphorylation-regulated kinase 3 OS=Rattus norvegicus GN=Dyrk3 PE=2 SV=1	QQPPLGAGPLAADKRGR
>	RRRRRsp|Q9Z3	REVERSED DNA (cytosine-5)-methyltransferase 1 OS=Rattus norvegicus GN=Dnmt1 PE=1 SV=2	DLINGFLR
>	sp|B2RZ37|REE	Receptor expression-enhancing protein 5 OS=Rattus norvegicus GN=Reep5 PE=1 SV=1	KATVNLLGDEK
>	RRRRRsp|Q9EQ	REVERSED C-terminal-binding protein 2 OS=Rattus norvegicus GN=Ctbp2 PE=1 SV=2	IRAAGSAVER
>	sp|P52481|CAP	Adenylyl cyclase-associated protein 2 OS=Rattus norvegicus GN=Cap2 PE=2 SV=1	VLAGDVETHAEMVHGAFQAQR
>	sp|P52481|CAP	Adenylyl cyclase-associated protein 2 OS=Rattus norvegicus GN=Cap2 PE=2 SV=1	AFLLMVSQHQQPQENEVAVLLKPISEK
>	sp|P52481|CAP	Adenylyl cyclase-associated protein 2 OS=Rattus norvegicus GN=Cap2 PE=2 SV=1	AFLLMVSQHQQPQENEVAVLLKPISEK
>	sp|P52481|CAP	Adenylyl cyclase-associated protein 2 OS=Rattus norvegicus GN=Cap2 PE=2 SV=1	VLAGDVETHAEMVHGAFQAQR
>	sp|P52481|CAP	Adenylyl cyclase-associated protein 2 OS=Rattus norvegicus GN=Cap2 PE=2 SV=1	VLAGDVETHAEMVHGAFQAQR

>	sp|P52481|CAF	Adenylyl cyclase-associated protein 2 OS=Rattus norvegicus GN=Cap2 PE=2 SV=1	VLAGDVETHAEMVHGAFQAQR
>	sp|P52481|CAF	Adenylyl cyclase-associated protein 2 OS=Rattus norvegicus GN=Cap2 PE=2 SV=1	VLAGDVETHAEMVHGAFQAQR
>	RRRRRsp|P1308	REVERSED Succinyl-CoA ligase [GDP-forming] subunit alpha, mitochondrial OS=Rattus norvegicus GN=Suclg1 PE=2 SV=2	AGGKGGAIIAGAHGMRR
>	sp|Q00495|CSF	Macrophage colony-stimulating factor 1 receptor OS=Rattus norvegicus GN=Csf1r PE=2 SV=1	LEFITQR
>	sp|Q91ZF7|AAI	Alanine and arginine-rich domain-containing protein OS=Rattus norvegicus GN=Aard PE=2 SV=1	QLERAFQRAAAR
>	sp|Q7TSA0|MII	Mitochondrial Rho GTPase 2 OS=Rattus norvegicus GN=Rhot2 PE=2 SV=1	LDQEK
>	RRRRRsp|P0715	REVERSED Dolichyl-diphosphooligosaccharide--protein glycosyltransferase subunit 1 OS=Rattus norvegicus GN=Rpn1 PE=2 SV=1	GAVLREAEVASK
>	sp|Q9JKE3|SCA	Secretory carrier-associated membrane protein 5 OS=Rattus norvegicus GN=Scamp5 PE=2 SV=1	AQEEWTTGAWK
>	sp|Q9JKE3|SCA	Secretory carrier-associated membrane protein 5 OS=Rattus norvegicus GN=Scamp5 PE=2 SV=1	AQEEWTTGAWK
>	sp|P09217|KPC	Protein kinase C zeta type OS=Rattus norvegicus GN=Prkcz PE=1 SV=2	LDNVLLDADGHIK
>	sp|Q9EST9|FGF	Fibroblast growth factor 20 OS=Rattus norvegicus GN=Fgf20 PE=2 SV=1	GGPGSVELAHLHGILRR
>	RRRRRsp|Q9QV	REVERSED Neuropilin-1 OS=Rattus norvegicus GN=Nrp1 PE=1 SV=1	SIAGQTGVATVFR
>	sp|Q64017|GP1	Probable G-protein coupled receptor 176 OS=Rattus norvegicus GN=Gpr176 PE=2 SV=2	VPRVSRVER
>	sp|P51650|SSD	Succinate-semialdehyde dehydrogenase, mitochondrial OS=Rattus norvegicus GN=Aldh5a1 PE=1 SV=2	VSMELGGLAPFIVFDSANVDQAVAGAMASK
>	RRRRRsp|P1448	REVERSED Fibrinogen beta chain OS=Rattus norvegicus GN=Fgb PE=1 SV=4	TLQSIK
>	RRRRRsp|P1707	REVERSED 40S ribosomal protein S19 OS=Rattus norvegicus GN=Rps19 PE=2 SV=3	TMSGVGAGGRLYLHRATSAAR
>	sp|Q08290|CNI	Calponin-1 OS=Rattus norvegicus GN=Cnn1 PE=2 SV=1	LQPGSVKK
>	sp|P56225|PO5	POU domain, class 5, transcription factor 2 OS=Rattus norvegicus GN=Pou5f2 PE=2 SV=2	VDTPTWLSSQAATSR
>	RRRRRsp|Q8VD	REVERSED Ribonuclease pancreatic delta-type OS=Rattus norvegicus GN=Rnase1d PE=3 SV=1	VTAWSEHLFTNVR
>	sp|P68035|ACT	Actin, alpha cardiac muscle 1 OS=Rattus norvegicus GN=Actc1 PE=1 SV=1	VAPEEHPTLLTEAPLNPK
>	sp|P68035|ACT	Actin, alpha cardiac muscle 1 OS=Rattus norvegicus GN=Actc1 PE=1 SV=1	LCYVALDFENEMATAASSSSLEK
>	sp|P68035|ACT	Actin, alpha cardiac muscle 1 OS=Rattus norvegicus GN=Actc1 PE=1 SV=1	AGFAGDDAPR
>	sp|P68035|ACT	Actin, alpha cardiac muscle 1 OS=Rattus norvegicus GN=Actc1 PE=1 SV=1	AGFAGDDAPR
>	sp|P68035|ACT	Actin, alpha cardiac muscle 1 OS=Rattus norvegicus GN=Actc1 PE=1 SV=1	AGFAGDDAPR
>	sp|P68035|ACT	Actin, alpha cardiac muscle 1 OS=Rattus norvegicus GN=Actc1 PE=1 SV=1	AGFAGDDAPR
>	sp|P68035|ACT	Actin, alpha cardiac muscle 1 OS=Rattus norvegicus GN=Actc1 PE=1 SV=1	AVFPSIVGRPR
>	sp|P68035|ACT	Actin, alpha cardiac muscle 1 OS=Rattus norvegicus GN=Actc1 PE=1 SV=1	AVFPSIVGRPR
>	sp|P68035|ACT	Actin, alpha cardiac muscle 1 OS=Rattus norvegicus GN=Actc1 PE=1 SV=1	AVFPSIVGRPR
>	sp|P68035|ACT	Actin, alpha cardiac muscle 1 OS=Rattus norvegicus GN=Actc1 PE=1 SV=1	AVFPSIVGRPR
>	sp|P68035|ACT	Actin, alpha cardiac muscle 1 OS=Rattus norvegicus GN=Actc1 PE=1 SV=1	AVFPSIVGRPR
>	sp|P68035|ACT	Actin, alpha cardiac muscle 1 OS=Rattus norvegicus GN=Actc1 PE=1 SV=1	AVFPSIVGRPR
>	sp|P68035|ACT	Actin, alpha cardiac muscle 1 OS=Rattus norvegicus GN=Actc1 PE=1 SV=1	AVFPSIVGRPR
>	sp|P68035|ACT	Actin, alpha cardiac muscle 1 OS=Rattus norvegicus GN=Actc1 PE=1 SV=1	AVFPSIVGRPR
>	sp|P68035|ACT	Actin, alpha cardiac muscle 1 OS=Rattus norvegicus GN=Actc1 PE=1 SV=1	AVFPSIVGRPR
>	sp|P68035|ACT	Actin, alpha cardiac muscle 1 OS=Rattus norvegicus GN=Actc1 PE=1 SV=1	AVFPSIVGRPR
>	sp|P68035|ACT	Actin, alpha cardiac muscle 1 OS=Rattus norvegicus GN=Actc1 PE=1 SV=1	AVFPSIVGRPR
>	sp|P68035|ACT	Actin, alpha cardiac muscle 1 OS=Rattus norvegicus GN=Actc1 PE=1 SV=1	AVFPSIVGRPR
>	sp|P68035|ACT	Actin, alpha cardiac muscle 1 OS=Rattus norvegicus GN=Actc1 PE=1 SV=1	AVFPSIVGRPR
>	sp|P68035|ACT	Actin, alpha cardiac muscle 1 OS=Rattus norvegicus GN=Actc1 PE=1 SV=1	DSYVGDEAQSK
>	sp|P68035|ACT	Actin, alpha cardiac muscle 1 OS=Rattus norvegicus GN=Actc1 PE=1 SV=1	DSYVGDEAQSK
>	sp|P68035|ACT	Actin, alpha cardiac muscle 1 OS=Rattus norvegicus GN=Actc1 PE=1 SV=1	DSYVGDEAQSK
>	sp|P68035|ACT	Actin, alpha cardiac muscle 1 OS=Rattus norvegicus GN=Actc1 PE=1 SV=1	DSYVGDEAQSK
>	sp|P68035|ACT	Actin, alpha cardiac muscle 1 OS=Rattus norvegicus GN=Actc1 PE=1 SV=1	DSYVGDEAQSK
>	sp|P68035|ACT	Actin, alpha cardiac muscle 1 OS=Rattus norvegicus GN=Actc1 PE=1 SV=1	DSYVGDEAQSK
>	sp|P68035|ACT	Actin, alpha cardiac muscle 1 OS=Rattus norvegicus GN=Actc1 PE=1 SV=1	DSYVGDEAQSKR
>	sp|P68035|ACT	Actin, alpha cardiac muscle 1 OS=Rattus norvegicus GN=Actc1 PE=1 SV=1	DSYVGDEAQSKR
>	sp|P68035|ACT	Actin, alpha cardiac muscle 1 OS=Rattus norvegicus GN=Actc1 PE=1 SV=1	EITALAPSTMK
>	sp|P68035|ACT	Actin, alpha cardiac muscle 1 OS=Rattus norvegicus GN=Actc1 PE=1 SV=1	EITALAPSTMK
>	sp|P68035|ACT	Actin, alpha cardiac muscle 1 OS=Rattus norvegicus GN=Actc1 PE=1 SV=1	EITALAPSTMK
>	sp|P68035|ACT	Actin, alpha cardiac muscle 1 OS=Rattus norvegicus GN=Actc1 PE=1 SV=1	EITALAPSTMK
>	sp|P68035|ACT	Actin, alpha cardiac muscle 1 OS=Rattus norvegicus GN=Actc1 PE=1 SV=1	EITALAPSTMK
>	sp|P68035|ACT	Actin, alpha cardiac muscle 1 OS=Rattus norvegicus GN=Actc1 PE=1 SV=1	EKLCYVALDFENEMATAASSSSLEK
>	sp|P68035|ACT	Actin, alpha cardiac muscle 1 OS=Rattus norvegicus GN=Actc1 PE=1 SV=1	HQGVMVGMGQK
>	sp|P68035|ACT	Actin, alpha cardiac muscle 1 OS=Rattus norvegicus GN=Actc1 PE=1 SV=1	HQGVMVGMGQK
>	sp|P68035|ACT	Actin, alpha cardiac muscle 1 OS=Rattus norvegicus GN=Actc1 PE=1 SV=1	HQGVMVGMGQK
>	sp|P68035|ACT	Actin, alpha cardiac muscle 1 OS=Rattus norvegicus GN=Actc1 PE=1 SV=1	IIAPPER
>	sp|P68035|ACT	Actin, alpha cardiac muscle 1 OS=Rattus norvegicus GN=Actc1 PE=1 SV=1	IWHHTFYNELR
>	sp|P68035|ACT	Actin, alpha cardiac muscle 1 OS=Rattus norvegicus GN=Actc1 PE=1 SV=1	KYSVWIGGSIL
>	sp|P68035|ACT	Actin, alpha cardiac muscle 1 OS=Rattus norvegicus GN=Actc1 PE=1 SV=1	KYSVWIGGSILASLSTFQQMWISKQEYDEAGPSIVHR
>	sp|P68035|ACT	Actin, alpha cardiac muscle 1 OS=Rattus norvegicus GN=Actc1 PE=1 SV=1	KYSVWIGGSILASLSTFQQMWISKQEYDEAGPSIVHR
>	sp|P68035|ACT	Actin, alpha cardiac muscle 1 OS=Rattus norvegicus GN=Actc1 PE=1 SV=1	LCYVALDFENEMATAASSSSLEK
>	sp|P68035|ACT	Actin, alpha cardiac muscle 1 OS=Rattus norvegicus GN=Actc1 PE=1 SV=1	LCYVALDFENEMATAASSSSLEK
>	sp|P68035|ACT	Actin, alpha cardiac muscle 1 OS=Rattus norvegicus GN=Actc1 PE=1 SV=1	LDLAGR
>	sp|P68035|ACT	Actin, alpha cardiac muscle 1 OS=Rattus norvegicus GN=Actc1 PE=1 SV=1	SYELPDGQVITIGNER
>	sp|P68035|ACT	Actin, alpha cardiac muscle 1 OS=Rattus norvegicus GN=Actc1 PE=1 SV=1	SYELPDGQVITIGNER
>	sp|P68035|ACT	Actin, alpha cardiac muscle 1 OS=Rattus norvegicus GN=Actc1 PE=1 SV=1	SYELPDGQVITIGNER
>	sp|P68035|ACT	Actin, alpha cardiac muscle 1 OS=Rattus norvegicus GN=Actc1 PE=1 SV=1	SYELPDGQVITIGNER
>	sp|P68035|ACT	Actin, alpha cardiac muscle 1 OS=Rattus norvegicus GN=Actc1 PE=1 SV=1	SYELPDGQVITIGNER
>	sp|P68035|ACT	Actin, alpha cardiac muscle 1 OS=Rattus norvegicus GN=Actc1 PE=1 SV=1	SYELPDGQVITIGNER
>	sp|P68035|ACT	Actin, alpha cardiac muscle 1 OS=Rattus norvegicus GN=Actc1 PE=1 SV=1	SYELPDGQVITIGNER
>	sp|P68035|ACT	Actin, alpha cardiac muscle 1 OS=Rattus norvegicus GN=Actc1 PE=1 SV=1	SYELPDGQVITIGNER
>	sp|P68035|ACT	Actin, alpha cardiac muscle 1 OS=Rattus norvegicus GN=Actc1 PE=1 SV=1	SYELPDGQVITIGNER
>	sp|P68035|ACT	Actin, alpha cardiac muscle 1 OS=Rattus norvegicus GN=Actc1 PE=1 SV=1	SYELPDGQVITIGNER
>	sp|P68035|ACT	Actin, alpha cardiac muscle 1 OS=Rattus norvegicus GN=Actc1 PE=1 SV=1	SYELPDGQVITIGNER

>	sp|P68035|ACT	Actin, alpha cardiac muscle 1 OS=Rattus norvegicus GN=Actc1 PE=1 SV=1	SYELPDGQVITIGNER
>	sp|P68035|ACT	Actin, alpha cardiac muscle 1 OS=Rattus norvegicus GN=Actc1 PE=1 SV=1	SYELPDGQVITIGNER
>	sp|P68035|ACT	Actin, alpha cardiac muscle 1 OS=Rattus norvegicus GN=Actc1 PE=1 SV=1	SYELPDGQVITIGNER
>	sp|P68035|ACT	Actin, alpha cardiac muscle 1 OS=Rattus norvegicus GN=Actc1 PE=1 SV=1	YSVWIGGSILASLSTFQQMWISKQEYDEAGPSIVHR
>	sp|P68035|ACT	Actin, alpha cardiac muscle 1 OS=Rattus norvegicus GN=Actc1 PE=1 SV=1	YSVWIGGSILASLSTFQQMWISKQEYDEAGPSIVHR
>	sp|P68035|ACT	Actin, alpha cardiac muscle 1 OS=Rattus norvegicus GN=Actc1 PE=1 SV=1	YSVWIGGSILASLSTFQQMWISKQEYDEAGPSIVHR
>	sp|P68035|ACT	Actin, alpha cardiac muscle 1 OS=Rattus norvegicus GN=Actc1 PE=1 SV=1	AVFPSIVGRPR
>	sp|P68035|ACT	Actin, alpha cardiac muscle 1 OS=Rattus norvegicus GN=Actc1 PE=1 SV=1	SYELPDGQVITIGNER
>	sp|P68035|ACT	Actin, alpha cardiac muscle 1 OS=Rattus norvegicus GN=Actc1 PE=1 SV=1	YPIEHGIITNWDDMEK
>	sp|P68035|ACT	Actin, alpha cardiac muscle 1 OS=Rattus norvegicus GN=Actc1 PE=1 SV=1	LCYVALDFENEMATAASSSSLEK
>	sp|P68035|ACT	Actin, alpha cardiac muscle 1 OS=Rattus norvegicus GN=Actc1 PE=1 SV=1	AVFPSIVGRPR
>	sp|P68035|ACT	Actin, alpha cardiac muscle 1 OS=Rattus norvegicus GN=Actc1 PE=1 SV=1	YSVWIGGSILASLSTFQQMWISKQEYDEAGPSIVHR
>	sp|P68035|ACT	Actin, alpha cardiac muscle 1 OS=Rattus norvegicus GN=Actc1 PE=1 SV=1	YPIEHGIITNWDDMEK
>	sp|P68035|ACT	Actin, alpha cardiac muscle 1 OS=Rattus norvegicus GN=Actc1 PE=1 SV=1	SYELPDGQVITIGNER
>	sp|P68035|ACT	Actin, alpha cardiac muscle 1 OS=Rattus norvegicus GN=Actc1 PE=1 SV=1	AGFAGDDAPR
>	sp|P68035|ACT	Actin, alpha cardiac muscle 1 OS=Rattus norvegicus GN=Actc1 PE=1 SV=1	IIAPPER
>	sp|P68035|ACT	Actin, alpha cardiac muscle 1 OS=Rattus norvegicus GN=Actc1 PE=1 SV=1	AGFAGDDAPR
>	sp|P68035|ACT	Actin, alpha cardiac muscle 1 OS=Rattus norvegicus GN=Actc1 PE=1 SV=1	AGFAGDDAPR
>	sp|P68035|ACT	Actin, alpha cardiac muscle 1 OS=Rattus norvegicus GN=Actc1 PE=1 SV=1	DLYANNVLSGGTTMYPGIADR
>	sp|P68035|ACT	Actin, alpha cardiac muscle 1 OS=Rattus norvegicus GN=Actc1 PE=1 SV=1	SYELPDGQVITIGNER
>	sp|P68035|ACT	Actin, alpha cardiac muscle 1 OS=Rattus norvegicus GN=Actc1 PE=1 SV=1	SSSSLEKSYELPDGQVITIGNER
>	sp|P68035|ACT	Actin, alpha cardiac muscle 1 OS=Rattus norvegicus GN=Actc1 PE=1 SV=1	AGFAGDDAPR
>	sp|P68035|ACT	Actin, alpha cardiac muscle 1 OS=Rattus norvegicus GN=Actc1 PE=1 SV=1	YSVWIGGSILASLSTFQQMWISKQEYDEAGPSIVHR
>	sp|P68035|ACT	Actin, alpha cardiac muscle 1 OS=Rattus norvegicus GN=Actc1 PE=1 SV=1	IIAPPER
>	sp|P68035|ACT	Actin, alpha cardiac muscle 1 OS=Rattus norvegicus GN=Actc1 PE=1 SV=1	IIAPPER
>	sp|P68035|ACT	Actin, alpha cardiac muscle 1 OS=Rattus norvegicus GN=Actc1 PE=1 SV=1	IIAPPER
>	sp|P68035|ACT	Actin, alpha cardiac muscle 1 OS=Rattus norvegicus GN=Actc1 PE=1 SV=1	IIAPPER
>	sp|P68035|ACT	Actin, alpha cardiac muscle 1 OS=Rattus norvegicus GN=Actc1 PE=1 SV=1	DSYVGDEAQSKR
>	sp|P68035|ACT	Actin, alpha cardiac muscle 1 OS=Rattus norvegicus GN=Actc1 PE=1 SV=1	IIAPPER
>	sp|P68035|ACT	Actin, alpha cardiac muscle 1 OS=Rattus norvegicus GN=Actc1 PE=1 SV=1	AVFPSIVGRPR
>	sp|P68035|ACT	Actin, alpha cardiac muscle 1 OS=Rattus norvegicus GN=Actc1 PE=1 SV=1	LCYVALDFENEMATAASSSSLEK
>	sp|P68035|ACT	Actin, alpha cardiac muscle 1 OS=Rattus norvegicus GN=Actc1 PE=1 SV=1	EKLCYVALDFENEMATAASSSSLEK
>	sp|P68035|ACT	Actin, alpha cardiac muscle 1 OS=Rattus norvegicus GN=Actc1 PE=1 SV=1	EKLCYVALDFENEMATAASSSSLEK
>	sp|P68035|ACT	Actin, alpha cardiac muscle 1 OS=Rattus norvegicus GN=Actc1 PE=1 SV=1	LDLAGR
>	sp|P68035|ACT	Actin, alpha cardiac muscle 1 OS=Rattus norvegicus GN=Actc1 PE=1 SV=1	LCYVALDFENEMATAASSSSLEK
>	sp|P68035|ACT	Actin, alpha cardiac muscle 1 OS=Rattus norvegicus GN=Actc1 PE=1 SV=1	LCYVALDFENEMATAASSSSLEK
>	sp|P68035|ACT	Actin, alpha cardiac muscle 1 OS=Rattus norvegicus GN=Actc1 PE=1 SV=1	EKLCYVALDFENEMATAASSSSLEK
>	sp|P68035|ACT	Actin, alpha cardiac muscle 1 OS=Rattus norvegicus GN=Actc1 PE=1 SV=1	LCYVALDFENEMATAASSSSLEK
>	sp|P68035|ACT	Actin, alpha cardiac muscle 1 OS=Rattus norvegicus GN=Actc1 PE=1 SV=1	SYELPDGQVITIGNER
>	sp|P68035|ACT	Actin, alpha cardiac muscle 1 OS=Rattus norvegicus GN=Actc1 PE=1 SV=1	LCYVALDFENEMATAASSSSLEK
>	sp|P68035|ACT	Actin, alpha cardiac muscle 1 OS=Rattus norvegicus GN=Actc1 PE=1 SV=1	LDLAGRDLTDYLMK
>	sp|P68035|ACT	Actin, alpha cardiac muscle 1 OS=Rattus norvegicus GN=Actc1 PE=1 SV=1	LCYVALDFENEMATAASSSSLEK
>	sp|P68035|ACT	Actin, alpha cardiac muscle 1 OS=Rattus norvegicus GN=Actc1 PE=1 SV=1	LCYVALDFENEMATAASSSSLEK
>	sp|P68035|ACT	Actin, alpha cardiac muscle 1 OS=Rattus norvegicus GN=Actc1 PE=1 SV=1	EKLCYVALDFENEMATAASSSSLEK
>	sp|P68035|ACT	Actin, alpha cardiac muscle 1 OS=Rattus norvegicus GN=Actc1 PE=1 SV=1	DSYVGDEAQSK
>	sp|P68035|ACT	Actin, alpha cardiac muscle 1 OS=Rattus norvegicus GN=Actc1 PE=1 SV=1	LCYVALDFENEMATAASSSSLEK
>	sp|P68035|ACT	Actin, alpha cardiac muscle 1 OS=Rattus norvegicus GN=Actc1 PE=1 SV=1	EITALAPSTMK
>	sp|P68035|ACT	Actin, alpha cardiac muscle 1 OS=Rattus norvegicus GN=Actc1 PE=1 SV=1	YPIEHGIITNWDDMEK
>	sp|P68035|ACT	Actin, alpha cardiac muscle 1 OS=Rattus norvegicus GN=Actc1 PE=1 SV=1	AGFAGDDAPR
>	sp|P68035|ACT	Actin, alpha cardiac muscle 1 OS=Rattus norvegicus GN=Actc1 PE=1 SV=1	GILTLK
>	sp|P68035|ACT	Actin, alpha cardiac muscle 1 OS=Rattus norvegicus GN=Actc1 PE=1 SV=1	ILTER
>	sp|P68035|ACT	Actin, alpha cardiac muscle 1 OS=Rattus norvegicus GN=Actc1 PE=1 SV=1	SYELPDGQVITIGNER
>	sp|P68035|ACT	Actin, alpha cardiac muscle 1 OS=Rattus norvegicus GN=Actc1 PE=1 SV=1	LCYVALDFENEMATAASSSSLEK
>	sp|P68035|ACT	Actin, alpha cardiac muscle 1 OS=Rattus norvegicus GN=Actc1 PE=1 SV=1	KYSVWIGGSILASLSTFQQMWISKQEYDEAGPSIVHR
>	sp|P68035|ACT	Actin, alpha cardiac muscle 1 OS=Rattus norvegicus GN=Actc1 PE=1 SV=1	GILTLK
>	sp|P68035|ACT	Actin, alpha cardiac muscle 1 OS=Rattus norvegicus GN=Actc1 PE=1 SV=1	VAPEEHPTLLTEAPLNPK
>	sp|P68035|ACT	Actin, alpha cardiac muscle 1 OS=Rattus norvegicus GN=Actc1 PE=1 SV=1	HQGVMVGMGQK
>	sp|P68035|ACT	Actin, alpha cardiac muscle 1 OS=Rattus norvegicus GN=Actc1 PE=1 SV=1	AVFPSIVGRPR
>	sp|P68035|ACT	Actin, alpha cardiac muscle 1 OS=Rattus norvegicus GN=Actc1 PE=1 SV=1	VAPEEHPTLLTEAPLNPK
>	sp|P68035|ACT	Actin, alpha cardiac muscle 1 OS=Rattus norvegicus GN=Actc1 PE=1 SV=1	VAPEEHPTLLTEAPLNPK
>	sp|P68035|ACT	Actin, alpha cardiac muscle 1 OS=Rattus norvegicus GN=Actc1 PE=1 SV=1	GILTLK
>	sp|P68035|ACT	Actin, alpha cardiac muscle 1 OS=Rattus norvegicus GN=Actc1 PE=1 SV=1	VAPEEHPTLLTEAPLNPK
>	sp|P68035|ACT	Actin, alpha cardiac muscle 1 OS=Rattus norvegicus GN=Actc1 PE=1 SV=1	HQGVMVGMGQK
>	sp|P68035|ACT	Actin, alpha cardiac muscle 1 OS=Rattus norvegicus GN=Actc1 PE=1 SV=1	LDLAGR
>	sp|P68035|ACT	Actin, alpha cardiac muscle 1 OS=Rattus norvegicus GN=Actc1 PE=1 SV=1	EITALAPSTMK
>	sp|P68035|ACT	Actin, alpha cardiac muscle 1 OS=Rattus norvegicus GN=Actc1 PE=1 SV=1	IWHHTFYNELR
>	sp|P31424|GRI	Metabotropic glutamate receptor 5 OS=Rattus norvegicus GN=Grm5 PE=1 SV=2	LSVHINK
>	RRRRRsp|Q4V8	REVERSED Leucine-rich repeat-containing protein 8A OS=Rattus norvegicus GN=Lrrc8a PE=2 SV=1	LNERLFALAPAEIK
>	RRRRRsp|Q6IFV	REVERSED Keratin, type I cytoskeletal 10 OS=Rattus norvegicus GN=Krt10 PE=2 SV=1	SLTLEDLVRR
>	RRRRRsp|Q5XI5	REVERSED Protein FAM194A OS=Rattus norvegicus GN=Fam194a PE=2 SV=1	ELLENGMVK
>	sp|P11951|CX6	Cytochrome c oxidase subunit 6C-2 OS=Rattus norvegicus GN=Cox6c2 PE=1 SV=3	QAGVFQSAK
>	sp|Q5PQX1|TO	Torsin-1A-interacting protein 1 OS=Rattus norvegicus GN=Tor1aip1 PE=1 SV=1	GTTFLEK
>	sp|Q6AXT3|AR	ADP-ribosylation factor-like protein 13A OS=Rattus norvegicus GN=Arl13a PE=2 SV=1	DLKGREK
>	sp|Q68FX1|MP	Mannose-6-phosphate isomerase OS=Rattus norvegicus GN=Mpi PE=2 SV=1	ILDNRISQK

>	sp|Q4QQT2|F1	Protein FAM118B OS=Rattus norvegicus GN=Fam118b PE=2 SV=1	LLPSLK
>	sp|P62959|HIN	Histidine triad nucleotide-binding protein 1 OS=Rattus norvegicus GN=Hint1 PE=1 SV=5	AQVAQPGGDTIFGK
>	sp|P51146|RA	Ras-related protein Rab-4B OS=Rattus norvegicus GN=Rab4b PE=2 SV=1	LQIWDTAGQER
>	sp|P51146|RA	Ras-related protein Rab-4B OS=Rattus norvegicus GN=Rab4b PE=2 SV=1	LQIWDTAGQER
>	sp|P51146|RA	Ras-related protein Rab-4B OS=Rattus norvegicus GN=Rab4b PE=2 SV=1	LQIWDTAGQER
>	sp|P51146|RA	Ras-related protein Rab-4B OS=Rattus norvegicus GN=Rab4b PE=2 SV=1	LQIWDTAGQER
>	sp|P51146|RA	Ras-related protein Rab-4B OS=Rattus norvegicus GN=Rab4b PE=2 SV=1	TILNKIDSGELDPER
>	RRRRRsp|P267	REVERSED Adenylate cyclase type 4 OS=Rattus norvegicus GN=Adcy4 PE=2 SV=1	LFDLR
>	RRRRRsp|Q9Q	REVERSED Ras GTPase-activating protein SynGAP OS=Rattus norvegicus GN=Syngap1 PE=1 SV=2	AMFSQLDISSNLDRMMYGQMEASPGR
>	RRRRRsp|Q6AY	REVERSED Spermatogenesis-associated protein 1 OS=Rattus norvegicus GN=Spata1 PE=2 SV=1	SDSINALNRPR
>	sp|O54767|ZP	Zona pellucida sperm-binding protein 2 OS=Rattus norvegicus GN=Zp2 PE=1 SV=1	RAHIK
>	RRRRRsp|Q66H	REVERSED Fatty acyl-CoA reductase 1 OS=Rattus norvegicus GN=Far1 PE=2 SV=1	FYSLFK
>	sp|Q3B7T9|RFI	Rab11 family-interacting protein 1 OS=Rattus norvegicus GN=Rab11fip1 PE=2 SV=1	SSLLSLVTGKK
>	sp|P57790|KE	Kelch-like ECH-associated protein 1 OS=Rattus norvegicus GN=Keap1 PE=2 SV=1	LIEFAYTASISVGEK
>	sp|P04785|PDI	Protein disulfide-isomerase OS=Rattus norvegicus GN=P4hb PE=1 SV=2	TLDGFKK
>	sp|Q4FZX7|SR	Signal recognition particle receptor subunit beta OS=Rattus norvegicus GN=Srprb PE=2 SV=1	LIQQQLEK
>	RRRRRsp|Q4V8	REVERSED DCN1-like protein 3 OS=Rattus norvegicus GN=Dcun1d3 PE=2 SV=1	DEQKAETLLSPFR
>	sp|Q03386|GN	Ral guanine nucleotide dissociation stimulator OS=Rattus norvegicus GN=Ralgds PE=1 SV=1	FATLEMNPRRTQR
>	sp|P84083|AR	ADP-ribosylation factor 5 OS=Rattus norvegicus GN=Arf5 PE=1 SV=2	ILMVGLDAAGK
>	sp|P84083|AR	ADP-ribosylation factor 5 OS=Rattus norvegicus GN=Arf5 PE=1 SV=2	ILMVGLDAAGK
>	sp|P84083|AR	ADP-ribosylation factor 5 OS=Rattus norvegicus GN=Arf5 PE=1 SV=2	ILMVGLDAAGK
>	sp|P84083|AR	ADP-ribosylation factor 5 OS=Rattus norvegicus GN=Arf5 PE=1 SV=2	ILMVGLDAAGK
>	sp|P84083|AR	ADP-ribosylation factor 5 OS=Rattus norvegicus GN=Arf5 PE=1 SV=2	ILMVGLDAAGK
>	sp|P84083|AR	ADP-ribosylation factor 5 OS=Rattus norvegicus GN=Arf5 PE=1 SV=2	ILMVGLDAAGK
>	sp|P84083|AR	ADP-ribosylation factor 5 OS=Rattus norvegicus GN=Arf5 PE=1 SV=2	ILMVGLDAAGK
>	sp|P84083|AR	ADP-ribosylation factor 5 OS=Rattus norvegicus GN=Arf5 PE=1 SV=2	LGEIVTTIPTIGFNVETVEYK
>	sp|P84083|AR	ADP-ribosylation factor 5 OS=Rattus norvegicus GN=Arf5 PE=1 SV=2	LGEIVTTIPTIGFNVETVEYK
>	sp|P84083|AR	ADP-ribosylation factor 5 OS=Rattus norvegicus GN=Arf5 PE=1 SV=2	LGEIVTTIPTIGFNVETVEYK
>	sp|P84083|AR	ADP-ribosylation factor 5 OS=Rattus norvegicus GN=Arf5 PE=1 SV=2	LGEIVTTIPTIGFNVETVEYK
>	sp|P84083|AR	ADP-ribosylation factor 5 OS=Rattus norvegicus GN=Arf5 PE=1 SV=2	LGEIVTTIPTIGFNVETVEYK
>	sp|P84083|AR	ADP-ribosylation factor 5 OS=Rattus norvegicus GN=Arf5 PE=1 SV=2	LGEIVTTIPTIGFNVETVEYK
>	sp|P84083|AR	ADP-ribosylation factor 5 OS=Rattus norvegicus GN=Arf5 PE=1 SV=2	ILMVGLDAAGK
>	sp|P84083|AR	ADP-ribosylation factor 5 OS=Rattus norvegicus GN=Arf5 PE=1 SV=2	LGEIVTTIPTIGFNVETVEYK
>	sp|P84083|AR	ADP-ribosylation factor 5 OS=Rattus norvegicus GN=Arf5 PE=1 SV=2	LGEIVTTIPTIGFNVETVEYK
>	sp|P84083|AR	ADP-ribosylation factor 5 OS=Rattus norvegicus GN=Arf5 PE=1 SV=2	LKLGEIVTTIPTIGFNVETVEYK
>	sp|P84083|AR	ADP-ribosylation factor 5 OS=Rattus norvegicus GN=Arf5 PE=1 SV=2	LKLGEIVTTIPTIGFNVETVEYK
>	sp|P84083|AR	ADP-ribosylation factor 5 OS=Rattus norvegicus GN=Arf5 PE=1 SV=2	MLQEDELRDAVLLVFANK
>	sp|P84083|AR	ADP-ribosylation factor 5 OS=Rattus norvegicus GN=Arf5 PE=1 SV=2	LGEIVTTIPTIGFNVETVEYK
>	RRRRRsp|Q5XI	REVERSED Coiled-coil domain-containing protein 81 OS=Rattus norvegicus GN=Ccdc81 PE=2 SV=1	LLTER
>	sp|Q6AXQ7|IQ	IQ and AAA domain-containing protein 1-like OS=Rattus norvegicus GN=Iqca1p1 PE=2 SV=2	QGRLRATFMR
>	sp|Q07008|NO	Neurogenic locus notch homolog protein 1 OS=Rattus norvegicus GN=Notch1 PE=2 SV=2	RSAVGWATTSLLPGTNGGR
>	RRRRRsp|Q5M	REVERSED SHC-transforming protein 1 OS=Rattus norvegicus GN=Shc1 PE=1 SV=1	GLISSLPR
>	sp|Q9JJ54|HN	Heterogeneous nuclear ribonucleoprotein D0 OS=Rattus norvegicus GN=Hnrnpd PE=1 SV=1	GFGFVLFK
>	sp|Q5XIT9|MC	Methylcrotonoyl-CoA carboxylase beta chain, mitochondrial OS=Rattus norvegicus GN=Mccc2 PE=2 SV=1	ISVMGGEQAATVLATVARDQRAR
>	sp|Q06518|NO	Nitric oxide synthase, inducible OS=Rattus norvegicus GN=Nos2 PE=1 SV=2	EIRFTVLVK
>	RRRRRsp|Q5M	REVERSED Leucine-rich repeat-containing protein 41 OS=Rattus norvegicus GN=Lrrc41 PE=2 SV=1	APAAER
>	sp|P04897|GN	Guanine nucleotide-binding protein G(i) subunit alpha-2 OS=Rattus norvegicus GN=Gnai2 PE=1 SV=3	LLLLGAGESGK
>	sp|P04897|GN	Guanine nucleotide-binding protein G(i) subunit alpha-2 OS=Rattus norvegicus GN=Gnai2 PE=1 SV=3	LLLLGAGESGK
>	sp|P04897|GN	Guanine nucleotide-binding protein G(i) subunit alpha-2 OS=Rattus norvegicus GN=Gnai2 PE=1 SV=3	LLLLGAGESGK
>	sp|P04897|GN	Guanine nucleotide-binding protein G(i) subunit alpha-2 OS=Rattus norvegicus GN=Gnai2 PE=1 SV=3	LLLLGAGESGK
>	sp|P04897|GN	Guanine nucleotide-binding protein G(i) subunit alpha-2 OS=Rattus norvegicus GN=Gnai2 PE=1 SV=3	NVQFVFDAVTDVIIK
>	sp|P04897|GN	Guanine nucleotide-binding protein G(i) subunit alpha-2 OS=Rattus norvegicus GN=Gnai2 PE=1 SV=3	TTGIVETHFTFK
>	sp|P04897|GN	Guanine nucleotide-binding protein G(i) subunit alpha-2 OS=Rattus norvegicus GN=Gnai2 PE=1 SV=3	LLLLGAGESGK
>	sp|P04897|GN	Guanine nucleotide-binding protein G(i) subunit alpha-2 OS=Rattus norvegicus GN=Gnai2 PE=1 SV=3	RLWADHGVQACFGR
>	sp|P04897|GN	Guanine nucleotide-binding protein G(i) subunit alpha-2 OS=Rattus norvegicus GN=Gnai2 PE=1 SV=3	LFDSICNNKWFTDTSIILFLNKKDLFEEK
>	sp|P85515|AC	Alpha-centractin OS=Rattus norvegicus GN=Actr1a PE=1 SV=1	FRAPELLFRPDLIGEESEGIHEVLVFAIQK
>	sp|P85515|AC	Alpha-centractin OS=Rattus norvegicus GN=Actr1a PE=1 SV=1	IDIAGR
>	sp|P85515|AC	Alpha-centractin OS=Rattus norvegicus GN=Actr1a PE=1 SV=1	IDIAGR
>	sp|P85515|AC	Alpha-centractin OS=Rattus norvegicus GN=Actr1a PE=1 SV=1	VMAGALEGDIFIGPK
>	sp|P85515|AC	Alpha-centractin OS=Rattus norvegicus GN=Actr1a PE=1 SV=1	IDIAGR
>	RRRRRsp|P025	REVERSED Myosin-7 OS=Rattus norvegicus GN=Myh7 PE=2 SV=2	INWQIILLSDRR
>	sp|P24062|IGF	Insulin-like growth factor 1 receptor OS=Rattus norvegicus GN=Igf1r PE=2 SV=2	LTVITEYLLLFR
>	RRRRRsp|Q5I0	REVERSED Multidrug and toxin extrusion protein 1 OS=Rattus norvegicus GN=Slc47a1 PE=1 SV=1	VLIGGVLVLVVGLFLLGRR
>	sp|O35821|MB	Myb-binding protein 1A OS=Rattus norvegicus GN=Mybbp1a PE=2 SV=2	LSVDLTAFLGMLQGK
>	RRRRRsp|P144	REVERSED Fumarate hydratase, mitochondrial OS=Rattus norvegicus GN=Fh PE=1 SV=1	EAFGIRTNLGTGVATGGAALEYIR
>	RRRRRsp|P706	REVERSED Protein-tyrosine kinase 2-beta OS=Rattus norvegicus GN=Ptk2b PE=1 SV=1	AQDVADLLNKADVALTHSATLMQR
>	sp|P58821|CAL	Neuron-specific vesicular protein calcyon OS=Rattus norvegicus GN=Caly PE=1 SV=1	LGCSFSGKPGK
>	sp|Q3KRF3|CA	Uncharacterized protein C1orf131 homolog OS=Rattus norvegicus PE=2 SV=1	LAPSILSSGR
>	sp|B0BNK8|FA	Protein FAM46B OS=Rattus norvegicus GN=Fam46b PE=2 SV=1	LSWPQVKRLDALLK
>	RRRRRsp|Q5FV	REVERSED TBCC domain-containing protein 1 OS=Rattus norvegicus GN=Tbccd1 PE=2 SV=1	TRSVTGEILFSLATVAER
>	RRRRRsp|P511	REVERSED Nuclear receptor subfamily 4 group A member 3 OS=Rattus norvegicus GN=Nr4a3 PE=2 SV=1	DIVSPPSVLDELK
>	RRRRRsp|Q088	REVERSED Dynamin-3 OS=Rattus norvegicus GN=Dnm3 PE=1 SV=2	NIISMYSDVLNRITEVQR
>	sp|P21932|AD	Adenylate cyclase type 3 OS=Rattus norvegicus GN=Adcy3 PE=2 SV=1	IEVHDQK
>	sp|Q91ZN1|CO	Coronin-1A OS=Rattus norvegicus GN=Coro1a PE=1 SV=3	LDRLEETVQAK

>	sp|O35780|BH	Class E basic helix-loop-helix protein 40 OS=Rattus norvegicus GN=Bhlhe40 PE=2 SV=1	SSQLVTHLHRVVSELLQGSASR
>	RRRRRsp|Q626	REVERSED Urea transporter 2 OS=Rattus norvegicus GN=Slc14a2 PE=1 SV=2	EQSLFYIR
>	RRRRRsp|P004	REVERSED Cytochrome c oxidase subunit 2 OS=Rattus norvegicus GN=Mtco2 PE=2 SV=2	GPIADTKLGLSPIAWSHLVDESSILMR
>	RRRRRsp|Q631	REVERSED Dual specificity protein kinase CLK3 OS=Rattus norvegicus GN=Clk3 PE=2 SV=2	AAERYKGVNR
>	RRRRRsp|P067	REVERSED DNA polymerase beta OS=Rattus norvegicus GN=Polb PE=1 SV=4	TLFNISSSTDDQR
>	sp|Q4V7D7|RB	Pre-mRNA-splicing factor RBM22 OS=Rattus norvegicus GN=Rbm22 PE=2 SV=1	LLKRASTMPR
>	RRRRRsp|D3Z	REVERSED Serine/threonine-protein kinase BRSK2 OS=Rattus norvegicus GN=Brsk2 PE=2 SV=1	VLGTQGK
>	RRRRRsp|D3Z	REVERSED Serine/threonine-protein kinase BRSK2 OS=Rattus norvegicus GN=Brsk2 PE=2 SV=1	VLGTQGK
>	RRRRRsp|B1H2	REVERSED Uncharacterized protein C1orf177 homolog OS=Rattus norvegicus PE=2 SV=1	IAQFHFHPLQTLR
>	sp|Q924S5|LO	Lon protease homolog, mitochondrial OS=Rattus norvegicus GN=Lonp1 PE=2 SV=1	TIRDIIALNPLYR
>	sp|Q8CGX0|IF2	Insulin-like growth factor 2 mRNA-binding protein 1 OS=Rattus norvegicus GN=Igf2bp1 PE=1 SV=1	ITISSLQDLTLYNPERTITVK
>	sp|Q499N4|CO	4-hydroxybenzoate polyprenyltransferase, mitochondrial OS=Rattus norvegicus GN=Coq2 PE=2 SV=1	ALSLSAAAVVNSAPRPLQPYLR
>	sp|P41740|AN	Atrial natriuretic peptide receptor 3 OS=Rattus norvegicus GN=Npr3 PE=2 SV=1	ALFSLVDRVAAAR
>	RRRRRsp|Q6AY	REVERSED Microfibril-associated glycoprotein 3 OS=Rattus norvegicus GN=Mfap3 PE=2 SV=1	LTVSYSAR
>	sp|P00759|KLK	Tonin OS=Rattus norvegicus GN=Klk2 PE=1 SV=1	VIDLPTK
>	RRRRRsp|P605	REVERSED Pannexin-2 OS=Rattus norvegicus GN=Panx2 PE=1 SV=2	IFDLR
>	sp|Q80WE1|F	Fragile X mental retardation protein 1 homolog OS=Rattus norvegicus GN=Fmr1 PE=2 SV=2	RGGGGRGQGGR
>	sp|Q642B2|ZN	Zinc finger protein 672 OS=Rattus norvegicus GN=Znf672 PE=2 SV=1	GFLESATLVRHQR
>	sp|Q5U2R6|K1	UPF0498 protein KIAA1191 homolog OS=Rattus norvegicus GN=Aa2-141 PE=2 SV=1	QTQESIQR
>	sp|Q6MG61|C	Chloride intracellular channel protein 1 OS=Rattus norvegicus GN=Clic1 PE=1 SV=1	GVTFNVTTVDTKR
>	RRRRRsp|P218	REVERSED Cysteine dioxygenase type 1 OS=Rattus norvegicus GN=Cdo1 PE=1 SV=1	LNGQLLK
>	sp|B3GNI6|SEP	Septin-11 OS=Rattus norvegicus GN=Sept11 PE=1 SV=1	VNIIPIIAK
>	sp|B3GNI6|SEP	Septin-11 OS=Rattus norvegicus GN=Sept11 PE=1 SV=1	VNIIPIIAK
>	sp|Q63164|DY	Dynein heavy chain 1, axonemal OS=Rattus norvegicus GN=Dnah1 PE=2 SV=2	LAELR
>	RRRRRsp|Q5BJ	REVERSED Putative ATP-dependent RNA helicase DHX30 OS=Rattus norvegicus GN=Dhx30 PE=2 SV=1	NEAEAK
>	RRRRRsp|P007	REVERSED Carboxypeptidase A1 OS=Rattus norvegicus GN=Cpa1 PE=2 SV=2	LGTDRLEFTFSYKIGQSYTWDITSGSAQYITDIISGYK
>	sp|Q62812|MY	Myosin-9 OS=Rattus norvegicus GN=Myh9 PE=1 SV=3	LDPHLVLDQLR
>	sp|Q62812|MY	Myosin-9 OS=Rattus norvegicus GN=Myh9 PE=1 SV=3	LDPHLVLDQLR
>	sp|Q62812|MY	Myosin-9 OS=Rattus norvegicus GN=Myh9 PE=1 SV=3	LDPHLVLDQLR
>	sp|Q62812|MY	Myosin-9 OS=Rattus norvegicus GN=Myh9 PE=1 SV=3	LDPHLVLDQLR
>	sp|Q62812|MY	Myosin-9 OS=Rattus norvegicus GN=Myh9 PE=1 SV=3	AGKLDPHLVLDQLR
>	sp|P09810|TP	Tryptophan 5-hydroxylase 1 OS=Rattus norvegicus GN=Tph1 PE=2 SV=2	LPLLSK
>	sp|P09810|TP	Tryptophan 5-hydroxylase 1 OS=Rattus norvegicus GN=Tph1 PE=2 SV=2	LPLLSK
>	sp|P09810|TP	Tryptophan 5-hydroxylase 1 OS=Rattus norvegicus GN=Tph1 PE=2 SV=2	LPLLSK
>	sp|P09810|TP	Tryptophan 5-hydroxylase 1 OS=Rattus norvegicus GN=Tph1 PE=2 SV=2	LPLLSK
>	sp|Q8K1Q4|PR	ProSAP-interacting protein 1 OS=Rattus norvegicus GN=Prosapip1 PE=1 SV=2	LAELR
>	sp|Q6IV68|FA	Fanconi anemia group D2 protein homolog OS=Rattus norvegicus GN=Fancd2 PE=2 SV=2	IAELR
>	RRRRRsp|P975	REVERSED Cullin-associated NEDD8-dissociated protein 1 OS=Rattus norvegicus GN=Cand1 PE=1 SV=1	AATLSDSYNK
>	sp|Q5M9F8|N	N-terminal kinase-like protein OS=Rattus norvegicus GN=Scyl1 PE=2 SV=1	ATALRNPGK
>	RRRRRsp|Q9EP	REVERSED Solute carrier organic anion transporter family member 1C1 OS=Rattus norvegicus GN=Slco1c1 PE=1 SV=1	ATIFSSRK
>	RRRRRsp|Q5BJ	REVERSED Ribosome-releasing factor 2, mitochondrial OS=Rattus norvegicus GN=Gfm2 PE=2 SV=2	LITER
>	sp|A9ZSY0|CN	Centlein (Fragment) OS=Rattus norvegicus GN=Cntln PE=2 SV=1	LAELR
>	sp|A9ZSY0|CN	Centlein (Fragment) OS=Rattus norvegicus GN=Cntln PE=2 SV=1	NEKAISEQTLEK
>	sp|Q8VI04|AS	L-asparaginase OS=Rattus norvegicus GN=Asrgl1 PE=1 SV=1	LITER
>	sp|P0C6B8|SV	Sushi, von Willebrand factor type A, EGF and pentraxin domain-containing protein 1 OS=Rattus norvegicus GN=Svep1 PE=1 SV=1	LLSDFPVVSTATRVAIVTFSSK
>	sp|O70277|TRI	Tripartite motif-containing protein 3 OS=Rattus norvegicus GN=Trim3 PE=1 SV=1	SPGGPGSHVR
>	RRRRRsp|Q074	REVERSED Heat shock 70 kDa protein 1A/1B OS=Rattus norvegicus GN=Hspa1a PE=2 SV=2	TRDLGYAIAAATPENIIRLVNLGAIVGADKTAQR
>	sp|P39052|DY	Dynamin-2 OS=Rattus norvegicus GN=Dnm2 PE=1 SV=1	LDLMDEGTDAR
>	sp|P39052|DY	Dynamin-2 OS=Rattus norvegicus GN=Dnm2 PE=1 SV=1	FPFELVK
>	sp|P39052|DY	Dynamin-2 OS=Rattus norvegicus GN=Dnm2 PE=1 SV=1	TLNQQLTNHIR
>	sp|P39052|DY	Dynamin-2 OS=Rattus norvegicus GN=Dnm2 PE=1 SV=1	GISPVPINLR
>	sp|Q7M6Z5|KI	Kinesin-like protein KIF27 OS=Rattus norvegicus GN=Kif27 PE=2 SV=1	IAEIR
>	sp|P14650|PER	Thyroid peroxidase OS=Rattus norvegicus GN=Tpo PE=2 SV=1	LITER
>	sp|Q9Z1C8|RP	Rap guanine nucleotide exchange factor 3 OS=Rattus norvegicus GN=Rapgef3 PE=2 SV=2	VIDNQRELSRLSR
>	sp|Q6WRH9|IG	Immunoglobulin superfamily member 10 OS=Rattus norvegicus GN=Igsf10 PE=2 SV=1	LSDSADFTCVVRSEGGESVLVVQLEVLEMLRRPTFR
>	sp|P19356|HE	Porphobilinogen deaminase OS=Rattus norvegicus GN=Hmbs PE=1 SV=2	TLETLPEKSAVGTSSLR
>	sp|Q08877|DY	Dynamin-3 OS=Rattus norvegicus GN=Dnm3 PE=1 SV=2	ALLQMVQQFAVDFEK
>	sp|Q08877|DY	Dynamin-3 OS=Rattus norvegicus GN=Dnm3 PE=1 SV=2	ALLQMVQQFAVDFEK
>	sp|Q08877|DY	Dynamin-3 OS=Rattus norvegicus GN=Dnm3 PE=1 SV=2	ALLQMVQQFAVDFEK
>	sp|Q08877|DY	Dynamin-3 OS=Rattus norvegicus GN=Dnm3 PE=1 SV=2	ALLQMVQQFAVDFEKR
>	sp|Q08877|DY	Dynamin-3 OS=Rattus norvegicus GN=Dnm3 PE=1 SV=2	RPLVLQLVTSKAEYAEFLHCK
>	sp|Q08877|DY	Dynamin-3 OS=Rattus norvegicus GN=Dnm3 PE=1 SV=2	LDLMDEGTDAR
>	sp|Q08877|DY	Dynamin-3 OS=Rattus norvegicus GN=Dnm3 PE=1 SV=2	VLNQQLTNHIR
>	sp|Q08877|DY	Dynamin-3 OS=Rattus norvegicus GN=Dnm3 PE=1 SV=2	FPFEIVK
>	sp|Q08877|DY	Dynamin-3 OS=Rattus norvegicus GN=Dnm3 PE=1 SV=2	LTLSAPLPRPASSRGPAPAIPSPGPHS
>	sp|Q08877|DY	Dynamin-3 OS=Rattus norvegicus GN=Dnm3 PE=1 SV=2	RSPPPSPTTQR
>	RRRRRsp|Q924	REVERSED Lon protease homolog, mitochondrial OS=Rattus norvegicus GN=Lonp1 PE=2 SV=1	EATLAK
>	sp|Q6REY9|RH	Rho GTPase-activating protein 20 OS=Rattus norvegicus GN=Arhgap20 PE=2 SV=2	LINQSLVMGIEVGK
>	RRRRRsp|Q0V8	REVERSED Contactin-associated protein like 5-1 OS=Rattus norvegicus GN=Cntnap5a PE=2 SV=1	LTNLLR
>	RRRRRsp|P0C5	REVERSED Patatin-like phospholipase domain-containing protein 2 OS=Rattus norvegicus GN=Pnpla2 PE=2 SV=1	LNFQISTNTIR
>	RRRRRsp|P096	REVERSED Potassium-transporting ATPase alpha chain 1 OS=Rattus norvegicus GN=Atp4a PE=2 SV=3	MVLLHR
>	sp|P97678|KC	Calcium-activated potassium channel subunit beta-1 OS=Rattus norvegicus GN=Kcnmb1 PE=2 SV=4	LVMAQK
